# Supplementary material for: Diversity-oriented synthesis of P-stereogenic and axially chiral monodentate biaryl phosphines enabled by C-P bond cleavage
Source: Nat Commun. 2023 Jul 24;14:4437. doi: 10.1038/s41467-023-40138-8 (PMC10363526; doi:10.1038/s41467-023-40138-8)
Supplement: Supplementary file 1 — Supplementary Information [file 41467_2023_40138_MOESM1_ESM.pdf]

Supplementary Information

Diversity-oriented Synthesis of P-stereogenic and Axially Chiral Monodentate Biaryl Phosphines Enabled by C-P Bond Cleavage

Liangzhi Pang[1], Zhan Huang[1], Qilin Sun[1], Gen Li[1], Jiaojiao Liu[1], Baoli Li[1], Congyue Ma[1], Jiaxu Guo[1], Chuanzhi Yao[1], Jie Yu[*,1,2] & Qiankun Li[*,1,2]

[1]Department of Applied Chemistry, Anhui Agricultural University, Hefei, Anhui 230036, China

[2]School of Plant Protection, Anhui Province Engineering Laboratory for Green Pesticide Development and Application, and Anhui Province Key Laboratory of Crop Integrated Pest Management, Anhui Agricultural University, Hefei, Anhui 230036, China

Correspondence to: liqk@ahau.edu.cn; jieyu@ustc.edu.cn

Table of Contents

1	Supplementary Methods		S2
2	Supplementary Discussion		S3
	2.1 Optimization of reaction conditions		S3
	2.2 X-ray crystal structure		S7
3	Supplementary Notes		S11
	3.1	Preparation of phosphonium salts	S11
	3.2	Enantioselective alkynylation of C-P bond with terminal alkynes	S14
	3.3	Enantioselective silylation of C-P bond	S27
	3.4	Enantioselective borylation of C-P bond	S35
	3.5	Enantioselective reduction of C-P bond with water as a hydride source	S38
	3.6	Using HCOONa as a hydride donor instead of B_2pin_2/H_2O	S45
	3.7	Synthetic applications	S46
	3.8	Deprotection of the phosphine sulphides and boranes	S48
	3.9	Testing the developed ligands in [3+2] annulation of MBH carbonates	S50
	3.10	NMR and HPLC spectra	S51
4	Supplementary References		S400

1. Supplementary Methods

All reagents were obtained commercially unless otherwise noted. Visualization was accomplished with UV light (254 nm) or $KMnO_4$ stain. Flash column chromatography was performed using silica gel (300-400 mesh). Nuclear Magnetic Resonance (NMR) spectra were acquired on an Agilent 600 NMR spectrometer. ^{1}H NMR spectra were reported in δ ppm relative to $SiMe_4$ (δ 0.00) or the residue solvents from the deuterium solvents. ^{13}C NMR spectra were reported relative to $CDCl_3$ (δ 77.16) or the other deuterium solvents. NMR acquisitions were performed at 295 K unless otherwise noted. Abbreviations are: s, singlet; d, doublet; t, triplet; q, quartet, p, pentet; bs, broad singlet; m, multiplet. High-resolution mass spectra (ESI) were measured on waters G2-Xs QTOF mass spectrometer. Optical rotation was measured using an Anton Paar MCP-100 polarimeter. HPLC analysis was conducted on a Waters-Breeze system equipped with Daicel chiral-stationary-phase columns (4.6 mm×250 mm). Single crystal structures of the compounds were determined by measuring X-ray intensity data on a XtaLAB Synergy, Dualflex, HyPix diffractometer.

2. Supplementary Discussion

2.1 Optimization of reaction conditions

Supplementary Table 1. Chiral Phosphine Ligand

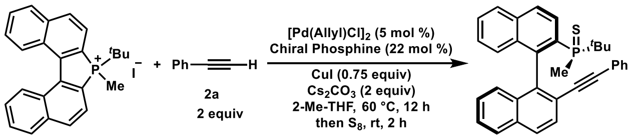

1a 3a

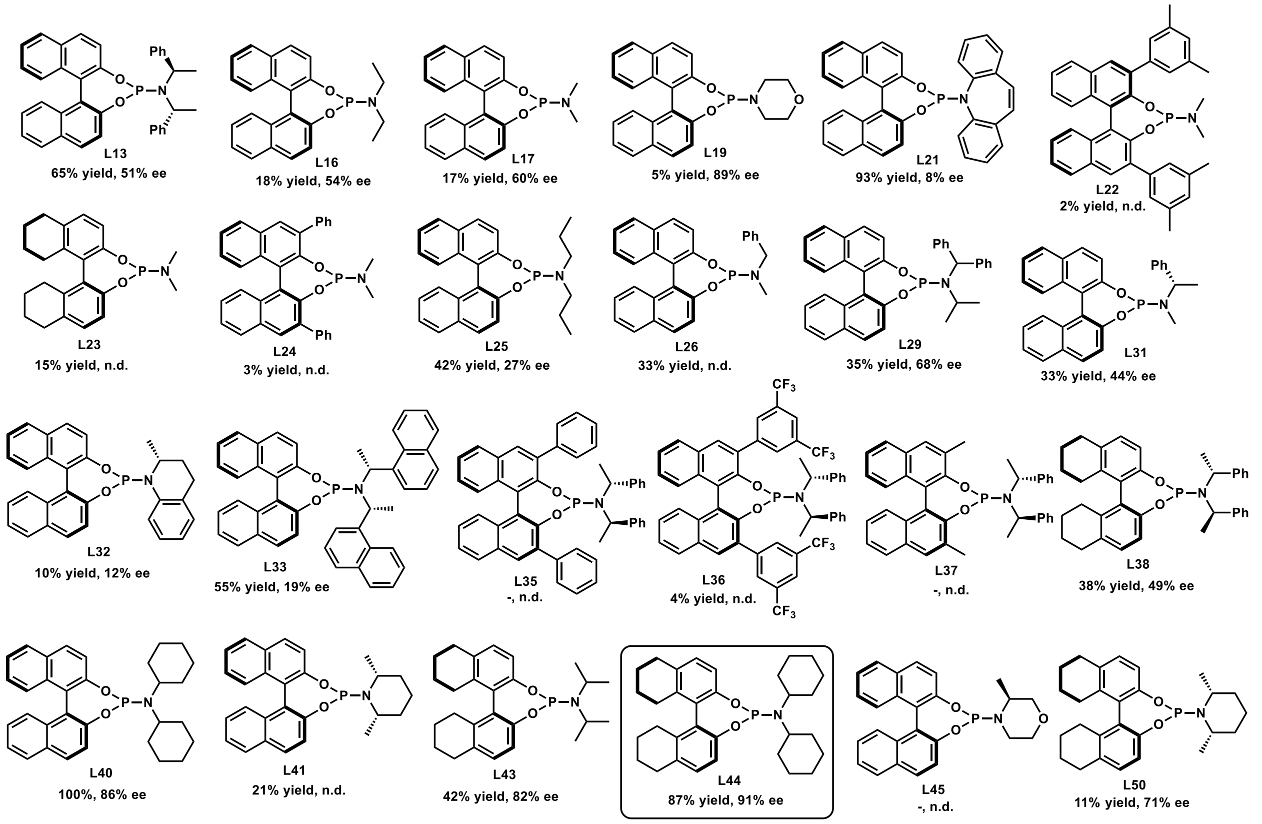

Supplementary Table 2. Cu(I) Salts

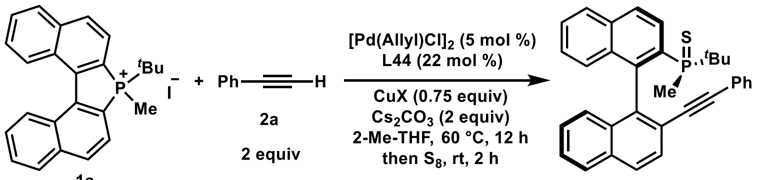

1a 3a

Entry	CuX	yield (%)/3a	ee (%)/3a	dr/3a	No.
1	CuCl	47	90	>25:1	PLZ-6-174A
2	CuBr	90	91	>25:1	PLZ-6-174B
3	CuI	87	91	>25:1	PLZ-6-174C

Supplementary Table 3. Solvents

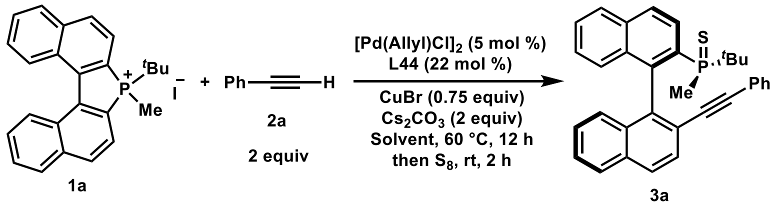

Entry	solvent	yield (%)/3a	ee (%)/3a	dr/3a	No.
1	TBME	19	95	>25:1	PLZ-7-6A
2	DME	55	91	>25:1	PLZ-7-6B
3	Toluene	16	89	>25:1	PLZ-7-6C
4	CH_3CN	trace	n.d.	n.d.	PLZ-7-6D
5	2-Me-THF	90	91	>25:1	PLZ-6-174B

Supplementary Table 4. Base

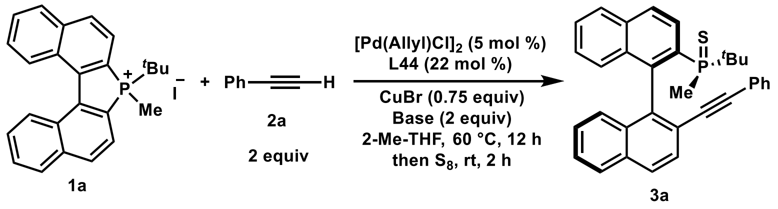

Entry	Base	Yield (%)/3a	ee (%)/3a	dr/3a	No.
1	K_2CO_3	78	93	>25:1	PLZ-7-14A
2	DBU	30	90	>25:1	PLZ-7-14B
3	Et_3N	-	n.d.	n.d.	PLZ-7-14C
4	K_3PO_4	76	93	>25:1	PLZ-7-14D
5	Cs_2CO_3	90	91	>25:1	PLZ-6-174B

Supplementary Table 5. Temperature

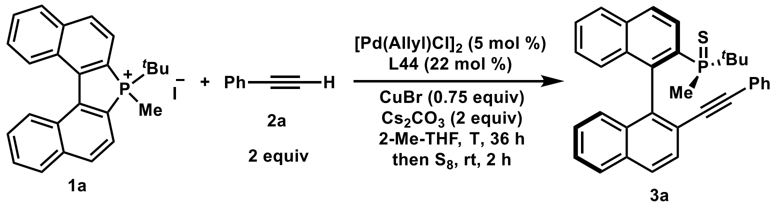

Entry	T (°C)	Yield (%)/3a	ee (%)/3a	dr/3a	No.
1 [a]	60	90	91	>25:1	PLZ-6-174B
2	50	92	92	>25:1	PLZ-7-19A
3	45	93	93	>25:1	PLZ-7-19B
4	40	81	94	>25:1	PLZ-7-19C
5 [b]	45	98	92	>25:1	PLZ-7-48E

[a] 12 h; [b] $[Pd(Allyl)Cl]_2$ (2.5 mol %), **L44** (11 mol %).

Supplementary Table 6. Amount of CuBr

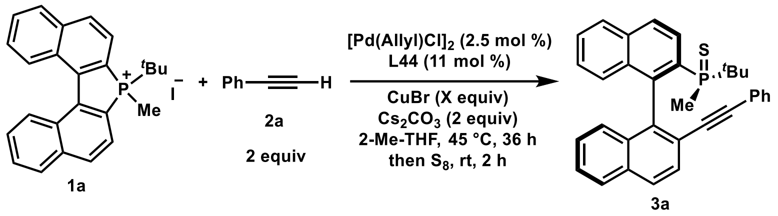

Entry	CuBr (equiv)	yield (%)/3a	ee (%)/3a	dr/3a	No.
1	0	11	90	>25:1	PLZ-7-80A
2	0.1	11	89	>25:1	PLZ-7-80B
3	0.25	7	90	>25:1	PLZ-7-80C
4	0.5	8	91	>25:1	PLZ-7-80D
5	0.75	98	92	>25:1	PLZ-7-48E
6	1.0	>99 (83)	93	>25:1	PLZ-7-48F
7	1.5	>99	93	>25:1	PLZ-7-58A
8	2.0	82	94	>25:1	PLZ-7-58B

Supplementary Table 7. Additives

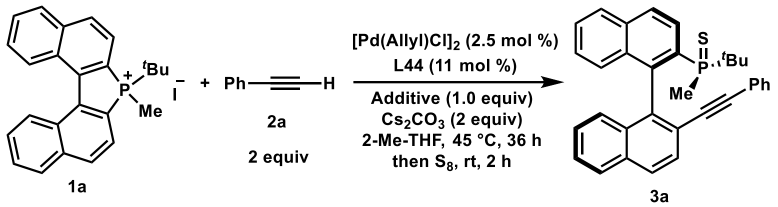

Entry	additives	Yield (%)/3a	ee (%)/3a	dr/3a	No.
1	-	11	90	>25:1	PLZ-7-80A
2	CuBr	>99 (83)	93	>25:1	PLZ-7-48F
3	CuI	58	93	>25:1	PLZ-7-91B
4	CuCl	>99	92	>25:1	PLZ-7-91C
5	Cu_2O	30	92	>25:1	PLZ-7-91A
6	CuCN	6	n.d.	>25:1	PLZ-7-91F
7	$CuBr_2$	-	n.d.	n.d.	PLZ-7-91D
8	$NiCl_2$	27	93	>25:1	PLZ-7-91H
9	LiCl	-	n.d.	n.d.	PLZ-7-91G
10	$ZnCl_2$	-	n.d.	n.d.	PLZ-7-91I
11	$MgCl_2$	-	n.d.	n.d.	PLZ-7-91J
12	$FeCl_2$	-	n.d.	n.d.	PLZ-7-91K
13	$CoCl_2$	-	n.d.	n.d.	PLZ-7-91L

2.2. X-ray crystal structure

The absolute configuration of **3c** was determined by X-ray crystallographic analysis. By analogy, the absolute configurations of products **3** were deduced. CCDC 2227153 contains the supplementary crystallographic data of **3c**.

Supplementary Table 8. Crystal data and structure refinement for 3c (CCDC 2227153)

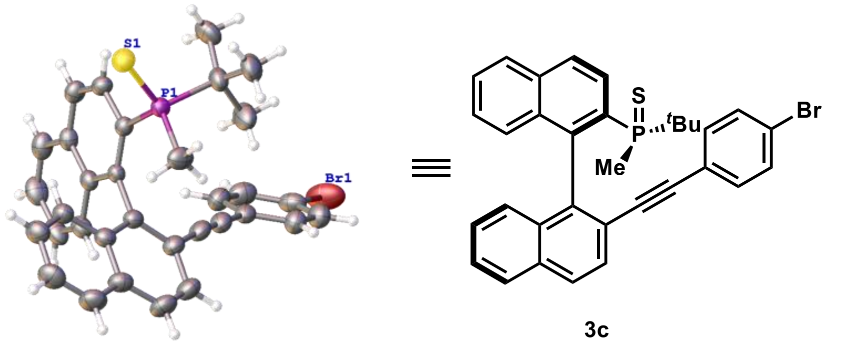

3c

Identification code	7-97A
Empirical formula	$C_{33}H_{28}BrPS$
Formula weight	567.49
Temperature/K	295.37(10)
Crystal system	orthorhombic
Space group	$P2_12_12_1$
a/Å	11.1725(2)
b/Å	11.8554(2)
c/Å	21.5888(3)
α/°	90
β/°	90
γ/°	90
Volume/Å^3	2859.53(8)
Z	4
ρ_{calc}g/cm^3	1.318
μ/mm^{-1}	3.306
F(000)	1168.0
Crystal size/mm^3	0.12 × 0.11 × 0.09
Radiation	Cu Kα (λ = 1.54184)
2Θ range for data collection/	8.19 to 151.028
Index ranges	$-14 \le h \le 13$, $-11 \le k \le 14$, $-26 \le l \le 20$
Reflections collected	10881
Independent reflections	5008 [R_{int} = 0.0305, R_{sigma} = 0.0340]
Data/restraints/parameters	5008/0/329
Goodness-of-fit on F^2	1.110
Final R indexes [I>=2σ (I)]	R_1= 0.0510, wR_2= 0.1466
Final R indexes [all data]	R_1= 0.0580, wR_2= 0.1513
Largest diff. peak/hole / e Å^{-3}	0.66/-0.74
Flack/Hooft parameter	-0.010(19)/0.001(10)

Crystal structure determination of 3c

Crystal Data for $C_{33}H_{28}BrPS$ (M =567.49 g/mol): orthorhombic, space group $P2_12_12_1$(no. 19), a = 11.1725(2) Å, b = 11.8554(2) Å, c = 21.5888(3) Å, V = 2859.53(8) Å^3, Z = 4, T = 295.37(10) K, μ(Cu Kα) = 3.306 mm^{-1}, $Dcalc$ = 1.318 g/cm^3, 10881 reflections measured (8.19° ≤ 2Θ ≤ 151.028°), 5008 unique (R_{int} = 0.0305, R_{sigma} = 0.0340) which were used in all calculations. The final R_1 was 0.0510 (I > 2σ(I)) and wR_2 was 0.1513 (all data).

The absolute configuration of **5o** was determined by X-ray crystallographic analysis. By analogy, the absolute configurations of products **5** were deduced. CCDC 2227154 contains the supplementary crystallographic data of **5o**.

Supplementary Table 9. Crystal data and structure refinement for 5o (CCDC 2227154)

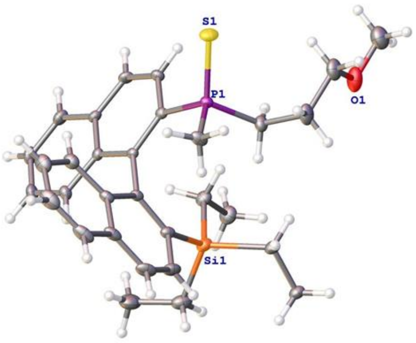

≡

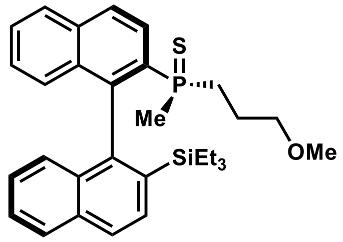

5o

Identification code	9-140
Empirical formula	$C_{31}H_{39}OPSSi$
Formula weight	518.74
Temperature/K	199.99(10)
Crystal system	monoclinic
Space group	$P2_1$
a/Å	11.6082(3)
b/Å	10.3595(2)
c/Å	12.0994(3)
α/°	90
β/°	96.928(2)
γ/°	90
Volume/Å^3	1444.40(6)
Z	2
ρ_{calc}g/cm^3	1.193
μ/mm^{-1}	2.068
F(000)	556.0
Crystal size/mm^3	0.13 × 0.12 × 0.1
Radiation	Cu Kα (λ = 1.54184)
2Θ range for data collection/°	7.36 to 147.57
Index ranges	-14 ≤ h ≤ 14, -12 ≤ k ≤ 12, -14 ≤ l ≤ 13
Reflections collected	7247
Independent reflections	4573 [R_{int} = 0.0248, R_{sigma} = 0.0345]
Data/restraints/parameters	4573/26/318
Goodness-of-fit on F^2	1.070
Final R indexes [I>=2σ (I)]	R_1 = 0.0512, wR_2 = 0.1388
Final R indexes [all data]	R_1 = 0.0521, wR_2 = 0.1402
Largest diff. peak/hole / e Å^{-3}	0.52/-0.77
Flack/Hooft parameter	0.019(16)/0.019(10)

Crystal structure determination of 5o

Crystal Data for $C_{31}H_{39}OPSSi$ (M =518.74 g/mol): monoclinic, space group $P2_1$ (no. 4), a = 11.6082(3) Å, b = 10.3595(2) Å, c = 12.0994(3) Å, β = 96.928(2) °, V = 1444.40(6) Å^3, Z = 2, T = 199.99(10) K, μ(Cu Kα) = 2.068 mm^{-1}, $Dcalc$ = 1.193 g/cm^3, 7247 reflections measured (7.36° ≤ 2Θ ≤ 147.57°), 4573 unique (R_{int} = 0.0248, R_{sigma} = 0.0345) which were used in all calculations. The final R_1 was 0.0512 (I > 2σ(I)) and wR_2 was 0.1402 (all data).

The absolute configuration of **7h** was determined by X-ray crystallographic analysis. By analogy, the absolute configurations of products **7** were deduced. CCDC 2227155 contains the supplementary crystallographic data of **7h**.

Supplementary Table 10. Crystal data and structure refinement for 7h (CCDC 2227155)

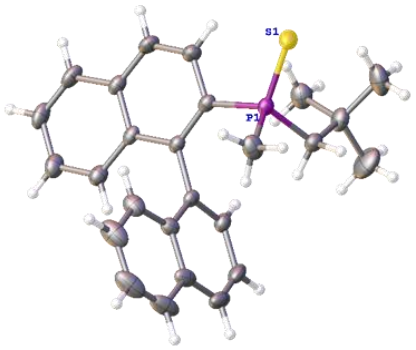

≡

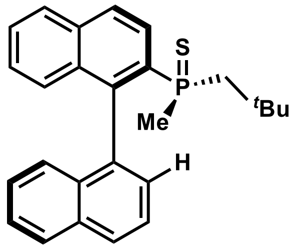

7h

Identification code	9-50
Empirical formula	$C_{52}H_{54}P_2S_2$
Formula weight	805.01
Temperature/K	149.97(10)
Crystal system	monoclinic
Space group	$P2_1$
a/Å	11.1729(3)
b/Å	19.3151(3)
c/Å	11.3800(2)
α/°	90
β/°	116.119(3)
γ/°	90
Volume/Å^3	2205.08(9)
Z	2
ρ_{calc}g/cm^3	1.212
μ/mm^{-1}	2.033
F(000)	856.0
Crystal size/mm^3	0.14 × 0.12 × 0.1
Radiation	Cu Kα (λ = 1.54184)
2Θ range for data collection/°	8.654 to 143.472
Index ranges	-13 ≤ h ≤ 11, -22 ≤ k ≤ 17, -11 ≤ l ≤ 14
Reflections collected	12961
Independent reflections	6906 [R_{int} = 0.0327, R_{sigma} = 0.0375]
Data/restraints/parameters	6906/1/513
Goodness-of-fit on F^2	1.035
Final R indexes [I>=2σ (I)]	R_1 = 0.0389, wR_2 = 0.1016
Final R indexes [all data]	R_1 = 0.0400, wR_2 = 0.1022
Largest diff. peak/hole / e Å^{-3}	0.21/-0.48
Flack/Hooft parameter	-0.001(15)/-0.012(7)

Crystal structure determination of 7h

Crystal Data for $C_{52}H_{54}P_2S_2$ (M =805.01 g/mol): monoclinic, space group P2$_1$ (no. 4), a = 11.1729(3) Å, b = 19.3151(3) Å, c = 11.3800(2) Å, β = 116.119(3) °, V = 2205.08(9) Å^3, Z = 2, T = 149.97(10) K, μ(Cu Kα) = 2.033 mm^{-1}, $Dcalc$ = 1.212 g/cm^3, 12961 reflections measured (8.654° ≤ 2Θ ≤ 143.472°), 6906 unique (R_{int} = 0.0327, R_{sigma} = 0.0375) which were used in all calculations. The final R_1 was 0.0389 (I > 2σ(I)) and wR_2 was 0.1022 (all data).

3 Supplementary Notes

3.1 Preparation of phosphonium salts

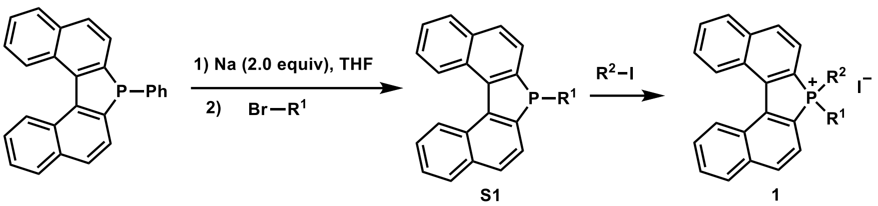

Typical Procedure: According to the reported procedure[1], Sodium (230 mg, 10.0 mmol, small pieces) was added to a mixture of 7-Phenyldinaphtho[2,1-*b*:1',2'-*d*]phosphole (1.80 g, 5.0 mmol) in dry THF (15 mL) at room temperature. The solution was freeze with liquid N_2 and the tube was flushed with N_2. The tube was then sealed and placed in oil bath at 70 °C for 7 h. Then the mixture was cooled to room temperature, *t*-BuCl (0.55 mL, 5.0 mmol) was added. After warming at 70 °C for further 4 h, the dark red solution was cooled to 0 ℃, the appropriate alkyl halide (5.5 mmol) was added dropwise, and was heated at 70 °C for 10 h. After cooling to room temperature, The THF was rotoevaporated and the crude product was purified by flash chromatography to afford phosphole **S1** which was used directly in the next step.

A solution of phosphole **S1** was stirred with MeI (5 mL) for 4 h at room temperature or EtI (5 mL) for 6 h at 40 °C. The solvent was evaporated over vacuum and the crude product was purified by flash chromatography using CH_2Cl_2/AcOEt (1:1) then CH_2Cl_2/MeOH (30:1) as eluent to afford the corresponding phosphonium salts **1** as a yellow solid.

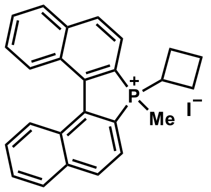

(**1b**) Yellow solid, 61% yield, M.p. 163-164 °C. **¹H NMR** (600 MHz, Chloroform-*d*) δ 8.73 (s, 1H), 8.53 (s, 1H), 8.23 (s, 4H), 8.10 (s, 2H), 7.77 (s, 2H), 7.65 (s, 2H), 5.04 (s, 1H), 2.86 (d, *J* = 14.3 Hz, 3H), 2.77-1.78 (m, 6H). **¹³C NMR** (151 MHz, Chloroform-*d*) δ 144.70, 137.95, 131.93, 129.22, 128.96 (d, *J* = 10.3 Hz), 127.71, 126.86, 126.05 (d, *J* = 10.8 Hz), 120.00 (d, *J* = 90.6 Hz), 27.73 (d, *J* = 42.2 Hz), 22.98, 20.01 (d, *J* = 17.9 Hz), 7.86 (d, *J* = 47.4 Hz). **³¹P NMR** (243 MHz, Chloroform-*d*) δ 34.77. **HRMS (ESI)** calcd for: $C_{25}H_{22}P^+$ $[M – I^-]^+$ 353.1454; found: 353.1453.

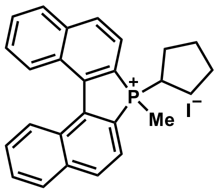

(**1c**) Yellow solid, 63% yield, M.p. 166 °C. **¹H NMR** (600 MHz, Chloroform-*d*) δ 8.71 (s, 1H), 8.51 (s, 1H), 8.32-8.16 (m, 4H), 8.10 (d, *J* = 8.2 Hz, 2H), 7.77 (t, *J* = 7.4 Hz, 2H), 7.65 (t, *J* = 7.5 Hz, 2H), 4.47-4.32 (m, 1H), 2.93 (d, *J* = 14.4 Hz, 3H), 2.48-1.53 (m, 8H). **¹³C NMR** (151 MHz, Chloroform-*d*) δ 144.57, 137.88, 131.92 (d, *J* = 10.0 Hz), 129.21, 129.19, 128.89 (d, *J* = 9.3 Hz), 127.68, 126.86, 125.81, 120.58 (d, *J* = 86.5 Hz), 32.45 (d, *J* = 47.3 Hz), 27.10, 26.34 (d, *J* = 10.0 Hz), 8.70 (d, *J* = 48.3 Hz). **³¹P NMR** (243 MHz, Chloroform-*d*) δ 37.66. **HRMS (ESI)** calcd for: $C_{26}H_{24}P^+$ $[M – I^-]^+$ 367.1610; found: 367.1616.

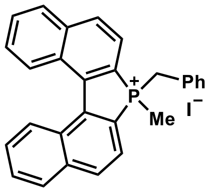

(**1d**) Yellow solid, 50% yield, M.p. 275-276 °C. **¹H NMR** (600 MHz, Chloroform-*d*) δ 8.89 (s, 1H), 8.62 (s, 1H), 8.18 (s, 2H), 8.13-7.91 (m, 4H), 7.72 (t, *J* = 7.4 Hz, 2H), 7.56 (t, *J* = 7.2 Hz, 2H), 7.13 (d, *J* = 6.0 Hz, 2H), 7.02-6.90 (m, 3H), 5.42 (s, 1H), 4.90 (s, 1H), 3.04 (d, *J* = 14.7 Hz, 3H). **¹³C NMR** (151 MHz, Chloroform-*d*) δ 144.70 (d, *J* = 21.3 Hz), 137.68, 131.50 (d, *J* = 13.5 Hz), 129.64, 129.60, 128.96, 128.70 (d, *J* = 11.0 Hz), 128.07 (d, *J* = 3.5 Hz), 127.72 (d, *J* = 4.1 Hz), 127.42, 127.20 (d, *J* = 9.3 Hz), 126.62 (d, *J* = 11.2 Hz), 126.51, 120.27 (d, *J* = 90.0 Hz), 31.03 (d, *J* = 42.4 Hz), 9.12 (d, *J* = 47.1 Hz). **³¹P NMR**

(243 MHz, Chloroform-*d*) δ 34.90. **HRMS (ESI)** calcd for: $C_{28}H_{22}P^+$ [M – I$^-$]$^+$ 389.1454; found: 389.1455.

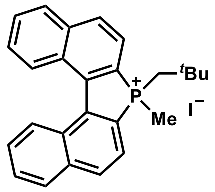

(**1e**) Yellow solid, 52% yield, M.p. > 300 °C. **^{1}H NMR** (600 MHz, Chloroform-*d*) δ 9.03 (s, 2H), 8.24 (dd, *J* = 7.5, 3.3 Hz, 4H), 8.09 (d, *J* = 8.1 Hz, 2H), 7.76 (t, *J* = 7.2 Hz, 2H), 7.63 (t, *J* = 6.9 Hz, 2H), 4.37 (s, 1H), 4.18 (s, 1H), 2.97 (d, *J* = 14.6 Hz, 3H), 0.78 (s, 9H). **^{13}C NMR** (151 MHz, Dimethylsulfoxide-*d6*) δ 143.21, 137.24, 131.23 (d, *J* = 11.1 Hz), 129.07, 129.02, 128.44 (d, *J* = 10.8 Hz), 127.10, 126.92, 125.55 (d, *J* = 11.8 Hz), 35.19 (d, *J* = 41.5 Hz), 31.46 (d, *J* = 5.7 Hz), 30.30 (d, *J* = 8.1 Hz), 8.39 (d, *J* = 50.1 Hz). **^{31}P NMR** (243 MHz, Dimethylsulfoxide-*d6*) δ 31.12. **HRMS (ESI)** calcd for: $C_{26}H_{26}P^+$ [M – I$^-$]$^+$ 369.1767; found: 369.1765.

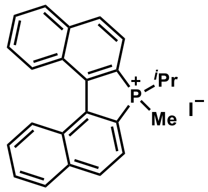

(**1f**) Yellow solid, 59% yield, M.p. 170-171 °C. **^{1}H NMR** (600 MHz, Chloroform-*d*) δ 8.65 (s, 2H), 8.31-8.15 (m, 4H), 8.11 (d, *J* = 8.2 Hz, 2H), 7.78 (t, *J* = 7.4 Hz, 2H), 7.65 (t, *J* = 7.6 Hz, 2H), 4.50-4.32 (m, 1H), 2.97 (d, *J* = 14.4 Hz, 3H), 1.46-1.06 (m, 6H). **^{13}C NMR** (151 MHz, Chloroform-*d*) δ 144.89 (d, *J* = 16.9 Hz), 137.97, 131.98, 129.30, 129.27, 129.03 (d, *J* = 9.8 Hz), 127.74, 126.94, 126.04 (d, *J* = 10.5 Hz), 119.75 (d, *J* = 86.0 Hz), 24.94 (d, *J* = 44.5 Hz), 15.66, 7.88 (d, *J* = 47.0 Hz). **^{31}P NMR** (243 MHz, Chloroform-*d*) δ 41.50. **HRMS (ESI)** calcd for: $C_{24}H_{22}P^+$ [M – I$^-$]$^+$ 341.1454; found: 341.1455.

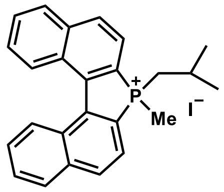

(**1g**) Yellow solid, 65% yield, M.p. 170 °C. **^{1}H NMR** (600 MHz, Chloroform-*d*) δ 8.81 (s, 2H), 8.30-8.17 (m, 4H), 8.10 (d, *J* = 8.2 Hz, 2H), 7.77 (t, *J* = 7.6 Hz, 2H), 7.64 (t, *J* = 7.6 Hz, 2H), 3.84 (br, 1H), 3.67 (br, 1H), 2.91 (d, *J* = 14.6 Hz, 3H), 1.77-1.67 (m, 1H), 0.99-0.60 (m, 6H). **^{13}C NMR** (151 MHz, Chloroform-*d*) δ 144.31 (d, *J* = 24.5 Hz), 137.91, 131.87 (d, *J* = 12.2 Hz), 129.27, 129.15, 129.09, 127.73, 126.92 (d, *J* = 12.7 Hz), 126.71, 32.04 (d, *J* = 43.4 Hz), 24.67 (d, *J* = 4.6 Hz), 23.65 (d, *J* = 10.3 Hz), 10.98 (d, *J* = 49.5 Hz). **^{31}P NMR** (243 MHz, Chloroform-*d*) δ 34.11. **HRMS (ESI)** calcd for: $C_{25}H_{24}P^+$ [M – I$^-$]$^+$ 355.1610; found: 355.1611.

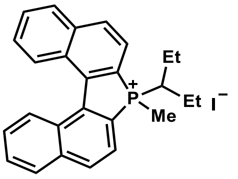

(**1h**) Yellow solid, 63% yield, M.p. 151-152 °C. **^{1}H NMR** (600 MHz, Chloroform-*d*) δ 8.65 (s, 2H), 8.40-8.17 (m, 4H), 8.11 (d, *J* = 8.0 Hz, 2H), 7.78 (t, *J* = 7.1 Hz, 2H), 7.65 (t, *J* = 7.0 Hz, 2H), 4.31-4.16 (m, 1H), 2.90 (d, *J* = 14.1 Hz, 3H), 2.10-1.55 (m, 4H), 0.94 (s, 6H). **^{13}C NMR** (151 MHz, Chloroform-*d*) δ 144.43 (d, *J* = 16.6 Hz), 137.78, 131.86, 129.22, 129.17, 129.00 (d, *J* = 10.9 Hz), 127.61, 126.87, 125.93, 36.27 (d, *J* = 40.5 Hz), 20.34, 11.78 (d, *J* = 10.8 Hz), 8.74 (d, *J* = 47.5 Hz). **^{31}P NMR** (243 MHz, Chloroform-*d*) δ 38.58. **HRMS (ESI)** calcd for: $C_{26}H_{26}P^+$ [M – I$^-$]$^+$ 369.1767; found: 369.1776.

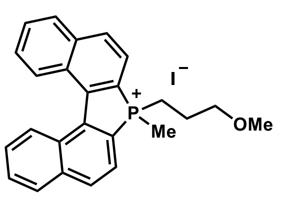

(**1i**) Yellow solid, 59% yield, M.p.123-124 °C. **^{1}H NMR** (600 MHz, Chloroform-*d*) δ 8.87 (t, *J* = 7.8 Hz, 2H), 8.23 (t, *J* = 7.0 Hz, 4H), 8.10 (d, *J* = 8.1 Hz, 2H), 7.76 (t, *J* = 6.9 Hz, 2H), 7.64 (t, *J* = 7.1 Hz, 2H), 3.86 (s, 1H), 3.73 (s, 1H), 3.34 (s, 2H), 3.10 (s, 3H), 3.02 (d, *J* = 14.8 Hz, 3H), 1.69 (s, 1H), 1.52 (s, 1H). **^{13}C NMR** (151 MHz, Chloroform-*d*) δ 144.45 (d, *J* = 18.1 Hz), 137.99 (d, *J* = 1.9 Hz), 131.92 (d, *J* = 12.4 Hz), 129.27, 129.18, 129.06 (d, *J* = 10.8 Hz), 127.82, 126.80, 126.44 (d, *J* = 11.6 Hz), 121.13 (d, *J* = 92.6 Hz), 70.82 (d, *J* = 14.5 Hz), 58.41, 22.52 (d, *J* = 4.3 Hz), 21.33 (d, *J* = 47.4 Hz), 10.26 (d, *J* = 49.4 Hz). **^{31}P NMR** (243 MHz, Chloroform-*d*) δ 36.29. **HRMS (ESI)** calcd for: $C_{25}H_{24}OP^+$ [M – I$^-$]$^+$ 371.1559; found: 371.1560.

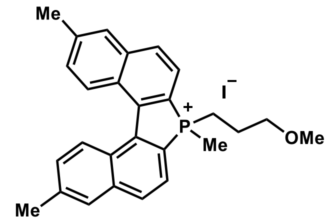

(**1j**) Yellow solid, 38% yield, M.p.136 °C. **^{1}H NMR** (600 MHz, Chloroform-*d*) δ 8.74 (s, 2H), 8.18-8.03 (m, 4H), 7.84 (s, 2H), 7.45 (d, *J* = 8.3 Hz, 2H), 4.02-3.50 (m, 2H), 3.48-3.24 (m, 2H), 3.11 (s, 3H), 2.95 (d, *J* = 14.8 Hz, 3H), 2.62 (s, 6H), 1.75-1.35 (m, 2H). **^{13}C NMR** (151 MHz, Chloroform-*d*) δ 144.52 (d, *J* = 18.1 Hz), 139.58, 138.40 (d, *J* = 2.0 Hz), 131.18 (d, *J* = 12.4 Hz), 129.06, 128.12, 127.74, 126.43 (d, *J* = 11.6 Hz), 119.89 (d, *J* = 90.5 Hz), 70.89 (d, *J* = 14.6 Hz), 58.48, 22.56 (d, *J* = 4.1 Hz), 21.82, 21.44 (d, *J* = 47.9 Hz), 10.32 (d, *J* = 49.7 Hz). **^{31}P NMR** (243 MHz, Chloroform-*d*) δ 35.54. **HRMS (ESI)** calcd for: $C_{27}H_{28}OP^+$ $[M – I^-]^+$ 399.1872; found: 399.1887.

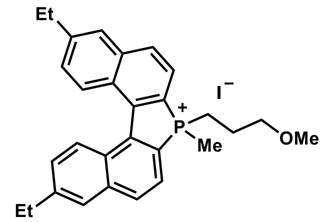

(**1k**) Yellow solid, 58% yield, M.p.124 °C. **^{1}H NMR** (600 MHz, Chloroform-*d*) δ 8.77 (t, *J* = 8.0 Hz, 2H), 8.19-8.10 (m, 4H), 7.85 (s, 2H), 7.50 (d, *J* = 8.7 Hz, 2H), 3.99-3.51 (m, 2H), 3.33 (t, *J* = 5.9 Hz, 2H), 3.11 (s, 3H), 3.02-2.88 (m, 7H), 1.81-1.35 (m, 2H), 1.42 (t, *J* = 7.6 Hz, 6H). **^{13}C NMR** (151 MHz, Chloroform-*d*) δ 145.65, 144.55 (d, *J* = 18.1 Hz), 138.52 (d, *J* = 1.9 Hz), 131.37 (d, *J* = 12.5 Hz), 128.05, 127.91, 126.80, 126.39 (d, *J* = 11.4 Hz), 119.93 (d, *J* = 89.4 Hz), 70.90 (d, *J* = 14.7 Hz), 58.49, 29.02, 22.54 (d, *J* = 4.2 Hz), 21.44 (d, *J* = 48.0 Hz), 15.01, 10.35 (d, *J* = 49.7 Hz). **^{31}P NMR** (243 MHz, Chloroform-*d*) δ 35.51. **HRMS (ESI)** calcd for: $C_{29}H_{32}OP^+$ $[M – I^-]^+$ 427.2185; found: 427.2200.

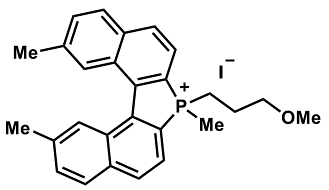

(**1l**) Yellow solid, 44% yield, M.p.128-129 °C. **^{1}H NMR** (600 MHz, Chloroform-*d*) δ 8.66 (s, 2H), 8.18-8.12 (m, 2H), 8.05 (s, 2H), 7.98 (d, *J* = 8.4 Hz, 2H), 7.60 (d, *J* = 8.3 Hz, 2H), 4.04-3.48 (m, 2H), 3.35 (t, *J* = 5.9 Hz, 2H), 3.15 (s, 3H), 2.91 (d, *J* = 14.7 Hz, 3H), 2.53 (s, 6H), 1.85-1.40 (m, 2H). **^{13}C NMR** (151 MHz, Chloroform-*d*) δ 143.96 (d, *J* = 18.2 Hz), 136.84, 136.53 (d, *J* = 1.8 Hz), 131.60 (d, *J* = 12.4 Hz), 131.50, 129.12, 127.35, 125.51 (d, *J* = 12.0 Hz), 70.96 (d, *J* = 14.5 Hz), 58.61, 22.69 (d, *J* = 4.3 Hz), 21.96, 21.50 (d, *J* = 47.8 Hz), 10.29 (d, *J* = 49.5 Hz). **^{31}P NMR** (243 MHz, Chloroform-*d*) δ 35.42. **HRMS (ESI)** calcd for: $C_{27}H_{28}OP^+$ $[M – I^-]^+$ 399.1872; found: 399.1886.

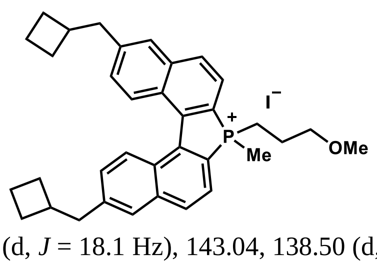

(**1m**) Yellow solid, 13% yield, M.p.110-111 °C. **^{1}H NMR** (600 MHz, Chloroform-*d*) δ 8.68 (t, *J* = 8.2 Hz, 2H), 8.17-8.06 (m, 4H), 7.78 (s, 2H), 7.43 (d, *J* = 8.8 Hz, 2H), 3.92-3.43 (m, 2H), 3.35 (t, *J* = 6.0 Hz, 2H), 3.14 (s, 3H), 2.96 (d, *J* = 7.5 Hz, 4H), 2.90 (d, *J* = 14.8 Hz, 3H), 2.80-2.68 (m, 2H), 2.20-2.07 (m, 4H), 2.01-1.87 (m, 4H), 1.87-1.76 (m, 4H), 1.75-1.45 (m, 2H). **^{13}C NMR** (151 MHz, Chloroform-*d*) δ 144.61 (d, *J* = 18.1 Hz), 143.04, 138.50 (d, *J* = 1.9 Hz), 131.47 (d, *J* = 12.4 Hz), 128.56, 127.87, 127.78 (d, *J* = 11.1 Hz), 127.67, 126.28 (d, *J* = 11.8 Hz), 120.01 (d, *J* = 91.1 Hz), 71.00 (d, *J* = 14.7 Hz), 58.61, 43.14, 36.90, 28.52, 22.71 (d, *J* = 4.0 Hz), 21.55 (d, *J* = 47.8 Hz), 18.54, 10.33 (d, *J* = 49.9 Hz). **^{31}P NMR** (243 MHz, Chloroform-*d*) δ 35.26. **HRMS (ESI)** calcd for: $C_{35}H_{40}OP^+$ $[M – I^-]^+$ 507.2811; found: 507.2821.

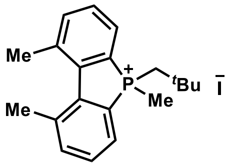

(**1n**) White solid, 22% yield, M.p.275-276 °C. **^{1}H NMR** (600 MHz, Chloroform-*d*) δ 8.76-8.65 (m, 2H), 7.61 (d, *J* = 7.5 Hz, 2H), 7.59-7.55 (m, 2H), 3.88 (d, *J* = 14.2 Hz, 2H), 2.73 (d, *J* = 14.5 Hz, 3H), 2.54 (s, 6H), 0.77 (s, 9H). **^{13}C NMR** (151 MHz, Chloroform-*d*) δ 143.72 (d, *J* = 17.8 Hz), 138.70 (d, *J* = 2.5 Hz), 136.00 (d, *J* = 9.6 Hz), 132.14 (d, *J* = 11.2 Hz), 129.80 (d, *J* = 13.4 Hz), 123.96 (d, *J* = 85.7 Hz), 37.97 (d, *J* = 41.8 Hz), 32.48 (d, *J* = 5.6 Hz), 31.03 (d, *J* = 8.2 Hz), 23.40, 12.72 (d, *J* = 52.0 Hz). **^{31}P NMR** (243 MHz, Chloroform-*d*) δ 27.49. **HRMS (ESI)** calcd for: $C_{20}H_{26}P^{+}$ $[M – I^{-}]^{+}$ 297.1767; found: 297.1770.

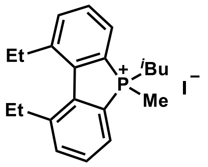

(**1o**) Yellow solid, 28% yield, M.p.191-192 °C. **^{1}H NMR** (600 MHz, Chloroform-*d*) δ 8.76-8.61 (m, 2H), 7.65 (d, *J* = 7.2 Hz, 2H), 7.58-7.49 (m, 2H), 3.59-3.48 (m, 2H), 2.86-2.68 (m, 7H), 1.62-1.52 (m, 1H), 1.18-1.08 (m, 6H), 0.64 (d, *J* = 3.8 Hz, 6H). **^{13}C NMR** (151 MHz, Chloroform-*d*) δ 143.19 (d, *J* = 17.3 Hz), 142.52 (d, *J* = 9.3 Hz), 136.09, 131.49 (d, *J* = 11.1 Hz), 129.96 (d, *J* = 13.3 Hz), 122.57 (d, *J* = 87.0 Hz), 32.85 (d, *J* = 44.8 Hz), 27.59, 24.43 (d, *J* = 4.5 Hz), 23.24 (d, *J* = 10.3 Hz), 15.26, 11.95 (d, *J* = 50.4 Hz). **^{31}P NMR** (243 MHz, Chloroform-*d*). δ 31.45. **HRMS (ESI)** calcd for: $C_{21}H_{28}P^{+}$ $[M – I^{-}]^{+}$ 311.1923; found: 311.1927.

3.2. Enantioselective alkynylation of C-P bond with terminal alkynes

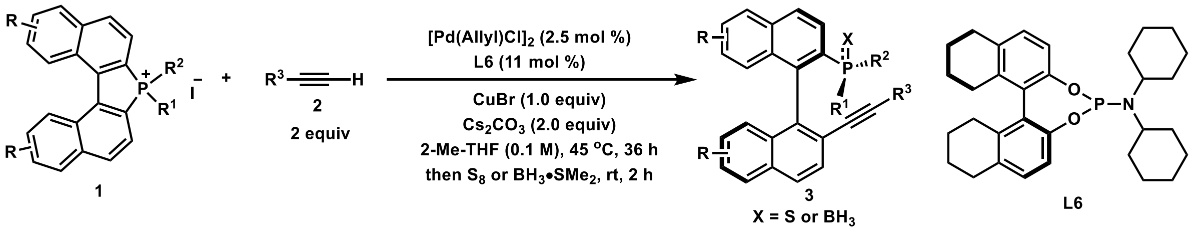

Typical Procedure: To a 10 mL Schlenk tube containing anhydrous Cs_2CO_3 (0.4 mmol, 2 equiv) was added phosphonium salts **1** (0.2 mmol), CuBr (0.2 mmol, 1 equiv), $[Pd(allyl)Cl]_2$ (0.005 mmol, 2.5 mol %), chiral phosphine ligand **L** (0.022 mmol, 11 mol %), 2-Me-THF (2 mL, 0.1 M), and alkyne (0.4 mmol, 2 equiv) sequentially under nitrogen. The Schlenk tube was then sealed and stirred for 36 h at 45 ℃. The reaction mixture was cooled to rt, S_8 (5 equiv, 1 mmol) or BH_3•SMe_2 (2 equiv, 10 M in Me_2S) was added and stirred for 2 h at rt. The reaction mixture was then filtered through a pad of celite eluenting with CH_2Cl_2/EtOAc (20 mL). The filtrate was concentrated, and the residue was purified by silica gel chromatography to afford the corresponding product.

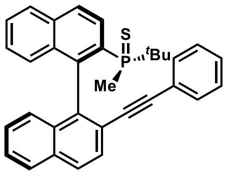

(**3a**) Brown solid, R_f = 0.24 (petroleum ether/ethyl acetate = 20:1), 83% yield, 93% ee, M.p. 95 °C, **^{1}H NMR** (600 MHz, Chloroform-*d*) δ 8.60 (dd, *J* = 11.1, 9.8 Hz, 1H), 8.06 (d, *J* = 8.8 Hz, 1H), 7.97 (d, *J* = 8.5 Hz, 1H), 7.93 (d, *J* = 8.1 Hz, 1H), 7.89 (d, *J* = 8.1 Hz, 1H), 7.73 (d, *J* = 8.5 Hz, 1H), 7.49 (dt, *J* = 14.9, 7.4 Hz, 2H), 7.40-7.33 (m, 1H), 7.30-7.25 (m, 1H), 7.24 (d, *J* = 8.4 Hz, 1H), 7.20 (d, *J* = 8.6 Hz, 1H), 7.17-7.12 (m, 1H), 7.08 (t, *J* = 7.4 Hz, 2H), 6.70 (d, *J* = 7.6 Hz, 2H), 1.18 (d, *J* = 16.5 Hz, 9H), 0.75 (d, *J* = 12.8 Hz, 3H). **^{13}C NMR** (151 MHz, Chloroform-*d*) δ 139.44 (d, *J* = 3.4 Hz), 139.19 (d, *J* = 4.9 Hz), 134.84, 134.33 (d, *J* = 2.1 Hz), 132.55, 132.41 (d, *J* = 10.5 Hz), 131.04, 130.81 (d, *J* = 12.6 Hz), 129.76 (d, *J* = 67.4 Hz), 128.98, 128.34, 128.17, 128.13, 128.10, 127.88, 127.87, 127.42, 127.39, 127.34, 127.29, 127.11,

126.36, 122.63, 122.32, 95.88, 89.84, 36.21 (d, J = 50.0 Hz), 25.68 (d, J = 2.0 Hz), 15.29 (d, J = 52.1 Hz). **^{31}P NMR** (243 MHz, Chloroform-*d*) δ 61.52. The enantiomeric excess was determined by Daicel Chiralpak ID, *n*-hexane/isopropanol = 90/10, 1 mL/min, λ = 254 nm, *t* (minor) = 14.43 min, *t* (major) = 16.45 min. $[\alpha]_D^{25}$ = 161.9 (c = 0.687, CH_2Cl_2). **HRMS (ESI)** calcd for: $C_{33}H_{30}PS^+$ $[M + H]^+$ 489.1800; found: 489.1807.

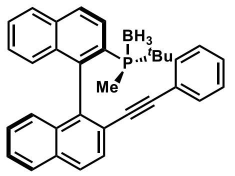

(**3b**) Yellow solid, R_f = 0.49 (petroleum ether/ethyl acetate = 20:1), 62% yield, M.p. 75 °C, **3b** was deprotected with DABCO, then reacted with S_8 in CH_2Cl_2 to afford **3a** (93% ee). **^{1}H NMR** (600 MHz, Chloroform-*d*) δ 8.09 (dd, J = 10.7, 9.2 Hz, 1H), 7.93 (d, J = 8.7 Hz, 1H), 7.87 (d, J = 8.5 Hz, 1H), 7.83 (d, J = 8.2 Hz, 1H), 7.79 (d, J = 8.2 Hz, 1H), 7.63 (d, J = 8.5 Hz, 1H), 7.43-7.34 (m, 2H), 7.23 (t, J = 7.6 Hz, 1H), 7.19-7.14 (m, 1H), 7.10 (d, J = 8.6 Hz, 1H), 7.07 (d, J = 8.7 Hz, 1H), 7.04 (d, J = 7.4 Hz, 1H), 6.99 (t, J = 7.5 Hz, 2H), 6.62 (d, J = 7.5 Hz, 2H), 1.03 (d, J = 13.8 Hz, 9H), 0.70-0.36 (m, 3H), 0.28 (d, J = 10.2 Hz, 3H). **^{13}C NMR** (151 MHz, Chloroform-*d*) δ 141.49, 139.58 (d, J = 3.0 Hz), 134.76, 134.15 (d, J = 1.9 Hz), 133.00 (d, J = 7.4 Hz), 132.65, 131.45, 131.34, 131.12, 128.92, 128.31, 128.20, 128.14, 128.05, 127.90, 127.77, 127.72, 127.31, 127.22, 127.04, 126.98, 126.51, 122.80, 122.63, 95.69, 89.98, 30.86 (d, J = 31.8 Hz), 26.71 (d, J = 2.4 Hz), 5.97 (d, J = 36.2 Hz). **^{31}P NMR** (243 MHz, Chloroform-*d*) δ 33.88. The enantiomeric excess was determined by Daicel Chiralpak ID, *n*-hexane/isopropanol = 90/10, 1 mL/min, λ = 254 nm, *t* (minor) = 13.10 min, *t* (major) = 15.03 min. $[\alpha]_D^{25}$ = 175.0 (c = 0.468, CH_2Cl_2). **HRMS (ESI)** calcd for: $C_{33}H_{32}BNaP^+$ $[M + Na]^+$ 493.2227; found: 493.2244.

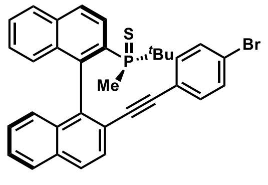

(**3c**) Yellow solid, R_f = 0.41 (petroleum ether/ethyl acetate = 20:1), 80% yield, 92% ee, M.p. 123-125 °C, **^{1}H NMR** (600 MHz, Chloroform-*d*) δ 8.59 (dd, J = 12.1, 8.9 Hz, 1H), 8.07 (d, J = 8.7 Hz, 1H), 7.98 (d, J = 8.5 Hz, 1H), 7.94 (d, J = 8.1 Hz, 1H), 7.91 (d, J = 8.1 Hz, 1H), 7.71 (d, J = 8.5 Hz, 1H), 7.55-7.48 (m, 2H), 7.39 (t, J = 7.6 Hz, 1H), 7.30-7.26 (m, 1H), 7.26-7.21 (m, 3H), 7.18 (d, J = 8.6 Hz, 1H), 6.51 (d, J = 8.4 Hz, 2H), 1.16 (d, J = 16.5 Hz, 9H), 0.73 (d, J = 12.9 Hz, 3H). **^{13}C NMR** (151 MHz, Chloroform-*d*) δ 139.77 (d, J = 3.4 Hz), 139.09 (d, J = 4.9 Hz), 134.86, 134.34 (d, J = 2.4 Hz), 132.69, 132.41, 131.57, 130.92, 130.84, 129.82 (d, J = 67.4 Hz), 129.11, 128.28, 128.22, 127.99, 127.94, 127.91, 127.53, 127.44, 127.40, 127.20, 126.43, 122.72, 121.96, 121.57, 94.76, 91.04, 36.26 (d, J = 50.0 Hz), 25.69 (d, J = 2.2 Hz), 15.32 (d, J = 52.1 Hz). **^{31}P NMR** (243 MHz, Chloroform-*d*) δ 61.41. The enantiomeric excess was determined by Daicel Chiralpak IA, *n*-hexane/isopropanol = 95/5, 1 mL/min, λ = 254 nm, *t* (minor) = 12.00 min, *t* (major) = 19.68 min. $[\alpha]_D^{25}$ = 135.9 (c = 0.788, CH_2Cl_2). **HRMS (ESI)** calcd for: $C_{33}H_{29}{}^{79}BrPS^+$ $[M + H]^+$ 567.0905; found: 567.0914.

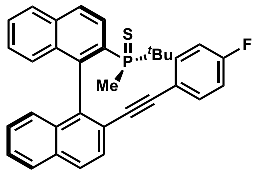

(**3d**) Yellow solid, R_f = 0.39 (petroleum ether/ethyl acetate = 20:1), 71% yield, 92% ee, M.p. 197-198 °C. **^{1}H NMR**(600 MHz, Chloroform-*d*) δ 8.60 (dd, J = 12.1, 8.9 Hz, 1H), 8.07 (d, J = 8.6 Hz, 1H), 7.99 (d, J = 8.5 Hz, 1H), 7.95 (d, J = 8.0 Hz, 1H), 7.91 (d, J = 8.2 Hz, 1H), 7.72 (d, J = 8.5 Hz, 1H), 7.57-7.49 (m, 2H), 7.39 (t, J = 7.5 Hz, 1H), 7.33-7.27 (m, 1H), 7.24 (d, J = 8.5 Hz, 1H), 7.19 (d, J = 8.6 Hz, 1H), 6.80 (t, J = 8.6 Hz, 2H), 6.65 (dd, J = 8.5, 5.5 Hz, 2H), 1.17 (d, J = 16.5 Hz, 9H), 0.73 (d, J = 12.9 Hz, 3H). **^{13}C NMR** (151 MHz, Chloroform-*d*) δ 162.57 (d, J = 250.2 Hz), 139.55 (d, J = 3.3 Hz), 139.22 (d, J = 4.9 Hz), 134.91, 134.39 (d, J = 2.3 Hz), 132.97 (d, J = 8.4 Hz), 132.64, 132.44 (d, J = 10.5 Hz), 130.92 (d, J = 12.5 Hz), 129.10, 128.27, 128.22, 128.02, 127.99, 127.96, 127.52, 127.48, 127.45, 127.44, 127.21, 126.44, 122.18, 118.79 (d, J = 3.4 Hz), 115.63 (d, J = 22.1 Hz), 94.82, 89.64, 36.28 (d, J = 49.9 Hz), 25.73 (d, J = 2.2 Hz), 15.33 (d, J = 52.1 Hz). **^{19}F**

NMR (564 MHz, Chloroform-*d*) δ -110.36. **^{31}P NMR** (243 MHz, Chloroform-*d*) δ 61.45. The enantiomeric excess was determined by Daicel Chiralpak IA, *n*-hexane/isopropanol = 90/10, 1 mL/min, λ = 254 nm, *t* (minor) = 8.24 min, *t* (major) = 10.70 min. $[\alpha]_D^{25}$ = 182.7 (c = 0.513, CH_2Cl_2). **HRMS (ESI)** calcd for: $C_{33}H_{29}FPS^+$ $[M + H]^+$ 507.1706; found: 507.1714.

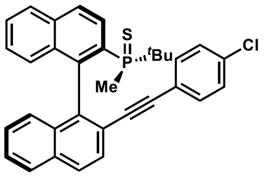

(**3e**) Yellow solid, R_f = 0.30 (petroleum ether/ethyl acetate/dichloromethane = 20:1), 85% yield, 92% ee, M.p. 106-107 °C. **^{1}H NMR** (600 MHz, Chloroform-*d*) δ 8.59 (dd, *J* = 12.0, 9.0 Hz, 1H), 8.07 (d, *J* = 8.8 Hz, 1H), 7.99 (d, *J* = 8.5 Hz, 1H), 7.95 (d, *J* = 8.1 Hz, 1H), 7.92 (d, *J* = 8.1 Hz, 1H), 7.72 (d, *J* = 8.5 Hz, 1H), 7.57-7.48 (m, 2H), 7.40 (t, *J* = 7.5 Hz, 1H), 7.29 (t, *J* = 7.5 Hz, 1H), 7.25 (d, *J* = 9.8 Hz, 1H), 7.18 (d, *J* = 8.6 Hz, 1H), 7.08 (d, *J* = 8.4 Hz, 2H), 6.59 (d, *J* = 8.4 Hz, 2H), 1.17 (d, *J* = 16.5 Hz, 9H), 0.73 (d, *J* = 12.9 Hz, 3H). **^{13}C NMR** (151 MHz, Chloroform-*d*) δ 139.78 (d, *J* = 3.5 Hz), 139.13 (d, *J* = 4.9 Hz), 134.90, 134.53, 134.39 (d, *J* = 2.2 Hz), 132.72, 132.42 (d, *J* = 10.5 Hz), 132.25, 130.96, 130.88, 130.09, 129.65, 129.13, 128.66, 128.30, 128.23, 128.00, 127.96, 127.54, 127.44, 127.22, 126.48, 122.02, 121.16, 94.73, 90.89, 36.29 (d, *J* = 50.0 Hz), 25.73 (d, *J* = 2.2 Hz), 15.35 (d, *J* = 52.1 Hz). **^{31}P NMR** (243 MHz, Chloroform-*d*) δ 61.42. The enantiomeric excess was determined by Daicel Chiralpak IA, *n*-hexane/isopropanol = 90/10, 1 mL/min, λ = 254 nm, *t* (minor) = 8.54 min, *t* (major) = 12.24 min. $[\alpha]_D^{25}$ = 158.1 (c = 0.704, CH_2Cl_2). **HRMS (ESI)** calcd for: $C_{33}H_{29}ClPS^+$ $[M + H]^+$ 523.1411; found: 523.1415.

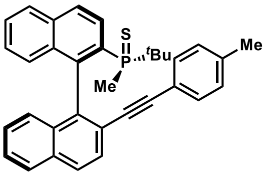

(**3f**) Yellow solid, R_f = 0.45 (petroleum ether/ethyl acetate = 20:1), 97% yield, 91% ee, M.p. 96-98 °C. **^{1}H NMR** (600 MHz, Chloroform-*d*) δ 8.59 (dd, *J* = 12.1, 8.9 Hz, 1H), 8.06 (d, *J* = 8.8 Hz, 1H), 7.98 (d, *J* = 8.5 Hz, 1H), 7.94 (d, *J* = 8.0 Hz, 1H), 7.91 (d, *J* = 8.2 Hz, 1H), 7.72 (d, *J* = 8.5 Hz, 1H), 7.56-7.46 (m, 2H), 7.38 (t, *J* = 7.6 Hz, 1H), 7.34-7.27 (m, 1H), 7.23 (d, *J* = 8.5 Hz, 1H), 7.20 (d, *J* = 8.6 Hz, 1H), 6.91 (d, *J* = 7.9 Hz, 2H), 6.59 (d, *J* = 8.0 Hz, 2H), 2.24 (s, 3H), 1.18 (d, *J* = 16.5 Hz, 9H), 0.74 (d, *J* = 12.9 Hz, 3H). **^{13}C NMR** (151 MHz, Chloroform-*d*) δ 139.33 (d, *J* = 5.0 Hz), 139.28 (d, *J* = 3.4 Hz), 138.62, 134.92, 134.39 (d, *J* = 2.3 Hz), 132.50, 132.43, 131.01, 130.90, 130.81, 129.93, 129.49, 129.01, 128.98, 128.16, 127.91 (d, *J* = 2.6 Hz), 127.50, 127.44, 127.36, 127.25, 127.13, 126.38, 122.57, 119.63, 96.21, 89.31, 36.26 (d, *J* = 49.9 Hz), 25.72 (d, *J* = 2.2 Hz), 21.49, 15.29 (d, *J* = 52.2 Hz). **^{31}P NMR** (243 MHz, Chloroform-*d*) δ 61.52. The enantiomeric excess was determined by Daicel Chiralpak IA, *n*-hexane/isopropanol = 90/10, 1 mL/min, λ = 254 nm, *t* (minor) = 8.45 min, *t* (major) = 14.00 min. $[\alpha]_D^{25}$ = 161.8 (c = 0.484, CH_2Cl_2). **HRMS (ESI)** calcd for: $C_{34}H_{32}PS^+$ $[M + H]^+$ 503.1957; found: 503.1961.

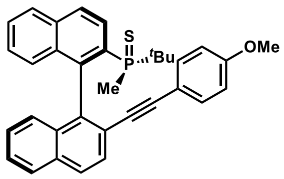

(**3g**) Yellow solid, R_f = 0.15 (petroleum ether/ethyl acetate = 20:1), 96% yield, 92% ee, M.p. 89-90 °C. **^{1}H NMR** (600 MHz, Chloroform-*d*) δ 8.59 (dd, *J* = 11.9, 9.1 Hz, 1H), 8.06 (d, *J* = 8.9 Hz, 1H), 7.97 (d, *J* = 8.5 Hz, 1H), 7.94 (d, *J* = 8.2 Hz, 1H), 7.90 (d, *J* = 8.2 Hz, 1H), 7.71 (d, *J* = 8.5 Hz, 1H), 7.52 (t, *J* = 7.4 Hz, 1H), 7.49 (t, *J* = 7.5 Hz, 1H), 7.38 (t, *J* = 7.6 Hz, 1H), 7.29 (t, *J* = 7.6 Hz, 1H), 7.25-7.17 (m, 2H), 6.63 (s, 4H), 3.72 (s, 3H), 1.18 (d, *J* = 16.5 Hz, 9H), 0.74 (d, *J* = 12.9 Hz, 3H). **^{13}C NMR** (151 MHz, Chloroform-*d*) δ 159.80, 139.43 (d, *J* = 5.0 Hz), 139.01 (d, *J* = 3.4 Hz), 134.94, 134.39 (d, *J* = 2.2 Hz), 132.59, 132.49 (d, *J* = 10.5 Hz), 132.43, 130.88 (d, *J* = 12.7 Hz), 129.72 (d, *J* = 67.6 Hz), 128.97, 128.17, 128.14, 128.11, 127.92, 127.90, 127.54, 127.37 (d, *J* = 12.2 Hz), 127.17, 127.13, 126.34,

122.73, 114.82, 113.97, 96.16, 88.76, 55.31, 36.26 (d, *J* = 49.9 Hz), 25.74 (d, *J* = 2.2 Hz), 15.30 (d, *J* = 52.1 Hz). **^{31}P NMR** (243 MHz, Chloroform-*d*) δ 61.53. The enantiomeric excess was determined by Daicel Chiralpak IA, *n*-hexane/isopropanol = 90/10, 1 mL/min, λ = 254 nm, *t* (minor) = 8.72 min, *t* (major) = 13.41 min. $[\alpha]_D^{25}$ = 161.9 (c = 0.902, CH_2Cl_2). **HRMS (ESI)** calcd for: $C_{34}H_{32}OPS^+$ $[M + H]^+$ 519.1906; found: 519.1922.

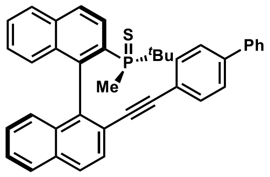

(**3h**) Light yellow solid, R_f = 0.27 (petroleum ether/ethyl acetate = 20:1), 79% yield, 91% ee, M.p. 191 °C. **^{1}H NMR** (600 MHz, Chloroform-*d*) δ 8.61 (dd, *J* = 12.1, 9.0 Hz, 1H), 8.09 (d, *J* = 8.8 Hz, 1H), 8.00 (d, *J* = 8.5 Hz, 1H), 7.96 (d, *J* = 8.2 Hz, 1H), 7.92 (d, *J* = 8.1 Hz, 1H), 7.75 (d, *J* = 8.5 Hz, 1H), 7.57-7.45 (m, 4H), 7.39 (t, *J* = 7.5 Hz, 3H), 7.37-7.28 (m, 4H), 7.25 (d, *J* = 7.3 Hz, 1H), 7.22 (d, *J* = 8.6 Hz, 1H), 6.76 (d, *J* = 8.1 Hz, 2H), 1.20 (d, *J* = 16.5 Hz, 9H), 0.75 (d, *J* = 12.9 Hz, 3H). **^{13}C NMR** (151 MHz, Chloroform-*d*) δ 141.10, 140.09, 139.44 (d, *J* = 3.3 Hz), 139.20 (d, *J* = 4.8 Hz), 134.85, 134.33 (d, *J* = 2.2 Hz), 132.53, 132.41 (d, *J* = 10.5 Hz), 131.46, 130.84 (d, *J* = 12.6 Hz), 129.74 (d, *J* = 67.4 Hz), 129.01, 128.86, 128.16, 128.15, 128.08, 127.91 (d, *J* = 5.4 Hz), 127.73, 127.42, 127.41 (d, *J* = 12.1 Hz),127.32, 127.15, 126.90, 126.86, 126.34, 122.32, 121.46, 95.86, 90.57, 36.23 (d, *J* = 49.9 Hz), 25.68 (d, *J* = 2.1 Hz), 15.28 (d, *J* = 52.1 Hz). **^{31}P NMR** (243 MHz, Chloroform-*d*) δ 61.55. The enantiomeric excess was determined by Daicel Chiralpak IA, *n*-hexane/isopropanol = 90/10, 1 mL/min, λ = 254 nm, *t* (minor) = 10.57 min, *t* (major) = 15.23 min. $[\alpha]_D^{25}$ = 138.1 (c = 0.823, CH_2Cl_2). **HRMS (ESI)** calcd for: $C_{39}H_{34}PS^+$ $[M + H]^+$ 565.2113; found: 565.2107.

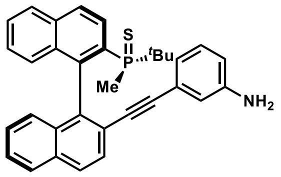

(**3i**) White solid, R_f = 0.35 (petroleum ether/ethyl acetate = 5:1), 74% yield, 90% ee, M.p. 88-89 °C. **^{1}H NMR** (600 MHz, Chloroform-*d*) δ 8.60 (dd, *J* = 11.8, 9.2 Hz, 1H), 8.06 (d, *J* = 8.8 Hz, 1H), 7.98 (d, *J* = 8.5 Hz, 1H), 7.94 (d, *J* = 8.2 Hz, 1H), 7.90 (d, *J* = 8.1 Hz, 1H), 7.72 (d, *J* = 8.4 Hz, 1H), 7.53 (t, *J* = 7.4 Hz, 1H), 7.49 (t, *J* = 7.4 Hz, 1H), 7.38 (t, *J* = 7.6 Hz, 1H), 7.29 (t, *J* = 7.6 Hz, 1H), 7.22 (d, *J* = 9.3 Hz, 1H), 7.20 (d, *J* = 8.9 Hz, 1H), 6.88 (t, *J* = 7.8 Hz, 1H), 6.48 (d, *J* = 7.7 Hz, 1H), 6.13 (d, *J* = 7.5 Hz, 1H), 5.97 (s, 1H), 3.50 (s, 2H), 1.18 (d, *J* = 16.5 Hz, 9H), 0.73 (d, *J* = 12.9 Hz, 3H). **^{13}C NMR** (151 MHz, Chloroform-*d*) δ 146.13, 139.32 (d, *J* = 3.5 Hz), 139.28 (d, *J* = 5.0 Hz), 134.89, 134.38 (d, *J* = 2.3 Hz), 132.52, 132.43 (d, *J* = 10.5 Hz), 130.85 (d, *J* = 12.6 Hz), 129.72 (d, *J* = 67.6 Hz), 129.09, 128.98, 128.21, 128.16, 128.14, 127.92, 127.86, 127.53, 127.39 (d, *J* = 12.2 Hz), 127.28, 127.16, 126.35, 123.32, 122.46, 121.48, 117.29, 115.45, 96.33, 89.23, 36.25 (d, *J* = 49.9 Hz), 25.70 (d, *J* = 2.2 Hz), 15.26 (d, *J* = 52.2 Hz). **^{31}P NMR** (243 MHz, Chloroform-*d*) δ 61.55. The enantiomeric excess was determined by Daicel Chiralpak IA, *n*-hexane/isopropanol = 70/30, 1 mL/min, λ = 254 nm, *t* (minor) = 8.93, *t* (major) = 10.43, $[\alpha]_D^{25}$ = 151.6 (c = 0.670, CH_2Cl_2). **HRMS (ESI)** calcd for: $C_{33}H_{31}NPS^+$ $[M + H]^+$ 504.1909; found: 504.1915.

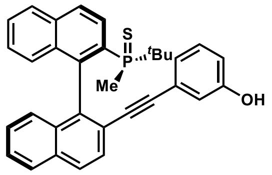

(**3j**) White solid, R_f = 0.37 (petroleum ether/ethyl acetate = 3:1), 65% yield, 93% ee, M.p. 220 °C. **^{1}H NMR** (600 MHz, Chloroform-*d*) δ 8.58 (dd, *J* = 11.7, 9.3 Hz, 1H), 8.05 (d, *J* = 8.8 Hz, 1H), 7.98 (d, *J* = 8.5 Hz, 1H), 7.93 (d, *J* = 8.2 Hz, 1H), 7.90 (d, *J* = 8.3 Hz, 1H), 7.72 (d, *J* = 8.5 Hz, 1H), 7.52 (t, *J* = 7.4 Hz, 1H), 7.48 (t, *J* = 7.5 Hz, 1H), 7.34 (t, *J* = 7.6 Hz, 1H), 7.29 (t, *J* = 7.6 Hz, 1H), 7.21 (d, *J* = 8.5 Hz, 1H), 7.19 (d, *J* = 8.7 Hz, 1H), 6.94 (t, *J* = 7.8 Hz, 1H), 6.65 (d, *J* = 8.1 Hz, 1H), 6.27 (d, *J* = 7.6 Hz, 1H), 6.17 (s, 1H), 5.09 (s, 1H), 1.18 (d, *J* = 16.6 Hz, 9H), 0.73 (d, *J* = 12.8 Hz, 3H). **^{13}C NMR** (151 MHz, Chloroform-*d*) δ 155.65, 139.41 (d, *J* = 3.1 Hz), 139.38 (d, *J* = 4.6 Hz), 134.87,

134.42 (d, J = 2.3 Hz), 132.60, 132.47 (d, J = 10.5 Hz), 130.73 (d, J = 12.6 Hz), 129.40 (d, J = 67.8 Hz), 129.36, 129.05, 128.23, 128.22, 128.15, 128.03, 127.99, 127.56 (d, J = 12.2 Hz), 127.45, 127.38, 127.23, 126.40, 123.72, 123.56, 122.33, 117.86, 116.13, 95.76, 89.64, 36.31 (d, J = 49.8 Hz), 25.72 (d, J = 2.2 Hz), 15.16 (d, J = 52.0 Hz). **^{31}P NMR** (243 MHz, Chloroform-*d*) δ 61.67. The enantiomeric excess was determined by Daicel Chiralpak IA, *n*-hexane/isopropanol = 90/10, 1 mL/min, λ = 254 nm, *t* (minor) = 18.32 min, *t* (major) = 23.02 min. $[\alpha]_D^{25}$ = 166.9 (c = 0.461, CH_2Cl_2). **HRMS (ESI)** calcd for: $C_{33}H_{30}OPS^+$ $[M + H]^+$ 505.1749; found: 505.1754.

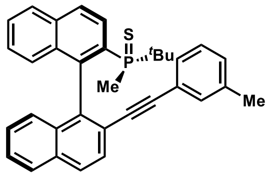

(**3k**) Light yellow solid, R_f = 0.29 (petroleum ether/ethyl acetate = 20:1), 69% yield, 90% ee, M.p. 83-84 °C. **^{1}H NMR** (600 MHz, Chloroform-*d*) δ 8.60 (dd, J = 12.1, 9.0 Hz, 1H), 8.07 (d, J = 8.8 Hz, 1H), 7.98 (d, J = 8.5 Hz, 1H), 7.94 (d, J = 8.2 Hz, 1H), 7.91 (d, J = 8.2 Hz, 1H), 7.72 (d, J = 8.5 Hz, 1H), 7.56-7.51 (m, 1H), 7.51-7.47 (m, 1H), 7.39 (t, J = 7.6 Hz, 1H), 7.29 (t, J = 7.6 Hz, 1H), 7.24 (d, J = 8.7 Hz, 1H), 7.20 (d, J = 8.6 Hz, 1H), 7.02-6.93 (m, 2H), 6.54-6.42 (m, 2H), 2.17 (s, 3H), 1.18 (d, J = 16.5 Hz, 9H), 0.73 (d, J = 12.9 Hz, 3H). **^{13}C NMR** (151 MHz, Chloroform-*d*) δ 139.50 (d, J = 3.5 Hz), 139.33 (d, J = 5.0 Hz), 137.90, 134.97, 134.48 (d, J = 2.3 Hz), 132.60, 132.51 (d, J = 10.5 Hz), 131.83, 130.94 (d, J = 12.7 Hz), 129.82 (d, J = 67.4 Hz), 129.30, 129.02, 128.19, 128.17, 128.12, 128.11, 127.94, 127.93, 127.55, 127.46, 127.38, 127.34, 127.18, 126.46, 122.54 (d, J = 3.2 Hz), 96.31, 89.66, 36.30 (d, J = 50.0 Hz), 25.75 (d, J = 2.2 Hz), 21.17, 15.35 (d, J = 52.1 Hz). **^{31}P NMR** (243 MHz, Chloroform-*d*) δ 61.54. The enantiomeric excess was determined by Daicel Chiralpak IA, *n*-hexane/isopropanol = 90/10, 1 mL/min, λ = 254 nm, *t* (minor) = 7.85 min, *t* (major) = 9.86 min. $[\alpha]_D^{25}$ = 157.0 (c = 0.513, CH_2Cl_2). **HRMS (ESI)** calcd for: $C_{34}H_{32}PS^+$ $[M + H]^+$ 503.1957; found: 503.1964.

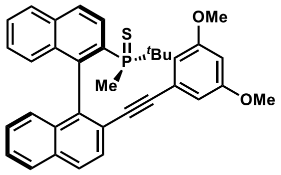

(**3l**) Yellow solid, R_f = 0.15 (petroleum ether/ethyl acetate = 20:1), 81% yield, 92% ee, M.p. 82-84 °C. **^{1}H NMR** (600 MHz, Chloroform-*d*) δ 8.60 (dd, J = 12.1, 9.0 Hz, 1H), 8.05 (d, J = 8.8 Hz, 1H), 7.99 (d, J = 8.5 Hz, 1H), 7.96-7.88 (m, 2H), 7.72 (d, J = 8.5 Hz, 1H), 7.58-7.46 (m, 2H), 7.40 (t, J = 7.6 Hz, 1H), 7.33-7.28 (m, 1H), 7.25 (d, J = 6.7 Hz, 1H), 7.21 (d, J = 8.6 Hz, 1H), 6.28 (s, 1H), 5.79 (d, J = 1.9 Hz, 2H), 3.61 (s, 6H), 1.18 (d, J = 16.5 Hz, 9H), 0.73 (d, J = 12.9 Hz, 3H). **^{13}C NMR** (151 MHz, Chloroform-*d*) δ 160.35, 139.71 (d, J = 3.4 Hz), 139.26 (d, J = 4.9 Hz), 134.84, 134.31 (d, J = 2.2 Hz), 132.58, 132.44 (d, J = 10.4 Hz), 130.94 (d, J = 12.7 Hz), 129.81 (d, J = 67.3 Hz), 129.04, 128.19, 128.16, 127.95, 127.89, 127.80, 127.50, 127.37, 127.24 (d, J = 12.2 Hz), 127.17, 126.32, 123.85, 122.16, 108.72, 102.04, 96.04, 89.45, 55.33, 36.24 (d, J = 49.9 Hz), 25.66 (d, J = 2.2 Hz), 15.27 (d, J = 52.1 Hz). **^{31}P NMR** (243 MHz, Chloroform-*d*) δ 61.52. The enantiomeric excess was determined by Daicel Chiralpak IA, *n*-hexane/isopropanol = 90/10, 1 mL/min, λ = 254 nm, *t* (minor) = 11.31 min, *t* (major) = 12.53 min. $[\alpha]_D^{25}$ = 166.7 (c = 0.752, CH_2Cl_2). **HRMS (ESI)** calcd for: $C_{35}H_{34}O_2PS^+$ $[M + H]^+$ 549.2012; found: 549.2026.

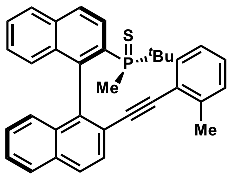

(**3m**) White solid, R_f = 0.34 (petroleum ether/ethyl acetate = 20:1), 91% yield, 93% ee, M.p. 109-110 °C. **^{1}H NMR** (600 MHz, Chloroform-*d*) δ 8.60 (dd, J = 12.1, 8.9 Hz, 1H), 8.04 (d, J = 8.8 Hz, 1H), 7.99 (d, J = 8.5 Hz, 1H), 7.92 (t, J = 8.0 Hz, 2H), 7.76 (d, J = 8.5 Hz, 1H), 7.56-7.45 (m, 2H), 7.38 (t, J = 7.6 Hz, 1H), 7.29 (t, J = 7.6 Hz, 1H), 7.21 (t, J = 8.1 Hz, 2H), 7.07 (t, J = 6.6 Hz, 1H), 7.02-6.90 (m, 3H), 1.38 (s, 3H), 1.17 (d, J = 16.5 Hz, 9H), 0.73 (d, J = 12.9 Hz, 3H). **^{13}C NMR** (151 MHz, Chloroform-*d*) δ 139.89, 139.24 (d, J = 4.9 Hz), 138.79 (d, J = 3.4 Hz), 134.85, 134.52 (d, J = 2.3 Hz), 132.47

(d, J = 10.3 Hz), 132.45, 131.65, 131.06 (d, J = 12.7 Hz), 129.63 (d, J = 67.2 Hz), 129.26, 128.95, 128.48, 128.43, 128.11, 128.10, 127.88, 127.85, 127.42, 127.35 (d, J = 12.2 Hz), 127.23, 127.20, 126.30, 125.37, 122.53, 122.36, 94.58, 93.38, 36.17 (d, J = 49.8 Hz), 25.62 (d, J = 2.2 Hz), 19.49, 15.17 (d, J = 52.2 Hz). **^{31}P NMR** (243 MHz, Chloroform-*d*) δ 61.69. The enantiomeric excess was determined by Daicel Chiralpak ID, *n*-hexane/isopropanol = 90/10, 1 mL/min, λ = 254 nm, *t* (minor) = 12.68 min, *t* (major) = 16.87 min. $[\alpha]_D^{25}$ = 204.0 (c = 0.707, CH_2Cl_2). **HRMS (ESI)** calcd for: $C_{34}H_{32}PS^+$ [M + H]$^+$ 503.1957; found: 503.1961.

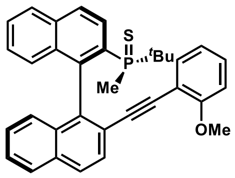

(**3n**) Yellow solid, R_f = 0.14 (petroleum ether/ethyl acetate = 20:1), 97% yield, 94% ee, M.p. 126-127 °C. **^{1}H NMR** (600 MHz, Chloroform-*d*) δ 8.61 (dd, J = 12.1, 8.9 Hz, 1H), 8.06 (d, J = 8.8 Hz, 1H), 7.97 (d, J = 8.5 Hz, 1H), 7.92 (d, J = 8.2 Hz, 1H), 7.89 (d, J = 8.1 Hz, 1H), 7.78 (d, J = 8.5 Hz, 1H), 7.56-7.44 (m, 2H), 7.36 (t, J = 7.6 Hz, 1H), 7.30-7.25 (m, 1H), 7.22 (d, J = 8.6 Hz, 1H), 7.19 (d, J = 8.5 Hz, 1H), 7.16-7.08 (m, 1H), 6.72-6.62 (m, 2H), 6.59 (dd, J = 7.5, 1.6 Hz, 1H), 3.51 (s, 3H), 1.18 (d, J = 16.6 Hz, 9H), 0.75 (d, J = 12.9 Hz, 3H). **^{13}C NMR** (151 MHz, Chloroform-*d*) δ 159.78, 139.36 (d, J = 4.9 Hz), 138.94 (d, J = 3.4 Hz), 134.88, 134.36 (d, J = 2.2 Hz), 133.11, 132.57 (d, J = 10.5 Hz), 132.44, 130.81 (d, J = 12.7 Hz), 129.82, 129.68 (d, J = 67.7 Hz), 128.85, 128.47, 128.10, 128.02, 127.81, 127.72, 127.55, 127.24 (d, J = 12.2 Hz), 127.13, 127.04, 126.30, 122.74, 120.25, 112.09, 110.77, 93.59, 92.49, 55.43, 36.19 (d, J = 50.0 Hz), 25.67 (d, J = 2.3 Hz), 15.20 (d, J = 52.1 Hz). **^{31}P NMR** (243 MHz, Chloroform-*d*) δ 61.66. The enantiomeric excess was determined by Daicel Chiralpak IA, *n*-hexane/isopropanol = 90/10, 1 mL/min, λ = 254 nm, *t* (minor) = 8.88 min, *t* (major) = 12.75 min. $[\alpha]_D^{25}$ = 151.2 (c = 0.927, CH_2Cl_2). **HRMS (ESI)** calcd for: $C_{34}H_{32}OPS^+$ [M + H]$^+$ 519.1906; found: 519.1904.

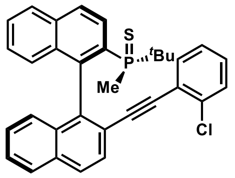

(**3o**) Yellow solid, R_f = 0.30 (petroleum ether/ethyl acetate = 20:1), 93% yield, 92% ee, M.p. 102 °C. **^{1}H NMR** (600 MHz, Chloroform-*d*) δ 8.59 (dd, J = 12.1, 8.9 Hz, 1H), 8.05 (d, J = 8.9 Hz, 1H), 8.00 (d, J = 8.5 Hz, 1H), 7.92 (t, J = 8.3 Hz, 2H), 7.78 (d, J = 8.5 Hz, 1H), 7.56-7.48 (m, 2H), 7.42-7.36 (m, 1H), 7.32-7.27 (m, 1H), 7.23 (d, J = 8.5 Hz, 1H), 7.19 (d, J = 8.6 Hz, 1H), 7.15 (dd, J = 8.0, 1.0 Hz, 1H), 7.09 (td, J = 7.8, 1.6 Hz, 1H), 7.02 (td, J = 7.6, 1.2 Hz, 1H), 6.87 (dd, J = 7.7, 1.5 Hz, 1H), 1.18 (d, J = 16.6 Hz, 9H), 0.73 (d, J = 12.9 Hz, 3H). **^{13}C NMR** (151 MHz, Chloroform-*d*) δ 139.65 (d, J = 3.4 Hz), 138.96 (d, J = 4.8 Hz), 135.48, 134.86, 134.61 (d, J = 2.3 Hz), 132.90, 132.71, 132.59 (d, J = 10.5 Hz), 130.98 (d, J = 12.6 Hz), 129.67 (d, J = 67.4 Hz), 129.34, 129.12, 129.01, 128.37, 128.18, 128.15, 127.86, 127.82, 127.50, 127.48, 127.40, 127.16, 126.45, 126.24, 122.64, 121.92, 94.53, 92.13, 36.27 (d, J = 49.9 Hz), 25.65 (d, J = 2.2 Hz), 15.23 (d, J = 52.1 Hz). **^{31}P NMR** (243 MHz, Chloroform-*d*) δ 61.59. The enantiomeric excess was determined by Daicel Chiralpak ID, *n*-hexane/isopropanol = 90/10, 1 mL/min, λ = 254 nm, *t* (minor) = 14.90 min, *t* (major) = 19.00 min. $[\alpha]_D^{25}$ = 171.3 (c = 0.890, CH_2Cl_2). **HRMS (ESI)** calcd for: $C_{33}H_{29}{}^{35}ClPS^+$ [M + H]$^+$ 523.1411; found: 523.1422.

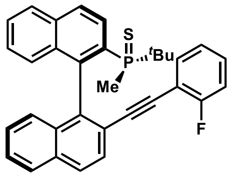

(**3p**) Light yellow solid, R_f = 0.20 (petroleum ether/ethyl acetate = 20:1), 75% yield, 92% ee, M.p. 149 °C. **^{1}H NMR** (600 MHz, Chloroform-*d*) δ 8.59 (dd, J = 12.1, 8.9 Hz, 1H), 8.07 (d, J = 8.4 Hz, 1H), 7.99 (d, J = 8.5 Hz, 1H), 7.93 (d, J = 8.6 Hz, 1H), 7.91 (d, J = 8.6 Hz, 1H), 7.77 (d, J = 8.5 Hz, 1H), 7.58-7.46 (m, 2H), 7.43-7.36 (m, 1H), 7.31-7.26 (m, 1H), 7.24 (d, J = 8.5 Hz, 1H), 7.18 (d, J = 8.6 Hz, 1H), 7.17-7.09 (m, 1H), 6.92-6.82 (m, 2H), 6.67-6.57 (m, 1H), 1.18 (d, J = 16.6 Hz, 9H), 0.74 (d, J = 12.9 Hz, 3H). **^{13}C NMR** (151 MHz, Chloroform-*d*) δ 162.21 (d, J = 253.3 Hz), 139.77 (d, J =

3.3 Hz), 138.97 (d, J = 4.8 Hz), 134.91, 134.50 (d, J = 2.1 Hz), 133.09, 132.79, 132.49 (d, J = 10.5 Hz), 130.88 (d, J = 12.7 Hz), 130.12 (d, J = 7.8 Hz), 129.83 (d, J = 67.7 Hz), 129.05, 128.26, 128.24, 128.21, 127.94, 127.87, 127.55 (d, J = 12.1 Hz), 127.54, 127.38, 127.14, 126.56, 123.84 (d, J = 3.7 Hz), 122.06, 115.44 (d, J = 20.6 Hz), 111.39 (d, J = 15.8 Hz), 94.63, 89.05, 36.29 (d, J = 49.9 Hz), 25.71 (d, J = 2.0 Hz), 15.37 (d, J = 52.1 Hz). **^{19}F NMR** (564 MHz, Chloroform-*d*) δ -109.92 (dd, J = 14.3, 7.0 Hz). **^{31}P NMR** (243 MHz, Chloroform-*d*) δ 61.53. The enantiomeric excess was determined by Daicel Chiralpak IA, *n*-hexane/isopropanol = 90/10, 1 mL/min, λ = 254 nm, *t* (minor) = 8.36 min, *t* (major) = 11.73 min. $[\alpha]_D^{25}$ = 160.9 (c = 0.647, CH_2Cl_2). **HRMS (ESI)** calcd for: $C_{33}H_{29}FPS^+$ $[M + H]^+$ 507.1706; found: 507.1715.

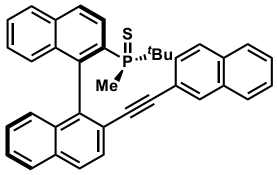

(**3q**) Yellow solid, R_f = 0.36 (petroleum ether/ethyl acetate = 20:1), 55% yield, 94% ee, M.p. 107-109 °C. **^{1}H NMR** (600 MHz, Chloroform-*d*) δ 8.62 (dd, J = 12.0, 9.0 Hz, 1H), 8.11 (d, J = 8.9 Hz, 1H), 8.01 (d, J = 8.6 Hz, 1H), 7.99 (d, J = 8.3 Hz, 1H), 7.92 (d, J = 8.1 Hz, 1H), 7.77 (d, J = 8.5 Hz, 1H), 7.72-7.66 (m, 1H), 7.61-7.48 (m, 4H), 7.45-7.37 (m, 3H), 7.34-7.29 (m, 1H), 7.28 (d, J = 8.5 Hz, 1H), 7.25 (d, J = 8.4 Hz, 1H), 7.17 (s, 1H), 6.68 (d, J = 8.4 Hz, 1H), 1.18 (d, J = 16.5 Hz, 9H), 0.76 (d, J = 12.9 Hz, 3H). **^{13}C NMR** (151 MHz, Chloroform-*d*) δ 139.71 (d, J = 3.4 Hz), 139.32 (d, J = 4.9 Hz), 134.97, 134.50 (d, J = 2.3 Hz), 132.85, 132.84, 132.65, 132.53 (d, J = 10.4 Hz), 131.20, 131.02, 130.93, 129.85 (d, J = 67.5 Hz), 129.10, 128.26, 128.23, 128.07, 128.02, 127.97, 127.92, 127.77, 127.65, 127.59, 127.49 (d, J = 12.3 Hz), 127.42, 127.25, 126.88, 126.65, 126.46, 122.41, 119.97, 96.53, 90.38, 36.32 (d, J = 49.9 Hz), 25.73 (d, J = 2.3 Hz), 15.35 (d, J = 52.1 Hz). **^{31}P NMR** (243 MHz, Chloroform-*d*) δ 61.53. The enantiomeric excess was determined by Daicel Chiralpak IA, *n*-hexane/isopropanol = 90/10, 1 mL/min, λ = 254 nm, *t* (minor) = 9.79 min, *t* (major) = 18.45 min. $[\alpha]_D^{25}$ = 152.8 (c = 0.490, CH_2Cl_2). **HRMS (ESI)** calcd for: $C_{37}H_{32}PS^+$ $[M + H]^+$ 539.1957; found: 539.1960.

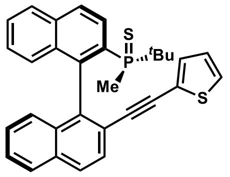

(**3r**) Yellow solid, R_f = 0.26 (petroleum ether/ethyl acetate = 20:1), 89% yield, 90% ee, M.p. 145-146 °C. **^{1}H NMR** (600 MHz, Chloroform-*d*) δ 8.59 (dd, J = 12.1, 8.9 Hz, 1H), 8.06 (d, J = 8.8 Hz, 1H), 7.97 (d, J = 8.5 Hz, 1H), 7.92 (d, J = 8.2 Hz, 1H), 7.89 (d, J = 8.2 Hz, 1H), 7.70 (d, J = 8.5 Hz, 1H), 7.55-7.45 (m, 2H), 7.37 (t, J = 7.4 Hz, 1H), 7.27 (t, J = 7.5 Hz, 1H), 7.23 (d, J = 8.5 Hz, 1H), 7.16 (d, J = 8.6 Hz, 1H), 7.07 (d, J = 4.8 Hz, 1H), 6.77 (dd, J = 4.8, 3.8 Hz, 1H), 6.60 (d, J = 3.0 Hz, 1H), 1.20 (d, J = 16.5 Hz, 9H), 0.75 (d, J = 12.9 Hz, 3H). **^{13}C NMR** (151 MHz, Chloroform-*d*) δ 139.33 (d, J = 3.4 Hz), 138.95 (d, J = 5.0 Hz), 134.82, 134.39 (d, J = 2.3 Hz), 132.57, 132.39 (d, J = 10.5 Hz), 131.61, 130.73 (d, J = 12.6 Hz), 129.67 (d, J = 67.5 Hz), 129.05, 128.22, 128.19, 127.96, 127.93, 127.74, 127.70, 127.53 (d, J = 12.2 Hz), 127.42, 127.28, 127.18, 127.04, 126.36, 122.59, 122.01, 93.61, 89.04, 36.27 (d, J = 49.9 Hz), 25.71 (d, J = 2.1 Hz), 15.28 (d, J = 52.1 Hz). **^{31}P NMR** (243 MHz, Chloroform-*d*) δ 61.43. The enantiomeric excess was determined by Daicel Chiralpak IA, *n*-hexane/isopropanol = 95/5, 1 mL/min, λ = 254 nm, *t* (minor) = 12.11 min, *t* (major) = 17.40 min. $[\alpha]_D^{25}$ = 160.0 (c = 0.810, CH_2Cl_2). **HRMS (ESI)** calcd for: $C_{31}H_{28}PS_2^+$ $[M + H]^+$ 495.1365; found: 495.1381.

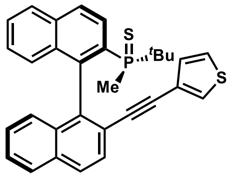

(**3s**) Yellow solid, R_f = 0.37 (petroleum ether/ethyl acetate = 20:1), 88% yield, 93% ee, M.p. 140 °C. **^{1}H NMR** (600 MHz, Chloroform-*d*) δ 8.59 (dd, J = 12.0, 9.0 Hz, 1H), 8.06 (d, J = 8.8 Hz, 1H), 7.97 (d, J = 8.5 Hz, 1H), 7.93 (d, J = 8.1 Hz, 1H), 7.90 (d, J = 8.2 Hz, 1H), 7.71 (d, J = 8.5 Hz, 1H), 7.56-7.45 (m, 2H), 7.37 (t, J = 7.6 Hz, 1H), 7.28 (t, J = 7.6 Hz, 1H), 7.22 (d, J = 8.5 Hz, 1H), 7.19 (d, J = 8.6 Hz, 1H), 7.06 (dd, J = 4.8, 3.0 Hz, 1H), 6.79 (d, J = 2.6 Hz, 1H), 6.41 (d, J =

4.9 Hz, 1H), 1.19 (d, *J* = 16.5 Hz, 9H), 0.74 (d, *J* = 12.9 Hz, 3H). **^{13}C NMR** (151 MHz, Chloroform-*d*) δ 139.34 (d, *J* = 3.5 Hz), 139.23 (d, *J* = 4.9 Hz), 134.88, 134.36 (d, *J* = 2.3 Hz), 132.54, 132.46 (d, *J* = 10.5 Hz), 130.85 (d, *J* = 12.6 Hz), 129.76 (d, *J* = 67.6 Hz), 129.28, 129.02, 128.46, 128.19, 128.18, 128.03, 127.93, 127.91, 127.46, 127.38, 127.32, 127.15, 126.38, 125.33, 122.34, 121.77, 91.08, 89.43, 36.27 (d, *J* = 49.9 Hz), 25.75 (d, *J* = 2.3 Hz), 15.31 (d, *J* = 52.2 Hz). **^{31}P NMR** (243 MHz, Chloroform-*d*) δ 61.46. The enantiomeric excess was determined by Daicel Chiralpak IA, *n*-hexane/isopropanol = 90/10, 1 mL/min, λ = 254 nm, *t* (minor) = 9.44 min, *t* (major) = 13.05 min. $[\alpha]_D^{25}$ = 156.9 (c = 0.667, CH_2Cl_2). **HRMS (ESI)** calcd for: $C_{31}H_{28}PS_2^+$ [M + H]$^+$ 495.1365; found: 495.1378.

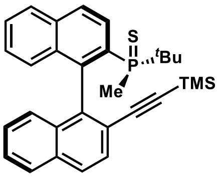

(**3t**) Yellow solid, R_f = 0.43 (petroleum ether/ethyl acetate = 20:1), 65% yield, 92% ee, M.p. 154-156 °C. **^{1}H NMR** (600 MHz, Chloroform-*d*) δ 8.59 (dd, *J* = 11.8, 9.2 Hz, 1H), 8.03 (d, *J* = 8.8 Hz, 1H), 7.94 (d, *J* = 8.5 Hz, 1H), 7.91 (d, *J* = 8.4 Hz, 1H), 7.89 (d, *J* = 8.4 Hz, 1H), 7.64 (d, *J* = 8.5 Hz, 1H), 7.56-7.46 (m, 2H), 7.38 (t, *J* = 7.7 Hz, 1H), 7.29 (d, *J* = 8.2 Hz, 1H), 7.26 (t, *J* = 7.9 Hz, 1H), 7.13 (d, *J* = 8.6 Hz, 1H), 1.21 (d, *J* = 16.5 Hz, 9H), 0.66 (d, *J* = 12.9 Hz, 3H), -0.33 (s, 9H). **^{13}C NMR** (151 MHz, Chloroform-*d*) δ 140.42 (d, *J* = 3.5 Hz), 139.16 (d, *J* = 5.0 Hz), 134.82, 134.43 (d, *J* = 2.3 Hz), 132.65, 132.40 (d, *J* = 10.5 Hz), 131.00 (d, *J* = 12.7 Hz), 129.35 (d, *J* = 67.3 Hz), 128.88, 128.16, 128.14, 128.03, 127.82, 127.80, 127.51, 127.43, 127.27 (d, *J* = 12.2 Hz), 126.97, 126.47, 122.28, 105.14, 101.51, 36.28 (d, *J* = 49.9 Hz), 25.85 (d, *J* = 2.3 Hz), 15.27 (d, *J* = 52.1 Hz), -0.75. **^{31}P NMR** (243 MHz, Chloroform-*d*) δ 61.57. The enantiomeric excess was determined by Daicel Chiralpak IE, *n*-hexane/isopropanol = 90/10, 1 mL/min, λ = 254 nm, *t* (major) = 14.30 min, *t* (minor) = 15.28 min. $[\alpha]_D^{25}$ = 183.3 (c = 0.402, CH_2Cl_2). **HRMS (ESI)** calcd for: $C_{30}H_{34}PSSi^+$ [M + H]$^+$ 485.1883; found: 485.1891.

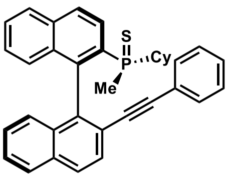

(**3u**) Light yellow solid, R_f = 0.20 (petroleum ether/ethyl acetate = 20:1), 84% yield, 94% ee, M.p. 96-97 °C. **^{1}H NMR** (600 MHz, Chloroform-*d*) δ 8.82 (dd, *J* = 13.3, 8.8 Hz, 1H), 8.14 (d, *J* = 8.7 Hz, 1H), 8.02 (d, *J* = 8.5 Hz, 1H), 7.99 (d, *J* = 8.2 Hz, 1H), 7.93 (d, *J* = 8.2 Hz, 1H), 7.77 (d, *J* = 8.5 Hz, 1H), 7.56-7.52 (m, 1H), 7.52-7.48 (m, 1H), 7.34 (t, *J* = 7.6 Hz, 1H), 7.30-7.24 (m, 1H), 7.19 (d, *J* = 8.4 Hz, 1H), 7.15 (t, *J* = 7.5 Hz, 1H), 7.12 (d, *J* = 8.7 Hz, 1H), 7.08 (t, *J* = 7.6 Hz, 2H), 6.67 (d, *J* = 7.8 Hz, 2H), 2.25-2.09 (m, 1H), 1.67-1.59 (m, 1H), 1.52-1.30 (m, 5H), 1.29-1.17 (m, 2H), 1.14-0.98 (m, 2H), 0.81 (d, *J* = 13.0 Hz, 3H). **^{13}C NMR** (151 MHz, Chloroform-*d*) δ 139.66 (d, *J* = 3.7 Hz), 139.59 (d, *J* = 6.4 Hz), 134.67 (d, *J* = 2.3 Hz), 134.35, 132.80 (d, *J* = 10.4 Hz), 132.66, 131.28, 130.92 (d, *J* = 12.9 Hz), 129.24, 128.81 (d, *J* = 70.1 Hz), 128.49, 128.25, 128.19, 128.07, 127.98, 127.95, 127.80, 127.75 (d, *J* = 12.8 Hz), 127.37, 127.19, 127.10, 126.91, 122.69, 122.49, 95.83, 88.93, 40.56 (d, *J* = 52.3 Hz), 26.29 (d, *J* = 14.4 Hz), 25.67 (d, *J* = 11.2 Hz), 25.62 (d, *J* = 2.2 Hz), 25.59 (d, *J* = 2.7 Hz), 25.25 (d, *J* = 2.0 Hz), 18.70 (d, *J* = 54.4 Hz). **^{31}P NMR** (243 MHz, Chloroform-*d*) δ 52.17. The enantiomeric excess was determined by Daicel Chiralpak IA, *n*-hexane/isopropanol = 90/10, 1 mL/min, λ = 254 nm, *t* (minor) = 7.09 min, *t* (major) = 10.01 min. $[\alpha]_D^{25}$ = 124.6 (c = 0.786, CH_2Cl_2). **HRMS (ESI)** calcd for: $C_{35}H_{32}PS^+$ [M + H]$^+$ 515.1957; found: 515.1965.

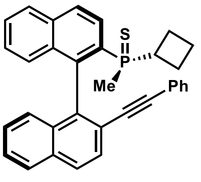

(**3v**) Light yellow solid, R_f = 0.34 (petroleum ether/ethyl acetate = 20:1), 64% yield, 95% ee, M.p. 149 °C. **^{1}H NMR** (600 MHz, Chloroform-*d*) δ 8.68 (dd, *J* = 13.2, 8.8 Hz, 1H), 8.10 (d, *J* = 8.6 Hz, 1H), 8.01 (d, *J* = 8.5 Hz, 1H), 7.95 (d, *J* = 8.1 Hz, 1H), 7.92 (d, *J* = 8.2 Hz, 1H), 7.77 (d, *J* = 8.5 Hz, 1H), 7.55-7.47 (m, 2H), 7.34 (t, *J* = 7.2 Hz, 1H), 7.29-7.24 (m, 1H), 7.20 (d, *J* = 8.7 Hz, 1H), 7.17 (d,

J = 7.4 Hz, 1H), 7.14-7.07 (m, 3H), 6.75 (d, *J* = 7.2 Hz, 2H), 3.28-3.16 (m, 1H), 2.47-2.37 (m, 1H), 2.35-2.27 (m, 1H), 1.88-1.59 (m, 4H), 0.74 (d, *J* = 13.1 Hz, 3H). **^{13}C NMR** (151 MHz, Chloroform-*d*) δ 139.75 (d, *J* = 3.8 Hz), 138.93 (d, *J* = 6.5 Hz), 134.60 (d, *J* = 2.2 Hz), 134.07, 132.74 (d, *J* = 10.5 Hz), 132.66, 131.24, 129.78, 129.69, 129.18, 128.53, 128.24, 128.20, 128.09, 128.00, 127.83, 127.78, 127.36, 127.16, 126.94, 126.85, 122.43 (d, *J* = 10.2 Hz), 95.61, 88.94, 36.60 (d, *J* = 50.1 Hz), 21.90 (d, *J* = 4.8 Hz), 21.80 (d, *J* = 4.3 Hz), 19.55 (d, *J* = 19.1 Hz), 18.04 (d, *J* = 55.2 Hz). **^{31}P NMR** (243 MHz, Chloroform-*d*) δ 48.86. The enantiomeric excess was determined by Daicel Chiralpak IA, *n*-hexane/isopropanol = 90/10, 1 mL/min, λ = 254 nm, *t* (minor) = 6.75 min, *t* (major) = 11.86 min. $[\alpha]_D^{25}$ = 155.1 (c = 0.411, CH_2Cl_2). **HRMS (ESI)** calcd for: $C_{33}H_{28}PS^+$ $[M + H]^+$ 487.1644; found: 487.1636.

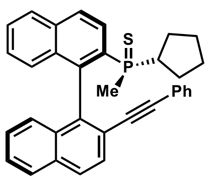

(**3w**) Light yellow solid, R_f = 0.36 (petroleum ether/ethyl acetate = 20:1), 81% yield, 91% ee, M.p. 161-162 °C. **^{1}H NMR** (600 MHz, Chloroform-*d*) δ 8.85 (dd, *J* = 13.1, 8.9 Hz, 1H), 8.12 (d, *J* = 8.7 Hz, 1H), 8.00 (d, *J* = 8.5 Hz, 1H), 7.97 (d, *J* = 8.2 Hz, 1H), 7.91 (d, *J* = 8.2 Hz, 1H), 7.76 (d, *J* = 8.4 Hz, 1H), 7.52 (t, *J* = 7.5 Hz, 1H), 7.49 (t, *J* = 7.5 Hz, 1H), 7.31 (t, *J* = 7.6 Hz, 1H), 7.29-7.24 (m, 1H), 7.17-7.13 (m, 2H), 7.13-7.06 (m, 3H), 6.73 (d, *J* = 7.8 Hz, 2H), 2.71-2.59 (m, 1H), δ 1.80-1.66 (m, 2H), 1.66-1.55 (m, 2H), 1.48-1.40 (m, 2H), 1.34-1.26 (m, 1H), 1.12-1.01 (m, 1H), 0.81 (d, *J* = 13.1 Hz, 3H). **^{13}C NMR** (151 MHz, Chloroform-*d*) δ 139.73 (d, *J* = 3.6 Hz), 139.05 (d, *J* = 6.6 Hz), 134.69 (d, *J* = 2.1 Hz), 134.40, 132.68 (d, *J* = 10.2 Hz), 132.66, 131.22, 130.41 (d, *J* = 13.1 Hz), 129.89 (d, *J* = 70.6 Hz), 129.19, 128.56, 128.24, 128.19, 128.06, 128.01, 127.93, 127.89, 127.83, 127.37, 127.17, 127.04, 126.80, 122.37, 122.30, 95.52, 88.87, 41.78 (d, *J* = 54.9 Hz), 27.30, 26.71, 26.13 (d, *J* = 11.3 Hz), 25.96 (d, *J* = 11.9 Hz), 19.92 (d, *J* = 55.3 Hz). **^{31}P NMR** (243 MHz, Chloroform-*d*) δ 52.17. The enantiomeric excess was determined by Daicel Chiralpak IA, *n*-hexane/isopropanol = 90/10, 1 mL/min, λ = 254 nm, *t* (minor) = 6.36 min, *t* (major) = 10.65 min. $[\alpha]_D^{25}$ = 142.4 (c = 0.424, CH_2Cl_2). **HRMS (ESI)** calcd for: $C_{34}H_{30}PS^+$ $[M + H]^+$ 501.1800; found: 501.1802.

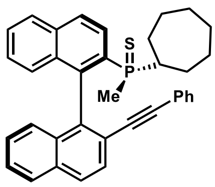

(**3x**) Light yellow solid, R_f = 0.34 (petroleum ether/ethyl acetate = 20:1), 55% yield, 95% ee, M.p. 75-76 °C. **^{1}H NMR** (600 MHz, Chloroform-*d*) δ 8.69 (dd, *J* = 12.8, 8.9 Hz, 1H), 8.14 (d, *J* = 8.7 Hz, 1H), 8.01 (d, *J* = 8.5 Hz, 1H), 7.98 (d, *J* = 8.2 Hz, 1H), 7.92 (d, *J* = 8.2 Hz, 1H), 7.75 (d, *J* = 8.5 Hz, 1H), 7.57-7.52 (m, 1H), 7.50 (t, *J* = 7.6 Hz, 1H), 7.34 (t, *J* = 7.4 Hz, 1H), 7.28 (t, *J* = 7.5 Hz, 1H), 7.22 (d, *J* = 8.4 Hz, 1H), 7.18-7.11 (m, 2H), 7.08 (t, *J* = 7.5 Hz, 2H), 6.65 (d, *J* = 7.3 Hz, 2H), 2.33-2.20 (m, 1H), 1.78-1.69 (m, 1H), 1.69-1.60 (m, 1H), 1.57-1.50 (m, 1H), 1.50-1.40 (m, 3H), 1.38-1.24 (m, 4H), 1.21-1.09 (m, 2H), 0.82 (d, *J* = 12.8 Hz, 3H). **^{13}C NMR** (151 MHz, Chloroform-*d*) δ 139.60 (d, *J* = 3.7 Hz), 139.42 (d, *J* = 6.2 Hz), 134.55 (d, *J* = 1.9 Hz), 134.21, 132.79 (d, *J* = 10.4 Hz), 132.71, 131.29, 130.01 (d, *J* = 12.6 Hz), 129.79 (d, *J* = 70.5 Hz), 129.21, 128.47, 128.19, 128.14, 128.05, 128.04, 127.93, 127.88, 127.80, 127.42, 127.24, 127.13, 127.05, 122.61, 122.53, 95.88, 89.19, 42.20 (d, *J* = 50.1 Hz), 28.57, 28.00 (d, *J* = 15.6 Hz), 27.90, 27.43, 27.20, 27.17 (d, *J* = 16.8 Hz), 18.18 (d, *J* = 53.9 Hz). **^{31}P NMR** (243 MHz, Chloroform-*d*) δ 54.47. The enantiomeric excess was determined by Daicel Chiralpak IA, *n*-hexane/isopropanol = 90/10, 1 mL/min, λ = 254 nm, *t* (minor) = 8.80 min, *t* (major) = 10.83 min. $[\alpha]_D^{25}$ = 141.7 (c = 0.453, CH_2Cl_2). **HRMS (ESI)** calcd for: $C_{36}H_{34}PS^+$ $[M + H]^+$ 529.2113; found: 529.2122.

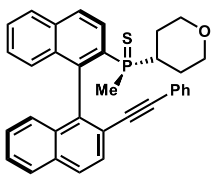

(**3y**) Light yellow solid, R_f = 0.49 (petroleum ether/ethyl acetate = 5:1), 84% yield, 95% ee, M.p. 84-85 °C. **^{1}H NMR** (600 MHz, Chloroform-*d*) δ 8.81 (dd, *J* = 13.2, 9.0 Hz, 1H), 8.15 (d, *J* = 8.6 Hz, 1H), 8.02 (d, *J* = 8.5 Hz, 1H), 7.99 (d, *J* = 8.1 Hz, 1H), 7.92 (d, *J* = 8.0 Hz, 1H), 7.79 (d, *J* = 8.4 Hz, 1H), 7.54 (t, *J* = 7.2 Hz, 1H), 7.50 (t, *J* = 7.3 Hz, 1H), 7.36-7.29 (m, 1H), 7.27 (t, *J* = 7.5 Hz, 1H), 7.17 (d, *J* = 8.4 Hz, 1H), 7.13 (d, *J* = 8.3 Hz, 2H), 7.07 (t, *J* = 7.3 Hz, 2H), 6.64 (d, *J* = 7.4 Hz, 2H), 3.91-3.76 (m, 1H), 3.51-3.37 (m, 1H), 3.21 (t, *J* = 11.5 Hz, 1H), 2.83 (t, *J* = 11.6 Hz, 1H), 2.52-2.38 (m, 1H), 1.95-1.69 (m, 2H), 1.29-1.11 (m, 2H), 0.81 (d, *J* = 13.0 Hz, 3H). **^{13}C NMR** (151 MHz, Chloroform-*d*) δ 139.63 (d, *J* = 6.6 Hz), 139.47 (d, *J* = 3.6 Hz), 134.66 (d, *J* = 2.0 Hz), 134.24, 132.68 (d, *J* = 10.6 Hz), 132.61, 131.10, 130.61 (d, *J* = 13.1 Hz), 129.34, 128.68, 128.30, 128.24, 128.10, 128.03, 127.90, 127.86, 127.78, 127.42, 127.29, 126.99, 126.64, 122.31, 122.07, 95.74, 89.00, 67.33 (d, *J* = 14.2 Hz), 67.21 (d, *J* = 14.4 Hz), 38.09 (d, *J* = 54.5 Hz), 25.10 (d, *J* = 60.0 Hz), 18.18 (d, *J* = 55.0 Hz). **^{31}P NMR** (243 MHz, Chloroform-*d*) δ 50.64. The enantiomeric excess was determined by Daicel Chiralpak IA, *n*-hexane/isopropanol = 90/10, 1 mL/min, λ = 254 nm, *t* (minor) = 10.34 min, *t* (major) = 14.16 min. $[\alpha]_D^{25}$ = 154.3 (c = 0.735, CH_2Cl_2). **HRMS (ESI)** calcd for: $C_{34}H_{30}OPS^+$ [M + H]$^+$ 517.1749; found: 517.1756.

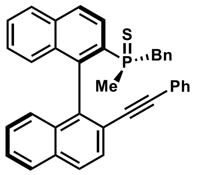

(**3z**) Light yellow solid, R_f = 0.26 (petroleum ether/ethyl acetate = 20:1), 75% yield, 97% ee, M.p. 76-77 °C. **^{1}H NMR** (600 MHz, Chloroform-*d*) δ 8.64 (dd, *J* = 13.7, 8.8 Hz, 1H), 8.10 (d, *J* = 8.8 Hz, 1H), 8.06 (d, *J* = 8.5 Hz, 1H), 7.98 (d, *J* = 8.2 Hz, 1H), 7.94 (d, *J* = 8.2 Hz, 1H), 7.82 (d, *J* = 8.5 Hz, 1H), 7.56 (t, *J* = 7.4 Hz, 1H), 7.51 (t, *J* = 7.5 Hz, 1H), 7.37-7.29 (m, 2H), 7.21-7.15 (m, 3H), 7.14-7.07 (m, 5H), 7.04 (d, *J* = 7.2 Hz, 2H), 6.76 (d, *J* = 7.3 Hz, 2H), 3.65-3.56 (m, 1H), 3.39 (t, *J* = 14.9 Hz, 1H), 0.73 (d, *J* = 13.3 Hz, 3H). **^{13}C NMR** (151 MHz, Chloroform-*d*) δ 139.65 (d, *J* = 3.9 Hz), 138.89 (d, *J* = 6.8 Hz), 134.74 (d, *J* = 2.3 Hz), 134.21, 132.74, 132.67 (d, *J* = 10.8 Hz), 131.76 (d, *J* = 8.1 Hz), 131.38, 130.42, 130.38, 129.86, 129.77, 129.40, 128.66, 128.35, 128.31, 128.28, 128.13, 128.10, 128.08, 128.05, 127.96, 127.47, 127.34, 127.11, 127.08, 126.74, 122.34, 95.97, 89.08, 43.41 (d, *J* = 47.7 Hz), 19.67 (d, *J* = 56.6 Hz). **^{31}P NMR** (243 MHz, Chloroform-*d*) δ 44.00. The enantiomeric excess was determined by Daicel Chiralpak IA, *n*-hexane/isopropanol = 90/10, 1 mL/min, λ = 254 nm, *t* (minor) = 9.77 min, *t* (major) = 12.13 min. $[\alpha]_D^{25}$ = 155.1 (c = 0.411, CH_2Cl_2). **HRMS (ESI)** calcd for: $C_{36}H_{28}PS^+$ [M + H]$^+$ 523.1644; found: 523.1648.

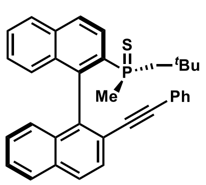

(**3aa**) Light yellow solid, R_f = 0.36 (petroleum ether/ethyl acetate = 20:1), 73% yield, 96% ee, M.p. 143-144 °C. **^{1}H NMR** (600 MHz, Chloroform-*d*) δ 8.91 (dd, *J* = 14.0, 8.9 Hz, 1H), 8.15 (d, *J* = 8.8 Hz, 1H), 8.01 (d, *J* = 8.5 Hz, 1H), 7.96 (d, *J* = 8.2 Hz, 1H), 7.91 (d, *J* = 8.2 Hz, 1H), 7.76 (d, *J* = 8.5 Hz, 1H), 7.54-7.45 (m, 2H), 7.30 (t, *J* = 7.6 Hz, 1H), 7.28-7.23 (m, 1H), 7.19-7.12 (m, 2H), 7.12-7.06 (m, 3H), 6.75 (d, *J* = 7.6 Hz, 2H), 2.30 (dd, *J* = 14.9, 9.7 Hz, 1H), 2.11 (t, *J* = 14.3 Hz, 1H), 0.99 (d, *J* = 13.3 Hz, 3H), 0.95 (s, 9H). **^{13}C NMR** (151 MHz, Chloroform-*d*) δ 139.77 (d, *J* = 3.4 Hz), 138.22 (d, *J* = 6.5 Hz), 134.51 (d, *J* = 1.9 Hz), 134.33, 132.66, 132.59 (d, *J* = 10.6 Hz), 132.16 (d, *J* = 73.2 Hz), 131.31, 129.82, 129.73, 129.22, 128.56, 128.51, 128.42, 128.23, 128.13, 128.04, 127.83, 127.79, 127.34, 127.21, 127.10, 126.83, 122.42 (d, *J* = 3.3 Hz), 95.93, 89.41, 47.94 (d, *J* = 49.7 Hz), 32.76 (d, *J* = 4.5 Hz), 31.22 (d, *J* = 7.3 Hz), 23.40 (d, *J* = 54.1 Hz). **^{31}P NMR** (243 MHz, Chloroform-*d*) δ 41.95. The enantiomeric excess was determined by Daicel Chiralpak IA, *n*-hexane/isopropanol = 90/10, 1 mL/min, λ = 254 nm, *t* (minor) = 5.61 min, *t* (major) = 7.68 min. $[\alpha]_D^{25}$ = 91.4 (c = 0.699, CH_2Cl_2). **HRMS (ESI)** calcd for: $C_{34}H_{32}PS^+$ [M + H]$^+$ 503.1957; found: 503.1965.

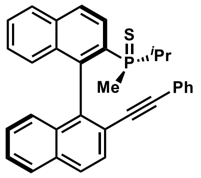

(**3ab**) Light yellow solid, R_f = 0.27 (petroleum ether/ethyl acetate = 20:1), 72% yield, 95% ee, M.p. 189-190 ºC. **^{1}H NMR** (600 MHz, Chloroform-*d*) δ 8.84 (dd, *J* = 13.3, 8.8 Hz, 1H), 8.13 (d, *J* = 8.8 Hz, 1H), 8.01 (d, *J* = 8.5 Hz, 1H), 7.98 (d, *J* = 8.2 Hz, 1H), 7.92 (d, *J* = 8.2 Hz, 1H), 7.76 (d, *J* = 8.5 Hz, 1H), 7.53 (t, *J* = 7.5 Hz, 1H), 7.49 (t, *J* = 7.5 Hz, 1H), 7.32 (t, *J* = 7.6 Hz, 1H), 7.29-7.24 (m, 1H), 7.17-7.13 (m, 2H), 7.12 (d, *J* = 8.8 Hz, 1H), 7.09 (t, *J* = 7.6 Hz, 2H), 6.73 (d, *J* = 7.4 Hz, 2H), 2.58-2.46 (m, 1H), 1.00 (dd, *J* = 18.8, 6.9 Hz, 3H), 0.90-0.79 (m, 6H). **^{13}C NMR** (151 MHz, Chloroform-*d*) δ 139.57 (d, *J* = 6.6 Hz), 139.51 (d, *J* = 3.7 Hz), 134.71 (d, *J* = 2.1 Hz), 134.34, 132.76 (d, *J* = 10.5 Hz), 132.66, 131.30, 130.82, 130.73, 129.23, 128.52, 128.24, 128.21, 128.08, 127.98, 127.92, 127.83, 127.81, 127.39, 127.21, 127.08, 126.87, 122.49 (d, *J* = 8.9 Hz), 95.73, 88.86, 31.11 (d, *J* = 52.7 Hz), 18.72 (d, *J* = 54.3 Hz), 16.26, 15.98. **^{31}P NMR** (243 MHz, Chloroform-*d*) δ 55.75. The enantiomeric excess was determined by Daicel Chiralpak ID, *n*-hexane/isopropanol = 90/10, 1 mL/min, λ = 254 nm, *t* (minor) = 9.83 min, *t* (major) = 10.63 min. $[\alpha]_D^{25}$ = 86.9 (c = 0.373, CH_2Cl_2). **HRMS (ESI)** calcd for: $C_{32}H_{28}PS^+$ $[M + H]^+$ 475.1644; found: 475.1652.

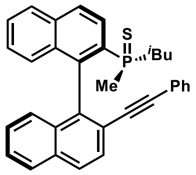

(**3ac**) Light yellow solid, R_f = 0.26 (petroleum ether/ethyl acetate = 20:1), 64% yield, 94% ee, M.p. 58-59 ºC. **^{1}H NMR** (600 MHz, Chloroform-*d*) δ 8.85-8.74 (m, 1H), 8.14 (d, *J* = 8.8 Hz, 1H), 8.01 (d, *J* = 8.5 Hz, 1H), 7.96 (d, *J* = 8.3 Hz, 1H), 7.92 (d, *J* = 8.3 Hz, 1H), 7.76 (d, *J* = 8.5 Hz, 1H), 7.52 (t, *J* = 7.5 Hz, 1H), 7.51-7.46 (m, 1H), 7.31 (t, *J* = 7.6 Hz, 1H), 7.27 (t, *J* = 7.7 Hz, 1H), 7.15 (t, *J* = 9.0 Hz, 2H), 7.13 (d, *J* = 8.9 Hz, 1H), 7.09 (t, *J* = 7.5 Hz, 2H), 6.74 (d, *J* = 7.8 Hz, 2H), 2.17-1.90 (m, 3H), 0.94 (d, *J* = 13.3 Hz, 3H), 0.81 (d, *J* = 6.3 Hz, 3H), 0.75 (d, *J* = 6.5 Hz, 3H). **^{13}C NMR** (151 MHz, Chloroform-*d*) δ 139.59 (d, *J* = 3.6 Hz), 138.89 (d, *J* = 6.6 Hz), 134.59 (d, *J* = 2.1 Hz), 134.16, 132.68, 132.67 (d, *J* = 10.4 Hz), 131.28, 129.48, 129.39, 129.22, 128.53, 128.46, 128.38, 128.21, 128.10, 128.06, 127.91, 127.77, 127.35, 127.25, 127.06, 126.84, 122.48 (d, *J* = 11.2 Hz), 95.83, 89.18, 44.09 (d, *J* = 51.6 Hz), 24.61 (d, *J* = 10.9 Hz), 24.35 (d, *J* = 3.4 Hz), 24.18 (d, *J* = 8.1 Hz), 21.84 (d, *J* = 54.8 Hz). **^{31}P NMR** (243 MHz, Chloroform-*d*) δ 43.97. The enantiomeric excess was determined by Daicel Chiralpak IA, *n*-hexane/isopropanol = 90/10, 1 mL/min, λ = 254 nm, *t* (minor) = 6.22 min, *t* (major) = 9.05 min. $[\alpha]_D^{25}$ = 85.0 (c = 0.534, CH_2Cl_2). **HRMS (ESI)** calcd for: $C_{33}H_{30}PS^+$ $[M + H]^+$ 489.1800; found: 489.1803.

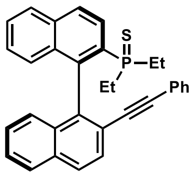

(**3ad**) Light yellow solid, R_f = 0.32 (petroleum ether/ethyl acetate = 20:1), 62% yield, 96% ee, M.p. 64-65 ºC. **^{1}H NMR** (600 MHz, Chloroform-*d*) δ 8.88 (dd, *J* = 13.5, 8.8 Hz, 1H), 8.14 (d, *J* = 8.8 Hz, 1H), 8.02 (d, *J* = 8.5 Hz, 1H), 7.97 (d, *J* = 8.2 Hz, 1H), 7.94 (d, *J* = 8.2 Hz, 1H), 7.75 (d, *J* = 8.5 Hz, 1H), 7.56-7.47 (m, 2H), 7.30 (t, *J* = 7.3 Hz, 1H), 7.28-7.23 (m, 1H), 7.15 (t, *J* = 7.4 Hz, 1H), 7.13-7.06 (m, 4H), 6.77-6.71 (m, 2H), 2.14-1.98 (m, 1H), 1.73-1.56 (m, 1H), 1.22-1.12 (m, 1H), 1.10-1.01 (m, 1H), 0.96 (dt, *J* = 19.8, 7.5 Hz, 3H), 0.78 (dt, *J* = 19.9, 7.5 Hz, 3H). **^{13}C NMR** (151 MHz, Chloroform-*d*) δ 139.87 (d, *J* = 6.9 Hz), 139.45 (d, *J* = 3.4 Hz), 134.70 (d, *J* = 2.2 Hz), 133.77, 132.89 (d, *J* = 10.4 Hz), 132.68, 131.34, 131.13 (d, *J* = 12.6 Hz), 129.23, 128.49, 128.33, 128.21, 128.20, 128.07, 127.97, 127.95, 127.89, 127.37, 127.28, 127.18, 127.08, 126.88, 122.91, 122.48, 95.85, 88.87, 25.77 (d, *J* = 53.3 Hz), 24.56 (d, *J* = 53.3 Hz), 7.26 (d, *J* = 4.5 Hz), 7.10 (d, *J* = 4.5 Hz). **^{31}P NMR** (243 MHz, Chloroform-*d*) δ 58.51. The enantiomeric excess was determined by Daicel Chiralpak IA, *n*-hexane/isopropanol = 90/10, 1 mL/min, λ = 254 nm, *t* (minor) = 6.11 min, *t* (major) = 9.61 min. $[\alpha]_D^{25}$ = 53.1 (c = 0.488, CH_2Cl_2). **HRMS (ESI)** calcd for: $C_{32}H_{28}PS^+$ $[M + H]^+$ 475.1644; found: 475.1650.

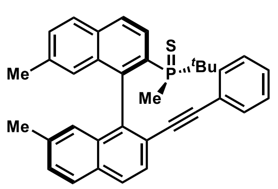

(**3ae**) Yellow solid, R_f = 0.16 (petroleum ether/ethyl acetate = 20:1), 63% yield, 95% ee, M.p. 98 °C. **^{1}H NMR** (600 MHz, Chloroform-*d*) δ 8.50 (dd, *J* = 12.2, 8.9 Hz, 1H), 8.02 (d, *J* = 8.8 Hz, 1H), 7.93 (d, *J* = 8.4 Hz, 1H), 7.84 (d, *J* = 8.3 Hz, 1H), 7.81 (d, *J* = 8.3 Hz, 1H), 7.66 (d, *J* = 8.5 Hz, 1H), 7.36 (d, *J* = 8.3 Hz, 1H), 7.34 (d, *J* = 8.3 Hz, 1H), 7.16 (t, *J* = 7.4 Hz, 1H), 7.10 (t, *J* = 7.4 Hz, 2H), 7.01 (s, 1H), 6.96 (s, 1H), 6.70 (d, *J* = 7.1 Hz, 2H), 2.30 (s, 3H), 2.24 (s, 3H), 1.17 (d, *J* = 16.5 Hz, 9H), 0.71 (d, *J* = 12.9 Hz, 3H). **^{13}C NMR** (151 MHz, Chloroform-*d*) δ 139.03 (d, *J* = 3.3 Hz), 138.72 (d, *J* = 4.9 Hz), 138.12, 136.78, 135.18, 132.74 (d, *J* = 5.7 Hz), 132.70 (d, *J* = 2.3 Hz), 131.11, 130.97, 130.19, 129.99, 129.91, 129.68, 128.65, 128.25, 128.20, 127.99, 127.75, 127.29, 127.14 (d, *J* = 12.4 Hz), 126.40, 125.48, 122.92, 122.31, 95.55, 90.22, 36.26 (d, *J* = 49.9 Hz), 25.78 (d, *J* = 2.0 Hz), 22.12, 22.04, 15.28 (d, *J* = 52.1 Hz). **^{31}P NMR** (243 MHz, Chloroform-*d*) δ 61.40. The enantiomeric excess was determined by Daicel Chiralpak IE, *n*-hexane/isopropanol = 90/10, 1 mL/min, λ = 254 nm, *t* (major) = 10.94 min, *t* (minor) = 12.03 min. $[\alpha]_D^{25}$ = 226.3 (c = 0.588, CH_2Cl_2). **HRMS (ESI)** calcd for: $C_{35}H_{34}PS^+$ $[M + H]^+$ 517.2113; found: 517.2120.

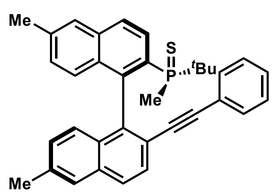

(**3af**) Light yellow solid, R_f = 0.42 (petroleum ether/ethyl acetate = 20:1), 84% yield, 93% ee, M.p. 86 °C. **^{1}H NMR** (600 MHz, Chloroform-*d*) δ 8.53 (dd, *J* = 12.1, 8.9 Hz, 1H), 7.97 (d, *J* = 8.9 Hz, 1H), 7.88 (d, *J* = 8.5 Hz, 1H), 7.75-7.63 (m, 3H), 7.21 (d, *J* = 8.5 Hz, 1H), 7.16 (t, *J* = 7.3 Hz, 1H), 7.14-7.04 (m, 5H), 6.74 (d, *J* = 7.5 Hz, 2H), 2.48 (s, 3H), 2.46 (s, 3H), 1.18 (d, *J* = 16.5 Hz, 9H), 0.75 (d, *J* = 12.9 Hz, 3H). **^{13}C NMR** (151 MHz, Chloroform-*d*) δ 139.45 (d, *J* = 3.3 Hz), 139.18 (d, *J* = 5.1 Hz), 137.83, 137.31, 134.59 (d, *J* = 2.0 Hz), 133.13, 132.80, 131.05, 130.82 (d, *J* = 12.3 Hz), 130.72, 130.34, 129.37, 128.23, 128.19, 128.15, 127.24, 127.15, 126.90, 126.80, 126.72, 126.23, 122.85, 121.22, 95.28, 90.07, 36.17 (d, *J* = 50.0 Hz), 25.69, 21.64, 21.59, 15.25 (d, *J* = 52.1 Hz). **^{31}P NMR** (243 MHz, Chloroform-*d*) δ 61.29. The enantiomeric excess was determined by Daicel Chiralpak IA, *n*-hexane/isopropanol = 90/10, 1 mL/min, λ = 254 nm, *t* (major) = 7.07 min, *t* (minor) = 9.84 min. $[\alpha]_D^{25}$ = 211.0 (c = 0.780, CH_2Cl_2). **HRMS (ESI)** calcd for: $C_{35}H_{34}PS^+$ $[M + H]^+$ 517.2113; found: 517.2125.

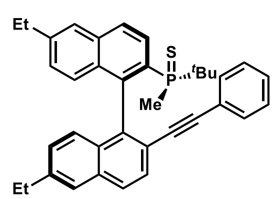

(**3ag**) Yellow solid, R_f = 0.37 (petroleum ether/ethyl acetate = 20:1), 90% yield, 90% ee, M.p. 88 °C. **^{1}H NMR** (600 MHz, Chloroform-*d*) δ 8.55 (dd, *J* = 11.9, 9.2 Hz, 1H), 7.99 (d, *J* = 8.7 Hz, 1H), 7.91 (d, *J* = 8.4 Hz, 1H), 7.75-7.62 (m, 3H), 7.24 (d, *J* = 9.1 Hz, 1H), 7.19-7.05 (m, 6H), 6.71 (d, *J* = 7.3 Hz, 2H), 2.83-2.70 (m, 4H), 1.34-1.22 (m, 6H), 1.18 (d, *J* = 16.5 Hz, 9H), 0.75 (d, *J* = 12.9 Hz, 3H). **^{13}C NMR** (151 MHz, Chloroform-*d*) δ 144.11, 143.59, 139.57 (d, *J* = 3.4 Hz), 139.27 (d, *J* = 5.0 Hz), 134.79 (d, *J* = 2.3 Hz), 133.48, 132.95, 131.14, 131.02 (d, *J* = 10.7 Hz), 130.90 (d, *J* = 12.7 Hz), 129.41, 128.53 (d, *J* = 68.6 Hz), 128.48, 128.36, 128.24, 128.22, 128.19, 127.53, 127.01 (d, *J* = 12.3 Hz), 126.44, 125.85, 125.62, 122.97, 121.36, 95.41, 90.21, 36.26 (d, *J* = 50.1 Hz), 28.98, 28.97, 25.75 (d, *J* = 2.2 Hz), 15.31 (d, *J* = 52.1 Hz), 15.28, 15.17. **^{31}P NMR** (243 MHz, Chloroform-*d*) δ 61.32. The enantiomeric excess was determined by Daicel Chiralpak ID, *n*-hexane/isopropanol = 90/10, 1 mL/min, λ = 254 nm, *t* (minor) = 12.64 min, *t* (major) = 13.65 min. $[\alpha]_D^{25}$ = 219.7 (c = 0.770, CH_2Cl_2). **HRMS (ESI)** calcd for: $C_{37}H_{38}PS^+$ $[M + H]^+$ 545.2426; found: 545.2418.

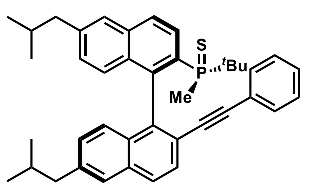

(**3ah**) Light yellow solid, R_f = 0.54 (petroleum ether/ethyl acetate = 20:1), 79% yield, 92% ee, M.p. 87-88 °C. **^{1}H NMR** (600 MHz, Chloroform-*d*) 8.56 (dd, *J* = 12.2, 9.0 Hz, 1H), 7.99 (d, *J* = 8.7 Hz, 1H), 7.90 (d, *J* = 8.5 Hz, 1H), 7.74-7.65 (m, 2H), 7.63 (s, 1H), 7.20 (q, *J* = 8.7 Hz, 2H), 7.17-7.10 (m, 3H), 7.07 (t, *J* = 7.5 Hz, 2H), 6.67 (d, *J* = 7.4 Hz, 2H), 2.67-2.54 (m, 4H), 2.02-1.85 (m, 2H), 1.16 (d, *J* = 16.5 Hz, 9H), 0.96-0.83 (m, 12H), 0.72 (d, *J* = 12.9 Hz, 3H). **^{13}C NMR** (151 MHz, Chloroform-*d*) δ 141.56, 141.10, 139.51 (d, *J* = 3.3 Hz), 139.19 (d, *J* = 5.0 Hz), 134.52, 133.48, 132.70, 131.00, 130.89 (d, *J* = 12.3 Hz), 130.08, 129.11, 128.43 (d, *J* = 68.8 Hz), 128.42, 128.17, 128.11, 128.03, 127.42, 127.34, 127.18, 126.93, 126.85, 126.23, 122.82, 121.37, 95.46, 90.12, 45.48 (d, *J* = 10.6 Hz), 36.35, 36.02, 30.03, 29.97, 25.64 (d, *J* = 1.8 Hz), 22.58, 22.52, 22.51, 22.35, 15.21 (d, *J* = 52.0 Hz). **^{31}P NMR** (243 MHz, Chloroform-*d*) δ 61.44. The enantiomeric excess was determined by Daicel Chiralpak IE, *n*-hexane/isopropanol = 80/20, 1 mL/min, λ = 254 nm, *t* (major) = 10.66 min, *t* (minor) = 12.22 min. $[\alpha]_D^{25}$ = 222.8 (c = 0.884, CH_2Cl_2). **HRMS (ESI)** calcd for: $C_{41}H_{46}PS^+$ $[M + H]^+$ 601.3052; found: 601.3060.

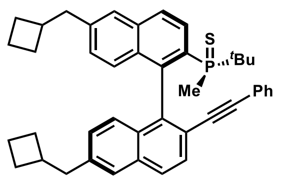

(**3ai**) Yellow solid, R_f = 0.50 (petroleum ether/ethyl acetate = 20:1), 91% yield, 94% ee, M.p. 78-79 °C. **^{1}H NMR** (600 MHz, Chloroform-*d*) δ 8.54 (dd, *J* = 12.1, 9.0 Hz, 1H), 7.98 (d, *J* = 8.7 Hz, 1H), 7.89 (d, *J* = 8.5 Hz, 1H), 7.68 (d, *J* = 8.5 Hz, 1H), 7.65 (s, 1H), 7.61 (s, 1H), 7.21-7.03 (m, 7H), 6.67 (d, *J* = 7.4 Hz, 2H), 2.82 (t, *J* = 6.6 Hz, 4H), 2.70-2.53 (m, 2H), 2.13-1.92 (m, 4H), 1.90-1.78 (m, 4H), 1.77-1.66 (m, 4H), 1.16 (d, *J* = 16.5 Hz, 9H), 0.71 (d, *J* = 12.9 Hz, 3H). **^{13}C NMR** δ 141.11, 140.68, 139.45 (d, *J* = 3.3 Hz), 139.14 (d, *J* = 4.9 Hz), 134.55 (d, *J* = 1.9 Hz), 133.39, 132.73, 130.97, 130.89 (d, *J* = 10.8 Hz), 130.79 (d, *J* = 12.8 Hz), 129.68, 128.70, 128.40, 128.14, 128.06, 127.99, 127.34, 126.90, 126.82, 126.56, 126.38, 126.24, 122.80, 121.27, 95.39, 90.09, 43.01, 42.93, 36.98, 36.86, 36.14 (d, *J* = 50.0 Hz), 28.41, 28.40, 28.28, 28.23, 25.61 (d, *J* = 1.7 Hz), 18.41, 18.39, 15.17 (d, *J* = 52.1 Hz). **^{31}P NMR** (243 MHz, Chloroform-*d*) δ 61.37. The enantiomeric excess was determined by Daicel Chiralpak ID, *n*-hexane/isopropanol = 95/5, 1 mL/min, λ = 254 nm, *t* (minor) = 12.87 min, *t* (major) = 20.16 min. $[\alpha]_D^{25}$ = 205.2 (c = 0.101, CH_2Cl_2). **HRMS (ESI)** calcd for: $C_{43}H_{46}PS^+$ $[M + H]^+$ 625.3052; found: 625.3060.

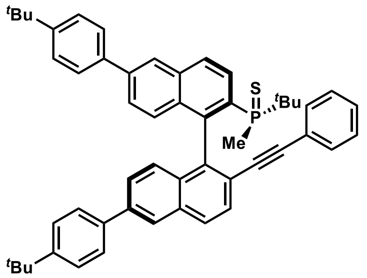

(**3aj**) Browm solid, R_f = 0.39 (petroleum ether/ethyl acetate = 20:1), 80% yield, 85% ee, M.p. 187-188 °C. **^{1}H NMR** (600 MHz, Chloroform-*d*) δ 8.68-8.58 (m, 1H), 8.18-8.08 (m, 3H), 8.03 (d, *J* = 8.4 Hz, 1H), 7.76 (d, *J* = 8.5 Hz, 1H), 7.68 (d, *J* = 8.7 Hz, 1H), 7.66-7.60 (m, 4H), 7.58 (d, *J* = 8.9 Hz, 1H), 7.53-7.44 (m, 4H), 7.34 (d, *J* = 8.7 Hz, 1H), 7.31 (d, *J* = 8.7 Hz, 1H), 7.14 (t, *J* = 7.2 Hz, 1H), 7.12-7.05 (m, 2H), 6.74 (d, *J* = 7.4 Hz, 2H), 1.36 (s, 9H), 1.35 (s, 9H), 1.21 (d, *J* = 16.5 Hz, 9H), 0.84 (d, *J* = 12.8 Hz, 3H). **^{13}C NMR** (151 MHz, Chloroform-*d*) δ 150.97, 150.94, 140.28, 139.81, 139.29 (d, *J* = 3.0 Hz), 139.05 (d, *J* = 4.9 Hz), 137.35, 137.21, 134.75 (d, *J* = 1.6 Hz), 133.88, 132.95, 131.49 (d, *J* = 10.4 Hz), 131.23 (d, *J* = 12.6 Hz), 131.07, 129.39 (d, *J* = 67.2 Hz), 129.21, 128.56, 128.33, 128.20, 128.00, 127.75, 127.63 (d, *J* = 12.2 Hz), 127.05, 126.87, 126.70, 125.96, 125.92, 125.53, 125.26, 122.66, 122.01, 96.01, 89.99, 36.26 (d, *J* = 50.0 Hz), 34.64, 34.63, 31.41, 31.40, 25.69, 15.37 (d, *J* = 52.1 Hz). **^{31}P NMR** (243 MHz, Chloroform-*d*) δ 61.50. The enantiomeric excess was determined by Daicel Chiralpak IA, *n*-hexane/isopropanol = 90/10, 1 mL/min, λ = 254 nm, *t* (minor) = 10.45 min, *t* (major) = 21.80 min. $[\alpha]_D^{25}$ = 134.7 (c = 0.107, CH_2Cl_2). **HRMS (ESI)** calcd for: $C_{53}H_{54}PS^+$ [M +

Na]$^+$ 775.3498; found: 775.3517.

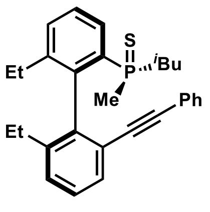

(**3ak**) Colorless oil, R_f = 0.55 (petroleum ether/ethyl acetate = 20:1), 41% yield, 83% ee. **^{1}H NMR** (600 MHz, Chloroform-*d*) δ 8.46-8.33 (m, 1H), 7.60-7.46 (m, 3H), 7.41 (t, *J* = 7.7 Hz, 1H), 7.36 (d, *J* = 7.7 Hz, 1H), 7.25-7.15 (m, 3H), 6.97 (d, *J* = 6.9 Hz, 2H), 2.45 (td, *J* = 15.2, 7.5 Hz, 1H), 2.34-2.16 (m, 3H), 2.07-1.95 (m, 2H), 1.95-1.84 (m, 1H), 1.24 (d, *J* = 13.3 Hz, 3H), 1.19-1.06 (m, 6H), 0.79 (d, *J* = 6.1 Hz, 3H), 0.75 (d, *J* = 6.1 Hz, 3H). **^{13}C NMR** (151 MHz, Chloroform-*d*) δ 143.79, 143.25 (d, *J* = 8.9 Hz), 141.45 (d, *J* = 3.1 Hz), 139.64 (d, *J* = 7.0 Hz), 132.91 (d, *J* = 73.2 Hz), 132.21 (d, *J* = 13.2 Hz), 131.39, 131.14 (d, *J* = 2.8 Hz), 129.53, 128.76, 128.48, 128.37, 128.22, 128.10 (d, *J* = 13.6 Hz), 124.08, 122.86, 94.43, 89.36, 44.97 (d, *J* = 51.9 Hz), 26.70, 26.00, 24.68 (d, *J* = 10.3 Hz), 24.51 (d, *J* = 3.7 Hz), 24.38 (d, *J* = 8.1 Hz), 21.14 (d, *J* = 54.2 Hz), 14.60, 13.55. **^{31}P NMR** (243 MHz, Chloroform-*d*) δ 43.46. The enantiomeric excess was determined by Daicel Chiralpak AS-H, *n*-hexane/isopropanol = 90/10, 1 mL/min, λ = 230 nm, t (minor) = 6.93 min, t (major) = 8.33 min. $[\alpha]_D^{25}$ = -73.9 (c = 0.138, CH_2Cl_2). **HRMS (ESI)** calcd for: $C_{29}H_{33}NaPS^+$ [M + Na]$^+$ 467.1933; found: 467.1942.

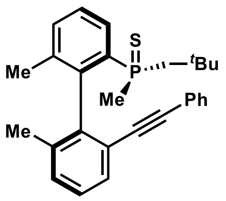

(**3al**) Yellow oil, R_f = 0.43 (petroleum ether/ethyl acetate = 20:1), 55% yield, 93% ee. **^{1}H NMR** (600 MHz, Chloroform-*d*) δ 8.57 (dd, *J* = 16.0, 7.6 Hz, 1H), 7.55-7.43 (m, 3H), 7.34 (t, *J* = 7.6 Hz, 1H), 7.28 (d, *J* = 7.7 Hz, 1H), 7.25-7.18 (m, 3H), 6.98 (d, *J* = 7.0 Hz, 2H), 2.27 (dd, *J* = 15.2, 10.0 Hz, 1H), 2.04-1.93 (m, 7H), 1.39 (d, *J* = 13.3 Hz, 3H), 0.94 (s, 9H). **^{13}C NMR** (151 MHz, Chloroform-*d*) δ 142.36, 140.13 (d, *J* = 7.3 Hz), 138.08, 137.22 (d, *J* = 9.0 Hz), 133.86 (d, *J* = 72.6 Hz), 133.18, 133.15, 133.09, 131.48, 130.65, 129.85, 128.53, 128.39, 127.97 (d, *J* = 13.7 Hz), 123.83, 122.83, 94.17, 88.95, 48.58 (d, *J* = 50.0 Hz), 32.83 (d, *J* = 4.5 Hz), 31.29 (d, *J* = 7.1 Hz), 22.80 (d, *J* = 53.3 Hz), 20.77, 20.21. **^{31}P NMR** (243 MHz, Chloroform-*d*) δ 41.26. The enantiomeric excess was determined by Daicel Chiralpak IC, *n*-hexane/isopropanol = 95/5, 1 mL/min, λ = 230 nm, t (minor) = 14.81 min, t (major) = 18.18 min. $[\alpha]_D^{25}$ = -48.7 (c = 0.191, CH_2Cl_2). **HRMS (ESI)** calcd for: $C_{28}H_{31}PSNa^+$ [M + Na]$^+$ 453.1776; found: 453.1776.

3.3. Enantioselective silylation of C-P bond

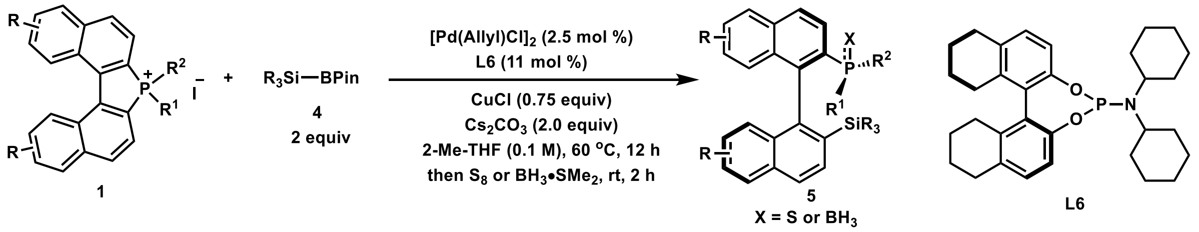

Typical Procedure: To a 10 mL Schlenk tube containing anhydrous Cs_2CO_3 (0.4 mmol, 2 equiv) was added phosphonium salts **1** (0.2 mmol), CuCl (0.15 mmol, 0.75 equiv), $[Pd(allyl)Cl]_2$ (0.005 mmol, 2.5 mol %), chiral phosphine ligand **L** (0.022 mmol, 11 mol %), 2-Me-THF (2 mL, 0.1 M), and R_3Si-BPin (0.4 mmol, 2 equiv) sequentially under nitrogen. The Schlenk tube was then sealed and stirred for 12 h at 60 ℃. The reaction mixture was cooled to rt, S_8 (5 equiv, 1 mmol) or BH_3•SMe_2 (2 equiv, 10 M in Me_2S) was added and stirred for 2 h at rt. The reaction mixture was

then filtered through a pad of celite eluenting with CH_2Cl_2/EtOAc (20 mL). The filtrate was concentrated, and the residue was purified by silica gel chromatography to afford the corresponding product.

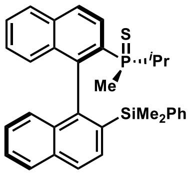

(5a) Yellow solid, R_f = 0.40 (petroleum ether/ethyl acetate = 20:1), 74% yield, 89% ee, M.p. 63 °C. **^{1}H NMR** (600 MHz, Chloroform-*d*) δ 8.59 (dd, *J* = 12.8, 8.8 Hz, 1H), 8.06 (d, *J* = 8.7 Hz, 1H), 7.96 (d, *J* = 8.4 Hz, 1H), 7.93 (d, *J* = 8.1 Hz, 1H), 7.87 (d, *J* = 8.2 Hz, 1H), 7.84 (d, *J* = 8.4 Hz, 1H), 7.53 (t, *J* = 7.4 Hz, 1H), 7.46 (t, *J* = 7.5 Hz, 1H), 7.36 (d, *J* = 7.7 Hz, 2H), 7.30 (d, *J* = 7.0 Hz, 1H), 7.28-7.20 (m, 4H), 7.17 (d, *J* = 8.5 Hz, 1H), 7.08 (d, *J* = 8.6 Hz, 1H), 2.10-1.93 (m, 1H), 0.98 (dd, *J* = 18.3, 6.8 Hz, 3H), 0.84 (dd, *J* = 18.4, 6.9 Hz, 3H), 0.79 (d, *J* = 13.0 Hz, 3H), -0.14 (s, 3H), -0.25 (s, 3H). **^{13}C NMR** (151 MHz, Chloroform-*d*) δ 142.61 (d, *J* = 3.6 Hz), 142.30 (d, *J* = 6.9 Hz), 138.74, 136.47, 134.68, 134.40 (d, *J* = 2.4 Hz), 134.35, 134.23, 134.17, 133.34, 132.08, 130.11 (d, *J* = 12.7 Hz), 129.22 (d, *J* = 70.6 Hz), 129.12, 128.05, 128.03, 127.97, 127.87, 127.79, 127.75, 127.19, 127.18, 127.00, 30.86 (d, *J* = 52.8 Hz), 18.52 (d, *J* = 54.6 Hz), 16.24 (d, *J* = 1.0 Hz), 16.11 (d, *J* = 1.3 Hz), -0.56, -1.66. **^{31}P NMR** (243 MHz, Chloroform-*d*) δ 54.38. The enantiomeric excess was determined by Daicel Chiralpak IA, *n*-hexane/isopropanol = 97.5/2.5, 1 mL/min, λ = 254 nm, *t* (minor) = 9.88 min, *t* (major) = 12.82 min. $[\alpha]_D^{25}$ = -7.1 (c = 0.103, CH_2Cl_2). **HRMS (ESI)** calcd for: $C_{32}H_{33}NaPSSi^+$ [M + Na]$^+$ 531.1702; found: 531.1704.

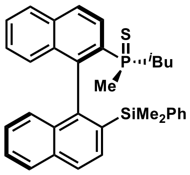

(5b) Yellow oil, R_f = 0.34 (petroleum ether/ethyl acetate = 20:1), 79% yield, 93% ee, **^{1}H NMR** (600 MHz, Chloroform-*d*) δ 8.62 (dd, *J* = 13.6, 8.8 Hz, 1H), 8.07 (d, *J* = 8.8 Hz, 1H), 7.98-7.92 (m, 1H), 7.89 (t, *J* = 7.6 Hz, 2H), 7.77 (d, *J* = 8.4 Hz, 1H), 7.51-7.42 (m, 2H), 7.30-7.22 (m, 4H), 7.20 (t, *J* = 7.2 Hz, 2H), 7.18-7.13 (m, 1H), 7.13-7.04 (m, 2H), 2.12-2.03 (m, 1H), 1.51 (td, *J* = 14.8, 4.8 Hz, 1H), 1.21 (d, *J* = 12.8 Hz, 3H), 0.90-0.85 (m, 1H), 0.85-0.80 (m, 3H), 0.62 (d, *J* = 6.6 Hz, 3H), 0.03 (s, 3H), -0.22 (s, 3H). **^{13}C NMR** (151 MHz, Chloroform-*d*) δ 142.61 (d, *J* = 3.5 Hz), 140.60 (d, *J* = 7.2 Hz), 138.40, 137.79, 134.23 (d, *J* = 2.4 Hz), 134.13, 133.77, 133.61 (d, *J* = 11.0 Hz), 133.52, 132.41 (d, *J* = 74.2 Hz), 131.73, 129.02, 128.75, 128.66, 128.09, 127.94, 127.91, 127.80, 127.74, 127.08, 126.94, 126.90, 126.85, 41.79 (d, *J* = 51.2 Hz), 25.19 (d, *J* = 11.6 Hz), 24.12 (d, *J* = 3.4 Hz), 24.08 (d, *J* = 6.8 Hz), 22.88 (d, *J* = 53.6 Hz), -1.20, -1.40. **^{31}P NMR** (243 MHz, Chloroform-*d*) δ 44.49. The enantiomeric excess was determined by Daicel Chiralpak IE, *n*-hexane/isopropanol = 90/10, 1 mL/min, λ = 254 nm, *t* (major) = 9.14 min, *t* (minor) = 9.89 min. $[\alpha]_D^{25}$ = 23.0 (c = 0.618, CH_2Cl_2). **HRMS (ESI)** calcd for: $C_{33}H_{35}NaSiPS^+$ [M + Na]$^+$ 545.1859; found: 545.1866.

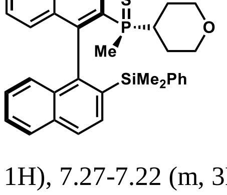

(5c) Yellow solid, R_f = 0.22 (petroleum ether/ethyl acetate = 5:1), 94% yield, 93% ee, M.p. 83-84 °C. **^{1}H NMR** (600 MHz, Chloroform-*d*) δ 8.34 (dd, *J* = 12.4, 8.8 Hz, 1H), 8.04 (d, *J* = 8.9 Hz, 1H), 8.00 (d, *J* = 8.4 Hz, 1H), 7.93 (d, *J* = 8.2 Hz, 1H), 7.90 (d, *J* = 8.2 Hz, 1H), 7.84 (d, *J* = 8.4 Hz, 1H), 7.53 (t, *J* = 7.4 Hz, 1H), 7.48 (t, *J* = 7.4 Hz, 1H), 7.37-7.32 (m, 2H), 7.29 (d, *J* = 7.2 Hz, 1H), 7.27-7.22 (m, 3H), 7.22-7.17 (m, 1H), 7.14 (d, *J* = 8.6 Hz, 1H), 7.08 (d, *J* = 8.5 Hz, 1H), 3.87-3.79 (m, 1H), 3.77-3.67 (m, 1H), 2.84 (t, *J* = 10.9 Hz, 1H), 2.68 (t, *J* = 11.6 Hz, 1H), 1.87-1.70 (m, 2H), 1.69-1.57 (m, 1H), 1.51 (d, *J* = 12.9 Hz, 1H), 1.13 (d, *J* = 13.0 Hz, 1H), 1.03 (d, *J* = 12.9 Hz, 3H), -0.15 (s, 6H). **^{13}C NMR** (151 MHz, Chloroform-*d*) δ 142.60 (d, *J* = 7.3 Hz), 142.20 (d, *J* = 4.0 Hz), 138.76, 136.61, 134.43, 134.37 (d, *J* = 2.3 Hz), 134.32, 134.22 (d, *J* =

10.9 Hz), 133.46, 132.03, 129.22, 129.20, 129.14, 128.69 (d, J = 6.7 Hz), 128.22, 128.15, 128.02, 127.99, 127.98, 127.94, 127.92, 127.78, 127.12, 126.96 (d, J = 9.3 Hz), 67.43 (d, J = 14.1 Hz), 66.92 (d, J = 13.5 Hz), 37.78 (d, J = 53.9 Hz), 25.95 (d, J = 3.4 Hz), 25.37 (d, J = 2.9 Hz), 17.74 (d, J = 55.1 Hz), -0.45, -1.14. **^{31}P NMR** (243 MHz, Chloroform-*d*) δ 48.63. The enantiomeric excess was determined by Daicel Chiralpak IE, *n*-hexane/isopropanol = 70/30, 1 mL/min, λ = 254 nm, *t* (major) = 12.56 min, *t* (minor) = 17.09 min. $[\alpha]_D^{25}$ = 32.8 (c = 0.347, CH_2Cl_2). **HRMS (ESI)** calcd for: $C_{34}H_{35}ONaSiPS^+$ $[M + Na]^+$ 573.1808; found: 573.1821.

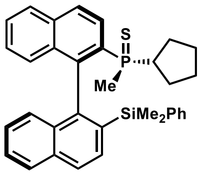

(5d) White solid, R_f = 0.40 (petroleum ether/ethyl acetate = 20:1), 69% yield, 83% ee, M.p. 72-73 °C. **^{1}H NMR** (600 MHz, Chloroform-*d*) δ 8.64 (dd, J = 13.0, 8.8 Hz, 1H), 8.05 (d, J = 8.7 Hz, 1H), 7.98 (d, J = 8.4 Hz, 1H), 7.92 (d, J = 8.2 Hz, 1H), 7.88 (d, J = 8.2 Hz, 1H), 7.84 (d, J = 8.4 Hz, 1H), 7.51 (t, J = 7.3 Hz, 1H), 7.47 (t, J = 7.2 Hz, 1H), 7.38-7.33 (m, 2H), 7.32-7.28 (m, 1H), 7.28-7.23 (m, 3H), 7.21 (t, J = 7.4 Hz, 1H), 7.14 (d, J = 8.1 Hz, 1H), 7.13 (d, J = 8.3 Hz, 1H), 2.09-1.99 (m, 1H), 1.99-1.89 (m, 1H), 1.67-1.50 (m, 3H), 1.48-1.38 (m, 1H), 1.36-1.28 (m, 1H), 1.28-1.20 (m, 1H), 1.19-1.11 (m, 1H), 0.75 (d, J = 13.1 Hz, 3H), -0.16 (s, 3H), -0.28 (s, 3H). **^{13}C NMR** (151 MHz, Chloroform-*d*) δ 142.98 (d, J = 3.6 Hz), 141.28 (d, J = 6.8 Hz), 138.63, 135.87, 134.73, 134.35, 134.13, 134.01 (d, J = 10.8 Hz), 133.34, 132.09, 130.63 (d, J = 71.5 Hz), 129.94 (d, J = 13.1 Hz), 129.15, 128.07, 127.99, 127.97, 127.95, 127.91, 127.86, 127.74, 127.25, 127.19, 127.05, 126.97, 41.36 (d, J = 54.4 Hz), 26.20 (d, J = 10.7 Hz), 26.20 (d, J = 10.7 Hz), 25.81 (d, J = 10.9 Hz), 19.66 (d, J = 55.6 Hz), -0.34, -1.82. **^{31}P NMR** (243 MHz, Chloroform-*d*) δ 51.84. The enantiomeric excess was determined by Daicel Chiralpak IE, *n*-Hexanes/isopropanol = 70/30, 1 mL/min, λ = 254 nm, *t* (major) = 7.36 min, *t* (minor) = 8.45 min. $[\alpha]_D^{25}$ = 63.9 (c = 0.195, CH_2Cl_2). **HRMS (ESI)** calcd for: $C_{34}H_{35}NaSiPS^+$ $[M + Na]^+$ 557.1859; found: 557.1877.

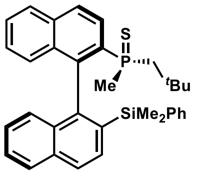

(5e) Light yellow solid, R_f = 0.51 (petroleum ether/ethyl acetate = 20:1), 71% yield, 94% ee, M.p. 49 °C. **^{1}H NMR** (600 MHz, Chloroform-*d*) δ 8.61 (dd, J = 13.6, 8.9 Hz, 1H), 8.07 (d, J = 8.8 Hz, 1H), 7.95 (d, J = 8.4 Hz, 1H), 7.89 (d, J = 8.0 Hz, 1H), 7.88 (d, J = 8.1 Hz, 1H), 7.77 (d, J = 8.4 Hz, 1H), 7.51-7.43 (m, 2H), 7.29-7.23 (m, 4H), 7.22-7.17 (m, 2H), 7.17-7.12 (m, 1H), 7.09 (t, J = 9.2 Hz, 2H), 1.67 (t, J = 14.6 Hz, 1H), 1.33 (d, J = 12.8 Hz, 3H), 1.12 (dd, J = 14.7, 8.7 Hz, 1H), 0.89 (s, 9H), 0.05 (s, 3H), -0.24 (s, 3H). **^{13}C NMR** (151 MHz, Chloroform-*d*) δ 142.91 (d, J = 3.3 Hz), 140.23 (d, J = 6.9 Hz), 138.53, 137.72, 134.17, 134.13 (d, J = 2.4 Hz), 133.96, 133.90, 133.66, 133.59, 133.48, 131.82, 129.02, 128.78 (d, J = 12.5 Hz), 128.48 (d, J = 13.8 Hz), 128.20, 128.12, 127.87, 127.80, 127.72, 127.07, 126.96, 126.91, 126.89, 45.36 (d, J = 49.7 Hz), 32.67 (d, J = 4.4 Hz), 31.25 (d, J = 7.2 Hz), 25.00 (d, J = 53.3 Hz), -1.05, -1.36. **^{31}P NMR** (243 MHz, Chloroform-*d*) δ 41.35. The enantiomeric excess was determined by Daicel Chiralpak ID, *n*-hexane/isopropanol = 90/10, 1 mL/min, λ = 254 nm, *t* (minor) = 6.19 min, *t* (major) = 6.99 min. $[\alpha]_D^{25}$ = 24.6 (c = 0.134, CH_2Cl_2). **HRMS (ESI)** calcd for: $C_{34}H_{37}NaSiPS^+$ $[M + Na]^+$ 559.2015; found: 559.2030.

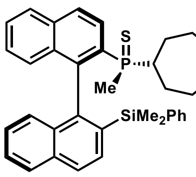

(5f) White solid, R_f = 0.29 (petroleum ether/ethyl acetate = 20:1), 91% yield, 92% ee, M.p. 72 °C. **^{1}H NMR** (600 MHz, Chloroform-*d*) δ 8.27 (dd, *J* = 11.8, 8.9 Hz, 1H), 8.04 (d, *J* = 8.7 Hz, 1H), 7.95 (d, *J* = 8.4 Hz, 1H), 7.91 (d, *J* = 8.2 Hz, 1H), 7.87 (d, *J* = 8.2 Hz, 1H), 7.79 (d, *J* = 8.4 Hz, 1H), 7.52 (t, *J* = 7.7 Hz, 1H), 7.47 (t, *J* = 7.5 Hz, 1H), 7.35-7.31 (m, 2H), 7.31-7.21 (m, 4H), 7.19 (t, *J* = 7.3 Hz, 1H), 7.16 (d, *J* = 8.4 Hz, 2H), 2.10-1.99 (m, 1H), 1.95-1.85 (m, 1H), 1.73-1.59 (m, 2H), 1.44-1.35 (m, 5H), 1.34-1.24 (m, 2H), 1.23-1.11 (m, 2H), 0.93 (d, *J* = 12.7 Hz, 3H), -0.04 (s, 3H), -0.28 (s, 3H). **^{13}C NMR** (151 MHz, Chloroform-*d*) δ 142.58 (d, *J* = 6.8 Hz), 142.35 (d, *J* = 3.8 Hz), 139.02, 136.83, 134.37 (d, *J* = 10.9 Hz), 134.31, 134.21, 133.46, 131.94, 130.03 (d, *J* = 71.9 Hz), 129.00, 128.68 (d, *J* = 11.9 Hz), 128.17 (d, *J* = 11.9 Hz), 128.08, 128.01, 127.91, 127.79, 127.64, 127.51, 127.10, 127.00, 126.88, 42.10 (d, *J* = 49.4 Hz), 29.06, 27.92 (d, *J* = 15.7 Hz), 27.57 (d, *J* = 15.8 Hz), 27.22 (d, *J* = 15.5 Hz), 27.07, 17.31 (d, *J* = 53.2 Hz), -0.78, -1.07. **^{31}P NMR** (243 MHz, Chloroform-*d*) δ 53.26. The enantiomeric excess was determined by Daicel Chiralpak IE, *n*-hexane/isopropanol = 90/10, 1 mL/min, λ = 254 nm, *t* (major) = 15.28 min, *t* (minor) = 20.48 min. $[\alpha]_D^{25}$ = 9.7 (c = 0.339, CH_2Cl_2). **HRMS (ESI)** calcd for: $C_{36}H_{39}NaSiPS^+$ $[M + Na]^+$ 585.2172; found: 585.2186.

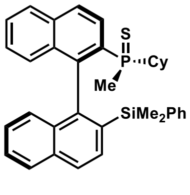

(5g) White solid, R_f = 0.32 (petroleum ether/ethyl acetate = 20:1), 68% yield, 93% ee, M.p. 60-61 °C. **^{1}H NMR** (600 MHz, Chloroform-*d*) δ 8.45 (dd, *J* = 12.4, 8.8 Hz, 1H), 8.03 (d, *J* = 8.7 Hz, 1H), 7.96 (d, *J* = 8.4 Hz, 1H), 7.91 (d, *J* = 8.2 Hz, 1H), 7.87 (d, *J* = 8.2 Hz, 1H), 7.82 (d, *J* = 8.3 Hz, 1H), 7.50 (t, *J* = 7.4 Hz, 1H), 7.46 (t, *J* = 7.5 Hz, 1H), 7.35-7.30 (m, 2H), 7.30-7.26 (m, 1H), 7.26-7.21 (m, 3H), 7.19-7.14 (m, 1H), 7.12 (d, *J* = 8.5 Hz, 1H), 7.09 (d, *J* = 8.6 Hz, 1H), 1.78-1.71 (m, 1H), 1.70-1.62 (m, 2H), 1.59 (d, *J* = 10.9 Hz, 1H), 1.51 (d, *J* = 14.4 Hz, 1H), 1.49-1.38 (m, 2H), 1.34-1.23 (m, 1H), 1.13-1.03 (m, 1H), 0.91 (d, *J* = 12.9 Hz, 3H), 0.88-0.77 (m, 2H), -0.02 (s, 3H), -0.26 (s, 3H). **^{13}C NMR** (151 MHz, Chloroform-*d*) δ 142.52 (d, *J* = 3.7 Hz), 142.26 (d, *J* = 7.0 Hz), 138.87, 136.49, 134.57, 134.30, 134.28, 134.09 (d, *J* = 10.7 Hz), 133.41, 131.99, 129.82 (d, *J* = 12.4 Hz), 129.23 (d, *J* = 71.0 Hz), 129.05, 128.07, 128.01, 127.92, 127.83, 127.77, 127.69, 127.08, 127.07, 126.99, 126.93, 40.16 (d, *J* = 51.8 Hz), 25.83, 25.74, 25.60 (d, *J* = 0.7 Hz), 25.46 (d, *J* = 2.1 Hz), 18.11 (d, *J* = 54.5 Hz), -0.92, -1.07. **^{31}P NMR** (243 MHz, Chloroform-*d*) δ 50.70. The enantiomeric excess was determined by Daicel Chiralpak IE, *n*-hexane/isopropanol = 90/10, 1 mL/min, λ = 254 nm, *t* (major) = 13.55 min, *t* (minor) = 16.64 min. $[\alpha]_D^{25}$ = 22.8 (c = 0.215, CH_2Cl_2). **HRMS (ESI)** calcd for: $C_{35}H_{37}NaSiPS^+$ $[M + Na]^+$ 571.2015; found: 571.2029.

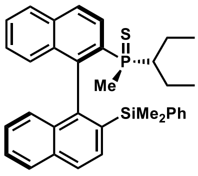

(5h) Light yellow solid, R_f = 0.43 (petroleum ether/ethyl acetate = 20:1), 84% yield, 90% ee, M.p. 50-51 °C. **^{1}H NMR** (600 MHz, Chloroform-*d*) δ 8.38 (dd, *J* = 12.2, 8.8 Hz, 1H), 8.05 (d, *J* = 8.0 Hz, 1H), 7.95 (d, *J* = 8.3 Hz, 1H), 7.91 (d, *J* = 8.2 Hz, 1H), 7.86 (d, *J* = 8.2 Hz, 1H), 7.80 (d, *J* = 8.4 Hz, 1H), 7.53-7.48 (m, 1H), 7.45 (t, *J* = 7.3 Hz, 1H), 7.34-7.20 (m, 6H), 7.20-7.12 (m, 3H), 1.80-1.63 (m, 2H), 1.61-1.46 (m, 2H), 1.32-1.21 (m, 1H), 0.96 (d, *J* = 12.8 Hz, 3H), 0.80 (t, *J* = 7.4 Hz, 6H), 0.02 (s, 3H), -0.34 (s, 3H). **^{13}C NMR** (151 MHz, Chloroform-*d*) δ 142.36 (d, *J* = 4.2 Hz), 142.34 (d, *J* = 3.7 Hz), 138.92, 136.79, 134.33, 134.24, 134.19 (d, *J* = 2.3 Hz), 134.16, 133.40, 131.90, 130.12 (d, *J* = 71.3 Hz), 129.12 (d, *J* = 12.4 Hz), 128.96, 128.10, 128.08, 127.99, 127.91, 127.88, 127.72, 127.61, 127.34, 127.04, 126.95, 126.85, 43.22 (d, *J* = 50.1 Hz), 21.71, 20.73, 18.63 (d, *J* = 53.2 Hz), 12.80 (d, *J* = 10.0 Hz), 12.48 (d, *J* = 12.4 Hz), -1.12. **^{31}P NMR** (243

MHz, Chloroform-*d*) δ 52.47. The enantiomeric excess was determined by Daicel Chiralpak IE, *n*-hexane/isopropanol = 90/10, 1 mL/min, λ = 254 nm, *t* (major) = 11.52 min, *t* (minor) = 15.10 min. $[\alpha]_D^{25}$ = -13.1 (c = 0.221, CH_2Cl_2). **HRMS (ESI)** calcd for: $C_{34}H_{37}NaSiPS^+$ $[M + Na]^+$ 559.2015; found: 559.2031.

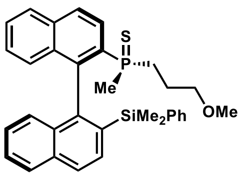

(5i) Light white oil, R_f = 0.33 (petroleum ether/ethyl acetate = 5:1), 92% yield, 95% ee, **^{1}H NMR** (600 MHz, Chloroform-*d*) δ 8.55 (dd, *J* = 13.5, 8.8 Hz, 1H), 8.07 (d, *J* = 8.8 Hz, 1H), 7.95 (d, *J* = 8.4 Hz, 1H), 7.93-7.86 (m, 2H), 7.78 (d, *J* = 8.4 Hz, 1H), 7.50 (t, *J* = 7.4 Hz, 1H), 7.46 (t, *J* = 7.4 Hz, 1H), 7.28-7.23 (m, 4H), 7.22-7.17 (m, 2H), 7.15 (t, *J* = 7.5 Hz, 1H), 7.09 (d, *J* = 8.2 Hz, 2H), 3.18-3.11 (m, 4H), 3.07-2.98 (m, 1H), 1.96-1.86 (m, 1H), 1.59-1.49 (m, 1H), 1.48-1.39 (m, 1H), 1.28-1.18 (m, 1H), 1.15 (d, *J* = 12.8 Hz, 3H), 0.08 (s, 3H), -0.25 (s, 3H). **^{13}C NMR** (151 MHz, Chloroform-*d*) δ 142.36 (d, *J* = 3.6 Hz), 140.98 (d, *J* = 7.4 Hz), 138.49, 137.67, 134.30 (d, *J* = 1.4 Hz), 134.13, 133.78, 133.61 (d, *J* = 11.1 Hz), 133.50, 131.73, 128.98, 128.74, 128.71, 128.65, 128.62, 128.11, 128.02, 127.92, 127.79, 127.75, 127.08, 126.96, 126.90, 126.85, 72.27 (d, *J* = 17.3 Hz), 58.35, 30.91 (d, *J* = 54.5 Hz), 23.18 (d, *J* = 2.6 Hz), 21.25 (d, *J* = 54.9 Hz), -1.23, -1.48. **^{31}P NMR** (243 MHz, Chloroform-*d*) δ 45.13. The enantiomeric excess was determined by Daicel Chiralpak IG, *n*-hexane/isopropanol = 80/20, 1 mL/min, λ = 254 nm, *t* (major) = 7.80 min, *t* (minor) = 8.57 min. $[\alpha]_D^{25}$ = 21.1 (c = 0.600, CH_2Cl_2). **HRMS (ESI)** calcd for: $C_{33}H_{35}ONaSiPS^+$ $[M + Na]^+$ 561.1808; found: 561.1819.

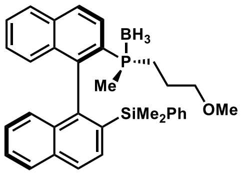

(5j) Colorless oil, R_f = 0.23 (petroleum ether/ethyl acetate = 20:1), 84% yield, 94% ee, **^{1}H NMR** (600 MHz, Chloroform-*d*) δ 8.03-7.97 (m, 2H), 7.95 (d, *J* = 8.4 Hz, 1H), 7.88 (d, *J* = 8.2 Hz, 2H), 7.79 (d, *J* = 8.4 Hz, 1H), 7.49-7.41 (m, 2H), 7.26-7.19 (m, 4H), 7.19-7.14 (m, 2H), 7.12-7.01 (m, 3H), 3.18 (s, 3H), 3.15-3.09 (m, 1H), 3.09-3.03 (m, 1H), 1.73-1.62 (m, 1H), 1.56-1.46 (m, 1H), 1.42-1.31 (m, 1H), 1.30-1.16 (m, 1H), 0.91 (d, *J* = 10.0 Hz, 3H), 0.83-0.15 (m, 3H), 0.08 (s, 3H), -0.25 (s, 3H). **^{13}C NMR** (151 MHz, Chloroform-*d*) δ 143.94 (d, *J* = 4.3 Hz), 142.26 (d, *J* = 3.6 Hz), 138.59, 137.44, 134.14, 134.09 (d, *J* = 1.9 Hz), 133.97, 133.93, 133.56, 131.61, 128.87, 128.72 (d, *J* = 13.9 Hz), 128.46 (d, *J* = 11.0 Hz), 128.10, 127.86, 127.85, 127.80, 127.74, 127.67, 127.54 (d, *J* = 49.2 Hz), 126.87, 126.86, 126.78, 126.51, 72.59 (d, *J* = 14.5 Hz), 58.40, 23.93 (d, *J* = 36.4 Hz), 23.64, 11.10 (d, *J* = 38.0 Hz), -1.04, -1.50. **^{31}P NMR** (243 MHz, Chloroform-*d*) δ 13.84 (d, *J* = 54.3 Hz). The enantiomeric excess was determined by Daicel Chiralpak IG, *n*-hexane/isopropanol = 95/5, 1 mL/min, λ = 254 nm, *t* (major) = 9.17 min, *t* (minor) = 10.30 min. $[\alpha]_D^{25}$ = 1.1 (c = 0.754, CH_2Cl_2). **HRMS (ESI)** calcd for: $C_{33}H_{38}ONaPBSi^+$ $[M + Na]^+$ 543.2415; found: 543.2418.

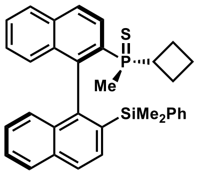

(5k) Yellow solid, R_f = 0.39 (petroleum ether/ethyl acetate = 20:1), 63% yield, 80% ee, M.p. 54-55 °C. **^{1}H NMR** (600 MHz, Chloroform-*d*) δ 8.32 (dd, *J* = 12.8, 8.8 Hz, 1H), 8.01 (d, *J* = 8.8 Hz, 1H), 7.98 (d, *J* = 8.4 Hz, 1H), 7.89 (d, *J* = 8.2 Hz, 2H), 7.84 (d, *J* = 8.4 Hz, 1H), 7.51-7.46 (m, 2H), 7.32-7.26 (m, 4H), 7.26-7.22 (m, 2H), 7.17 (d, *J* = 8.5 Hz, 1H), 7.13 (t, *J* = 7.7 Hz, 1H), 7.02 (d, *J* = 8.6 Hz, 1H), 2.67-2.55 (m, 1H), 2.53-2.44 (m, 1H), 2.23-2.11 (m, 1H), 1.84-1.70 (m, 2H), 1.70-1.61 (m, 1H), 1.55-1.47 (m, 1H), 0.73 (d, *J* = 12.9 Hz, 3H), -0.12 (s, 3H), -0.17 (s, 3H). **^{13}C NMR** (151 MHz, Chloroform-*d*) δ 142.86 (d, *J* = 3.9 Hz), 140.56 (d, *J* = 6.9 Hz), 138.58, 136.45, 134.26 (d, *J* = 2.3 Hz), 134.14, 133.82 (d, *J* = 10.8 Hz), 133.43, 131.84, 131.19 (d, *J* = 72.3 Hz), 129.11, 128.89 (d, *J* = 12.9 Hz), 128.39 (d, *J* = 12.2 Hz), 127.99, 127.95, 127.88,

127.87, 127.81, 127.29, 127.20, 127.10, 126.89, 36.24 (d, *J* = 50.1 Hz), 22.38 (d, *J* = 5.1 Hz), 22.11 (d, *J* = 4.1 Hz), 19.40 (d, *J* = 19.4 Hz), 18.16 (d, *J* = 55.7 Hz), -0.92, -1.47. **^{31}P NMR** (243 MHz, Chloroform-*d*) δ 47.92. The enantiomeric excess was determined by Daicel Chiralpak IG, *n*-hexane/isopropanol = 90/10, 1 mL/min, λ = 254 nm, *t* (major) = 9.03 min, *t* (minor) = 13.52 min. $[\alpha]_D^{25}$ = 53.6 (c = 0.153, CH_2Cl_2). **HRMS (ESI)** calcd for: $C_{33}H_{33}NaSiPS^+$ $[M + Na]^+$ 543.1702; found: 543.1718.

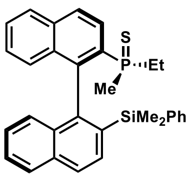

(5l) Light yellow solid, R_f = 0.24 (petroleum ether/ethyl acetate = 20:1), 98% yield, 95% ee, M.p. 50 °C. **^{1}H NMR** (600 MHz, Chloroform-*d*) δ 8.56 (dd, *J* = 13.3, 8.8 Hz, 1H), 8.07 (d, *J* = 8.7 Hz, 1H), 7.95 (d, *J* = 8.4 Hz, 1H), 7.91 (d, *J* = 8.3 Hz, 1H), 7.88 (d, *J* = 8.2 Hz, 1H), 7.78 (d, *J* = 8.4 Hz, 1H), 7.52-7.48 (m, 1H), 7.48-7.45 (m, 1H), 7.29-7.24 (m, 4H), 7.23-7.19 (m, 2H), 7.16 (t, *J* = 7.6 Hz, 1H), 7.10 (d, *J* = 8.5 Hz, 1H), 7.07 (d, *J* = 8.5 Hz, 1H), 1.61-1.50 (m, 1H), 1.30-1.20 (m, 1H), 1.09 (d, *J* = 12.9 Hz, 3H), 0.96 (dt, *J* = 20.2, 7.5 Hz, 3H), 0.02 (s, 3H), -0.23 (s, 3H). **^{13}C NMR** (151 MHz, Chloroform-*d*) δ 142.48 (d, *J* = 3.8 Hz), 141.04 (d, *J* = 7.4 Hz), 138.49, 137.50, 134.35 (d, *J* = 2.5 Hz), 134.14, 133.88, 133.73 (d, *J* = 11.0 Hz), 133.47, 131.80, 131.15 (d, *J* = 74.4 Hz), 129.04, 128.91 (d, *J* = 13.2 Hz), 128.62 (d, *J* = 12.4 Hz), 128.13, 128.09, 128.02, 127.96, 127.88, 127.81, 127.16, 126.97, 126.96, 27.46 (d, *J* = 54.0 Hz), 20.39 (d, *J* = 54.8 Hz), 7.08 (d, *J* = 4.4 Hz), -1.23, -1.29. **^{31}P NMR** (243 MHz, Chloroform-*d*) δ 47.20. The enantiomeric excess was determined by Daicel Chiralpak ID, *n*-hexane/isopropanol = 90/10, 1 mL/min, λ = 254 nm, *t* (minor) = 8.99 min, *t* (major) = 9.85 min. $[\alpha]_D^{25}$ = 8.16 (c = 0.147, CH_2Cl_2). **HRMS (ESI)** calcd for: $C_{31}H_{31}NaSiPS^+$ $[M + Na]^+$ 517.1546; found: 517.1558.

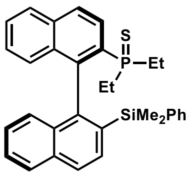

(5m) Brown solid, R_f = 0.36 (petroleum ether/ethyl acetate = 20:1), 79% yield, 96% ee, M.p. 53-54 °C. **^{1}H NMR** (600 MHz, Chloroform-*d*) δ 8.61 (dd, *J* = 12.9, 8.8 Hz, 1H), 8.05 (d, *J* = 8.8 Hz, 1H), 7.94 (d, *J* = 8.4 Hz, 1H), 7.89 (d, *J* = 8.2 Hz, 2H), 7.76 (d, *J* = 8.4 Hz, 1H), 7.50-7.43 (m, 2H), 7.28-7.15 (m, 6H), 7.11 (t, *J* = 7.6 Hz, 1H), 7.07-7.01 (m, 2H), 1.69-1.59 (m, 1H), 1.51-1.40 (m, 1H), 1.27-1.16 (m, 1H), 0.92-0.74 (m, 7H), 0.14 (s, 3H), -0.28 (s, 3H). **^{13}C NMR** (151 MHz, Chloroform-*d*) δ 142.32 (d, *J* = 3.5 Hz), 141.67 (d, *J* = 7.1 Hz), 138.53, 138.41, 134.26 (d, *J* = 2.3 Hz), 134.11, 133.70 (d, *J* = 10.9 Hz), 133.43, 131.84, 129.89, 129.81, 129.70 (d, *J* = 70.4 Hz), 128.88, 128.25, 128.11 (d, *J* = 12.1 Hz), 128.11, 127.94, 127.87, 127.80, 127.69, 126.94, 126.79, 126.75, 126.42, 25.79 (d, *J* = 52.5 Hz), 24.13 (d, *J* = 52.6 Hz), 7.54 (d, *J* = 4.0 Hz), 6.98 (d, *J* = 4.6 Hz), -1.08, -1.66. **^{31}P NMR** (243 MHz, Chloroform-*d*) δ 57.09. The enantiomeric excess was determined by Daicel Chiralpak IE, *n*-hexane/isopropanol = 90/10, 1 mL/min, λ = 254 nm, *t* (major) = 8.22 min, *t* (minor) = 8.83 min. $[\alpha]_D^{25}$ = -6.8 (c = 0.181, CH_2Cl_2). **HRMS (ESI)** calcd for: $C_{32}H_{33}NaSiPS^+$ $[M + Na]^+$ 531.1702; found: 531.1713.

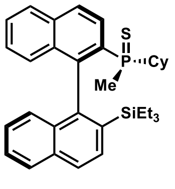

(5n) Yellow solid, R_f = 0.45 (petroleum ether/ethyl acetate = 20:1), 77% yield, 93% ee, M.p. 57 °C. **^{1}H NMR** (600 MHz, Chloroform-*d*) δ 8.46 (dd, *J* = 11.7, 9.4 Hz, 1H), 8.05 (d, *J* = 8.5 Hz, 1H), 7.96 (d, *J* = 8.2 Hz, 1H), 7.92 (d, *J* = 8.1 Hz, 1H), 7.87 (d, *J* = 8.1 Hz, 1H), 7.77 (d, *J* = 8.3 Hz, 1H), 7.52 (t, *J* = 7.3 Hz, 1H), 7.45 (t, *J* = 7.2 Hz, 1H), 7.28-7.21 (m, 2H), 7.16 (d, *J* = 8.5 Hz, 1H), 7.05 (d, *J* = 8.6 Hz, 1H), 1.91-1.69 (m, 4H), 1.65 (d, *J* = 10.5 Hz, 1H), 1.60-1.46 (m, 2H), 1.44-1.33 (m, 1H), 1.23-1.05 (m, 3H), 0.78-0.69 (m, 12H), 0.44-0.34 (m, 3H), 0.17-0.08 (m, 3H). **^{13}C NMR** (151 MHz, Chloroform-*d*) δ 142.63 (d, *J* = 3.8

Hz), 142.45 (d, J = 6.8 Hz), 137.11, 134.73, 134.21 (d, J = 2.3 Hz), 133.92 (d, J = 10.8 Hz), 133.15, 131.46, 129.92 (d, J = 12.6 Hz), 129.21 (d, J = 71.1 Hz), 128.25, 127.94, 127.84, 127.79, 127.56, 127.48, 127.33, 127.06, 126.81, 126.63, 40.30 (d, J = 51.9 Hz), 25.99 (d, J = 13.3 Hz), 25.90 (d, J = 13.5 Hz), 25.78 (d, J = 2.4 Hz), 25.66 (d, J = 1.4 Hz), 25.41 (d, J = 2.3 Hz), 17.70 (d, J = 54.5 Hz), 8.04, 4.27. **^{31}P NMR** (243 MHz, Chloroform-*d*) δ 50.79. The enantiomeric excess was determined by Daicel Chiralpak OD-H, *n*-hexane/isopropanol = 95/5, 1 mL/min, λ = 254 nm, *t* (major) = 5.19 min, *t* (minor) = 5.85 min. $[\alpha]_D^{25}$ = 77.7 (c = 0.208, CH_2Cl_2). **HRMS (ESI)** calcd for: $C_{33}H_{41}NaSiPS^+$ $[M + Na]^+$ 551.2328; found: 551.2341.

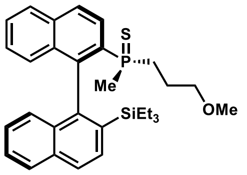

(5o) Colorless solid, R_f = 0.60 (petroleum ether/ethyl acetate = 5:1), 81% yield, 94% ee, M.p. 109-110 °C. **^{1}H NMR** (600 MHz, Chloroform-*d*) δ 8.58 (dd, J = 13.5, 8.8 Hz, 1H), 8.09 (d, J = 8.8 Hz, 1H), 7.98 (d, J = 8.3 Hz, 1H), 7.92 (d, J = 8.5 Hz, 1H), 7.90 (d, J = 8.6 Hz, 1H), 7.79 (d, J = 8.4 Hz, 1H), 7.52 (t, J = 7.4 Hz, 1H), 7.45 (t, J = 7.5 Hz, 1H), 7.27-7.21 (m, 2H), 7.15 (d, J = 8.6 Hz, 1H), 7.06 (d, J = 8.5 Hz, 1H), 3.26-3.20 (m, 1H), 3.18 (s, 3H), 3.13-3.07 (m, 1H), 2.01-1.90 (m, 1H), 1.66-1.53 (m, 1H), 1.53-1.42 (m, 1H), 1.29-1.19 (m, 1H), 1.14 (d, J = 12.8 Hz, 3H), 0.71 (t, J = 7.9 Hz, 9H), 0.40 (dq, J = 15.7, 7.8 Hz, 3H), 0.08 (dq, J = 15.7, 7.9 Hz, 3H). **^{13}C NMR** (151 MHz, Chloroform-*d*) δ 142.68 (d, J = 3.7 Hz), 141.20 (d, J = 7.4 Hz), 137.88, 134.25 (d, J = 2.4 Hz), 133.94, 133.63 (d, J = 11.2 Hz), 133.37, 131.71, 131.54, 131.21, 128.74 (d, J = 13.4 Hz), 128.52 (d, J = 12.5 Hz), 128.23, 128.00, 127.92, 127.37, 126.87, 126.79, 126.75, 126.70, 72.29 (d, J = 17.2 Hz), 58.31, 30.81 (d, J = 54.4 Hz), 23.21 (d, J = 2.8 Hz), 21.23 (d, J = 54.8 Hz), 7.83, 3.90. **^{31}P NMR** (243 MHz, Chloroform-*d*) δ 45.44. The enantiomeric excess was determined by Daicel Chiralpak IG, *n*-hexane/isopropanol = 95/5, 1 mL/min, λ = 254 nm, *t* (minor) = 14.08 min, *t* (major) = 15.25 min. $[\alpha]_D^{25}$ = 72.6 (c = 0.241, CH_2Cl_2). **HRMS (ESI)** calcd for: $C_{31}H_{39}ONaSiPS^+$ $[M + Na]^+$ 541.2121; found: 541.2129.

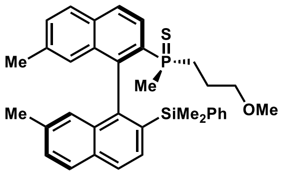

(5p) Brown solid, R_f = 0.50 (petroleum ether/ethyl acetate = 5:1), 90% yield, 88% ee, M.p. 40 °C. **^{1}H NMR** (600 MHz, Chloroform-*d*) δ 8.50 (dd, J = 13.6, 8.8 Hz, 1H), 8.03 (d, J = 8.5 Hz, 1H), 7.91 (d, J = 8.3 Hz, 1H), 7.81 (d, J = 8.3 Hz, 1H), 7.79 (d, J = 8.4 Hz, 1H), 7.72 (d, J = 8.3 Hz, 1H), 7.33 (d, J = 8.5 Hz, 1H), 7.31 (d, J = 8.5 Hz, 1H), 7.27-7.17 (m, 5H), 6.86 (d, J = 9.3 Hz, 2H), 3.19-3.15 (m, 4H), 3.10-3.03 (m, 1H), 2.23 (s, 3H), 2.08 (s, 3H), 1.96-1.86 (m, 1H), 1.58-1.51 (m, 1H), 1.52-1.41 (m, 1H), 1.32-1.22 (m, 1H), 1.10 (d, J = 12.9 Hz, 3H), 0.14 (s, 3H), -0.37 (s, 3H). **^{13}C NMR** (151 MHz, Chloroform-*d*) δ 141.69 (d, J = 3.8 Hz), 140.52 (d, J = 7.5 Hz), 138.80, 137.31, 136.75, 136.50, 133.94, 133.78 (d, J = 11.1 Hz), 132.58 (d, J = 2.3 Hz), 131.75, 130.90 (d, J = 75.3 Hz), 130.74, 130.24, 129.31, 128.81, 128.23 (d, J = 12.7 Hz), 127.85, 127.76 (d, J = 13.6 Hz), 127.68, 127.62, 127.40, 127.16, 125.84, 72.25 (d, J = 17.3 Hz), 58.29, 31.07 (d, J = 54.5 Hz), 23.21 (d, J = 2.8 Hz), 21.90, 21.74, 20.96 (d, J = 54.8 Hz), -1.06, -1.91. **^{31}P NMR** (243 MHz, Chloroform-*d*) δ 45.16. The enantiomeric excess was determined by Daicel Chiralpak IA, *n*-hexane/isopropanol = 95/5, 1 mL/min, λ = 254 nm, *t* (minor) = 7.44 min, *t* (major) = 15.18 min. $[\alpha]_D^{25}$ = 13.3 (c = 0.278, CH_2Cl_2). **HRMS (ESI)** calcd for: $C_{35}H_{39}ONaSiPS^+$ $[M + Na]^+$ 589.2121; found: 589.2123.

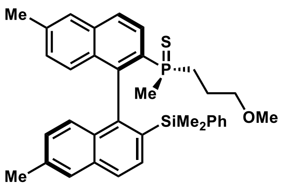

(5q) White oil, R_f = 0.44 (petroleum ether/ethyl acetate = 5:1), 86% yield, 93% ee, **^{1}H NMR** (600 MHz, Chloroform-*d*) δ 8.52 (dd, *J* = 13.4, 8.9 Hz, 1H), 7.96 (d, *J* = 8.8 Hz, 1H), 7.85 (d, *J* = 8.3 Hz, 1H), 7.73 (d, *J* = 8.3 Hz, 1H), 7.65 (d, *J* = 8.5 Hz, 2H), 7.28-7.22 (m, 3H), 7.19 (t, *J* = 7.2 Hz, 2H), 7.07 (d, *J* = 8.6 Hz, 1H), 6.99 (d, *J* = 8.6 Hz, 1H), 6.98-6.91 (m, 2H), 3.18-3.09 (m, 4H), 3.06-2.99 (m, 1H), 2.45 (s, 6H), 1.99-1.87 (m, 1H), 1.62-1.50 (m, 1H), 1.50-1.40 (m, 1H), 1.30-1.20 (m, 1H), 1.14 (d, *J* = 12.8 Hz, 3H), 0.06 (s, 3H), -0.24 (s, 3H). **^{13}C NMR** (151 MHz, Chloroform-*d*) δ 142.36 (d, *J* = 3.8 Hz), 140.85 (d, *J* = 7.5 Hz), 138.68, 137.97, 136.87, 136.16, 134.51 (d, *J* = 2.4 Hz), 134.10, 133.70, 132.08, 131.83 (d, *J* = 11.3 Hz), 131.73, 130.08 (d, *J* = 76.1 Hz), 129.10, 129.08, 128.81, 128.74 (d, *J* = 13.5 Hz), 127.91 (d, *J* = 12.6 Hz), 127.90, 127.66, 127.09, 127.02, 126.86, 126.69, 72.26 (d, *J* = 17.4 Hz), 58.30, 30.89 (d, *J* = 54.5 Hz), 23.15 (d, *J* = 2.8 Hz), 21.66, 21.63, 21.19 (d, *J* = 54.9 Hz), -1.25, -1.45. **^{31}P NMR** (243 MHz, Chloroform-*d*) δ 45.07. The enantiomeric excess was determined by Daicel Chiralpak IG, *n*-hexane/isopropanol = 95/5, 1 mL/min, λ = 254 nm, *t* (major) = 20.85 min, *t* (minor) = 29.81 min. $[\alpha]_D^{25}$ = 8.0 (c = 0.199, CH_2Cl_2). **HRMS (ESI)** calcd for: $C_{35}H_{39}ONaSiPS^+$ $[M + Na]^+$ 589.2121; found: 589.2127.

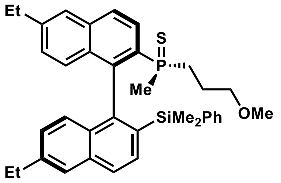

(5r) White solid, R_f = 0.51 (petroleum ether/ethyl acetate = 5:1), 88% yield, 91% ee, M.p. 36-37 °C. **^{1}H NMR** (600 MHz, Chloroform-*d*) δ 8.47 (dd, *J* = 13.6, 8.8 Hz, 1H), 7.92 (d, *J* = 8.8 Hz, 1H), 7.82 (d, *J* = 8.4 Hz, 1H), 7.68 (d, *J* = 8.3 Hz, 1H), 7.60 (d, *J* = 4.9 Hz, 2H), 7.21-7.14 (m, 3H), 7.15-7.08 (m, 2H), 7.04 (d, *J* = 7.9 Hz, 1H), 6.95 (d, *J* = 8.6 Hz, 1H), 6.90 (s, 2H), 3.13-3.04 (m, 4H), 3.00-2.91 (m, 1H), 2.75-2.63 (m, 4H), 1.92-1.80 (m, 1H), 1.54-1.44 (m, 1H), 1.44-1.34 (m, 1H), 1.27-1.17 (m, 7H), 1.08 (d, *J* = 12.8 Hz, 3H), 0.02 (s, 3H), -0.31 (s, 3H). **^{13}C NMR** (151 MHz, Chloroform-*d*) δ 144.11, 143.04, 142.37 (d, *J* = 3.8 Hz), 140.93 (d, *J* = 7.6 Hz), 138.68, 136.24, 134.58 (d, *J* = 2.4 Hz), 134.08, 133.75, 132.30, 132.01 (d, *J* = 11.2 Hz), 131.66, 130.03 (d, *J* = 75.9 Hz), 128.81, 128.72 (d, *J* = 13.5 Hz), 128.11, 128.06, 128.03, 127.63, 127.28, 126.81, 125.62, 125.45, 72.27 (d, *J* = 17.4 Hz), 58.30, 30.91 (d, *J* = 54.5 Hz), 28.88, 28.87, 23.16 (d, *J* = 2.9 Hz), 21.23 (d, *J* = 54.9 Hz), 15.14, 15.11, -1.21, -1.51. **^{31}P NMR** (243 MHz, Chloroform-*d*) δ 45.11. The enantiomeric excess was determined by Daicel Chiralpak IG, *n*-hexane/isopropanol = 95/5, 1 mL/min, λ = 254 nm, *t* (major) = 15.91 min, *t* (minor) = 19.54 min. $[\alpha]_D^{25}$ = 2.3 (c = 0.560, CH_2Cl_2). **HRMS (ESI)** calcd for: $C_{37}H_{43}O$ $NaSiPS^+$ $[M + Na]^+$ 617.2434; found: 617.2446.

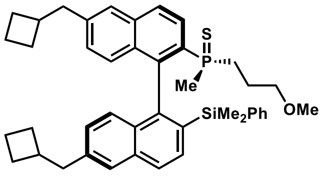

(5s) Light yellow oil, R_f = 0.64 (petroleum ether/ethyl acetate = 5:1), 87% yield, 92% ee, **^{1}H NMR** (600 MHz, Chloroform-*d*) δ 8.52 (dd, *J* = 13.6, 8.8 Hz, 1H), 7.97 (d, *J* = 8.6 Hz, 1H), 7.86 (d, *J* = 8.4 Hz, 1H), 7.73 (d, *J* = 8.4 Hz, 1H), 7.60 (d, *J* = 12.2 Hz, 2H), 7.28-7.15 (m, 5H), 7.05 (d, *J* = 8.7 Hz, 1H), 7.00 (d, *J* = 8.7 Hz, 1H), 6.97 (d, *J* = 8.7 Hz, 1H), 6.92 (d, *J* = 8.7 Hz, 1H), 3.19-3.11 (m, 4H), 3.07-2.99 (m, 1H), 2.84-2.77 (m, 4H), 2.68-2.55 (m, 2H), 2.09-1.97 (m, 4H), 1.97-1.79 (m, 5H), 1.79-1.68 (m, 4H), 1.56-1.49 (m, 1H), 1.49-1.41 (m, 1H), 1.28-1.19 (m, 1H), 1.10 (d, *J* = 12.9 Hz, 3H), 0.07 (s, 3H), -0.29 (s, 3H). **^{13}C NMR** (151 MHz, Chloroform-*d*) δ 142.32 (d, *J* = 3.7 Hz), 141.31, 140.96 (d, *J* = 7.5 Hz), 140.25, 138.68, 136.27, 134.50 (d, *J* = 2.0 Hz), 134.07, 133.64, 132.33, 132.07 (d, *J* = 11.2 Hz), 131.62, 129.99 (d, *J* = 76.1 Hz), 128.80, 128.72 (d, *J* = 13.5 Hz), 128.50, 128.47, 128.07, 128.00, 127.61, 127.26, 126.73, 126.45, 126.30, 72.26 (d, *J* = 17.4 Hz), 58.27, 43.01, 42.99, 36.97, 30.91 (d, *J* = 54.4 Hz), 28.42, 28.39, 23.16 (d, *J* = 2.6 Hz), 21.18 (d, *J* = 54.9 Hz), 18.45, 18.44, -1.23, -1.60. **^{31}P NMR** (243 MHz, Chloroform-*d*) δ 45.11. The enantiomeric excess was

determined by Daicel Chiralpak IA, *n*-hexane/isopropanol = 97.5/2.5, 1 mL/min, λ = 254 nm, *t* (minor) = 11.58 min, *t* (major) = 14.88 min. $[\alpha]_D^{25}$ = -7.2 (c = 0.097, CH_2Cl_2). **HRMS (ESI)** calcd for: $C_{43}H_{51}ONaPSSi^+$ $[M + Na]^+$ 697.3060; found: 697.3072.

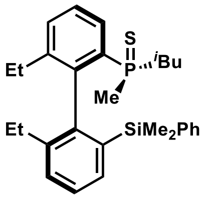

(**5t**) Yellow oil, R_f = 0.69 (petroleum ether/ethyl acetate = 20:1), 61% yield, 91% ee. **^{1}H NMR** (600 MHz, Chloroform-*d*) δ 8.21 (dd, *J* = 15.0, 7.9 Hz, 1H), 7.56 (d, *J* = 6.1 Hz, 1H), 7.49 (t, *J* = 6.8 Hz, 1H), 7.44-7.37 (m, 3H), 7.35 (d, *J* = 7.2 Hz, 2H), 7.32-7.23 (m, 3H), 2.54-2.42 (m, 1H), 2.22-2.01 (m, 5H), 1.66 (td, *J* = 14.7, 4.7 Hz, 1H), 1.26 (d, *J* = 12.8 Hz, 3H), 1.15 (t, *J* = 7.4 Hz, 3H), 0.98 (t, *J* = 7.4 Hz, 3H), 0.94 (d, *J* = 6.5 Hz, 3H), 0.81 (d, *J* = 6.5 Hz, 3H), 0.15 (s, 3H), -0.02 (d, *J* = 21.7 Hz, 3H). **^{13}C NMR** (151 MHz, Chloroform-*d*) δ 144.21 (d, *J* = 3.1 Hz), 142.70 (d, *J* = 9.3 Hz), 142.41, 141.75 (d, *J* = 7.4 Hz), 139.06, 137.68, 134.88 (d, *J* = 74.0 Hz), 134.22, 134.12, 131.41 (d, *J* = 12.8 Hz), 130.03 (d, *J* = 2.8 Hz), 129.06, 128.67, 128.29 (d, *J* = 13.4 Hz), 127.90, 127.84, 42.81 (d, *J* = 51.5 Hz), 26.12, 25.83, 25.27 (d, *J* = 10.7 Hz), 24.44 (d, *J* = 7.0 Hz), 24.25 (d, *J* = 3.2 Hz), 22.02 (d, *J* = 53.4 Hz), 13.50, 13.32, -0.43, -0.85. **^{31}P NMR** (243 MHz, Chloroform-*d*) δ 43.47. The enantiomeric excess was determined by Daicel Chiralpak IF, *n*-hexane/isopropanol = 97.5/2.5, 1 mL/min, λ = 230 nm, t (minor) = 8.65 min, t (major) = 9.69 min. $[\alpha]_D^{25}$ = -47.4 (c = 0.247, CH_2Cl_2). **HRMS (ESI)** calcd for: $C_{29}H_{39}NaPSSi^+$ $[M + Na]^+$ 501.2172; found: 501.2172.

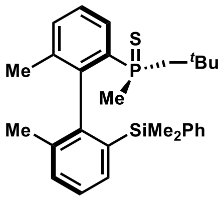

(**5u**) Yellow oil, R_f = 0.53 (petroleum ether/ethyl acetate = 20:1), 58% yield, 94% ee. **^{1}H NMR** (600 MHz, Chloroform-*d*) δ 8.28 (dd, *J* = 15.3, 8.0 Hz, 1H), 7.57 (d, *J* = 7.3 Hz, 1H), 7.42 (t, *J* = 7.6 Hz, 1H), 7.37-7.23 (m, 8H), 2.00 (s, 3H), 1.83 (t, *J* = 14.5 Hz, 1H), 1.72 (s, 3H), 1.46 (dd, *J* = 14.8, 8.3 Hz, 1H), 1.40 (d, *J* = 13.0 Hz, 3H), 1.03 (s, 9H), 0.20 (s, 3H), -0.01 (s, 3H). **^{13}C NMR** (151 MHz, Chloroform-*d*) δ 145.21 (d, *J* = 2.7 Hz), 142.02 (d, *J* = 7.5 Hz), 139.03, 137.51, 137.28 (d, *J* = 9.6 Hz), 136.85, 136.11 (d, *J* = 73.2 Hz), 134.30, 134.26, 132.80 (d, *J* = 2.8 Hz), 131.85 (d, *J* = 13.1 Hz), 131.53, 129.10, 128.19 (d, *J* = 13.5 Hz), 127.93, 127.71, 46.37 (d, *J* = 49.8 Hz), 32.90 (d, *J* = 4.2 Hz), 31.38 (d, *J* = 7.1 Hz), 24.46 (d, *J* = 53.2 Hz), 20.97, 20.66, -0.47, -0.83. **^{31}P NMR** (243 MHz, Chloroform-*d*) δ 40.72. The enantiomeric excess was determined by Daicel Chiralpak IC, *n*-hexane/isopropanol = 95/5, 1 mL/min, λ = 230 nm, t (minor) = 7.42 min, t (major) = 8.49 min. $[\alpha]_D^{25}$ = -69.6 (c = 0.205, CH_2Cl_2). **HRMS (ESI)** calcd for: $C_{28}H_{37}NaPSSi^+$ $[M + Na]^+$ 487.2015; found: 487.2024.

3.4. Enantioselective borylation of C-P bond

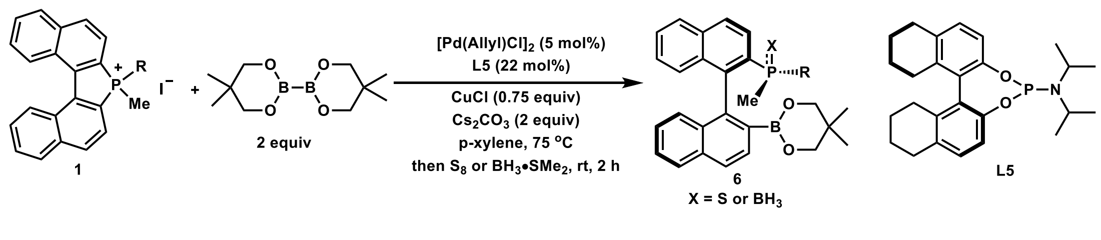

Typical Procedure: To a 10 mL Schlenk tube containing anhydrous Cs_2CO_3 (0.2 mmol, 2 equiv) was added phosphonium salts **1** (0.1 mmol), $B_2(OR)_2$ (0.2 mmol, 2 equiv), CuCl (0.075 mmol, 0.75 equiv), $[Pd(allyl)Cl]_2$ (0.005 mmol, 5 mol %), chiral phosphine ligand **L5** (0.022 mmol, 22 mol %), and *p*-xylene (1 mL, 0.1 M) sequentially under nitrogen. The Schlenk

tube was then sealed and stirred for 36 h at 35 ℃. The reaction mixture was cooled to rt, S_8 (5 equiv, 1 mmol) or BH_3•SMe_2 (2 equiv, 10 M in Me_2S) was added and stirred for 2 h at rt. The reaction mixture was then filtered through a pad of celite eluenting with CH_2Cl_2/EtOAc (20 mL). The filtrate was concentrated, and the residue was purified by preparative TLC.

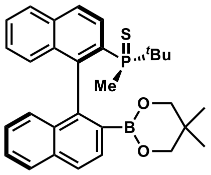

(6a) Yellow solid, R_f = 0.31 (petroleum ether/ethyl acetate = 20:1), 76% yield, 95% ee, M.p. 73 °C. **^{1}H NMR** (600 MHz, Chloroform-*d*) δ 8.53 (dd, *J* = 12.3, 8.9 Hz, 1H), 8.06 (d, *J* = 8.4 Hz, 1H), 7.98-7.93 (m, 2H), 7.90-7.83 (m, 2H), 7.51-7.40 (m, 2H), 7.33-7.27 (m, 1H), 7.21 (d, *J* = 8.5 Hz, 1H), 7.17 (t, *J* = 7.3 Hz, 1H), 7.11 (d, *J* = 8.6 Hz, 1H), 3.30 (d, *J* = 10.8 Hz, 2H), 3.08 (d, *J* = 10.8 Hz, 2H), 1.17 (d, *J* = 16.3 Hz, 9H), 0.64 (s, 6H), 0.57 (d, *J* = 12.9 Hz, 3H). **^{13}C NMR** (151 MHz, Chloroform-*d*) δ 143.50 (d, *J* = 3.6 Hz), 142.88 (d, *J* = 5.7 Hz), 135.02, 134.34, 134.14 (d, *J* = 11.0 Hz), 133.93 (d, *J* = 2.4 Hz), 130.93 (d, *J* = 13.0 Hz), 130.69, 128.33 (d, *J* = 68.7 Hz), 127.92, 127.90, 127.69, 127.45, 127.30, 127.27, 127.25, 127.15, 126.42 (d, *J* = 12.3 Hz), 126.22, 72.12, 36.34 (d, *J* = 49.9 Hz), 31.47, 25.59 (d, *J* = 2.4 Hz), 21.65, 15.41 (d, *J* = 52.1 Hz). **^{31}P NMR** (243 MHz, Chloroform-*d*) δ 61.54. The enantiomeric excess was determined by Daicel Chiralpak IE, *n*-hexane/isopropanol = 80/20, 1 mL/min, λ = 254 nm, *t* (major) = 15.65 min, *t* (minor) = 17.85 min. $[\alpha]_D^{25}$ = 25.7 (c = 0.236, CH_2Cl_2). **HRMS (ESI)** calcd for: $C_{30}H_{34}O_2NaSBP^+$ $[M + Na]^+$ 523.2002; found: 523.2014.

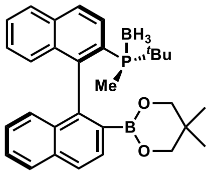

(6b) White solid, R_f = 0.31 (petroleum ether/ethyl acetate = 20:1), 71% yield, 94% ee, M.p. 87 °C. **^{1}H NMR** (600 MHz, Chloroform-*d*) δ 8.10 (dd, *J* = 11.1, 8.9 Hz, 1H), 8.05 (d, *J* = 8.4 Hz, 1H), 7.95 (d, *J* = 8.4 Hz, 1H), 7.91 (d, *J* = 8.6 Hz, 1H), 7.86 (t, *J* = 8.7 Hz, 2H), 7.48-7.40 (m, 2H), 7.28-7.22 (m, 1H), 7.18-7.14 (m, 1H), 7.12 (t, *J* = 10.0 Hz, 2H), 3.30 (d, *J* = 10.8 Hz, 2H), 3.09 (d, *J* = 10.8 Hz, 2H), 1.10 (d, *J* = 13.6 Hz, 9H), 0.85-0.32 (m, 3H), 0.63 (s, 6H), 0.23 (d, *J* = 10.2 Hz, 3H). **^{13}C NMR** (151 MHz, Chloroform-*d*) δ 145.03, 143.41 (d, *J* = 3.3 Hz), 134.88, 134.50 (d, *J* = 7.9 Hz), 134.37, 133.70 (d, *J* = 1.9 Hz), 131.44 (d, *J* = 16.9 Hz), 130.55, 127.87, 127.58, 127.51, 127.44, 127.31, 127.08, 126.69, 126.20 (d, *J* = 12.5 Hz), 126.06, 124.82 (d, *J* = 46.2 Hz), 72.12, 31.47, 30.87 (d, *J* = 32.0 Hz), 26.56 (d, *J* = 2.6 Hz), 21.65, 6.07 (d, *J* = 36.5 Hz). **^{31}P NMR** (243 MHz, Chloroform-*d*) δ 32.54 (d, *J* = 61.6 Hz). The enantiomeric excess was determined by Daicel Chiralpak AD-H, *n*-hexane/isopropanol = 97.5/2.5, 1 mL/min, λ = 254 nm, *t* (minor) = 7.97 min, *t* (major) = 9.94 min. $[\alpha]_D^{25}$ = 123.7 (c = 0.059, CH_2Cl_2). **HRMS (ESI)** calcd for: $C_{30}H_{37}B_2O_2NaP^+$ $[M + Na]^+$ 505.2609; found: 505.2628.

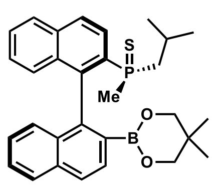

(6c) Yellow oil, R_f = 0.22 (petroleum ether/ethyl acetate = 20:1), 51% yield, 96% ee, **^{1}H NMR** (600 MHz, Chloroform-*d*) δ 8.64 (dd, *J* = 13.9, 8.8 Hz, 1H), 8.05-7.95 (m, 3H), 7.89 (t, *J* = 8.8 Hz, 2H), 7.51-7.42 (m, 2H), 7.30-7.23 (m, 1H), 7.23-7.14 (m, 2H), 7.09 (d, *J* = 8.5 Hz, 1H), 3.32 (d, *J* = 10.9 Hz, 2H), 3.16 (d, *J* = 10.9 Hz, 2H), 2.21-2.05 (m, 1H), 1.81 (td, *J* = 14.3, 5.3 Hz, 1H), 1.76-1.64 (m, 1H), 0.94 (d, *J* = 6.7 Hz, 3H), 0.87 (d, *J* = 13.3 Hz, 3H), 0.84 (d, *J* = 6.6 Hz, 3H), 0.56 (s, 6H). **^{13}C NMR** (151 MHz, Chloroform-*d*) δ 142.45 (d, *J* = 4.1 Hz), 141.89 (d, *J* = 7.1 Hz), 134.37, 134.26 (d, *J* = 2.5 Hz), 134.17, 133.80 (d, *J* = 11.3 Hz), 130.35, 130.35 (d, *J* = 75.8 Hz), 128.83 (d, *J* = 13.8 Hz), 128.02, 127.92, 127.69, 127.54 (d, *J* = 12.4 Hz), 127.51, 127.35, 127.16, 126.78, 126.51, 72.25, 43.71 (d, *J* = 51.3 Hz), 31.46, 25.19 (d, *J* = 11.2 Hz), 24.38 (d, *J* = 7.4 Hz), 24.21 (d, *J* = 3.4 Hz), 21.79 (d, *J* = 54.3 Hz), 21.53. **^{31}P NMR** (243 MHz, Chloroform-*d*) δ 44.30. The enantiomeric excess was determined by Daicel Chiralpak IG, *n*-hexane/isopropanol = 95/5, 1 mL/min, λ = 254 nm, *t* (major) = 17.80 min, *t* (minor)

= 22.68 min. $[\alpha]_D^{25}$ = 81.4 (c = 0.086, CH_2Cl_2). **HRMS (ESI)** calcd for: $C_{30}H_{34}BO_2NaPS^+$ $[M + Na]^+$ 523.2002; found: 523.2016.

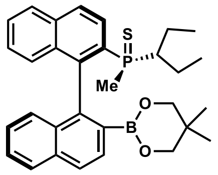

(6d) Yellow solid, R_f = 0.22 (petroleum ether/ethyl acetate = 20:1), 72% yield, 92% ee, M.p. 48 °C. **^{1}H NMR** (600 MHz, Chloroform-*d*) δ 8.56 (dd, *J* = 13.0, 8.8 Hz, 1H), 8.03 (d, *J* = 8.3 Hz, 1H), 8.00 (d, *J* = 8.9 Hz, 1H), 7.97 (d, *J* = 8.4 Hz, 1H), 7.88 (d, *J* = 8.2 Hz, 2H), 7.51-7.42 (m, 2H), 728-7.23 (m, 1H), 7.22-7.15 (m, 2H), 7.12 (d, *J* = 8.6 Hz, 1H), 3.33 (d, *J* = 10.8 Hz, 2H), 3.09 (d, *J* = 10.8 Hz, 2H), 1.75-1.56 (m, 3H), 1.53-1.43 (m, 1H), 1.34-1.27 (m, 1H), 0.93 (t, *J* = 7.3 Hz, 3H), 0.80-0.72 (m, 6H), 0.57 (s, 6H). **^{13}C NMR** (151 MHz, Chloroform-*d*) δ 142.87 (d, *J* = 6.8 Hz), 142.74 (d, *J* = 3.8 Hz), 134.46, 134.21, 134.17, 134.06 (d, *J* = 11.0 Hz), 130.63, 129.47 (d, *J* = 12.8 Hz), 128.63 (d, *J* = 72.6 Hz), 127.90, 127.86, 127.84, 127.69, 127.65, 127.31, 127.23, 127.09 (d, *J* = 12.3 Hz), 126.68, 126.40, 72.25, 43.76 (d, *J* = 50.4 Hz), 31.47, 21.53, 21.12, 21.03, 18.85 (d, *J* = 53.3 Hz), 13.78 (d, *J* = 10.2 Hz), 12.63 (d, *J* = 12.9 Hz). **^{31}P NMR** (243 MHz, Chloroform-*d*) δ 53.86. The enantiomeric excess was determined by Daicel Chiralpak IE, *n*-hexane/isopropanol = 80/20, 1 mL/min, λ = 254 nm, *t* (major) = 10.53 min, *t* (minor) = 14.06 min. $[\alpha]_D^{25}$ = 75.4 (c = 0.099, CH_2Cl_2). **HRMS (ESI)** calcd for: $C_{31}H_{36}O_2NaSBP^+$ $[M + Na]^+$ 537.2159; found: 537.2169.

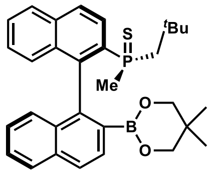

(6e) White oil, R_f = 0.25 (petroleum ether/ethyl acetate = 20:1), 80% yield, 92% ee, **^{1}H NMR** (600 MHz, Chloroform-*d*) δ 8.72 (dd, *J* = 14.1, 8.8 Hz, 1H), 8.05-7.96 (m, 3H), 7.88 (t, *J* = 9.1 Hz, 2H), 7.51-7.42 (m, 2H), 7.28-7.22 (m, 1H), 7.21-7.14 (m, 2H), 7.07 (d, *J* = 8.6 Hz, 1H), 3.33 (d, *J* = 10.8 Hz, 2H), 3.16 (d, *J* = 10.8 Hz, 2H), 2.05-1.87 (m, 2H), 1.04 (s, 9H), 0.92 (d, *J* = 13.4 Hz, 3H), 0.56 (s, 6H). **^{13}C NMR** (151 MHz, Chloroform-*d*) δ 142.64 (d, *J* = 3.7 Hz), 141.32 (d, *J* = 7.1 Hz), 134.34, 134.33, 134.14 (d, *J* = 2.3 Hz), 133.73 (d, *J* = 11.1 Hz), 131.44 (d, *J* = 75.2 Hz), 130.38, 129.09 (d, *J* = 14.1 Hz), 128.00, 127.87, 127.74, 127.64, 127.57 (d, *J* = 12.8 Hz), 127.47, 127.27, 127.14, 126.80, 126.45, 72.25, 47.33 (d, *J* = 49.6 Hz), 32.74 (d, *J* = 4.5 Hz), 31.47, 31.40 (d, *J* = 7.2 Hz), 23.52 (d, *J* = 53.8 Hz), 21.51. **^{31}P NMR** (243 MHz, Chloroform-*d*) δ 42.02. The enantiomeric excess was determined by Daicel Chiralpak IC, *n*-hexane/isopropanol = 95/5, 1 mL/min, λ = 254 nm, *t* (minor) = 12.47 min, *t* (major) = 13.99 min. $[\alpha]_D^{25}$ = 89.7 (c = 0.181, CH_2Cl_2). **HRMS (ESI)** calcd for: $C_{31}H_{36}BO_2NaPS^+$ $[M + Na]^+$ 537.2159; found: 537.2160.

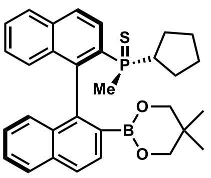

(6f) Yellow oil, R_f = 0.23 (petroleum ether/ethyl acetate = 20:1), 38% yield, 86% ee, **^{1}H NMR** (600 MHz, Chloroform-*d*) δ 8.71 (dd, *J* = 13.3, 8.9 Hz, 1H), 8.05-7.95 (m, 3H), 7.88 (d, *J* = 8.1 Hz, 2H), 7.51-7.43 (m, 2H), 7.28-7.22 (m, 1H), 7.21-7.15 (m, 2H), 7.09 (d, *J* = 8.6 Hz, 1H), 3.32 (d, *J* = 10.8 Hz, 2H), 3.14 (d, *J* = 10.8 Hz, 2H), 2.40-2.28 (m, 1H), 1.95-1.78 (m, 1H), 1.78-1.63 (m, 3H), 1.54-1.45 (m, 2H), 1.45-1.37 (m, 2H), 0.63 (d, *J* = 13.2 Hz, 3H), 0.56 (s, 6H). **^{13}C NMR** (151 MHz, Chloroform-*d*) δ 142.89 (d, *J* = 3.8 Hz), 142.21 (d, *J* = 7.0 Hz), 134.60, 134.38, 134.36, 133.77 (d, *J* = 11.1 Hz), 130.65, 130.02 (d, *J* = 13.3 Hz), 128.92 (d, *J* = 73.1 Hz), 127.98, 127.89, 127.69, 127.63, 127.48, 127.33, 127.24, 126.98 (d, *J* = 12.6 Hz), 126.88, 126.40, 72.35, 41.64 (d, *J* = 54.8 Hz), 31.48, 29.85, 26.99 (d, *J* = 66.7 Hz), 26.46 (d, *J* = 11.6 Hz), 26.29 (d, *J* = 11.0 Hz), 21.46, 19.65 (d, *J* = 55.4 Hz). **^{31}P NMR** (243 MHz, Chloroform-*d*) δ 52.21. The enantiomeric excess was determined by Daicel Chiralpak IE, *n*-hexane/isopropanol = 70/30, 1 mL/min, λ = 254 nm, *t* (major) = 8.62 min, *t* (minor) = 9.31 min. $[\alpha]_D^{25}$ =

81.9 (c = 0.085, CH_2Cl_2). **HRMS (ESI)** calcd for: $C_{31}H_{34}BO_2NaPS^+$ $[M + Na]^+$ 535.2002; found: 535.2001.

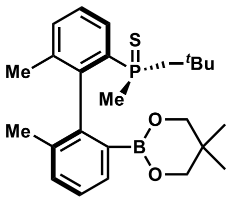

(**6g**) Yellow oil, R_f = 0.34 (petroleum ether/ethyl acetate = 20:1), 41% yield, 96% ee. **^{1}H NMR** (600 MHz, Chloroform-*d*) δ 8.38 (dd, *J* = 15.9, 7.7 Hz, 1H), 7.77-7.53 (m, 1H), 7.37-7.28 (m, 4H), 3.46 (d, *J* = 10.9 Hz, 2H), 3.40 (d, *J* = 10.9 Hz, 2H), 2.05 (dd, *J* = 14.9, 9.9 Hz, 1H), 1.94 (s, 3H), 1.91-1.83 (m, 4H), 1.29 (d, *J* = 13.4 Hz, 3H), 1.08 (s, 9H), 0.82 (s, 6H). **^{13}C NMR** (151 MHz, Chloroform-*d*) δ 144.27 (d, *J* = 3.0 Hz), 142.99 (d, *J* = 7.6 Hz), 136.95 (d, *J* = 9.8 Hz), 136.79, 133.39 (d, *J* = 74.5 Hz), 132.43, 132.34, 132.31 (d, *J* = 2.9 Hz), 132.06 (d, *J* = 13.6 Hz), 127.67, 127.01 (d, *J* = 13.8 Hz), 72.28, 47.95 (d, *J* = 49.6 Hz), 32.83 (d, *J* = 4.5 Hz), 31.47 (d, *J* = 7.1 Hz), 22.82 (d, *J* = 53.4 Hz), 21.86, 20.72, 20.68 (d, *J* = 0.8 Hz). **^{31}P NMR** (243 MHz, Chloroform-*d*) δ 41.47. The enantiomeric excess was determined by Daicel Chiralpak IC, *n*-hexane/isopropanol = 80/20, 1 mL/min, λ = 230 nm, t (minor) = 6.02 min, t (major) = 6.29 min. $[\alpha]_D^{25}$ = -12.5 (c = 0.149 CH_2Cl_2). **HRMS (ESI)** calcd for: $C_{25}H_{36}O_2NaPSB^+$ $[M + Na]^+$ 465.2159; found: 465.2165.

3.5. Enantioselective reduction of C-P bond with water as a hydride source

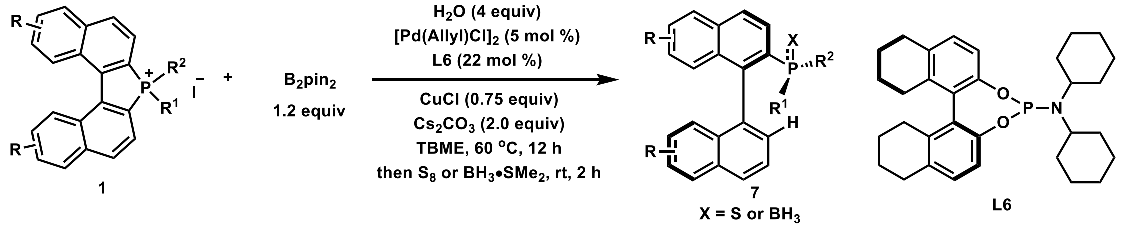

Typical Procedure: To a 10 mL Schlenk tube containing anhydrous Cs_2CO_3 (0.4 mmol, 2 equiv) was added phosphonium salts **1** (0.2 mmol), B_2pin_2 (0.24 mmol, 1.2 equiv), CuCl (0.15 mmol, 0.75 equiv), $[Pd(allyl)Cl]_2$ (0.01 mmol, 5 mol %), chiral phosphine ligand **L6** (0.044 mmol, 22 mol %), TBME (4 mL, 0.05 M), and H_2O (14 μL, 4 equiv) sequentially under nitrogen. The Schlenk tube was then sealed and stirred for 12 h at 60 ℃. The reaction mixture was cooled to rt, S_8 (5 equiv, 1 mmol) or BH_3•SMe_2 (2 equiv, 10 M in Me_2S) was added and stirred for 2 h at rt. The reaction mixture was then filtered through a pad of celite eluenting with CH_2Cl_2/EtOAc (20 mL). The filtrate was concentrated, and the residue was purified by silica gel chromatography to afford the corresponding product.

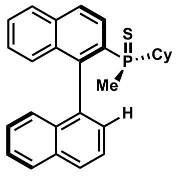

(**7a**) Yellow solid, R_f = 0.47 (petroleum ether/ethyl acetate = 20:1), 91% yield, 91% ee, M.p. 67-68 °C. **^{1}H NMR** (600 MHz, Chloroform-*d*) δ 8.80 (dd, *J* = 13.5, 8.8 Hz, 1H), 8.08 (d, *J* = 8.7 Hz, 1H), 8.05 (d, *J* = 8.2 Hz, 1H), 7.95 (d, *J* = 6.8 Hz, 2H), 7.64 (t, *J* = 7.6 Hz, 1H), 7.53 (t, *J* = 7.5 Hz, 1H), 7.49 (t, *J* = 7.5 Hz, 1H), 7.45 (d, *J* = 6.8 Hz, 1H), 7.29 (t, *J* = 7.6 Hz, 1H), 7.27-7.22 (m, 1H), 7.11 (d, *J* = 8.5 Hz, 1H), 7.07 (d, *J* = 8.7 Hz, 1H), 1.94-1.84 (m, 1H), 1.79-1.69 (m, 2H), 1.67-1.60 (m, 1H), 1.52-1.42 (m, 3H), 1.42-1.34 (m, 1H), 1.22-1.10 (m, 2H), 1.09-0.98 (m, 1H), 0.88 (d, *J* = 12.9 Hz, 3H). **^{13}C NMR** (151 MHz, Chloroform-*d*) δ 140.68 (d, *J* = 7.2 Hz), 136.25 (d, *J* = 3.8 Hz), 134.65 (d, *J* = 2.3 Hz), 134.11, 133.42, 133.37 (d, *J* = 10.7 Hz), 130.78, 130.69, 129.50, 128.72, 128.47, 128.14, 127.97, 127.89, 127.62, 127.23, 126.96, 126.73, 126.72, 124.98, 41.24 (d, *J* = 52.7 Hz), 26.45 (d, *J* = 4.7 Hz), 26.35 (d, *J* = 4.4 Hz), 26.07 (d, *J* = 2.3 Hz), 25.76 (d, *J* = 1.4 Hz), 25.31 (d, *J* = 1.7 Hz), 18.68 (d, *J* = 54.8 Hz).

^{31}**P NMR** (243 MHz, Chloroform-*d*) δ 52.40. The enantiomeric excess was determined by Daicel Chiralpak IA, *n*-hexane/isopropanol = 95/5, 1 mL/min, λ = 254 nm, *t* (minor) = 7.99 min, *t* (major) = 8.93 min. $[\alpha]_D^{25}$ = 71.1 (c = 0.410, CH_2Cl_2). **HRMS (ESI)** calcd for: $C_{27}H_{27}NaPS^+$ $[M + Na]^+$ 437.1463; found: 437.1465.

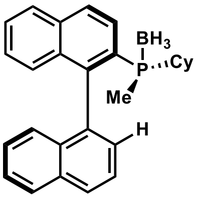

(**7b**) White solid, R_f = 0.55 (petroleum ether/ethyl acetate = 20:1), 87% yield, 89 % ee, M.p. 85-86 °C. ^{1}**H NMR** (600 MHz, Chloroform-*d*) δ 8.19 (dd, *J* = 11.7, 8.8 Hz, 1H), 8.04 (d, *J* = 8.3 Hz, 1H), 8.01 (d, *J* = 8.5 Hz, 1H), 7.95 (d, *J* = 8.3 Hz, 1H), 7.93 (d, *J* = 8.3 Hz, 1H), 7.64 (t, *J* = 7.6 Hz, 1H), 7.55-7.46 (m, 2H), 7.44 (d, *J* = 6.7 Hz, 1H), 7.29 (t, *J* = 7.4 Hz, 1H), 7.27-7.20 (m, 1H), 7.11 (d, *J* = 8.5 Hz, 1H), 7.08 (d, *J* = 8.6 Hz, 1H), 1.78-1.66 (m, 3H), 1.66-1.59 (m, 1H), 1.54-1.47 (m, 1H), 1.47-1.41 (m, 1H), 1.39-1.27 (m, 2H), 1.22-1.02 (m, 2H), 1.02-0.91 (m, 1H), 0.51 (d, *J* = 10.3 Hz, 3H), 0.69-0.28 (m, 3H). ^{13}**C NMR** (151 MHz, Chloroform-*d*) δ 143.07, 136.02 (d, *J* = 3.4 Hz), 134.33 (d, *J* = 2.0 Hz), 133.97, 133.53 (d, *J* = 7.4 Hz), 133.31, 130.96 (d, *J* = 17.4 Hz), 129.28, 128.61, 128.41, 127.99, 127.89, 127.65, 127.17, 126.97, 126.81, 126.57, 126.51, 125.85 (d, *J* = 48.5 Hz), 124.94, 35.96 (d, *J* = 34.8 Hz), 26.84, 26.78, 26.76, 26.70, 25.86 (d, *J* = 1.3 Hz), 8.16 (d, *J* = 38.2 Hz). ^{31}**P NMR** (243 MHz, Chloroform-*d*) δ 21.19 (d, *J* = 72.5 Hz). The enantiomeric excess was determined by Daicel Chiralpak IE, *n*-hexane/isopropanol = 90/10, 1 mL/min, λ = 254 nm, *t* (major) = 7.62 min, *t* (minor) = 8.39 min. $[\alpha]_D^{25}$ = 63.3, (c = 0.497, CH_2Cl_2). **HRMS (ESI)** calcd for: $C_{27}H_{30}BNaP^+$ $[M + Na]^+$ 419.2070; found: 419.2082.

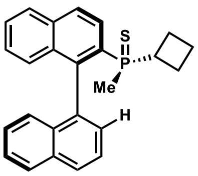

(**7c**) Brown solid, R_f = 0.34 (petroleum ether/ethyl acetate = 20:1), 80% yield, 92% ee, M.p. 56-57 °C. ^{1}**H NMR** (600 MHz, Chloroform-*d*) δ 8.78 (dd, *J* = 12.7, 9.4 Hz, 1H), 8.10-8.01 (m, 2H), 7.98-7.87 (m, 2H), 7.65 (t, *J* = 6.6 Hz, 1H), 7.56-7.40 (m, 3H), 7.35-7.18 (m, 2H), 7.12 (d, *J* = 8.3 Hz, 1H), 7.05 (d, *J* = 8.5 Hz, 1H), 2.87 (p, *J* = 8.3 Hz, 1H), 2.47-2.27 (m, 2H), 2.06-1.87 (m, 2H), 1.86-1.70 (m, 2H), 0.79 (d, *J* = 13.1 Hz, 3H). ^{13}**C NMR** (151 MHz, Chloroform-*d*) δ 140.45 (d, *J* = 7.4 Hz), 136.34 (d, *J* = 3.9 Hz), 134.56 (d, *J* = 2.3 Hz), 133.96, 133.36, 133.27 (d, *J* = 10.6 Hz), 130.04 (d, *J* = 13.0 Hz), 129.39, 128.67 (d, *J* = 71.1 Hz), 128.46, 128.40, 128.04, 127.96, 127.81, 127.53, 127.15, 126.90, 126.69, 126.61, 125.08, 36.62 (d, *J* = 50.0 Hz), 22.20 (d, *J* = 4.4 Hz), 21.67 (d, *J* = 4.6 Hz), 19.41 (d, *J* = 18.7 Hz), 18.63 (d, *J* = 55.4 Hz). ^{31}**P NMR** (243 MHz, Chloroform-*d*) δ 49.75. The enantiomeric excess was determined by Daicel Chiralpak IA, *n*-hexane/isopropanol = 95/5, 1 mL/min, λ = 254 nm, *t* (minor) = 7.35 min, *t* (major) = 8.51 min. $[\alpha]_D^{25}$ = 93.2 (c = 0.202, CH_2Cl_2). **HRMS (ESI)** calcd for: $C_{25}H_{24}PS^+$ $[M + H]^+$ 387.1311; found: 387.1334.

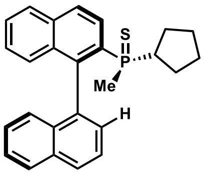

(**7d**) Orange solid, R_f = 0.43 (petroleum ether/ethyl acetate = 20:1), 85% yield, 93% ee, M.p. 159 °C. ^{1}**H NMR** (600 MHz, Chloroform-*d*) δ 8.90 (dd, *J* = 13.6, 8.8 Hz, 1H), 8.08 (d, *J* = 8.8 Hz, 1H), 8.04 (d, *J* = 8.3 Hz, 1H), 7.95 (d, *J* = 8.2 Hz, 2H), 7.66-7.60 (m, 1H), 7.53 (t, *J* = 7.5 Hz, 1H), 7.49 (t, *J* = 7.5 Hz, 1H), 7.43 (d, *J* = 6.8 Hz, 1H), 7.29 (t, *J* = 7.6 Hz, 1H), 7.27-7.21 (m, 1H), 7.11 (d, *J* = 8.5 Hz, 1H), 7.06 (d, *J* = 8.7 Hz, 1H), 2.44-2.34 (m, 1H), 1.91-1.65 (m, 4H), 1.56-1.39 (m, 4H), 0.82 (d, *J* = 13.1 Hz, 3H). ^{13}**C NMR** (151 MHz, Chloroform-*d*) δ 140.43 (d, *J* = 7.3 Hz), 136.40 (d, *J* = 3.8 Hz), 134.57 (d, *J* = 2.3 Hz), 134.10, 133.33, 133.22 (d, *J* = 10.6 Hz), 130.55 (d, *J* = 13.0 Hz), 129.38, 129.17 (d, *J* = 70.3 Hz), 128.38, 128.25, 128.02, 127.87 (d, *J* = 12.4 Hz), 127.78, 127.51, 127.14, 126.84, 126.67, 126.57, 125.00, 41.68 (d, *J* = 54.9 Hz), 27.20, 27.16, 26.59 (d, *J* = 10.3 Hz), 26.43 (d, *J* = 10.6 Hz), 19.98 (d, *J* = 55.5 Hz). ^{31}**P NMR** (243 MHz, Chloroform-*d*) δ 53.09. The enantiomeric

excess was determined by Daicel Chiralpak IA, *n*-hexane/isopropanol = 90/10, 1 mL/min, λ = 254 nm, *t* (minor) = 5.33 min, *t* (major) = 6.06 min. $[\alpha]_D^{25}$ = 2.5 (c = 0.685, CH_2Cl_2). **HRMS (ESI)** calcd for: $C_{26}H_{26}PS^+$ $[M + H]^+$ 401.1487; found: 401.1487.

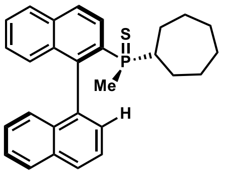

(**7e**) Yellow solid, R_f = 0.44 (petroleum ether/ethyl acetate = 20:1), 95% yield, 91% ee, M.p. 70 °C. **^{1}H NMR** (600 MHz, Chloroform-*d*) δ 8.83 (dd, *J* = 13.2, 8.9 Hz, 1H), 8.08 (d, *J* = 8.7 Hz, 1H), 8.04 (d, *J* = 8.2 Hz, 1H), 7.99-7.88 (m, 2H), 7.63 (t, *J* = 7.5 Hz, 1H), 7.56-7.42 (m, 3H), 7.27 (t, *J* = 7.6 Hz, 1H), 7.22 (t, *J* = 7.6 Hz, 1H), 7.11 (d, *J* = 8.4 Hz, 1H), 7.07 (d, *J* = 8.6 Hz, 1H), 2.06 (q, *J* = 10.1 Hz, 1H), 1.77-1.40 (m, 10H), 1.41-1.31 (m, 1H), 1.23-1.12 (m, 1H), 0.91 (d, *J* = 12.7 Hz, 3H). **^{13}C NMR** (151 MHz, Chloroform-*d*) δ 140.55 (d, *J* = 7.2 Hz), 136.04 (d, *J* = 3.7 Hz), 134.49 (d, *J* = 2.2 Hz), 133.91, 133.29, 133.25 (d, *J* = 12.1 Hz), 130.54 (d, *J* = 12.6 Hz), 129.43, 128.76, 128.69 (d, *J* = 69.4 Hz), 128.39, 128.01, 127.89 (d, *J* = 12.2 Hz), 127.78, 127.50, 127.08, 126.86, 126.60, 126.58, 124.77, 41.79 (d, *J* = 50.3 Hz), 28.05, 27.99, 27.92 (d, *J* = 16.6 Hz), 27.72, 27.65 (d, *J* = 17.0 Hz), 27.24, 18.90 (d, *J* = 54.4 Hz). **^{31}P NMR** (243 MHz, Chloroform-*d*) δ 55.23. The enantiomeric excess was determined by Daicel Chiralpak IE, *n*-hexane/isopropanol = 90/10, 1 mL/min, λ = 254 nm, *t* (major) = 15.26 min, *t* (minor) = 16.75 min. $[\alpha]_D^{25}$ = 68.2 (c = 0.127, CH_2Cl_2). **HRMS (ESI)** calcd for: $C_{28}H_{30}PS^+$ $[M + H]^+$ 429.1800; found: 429.1811.

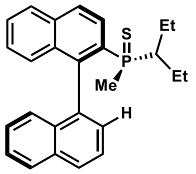

(**7f**) Yellow solid, R_f = 0.48 (petroleum ether/ethyl acetate = 20:1), 89% yield, 95% ee, M.p. 146 °C. **^{1}H NMR** (600 MHz, Chloroform-*d*) δ 8.86 (dd, *J* = 13.5, 8.8 Hz, 1H), 8.09 (d, *J* = 8.8 Hz, 1H), 8.04 (d, *J* = 8.3 Hz, 1H), 7.98-7.93 (m, 2H), 7.66-7.59 (m, 1H), 7.54 (t, *J* = 7.4 Hz, 1H), 7.49 (t, *J* = 7.4 Hz, 1H), 7.46 (d, *J* = 6.8 Hz, 1H), 7.29 (t, *J* = 7.3 Hz, 1H), 7.27-7.23 (m, 1H), 7.13-7.05 (m, 2H), 1.87-1.77 (m, 1H), 1.68-1.56 (m, 2H), 1.53-1.39 (m, 2H), 0.88 (d, *J* = 12.8 Hz, 3H), 0.84 (t, *J* = 7.5 Hz, 3H), 0.81 (t, *J* = 7.5 Hz, 3H). **^{13}C NMR** (151 MHz, Chloroform-*d*) δ 140.51 (d, *J* = 7.3 Hz), 136.18 (d, *J* = 3.8 Hz), 134.53 (d, *J* = 2.3 Hz), 134.07, 133.31, 133.26 (d, *J* = 10.7 Hz), 130.55 (d, *J* = 12.8 Hz), 129.43, 129.11 (d, *J* = 69.3 Hz), 128.66, 128.39, 128.04, 127.93 (d, *J* = 12.3 Hz), 127.81, 127.55, 127.12, 126.88, 126.63, 126.59, 124.82, 44.45 (d, *J* = 50.5 Hz), 21.60, 21.51, 20.03 (d, *J* = 54.1 Hz), 13.49 (d, *J* = 10.3 Hz), 12.61 (d, *J* = 11.2 Hz). **^{31}P NMR** (243 MHz, Chloroform-*d*) δ 54.51. The enantiomeric excess was determined by Daicel Chiralpak IE, *n*-hexane/isopropanol = 90/10, 1 mL/min, λ = 254 nm, *t* (major) = 11.50 min, *t* (minor) = 12.89 min. $[\alpha]_D^{25}$ = 38.9 (c = 0.126, CH_2Cl_2). **HRMS (ESI)** calcd for: $C_{26}H_{28}PS^+$ $[M + H]^+$ 403.1644; found: 403.1655.

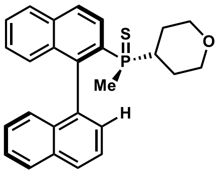

(**7g**) Yellow solid, R_f = 0.26 (petroleum ether/ethyl acetate = 5:1), 87% yield, 90% ee, M.p. 68-69 °C. **^{1}H NMR** (600 MHz, Chloroform-*d*) δ 8.71 (dd, *J* = 13.5, 8.8 Hz, 1H), 8.12-8.04 (m, 2H), 7.96 (d, *J* = 8.4 Hz, 1H), 7.94 (d, *J* = 8.4 Hz, 1H), 7.69-7.62 (m, 1H), 7.53 (t, *J* = 7.4 Hz, 1H), 7.49 (t, *J* = 6.4 Hz, 2H), 7.28 (t, *J* = 7.5 Hz, 1H), 7.26-7.22 (m, 1H), 7.11 (d, *J* = 8.6 Hz, 1H), 7.09 (d, *J* = 8.7 Hz, 1H), 4.00-3.85 (m, 2H), 3.22 (t, *J* = 11.7 Hz, 1H), 3.07 (t, *J* = 11.6 Hz, 1H), 2.09-1.97 (m, 1H), 1.89-1.80 (m, 1H), 1.80-1.69 (m, 1H), 1.36-1.27 (m, 2H), 0.94 (d, *J* = 13.0 Hz, 3H). **^{13}C NMR** (151 MHz, Chloroform-*d*) δ 140.80 (d, *J* = 7.2 Hz), 135.93 (d, *J* = 3.9 Hz), 134.60 (d, *J* = 2.4 Hz), 133.94, 133.36, 133.28 (d, *J* = 10.7 Hz), 130.07 (d, *J* = 13.1 Hz), 129.54, 128.61, 128.47, 128.12, 128.04, 128.02, 127.56 (d, *J* = 71.5 Hz), 127.52, 127.19, 127.06, 126.73, 126.46, 124.95, 67.79 (d, *J* = 14.4 Hz), 67.46 (d, *J* = 14.0 Hz), 38.72 (d, *J* = 54.6 Hz), 26.04 (d, *J* = 3.2 Hz), 25.11 (d, *J* = 2.7 Hz), 18.03 (d,

J = 55.3 Hz). **^{31}P NMR** (243 MHz, Chloroform-*d*) δ 50.64. The enantiomeric excess was determined by Daicel Chiralpak IE, *n*-hexane/isopropanol = 70/30, 1 mL/min, λ = 254 nm, *t* (major) = 13.67 min, *t* (minor) = 14.89 min. $[\alpha]_D^{25}$ = 61.4 (c = 0.375, CH_2Cl_2). **HRMS (ESI)** calcd for: $C_{26}H_{26}OPS^+$ [M + H]$^+$ 417.1436; found: 417.1445.

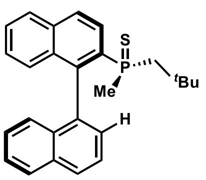

(**7h**) Yellow solid, R_f = 0.53 (petroleum ether/ethyl acetate = 20:1), 91% yield, 96% ee, M.p. 59 °C. **^{1}H NMR** (600 MHz, Chloroform-*d*) δ 8.97 (dd, J = 14.5, 8.8 Hz, 1H), 8.10 (d, J = 8.8 Hz, 1H), 8.04 (d, J = 8.3 Hz, 1H), 7.94 (d, J = 8.2 Hz, 2H), 7.67-7.61 (m, 1H), 7.55-7.49 (m, 2H), 7.48 (t, J = 7.5 Hz, 1H), 7.30-7.25 (m, 1H), 7.25-7.20 (m, 1H), 7.06 (t, J = 9.2 Hz, 2H), 2.17 (dd, J = 14.5, 12.3 Hz, 1H), 2.02 (dd, J = 14.7, 11.4 Hz, 1H), 1.02 (s, 9H), 0.94 (d, J = 13.2 Hz, 3H). **^{13}C NMR** (151 MHz, Chloroform-*d*) δ 140.05 (d, J = 7.2 Hz), 136.39 (d, J = 3.5 Hz), 134.50 (d, J = 1.4 Hz), 134.19, 133.36, 133.24 (d, J = 10.5 Hz), 130.78 (d, J = 70.7 Hz), 130.09 (d, J = 14.3 Hz), 129.42, 128.78, 128.39, 128.22 (d, J = 12.8 Hz), 128.03, 127.80, 127.55, 127.11, 126.91, 126.65, 126.54, 124.86, 48.32 (d, J = 50.1 Hz), 32.78 (d, J = 4.6 Hz), 31.48 (d, J = 7.3 Hz), 23.78 (d, J = 56.0 Hz). **^{31}P NMR** (243 MHz, Chloroform-*d*) δ 41.14. The enantiomeric excess was determined by Daicel Chiralpak AS-H, *n*-hexane/isopropanol = 95/5, 1 mL/min, λ = 254 nm, *t* (minor) = 9.25 min, *t* (major) = 13.31 min. $[\alpha]_D^{25}$ = 48.1 (c = 0.496, CH_2Cl_2). **HRMS (ESI)** calcd for: $C_{26}H_{28}PS^+$ [M + H]$^+$ 403.1644; found: 403.1650.

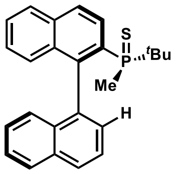

(**7i**) Orange solid, R_f = 0.37 (petroleum ether/ethyl acetate = 20:1), 80% yield, 88% ee, M.p. 79 °C. **^{1}H NMR** (600 MHz, Chloroform-*d*) δ 8.67 (dd, J = 12.2, 8.9 Hz, 1H), 8.04 (d, J = 8.8 Hz, 1H), 8.00 (d, J = 8.2 Hz, 1H), 7.95-7.88 (m, 2H), 7.59 (t, J = 7.6 Hz, 1H), 7.50 (t, J = 7.4 Hz, 1H), 7.48-7.42 (m, 2H), 7.30 (t, J = 7.5 Hz, 1H), 7.23 (t, J = 7.6 Hz, 1H), 7.15 (d, J = 8.5 Hz, 1H), 7.07 (d, J = 8.6 Hz, 1H), 1.19 (d, J = 16.4 Hz, 9H), 0.71 (d, J = 13.0 Hz, 3H). **^{13}C NMR** (151 MHz, Chloroform-*d*) δ 140.39 (d, J = 5.7 Hz), 136.57 (d, J = 3.5 Hz), 134.79, 134.48 (d, J = 2.4 Hz), 133.24 (d, J = 10.6 Hz), 133.17, 131.08 (d, J = 12.9 Hz), 129.12, 128.64, 128.48 (d, J = 67.0 Hz), 128.32, 127.89, 127.67, 127.57, 127.33 (d, J = 12.3 Hz), 126.95, 126.60, 126.07, 124.65, 35.92 (d, J = 49.8 Hz), 25.39 (d, J = 2.3 Hz), 15.43 (d, J = 52.4 Hz). **^{31}P NMR** (243 MHz, Chloroform-*d*) δ 62.18. The enantiomeric excess was determined by Daicel Chiralpak IC, *n*-hexane/isopropanol = 90/10, 1 mL/min, λ = 254 nm, *t* (major) = 11.27 min, *t* (minor) = 12.57 min. $[\alpha]_D^{25}$ = 38.9 (c = 0.329, CH_2Cl_2). **HRMS (ESI)** calcd for: $C_{25}H_{26}PS^+$ [M + H]$^+$ 389.1487; found: 389.1502.

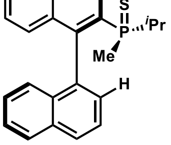

(**7j**) Yellow solid, R_f = 0.40 (petroleum ether/ethyl acetate = 20:1), 83% yield, 95% ee, M.p. 159 °C. **^{1}H NMR** (600 MHz, Chloroform-*d*) δ 8.86 (dd, J = 13.3, 9.0 Hz, 1H), 8.08 (d, J = 8.7 Hz, 1H), 8.04 (d, J = 8.2 Hz, 1H), 7.94 (d, J = 8.2 Hz, 2H), 7.63 (t, J = 7.5 Hz, 1H), 7.52 (t, J = 7.4 Hz, 1H), 7.49 (t, J = 7.5 Hz, 1H), 7.43 (d, J = 6.8 Hz, 1H), 7.28 (t, J = 7.5 Hz, 1H), 7.24 (t, J = 7.7 Hz, 1H), 7.11 (d, J = 8.4 Hz, 1H), 7.06 (d, J = 8.6 Hz, 1H), 2.26 (dq, J = 13.5, 6.7 Hz, 1H), 1.05 (dd, J = 18.7, 6.6 Hz, 3H), 0.98 (dd, J = 18.9, 6.7 Hz, 3H), 0.83 (d, J = 13.0 Hz, 3H). **^{13}C NMR** (151 MHz, Chloroform-*d*) δ 140.68 (d, J = 7.2 Hz), 136.32 (d, J = 3.9 Hz), 134.65 (d, J = 2.3 Hz), 134.12, 133.35, 133.29 (d, J = 10.6 Hz), 130.81 (d, J = 13.0 Hz), 129.46, 128.43, 128.30 (d, J = 69.9 Hz), 128.25 (d, J = 46.9 Hz), 127.93, 127.90, 127.85, 127.59, 127.23, 126.94, 126.73, 126.64, 125.00, 31.42 (d, J = 52.9 Hz), 18.82 (d, J = 54.8 Hz), 16.23, 15.82. **^{31}P NMR** (243 MHz, Chloroform-*d*) δ 56.41. The enantiomeric excess was determined by Daicel Chiralpak IA, *n*-hexane/isopropanol = 90/10, 1 mL/min, λ = 254 nm, *t* (minor) = 5.40 min, *t* (major)

= 6.17 min. $[\alpha]_D^{25}$ = 2.5 (c = 0.685, CH_2Cl_2). **HRMS (ESI)** calcd for: $C_{24}H_{24}PS^+$ $[M + H]^+$ 375.1331; found: 375.1345.

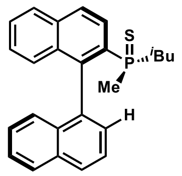

(**7k**) Yellow solid, R_f = 0.49 (petroleum ether/ethyl acetate = 20:1), 88% yield, 94% ee, M.p. 49-50 °C. **^{1}H NMR** (600 MHz, Chloroform-*d*) δ 8.90 (dd, *J* = 14.1, 8.9 Hz, 1H), 8.10 (d, *J* = 8.7 Hz, 1H), 8.05 (d, *J* = 8.2 Hz, 1H), 7.95 (d, *J* = 8.2 Hz, 2H), 7.64 (t, *J* = 7.4 Hz, 1H), 7.53 (t, *J* = 7.4 Hz, 1H), 7.51-7.45 (m, 2H), 7.29 (t, *J* = 7.6 Hz, 1H), 7.27-7.22 (m, 1H), 7.08 (t, *J* = 8.5 Hz, 2H), 2.20-2.08 (m, 1H), 2.03-1.94 (m, 1H), 1.83-1.74 (m, 1H), 0.94 (d, *J* = 6.6 Hz, 3H), 0.91 (d, *J* = 13.3 Hz, 3H), 0.82 (d, *J* = 6.6 Hz, 3H). **^{13}C NMR** (151 MHz, Chloroform-*d*) δ 140.55 (d, *J* = 7.5 Hz), 136.27 (d, *J* = 4.0 Hz), 134.62 (d, *J* = 2.4 Hz), 134.11, 133.38, 133.24 (d, *J* = 10.6 Hz), 130.05 (d, *J* = 13.9 Hz), 129.54 (d, *J* = 71.3 Hz), 129.48, 128.78, 128.43, 128.22 (d, *J* = 12.7 Hz), 128.10, 127.90, 127.56, 127.17, 126.98, 126.71, 126.57, 124.94, 44.65 (d, *J* = 51.8 Hz), 24.55 (d, *J* = 9.9 Hz), 24.46 (d, *J* = 8.8 Hz), 24.32 (d, *J* = 3.4 Hz), 22.21 (d, *J* = 56.1 Hz). **^{31}P NMR** (243 MHz, Chloroform-*d*) δ 43.92. The enantiomeric excess was determined by Daicel Chiralpak IA, *n*-hexane/isopropanol = 97.5/2.5, 1 mL/min, λ = 254 nm, *t* (minor) = 8.59 min, *t* (major) = 10.30 min. $[\alpha]_D^{25}$ = 25.5 (c = 0.540, CH_2Cl_2). **HRMS (ESI)** calcd for: $C_{25}H_{26}PS^+$ $[M + H]^+$ 389.1487; found: 389.1499.

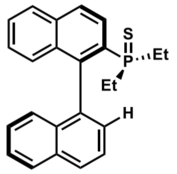

(**7l**) White solid, R_f = 0.27 (petroleum ether/ethyl acetate = 20:1), 87% yield, 93% ee, M.p. 115-116 °C. **^{1}H NMR** (600 MHz, Chloroform-*d*) δ 8.85 (dd, *J* = 13.1, 9.2 Hz, 1H), 8.09 (d, *J* = 8.7 Hz, 1H), 8.05 (d, *J* = 8.1 Hz, 1H), 8.01-7.92 (m, 2H), 7.69-7.58 (m, 1H), 7.57-7.42 (m, 3H), 7.30-7.20 (m, 2H), 7.07 (t, *J* = 8.9 Hz, 2H), 1.81-1.69 (m, 1H), 1.69-1.56 (m, 1H), 1.36-1.17 (m, 1H), 1.17-1.07 (m, 1H), 1.07-0.98 (m, 3H), 0.82-0.67 (m, 3H). **^{13}C NMR** (151 MHz, Chloroform-*d*) δ 141.02 (d, *J* = 7.5 Hz), 136.01 (d, *J* = 3.6 Hz), 134.64 (d, *J* = 2.3 Hz), 133.53, 133.37, 133.36 (d, *J* = 10.6 Hz), 131.15 (d, *J* = 12.7 Hz), 129.48, 128.63, 128.54, 128.05, 127.96 (d, *J* = 12.2 Hz), 127.89, 127.58, 126.90, 126.77, 126.71 (d, *J* = 67.2 Hz), 126.62, 126.56, 125.02, 26.75 (d, *J* = 53.6 Hz), 24.81 (d, *J* = 53.8 Hz), 6.87 (d, *J* = 4.6 Hz), 6.72 (d, *J* = 4.5 Hz). **^{31}P NMR** (243 MHz, Chloroform-*d*) δ 58.65. The enantiomeric excess was determined by Daicel Chiralpak IA, *n*-hexane/isopropanol = 97.5/2.5, 1 mL/min, λ = 254 nm, *t* (minor) = 10.69 min, *t* (major) = 11.50 min. $[\alpha]_D^{25}$ = 1.2 (c = 0.584, CH_2Cl_2). **HRMS (ESI)** calcd for: $C_{24}H_{24}PS^+$ $[M + H]^+$ 375.1331; found: 375.1339.

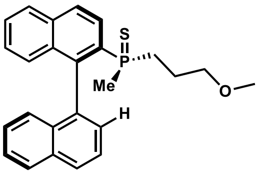

(**7m**) Yellow oil, R_f = 0.49 (petroleum ether/ethyl acetate = 5:1), 77% yield, 96% ee. **^{1}H NMR** (600 MHz, Chloroform-*d*) δ 8.80 (dd, *J* = 14.0, 8.8 Hz, 1H), 8.09 (d, *J* = 8.7 Hz, 1H), 8.04 (d, *J* = 8.2 Hz, 1H), 7.98-7.90 (m, 2H), 7.63 (t, *J* = 7.5 Hz, 1H), 7.56-7.50 (m, 2H), 7.48 (t, *J* = 7.5 Hz, 1H), 7.30-7.25 (m, 1H), 7.23 (t, *J* = 7.6 Hz, 1H), 7.09 (t, *J* = 7.5 Hz, 2H), 3.32-3.21 (m, 2H), 3.20 (s, 3H), 2.17-1.99 (m, 1H), 1.89-1.75 (m, 2H), 1.69-1.57 (m, 1H), 0.99 (d, *J* = 13.3 Hz, 3H). **^{13}C NMR** (151 MHz, Chloroform-*d*) δ 140.88 (d, *J* = 7.6 Hz), 135.97 (d, *J* = 4.0 Hz), 134.66 (d, *J* = 2.3 Hz), 133.93, 133.38, 133.29 (d, *J* = 10.7 Hz), 129.96 (d, *J* = 13.6 Hz), 129.41, 128.88, 128.69, 128.45, 128.16 (d, *J* = 12.8 Hz), 128.07, 127.94, 127.57, 127.11, 127.00, 126.62, 126.50, 125.20, 72.24 (d, *J* = 16.6 Hz), 58.48, 32.95 (d, *J* = 54.7 Hz), 23.07 (d, *J* = 2.9 Hz), 21.11 (d, *J* = 56.6 Hz). **^{31}P NMR** (243 MHz, Chloroform-*d*) δ 45.35. The enantiomeric excess was determined by Daicel Chiralpak AS-H, *n*-hexane/isopropanol = 70/30, 1 mL/min, λ = 254 nm, *t* (major) = 10.20 min, *t* (minor) = 12.29 min.

$[\alpha]_D^{25}$ = 42.0 (c = 0.248, CH_2Cl_2). **HRMS (ESI)** calcd for: $C_{25}H_{26}OPS^+$ $[M + H]^+$ 405.1436; found: 405.1448.

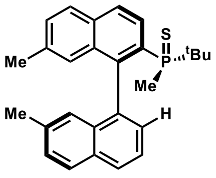

(**7n**) Yellow solid, R_f = 0.41 (petroleum ether/ethyl acetate = 20:1), 60% yield, 91% ee, M.p. 59 °C. **^{1}H NMR** (600 MHz, Chloroform-*d*) δ 8.58 (dd, *J* = 12.3, 8.9 Hz, 1H), 7.99 (d, *J* = 8.8 Hz, 1H), 7.95 (d, *J* = 8.2 Hz, 1H), 7.85-7.79 (m, 2H), 7.51 (t, *J* = 7.5 Hz, 1H), 7.39 (d, *J* = 6.8 Hz, 1H), 7.36 (d, *J* = 8.3 Hz, 1H), 7.30 (d, *J* = 8.3 Hz, 1H), 6.92 (s, 1H), 6.86 (s, 1H), 2.26 (s, 3H), 2.22 (s, 3H), 1.17 (d, *J* = 16.3 Hz, 9H), 0.68 (d, *J* = 13.1 Hz, 3H). **^{13}C NMR** (151 MHz, Chloroform-*d*) δ 140.00 (d, *J* = 5.8 Hz), 137.42, 136.71, 135.93 (d, *J* = 3.4 Hz), 135.11, 133.46 (d, *J* = 10.7 Hz), 132.83 (d, *J* = 2.2 Hz), 131.53, 130.25, 130.18, 130.17, 128.97, 128.84, 128.79, 128.17, 127.78, 127.04 (d, *J* = 12.5 Hz), 126.60, 124.87, 123.73, 35.97 (d, *J* = 49.9 Hz), 25.45, 25.44, 22.06, 15.38 (d, *J* = 52.4 Hz). **^{31}P NMR** (243 MHz, Chloroform-*d*) δ 62.11. The enantiomeric excess was determined by Daicel Chiralpak IE, *n*-hexane/isopropanol = 80/20, 1 mL/min, λ = 254 nm, *t* (major) = 7.35 min, *t* (minor) = 8.42 min. $[\alpha]_D^{25}$ = - 9.7 (c = 0.364, CH_2Cl_2). **HRMS (ESI)** calcd for: $C_{27}H_{30}PS^+$ $[M + H]^+$ 417.1800; found: 417.1809.

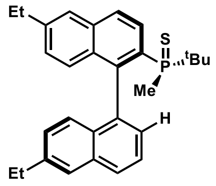

(**7o**) Brown solid, R_f = 0.47 (petroleum ether/ethyl acetate = 20:1), 90% yield, 89% ee, M.p. 99 °C. **^{1}H NMR** (600 MHz, Chloroform-*d*) δ 8.61 (dd, *J* = 12.2, 9.0 Hz, 1H), 7.97 (d, *J* = 8.8 Hz, 1H), 7.93 (d, *J* = 8.2 Hz, 1H), 7.73 -7.64 (m, 2H), 7.54 (t, *J* = 7.5 Hz, 1H), 7.38 (d, *J* = 6.8 Hz, 1H), 7.18 (d, *J* = 8.6 Hz, 1H), 7.11 (d, *J* = 8.6 Hz, 1H), 7.07 (d, *J* = 8.6 Hz, 1H), 7.01 (d, *J* = 8.8 Hz, 1H), 2.77 (q, *J* = 7.5 Hz, 4H), 1.32-1.26 (m, 6H), 1.18 (d, *J* = 16.4 Hz, 9H), 0.72 (d, *J* = 13.0 Hz, 3H). **^{13}C NMR** (151 MHz, Chloroform-*d*) δ 144.17, 142.61, 140.51 (d, *J* = 5.8 Hz), 136.55 (d, *J* = 3.3 Hz), 134.85 (d, *J* = 2.2 Hz), 133.48, 133.36, 131.79 (d, *J* = 10.7 Hz), 131.15 (d, *J* = 12.9 Hz), 128.85, 128.56, 128.17, 127.78, 127.75, 126.89 (d, *J* = 12.3 Hz), 126.08, 125.89, 125.61, 124.64, 35.93 (d, *J* = 50.0 Hz), 28.97, 28.94, 25.43 (d, *J* = 2.0 Hz), 15.60, 15.29, 15.25. **^{31}P NMR** (243 MHz, Chloroform-*d*) δ 61.96. The enantiomeric excess was determined by Daicel Chiralpak IC, *n*-hexane/isopropanol = 90/10, 1 mL/min, λ = 254 nm, *t* (major) = 10.26 min, *t* (minor) = 11.15 min. $[\alpha]_D^{25}$ = - 16.0 (c = 0.238, CH_2Cl_2). **HRMS (ESI)** calcd for: $C_{29}H_{34}PS^+$ $[M + H]^+$ 445.2113; found: 445.2121.

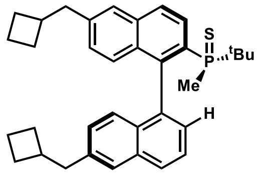

(**7p**) Orange solid, R_f = 0.44 (petroleum ether/ethyl acetate = 20:1), 75% yield, 86% ee, M.p. 81-82 °C. **^{1}H NMR** (600 MHz, Chloroform-*d*) δ 8.60 (dd, *J* = 12.3, 8.9 Hz, 1H), 7.95 (d, *J* = 8.7 Hz, 1H), 7.91 (d, *J* = 8.2 Hz, 1H), 7.63 (s, 2H), 7.53 (t, *J* = 7.6 Hz, 1H), 7.37 (d, *J* = 6.8 Hz, 1H), 7.12 (d, *J* = 8.6 Hz, 1H), 7.05 (d, *J* = 8.7 Hz, 2H), 6.99 (d, *J* = 8.7 Hz, 1H), 2.81 (d, *J* = 7.4 Hz, 4H), 2.69-2.55 (m, 2H), 2.13-1.95 (m, 4H), 1.91-1.80 (m, 4H), 1.80-1.68 (m, 4H), 1.17 (d, *J* = 16.3 Hz, 9H), 0.69 (d, *J* = 13.0 Hz, 3H). **^{13}C NMR** (151 MHz, Chloroform-*d*) δ 141.38, 140.51 (d, *J* = 5.8 Hz), 139.83, 136.54 (d, *J* = 3.3 Hz), 134.76 (d, *J* = 2.1 Hz), 133.38, 131.81 (d, *J* = 10.7 Hz), 131.12 (d, *J* = 12.7 Hz), 129.27, 128.63, 128.56, 127.81, 127.64, 127.23 (d, *J* = 68.1 Hz), 126.89 (d, *J* = 12.1 Hz), 126.73, 126.43, 126.00, 124.58, 43.13, 43.06, 37.05, 35.94 (d, *J* = 49.9 Hz), 28.52, 28.49, 28.47, 25.44 (d, *J* = 1.8 Hz), 18.50, 15.40 (d, *J* = 52.5 Hz). **^{31}P NMR** (243 MHz, Chloroform-*d*) δ 61.98. The enantiomeric excess was determined by Daicel Chiralpak IE, *n*-hexane/isopropanol = 90/10, 1 mL/min, λ = 254 nm, *t* (minor) = 12.93 min, *t* (major) = 15.02 min. $[\alpha]_D^{25}$ = - 36.3 (c = 0.747, CH_2Cl_2). **HRMS (ESI)** calcd for: $C_{35}H_{42}PS^+$ $[M + H]^+$ 525.2739; found: 525.2740.

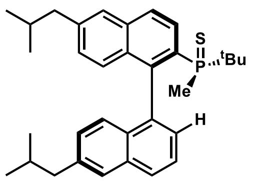

(**7q**) Orange solid, R_f = 0.59 (petroleum ether/ethyl acetate = 20:1), 79% yield, 80% ee, M.p. 88-89 °C. **^{1}H NMR** (600 MHz, Chloroform-*d*) δ 8.62 (dd, *J* = 12.3, 8.9 Hz, 1H), 7.96 (d, *J* = 8.8 Hz, 1H), 7.92 (d, *J* = 8.2 Hz, 1H), 7.650 (s, 1H), 7.645 (s, 1H), 7.59-7.48 (m, 1H), 7.38 (d, *J* = 6.8 Hz, 1H), 7.14 (d, *J* = 8.6 Hz, 1H), 7.08 (d, *J* = 8.7 Hz, 1H), 7.06 (d, *J* = 8.9 Hz, 1H), 7.01 (d, *J* = 8.7 Hz, 1H), 2.67-2.53 (m, 4H), 2.01-1.86 (m, 2H), 1.18 (d, *J* = 16.4 Hz, 9H), 0.95-0.86 (m, 12H), 0.70 (d, *J* = 13.0 Hz, 3H). **^{13}C NMR** (151 MHz, Chloroform-*d*) δ 141.73, 140.52 (d, *J* = 5.9 Hz), 140.20, 136.55 (d, *J* = 3.4 Hz), 134.68 (d, *J* = 2.3 Hz), 133.41, 133.30, 131.82 (d, *J* = 10.8 Hz), 131.13 (d, *J* = 12.9 Hz), 129.61, 129.03, 128.55, 127.87, 127.53, 127.23 (d, *J* = 68.1 Hz), 127.21, 126.87 (d, *J* = 12.3 Hz), 125.94, 124.59, 45.58, 45.50, 35.96 (d, *J* = 49.9 Hz), 30.12, 30.07, 25.44 (d, *J* = 2.1 Hz), 22.65, 22.60, 22.57, 22.52, 15.38 (d, *J* = 52.5 Hz). **^{31}P NMR** (243 MHz, Chloroform-*d*) δ 62.01. The enantiomeric excess was determined by Daicel Chiralpak IE, *n*-hexane/isopropanol = 95/5, 1 mL/min, λ = 254 nm, *t* (minor) = 15.63 min, *t* (major) = 17.89 min. $[\alpha]_D^{25}$ = - 18.8 (c = 0.723, CH_2Cl_2). **HRMS (ESI)** calcd for: $C_{33}H_{42}PS^+$ $[M + H]^+$ 501.2739; found: 501.2745.

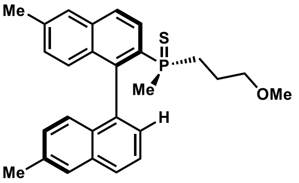

(7r) Yellow oil, R_f = 0.52 (petroleum ether/ethyl acetate = 5:1), 66% yield, 96% ee, M.p. 50 °C. **^{1}H NMR** (600 MHz, Chloroform-*d*) δ 8.75 (dd, *J* = 14.1, 8.8 Hz, 1H), 7.98 (d, *J* = 8.6 Hz, 1H), 7.93 (d, *J* = 8.1 Hz, 1H), 7.70 (s, 2H), 7.58 (t, *J* = 7.5 Hz, 1H), 7.44 (d, *J* = 6.7 Hz, 1H), 7.10 (d, *J* = 8.3 Hz, 1H), 7.07 (d, *J* = 8.7 Hz, 1H), 6.98 (t, *J* = 8.6 Hz, 2H), 3.29-3.17 (m, 2H), 3.20 (s, 3H), 2.48 (s, 3H), 2.47 (s, 3H), 2.17-2.04 (m, 1H), 1.88-1.74 (m, 2H), 1.69-1.57 (m, 1H), 0.98 (d, *J* = 13.3 Hz, 3H). **^{13}C NMR** (151 MHz, Chloroform-*d*) δ 140.88 (d, *J* = 7.7 Hz), 138.01, 136.38, 135.93 (d, *J* = 4.1 Hz), 134.95 (d, *J* = 2.1 Hz), 133.62, 132.23, 131.61 (d, *J* = 10.8 Hz), 130.14 (d, *J* = 13.8 Hz), 129.35, 129.28, 128.67, 127.90, 127.49, 127.44, 127.40, 127.02, 126.37, 125.23, 72.29 (d, *J* = 16.6 Hz), 58.47, 33.01 (d, *J* = 54.7 Hz), 23.06 (d, *J* = 2.6 Hz), 21.68, 21.16 (d, *J* = 56.6 Hz). **^{31}P NMR** (243 MHz, Chloroform-*d*) δ 45.29. The enantiomeric excess was determined by Daicel Chiralpak IA, *n*-hexane/isopropanol = 97.5/2.5, 1 mL/min, λ = 254 nm, *t* (minor) = 16.22 min, *t* (major) = 20.88 min. $[\alpha]_D^{25}$ = -6.6 (c = 0.297, CH_2Cl_2). **HRMS (ESI)** calcd for: $C_{27}H_{29}ONaPS^+$ $[M + Na]^+$ 455.1569; found: 455.1576.

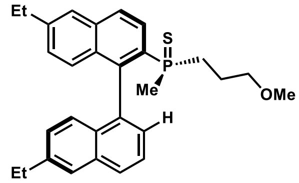

(7s) Yellow oil, R_f = 0.59 (petroleum ether/ethyl acetate = 5:1), 77% yield, 95% ee, M.p. 35 °C. **^{1}H NMR** (600 MHz, Chloroform-*d*) δ 8.76 (dd, *J* = 14.1, 8.8 Hz, 1H), 8.01 (d, *J* = 8.7 Hz, 1H), 7.96 (d, *J* = 8.3 Hz, 1H), 7.72 (s, 2H), 7.58 (dd, *J* = 7.9, 7.3 Hz, 1H), 7.44 (d, *J* = 6.8 Hz, 1H), 7.14 (d, *J* = 8.6 Hz, 1H), 7.10 (d, *J* = 8.7 Hz, 1H), 7.02 (d, *J* = 8.5 Hz, 1H), 7.01 (d, *J* = 8.7 Hz, 1H), 3.30-3.17 (m, 2H), 3.20 (s, 3H), 2.84-2.71 (m, 4H), 2.16-2.05 (m, 1H), 1.88-1.74 (m, 2H), 1.67-1.57 (m, 1H), 1.33-1.26 (m, 6H), 0.98 (d, *J* = 13.3 Hz, 3H). **^{13}C NMR** (151 MHz, Chloroform-*d*) δ 144.12, 142.52, 140.87 (d, *J* = 7.6 Hz), 135.91 (d, *J* = 4.0 Hz), 134.98, 133.63, 132.43, 131.80 (d, *J* = 10.9 Hz), 130.04 (d, *J* = 13.7 Hz), 128.82, 128.28, 128.20, 127.90, 127.63, 127.55, 127.20 (d, *J* = 73.2 Hz), 126.44, 125.96, 125.63, 125.14, 72.26 (d, *J* = 16.6 Hz), 58.44, 32.99 (d, *J* = 54.8 Hz), 28.92, 28.90, 23.03 (d, *J* = 2.4 Hz), 21.13 (d, *J* = 56.5 Hz), 15.19. **^{31}P NMR** (243 MHz, Chloroform-*d*) δ 45.28. The enantiomeric excess was determined by Daicel Chiralpak IA, *n*-hexane/isopropanol = 97.5/2.5, 1 mL/min, λ = 254 nm, *t* (minor) = 13.32 min, *t* (major) = 16.82 min. $[\alpha]_D^{25}$ = -13.0 (c = 0.300, CH_2Cl_2). **HRMS (ESI)** calcd for: $C_{29}H_{33}ONaPS^+$ $[M + H]^+$ 483.1882; found: 483.1897.

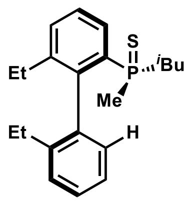

(**7t**) Yellow oil, R_f = 0.69 (petroleum ether/ethyl acetate = 20:1), 62% yield, 93% ee. **^{1}H NMR** (600 MHz, Chloroform-*d*) δ 8.66-8.53 (m, 1H), 7.52-7.45 (m, 2H), 7.42 (t, *J* = 7.4 Hz, 1H), 7.36 (d, *J* = 7.5 Hz, 1H), 7.29 (t, *J* = 7.2 Hz, 1H), 7.12 (d, *J* = 7.3 Hz, 1H), 2.34-2.22 (m, 2H), 2.22-2.10 (m, 2H), 2.10-1.98 (m, 1H), 1.93-1.82 (m, 1H), 1.81-1.70 (m, 1H), 1.22 (d, *J* = 13.3 Hz, 3H), 1.11 (t, *J* = 7.6 Hz, 3H), 1.05 (t, *J* = 7.5 Hz, 3H), 0.94 (d, *J* = 6.6 Hz, 3H), 0.80 (d, *J* = 6.6 Hz, 3H). **^{13}C NMR** (151 MHz, Chloroform-*d*) δ 143.23 (d, *J* = 9.1 Hz), 142.96, 141.16 (d, *J* = 7.7 Hz), 138.48 (d, *J* = 3.0 Hz), 133.49 (d, *J* = 13.5 Hz), 131.75 (d, *J* = 2.8 Hz), 131.02 (d, *J* = 70.4 Hz), 130.19, 129.02, 128.01, 127.88 (d, *J* = 13.7 Hz), 125.48, 44.59 (d, *J* = 52.1 Hz), 26.35, 26.00, 24.56 (d, *J* = 8.7 Hz), 24.45 (d, *J* = 9.8 Hz), 24.29 (d, *J* = 3.4 Hz), 21.55 (d, *J* = 55.7 Hz), 15.20, 13.43. **^{31}P NMR** (243 MHz, Chloroform-*d*) δ 43.54. The enantiomeric excess was determined by Daicel Chiralpak IC, *n*-hexane/isopropanol = 90/10, 1 mL/min, λ = 230 nm, t (major) = 8.99 min, t (minor) = 9.35 min. $[\alpha]_D^{25}$ = -36.3 (c = 0.193, CH_2Cl_2). **HRMS (ESI)** calcd for: $C_{21}H_{29}NaPS^+$ $[M + H]^+$ 367.1620; found: 367.1624.

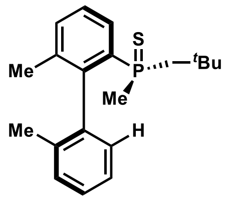

(**7u**) Yellow oil, R_f = 0.54 (petroleum ether/ethyl acetate = 20:1), 56% yield, 92% ee. **^{1}H NMR** (600 MHz, Chloroform-*d*) δ 8.67 (dd, *J* = 16.6, 7.0 Hz, 1H), 7.48-7.39 (m, 2H), 7.35 (t, *J* = 7.3 Hz, 1H), 7.32-7.27 (m, 2H), 7.15 (d, *J* = 7.3 Hz, 1H), 2.11-1.97 (m, 2H), 1.95 (s, 3H), 1.91 (s, 3H), 1.31 (d, *J* = 13.2 Hz, 3H), 1.02 (s, 9H). **^{13}C NMR** (151 MHz, Chloroform-*d*) δ 141.84 (d, *J* = 7.7 Hz), 139.58 (d, *J* = 2.8 Hz), 137.41, 137.23 (d, *J* = 9.4 Hz), 133.88 (d, *J* = 14.0 Hz), 133.46 (d, *J* = 2.7 Hz), 132.10 (d, *J* = 69.8 Hz), 130.74, 129.85, 128.77, 127.72 (d, *J* = 13.8 Hz), 125.97, 48.25 (d, *J* = 50.4 Hz), 32.87 (d, *J* = 4.6 Hz), 31.49 (d, *J* = 7.3 Hz), 22.95 (d, *J* = 55.9 Hz), 20.50, 20.03. **^{31}P NMR** (243 MHz, Chloroform-*d*) δ 40.48. The enantiomeric excess was determined by Daicel Chiralpak IC, *n*-hexane/isopropanol = 80/20, 1 mL/min, λ = 230 nm, t (major) = 6.60 min, t (minor) = 6.96 min. $[\alpha]_D^{25}$ = -44.0 (c = 0.138, CH_2Cl_2). **HRMS (ESI)** calcd for: $C_{20}H_{27}NaPS^+$ $[M + H]^+$ 353.1463; found: 353.1466.

3.6. Using HCOONa as a hydride donor instead of B_2pin_2/H_2O

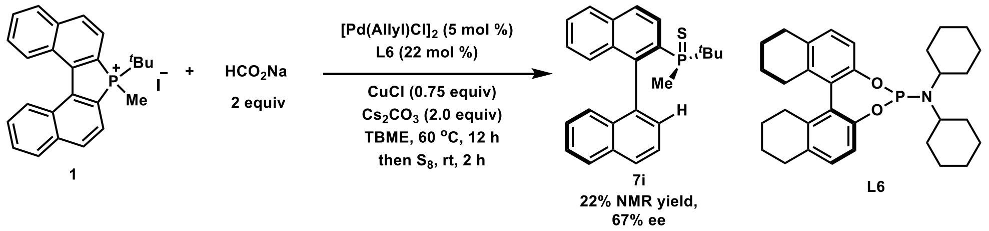

To a 10 mL Schlenk tube containing anhydrous Cs_2CO_3 (0.4 mmol, 2 equiv) was added phosphonium salts **1** (0.2 mmol), HCO_2Na (0.4 mmol, 2 equiv), CuCl (0.15 mmol, 0.75 equiv), $[Pd(allyl)Cl]_2$ (0.01 mmol, 5 mol %), chiral phosphine ligand **L6** (0.044 mmol, 22 mol %), and TBME (4 mL, 0.05 M) sequentially under nitrogen. The Schlenk tube was then sealed and stirred for 12 h at 60 ℃. The reaction mixture was cooled to rt, S_8 (5 equiv, 1 mmol) or BH_3•SMe_2 (2 equiv, 10 M in Me_2S) was added and stirred for 2 h at rt. The reaction mixture was then filtered through a pad of celite eluenting with CH_2Cl_2/EtOAc (20 mL). The filtrate was concentrated, and the residue was purified preparative TLC to afford the corresponding product **7i** in 22% NMR yield and 67% ee. The enantiomeric excess was determined by Daicel Chiralpak IC, *n*-hexane/isopropanol = 90/10, 1 mL/min, λ = 254 nm, *t* (major) = 11.40 min, *t* (minor) = 12.60 min.

3.7. Synthetic applications

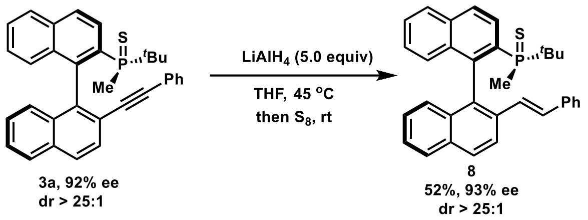

To a 10 mL oven dried Schlenk tube was added **3a** (48.9 mg, 0.1 mmol) and THF (2 mL). Then $LiAlH_4$ (0.5 mmol, 5 equiv) was added slowly at 0 °C under nitrogen. The Schlenk tube was then sealed and stirred for 16 h at 45 ℃. The reaction mixture was cooled to 0 °C. Saturated NH_4Cl aq was added, the aqueous solution was extracted with CH_2Cl_2 (3 × 20 mL). The combined organic layer was then washed with water and brine, and dried over anhydrous Na_2SO_4. Evaporation and the residue was dissolved in CH_2Cl_2 (2 mL), S_8 (16.1 mg) was added, and the reaction mixture was stirred for 10 min. at rt. The reaction mixture was then filtered, and the filtrate was concentrated, and the residue was purified by silica gel chromatography to afford the corresponding product **8** [2] as light yellow solid. R_f = 0.27 (petroleum ether/ethyl acetate = 20:1), 52% yield, 93% ee. **^{1}H NMR** (600 MHz, Chloroform-*d*) δ 8.61 (dd, *J* = 12.0, 9.0 Hz, 1H), 8.10 (d, *J* = 8.7 Hz, 1H), 8.05 (d, *J* = 8.8 Hz, 1H), 8.00 (d, *J* = 8.8 Hz, 1H), 7.95 (d, *J* = 8.1 Hz, 1H), 7.87 (d, *J* = 8.1 Hz, 1H), 7.52 (t, *J* = 7.3 Hz, 1H), 7.43 (t, *J* = 7.1 Hz, 1H), 7.30 (t, *J* = 7.3 Hz, 1H), 7.27-7.23 (m, 1H), 7.23-7.12 (m, 5H), 7.08 (d, *J* = 7.1 Hz, 2H), 7.01 (d, *J* = 8.5 Hz, 1H), 6.69 (d, *J* = 16.3 Hz, 1H), 1.13 (d, *J* = 16.4 Hz, 9H), 0.75 (d, *J* = 12.9 Hz, 3H). The enantiomeric excess was determined by Daicel Chiralpak ID, *n*-hexane/isopropanol = 90/10, 1 mL/min, λ = 254 nm, *t* (minor) = 13.00 min, *t* (major) = 15.32 min. $[\alpha]_D^{25}$ = 81.5 (c = 0.225, CH_2Cl_2).

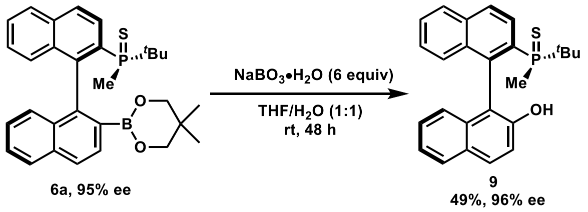

To a 15 mL vial was added **6a** (50.0 mg, 0.1 mmol), $NaBO_3•H_2O$ (92.4 mg, 0.6 mmol), THF (0.5 mL), and water (0.5 mL). The mixture was stirred at room temperature for 24 h. The reaction mixture was then filtered through a pad of celite eluenting with CH_2Cl_2/EtOAc (10 mL). The filtrate was concentrated, and the residue was purified by silica gel chromatography to afford the corresponding product **9** as a white solid. R_f = 0.40 (petroleum ether/ethyl acetate = 5:1), 49% yield, 96% ee. **^{1}H NMR** (600 MHz, Chloroform-*d*) δ 8.67 (dd, *J* = 12.0, 9.0 Hz, 1H), 8.09 (d, *J* = 8.7 Hz, 1H), 7.96 (t, *J* = 8.1 Hz, 2H), 7.85 (d, *J* = 8.1 Hz, 1H), 7.59 (t, *J* = 7.4 Hz, 1H), 7.38-7.20 (m, 5H), 6.98 (d, *J* = 8.4 Hz, 1H), 4.85 (s, 1H), 1.16 (d, *J* = 16.5 Hz, 9H), 0.85 (d, *J* = 12.9 Hz, 3H). **^{13}C NMR** (151 MHz, Chloroform-*d*) δ 150.96, 135.83, 134.88 (d, *J* = 2.2 Hz), 134.80 (d, *J* = 5.1 Hz), 132.45 (d, *J* = 10.2 Hz), 131.87, 131.79, 131.70 (d, *J* = 65.9 Hz), 131.21, 128.83, 128.56, 128.47, 128.30 (d, *J* = 12.1 Hz), 128.21, 128.17, 128.02, 126.59, 124.87, 124.37, 117.67, 36.04 (d, *J* = 49.7 Hz), 25.43 (d, *J* = 2.3 Hz), 15.28 (d, *J* = 52.7 Hz). **^{31}P NMR** (243 MHz, Chloroform-*d*) δ 62.07. The enantiomeric excess was determined by Daicel Chiralpak IG, *n*-hexane/isopropanol = 90/10, 1 mL/min, λ = 254 nm, *t* (major) = 13.42 min, *t* (minor) = 17.25 min. $[\alpha]_D^{25}$ = 70.2 (c = 0.085, CH_2Cl_2). **HRMS (ESI)** calcd for: $C_{25}H_{25}ONaPS^+$ $[M + Na]^+$ 427.1256; found: 427.1260.

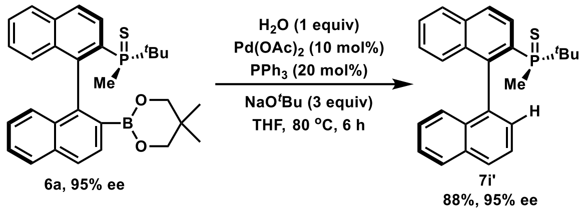

To a 10 mL Schlenk tube was added **6a** (14.8 mg, 0.03 mmol), H_2O (0.5 μL), $Pd(OAc)_2$ (0.7 mg, 0.03 mmol), PPh_3 (1.6 mg, 0.006 mmol), NaO^tBu (8.6 mg, 0.09 mmol), and THF (1 mL) sequentially under nitrogen. The Schlenk tube was then sealed and stirred for 12 h at 80 ℃. The reaction mixture was cooled to rt, and then filtered through a pad of celite eluenting with CH_2Cl_2/EtOAc (20 mL). The filtrate was concentrated, and the residue was purified by silica gel chromatography to afford the corresponding product 7i' as orange solid. R_f = 0.37 (petroleum ether/ethyl acetate = 20:1), 88% yield, 95% ee. **^{1}H NMR** (600 MHz, Chloroform-*d*) δ 8.66 (dd, *J* = 12.2, 8.9 Hz, 1H), 8.05 (d, *J* = 8.8 Hz, 1H), 8.01 (d, *J* = 8.3 Hz, 1H), 7.93 (d, *J* = 8.1 Hz, 2H), 7.60 (t, *J* = 7.6 Hz, 1H), 7.53 (t, *J* = 7.4 Hz, 1H), 7.51-7.41 (m, 2H), 7.32 (t, *J* = 7.6 Hz, 1H), 7.28-7.21 (m, 1H), 7.15 (d, *J* = 8.5 Hz, 1H), 7.07 (d, *J* = 8.6 Hz, 1H), 1.19 (d, *J* = 16.4 Hz, 9H), 0.71 (d, *J* = 13.0 Hz, 3H). The enantiomeric excess was determined by Daicel Chiralpak IC, *n*-hexane/isopropanol = 90/10, 1 mL/min, λ = 254 nm, *t* (major) = 11.52 min, *t* (minor) = 12.77 min. $[\alpha]_D^{25}$ = 35.3 (c = 0.085, CH_2Cl_2).

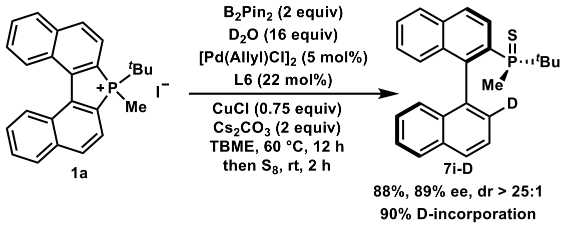

To a 10 mL Schlenk tube containing anhydrous Cs_2CO_3 (0.4 mmol, 2 equiv) was added phosphonium salts **1a** (0.2 mmol), B_2Pin_2 (0.24 mmol, 1.2 equiv), CuCl (0.15 mmol, 0.75 equiv), $[Pd(allyl)Cl]_2$ (0.01 mmol, 5 mol %), chiral phosphine ligand **L6** (0.044 mmol, 22 mol %), TBME (4 mL, 0.05 M), and D_2O (58 μL, 16 equiv) sequentially under nitrogen. The Schlenk tube was then sealed and stirred for 12 h at 60 ℃. The reaction mixture was cooled to rt, S_8 (5 equiv, 1 mmol) was added and stirred for 2 h at rt. The reaction mixture was then filtered through a pad of celite eluenting with CH_2Cl_2/EtOAc (20 mL). The filtrate was concentrated, and the residue was purified by silica gel chromatography to afford the corresponding product **7i-D** as an orange solid. R_f = 0.37 (petroleum ether/ethyl acetate = 20:1), 87% yield, 88% ee.**^{1}H NMR** (600 MHz, Acetone-*d6*) δ 8.65 (dd, *J* = 11.9, 9.2 Hz, 1H), 8.16 (dd, *J* = 8.5, 4.4 Hz, 2H), 8.06 (t, *J* = 7.6 Hz, 2H), 7.74 (d, *J* = 8.3 Hz, 1H), 7.64-7.57 (m, 1.1H), 7.55 (t, *J* = 7.5 Hz, 1H), 7.38 (t, *J* = 7.6 Hz, 1H), 7.33 (t, *J* = 7.7 Hz, 1H), 7.04 (t, *J* = 9.3 Hz, 2H), 1.18 (d, *J* = 16.3 Hz, 9H), 0.70-0.60 (m, 2.4H). **^{13}C NMR** (151 MHz, Chloroform-*d*) δ 140.39 (d, *J* = 5.7 Hz), 136.50 (d, *J* = 3.5 Hz), 134.80, 134.50 (d, *J* = 2.3 Hz), 133.26 (d, *J* = 10.7 Hz), 133.19, 131.15, 131.06, 129.14, 128.66, 128.48 (d, *J* = 66.8 Hz), 128.34, 127.92, 127.70, 127.61, 127.37 (d, *J* = 12.2 Hz), 126.97, 126.63, 126.09, 124.56, 38.56-31.73 (m), 25.40 (d, *J* = 1.9 Hz), 15.44 (d, *J* = 52.5 Hz). **^{31}P NMR** (243 MHz, Acetone-*d6*) δ 61.82. The enantiomeric excess was determined by Daicel Chiralpak IC, *n*-hexane/isopropanol = 90/10, 1 mL/min, λ = 254 nm, *t* (major) = 11.47 min, *t* (minor) = 12.74 min. $[\alpha]_D^{25}$ = 70.2 (c = 0.561, CH_2Cl_2). **HRMS (ESI)** calcd for: $C_{25}H_{24}DNaPS^+$ $[M + Na]^+$ 412.1370; found: 412.1381.

3.8. Deprotection of the phosphine sulphides and boranes

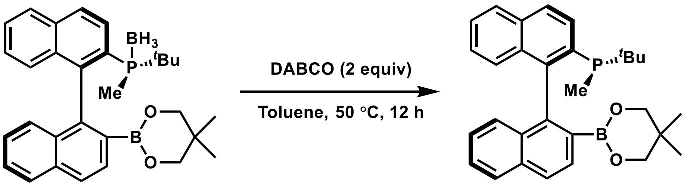

6b, 94% ee   L7, 41% yield

To a 25 mL Schlenk tube was added DABCO (67.2 mg, 0.60 mmol), **6b** (142.6 mg, 0.30 mmol), and toluene (2 mL) sequentially under nitrogen. The Schlenk tube was then sealed and stirred for 12 h at 50 ℃. The reaction mixture was cooled to rt and concentrated. The residue was purified by silica gel chromatography (petroleum ether /EtOAc 20:1) to afford the corresponding phosphine **L7** in 41% yield. Colorless oil, R_f = 0.27 (petroleum ether/ethyl acetate = 20:1), **^{1}H NMR** (600 MHz, Chloroform-*d*) δ 7.97 (d, *J* = 8.2 Hz, 1H), 7.91 (d, *J* = 8.2 Hz, 1H), 7.89-7.81 (m, 3H), 7.77 (d, *J* = 8.4 Hz, 1H), 7.37 (t, *J* = 7.2 Hz, 2H), 7.19 (d, *J* = 8.4 Hz, 1H), 7.12 (q, *J* = 8.0 Hz, 2H), 7.07 (d, *J* = 8.4 Hz, 1H), 3.30 (d, *J* = 10.8 Hz, 2H), 3.22 (d, *J* = 10.8 Hz, 2H), 1.23 (d, *J* = 5.1 Hz, 3H), 0.77 (d, *J* = 11.9 Hz, 9H), 0.54 (s, 6H). **^{13}C NMR** (151 MHz, Chloroform-*d*) δ 147.72 (d, *J* = 33.2 Hz), 144.40 (d, *J* = 8.8 Hz), 136.49 (d, *J* = 20.1 Hz), 134.31, 133.78 (d, *J* = 7.1 Hz), 133.30 (d, *J* = 16.9 Hz), 130.16, 128.85, 128.71, 128.69, 128.05, 128.03, 127.86, 127.39, 126.69, 126.20, 126.01, 125.98, 125.71, 124.94, 72.15, 31.39, 29.05 (d, *J* = 14.3 Hz), 28.29 (d, *J* = 15.5 Hz), 21.53, 8.41 (d, *J* = 20.9 Hz). **^{31}P NMR** (243 MHz, Chloroform-*d*) δ -23.22. $[\alpha]_D^{25}$ = -15.3 (c = 0.575, CH_2Cl_2). **HRMS (ESI)** calcd for: $C_{25}H_{27}BO_2P^+$ $[M – C_5H_8 + H]^+$ 401.1836; found: 401.1851.

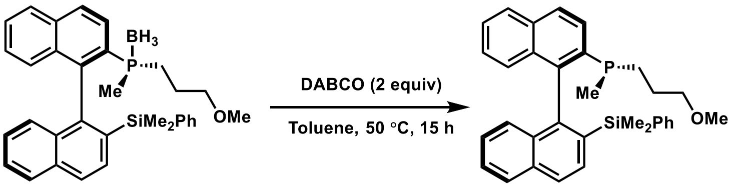

5j, 94% ee   L8, 46% yield

To a 25 mL Schlenk tube was added DABCO (35.8 mg, 0.32 mmol), **5j** (83.2 mg, 0.16 mmol), and toluene (2 mL) sequentially under nitrogen. The Schlenk tube was then sealed and stirred for 15 h at 50 ℃. The reaction mixture was cooled to rt and concentrated. The residue was purified by silica gel chromatography (petroleum ether / EtOAc 20:1) to afford the corresponding phosphine **L8** in 46% yield. Colorless oil, R_f = 0.48 (petroleum ether/ethyl acetate = 20:1), **^{1}H NMR** (600 MHz, Chloroform-*d*) δ 7.96 (d, *J* = 8.5 Hz, 1H), 7.92-7.83 (m, 3H), 7.75-7.67 (m, 2H), 7.47-7.38 (m, 2H), 7.29 (d, *J* = 6.9 Hz, 2H), 7.24 (d, *J* = 6.6 Hz, 1H), 7.22-7.12 (m, 5H), 7.04 (d, *J* = 8.5 Hz, 1H), 3.14 (s, 3H), 3.05-2.94 (m, 2H), 1.40-1.22 (m, 4H), 1.14 (d, *J* = 4.4 Hz, 3H), -0.10 (s, 3H), -0.19 (s, 3H). **^{13}C NMR** (151 MHz, Chloroform-*d*) δ 145.37 (d, *J* = 31.8 Hz), 144.94 (d, *J* = 8.0 Hz), 139.47, 137.55 (d, *J* = 15.1 Hz), 135.85 (d, *J* = 2.8 Hz), 134.25, 133.92 (d, *J* = 6.5 Hz), 133.65 (d, *J* = 2.6 Hz), 133.52 (d, *J* = 15.7 Hz), 131.93, 128.71, 128.26, 128.11, 127.81, 127.63, 127.60, 127.16, 126.81, 126.54, 126.45, 126.44, 126.34, 126.30, 125.83, 73.43 (d, *J* = 11.4 Hz), 58.37, 26.99 (d, *J* = 13.6 Hz), 25.74 (d, *J* = 12.9 Hz), 11.25 (d, *J* = 16.4 Hz), -1.26. **^{31}P NMR** (243 MHz, Chloroform-*d*) δ -47.75. $[\alpha]_D^{25}$ = -17.6 (c = 0.375, CH_2Cl_2). **HRMS (ESI)** calcd for: $C_{33}H_{36}OPSi^+$ $[M + H]^+$ 507.2268; found: 507.2279.

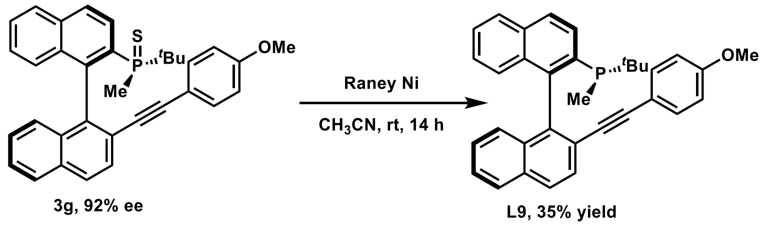

To a N_2-flushed Schlenk flask was loaded about 0.35 g of Raney Ni. The Raney Ni was washed with dried degassed CH_3CN (2 mL). To this flask was then added CH_3CN (2 mL) and **3g** (25.9 mg, 0.05 mmol). The resulting mixture was stirred under N_2 at rt for 14 h. The mixture was filtered through a short silica gel. The Raney Ni solid was washed with ether. The combined filtrate was concentrated under reduced pressure and the residue was purified by silica gel chromatography (petroleum ether /EtOAc 20:1) to afford product **L9** in 35% yield. White solid, R_f = 0.27 (petroleum ether/ethyl acetate = 20:1), **^{1}H NMR** (600 MHz, Chloroform-*d*) δ 7.98 (d, *J* = 8.5 Hz, 1H), 7.95-7.84 (m, 4H), 7.74 (d, *J* = 8.4 Hz, 1H), 7.47-7.37 (m, 2H), 7.26-7.16 (m, 3H), 7.09 (d, *J* = 8.5 Hz, 1H), 6.82 (d, *J* = 8.1 Hz, 2H), 6.66 (d, *J* = 8.0 Hz, 2H), 3.70 (s, 3H), 1.25 (d, *J* = 4.5 Hz, 3H), 0.78 (d, *J* = 12.1 Hz, 9H). **^{13}C NMR** (151 MHz, Chloroform-*d*) δ 159.47, 145.28 (d, *J* = 33.3 Hz), 141.48 (d, *J* = 7.8 Hz), 137.10 (d, *J* = 20.8 Hz), 133.73, 133.34, 132.97 (d, *J* = 7.5 Hz), 132.91, 132.74, 128.90 (d, *J* = 3.2 Hz), 128.23, 128.15, 128.12, 127.88, 127.81, 127.45 (d, *J* = 2.1 Hz), 127.12, 126.50, 126.33, 126.09, 125.97, 122.88 (d, *J* = 5.3 Hz), 115.67, 113.88, 94.15, 89.17, 55.30, 29.16 (d, *J* = 13.8 Hz), 28.10 (d, *J* = 15.5 Hz), 8.36 (d, *J* = 20.6 Hz). **^{31}P NMR** (243 MHz, Chloroform-*d*) δ -22.06. $[\alpha]_D^{25}$ = -74.8 (c = 0.086 CH_2Cl_2). **HRMS (ESI)** calcd for: $C_{34}H_{32}OP^+$ $[M + H]^+$ 487.2185; found: 487.2195.

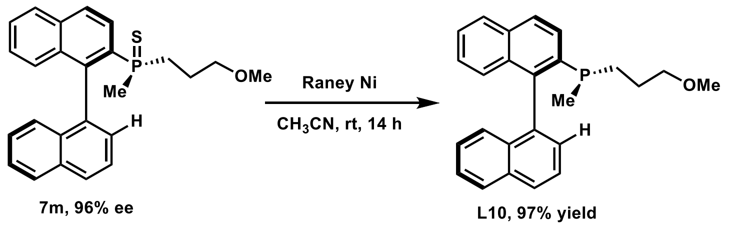

To a N_2-flushed Schlenk flask was loaded about 0.9 g of Raney Ni. The Raney Ni was washed with dried degassed CH_3CN (2 mL). To this flask was then added CH_3CN (2 mL) and **7m** (51.7 mg, 0.13 mmol). The resulting mixture was stirred under N_2 at rt for 14 h. The mixture was filtered through a short silica gel. The Raney Ni solid was washed with ether. The combined filtrate was concentrated under reduced pressure and the residue was purified by silica gel chromatography (petroleum ether /EtOAc 20:1) to afford product **L10** in 97% yield. Colorless oil, R_f = 0.29 (petroleum ether/ethyl acetate = 20:1), **^{1}H NMR** (600 MHz, Chloroform-*d*) δ 7.96 (d, *J* = 8.2 Hz, 2H), 7.92 (d, *J* = 8.1 Hz, 1H), 7.88 (d, *J* = 8.0 Hz, 1H), 7.75 (d, *J* = 8.1 Hz, 1H), 7.59 (t, *J* = 7.4 Hz, 1H), 7.48-7.36 (m, 3H), 7.25-7.18 (m, 2H), 7.18-7.10 (m, 2H), 3.14 (s, 3H), 3.11-2.99 (m, 2H), 1.63-1.50 (m, 1H), 1.45-1.30 (m, 3H), 1.22 (s, 3H). **^{13}C NMR** (151 MHz, Chloroform-*d*) δ 144.46 (d, *J* = 30.4 Hz), 137.85 (d, *J* = 8.0 Hz), 133.52, 133.46, 133.26 (d, *J* = 6.1 Hz), 128.75, 128.72, 128.42, 128.18, 128.16, 127.88, 127.20 (d, *J* = 2.2 Hz), 126.58, 126.44, 126.37, 126.20, 126.20, 126.06, 125.85, 125.28, 73.33 (d, *J* = 12.2 Hz), 58.38, 25.86 (d, *J* = 13.8 Hz), 25.70 (d, *J* = 12.7 Hz), 12.90 (d, *J* = 15.7 Hz). **^{31}P NMR** (243 MHz, Chloroform-*d*) δ -45.65. $[\alpha]_D^{25}$ = -36.7 (c = 0.462, CH_2Cl_2). **HRMS (ESI)** calcd for: $C_{25}H_{26}OP^+$ $[M + H]^+$ 373.1716; found: 373.1719.

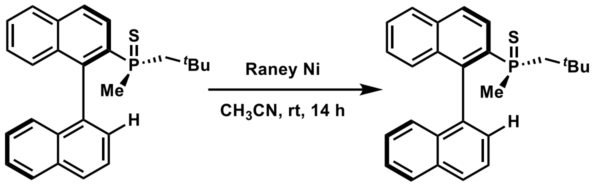

7h, 96% ee L11, 78% yield

To a N_2-flushed Schlenk flask was loaded about 2.4 g of Raney Ni. The Raney Ni was washed with dried degassed CH_3CN (5 mL). To this flask was then added CH_3CN (5 mL) and **7h** (134.8 mg, 0.34 mmol). The resulting mixture was stirred under N_2 at rt for 14 h. The mixture was filtered through a short silica gel. The Raney Ni solid was washed with ether. The combined filtrate was concentrated under reduced pressure and the residue was purified by silica gel chromatography (petroleum ether /EtOAc 20:1) to afford product **L11** in78% yield. Colorless oil, R_f = 0.78 (petroleum ether/ethyl acetate = 20:1), **^{1}H NMR** (600 MHz, Chloroform-*d*) δ 7.93 (d, *J* = 8.2 Hz, 2H), 7.90 (d, *J* = 8.1 Hz, 1H), 7.86 (d, *J* = 8.0 Hz, 1H), 7.78 (d, *J* = 8.4 Hz, 1H), 7.59 (t, *J* = 7.0 Hz, 1H), 7.45-7.36 (m, 3H), 7.25-7.09 (m, 4H), 1.59 (d, *J* = 14.0 Hz, 1H), 1.44 (d, *J* = 13.9 Hz, 1H), 1.26 (s, 3H), 0.51 (s, 9H). **^{13}C NMR** (151 MHz, Chloroform-*d*) δ 143.59 (d, *J* = 31.3 Hz), 139.18 (d, *J* = 14.0 Hz), 137.94 (d, *J* = 8.0 Hz), 133.73, 133.48 (d, *J* = 1.7 Hz), 133.28, 133.26 (d, *J* = 4.0 Hz), 128.78 (d, *J* = 3.7 Hz), 128.36, 128.03, 128.01, 127.86, 127.26 (d, *J* = 2.3 Hz), 126.86, 126.75 (d, *J* = 1.5 Hz), 126.32, 126.30, 125.93, 125.76, 125.42, 47.49 (d, *J* = 17.5 Hz), 31.18 (d, *J* = 14.1 Hz), 30.71 (d, *J* = 8.8 Hz), 15.38 (d, *J* = 15.6 Hz). **^{31}P NMR** (243 MHz, Chloroform-*d*) δ -54.30. $[\alpha]_D^{25}$ = -40.6 (c = 0.967, CH_2Cl_2). **HRMS (ESI)** calcd for: $C_{26}H_{28}P^+$ $[M + H]^+$ 371.1923; found: 371.1932.

3.9. Testing the developed ligand library in [3+2] annulation of MBH carbonates

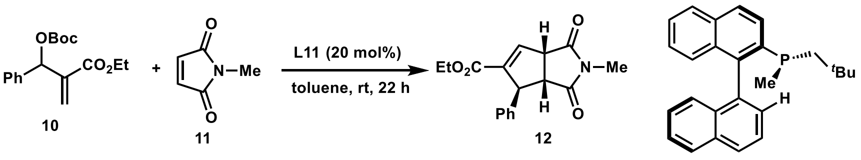

10 11 12 L11, 97% ee

To a 10 mL Schlenk tube was added **11** (13.3 mg, 0.12 mmol), toluene (1 mL), **L11** (7.4 mg, 0.02 mmol), and **10** (30.6 mg, 0.1 mmol) sequentially under nitrogen. The Schlenk tube was then sealed and stirred for 22 h at room temperature. The reaction mixture concentrated, and the residue was purified by silica gel chromatography to afford the corresponding product **12** [3] as a colorless oil. R_f = 0.22 (petroleum ether/ethyl acetate = 5:1), 92% yield, 93% ee. **^{1}H NMR** (600 MHz, Chloroform-*d*) δ 7.35 (t, *J* = 7.5 Hz, 2H), 7.28 (d, *J* = 7.8 Hz, 1H), 7.22 (d, *J* = 7.5 Hz, 2H), 6.91 (s, 1H), 4.57 (s, 1H), 4.19-4.15 (m, 1H), 4.15-4.03 (m, 2H), 3.42-3.30 (m, 1H), 3.02 (s, 3H), 1.17 (t, *J* = 7.1 Hz, 3H). The enantiomeric excess was determined by Daicel Chiralpak AD-H, *n*-hexane/isopropanol = 70/30, 1 mL/min, λ = 254 nm, *t* (major) = 6.46 min, *t* (minor) = 11.37 min. $[\alpha]_D^{25}$ = 229.6 (c = 0.278, CH_2Cl_2).

3.10. NMR and HPLC spectra

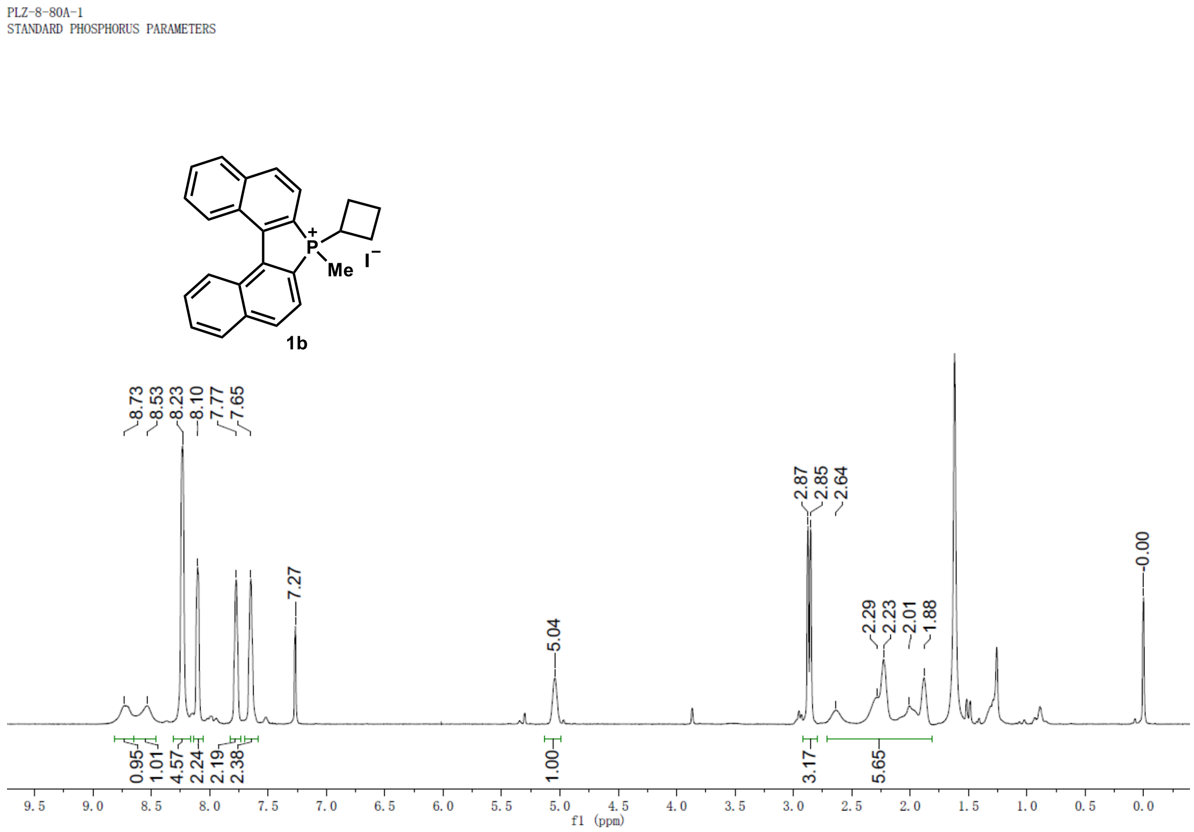

Supplementary Fig. 1. ^{1}H NMR spectrum of **1b**. The sample has been recorded in 600 MHz, $CDCl_3$ at 25 ℃.

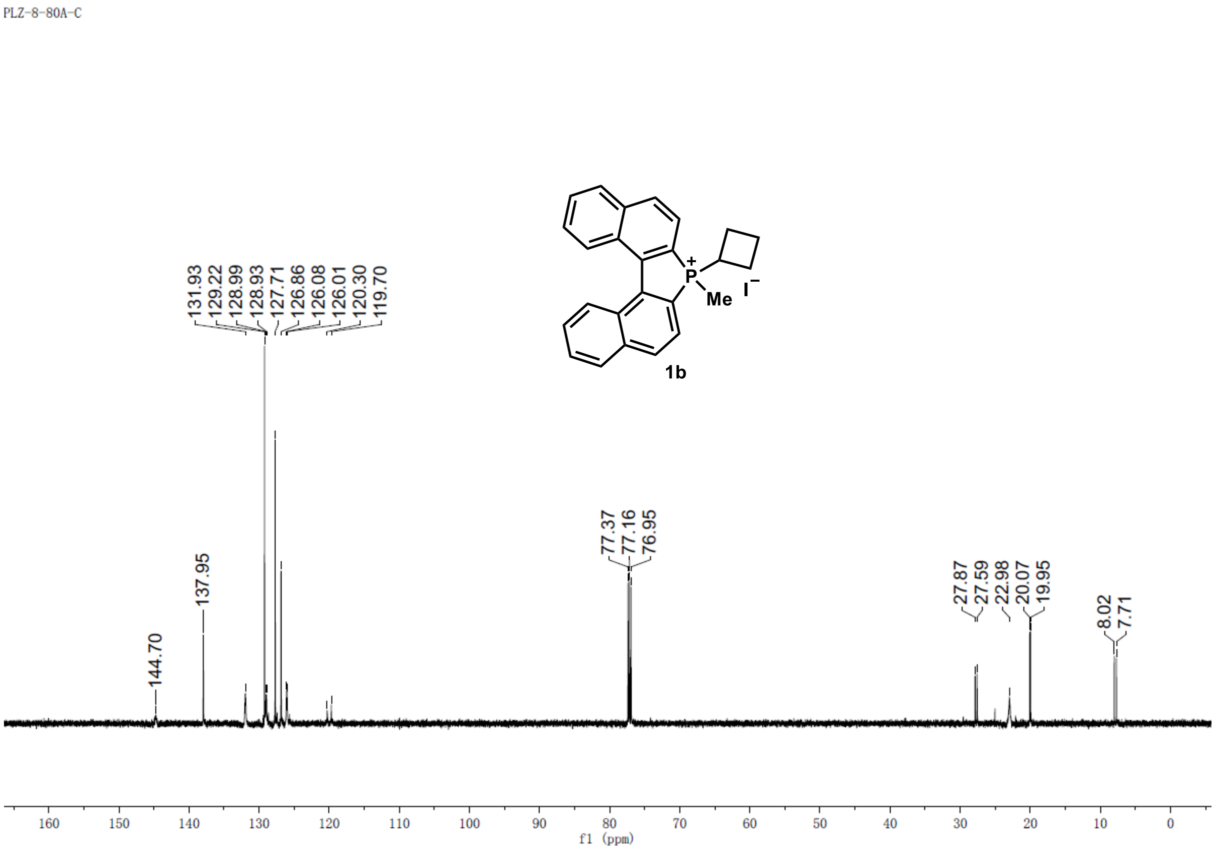

Supplementary Fig. 2.^{13}C NMR spectrum of **1b**. The sample has been recorded in 151 MHz, $CDCl_3$ at 25 ℃.

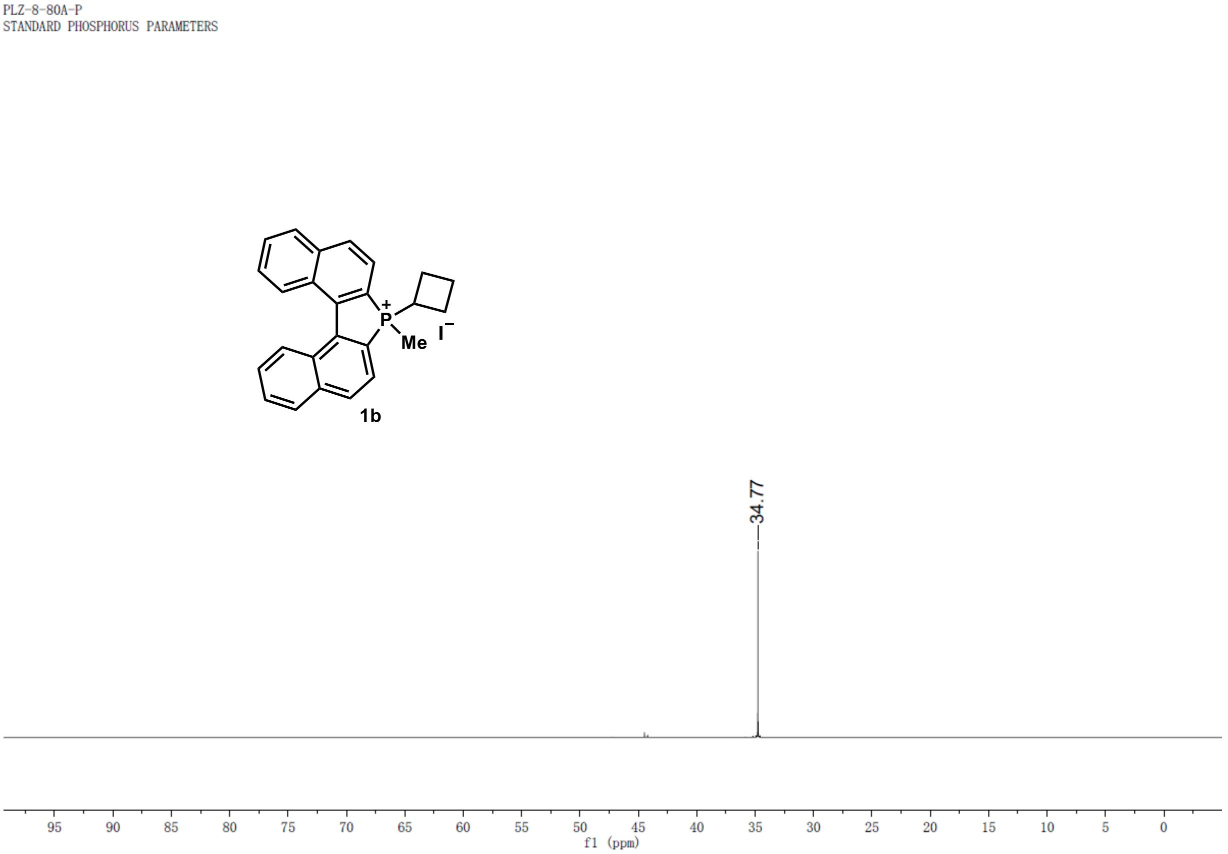

Supplementary Fig. 3.^{31}P NMR spectrum of **1b**. The sample has been recorded in 243 MHz, $CDCl_3$ at 25 ℃.

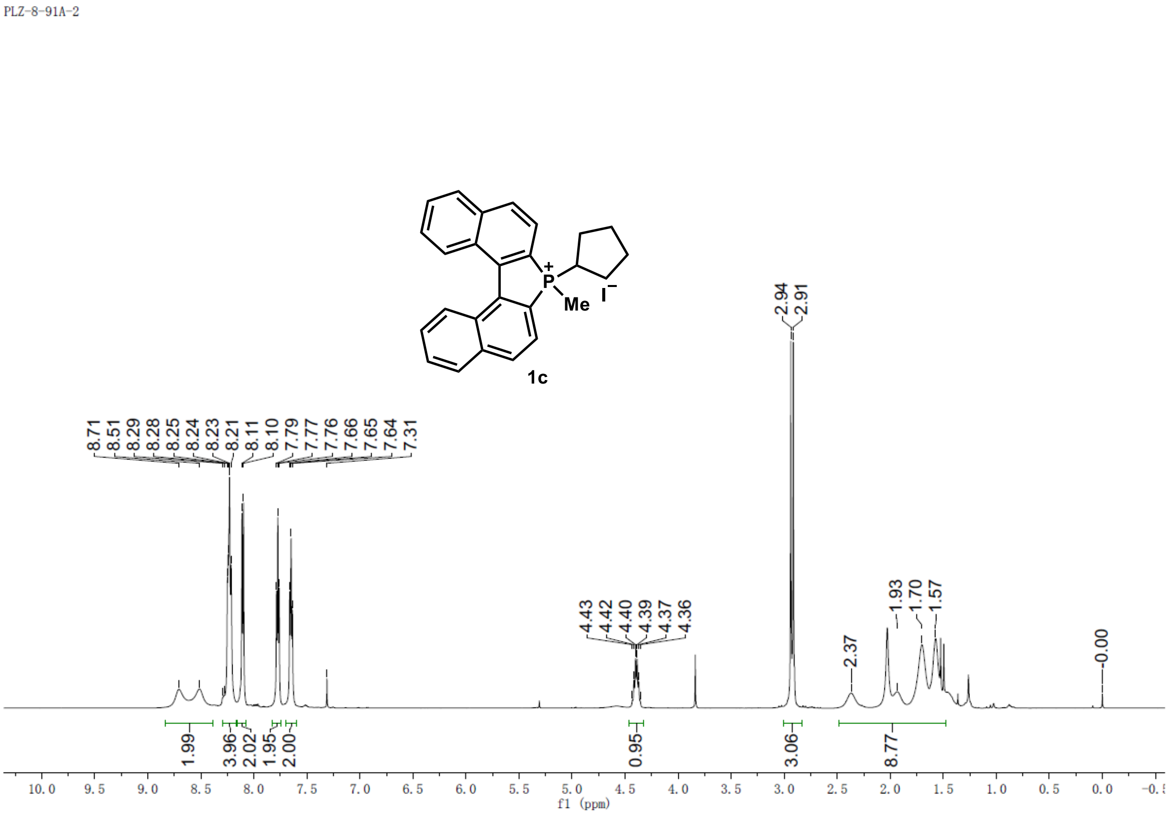

Supplementary Fig. 4. ^{1}H NMR spectrum of **1c**. The sample has been recorded in 600 MHz, $CDCl_3$ at 25 ℃.

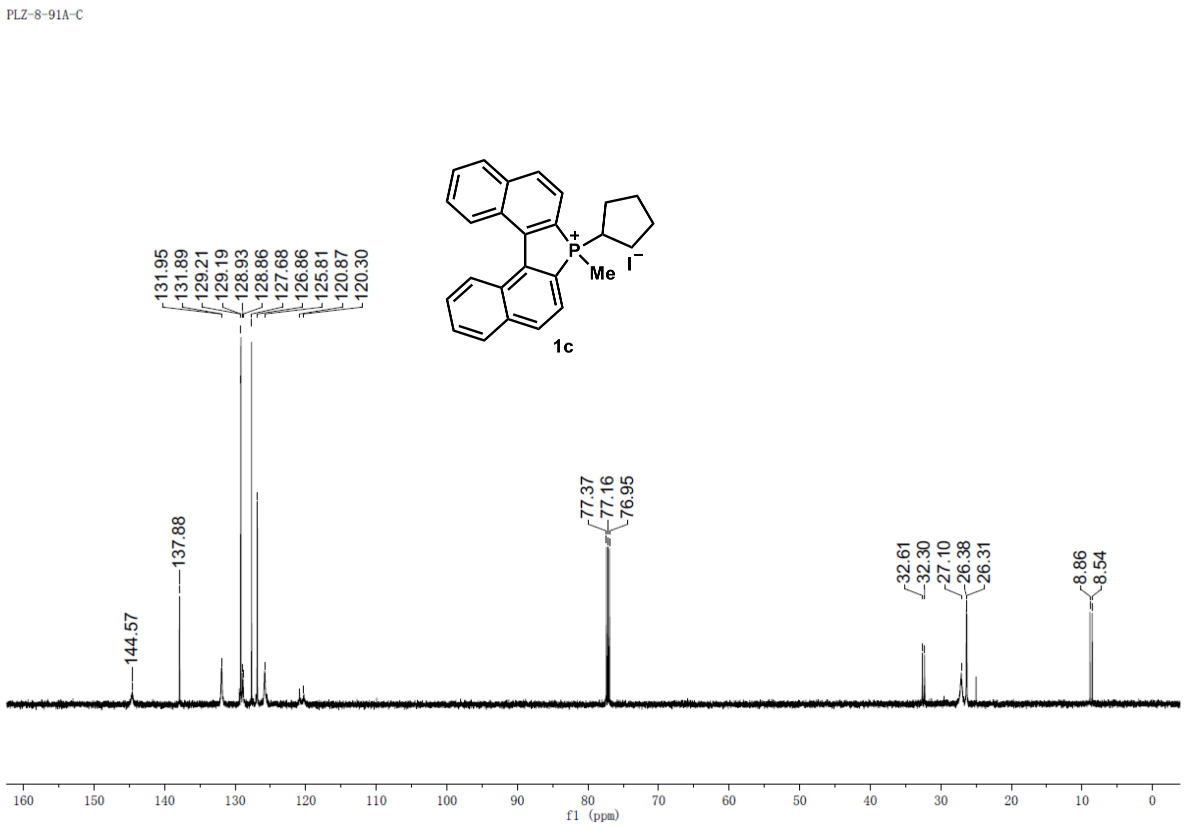

Supplementary Fig. 5.^{13}C NMR spectrum of **1c**. The sample has been recorded in 151 MHz, $CDCl_3$ at 25 ℃.

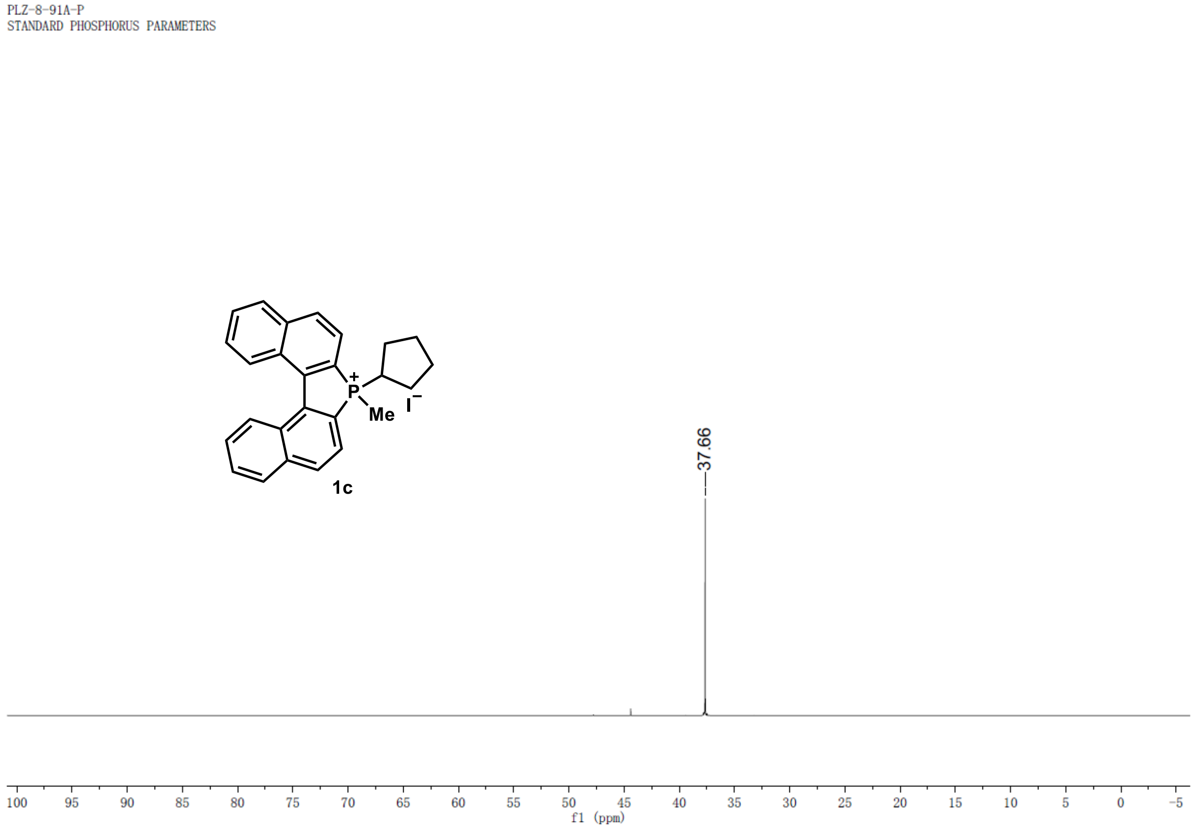

Supplementary Fig. 6.^{31}P NMR spectrum of **1c**. The sample has been recorded in 243 MHz, $CDCl_3$ at 25 ℃.

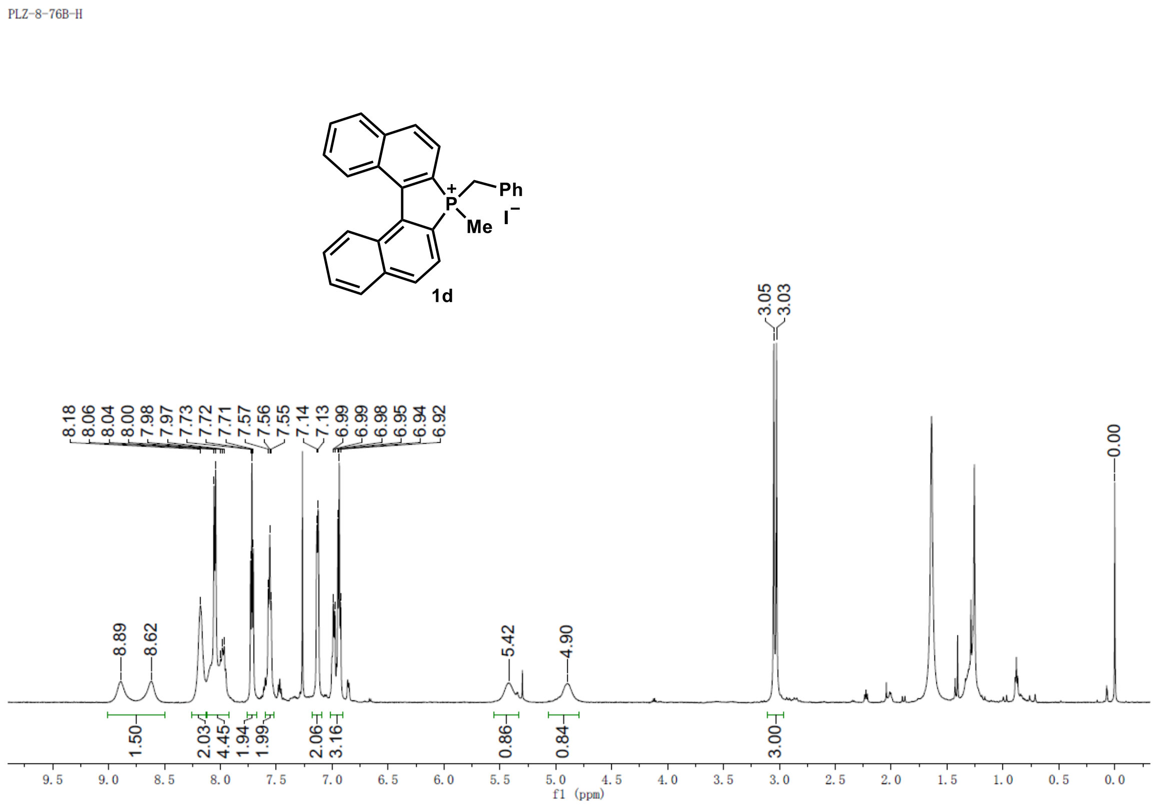

Supplementary Fig. 7. ^{1}H NMR spectrum of **1d**. The sample has been recorded in 600 MHz, $CDCl_3$ at 25 ℃.

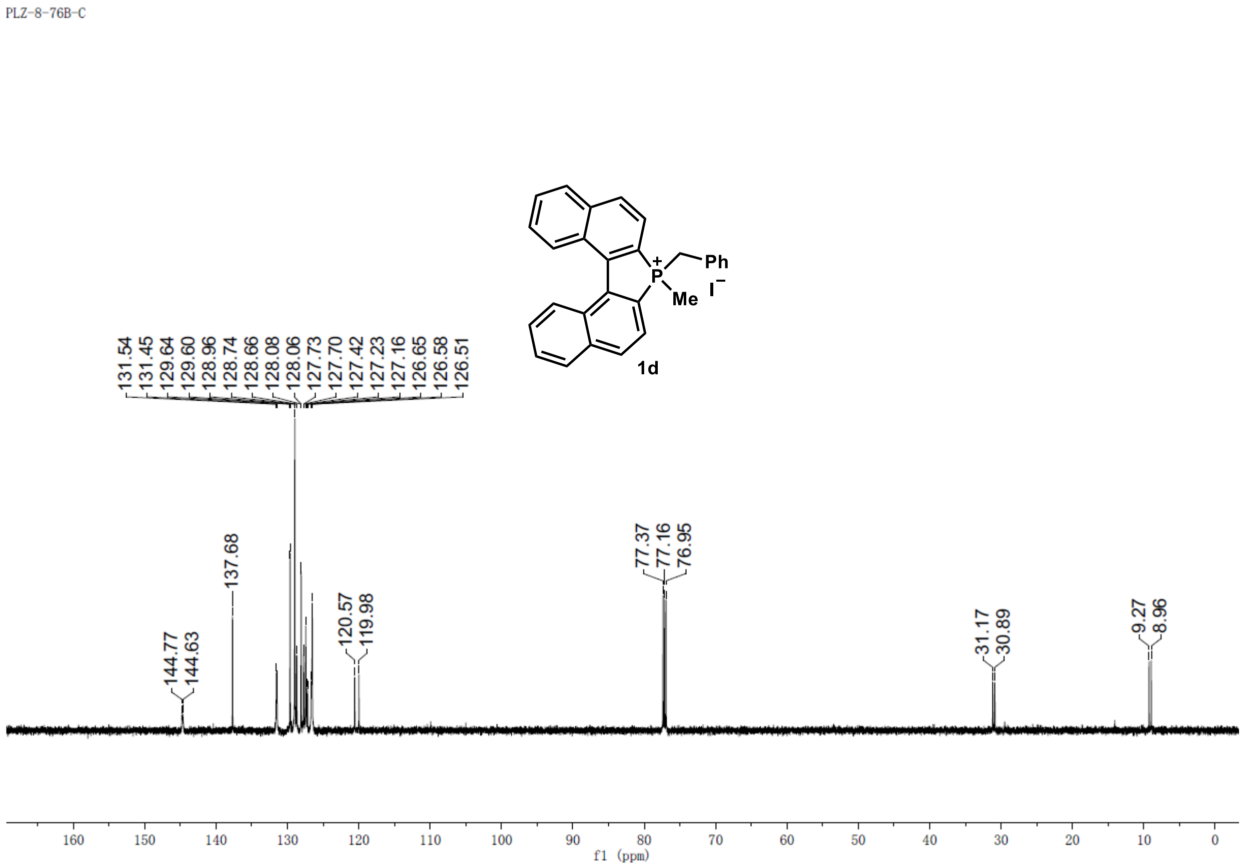

Supplementary Fig. 8.^{13}C NMR spectrum of **1d**. The sample has been recorded in 151 MHz, $CDCl_3$ at 25 ℃.

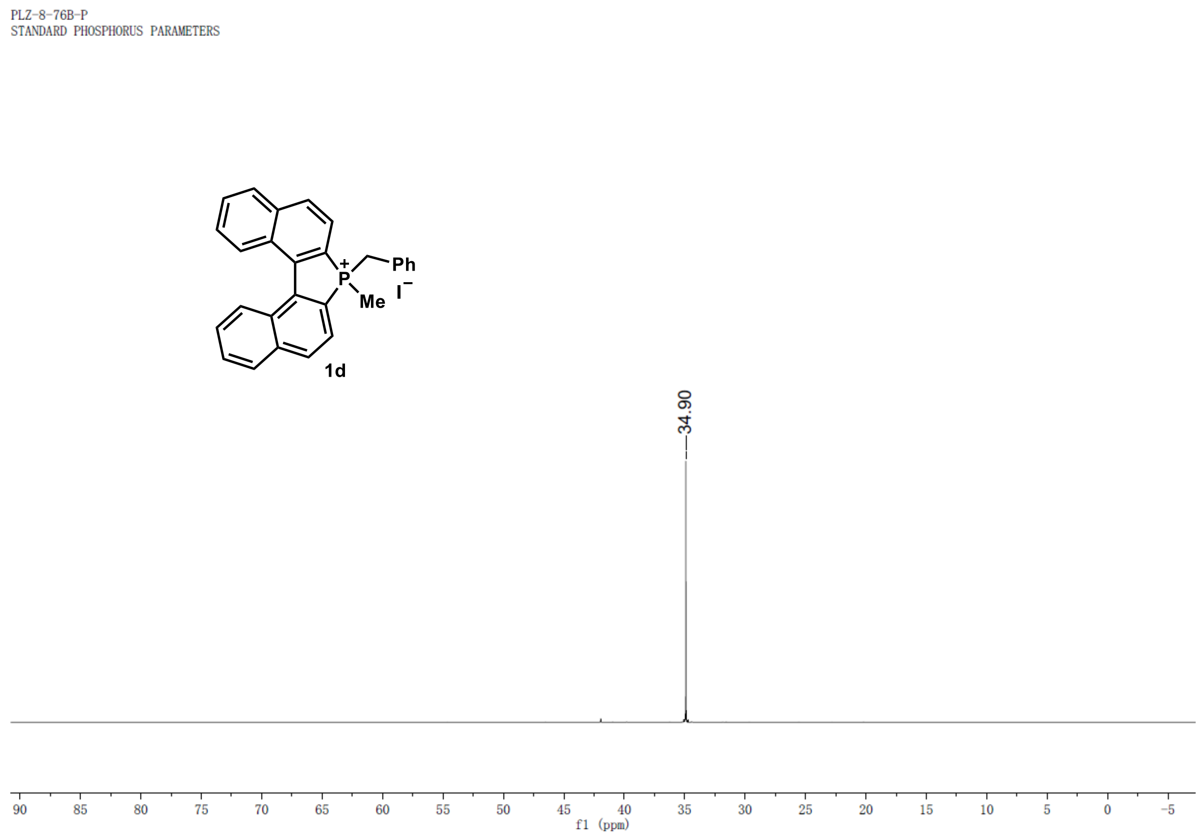

Supplementary Fig. 9.^{31}P NMR spectrum of **1d**. The sample has been recorded in 243 MHz, $CDCl_3$ at 25 ℃.

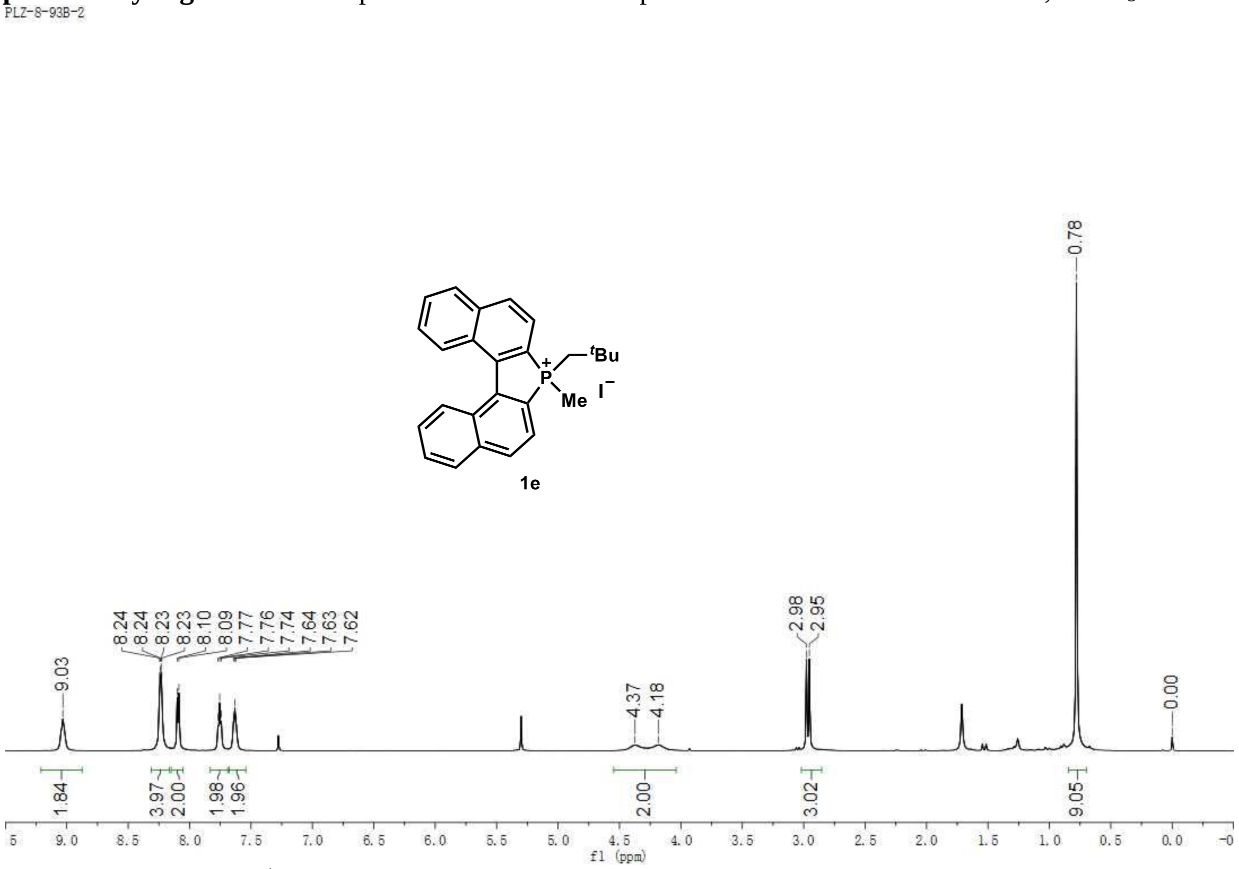

Supplementary Fig. 10. ^{1}H NMR spectrum of **1e**. The sample has been recorded in 600 MHz, $CDCl_3$ at 25 ℃.

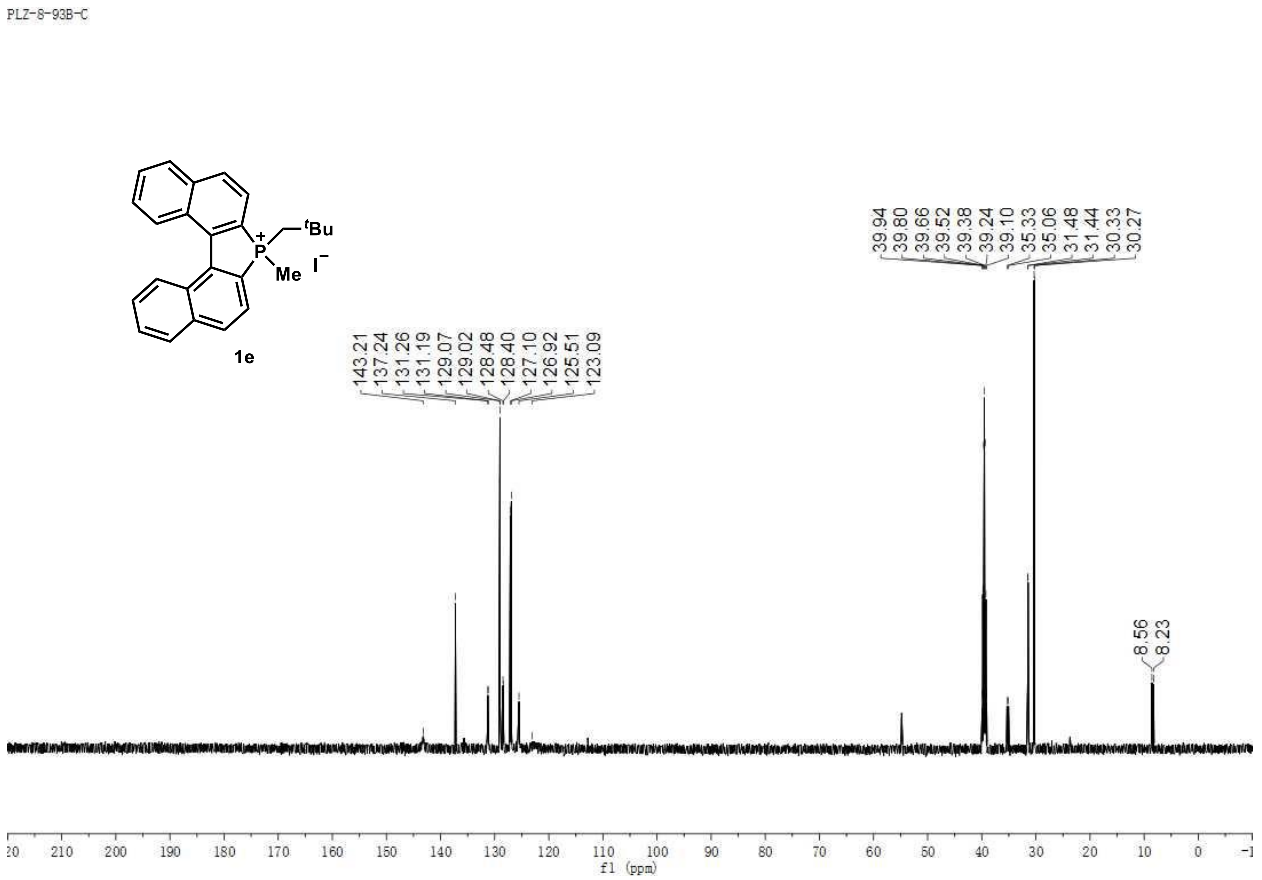

Supplementary Fig. 11.^{13}C NMR spectrum of **1e**. The sample has been recorded in 151 MHz, $CDCl_3$ at 25 ℃.

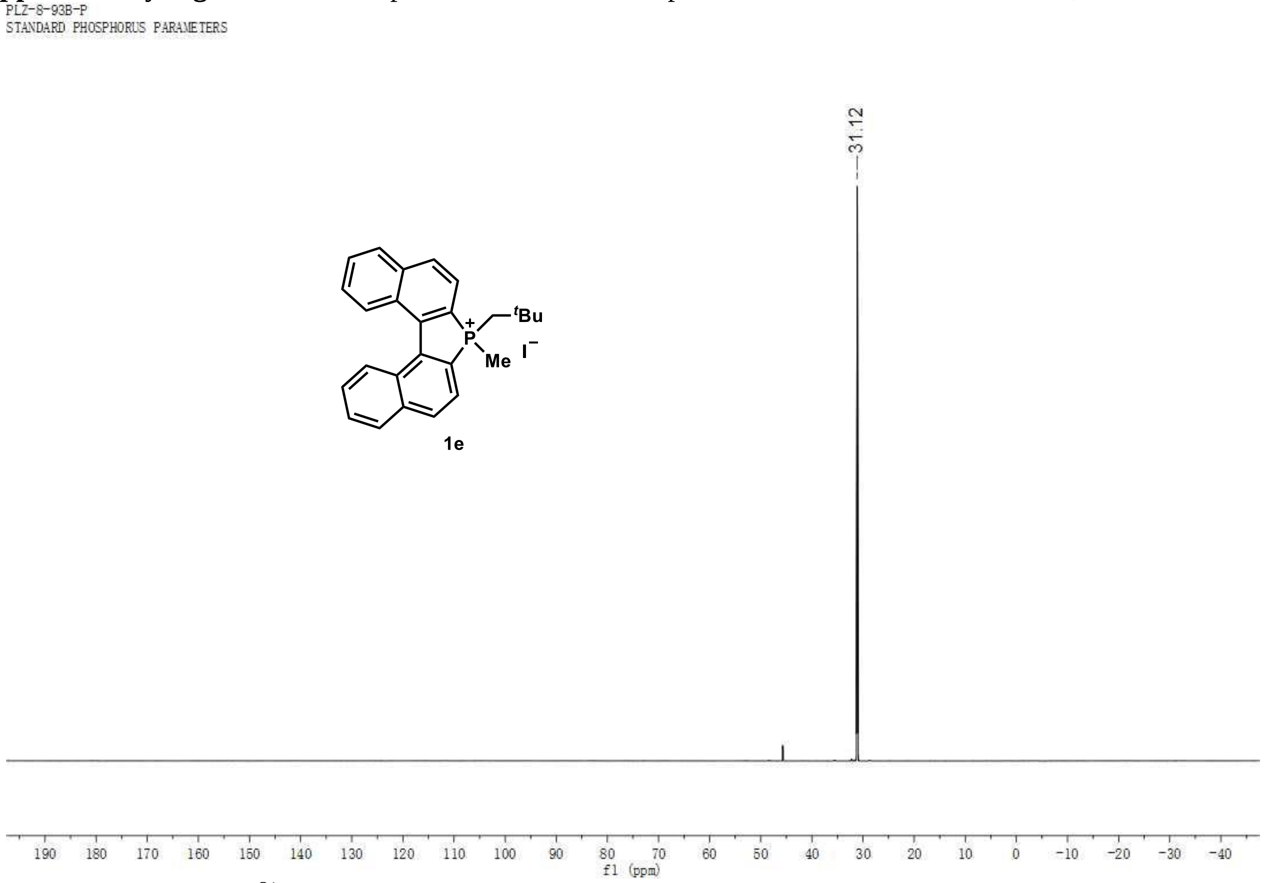

Supplementary Fig. 12.^{31}P NMR spectrum of **1e**. The sample has been recorded in 243 MHz, $CDCl_3$ at 25 ℃.

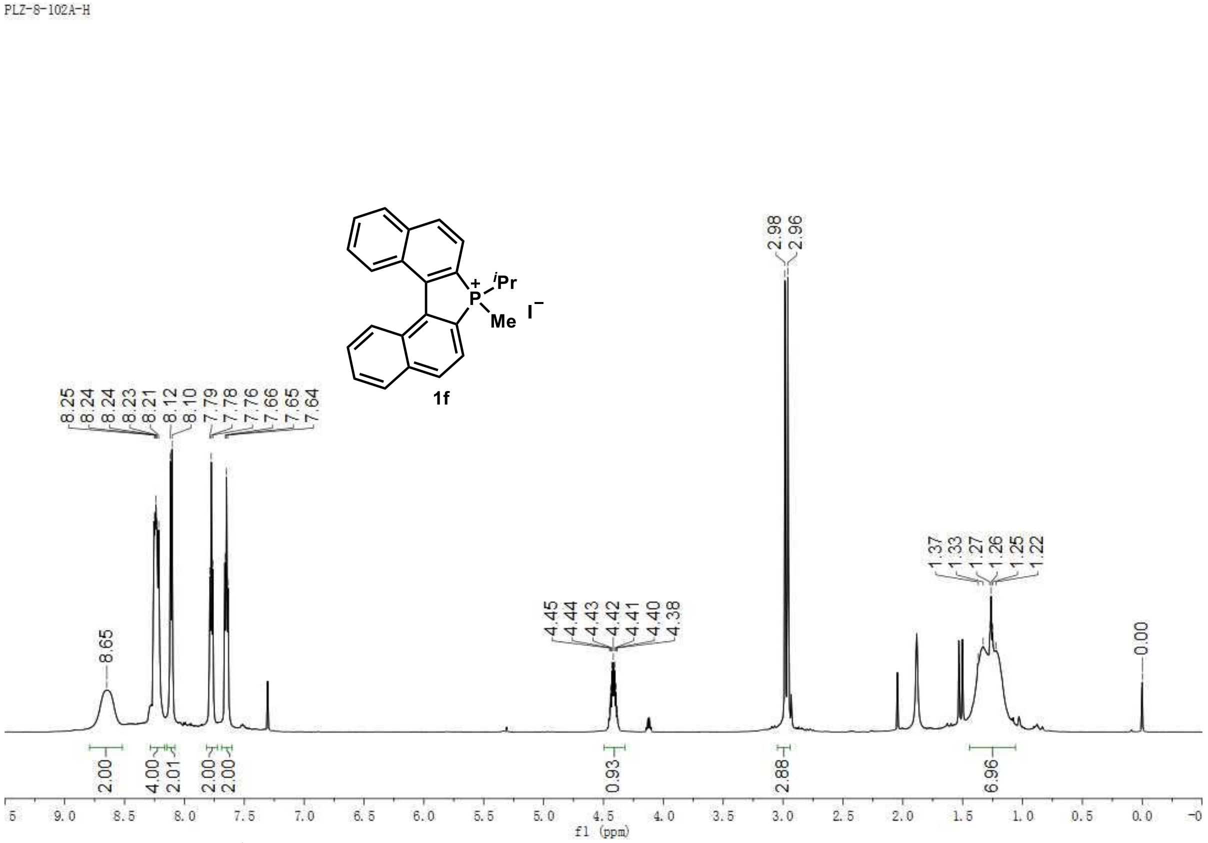

Supplementary Fig. 13. ^{1}H NMR spectrum of **1f**. The sample has been recorded in 600 MHz, $CDCl_3$ at 25 ℃.

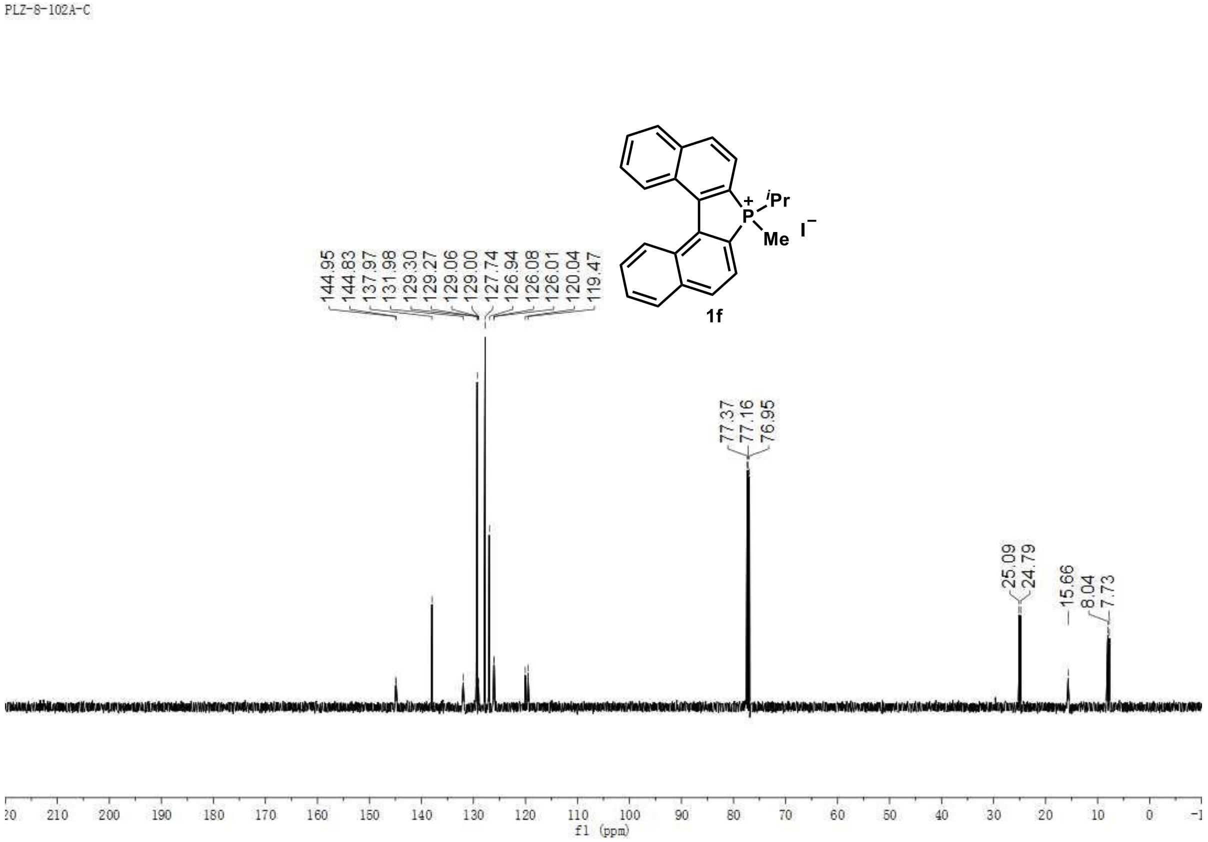

Supplementary Fig. 14. ^{13}C NMR spectrum of **1f**. The sample has been recorded in 151 MHz, $CDCl_3$ at 25 ℃.

PLZ-8-102A-P
STANDARD PHOSPHORUS PARAMETERS

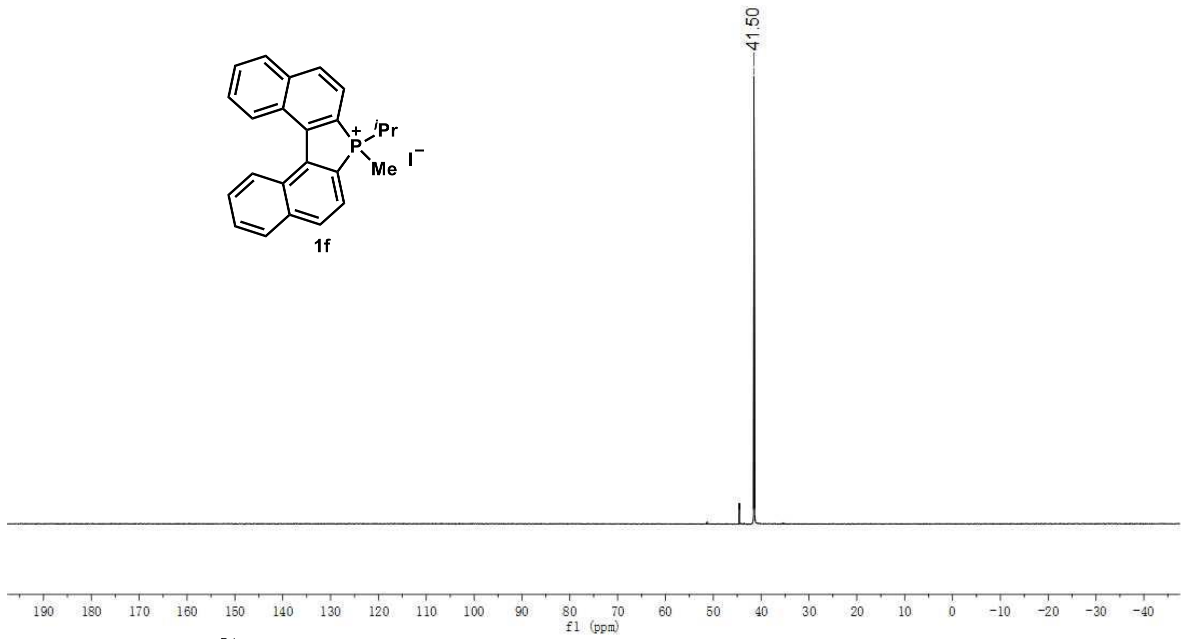

Supplementary Fig. 15.^{31}P NMR spectrum of **1f**. The sample has been recorded in 243 MHz, $CDCl_3$ at 25 ℃.

PLZ-8-102B

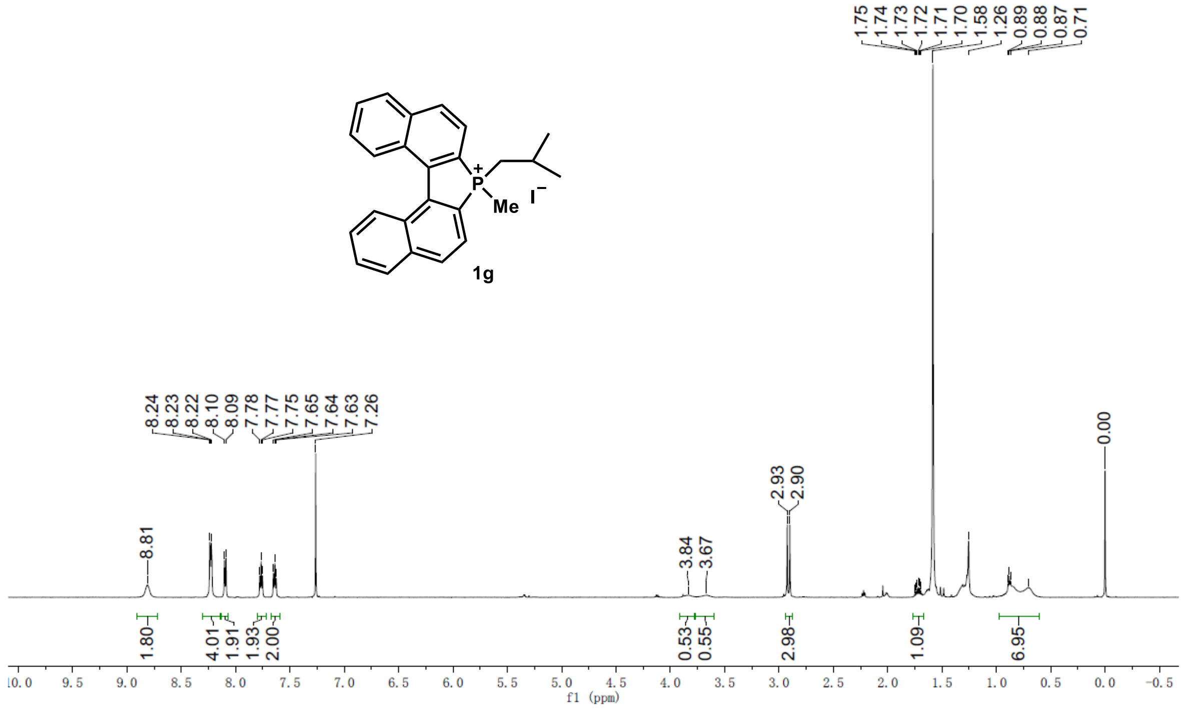

Supplementary Fig. 16. ^{1}H NMR spectrum of **1g**. The sample has been recorded in 600 MHz, $CDCl_3$ at 25 ℃.

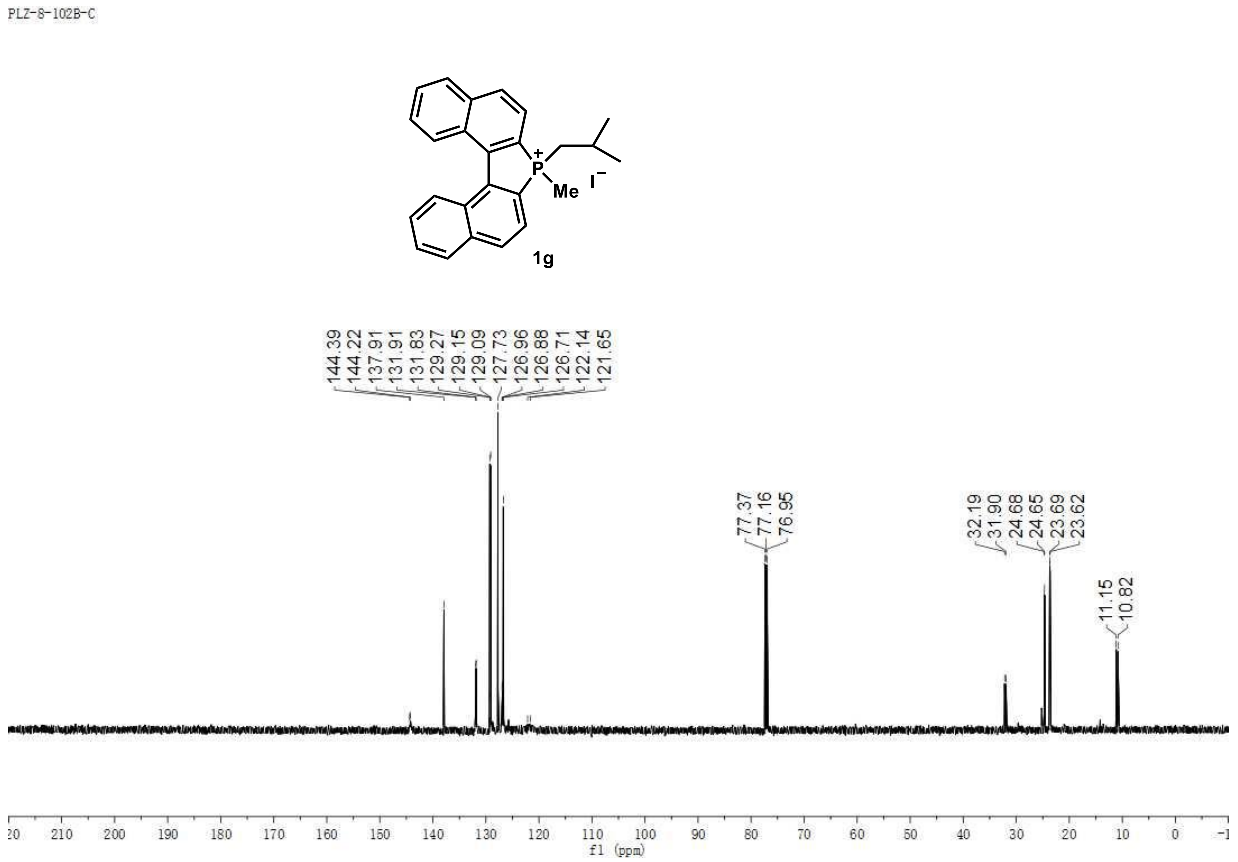

Supplementary Fig. 17.^{13}C NMR spectrum of **1g**. The sample has been recorded in 151 MHz, $CDCl_3$ at 25 ℃.

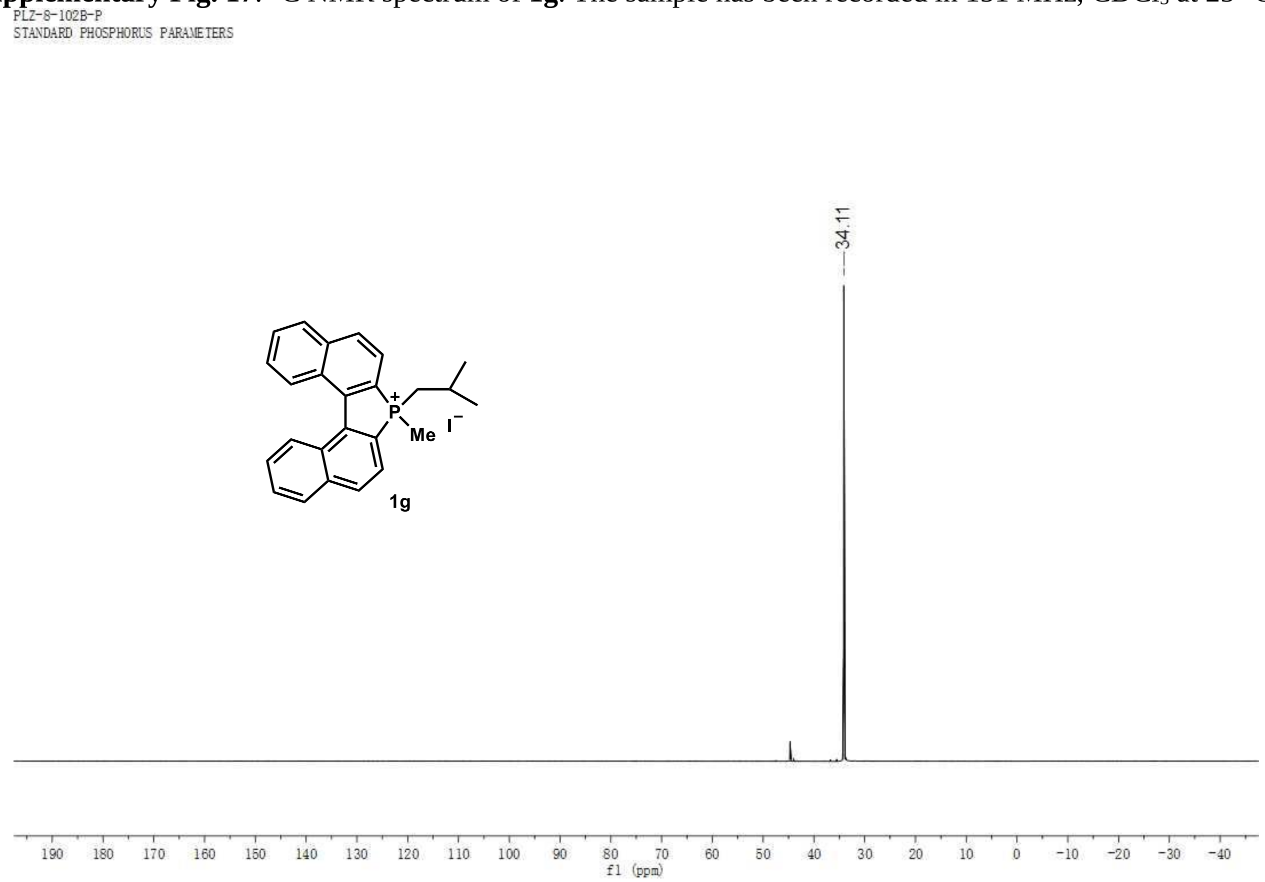

Supplementary Fig. 18.^{31}P NMR spectrum of **1g**. The sample has been recorded in 243 MHz, $CDCl_3$ at 25 ℃.

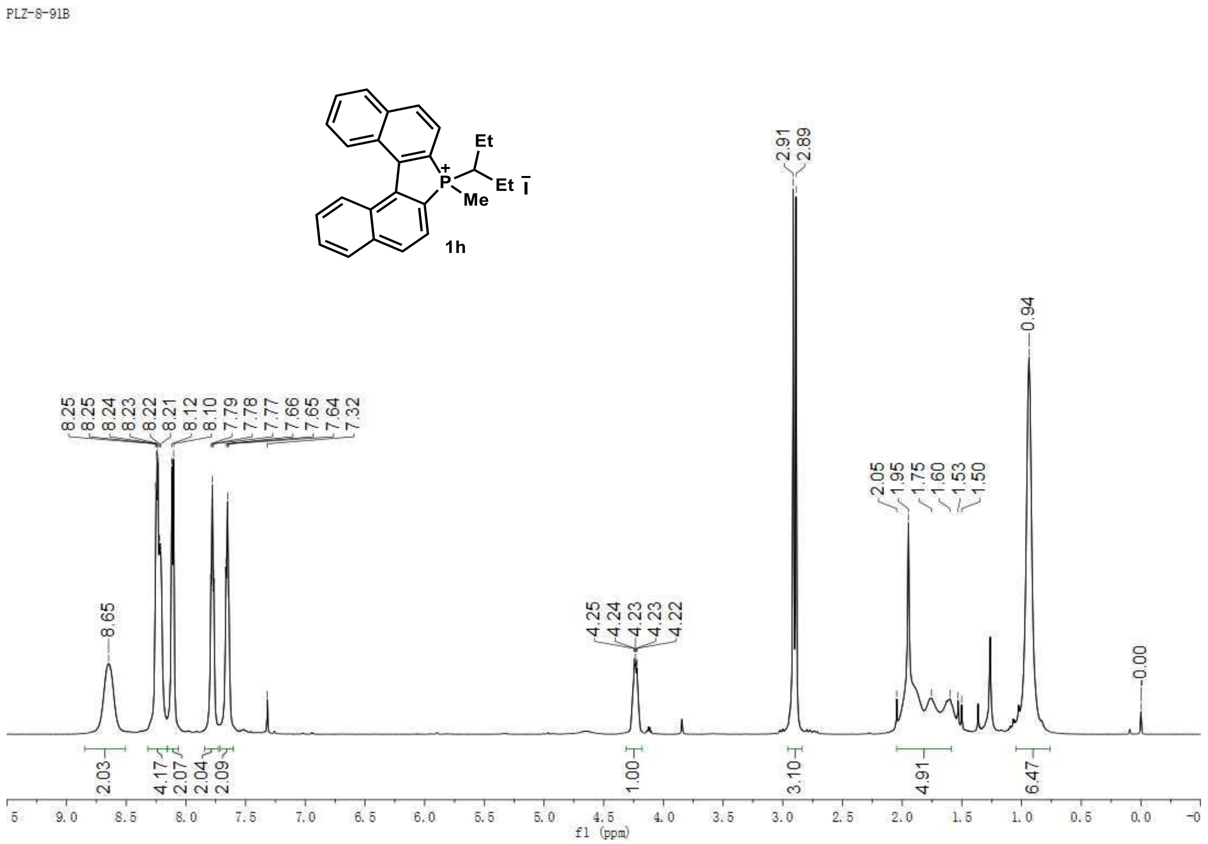

Supplementary Fig. 19. ^{1}H NMR spectrum of **1h**. The sample has been recorded in 600 MHz, $CDCl_3$ at 25 ℃.

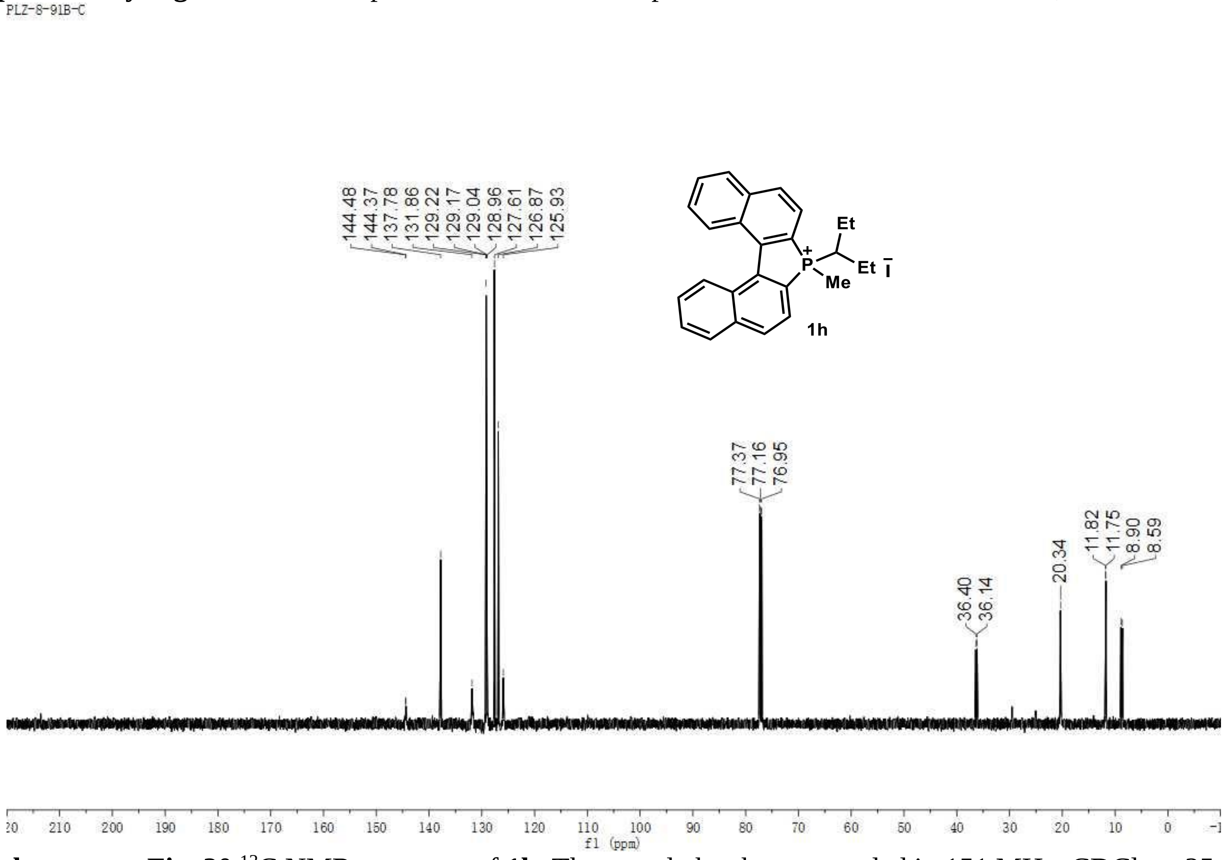

Supplementary Fig. 20.^{13}C NMR spectrum of **1h**. The sample has been recorded in 151 MHz, $CDCl_3$ at 25 ℃.

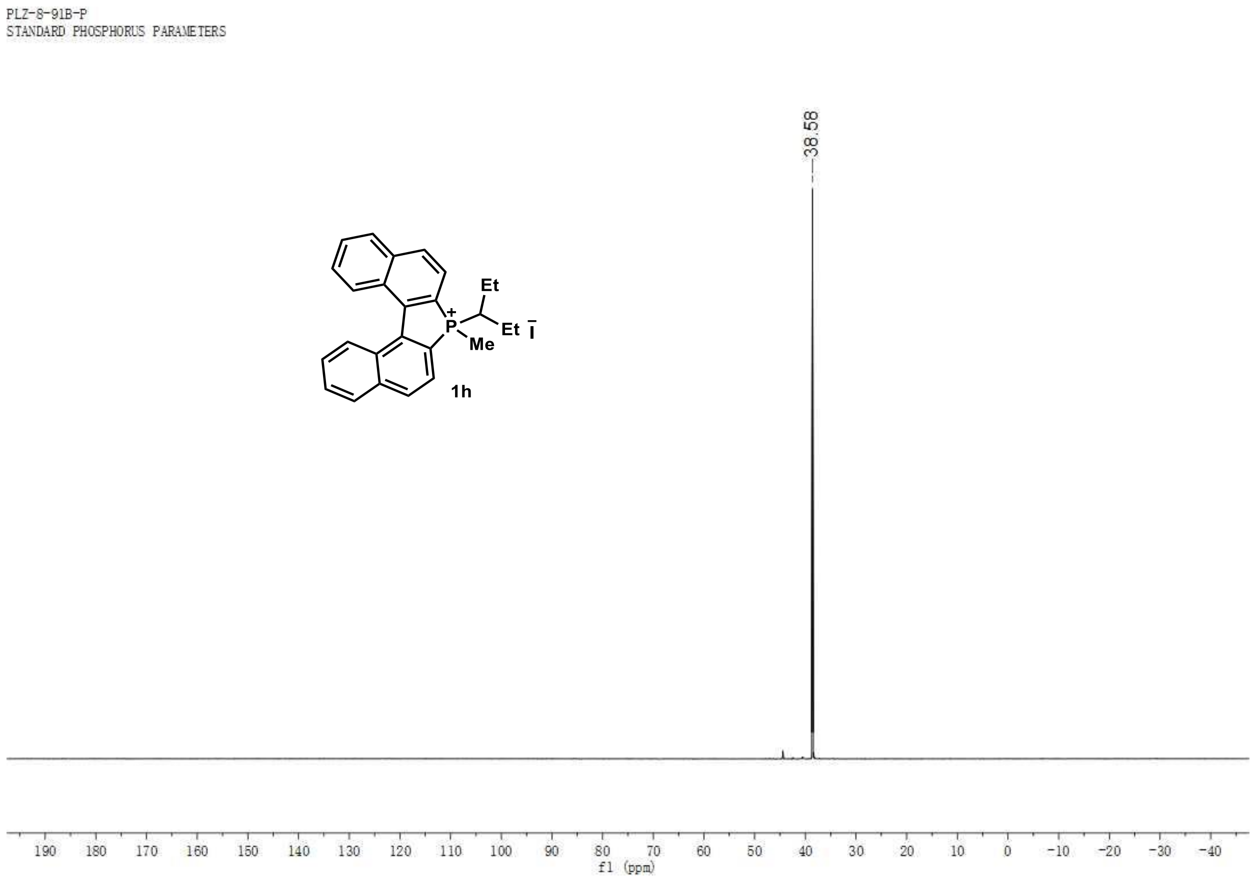

Supplementary Fig. 21.^{31}P NMR spectrum of **1h**. The sample has been recorded in 243 MHz, $CDCl_3$ at 25 ℃.

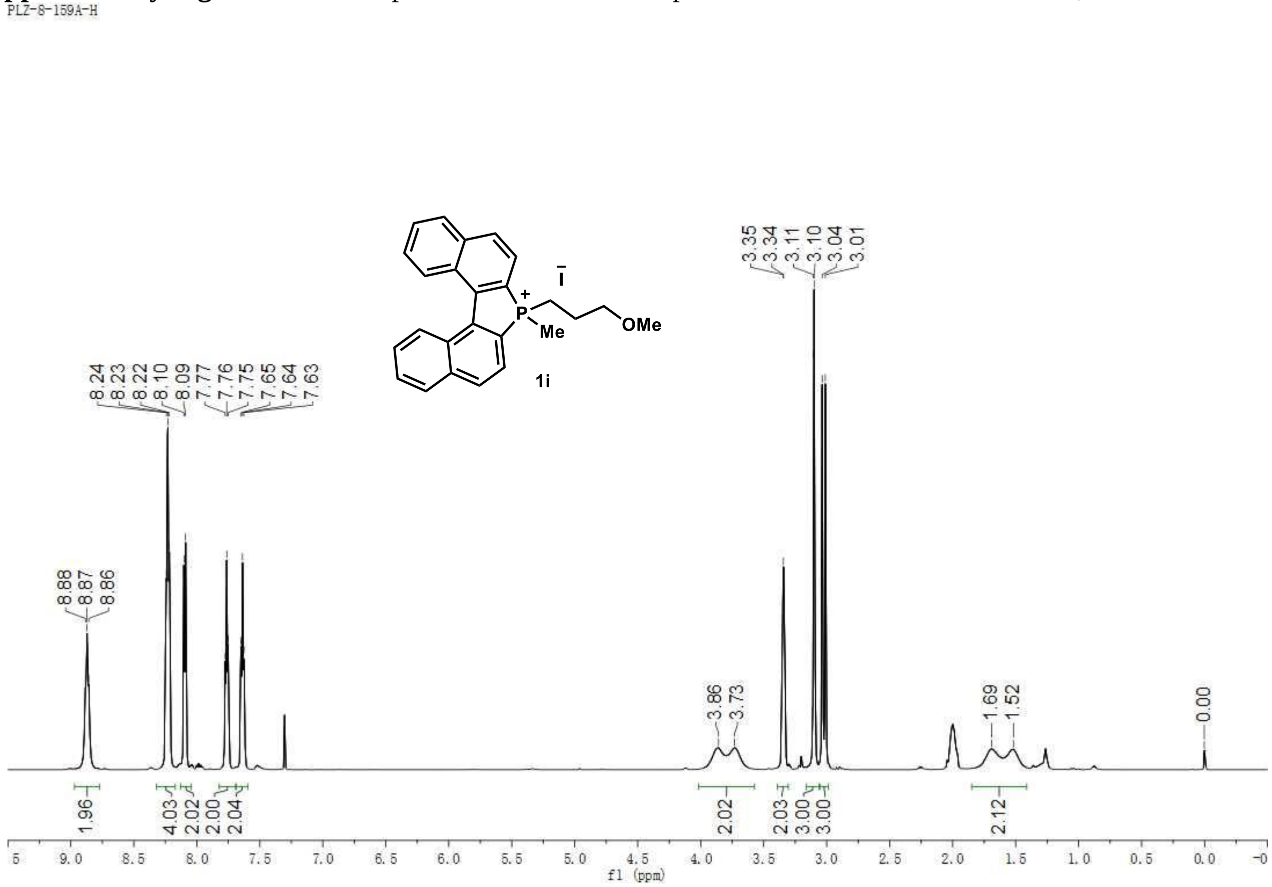

Supplementary Fig. 22. ^{1}H NMR spectrum of **1i**. The sample has been recorded in 600 MHz, $CDCl_3$ at 25 ℃.

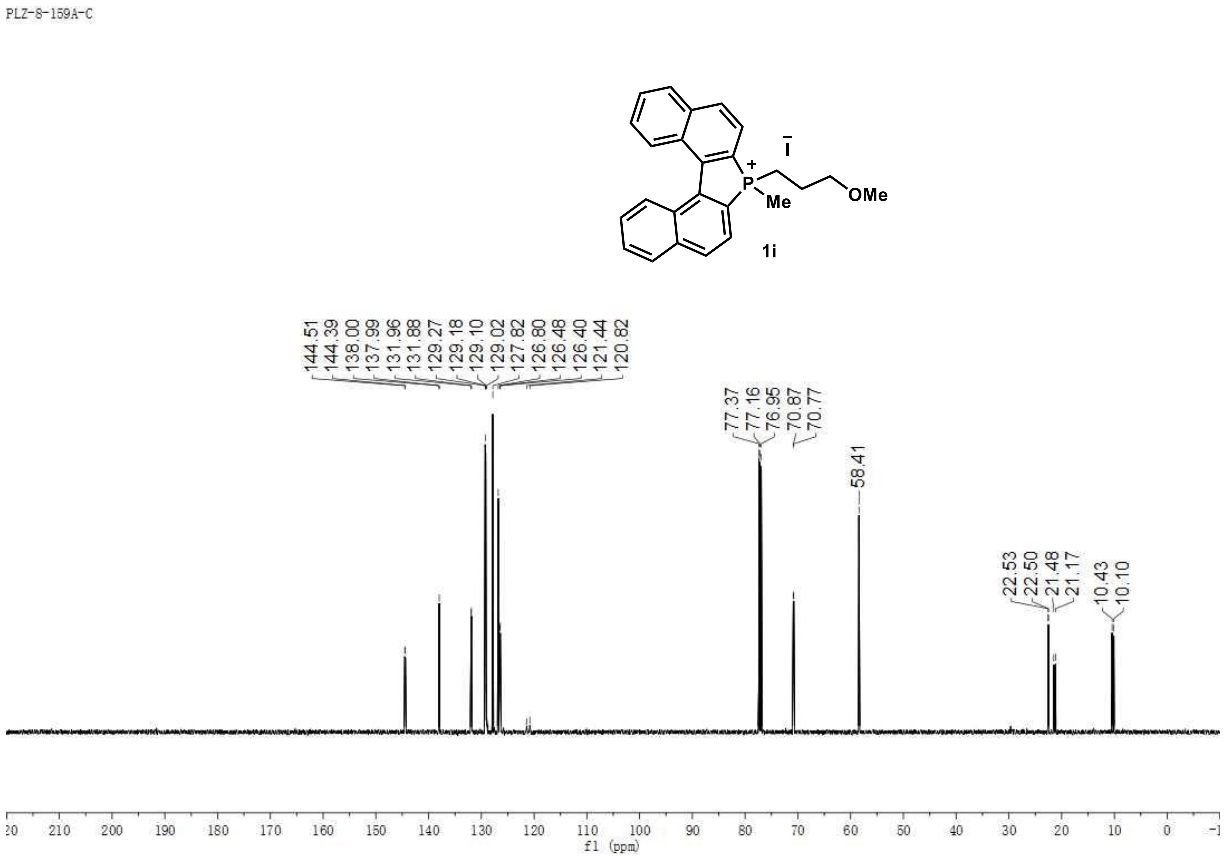

Supplementary Fig. 23.^{13}C NMR spectrum of **1i**. The sample has been recorded in 151 MHz, $CDCl_3$ at 25 ℃.

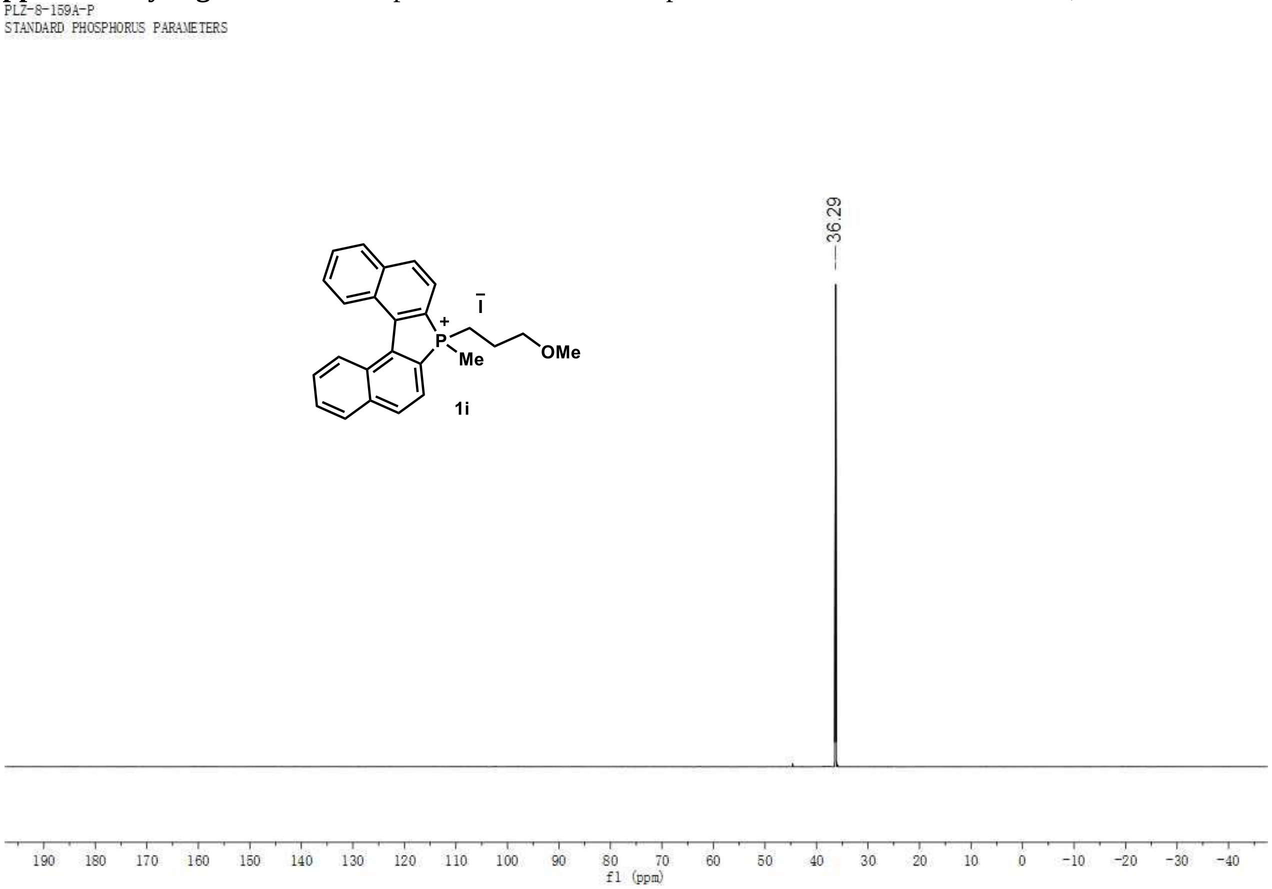

Supplementary Fig. 24.^{31}P NMR spectrum of **1i**. The sample has been recorded in 243 MHz, $CDCl_3$ at 25 ℃.

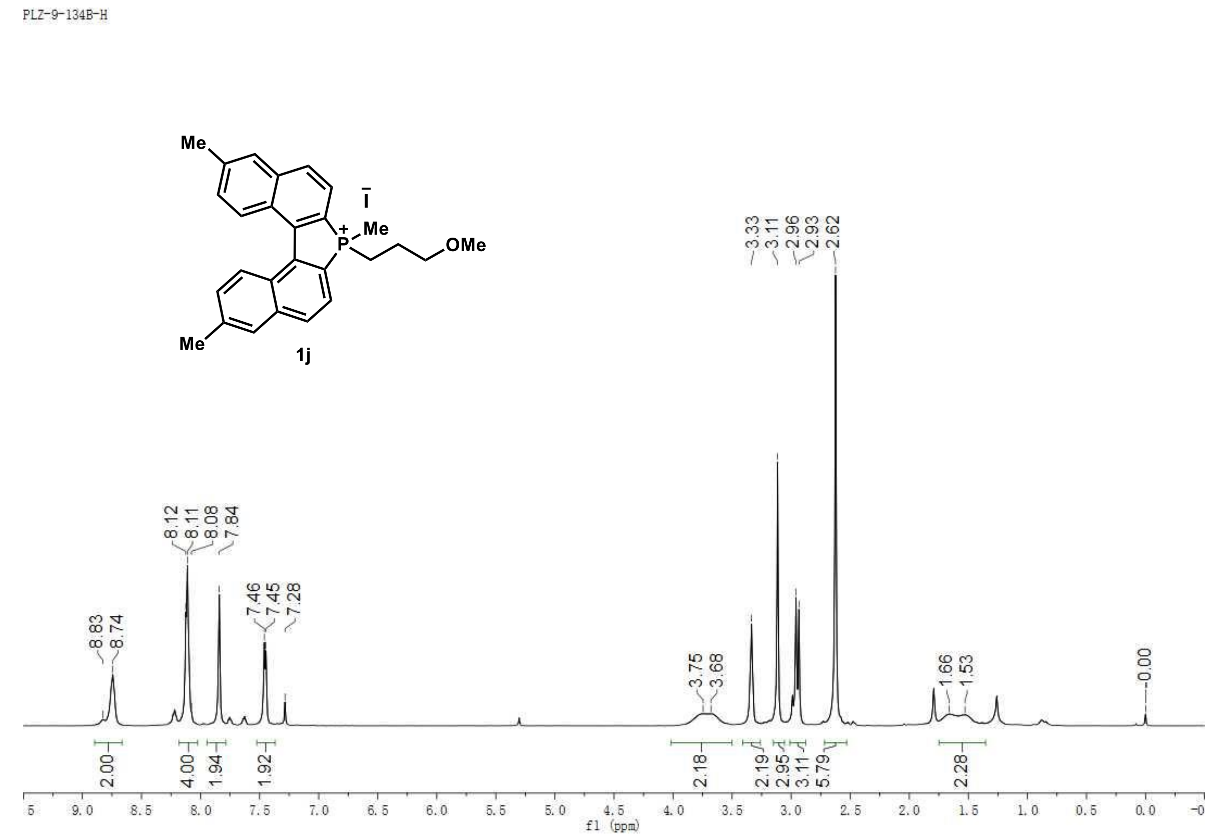

Supplementary Fig. 25. ^{1}H NMR spectrum of **1j**. The sample has been recorded in 600 MHz, $CDCl_3$ at 25 ℃.

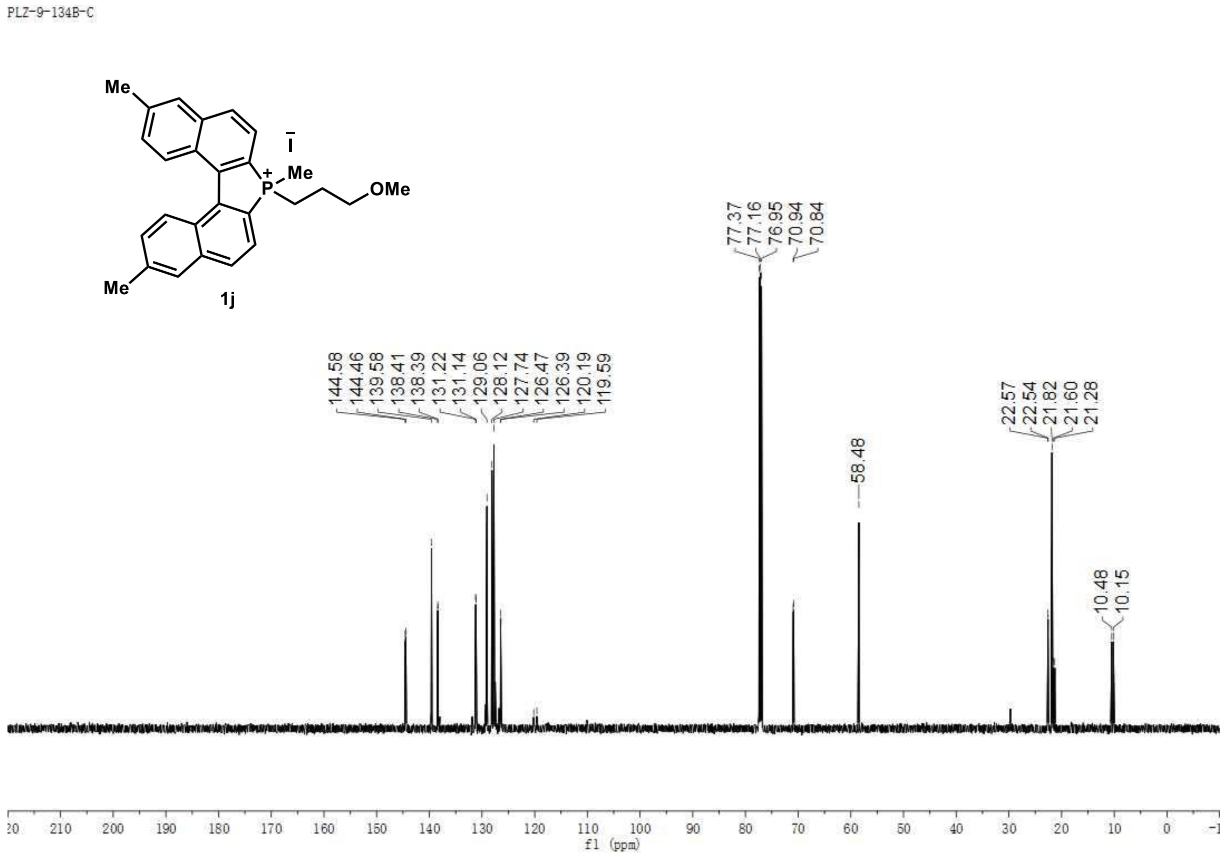

Supplementary Fig. 26.^{13}C NMR spectrum of **1j**. The sample has been recorded in 151 MHz, $CDCl_3$ at 25 ℃.

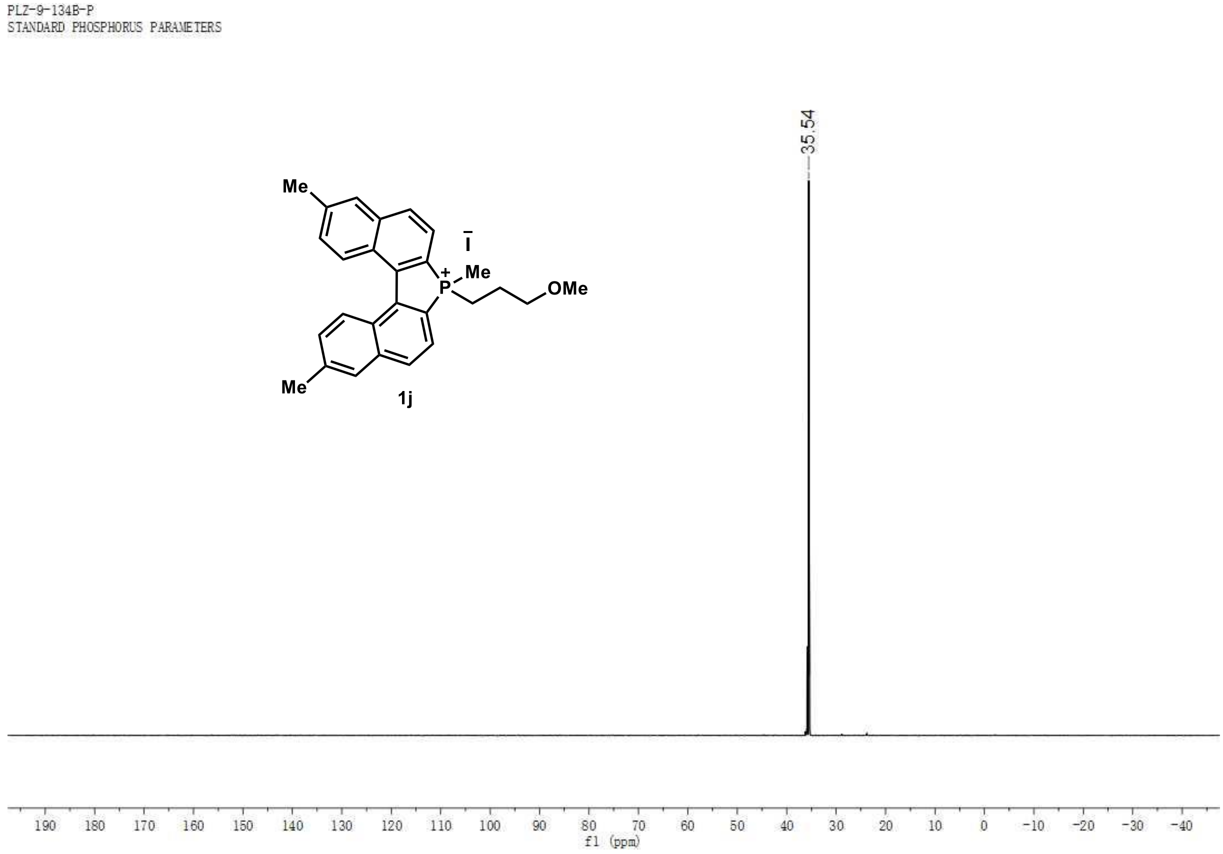

Supplementary Fig. 27.^{31}P NMR spectrum of **1j**. The sample has been recorded in 243 MHz, $CDCl_3$ at 25 ℃.

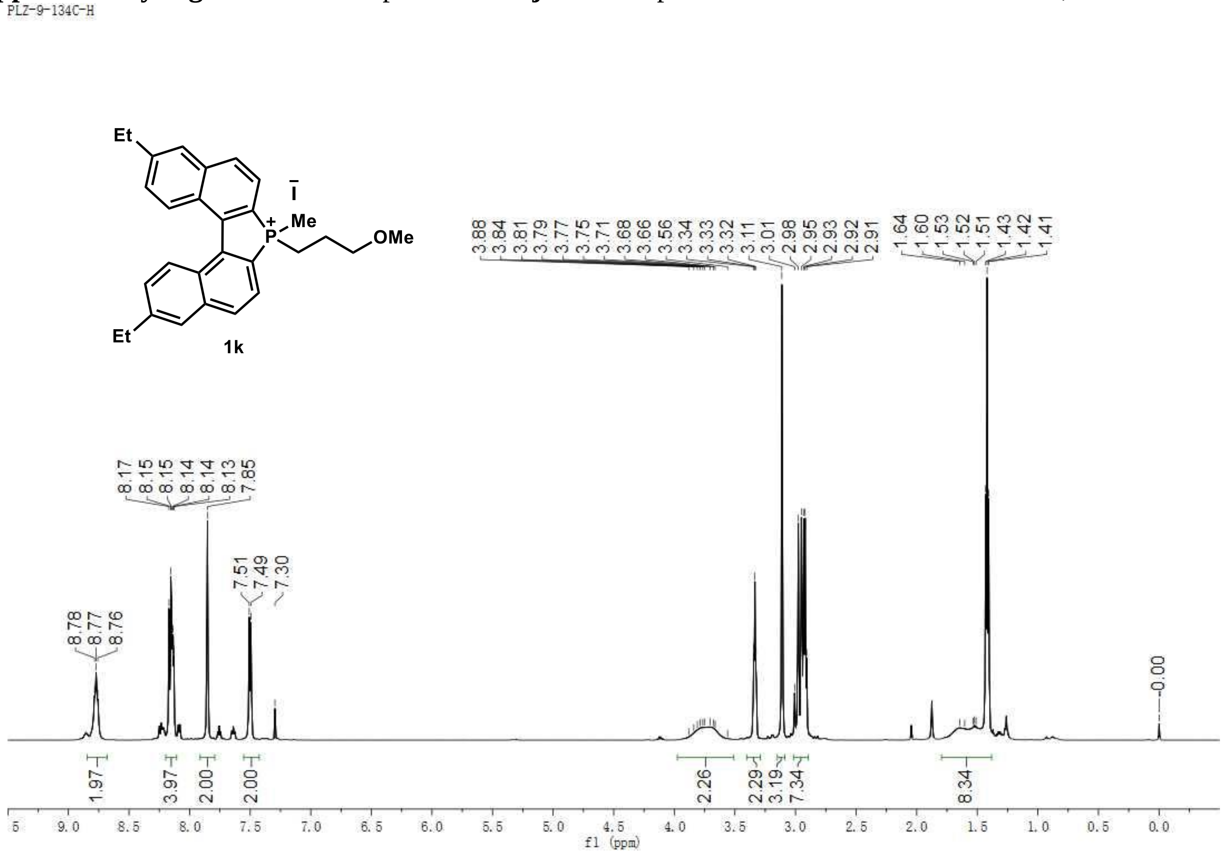

Supplementary Fig. 28. ^{1}H NMR spectrum of **1k**. The sample has been recorded in 600 MHz, $CDCl_3$ at 25 ℃.

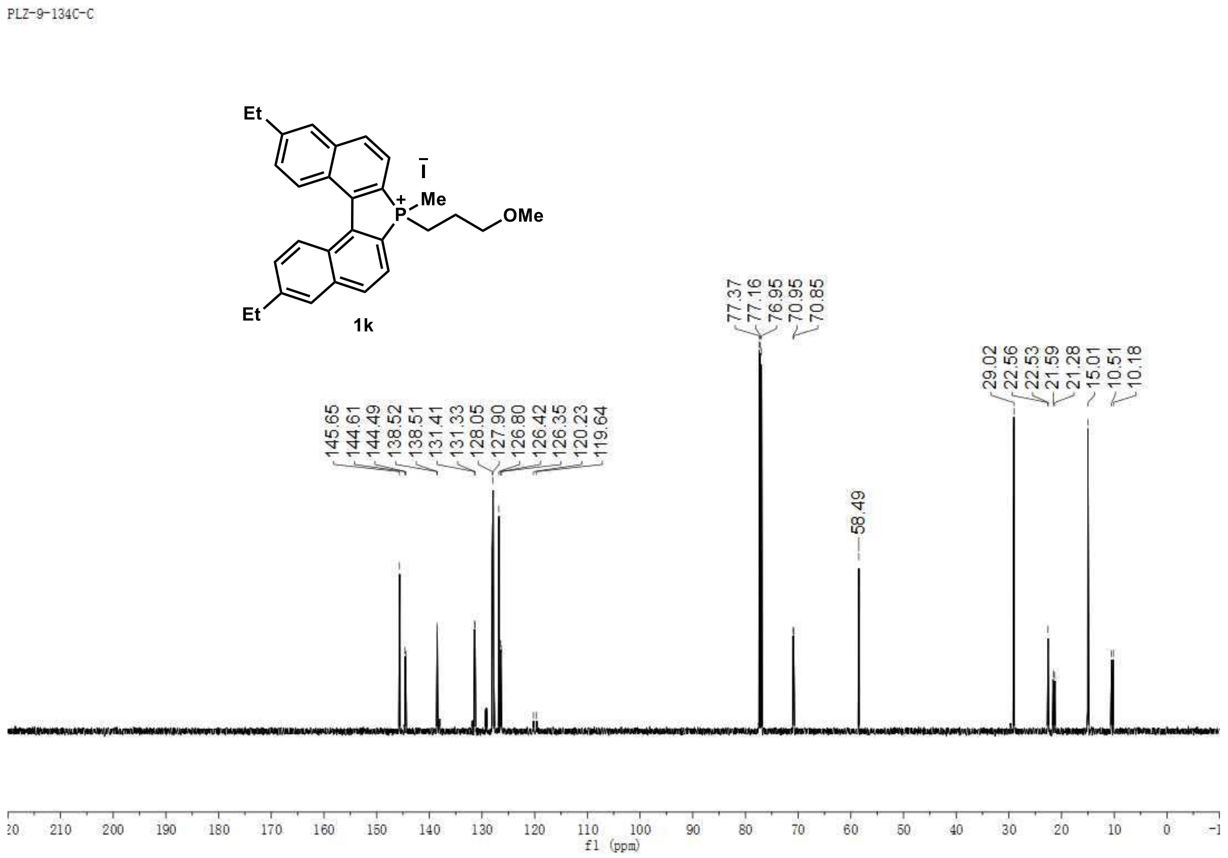

Supplementary Fig. 29.^{13}C NMR spectrum of **1k**. The sample has been recorded in 151 MHz, $CDCl_3$ at 25 ℃.

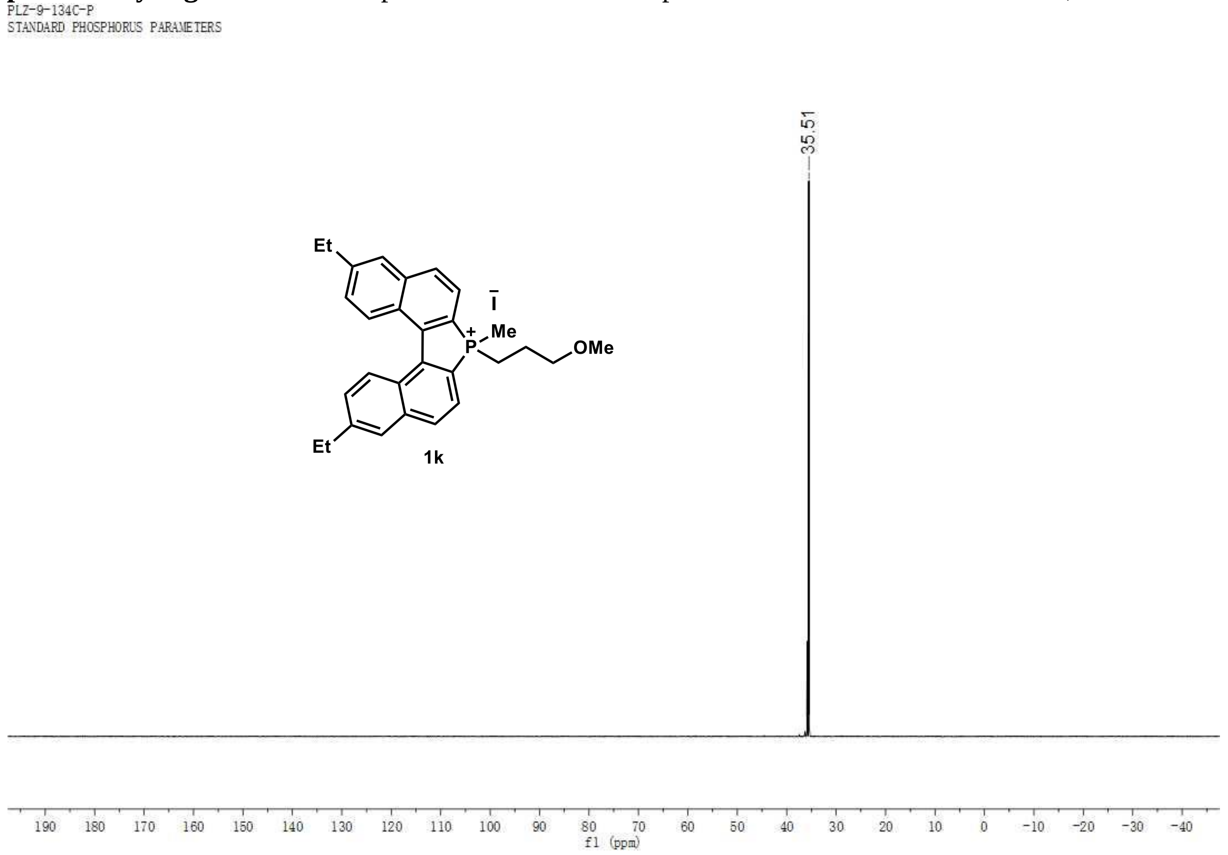

Supplementary Fig. 30.^{31}P NMR spectrum of **1k**. The sample has been recorded in 243 MHz, $CDCl_3$ at 25 ℃.

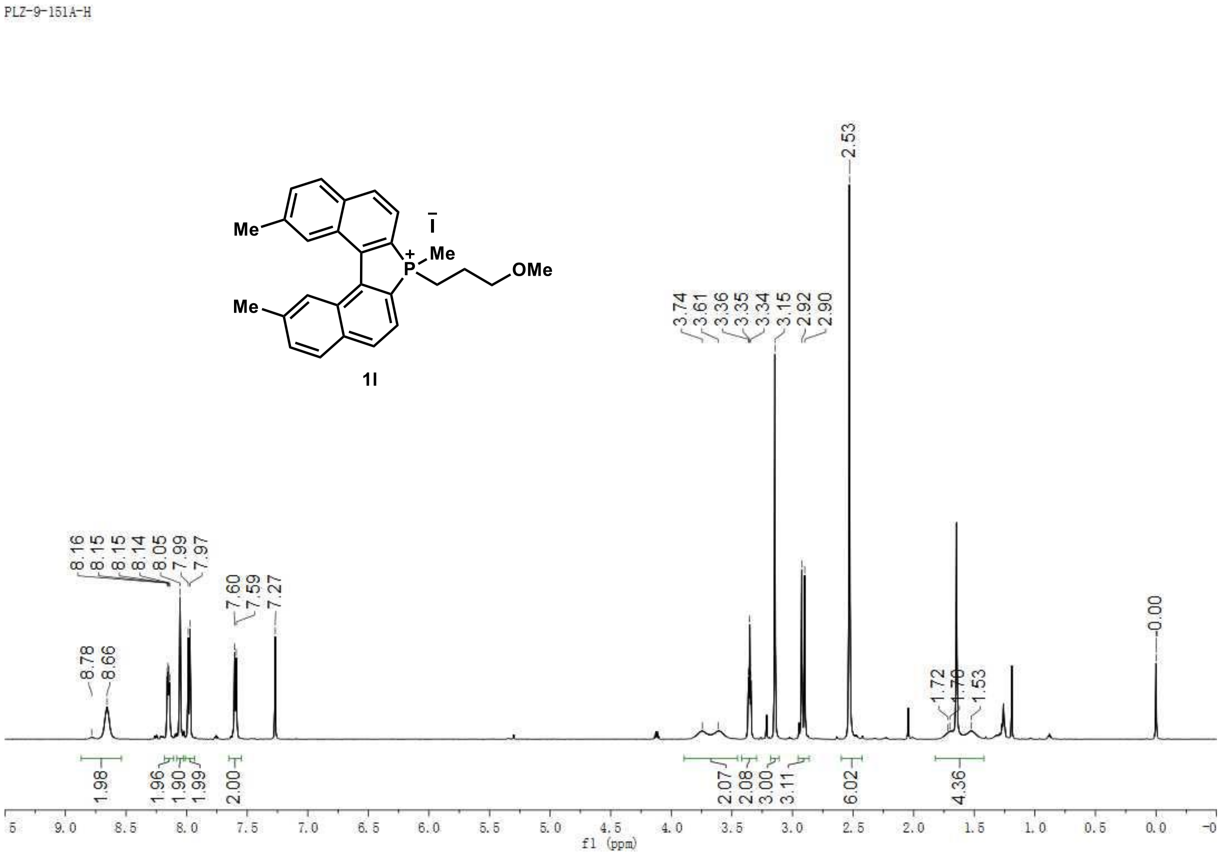

Supplementary Fig. 31. ^{1}H NMR spectrum of **1l**. The sample has been recorded in 600 MHz, $CDCl_3$ at 25 ℃.

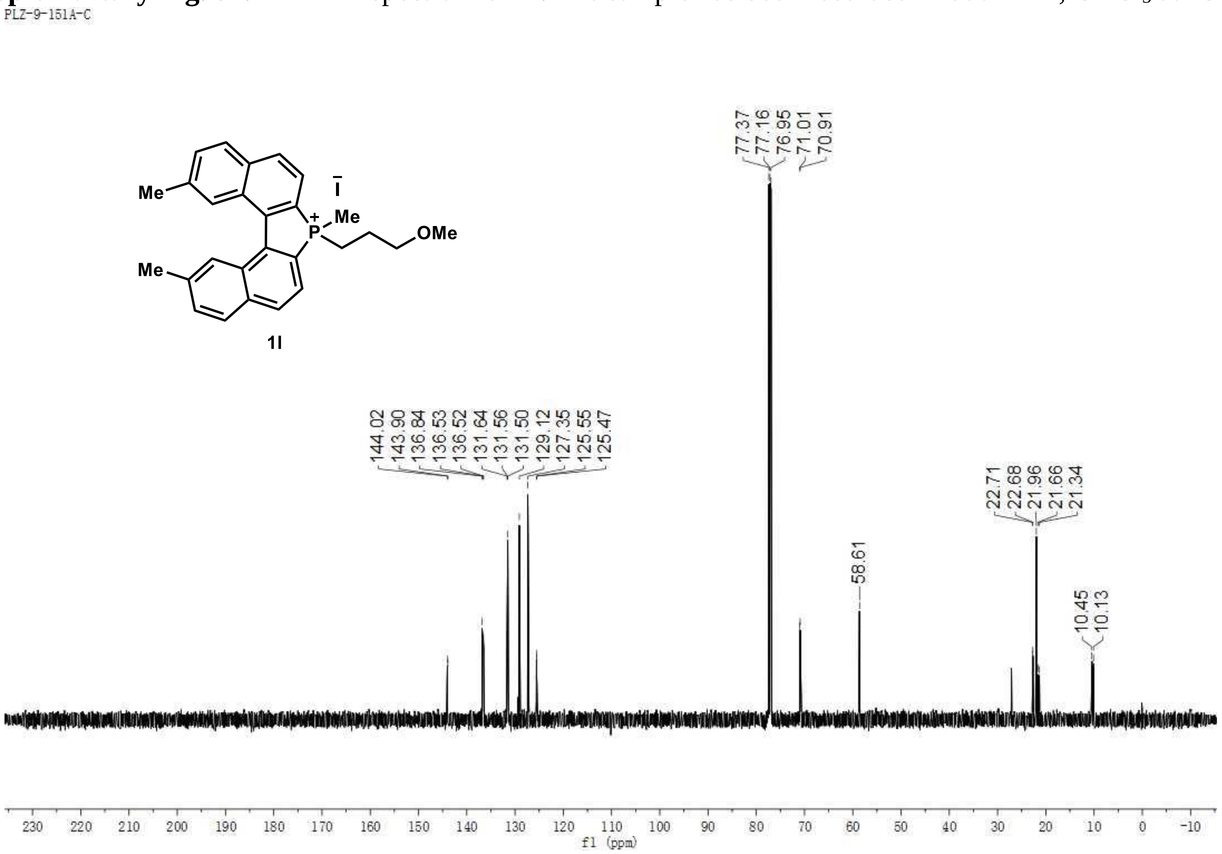

Supplementary Fig. 32.^{13}C NMR spectrum of **1l**. The sample has been recorded in 151 MHz, $CDCl_3$ at 25 ℃.

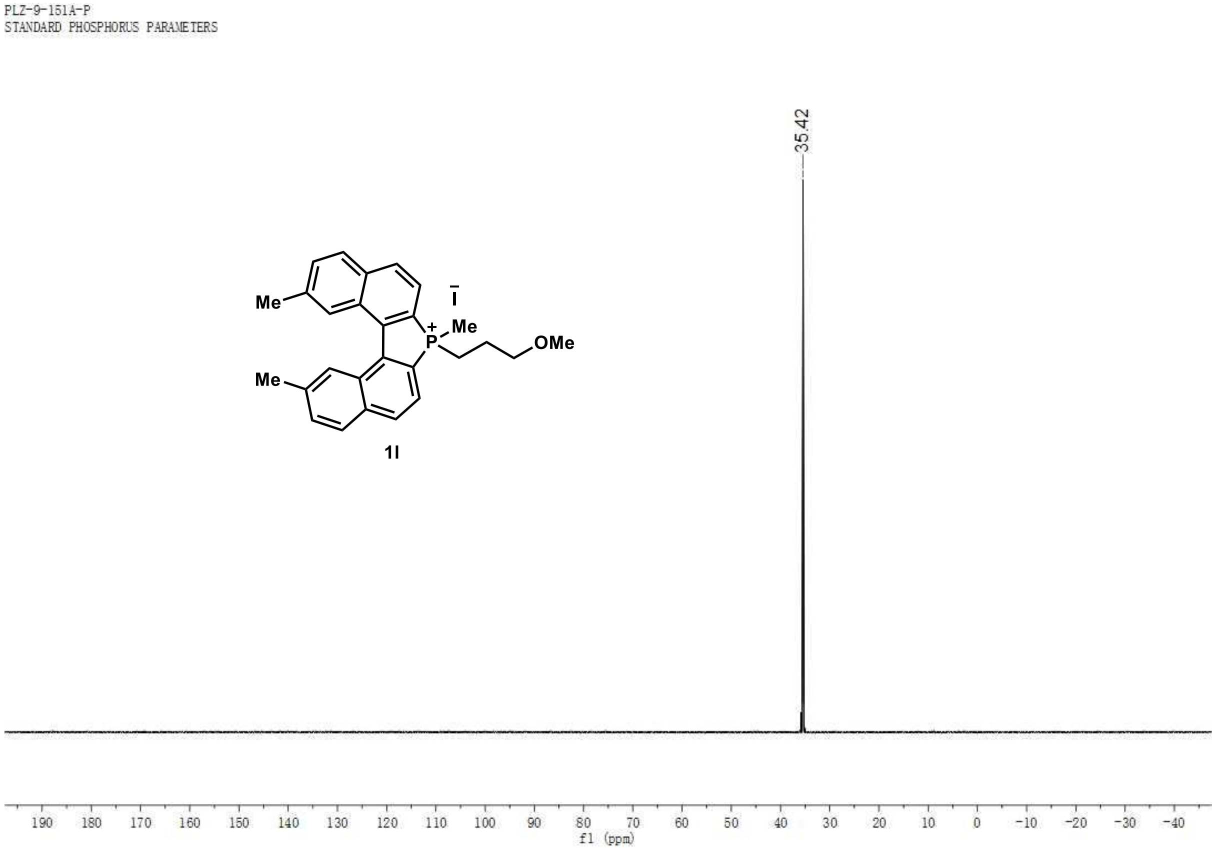

Supplementary Fig. 33.^{31}P NMR spectrum of **1l**. The sample has been recorded in 243 MHz, $CDCl_3$ at 25 ℃.

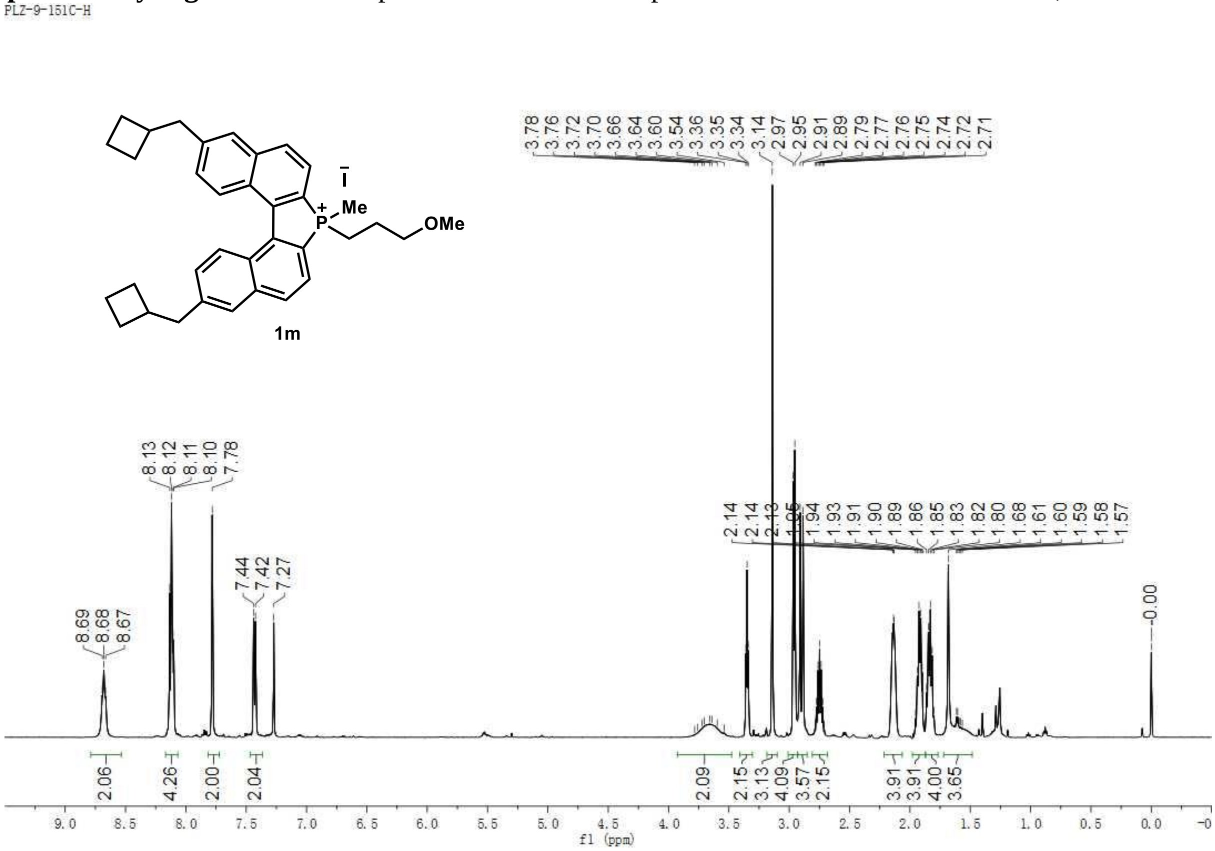

Supplementary Fig. 34. ^{1}H NMR spectrum of **1m**. The sample has been recorded in 600 MHz, $CDCl_3$ at 25 ℃.

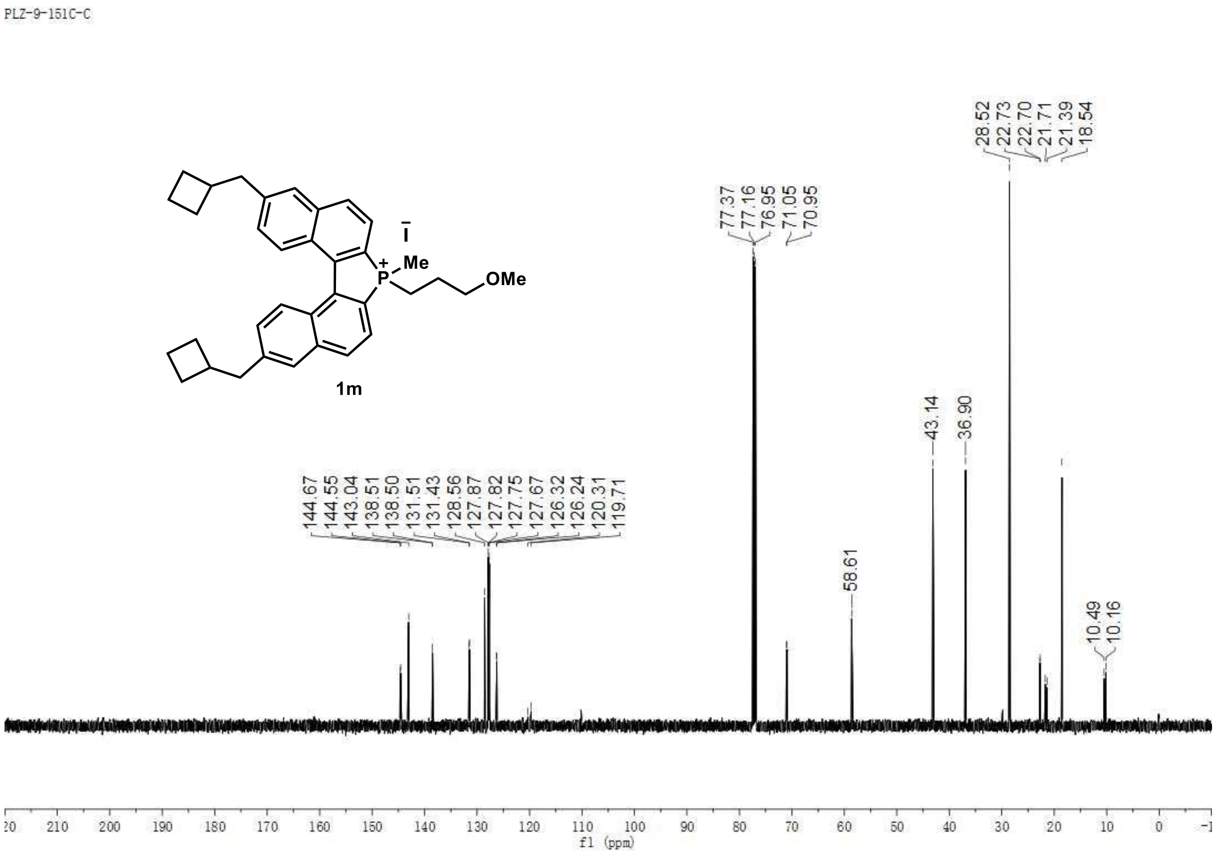

Supplementary Fig. 35.^{13}C NMR spectrum of **1m**. The sample has been recorded in 151 MHz, $CDCl_3$ at 25 ℃.

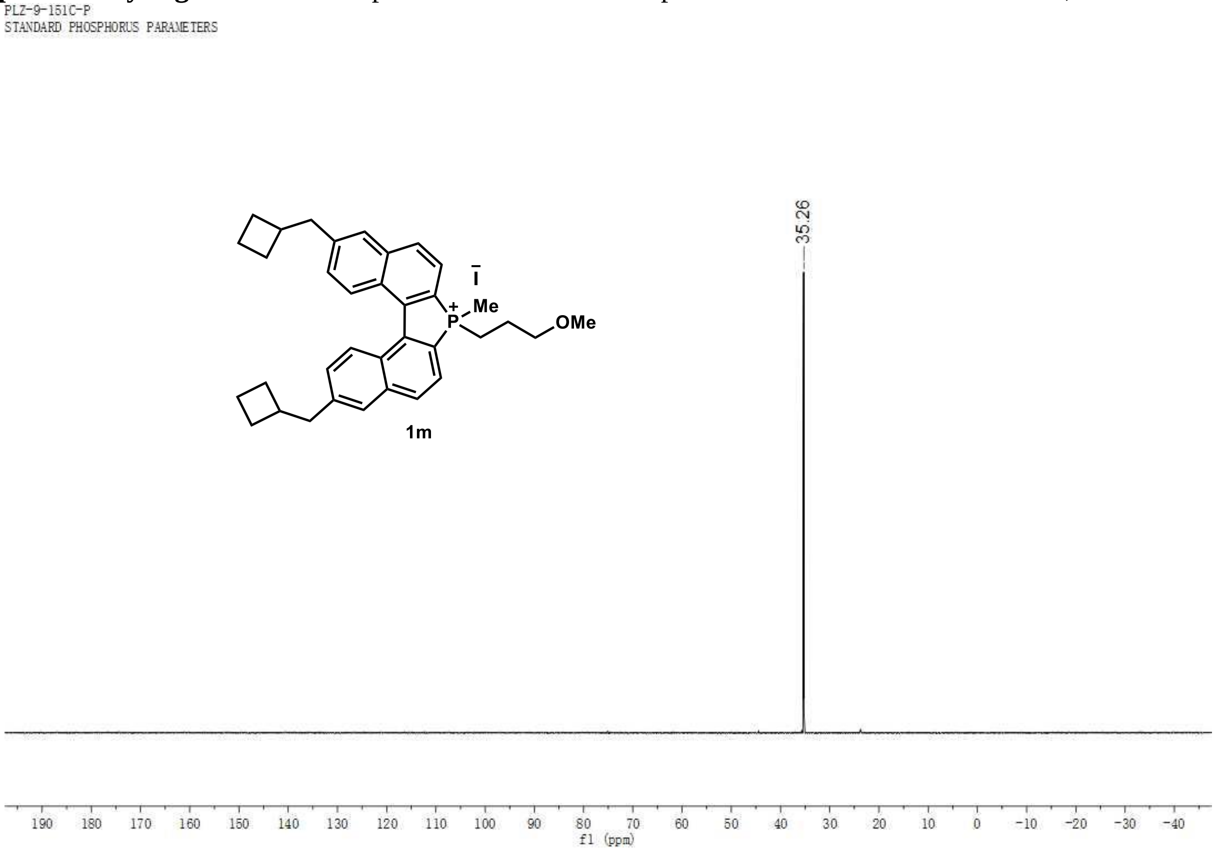

Supplementary Fig. 36.^{31}P NMR spectrum of **1m**. The sample has been recorded in 243 MHz, $CDCl_3$ at 25 ℃.

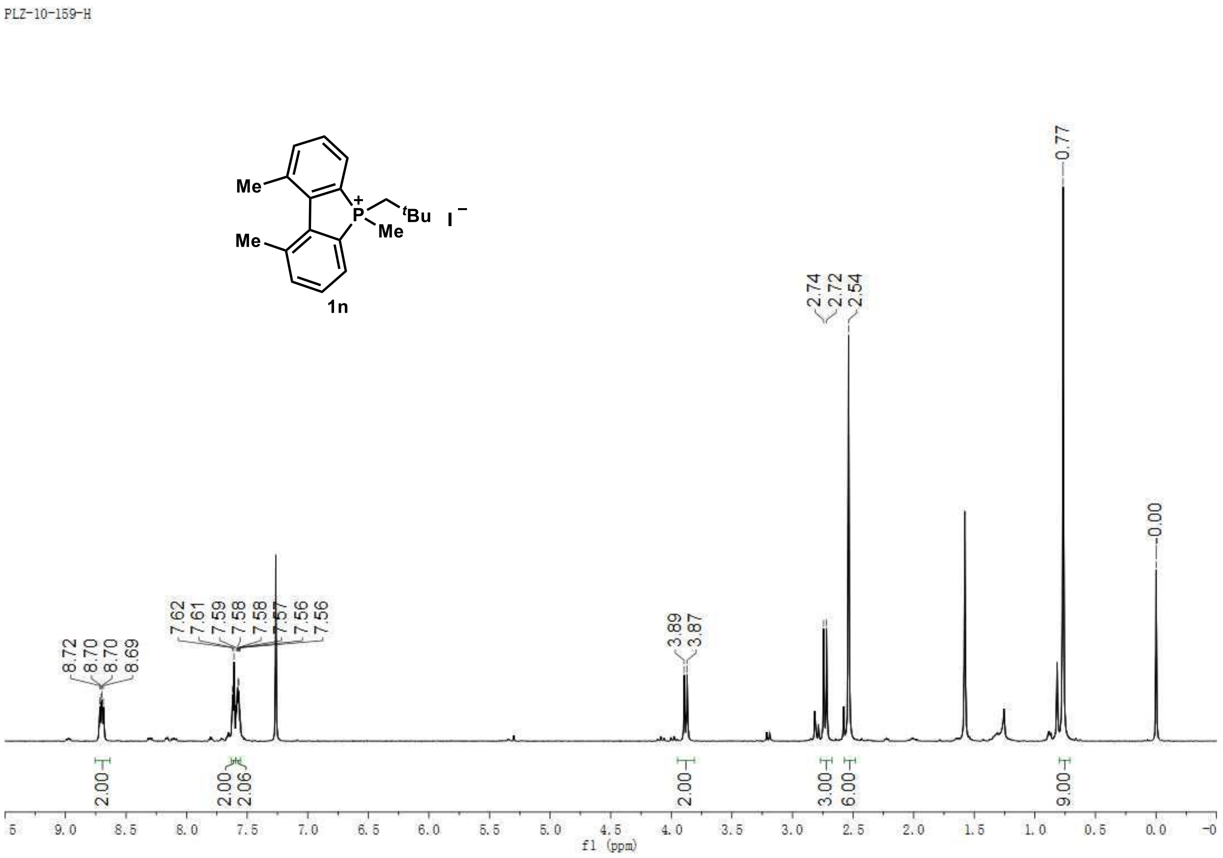

Supplementary Fig. 37. ^{1}H NMR spectrum of **1n**. The sample has been recorded in 600 MHz, $CDCl_3$ at 25 ℃.

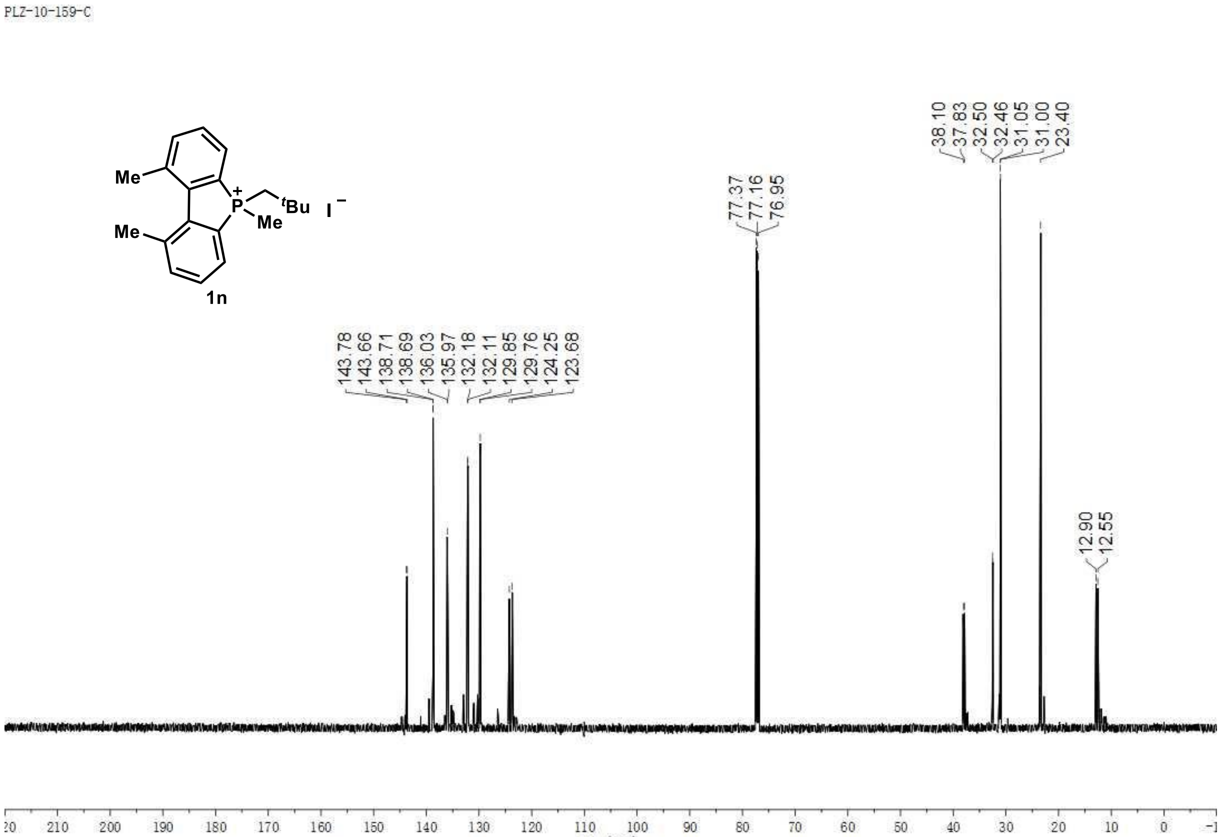

Supplementary Fig. 38.^{13}C NMR spectrum of **1n**. The sample has been recorded in 151 MHz, $CDCl_3$ at 25 ℃.

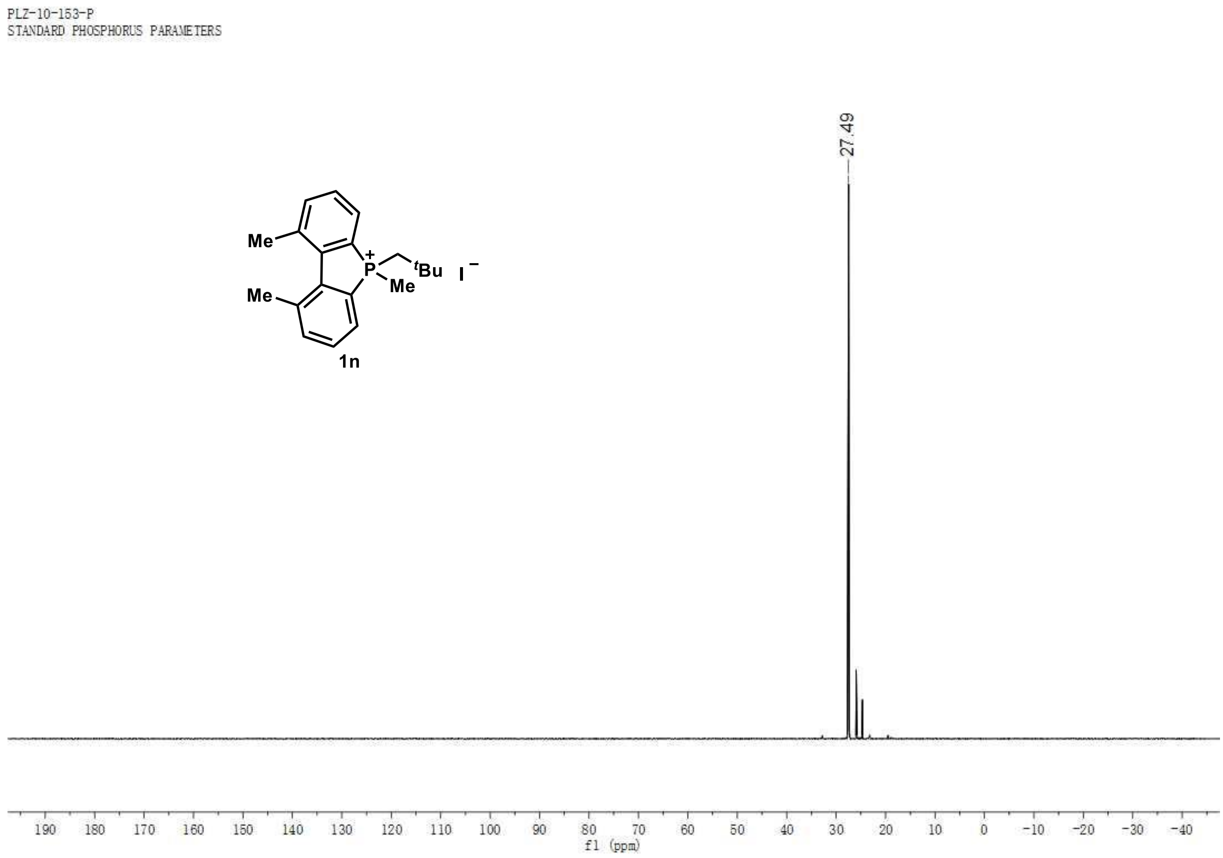

Supplementary Fig. 39.^{31}P NMR spectrum of **1n**. The sample has been recorded in 243 MHz, $CDCl_3$ at 25 ℃.

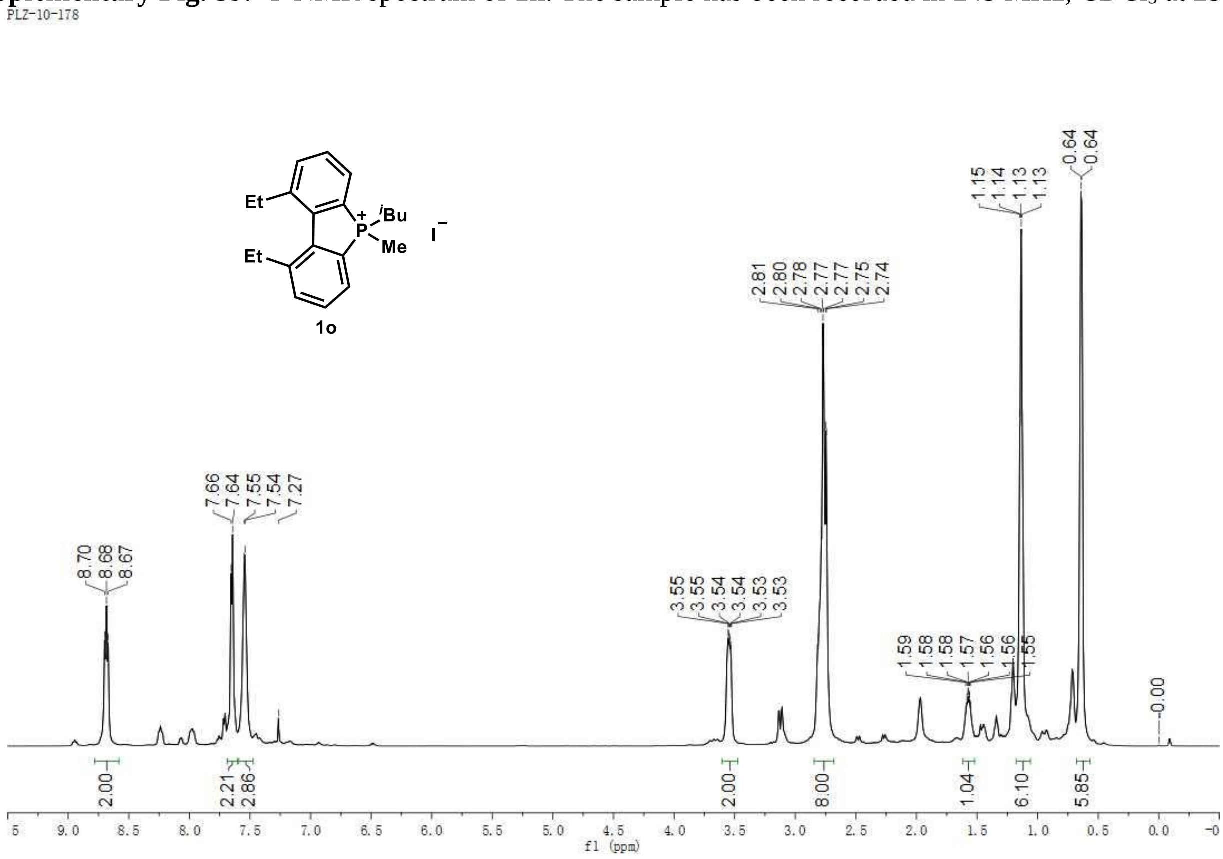

Supplementary Fig. 40. ^{1}H NMR spectrum of **1o**. The sample has been recorded in 600 MHz, $CDCl_3$ at 25 ℃.

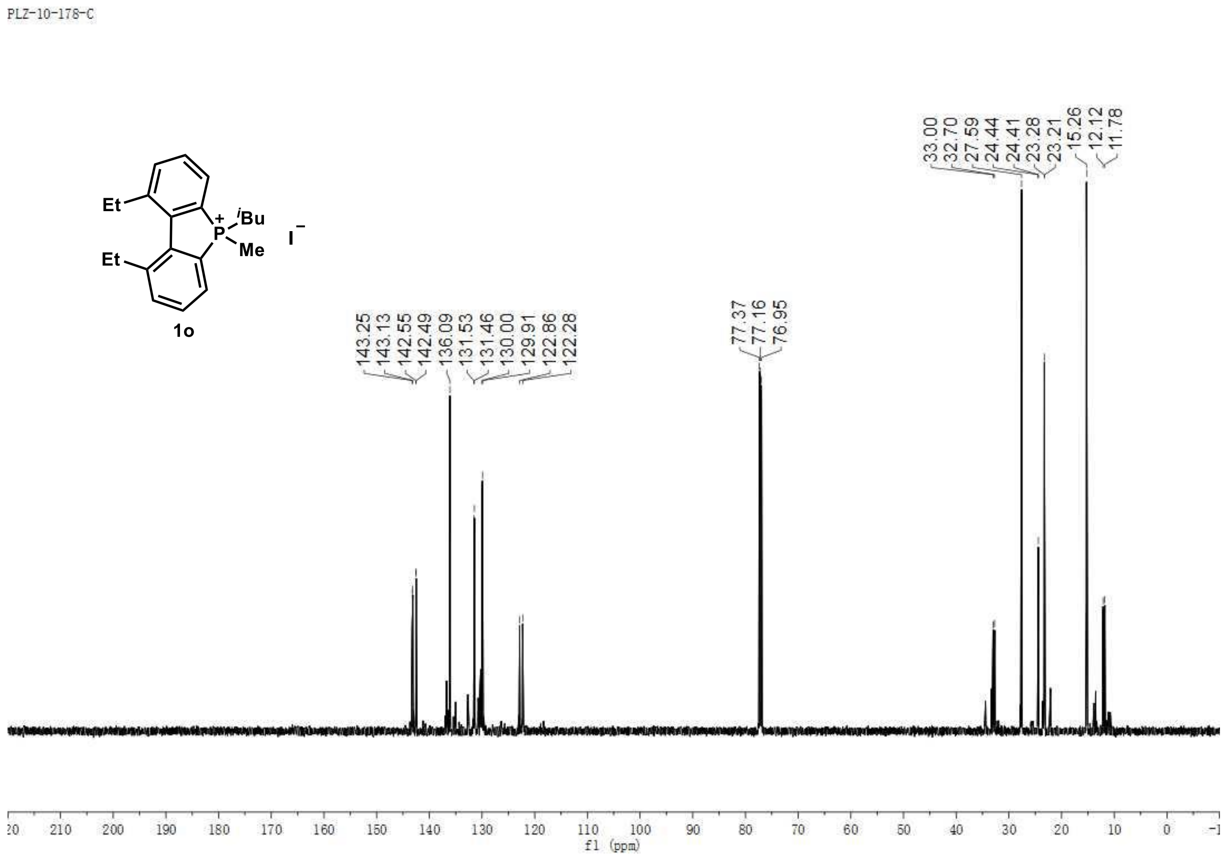

Supplementary Fig. 41. ^{13}C NMR spectrum of **1o**. The sample has been recorded in 151 MHz, $CDCl_3$ at 25 ℃.

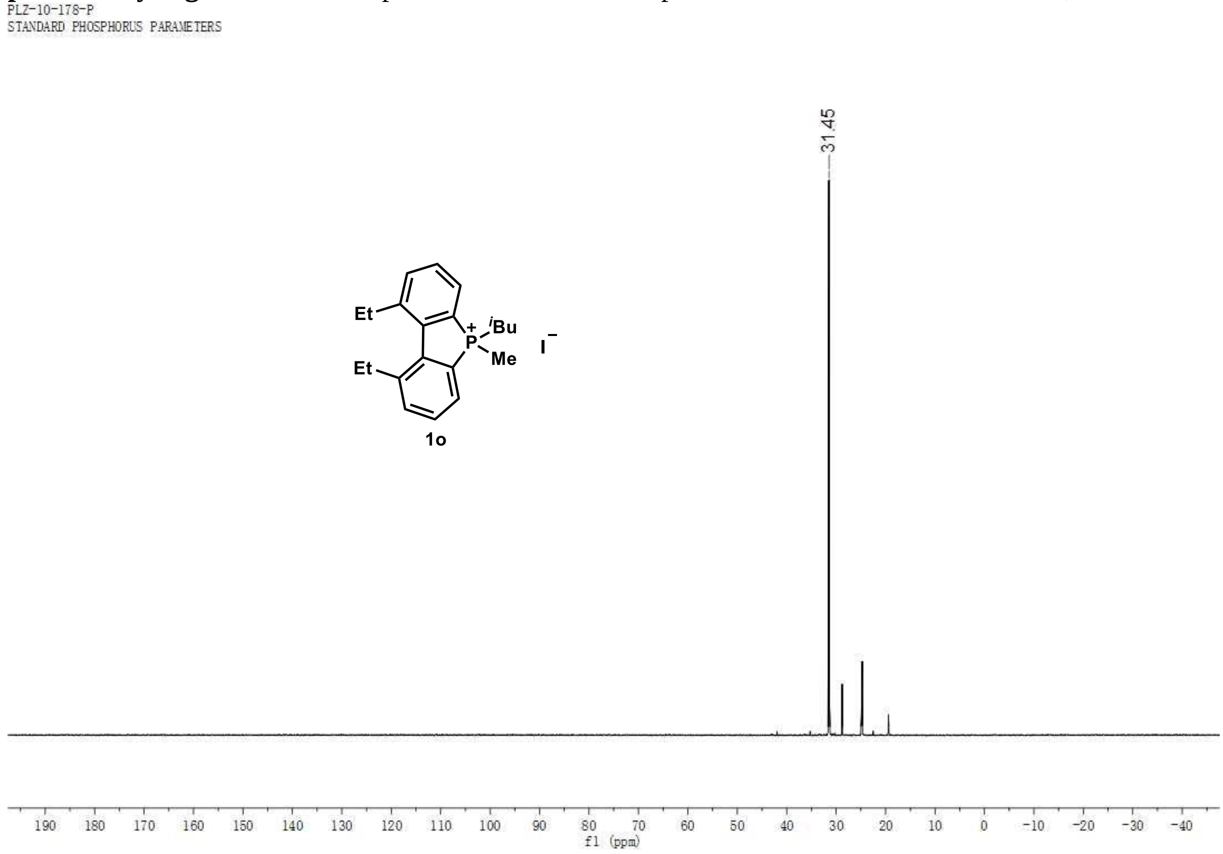

Supplementary Fig. 42. ^{31}P NMR spectrum of **1o**. The sample has been recorded in 243 MHz, $CDCl_3$ at 25 ℃.

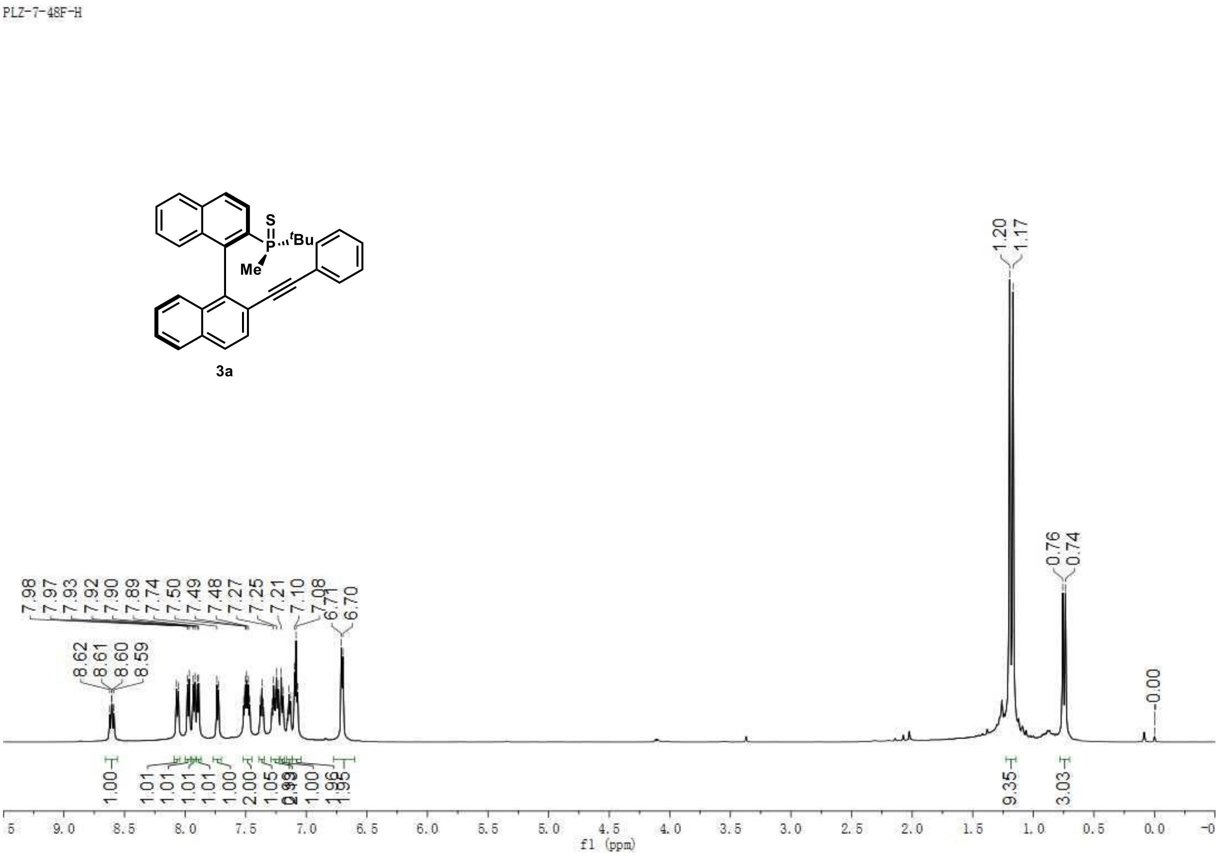

Supplementary Fig. 43. ^{1}H NMR spectrum of **3a**. The sample has been recorded in 600 MHz, $CDCl_3$ at 25 ℃.

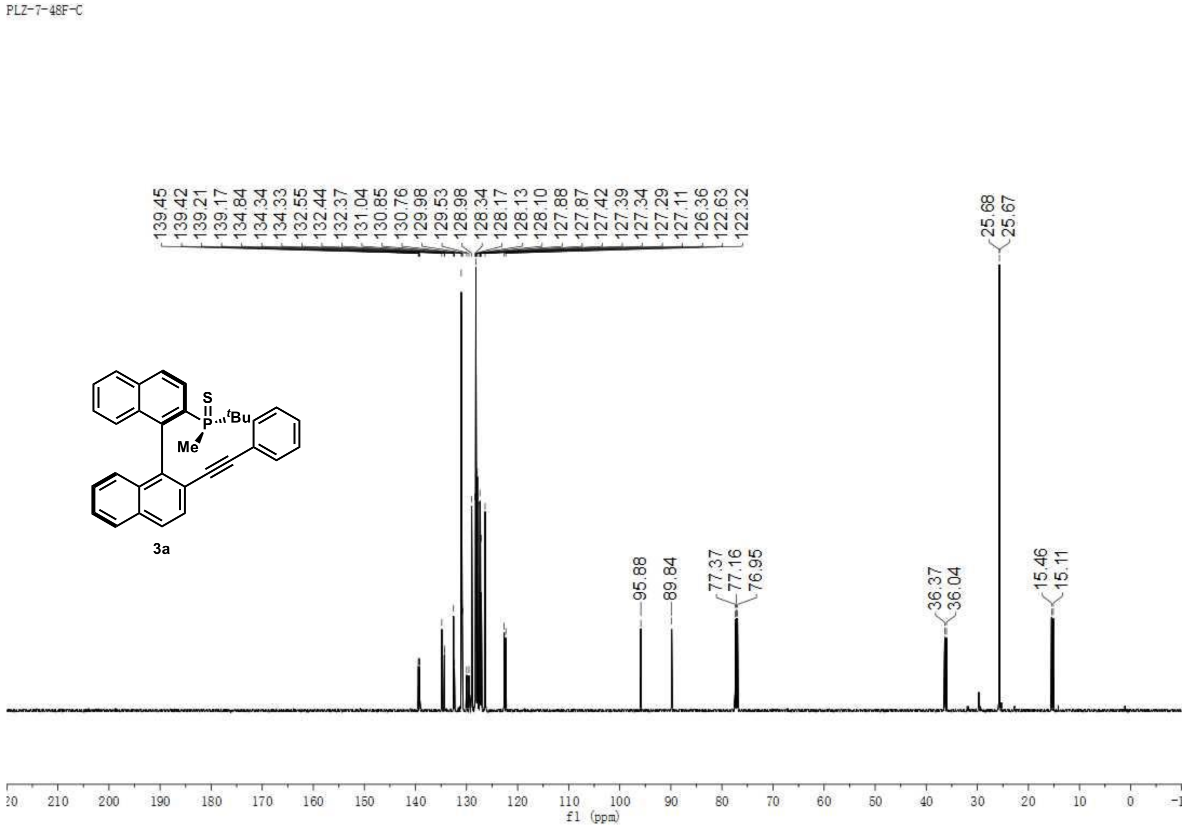

Supplementary Fig. 44.^{13}C NMR spectrum of **3a**. The sample has been recorded in 151 MHz, $CDCl_3$ at 25 ℃.

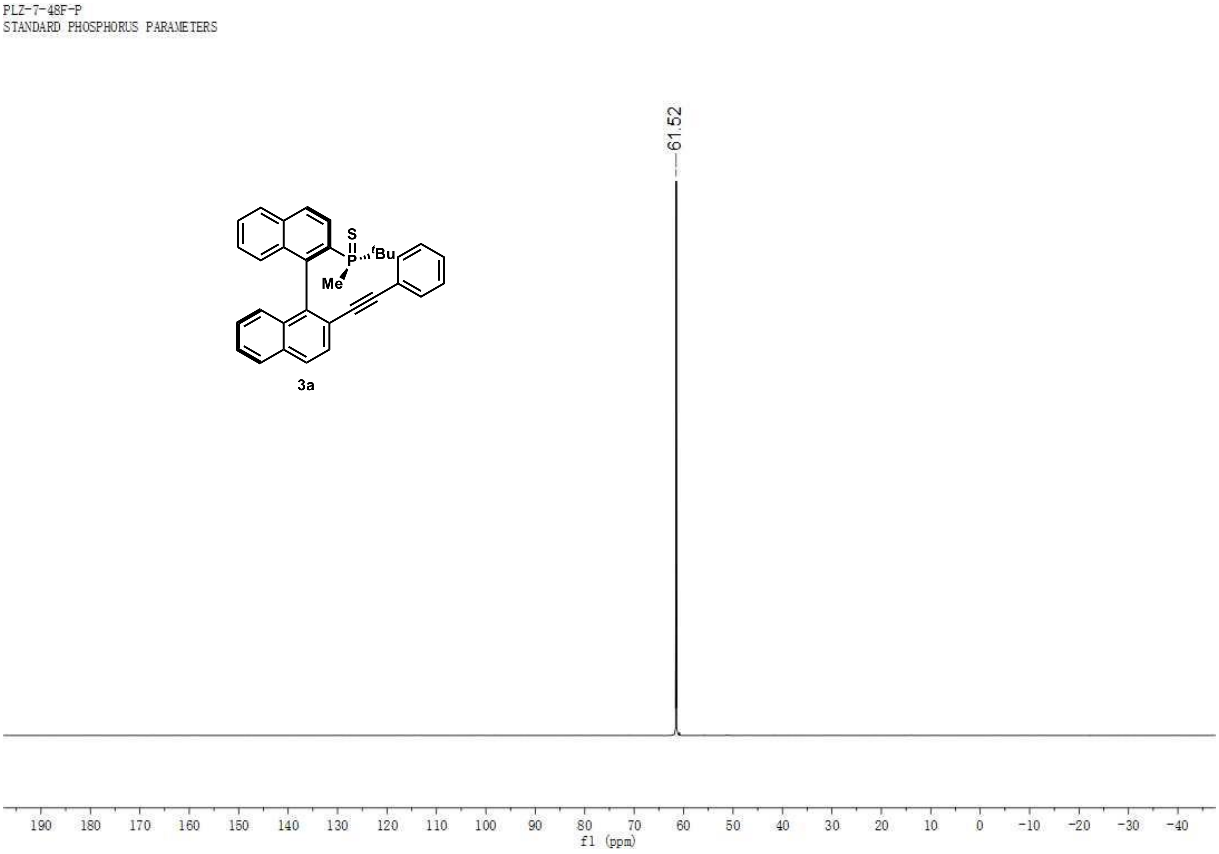

Supplementary Fig. 45.^{31}P NMR spectrum of **3a**. The sample has been recorded in 243 MHz, $CDCl_3$ at 25 ℃.

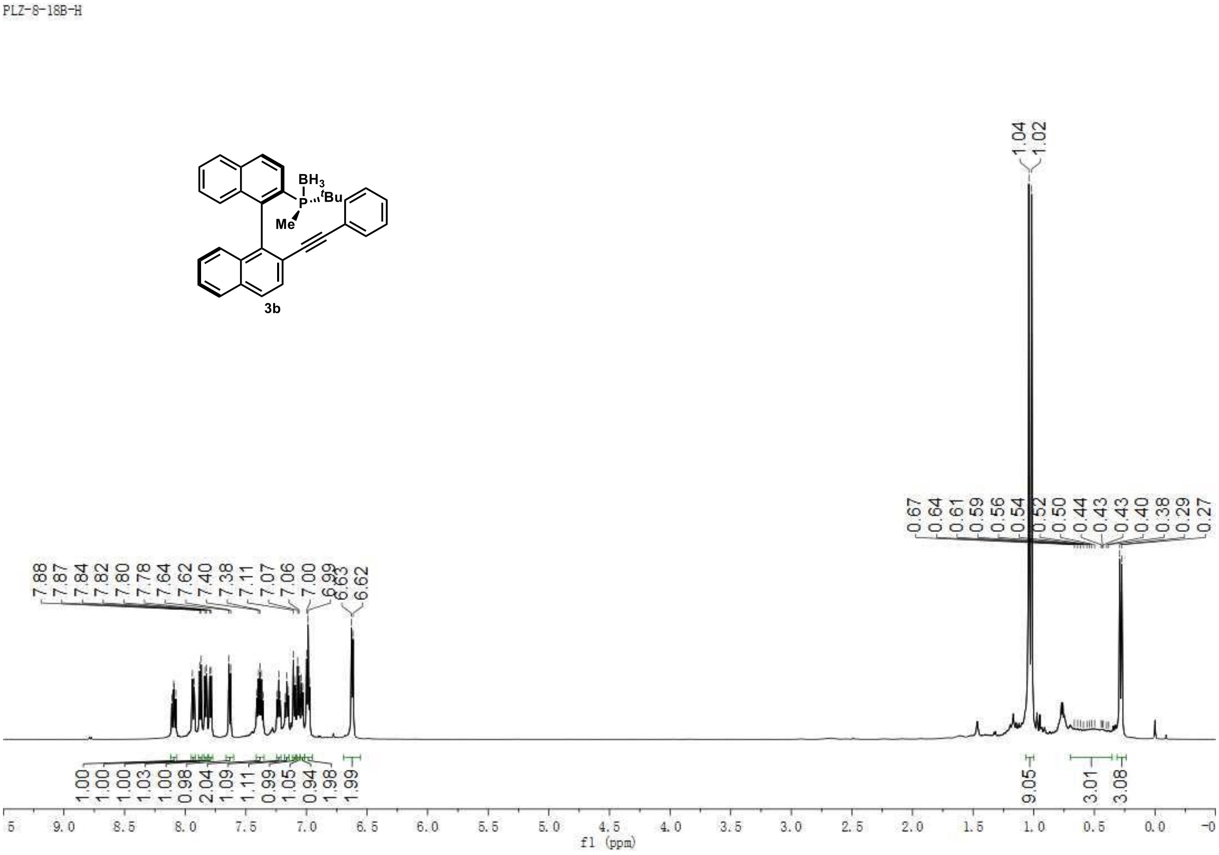

Supplementary Fig. 46. ^{1}H NMR spectrum of **3b**. The sample has been recorded in 600 MHz, $CDCl_3$ at 25 ℃.

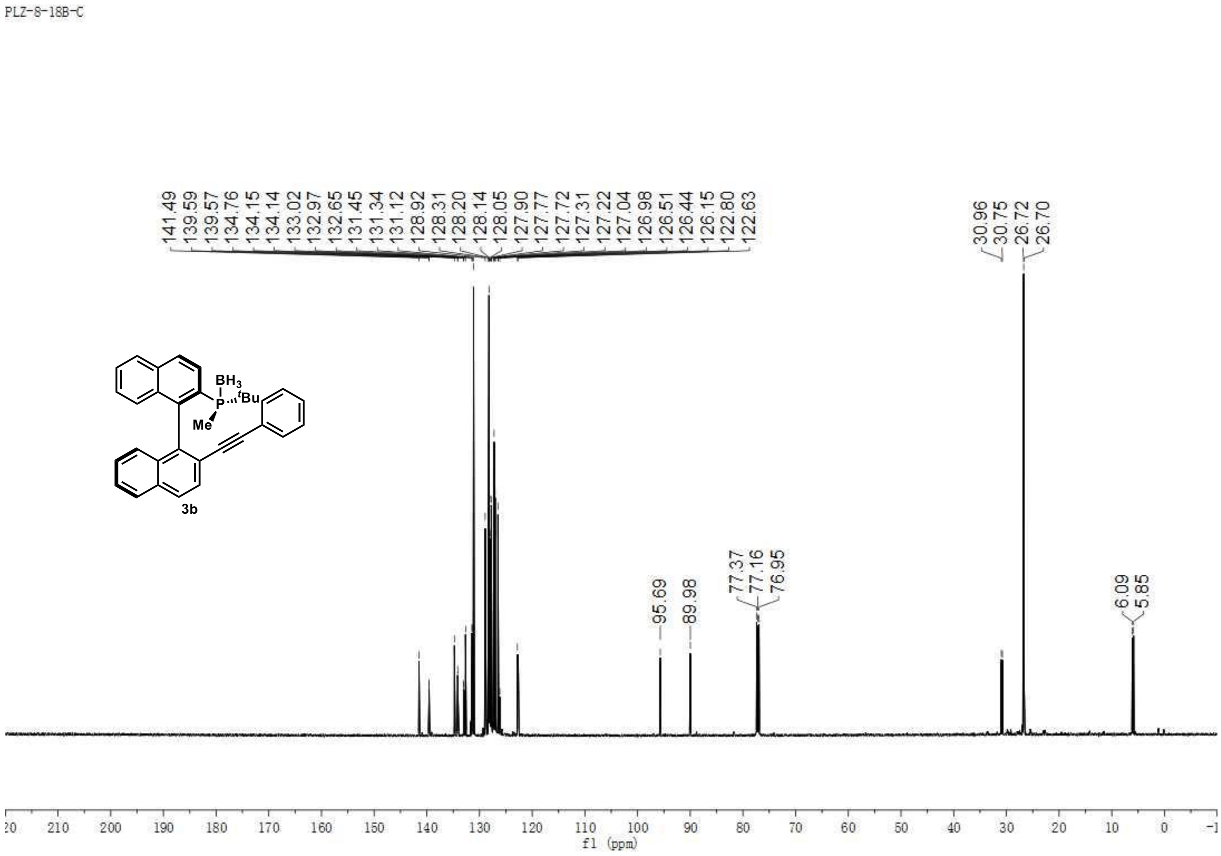

Supplementary Fig. 47.^{13}C NMR spectrum of **3b**. The sample has been recorded in 151 MHz, $CDCl_3$ at 25 ℃.

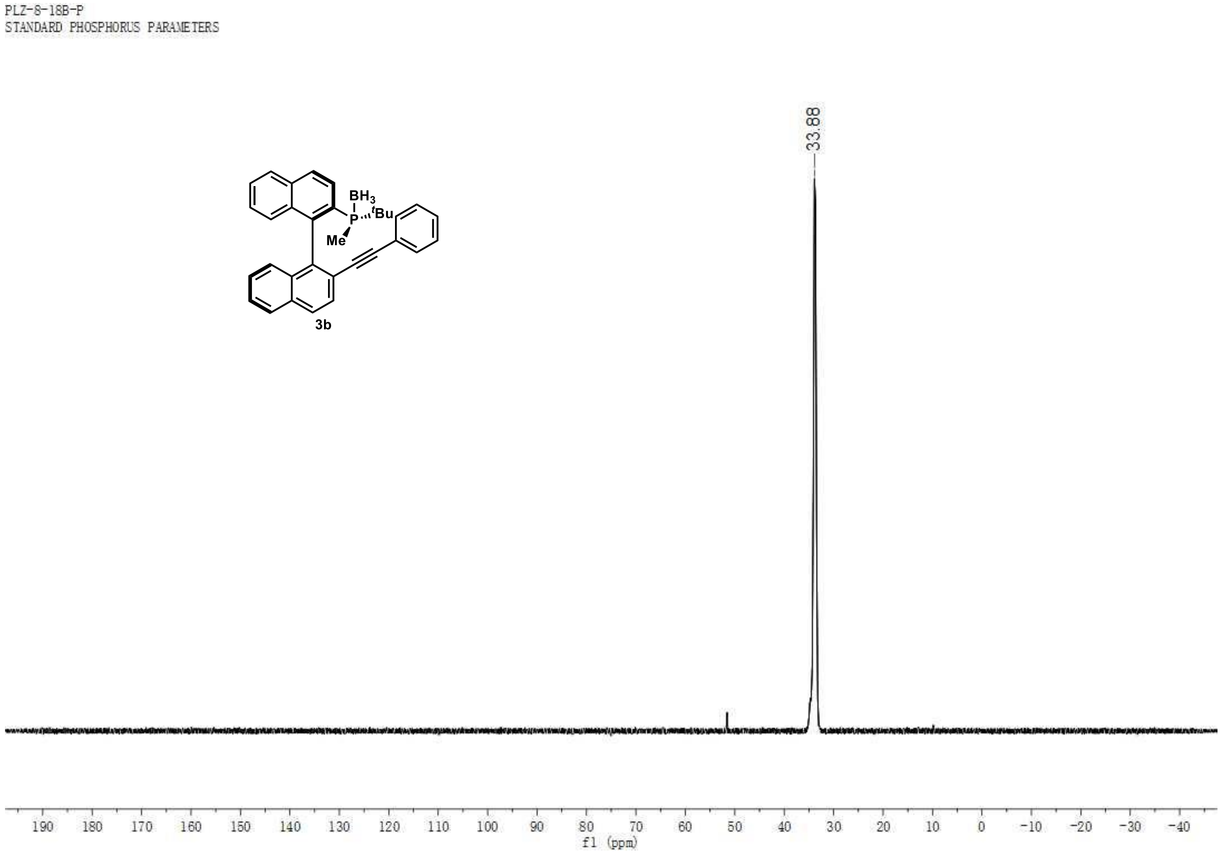

Supplementary Fig. 48.^{31}P NMR spectrum of **3b**. The sample has been recorded in 243 MHz, $CDCl_3$ at 25 ℃.

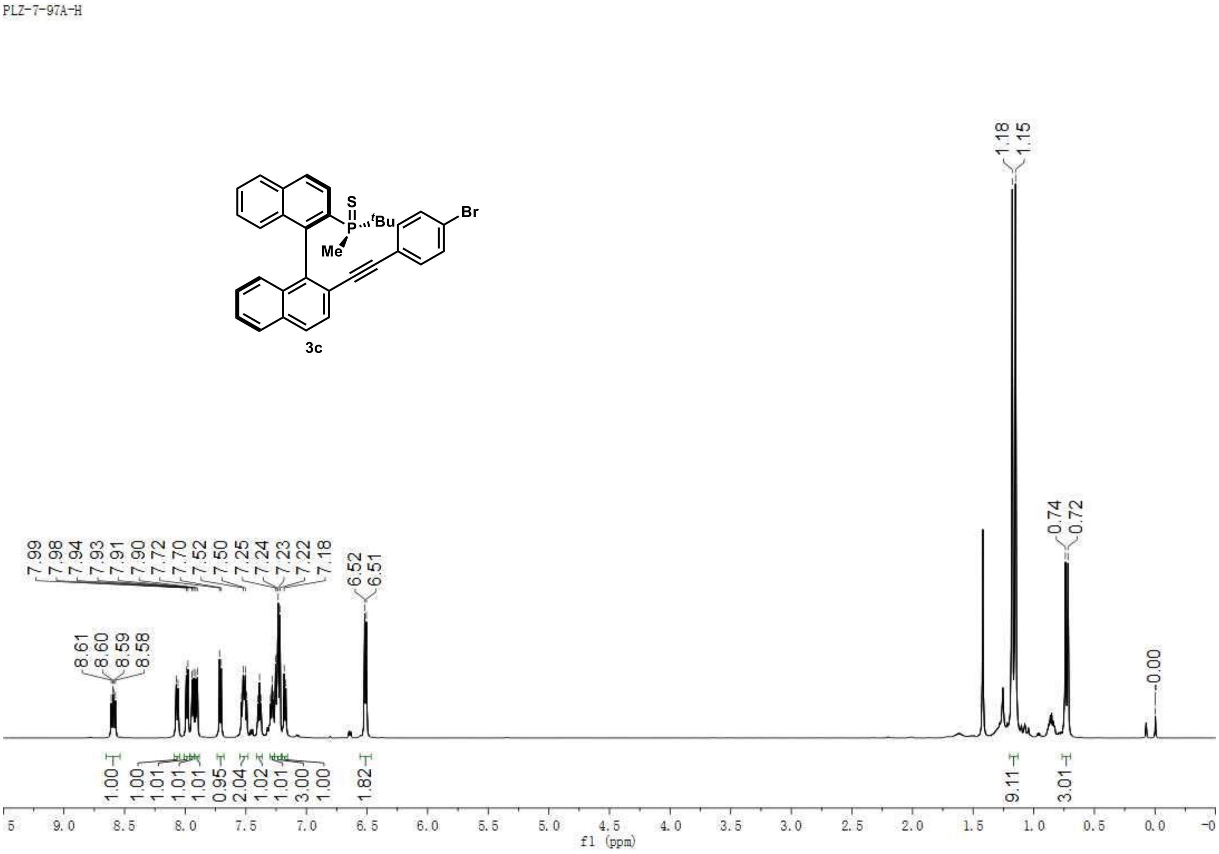

Supplementary Fig. 49. ^{1}H NMR spectrum of **3c**. The sample has been recorded in 600 MHz, $CDCl_3$ at 25 ℃.

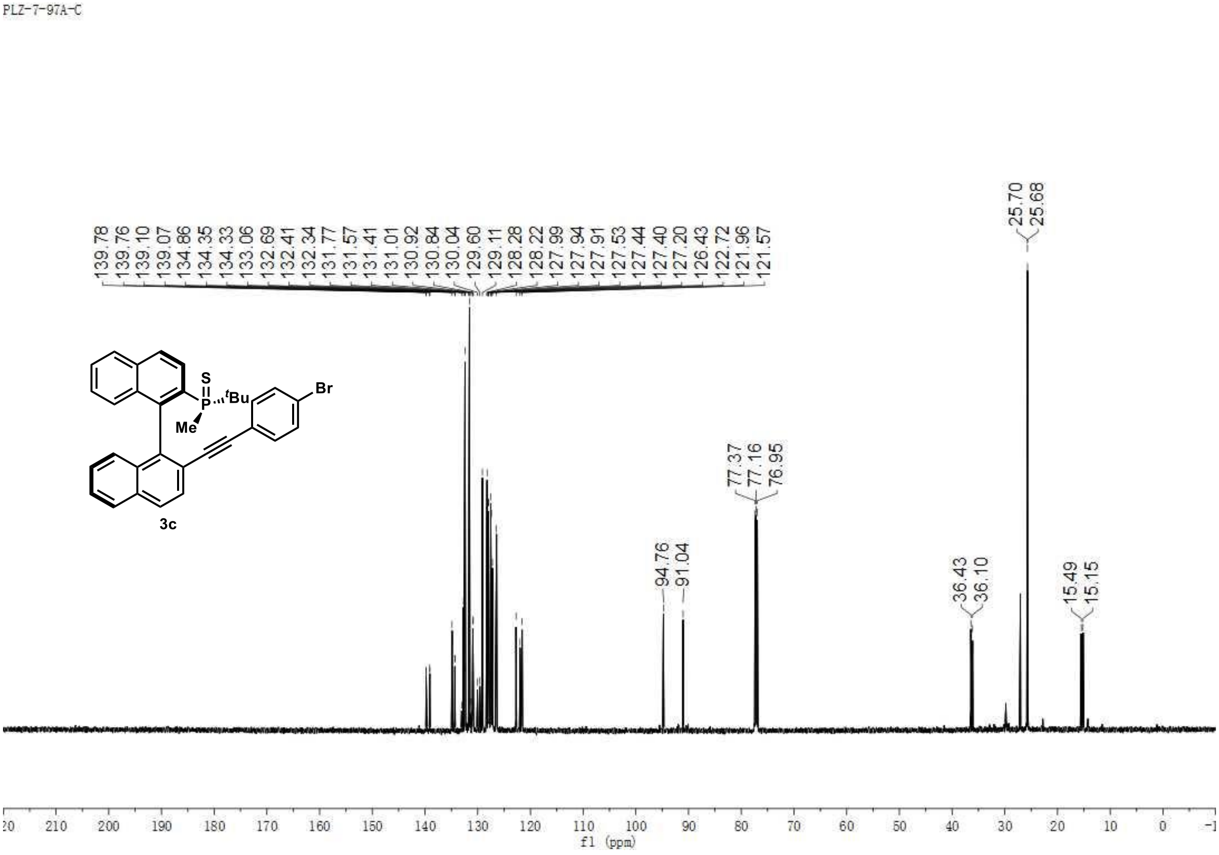

Supplementary Fig. 50.^{13}C NMR spectrum of **3c**. The sample has been recorded in 151 MHz, $CDCl_3$ at 25 ℃.

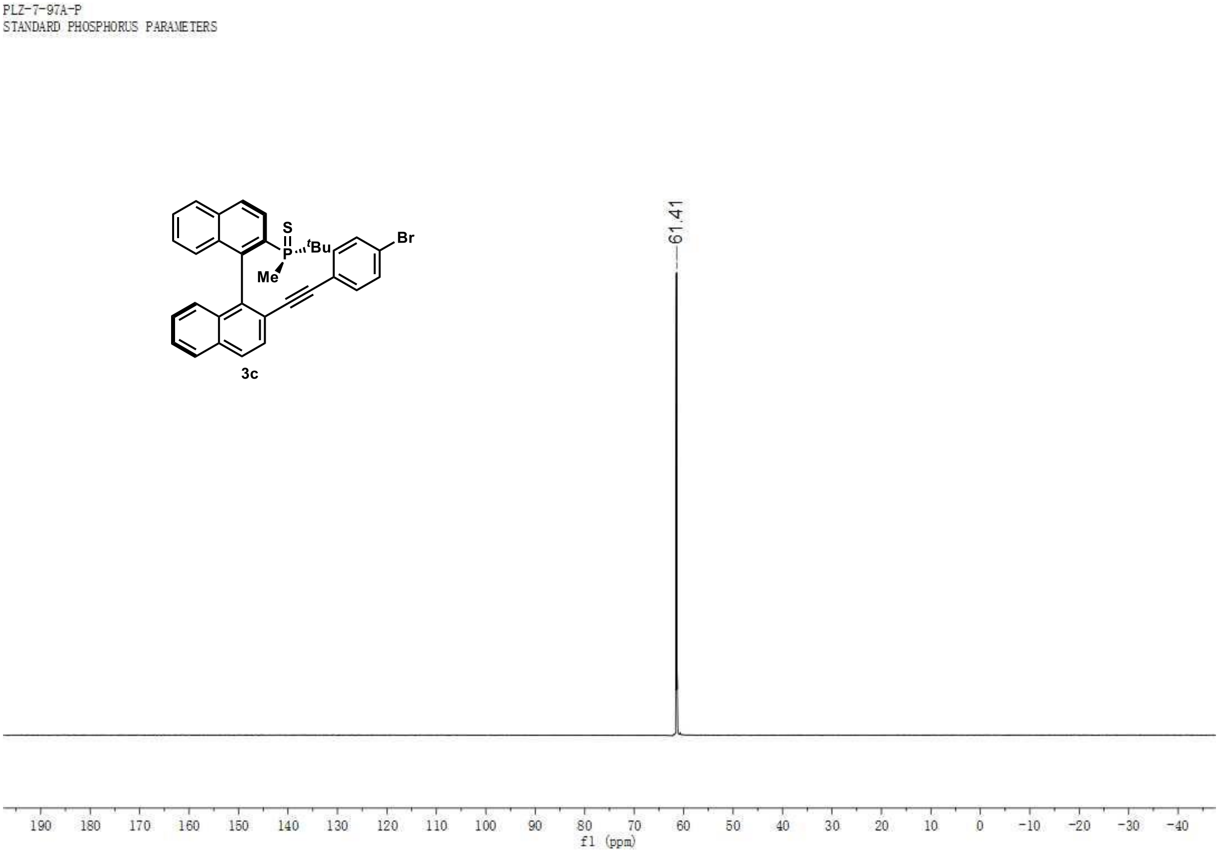

Supplementary Fig. 51.^{31}P NMR spectrum of **3c**. The sample has been recorded in 243 MHz, $CDCl_3$ at 25 ℃.

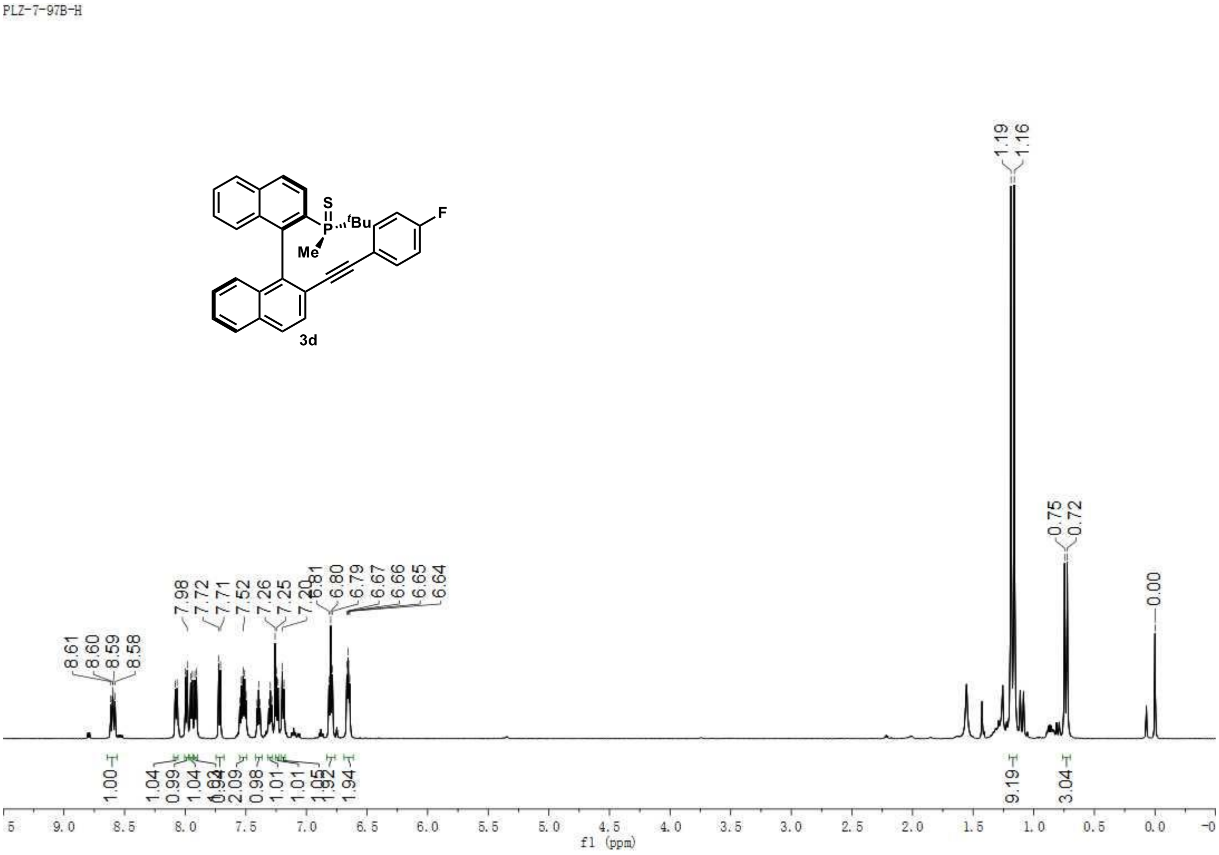

Supplementary Fig. 52. ^{1}H NMR spectrum of **3d**. The sample has been recorded in 600 MHz, $CDCl_3$ at 25 ℃.

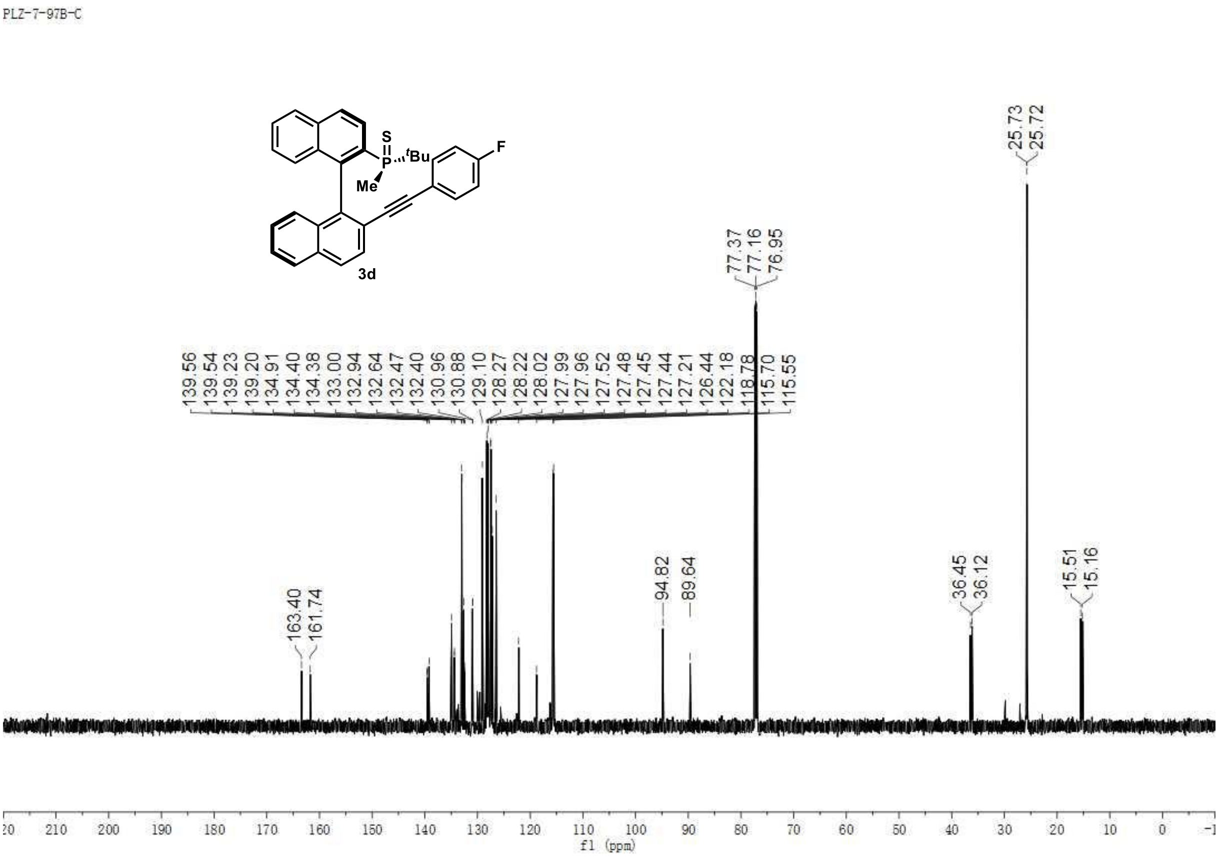

Supplementary Fig. 53.^{13}C NMR spectrum of **3d**. The sample has been recorded in 151 MHz, $CDCl_3$ at 25 ℃.

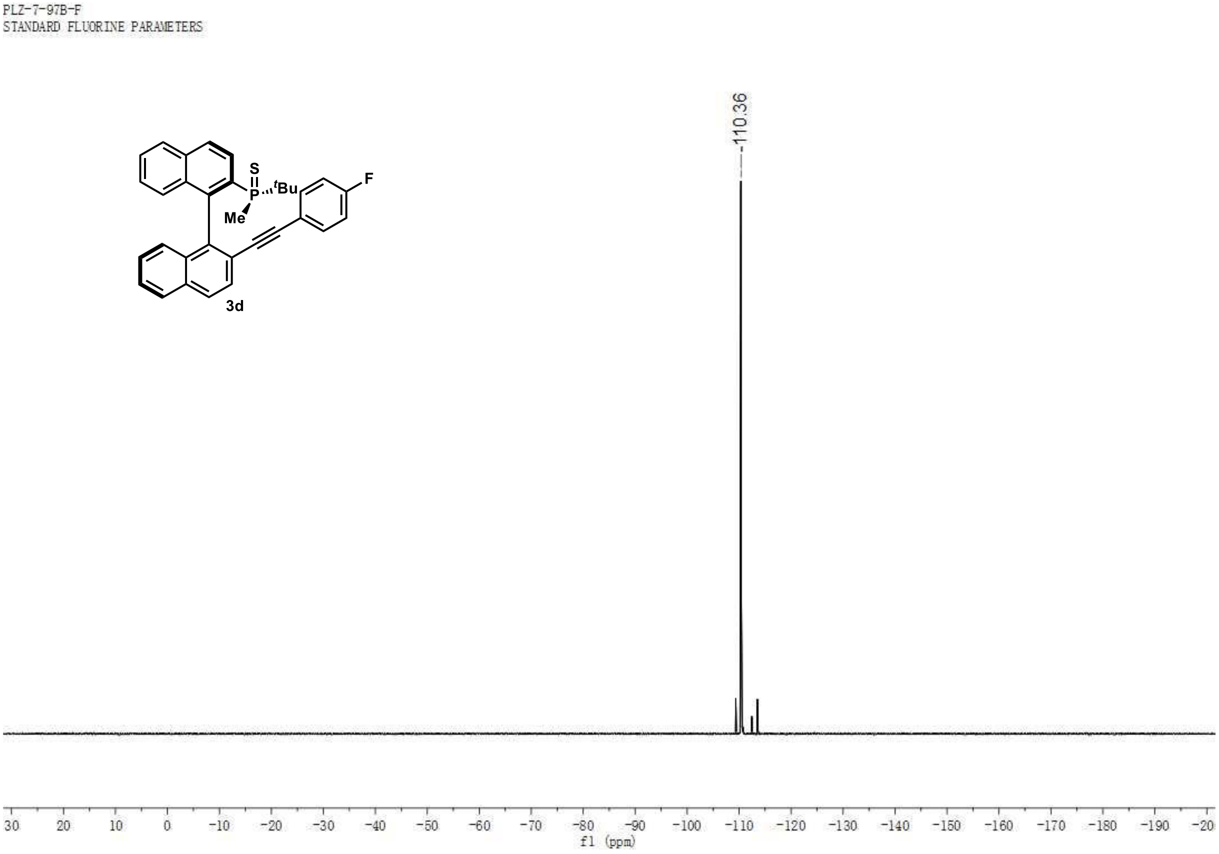

Supplementary Fig. 54.^{19}F NMR spectrum of **3d**. The sample has been recorded in 564 MHz, $CDCl_3$ at 25 ℃.

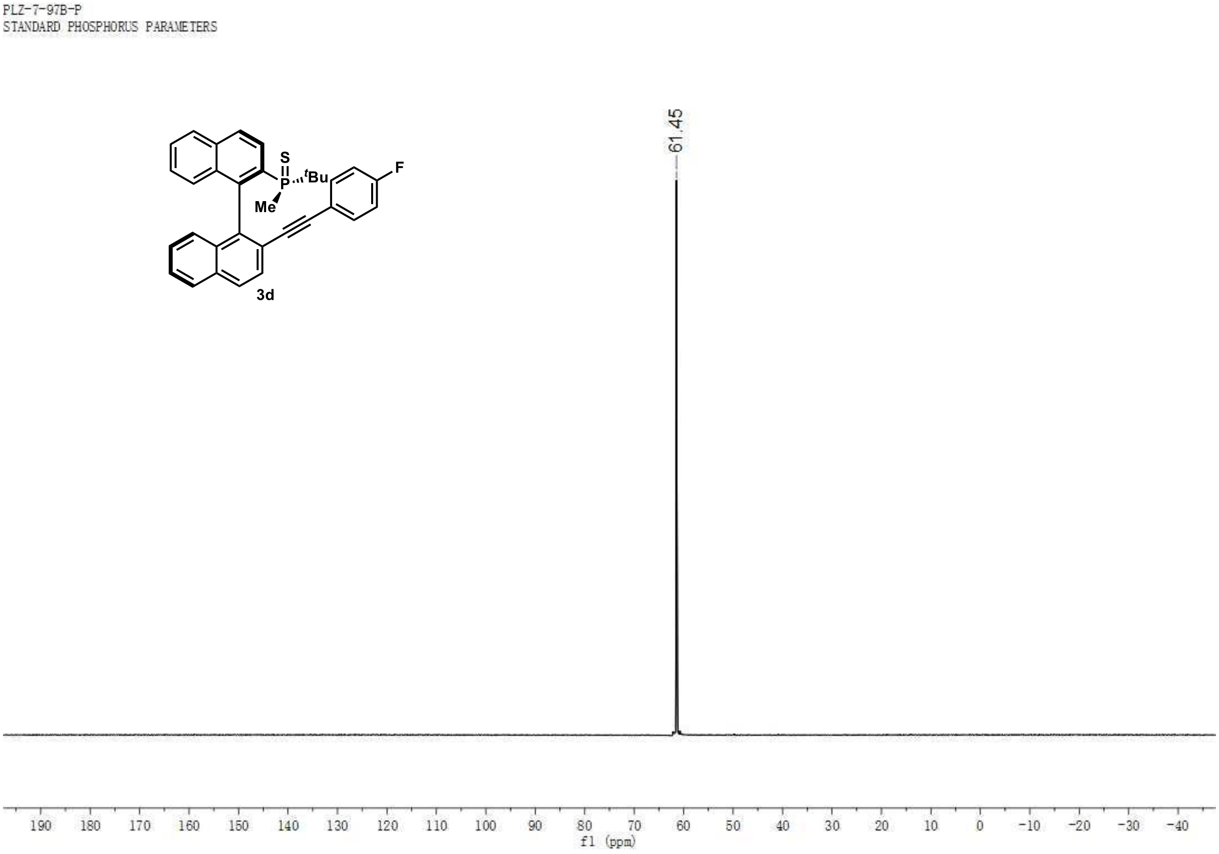

Supplementary Fig. 55.^{31}P NMR spectrum of **3d**. The sample has been recorded in 243 MHz, $CDCl_3$ at 25 ℃.

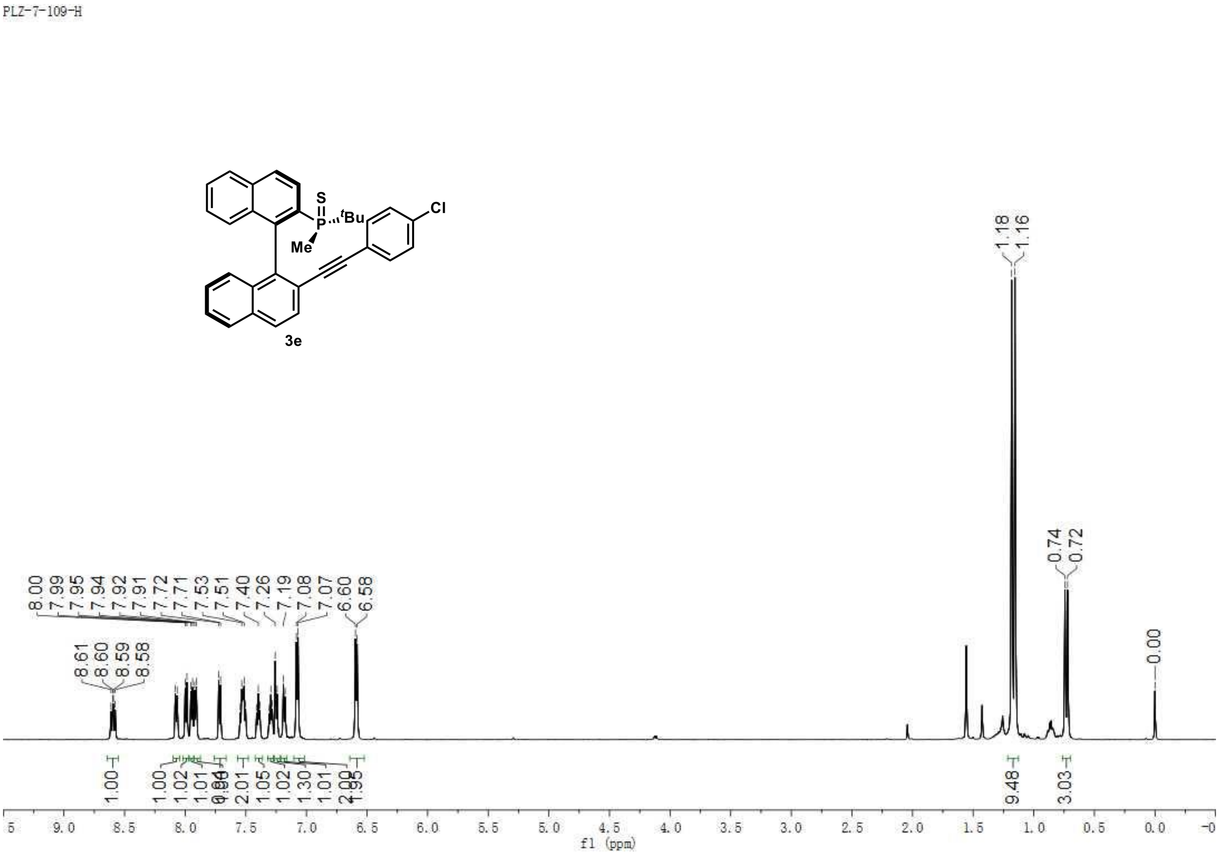

Supplementary Fig. 56. ^{1}H NMR spectrum of **3e**. The sample has been recorded in 600 MHz, $CDCl_3$ at 25 ℃.

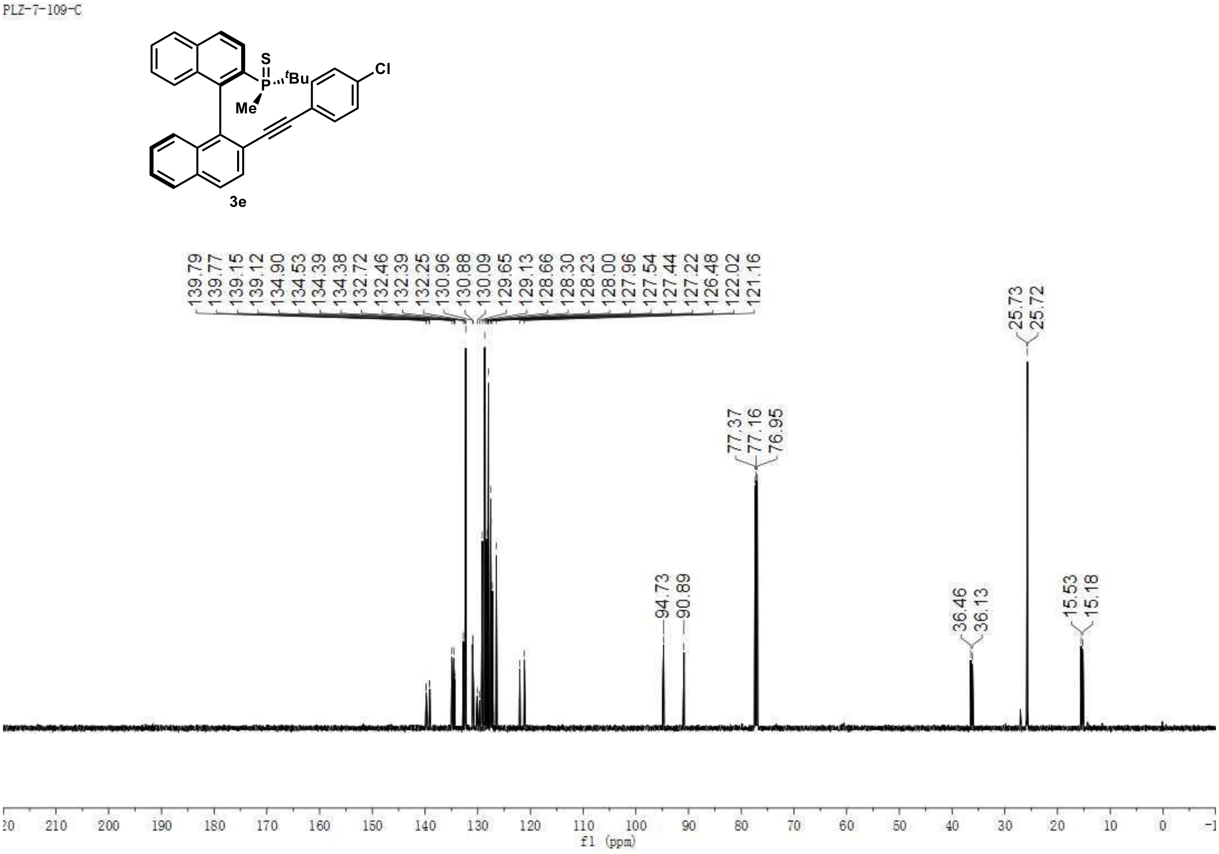

Supplementary Fig. 57.^{13}C NMR spectrum of **3e**. The sample has been recorded in 151 MHz, $CDCl_3$ at 25 ℃.

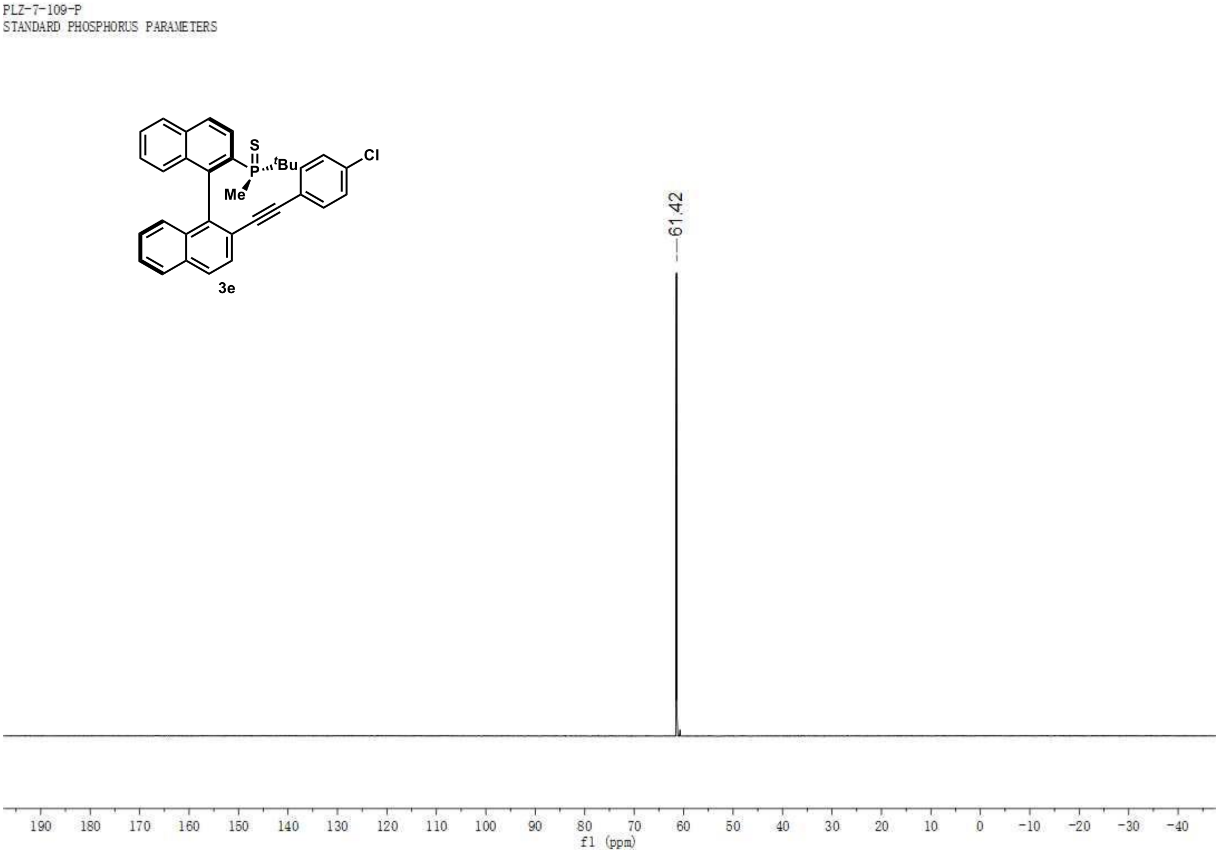

Supplementary Fig. 58.^{31}P NMR spectrum of **3e**. The sample has been recorded in 243 MHz, $CDCl_3$ at 25 ℃.

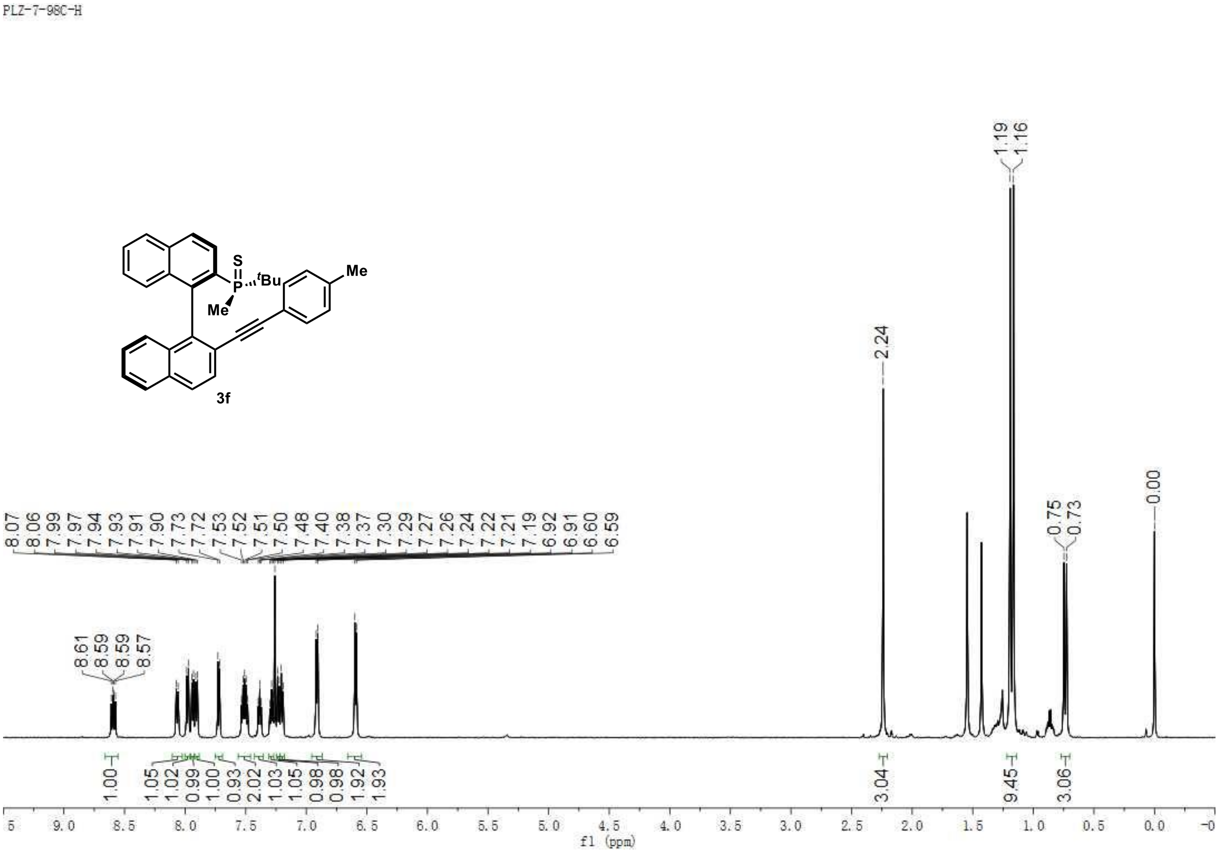

Supplementary Fig. 59. ^{1}H NMR spectrum of **3f**. The sample has been recorded in 600 MHz, $CDCl_3$ at 25 ℃.

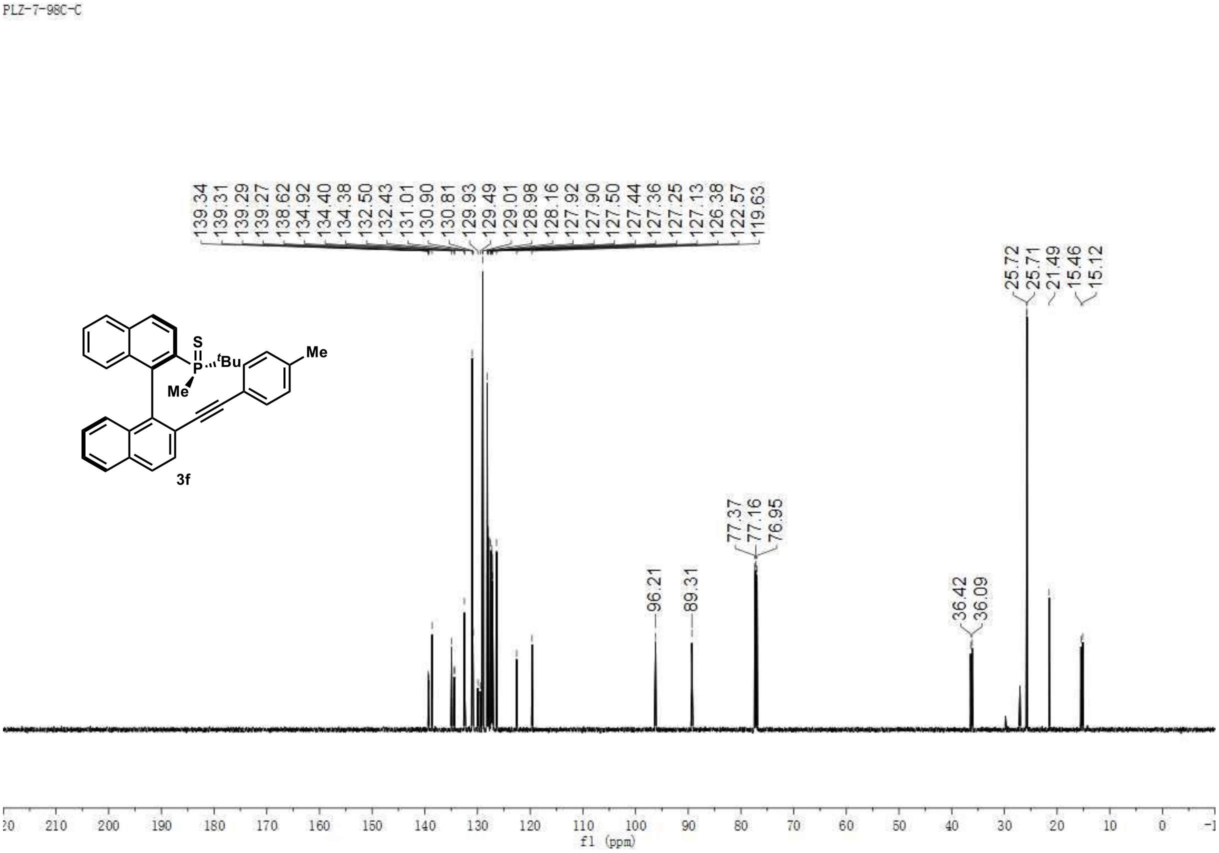

Supplementary Fig. 60.^{13}C NMR spectrum of **3f**. The sample has been recorded in 151 MHz, $CDCl_3$ at 25 ℃.

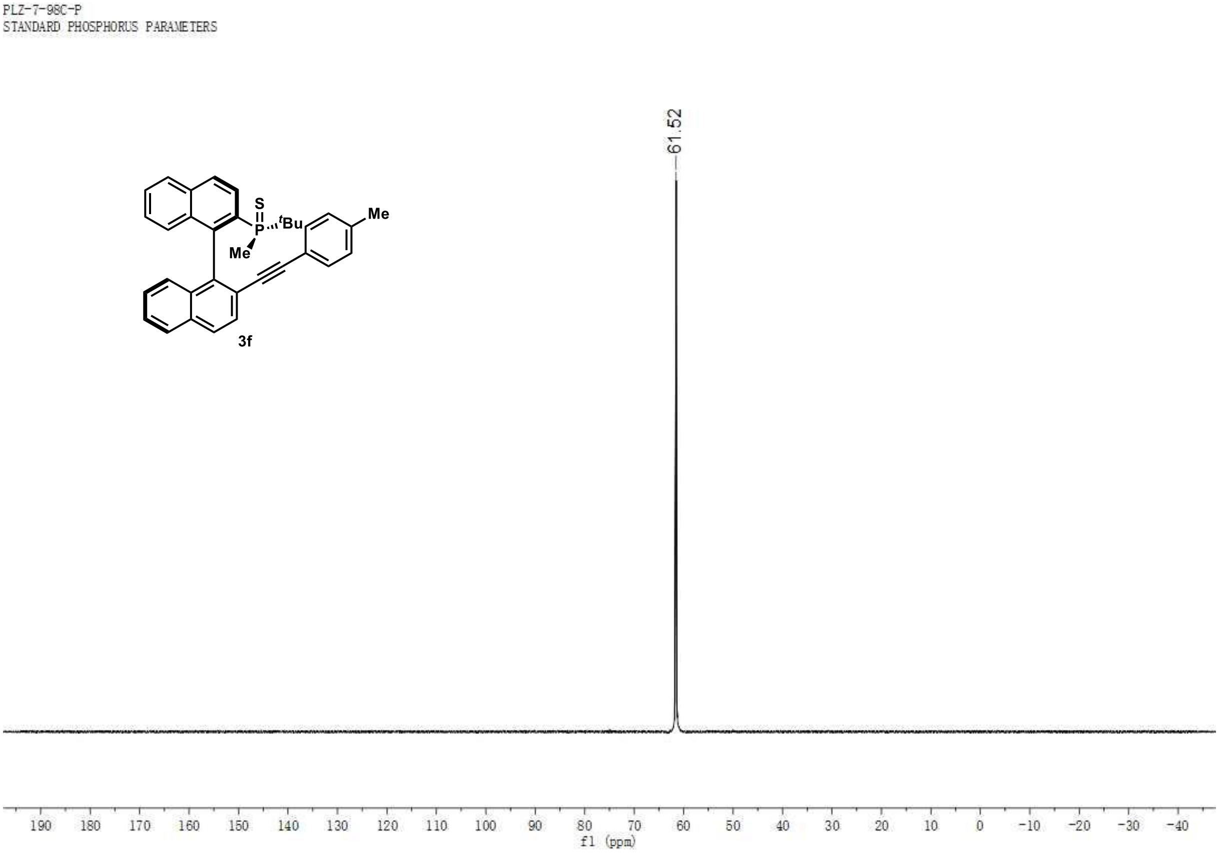

Supplementary Fig. 61.^{31}P NMR spectrum of **3f**. The sample has been recorded in 243 MHz, $CDCl_3$ at 25 ℃.

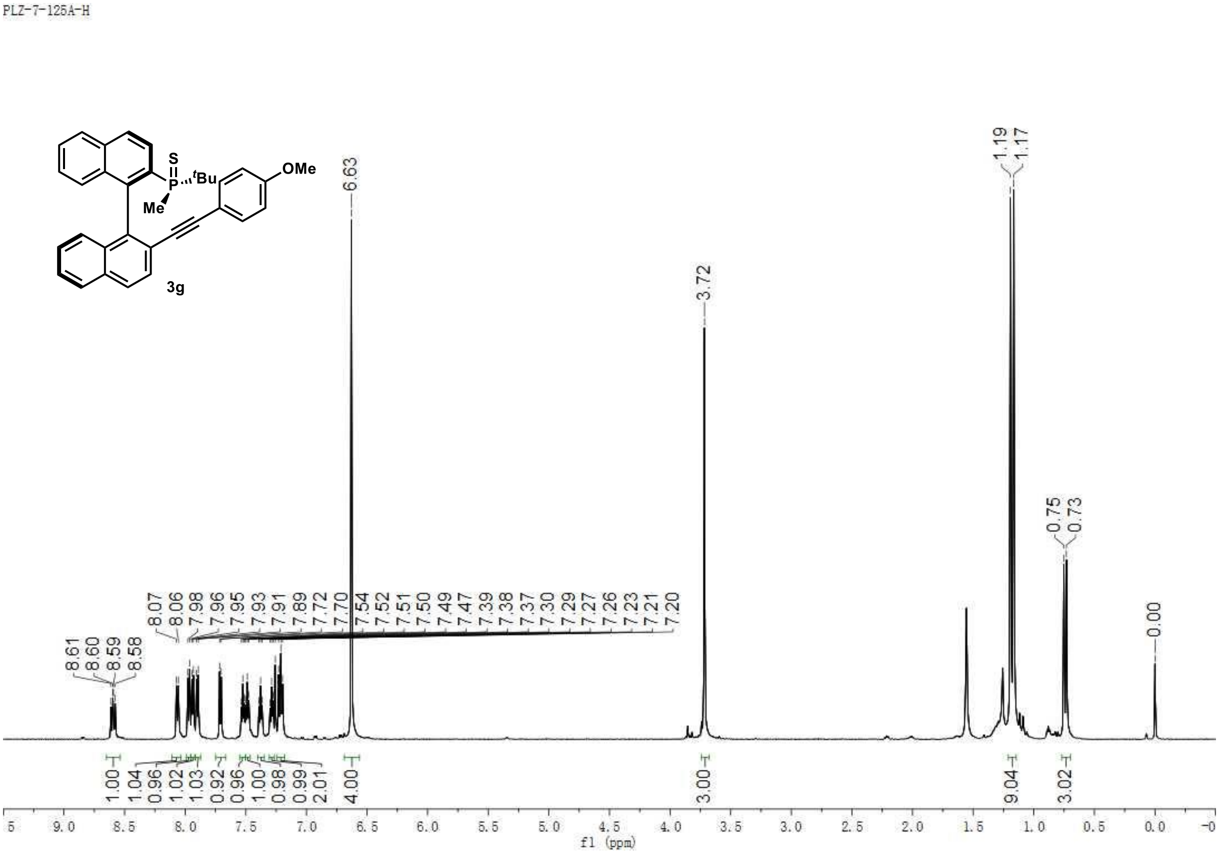

Supplementary Fig. 62. ^{1}H NMR spectrum of **3g**. The sample has been recorded in 600 MHz, $CDCl_3$ at 25 ℃.

PLZ-7-98E-C

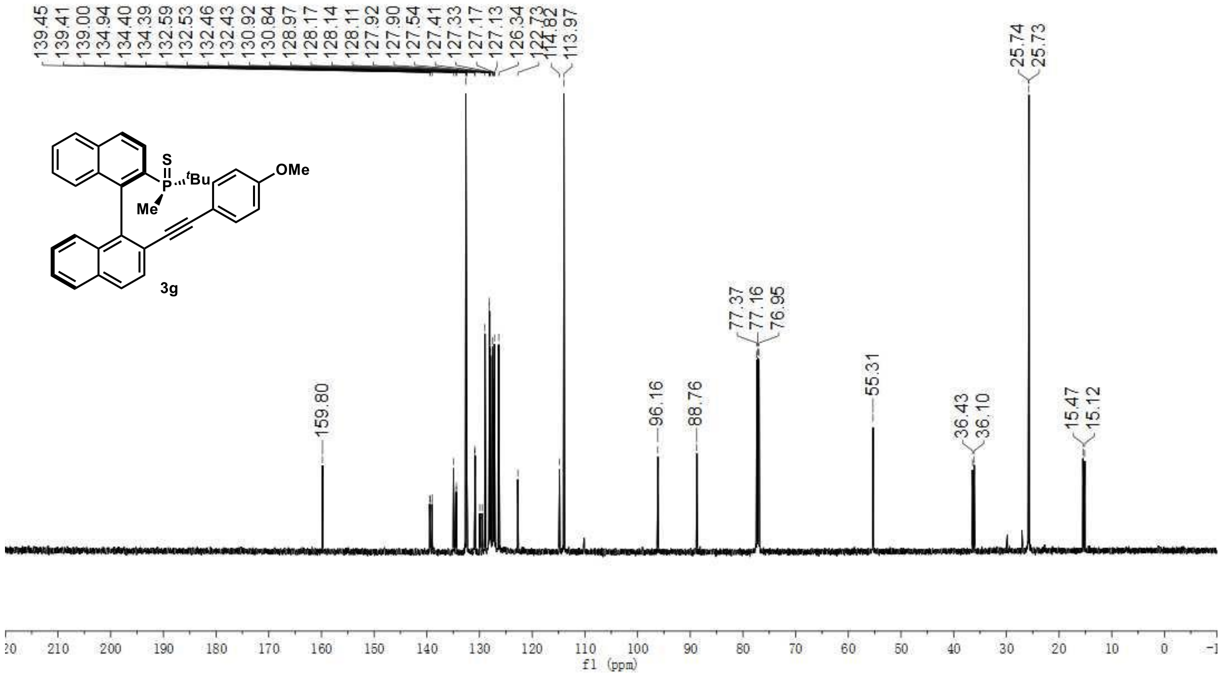

Supplementary Fig. 63.^{13}C NMR spectrum of **3g**. The sample has been recorded in 151 MHz, $CDCl_3$ at 25 ℃.

PLZ-7-98E-P
STANDARD PHOSPHORUS PARAMETERS

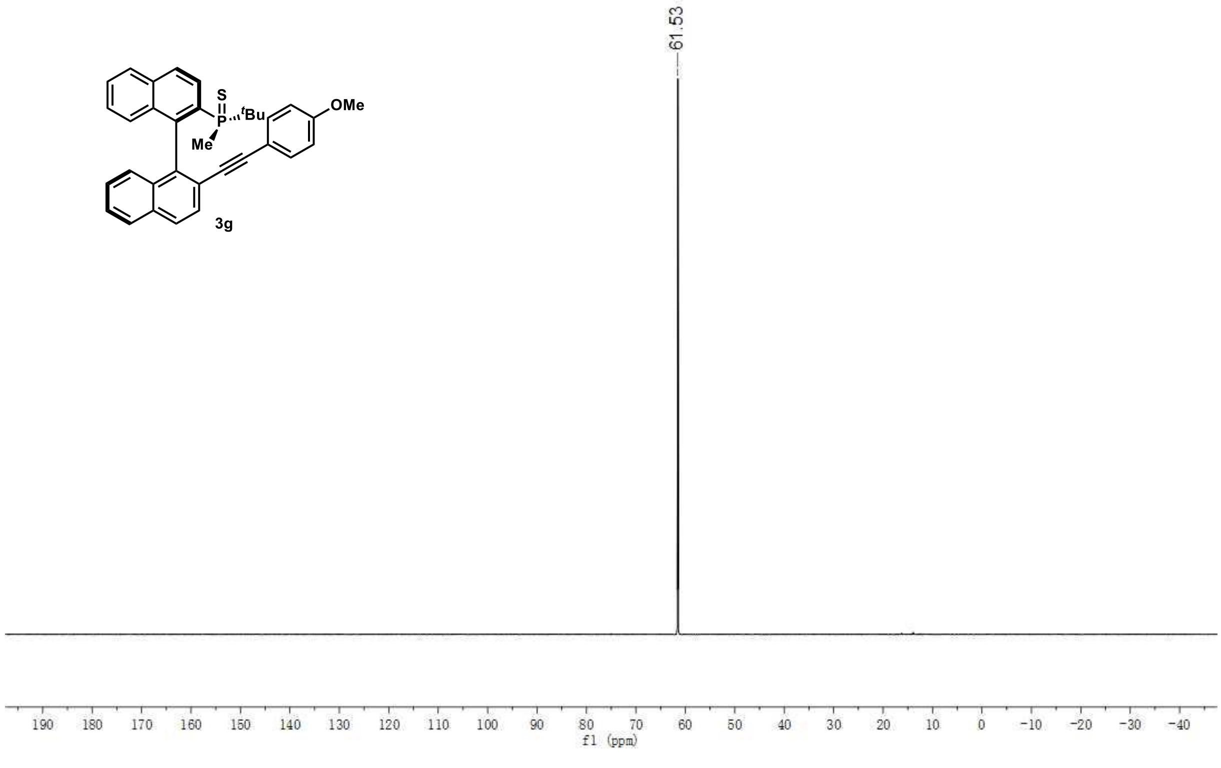

Supplementary Fig. 64.^{31}P NMR spectrum of **3g**. The sample has been recorded in 243 MHz, $CDCl_3$ at 25 ℃.

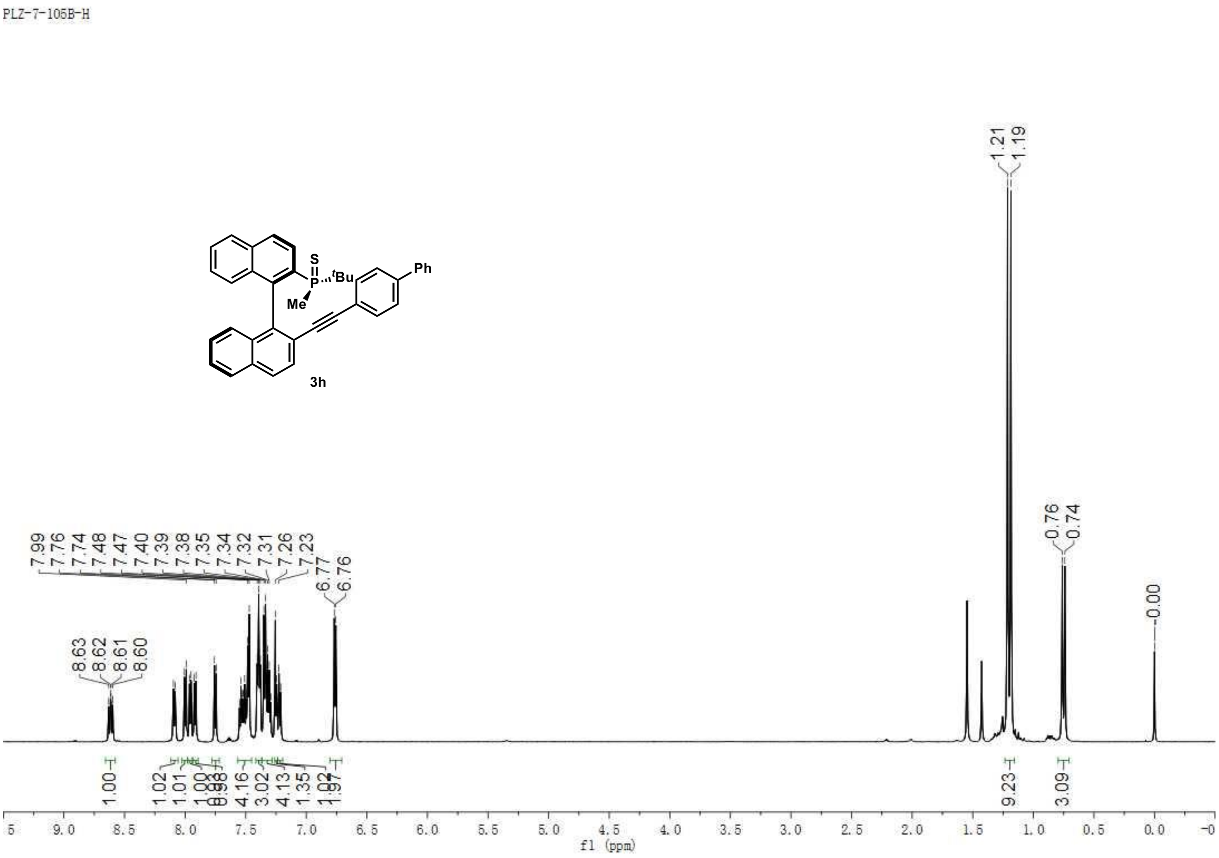

Supplementary Fig. 65. ^{1}H NMR spectrum of **3h**. The sample has been recorded in 600 MHz, $CDCl_3$ at 25 ℃.

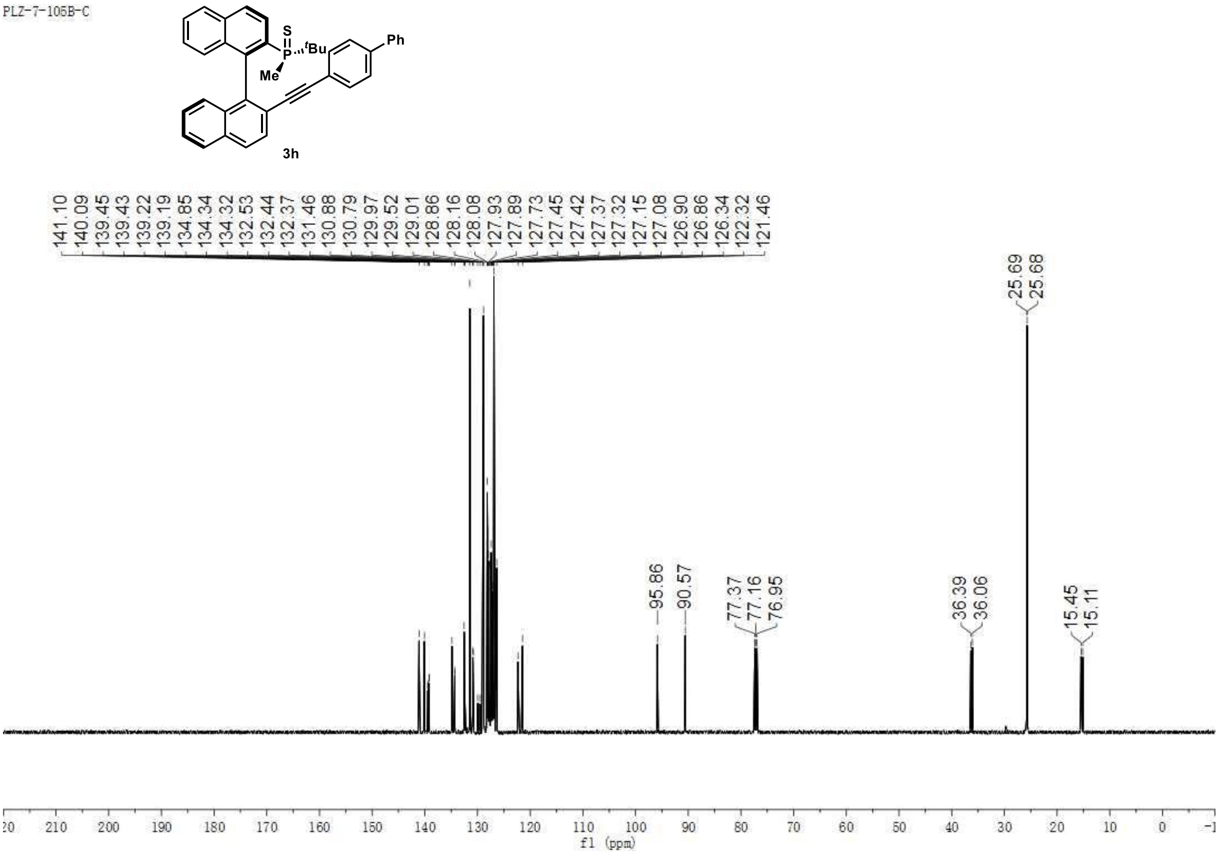

Supplementary Fig. 66.^{13}C NMR spectrum of **3h**. The sample has been recorded in 151 MHz, $CDCl_3$ at 25 ℃.

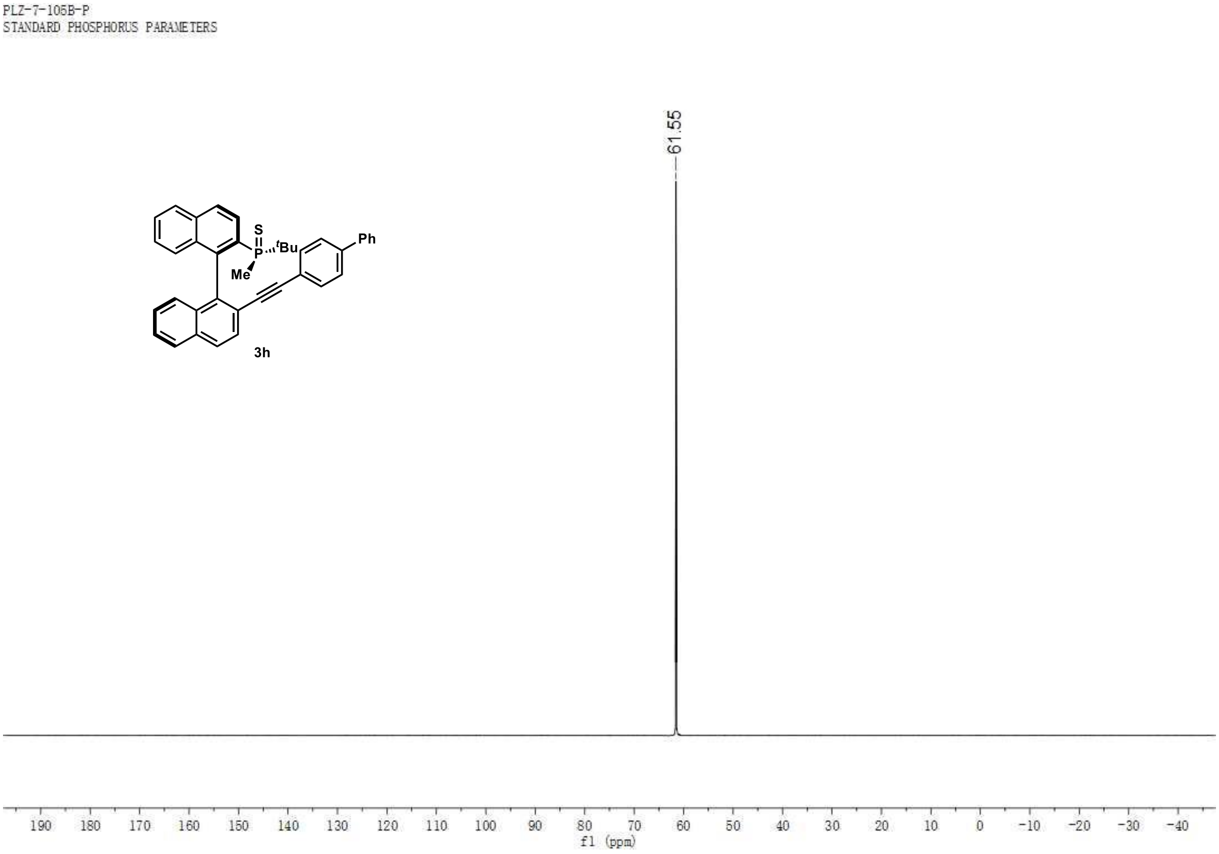

Supplementary Fig. 67.^{31}P NMR spectrum of **3h**. The sample has been recorded in 243 MHz, $CDCl_3$ at 25 ℃.

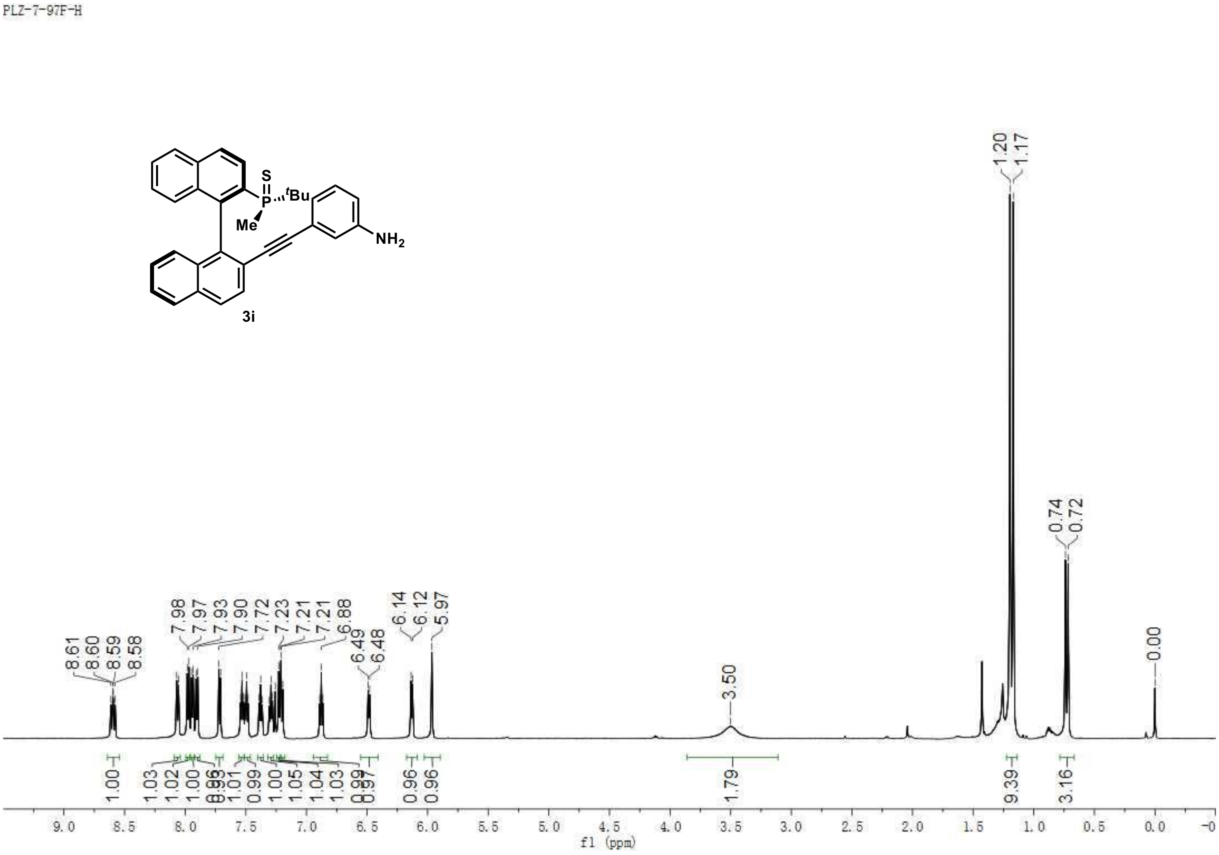

Supplementary Fig. 68. ^{1}H NMR spectrum of **3i**. The sample has been recorded in 600 MHz, $CDCl_3$ at 25 ℃.

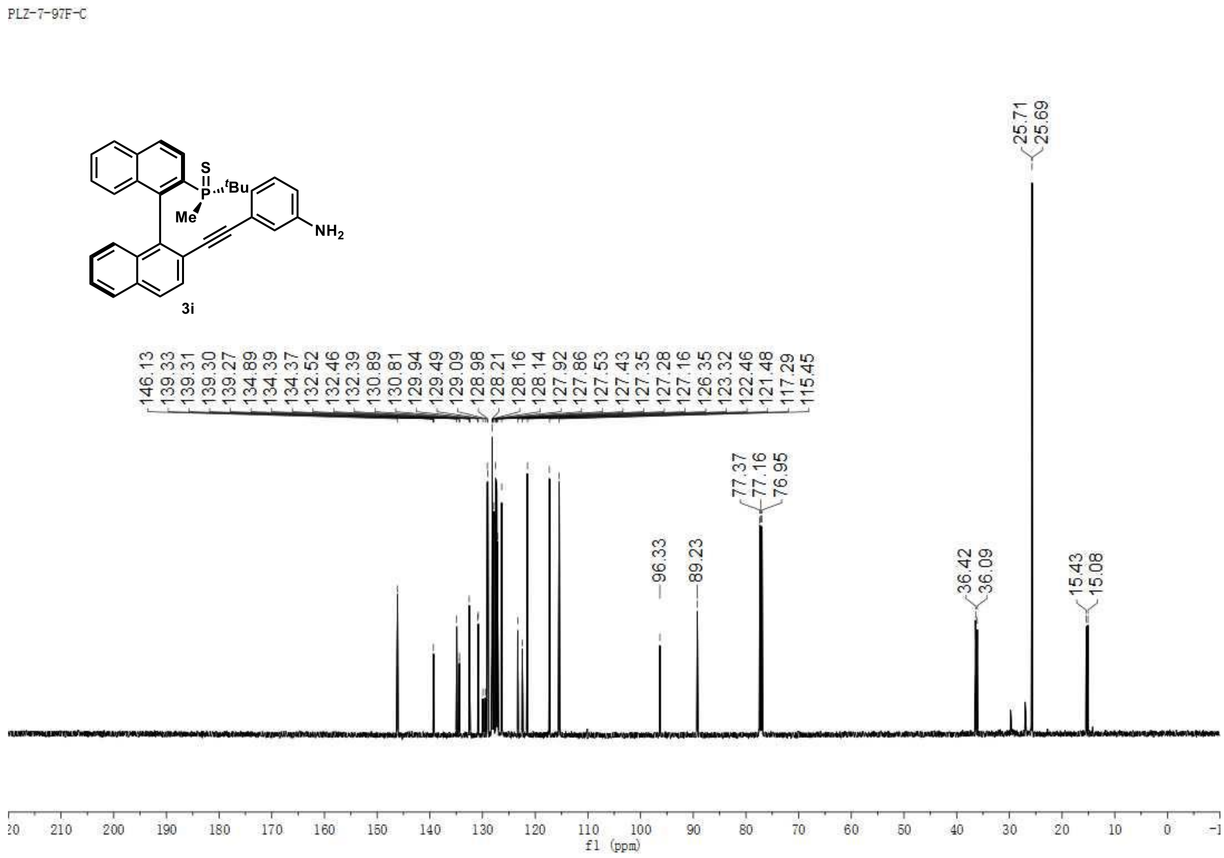

Supplementary Fig. 69.^{13}C NMR spectrum of **3i**. The sample has been recorded in 151 MHz, $CDCl_3$ at 25 ℃.

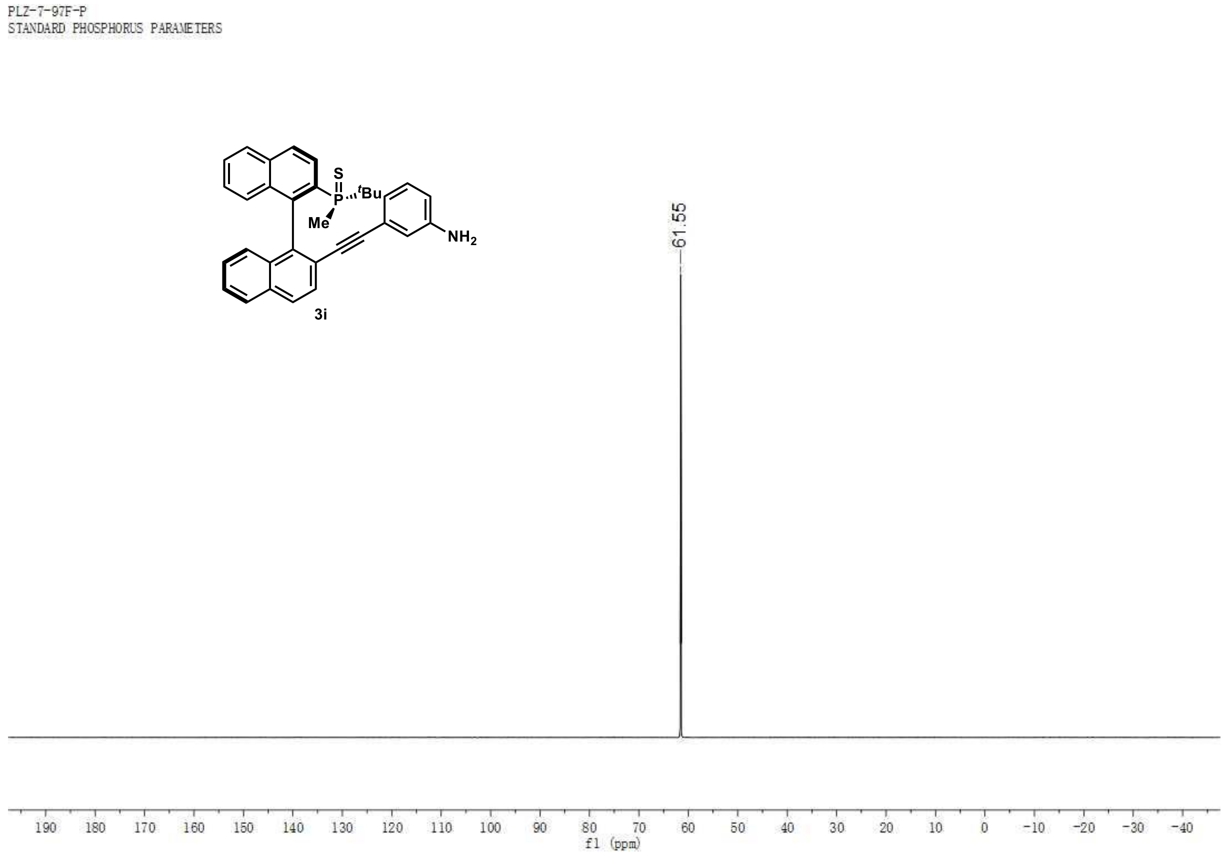

Supplementary Fig. 70.^{31}P NMR spectrum of **3i**. The sample has been recorded in 243 MHz, $CDCl_3$ at 25 ℃.

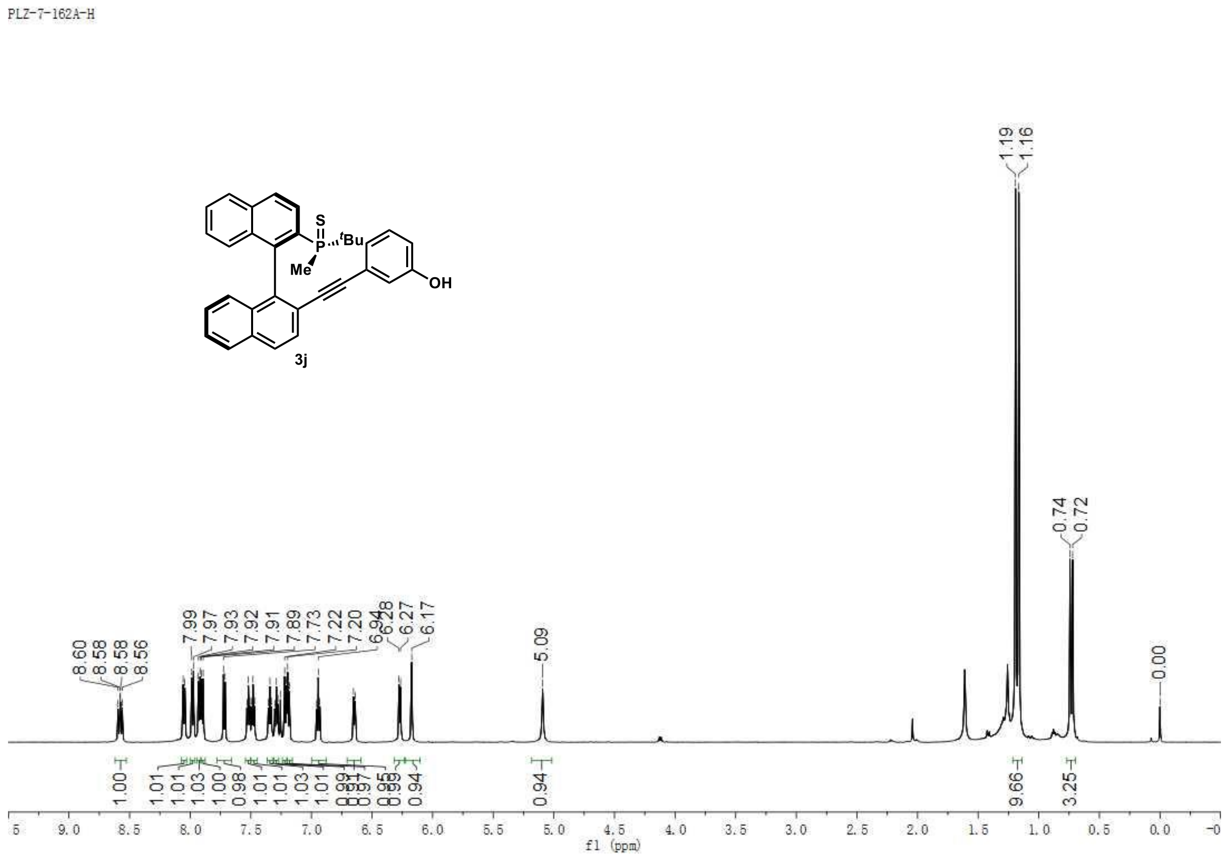

Supplementary Fig. 71. ^{1}H NMR spectrum of **3j**. The sample has been recorded in 600 MHz, $CDCl_3$ at 25 ℃.

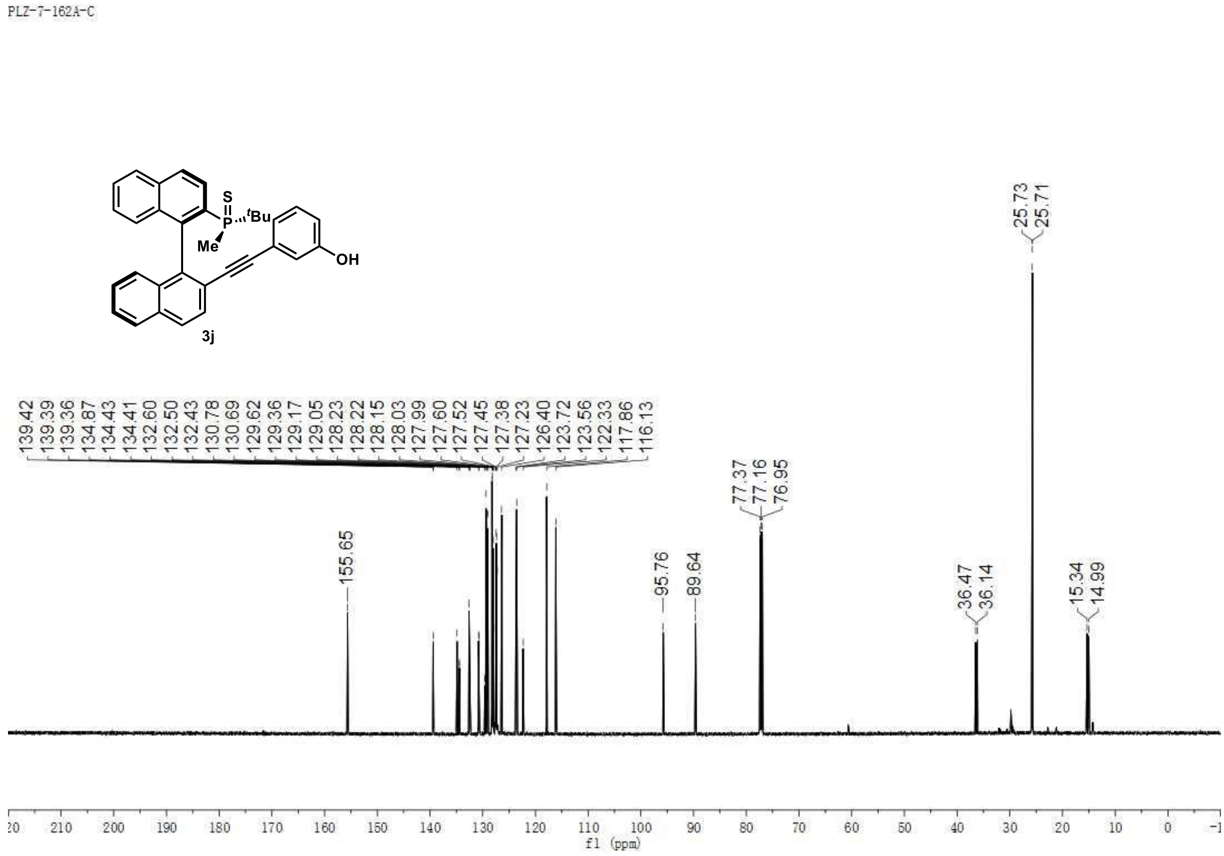

Supplementary Fig. 72.^{13}C NMR spectrum of **3j**. The sample has been recorded in 151 MHz, $CDCl_3$ at 25 ℃.

PLZ-7-162A-P
STANDARD PHOSPHORUS PARAMETERS

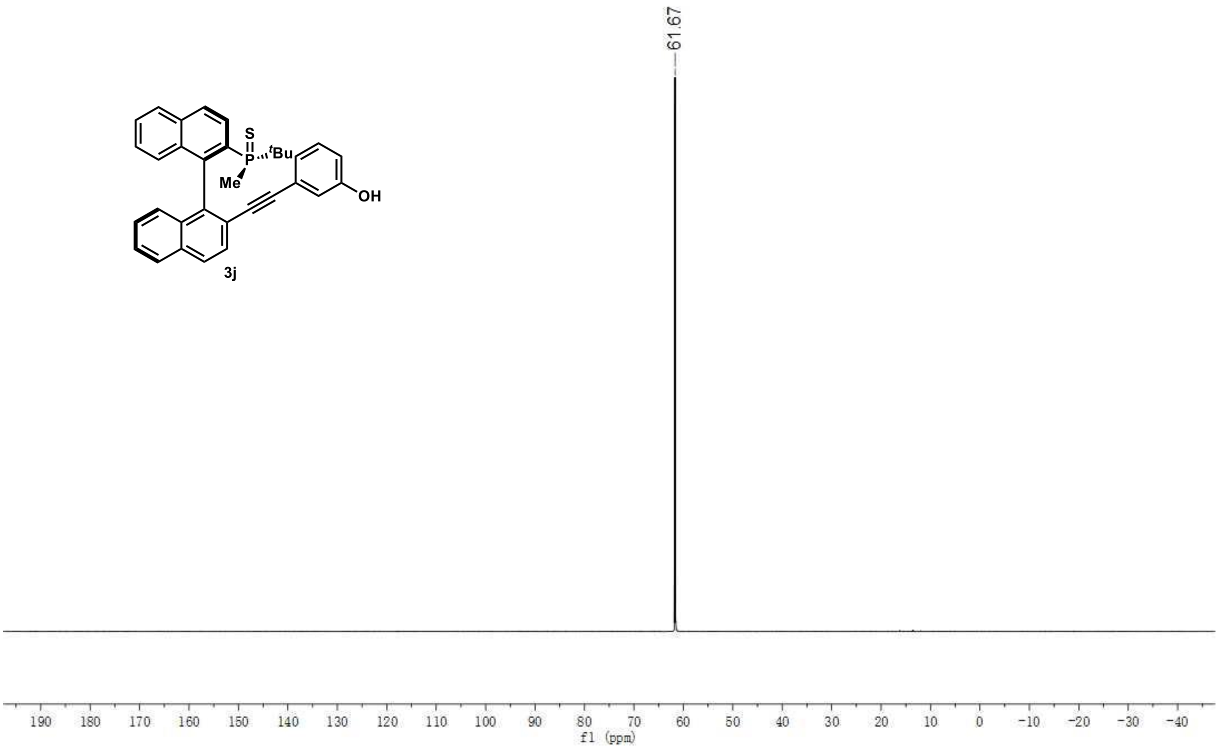

Supplementary Fig. 73.^{31}P NMR spectrum of **3j**. The sample has been recorded in 243 MHz, $CDCl_3$ at 25 ℃.

PLZ-7-130-H

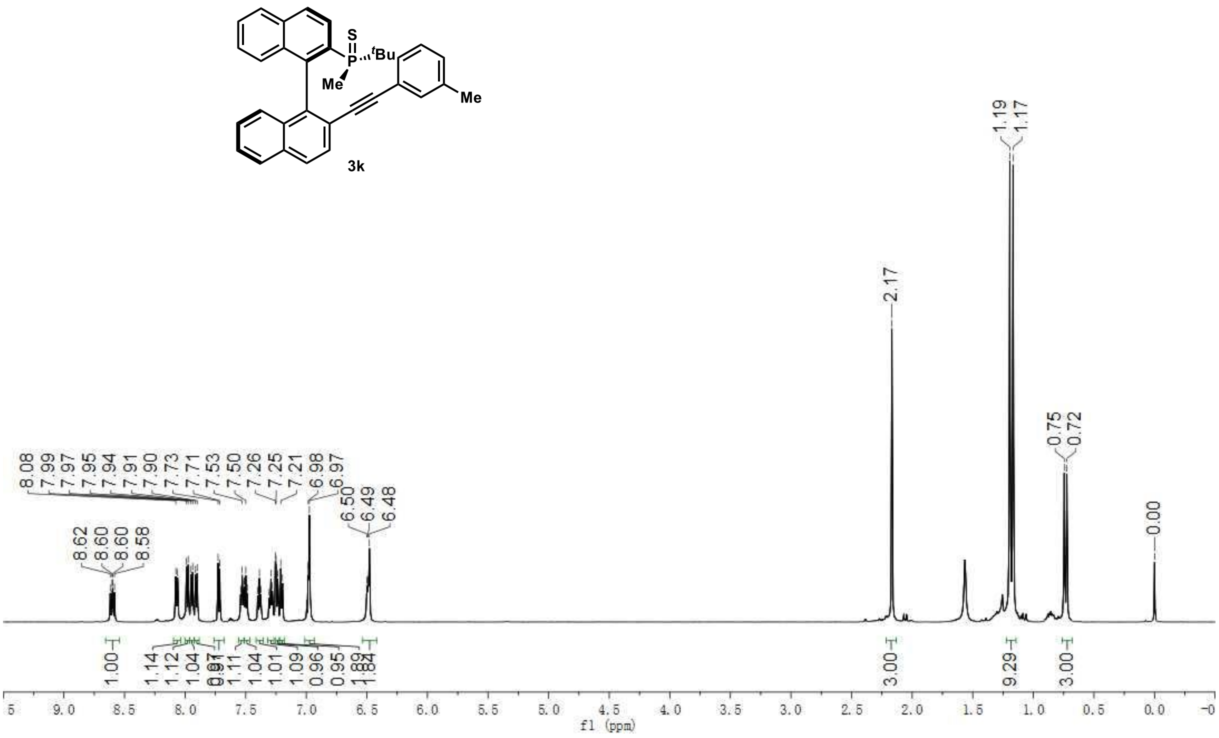

Supplementary Fig. 74. ^{1}H NMR spectrum of **3k**. The sample has been recorded in 600 MHz, $CDCl_3$ at 25 ℃.

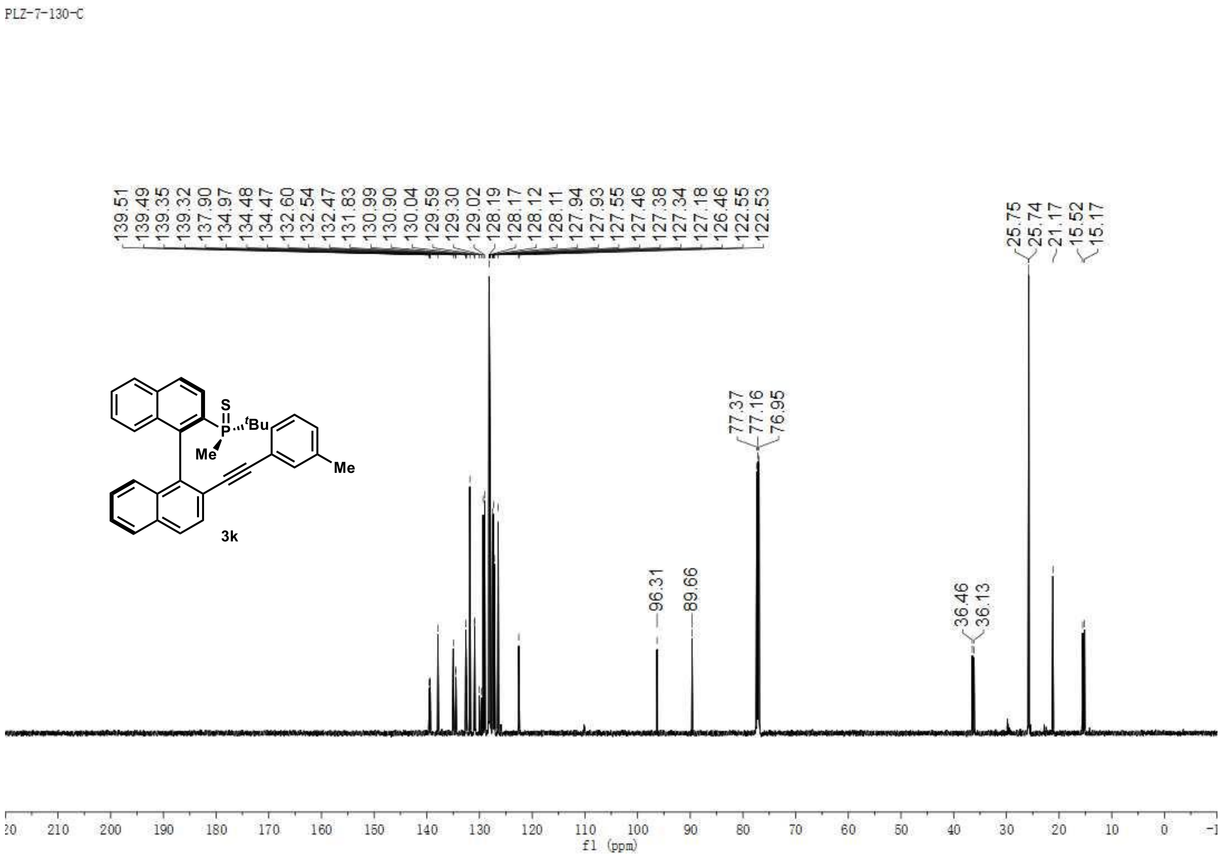

Supplementary Fig. 75.^{13}C NMR spectrum of **3k**. The sample has been recorded in 151 MHz, $CDCl_3$ at 25 ℃.

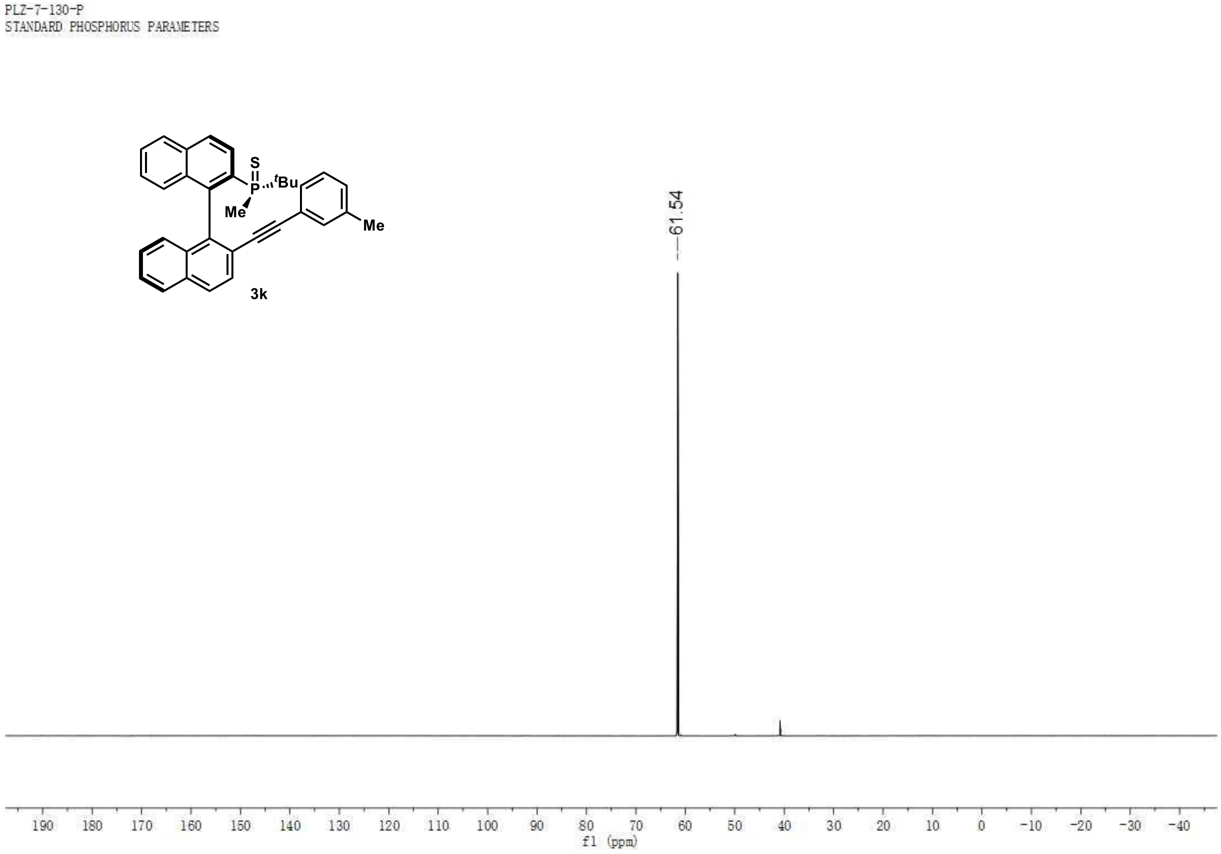

Supplementary Fig. 76.^{31}P NMR spectrum of **3k**. The sample has been recorded in 243 MHz, $CDCl_3$ at 25 ℃.

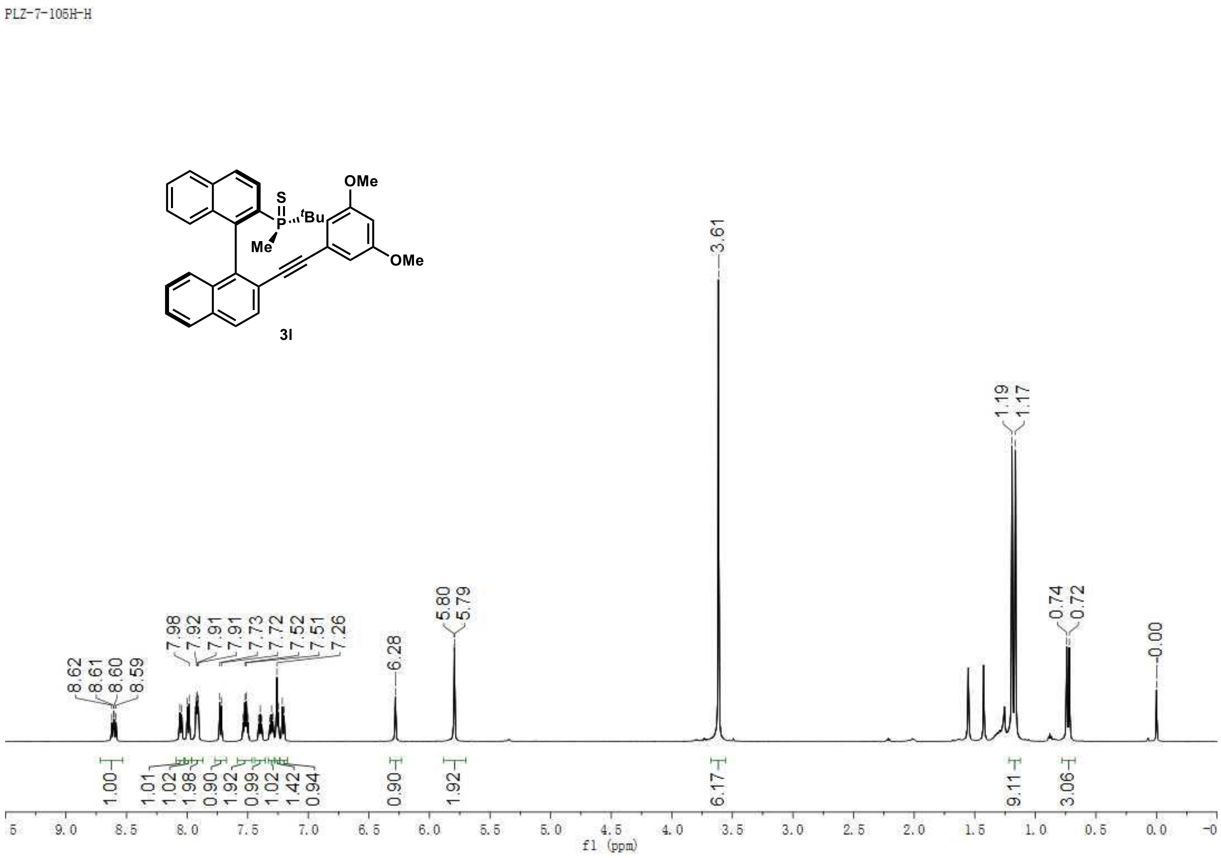

Supplementary Fig. 77. ^{1}H NMR spectrum of **3l**. The sample has been recorded in 600 MHz, $CDCl_3$ at 25 ℃.

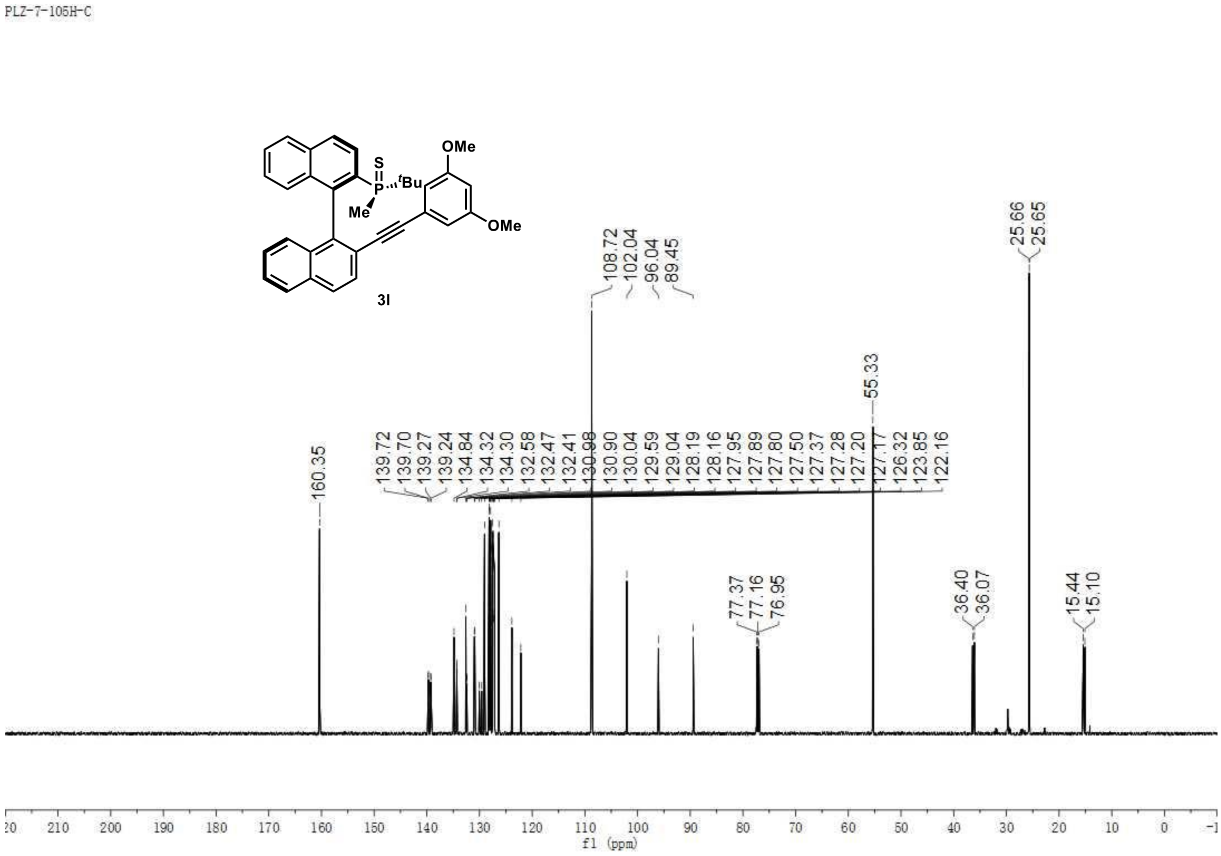

Supplementary Fig. 78.^{13}C NMR spectrum of **3l**. The sample has been recorded in 151 MHz, $CDCl_3$ at 25 ℃.

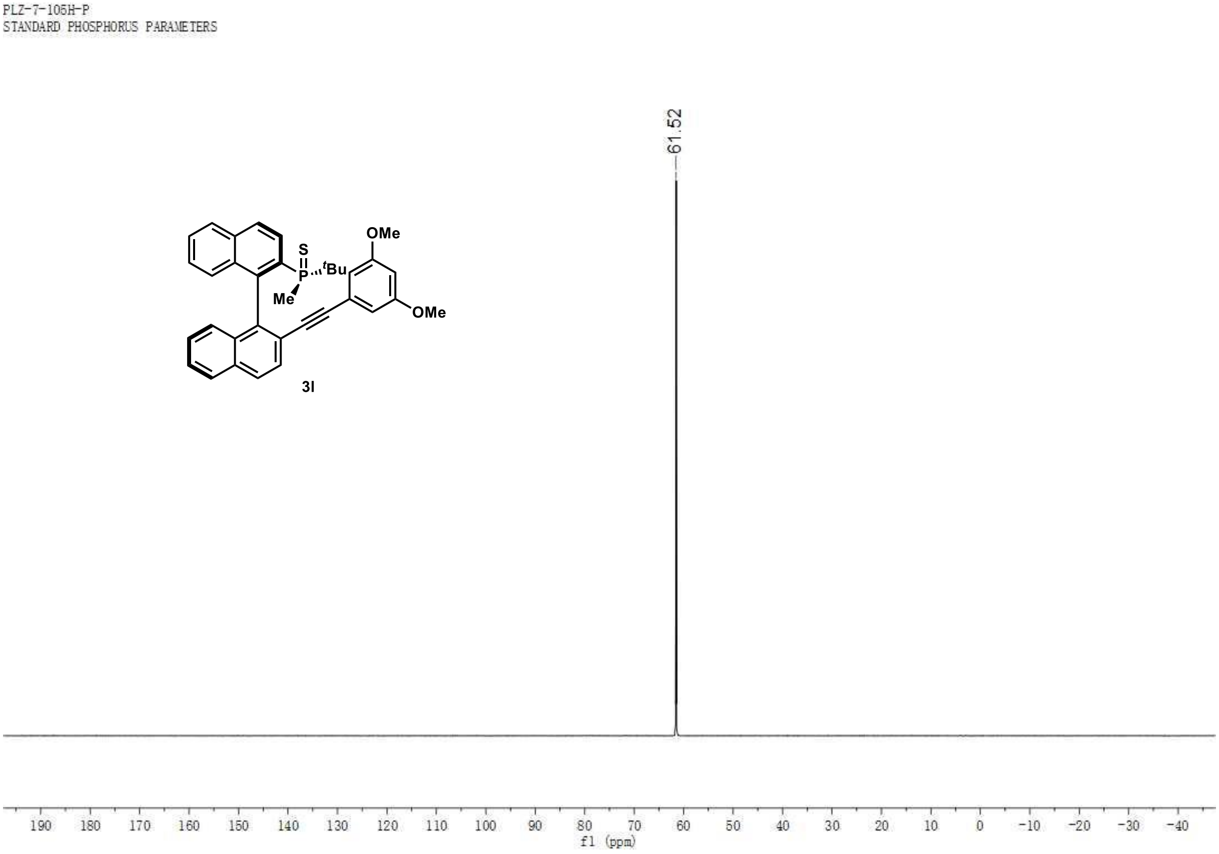

Supplementary Fig. 79.^{31}P NMR spectrum of **3l**. The sample has been recorded in 243 MHz, $CDCl_3$ at 25 ℃.

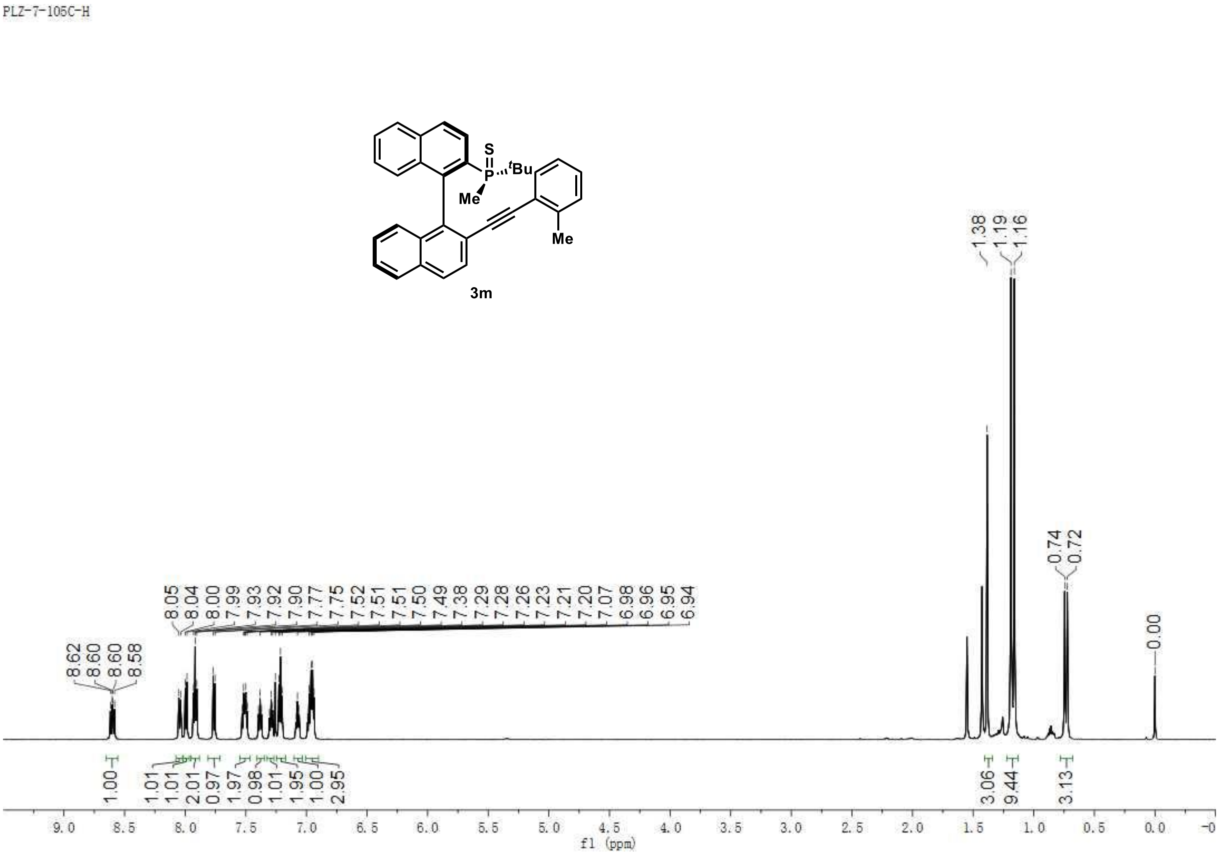

Supplementary Fig. 80. ^{1}H NMR spectrum of **3m**. The sample has been recorded in 600 MHz, $CDCl_3$ at 25 ℃.

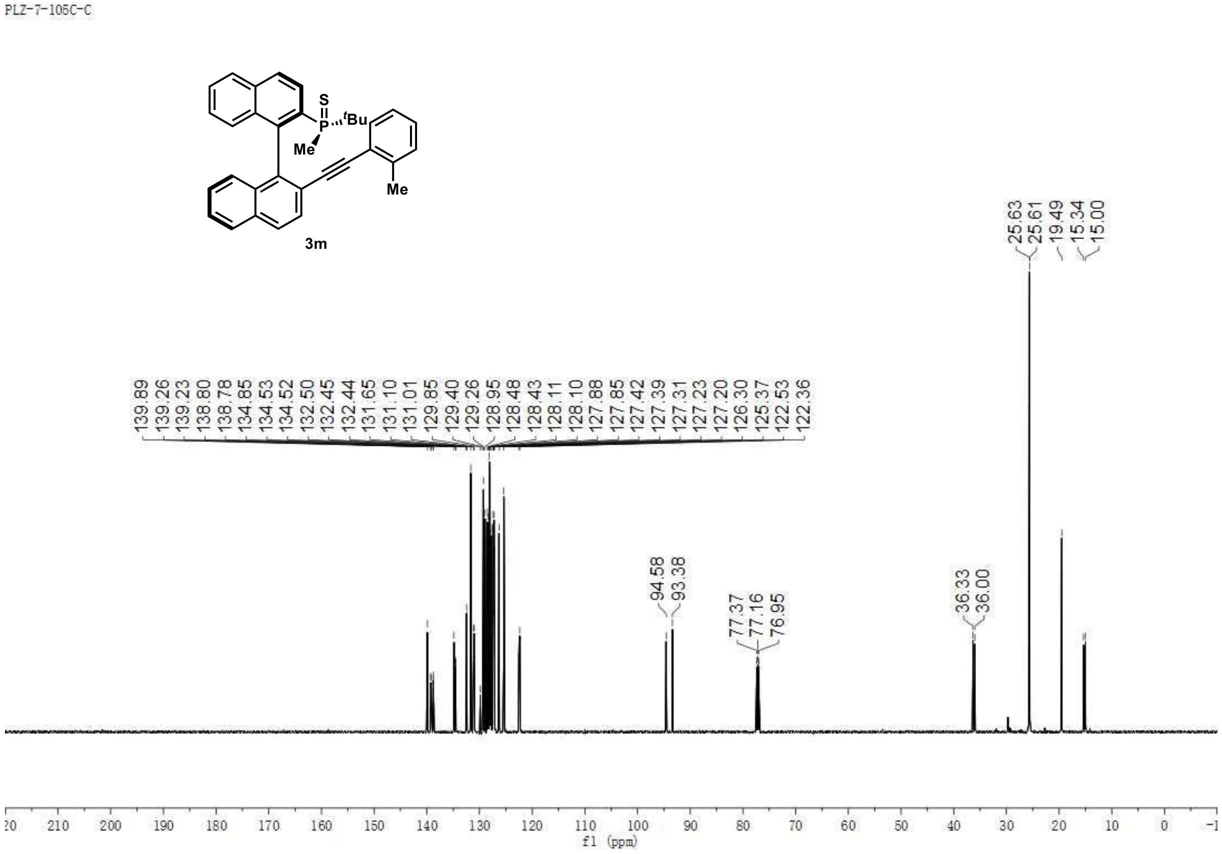

Supplementary Fig. 81.^{13}C NMR spectrum of **3m**. The sample has been recorded in 151 MHz, $CDCl_3$ at 25 ℃.

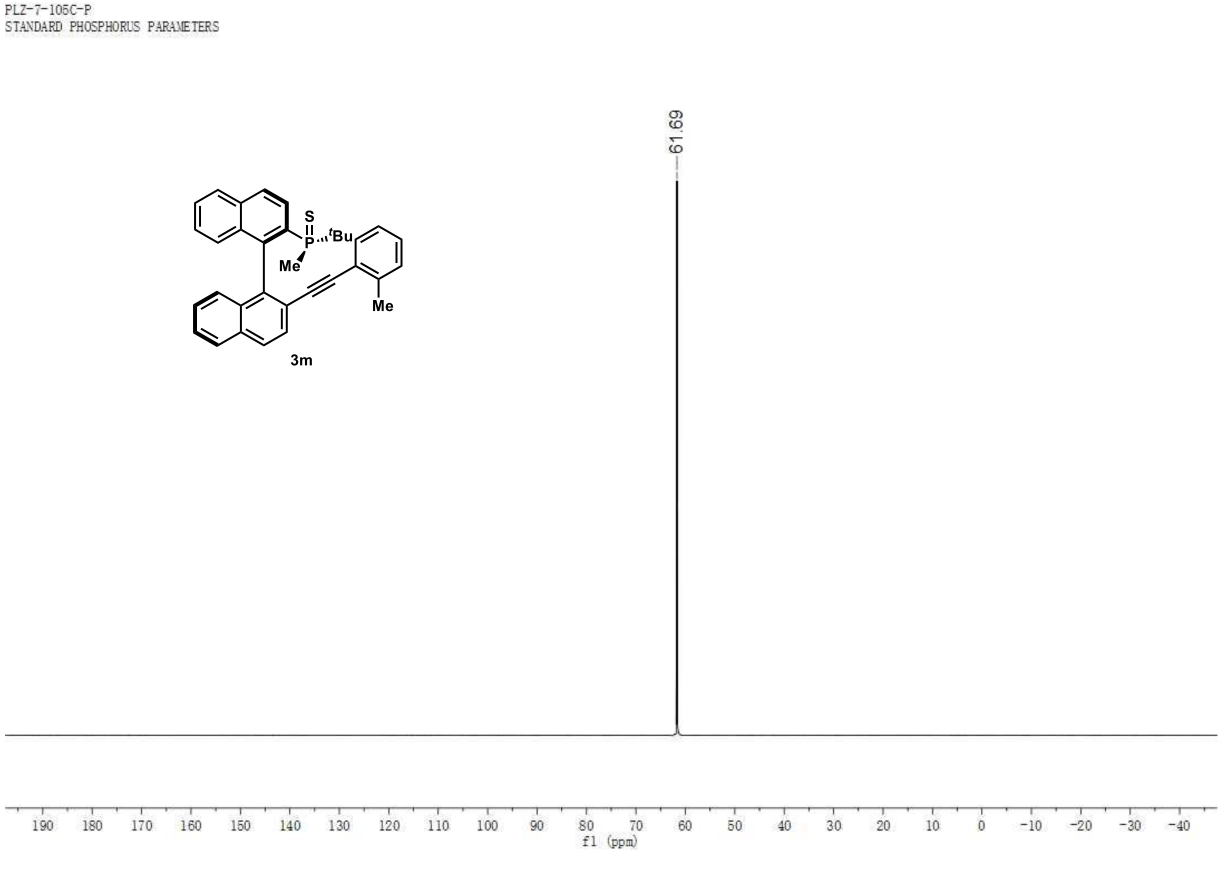

Supplementary Fig. 82.^{31}P NMR spectrum of **3m**. The sample has been recorded in 243 MHz, $CDCl_3$ at 25 ℃.

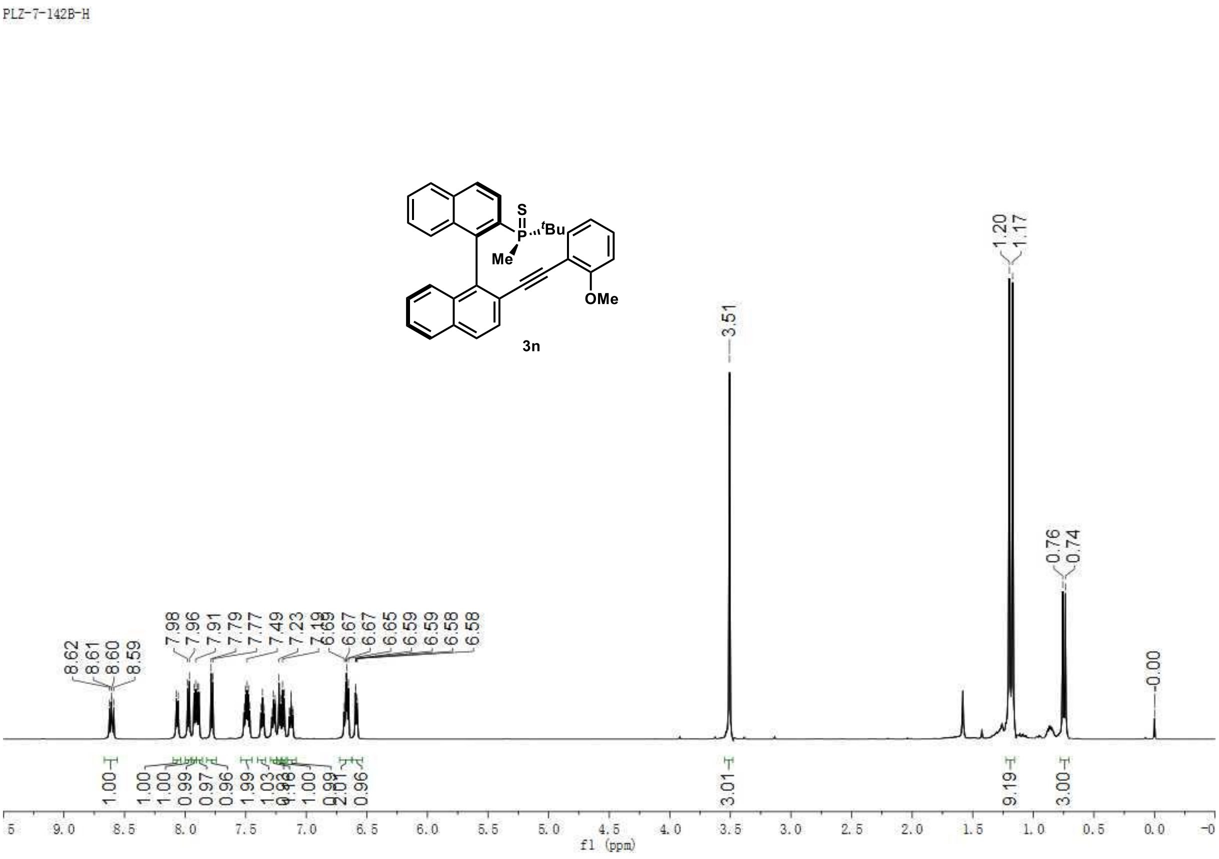

Supplementary Fig. 83. ^{1}H NMR spectrum of **3n**. The sample has been recorded in 600 MHz, $CDCl_3$ at 25 ℃.

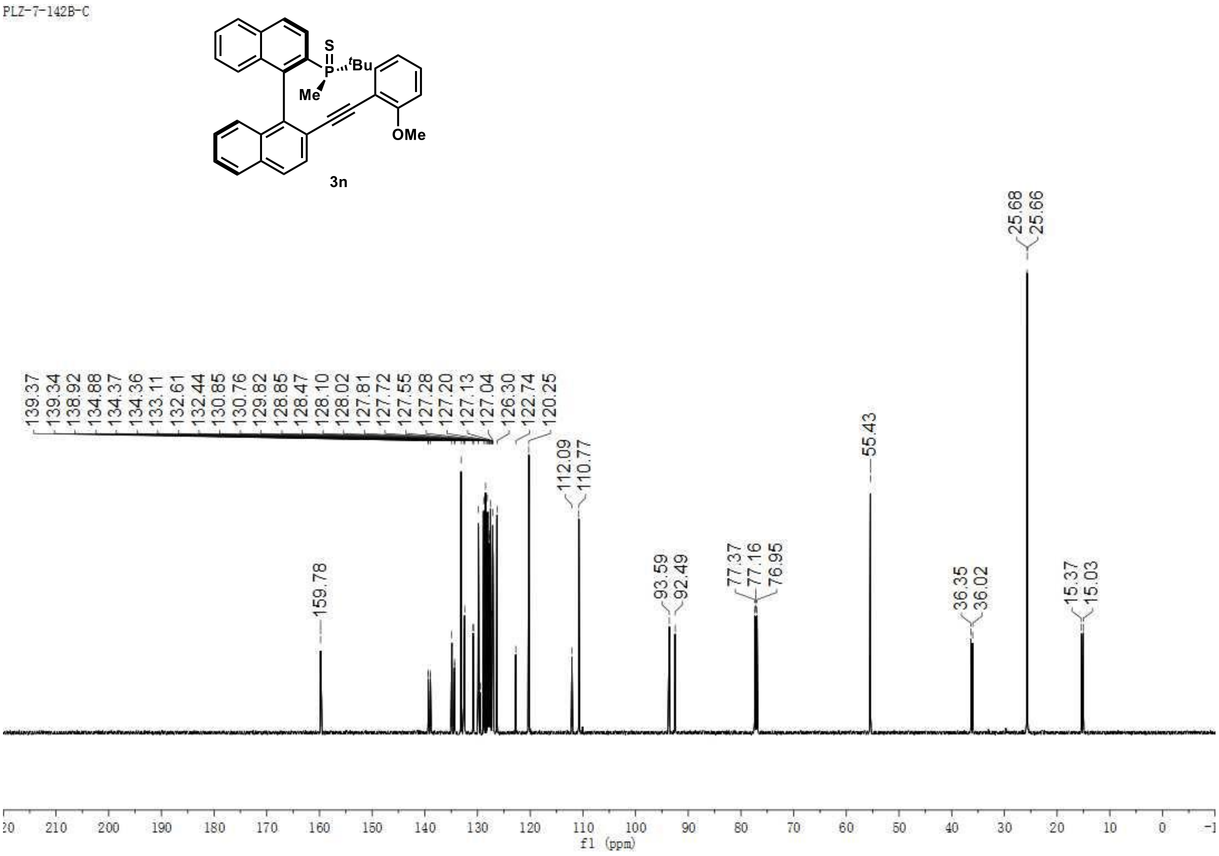

Supplementary Fig. 84.^{13}C NMR spectrum of **3n**. The sample has been recorded in 151 MHz, $CDCl_3$ at 25 ℃.

PLZ-7-142B-P
STANDARD PHOSPHORUS PARAMETERS

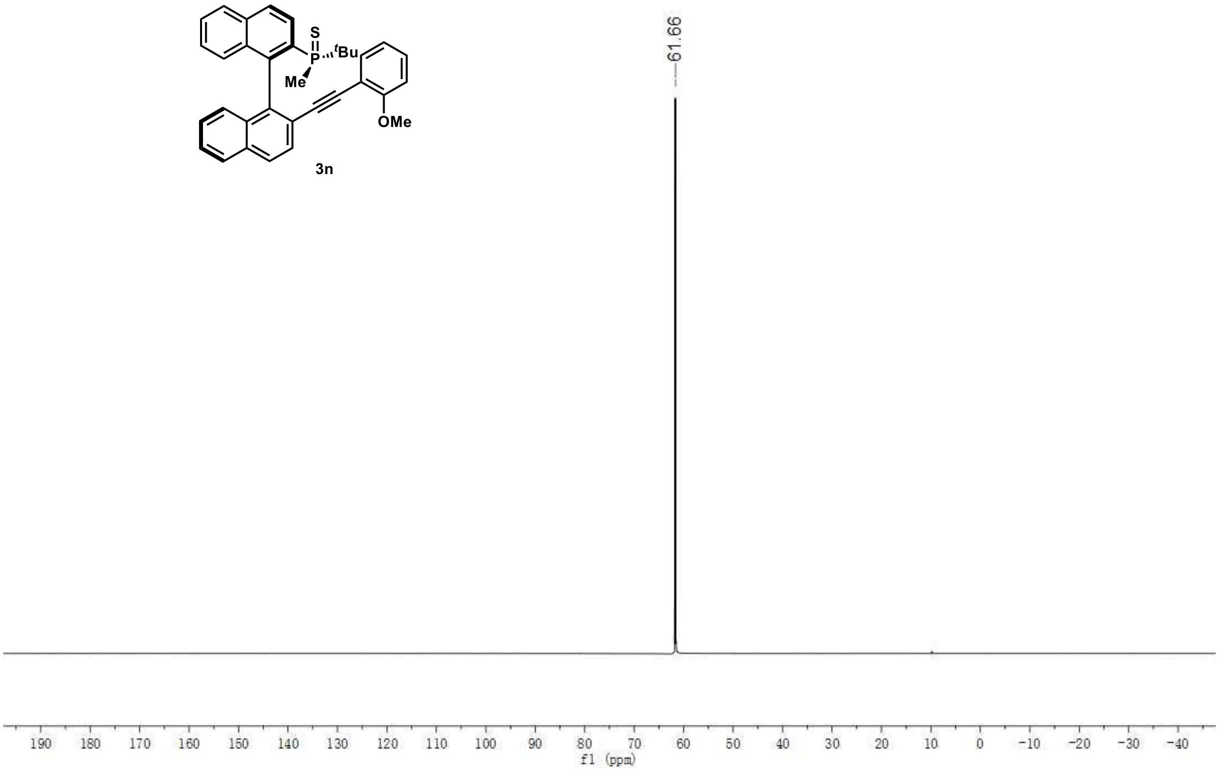

Supplementary Fig. 85.^{31}P NMR spectrum of **3n**. The sample has been recorded in 243 MHz, $CDCl_3$ at 25 ℃.

PLZ-7-142A-H

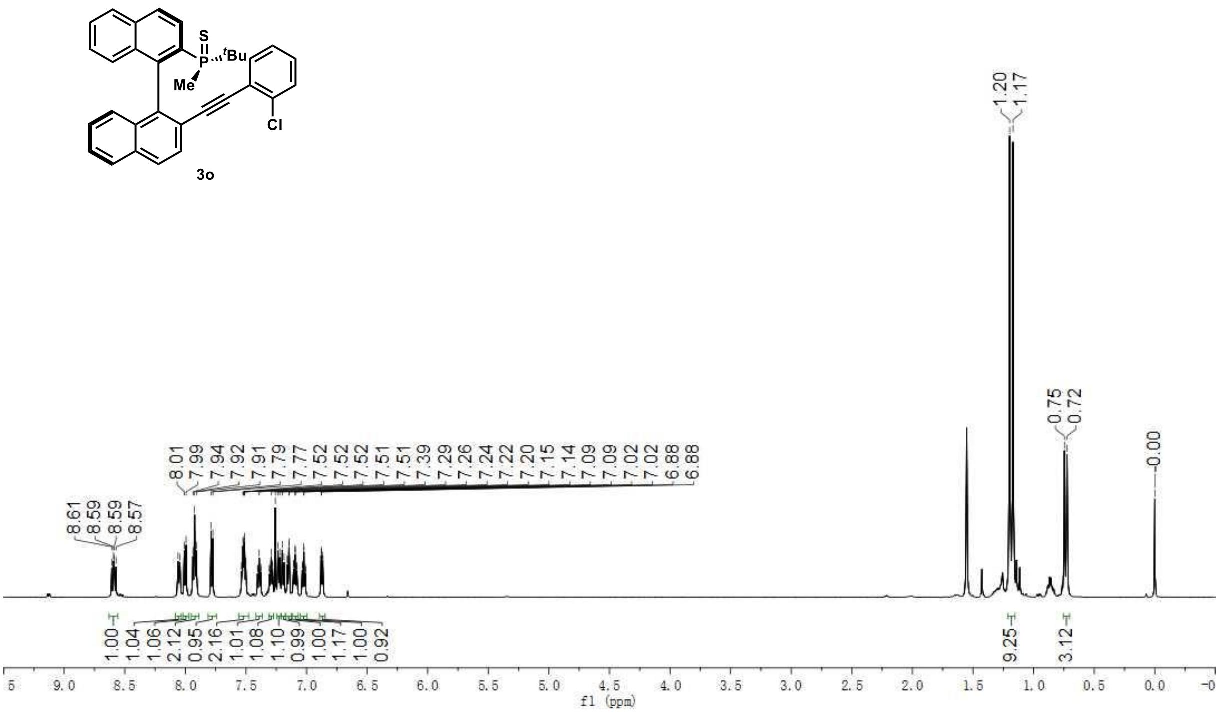

Supplementary Fig. 86. ^{1}H NMR spectrum of **3o**. The sample has been recorded in 600 MHz, $CDCl_3$ at 25 ℃.

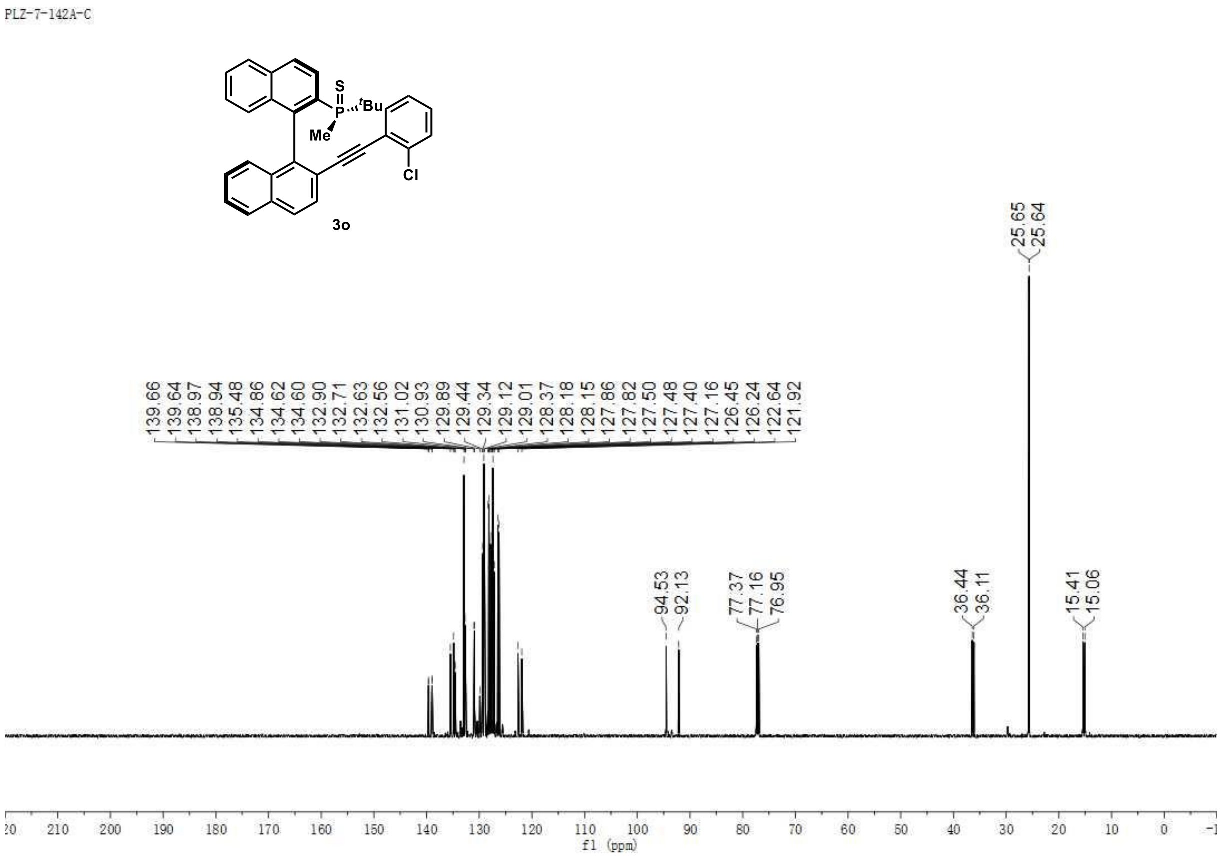

Supplementary Fig. 87.^{13}C NMR spectrum of **3o**. The sample has been recorded in 151 MHz, $CDCl_3$ at 25 ℃.

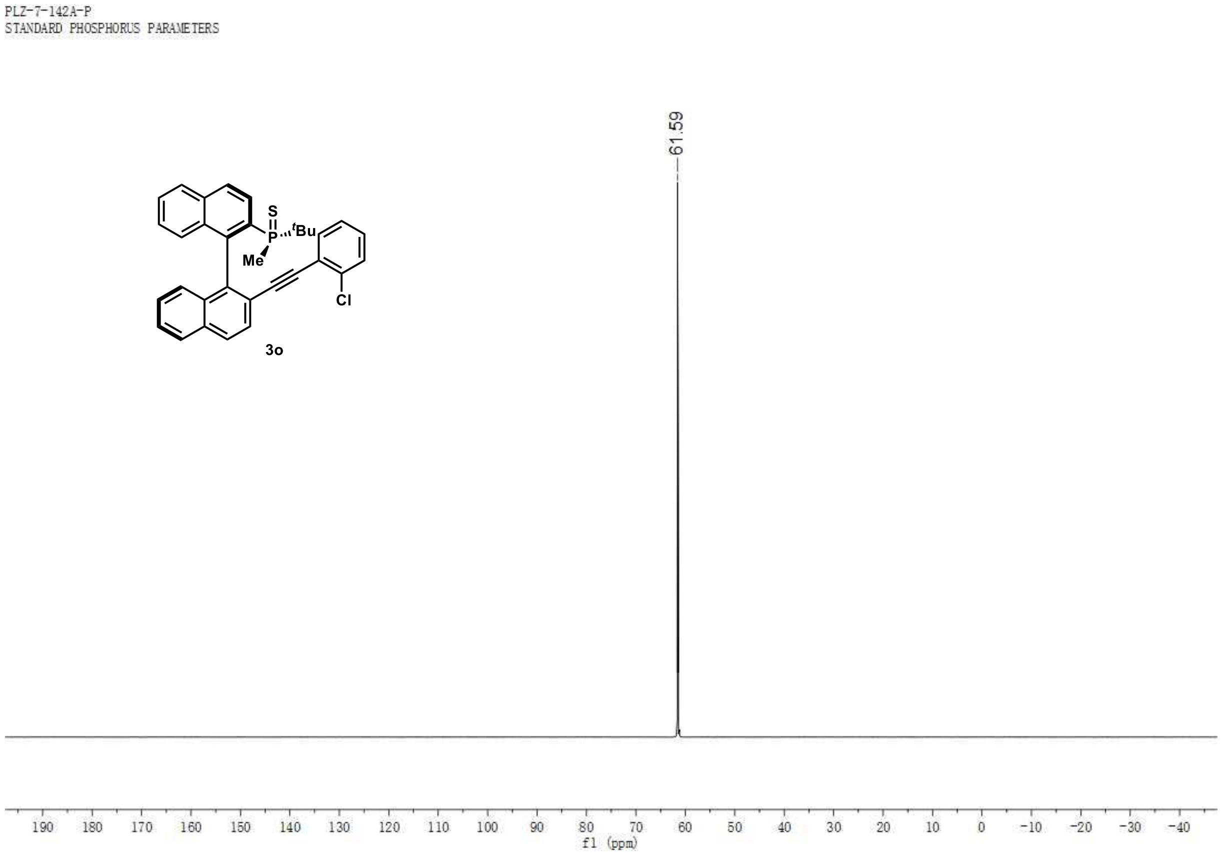

Supplementary Fig. 88.^{31}P NMR spectrum of **3o**. The sample has been recorded in 243 MHz, $CDCl_3$ at 25 ℃.

PLZ-7-145B-H

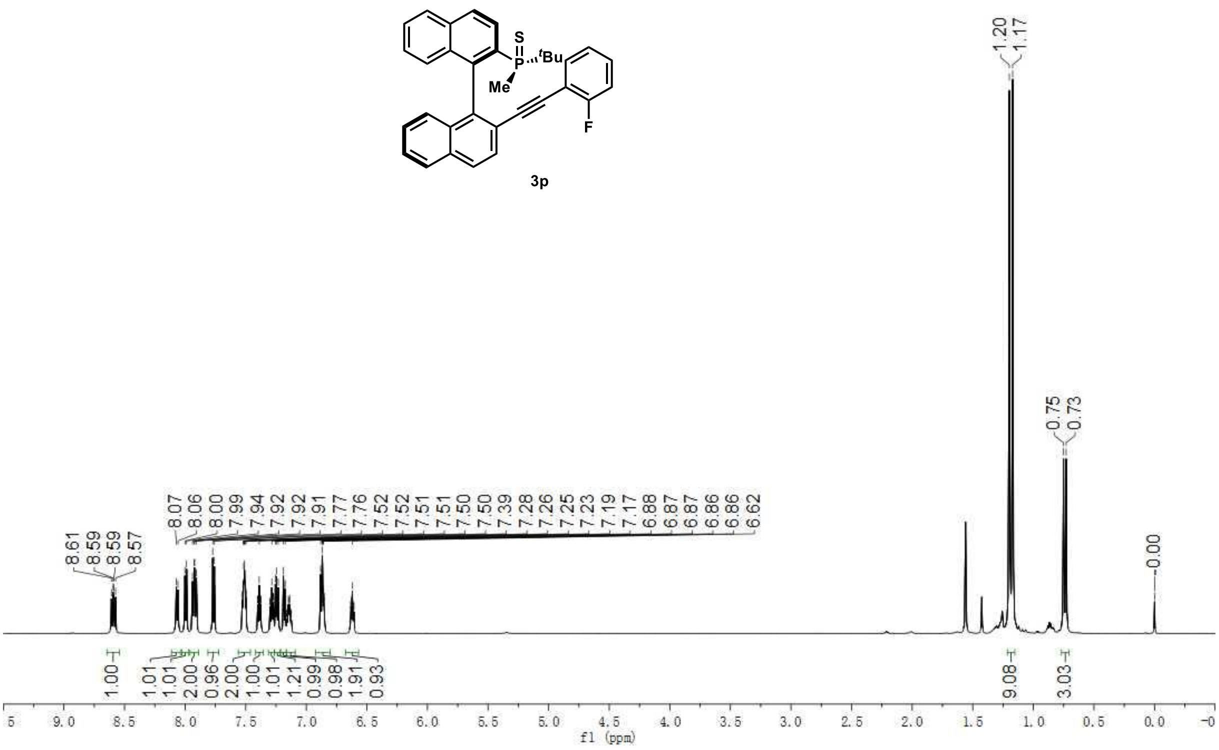

Supplementary Fig. 89. ^{1}H NMR spectrum of **3p**. The sample has been recorded in 600 MHz, $CDCl_3$ at 25 ℃.

PLZ-7-145B-C

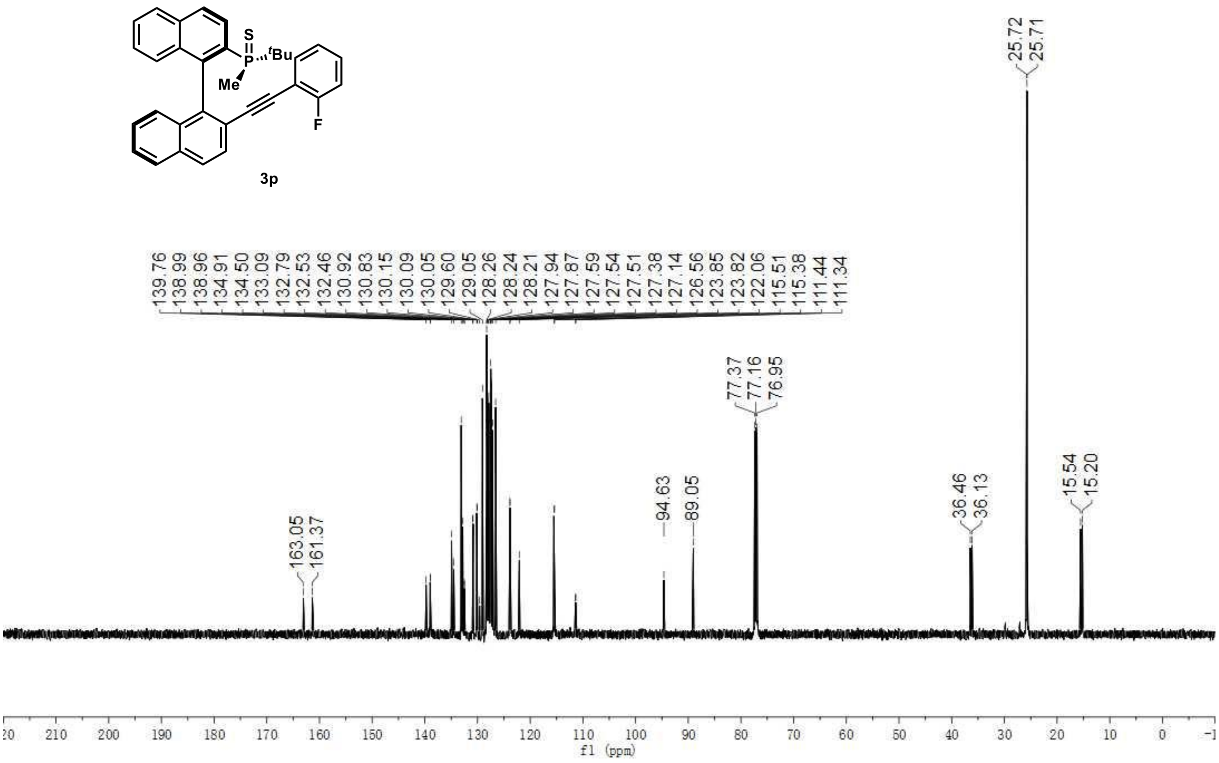

Supplementary Fig. 90.^{13}C NMR spectrum of **3p**. The sample has been recorded in 151 MHz, $CDCl_3$ at 25 ℃.

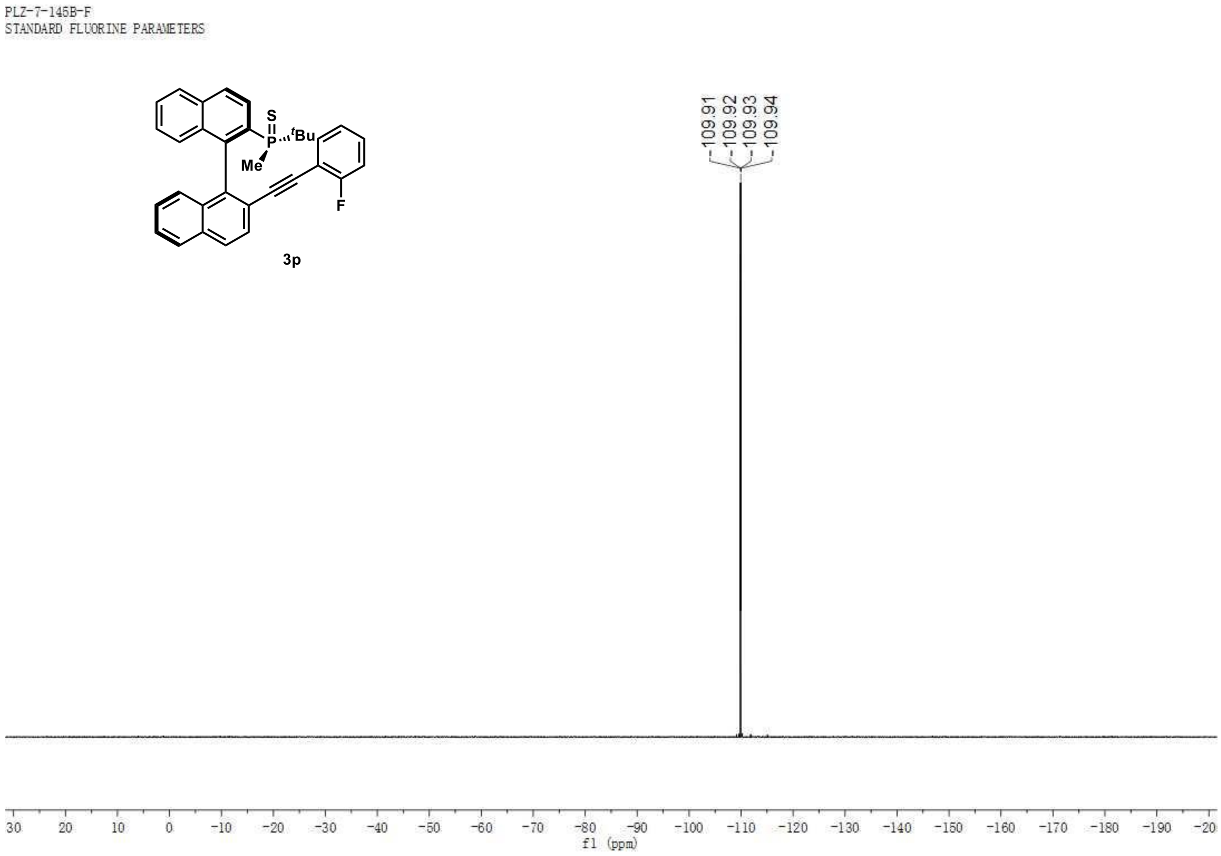

Supplementary Fig. 91.^{19}F NMR spectrum of **3p**. The sample has been recorded in 564 MHz, $CDCl_3$ at 25 ℃.

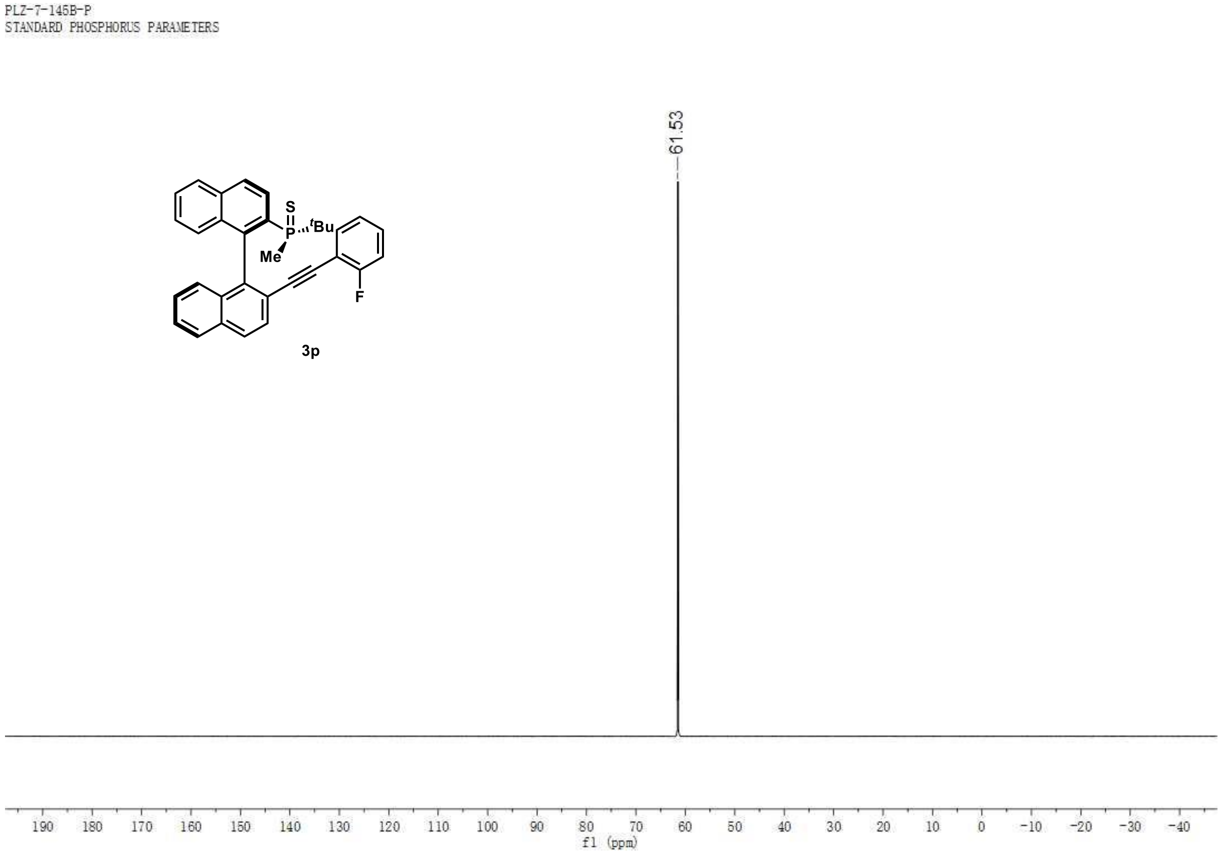

Supplementary Fig. 92.^{31}P NMR spectrum of **3p**. The sample has been recorded in 243 MHz, $CDCl_3$ at 25 ℃.

PLZ-7-98F-H

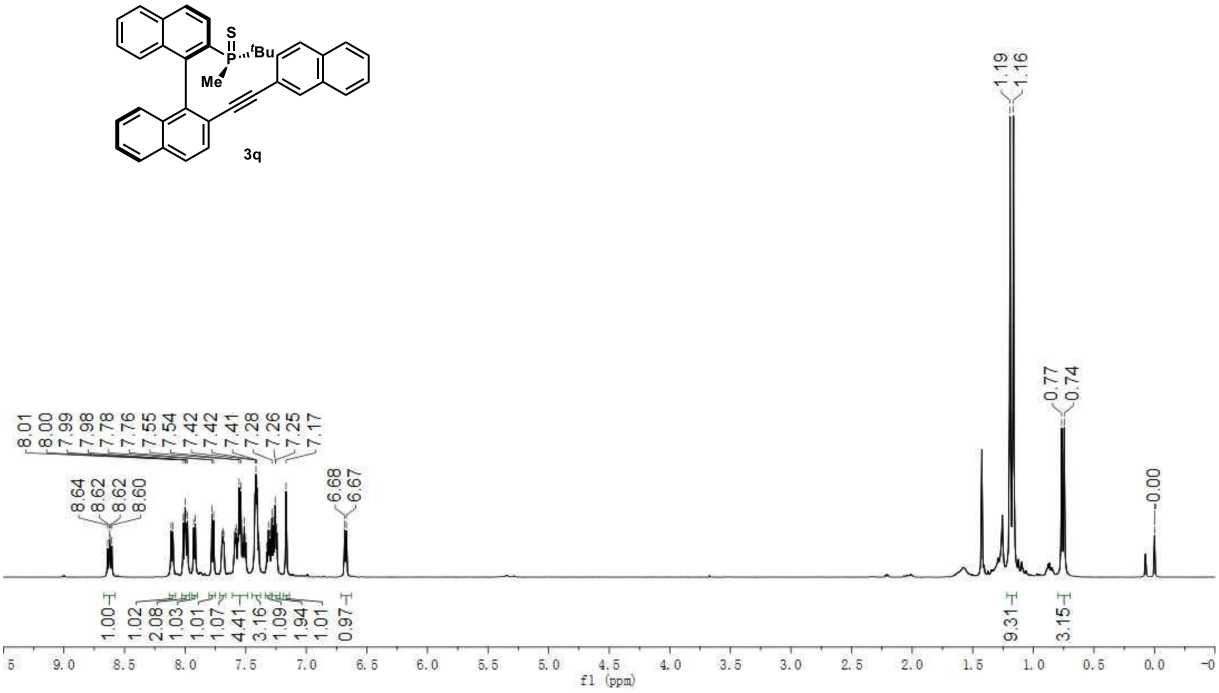

Supplementary Fig. 93. ^{1}H NMR spectrum of **3q**. The sample has been recorded in 600 MHz, $CDCl_3$ at 25 ℃.

PLZ-7-98F-C

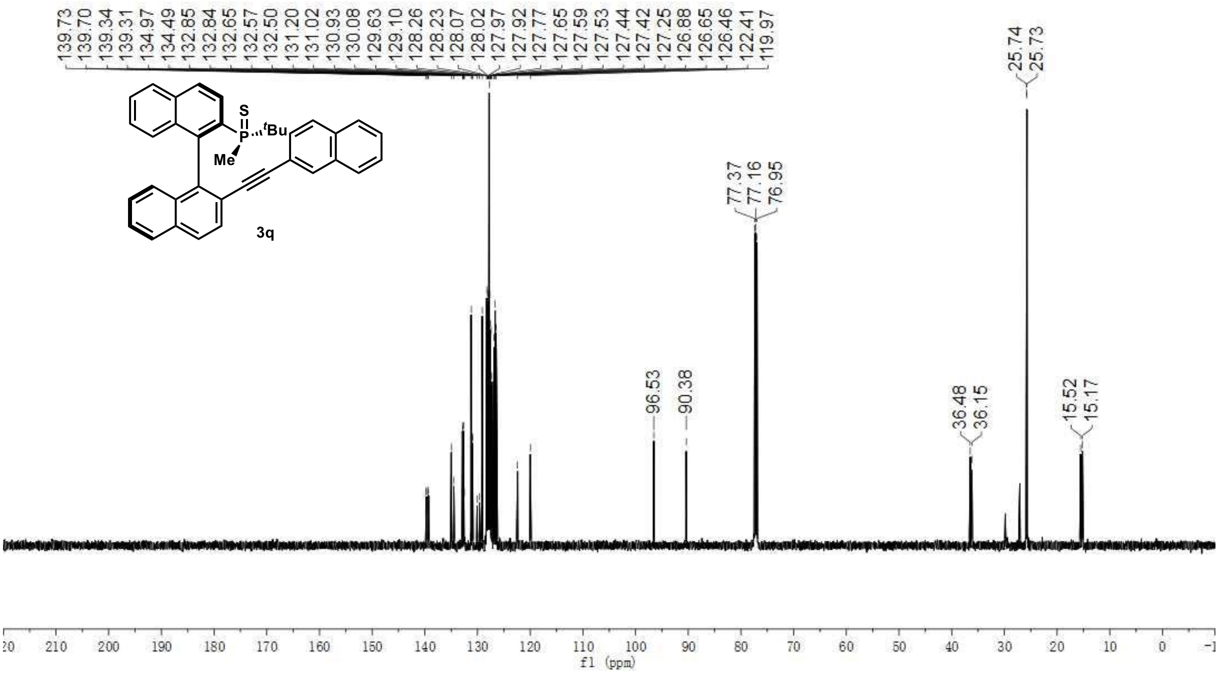

Supplementary Fig. 94.^{13}C NMR spectrum of **3q**. The sample has been recorded in 151 MHz, $CDCl_3$ at 25 ℃.

PLZ-7-98F-P
STANDARD PHOSPHORUS PARAMETERS

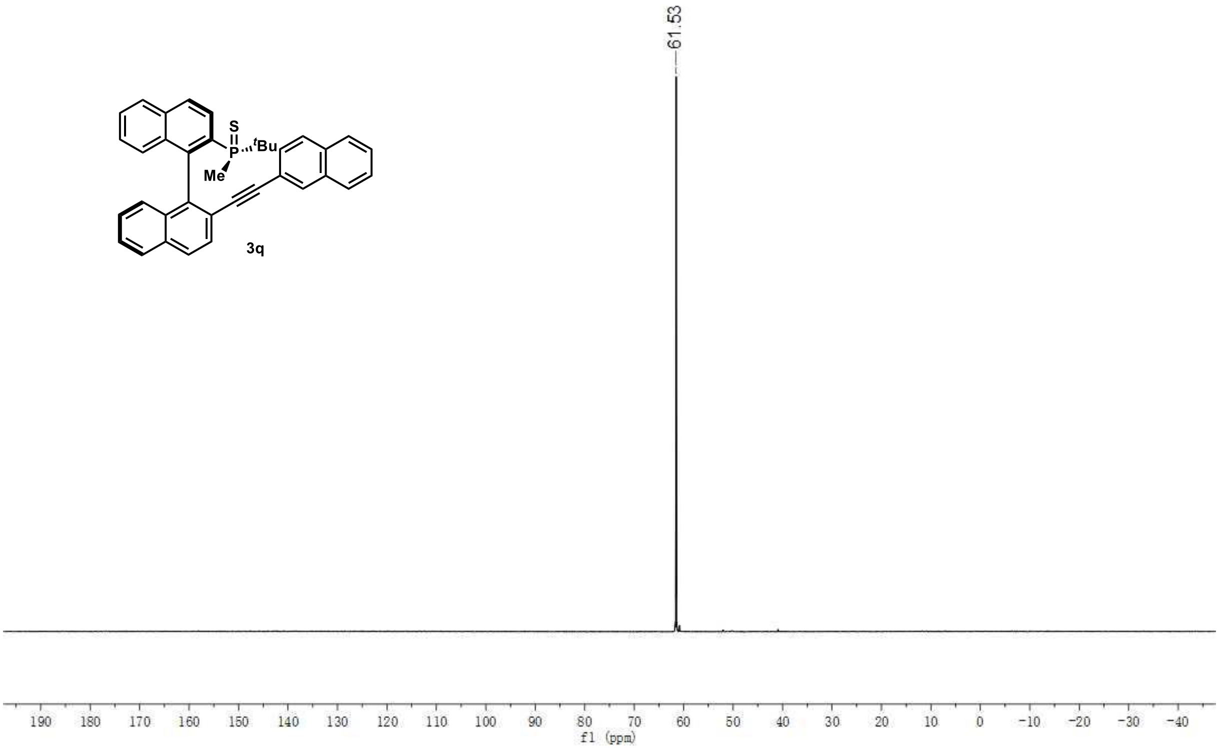

Supplementary Fig. 95.^{31}P NMR spectrum of **3q**. The sample has been recorded in 243 MHz, $CDCl_3$ at 25 ℃.

PLZ-7-165

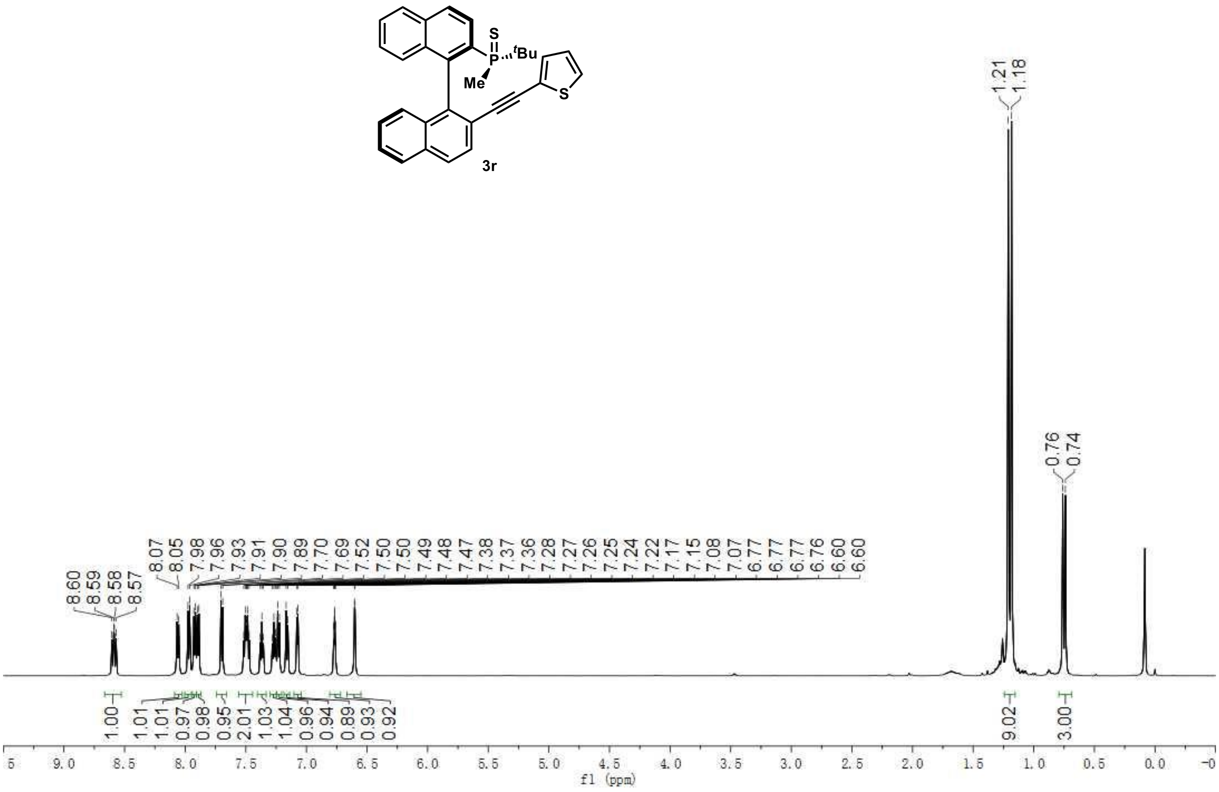

Supplementary Fig. 96. ^{1}H NMR spectrum of **3r**. The sample has been recorded in 600 MHz, $CDCl_3$ at 25 ℃.

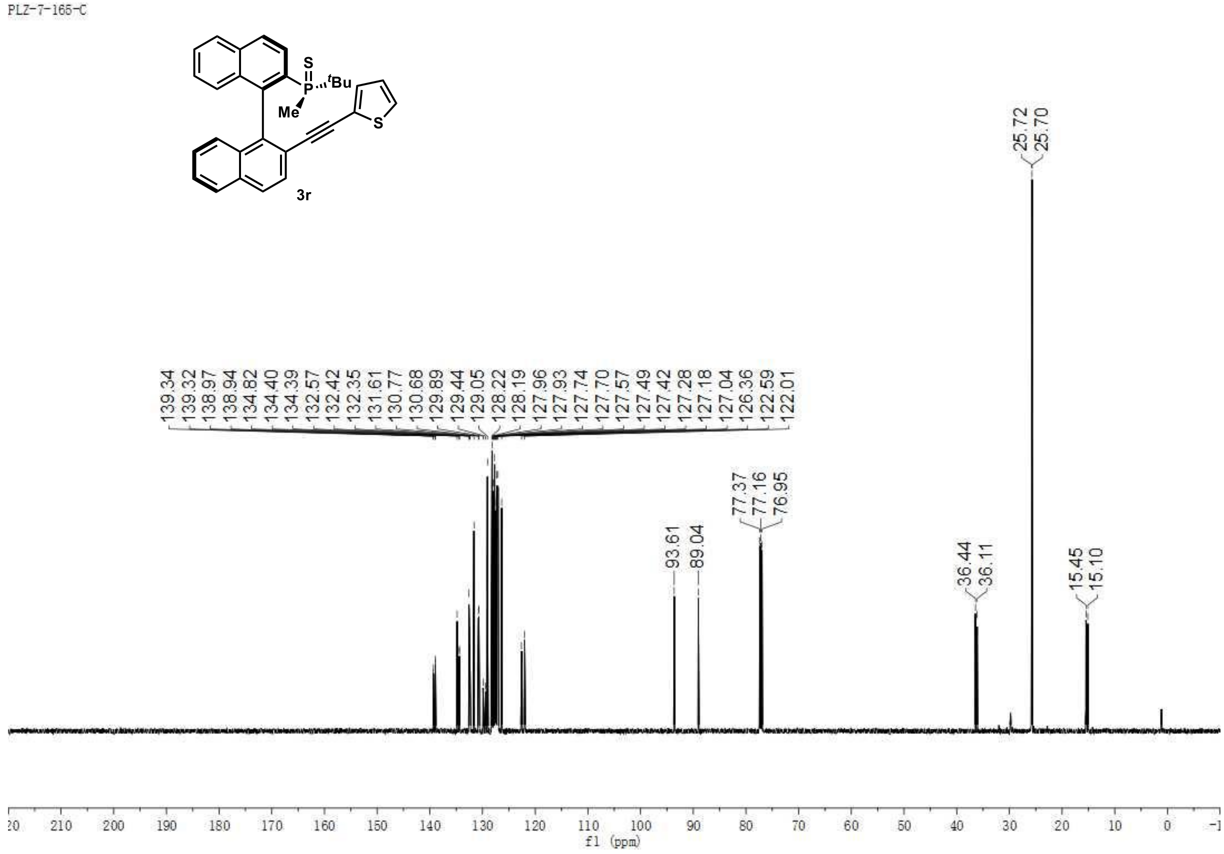

Supplementary Fig. 97.^{13}C NMR spectrum of **3r**. The sample has been recorded in 151 MHz, $CDCl_3$ at 25 ℃.

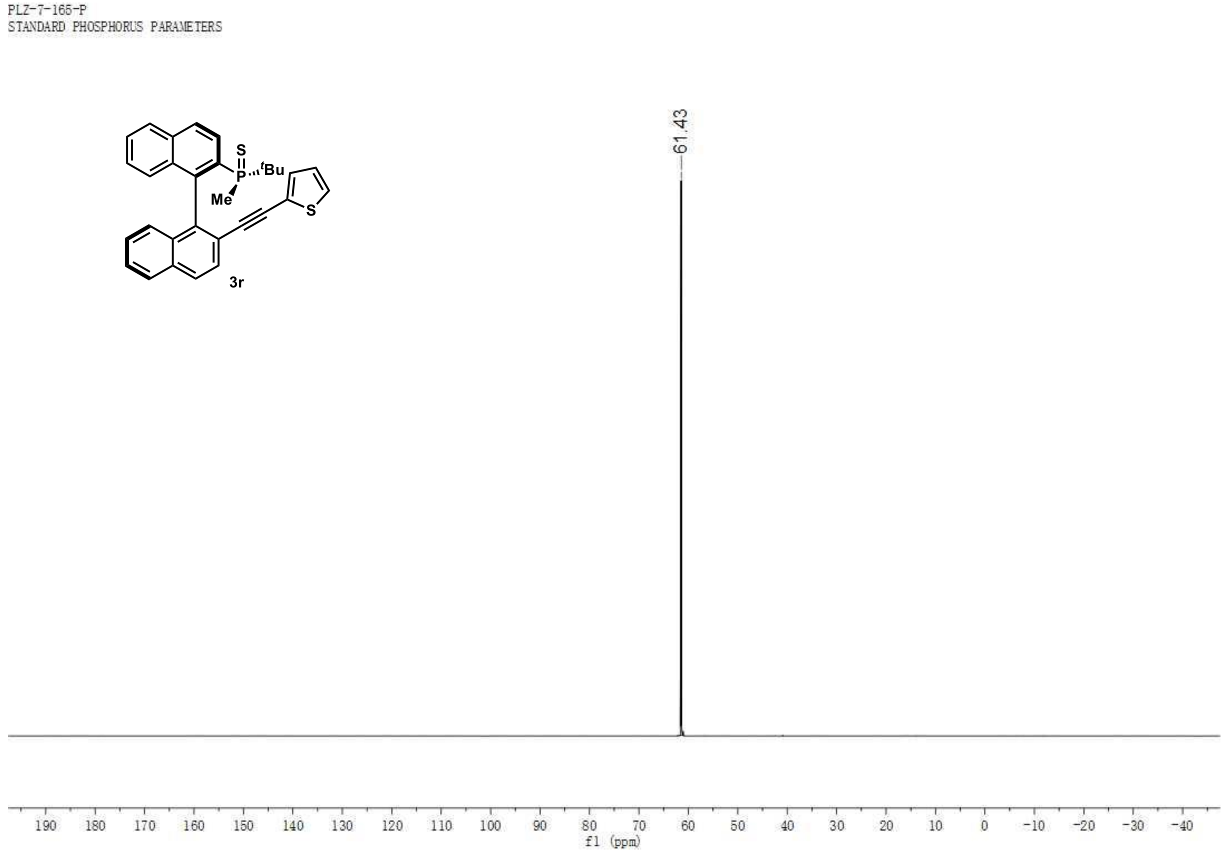

Supplementary Fig. 98.^{31}P NMR spectrum of **3r**. The sample has been recorded in 243 MHz, $CDCl_3$ at 25 ℃.

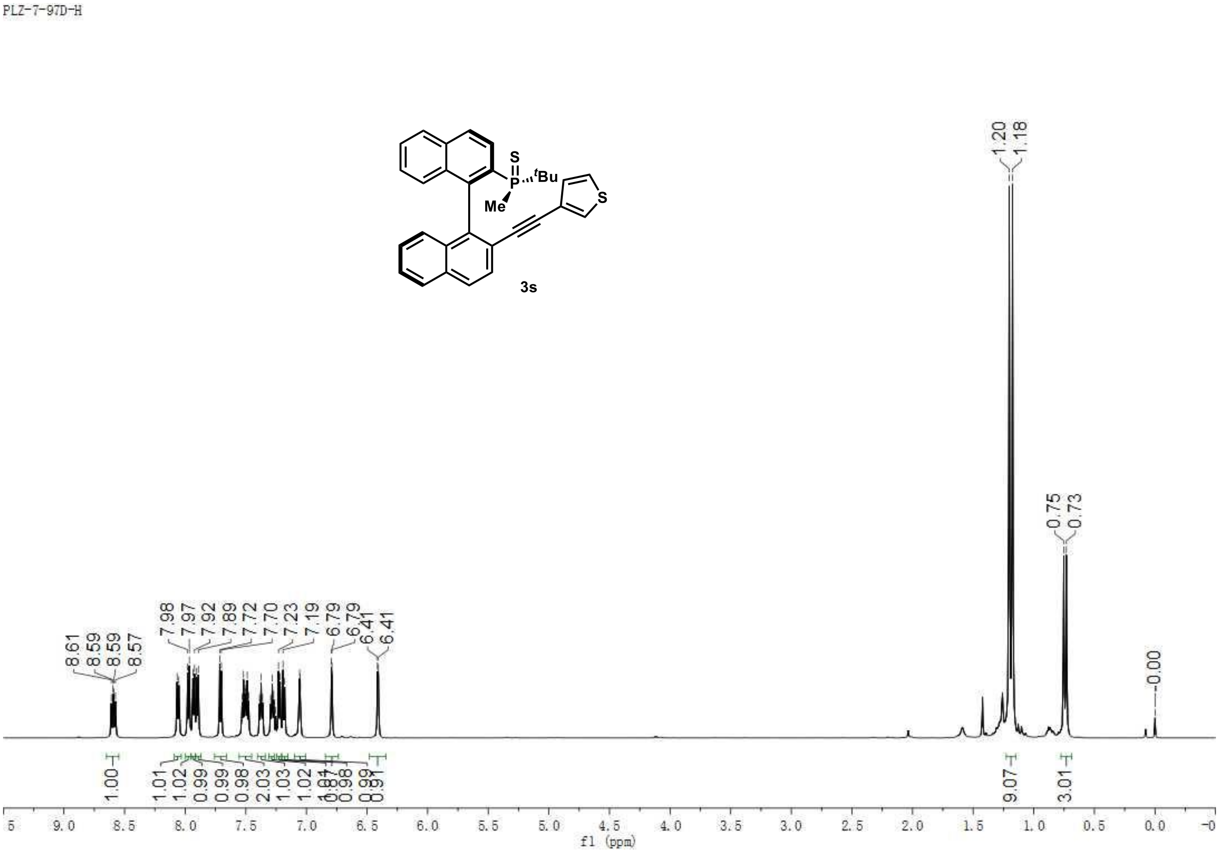

Supplementary Fig. 99. ^{1}H NMR spectrum of **3s**. The sample has been recorded in 600 MHz, $CDCl_3$ at 25 ℃.

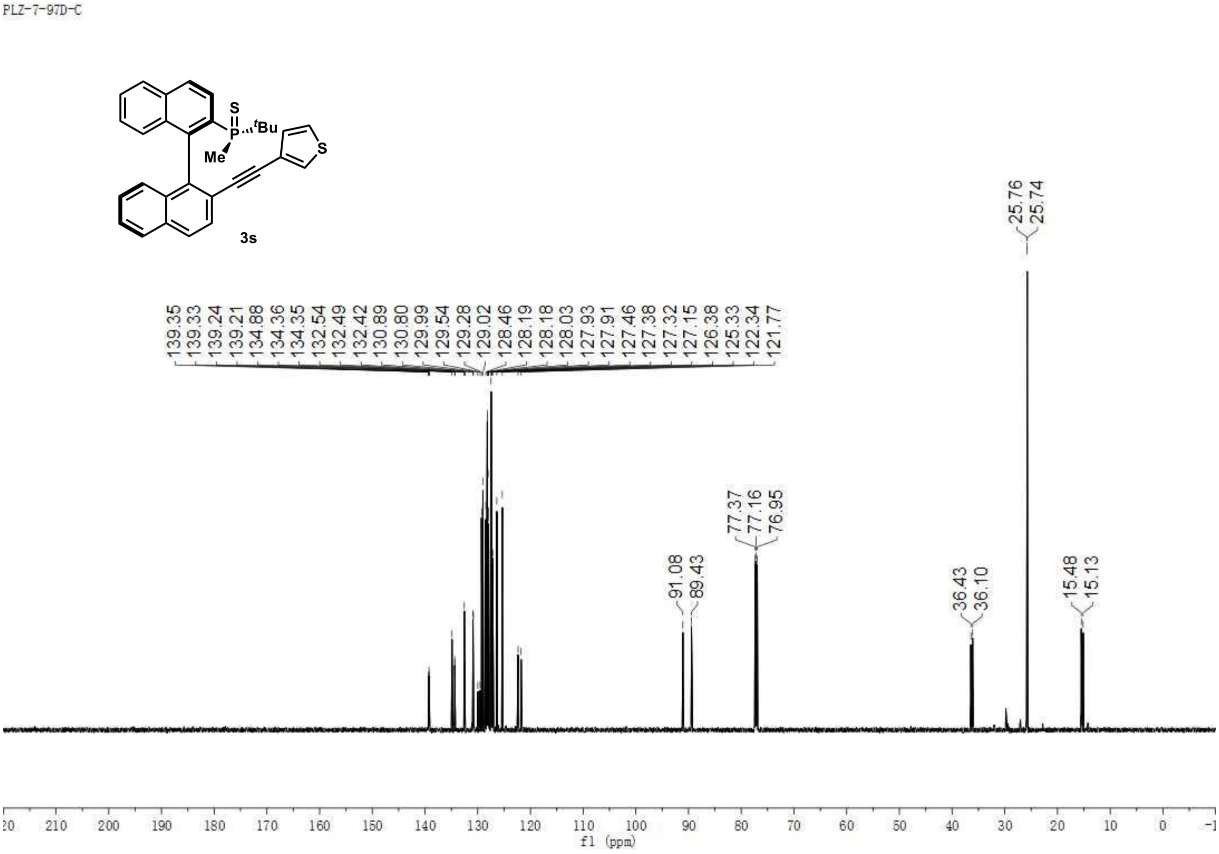

Supplementary Fig. 100.^{13}C NMR spectrum of **3s**. The sample has been recorded in 151 MHz, $CDCl_3$ at 25 ℃.

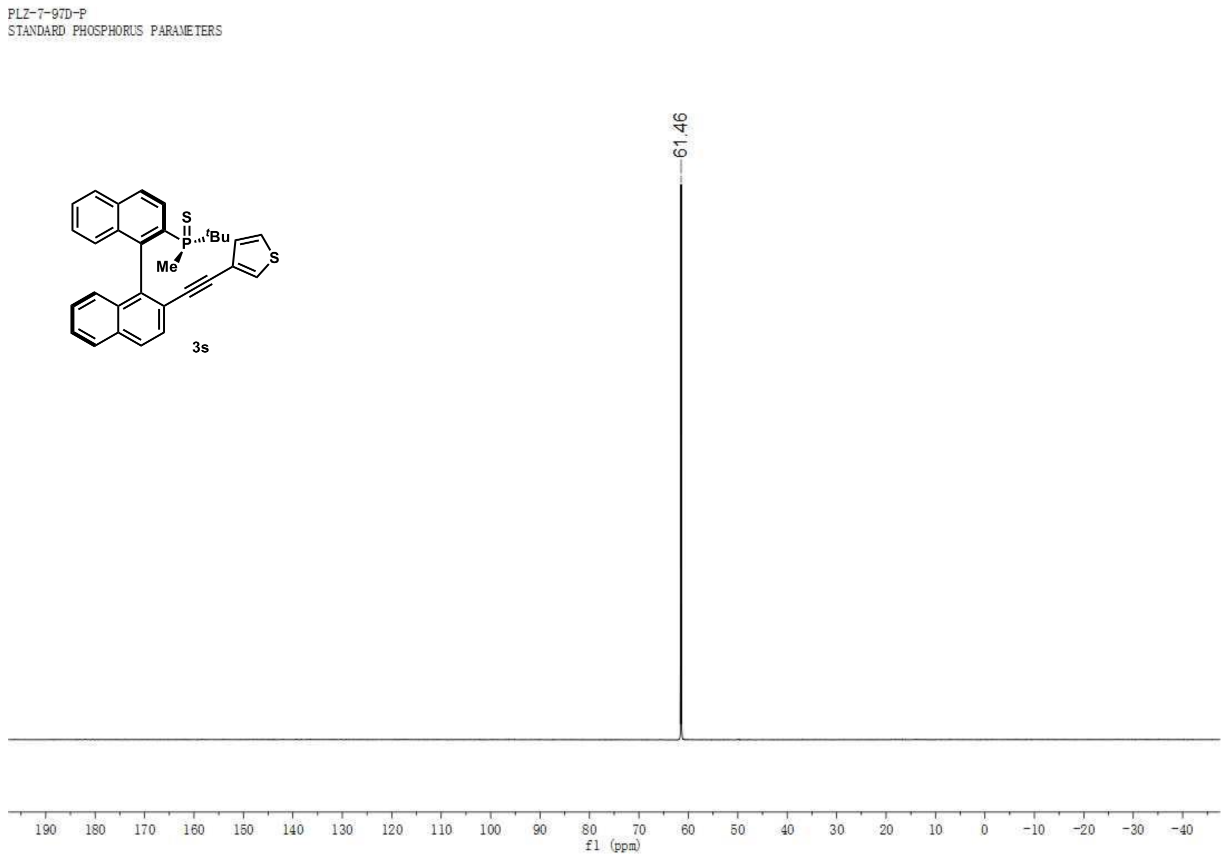

Supplementary Fig. 101.^{31}P NMR spectrum of **3s**. The sample has been recorded in 243 MHz, $CDCl_3$ at 25 ℃.

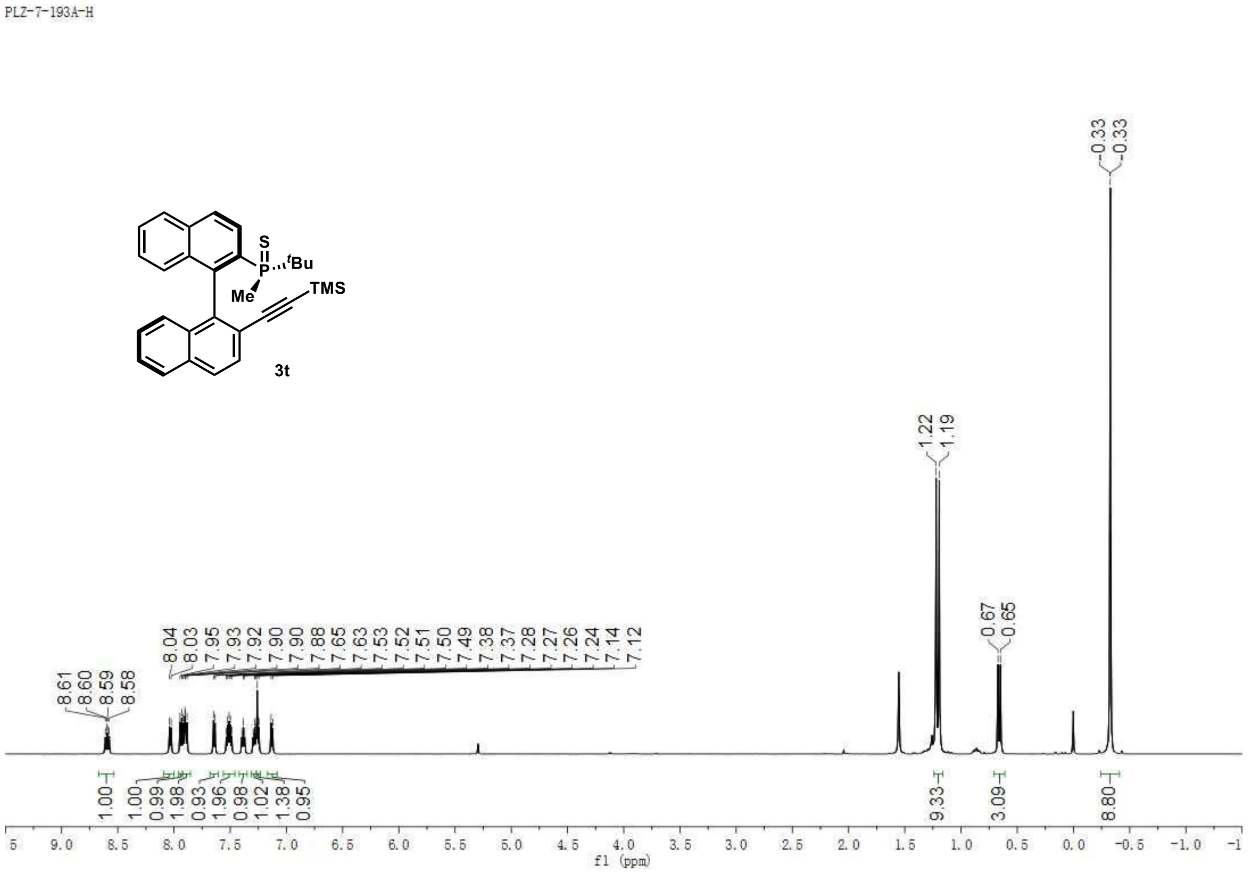

Supplementary Fig. 102. ^{1}H NMR spectrum of **3t**. The sample has been recorded in 600 MHz, $CDCl_3$ at 25 ℃.

PLZ-7-193A-C

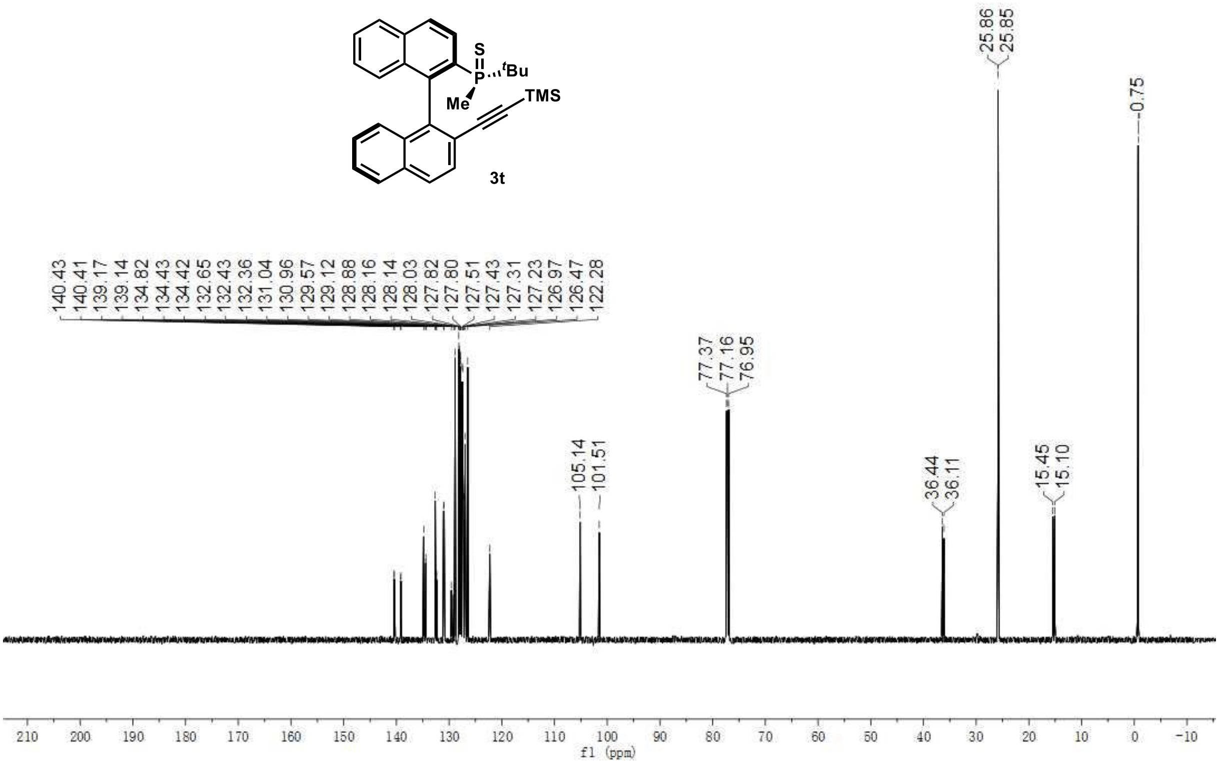

Supplementary Fig. 103.^{13}C NMR spectrum of **3t**. The sample has been recorded in 151 MHz, $CDCl_3$ at 25 ℃.

PLZ-7-193A-P
STANDARD PHOSPHORUS PARAMETERS

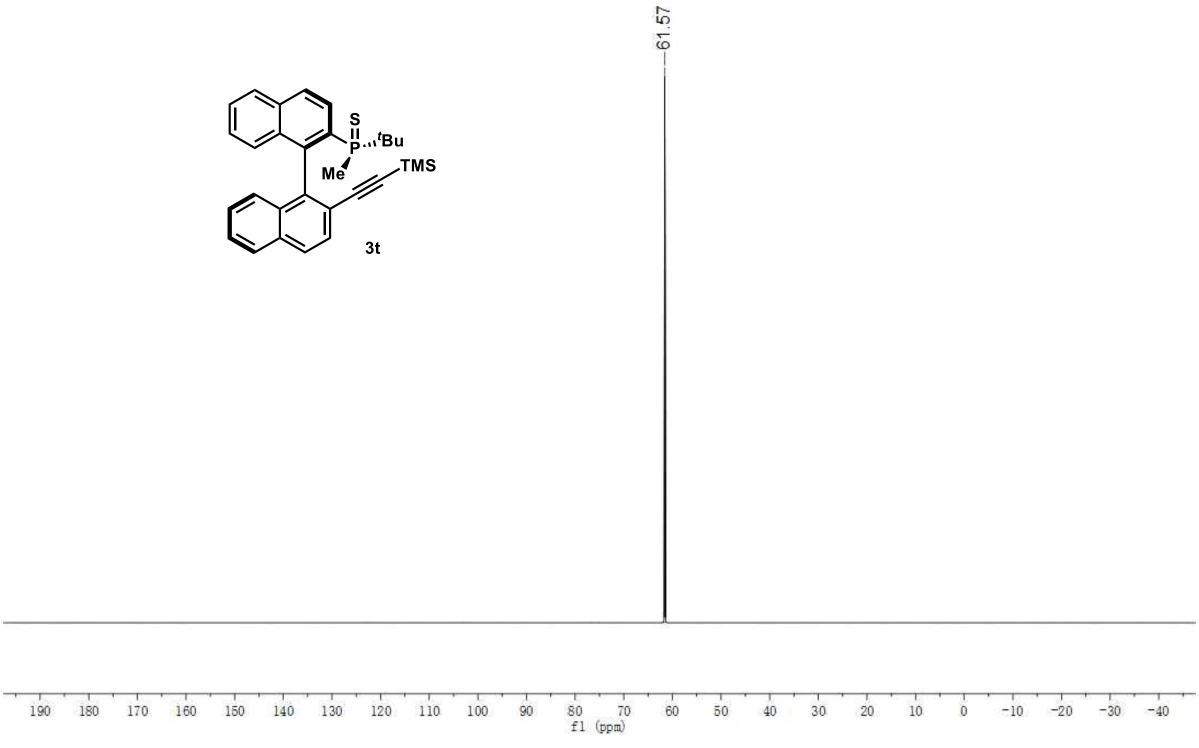

Supplementary Fig. 104.^{31}P NMR spectrum of **3t**. The sample has been recorded in 243 MHz, $CDCl_3$ at 25 ℃.

PLZ-7-126B-H

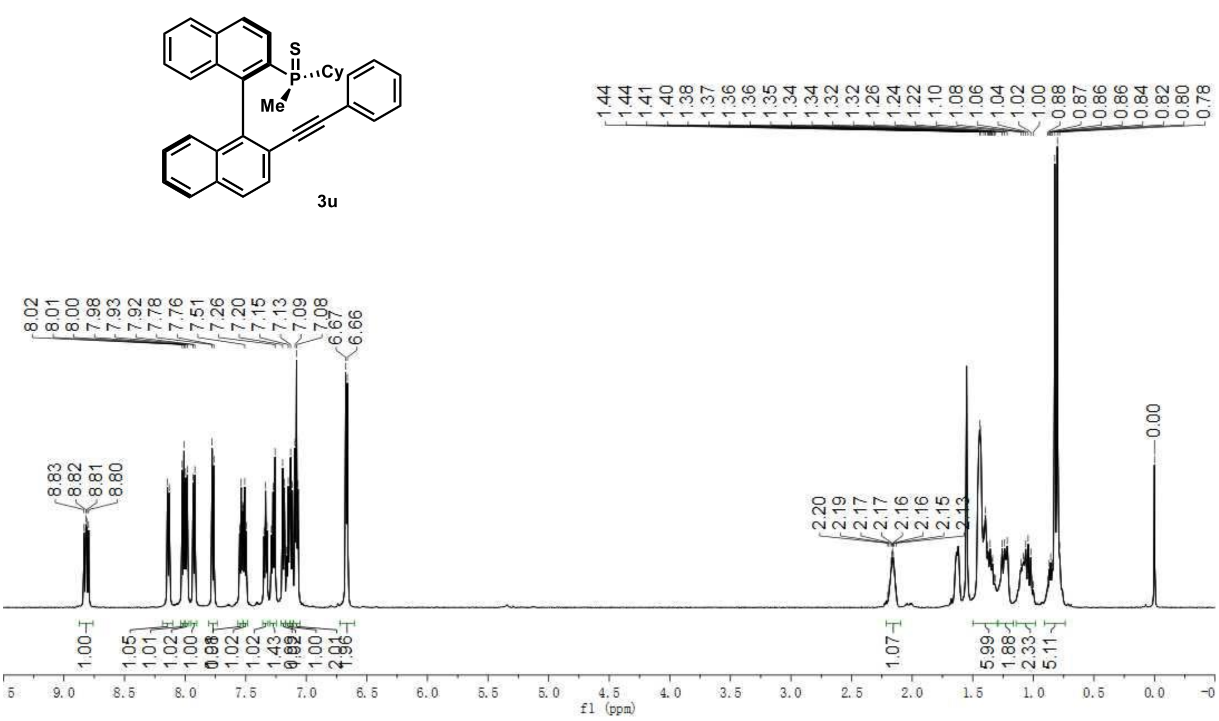

Supplementary Fig. 105. ^{1}H NMR spectrum of **3u**. The sample has been recorded in 600 MHz, $CDCl_3$ at 25 ℃.

PLZ-7-126B-C

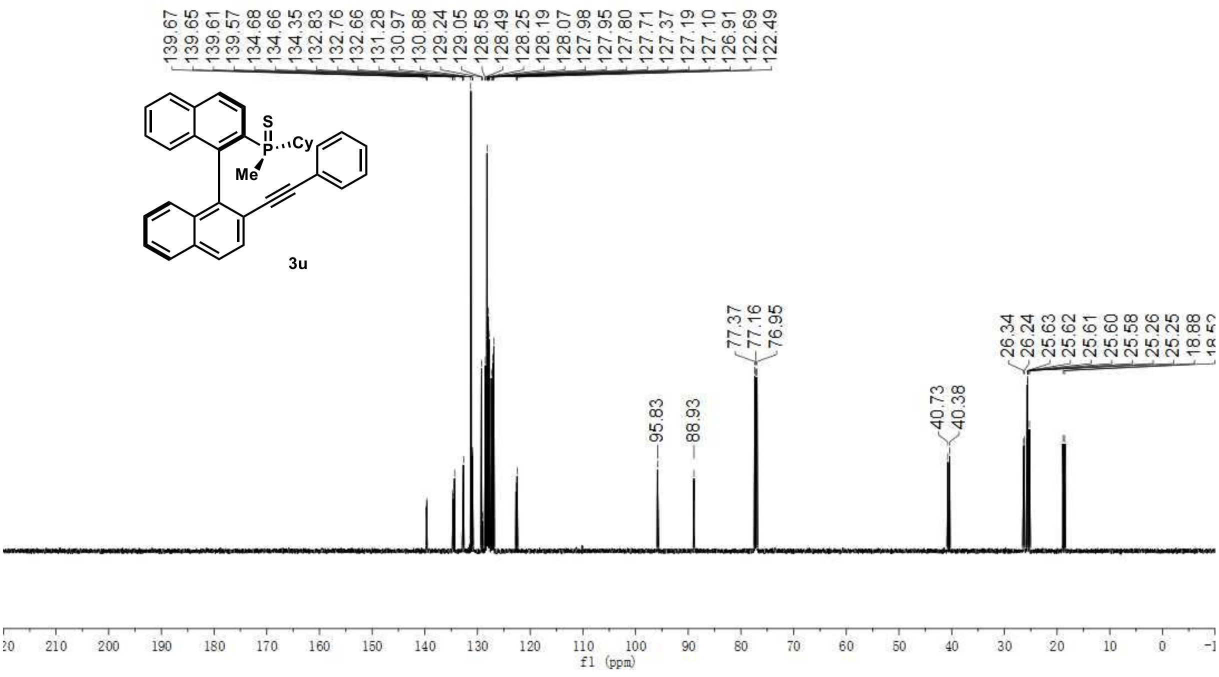

Supplementary Fig. 106.^{13}C NMR spectrum of **3u**. The sample has been recorded in 151 MHz, $CDCl_3$ at 25 ℃.

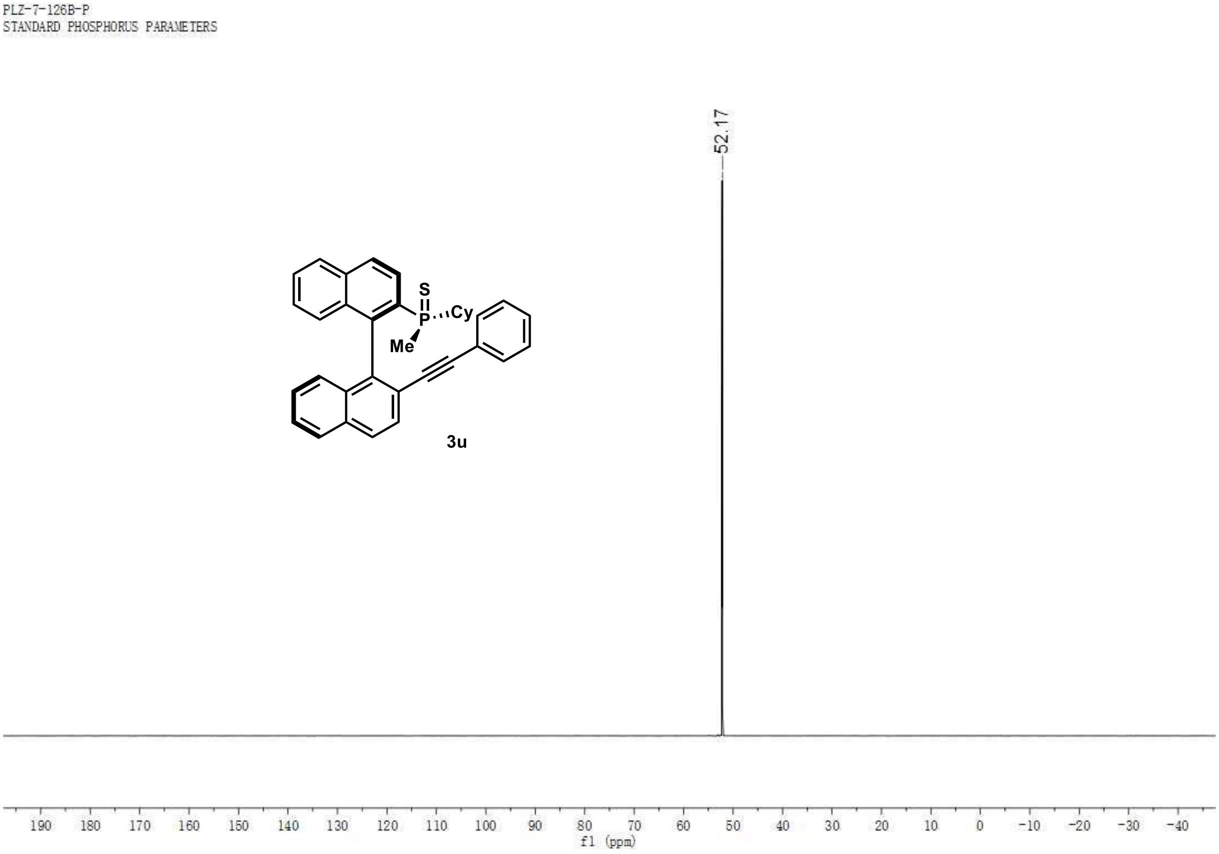

Supplementary Fig. 107.^{31}P NMR spectrum of **3u**. The sample has been recorded in 243 MHz, $CDCl_3$ at 25 ℃.

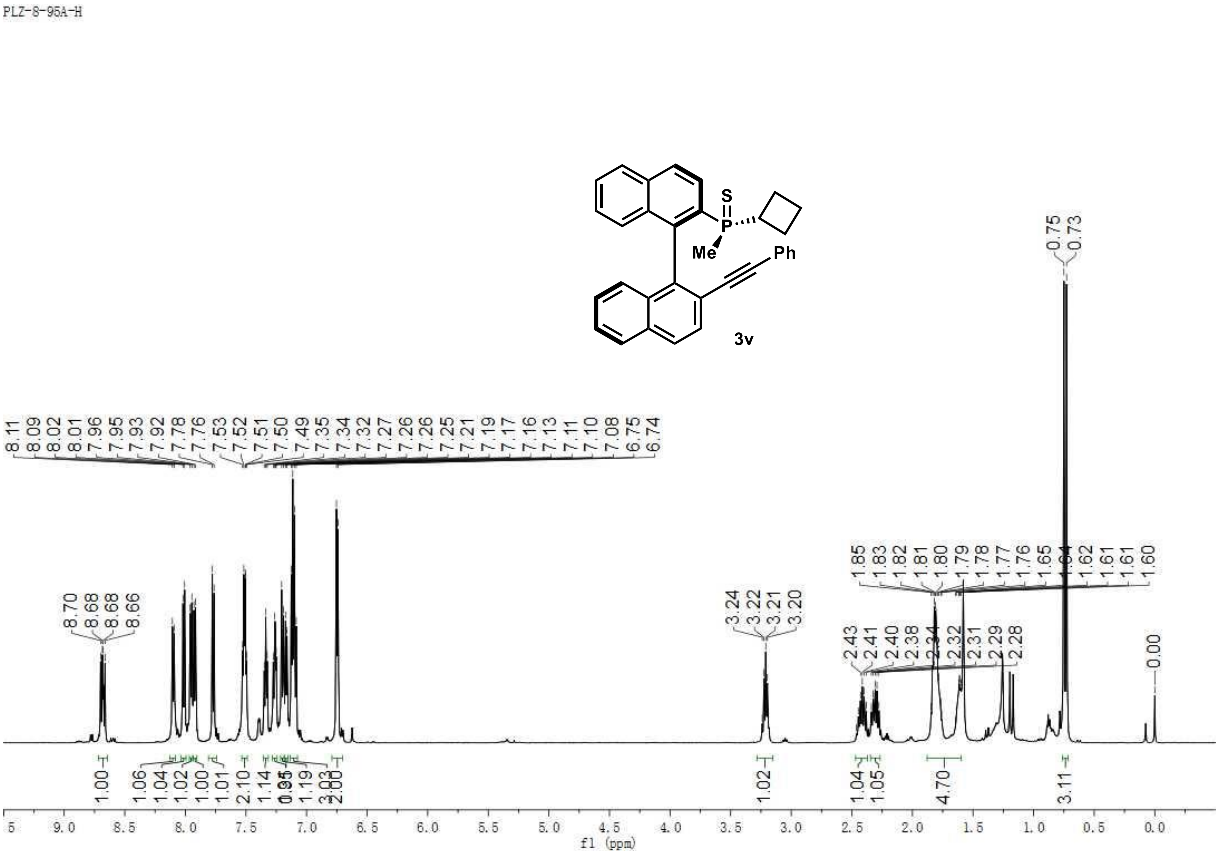

Supplementary Fig. 108. ^{1}H NMR spectrum of **3v**. The sample has been recorded in 600 MHz, $CDCl_3$ at 25 ℃.

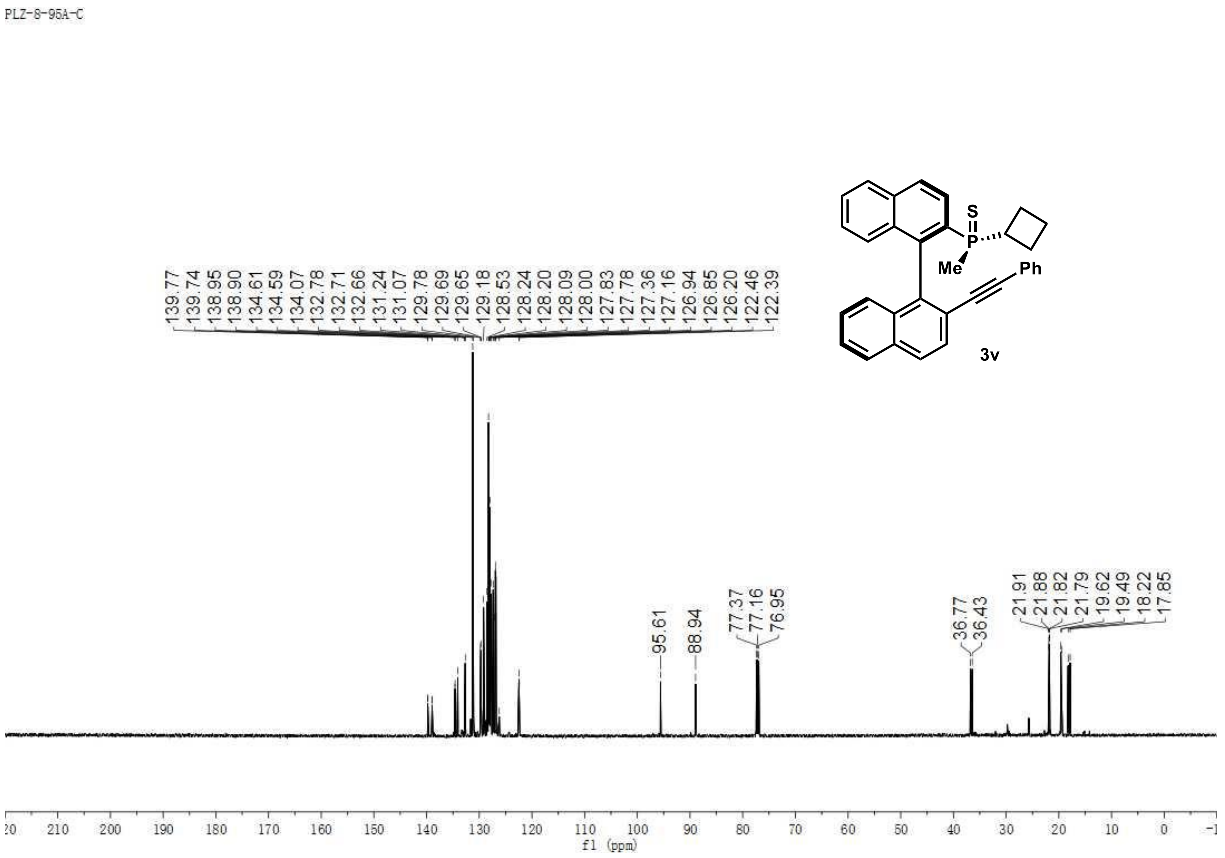

Supplementary Fig. 109.^{13}C NMR spectrum of **3v**. The sample has been recorded in 151 MHz, $CDCl_3$ at 25 ℃.

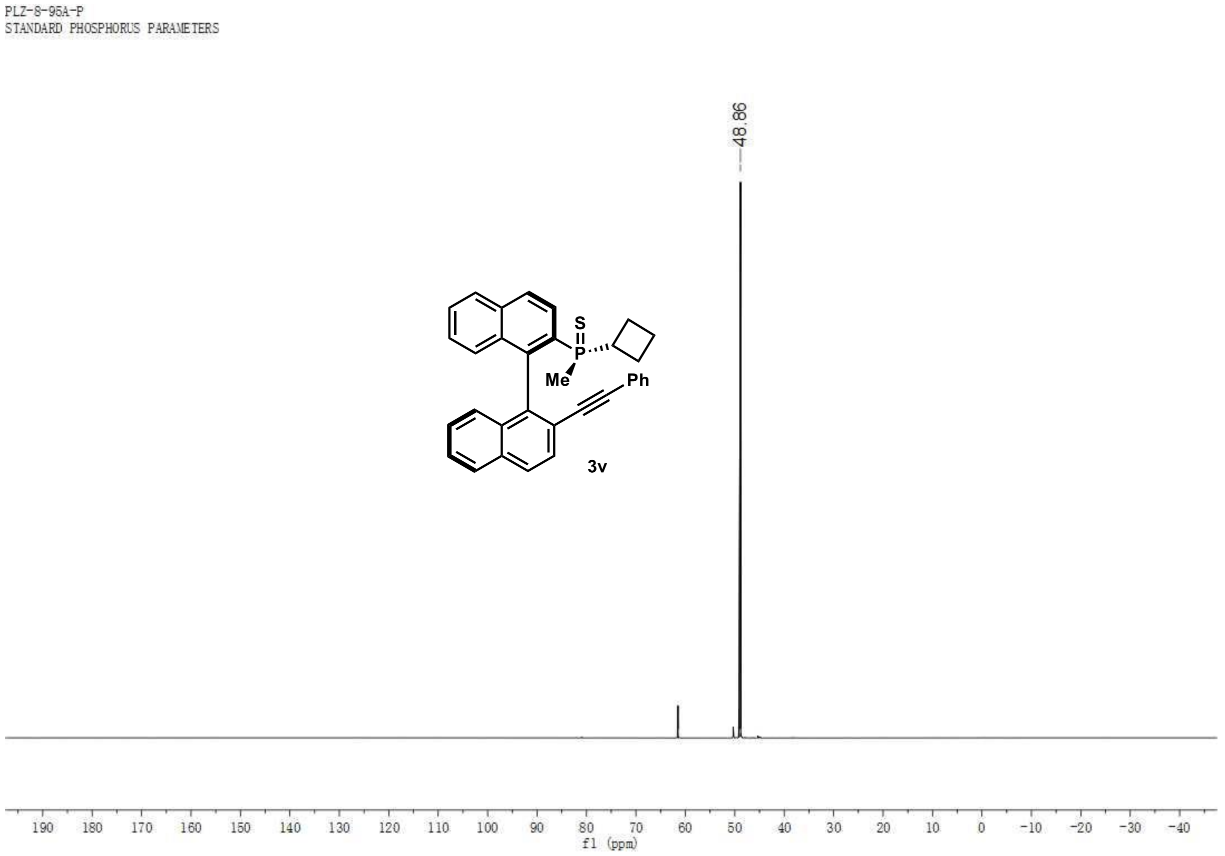

Supplementary Fig. 110.^{31}P NMR spectrum of **3v**. The sample has been recorded in 243 MHz, $CDCl_3$ at 25 ℃.

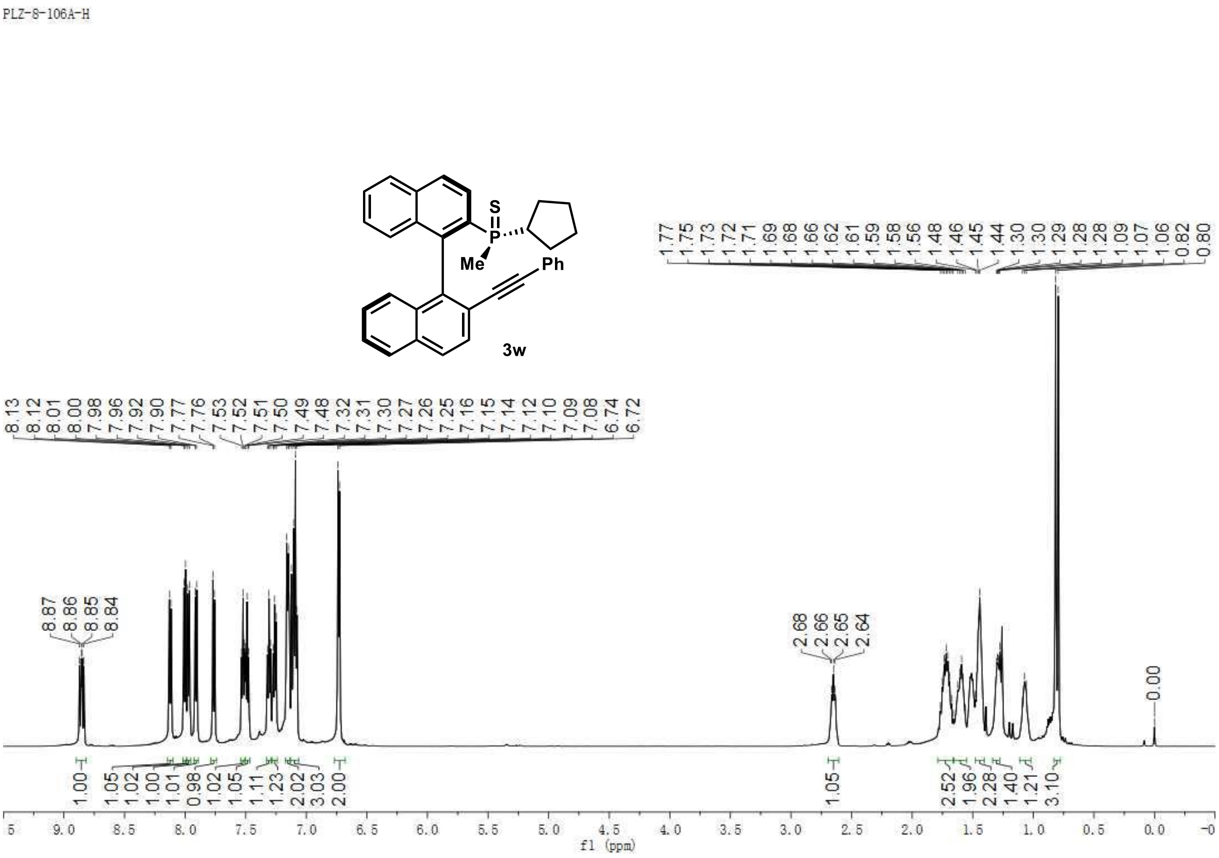

Supplementary Fig. 111. ^{1}H NMR spectrum of **3w**. The sample has been recorded in 600 MHz, $CDCl_3$ at 25 ℃.

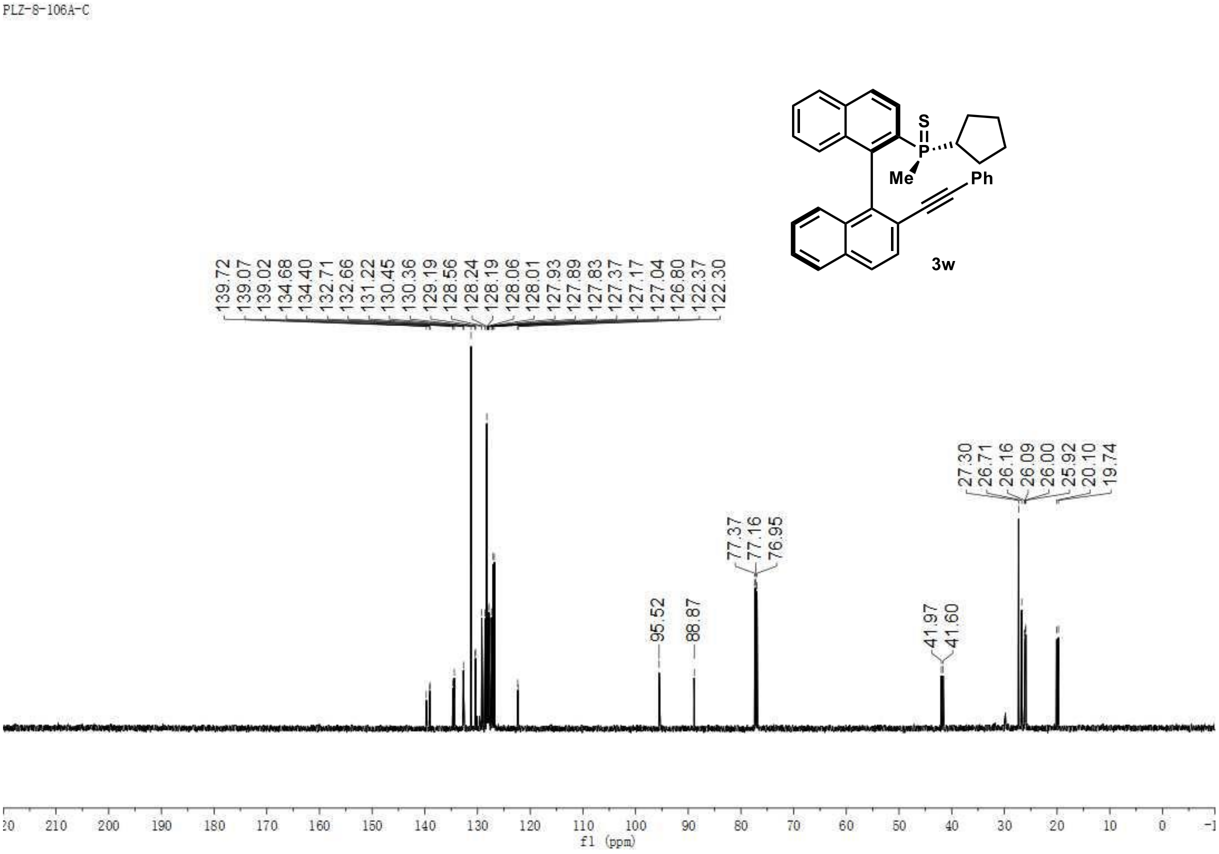

Supplementary Fig. 112.^{13}C NMR spectrum of **3w**. The sample has been recorded in 151 MHz, $CDCl_3$ at 25 ℃.

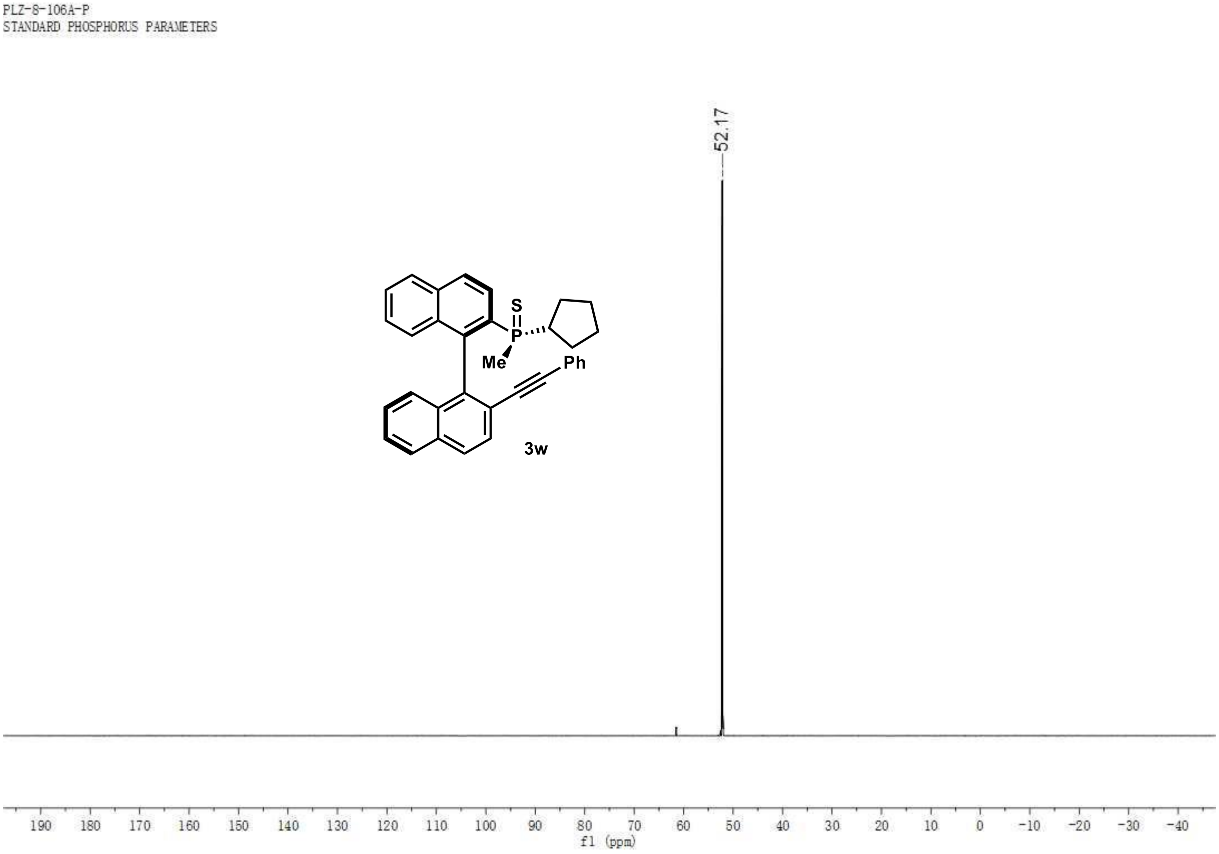

Supplementary Fig. 113.^{31}P NMR spectrum of **3w**. The sample has been recorded in 243 MHz, $CDCl_3$ at 25 ℃.

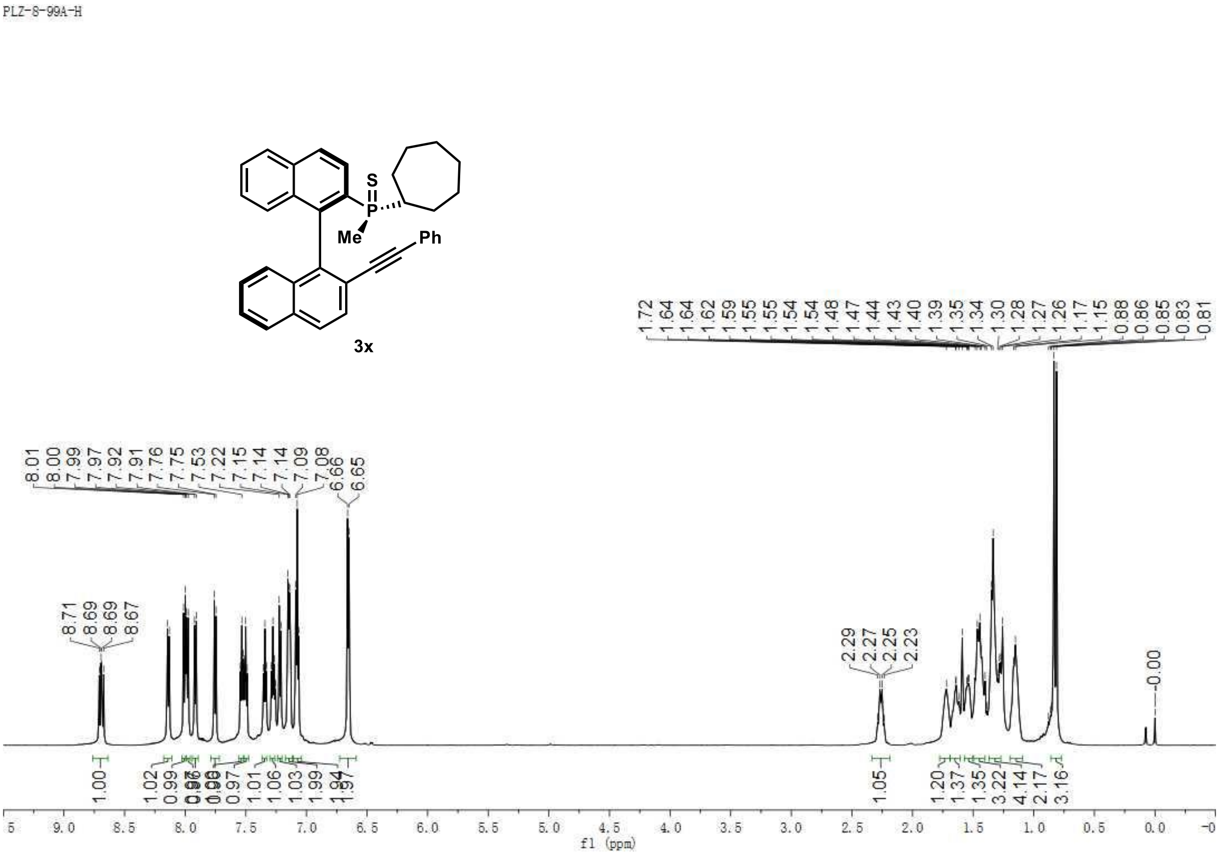

Supplementary Fig. 114. ^{1}H NMR spectrum of **3x**. The sample has been recorded in 600 MHz, $CDCl_3$ at 25 ℃.

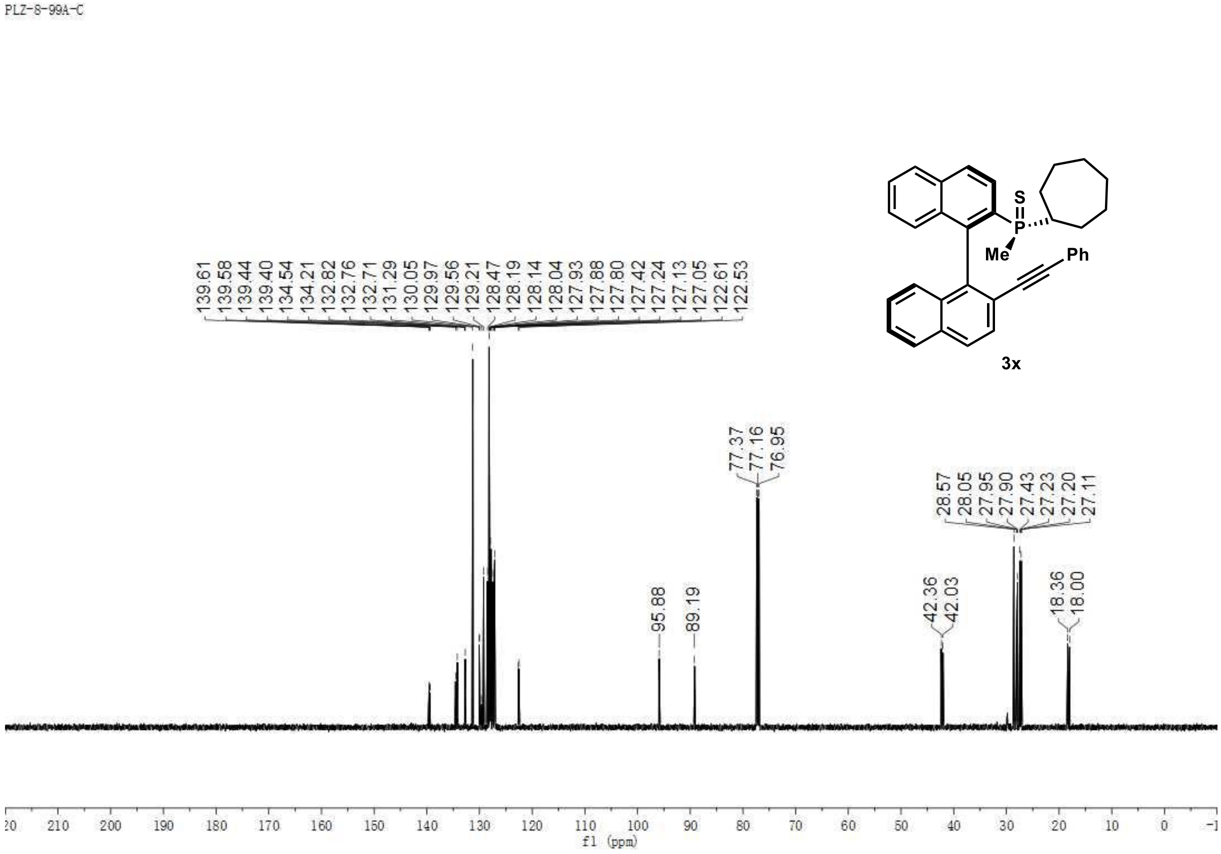

Supplementary Fig. 115.^{13}C NMR spectrum of **3x**. The sample has been recorded in 151 MHz, $CDCl_3$ at 25 ℃.

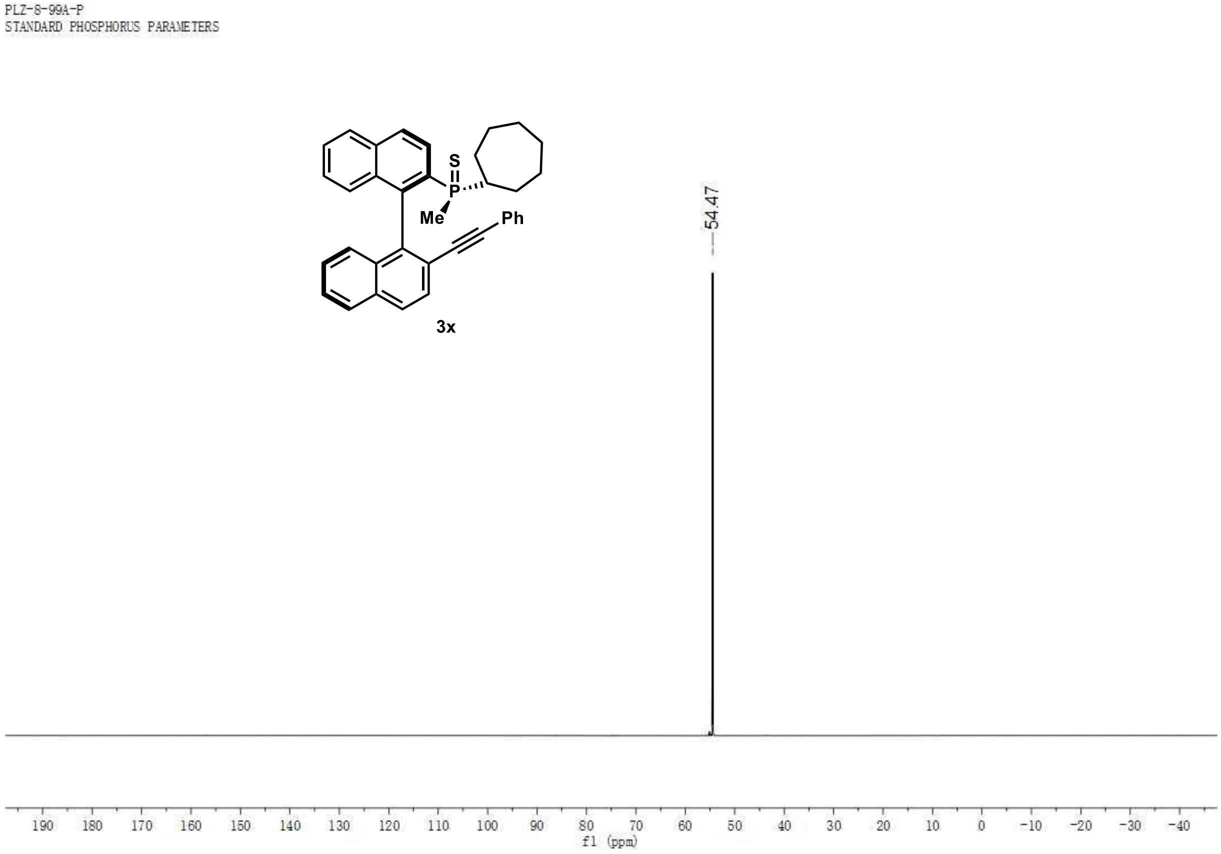

Supplementary Fig. 116.^{31}P NMR spectrum of **3x**. The sample has been recorded in 243 MHz, $CDCl_3$ at 25 ℃.

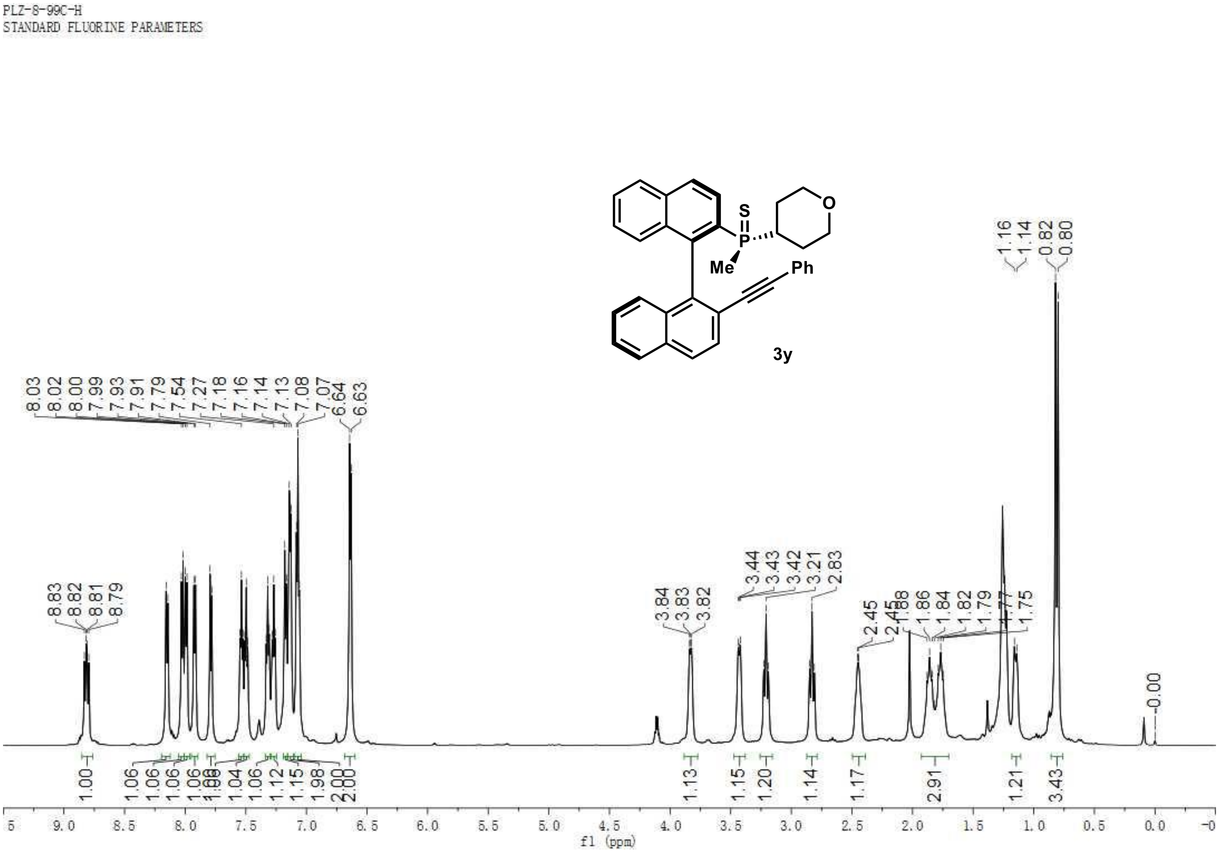

Supplementary Fig. 117. ^{1}H NMR spectrum of **3y**. The sample has been recorded in 600 MHz, $CDCl_3$ at 25 ℃.

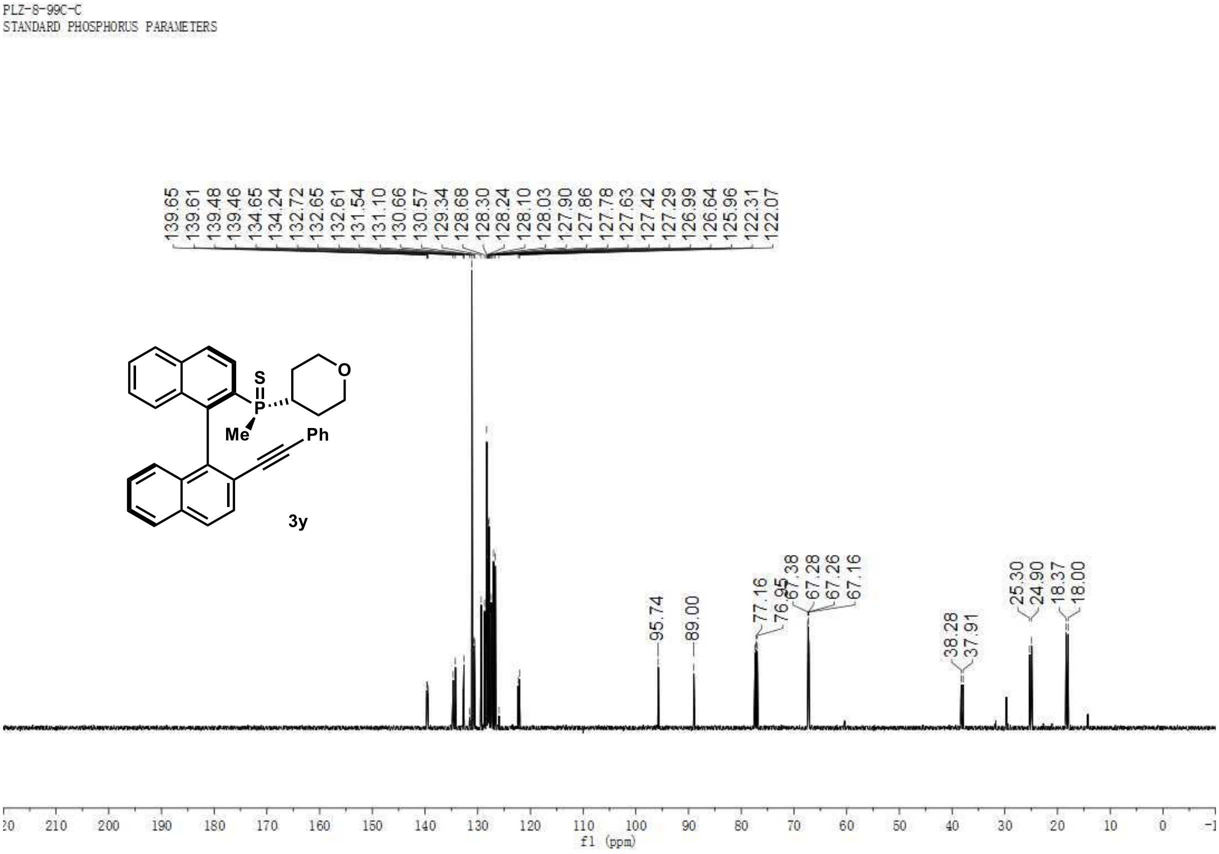

Supplementary Fig. 118. ^{13}C NMR spectrum of **3y**. The sample has been recorded in 151 MHz, $CDCl_3$ at 25 ℃.

PLZ-8-99C-P
STANDARD PHOSPHORUS PARAMETERS

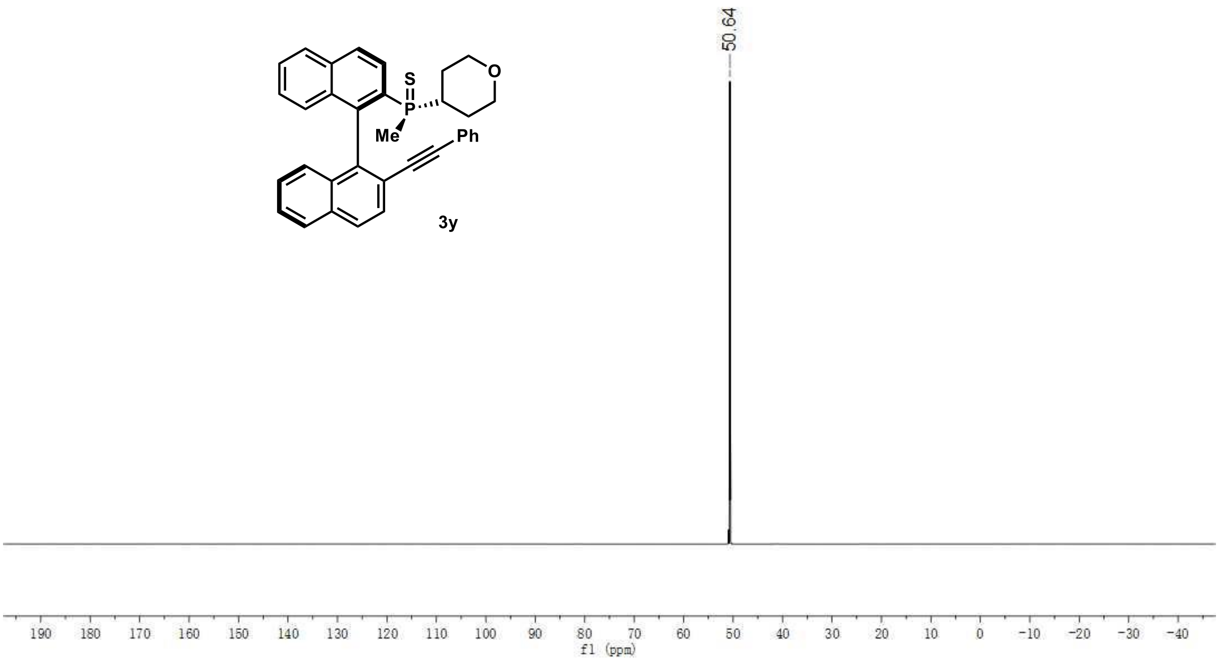

Supplementary Fig. 119.^{31}P NMR spectrum of **3y**. The sample has been recorded in 243 MHz, $CDCl_3$ at 25 ℃.

PLZ-8-95B-H

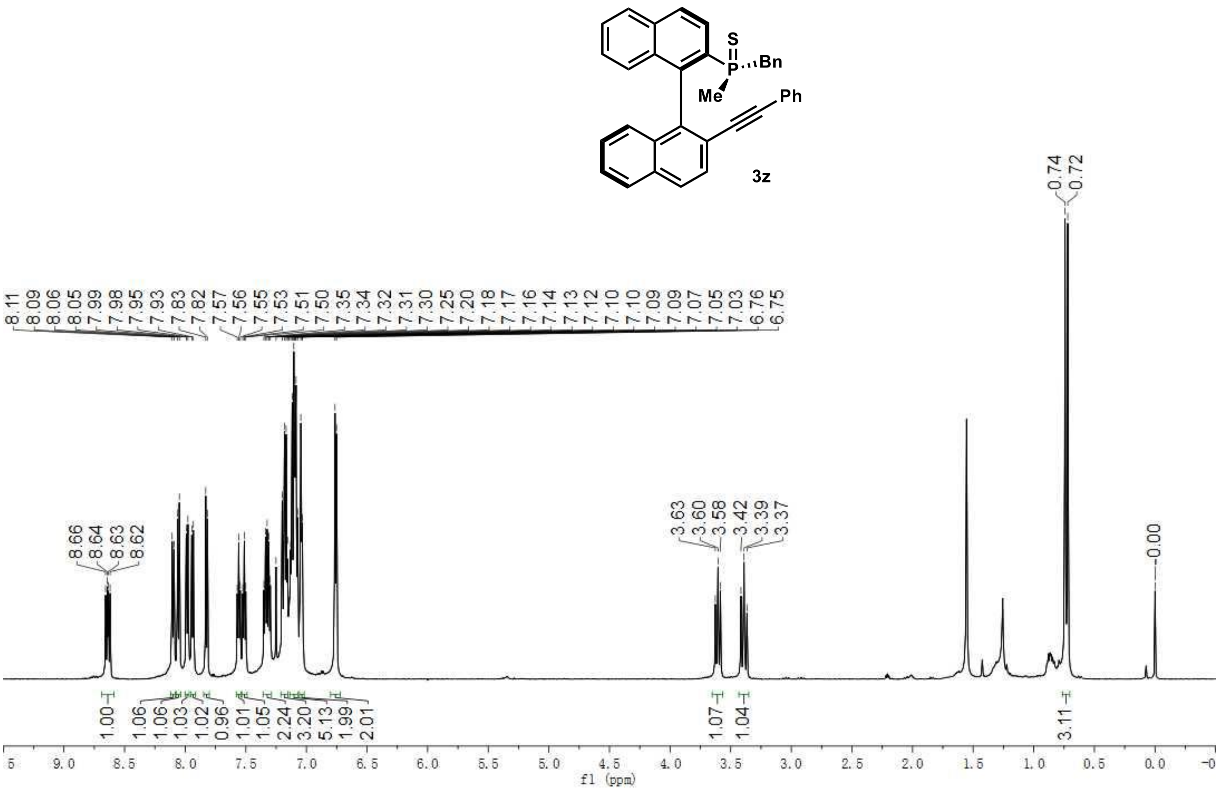

Supplementary Fig. 120. ^{1}H NMR spectrum of **3z**. The sample has been recorded in 600 MHz, $CDCl_3$ at 25 ℃.

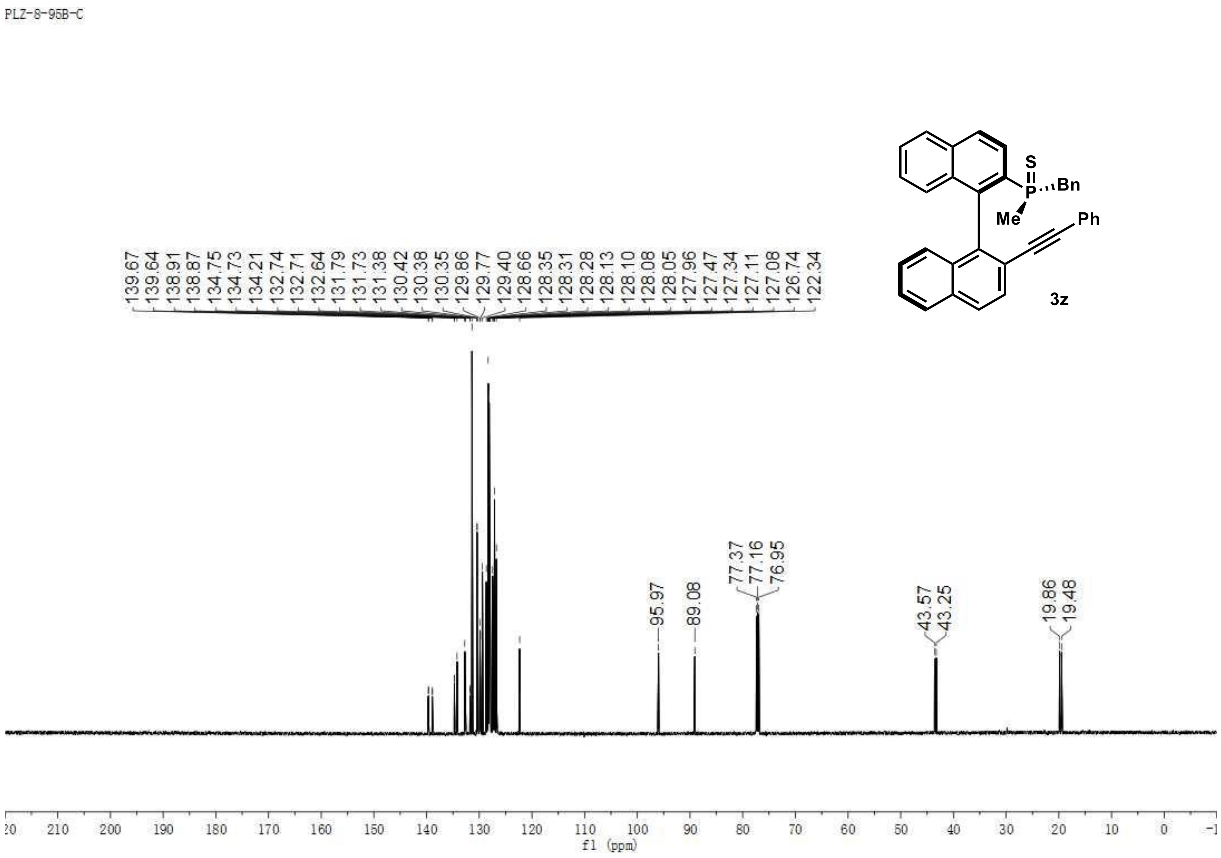

Supplementary Fig. 121.^{13}C NMR spectrum of **3z**. The sample has been recorded in 151 MHz, $CDCl_3$ at 25 ℃.

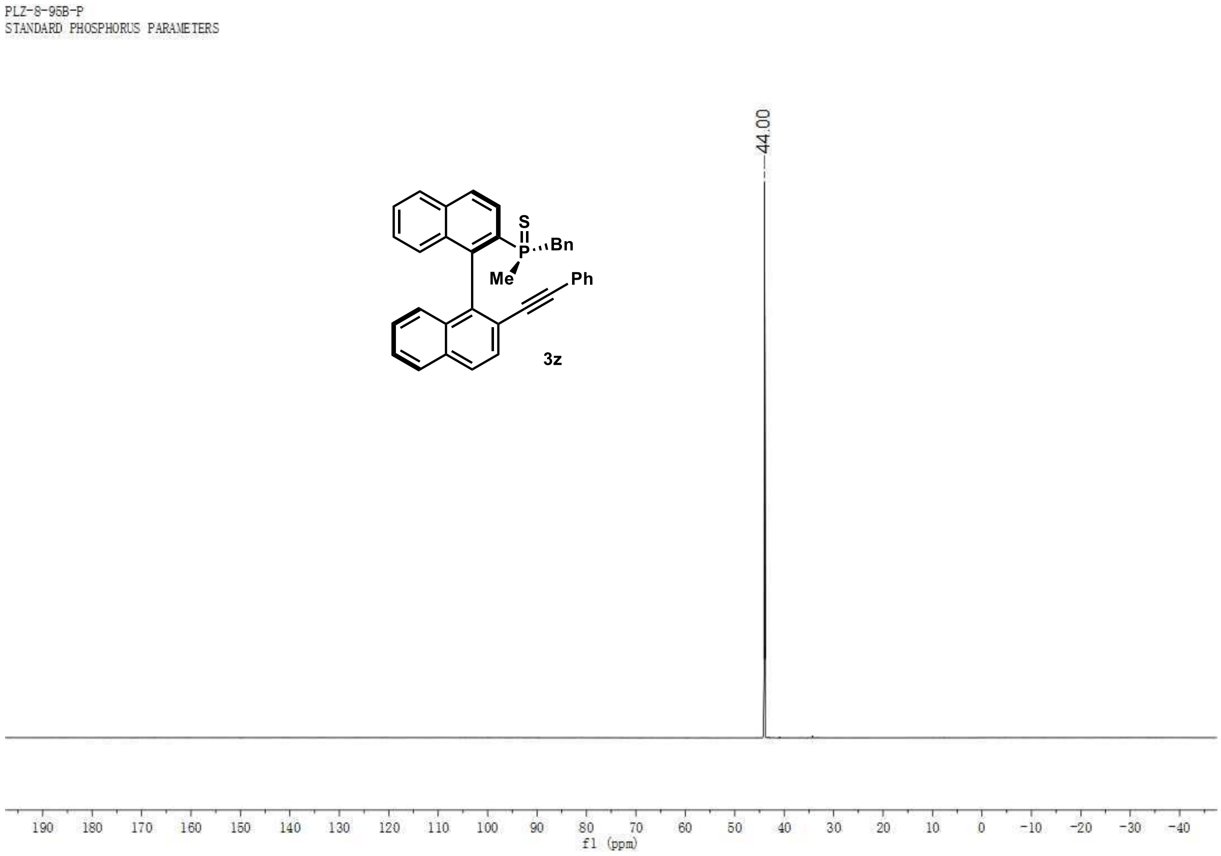

Supplementary Fig. 122.^{31}P NMR spectrum of **3z**. The sample has been recorded in 243 MHz, $CDCl_3$ at 25 ℃.

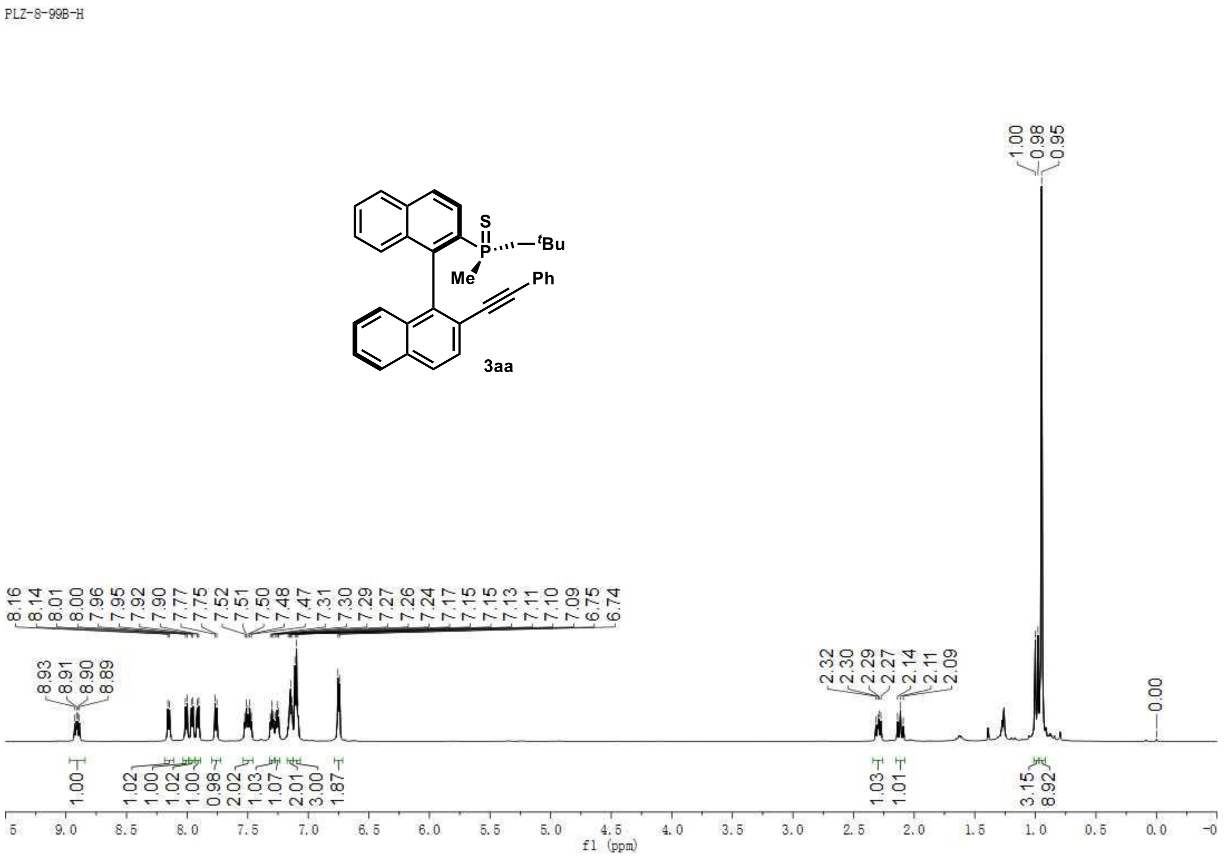

Supplementary Fig. 123. ^{1}H NMR spectrum of **3aa**. The sample has been recorded in 600 MHz, $CDCl_3$ at 25 ℃.

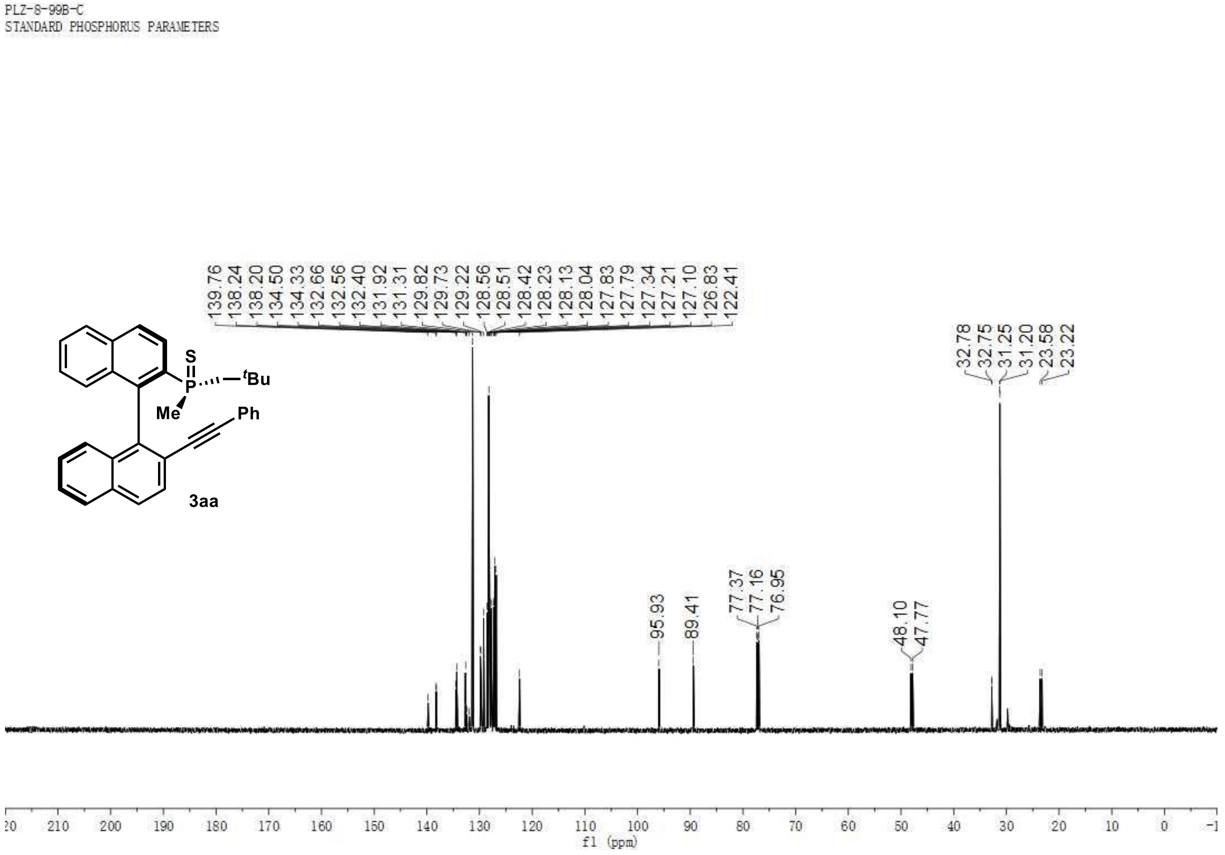

Supplementary Fig. 124.^{13}C NMR spectrum of **3aa**. The sample has been recorded in 151 MHz, $CDCl_3$ at 25 ℃.

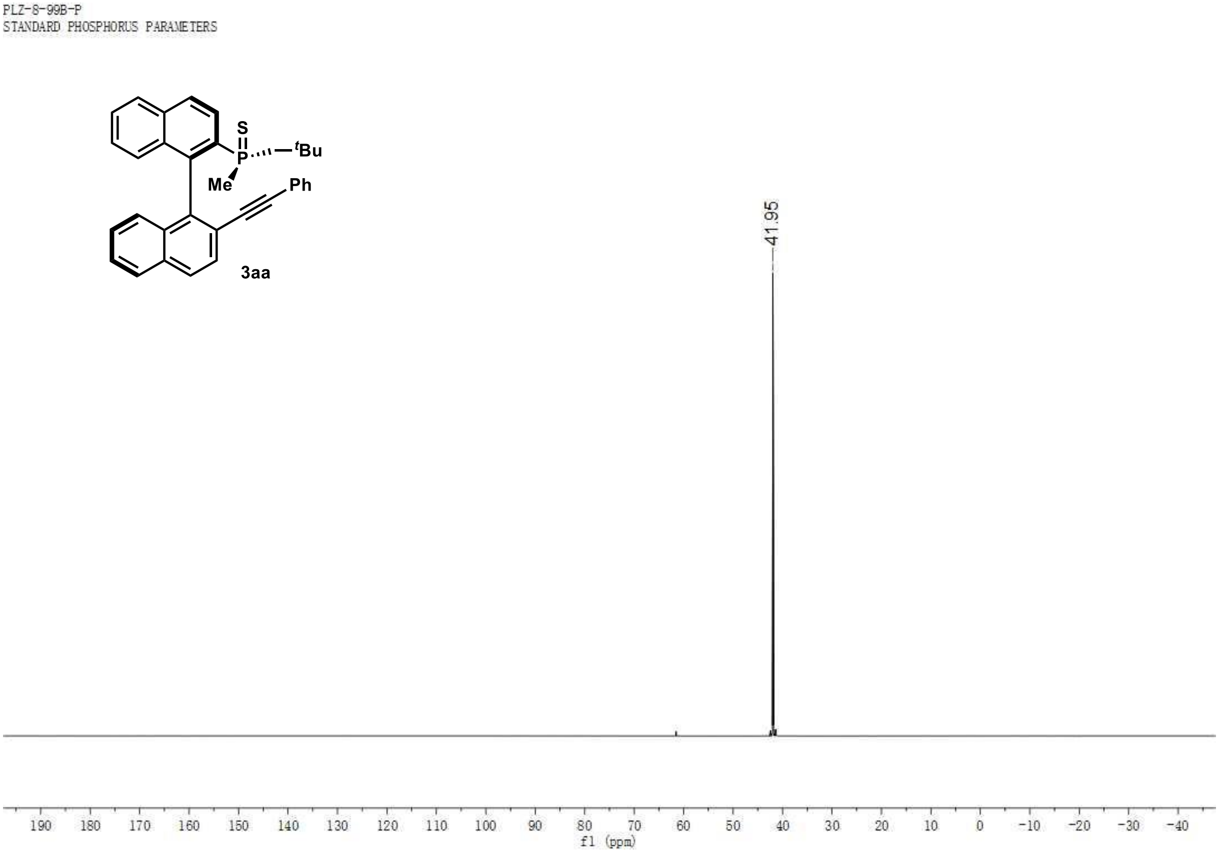

Supplementary Fig. 125.^{31}P NMR spectrum of **3aa**. The sample has been recorded in 243 MHz, $CDCl_3$ at 25 ℃.

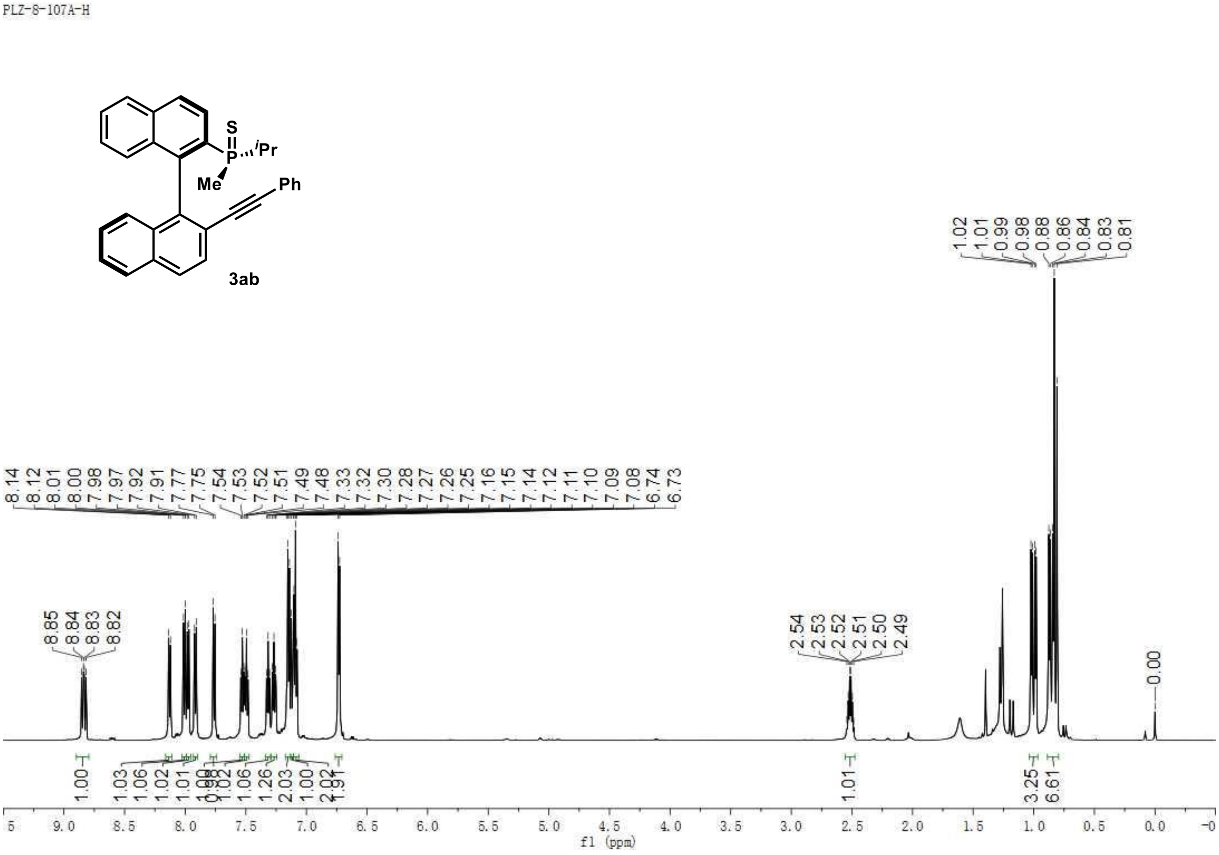

Supplementary Fig. 126. ^{1}H NMR spectrum of **3ab**. The sample has been recorded in 600 MHz, $CDCl_3$ at 25 ℃.

PLZ-8-107A-C

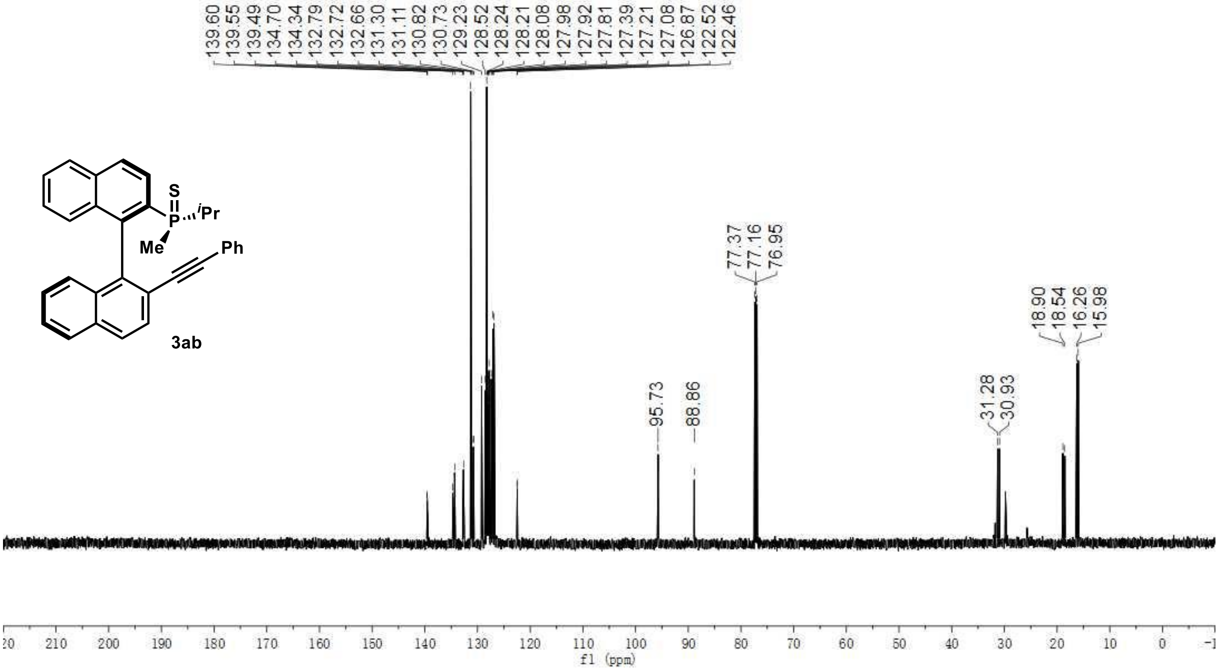

Supplementary Fig. 127.^{13}C NMR spectrum of **3ab**. The sample has been recorded in 151 MHz, $CDCl_3$ at 25 ℃.

PLZ-8-107A-P
STANDARD PHOSPHORUS PARAMETERS

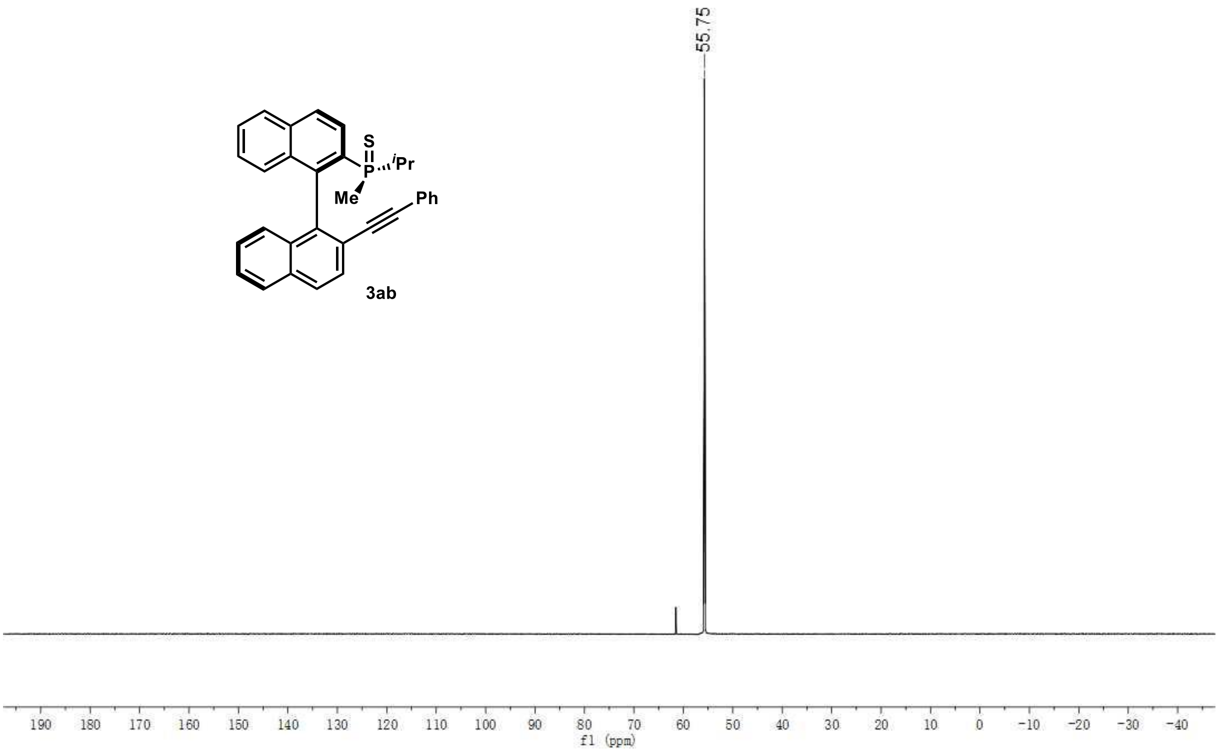

Supplementary Fig. 128.^{31}P NMR spectrum of **3ab**. The sample has been recorded in 243 MHz, $CDCl_3$ at 25 ℃.

PLZ-8-107B-H

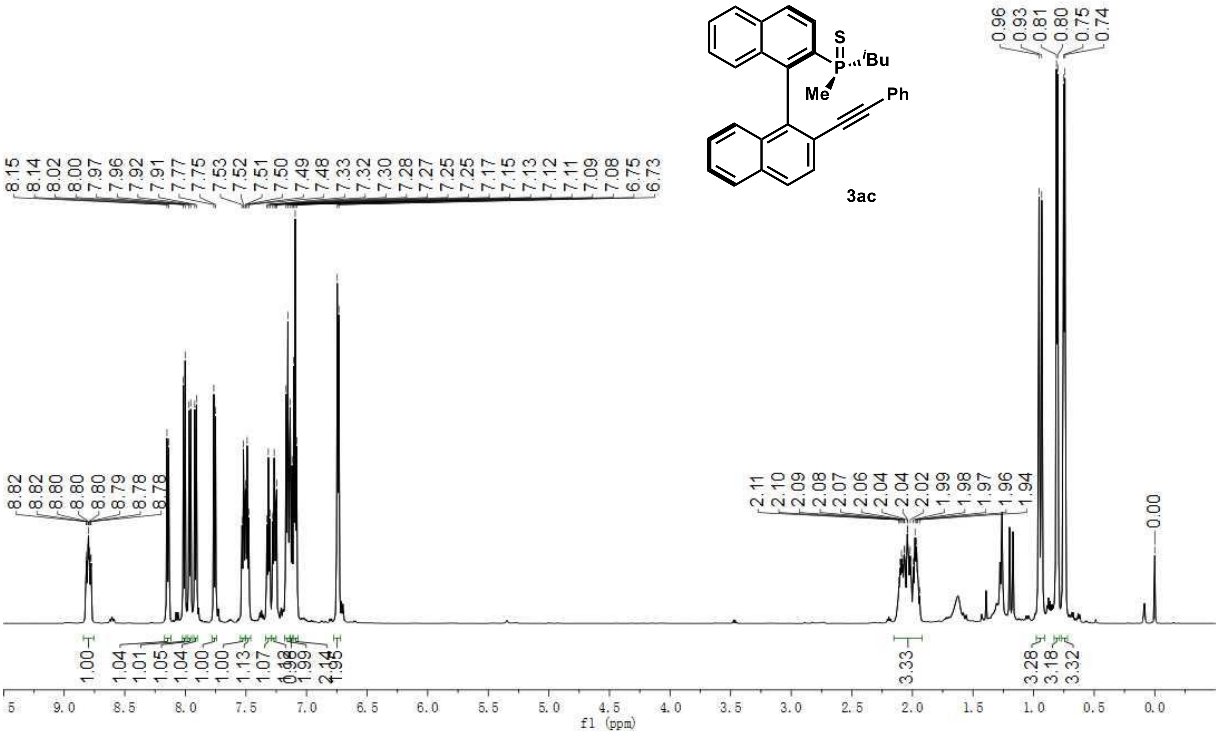

Supplementary Fig. 129. ^{1}H NMR spectrum of **3ac**. The sample has been recorded in 600 MHz, $CDCl_3$ at 25 ℃.

PLZ-8-107B-C

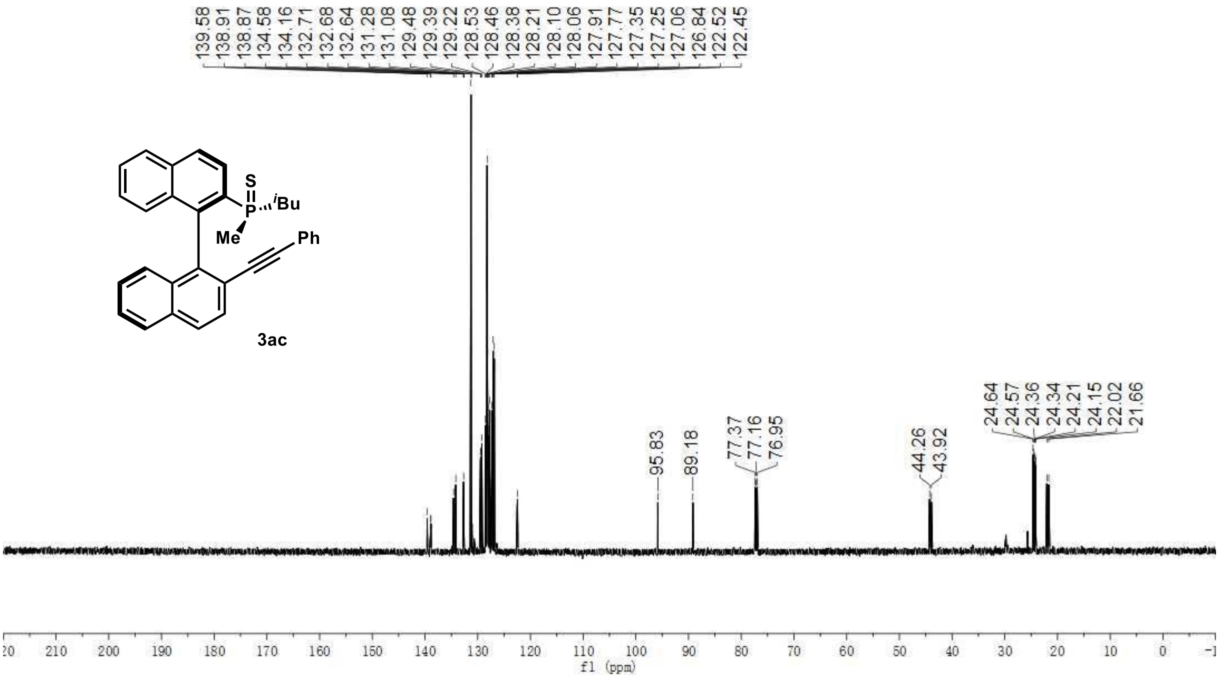

Supplementary Fig. 130.^{13}C NMR spectrum of **3ac**. The sample has been recorded in 151 MHz, $CDCl_3$ at 25 ℃.

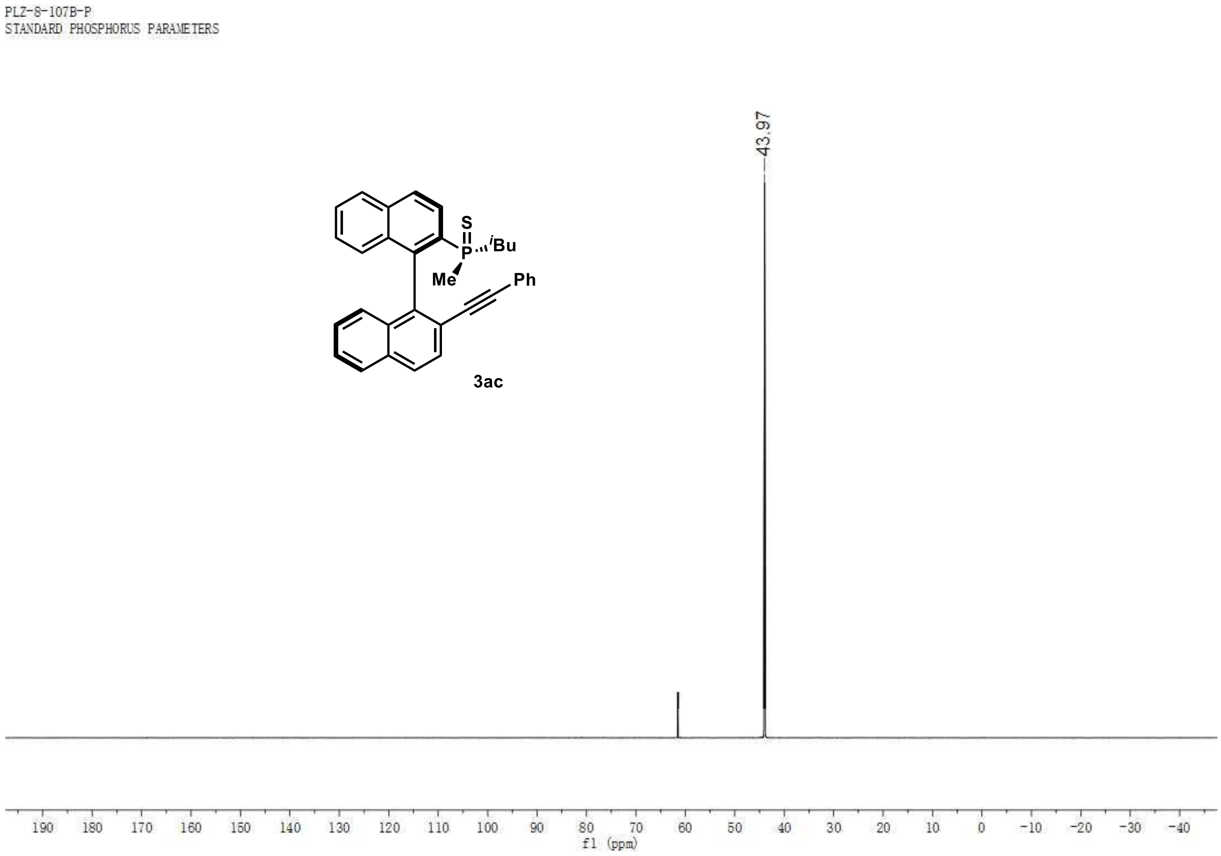

Supplementary Fig. 131.^{31}P NMR spectrum of **3ac**. The sample has been recorded in 243 MHz, $CDCl_3$ at 25 ℃.

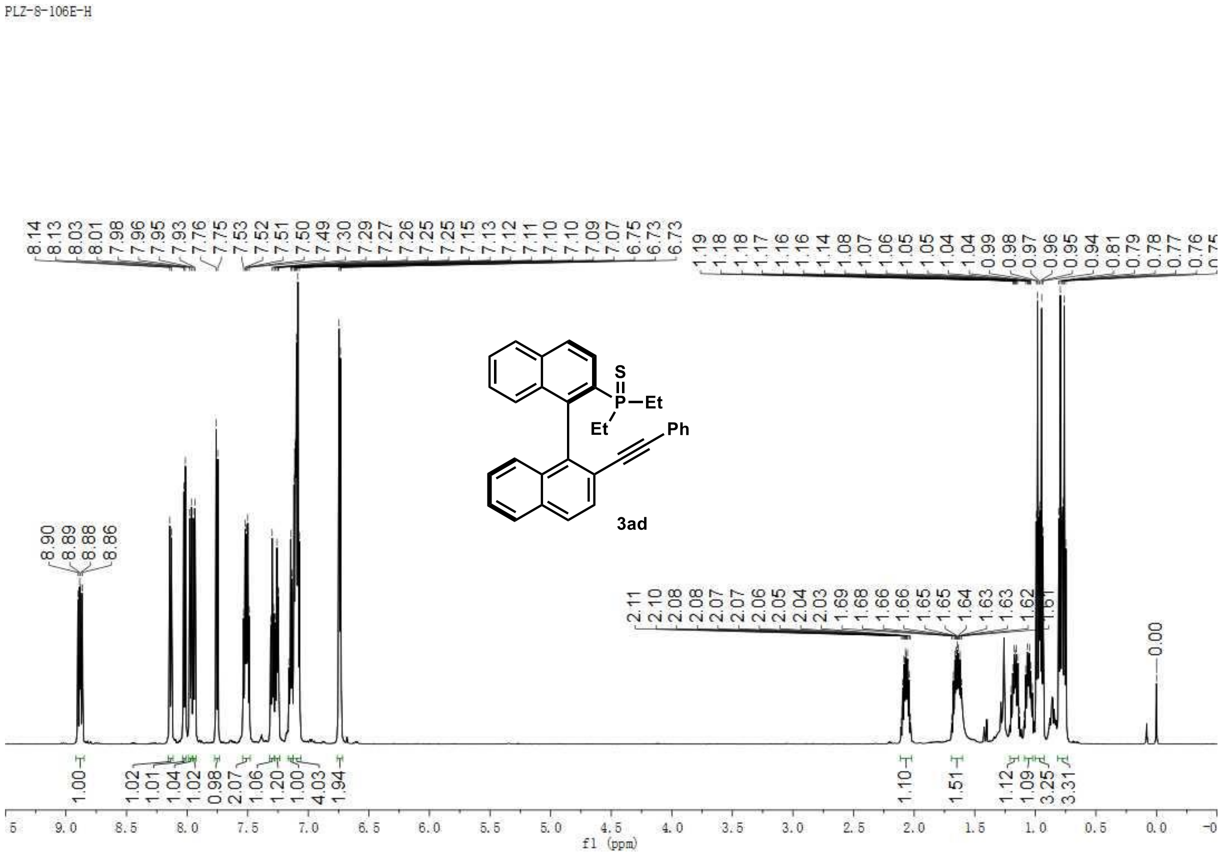

Supplementary Fig. 132. ^{1}H NMR spectrum of **3ad**. The sample has been recorded in 600 MHz, $CDCl_3$ at 25 ℃.

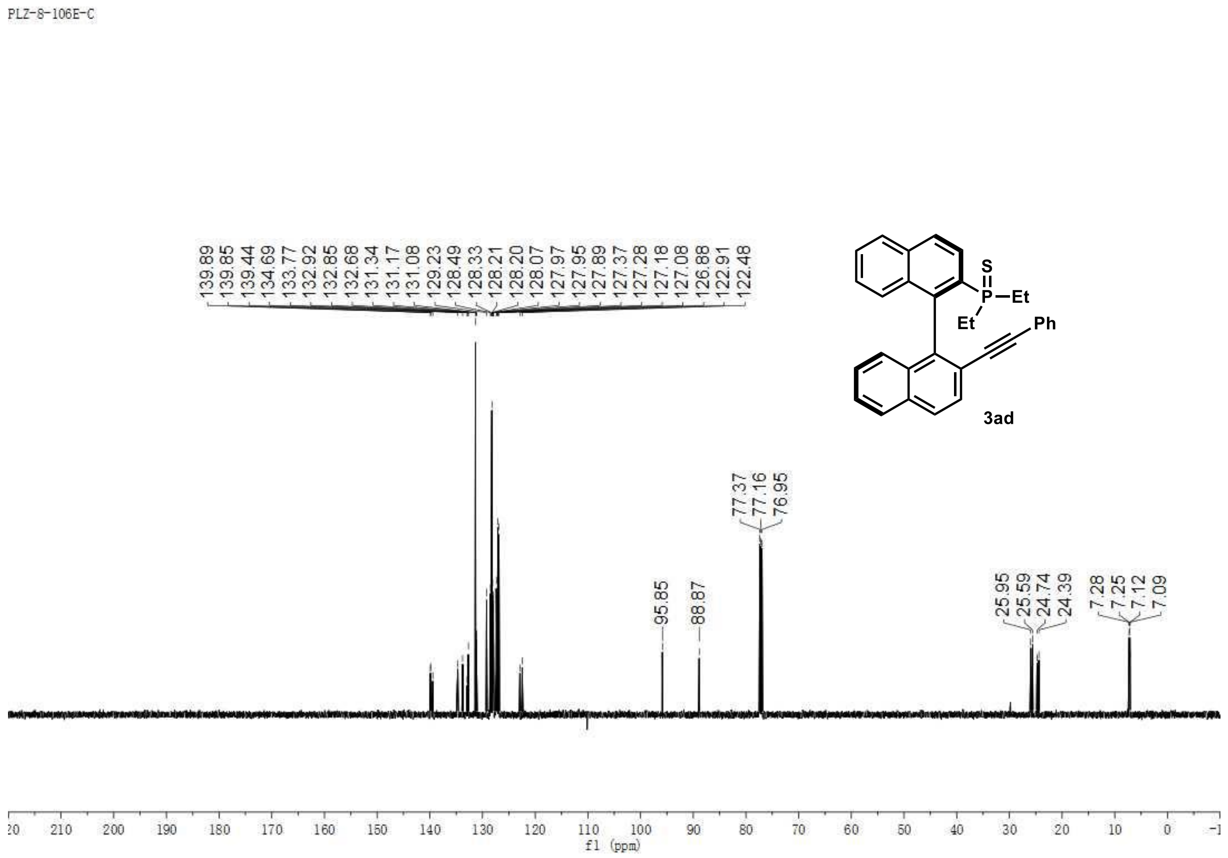

Supplementary Fig. 133.^{13}C NMR spectrum of **3ad**. The sample has been recorded in 151 MHz, $CDCl_3$ at 25 ℃.

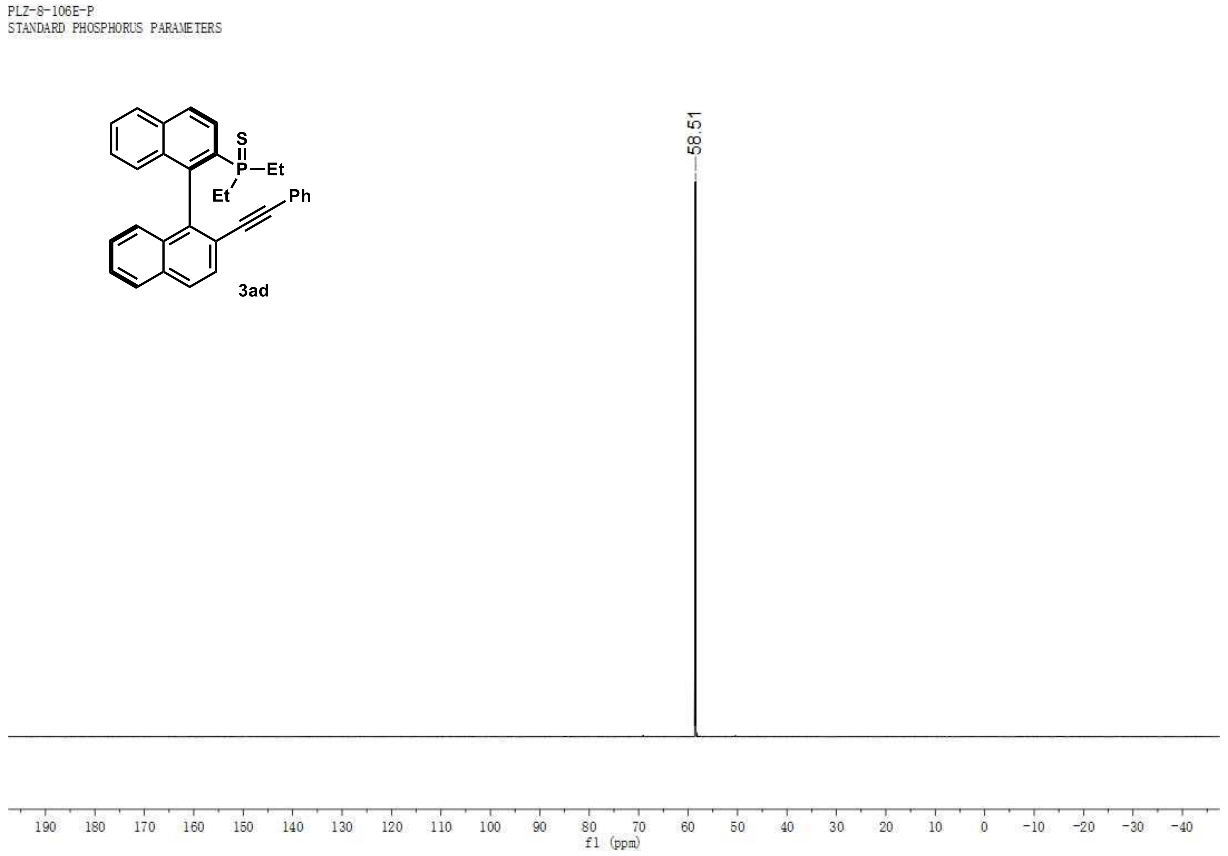

Supplementary Fig. 134.^{31}P NMR spectrum of **3ad**. The sample has been recorded in 243 MHz, $CDCl_3$ at 25 ℃.

PLZ-7-125C-H

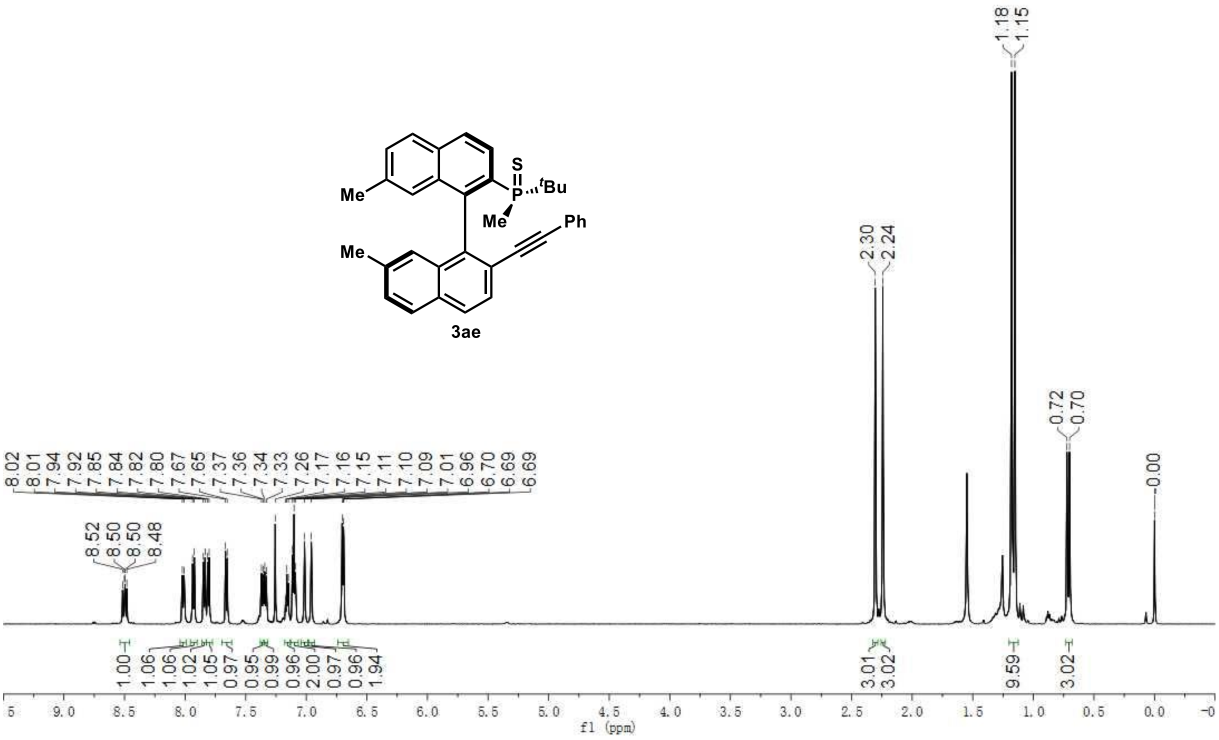

Supplementary Fig. 135. ^{1}H NMR spectrum of **3ae**. The sample has been recorded in 600 MHz, $CDCl_3$ at 25 ℃.

PLZ-7-108B-C

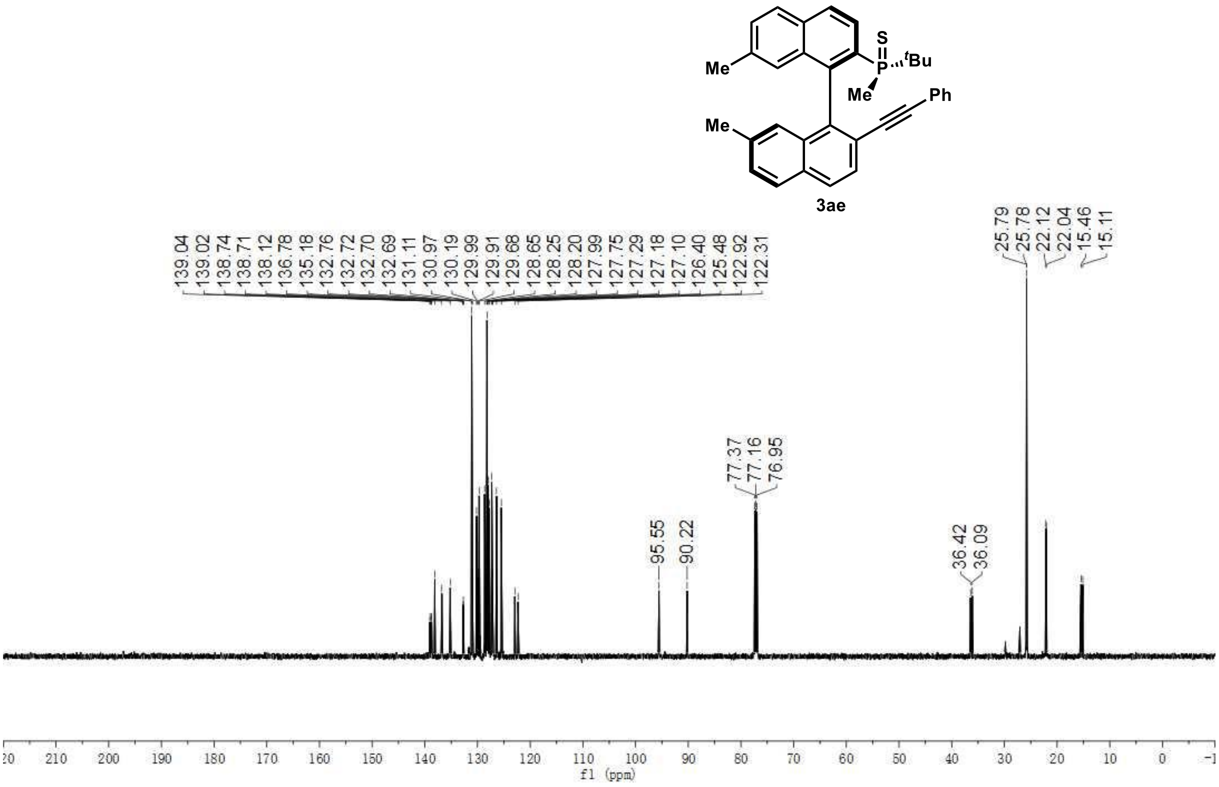

Supplementary Fig. 136.^{13}C NMR spectrum of **3ae**. The sample has been recorded in 151 MHz, $CDCl_3$ at 25 ℃.

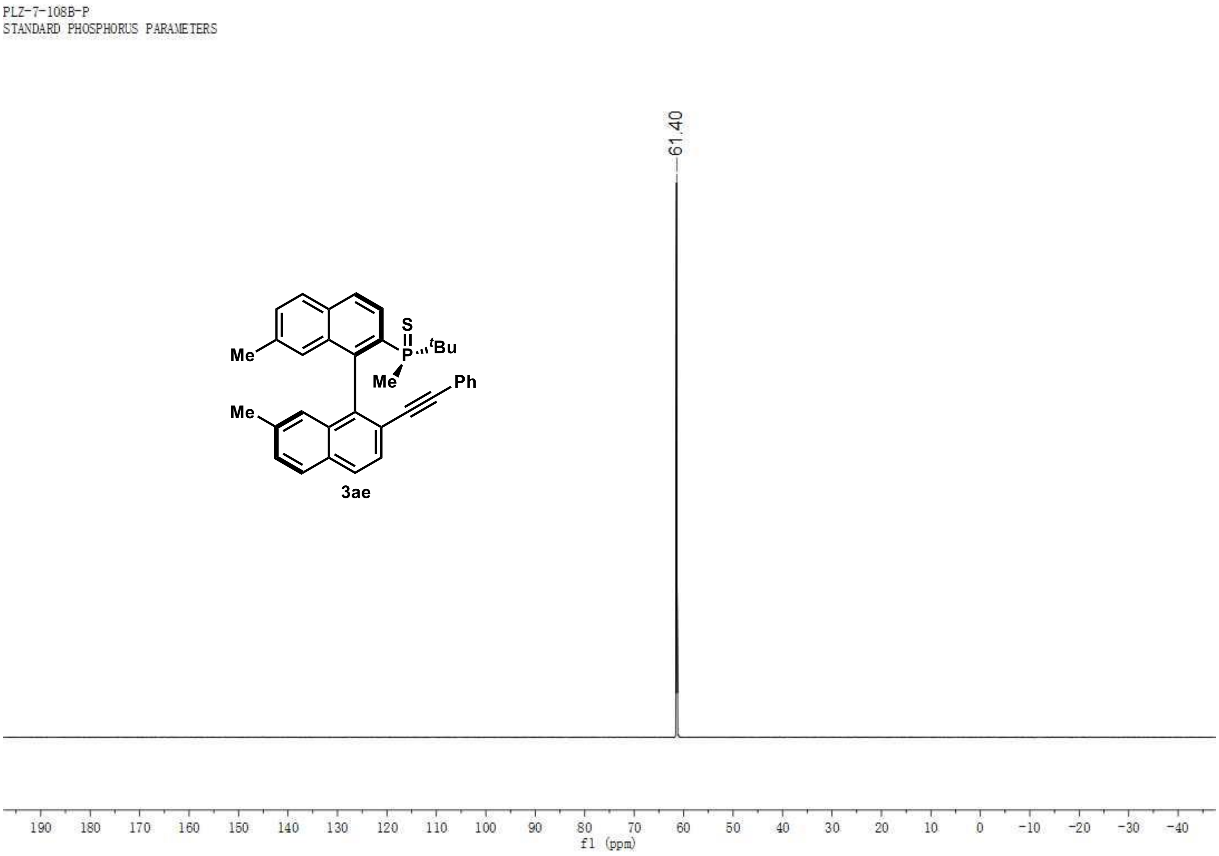

Supplementary Fig. 137.^{31}P NMR spectrum of **3ae**. The sample has been recorded in 243 MHz, $CDCl_3$ at 25 ℃.

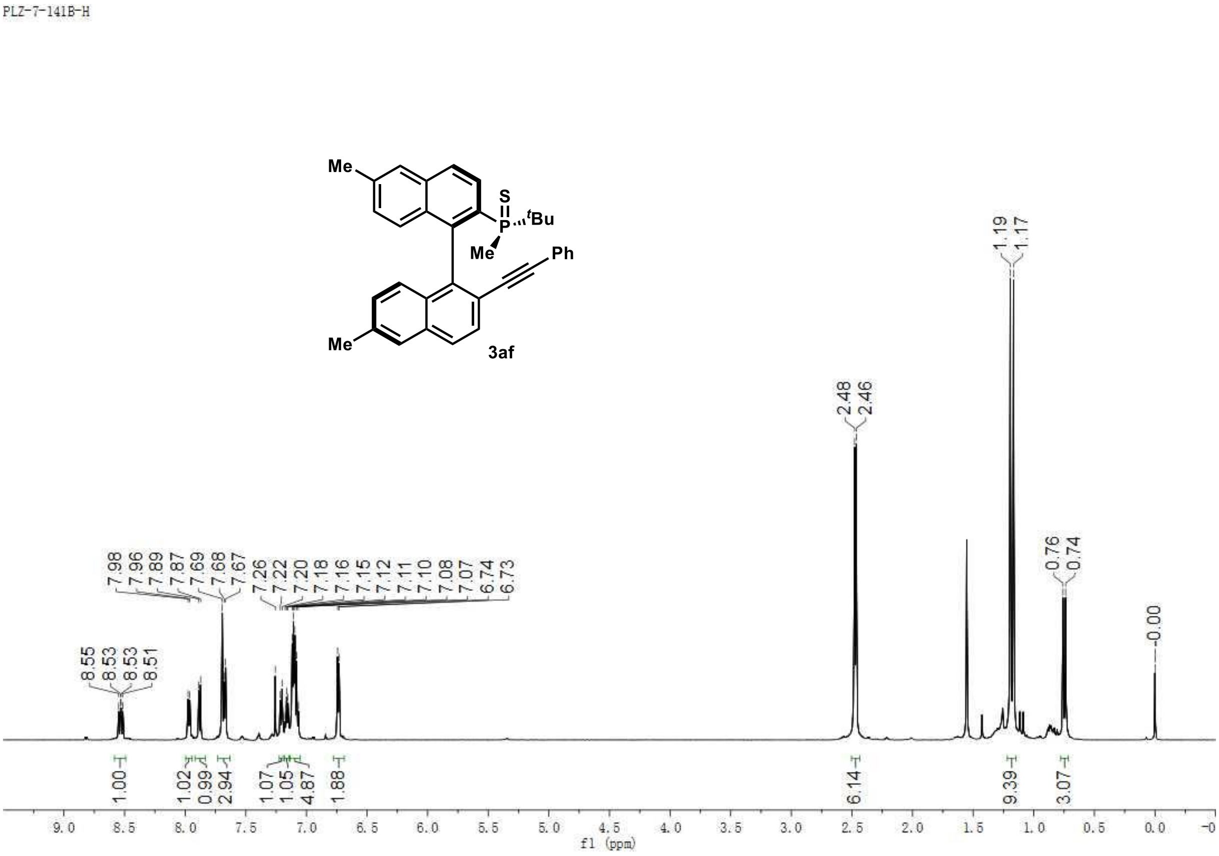

Supplementary Fig. 138. ^{1}H NMR spectrum of **3af**. The sample has been recorded in 600 MHz, $CDCl_3$ at 25 ℃.

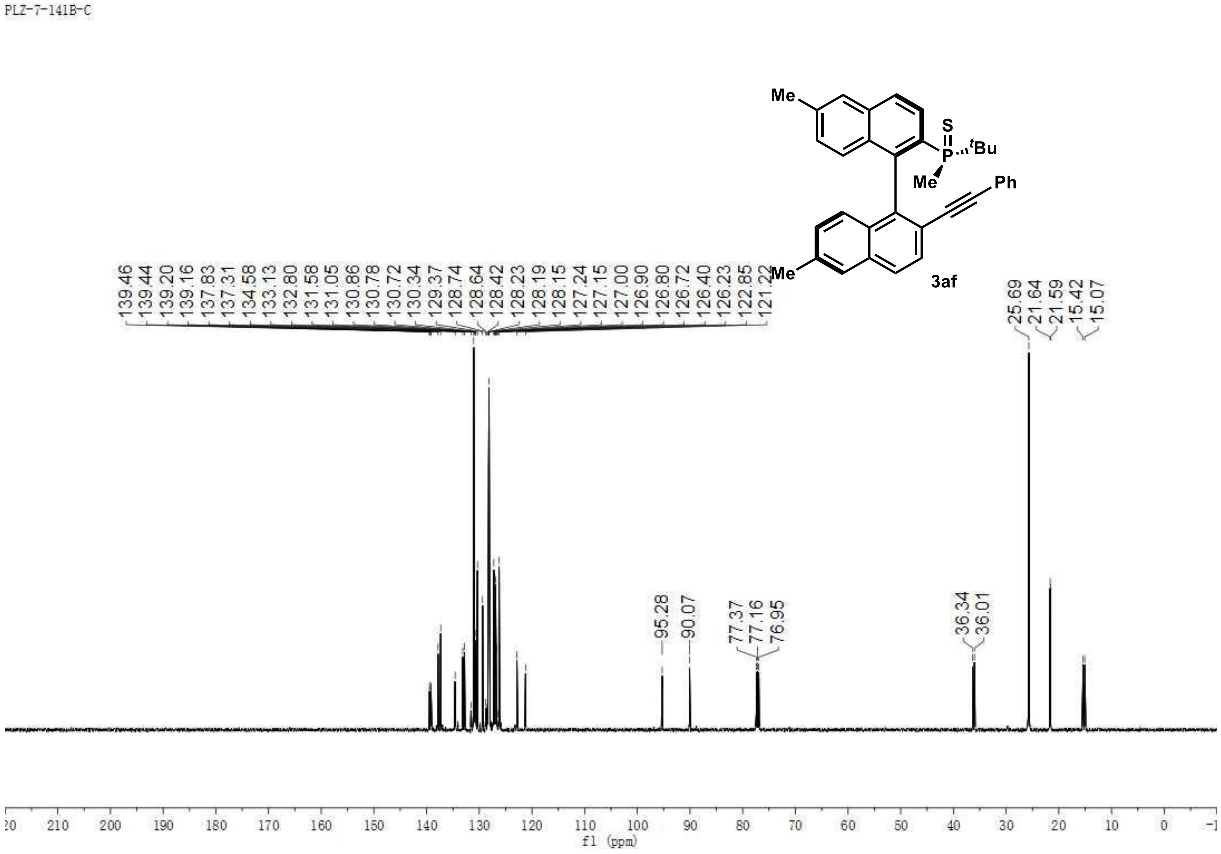

Supplementary Fig. 139.^{13}C NMR spectrum of **3af**. The sample has been recorded in 151 MHz, $CDCl_3$ at 25 ℃.

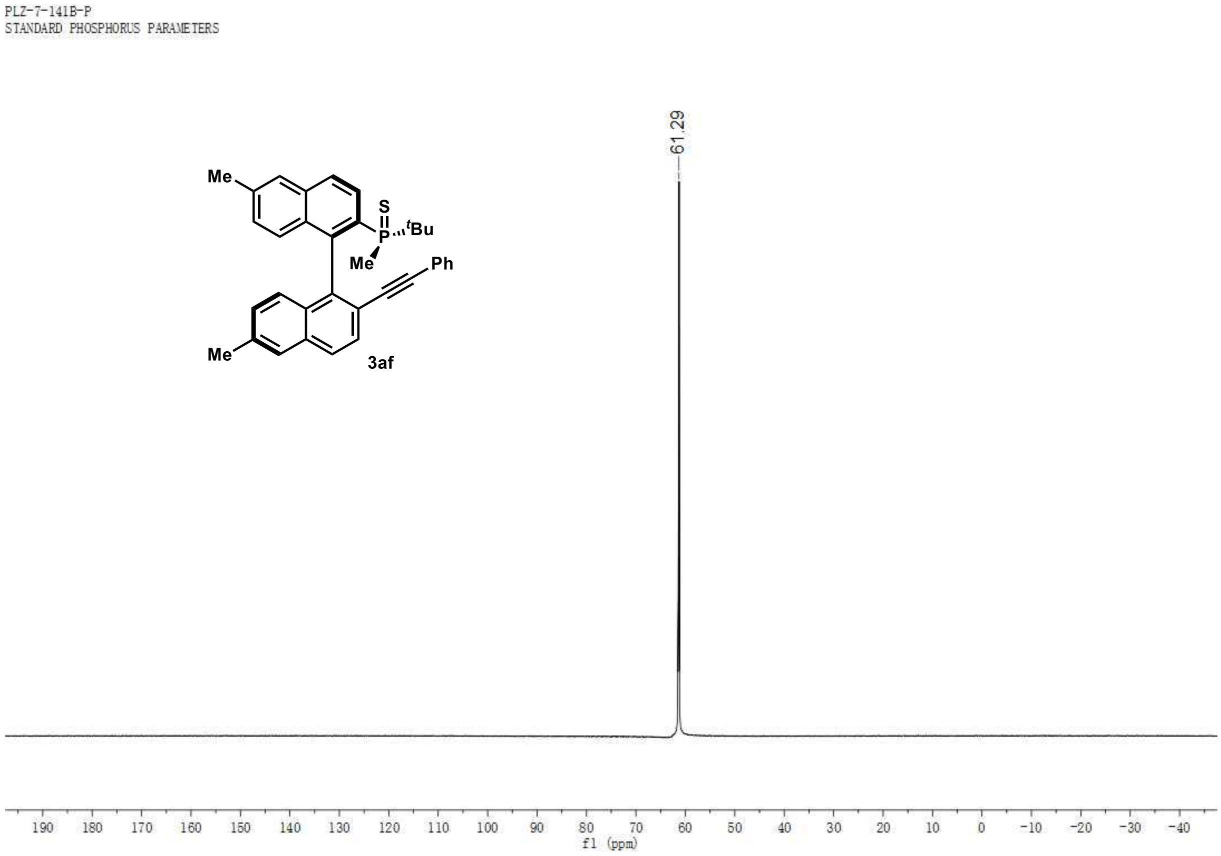

Supplementary Fig. 140.^{31}P NMR spectrum of **3af**. The sample has been recorded in 243 MHz, $CDCl_3$ at 25 ℃.

PLZ-7-108C-H

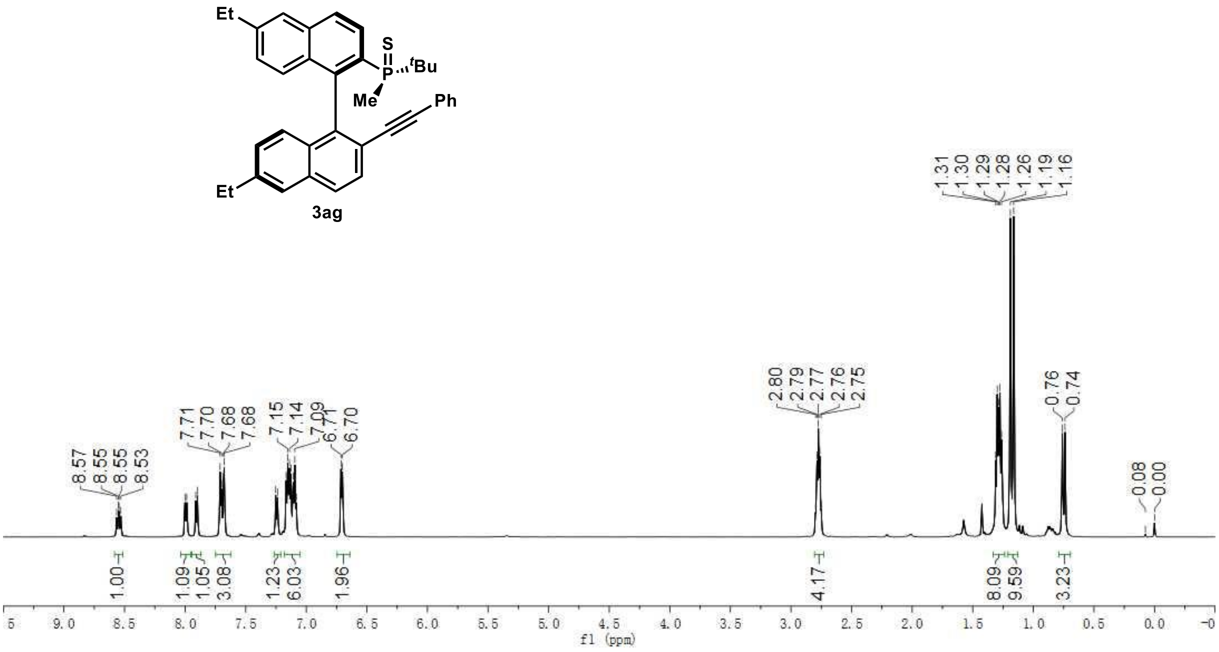

Supplementary Fig. 141. ^{1}H NMR spectrum of **3ag**. The sample has been recorded in 600 MHz, $CDCl_3$ at 25 ℃.

PLZ-7-108C-C

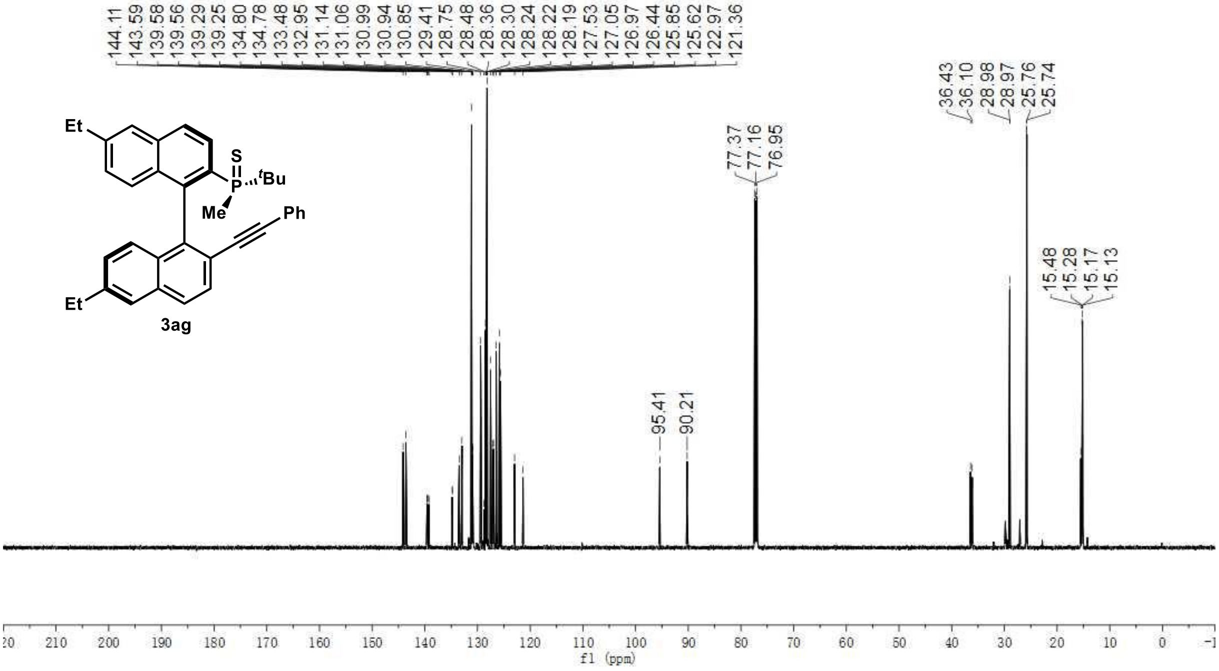

Supplementary Fig. 142.^{13}C NMR spectrum of **3ag**. The sample has been recorded in 151 MHz, $CDCl_3$ at 25 ℃.

PLZ-7-108C-P
STANDARD PHOSPHORUS PARAMETERS

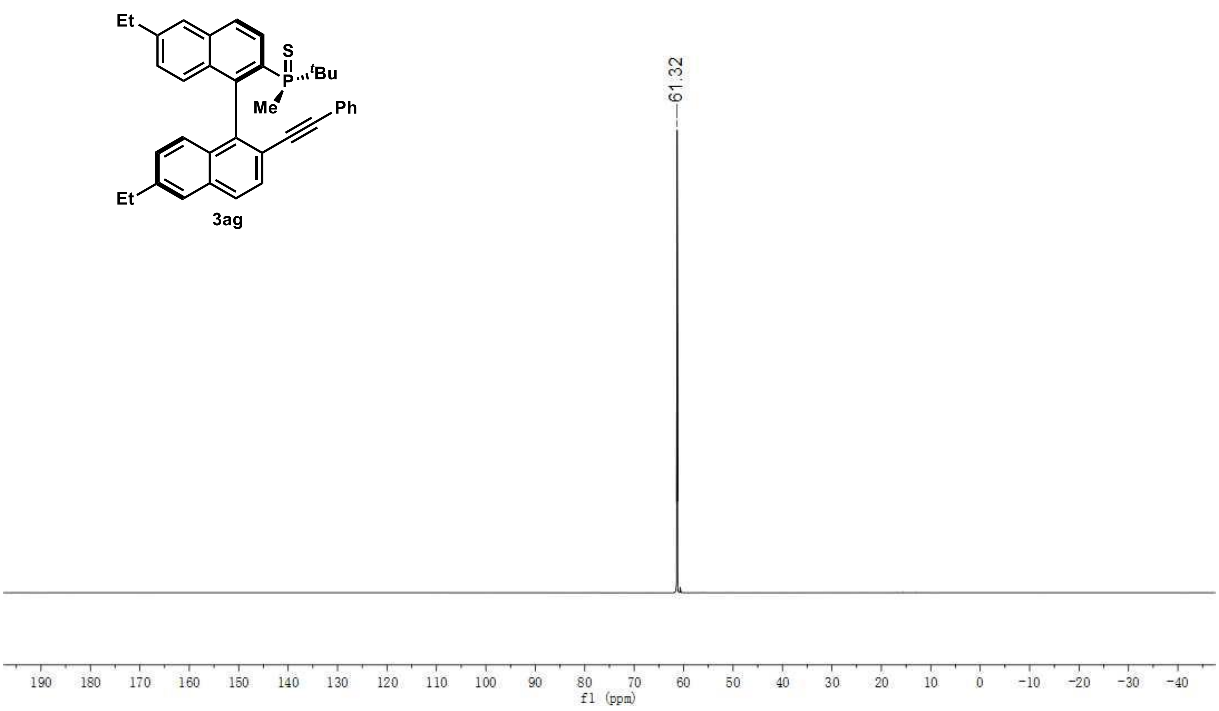

Supplementary Fig. 143.^{31}P NMR spectrum of **3ag**. The sample has been recorded in 243 MHz, $CDCl_3$ at 25 ℃.

PL2-7-36A-H

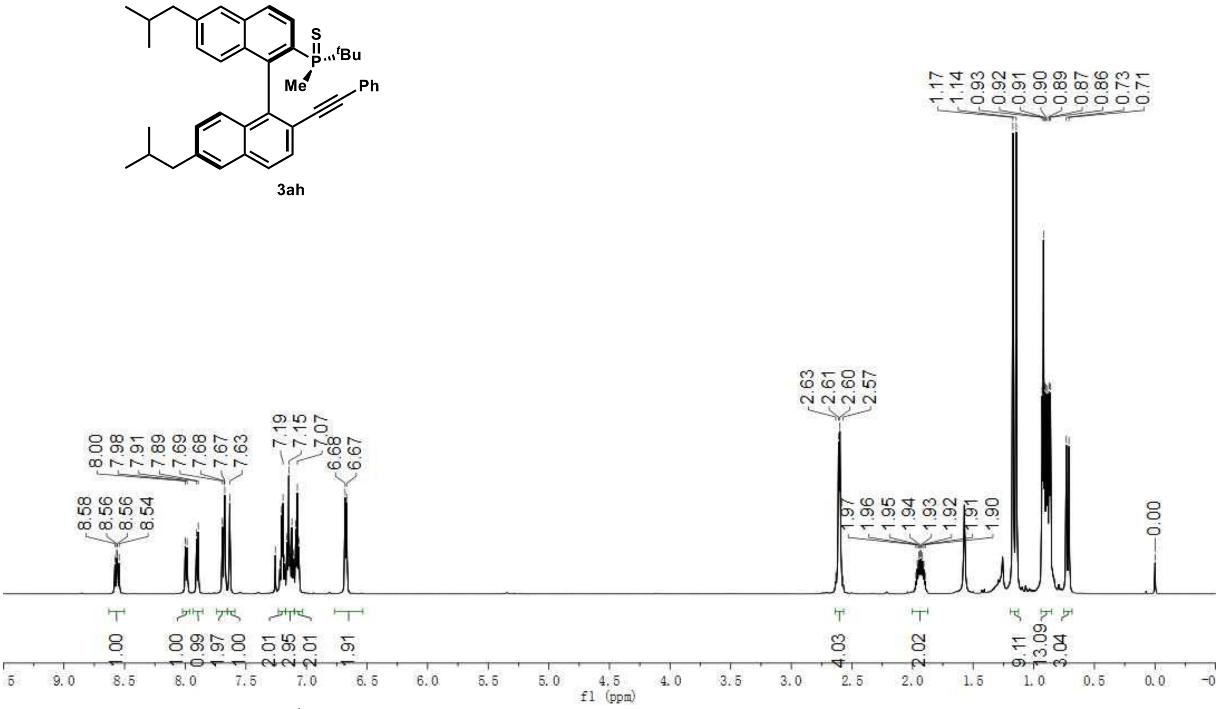

Supplementary Fig. 144. ^{1}H NMR spectrum of **3ah**. The sample has been recorded in 600 MHz, $CDCl_3$ at 25 ℃.

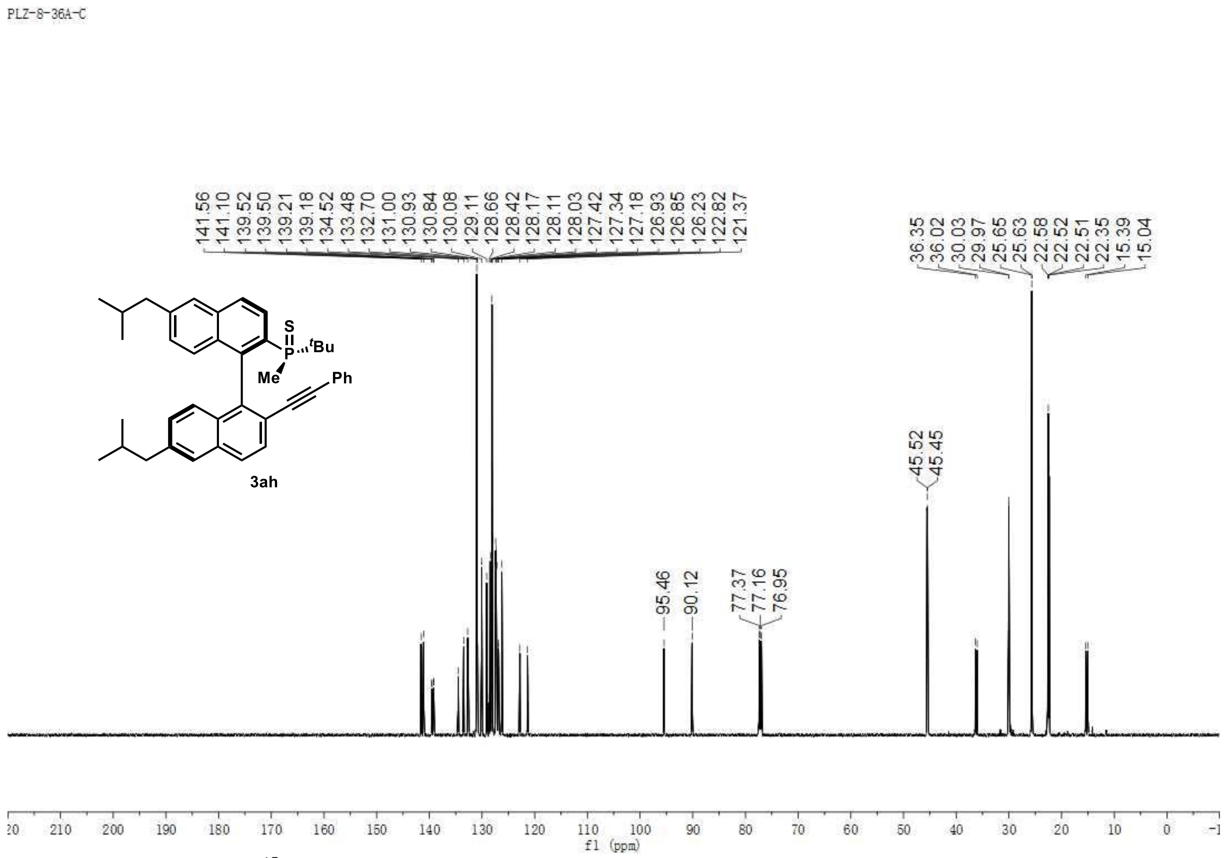

Supplementary Fig. 145. ^{13}C NMR spectrum of **3ah**. The sample has been recorded in 151 MHz, $CDCl_3$ at 25 ℃.

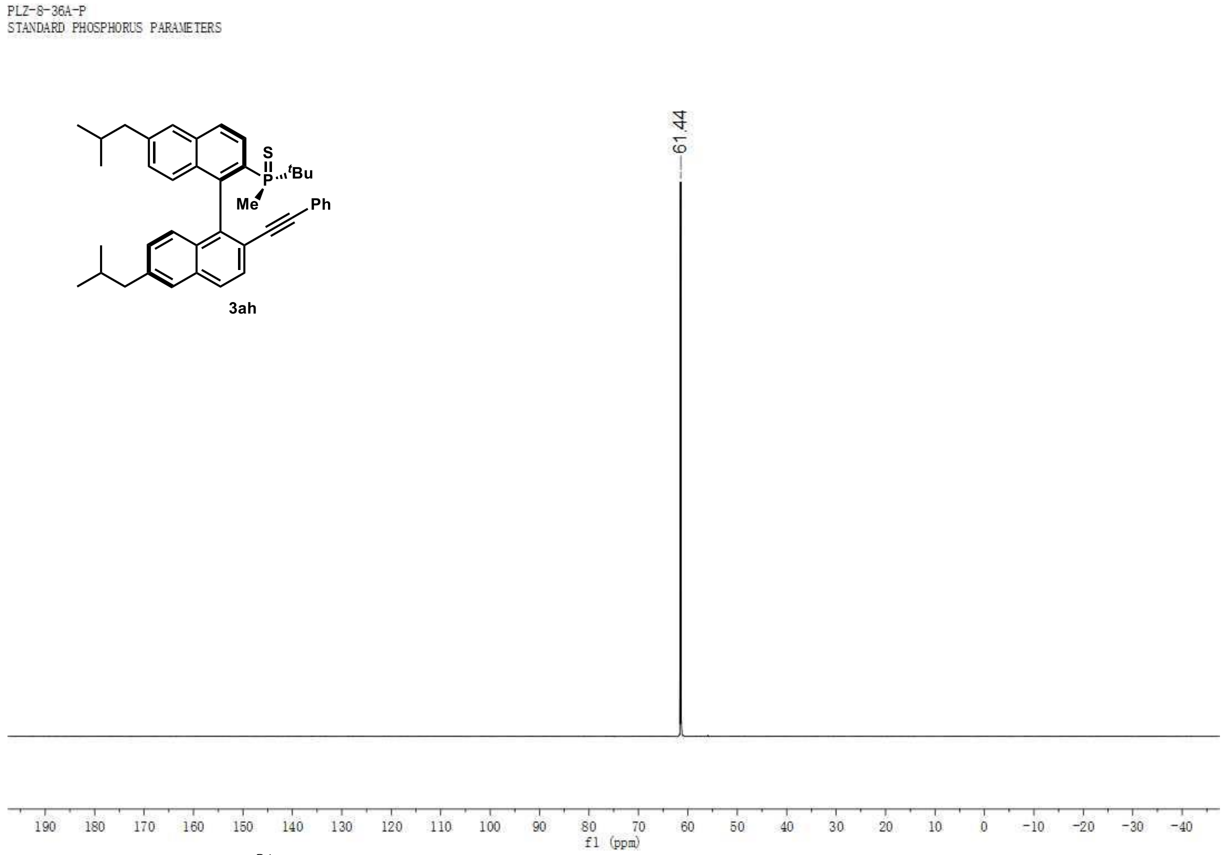

Supplementary Fig. 146.^{31}P NMR spectrum of **3ah**. The sample has been recorded in 243 MHz, $CDCl_3$ at 25 ℃.

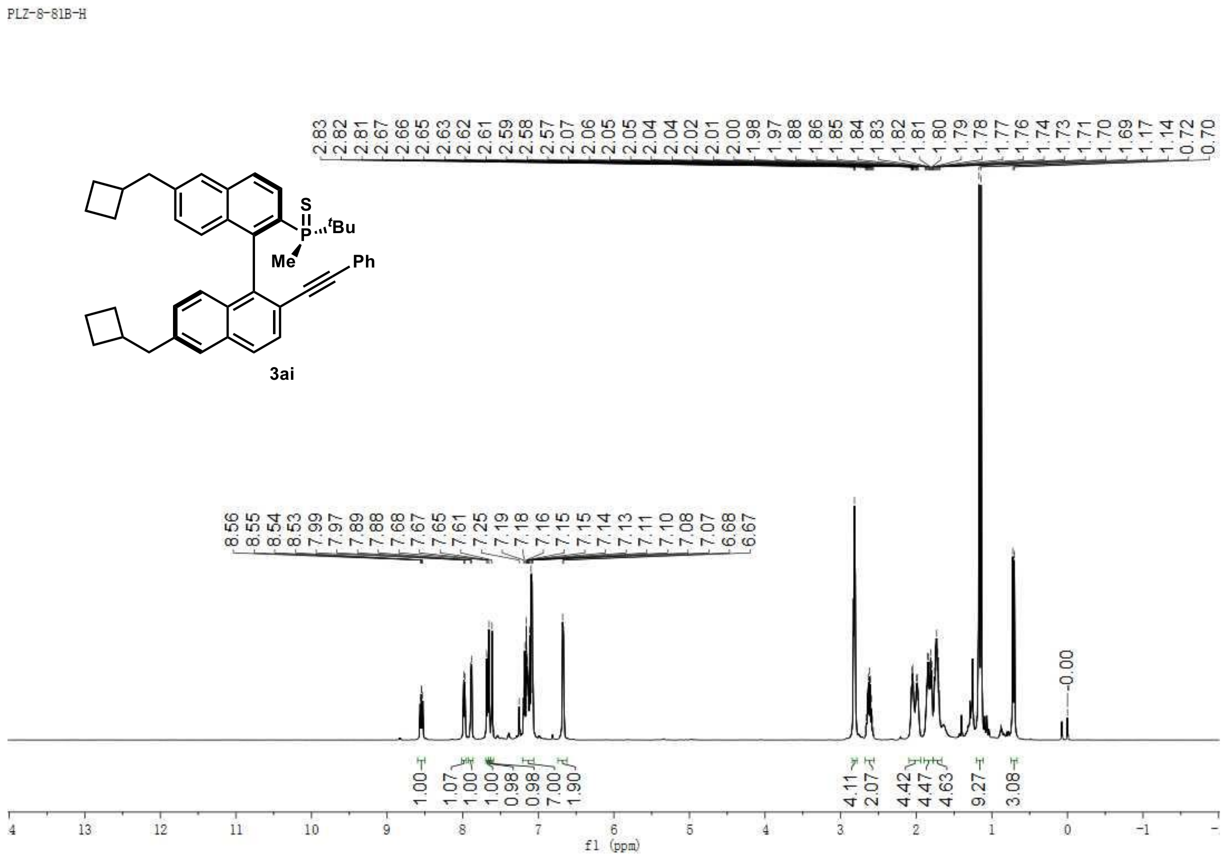

Supplementary Fig. 147. ^{1}H NMR spectrum of **3ai**. The sample has been recorded in 600 MHz, $CDCl_3$ at 25 ℃.

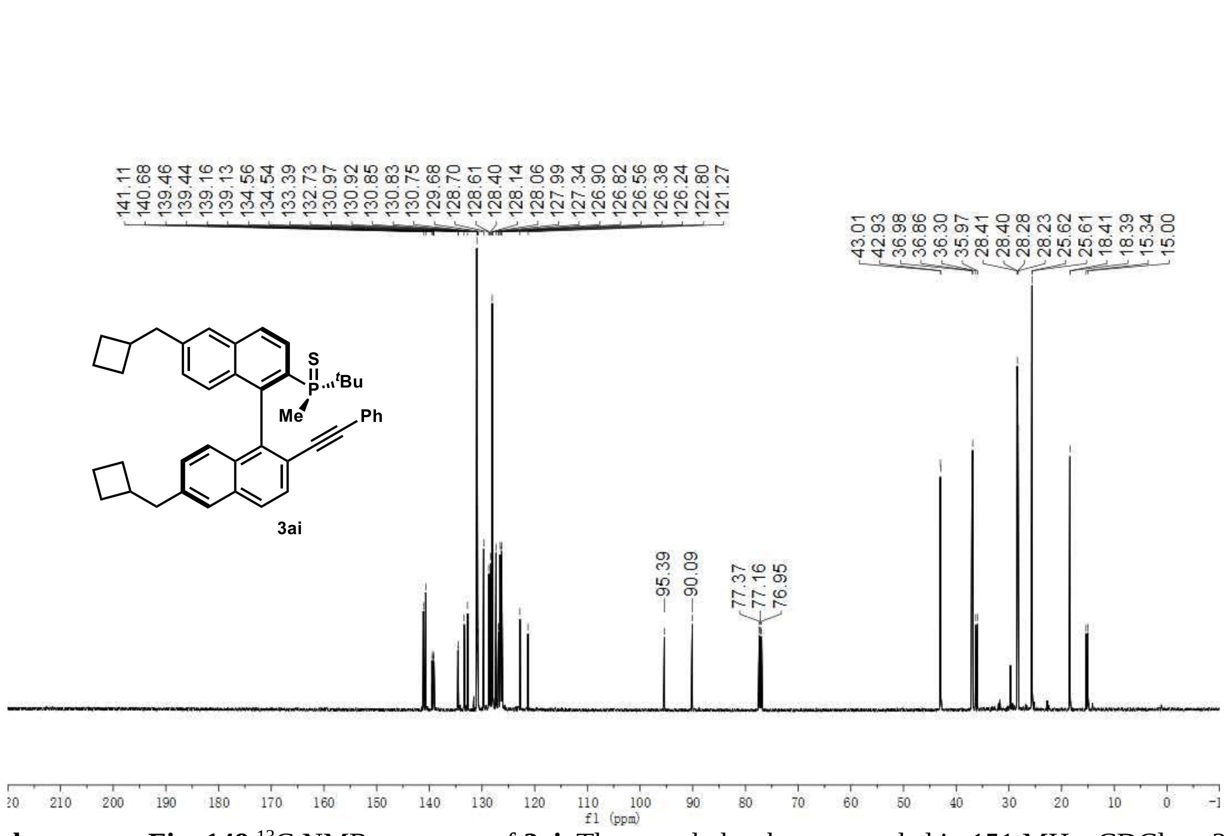

Supplementary Fig. 148.^{13}C NMR spectrum of **3ai**. The sample has been recorded in 151 MHz, $CDCl_3$ at 25 ℃.

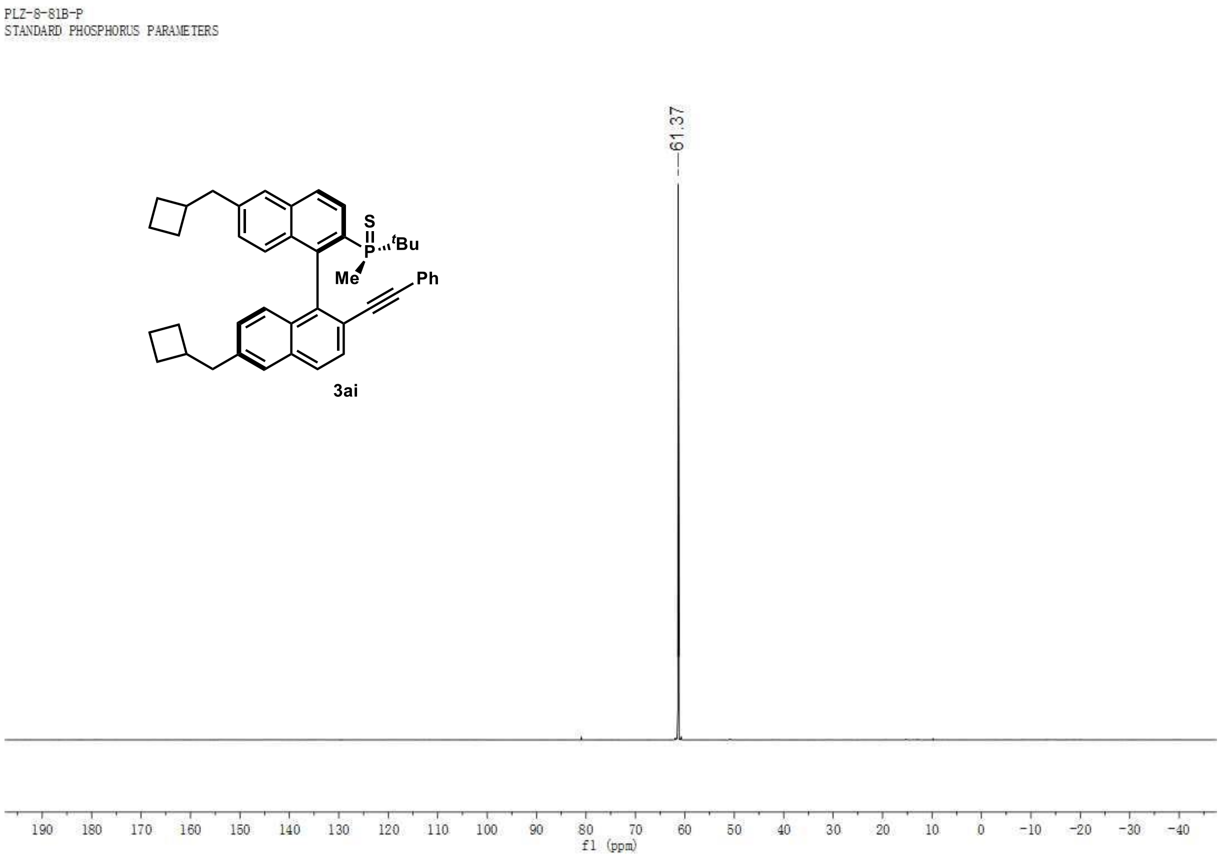

Supplementary Fig. 149.^{31}P NMR spectrum of **3ai**. The sample has been recorded in 243 MHz, $CDCl_3$ at 25 ℃.

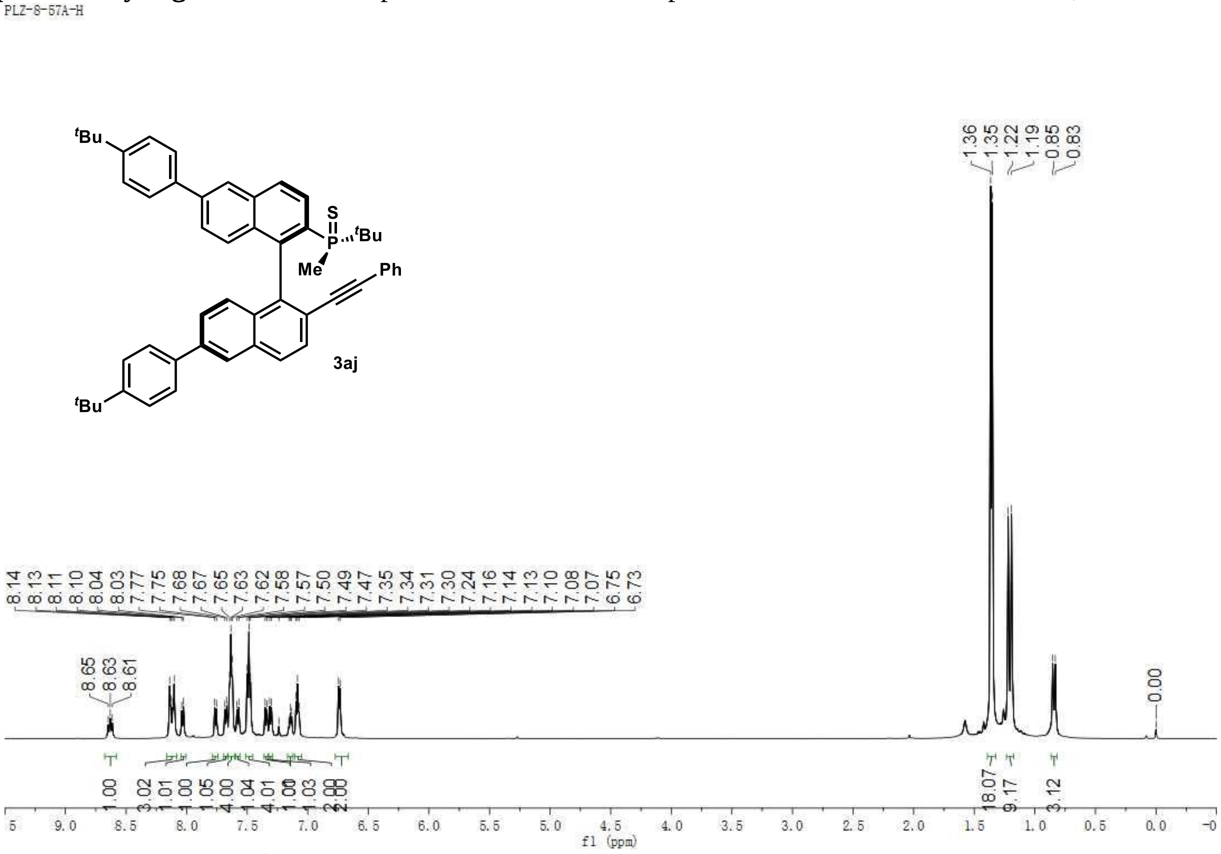

Supplementary Fig. 150. ^{1}H NMR spectrum of **3aj**. The sample has been recorded in 600 MHz, $CDCl_3$ at 25 ℃.

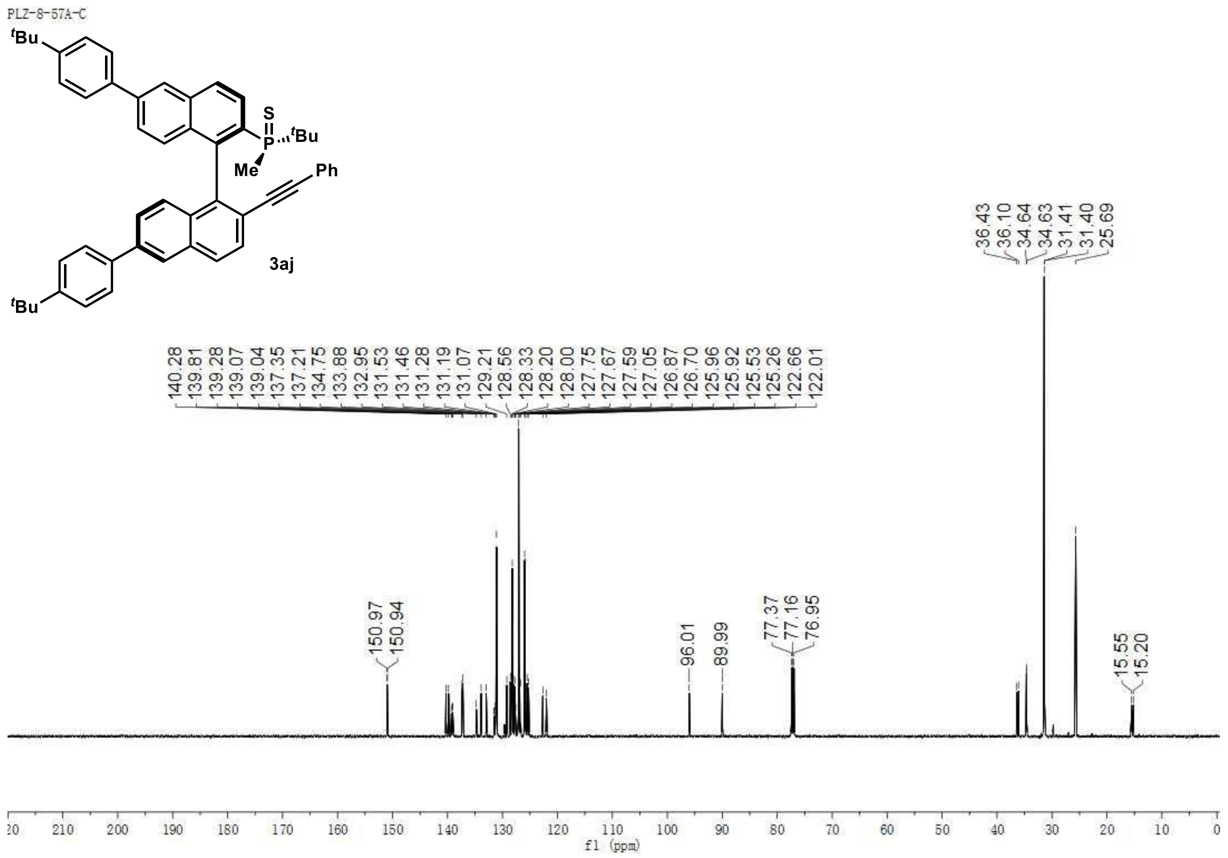

Supplementary Fig. 151.^{13}C NMR spectrum of **3aj**. The sample has been recorded in 151 MHz, $CDCl_3$ at 25 ℃.

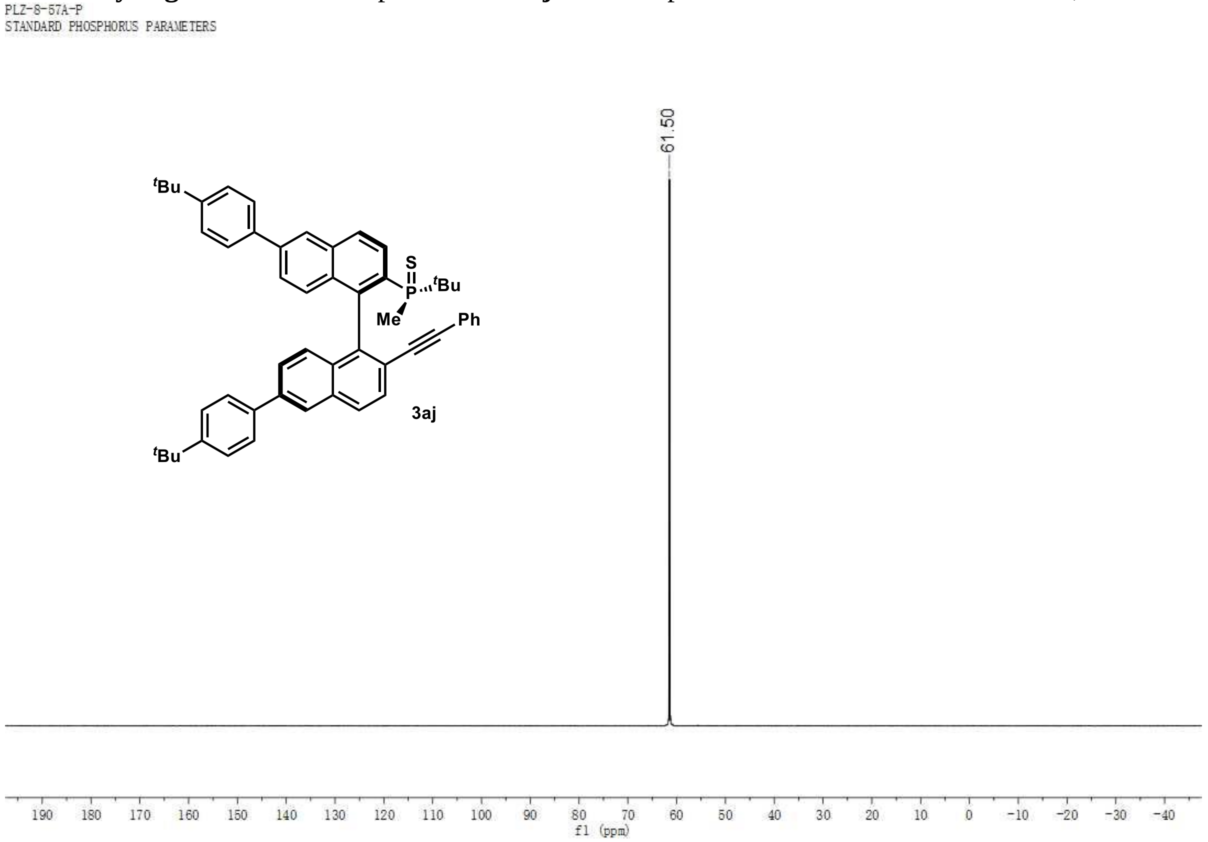

Supplementary Fig. 152.^{31}P NMR spectrum of **3aj**. The sample has been recorded in 243 MHz, $CDCl_3$ at 25 ℃.

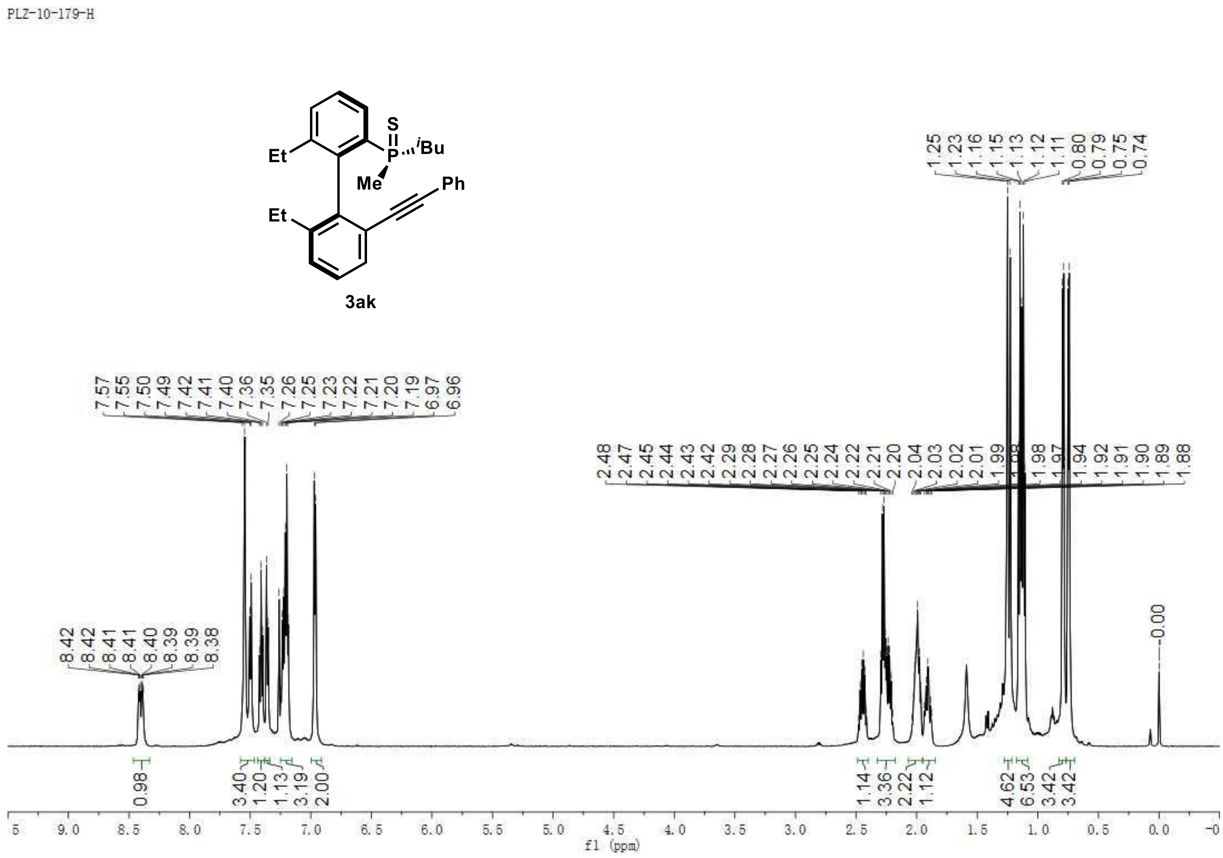

Supplementary Fig. 153. ^{1}H NMR spectrum of **3ak**. The sample has been recorded in 600 MHz, $CDCl_3$ at 25 ℃.

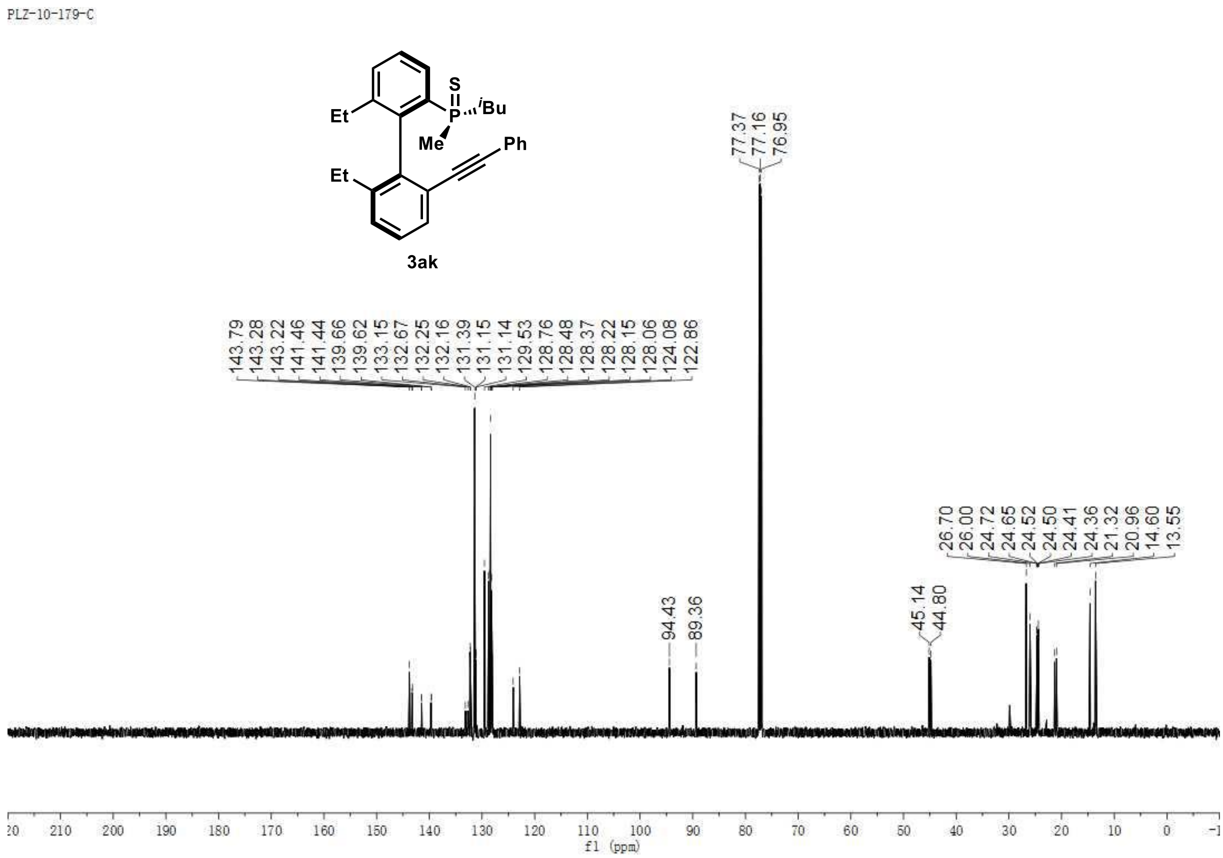

Supplementary Fig. 154.^{13}C NMR spectrum of **3ak**. The sample has been recorded in 151 MHz, $CDCl_3$ at 25 ℃.

PLZ-10-179-P
STANDARD PHOSPHORUS PARAMETERS

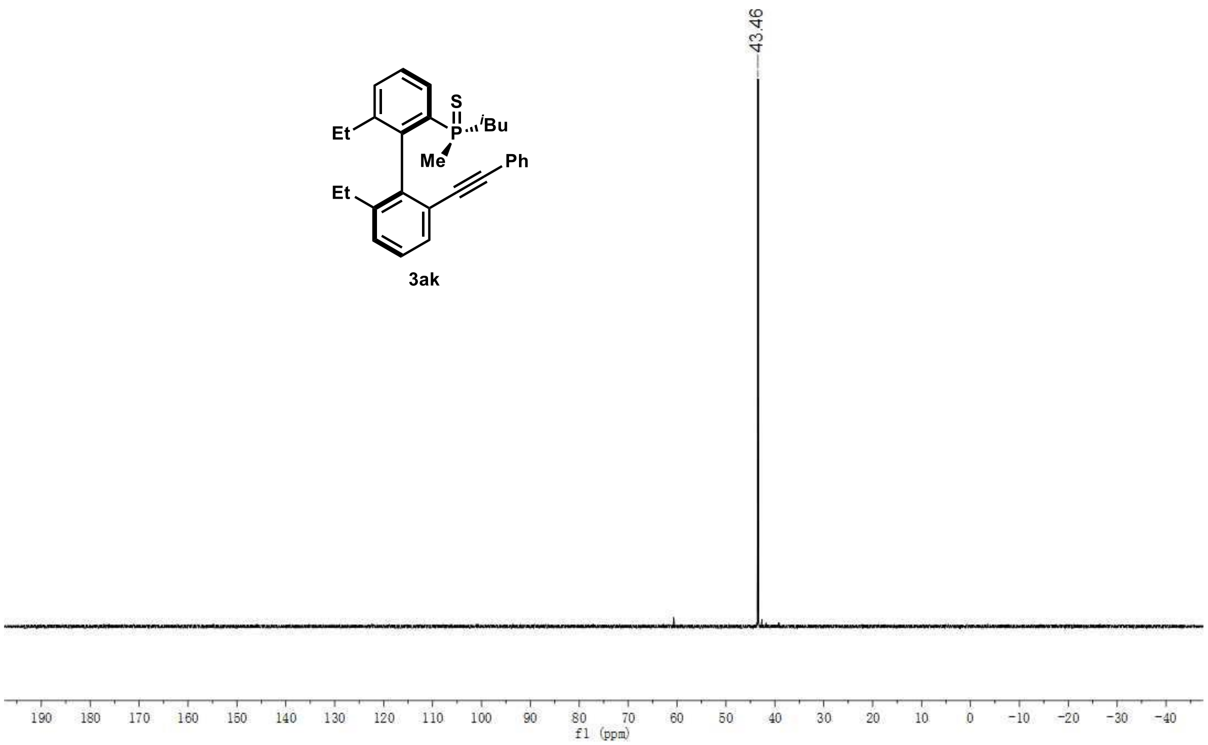

Supplementary Fig. 155.^{31}P NMR spectrum of **3ak**. The sample has been recorded in 243 MHz, $CDCl_3$ at 25 ℃.

PLZ-10-161-H-23-4-25

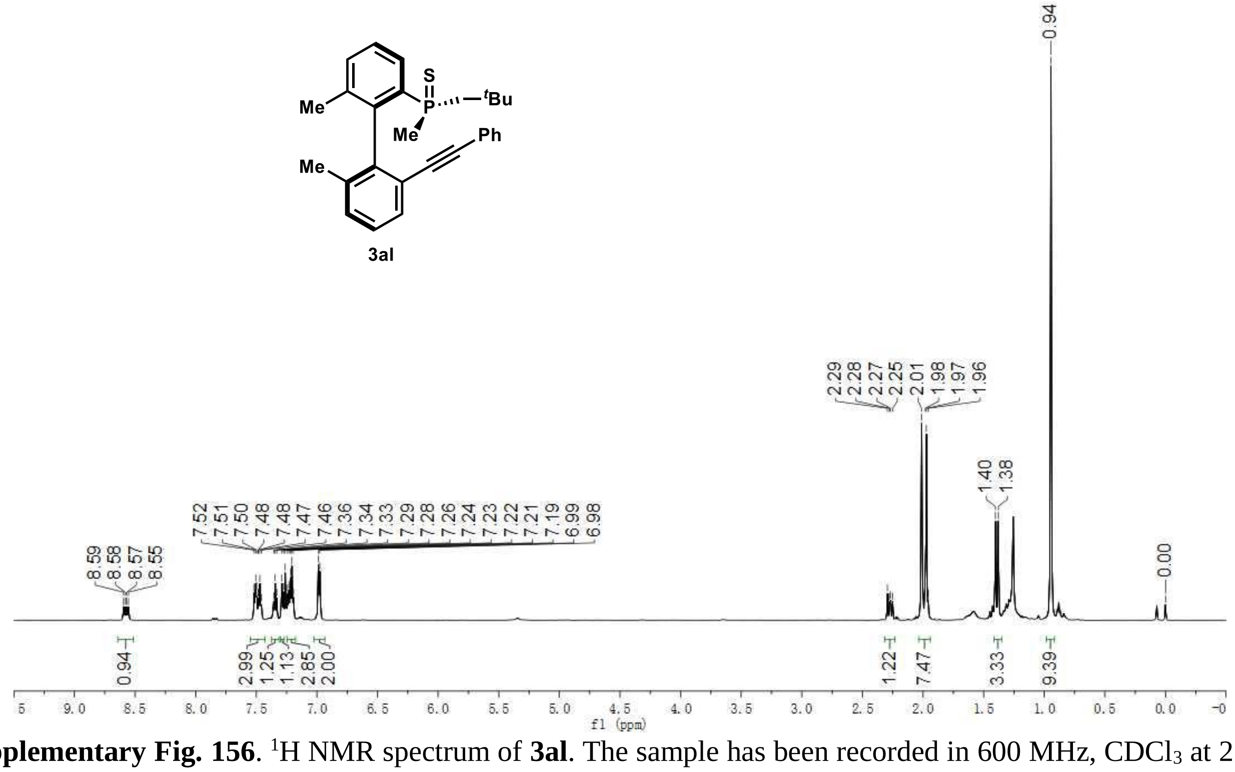

Supplementary Fig. 156. ^{1}H NMR spectrum of **3al**. The sample has been recorded in 600 MHz, $CDCl_3$ at 25 ℃.

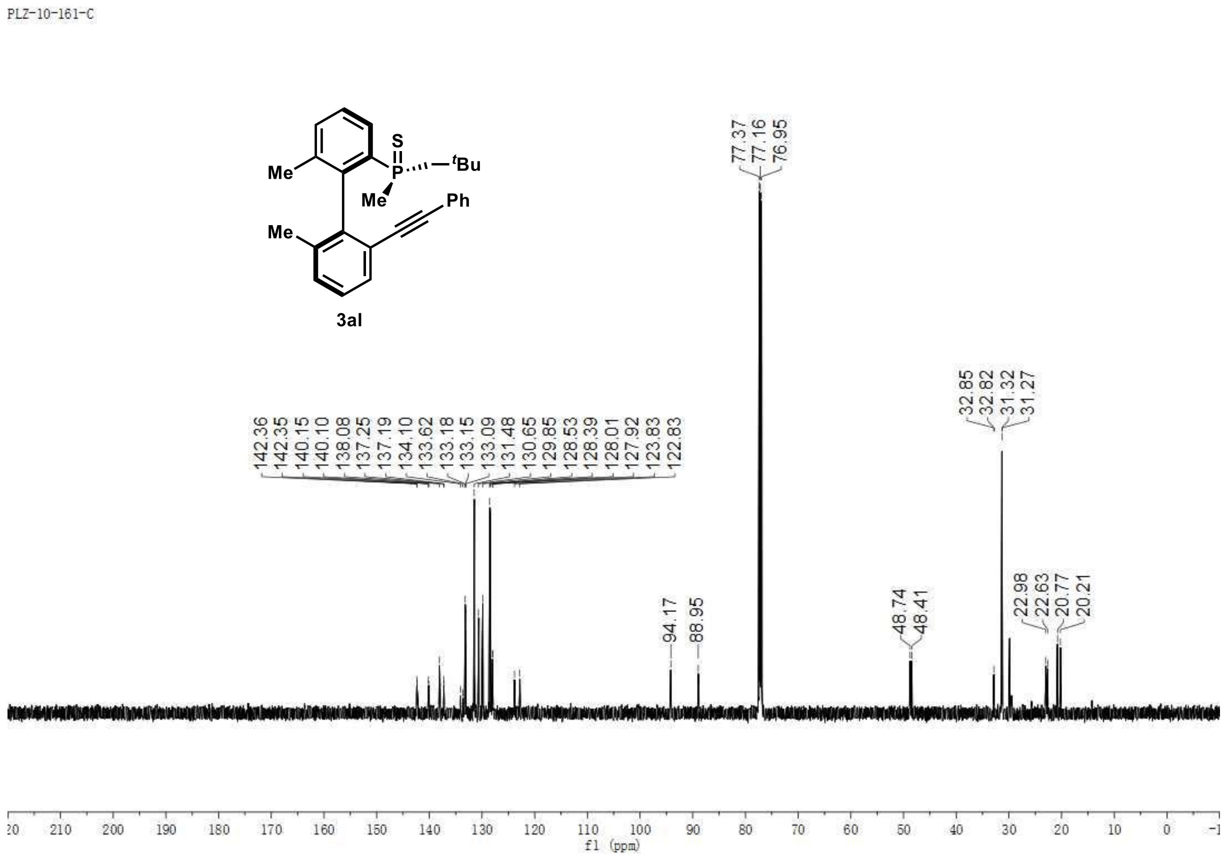

Supplementary Fig. 157.^{13}C NMR spectrum of **3al**. The sample has been recorded in 151 MHz, $CDCl_3$ at 25 ℃.

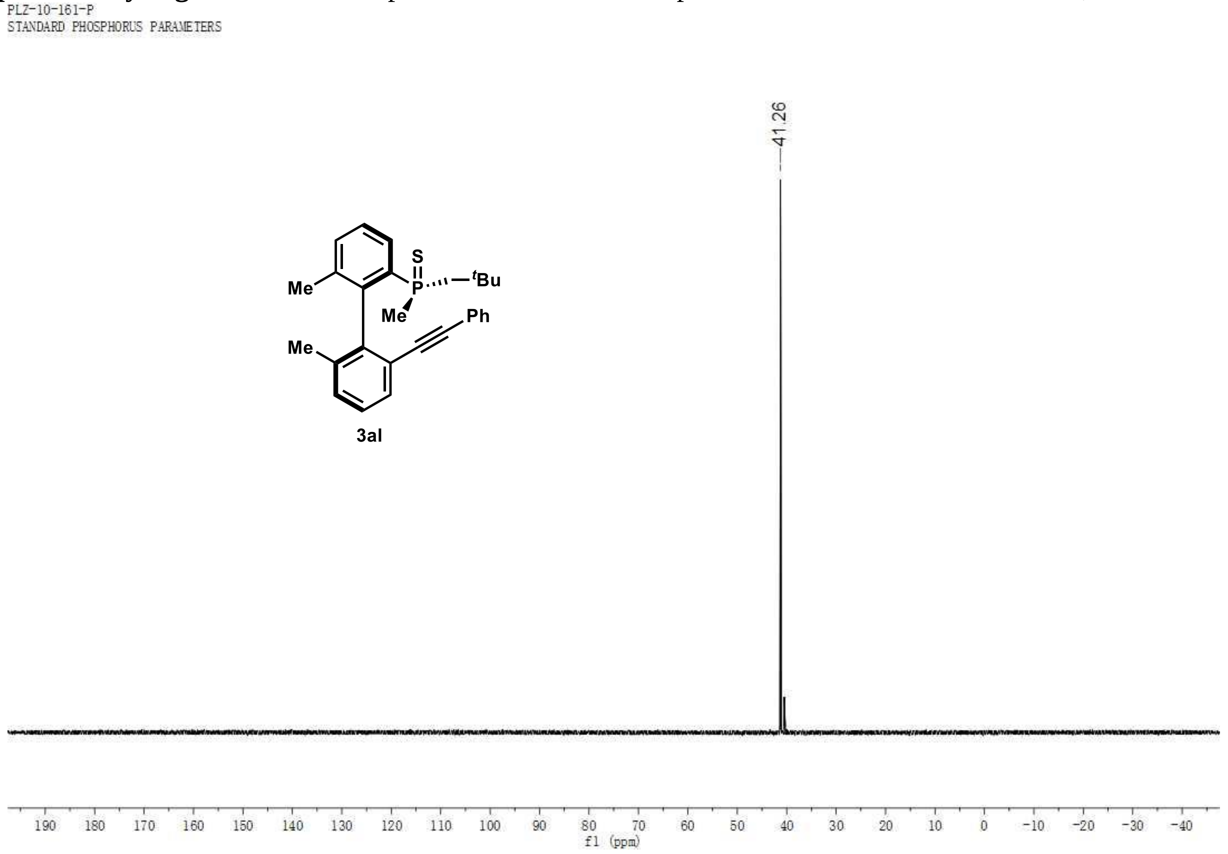

Supplementary Fig. 158.^{31}P NMR spectrum of **3al**. The sample has been recorded in 243 MHz, $CDCl_3$ at 25 ℃.

PLZ-9-92B-H

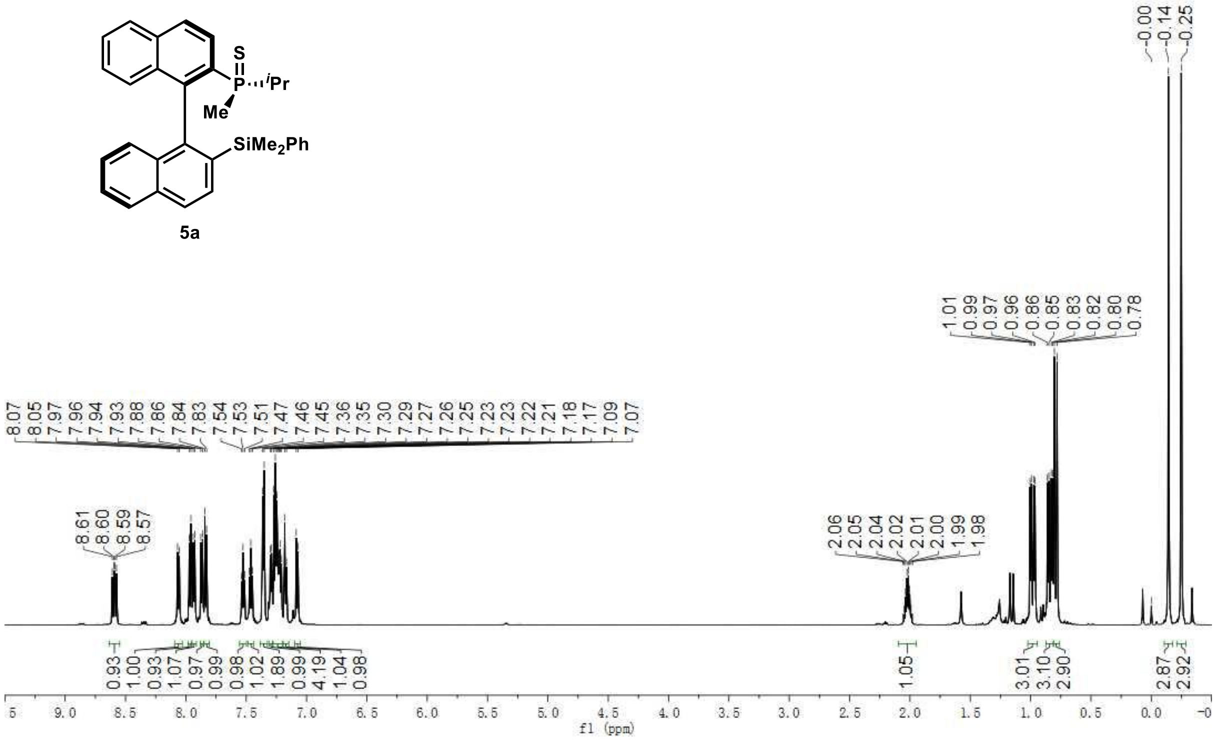

Supplementary Fig. 159. ^{1}H NMR spectrum of **5a**. The sample has been recorded in 600 MHz, $CDCl_3$ at 25 ℃.

PLZ-9-92B-C

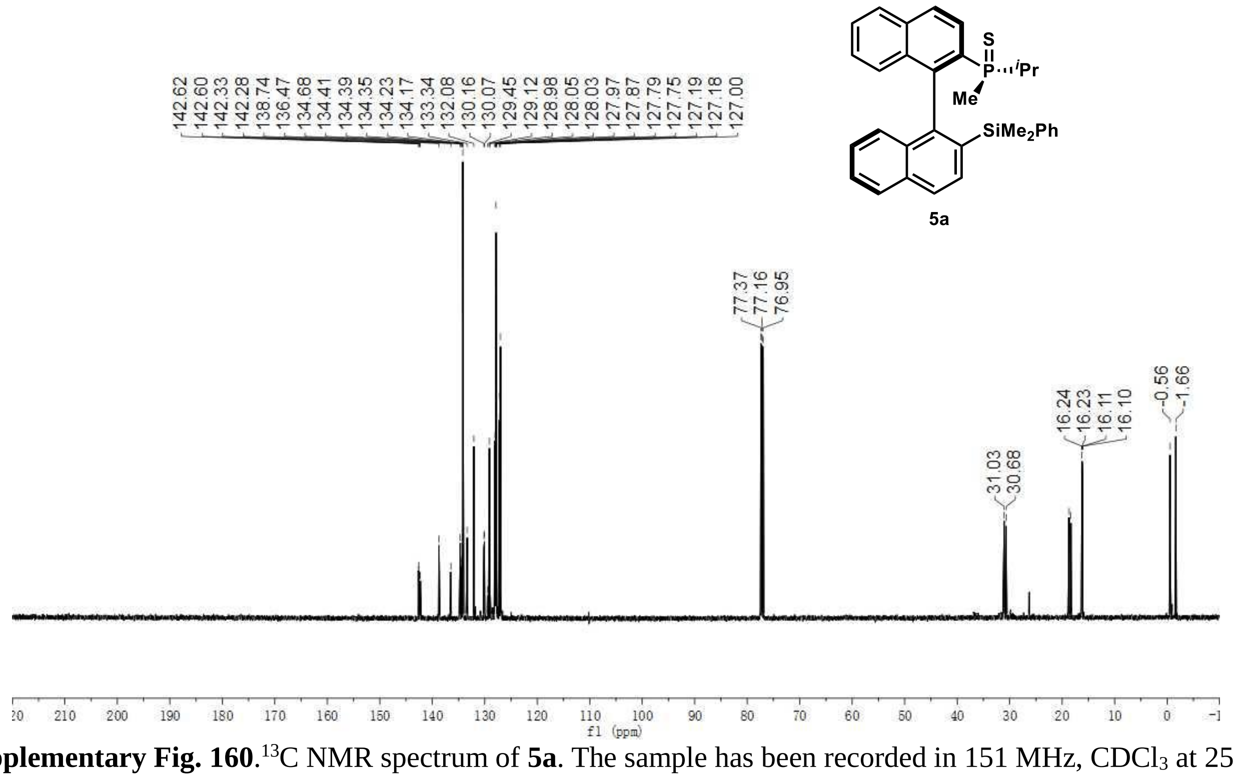

Supplementary Fig. 160.^{13}C NMR spectrum of **5a**. The sample has been recorded in 151 MHz, $CDCl_3$ at 25 ℃.

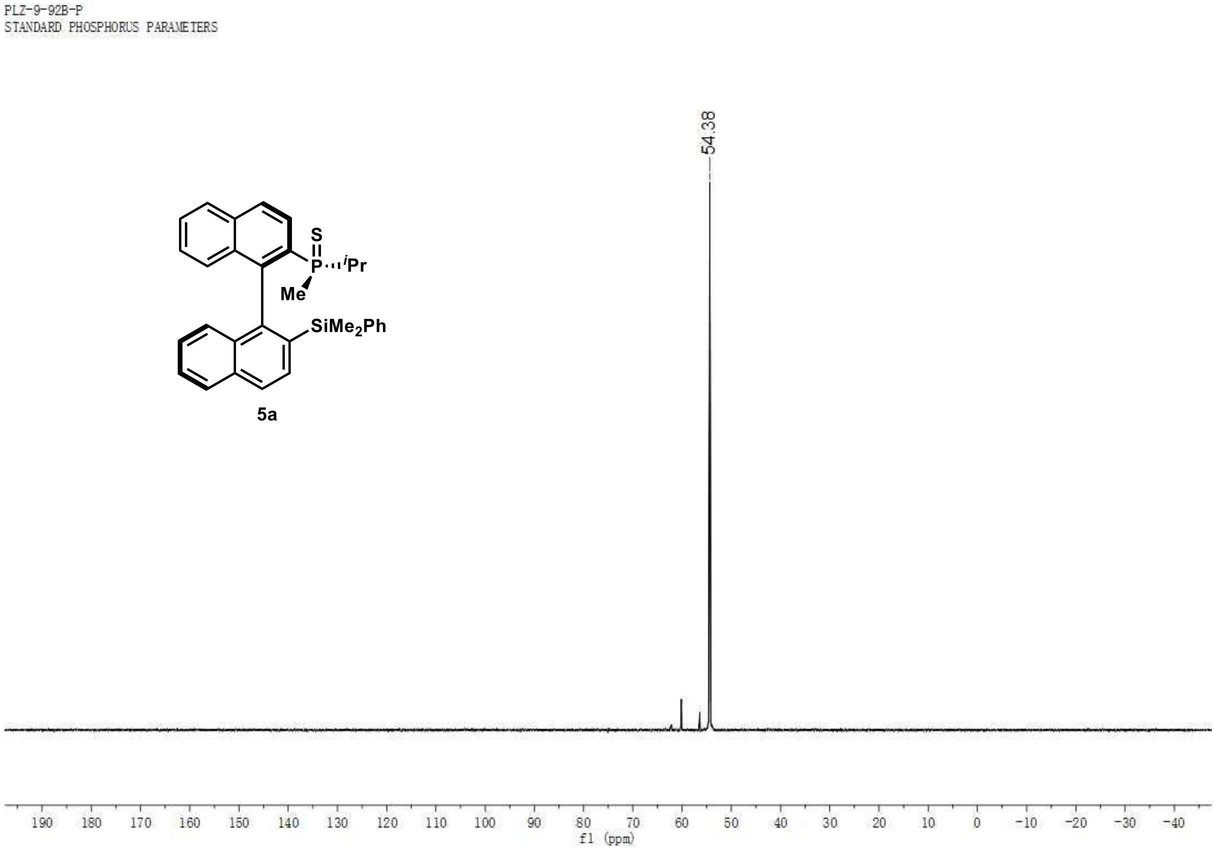

Supplementary Fig. 161.^{31}P NMR spectrum of **5a**. The sample has been recorded in 243 MHz, $CDCl_3$ at 25 ℃.

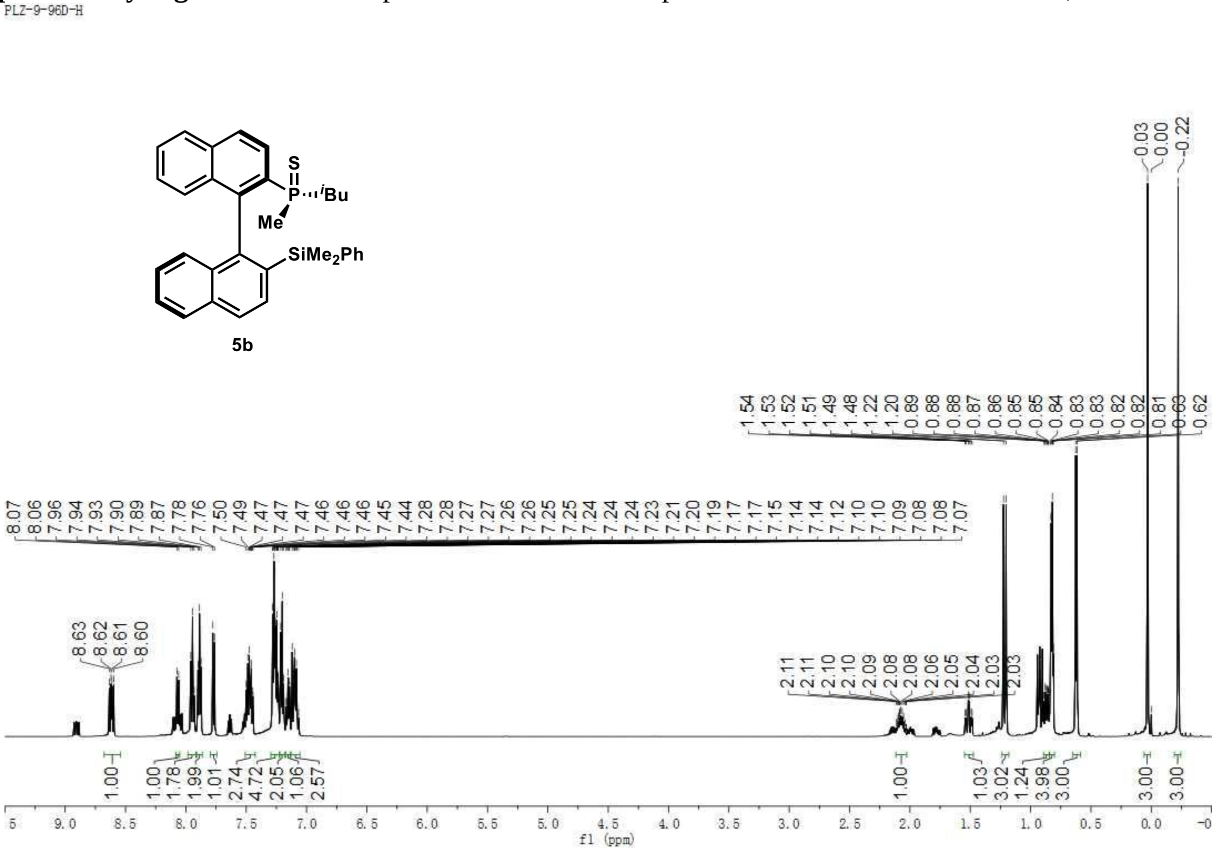

Supplementary Fig. 162. ^{1}H NMR spectrum of **5b**. The sample has been recorded in 600 MHz, $CDCl_3$ at 25 ℃.

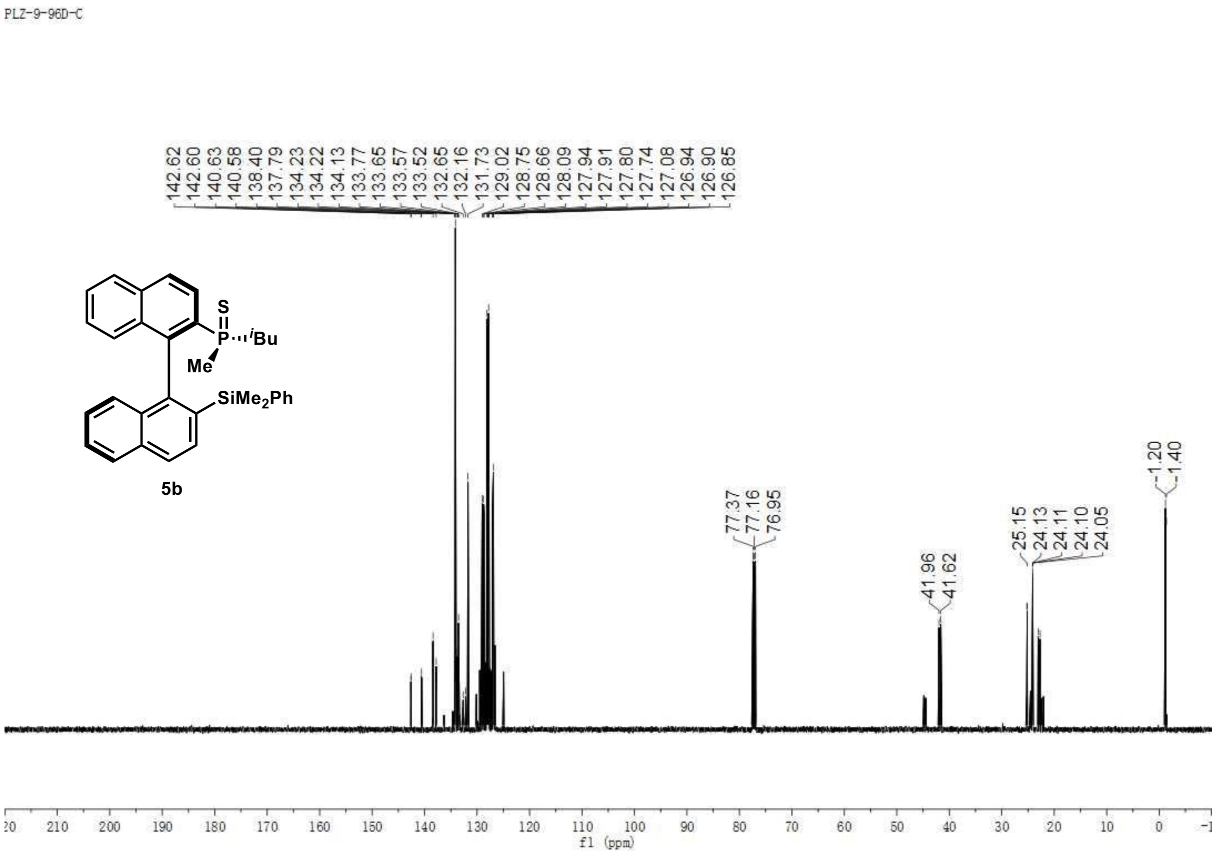

Supplementary Fig. 163.^{13}C NMR spectrum of **5b**. The sample has been recorded in 151 MHz, $CDCl_3$ at 25 ℃.

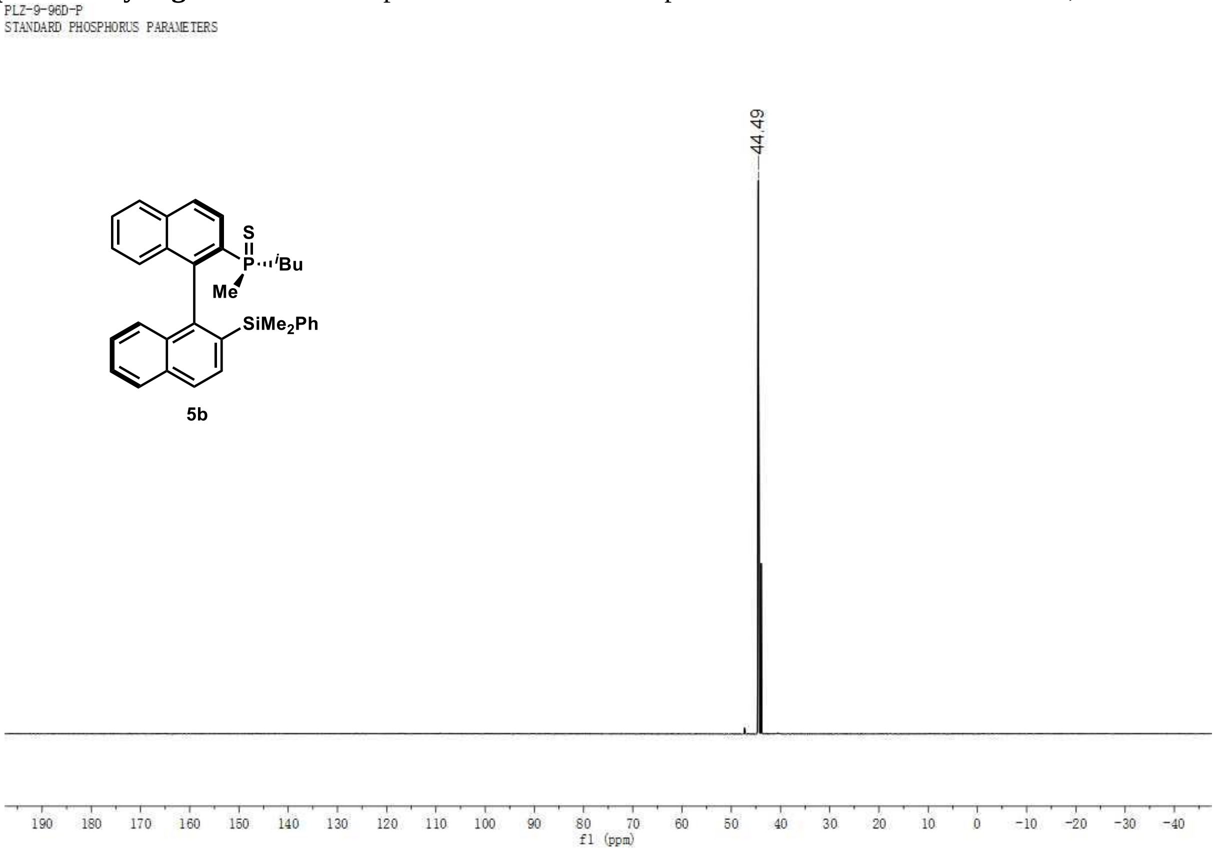

Supplementary Fig. 164.^{31}P NMR spectrum of **5b**. The sample has been recorded in 243 MHz, $CDCl_3$ at 25 ℃.

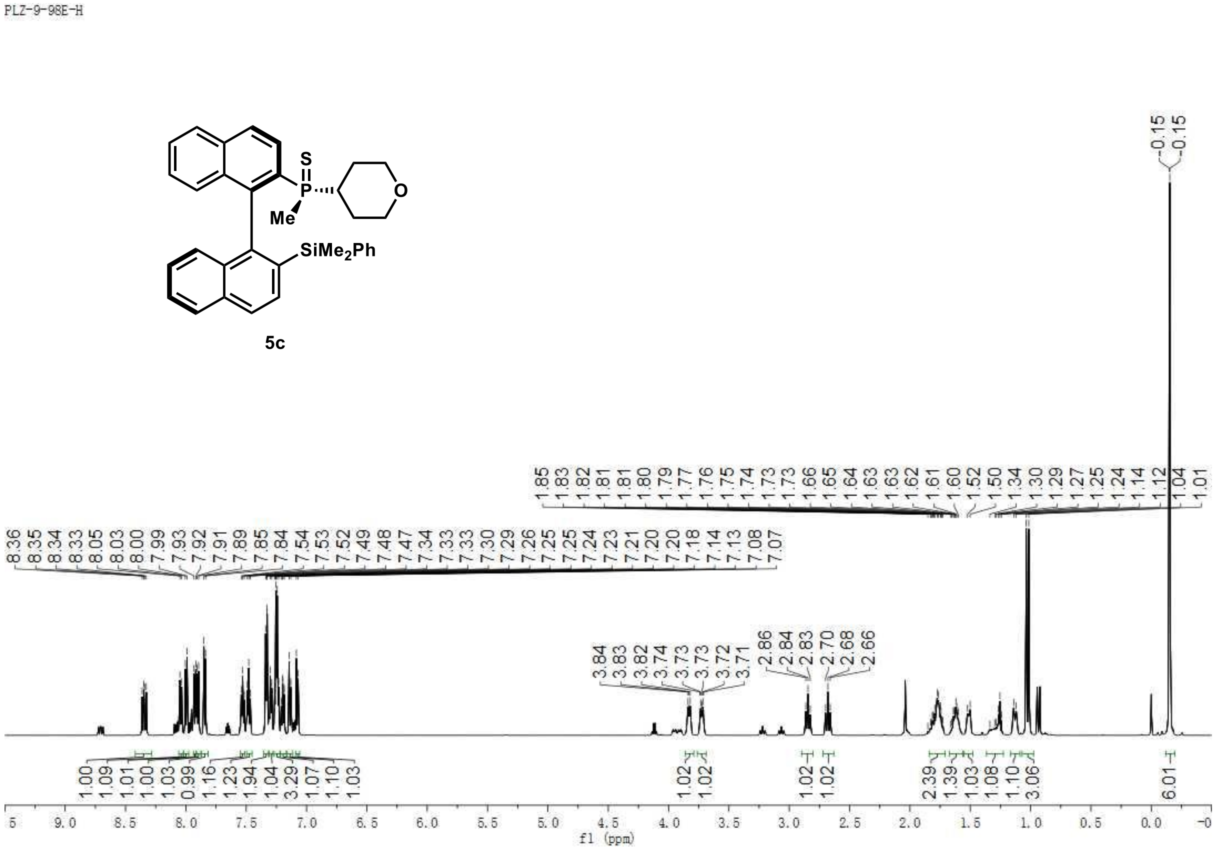

Supplementary Fig. 165. ^{1}H NMR spectrum of **5c**. The sample has been recorded in 600 MHz, $CDCl_3$ at 25 ℃.

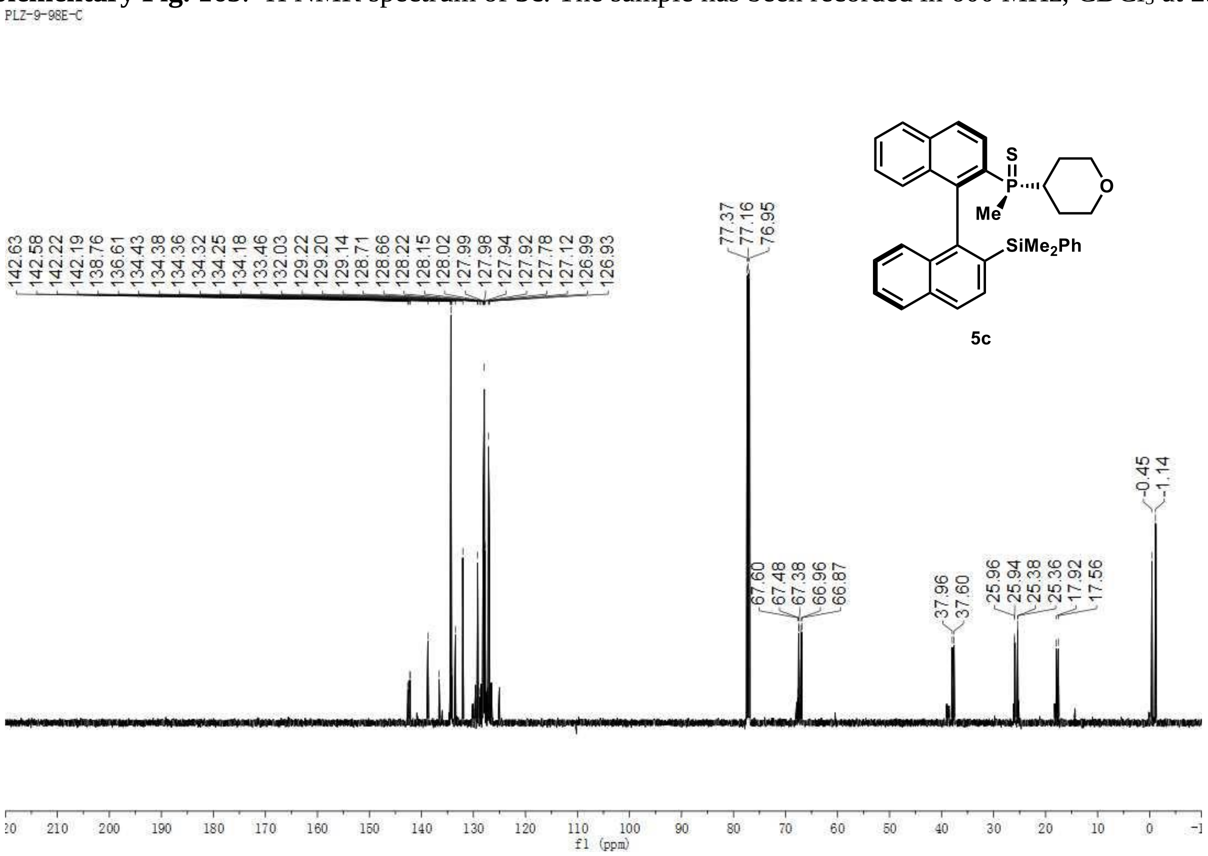

Supplementary Fig. 166.^{13}C NMR spectrum of **5c**. The sample has been recorded in 151 MHz, $CDCl_3$ at 25 ℃.

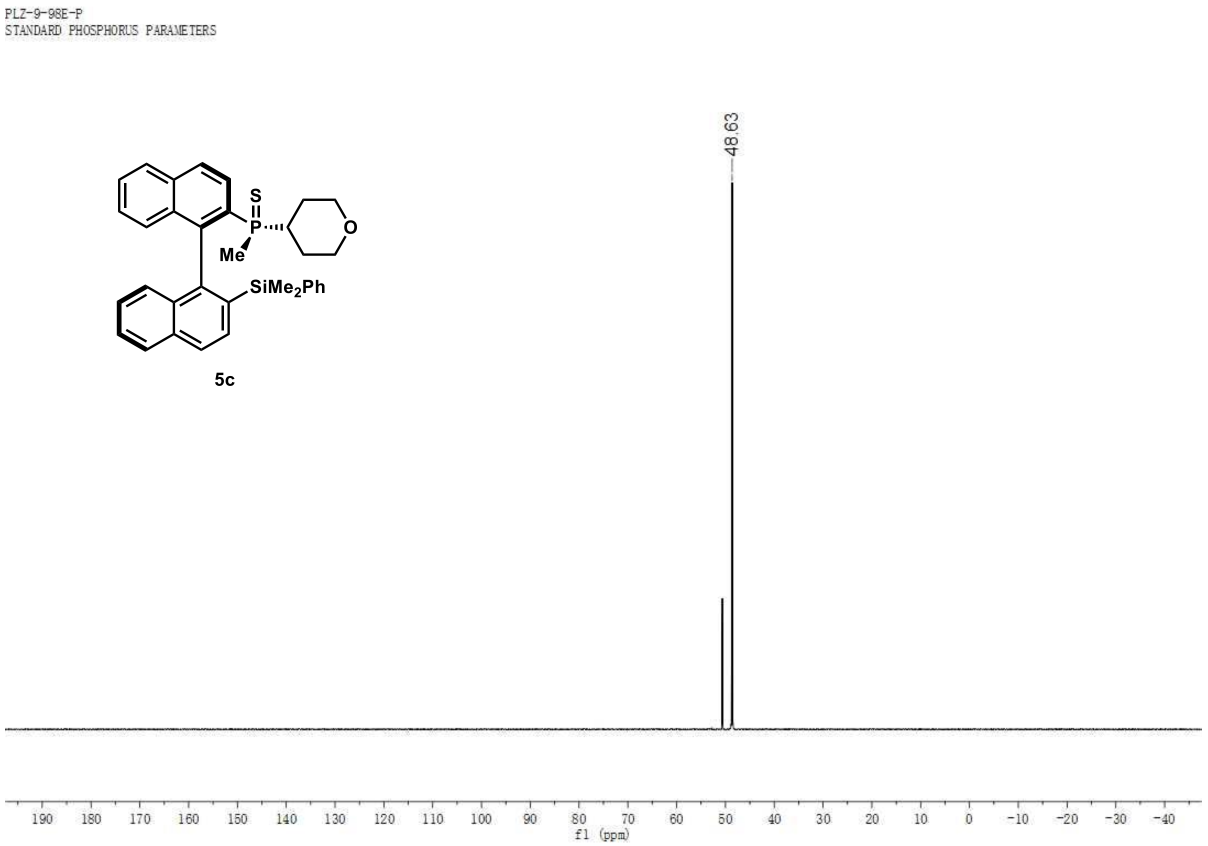

Supplementary Fig. 167.^{31}P NMR spectrum of **5c**. The sample has been recorded in 243 MHz, $CDCl_3$ at 25 ℃.

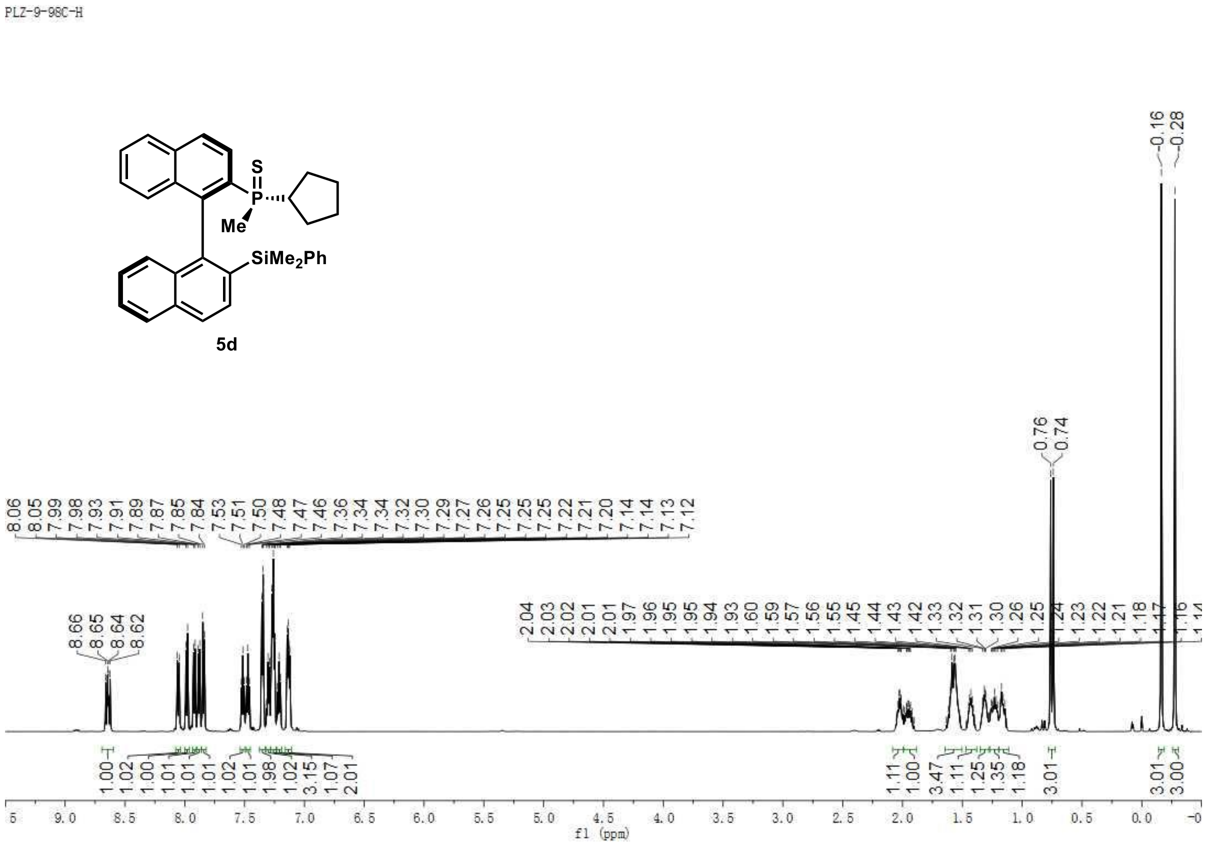

Supplementary Fig. 168. ^{1}H NMR spectrum of **5d**. The sample has been recorded in 600 MHz, $CDCl_3$ at 25 ℃.

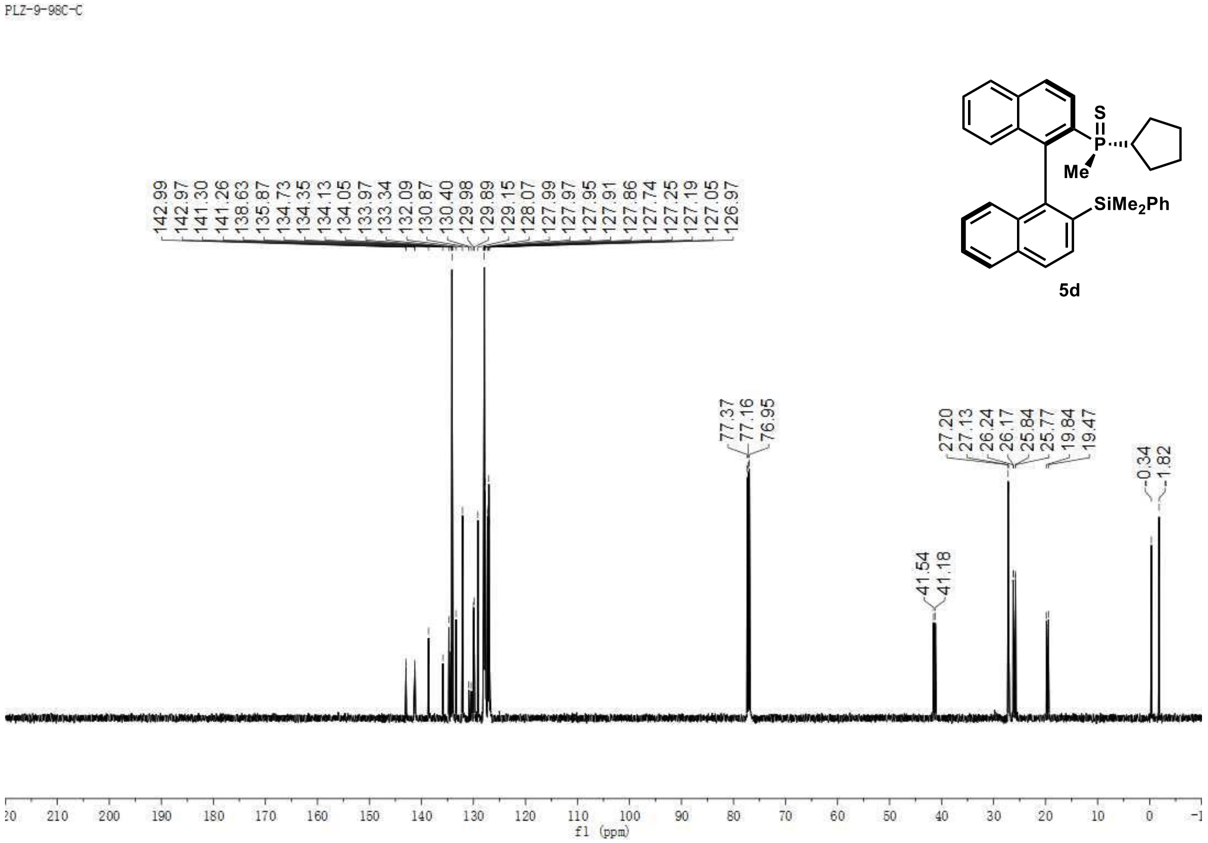

Supplementary Fig. 169.^{13}C NMR spectrum of **5d**. The sample has been recorded in 151 MHz, $CDCl_3$ at 25 ℃.

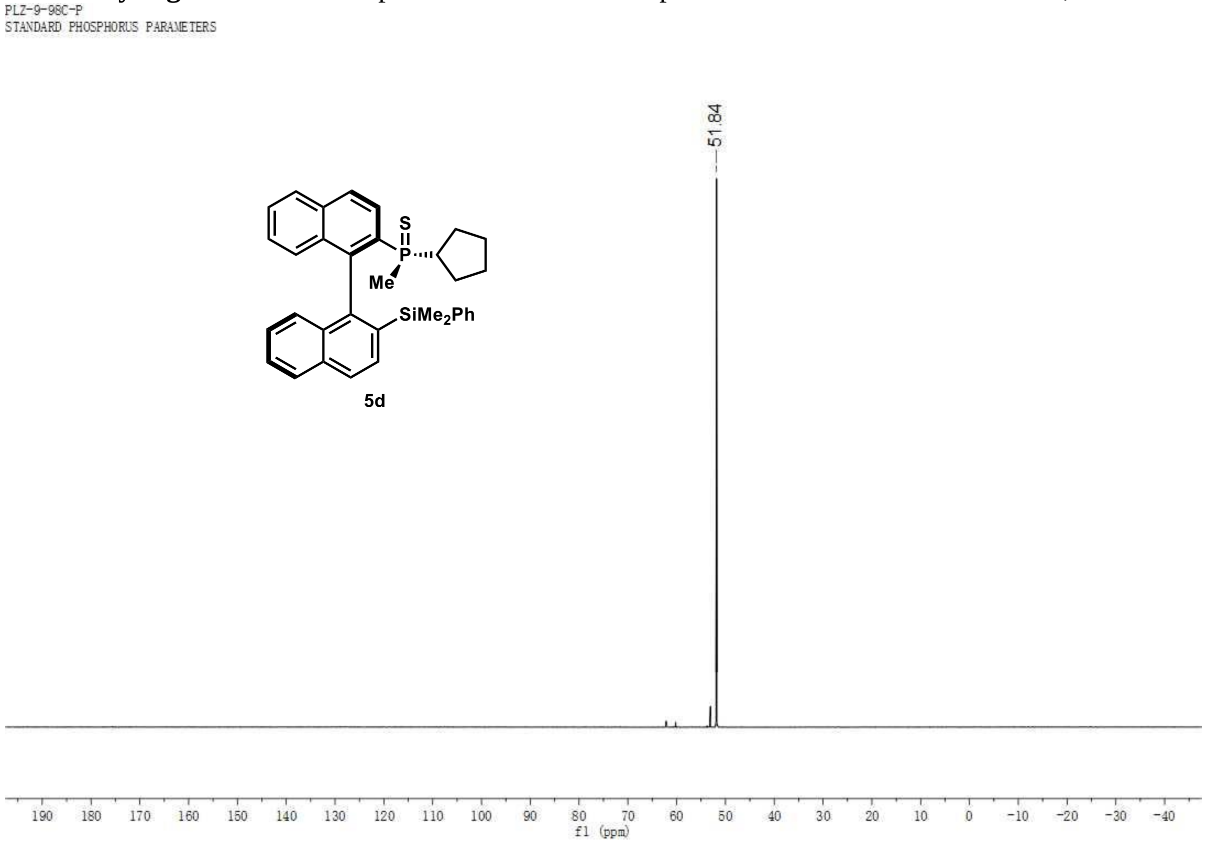

Supplementary Fig. 170.^{31}P NMR spectrum of **5d**. The sample has been recorded in 243 MHz, $CDCl_3$ at 25 ℃.

PLZ-9-101E-H

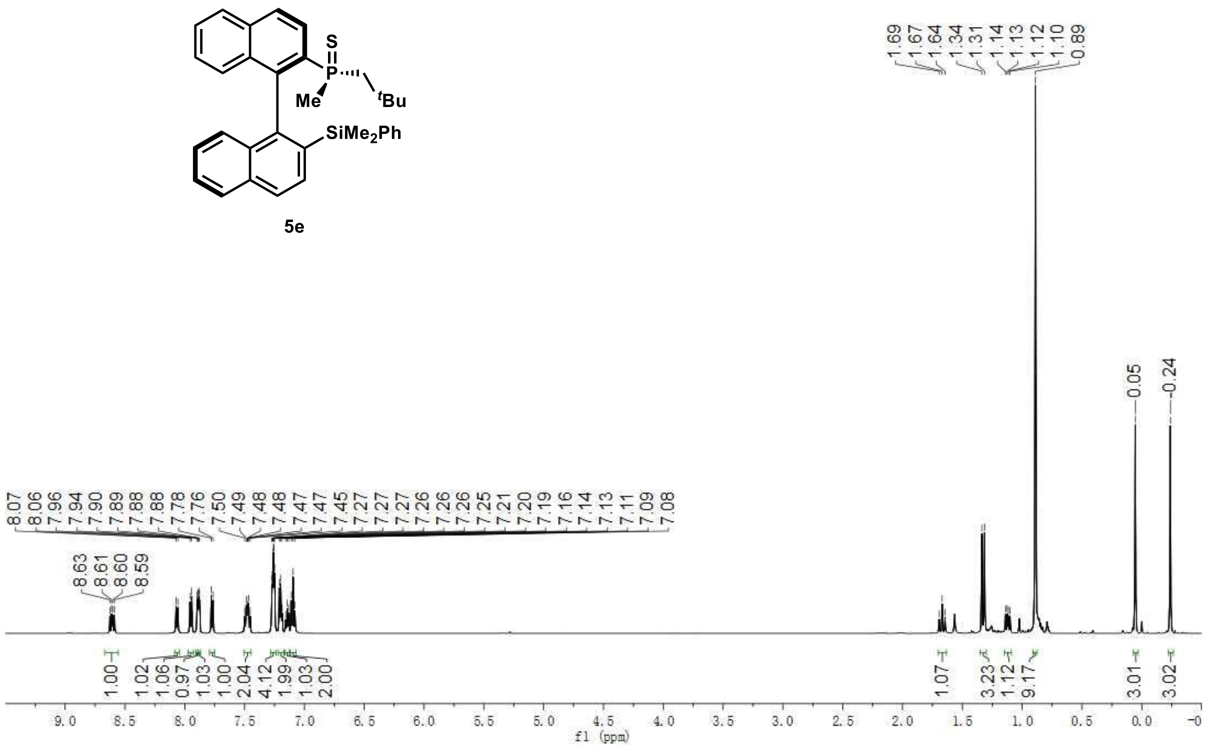

Supplementary Fig. 171. ^{1}H NMR spectrum of **5e**. The sample has been recorded in 600 MHz, $CDCl_3$ at 25 ℃.

PLZ-9-101E-C

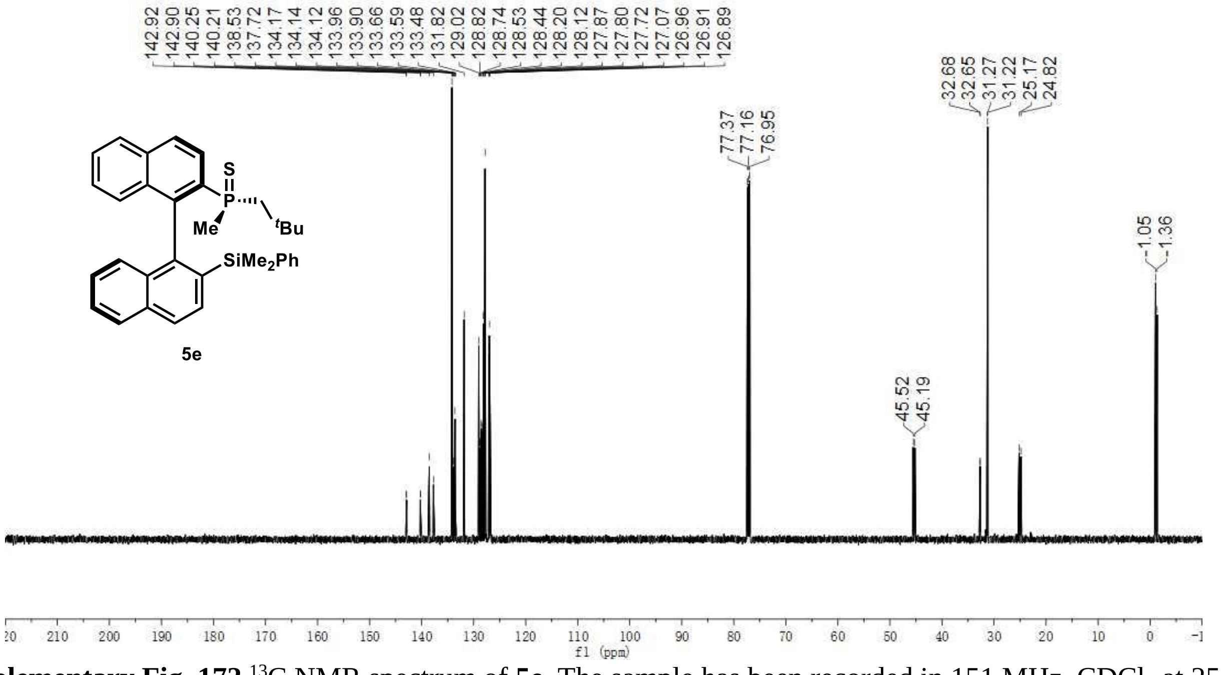

Supplementary Fig. 172.^{13}C NMR spectrum of **5e**. The sample has been recorded in 151 MHz, $CDCl_3$ at 25 ℃.

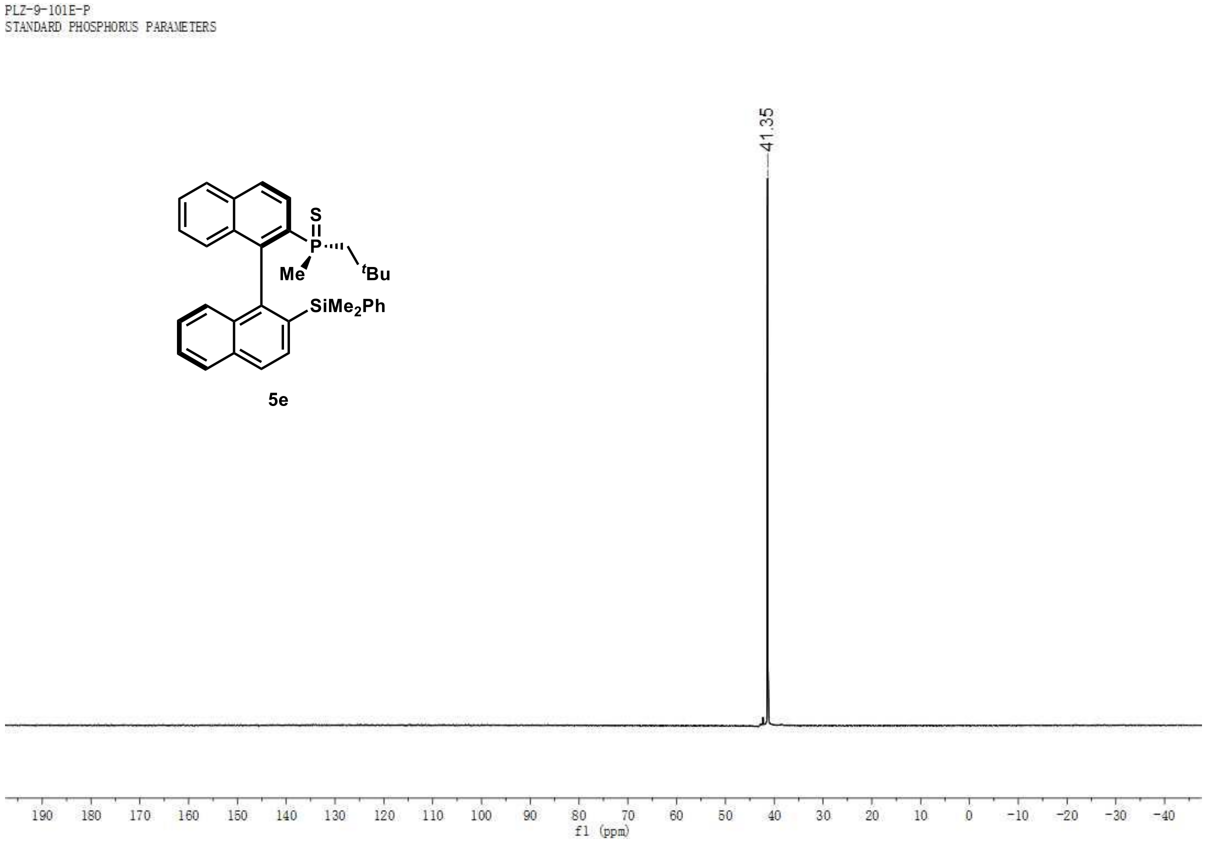

Supplementary Fig. 173.^{31}P NMR spectrum of **5e**. The sample has been recorded in 243 MHz, $CDCl_3$ at 25 ℃.

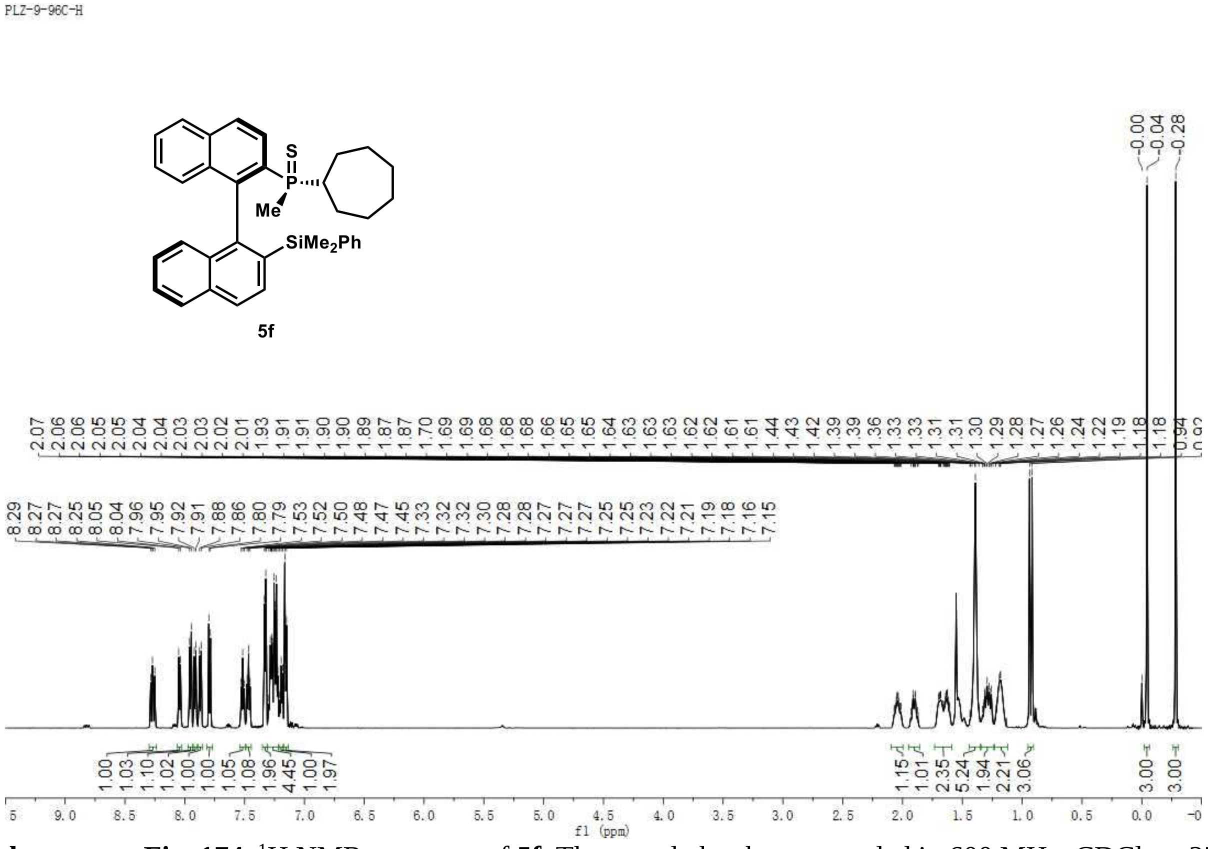

Supplementary Fig. 174. ^{1}H NMR spectrum of **5f**. The sample has been recorded in 600 MHz, $CDCl_3$ at 25 ℃.

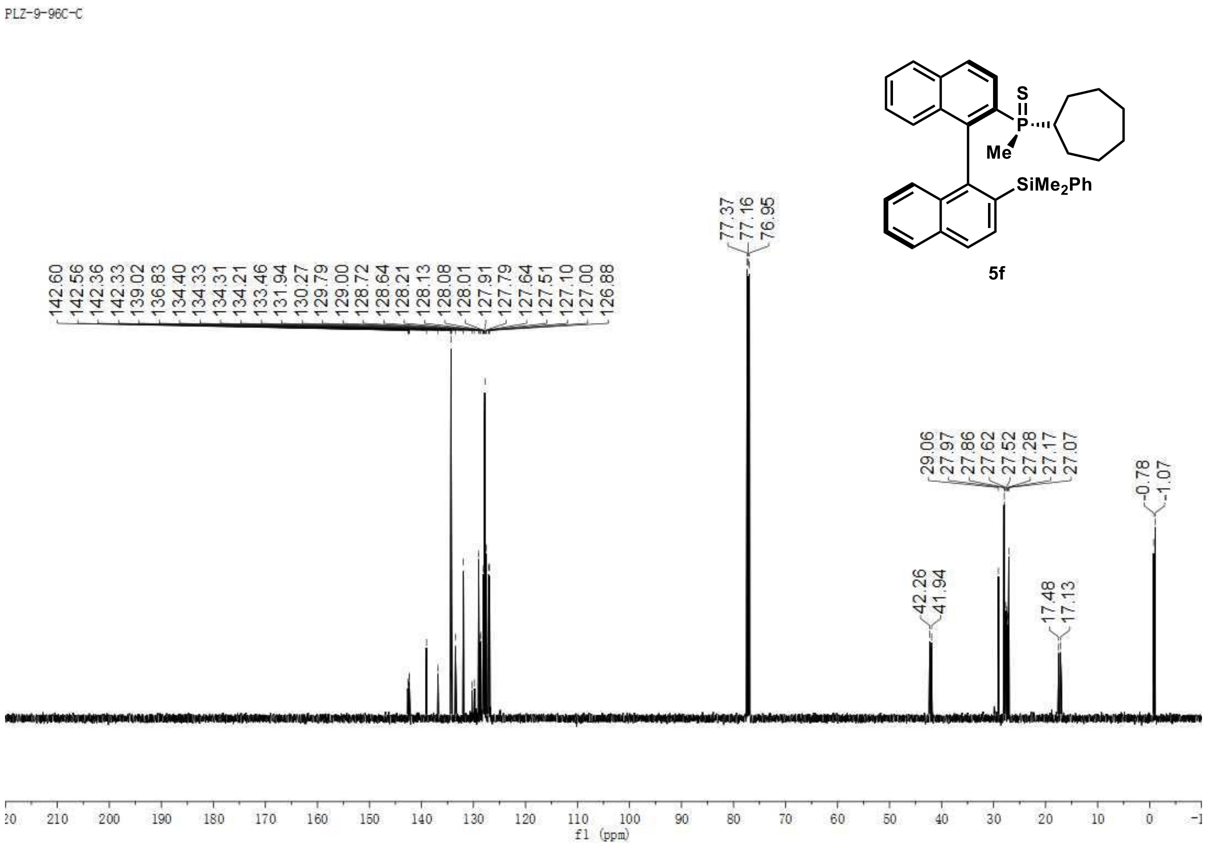

Supplementary Fig. 175.^{13}C NMR spectrum of **5f**. The sample has been recorded in 151 MHz, $CDCl_3$ at 25 ℃.

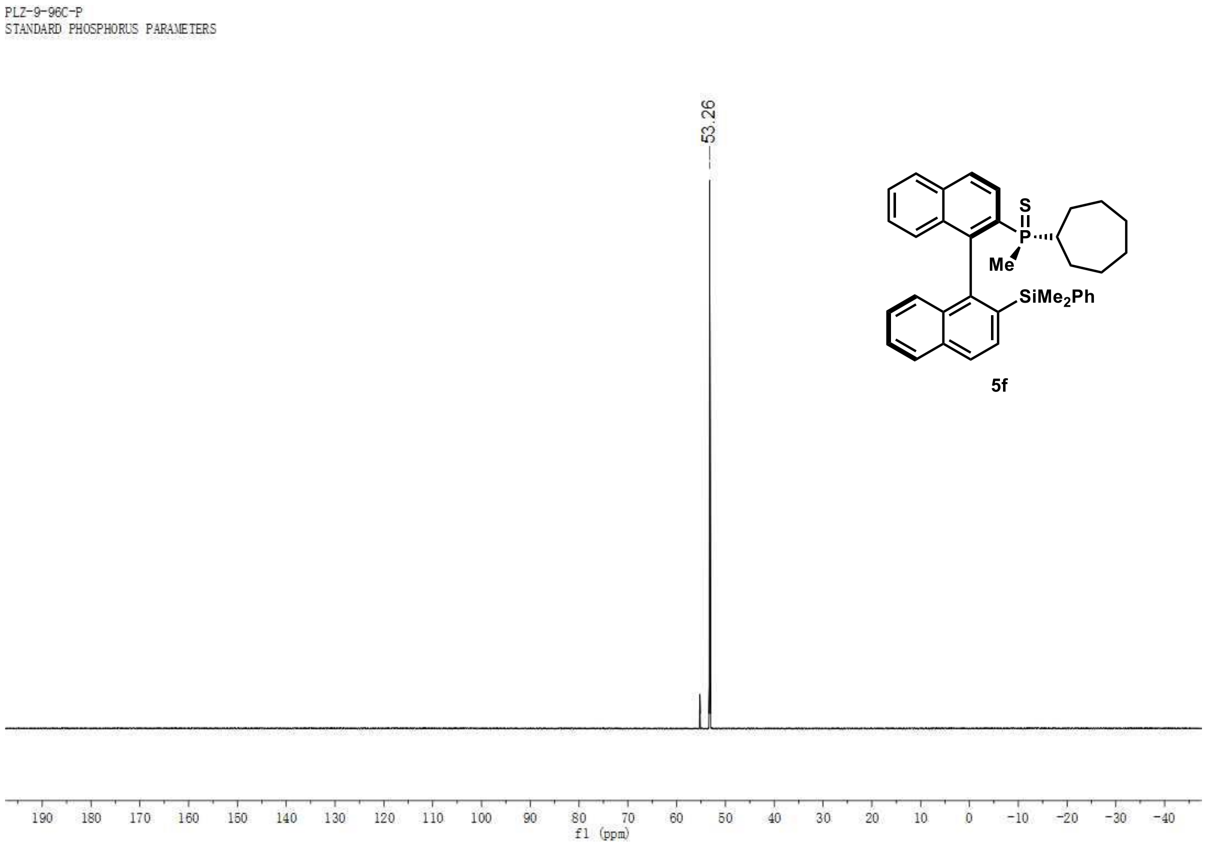

Supplementary Fig. 176.^{31}P NMR spectrum of **5f**. The sample has been recorded in 243 MHz, $CDCl_3$ at 25 ℃.

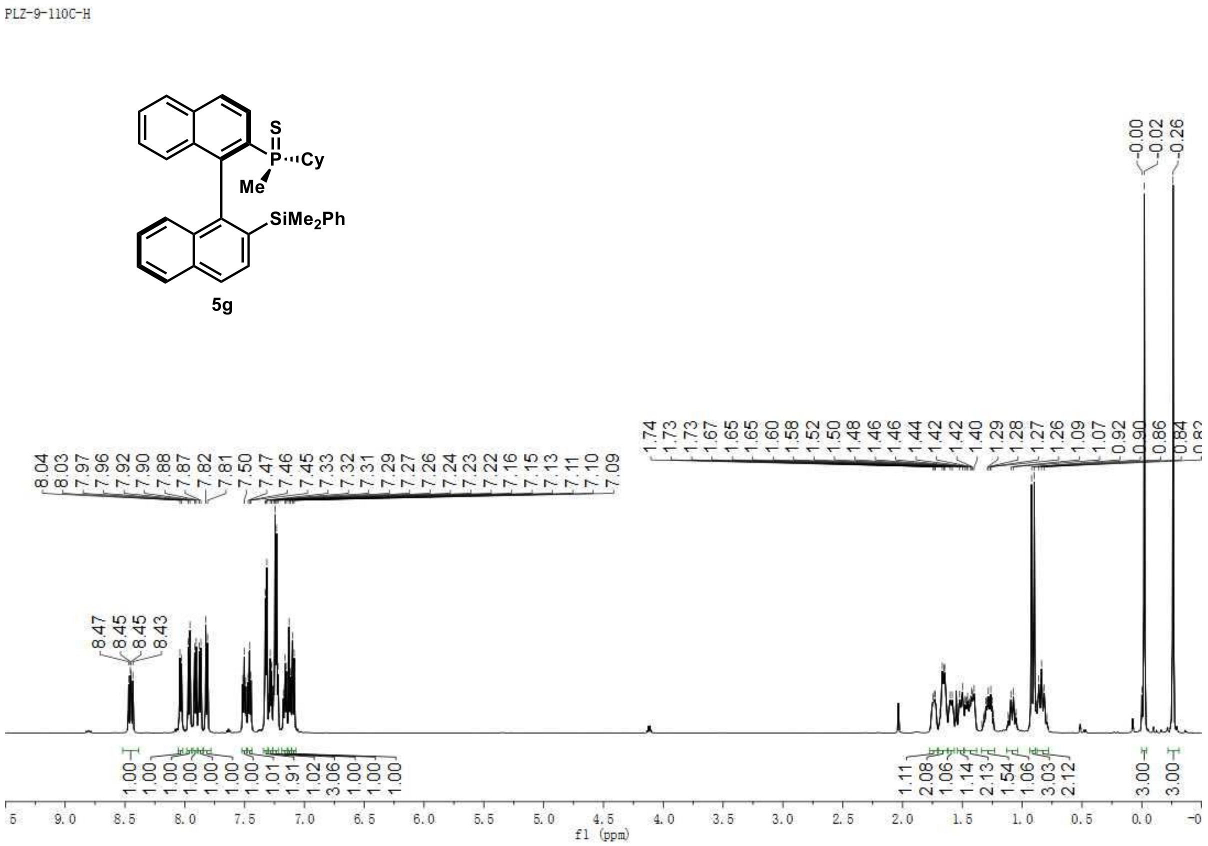

Supplementary Fig. 177. ^{1}H NMR spectrum of **5g**. The sample has been recorded in 600 MHz, $CDCl_3$ at 25 ℃.

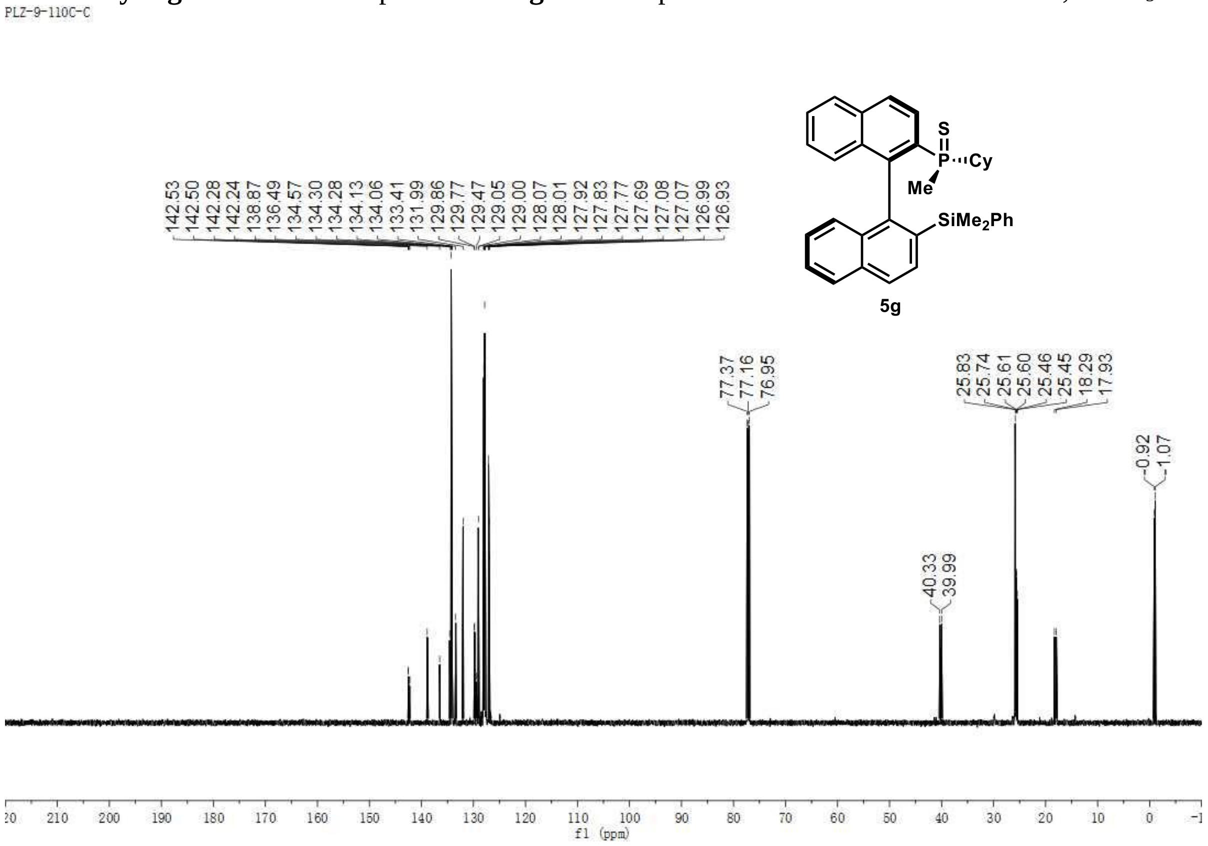

Supplementary Fig. 178.^{13}C NMR spectrum of **5g**. The sample has been recorded in 151 MHz, $CDCl_3$ at 25 ℃.

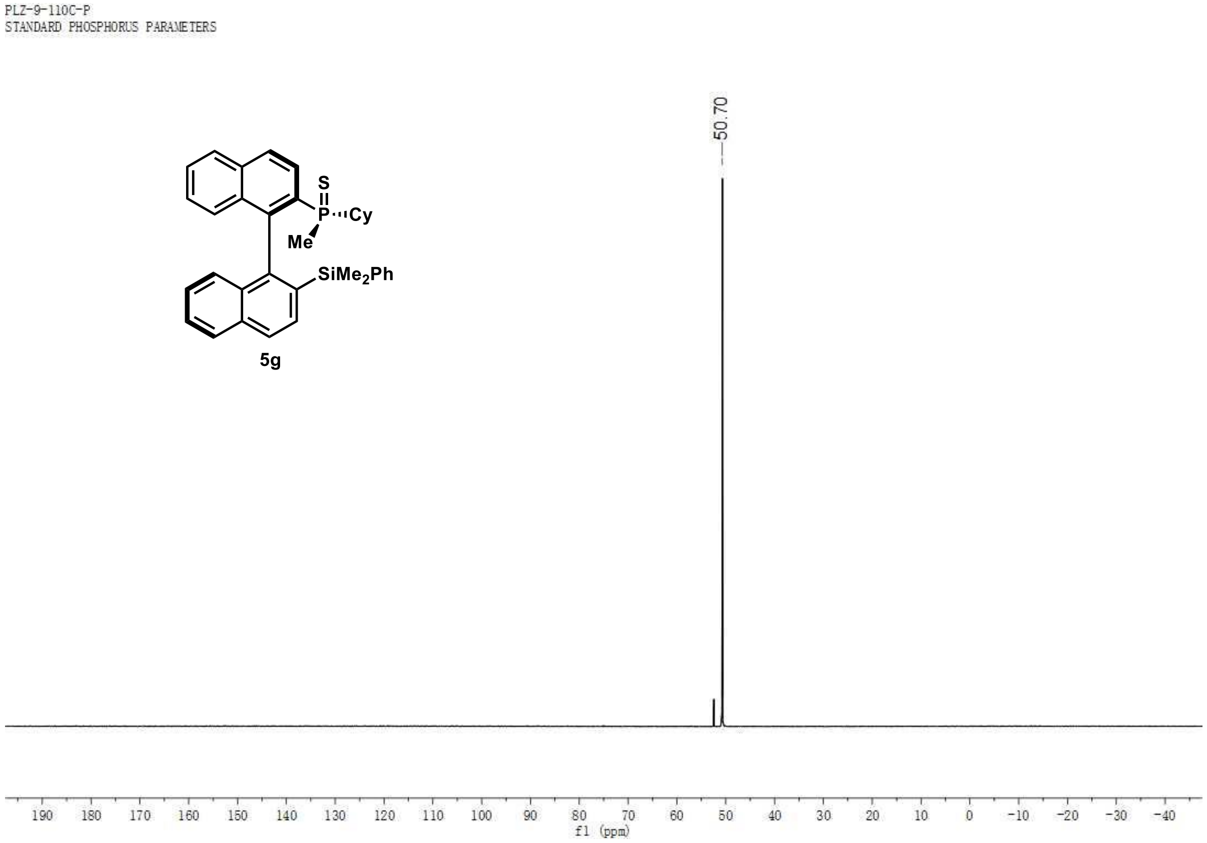

Supplementary Fig. 179.^{31}P NMR spectrum of **5g**. The sample has been recorded in 243 MHz, $CDCl_3$ at 25 ℃.

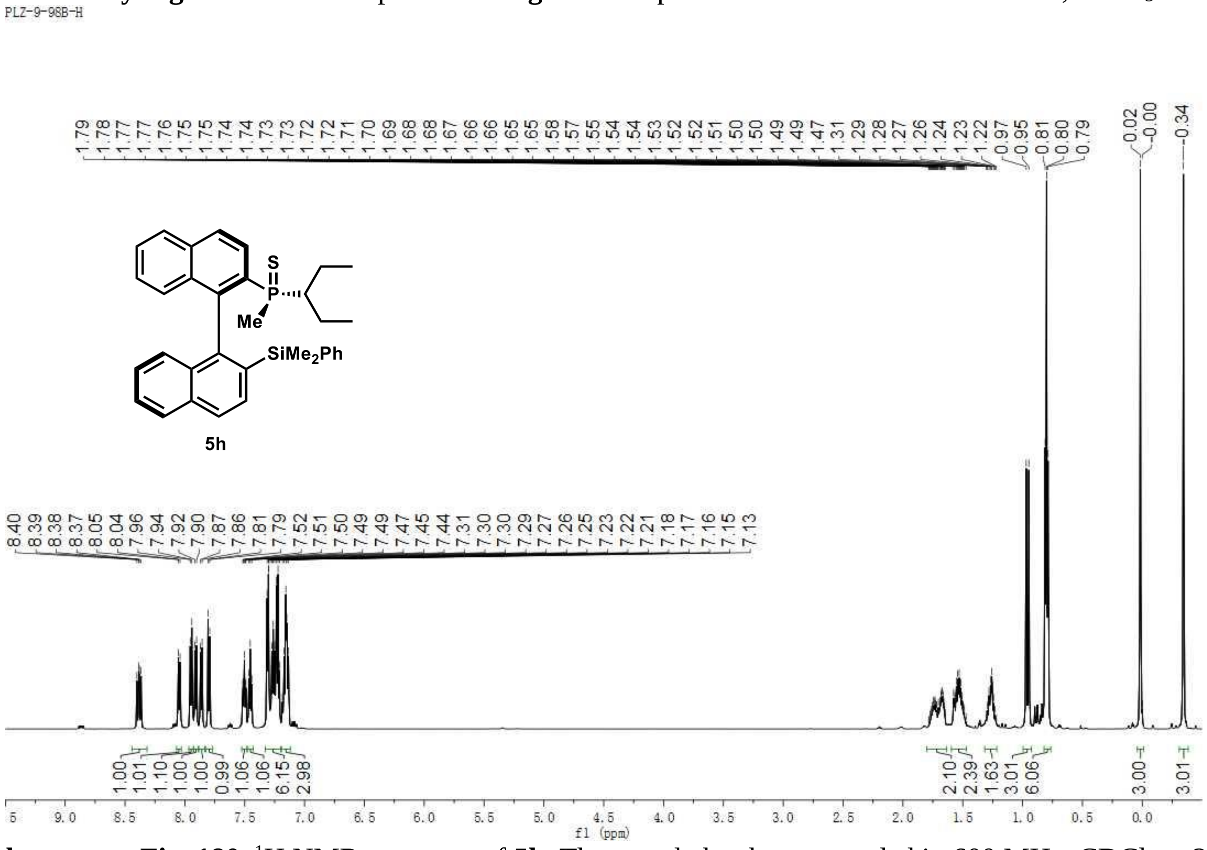

Supplementary Fig. 180. ^{1}H NMR spectrum of **5h**. The sample has been recorded in 600 MHz, $CDCl_3$ at 25 ℃.

PLZ-9-98B-C

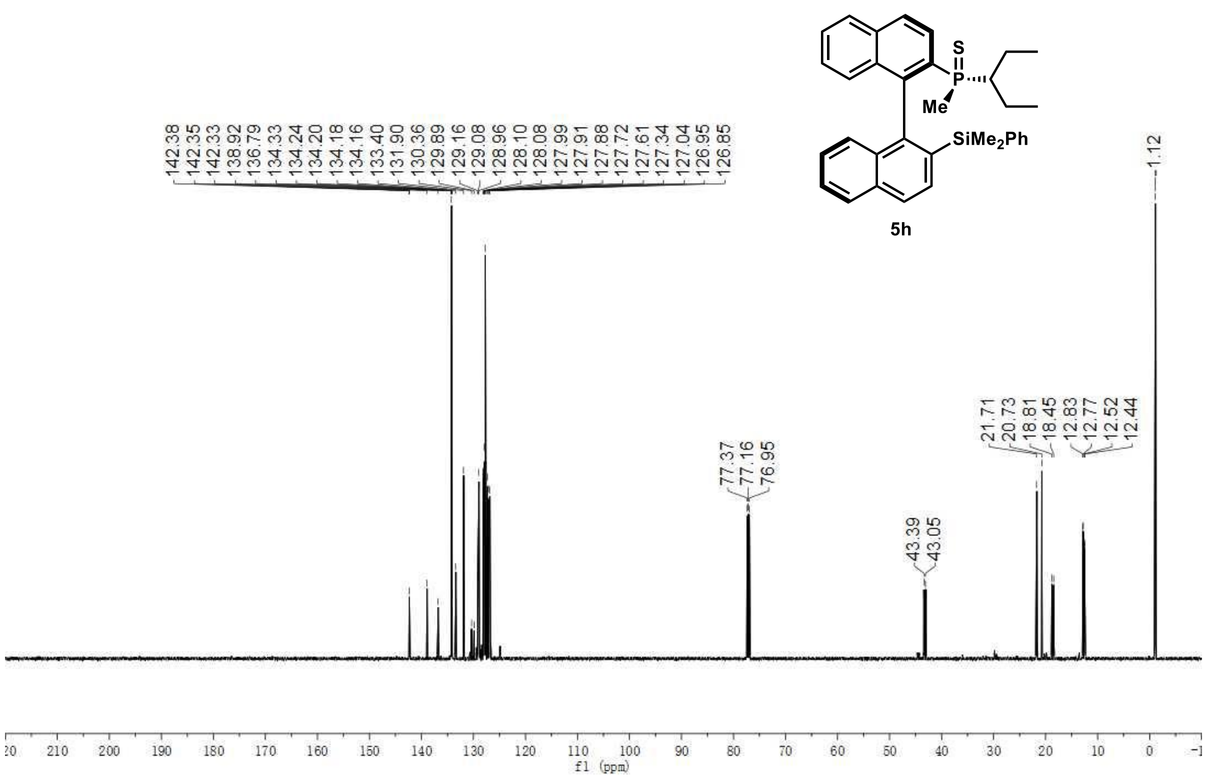

Supplementary Fig. 181.^{13}C NMR spectrum of **5h**. The sample has been recorded in 151 MHz, $CDCl_3$ at 25 ℃.

PLZ-9-98B-P
STANDARD PHOSPHORUS PARAMETERS

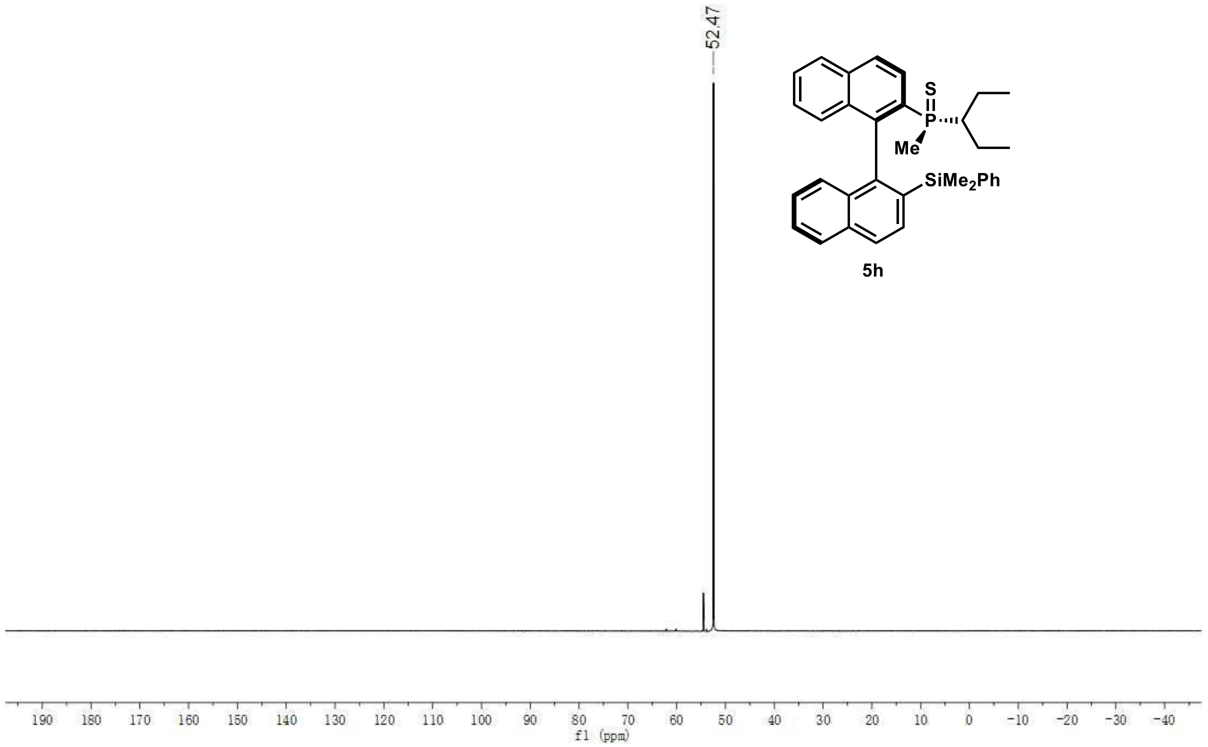

Supplementary Fig. 182.^{31}P NMR spectrum of **5h**. The sample has been recorded in 243 MHz, $CDCl_3$ at 25 ℃.

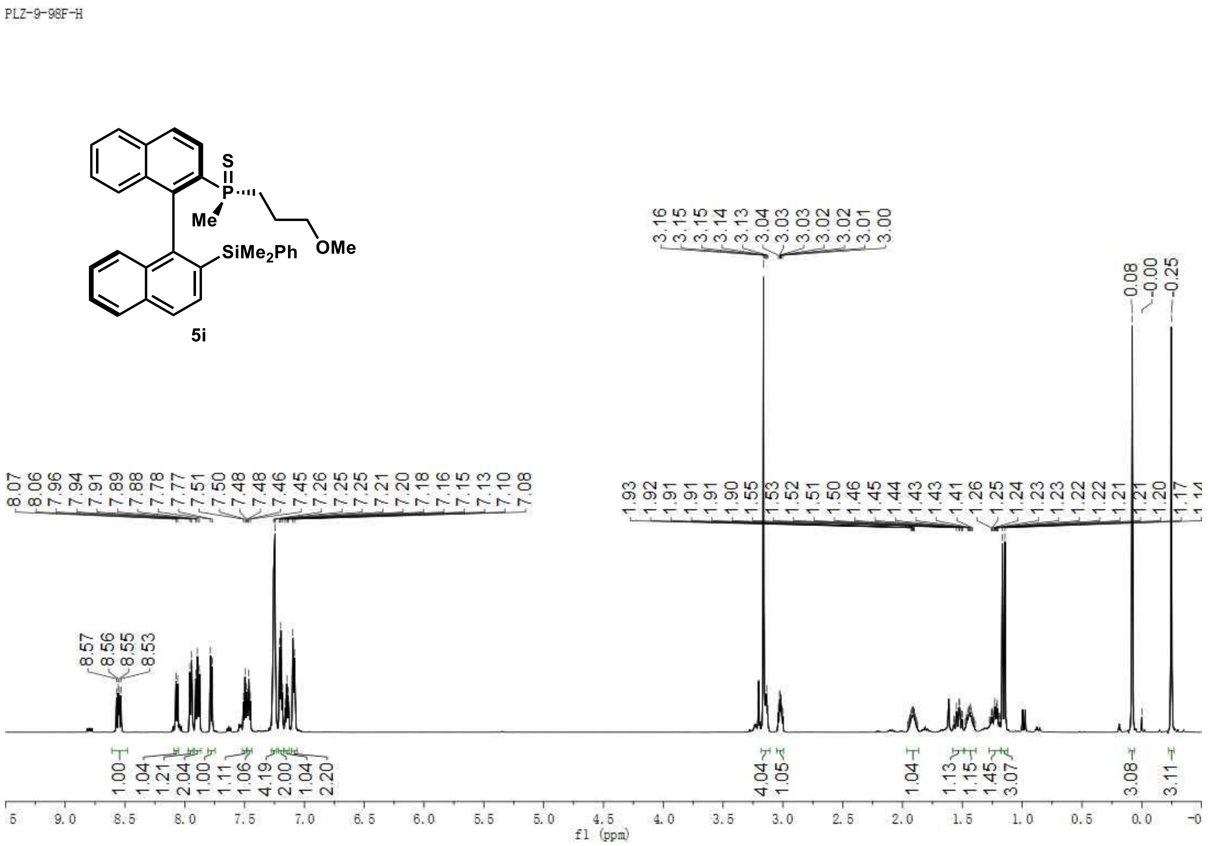

Supplementary Fig. 183. ^{1}H NMR spectrum of **5i**. The sample has been recorded in 600 MHz, $CDCl_3$ at 25 ℃.

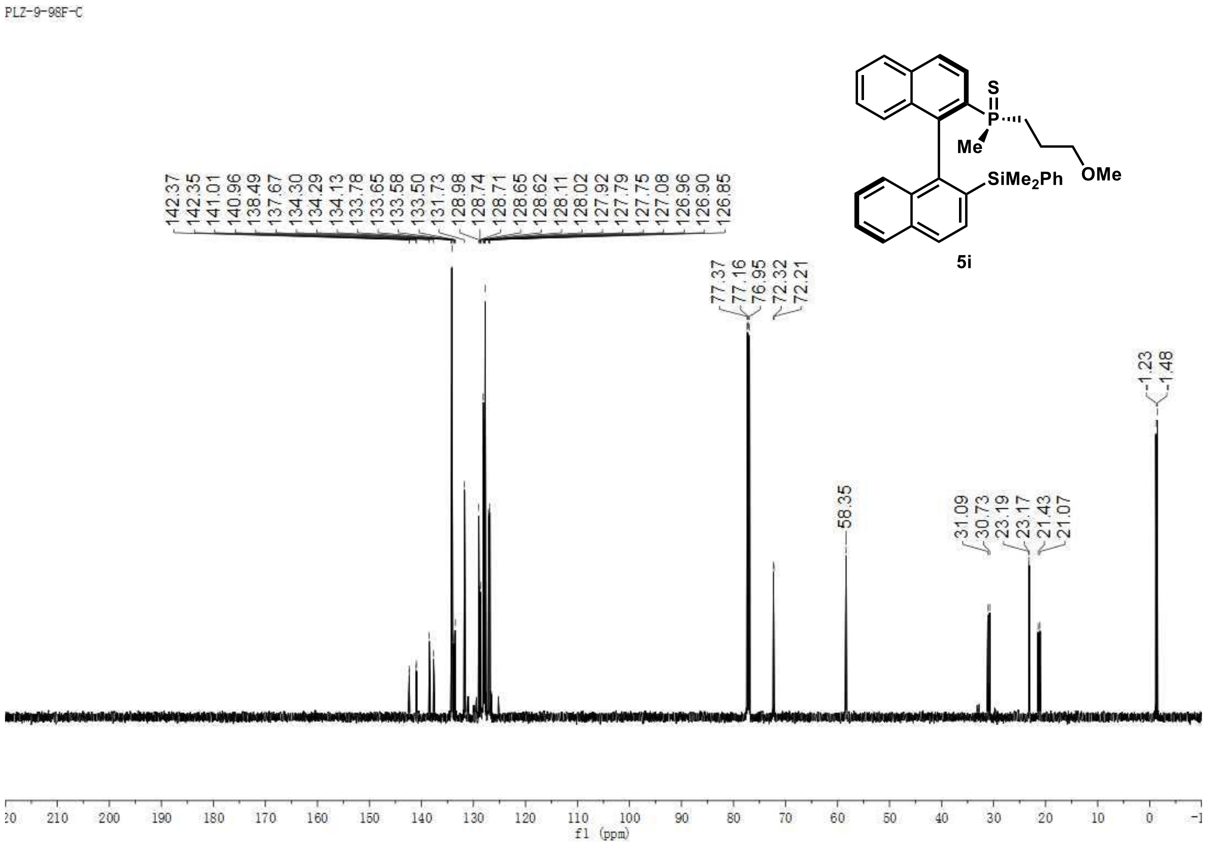

Supplementary Fig. 184.^{13}C NMR spectrum of **5i**. The sample has been recorded in 151 MHz, $CDCl_3$ at 25 ℃.

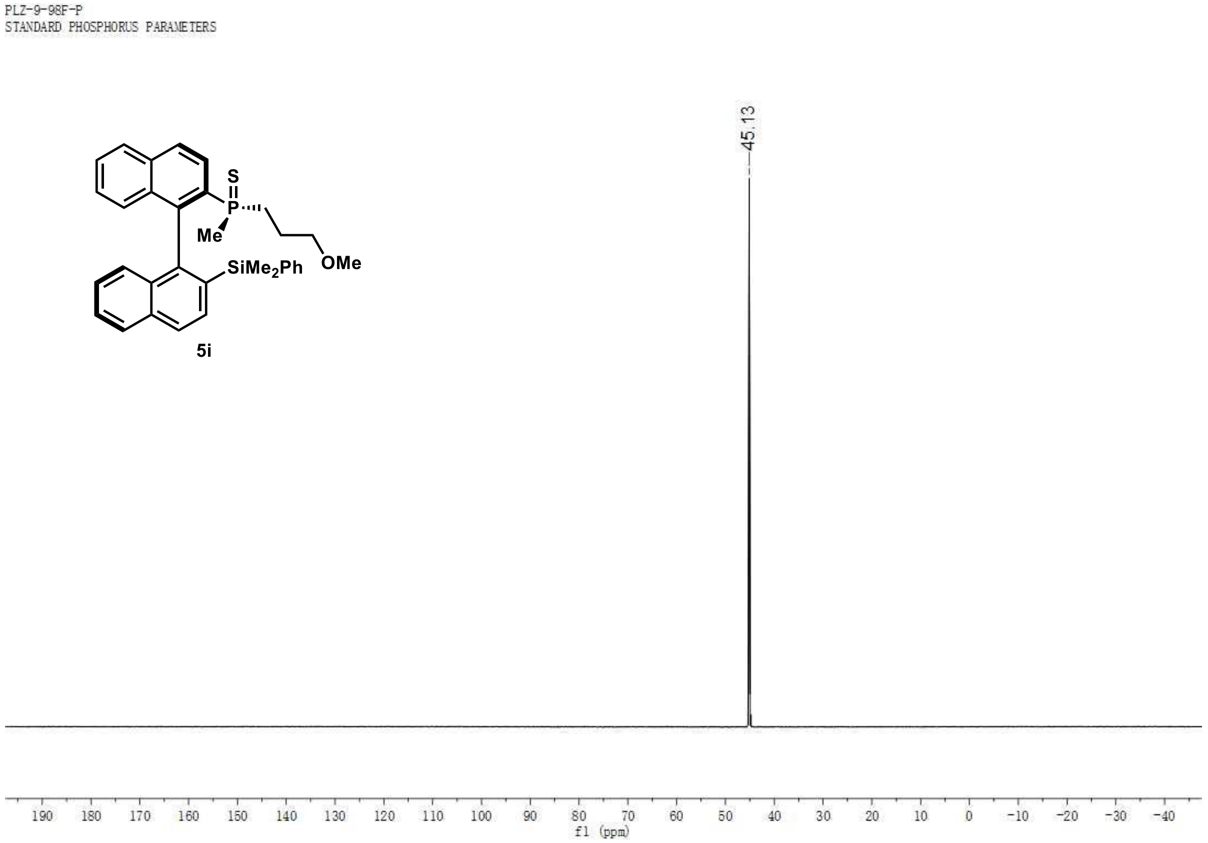

Supplementary Fig. 185.^{31}P NMR spectrum of **5i**. The sample has been recorded in 243 MHz, $CDCl_3$ at 25 ℃.

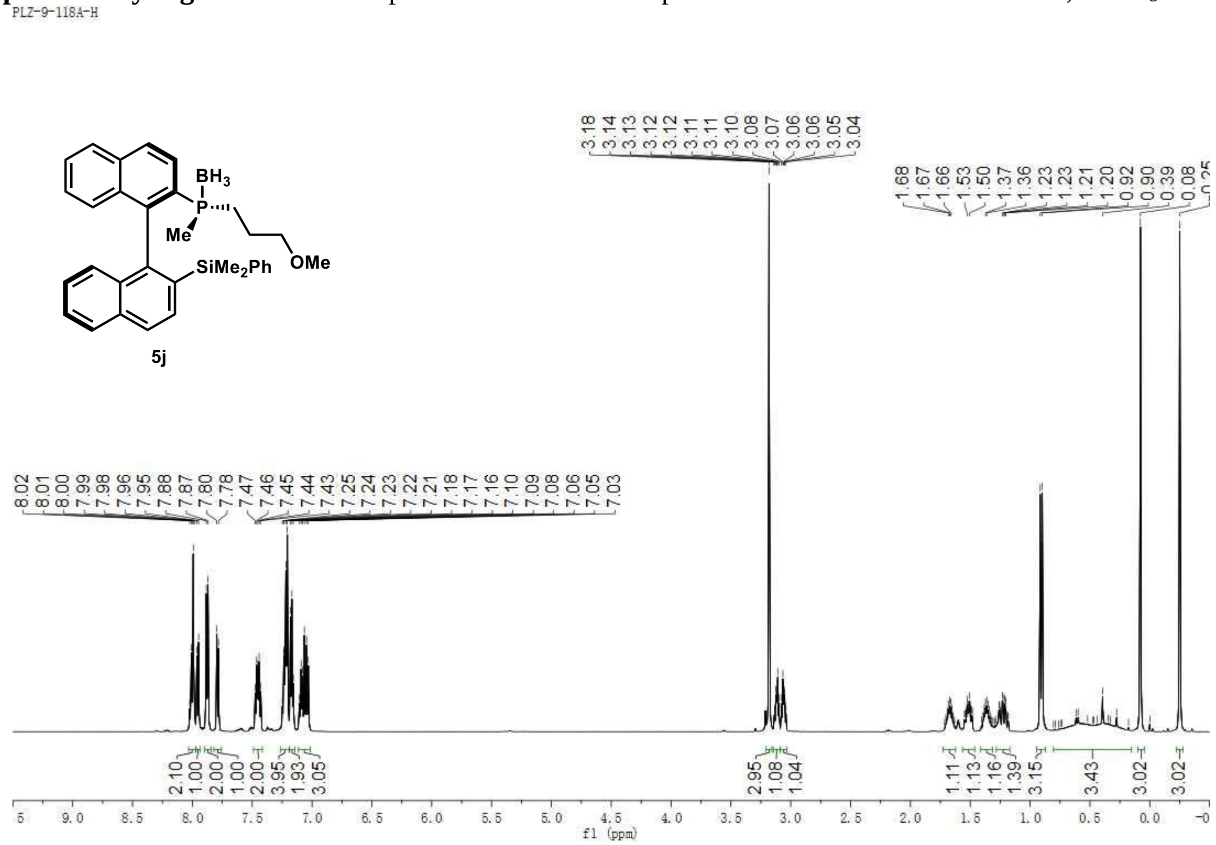

Supplementary Fig. 186. ^{1}H NMR spectrum of **5j**. The sample has been recorded in 600 MHz, $CDCl_3$ at 25 ℃.

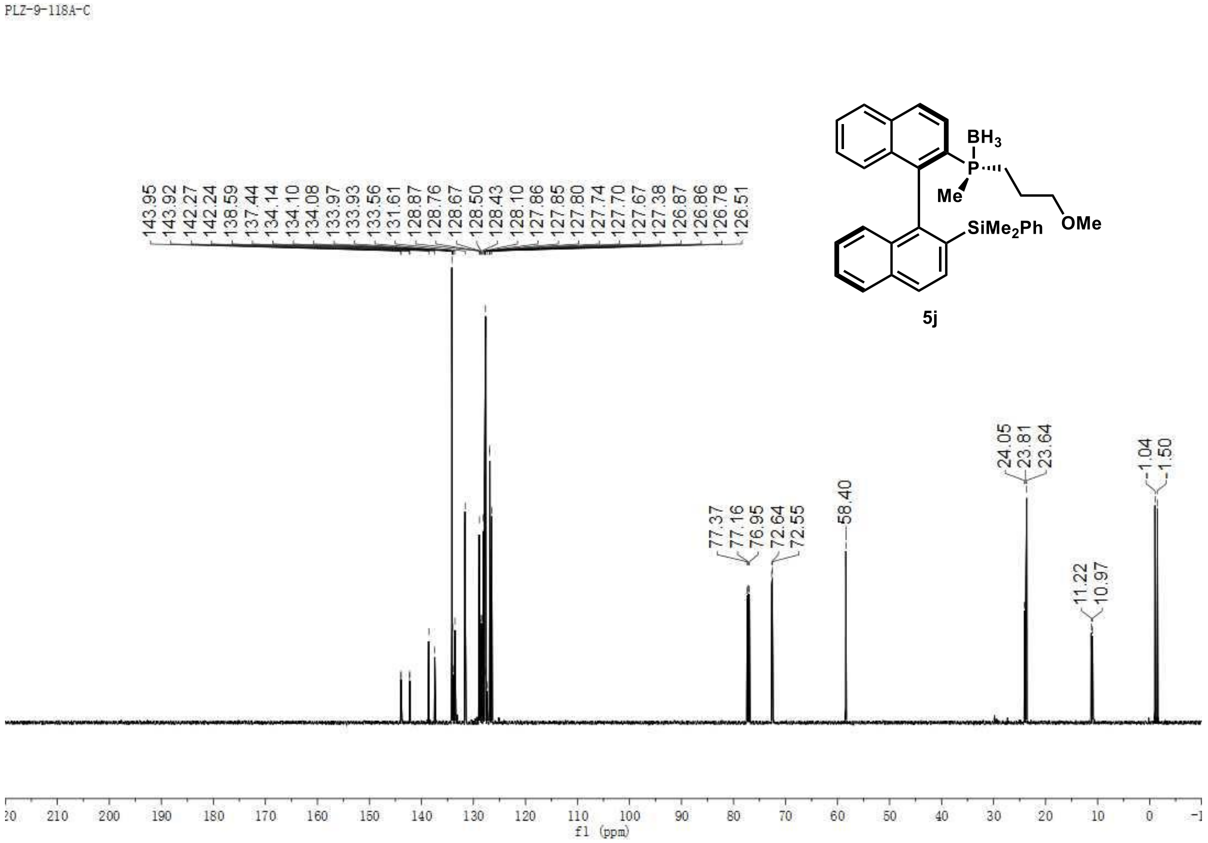

Supplementary Fig. 187.^{13}C NMR spectrum of **5j**. The sample has been recorded in 151 MHz, $CDCl_3$ at 25 ℃.

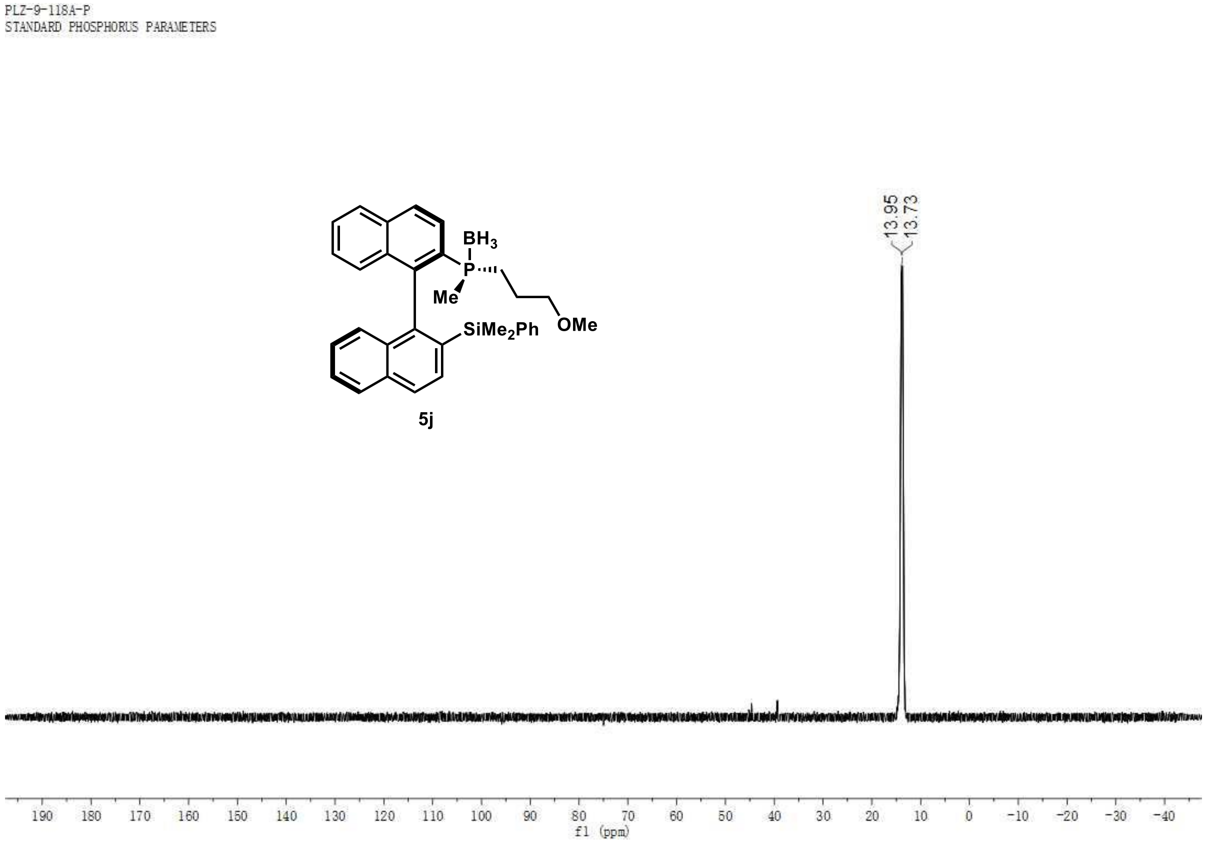

Supplementary Fig. 188.^{31}P NMR spectrum of **5j**. The sample has been recorded in 243 MHz, $CDCl_3$ at 25 ℃.

PLZ-9-98D-H

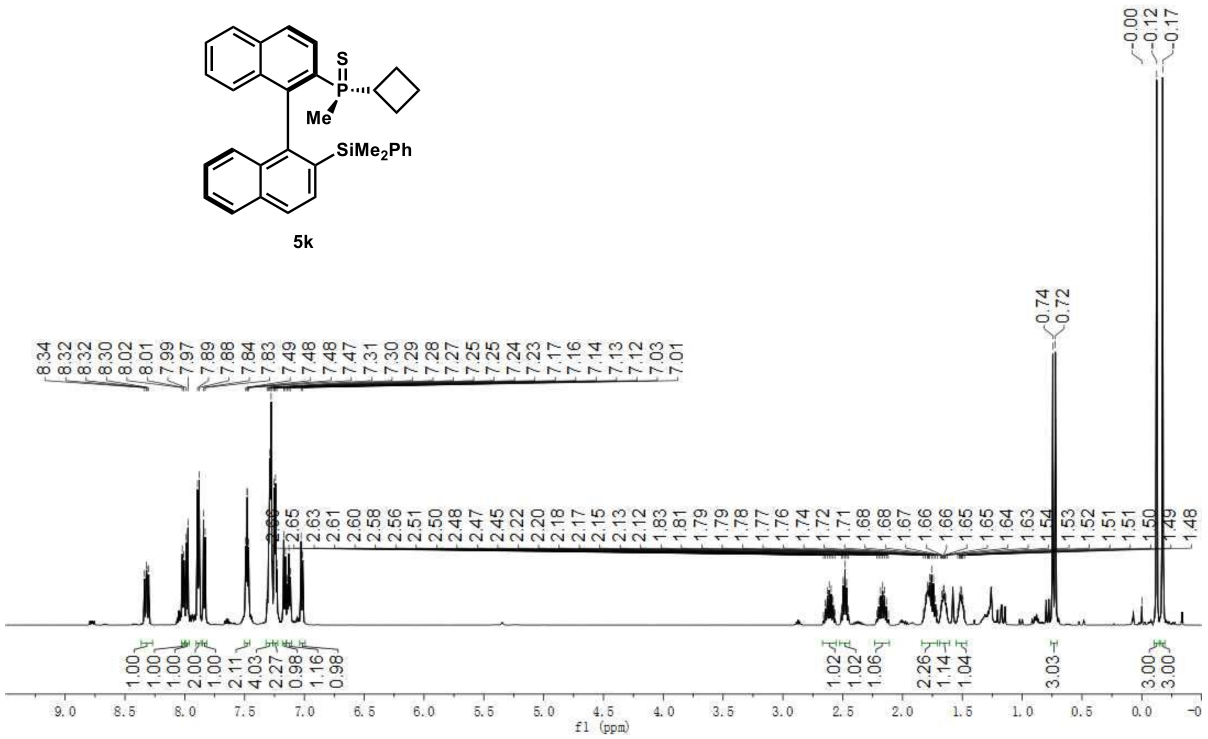

Supplementary Fig. 189. ^{1}H NMR spectrum of **5k**. The sample has been recorded in 600 MHz, $CDCl_3$ at 25 ℃.

PLZ-9-98D-C

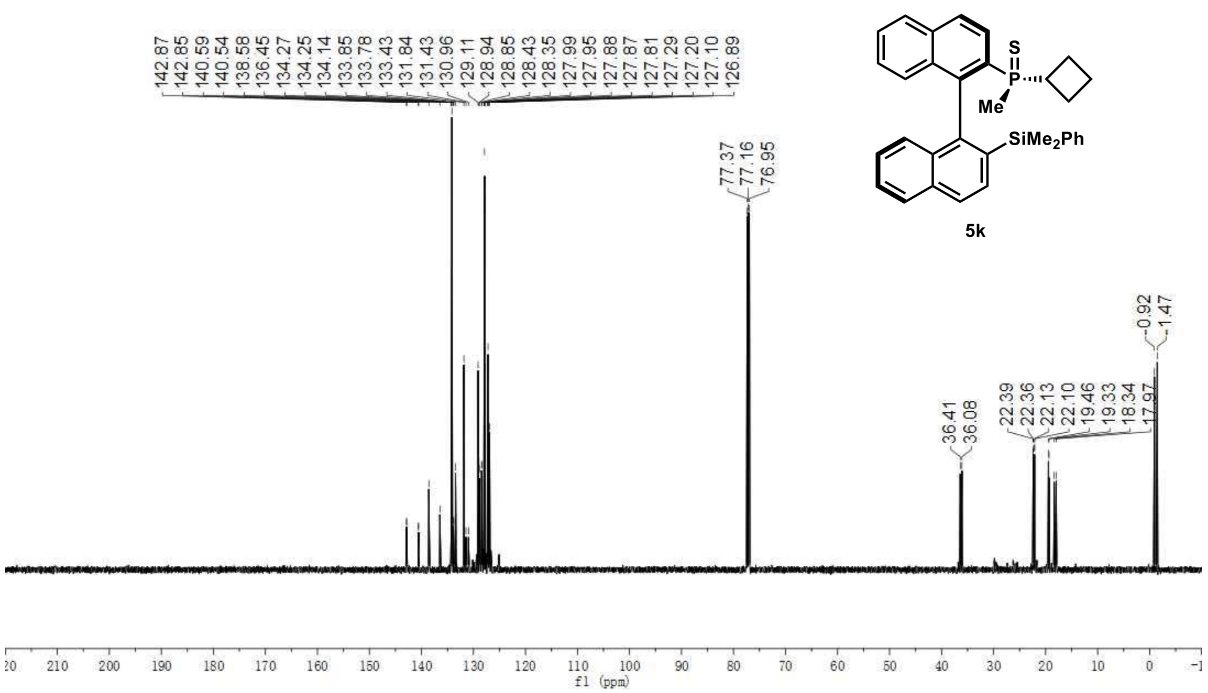

Supplementary Fig. 190. ^{13}C NMR spectrum of **5k**. The sample has been recorded in 151 MHz, $CDCl_3$ at 25 ℃.

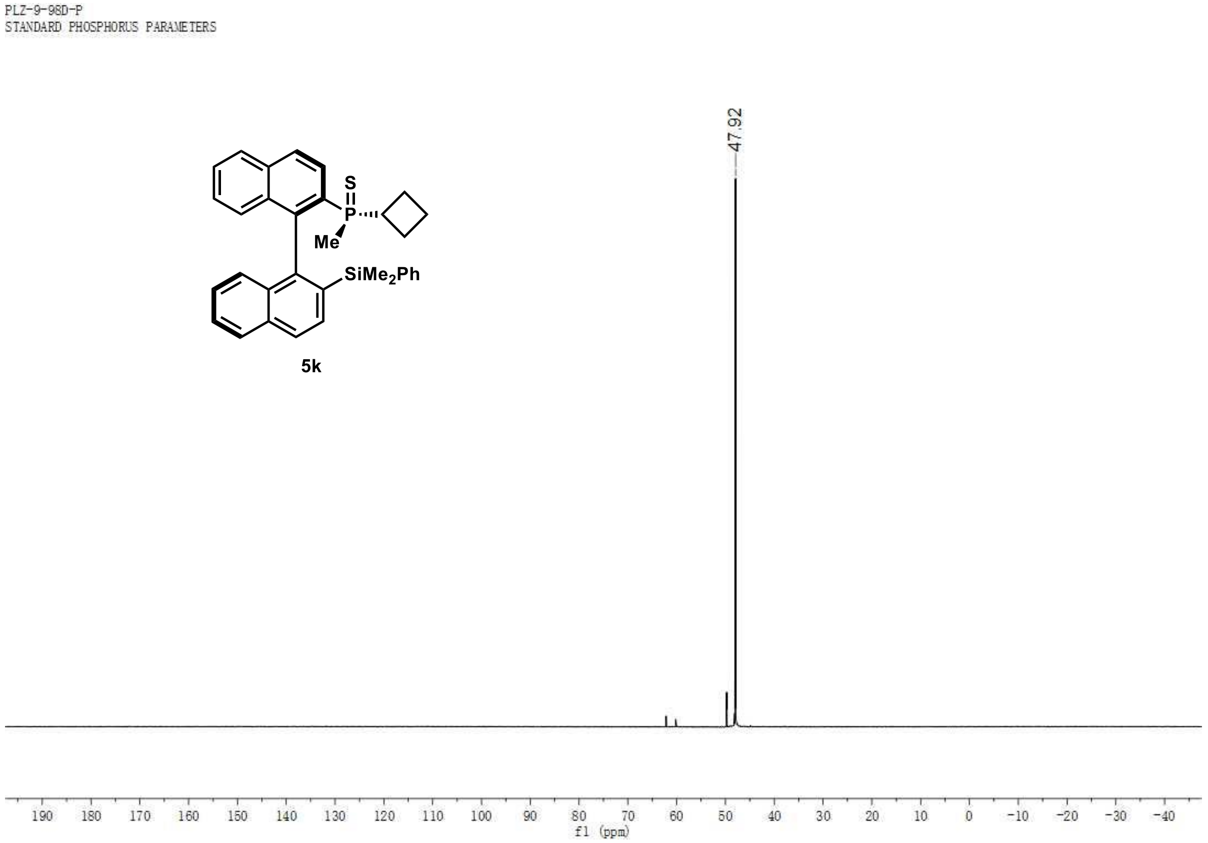

Supplementary Fig. 191.^{31}P NMR spectrum of **5k**. The sample has been recorded in 243 MHz, $CDCl_3$ at 25 ℃.

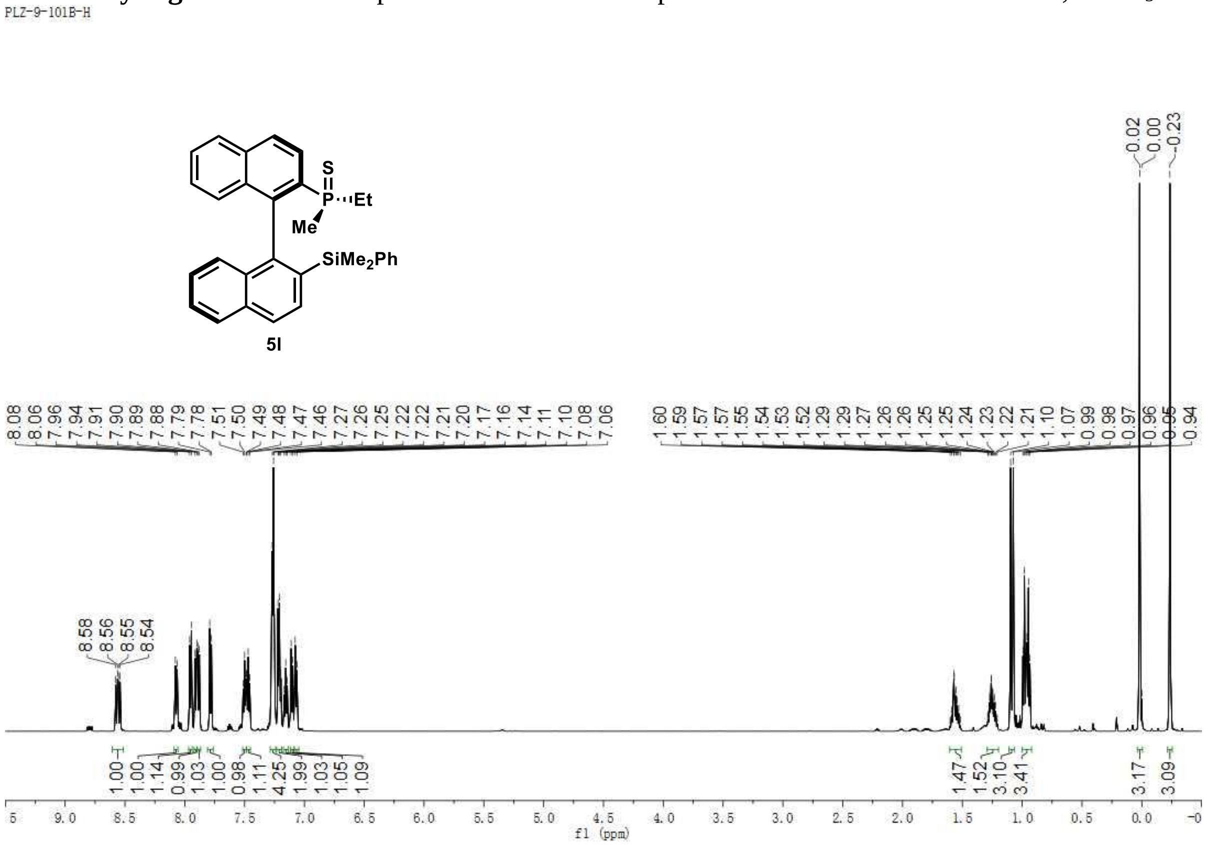

Supplementary Fig. 192. ^{1}H NMR spectrum of **5l**. The sample has been recorded in 600 MHz, $CDCl_3$ at 25 ℃.

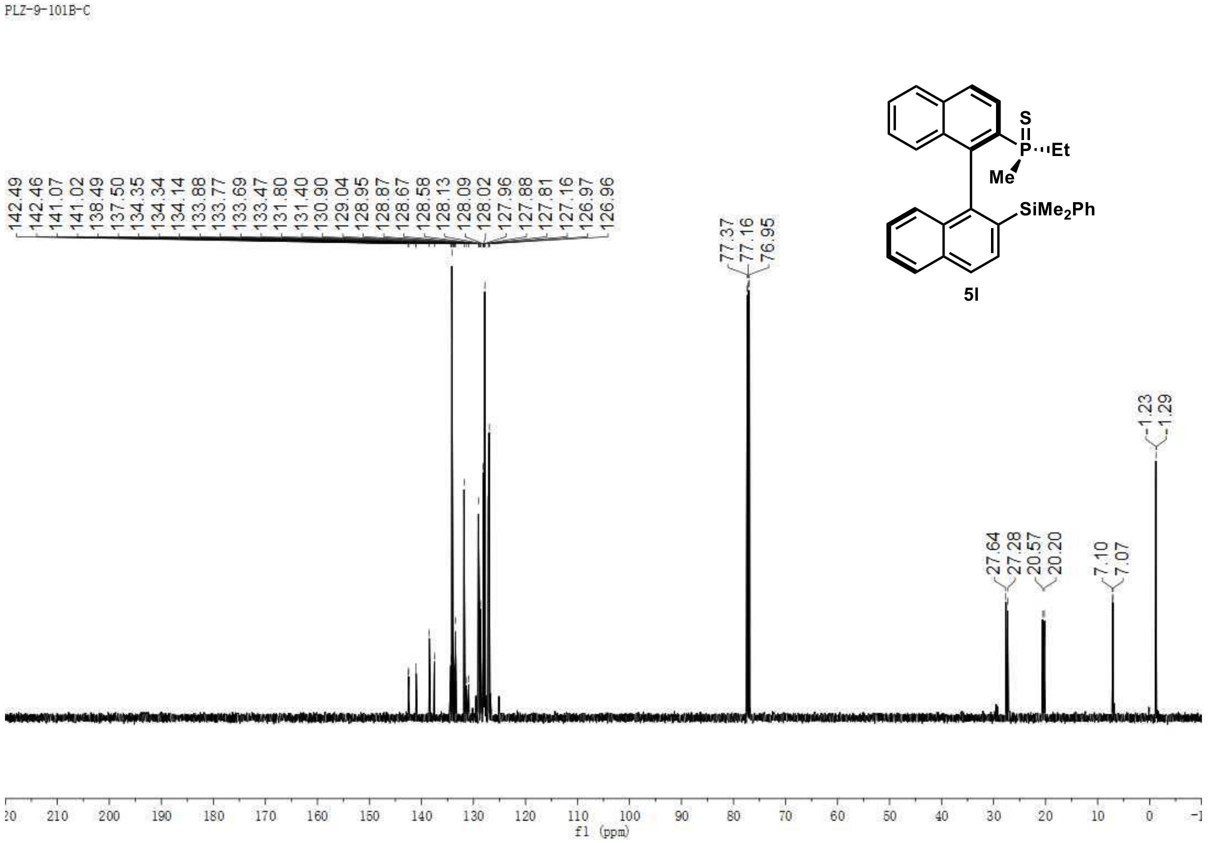

Supplementary Fig. 193.^{13}C NMR spectrum of **5l**. The sample has been recorded in 151 MHz, $CDCl_3$ at 25 ℃.

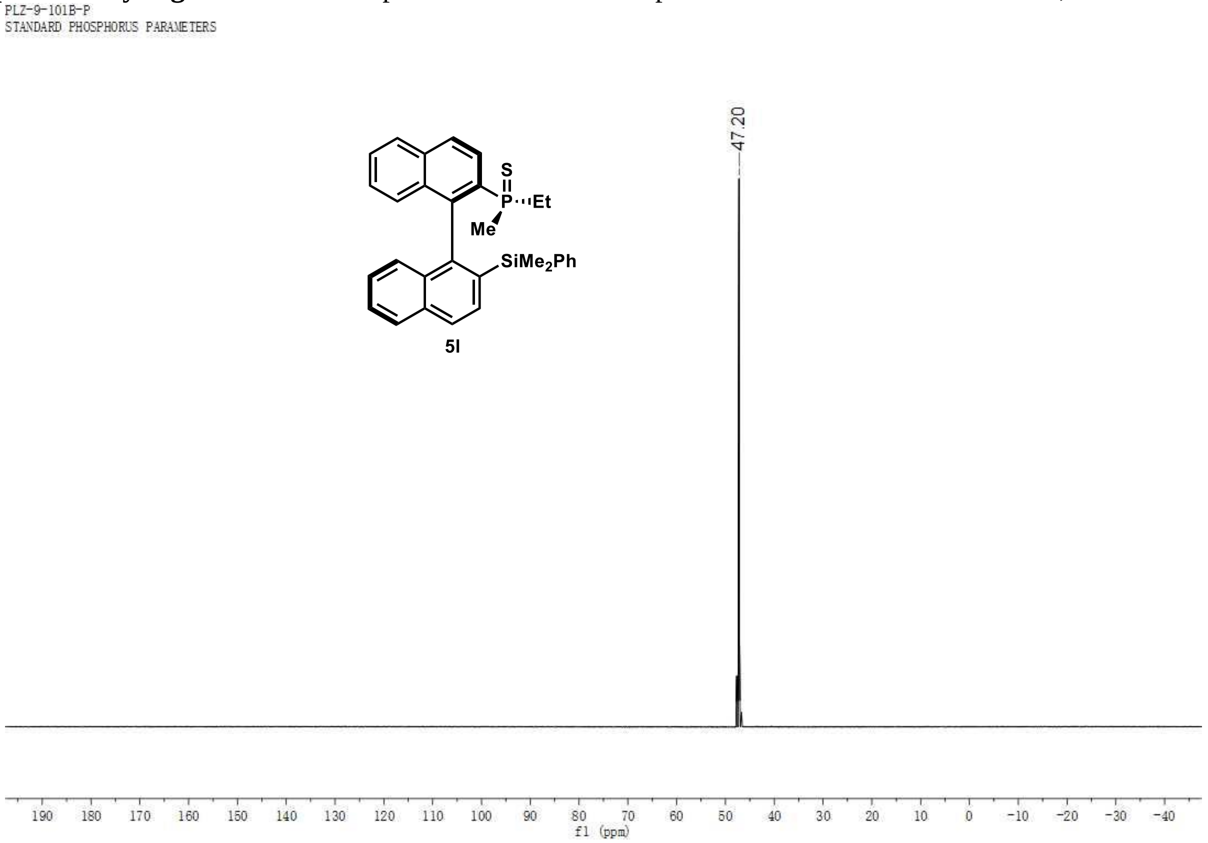

Supplementary Fig. 194.^{31}P NMR spectrum of **5l**. The sample has been recorded in 243 MHz, $CDCl_3$ at 25 ℃.

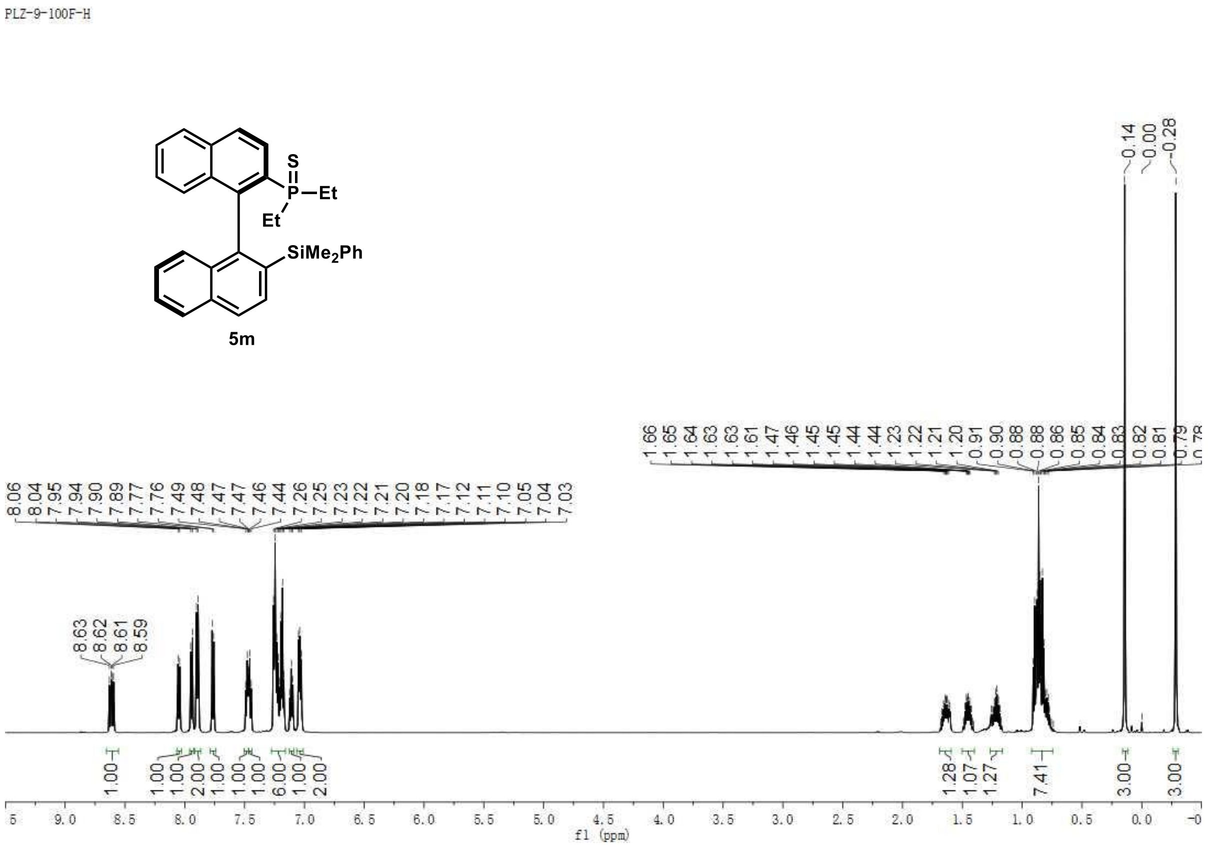

Supplementary Fig. 195. ^{1}H NMR spectrum of **5m**. The sample has been recorded in 600 MHz, $CDCl_3$ at 25 ℃.

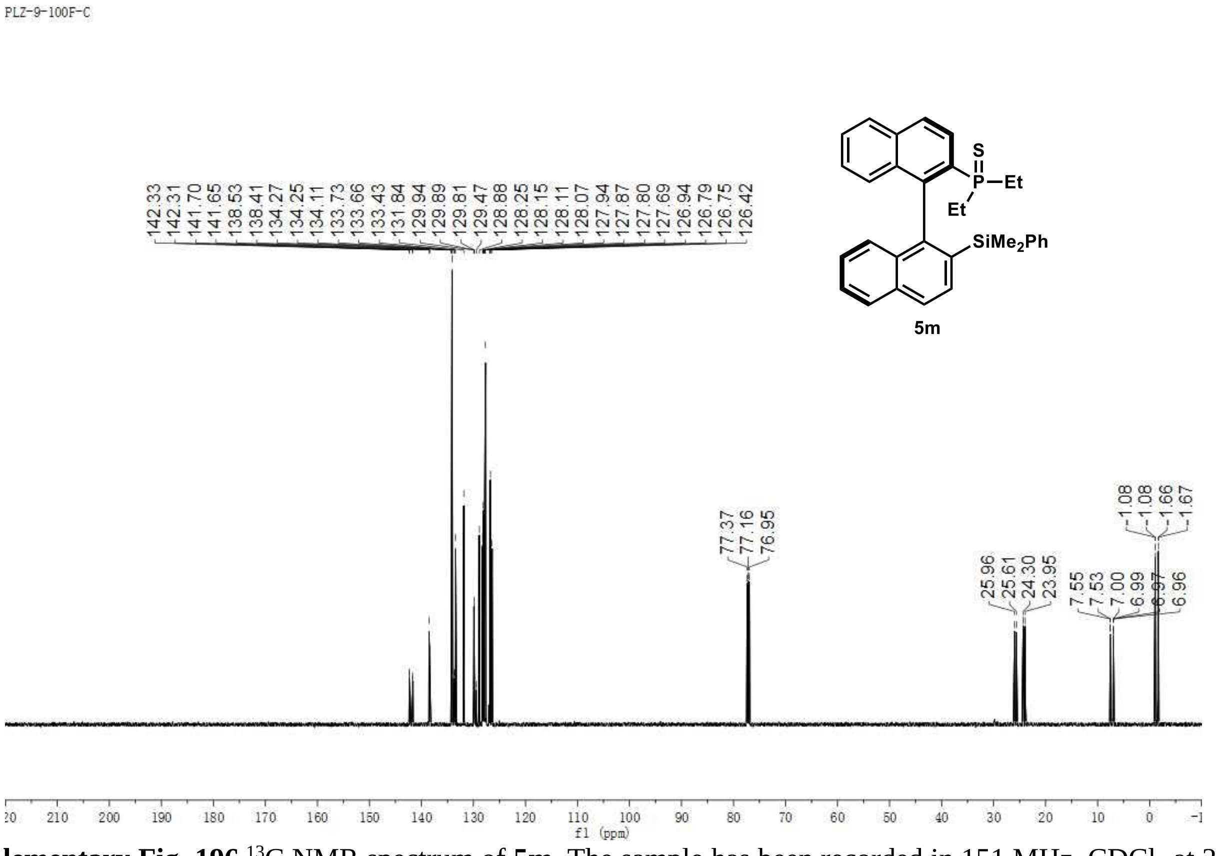

Supplementary Fig. 196.^{13}C NMR spectrum of **5m**. The sample has been recorded in 151 MHz, $CDCl_3$ at 25 ℃.

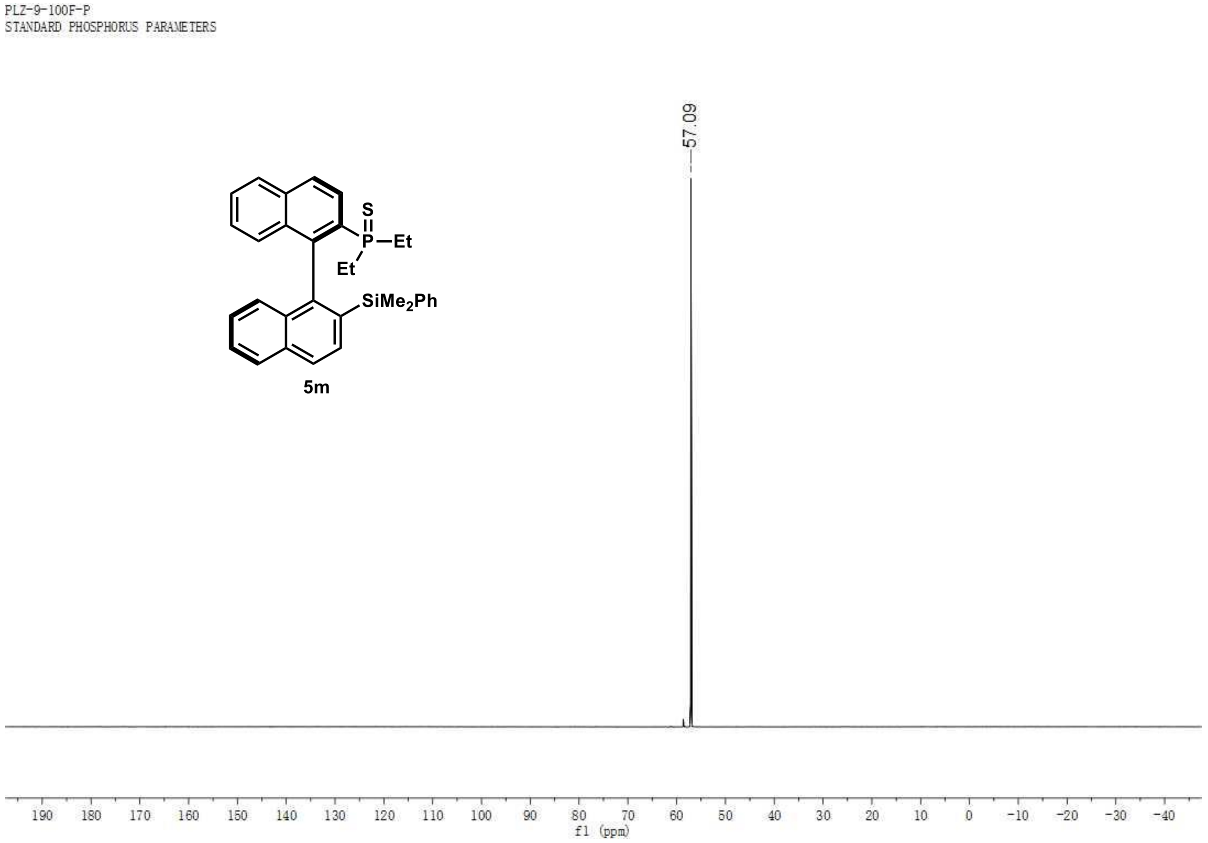

Supplementary Fig. 197.^{31}P NMR spectrum of **5m**. The sample has been recorded in 243 MHz, $CDCl_3$ at 25 ℃.

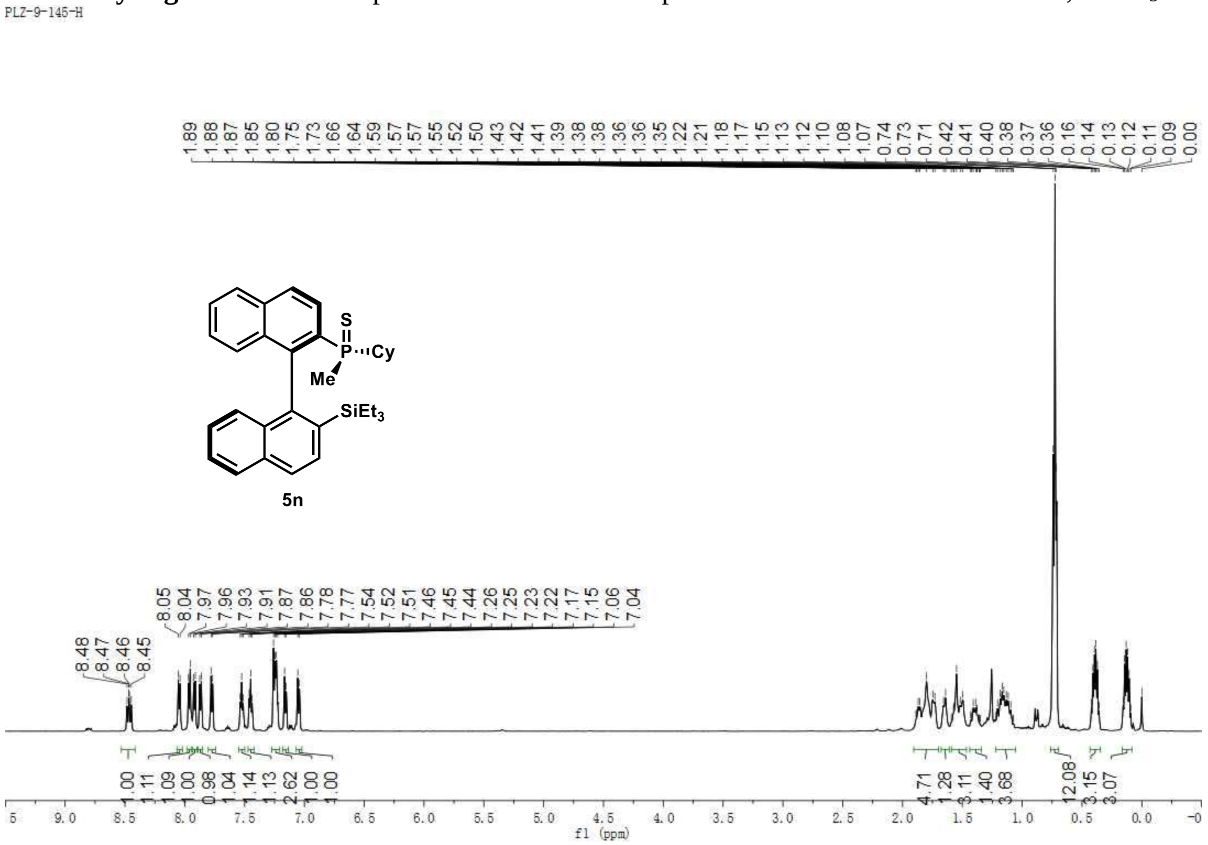

Supplementary Fig. 198. ^{1}H NMR spectrum of **5n**. The sample has been recorded in 600 MHz, $CDCl_3$ at 25 ℃.

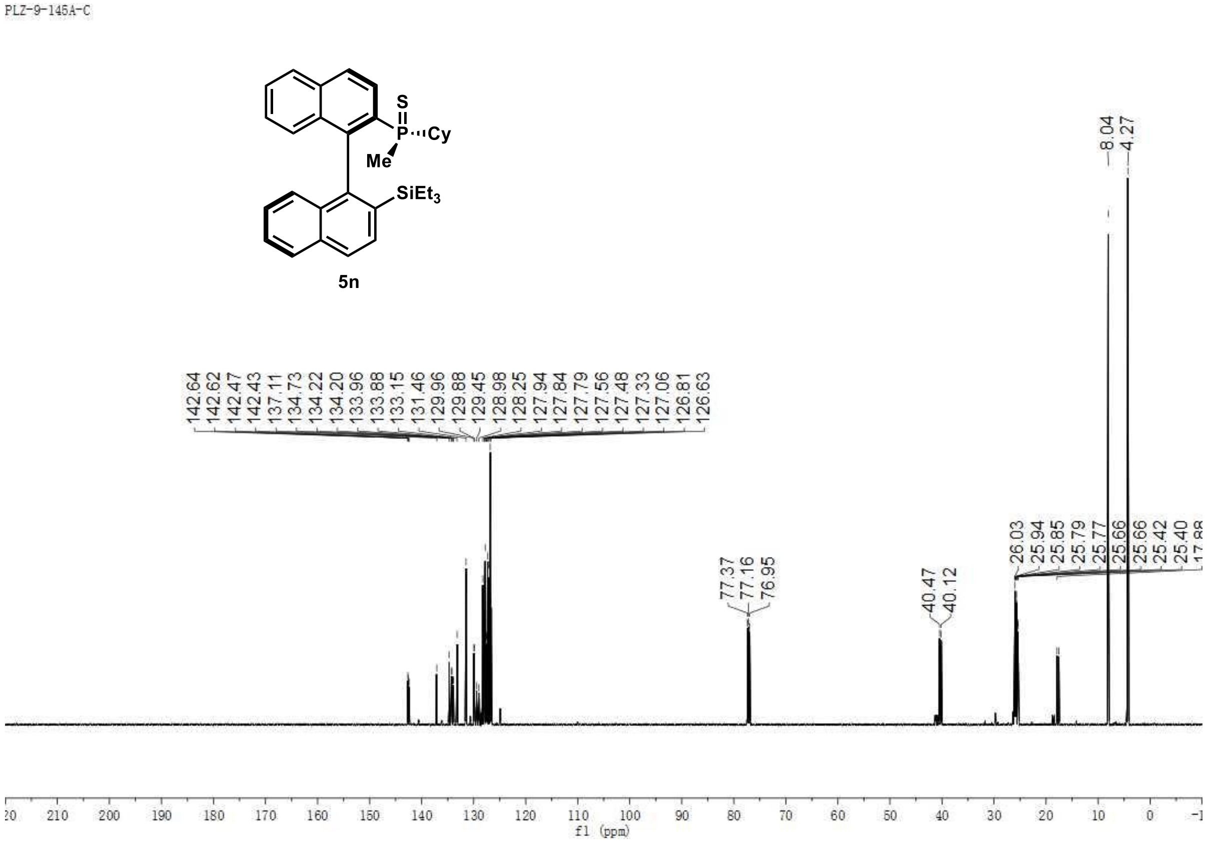

Supplementary Fig. 199.^{13}C NMR spectrum of **5n**. The sample has been recorded in 151 MHz, $CDCl_3$ at 25 ℃.

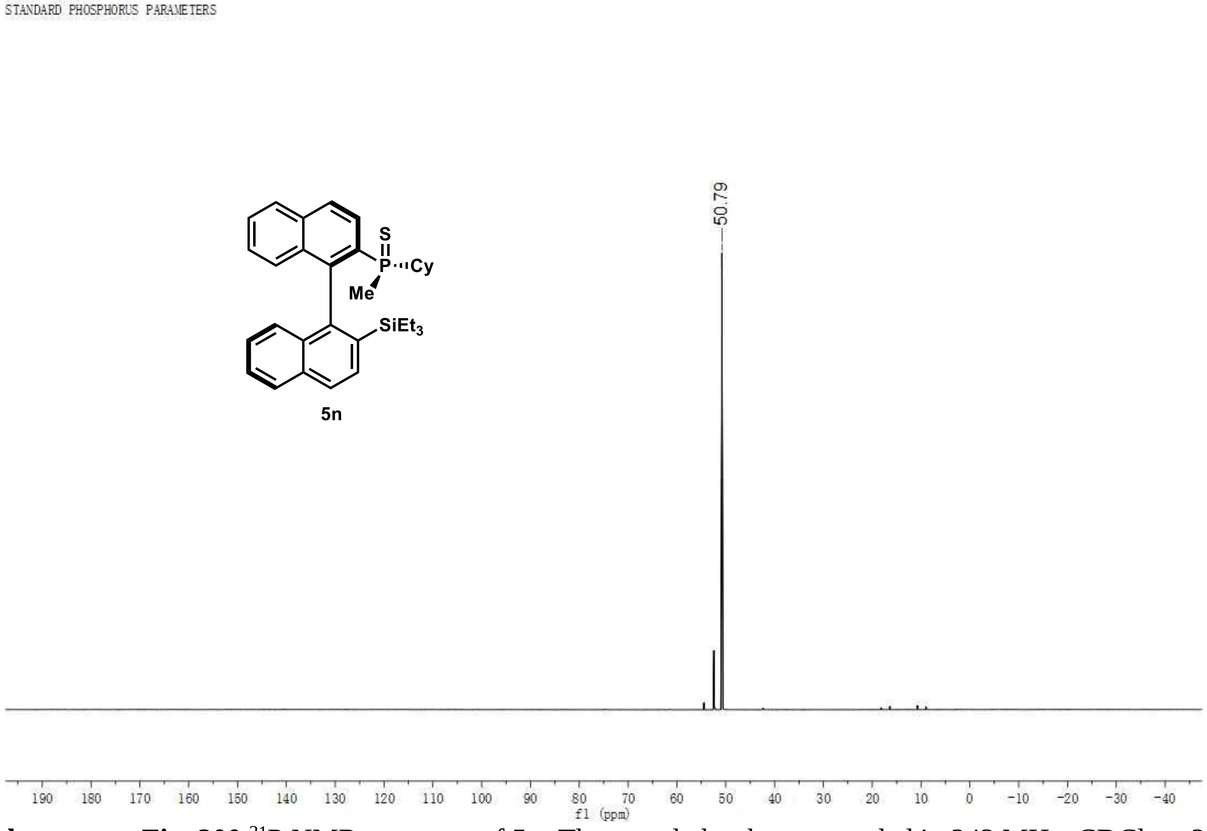

Supplementary Fig. 200.^{31}P NMR spectrum of **5n**. The sample has been recorded in 243 MHz, $CDCl_3$ at 25 ℃.

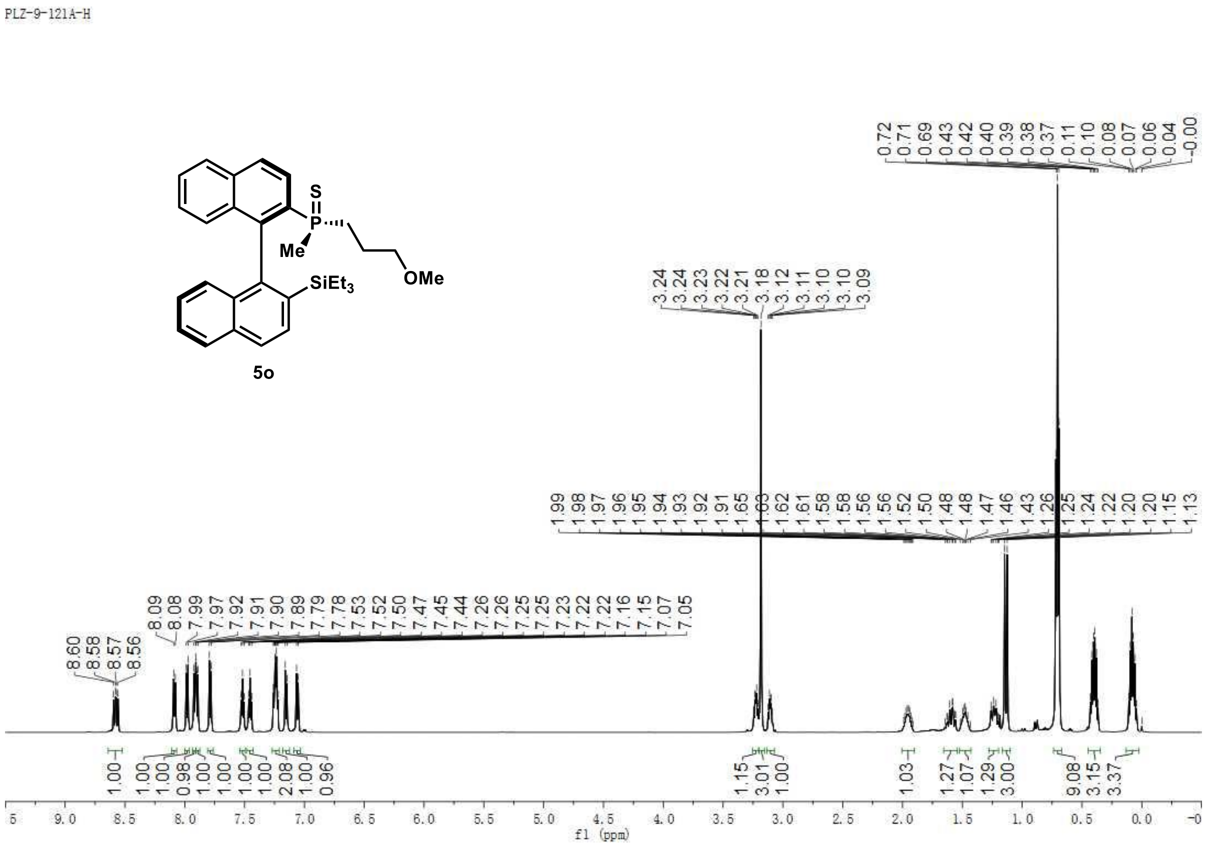

Supplementary Fig. 201. ^{1}H NMR spectrum of **5o**. The sample has been recorded in 600 MHz, $CDCl_3$ at 25 ℃.

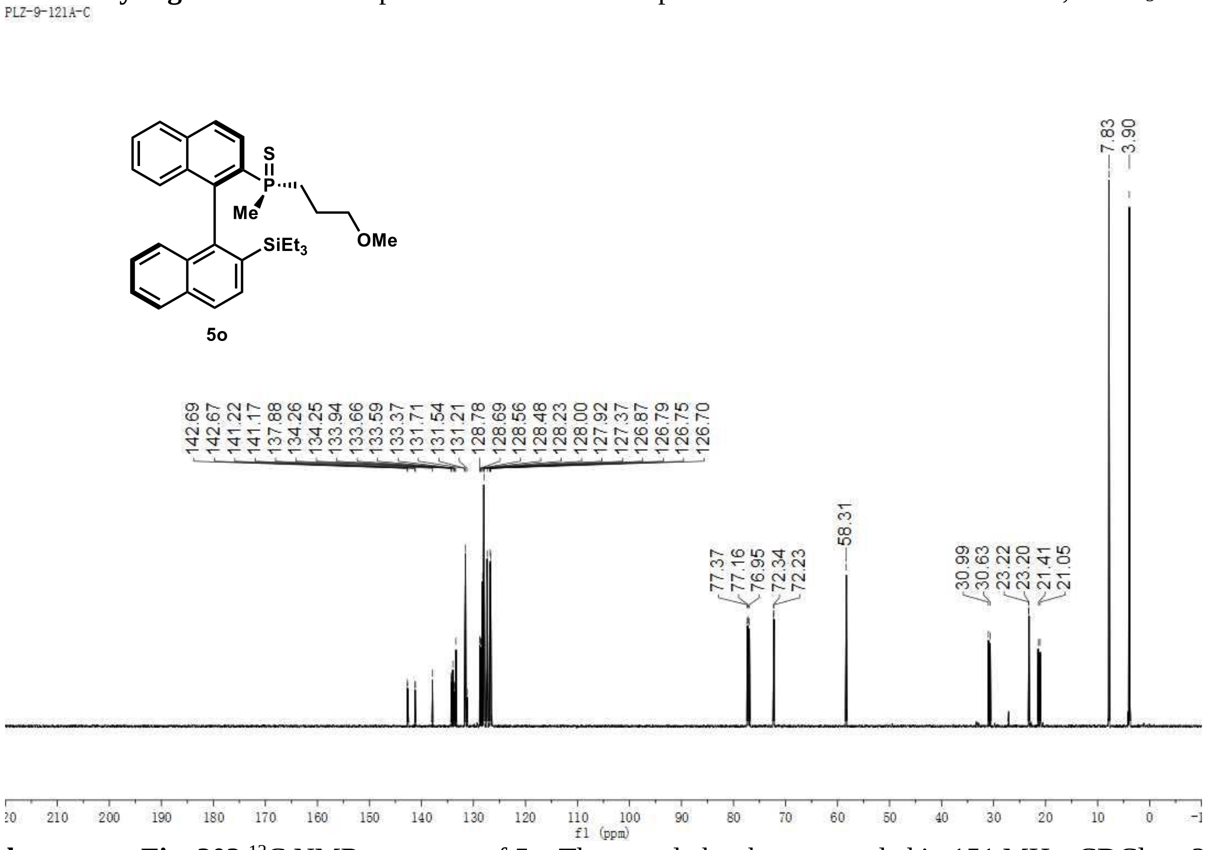

Supplementary Fig. 202.^{13}C NMR spectrum of **5o**. The sample has been recorded in 151 MHz, $CDCl_3$ at 25 ℃.

PLZ-9-121A-P
STANDARD PHOSPHORUS PARAMETERS

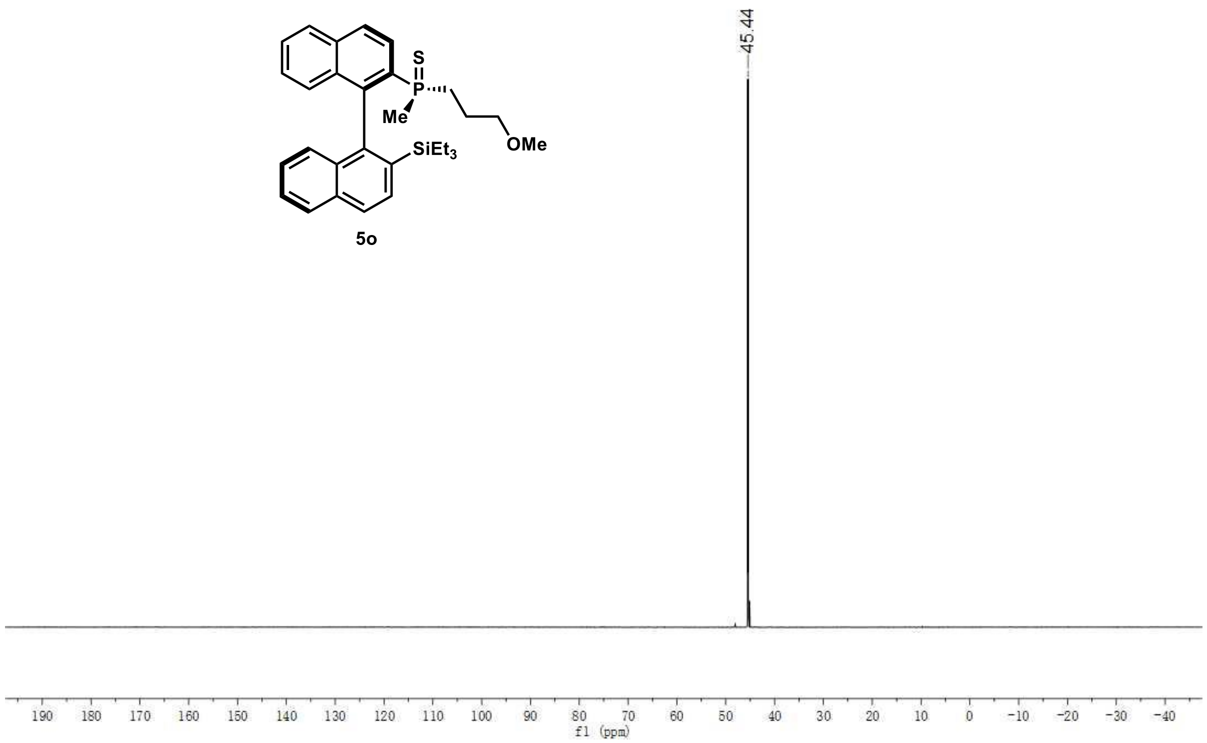

Supplementary Fig. 203.^{31}P NMR spectrum of **5o**. The sample has been recorded in 243 MHz, $CDCl_3$ at 25 ℃.

PLZ-9-159A-H

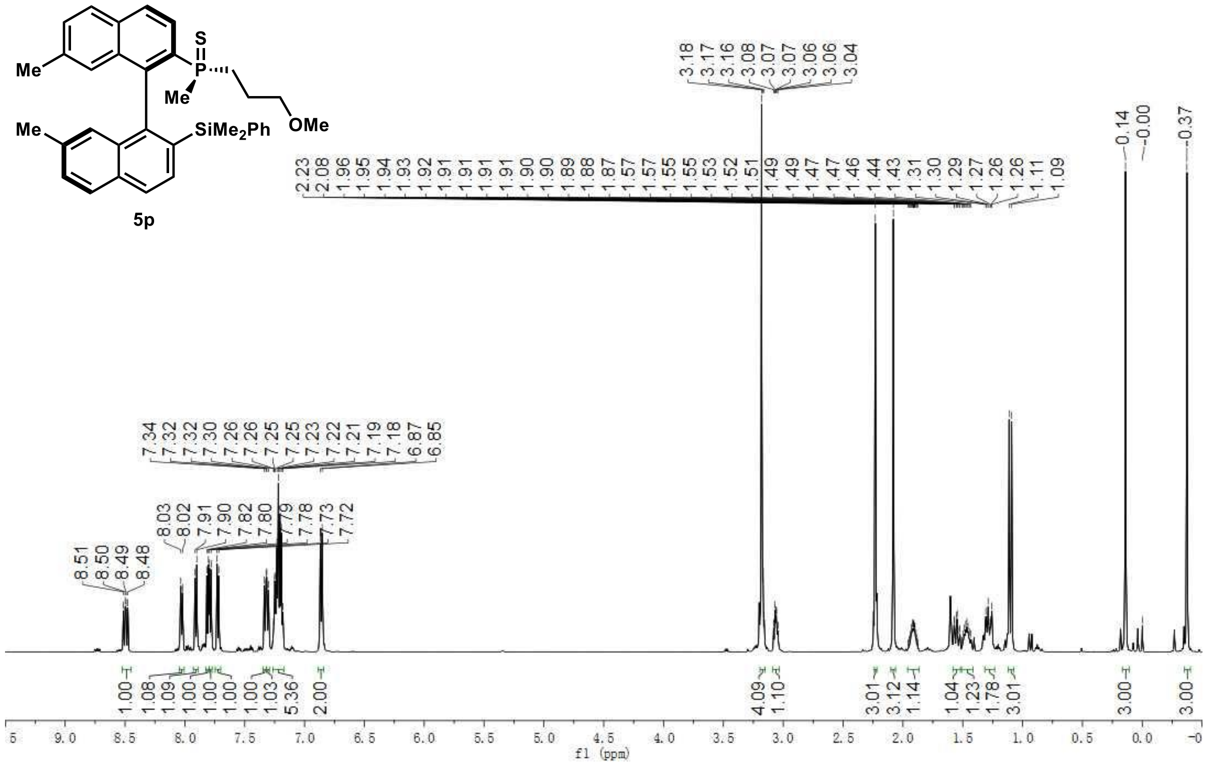

Supplementary Fig. 204. ^{1}H NMR spectrum of **5p**. The sample has been recorded in 600 MHz, $CDCl_3$ at 25 ℃.

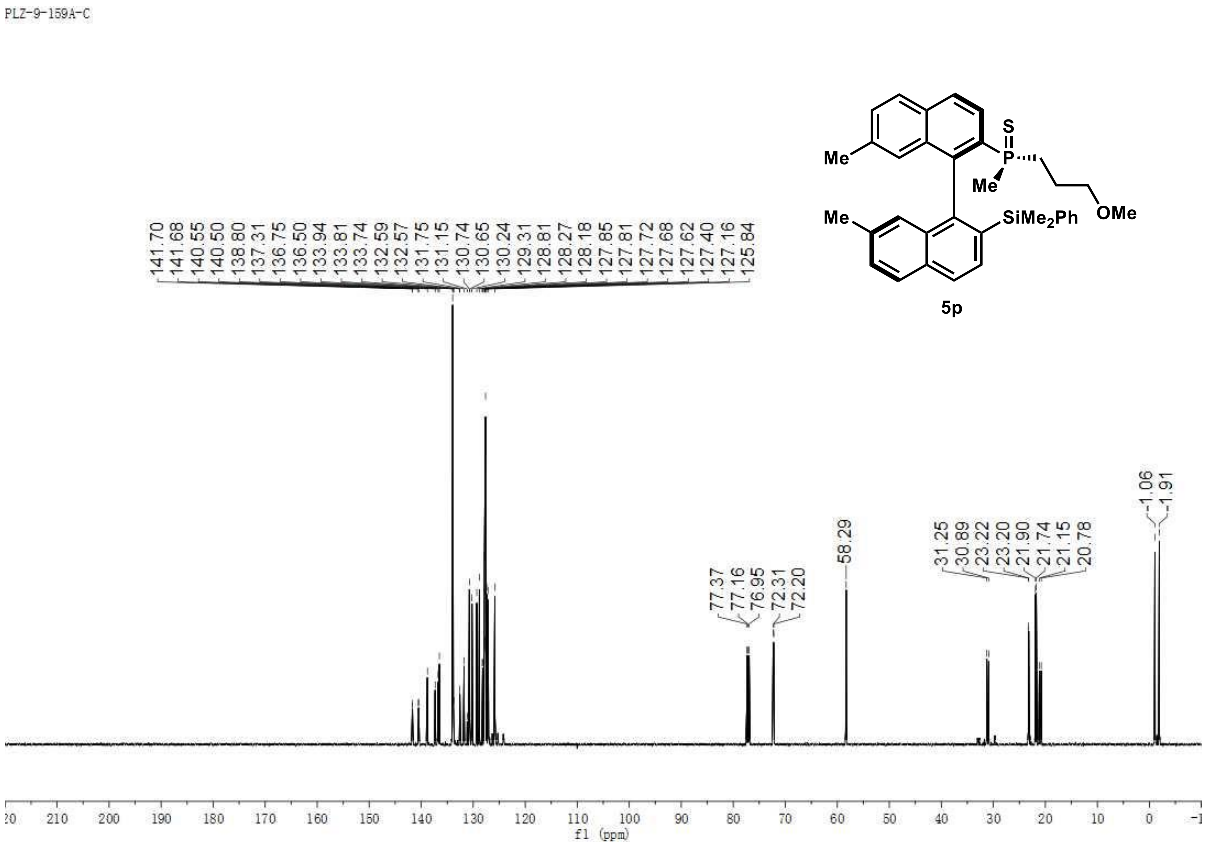

Supplementary Fig. 205.^{13}C NMR spectrum of **5p**. The sample has been recorded in 151 MHz, $CDCl_3$ at 25 ℃.

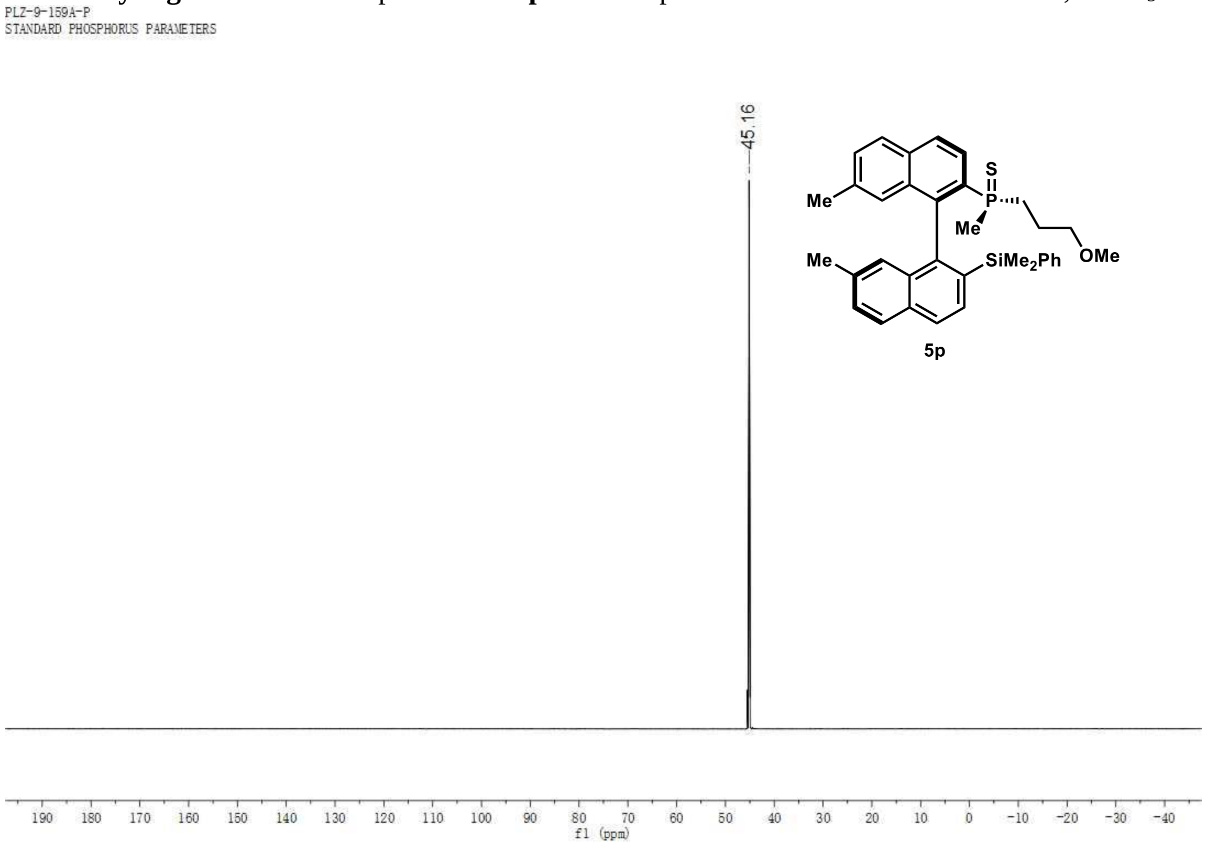

Supplementary Fig. 206.^{31}P NMR spectrum of **5p**. The sample has been recorded in 243 MHz, $CDCl_3$ at 25 ℃.

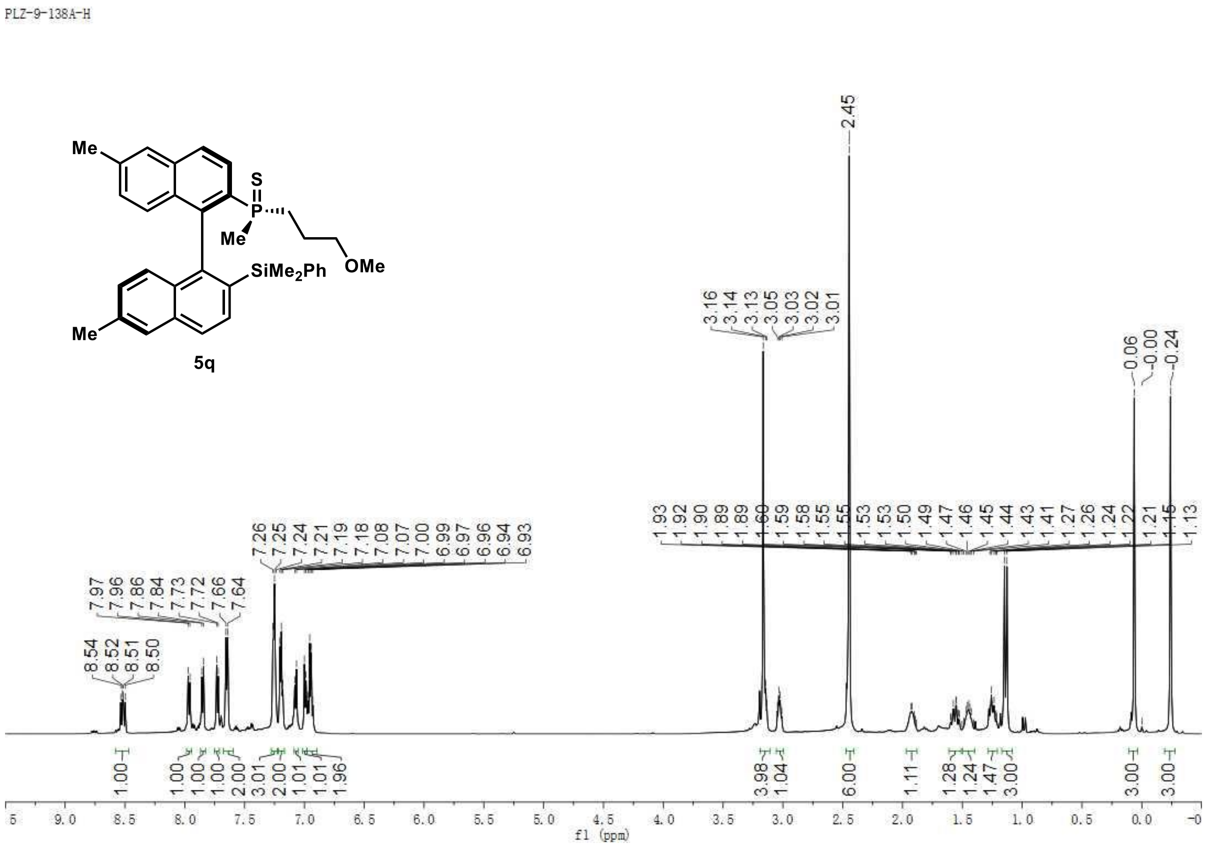

Supplementary Fig. 207. ^{1}H NMR spectrum of **5q**. The sample has been recorded in 600 MHz, $CDCl_3$ at 25 ℃.

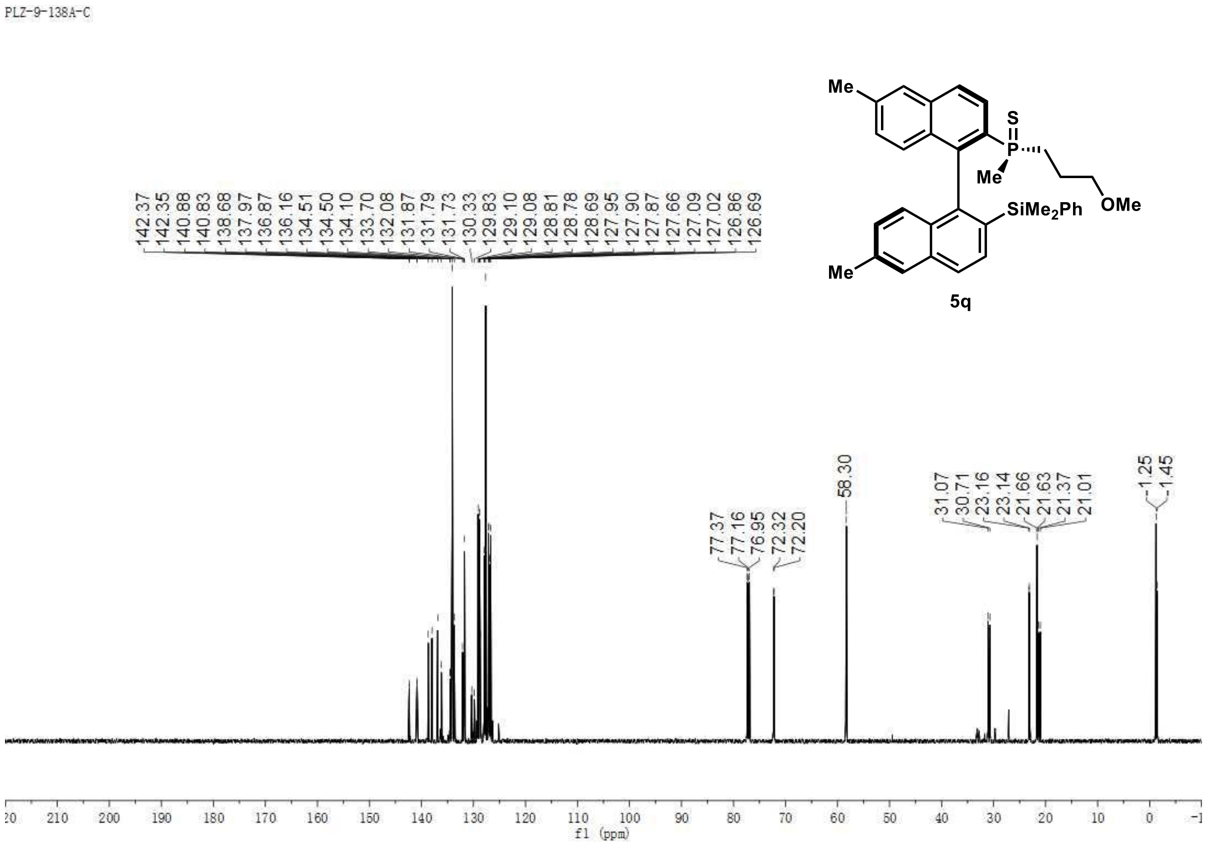

Supplementary Fig. 208.^{13}C NMR spectrum of **5q**. The sample has been recorded in 151 MHz, $CDCl_3$ at 25 ℃.

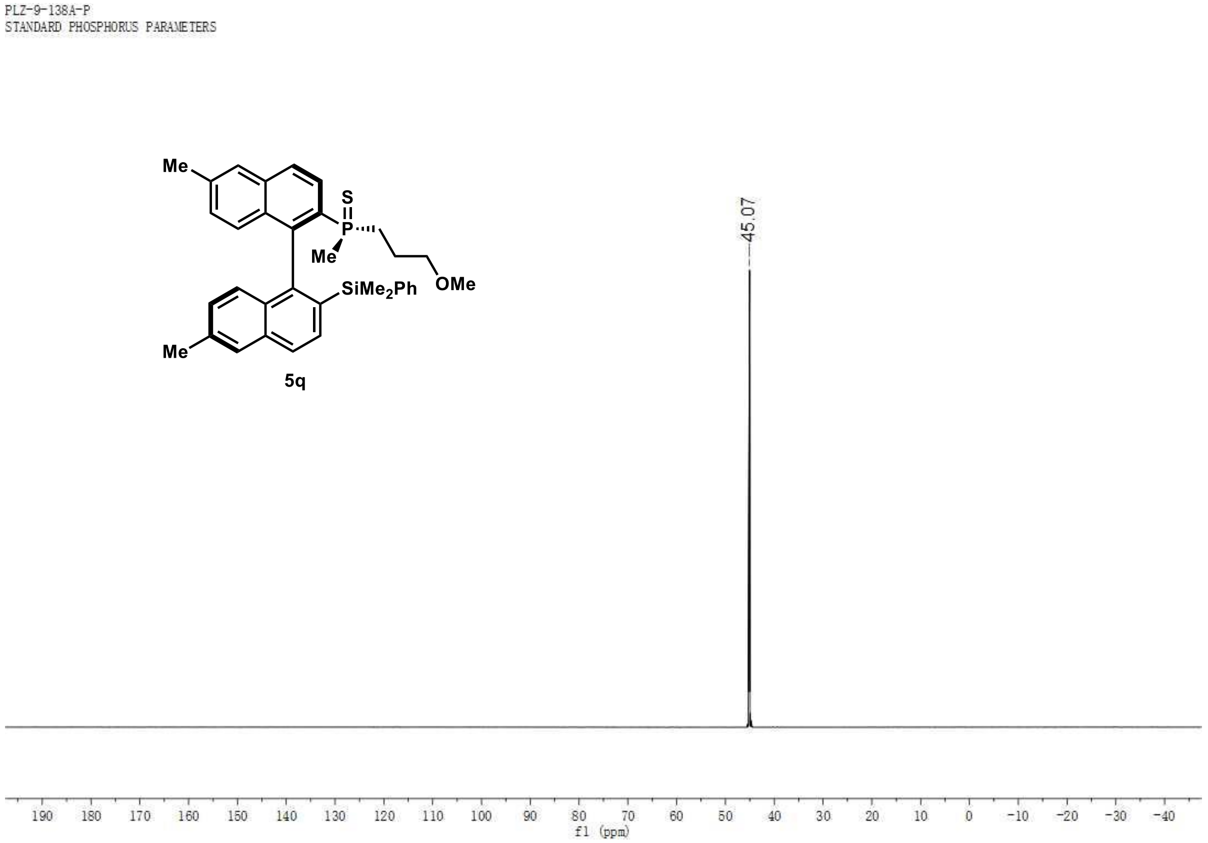

Supplementary Fig. 209.^{31}P NMR spectrum of **5q**. The sample has been recorded in 243 MHz, $CDCl_3$ at 25 ℃.

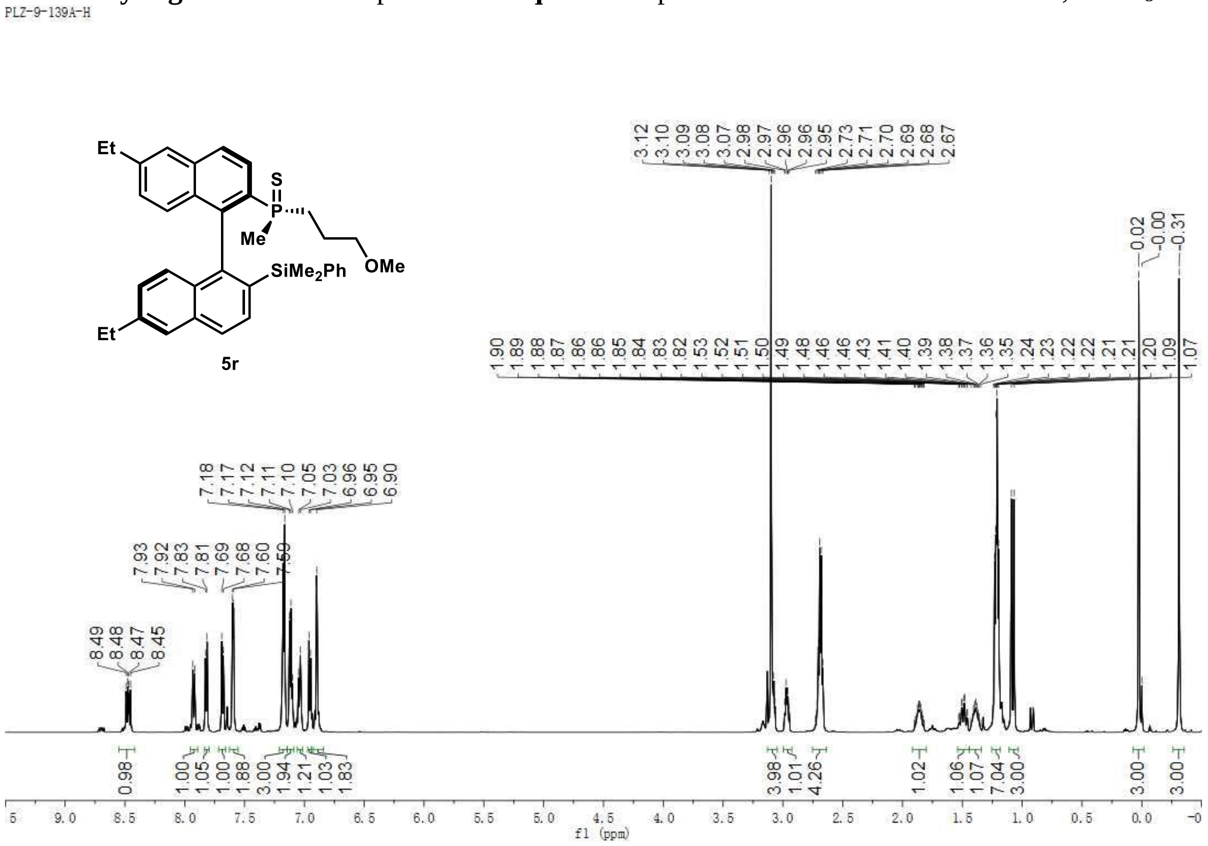

Supplementary Fig. 210. ^{1}H NMR spectrum of **5r**. The sample has been recorded in 600 MHz, $CDCl_3$ at 25 ℃.

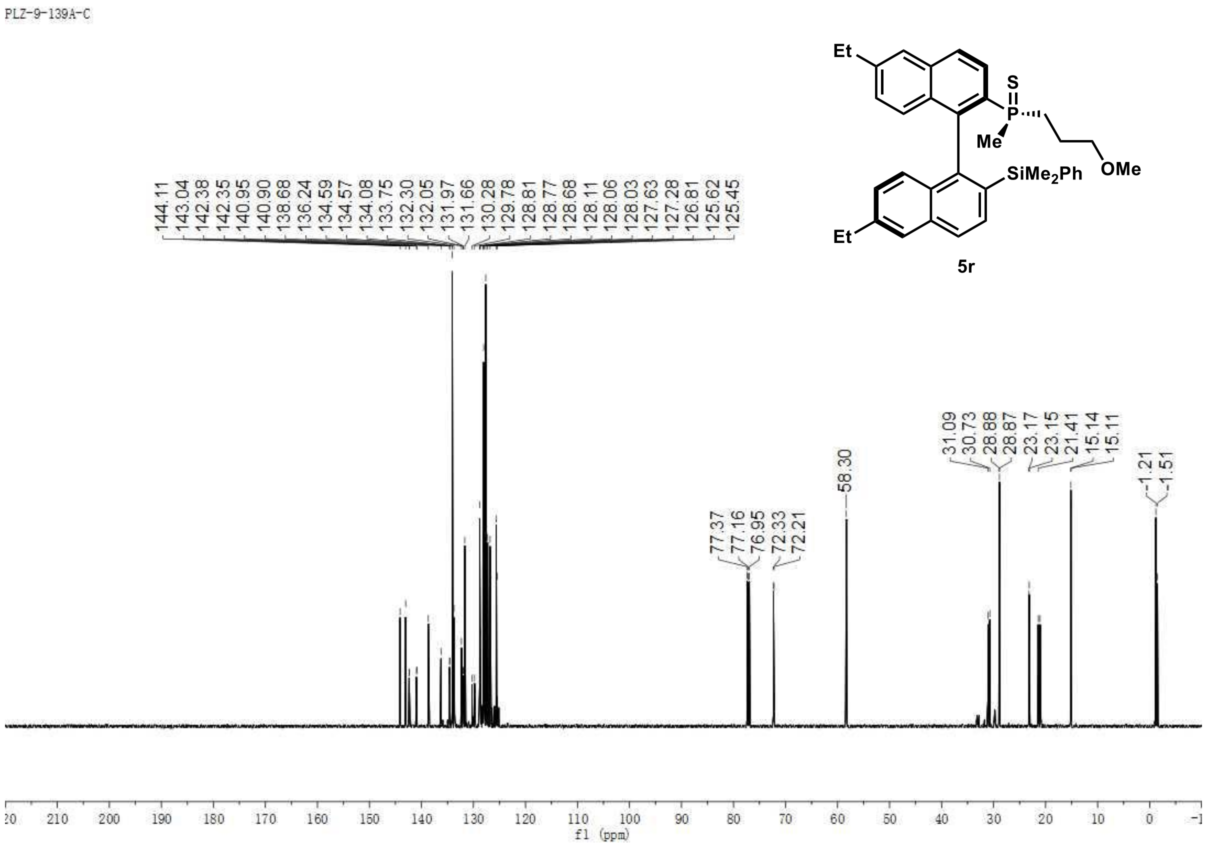

Supplementary Fig. 211.^{13}C NMR spectrum of **5r**. The sample has been recorded in 151 MHz, $CDCl_3$ at 25 ℃.

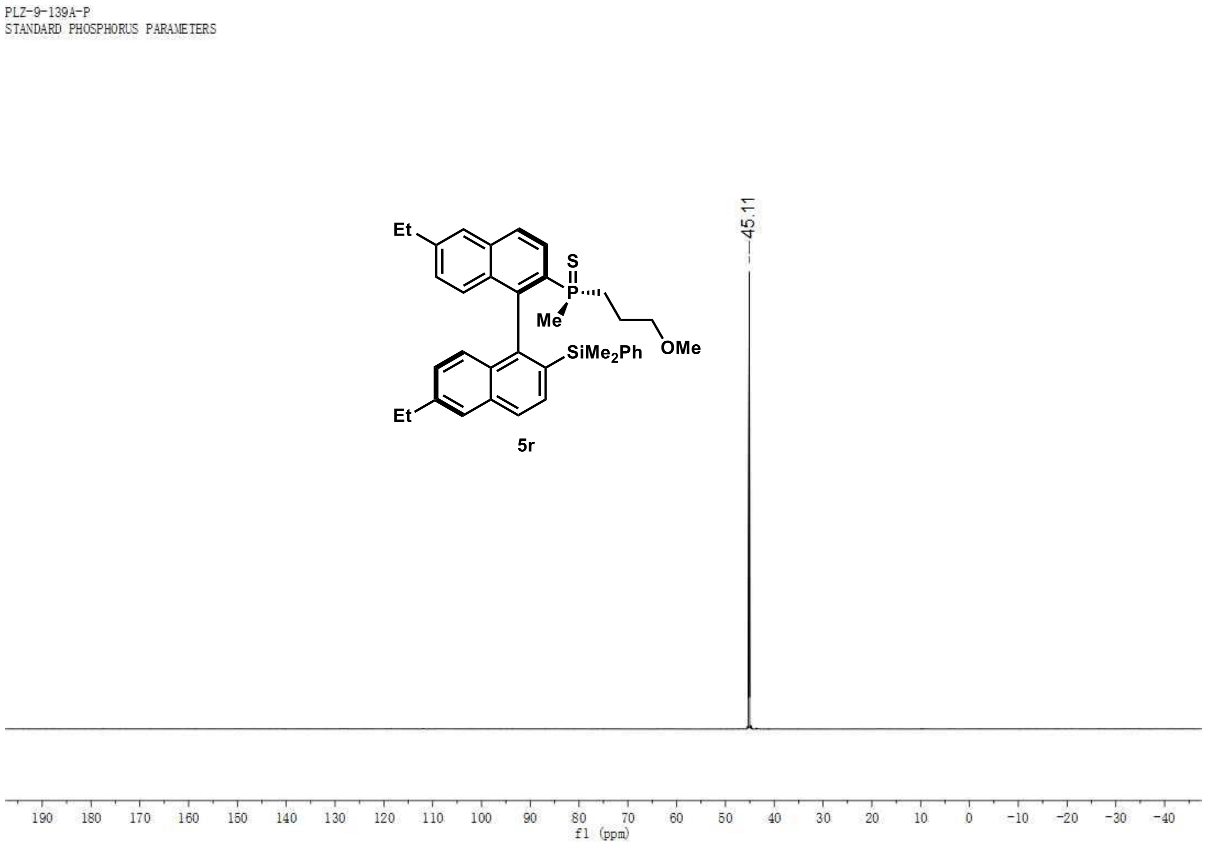

Supplementary Fig. 212.^{31}P NMR spectrum of **5r**. The sample has been recorded in 243 MHz, $CDCl_3$ at 25 ℃.

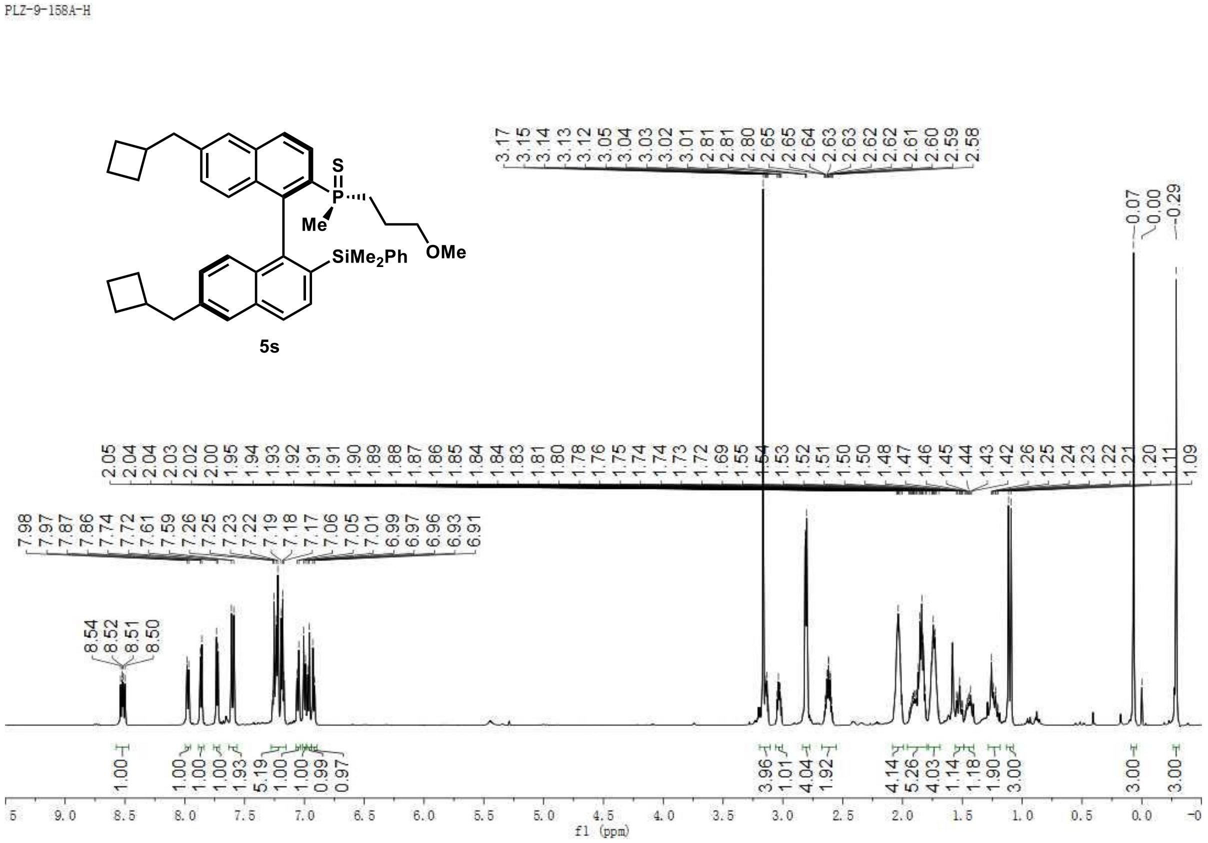

Supplementary Fig. 213. ^{1}H NMR spectrum of **5s**. The sample has been recorded in 600 MHz, $CDCl_3$ at 25 ℃.

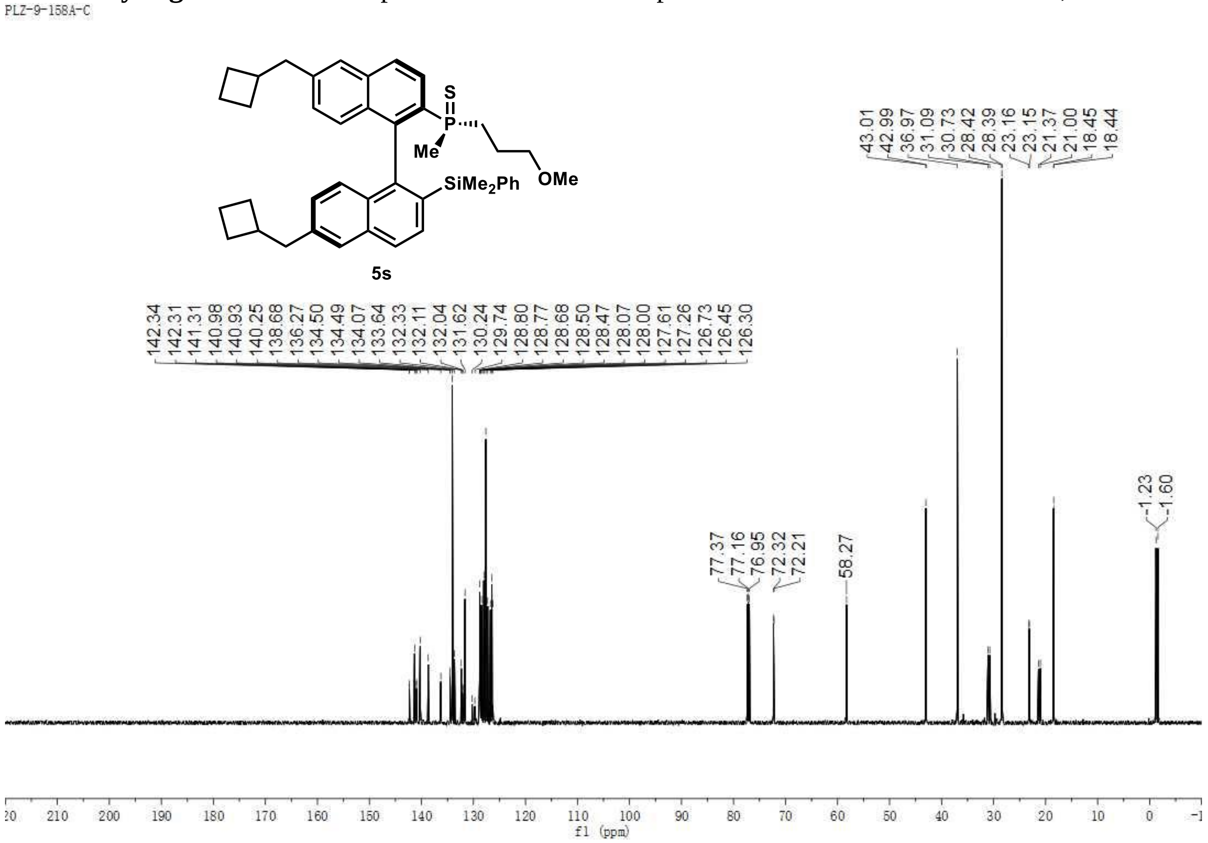

Supplementary Fig. 214.^{13}C NMR spectrum of **5s**. The sample has been recorded in 151 MHz, $CDCl_3$ at 25 ℃.

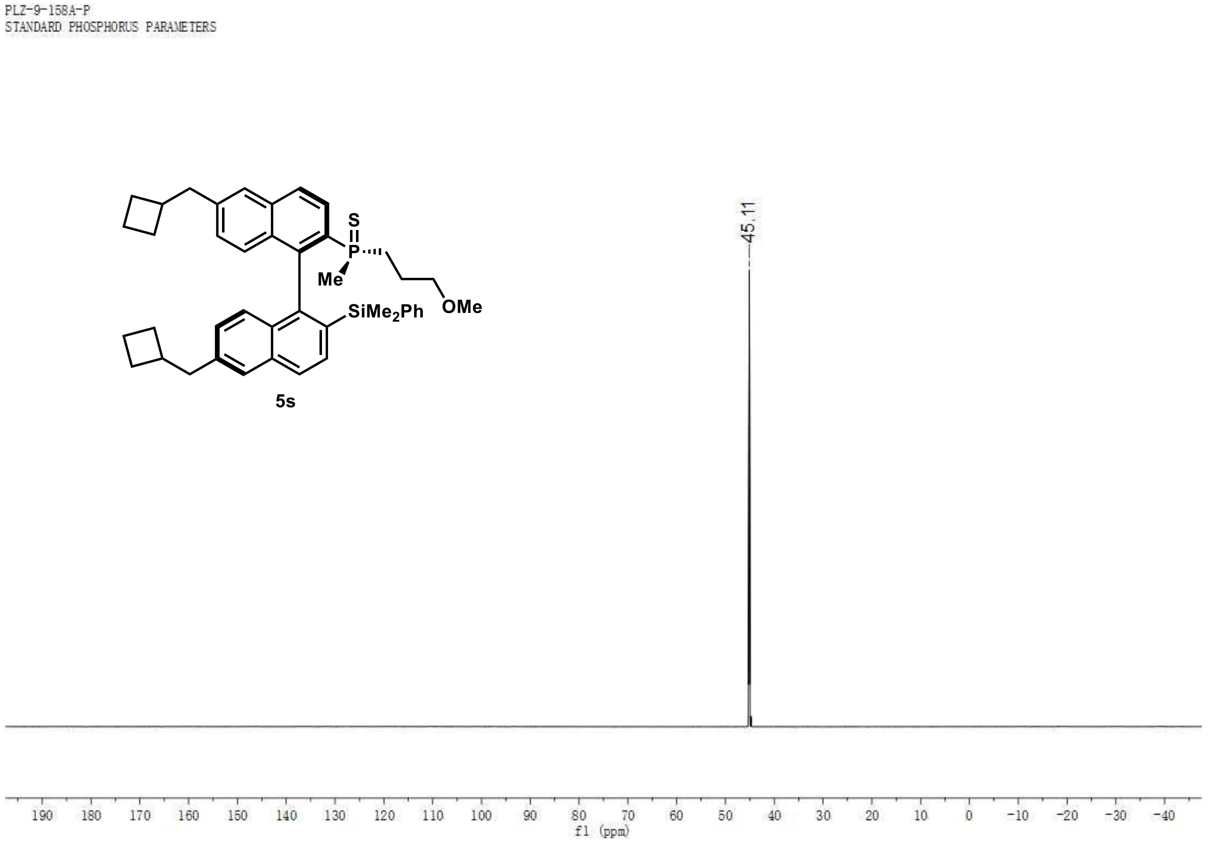

Supplementary Fig. 215.^{31}P NMR spectrum of **5s**. The sample has been recorded in 243 MHz, $CDCl_3$ at 25 ℃.

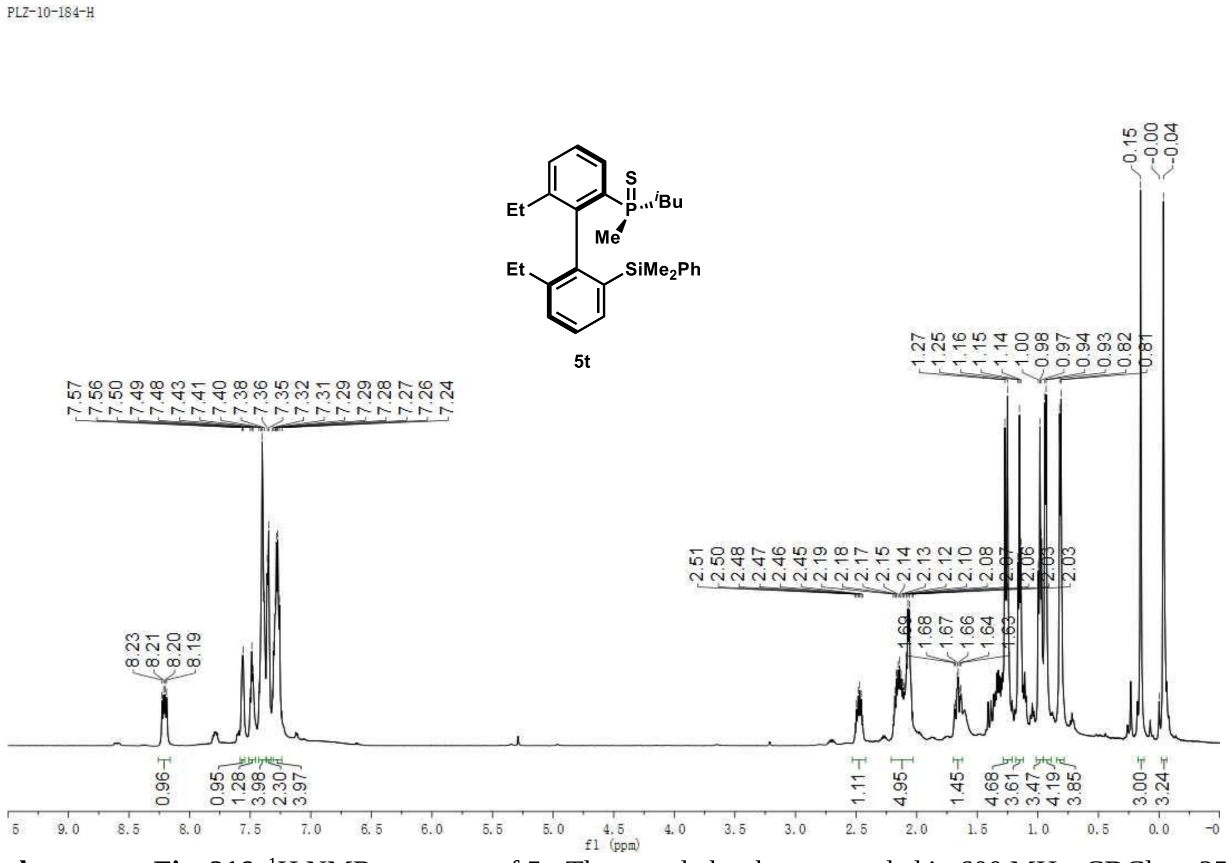

Supplementary Fig. 216. ^{1}H NMR spectrum of **5t**. The sample has been recorded in 600 MHz, $CDCl_3$ at 25 ℃.

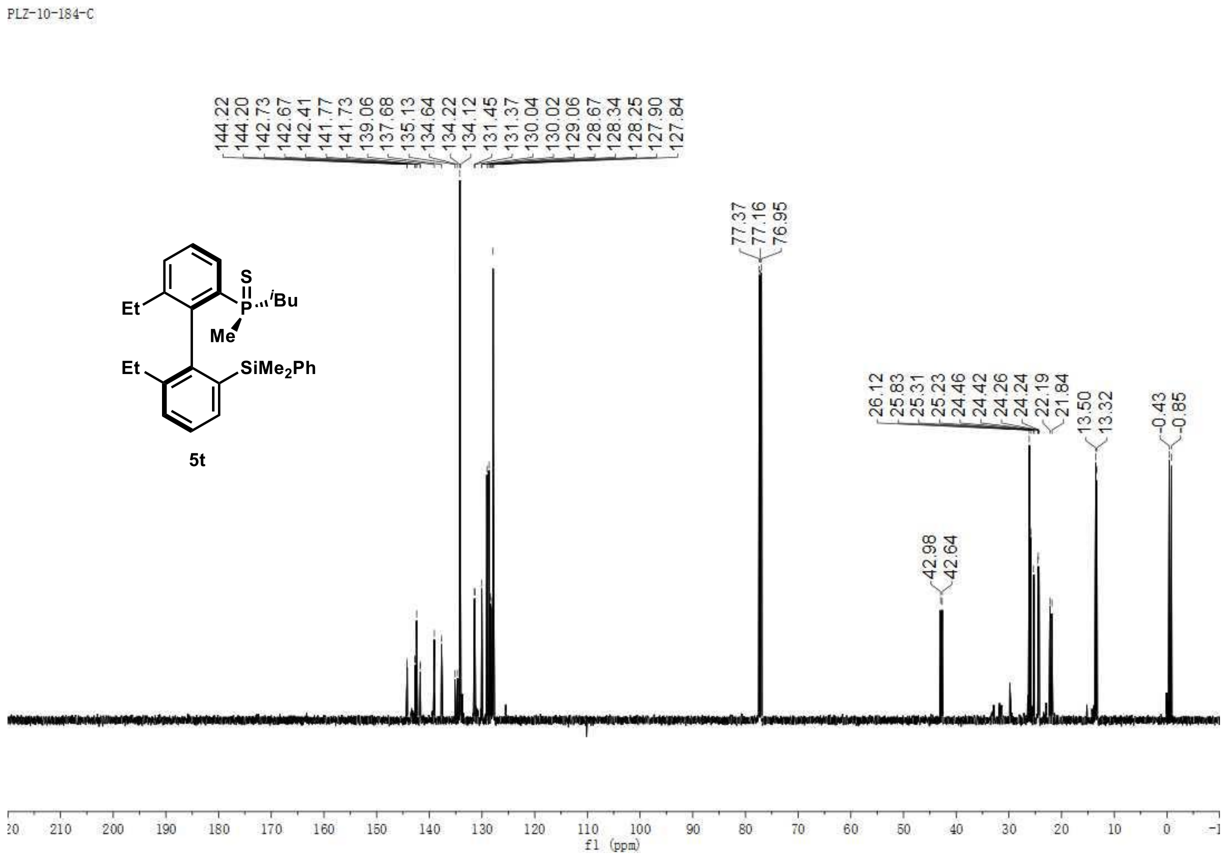

Supplementary Fig. 217.^{13}C NMR spectrum of **5t**. The sample has been recorded in 151 MHz, $CDCl_3$ at 25 ℃.

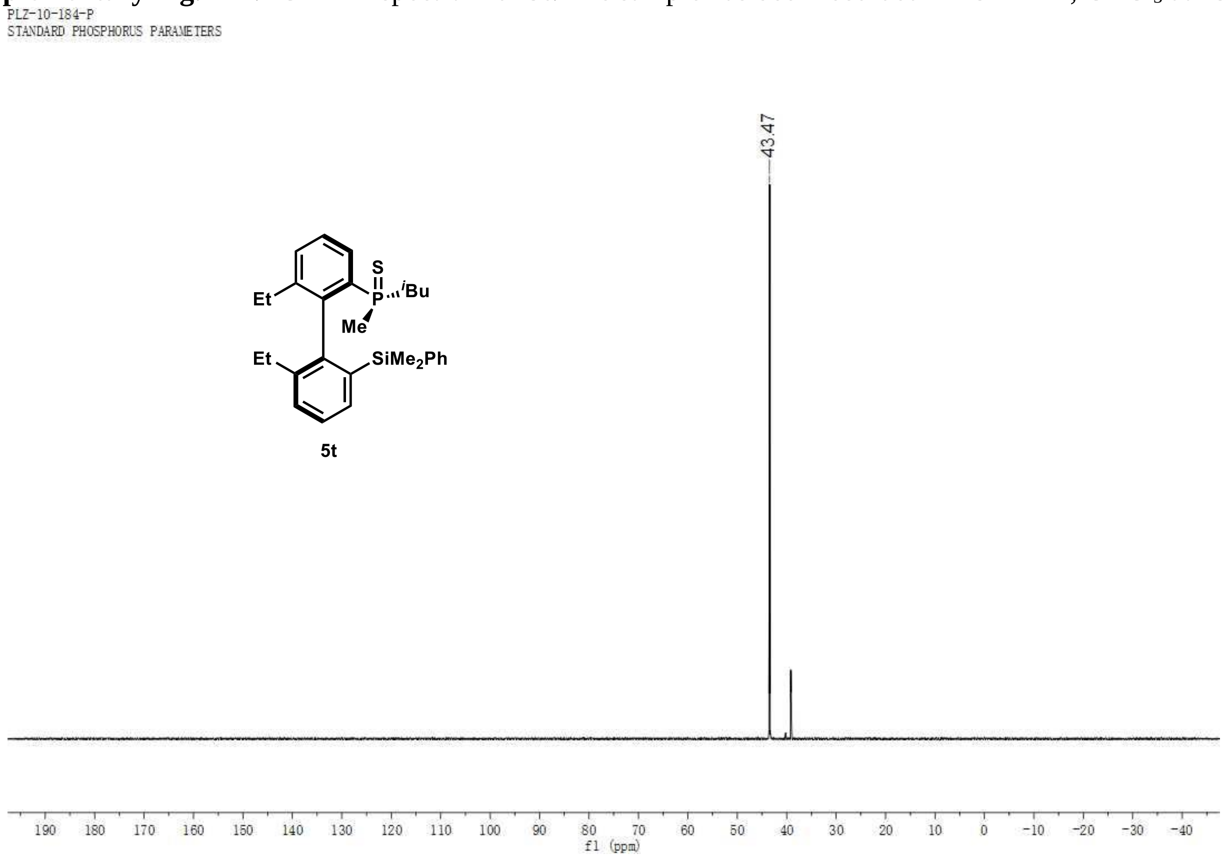

Supplementary Fig. 218.^{31}P NMR spectrum of **5t**. The sample has been recorded in 243 MHz, $CDCl_3$ at 25 ℃.

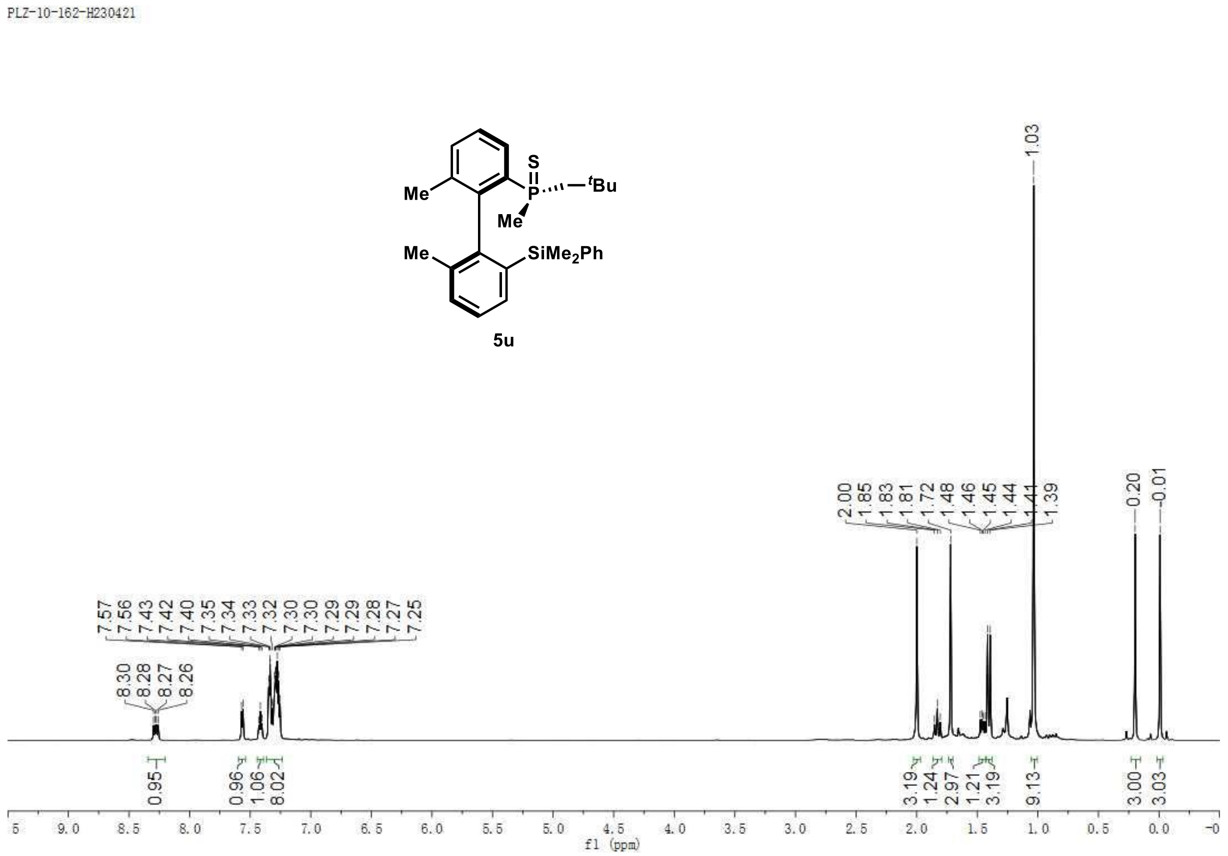

Supplementary Fig. 219. ^{1}H NMR spectrum of **5u**. The sample has been recorded in 600 MHz, $CDCl_3$ at 25 ℃.

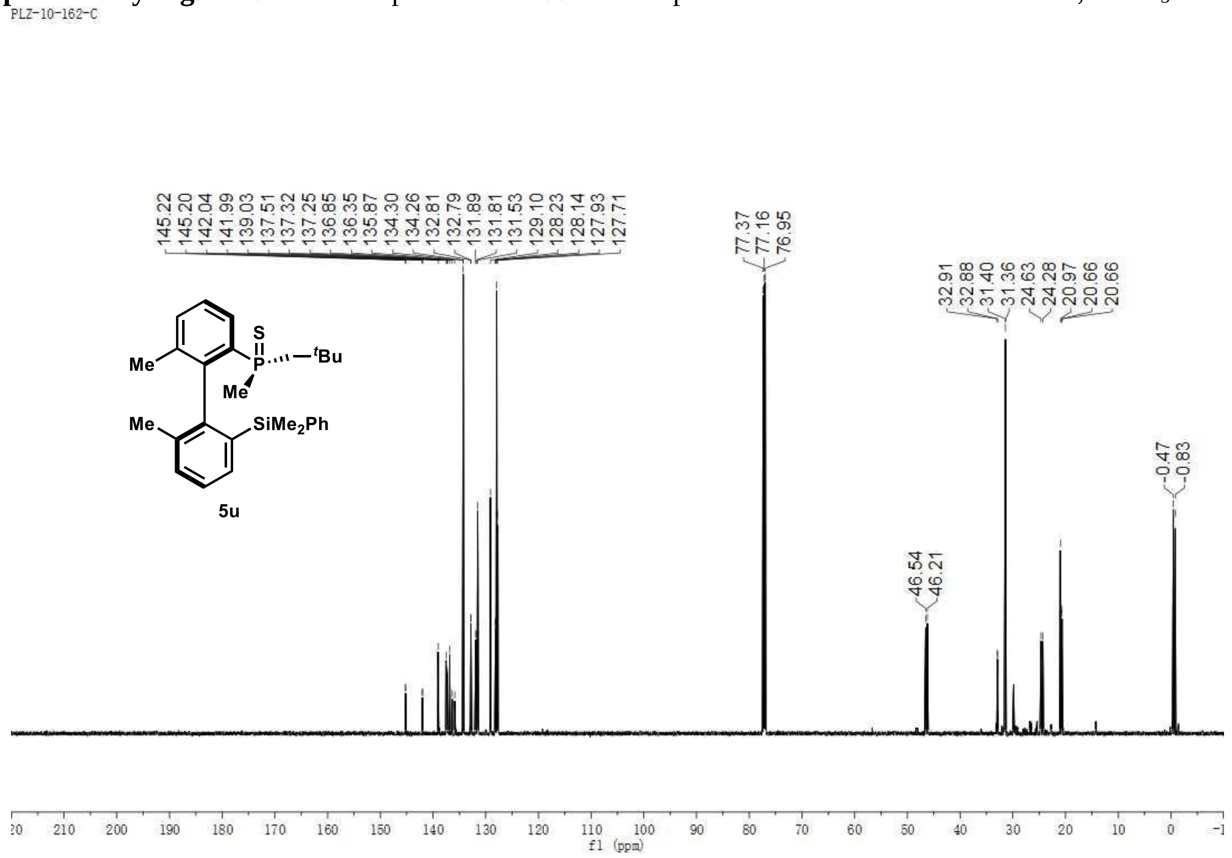

Supplementary Fig. 220.^{13}C NMR spectrum of **5u**. The sample has been recorded in 151 MHz, $CDCl_3$ at 25 ℃.

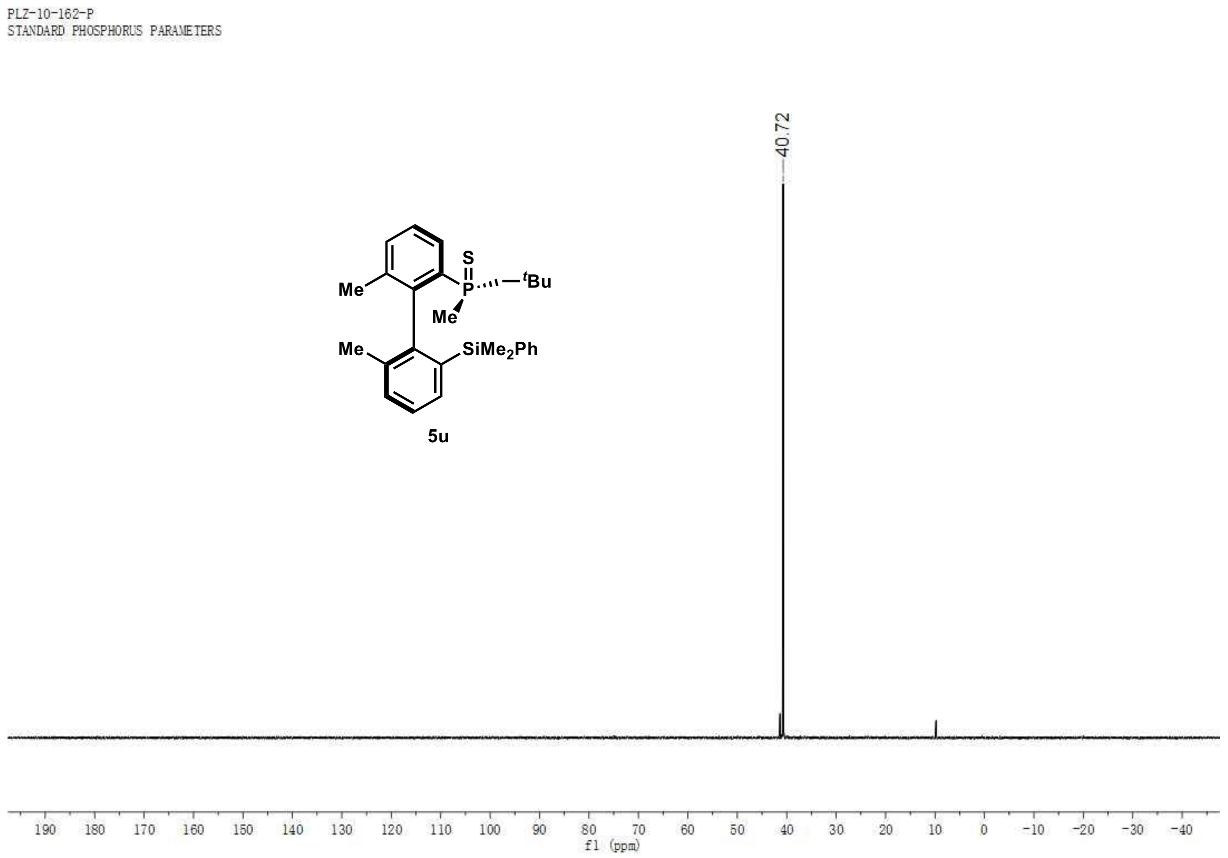

Supplementary Fig. 221.^{31}P NMR spectrum of **5u**. The sample has been recorded in 243 MHz, $CDCl_3$ at 25 ℃.

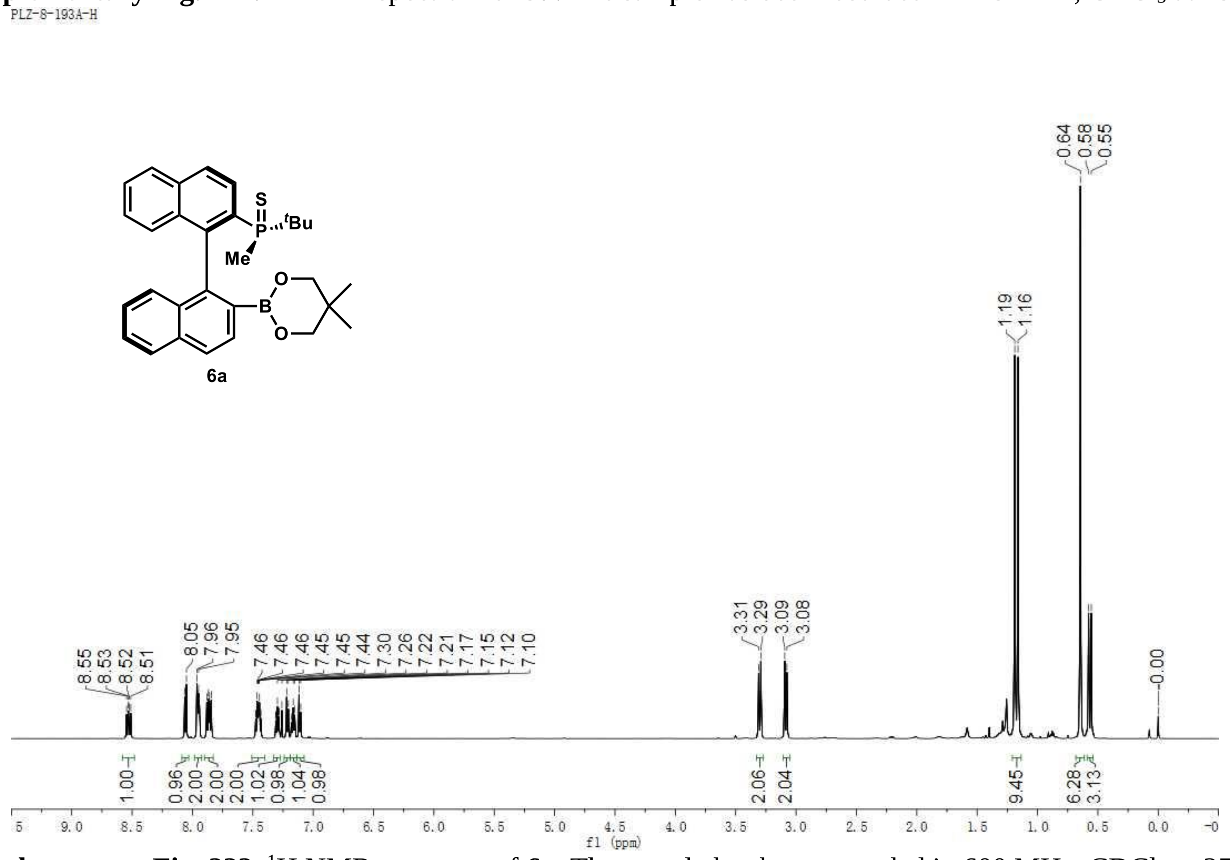

Supplementary Fig. 222. ^{1}H NMR spectrum of **6a**. The sample has been recorded in 600 MHz, $CDCl_3$ at 25 ℃.

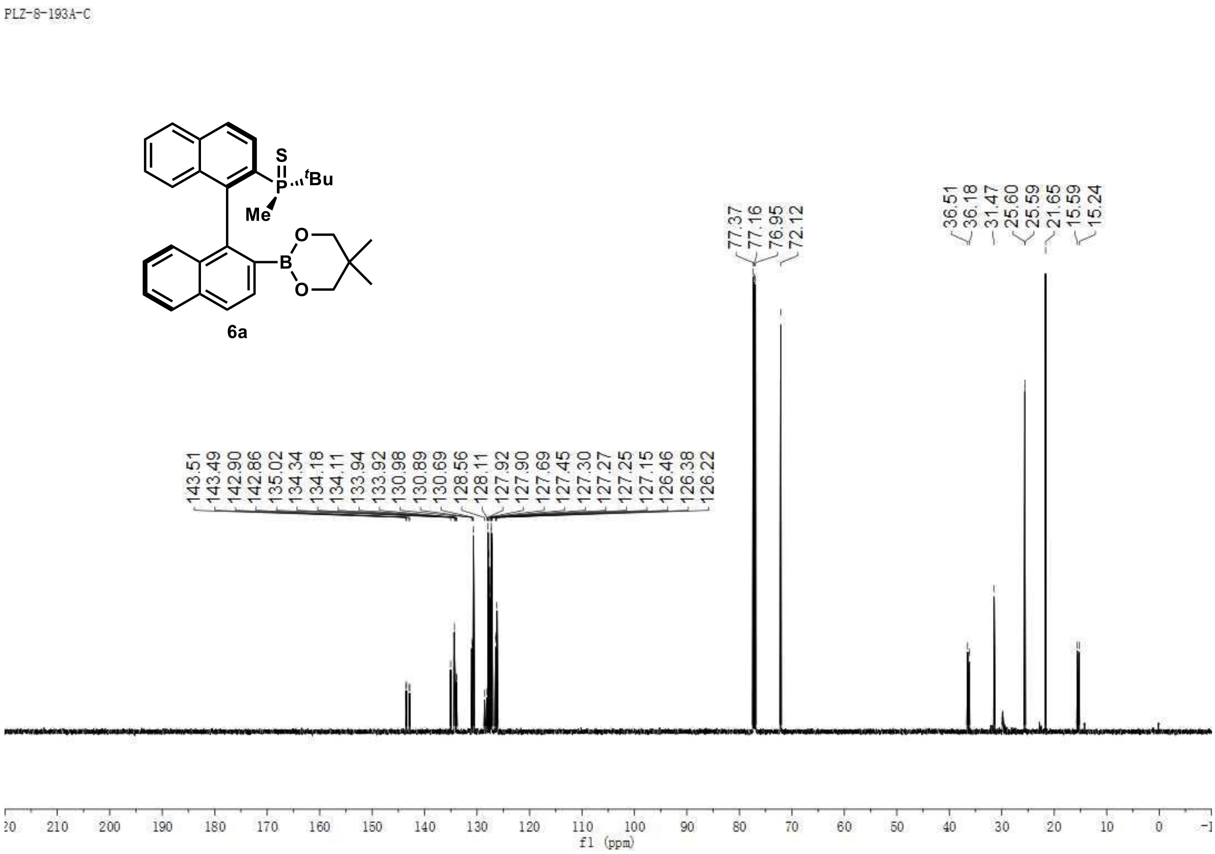

Supplementary Fig. 223.^{13}C NMR spectrum of **6a**. The sample has been recorded in 151 MHz, $CDCl_3$ at 25 ℃.

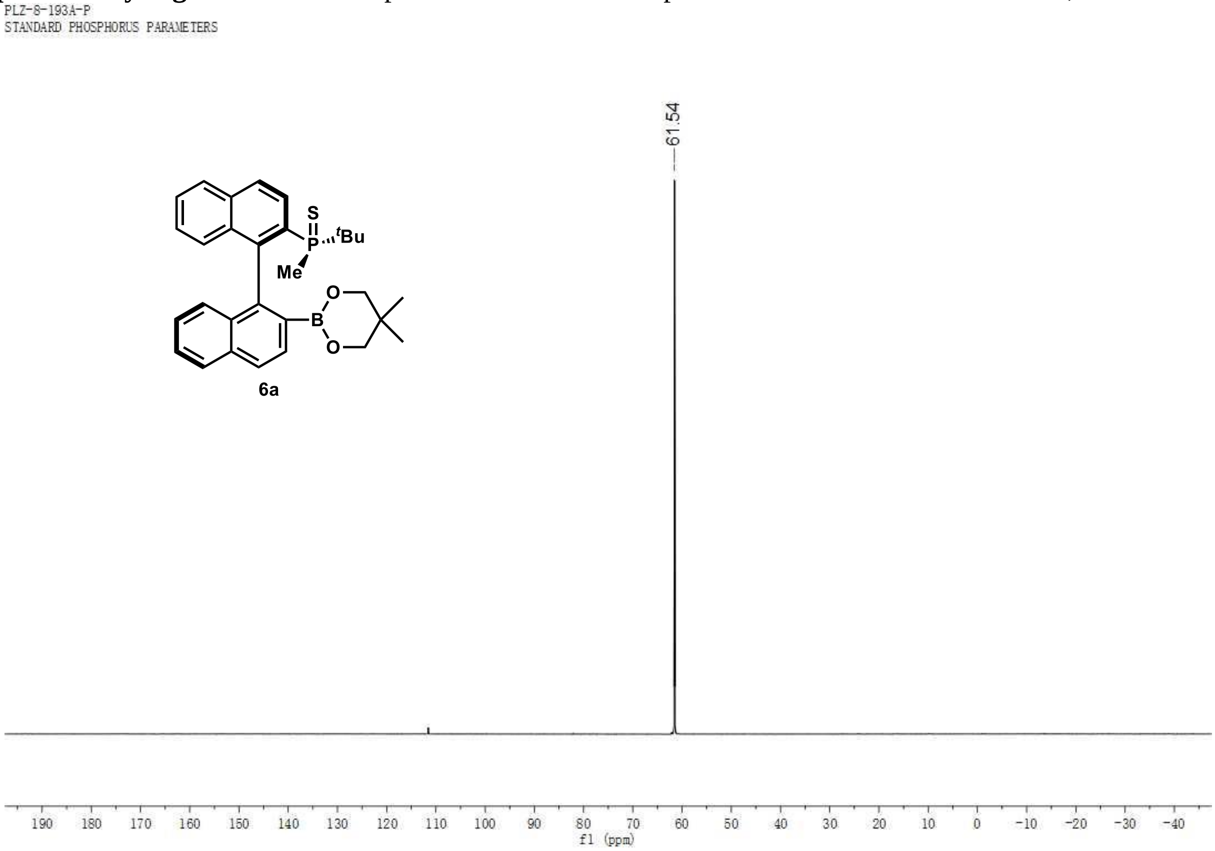

Supplementary Fig. 224.^{31}P NMR spectrum of **6a**. The sample has been recorded in 243 MHz, $CDCl_3$ at 25 ℃.

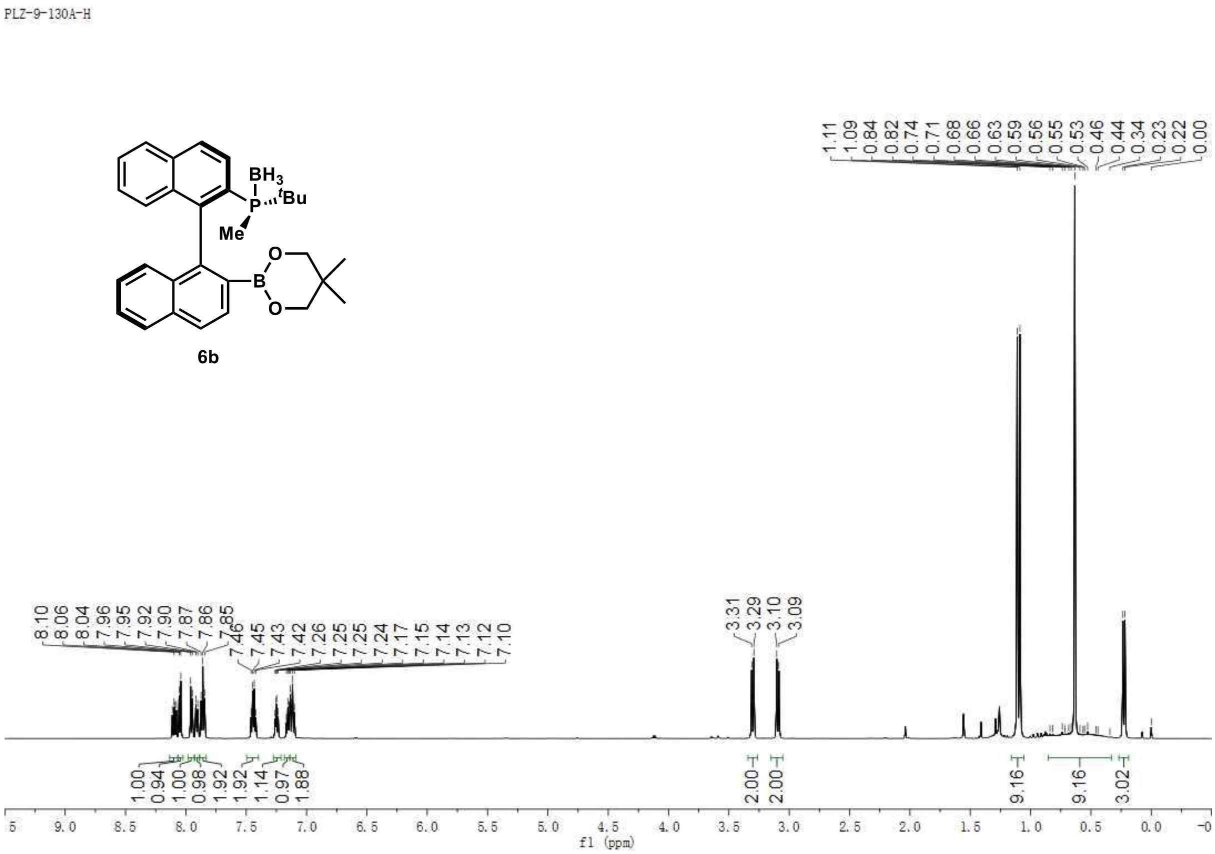

Supplementary Fig. 225. ^{1}H NMR spectrum of **6b**. The sample has been recorded in 600 MHz, $CDCl_3$ at 25 ℃.

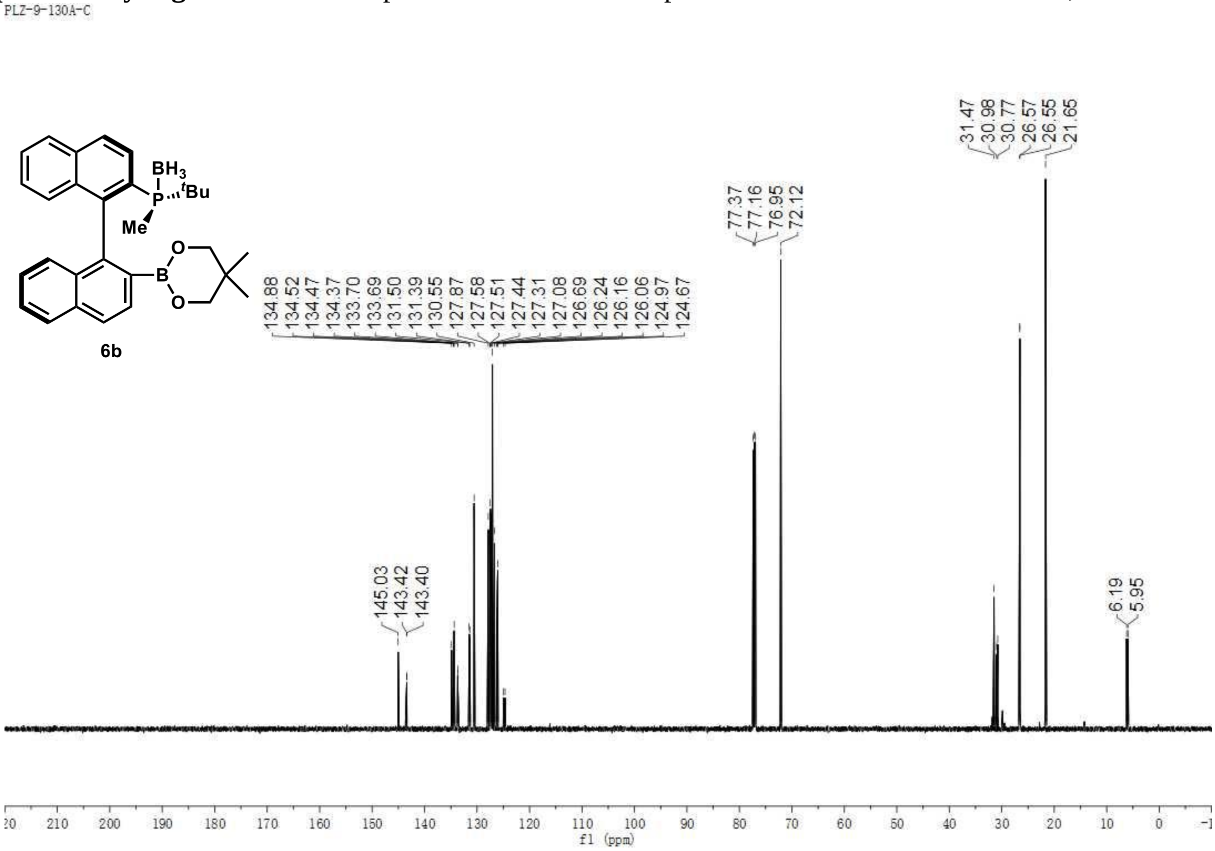

Supplementary Fig. 226.^{13}C NMR spectrum of **6b**. The sample has been recorded in 151 MHz, $CDCl_3$ at 25 ℃.

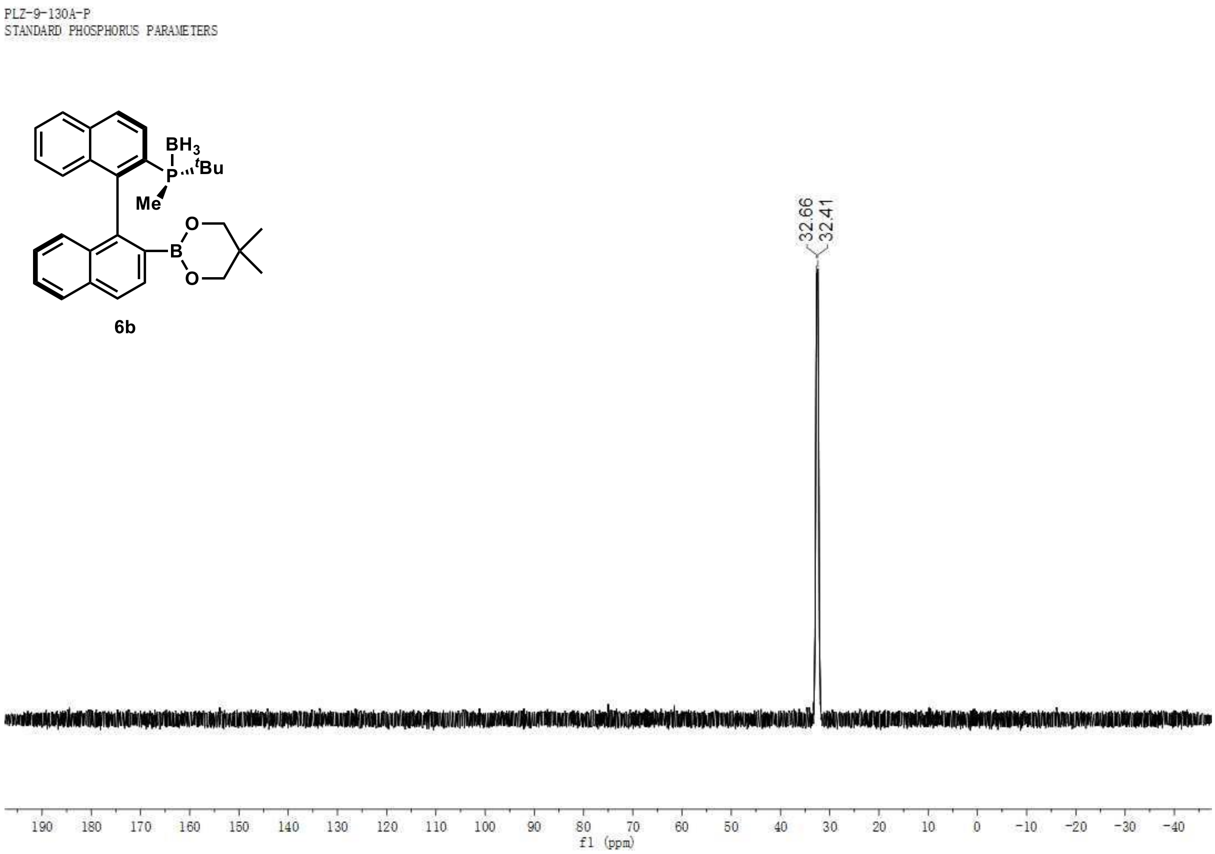

Supplementary Fig. 227.^{31}P NMR spectrum of **6b**. The sample has been recorded in 243 MHz, $CDCl_3$ at 25 ℃.

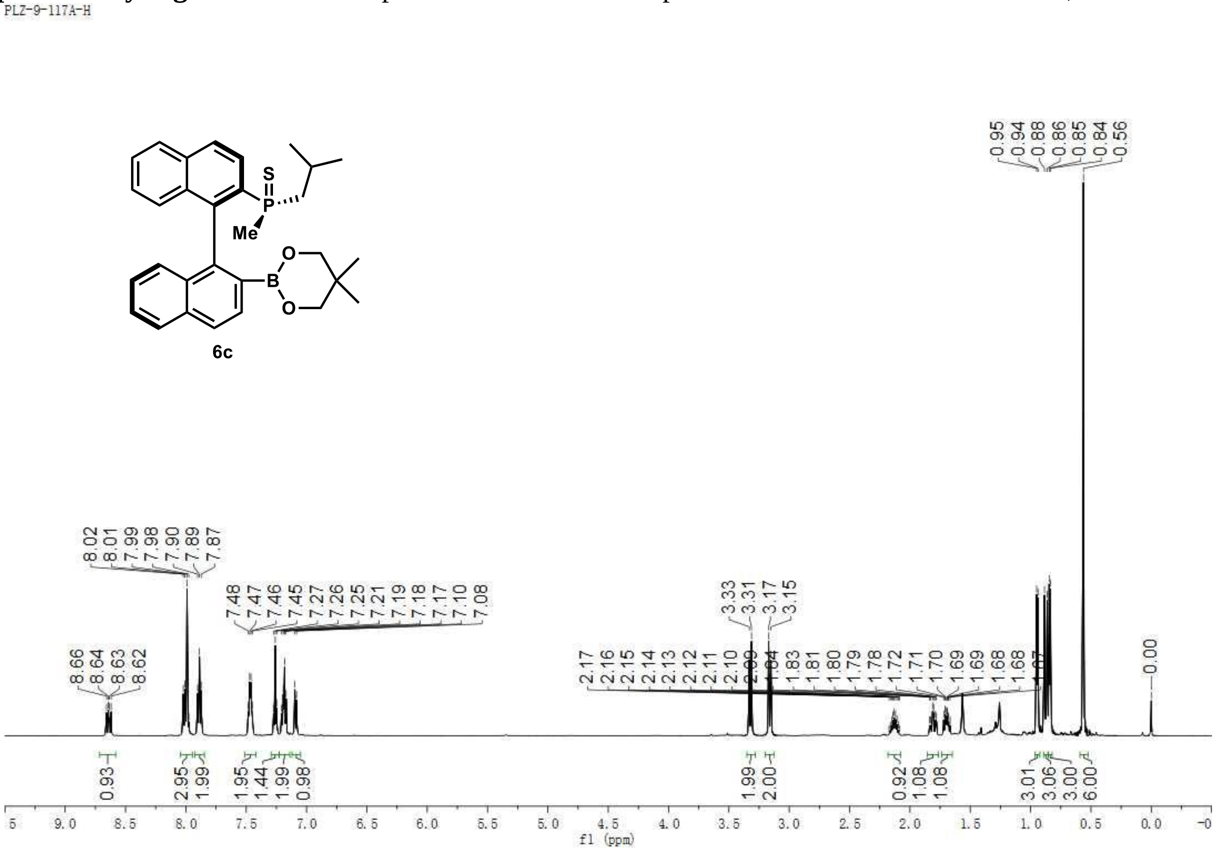

Supplementary Fig. 228. ^{1}H NMR spectrum of **6c**. The sample has been recorded in 600 MHz, $CDCl_3$ at 25 ℃.

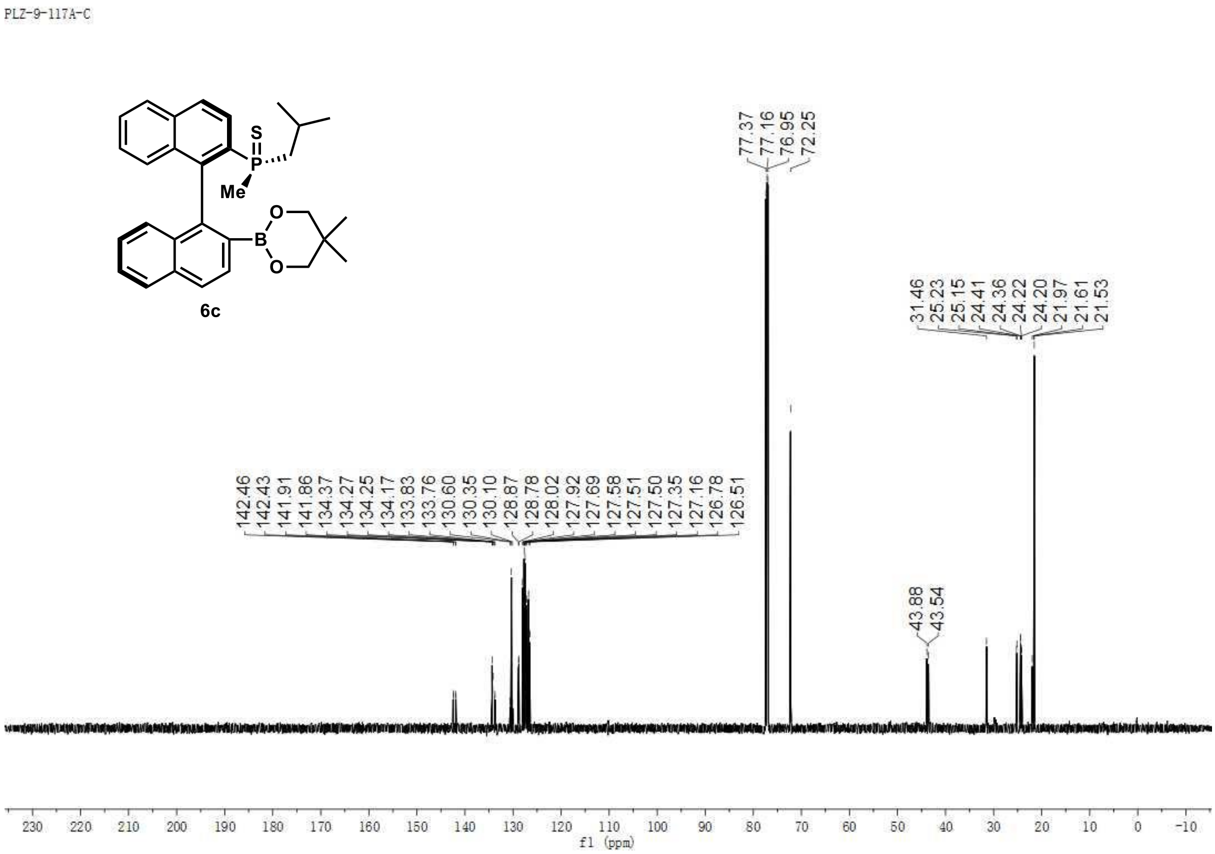

Supplementary Fig. 229.^{13}C NMR spectrum of **6c**. The sample has been recorded in 151 MHz, $CDCl_3$ at 25 ℃.

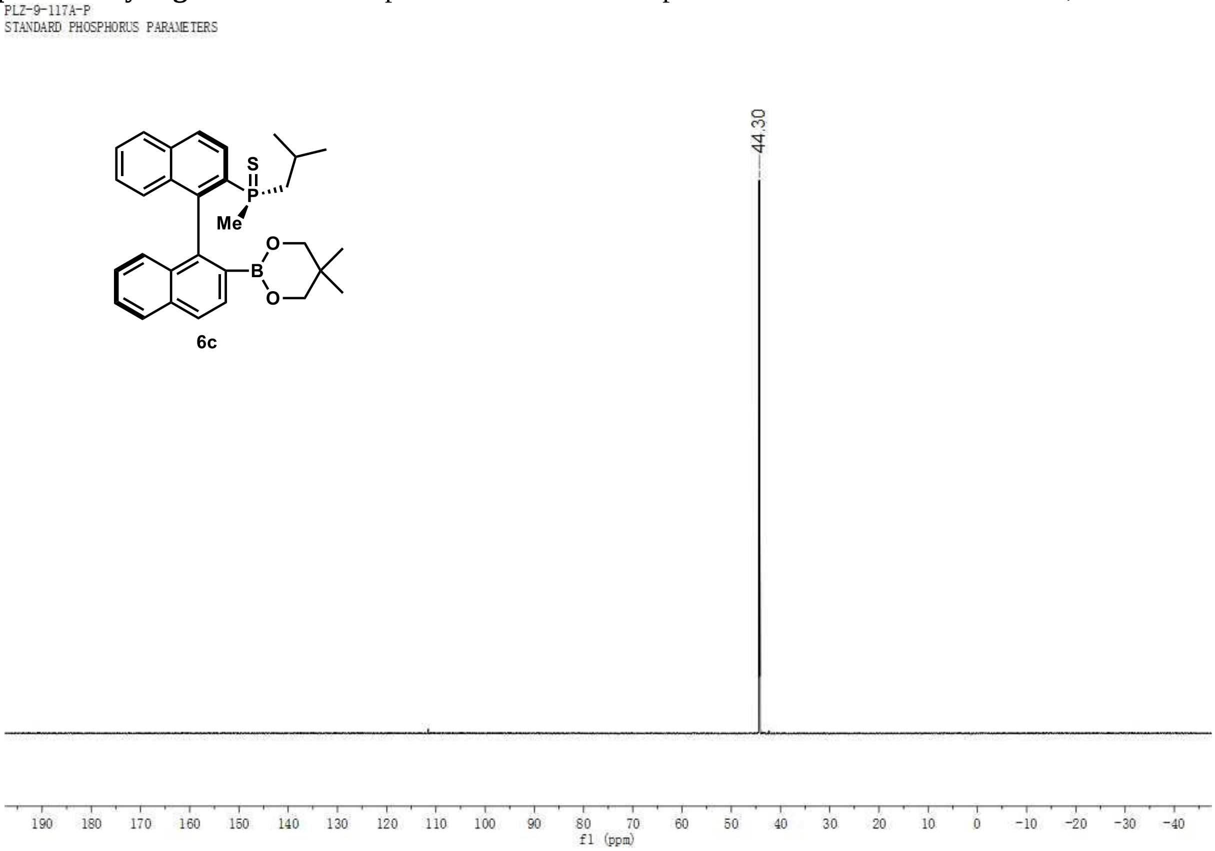

Supplementary Fig. 230.^{31}P NMR spectrum of **6c**. The sample has been recorded in 243 MHz, $CDCl_3$ at 25 ℃.

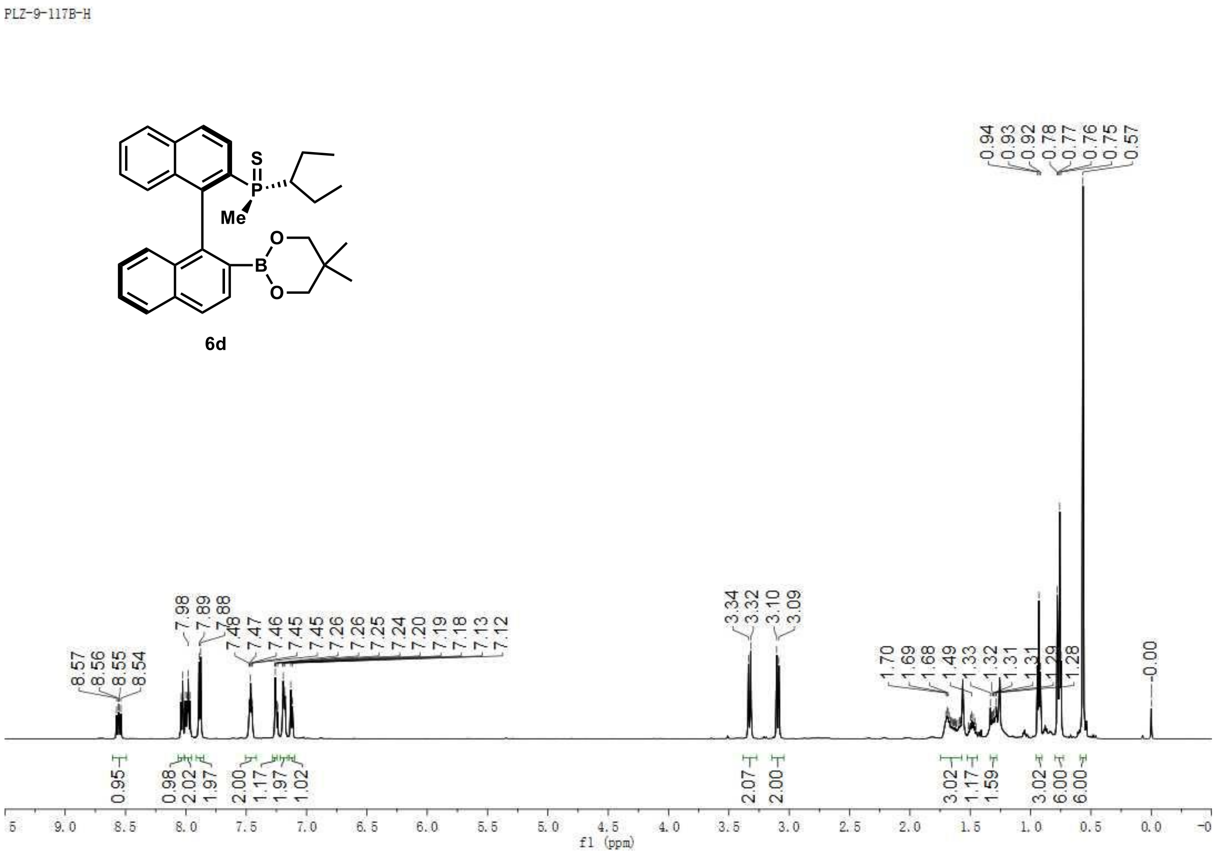

Supplementary Fig. 231. ^{1}H NMR spectrum of **6d**. The sample has been recorded in 600 MHz, $CDCl_3$ at 25 ℃.

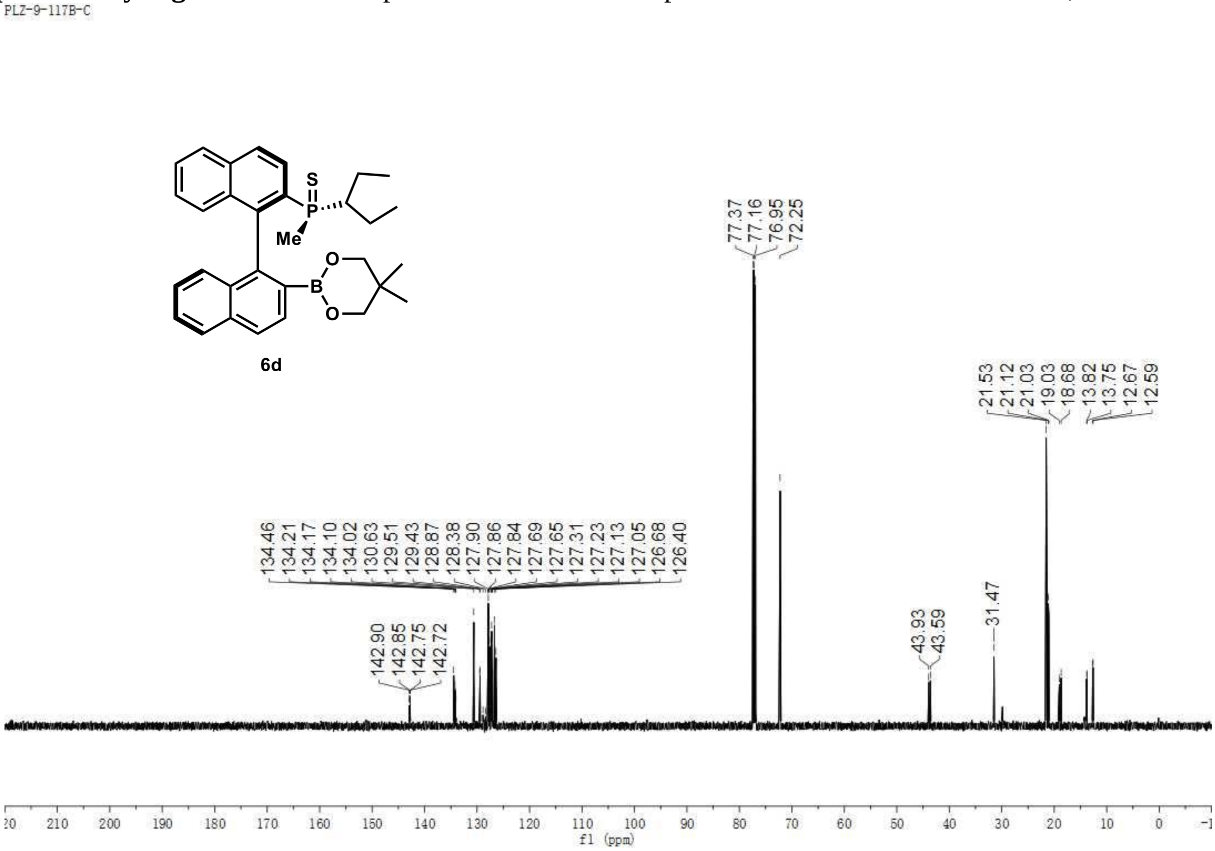

Supplementary Fig. 232.^{13}C NMR spectrum of **6d**. The sample has been recorded in 151 MHz, $CDCl_3$ at 25 ℃.

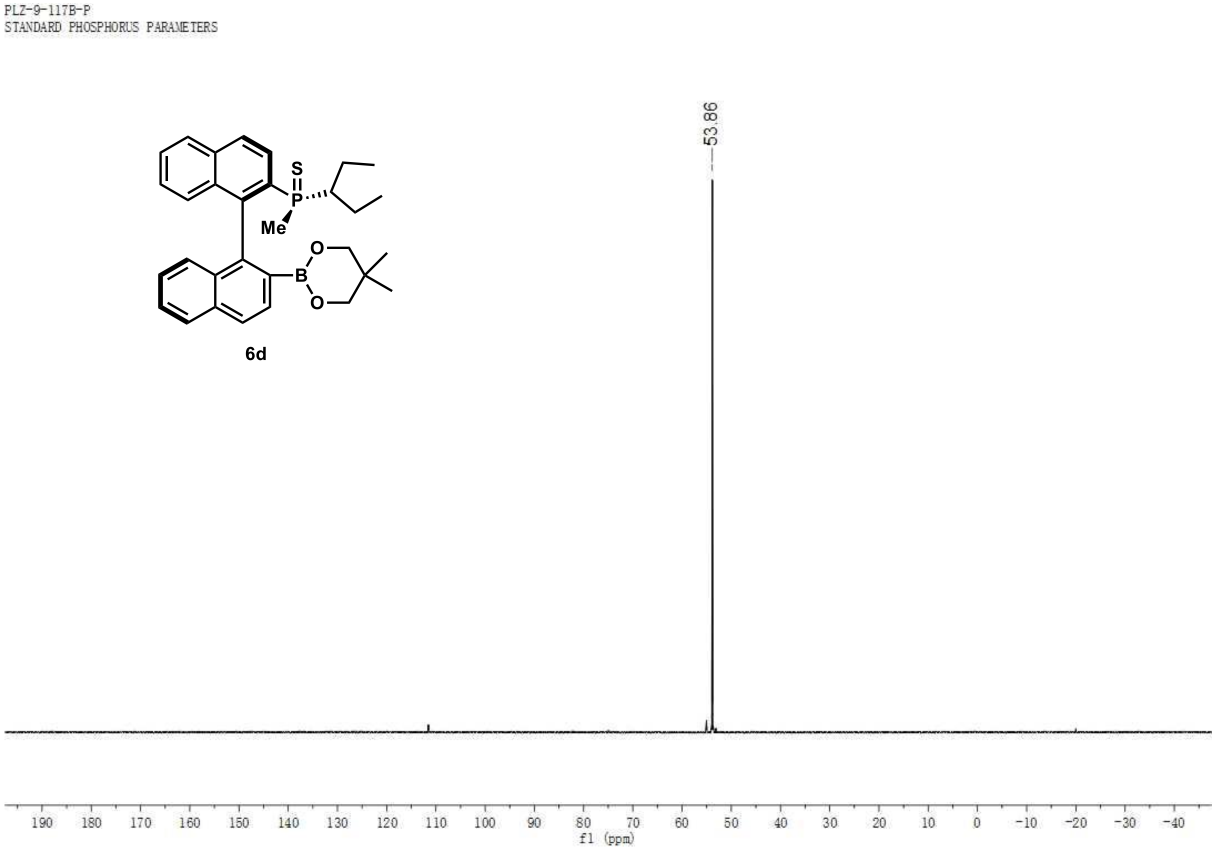

Supplementary Fig. 233.^{31}P NMR spectrum of **6d**. The sample has been recorded in 243 MHz, $CDCl_3$ at 25 ℃.

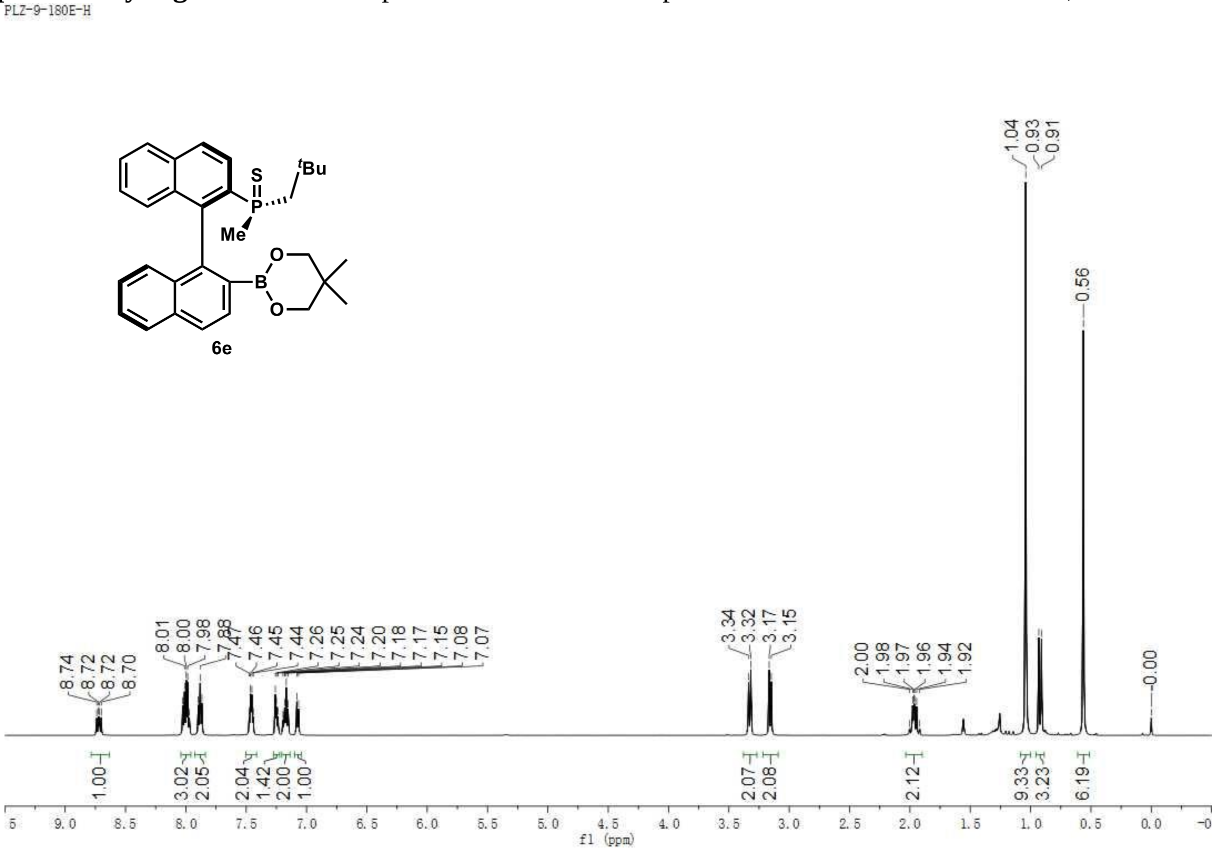

Supplementary Fig. 234. ^{1}H NMR spectrum of **6e**. The sample has been recorded in 600 MHz, $CDCl_3$ at 25 ℃.

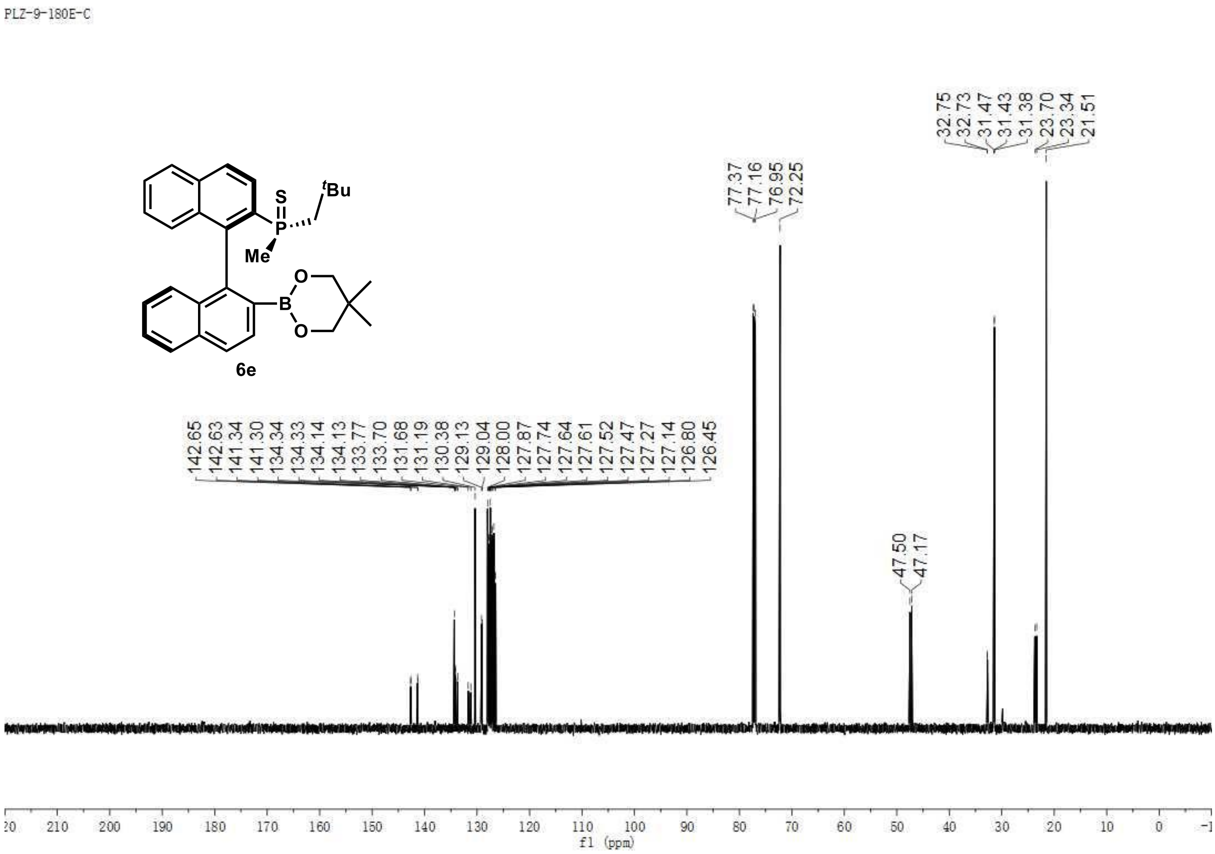

Supplementary Fig. 235.^{13}C NMR spectrum of **6e**. The sample has been recorded in 151 MHz, $CDCl_3$ at 25 ℃.

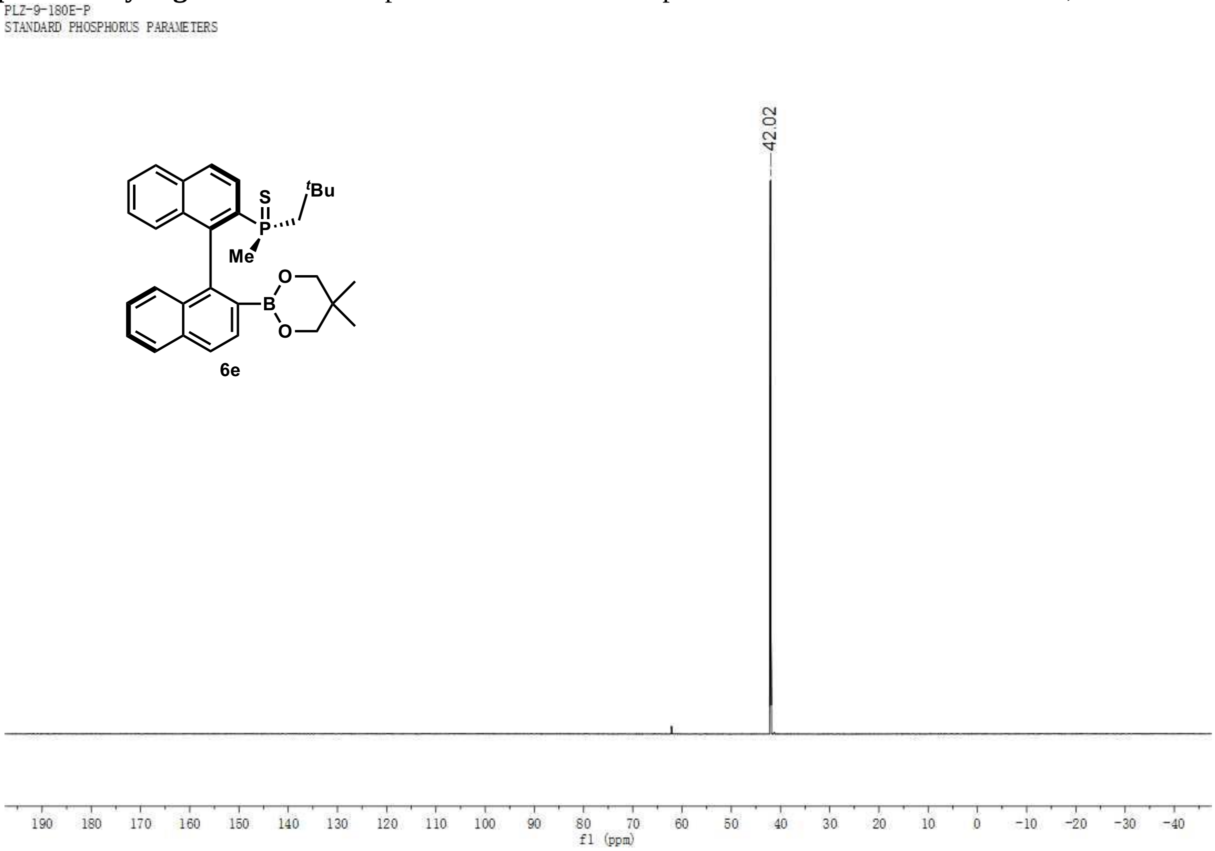

Supplementary Fig. 236.^{31}P NMR spectrum of **6e**. The sample has been recorded in 243 MHz, $CDCl_3$ at 25 ℃.

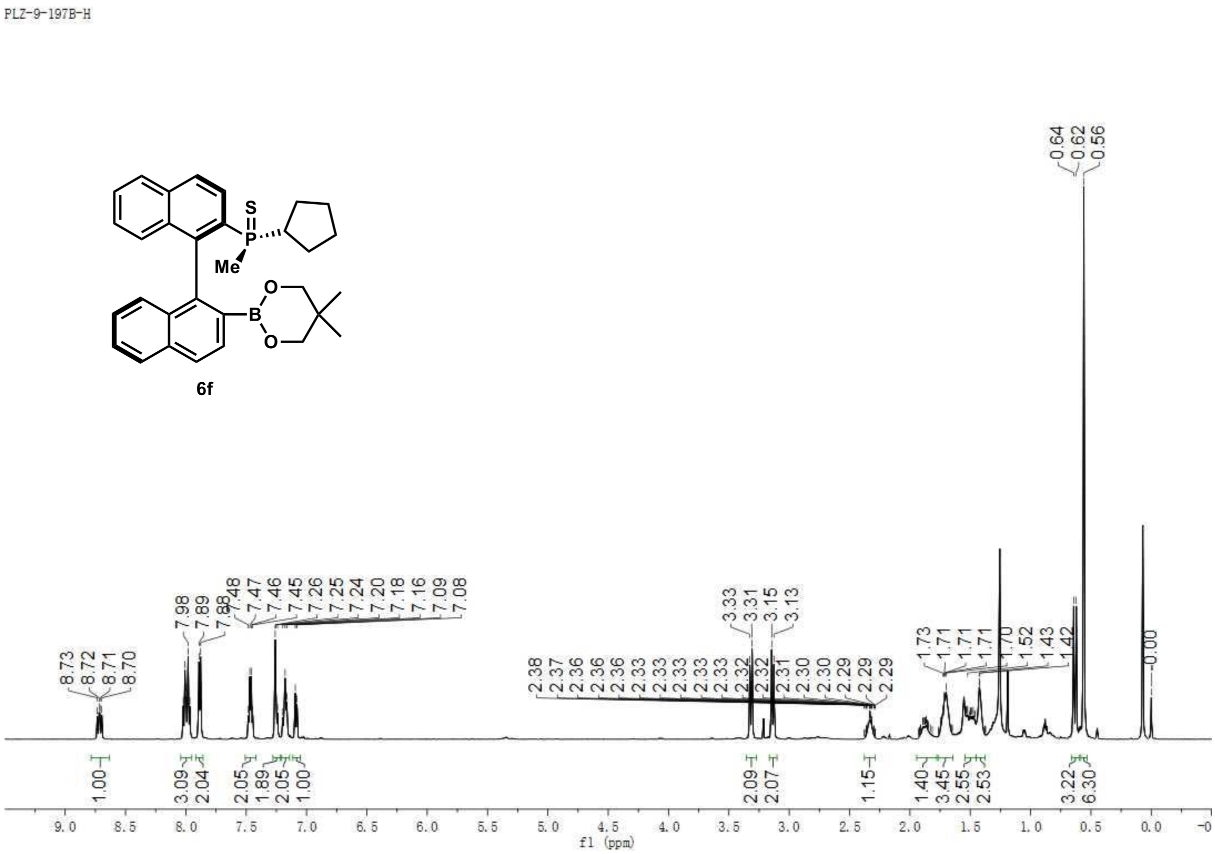

Supplementary Fig. 237. ^{1}H NMR spectrum of **6f**. The sample has been recorded in 600 MHz, $CDCl_3$ at 25 ℃.

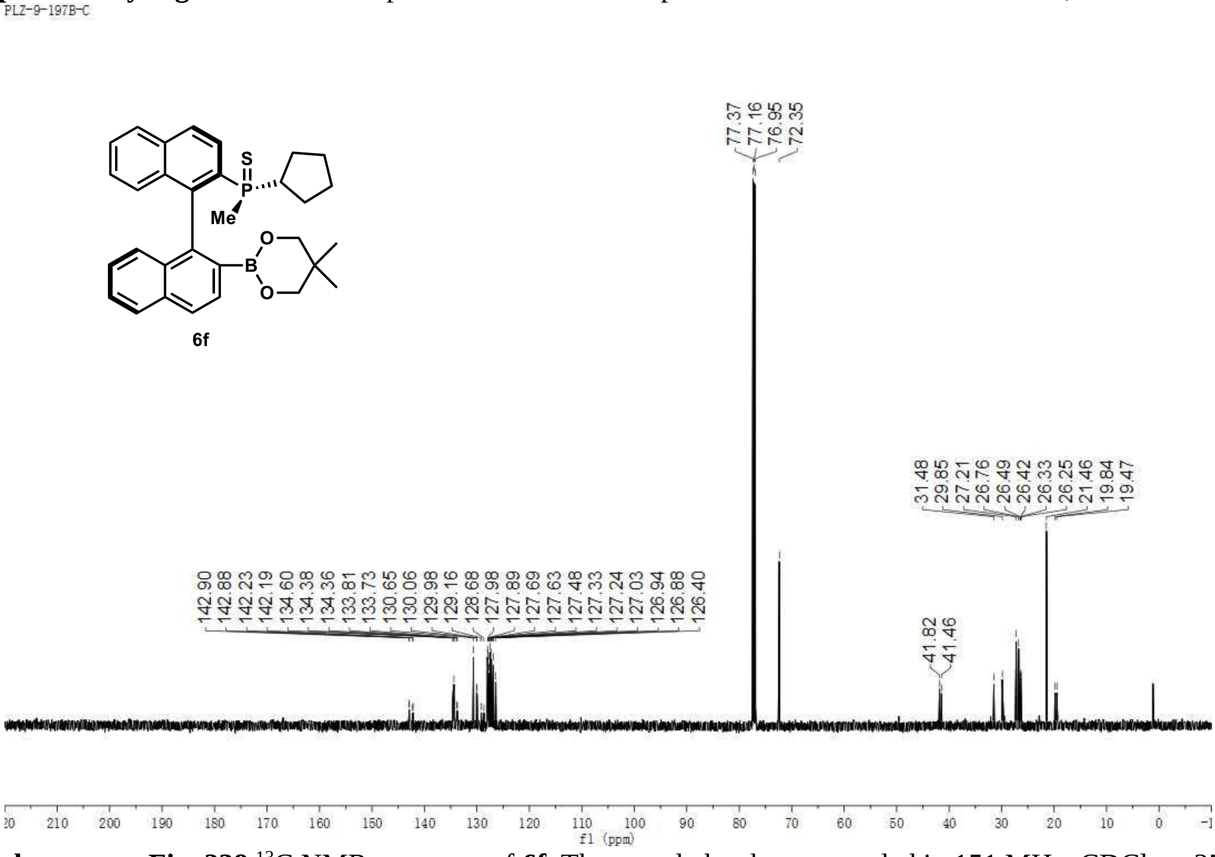

Supplementary Fig. 238.^{13}C NMR spectrum of **6f**. The sample has been recorded in 151 MHz, $CDCl_3$ at 25 ℃.

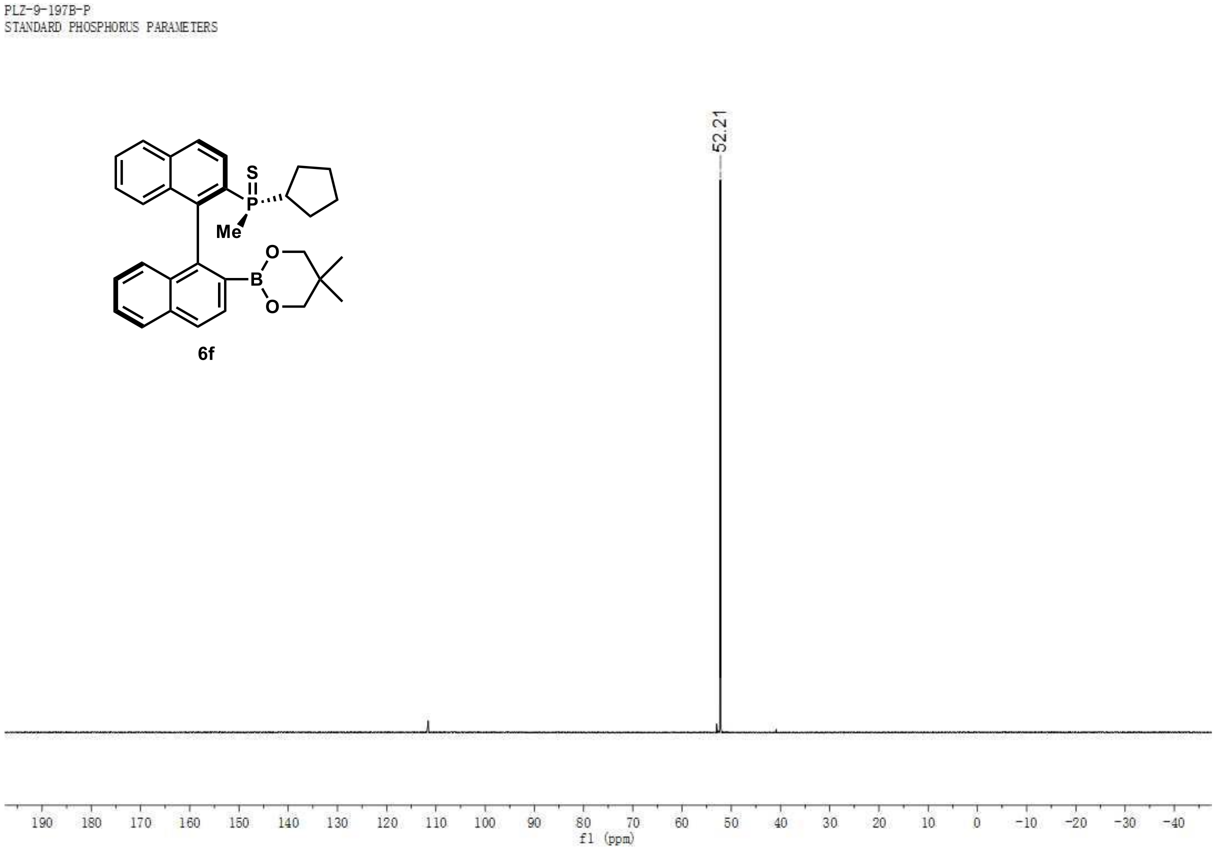

Supplementary Fig. 239. ^{31}P NMR spectrum of **6f**. The sample has been recorded in 243 MHz, $CDCl_3$ at 25 ℃.

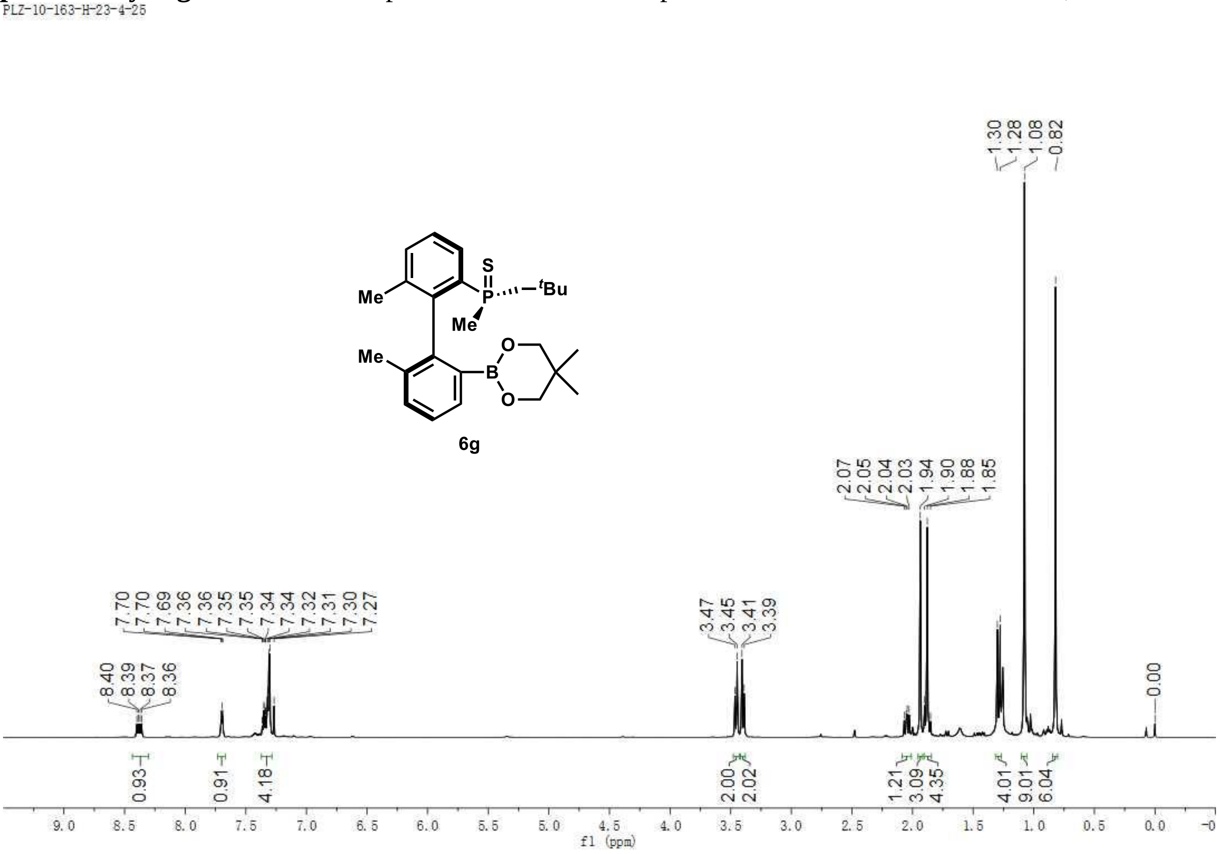

Supplementary Fig. 240. ^{1}H NMR spectrum of **6g**. The sample has been recorded in 600 MHz, $CDCl_3$ at 25 ℃.

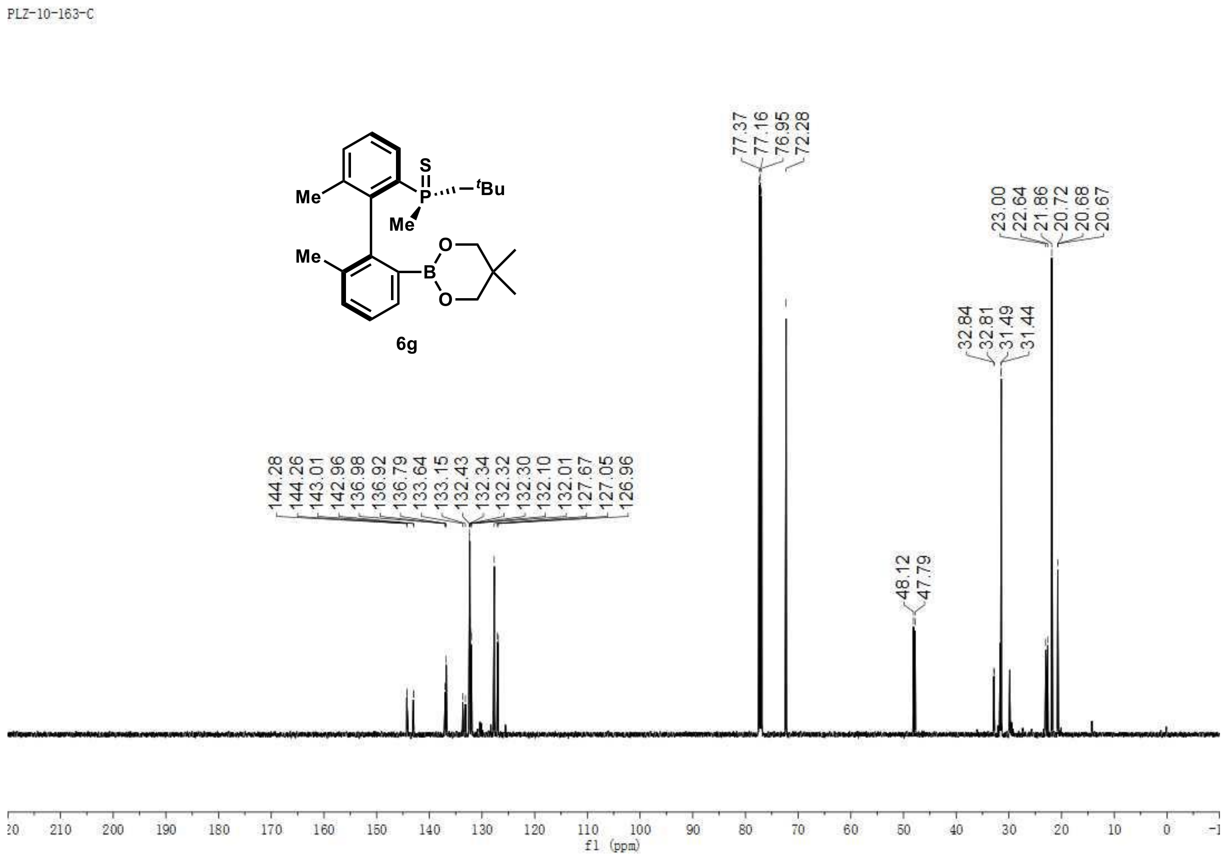

Supplementary Fig. 241.^{13}C NMR spectrum of **6g**. The sample has been recorded in 151 MHz, $CDCl_3$ at 25 ℃.

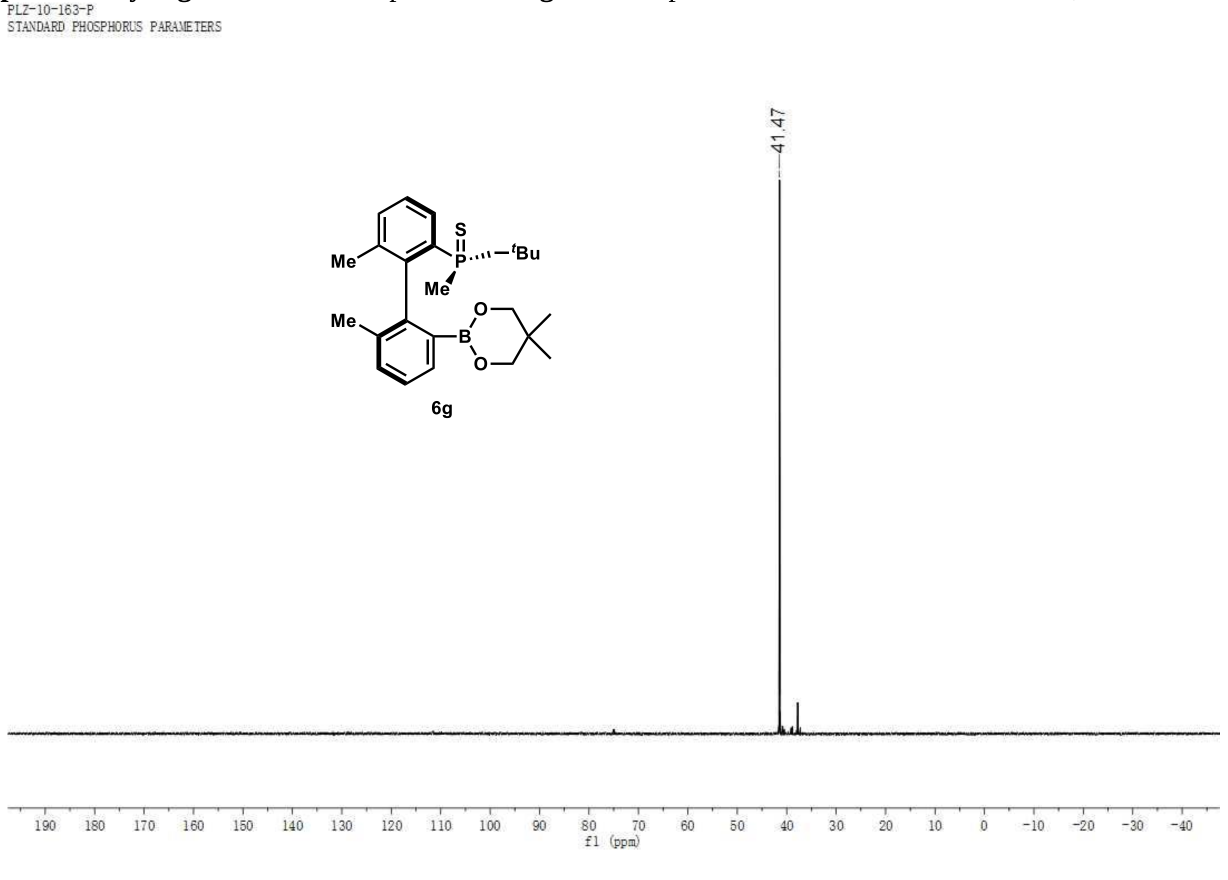

Supplementary Fig. 242.^{31}P NMR spectrum of **6g**. The sample has been recorded in 243 MHz, $CDCl_3$ at 25 ℃.

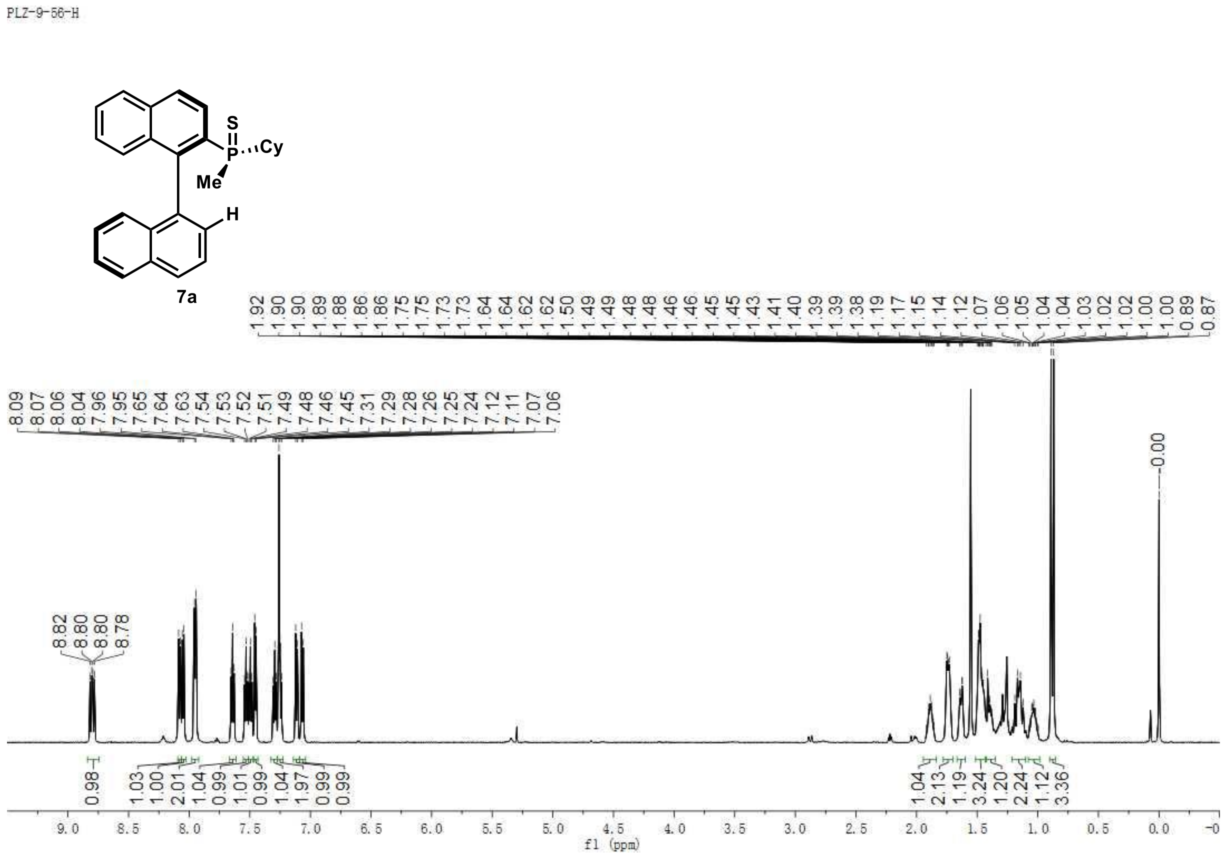

Supplementary Fig. 243. ^{1}H NMR spectrum of **7a**. The sample has been recorded in 600 MHz, $CDCl_3$ at 25 ℃.

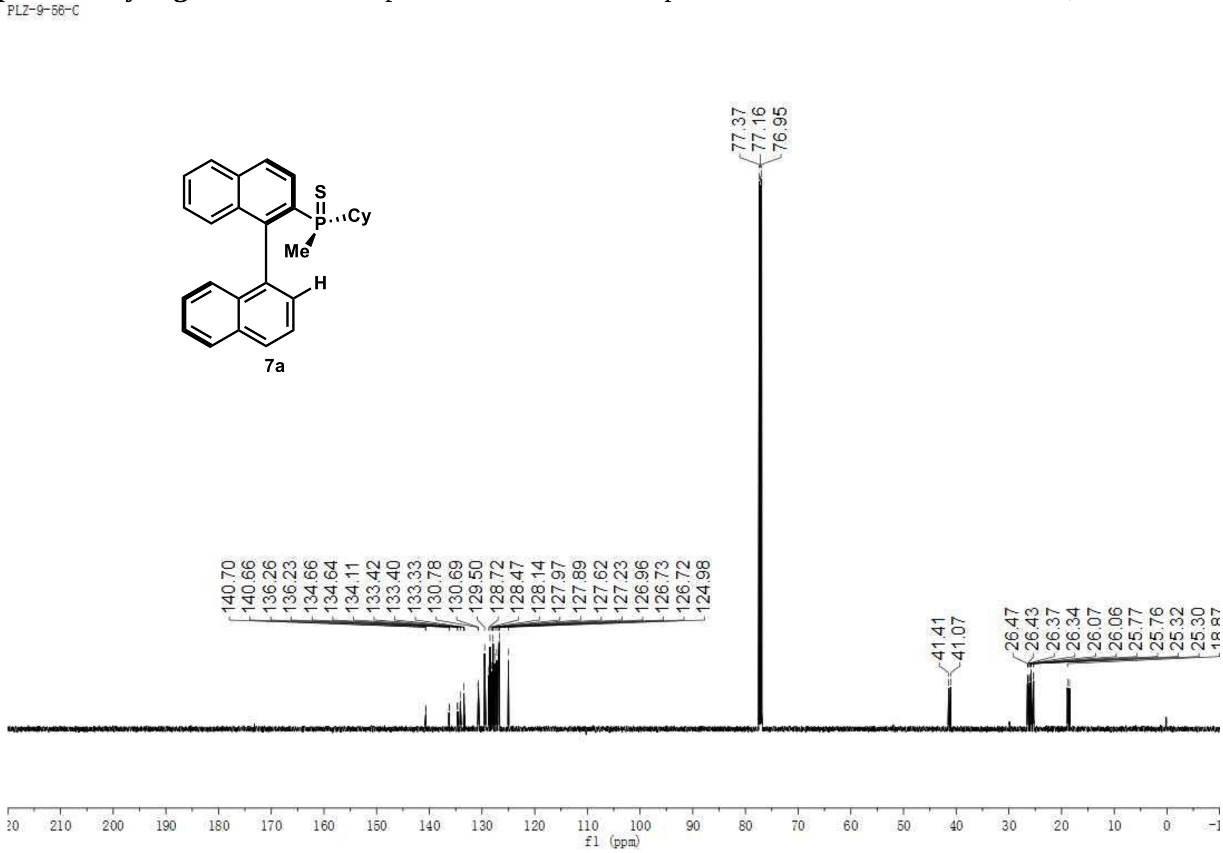

Supplementary Fig. 244.^{13}C NMR spectrum of **7a**. The sample has been recorded in 151 MHz, $CDCl_3$ at 25 ℃.

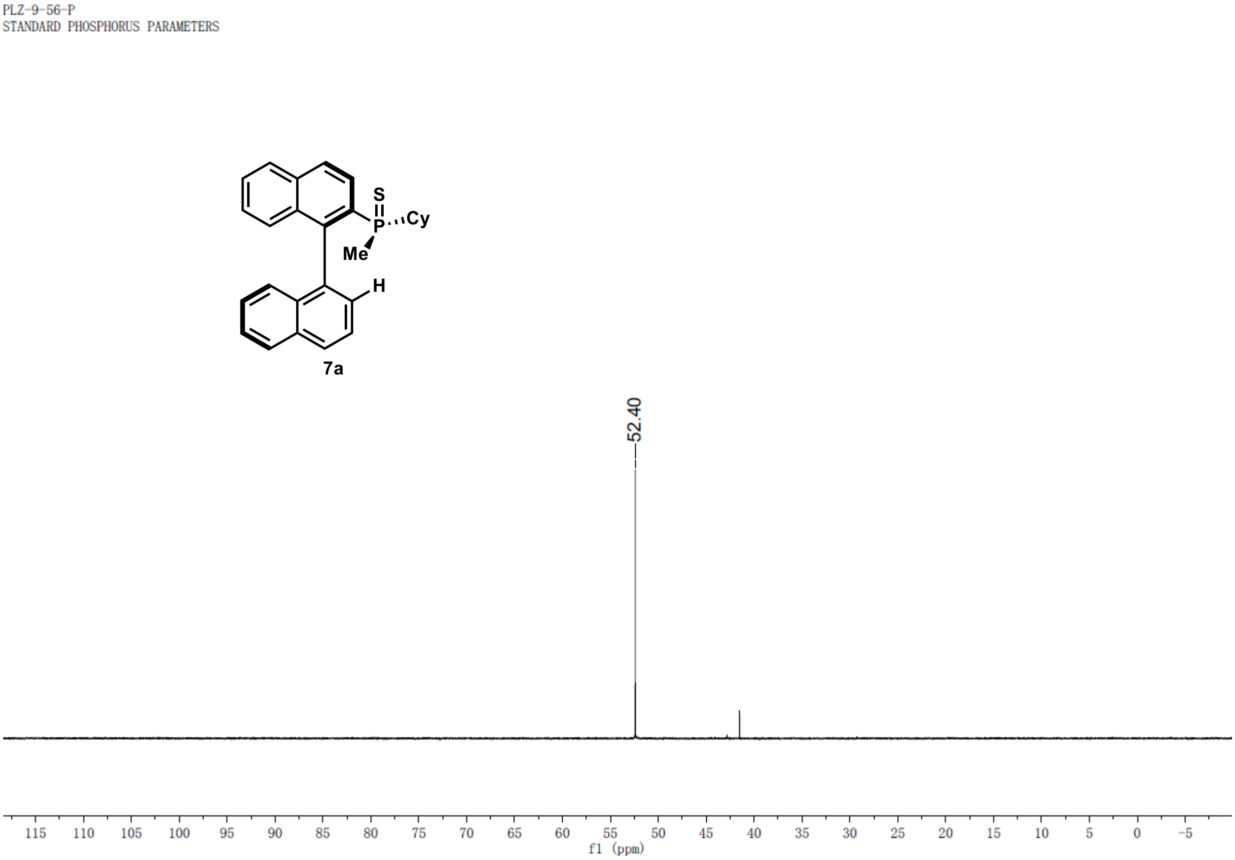

Supplementary Fig. 245.^{31}P NMR spectrum of **7a**. The sample has been recorded in 243 MHz, $CDCl_3$ at 25 ℃.

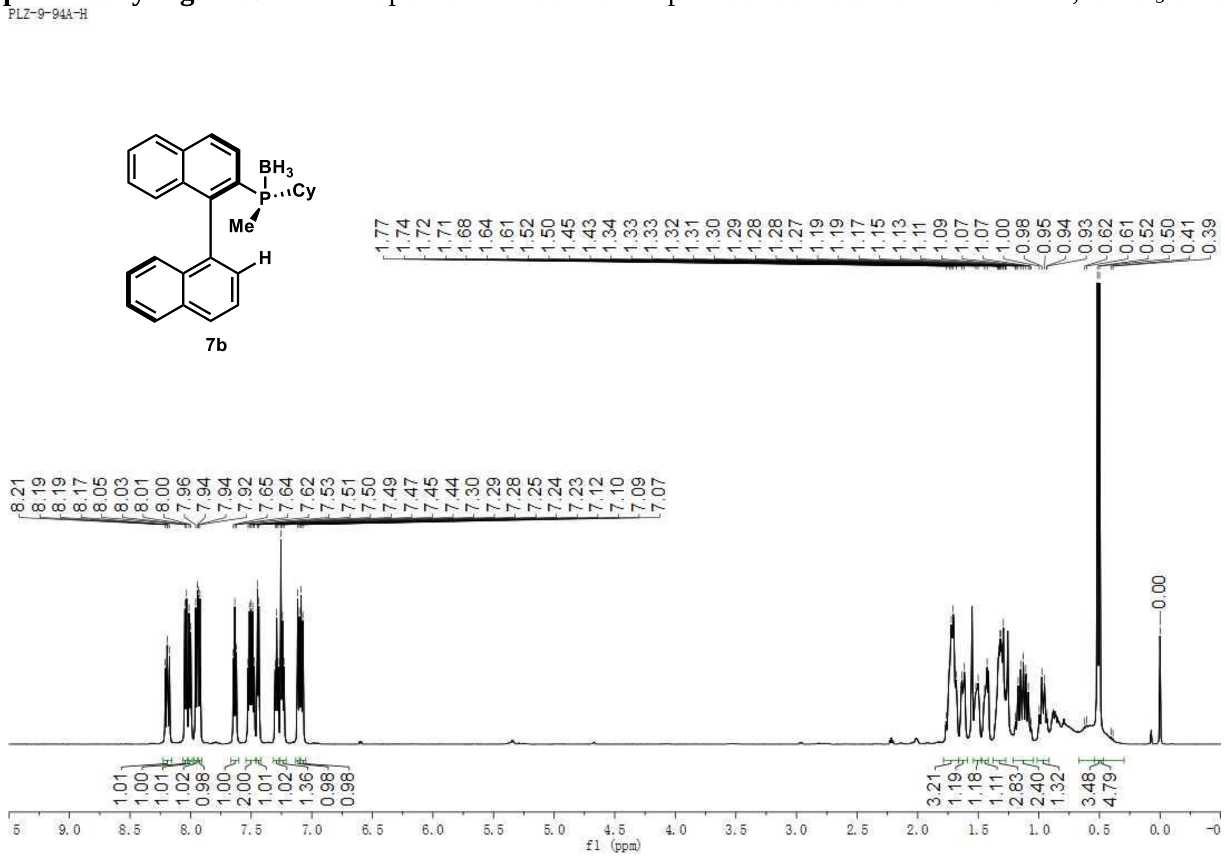

Supplementary Fig. 246. ^{1}H NMR spectrum of **7b**. The sample has been recorded in 600 MHz, $CDCl_3$ at 25 ℃.

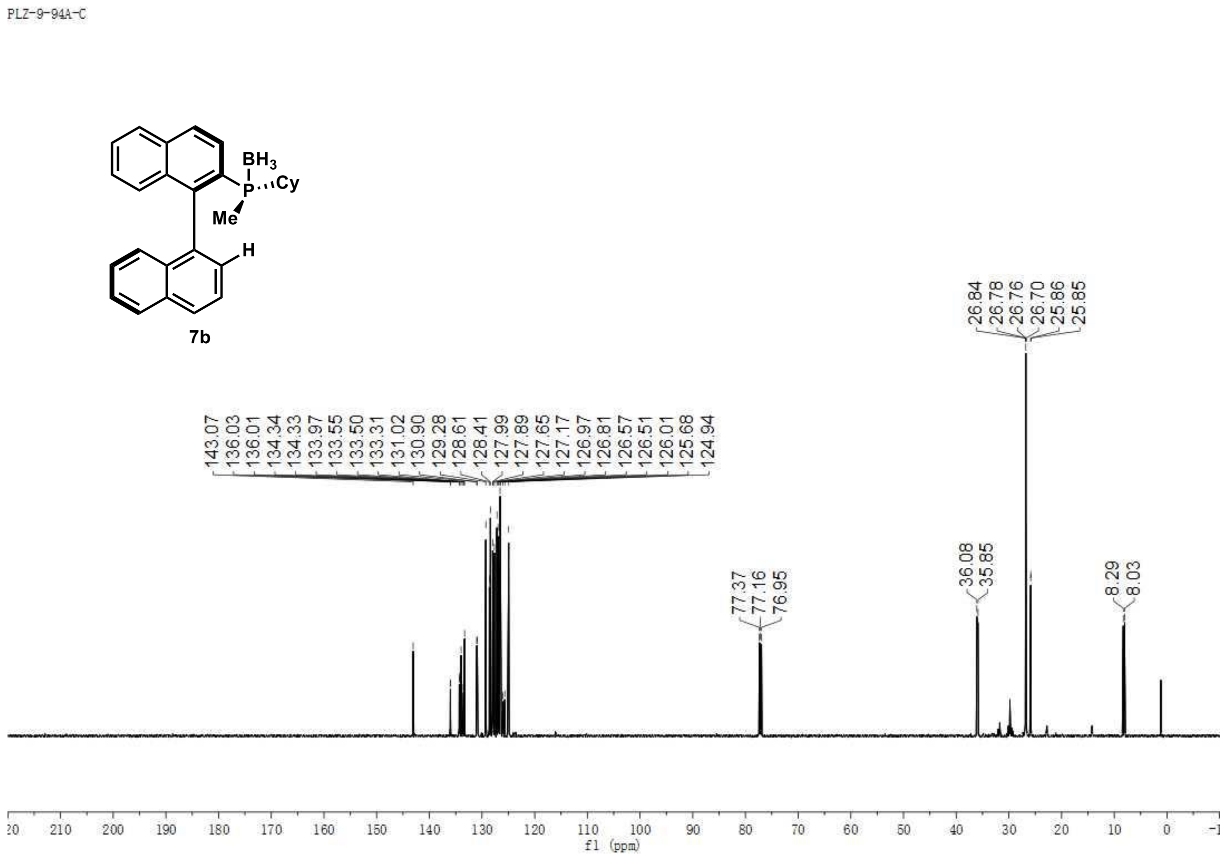

Supplementary Fig. 247.^{13}C NMR spectrum of **7b**. The sample has been recorded in 151 MHz, $CDCl_3$ at 25 ℃.

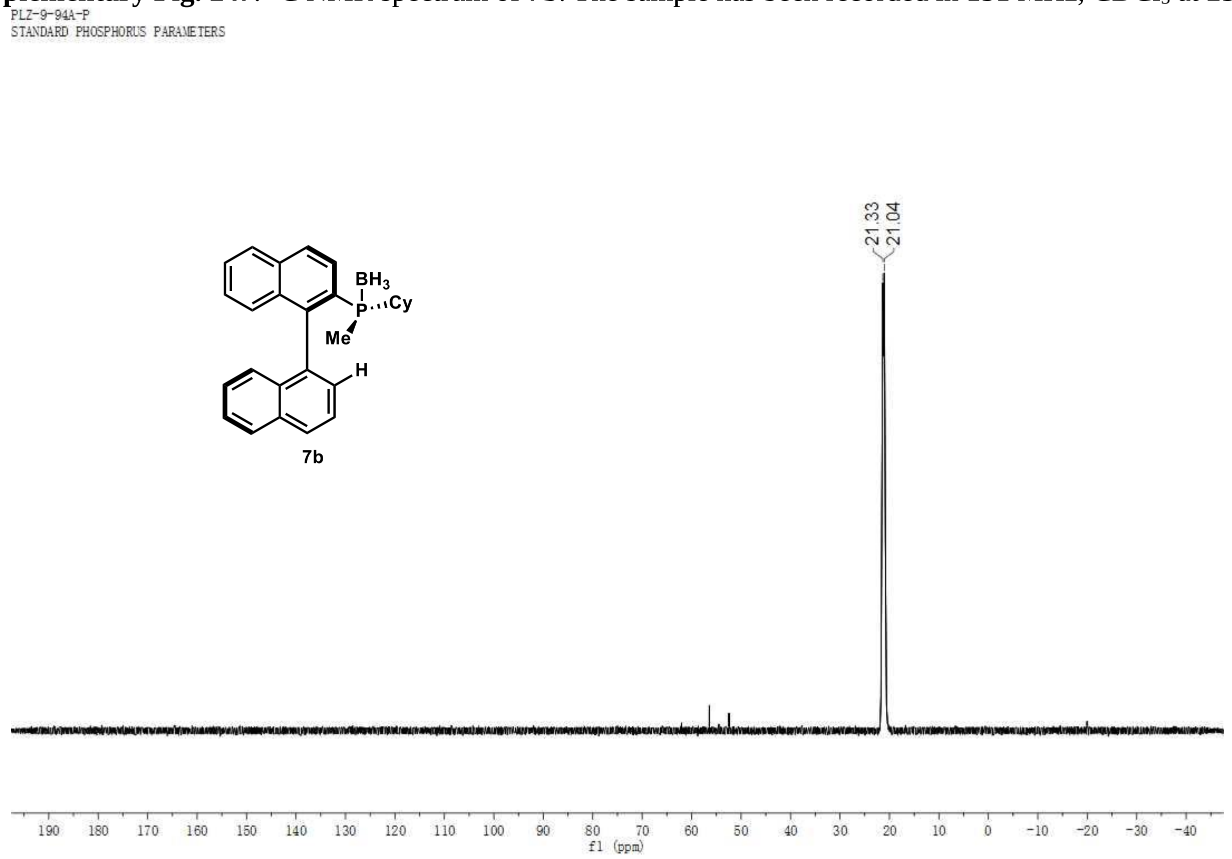

Supplementary Fig. 248.^{31}P NMR spectrum of **7b**. The sample has been recorded in 243 MHz, $CDCl_3$ at 25 ℃.

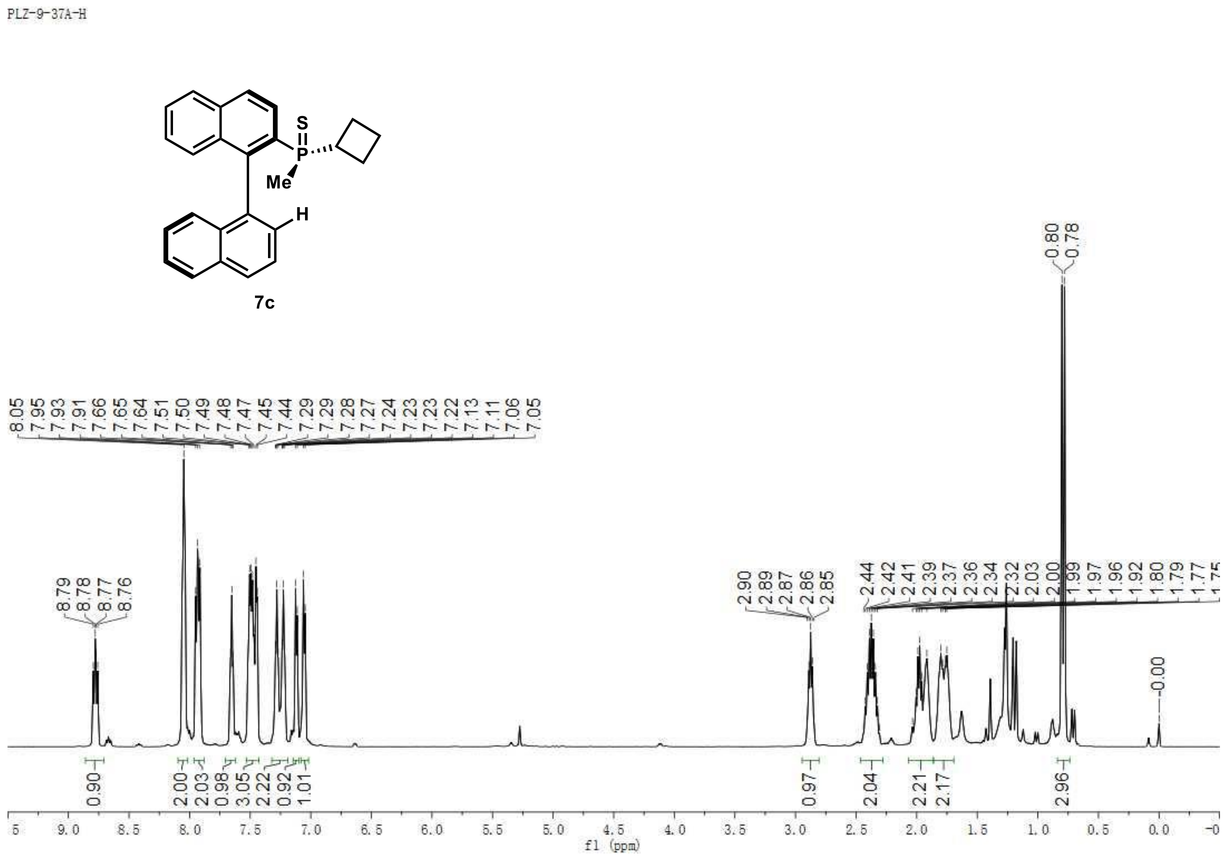

Supplementary Fig. 249. ^{1}H NMR spectrum of **7c**. The sample has been recorded in 600 MHz, $CDCl_3$ at 25 ℃.

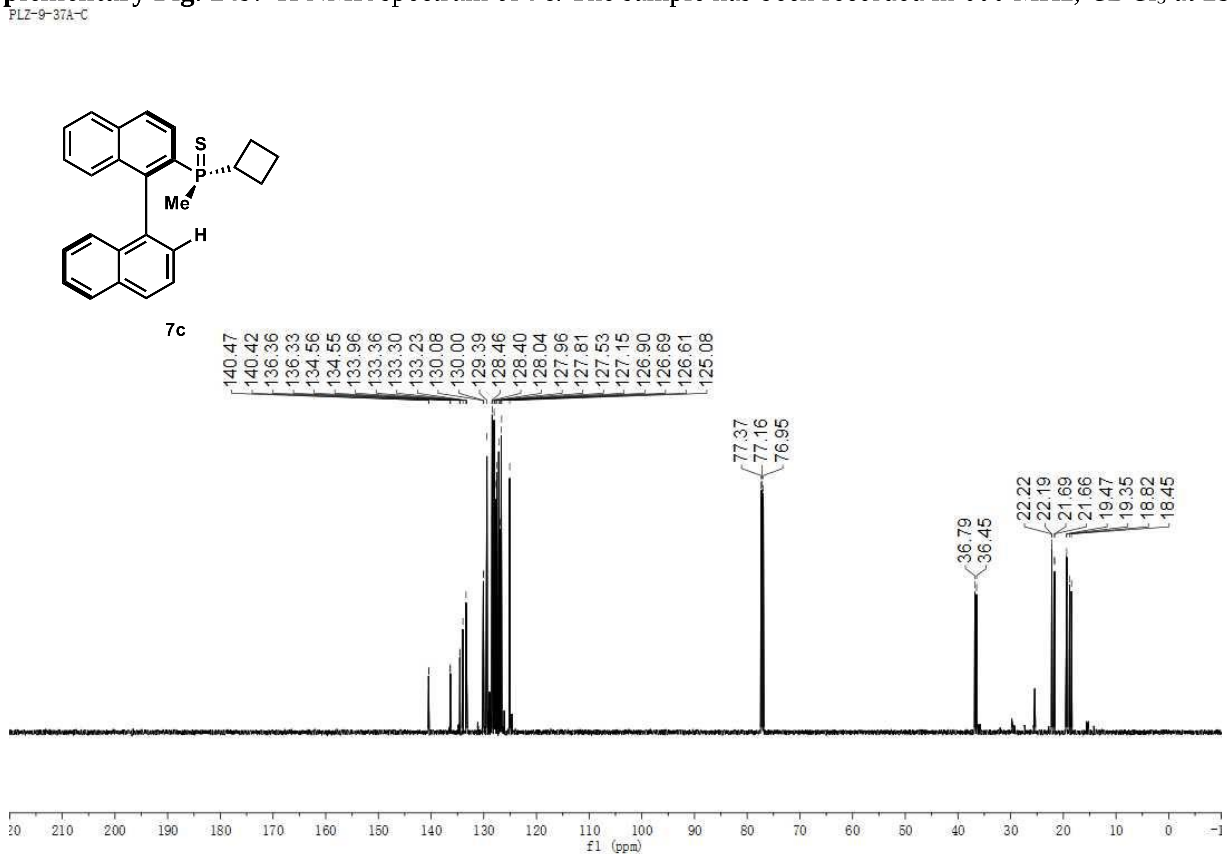

Supplementary Fig. 250.^{13}C NMR spectrum of **7c**. The sample has been recorded in 151 MHz, $CDCl_3$ at 25 ℃.

PLZ-9-37A-P
STANDARD PHOSPHORUS PARAMETERS

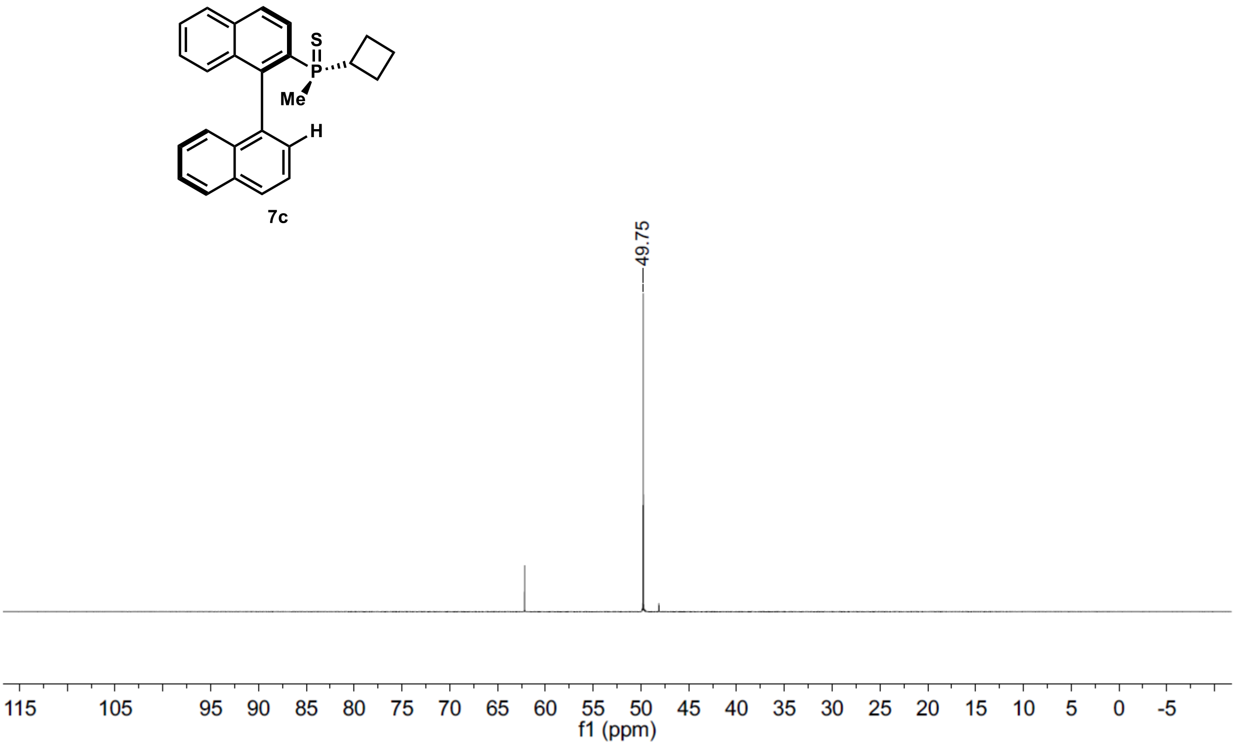

Supplementary Fig. 251.^{31}P NMR spectrum of **7c**. The sample has been recorded in 243 MHz, $CDCl_3$ at 25 ℃.

PLZ-9-3D-H

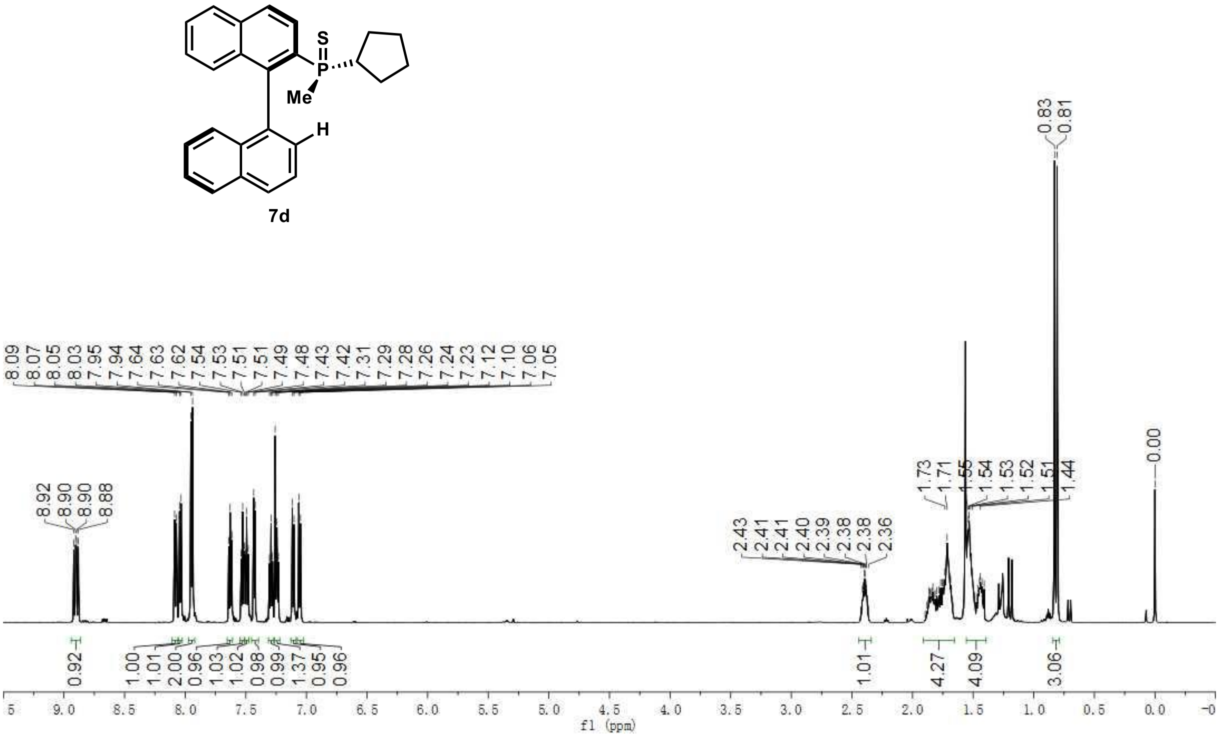

Supplementary Fig. 252. ^{1}H NMR spectrum of **7d**. The sample has been recorded in 600 MHz, $CDCl_3$ at 25 ℃.

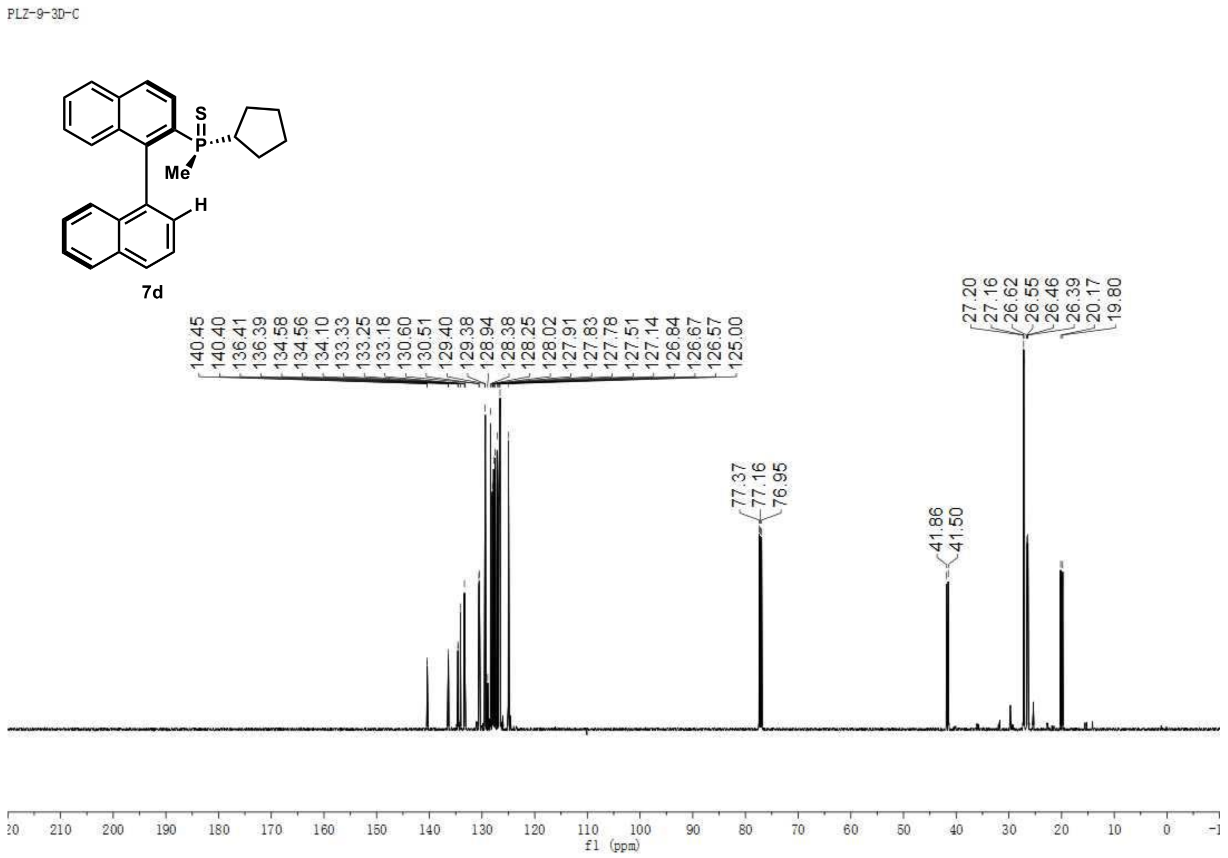

Supplementary Fig. 253.^{13}C NMR spectrum of **7d**. The sample has been recorded in 151 MHz, $CDCl_3$ at 25 ℃.

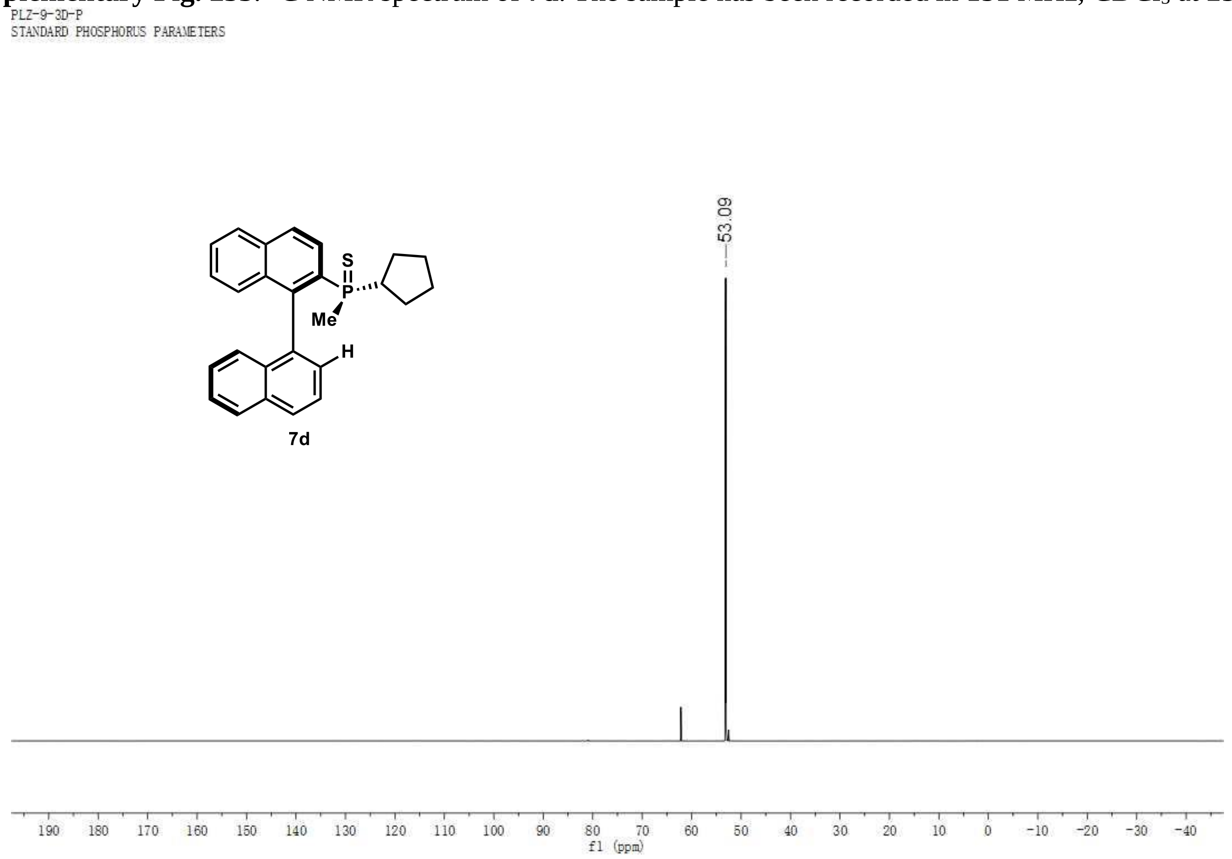

Supplementary Fig. 254.^{31}P NMR spectrum of **7d**. The sample has been recorded in 243 MHz, $CDCl_3$ at 25 ℃.

PLZ-9-12B-H

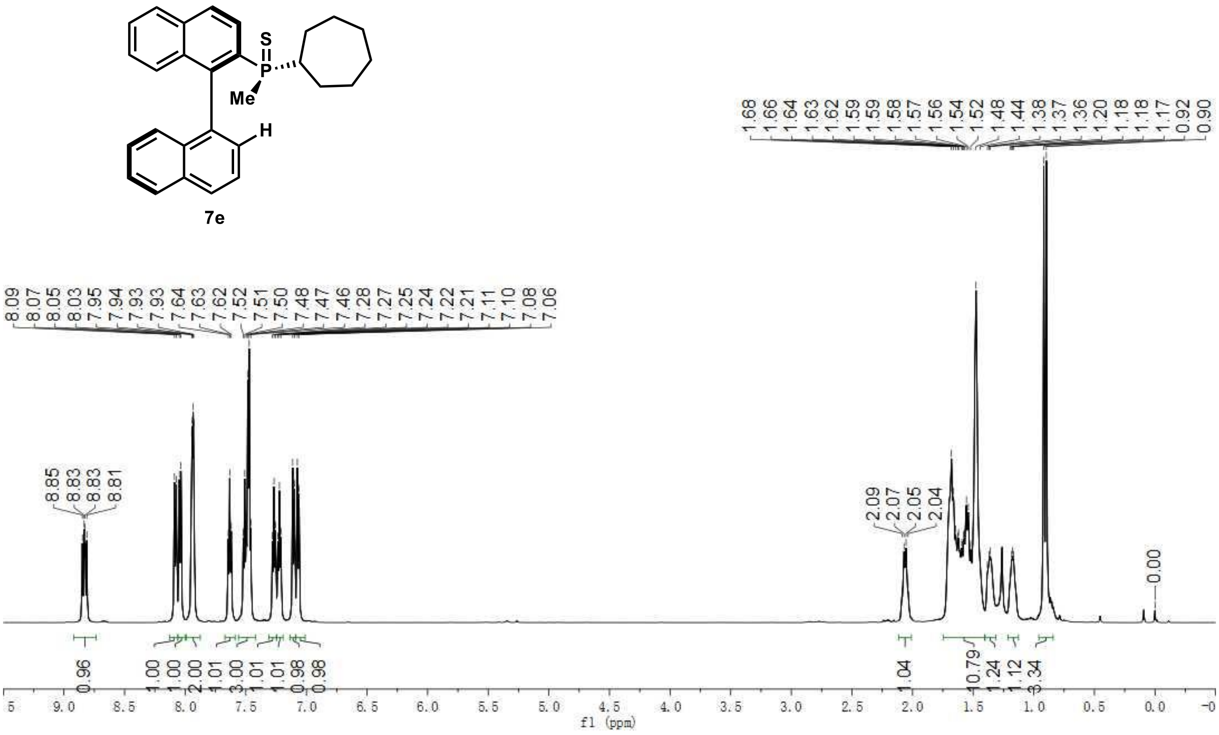

Supplementary Fig. 255. ^{1}H NMR spectrum of **7e**. The sample has been recorded in 600 MHz, $CDCl_3$ at 25 ℃.

PLZ-9-12B-C

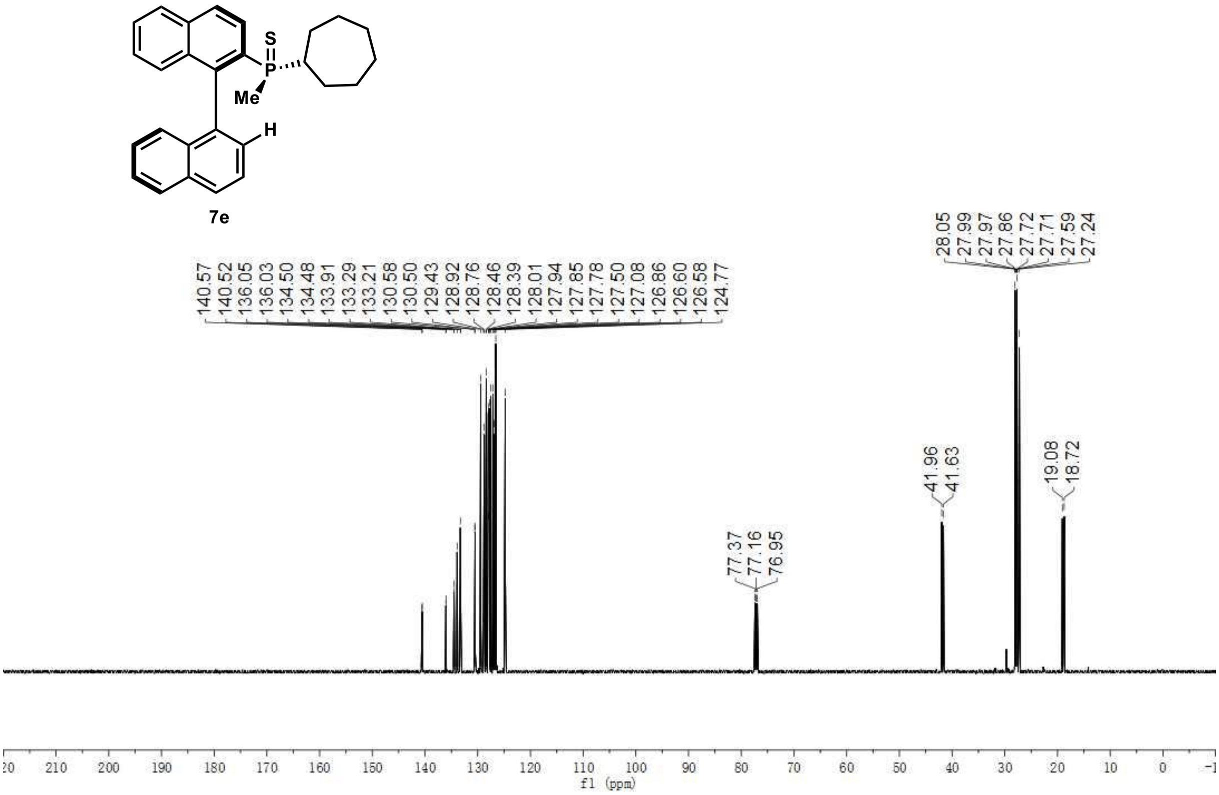

Supplementary Fig. 256.^{13}C NMR spectrum of **7e**. The sample has been recorded in 151 MHz, $CDCl_3$ at 25 ℃.

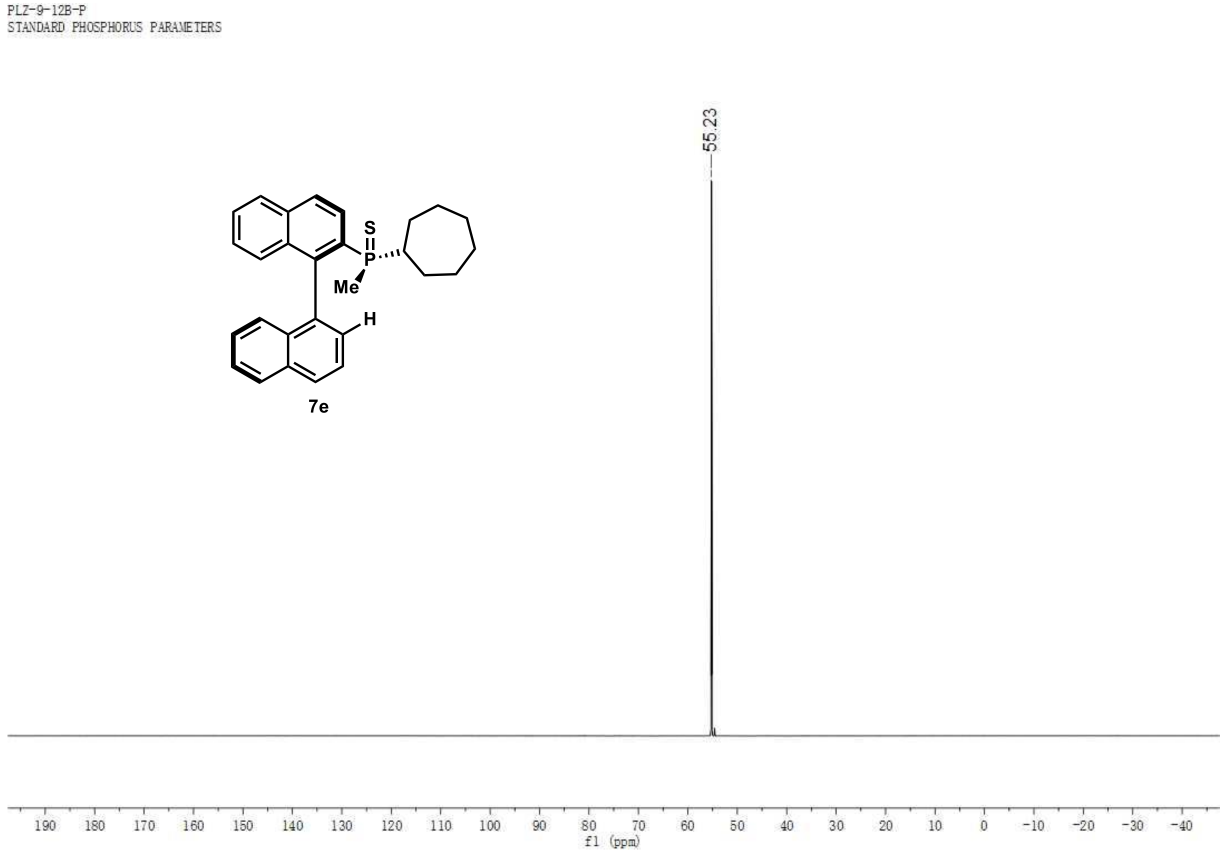

Supplementary Fig. 257.^{31}P NMR spectrum of **7e**. The sample has been recorded in 243 MHz, $CDCl_3$ at 25 ℃.

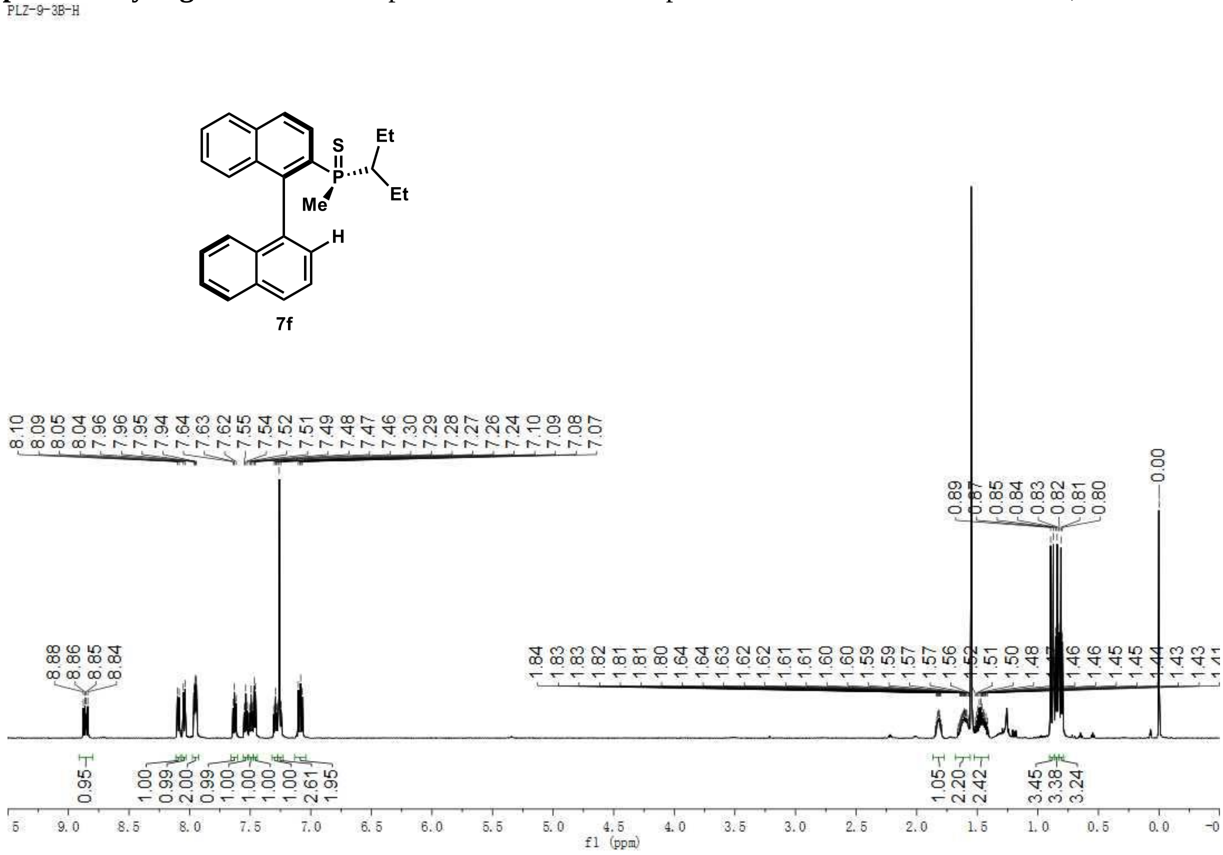

Supplementary Fig. 258. ^{1}H NMR spectrum of **7f**. The sample has been recorded in 600 MHz, $CDCl_3$ at 25 ℃.

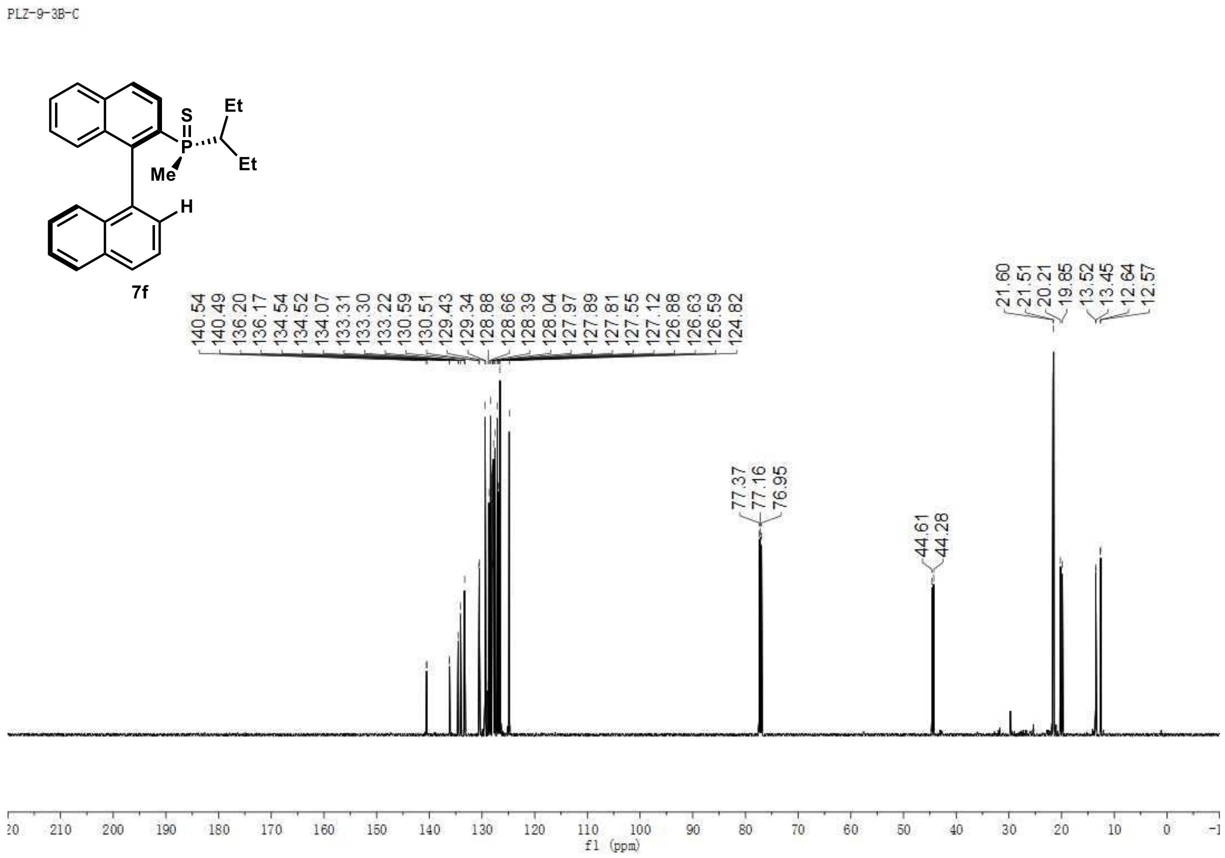

Supplementary Fig. 259.^{13}C NMR spectrum of **7f**. The sample has been recorded in 151 MHz, $CDCl_3$ at 25 ℃.

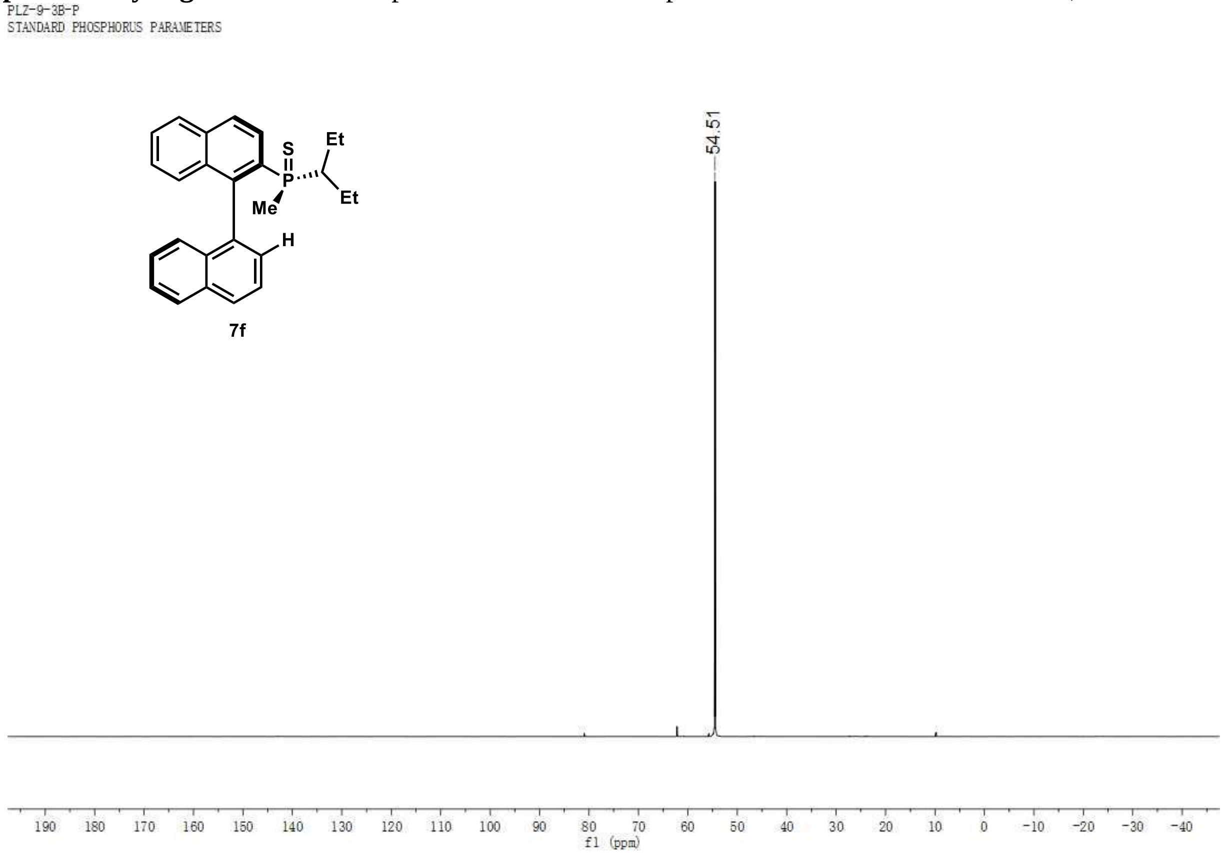

Supplementary Fig. 260.^{31}P NMR spectrum of **7f**. The sample has been recorded in 243 MHz, $CDCl_3$ at 25 ℃.

PLZ-9-3C-H

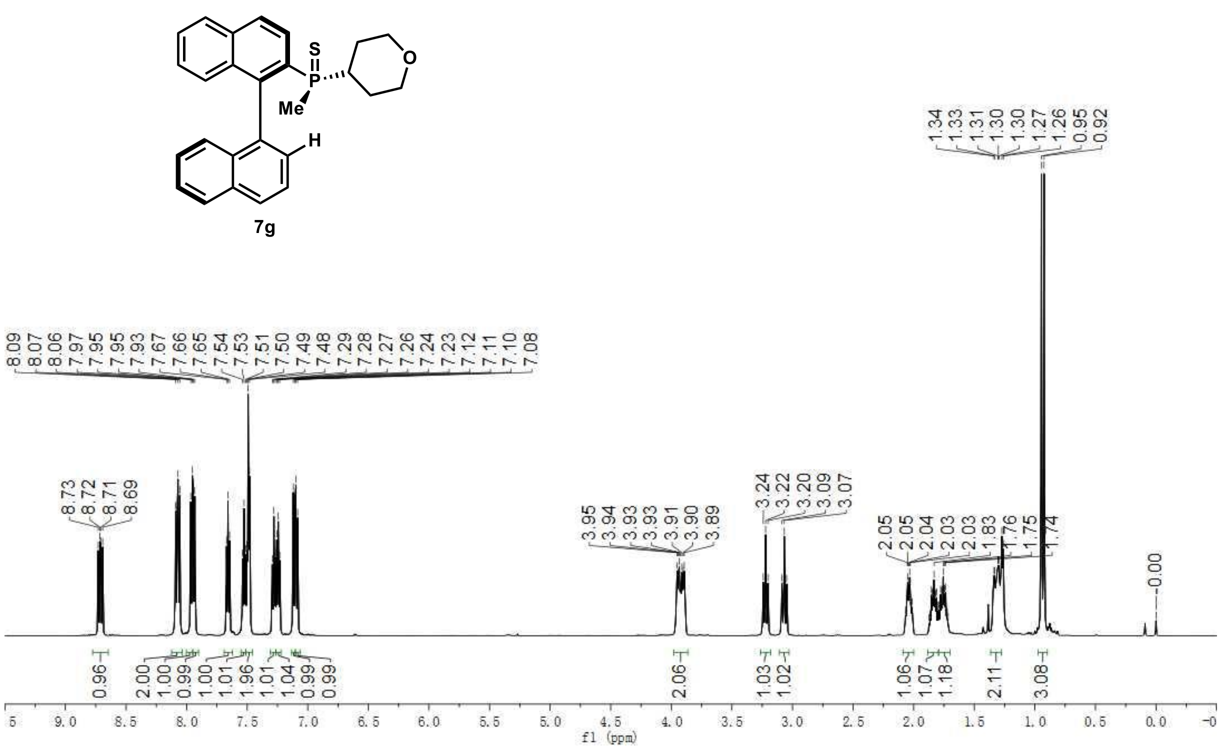

Supplementary Fig. 261. ^{1}H NMR spectrum of **7g**. The sample has been recorded in 600 MHz, $CDCl_3$ at 25 ℃.

PLZ-9-3C-C

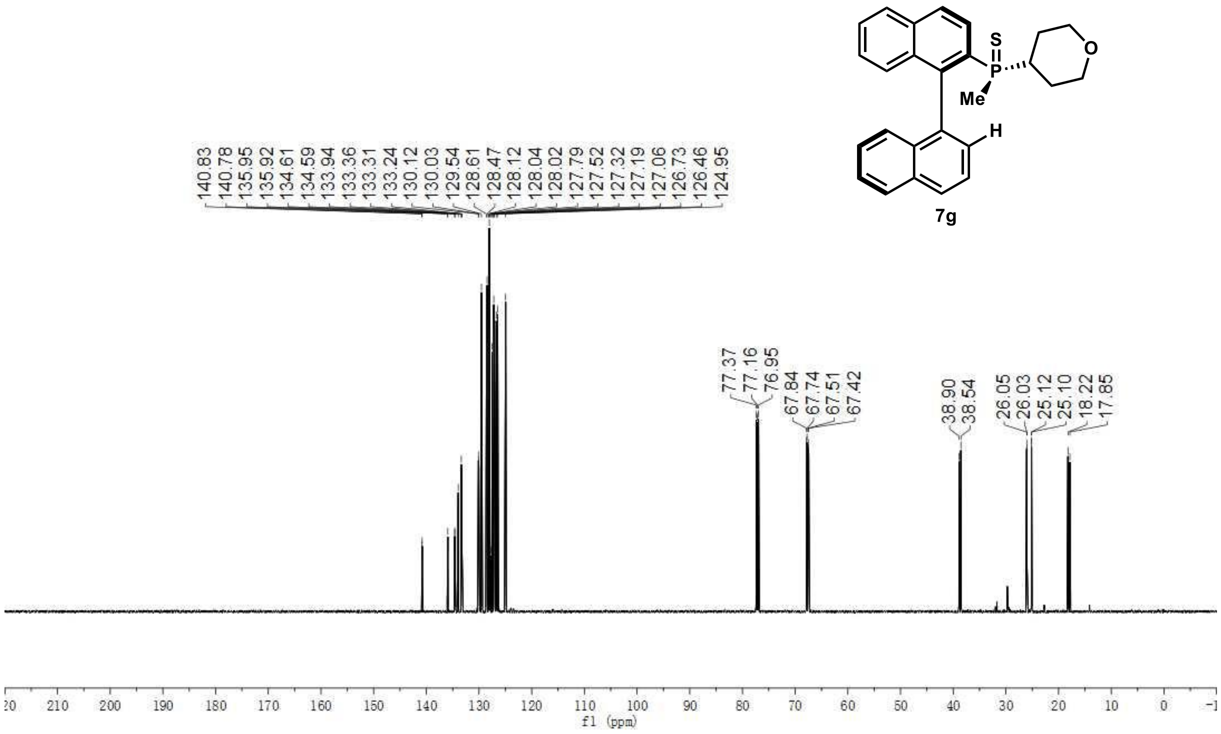

Supplementary Fig. 262.^{13}C NMR spectrum of **7g**. The sample has been recorded in 151 MHz, $CDCl_3$ at 25 ℃.

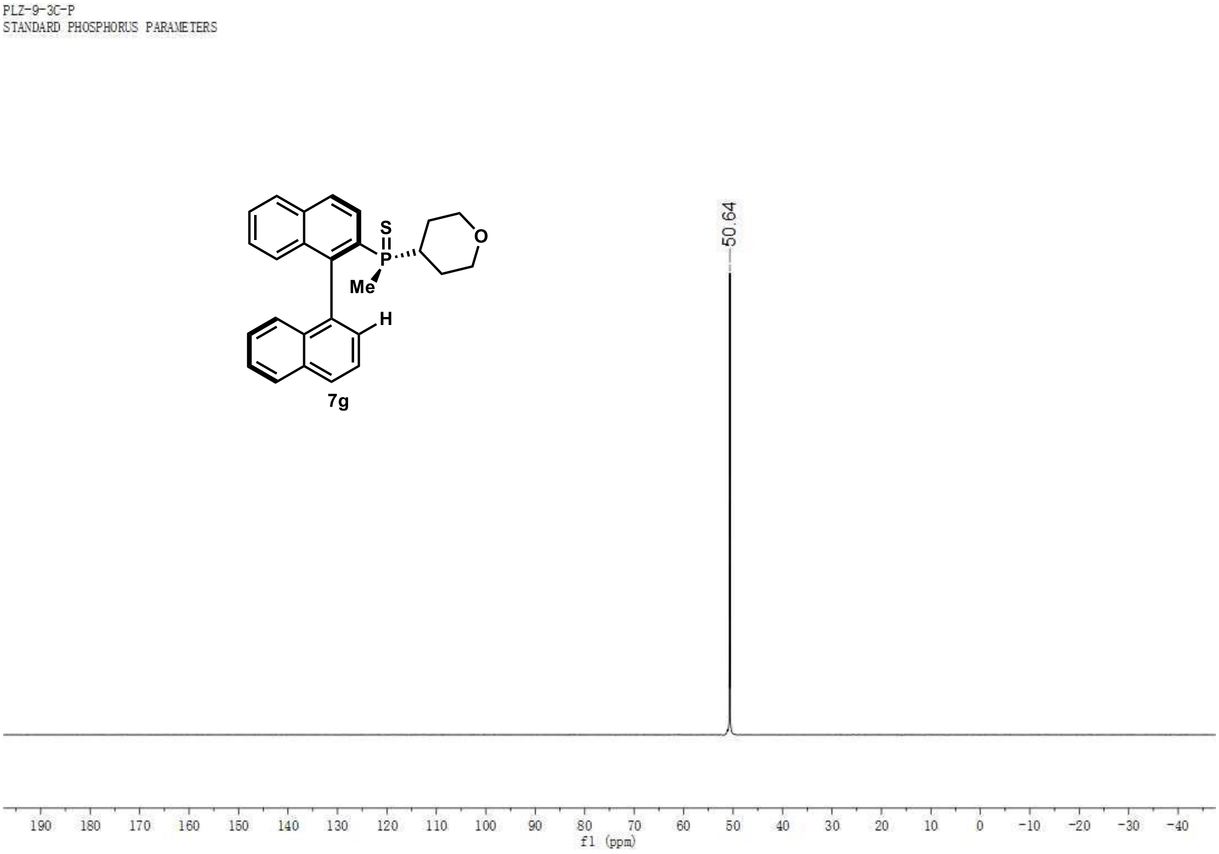

Supplementary Fig. 263. ^{31}P NMR spectrum of **7g**. The sample has been recorded in 243 MHz, $CDCl_3$ at 25 ℃.

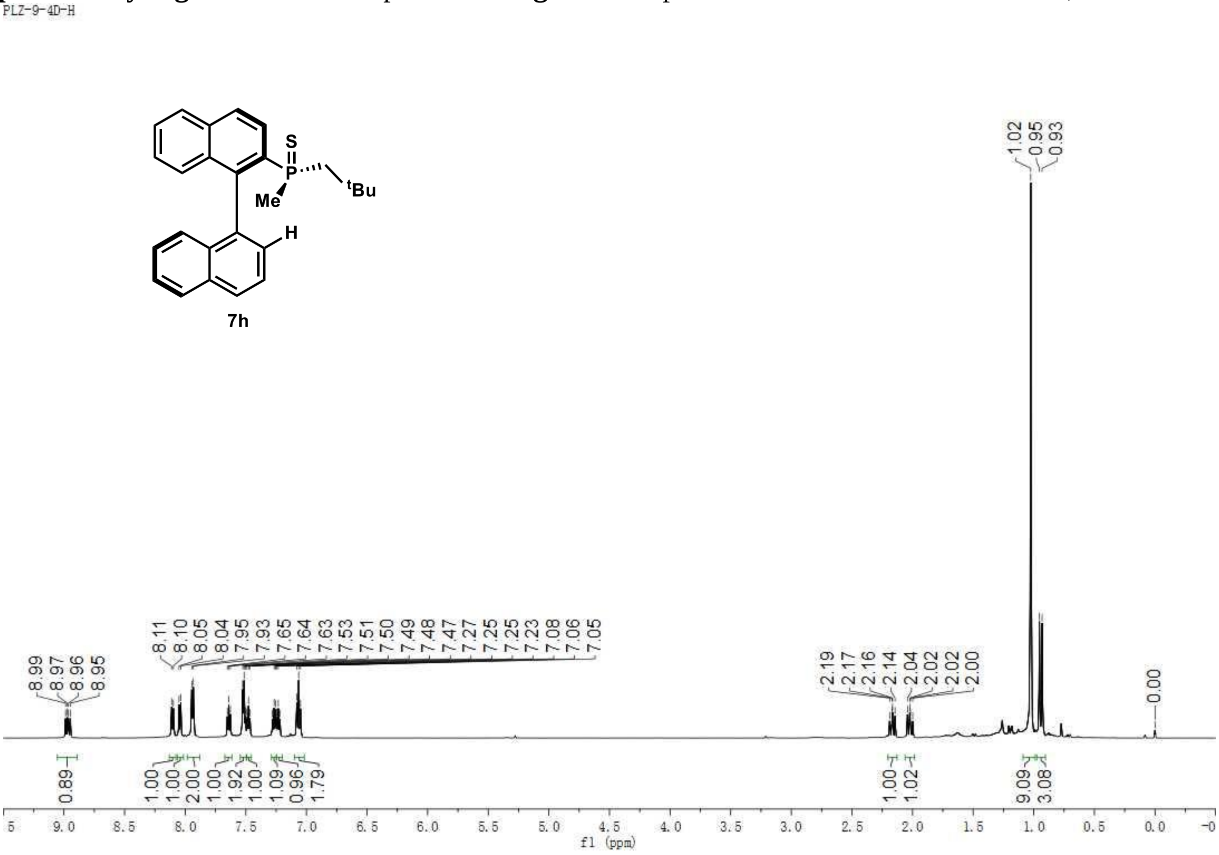

Supplementary Fig. 264. ^{1}H NMR spectrum of **7h**. The sample has been recorded in 600 MHz, $CDCl_3$ at 25 ℃.

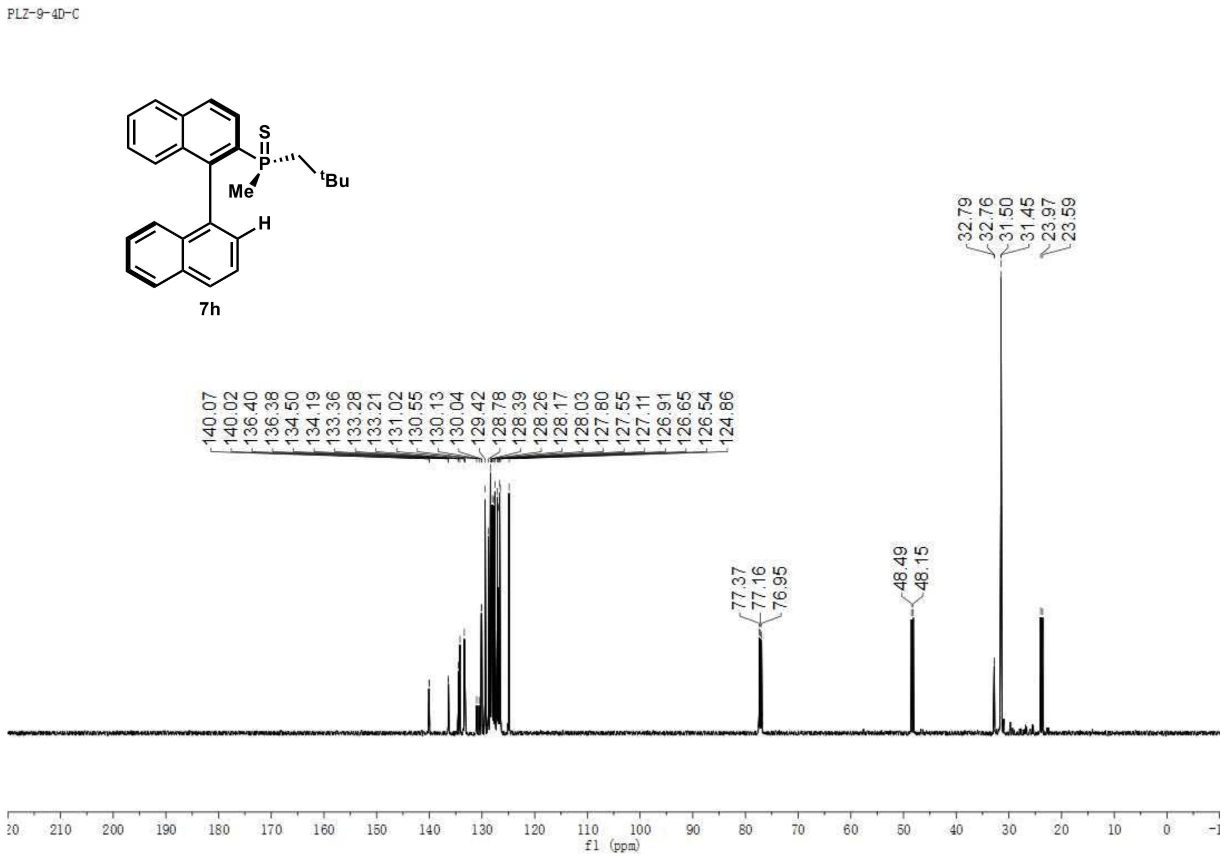

Supplementary Fig. 265.^{13}C NMR spectrum of **7h**. The sample has been recorded in 151 MHz, $CDCl_3$ at 25 ℃.

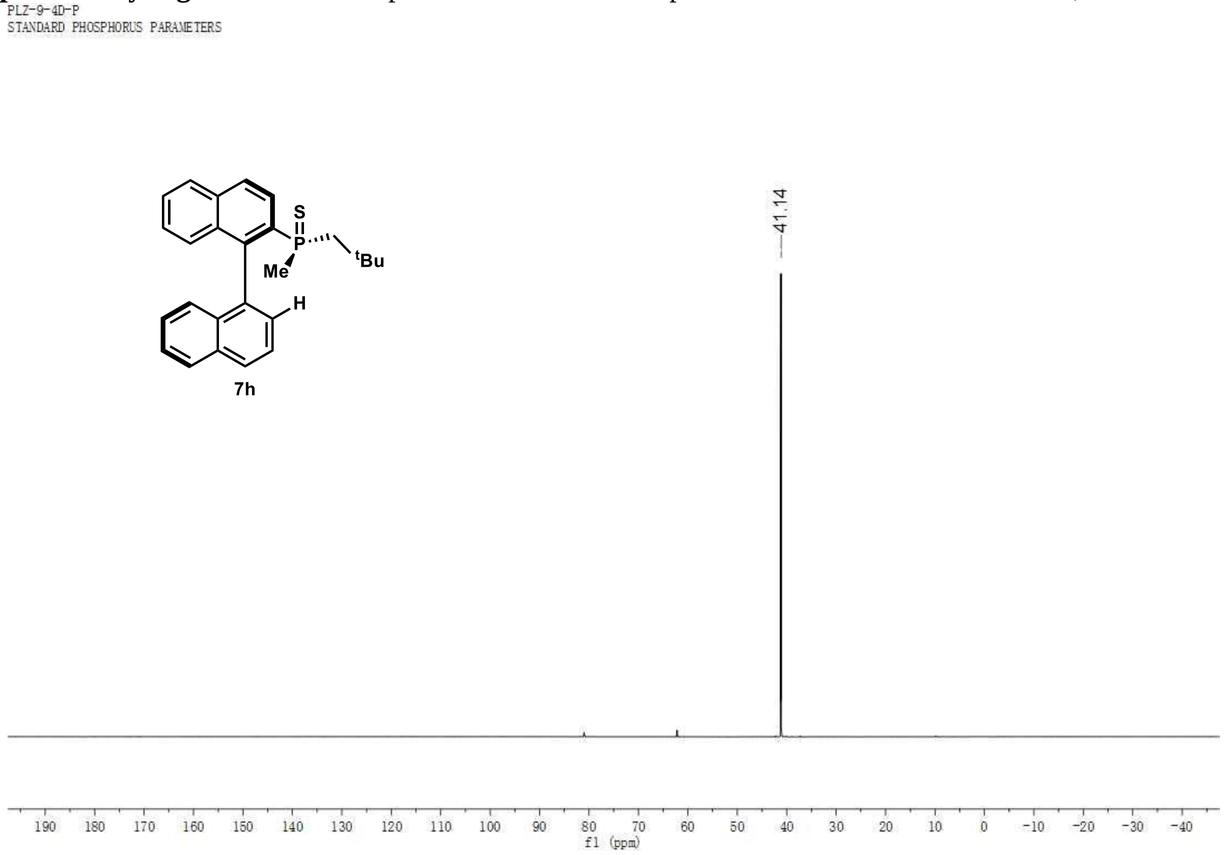

Supplementary Fig. 266.^{31}P NMR spectrum of **7h**. The sample has been recorded in 243 MHz, $CDCl_3$ at 25 ℃.

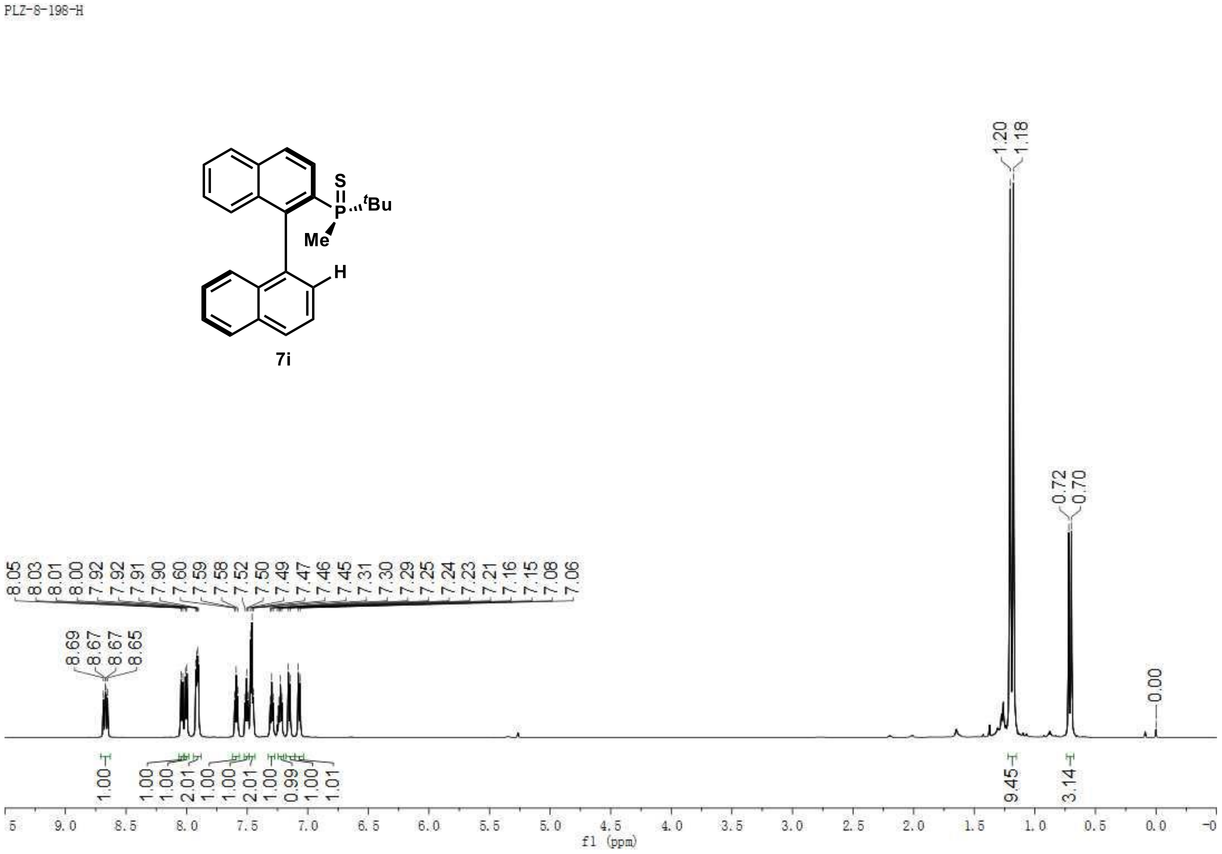

Supplementary Fig. 267. ^{1}H NMR spectrum of **7i**. The sample has been recorded in 600 MHz, $CDCl_3$ at 25 ℃.

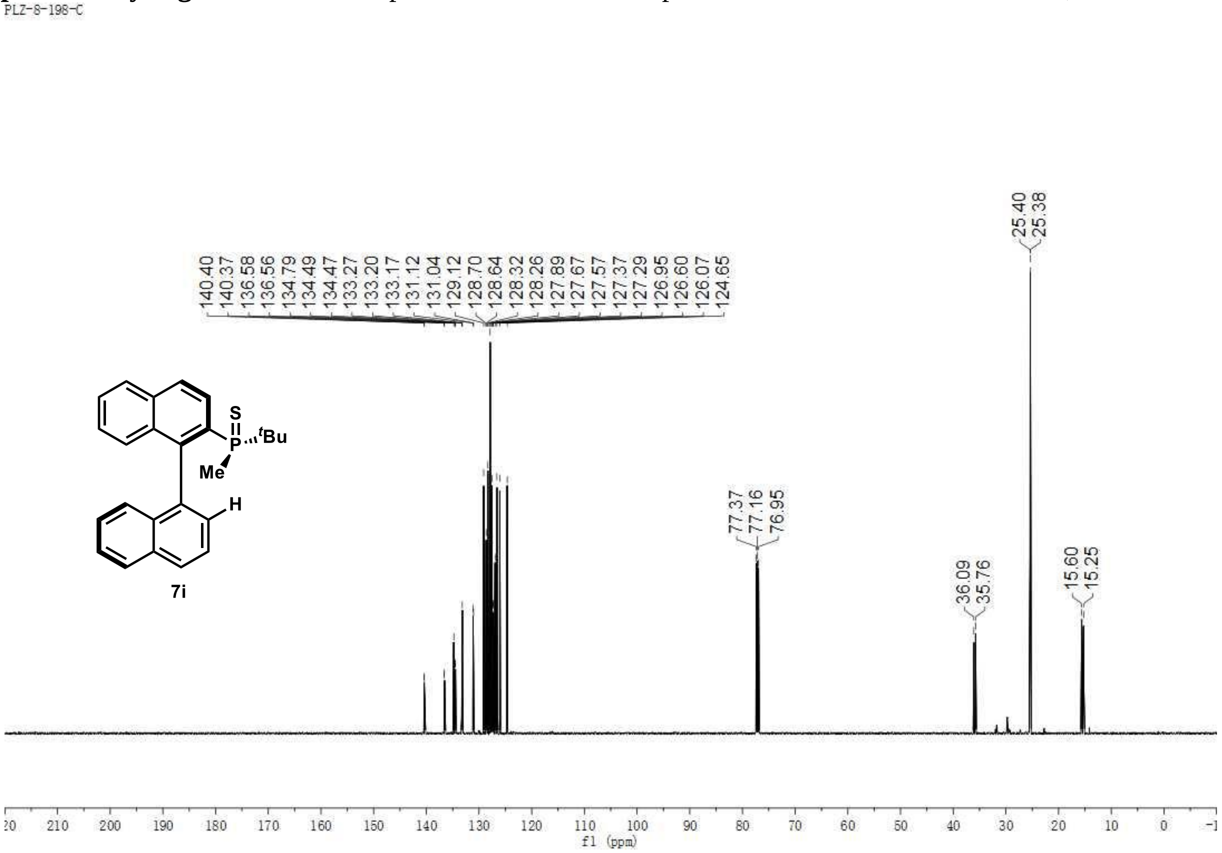

Supplementary Fig. 268. ^{13}C NMR spectrum of **7i.** The sample has been recorded in 151 MHz, $CDCl_3$ at 25 ℃.

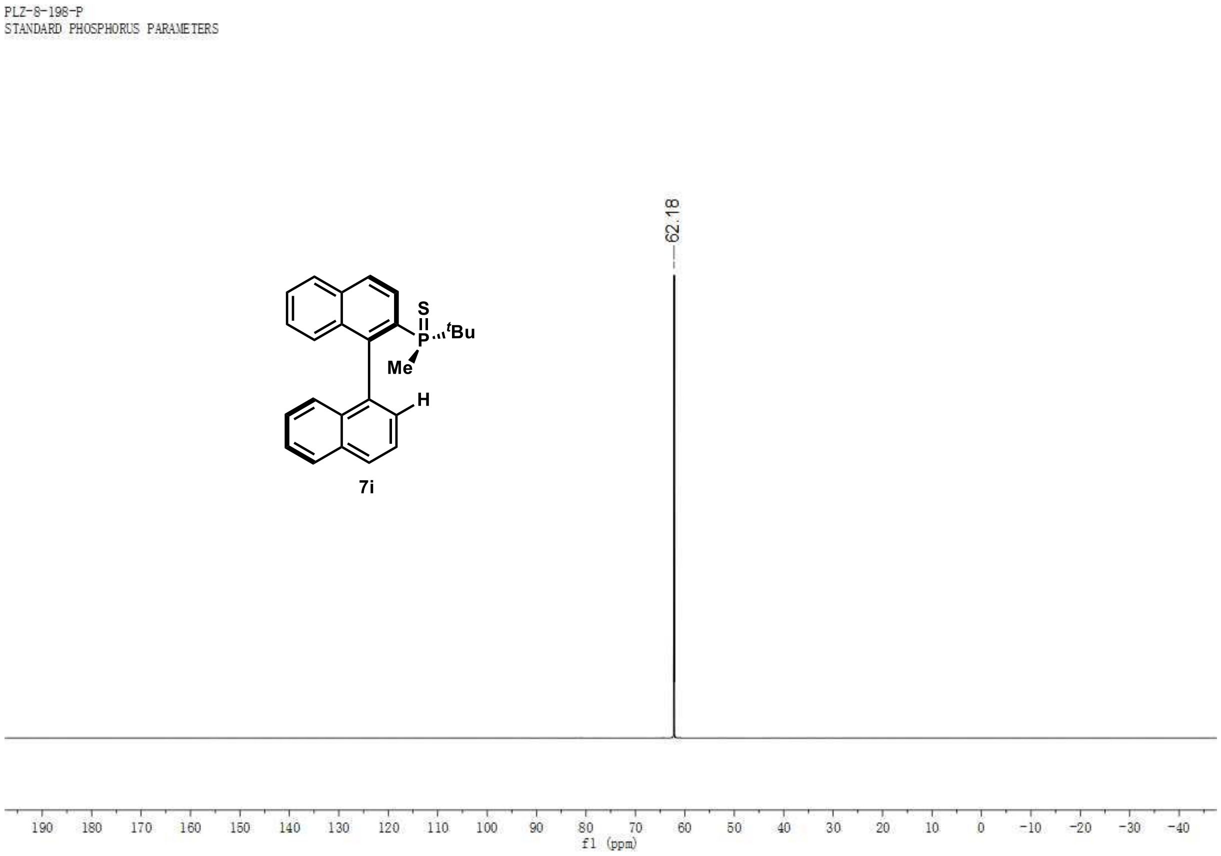

Supplementary Fig. 269.^{31}P NMR spectrum of **7i**. The sample has been recorded in 243 MHz, $CDCl_3$ at 25 ℃.

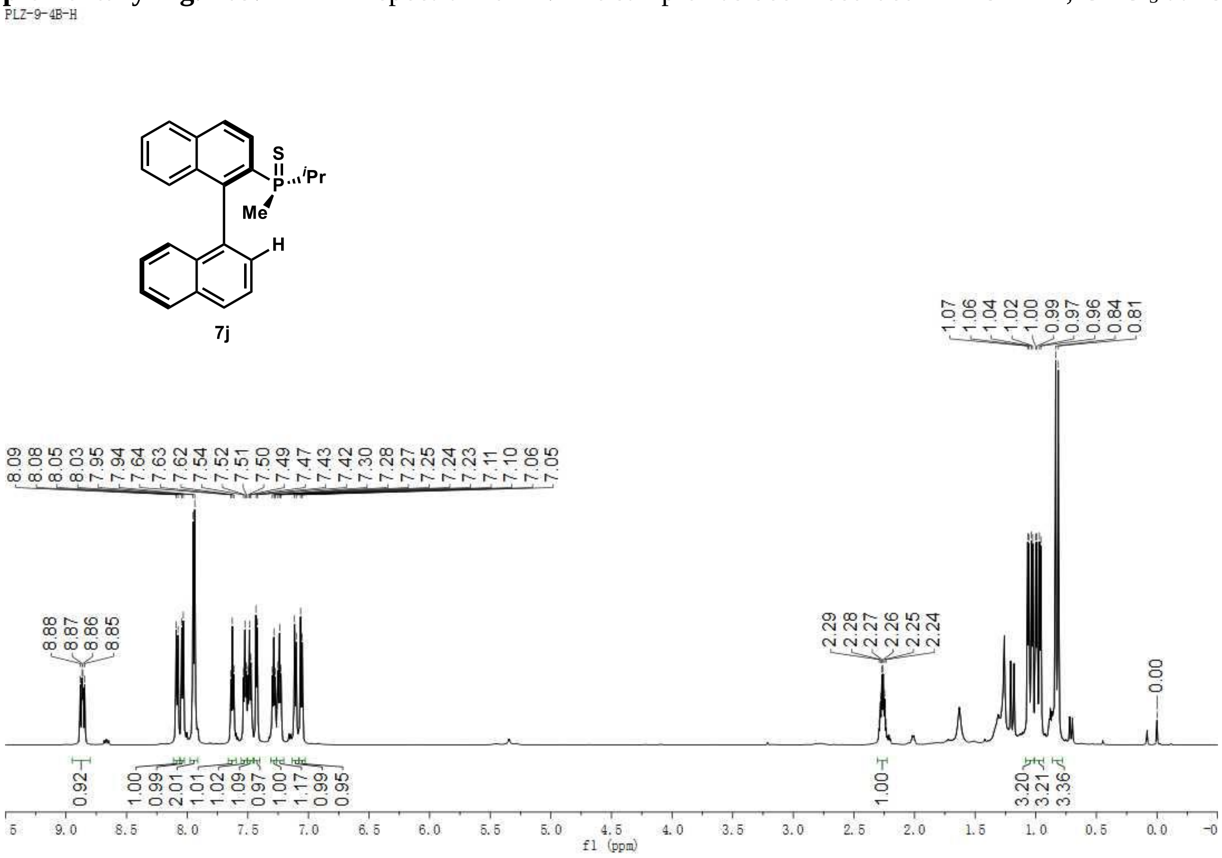

Supplementary Fig. 270. ^{1}H NMR spectrum of **7j**. The sample has been recorded in 600 MHz, $CDCl_3$ at 25 ℃.

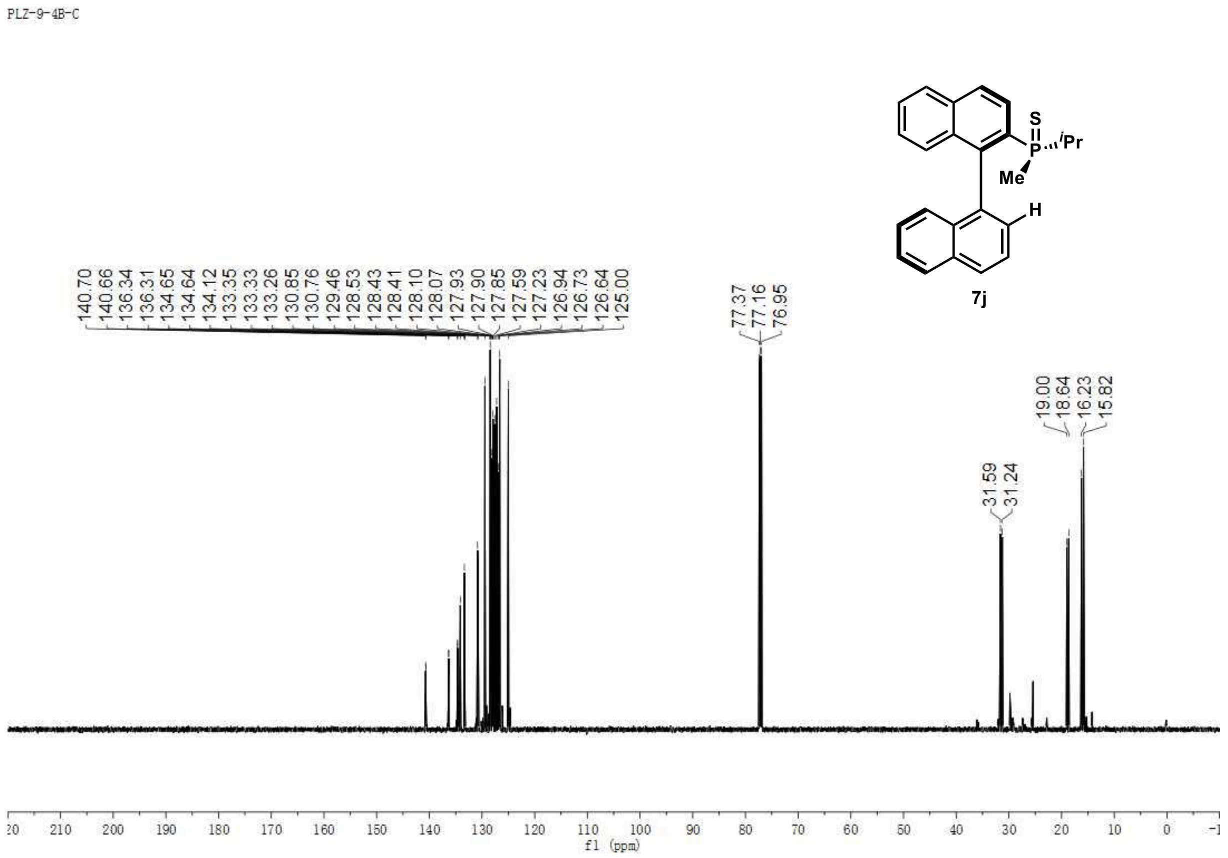

Supplementary Fig. 271.^{13}C NMR spectrum of **7j**. The sample has been recorded in 151 MHz, $CDCl_3$ at 25 ℃.

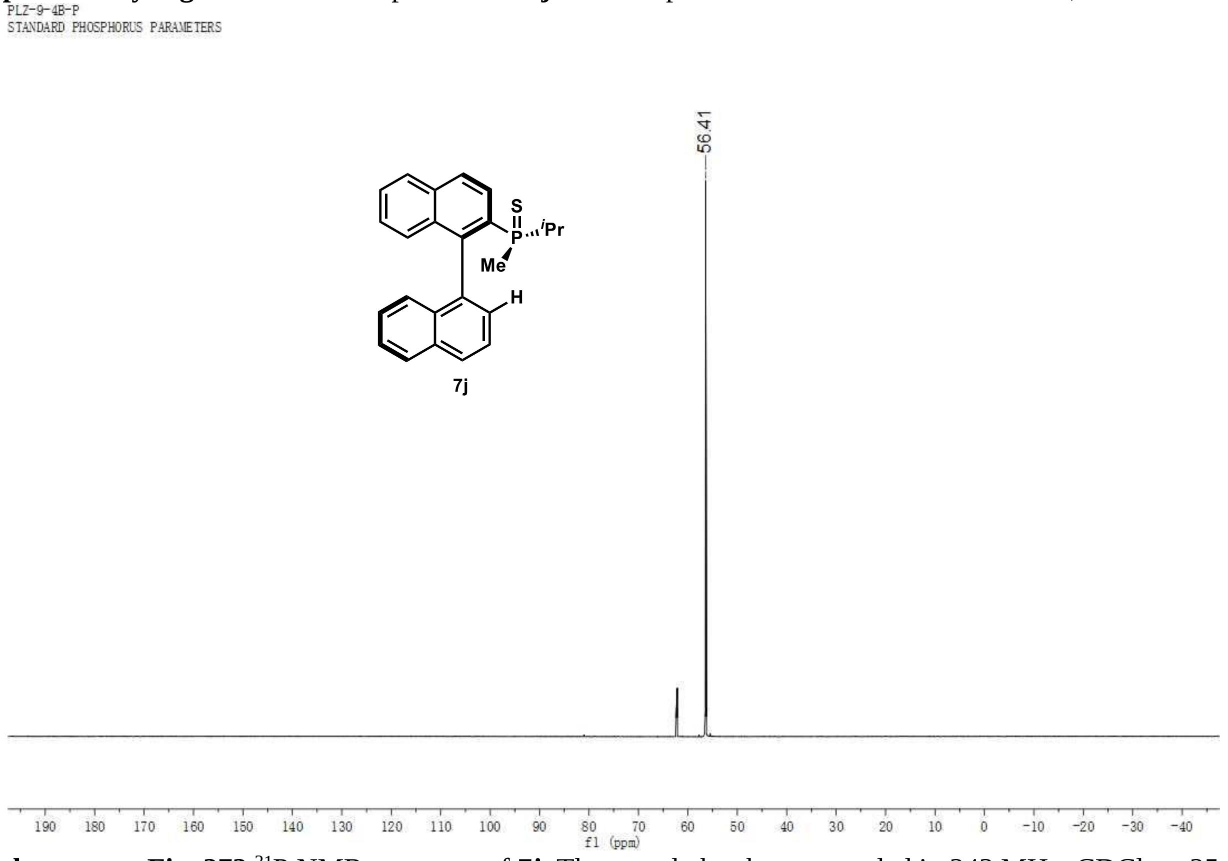

Supplementary Fig. 272.^{31}P NMR spectrum of **7j**. The sample has been recorded in 243 MHz, $CDCl_3$ at 25 ℃.

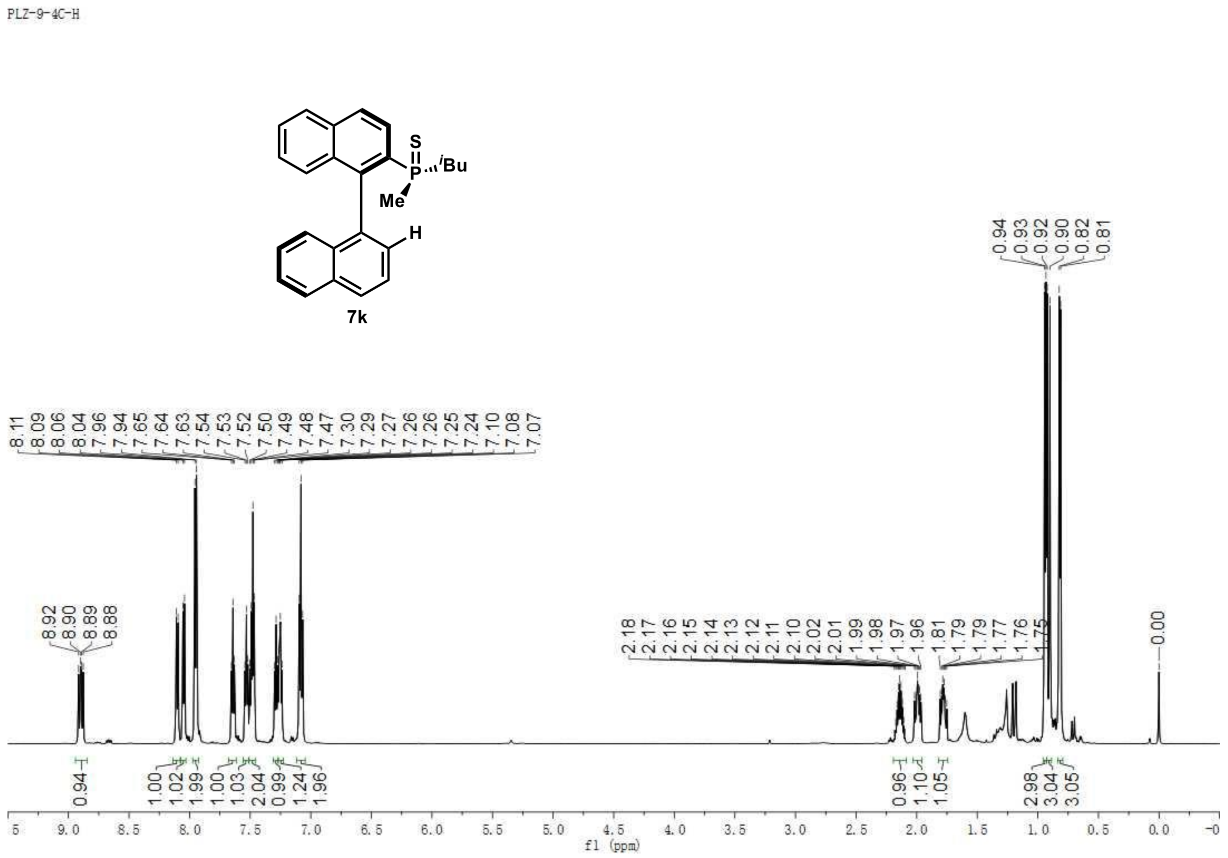

Supplementary Fig. 273. ^{1}H NMR spectrum of **7k**. The sample has been recorded in 600 MHz, $CDCl_3$ at 25 ℃.

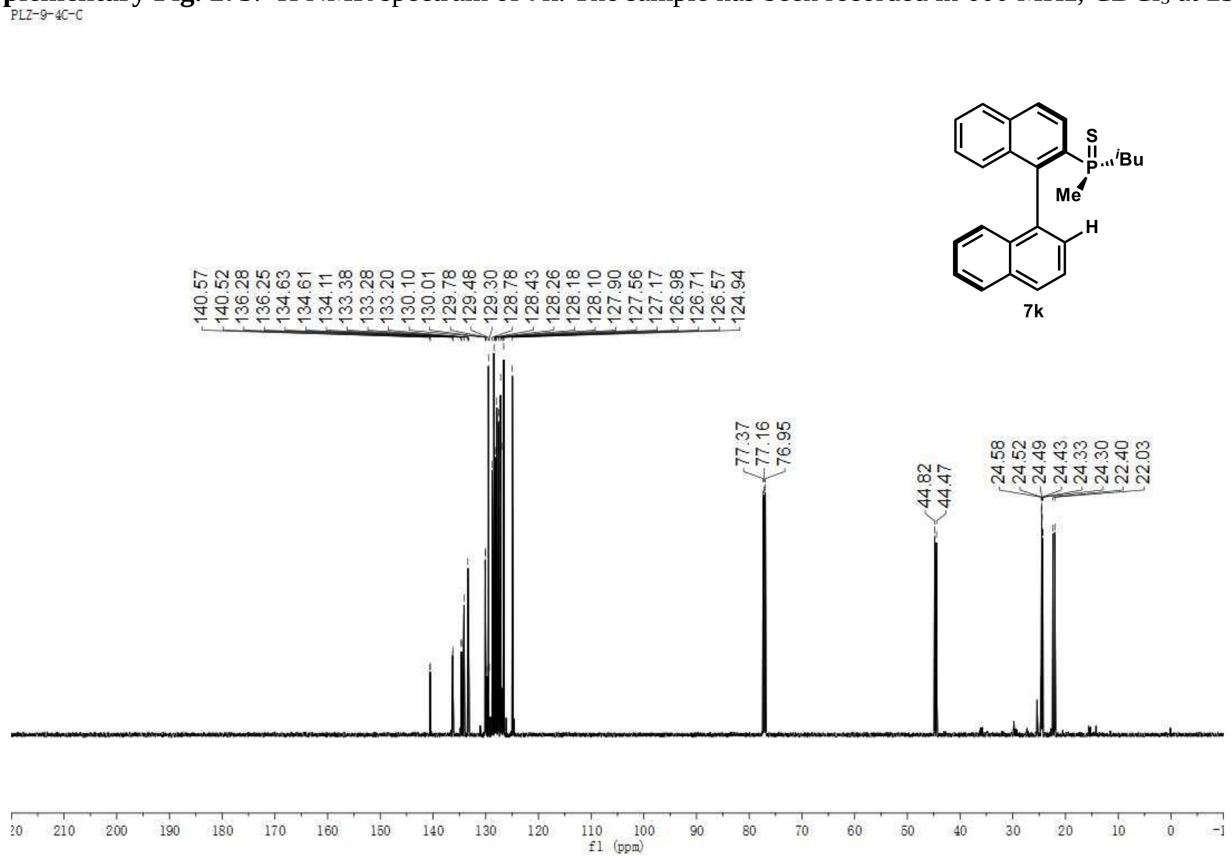

Supplementary Fig. 274. ^{13}C NMR spectrum of **7k**. The sample has been recorded in 151 MHz, $CDCl_3$ at 25 ℃.

PLZ-9-4C-P
STANDARD PHOSPHORUS PARAMETERS

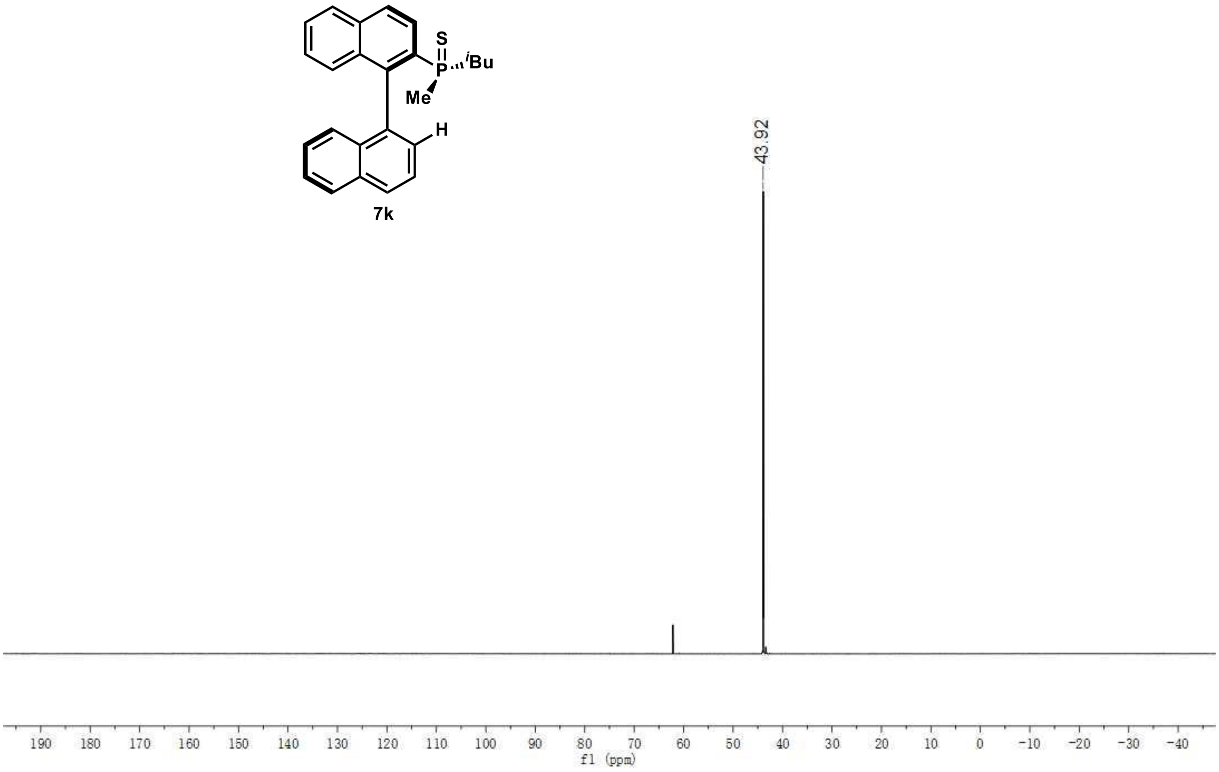

Supplementary Fig. 275.^{31}P NMR spectrum of **7k**. The sample has been recorded in 243 MHz, $CDCl_3$ at 25 ℃.

PLZ-9-37B-H

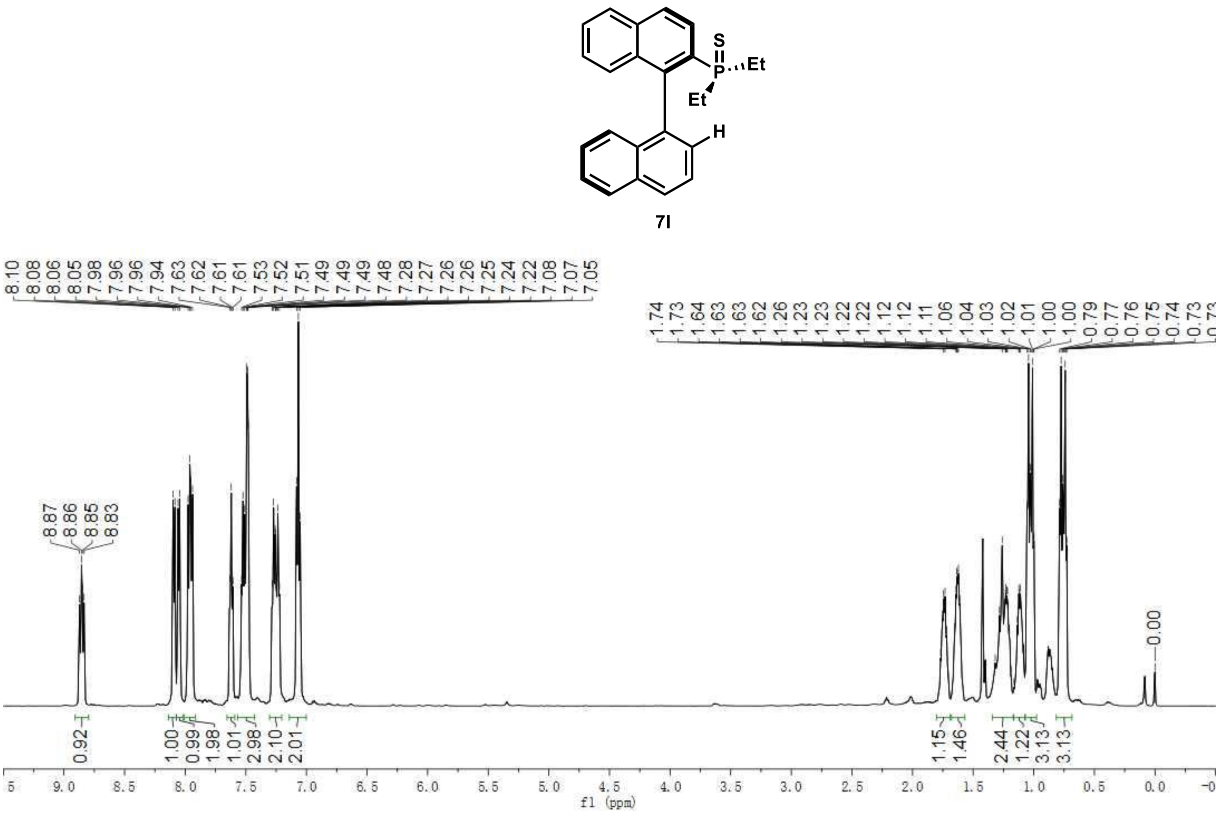

Supplementary Fig. 276. ^{1}H NMR spectrum of **7l**. The sample has been recorded in 600 MHz, $CDCl_3$ at 25 ℃.

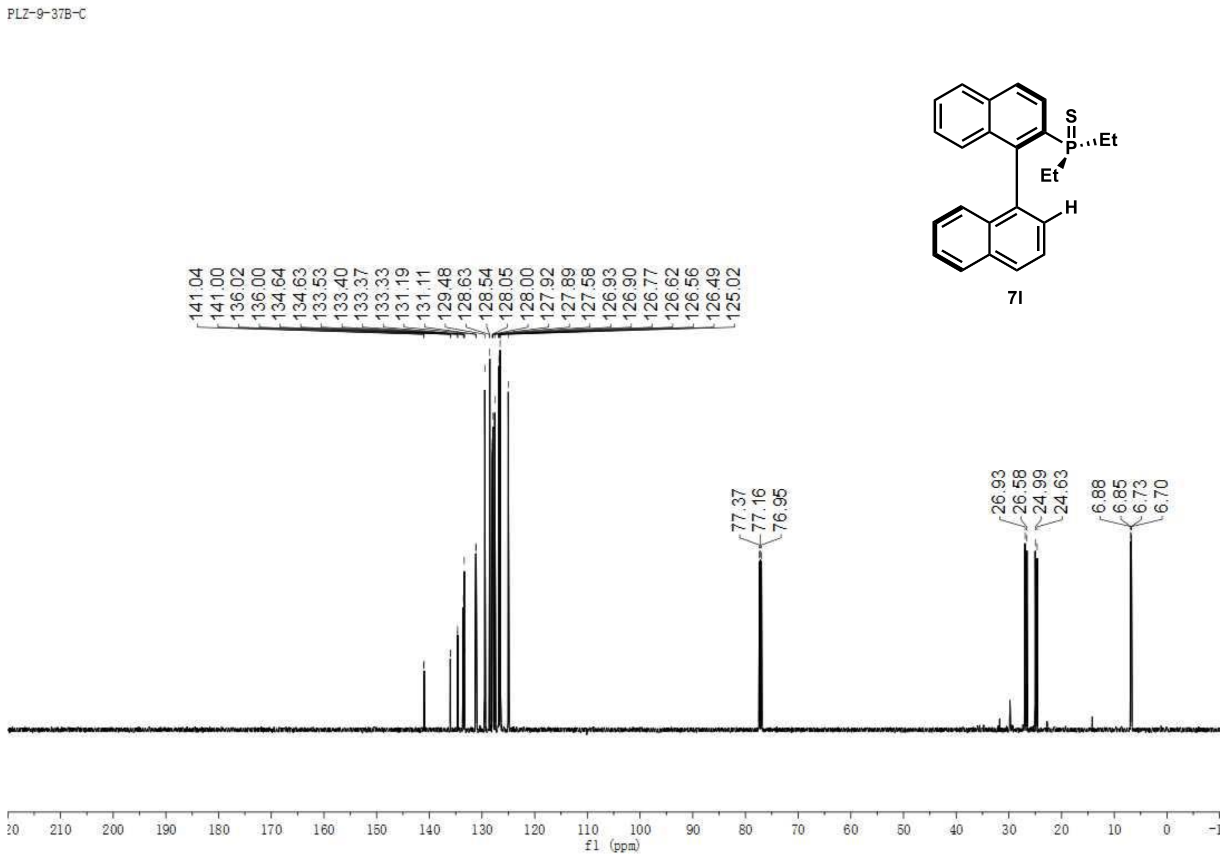

Supplementary Fig. 277.^{13}C NMR spectrum of **7l**. The sample has been recorded in 151 MHz, $CDCl_3$ at 25 ℃.

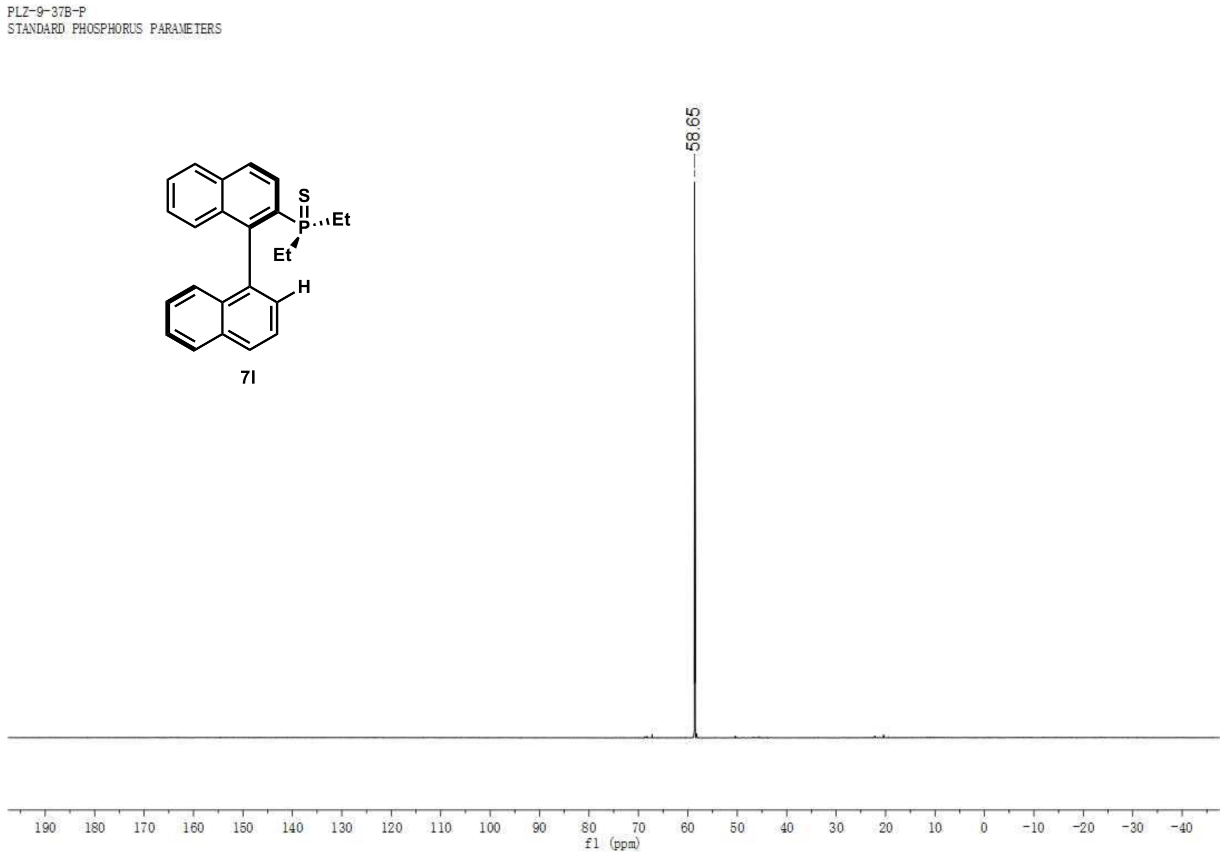

Supplementary Fig. 278.^{31}P NMR spectrum of **7l**. The sample has been recorded in 243 MHz, $CDCl_3$ at 25 ℃.

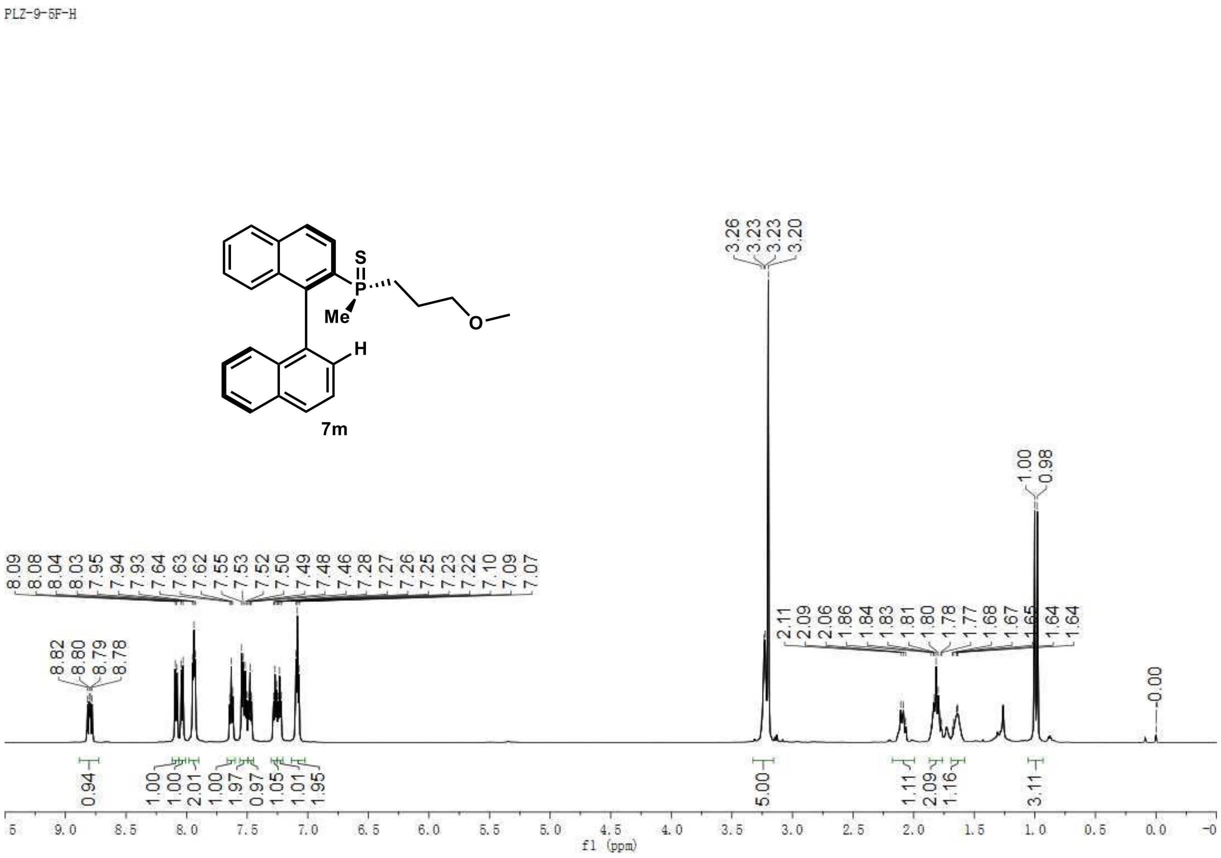

Supplementary Fig. 279. ^{1}H NMR spectrum of **7m**. The sample has been recorded in 600 MHz, $CDCl_3$ at 25 ℃.

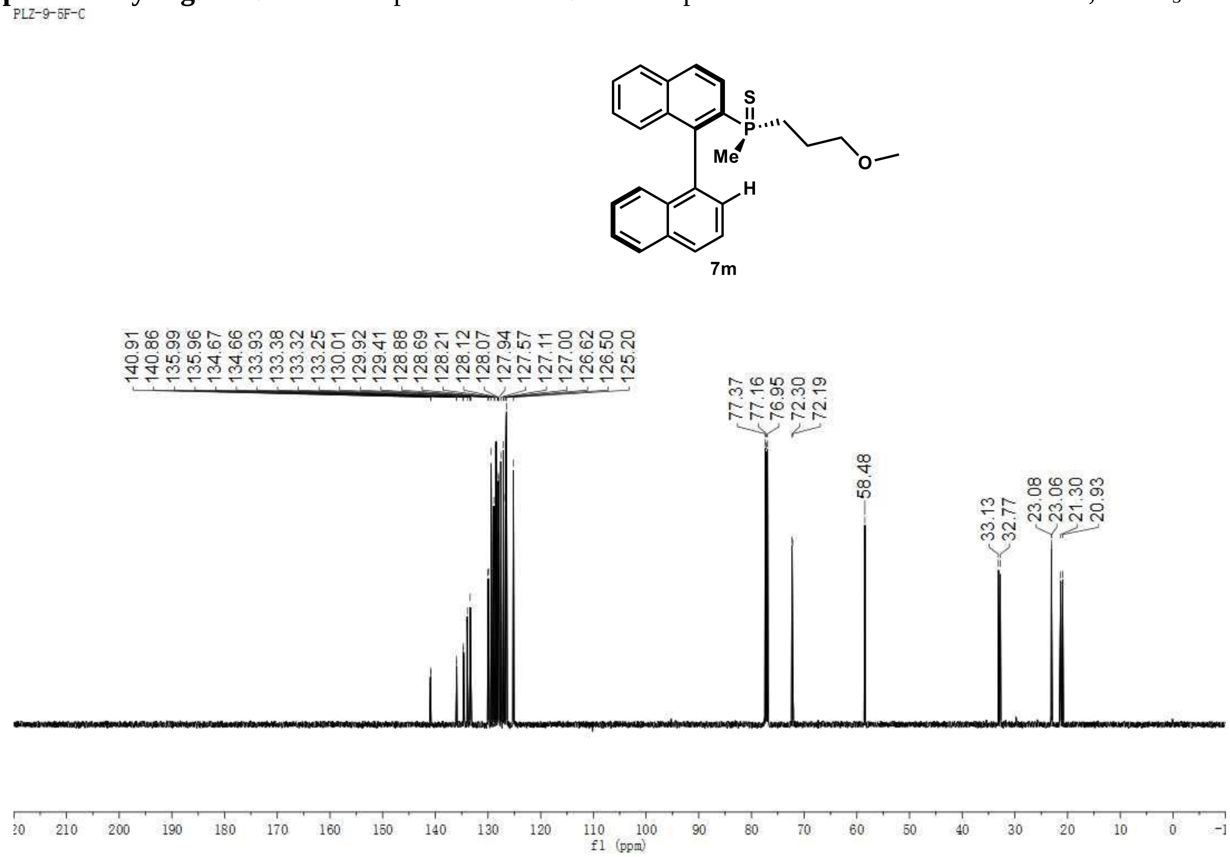

Supplementary Fig. 280.^{13}C NMR spectrum of **7m**. The sample has been recorded in 151 MHz, $CDCl_3$ at 25 ℃.

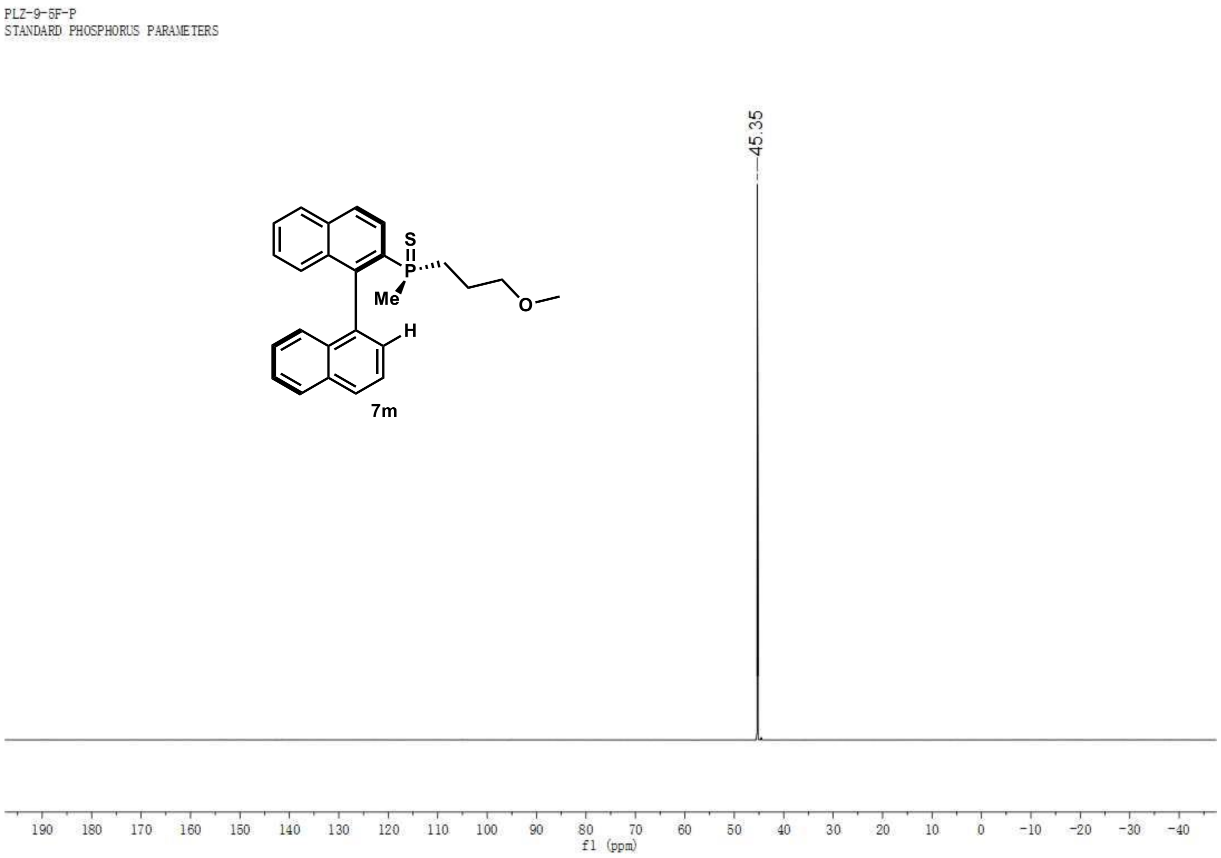

Supplementary Fig. 281.^{31}P NMR spectrum of **7m**. The sample has been recorded in 243 MHz, $CDCl_3$ at 25 ℃.

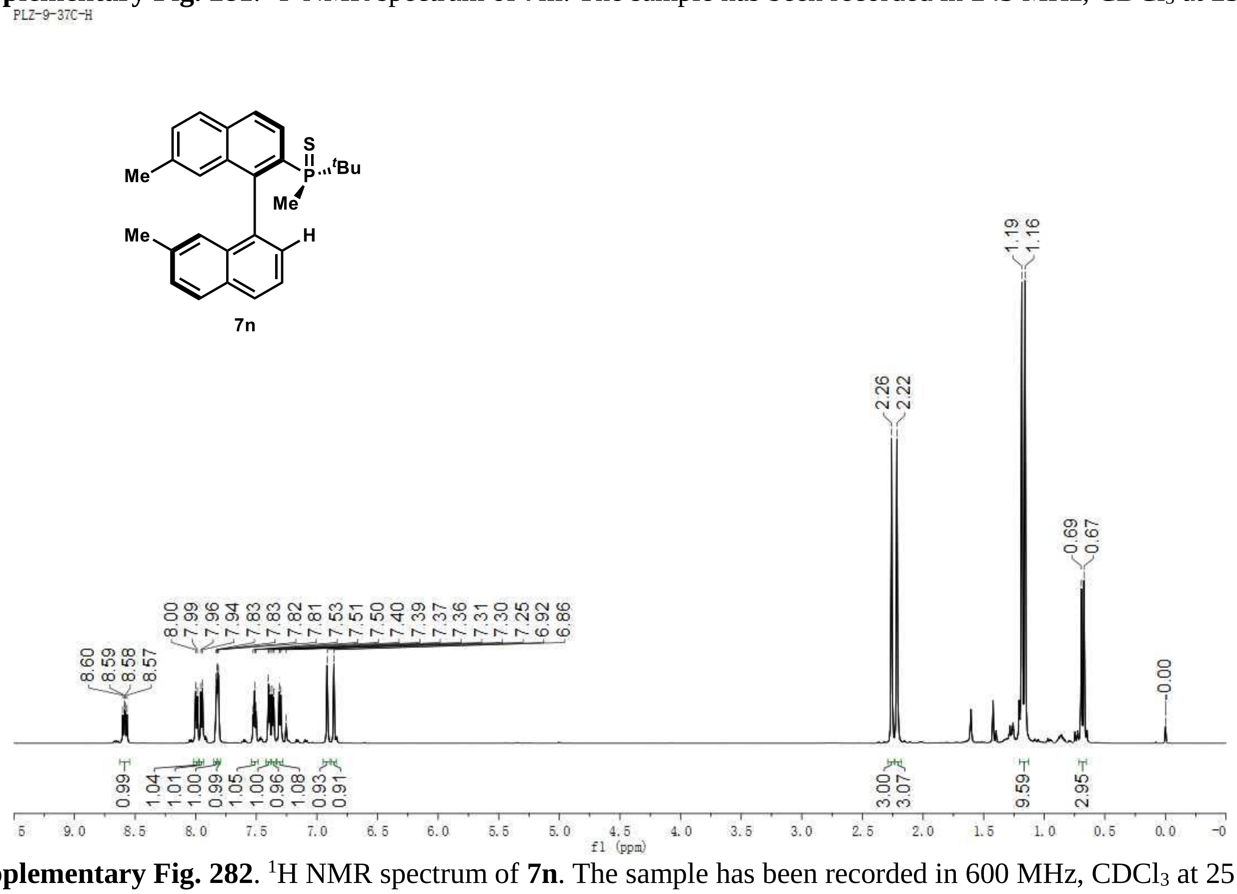

Supplementary Fig. 282. ^{1}H NMR spectrum of **7n**. The sample has been recorded in 600 MHz, $CDCl_3$ at 25 ℃.

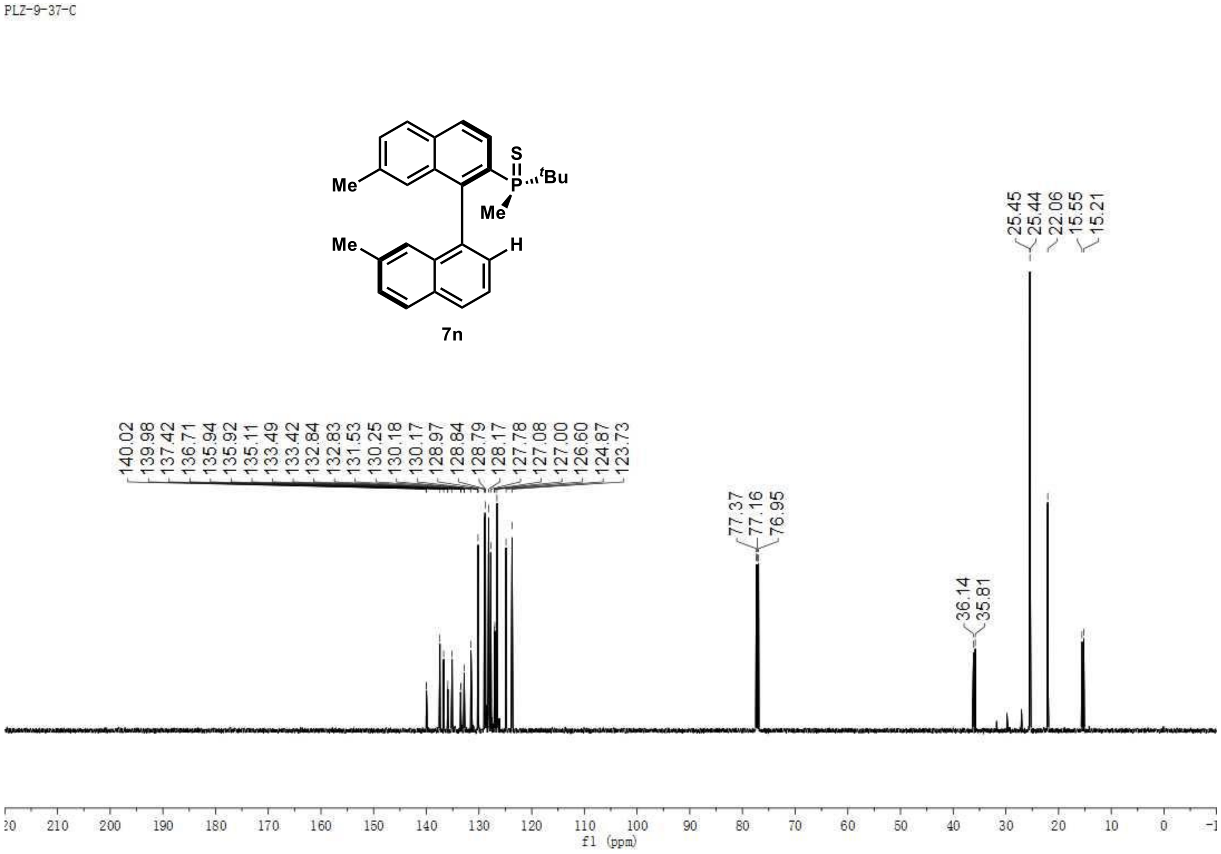

Supplementary Fig. 283.^{13}C NMR spectrum of **7n**. The sample has been recorded in 151 MHz, $CDCl_3$ at 25 ℃.

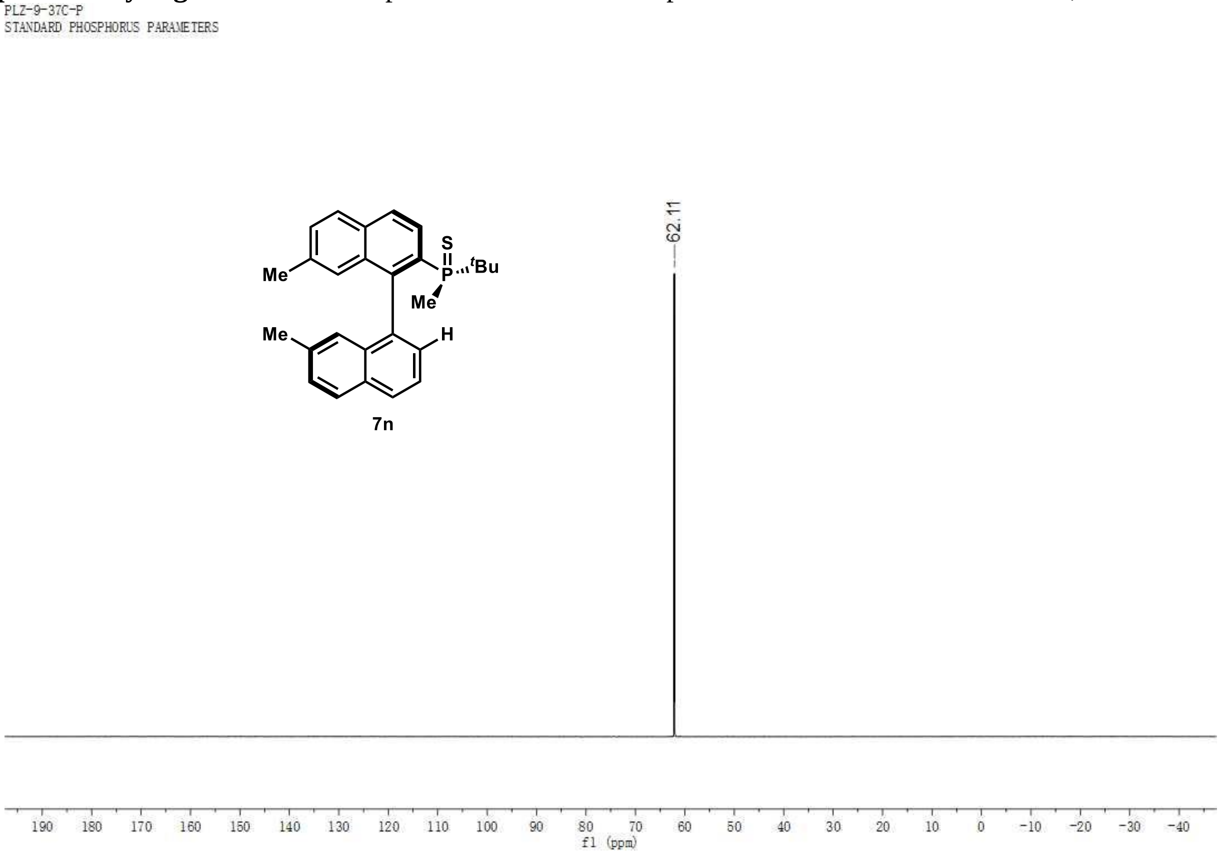

Supplementary Fig. 284.^{31}P NMR spectrum of **7n**. The sample has been recorded in 243 MHz, $CDCl_3$ at 25 ℃.

PLZ-9-37D-H

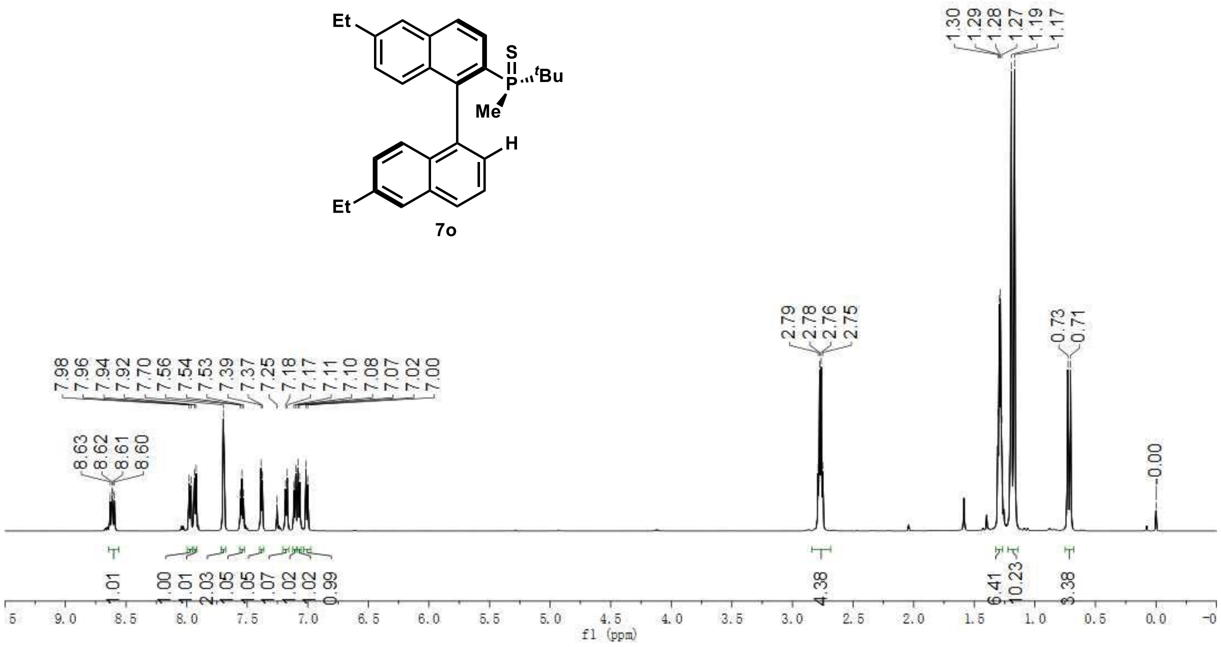

Supplementary Fig. 285. ^{1}H NMR spectrum of **7o**. The sample has been recorded in 600 MHz, $CDCl_3$ at 25 ℃.

PLZ-9-37D-C

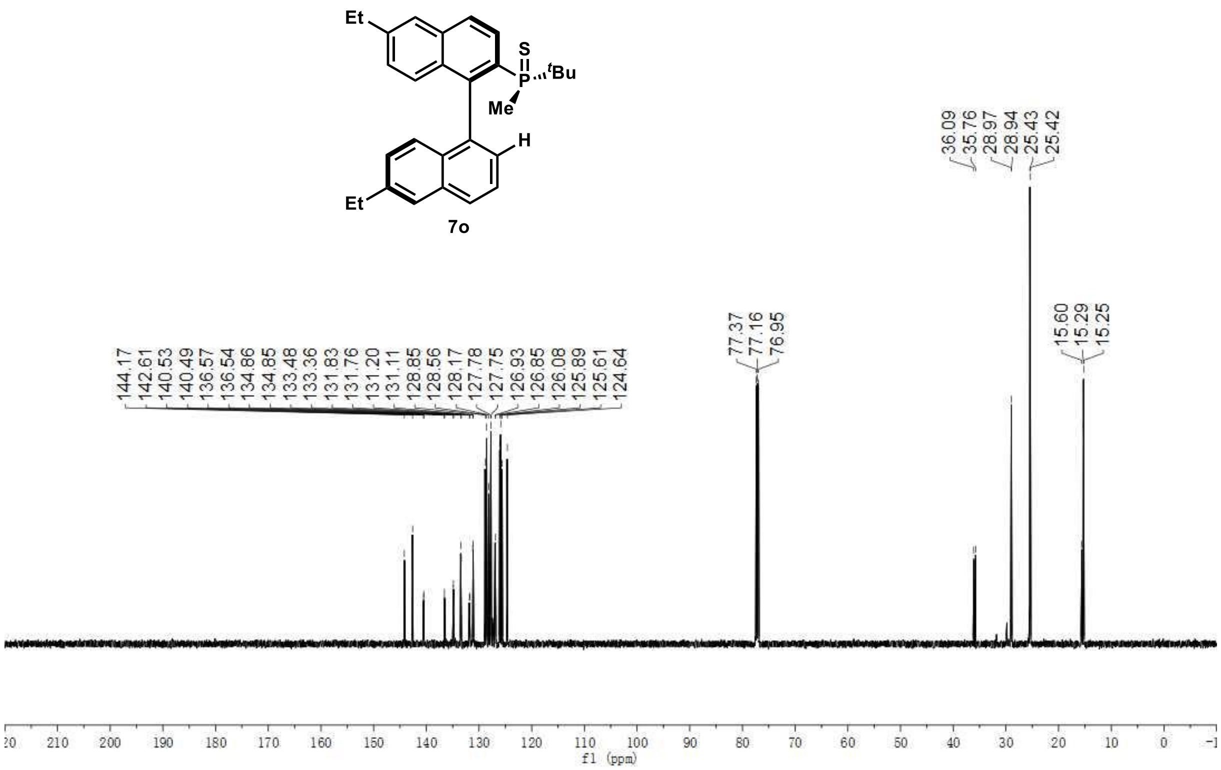

Supplementary Fig. 286.^{13}C NMR spectrum of **7o**. The sample has been recorded in 151 MHz, $CDCl_3$ at 25 ℃.

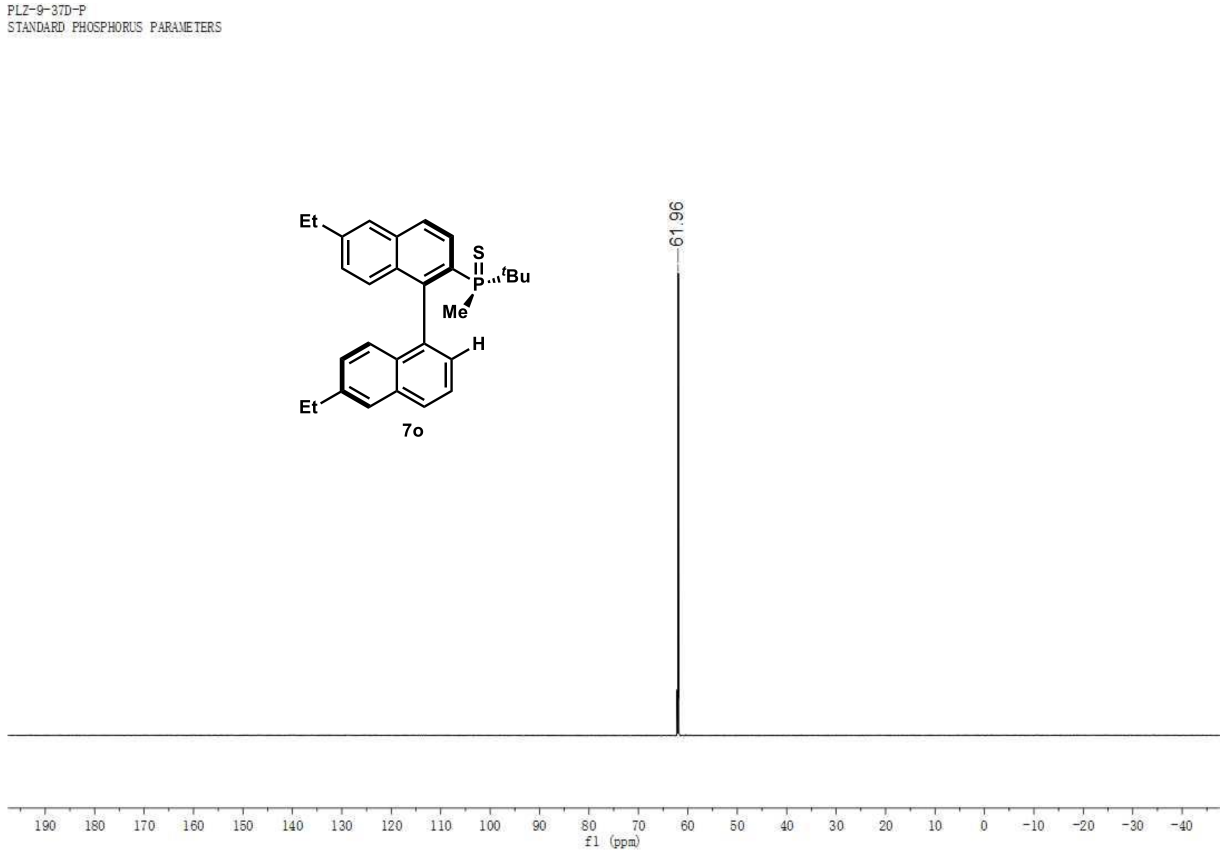

Supplementary Fig. 287.^{31}P NMR spectrum of **7o**. The sample has been recorded in 243 MHz, $CDCl_3$ at 25 ℃.

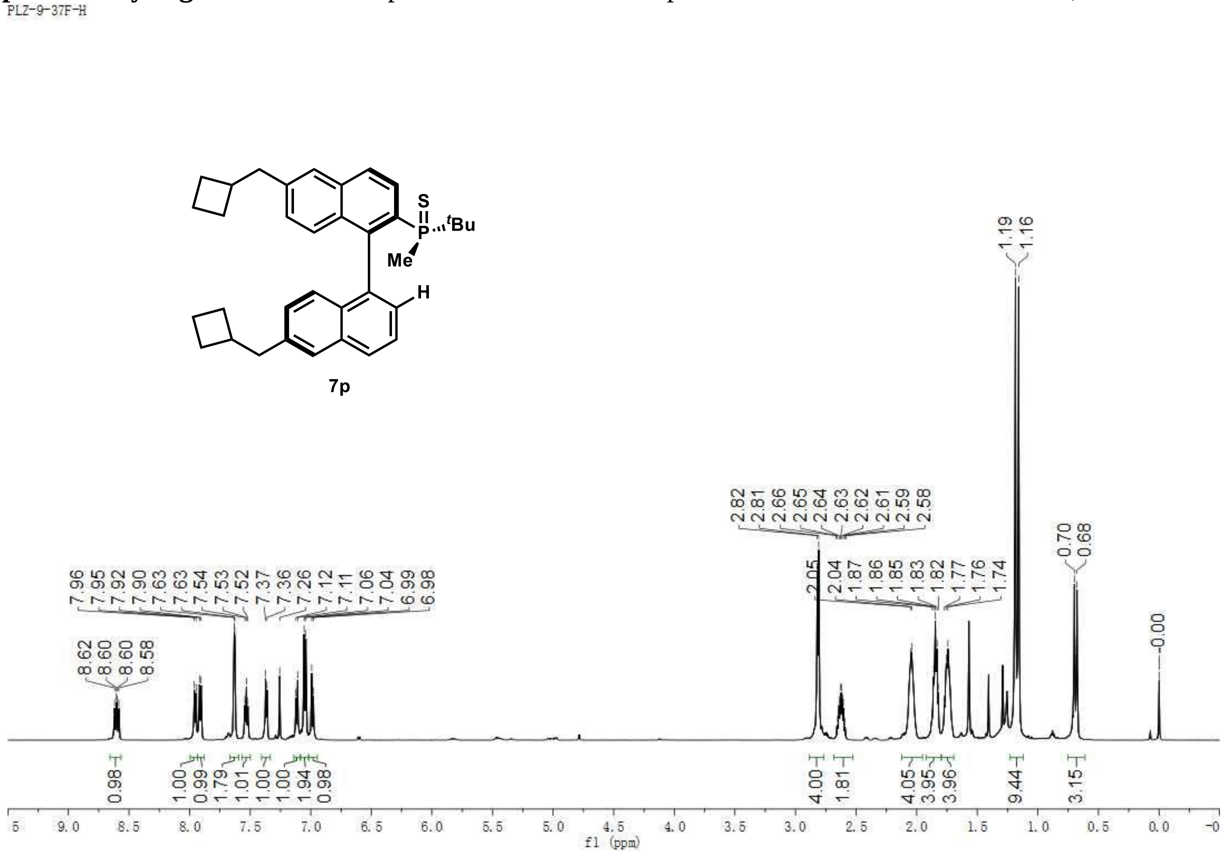

Supplementary Fig. 288. ^{1}H NMR spectrum of **7p**. The sample has been recorded in 600 MHz, $CDCl_3$ at 25 ℃.

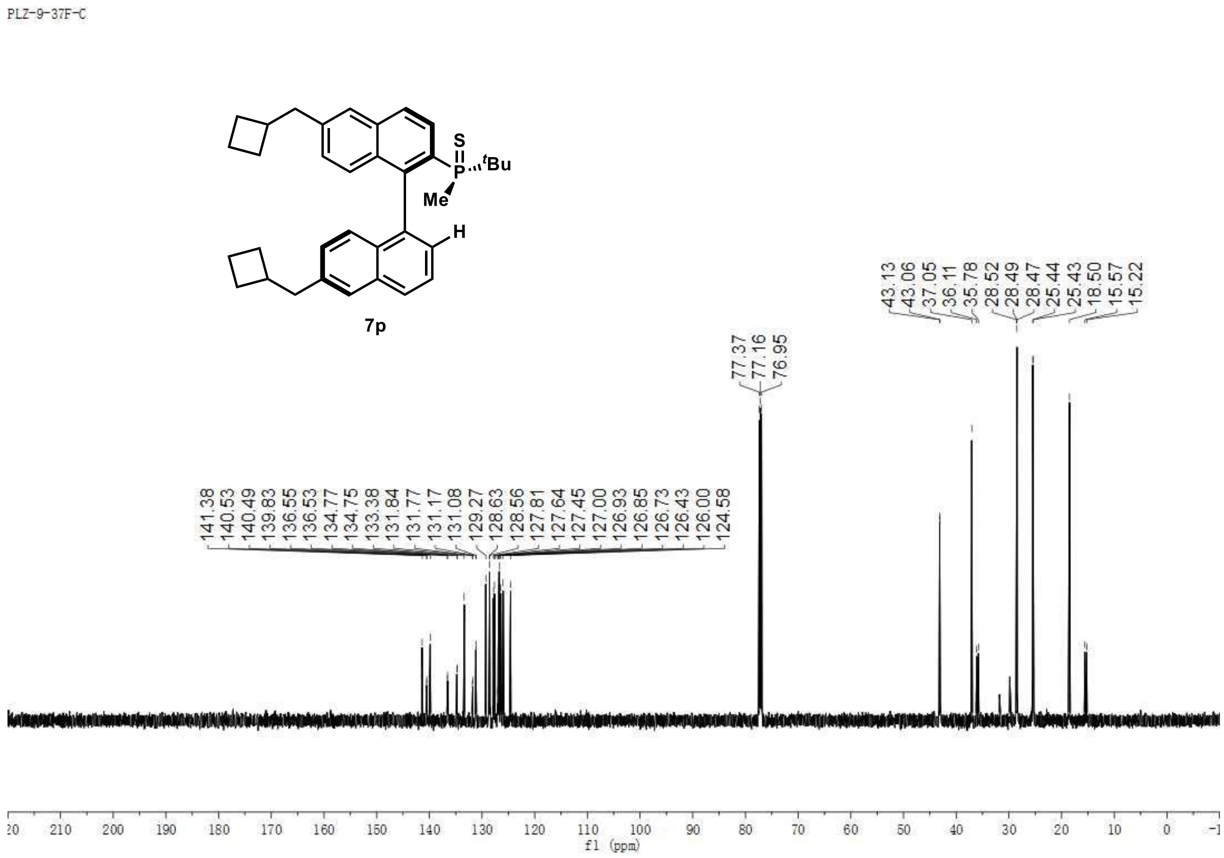

Supplementary Fig. 289. ^{13}C NMR spectrum of **7p**. The sample has been recorded in 151 MHz, $CDCl_3$ at 25 ℃.

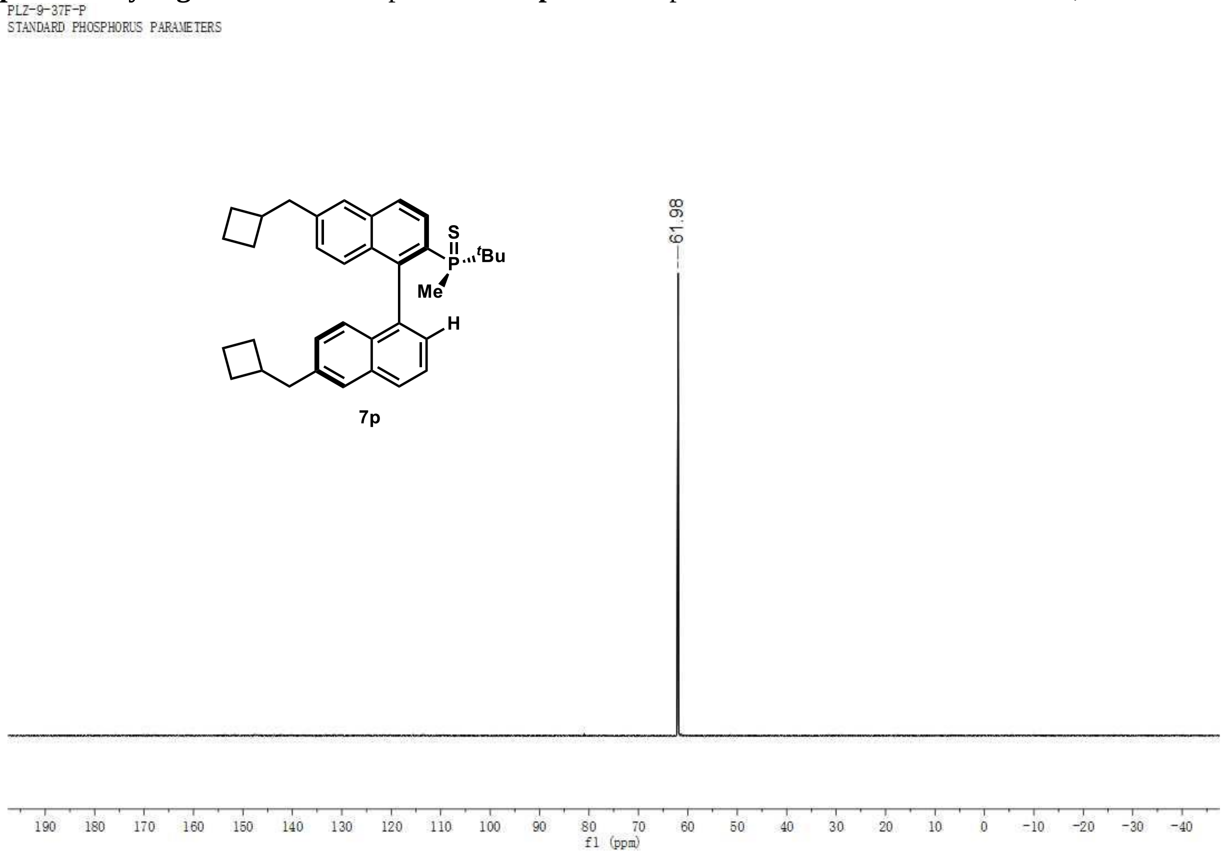

Supplementary Fig. 290. ^{31}P NMR spectrum of **7p**. The sample has been recorded in 243 MHz, $CDCl_3$ at 25 ℃.

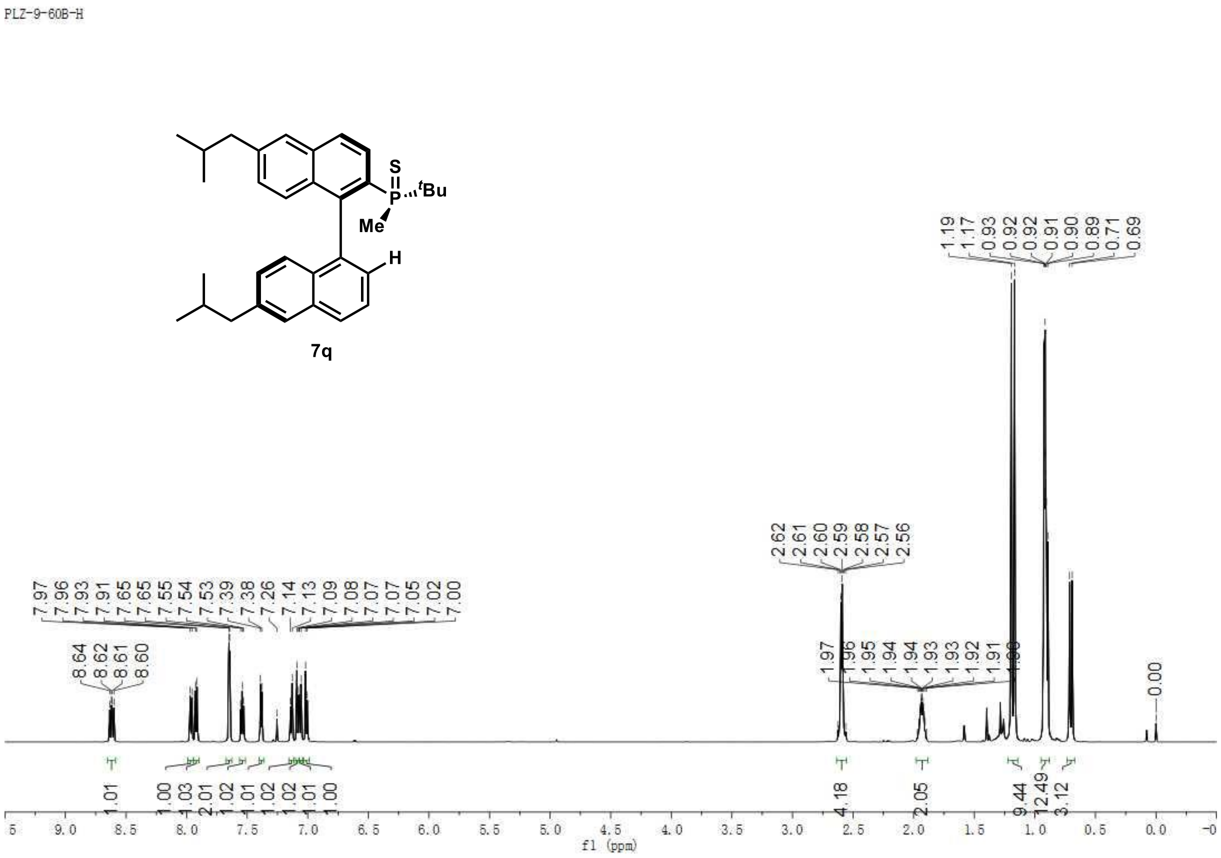

Supplementary Fig. 291. ^{1}H NMR spectrum of **7q**. The sample has been recorded in 600 MHz, $CDCl_3$ at 25 ℃.

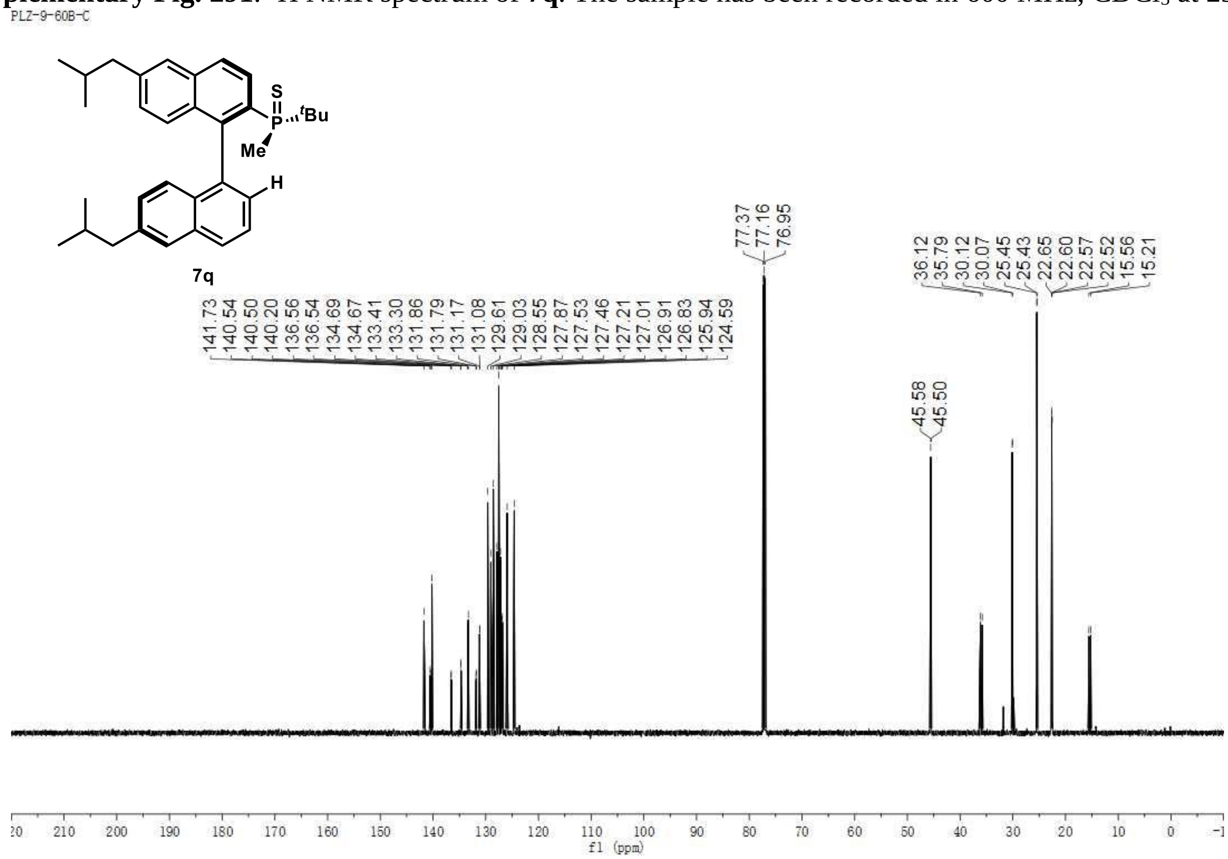

Supplementary Fig. 292.^{13}C NMR spectrum of **7q**. The sample has been recorded in 151 MHz, $CDCl_3$ at 25 ℃.

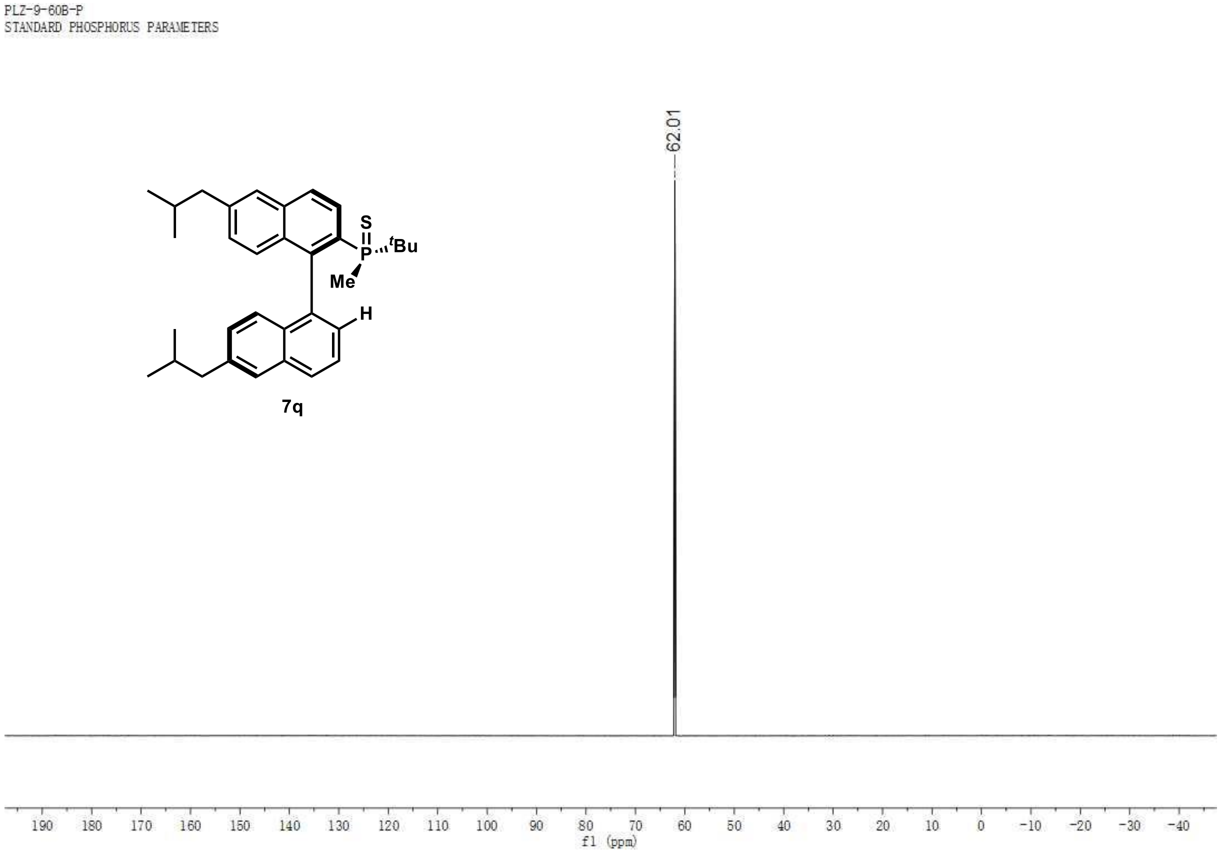

Supplementary Fig. 293.^{31}P NMR spectrum of **7q**. The sample has been recorded in 243 MHz, $CDCl_3$ at 25 ℃.

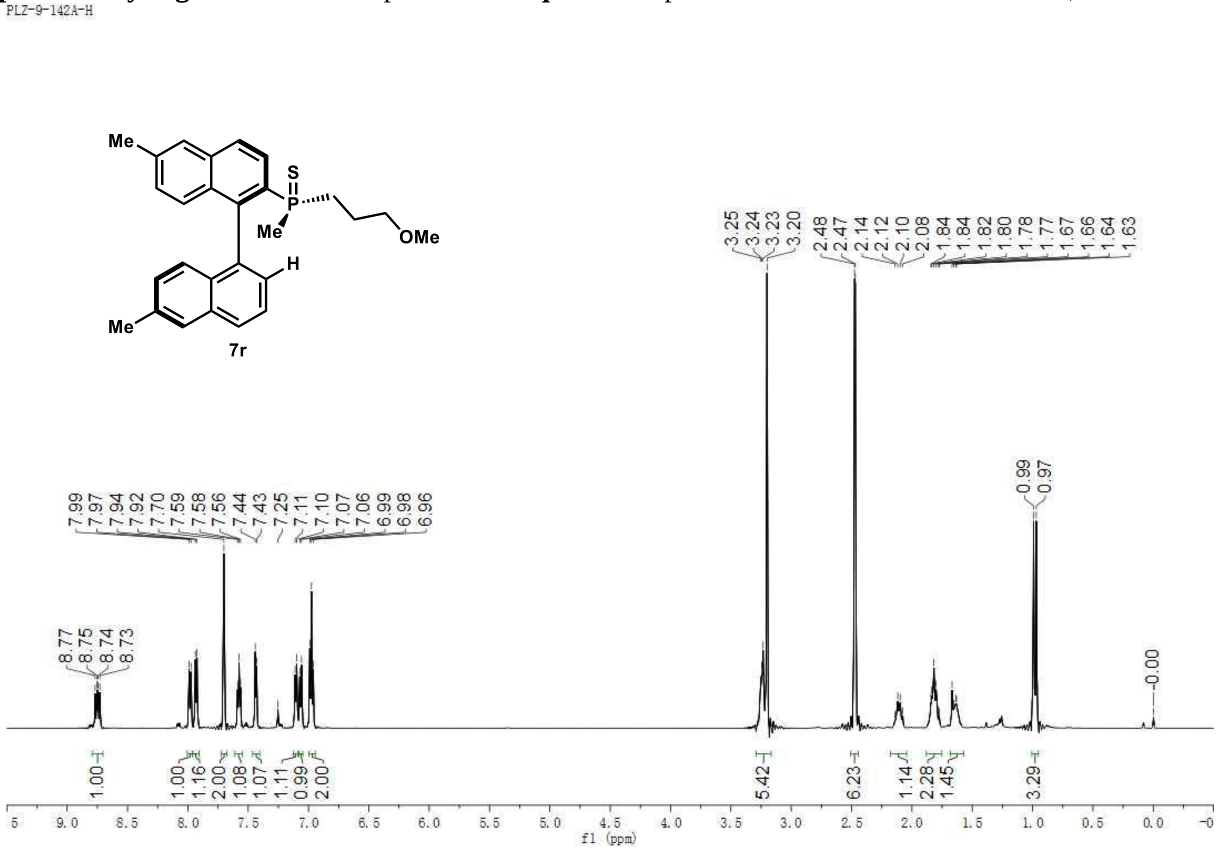

Supplementary Fig. 294. ^{1}H NMR spectrum of **7r**. The sample has been recorded in 600 MHz, $CDCl_3$ at 25 ℃.

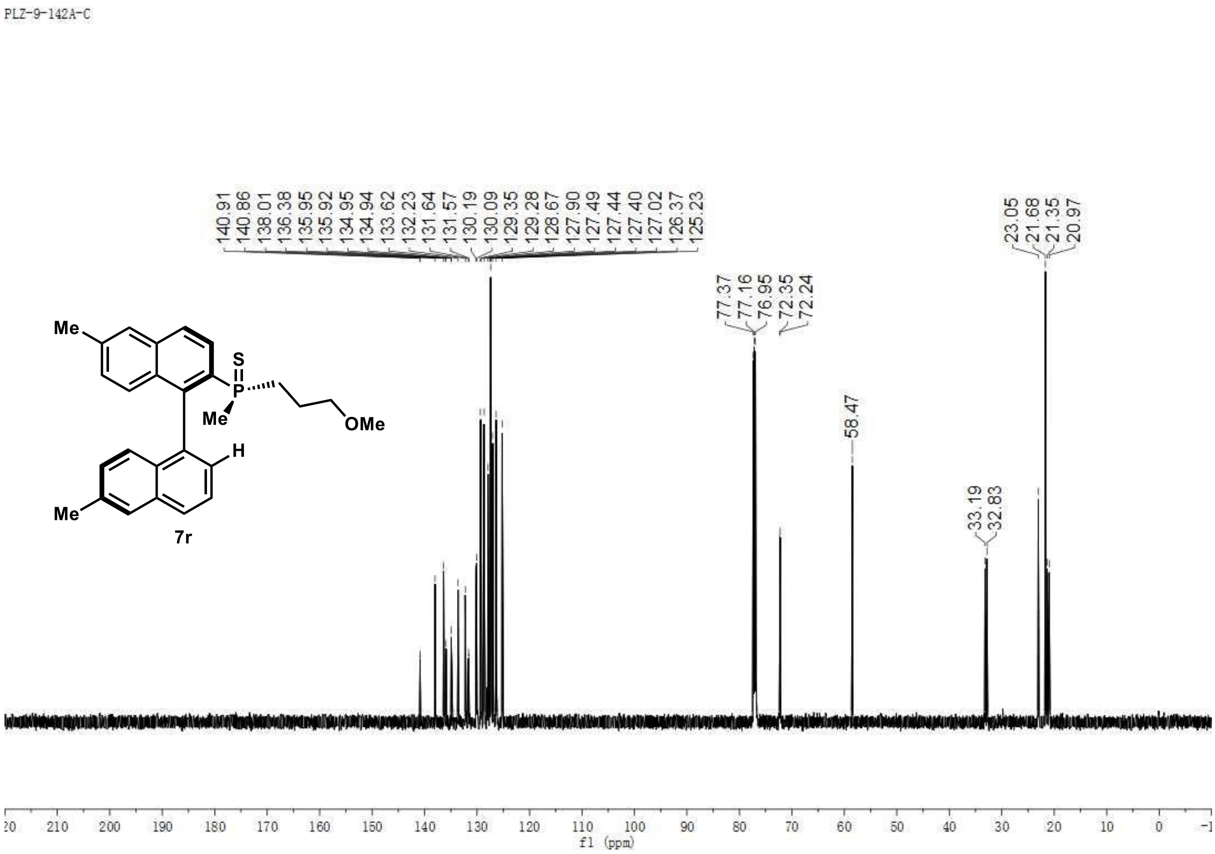

Supplementary Fig. 295.^{13}C NMR spectrum of **7r**. The sample has been recorded in 151 MHz, $CDCl_3$ at 25 ℃.

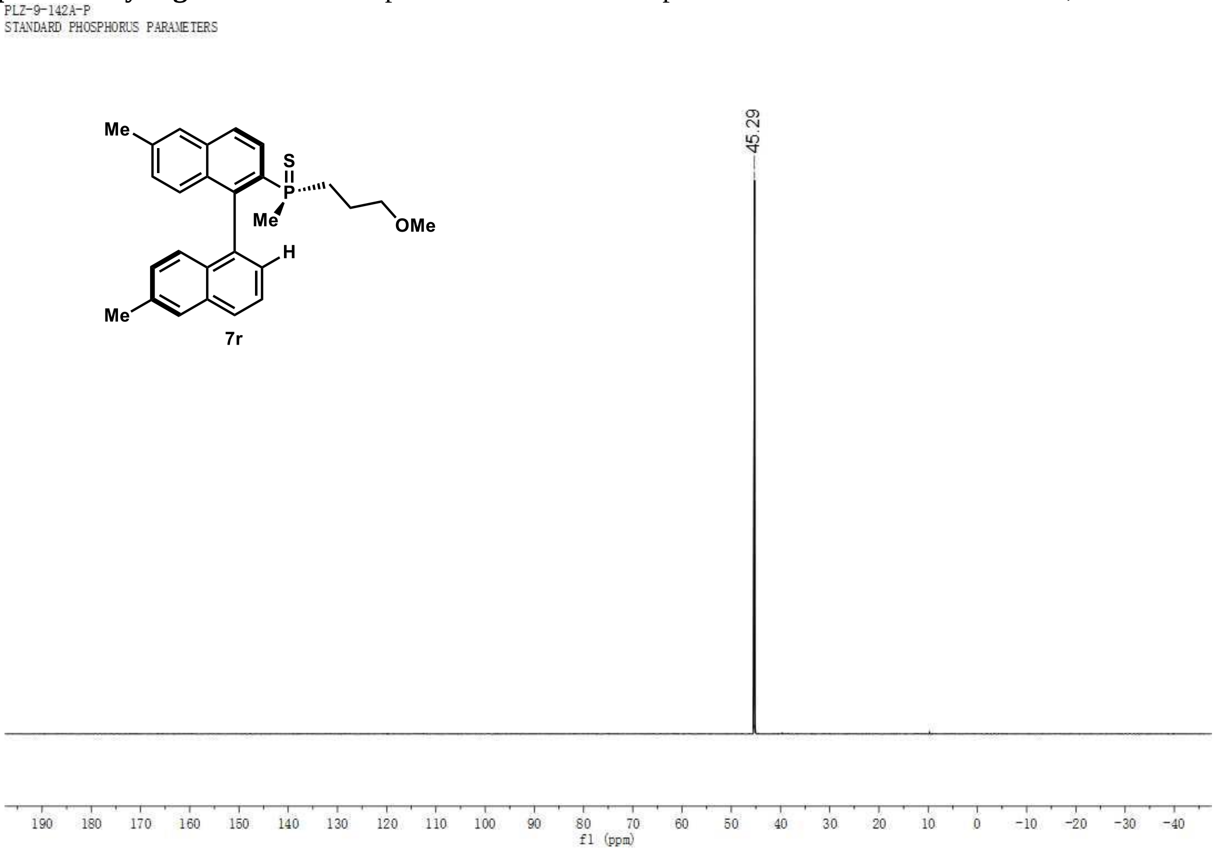

Supplementary Fig. 296.^{31}P NMR spectrum of **7r**. The sample has been recorded in 243 MHz, $CDCl_3$ at 25 ℃.

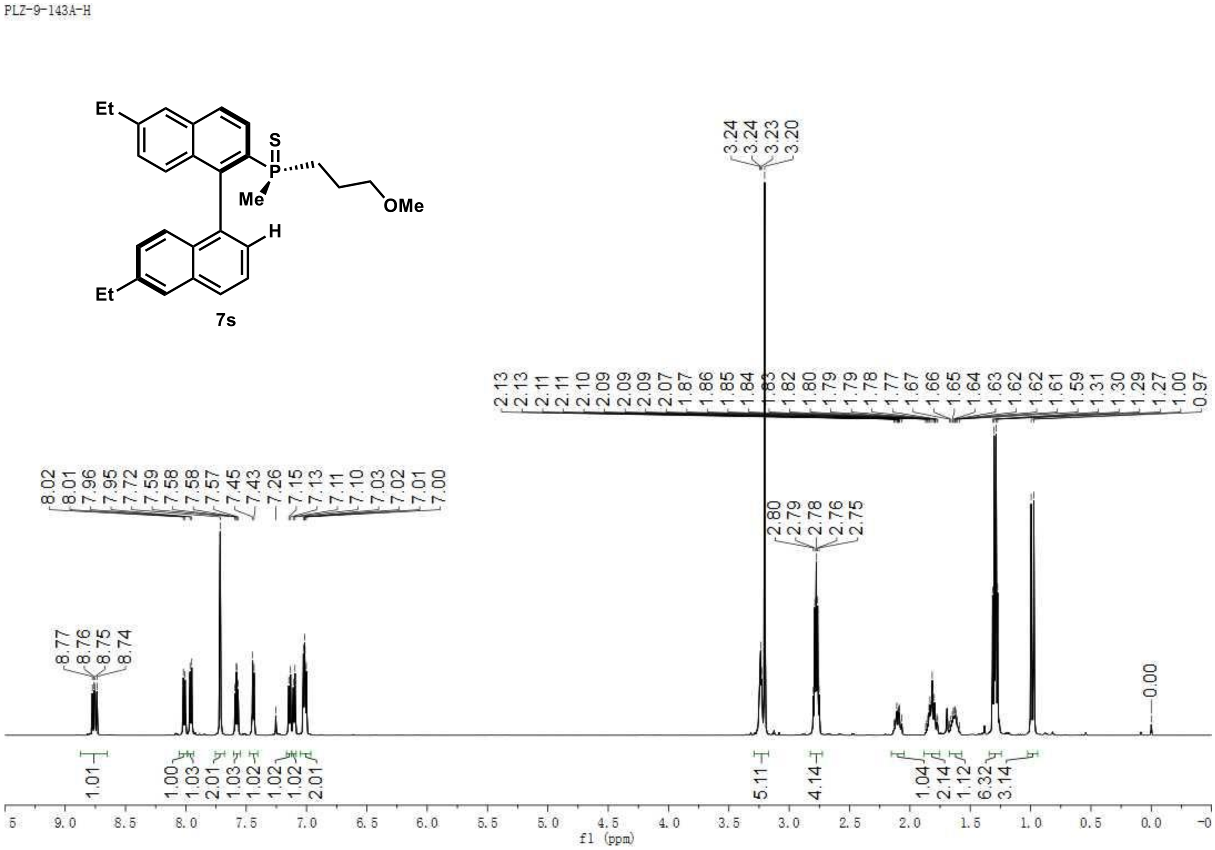

Supplementary Fig. 297. ^{1}H NMR spectrum of **7s**. The sample has been recorded in 600 MHz, $CDCl_3$ at 25 ℃.

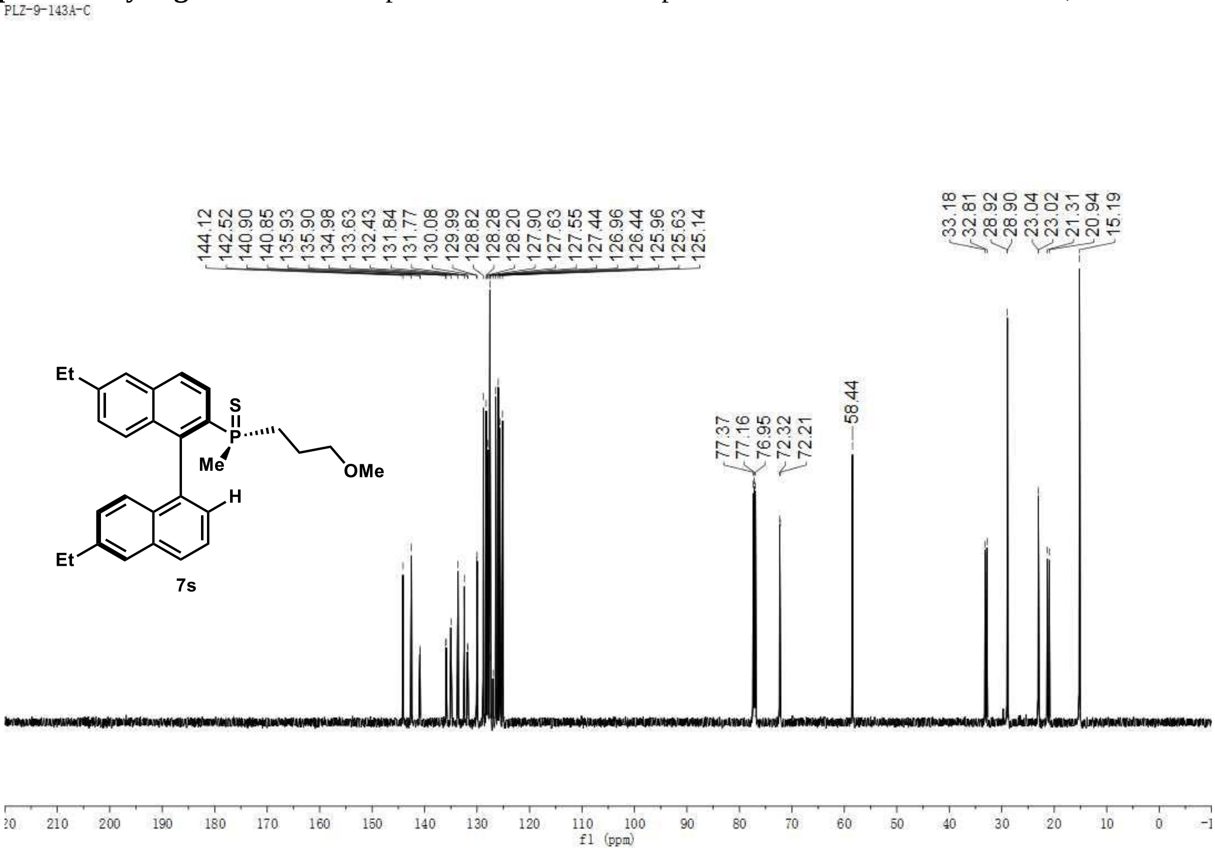

Supplementary Fig. 298.^{13}C NMR spectrum of **7s**. The sample has been recorded in 151 MHz, $CDCl_3$ at 25 ℃.

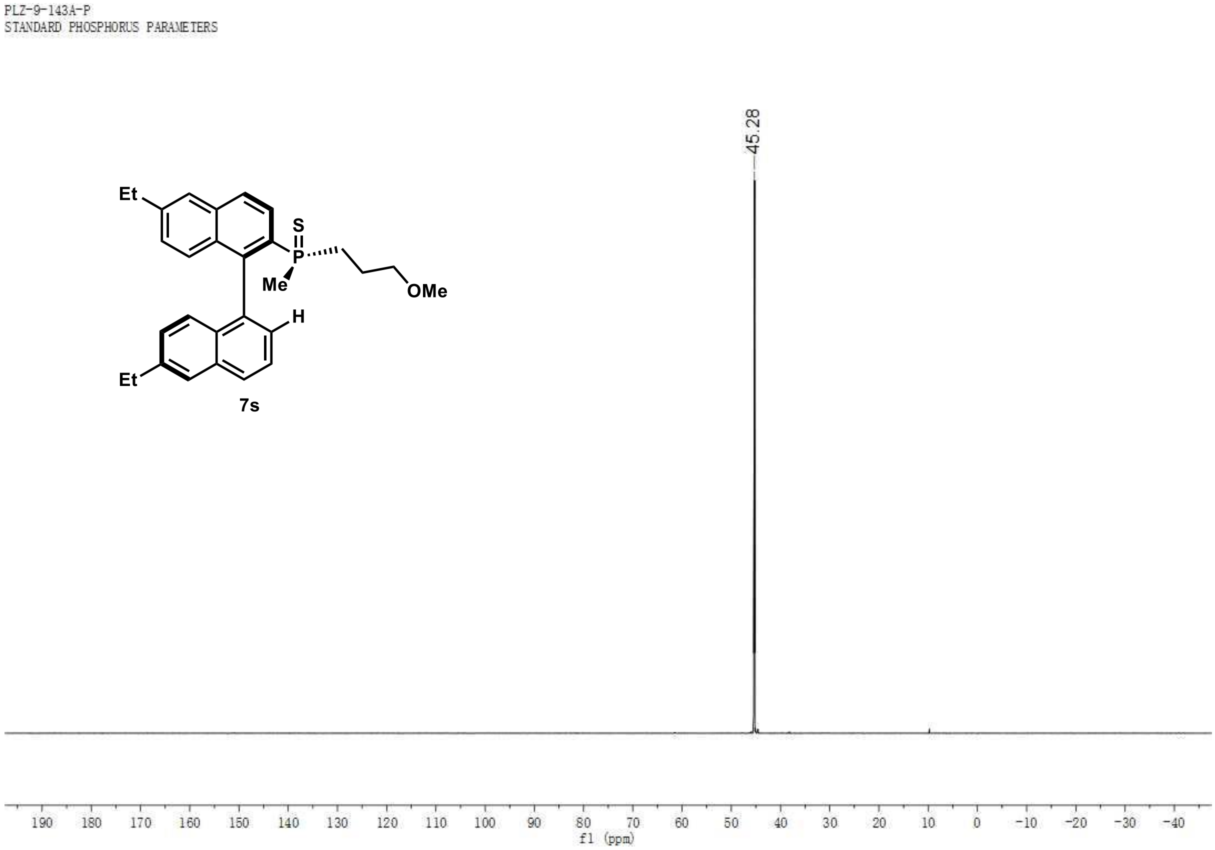

Supplementary Fig. 299.^{31}P NMR spectrum of **7s**. The sample has been recorded in 243 MHz, $CDCl_3$ at 25 ℃.

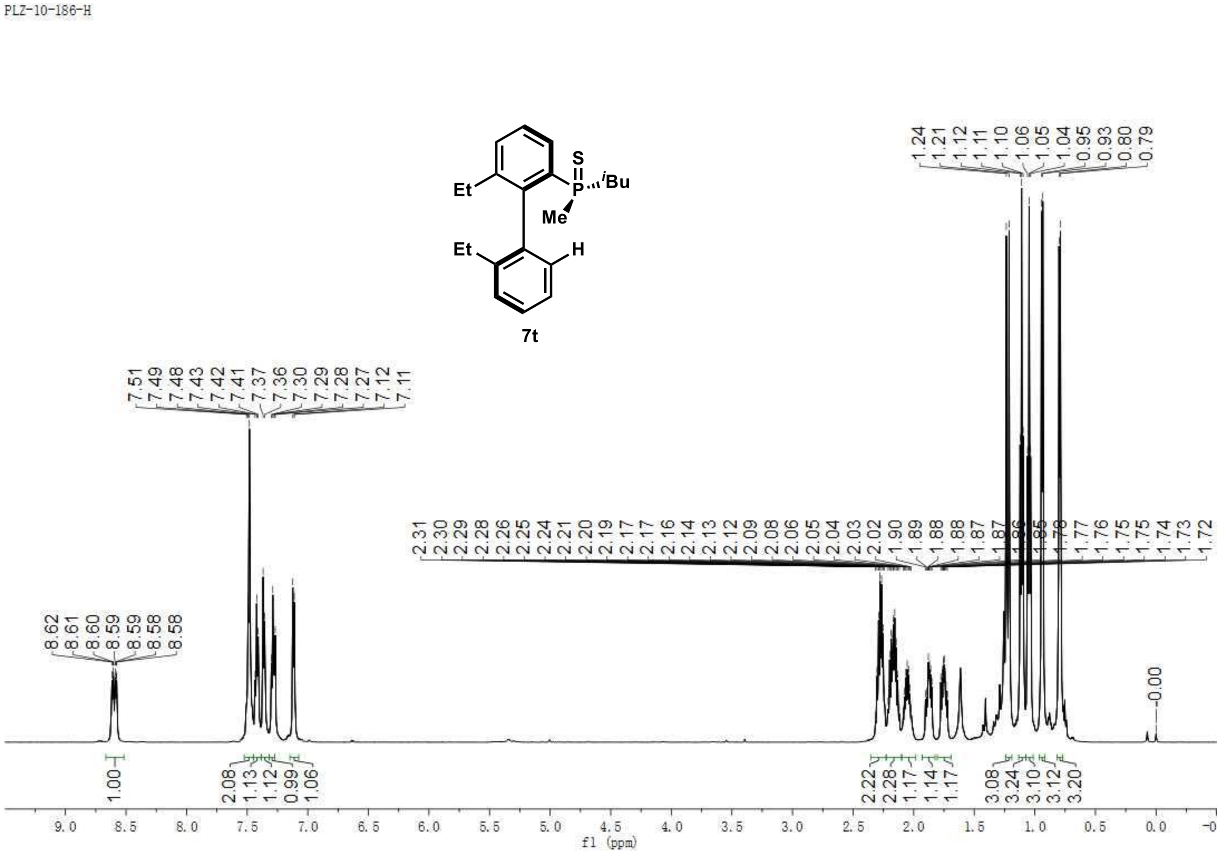

Supplementary Fig. 300. ^{1}H NMR spectrum of **7t**. The sample has been recorded in 600 MHz, $CDCl_3$ at 25 ℃.

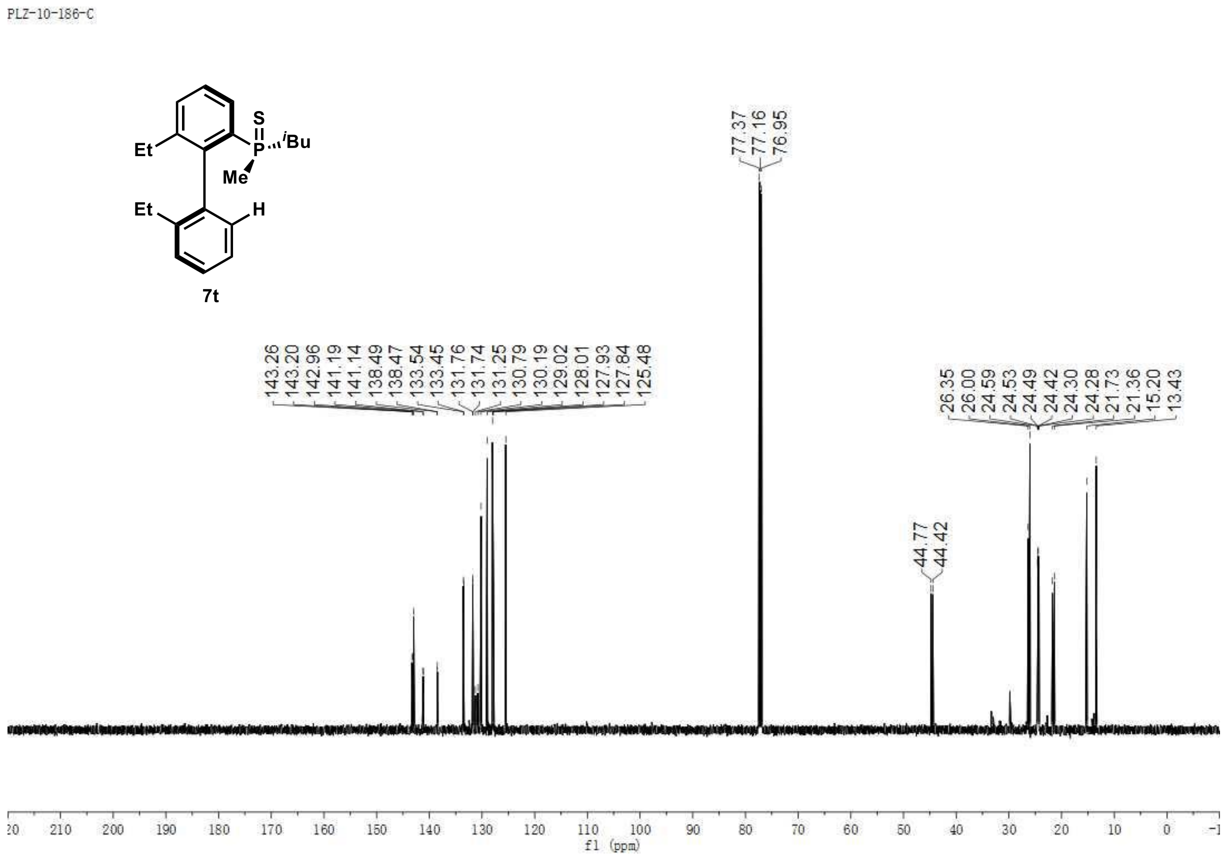

Supplementary Fig. 301.^{13}C NMR spectrum of **7t**. The sample has been recorded in 151 MHz, $CDCl_3$ at 25 ℃.

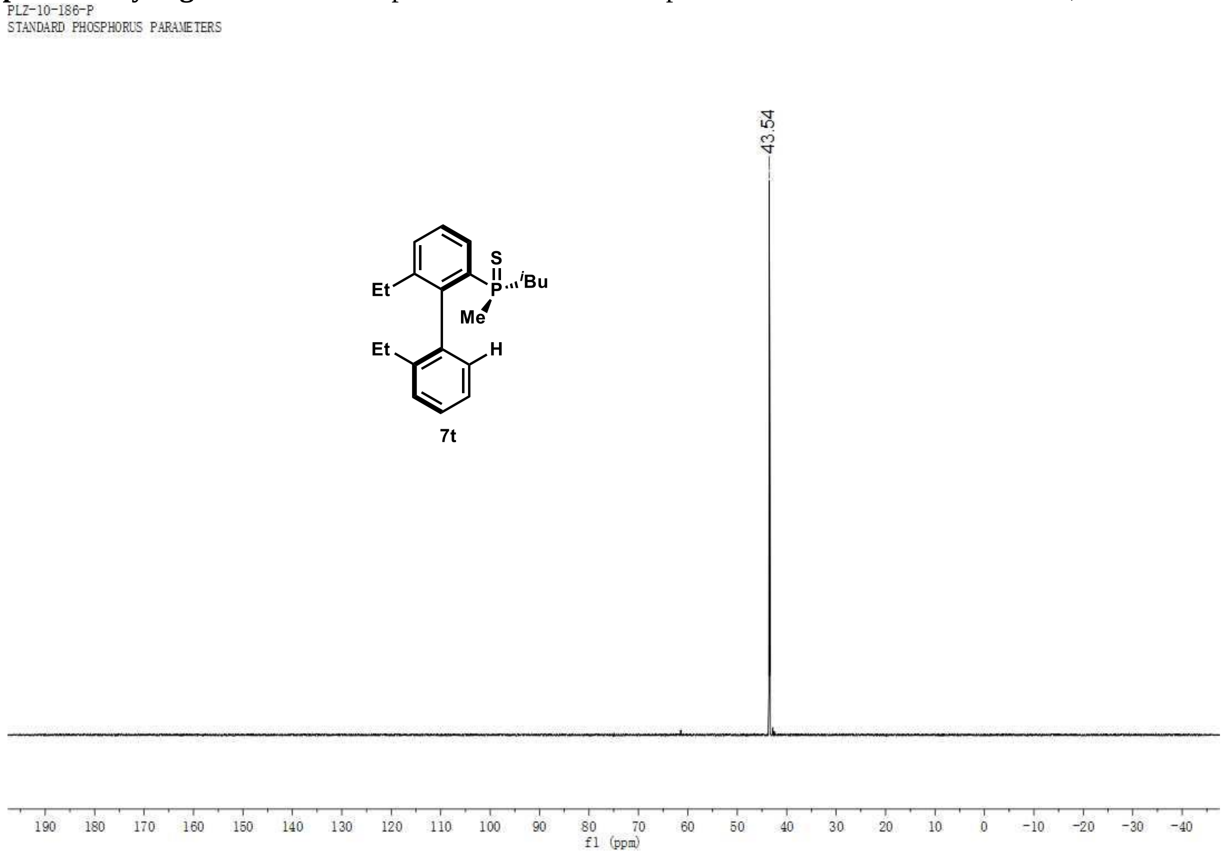

Supplementary Fig. 302.^{31}P NMR spectrum of **7t**. The sample has been recorded in 243 MHz, $CDCl_3$ at 25 ℃.

PLZ-10-164-H

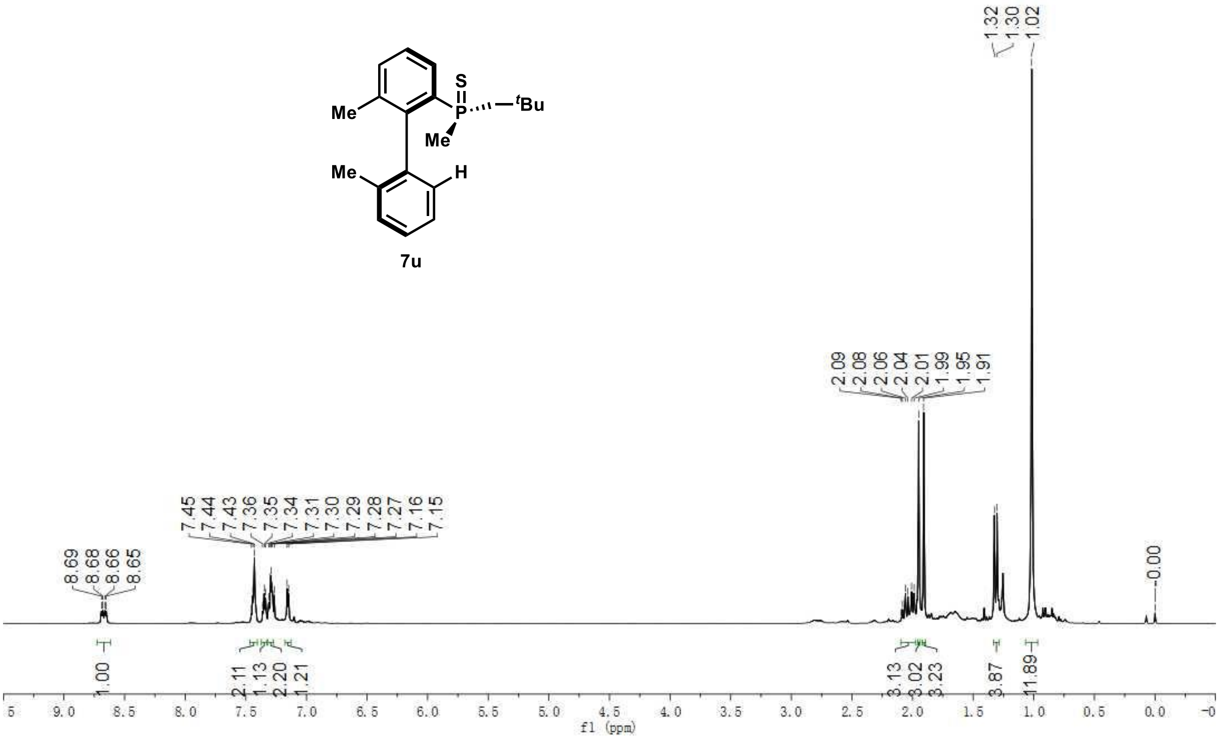

Supplementary Fig. 303. ^{1}H NMR spectrum of **7u**. The sample has been recorded in 600 MHz, $CDCl_3$ at 25 ℃.

PLZ-10-164-C

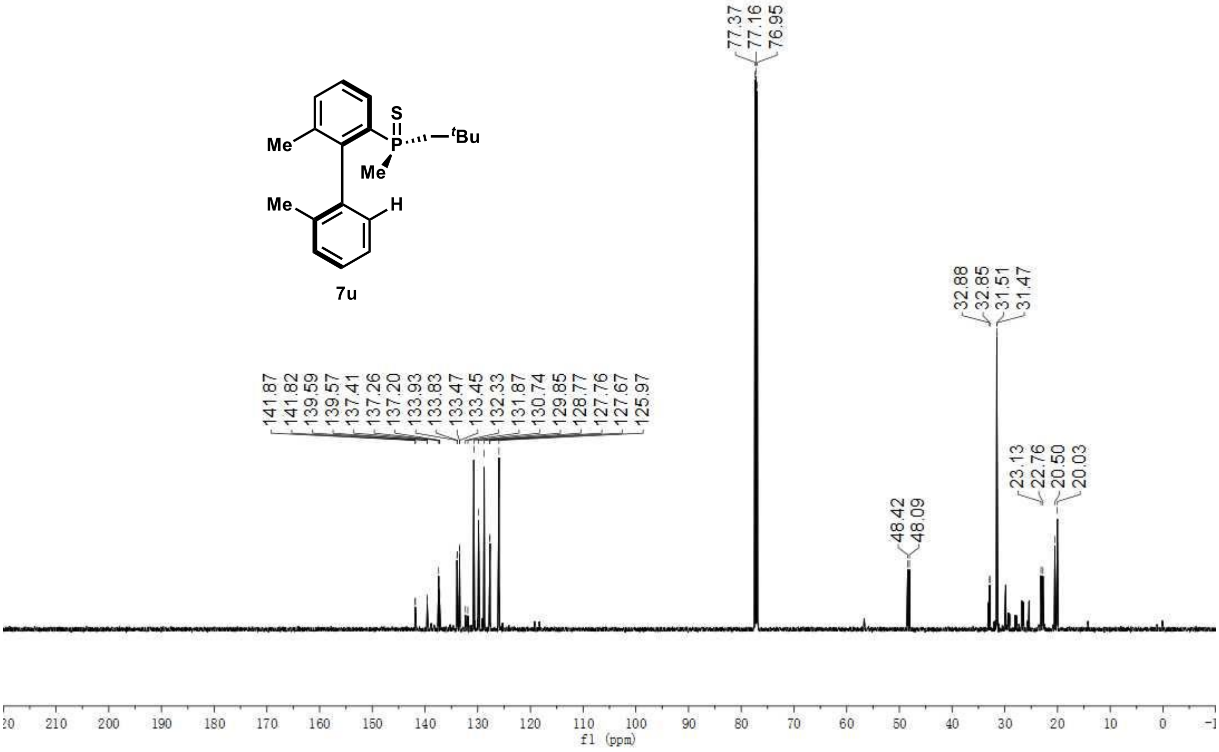

Supplementary Fig. 304. ^{13}C NMR spectrum of **7u**. The sample has been recorded in 151 MHz, $CDCl_3$ at 25 ℃.

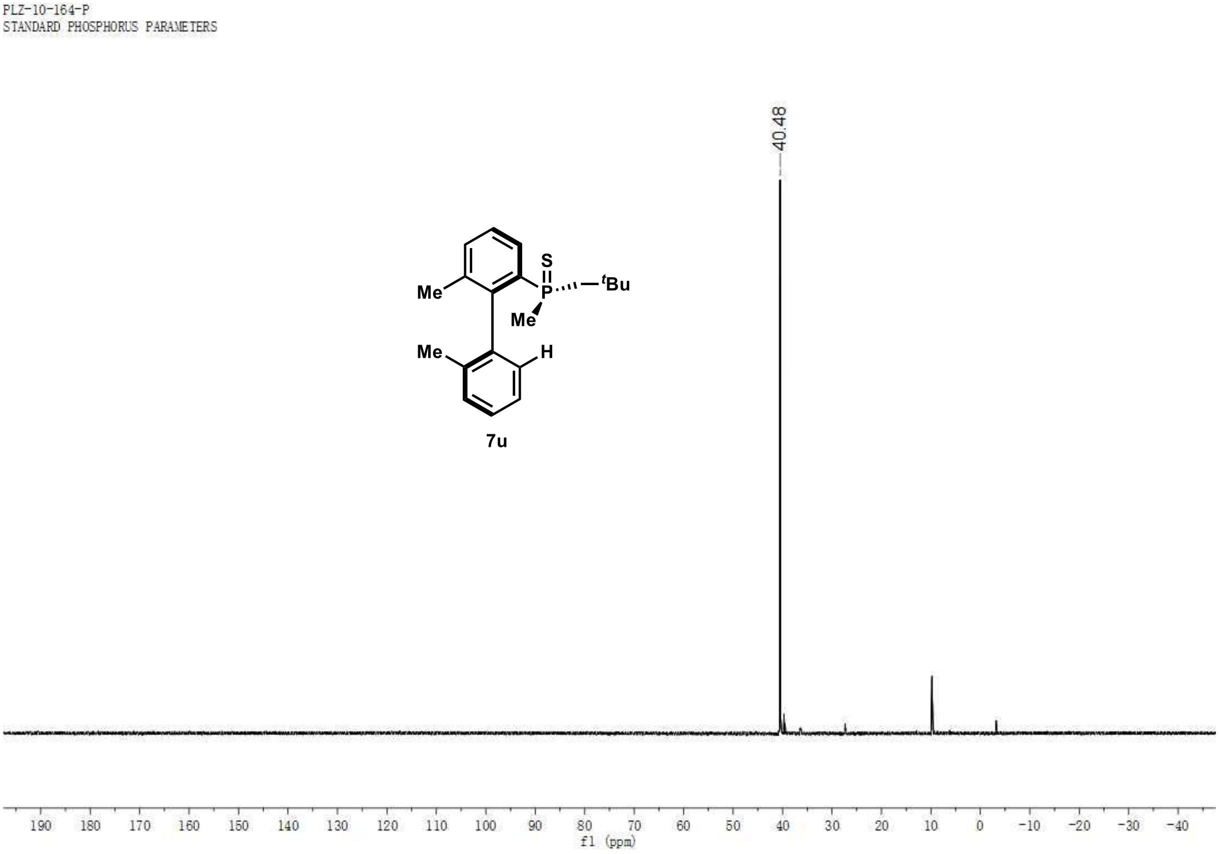

Supplementary Fig. 305.^{31}P NMR spectrum of **7u**. The sample has been recorded in 243 MHz, $CDCl_3$ at 25 ℃.

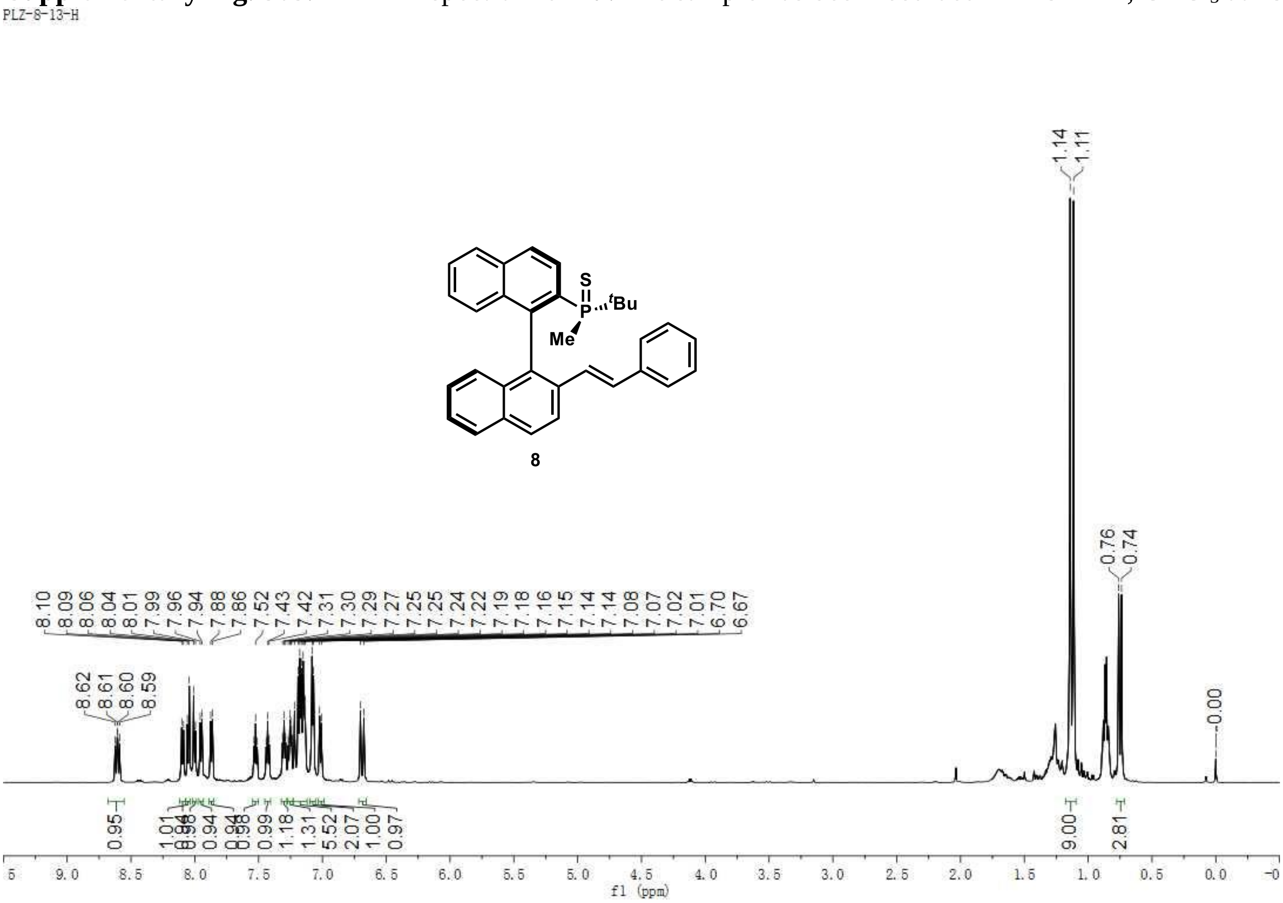

Supplementary Fig. 306. ^{1}H NMR spectrum of **8**. The sample has been recorded in 600 MHz, $CDCl_3$ at 25 ℃.

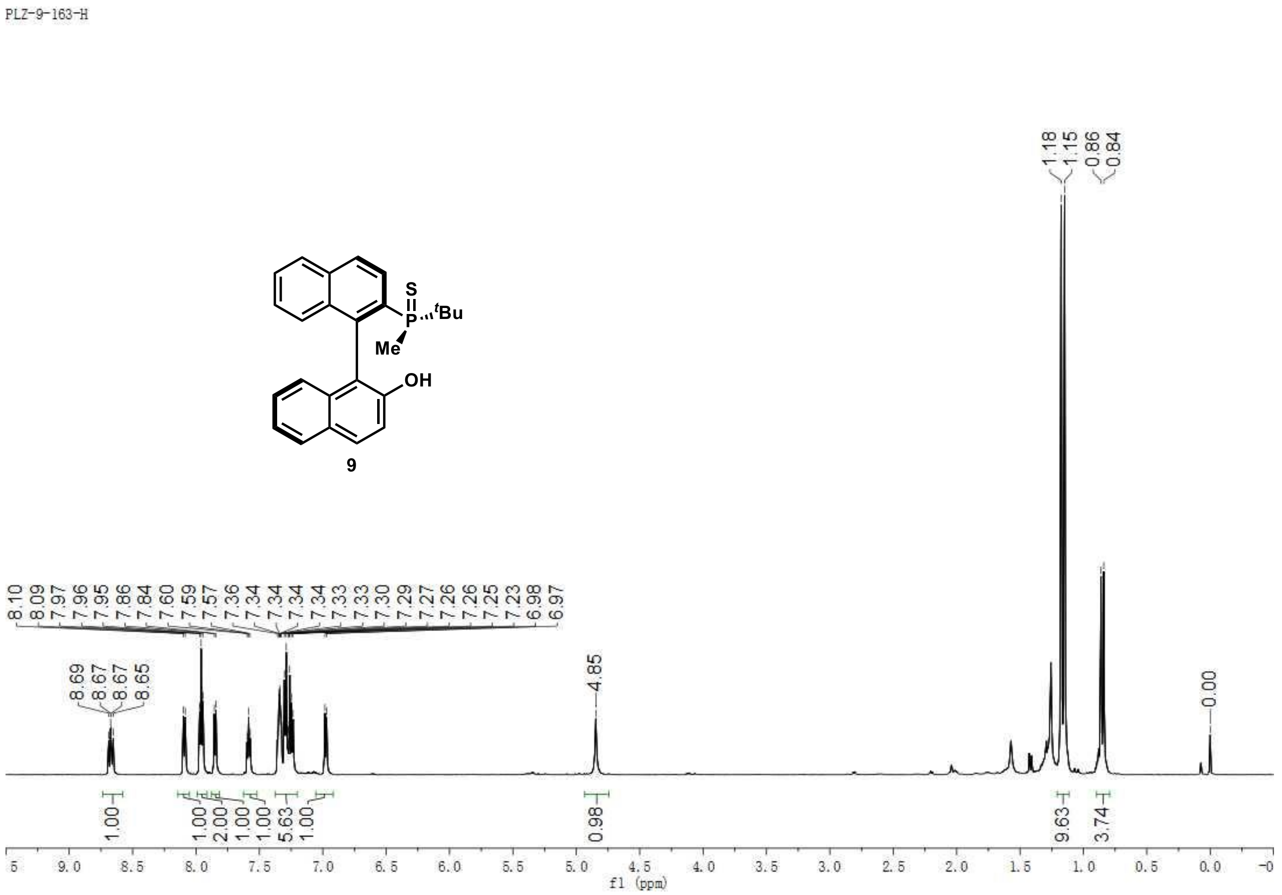

Supplementary Fig. 307. ^{1}H NMR spectrum of **9**. The sample has been recorded in 600 MHz, $CDCl_3$ at 25 ℃.

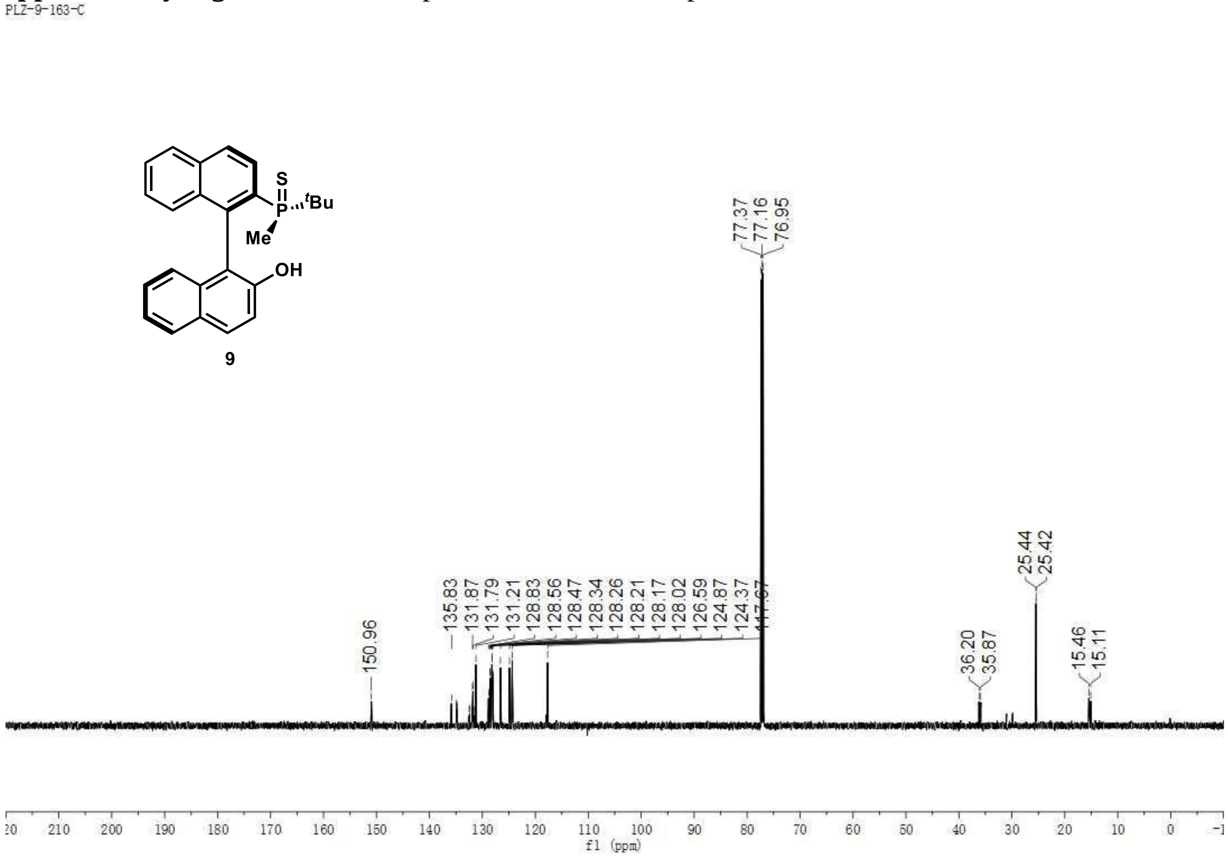

Supplementary Fig. 308.^{13}C NMR spectrum of **9**. The sample has been recorded in 151 MHz, $CDCl_3$ at 25 ℃.

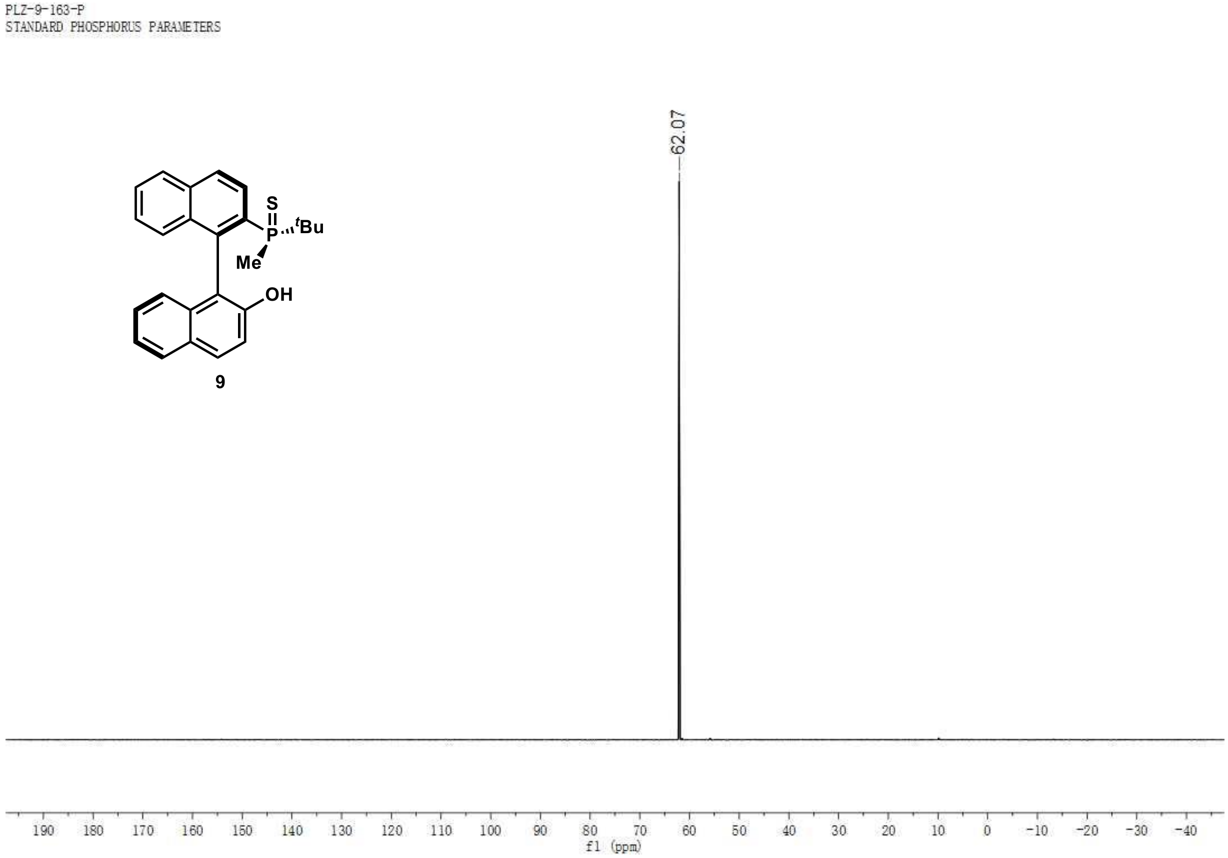

Supplementary Fig. 309.^{31}P NMR spectrum of **9**. The sample has been recorded in 243 MHz, $CDCl_3$ at 25 ℃.

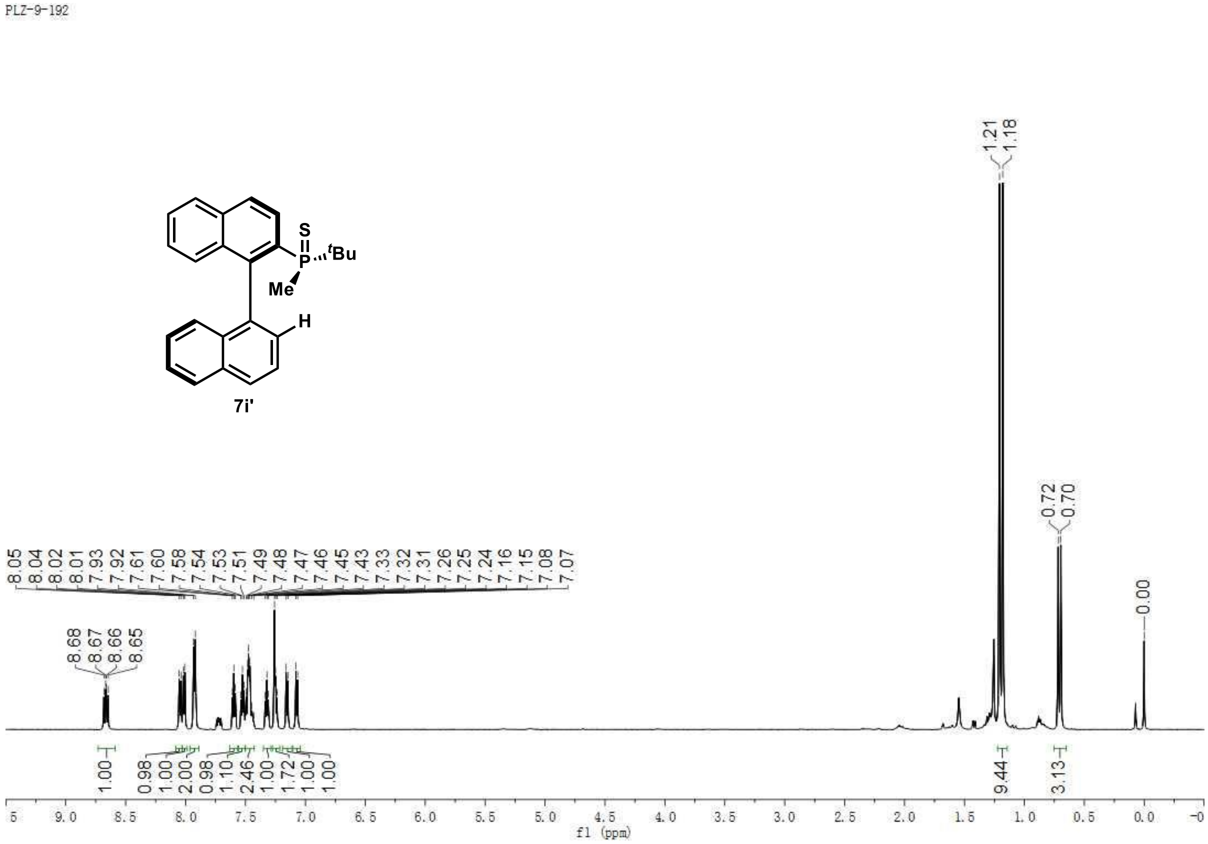

Supplementary Fig. 310. ^{1}H NMR spectrum of **7i'**. The sample has been recorded in 600 MHz, $CDCl_3$ at 25 ℃.

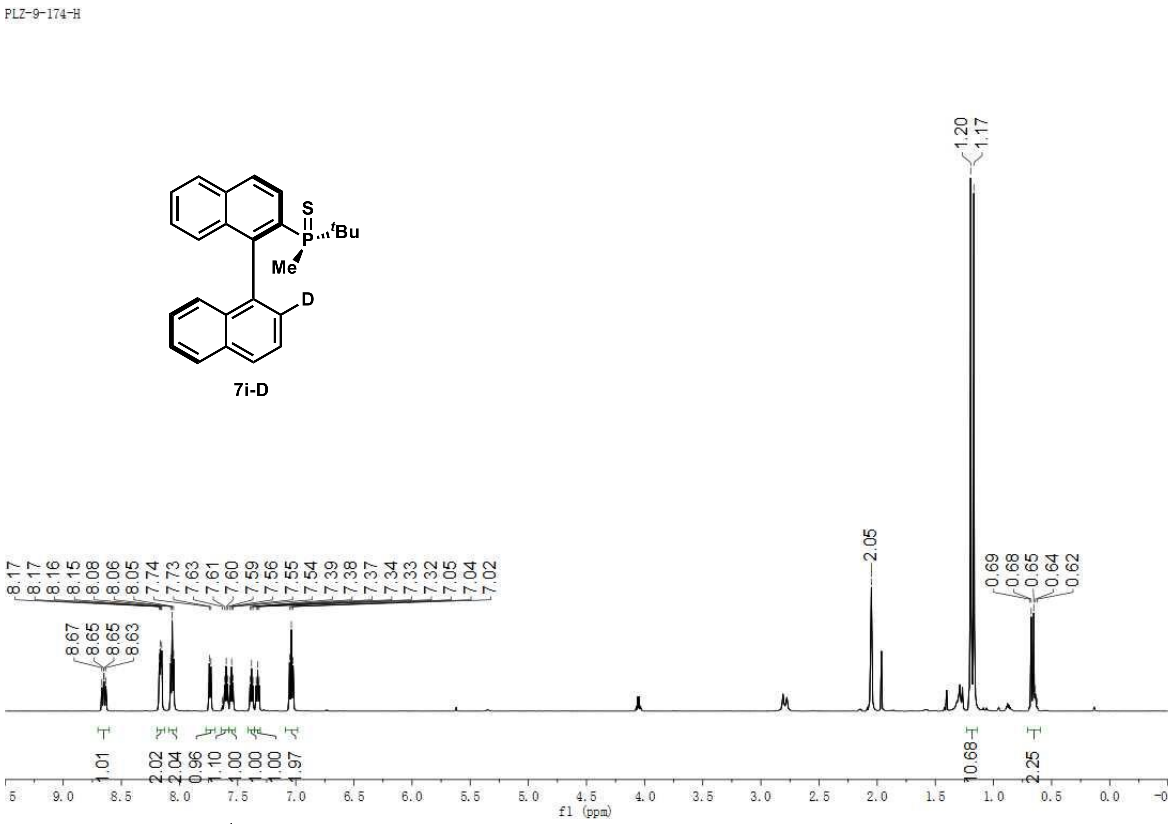

Supplementary Fig. 311. ^{1}H NMR spectrum of **7i-D**. The sample has been recorded in 600 MHz, $CDCl_3$ at 25 ℃.

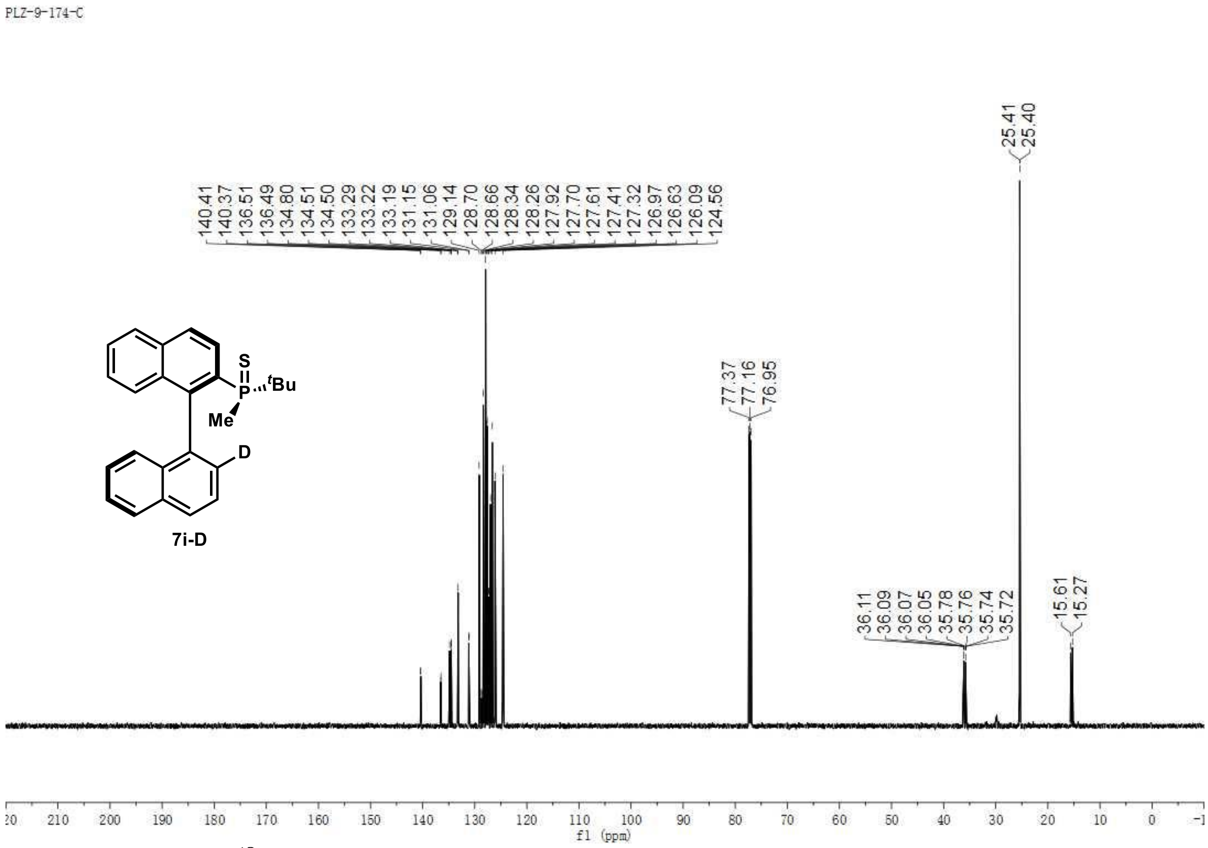

Supplementary Fig. 312.^{13}C NMR spectrum of **7i-D**. The sample has been recorded in 151 MHz, $CDCl_3$ at 25 ℃.

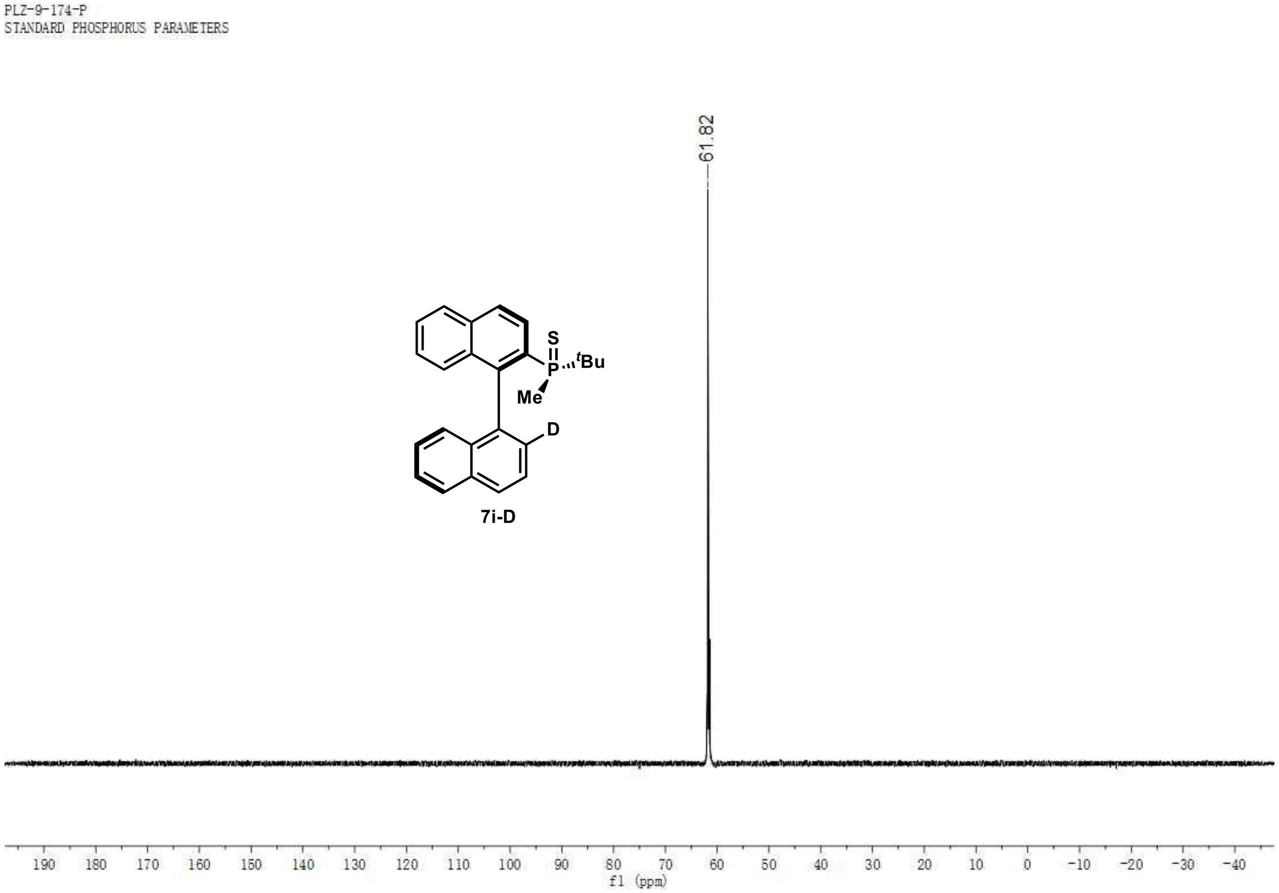

Supplementary Fig. 313.^{31}P NMR spectrum of **7i-D**. The sample has been recorded in 243 MHz, $CDCl_3$ at 25 ℃.

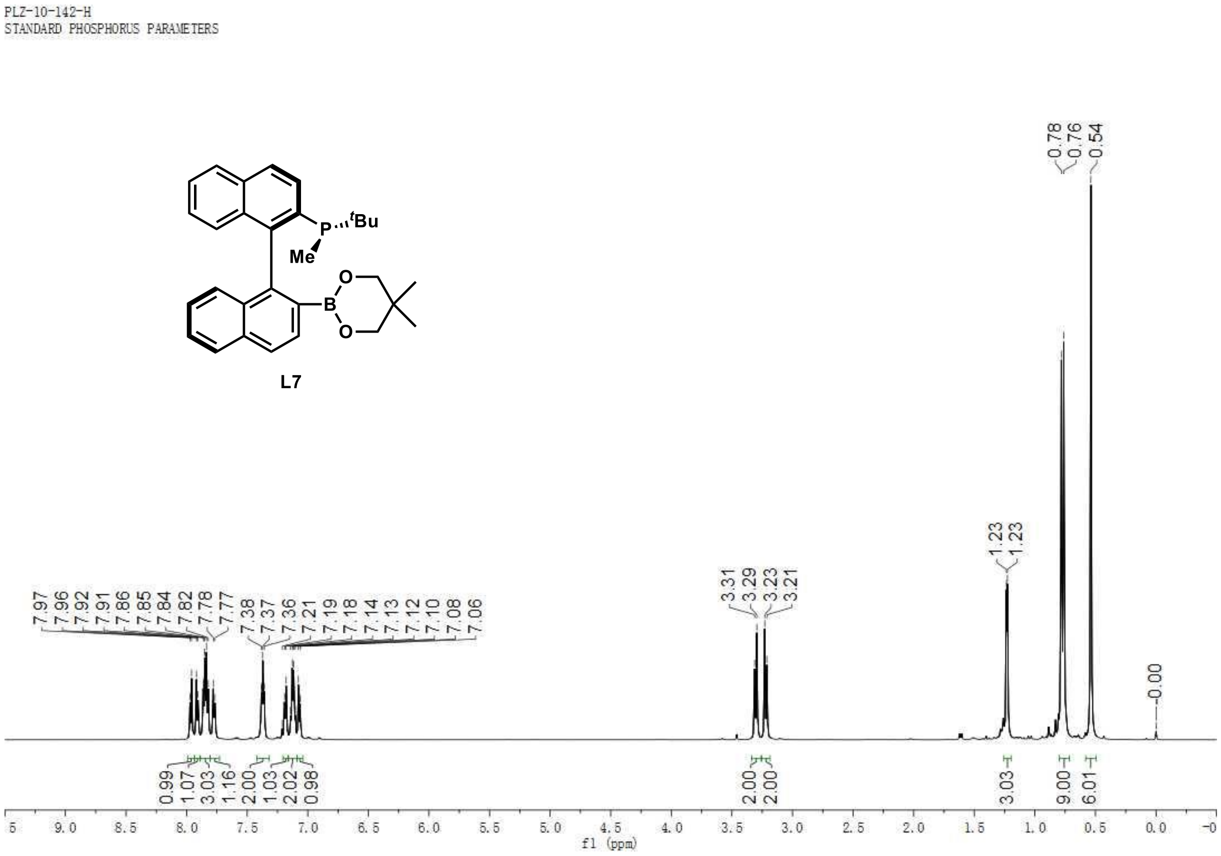

Supplementary Fig. 314. ^{1}H NMR spectrum of **L7**. The sample has been recorded in 600 MHz, $CDCl_3$ at 25 ℃.

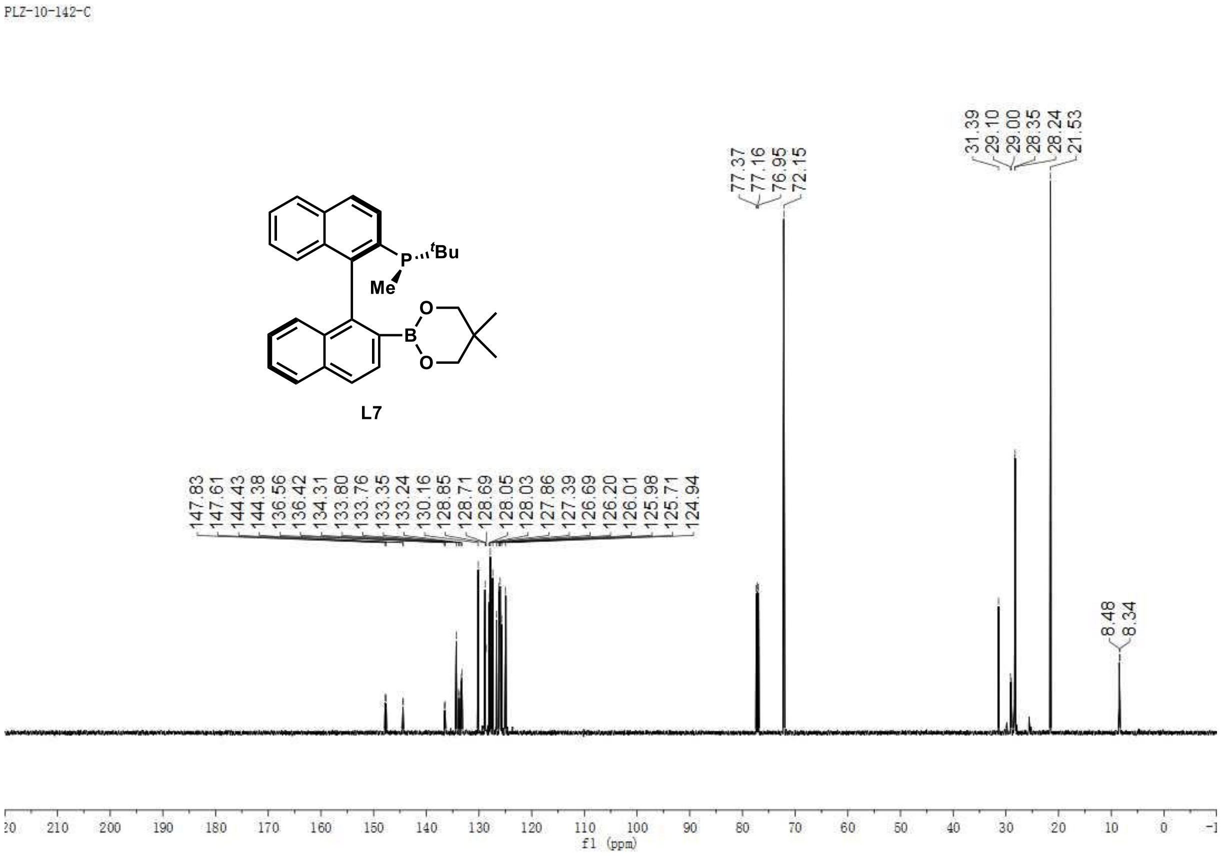

Supplementary Fig. 315.^{13}C NMR spectrum of **L7**. The sample has been recorded in 151 MHz, $CDCl_3$ at 25 ℃.

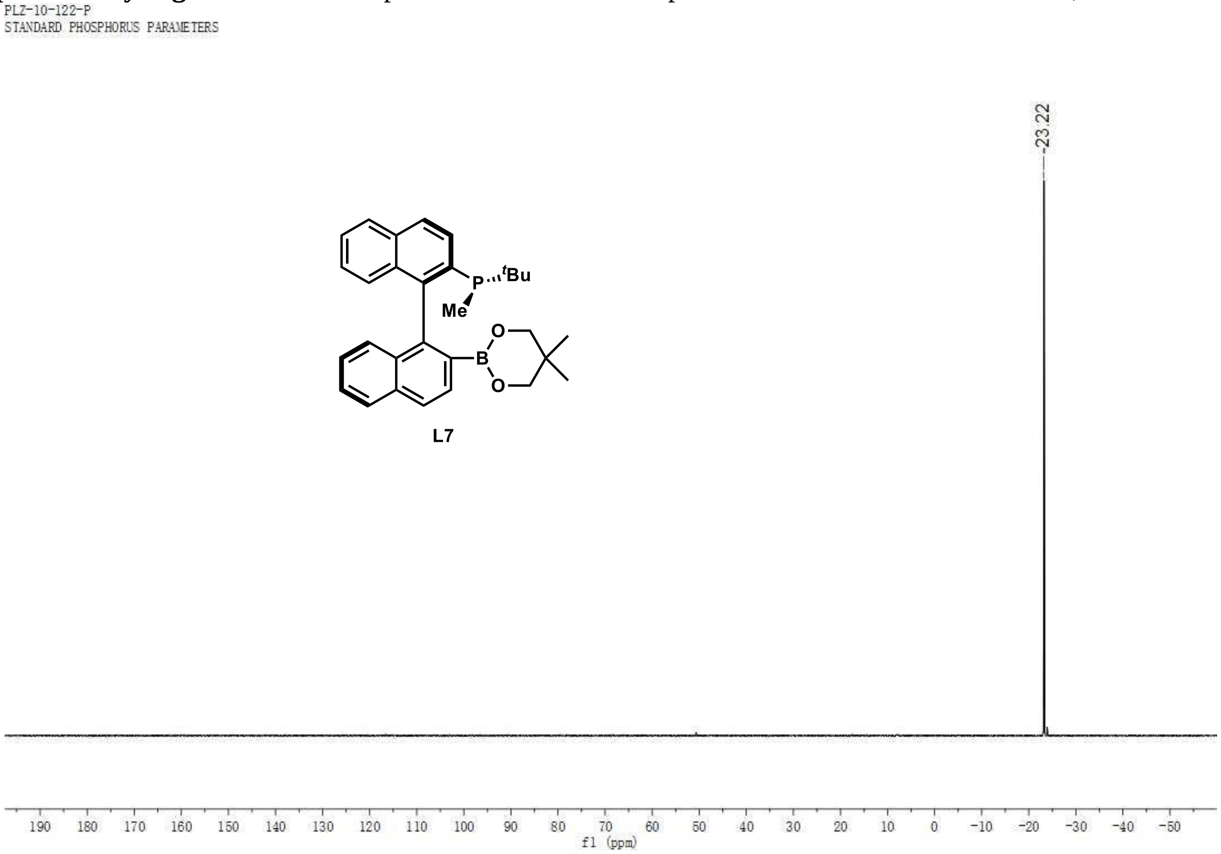

Supplementary Fig. 316.^{31}P NMR spectrum of **L7**. The sample has been recorded in 243 MHz, $CDCl_3$ at 25 ℃.

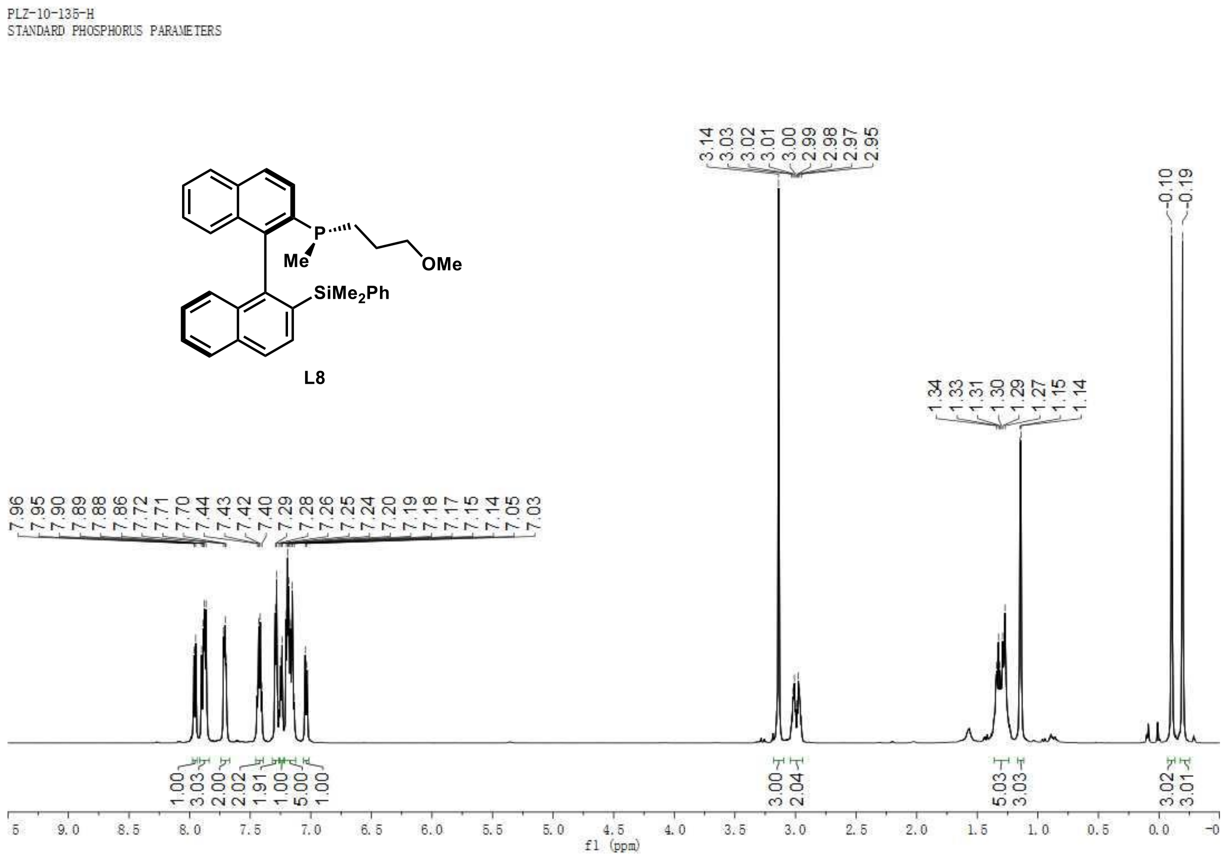

Supplementary Fig. 317. ^{1}H NMR spectrum of **L8**. The sample has been recorded in 600 MHz, $CDCl_3$ at 25 ℃.

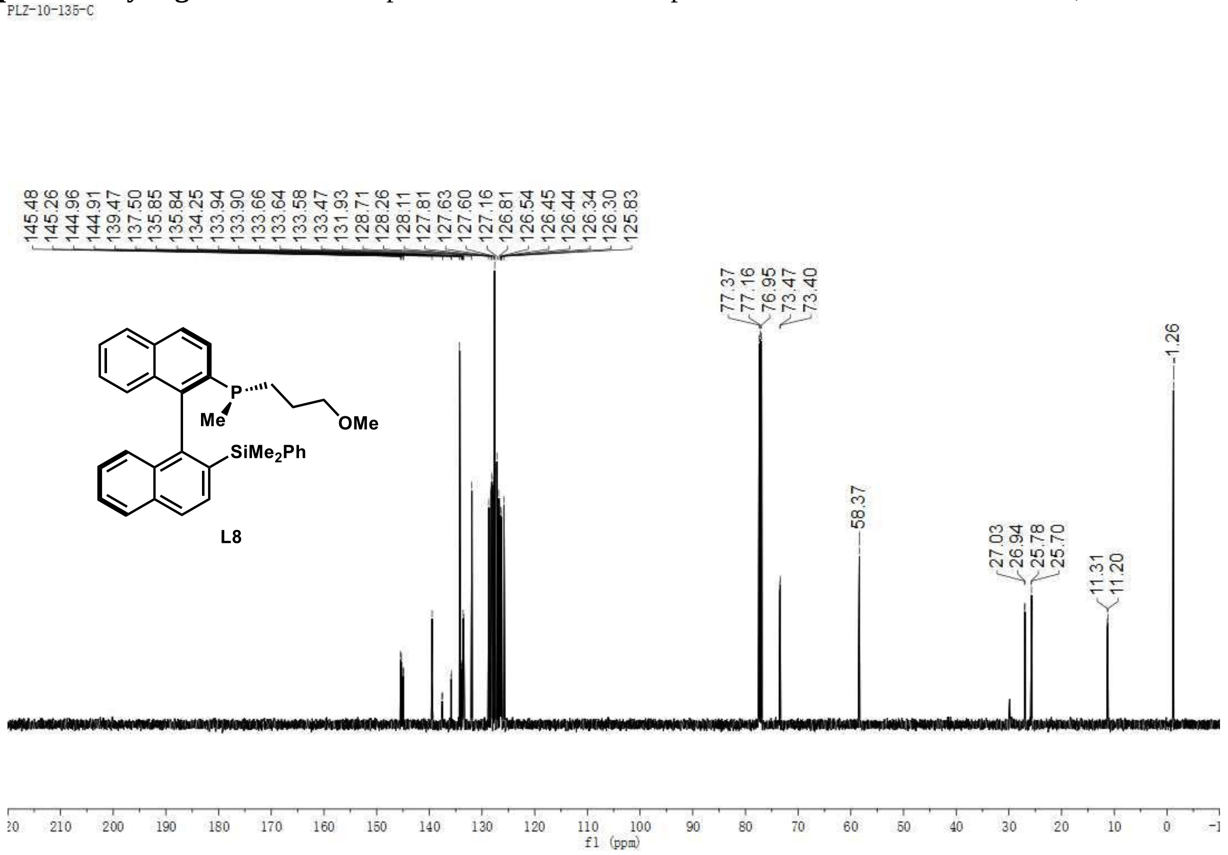

Supplementary Fig. 318.^{13}C NMR spectrum of **L8**. The sample has been recorded in 151 MHz, $CDCl_3$ at 25 ℃.

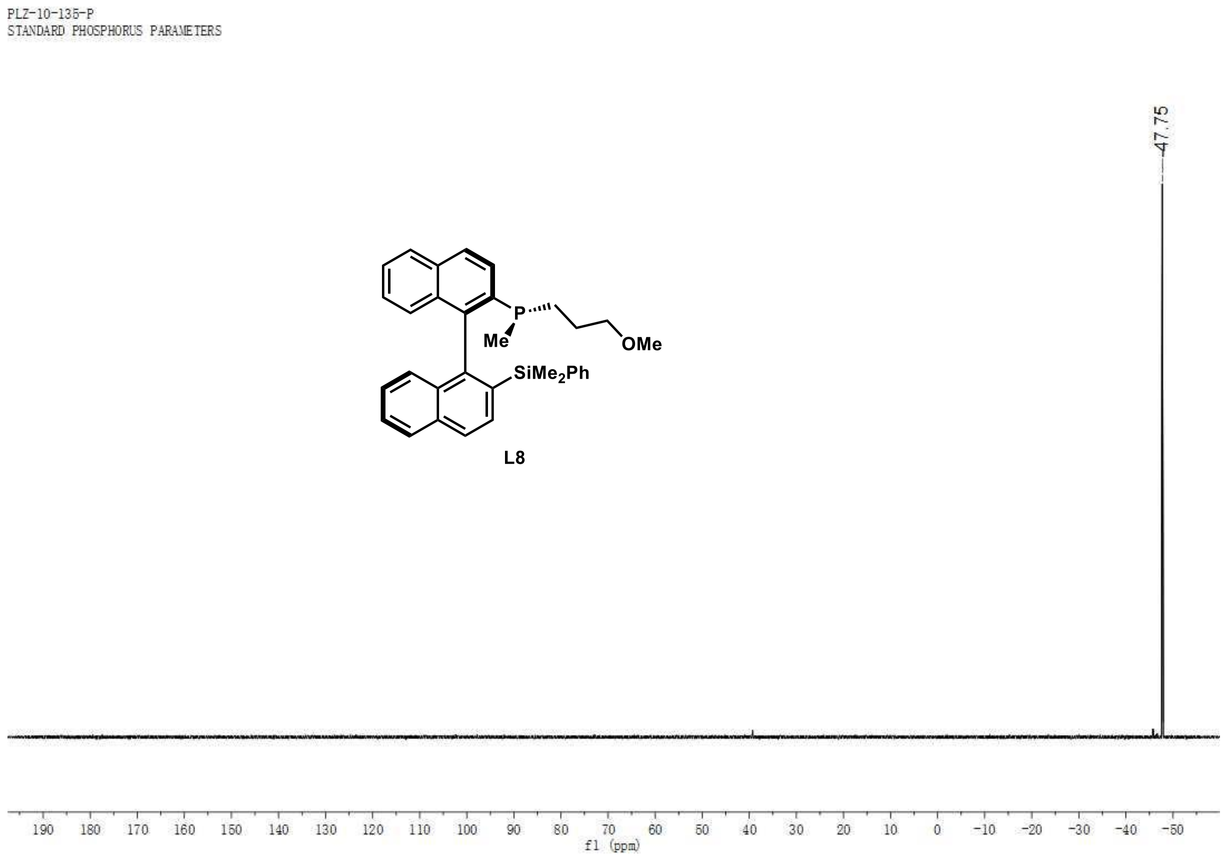

Supplementary Fig. 319. ^{31}P NMR spectrum of **L8**. The sample has been recorded in 243 MHz, $CDCl_3$ at 25 ℃.

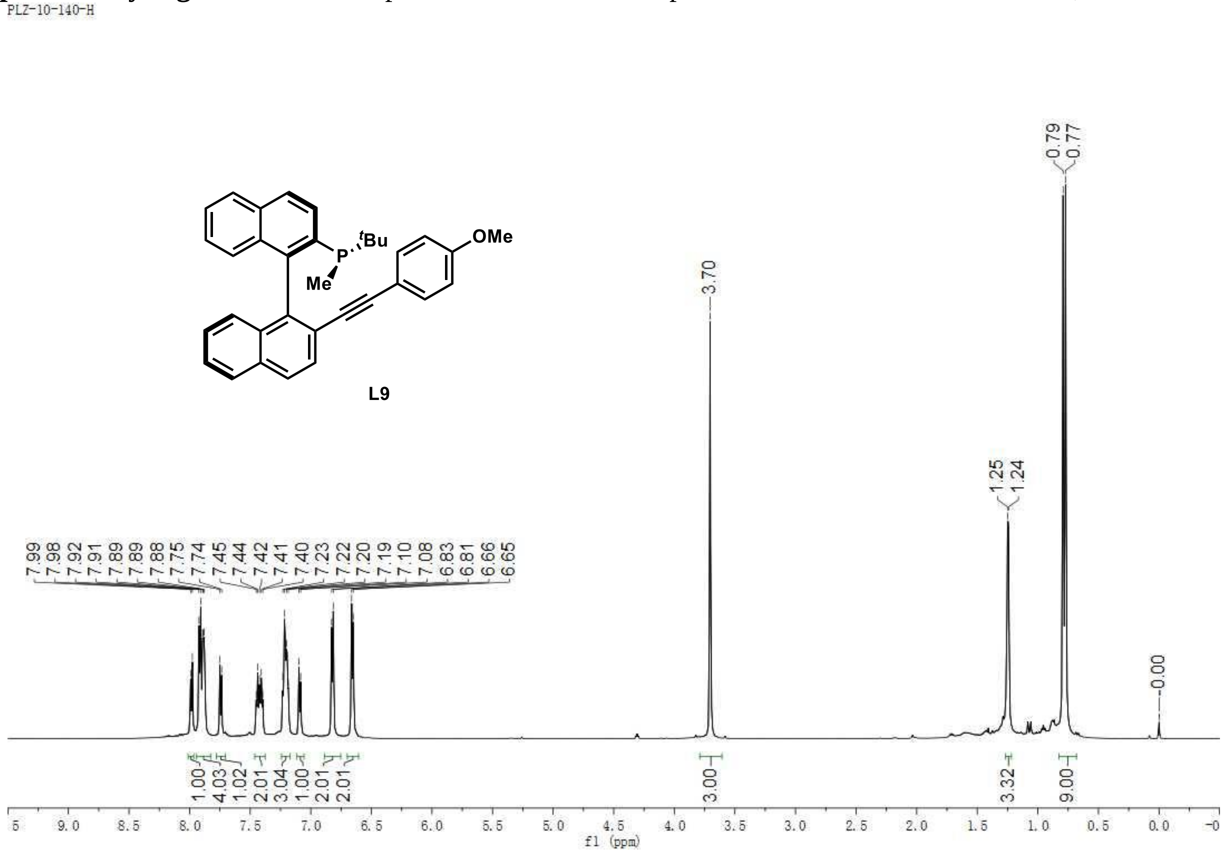

Supplementary Fig. 320. ^{1}H NMR spectrum of **L9**. The sample has been recorded in 600 MHz, $CDCl_3$ at 25 ℃.

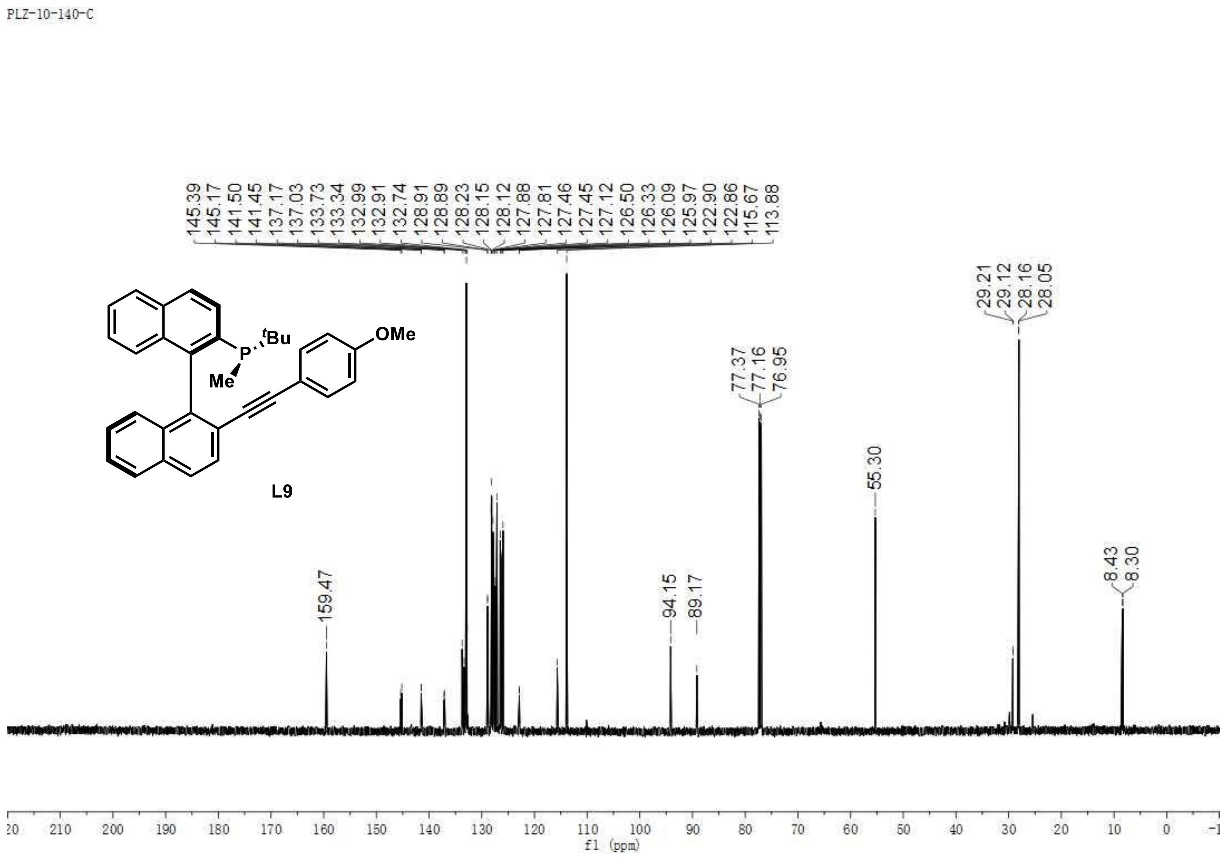

Supplementary Fig. 321.^{13}C NMR spectrum of **L9**. The sample has been recorded in 151 MHz, $CDCl_3$ at 25 ℃.

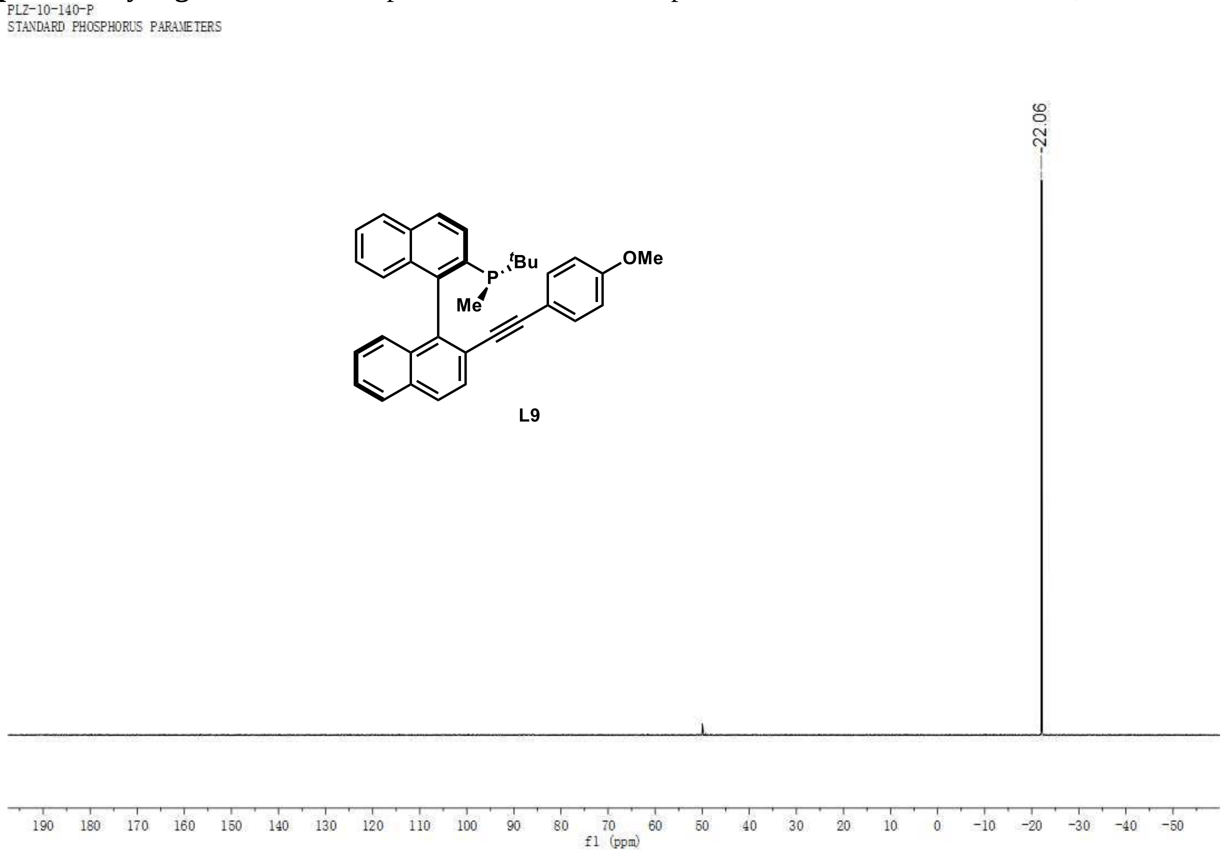

Supplementary Fig. 322.^{31}P NMR spectrum of **L9**. The sample has been recorded in 243 MHz, $CDCl_3$ at 25 ℃.

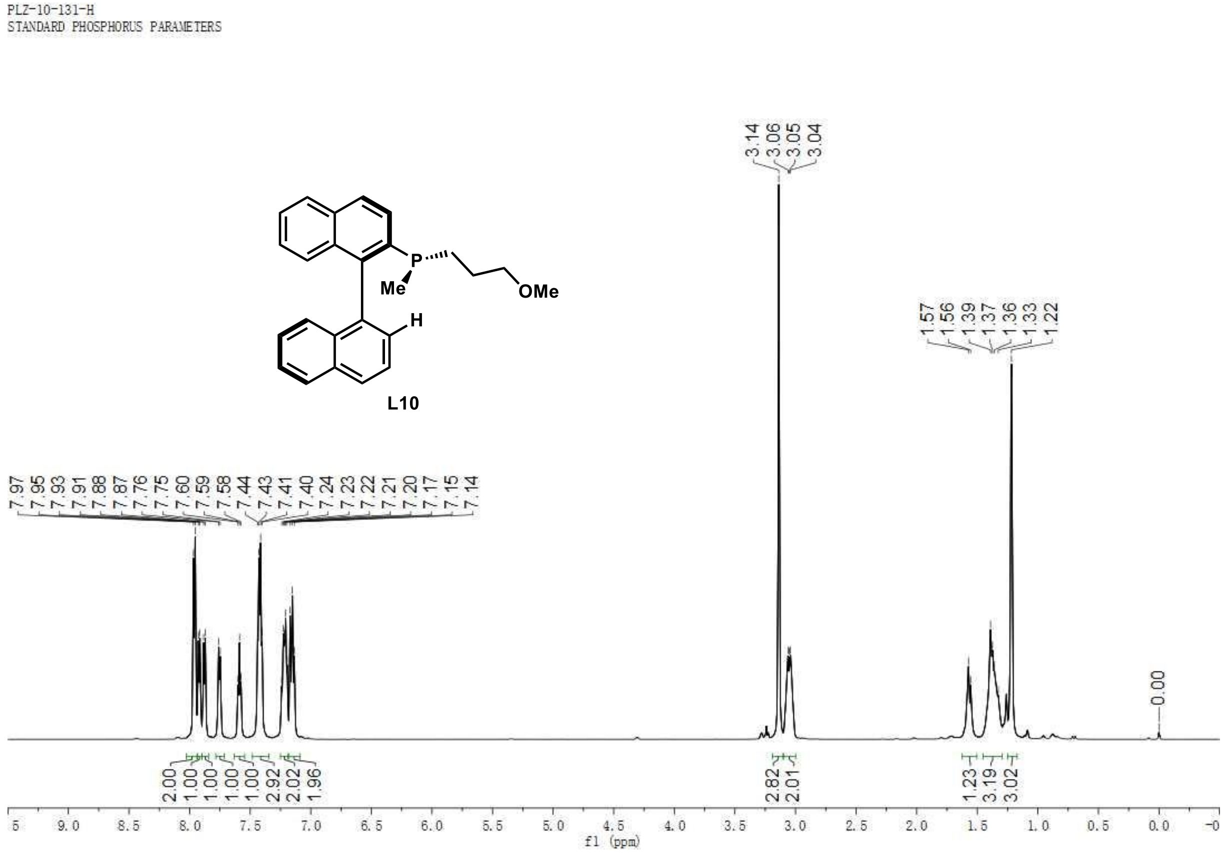

Supplementary Fig. 323. ^{1}H NMR spectrum of **L10**. The sample has been recorded in 600 MHz, $CDCl_3$ at 25 ℃.

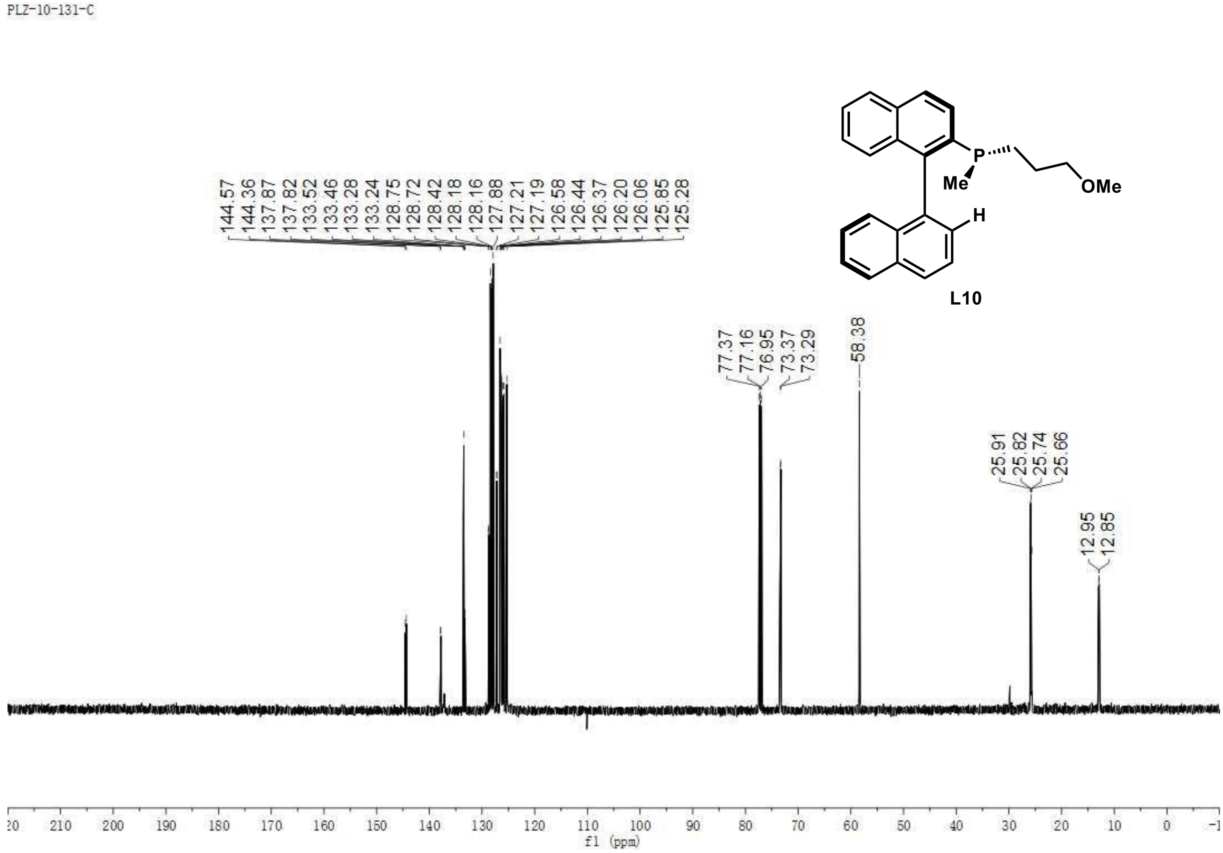

Supplementary Fig. 324.^{13}C NMR spectrum of **L10**. The sample has been recorded in 151 MHz, $CDCl_3$ at 25 ℃.

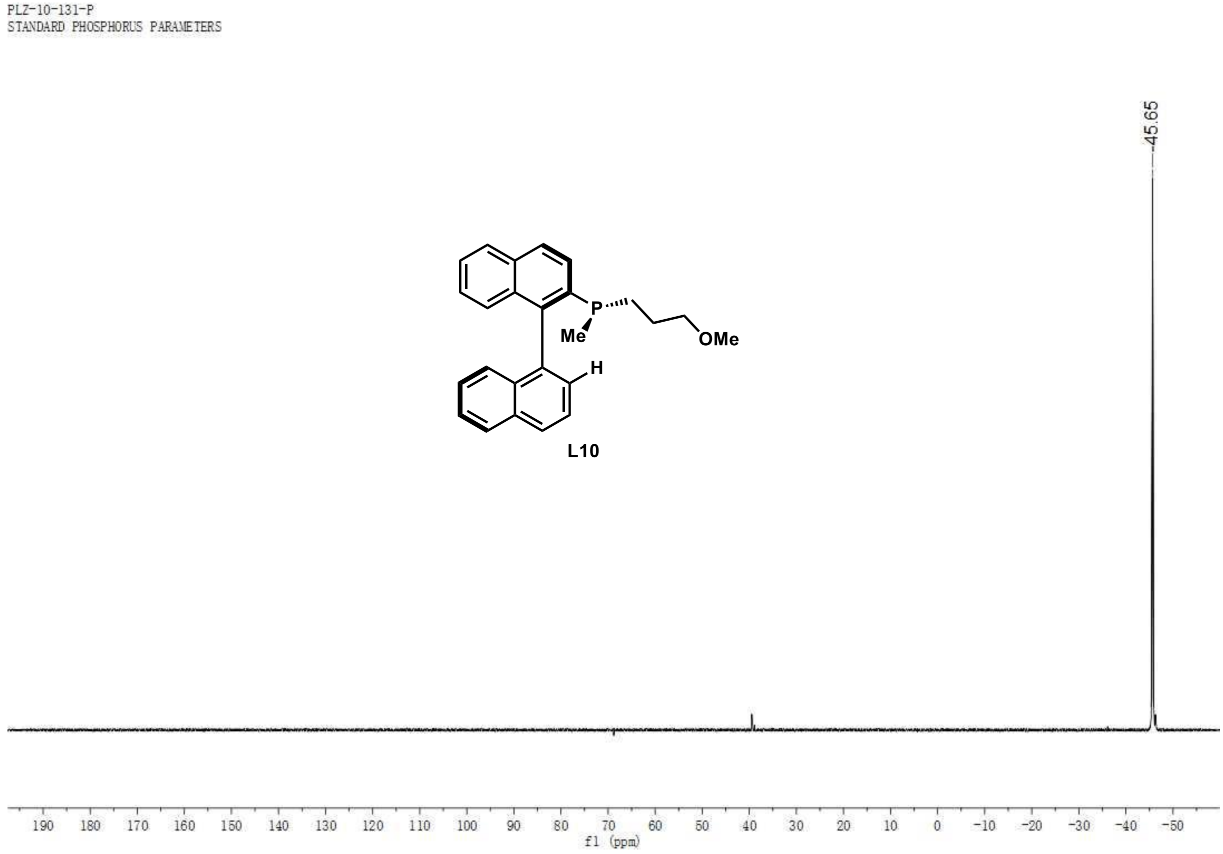

Supplementary Fig. 325.^{31}P NMR spectrum of **L10**. The sample has been recorded in 243 MHz, $CDCl_3$ at 25 ℃.

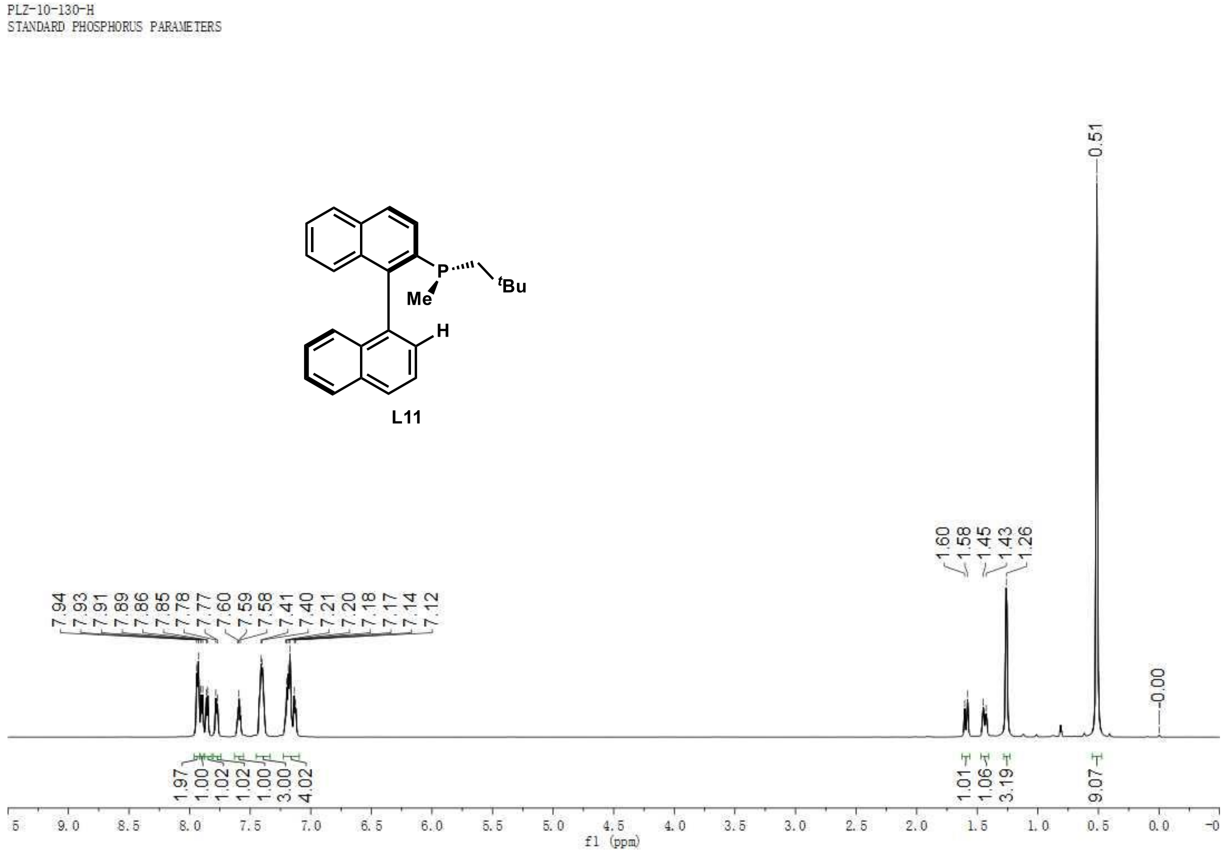

Supplementary Fig. 326. ^{1}H NMR spectrum of **L11**. The sample has been recorded in 600 MHz, $CDCl_3$ at 25 ℃.

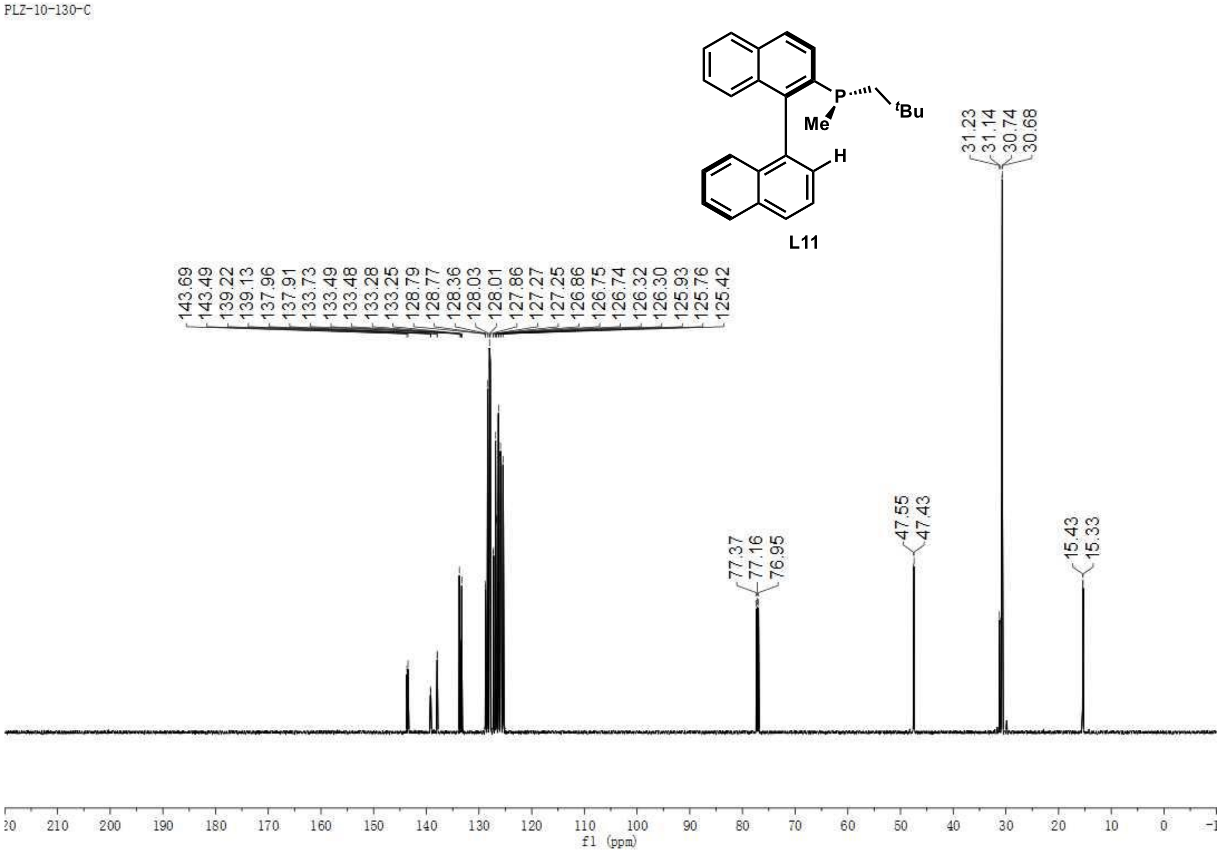

Supplementary Fig. 327.^{13}C NMR spectrum of **L11**. The sample has been recorded in 151 MHz, $CDCl_3$ at 25 ℃.

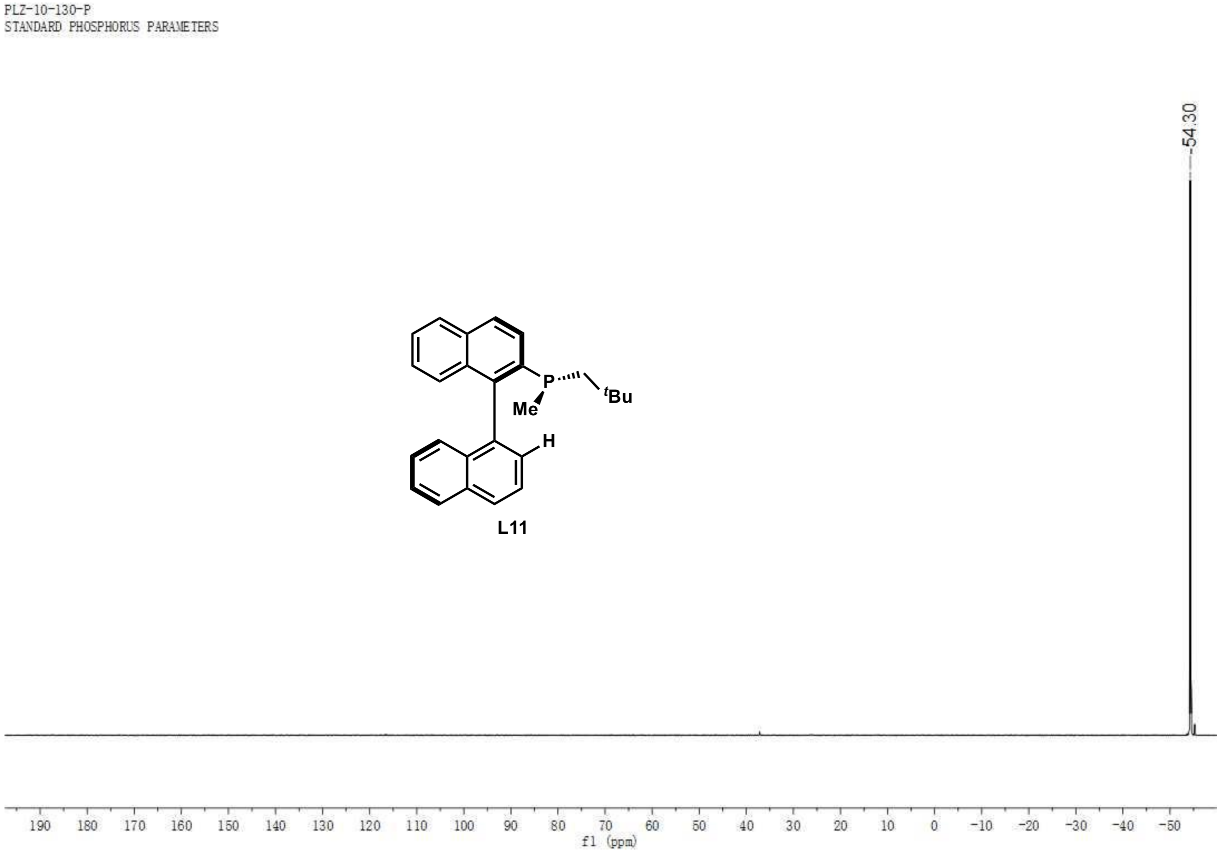

Supplementary Fig. 328.^{31}P NMR spectrum of **L11**. The sample has been recorded in 243 MHz, $CDCl_3$ at 25 ℃.

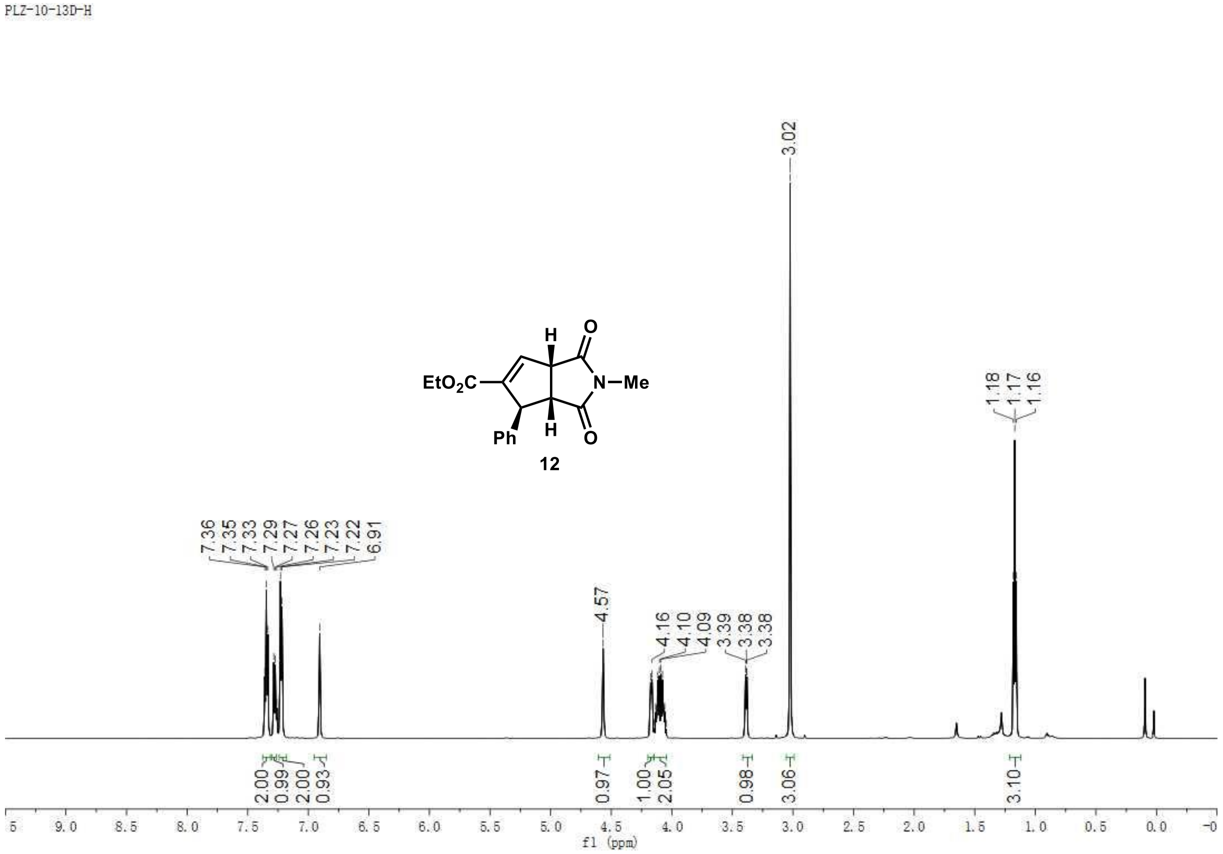

Supplementary Fig. 329. ^{1}H NMR spectrum of **12**. The sample has been recorded in 600 MHz, $CDCl_3$ at 25 ℃.

??????

Project Name lk

Reported by User: Breeze user (Breeze)

SAMPLE INFORMATION

Sample Name:	PLZ-7-15B-ID10%	Acquired By:	Breeze
Sample Type:	Unknown	Date Acquired:	5/25/2022 7:33:12 AM CST
Vial:	1	Acq. Method:	10% 254nm
Injection #:	1	Processed By:	Breeze
Injection Volume:	20.00 ul	Date Processed:	5/25/2022 7:52:16 AM CST
Run Time:	60.00 Minutes	Channel Name:	2998 Ch1 254nm@1.2nm
Sampling Rate:	10.00 per sec	Channel Desc.:	2998 Ch1 254nm@1.2nm
		Sample Set Name	

Sample Values Used in Calculation:

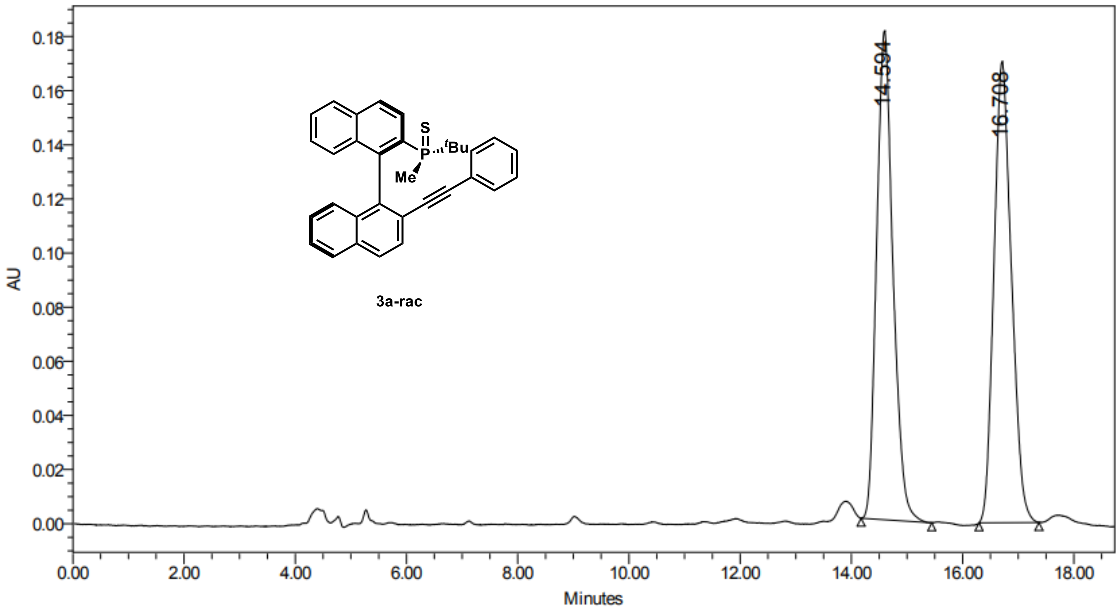

	RT (min)	Peak Type	Area (µV*sec)	% Area	Height (µV)	% Height	Integration Type	Points Across Peak	Start Time (min)	End Time (min)
1	14.594	Unknown	3683379	49.75	180610	51.46	bb	762	14.175	15.445
2	16.708	Unknown	3720841	50.25	170349	48.54	bb	647	16.295	17.373

Report Method: Detailed Individual Report

Page: 1 of 2

Printed: 5/25/2022 8:13:10 AM PRC

Supplementary Fig. 330. HPLC of **3a-rac.**

??????

Project Name lk

Reported by User: Breeze user (Breeze)

SAMPLE INFORMATION

Sample Name:	PLZ-7-48F-ID10%	Acquired By:	Breeze
Sample Type:	Unknown	Date Acquired:	5/25/2022 7:53:27 AM CST
Vial:	1	Acq. Method:	10% 254nm
Injection #:	2	Processed By:	Breeze
Injection Volume:	20.00 ul	Date Processed:	5/25/2022 8:12:56 AM CST
Run Time:	60.00 Minutes	Channel Name:	2998 Ch1 254nm@1.2nm
Sampling Rate:	10.00 per sec	Channel Desc.:	2998 Ch1 254nm@1.2nm
		Sample Set Name	

Sample Values Used in Calculation:

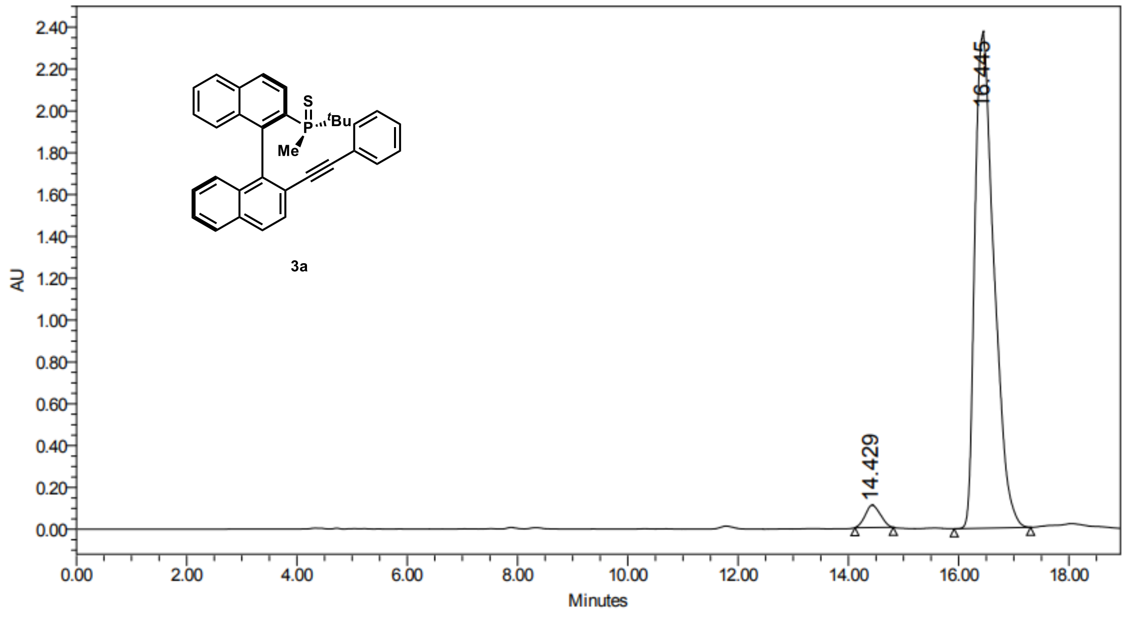

	RT (min)	Peak Type	Area (µV*sec)	% Area	Height (µV)	% Height	Integration Type	Points Across Peak	Start Time (min)
1	14.429	Unknown	2042662	3.46	107539	4.33	bb	417	14.118
2	16.445	Unknown	56927836	96.54	2374563	95.67	bb	831	15.920

Report Method: Detailed Individual Report

Page: 1 of 2

Printed: 5/25/2022 8:14:31 AM PRC

Supplementary Fig. 331. HPLC of **3a.**

??????

Project Name Ik

Reported by User: Breeze user (Breeze)

SAMPLE INFORMATION

Sample Name:	PLZ-6-15B-ID10%	Acquired By:	Breeze
Sample Type:	Unknown	Date Acquired:	7/17/2022 6:45:08 AM CST
Vial:	1	Acq. Method:	10% 254nm
Injection #:	2	Processed By:	Breeze
Injection Volume:	20.00 ul	Date Processed:	7/17/2022 8:53:01 AM CST
Run Time:	60.00 Minutes	Channel Name:	2998 Ch1 254nm@1.2nm
Sampling Rate:	10.00 per sec	Channel Desc.:	2998 Ch1 254nm@1.2nm
		Sample Set Name	

Sample Values
Used in Calculation:

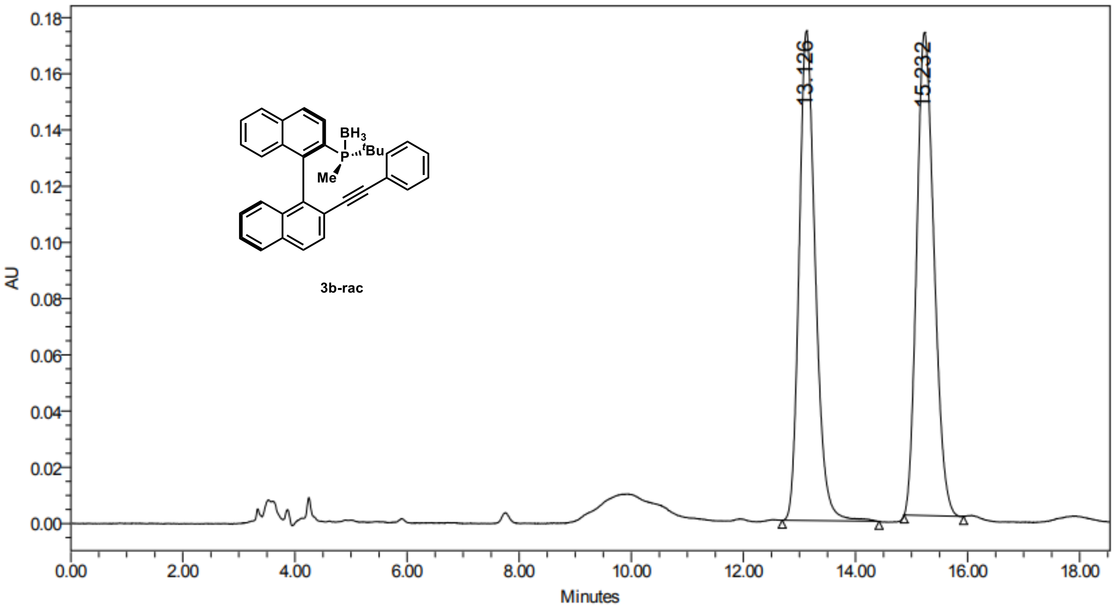

	RT (min)	Peak Type	Area (µV*sec)	% Area	Height (µV)	% Height	Integration Type	Points Across Peak	Start Time (min)	End Time (min)
1	13.126	Unknown	3503472	48.76	174286	50.35	bb	1038	12.692	14.422
2	15.232	Unknown	3681494	51.24	171871	49.65	bb	636	14.868	15.928

Report Method: Detailed Individual Report
Page: 1 of 2

Printed: 7/17/2022
8:53:48 AM PRC

Supplementary Fig. 332. HPLC of **3b-rac.**

??????

Project Name Ik

Reported by User: Breeze user (Breeze)

SAMPLE INFORMATION

Sample Name:	PLZ-8-68D-ID10%	Acquired By:	Breeze
Sample Type:	Unknown	Date Acquired:	7/17/2022 7:13:13 AM CST
Vial:	1	Acq. Method:	10% 254nm
Injection #:	3	Processed By:	Breeze
Injection Volume:	20.00 ul	Date Processed:	7/17/2022 8:53:24 AM CST
Run Time:	60.00 Minutes	Channel Name:	2998 Ch1 254nm@1.2nm
Sampling Rate:	10.00 per sec	Channel Desc.:	2998 Ch1 254nm@1.2nm
		Sample Set Name	

Sample Values
Used in Calculation:

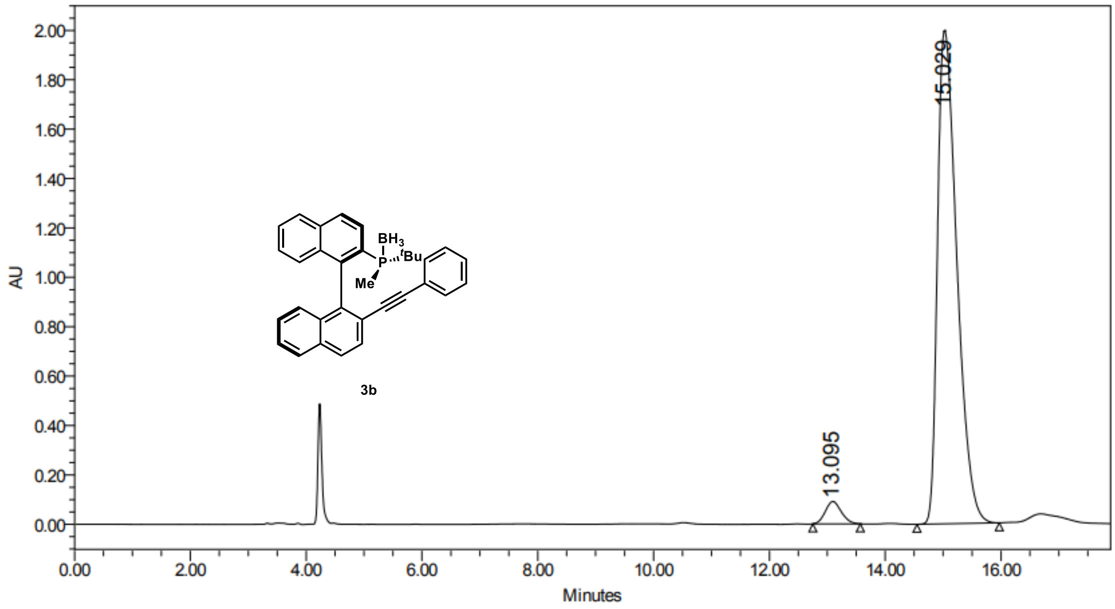

	RT (min)	Peak Type	Area (µV*sec)	% Area	Height (µV)	% Height	Integration Type	Points Across Peak	Start Time (min)
1	13.095	Unknown	1738162	3.61	90504	4.34	bb	493	12.750
2	15.029	Unknown	46461671	96.39	1996889	95.66	bb	852	14.550

Report Method: Detailed Individual Report
Page: 1 of 2

Printed: 7/17/2022
8:55:02 AM PRC

Supplementary Fig. 333. HPLC of **3b.**

??????
Project Name Ik
Reported by User: Breeze user (Breeze)

Breeze 2 HPLC System

SAMPLE INFORMATION

Sample Name:	PLZ-7-94A-IA5%	Acquired By:	Breeze
Sample Type:	Unknown	Date Acquired:	5/22/2022 4:25:12 AM CST
Vial:	1	Acq. Method:	5% 254nm
Injection #:	1	Processed By:	Breeze
Injection Volume:	20.00 ul	Date Processed:	5/22/2022 4:47:47 AM CST
Run Time:	60.00 Minutes	Channel Name:	2998 Ch1 254nm@1.2nm
Sampling Rate:	10.00 per sec	Channel Desc.:	2998 Ch1 254nm@1.2nm
		Sample Set Name	

Sample Values
Used in Calculation:

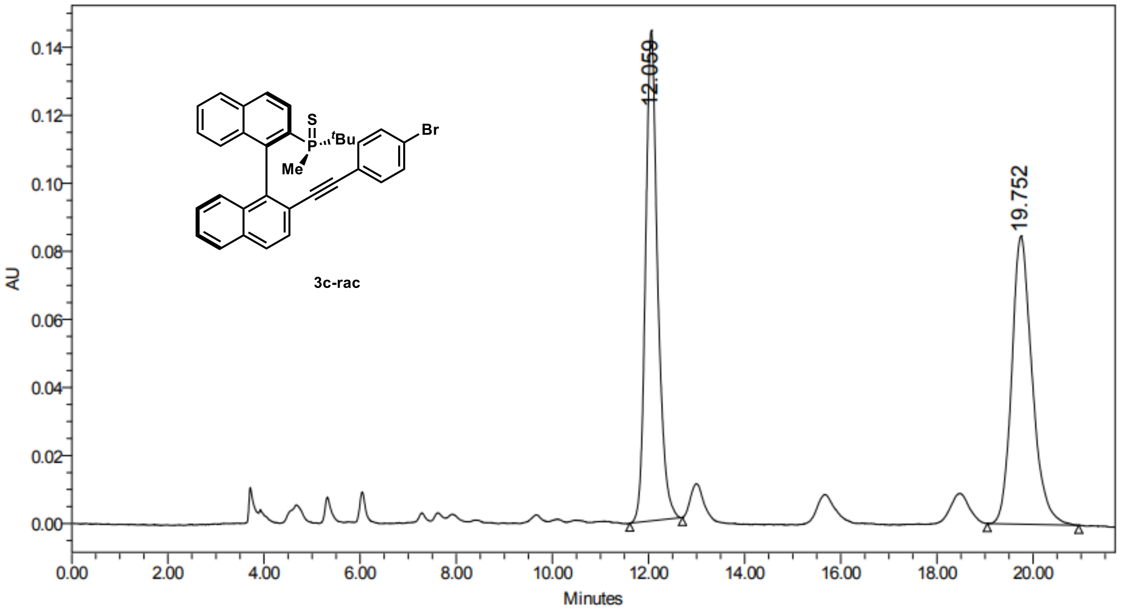

	RT (min)	Peak Type	Area (μV*sec)	% Area	Height (μV)	% Height	Integration Type	Points Across Peak	Start Time (min)	End Time (min)
1	12.059	Unknown	2525117	50.29	144173	63.00	bb	657	11.612	12.707
2	19.752	Unknown	2495899	49.71	84679	37.00	bb	1144	19.047	20.953

Report Method: Detailed Individual Report
Page: 1 of 2

Printed: 5/22/2022
4:48:07 AM PRC

Supplementary Fig. 334. HPLC of **3c-rac.**

??????
Project Name   lk
Reported by User: Breeze user (Breeze)

SAMPLE INFORMATION

Sample Name: PLZ-7-97A-IA5%
Sample Type: Unknown
Vial: 1
Injection #: 2
Injection Volume: 20.00 ul
Run Time: 60.00 Minutes
Sampling Rate: 10.00 per sec

Sample Values
Used in Calculation:

Acquired By: Breeze
Date Acquired: 5/22/2022 4:47:16 AM CST
Acq. Method: 5% 254nm
Processed By: Breeze
Date Processed: 5/22/2022 5:11:45 AM CST
Channel Name: 2998 Ch1 254nm@1.2nm
Channel Desc.: 2998 Ch1 254nm@1.2nm
Sample Set Name

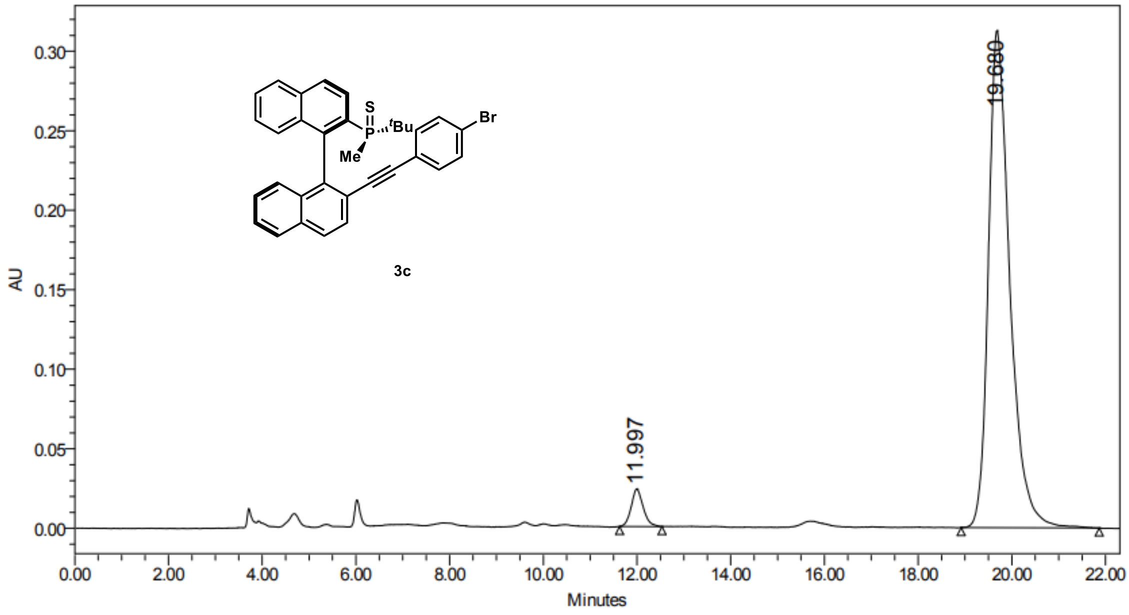

	RT (min)	Peak Type	Area (µV*sec)	% Area	Height (µV)	% Height	Integration Type	Points Across Peak	Start Time (min)	End Time (min)
1	11.997	Unknown	418207	4.04	23594	7.02	bb	544	11.627	12.533
2	19.680	Unknown	9925594	95.96	312690	92.98	bb	1769	18.917	21.865

Report Method: Detailed Individual Report
Page: 1 of 2

Printed: 5/22/2022
5:12:08 AM PRC

Supplementary Fig. 335. HPLC of **3c.**

??????

Project Name Ik

Reported by User: Breeze user (Breeze)

SAMPLE INFORMATION

Sample Name:	PLZ-7-94B-IA10%	Acquired By:	Breeze
Sample Type:	Unknown	Date Acquired:	5/22/2022 1:26:09 AM CST
Vial:	1	Acq. Method:	10% 254nm
Injection #:	5	Processed By:	Breeze
Injection Volume:	20.00 ul	Date Processed:	5/22/2022 1:51:07 AM CST
Run Time:	60.00 Minutes	Channel Name:	2998 Ch1 254nm@1.2nm
Sampling Rate:	10.00 per sec	Channel Desc.:	2998 Ch1 254nm@1.2nm
		Sample Set Name	

Sample Values Used in Calculation:

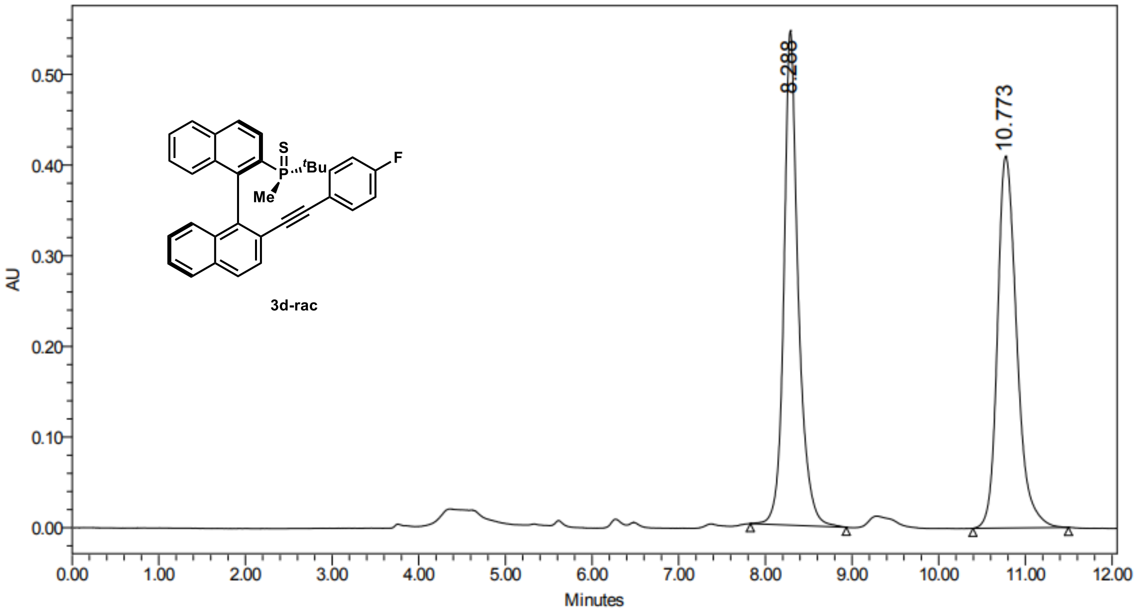

	RT (min)	Peak Type	Area (µV*sec)	% Area	Height (µV)	% Height	Integration Type	Points Across Peak	Start Time (min)	End Time (min)
1	8.288	Unknown	6279346	49.75	545471	57.09	bb	665	7.827	8.935
2	10.773	Unknown	6341975	50.25	410028	42.91	bb	661	10.395	11.497

Report Method: Detailed Individual Report

Page: 1 of 2

Printed: 5/22/2022 1:51:21 AM PRC

Supplementary Fig. 336. HPLC of **3d-rac.**

??????

Project Name Ik

Reported by User: Breeze user (Breeze)

SAMPLE INFORMATION

Sample Name:	PLZ-7-97B-IA10%	Acquired By:	Breeze
Sample Type:	Unknown	Date Acquired:	5/22/2022 1:38:45 AM CST
Vial:	1	Acq. Method:	10% 254nm
Injection #:	6	Processed By:	Breeze
Injection Volume:	20.00 ul	Date Processed:	5/22/2022 1:53:48 AM CST
Run Time:	60.00 Minutes	Channel Name:	2998 Ch1 254nm@1.2nm
Sampling Rate:	10.00 per sec	Channel Desc.:	2998 Ch1 254nm@1.2nm
		Sample Set Name	

Sample Values
Used in Calculation:

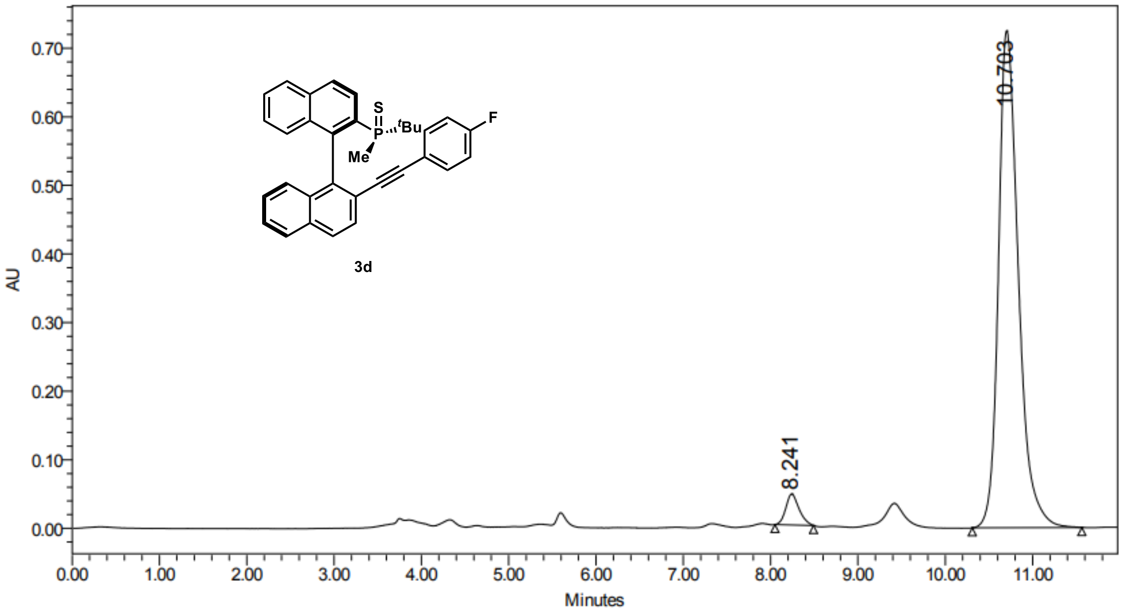

	RT (min)	Peak Type	Area (μV*sec)	% Area	Height (μV)	% Height	Integration Type	Points Across Peak	Start Time (min)	End Time (min)
1	8.241	Unknown	473398	3.96	45168	5.87	bb	264	8.050	8.490
2	10.703	Unknown	11486794	96.04	724463	94.13	bb	753	10.310	11.565

Report Method: Detailed Individual Report
Page: 1 of 2

Printed: 5/22/2022
1:54:00 AM PRC

Supplementary Fig. 337. HPLC of **3d.**

??????

Project Name lk

Reported by User: Breeze user (Breeze)

Breeze 2 HPLC System

SAMPLE INFORMATION

Sample Name:	PLZ-7-37A-IA10%	Acquired By:	Breeze
Sample Type:	Unknown	Date Acquired:	5/22/2022 1:11:38 AM CST
Vial:	1	Acq. Method:	10% 254nm
Injection #:	4	Processed By:	Breeze
Injection Volume:	20.00 ul	Date Processed:	5/22/2022 1:27:09 AM CST
Run Time:	60.00 Minutes	Channel Name:	2998 Ch1 254nm@1.2nm
Sampling Rate:	10.00 per sec	Channel Desc.:	2998 Ch1 254nm@1.2nm
		Sample Set Name	

Sample Values
Used in Calculation:

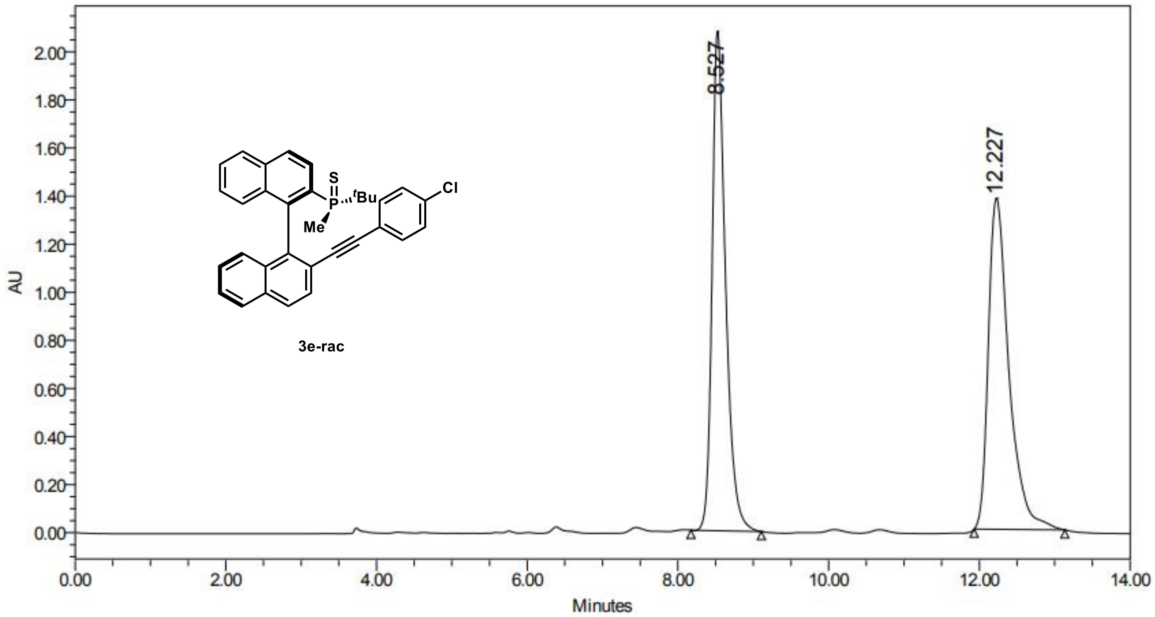

	RT (min)	Peak Type	Area (µV*sec)	% Area	Height (µV)	% Height	Integration Type	Points Across Peak	Start Time (min)
1	8.527	Unknown	25766888	49.63	2078680	60.13	bb	560	8.173
2	12.227	Unknown	26155846	50.37	1378452	39.87	bb	721	11.932

Report Method: Detailed Individual Report
Page: 1 of 2

Printed: 5/22/2022
1:27:23 AM PRC

Supplementary Fig. 338. HPLC of **3e-rac.**

??????

Project Name Ik

Reported by User: Breeze user (Breeze)

SAMPLE INFORMATION

Sample Name:	PLZ-7-109-IA10%	Acquired By:	Breeze
Sample Type:	Unknown	Date Acquired:	5/22/2022 12:57:01 AM CST
Vial:	1	Acq. Method:	10% 254nm
Injection #:	3	Processed By:	Breeze
Injection Volume:	20.00 ul	Date Processed:	5/22/2022 1:29:21 AM CST
Run Time:	60.00 Minutes	Channel Name:	2998 Ch1 254nm@1.2nm
Sampling Rate:	10.00 per sec	Channel Desc.:	2998 Ch1 254nm@1.2nm
		Sample Set Name	

Sample Values Used in Calculation:

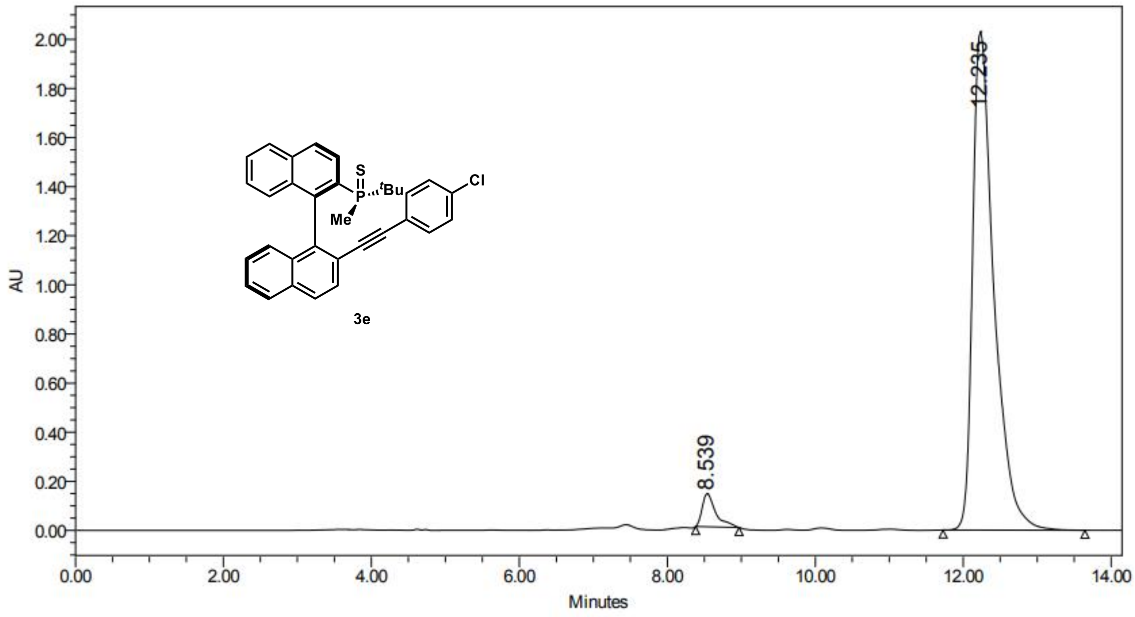

	RT (min)	Peak Type	Area (μV*sec)	% Area	Height (μV)	% Height	Integration Type	Points Across Peak	Start Time (min)
1	8.539	Unknown	1720788	4.07	135013	6.23	bb	352	8.383
2	12.235	Unknown	40573571	95.93	2030949	93.77	bb	1151	11.727

Report Method: Detailed Individual Report

Page: 1 of 2

Printed: 5/22/2022 1:30:52 AM PRC

Supplementary Fig. 339. HPLC of **3e.**

??????

Project Name Ik

Reported by User: Breeze user (Breeze)

Breeze 2 HPLC System

SAMPLE INFORMATION

Sample Name:	PLZ-7-15-IA10%	Acquired By:	Breeze
Sample Type:	Unknown	Date Acquired:	5/19/2022 10:33:25 AM CST
Vial:	1	Acq. Method:	10% 254nm
Injection #:	8	Processed By:	Breeze
Injection Volume:	20.00 ul	Date Processed:	5/19/2022 10:49:18 AM CST
Run Time:	60.00 Minutes	Channel Name:	2998 Ch1 254nm@1.2nm
Sampling Rate:	10.00 per sec	Channel Desc.:	2998 Ch1 254nm@1.2nm
		Sample Set Name	

Sample Values
Used in Calculation:

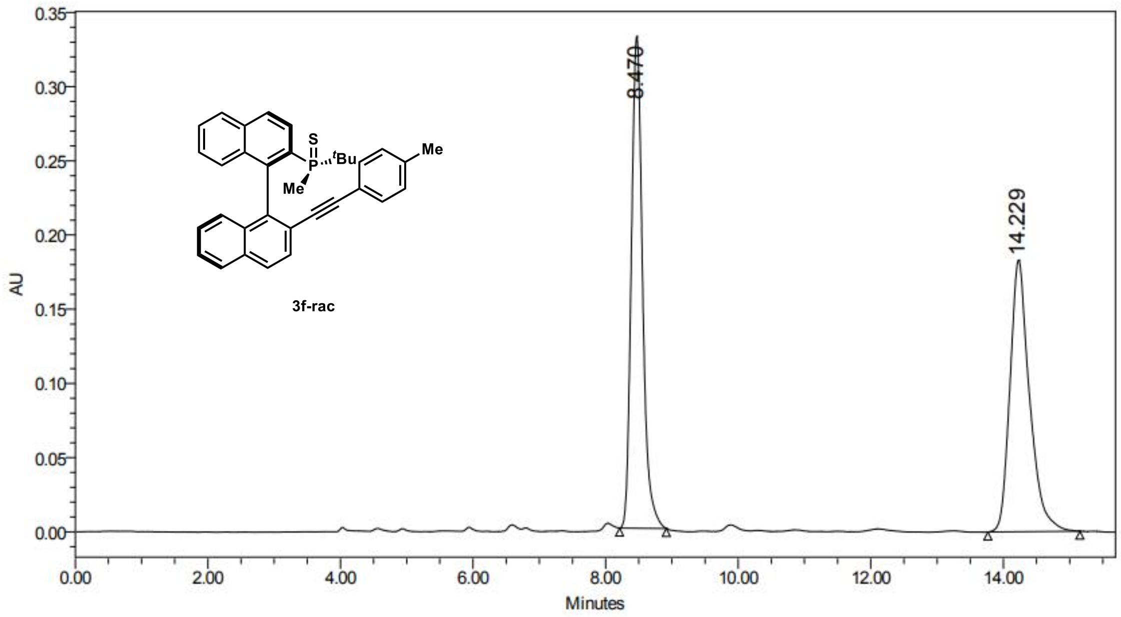

	RT (min)	Peak Type	Area (µV*sec)	% Area	Height (µV)	% Height	Integration Type	Points Across Peak	Start Time (min)	End Time (min)
1	8.470	Unknown	3895256	50.93	331469	64.42	bb	422	8.212	8.915
2	14.229	Unknown	3753228	49.07	183086	35.58	bb	832	13.767	15.153

Report Method: Detailed Individual Report | Printed: 5/19/2022
Page: 1 of 2 | 11:05:28 AM PRC

Supplementary Fig. 340. HPLC of **3f-rac.**

??????
Project Name Ik
Reported by User: Breeze user (Breeze)

SAMPLE INFORMATION

Sample Name:	PLZ-7-98C-IA10%	Acquired By:	Breeze
Sample Type:	Unknown	Date Acquired:	5/19/2022 10:50:27 AM CST
Vial:	1	Acq. Method:	10% 254nm
Injection #:	9	Processed By:	Breeze
Injection Volume:	20.00 ul	Date Processed:	5/19/2022 11:06:41 AM CST
Run Time:	60.00 Minutes	Channel Name:	2998 Ch1 254nm@1.2nm
Sampling Rate:	10.00 per sec	Channel Desc.:	2998 Ch1 254nm@1.2nm
		Sample Set Name	

Sample Values
Used in Calculation:

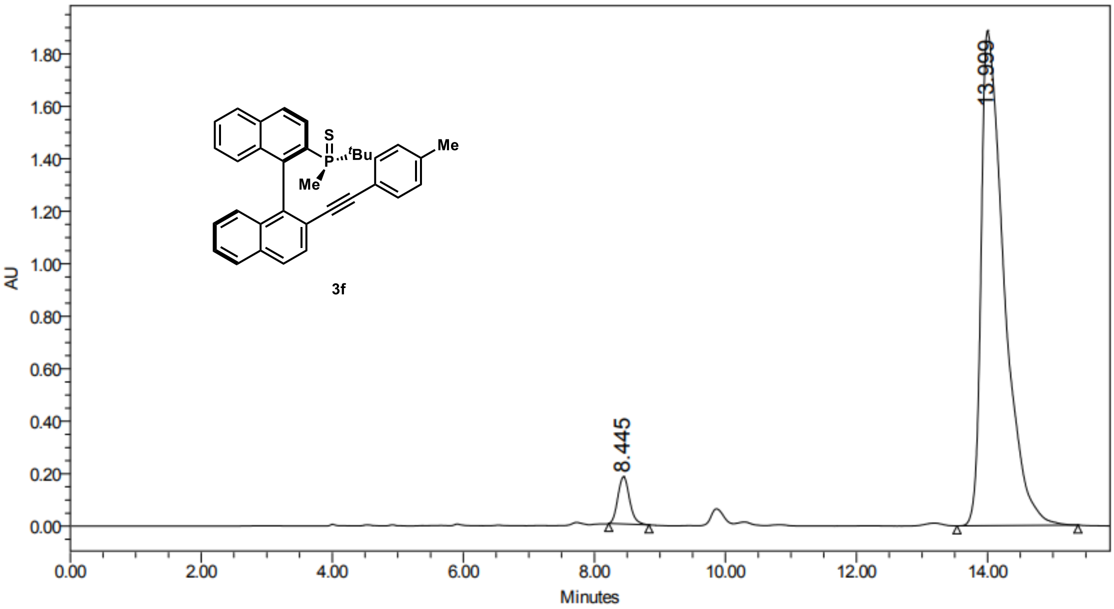

	RT (min)	Peak Type	Area (µV*sec)	% Area	Height (µV)	% Height	Integration Type	Points Across Peak	Start Time (min)
1	8.445	Unknown	2240413	4.65	180373	8.72	bb	369	8.218
2	13.999	Unknown	45969702	95.35	1887014	91.28	bb	1107	13.535

Report Method: Detailed Individual Report
Page: 1 of 2

Printed: 5/19/2022
11:07:05 AM PRC

Supplementary Fig. 341. HPLC of **3f.**

SHIMADZU LabSolutions 分析报告

<样品信息>

样品名	: PLZ		
样品ID	: PLZ-220915		
数据文件名	: PLZ-10-127-IA10%12.lcd		
方法文件名	: 322.lcm		
批处理文件名	:		
样品瓶号	: 1-1	样品类型	: 未知
进样体积	: 1 uL		
分析日期	: 2023/4/15 0:04:32	分析者	: System Administrator
处理日期	: 2023/4/15 0:37:04	处理者	: System Administrator

<色谱图>

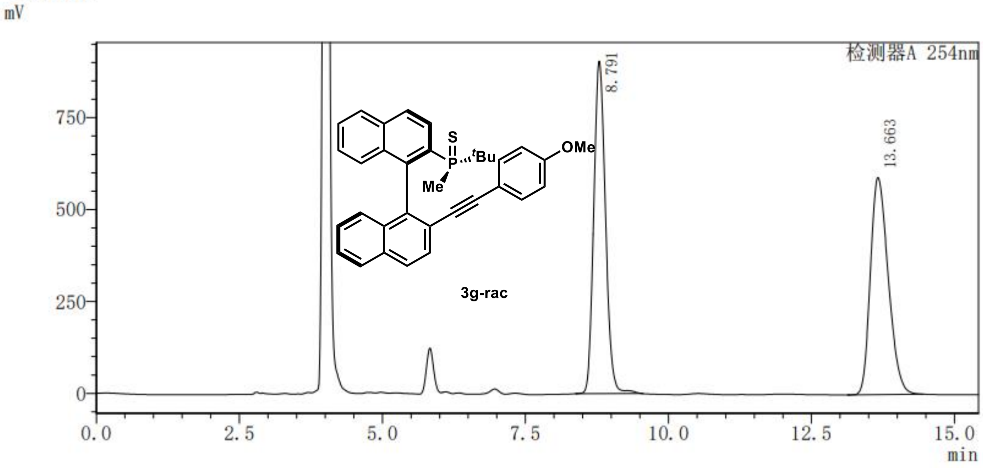

<峰表>

检测器A 254nm

峰号	保留时间	面积	高度	浓度	浓度单位	标记	化合物名
1	8.791	12897858	904193	50.173		M	
2	13.663	12809066	590168	49.827		M	
总计		25706924	1494361				

C:\LabSolutions\Sample\PLZ-10-127-IA10%12.lcd

Supplementary Fig. 342. HPLC of **3g-rac.**

SHIMADZU LabSolutions 分析报告

<样品信息>

样品名	: PLZ		
样品ID	: PLZ-220915		
数据文件名	: PLZ-7-125A-IA10%14.lcd		
方法文件名	: 322.lcm		
批处理文件名	:		
样品瓶号	: 1-1	样品类型	: 未知
进样体积	: 1 uL		
分析日期	: 2023/4/15 0:41:15	分析者	: System Administrator
处理日期	: 2023/4/15 0:58:34	处理者	: System Administrator

<色谱图>

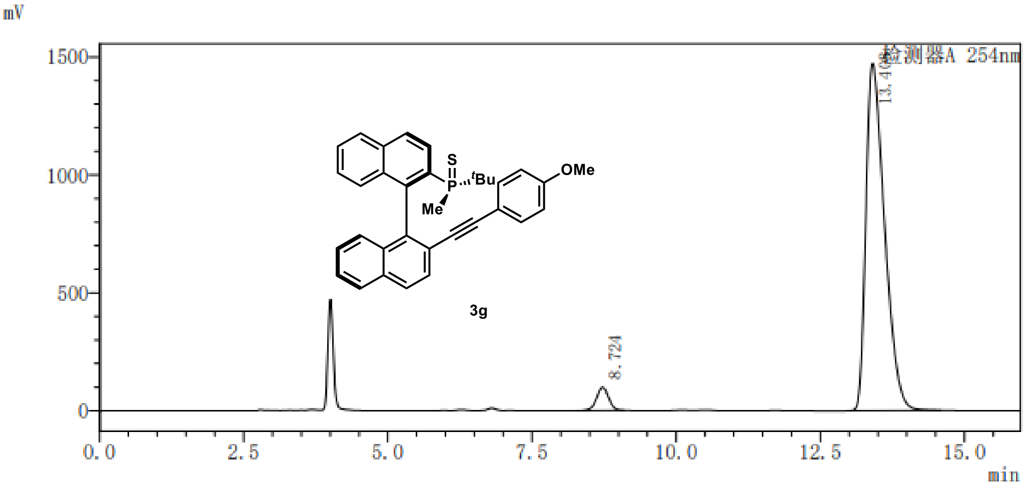

<峰表>

检测器A 254nm

峰号	保留时间	面积	高度	浓度	浓度单位	标记	化合物名
1	8.724	1445665	98777	4.170		M	
2	13.408	33219901	1472990	95.830		M	
总计		34665566	1571767				

C:\LabSolutions\Sample\PLZ-7-125A-IA10%14.lcd

Supplementary Fig. 343. HPLC of **3g.**

??????

Project Name lk

Reported by User: Breeze user (Breeze)

SAMPLE INFORMATION

Sample Name:	PLZ-7-7D-IA10%	Acquired By:	Breeze
Sample Type:	Unknown	Date Acquired:	5/18/2022 6:01:14 AM CST
Vial:	1	Acq. Method:	10% 254nm
Injection #:	5	Processed By:	Breeze
Injection Volume:	20.00 ul	Date Processed:	5/19/2022 8:35:28 AM CST
Run Time:	60.00 Minutes	Channel Name:	2998 Ch1 254nm@1.2nm
Sampling Rate:	10.00 per sec	Channel Desc.:	2998 Ch1 254nm@1.2nm
		Sample Set Name	

Sample Values Used in Calculation:

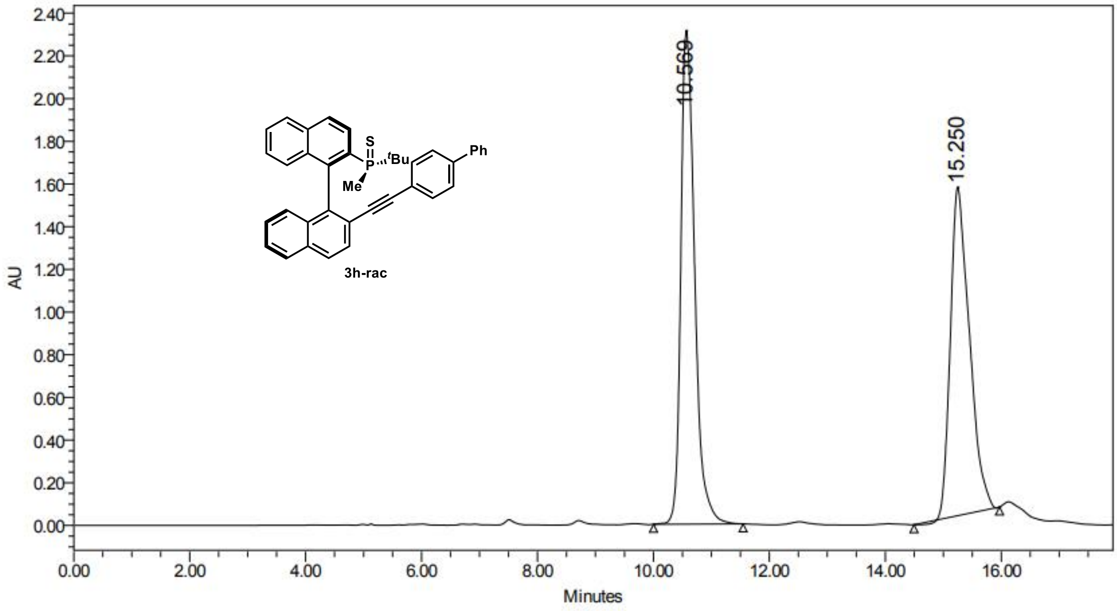

	RT (min)	Peak Type	Area (µV*sec)	% Area	Height (µV)	% Height	Integration Type	Points Across Peak	Start Time (min)
1	10.569	Unknown	38355178	52.06	2312595	60.04	bb	927	10.002
2	15.250	Unknown	35315961	47.94	1539129	39.96	bb	885	14.495

Report Method: Detailed Individual Report

Page: 1 of 2

Printed: 5/19/2022 8:36:22 AM PRC

Supplementary Fig. 344. HPLC of **3h-rac.**

??????

Project Name lk

Reported by User: Breeze user (Breeze)

SAMPLE INFORMATION

Sample Name:	PLZ-7-105B-IA10%	Acquired By:	Breeze
Sample Type:	Unknown	Date Acquired:	5/18/2022 6:20:49 AM CST
Vial:	1	Acq. Method:	10% 254nm
Injection #:	6	Processed By:	Breeze
Injection Volume:	20.00 ul	Date Processed:	5/19/2022 8:35:46 AM CST
Run Time:	60.00 Minutes	Channel Name:	2998 Ch1 254nm@1.2nm
Sampling Rate:	10.00 per sec	Channel Desc.:	2998 Ch1 254nm@1.2nm
		Sample Set Name	

Sample Values
Used in Calculation:

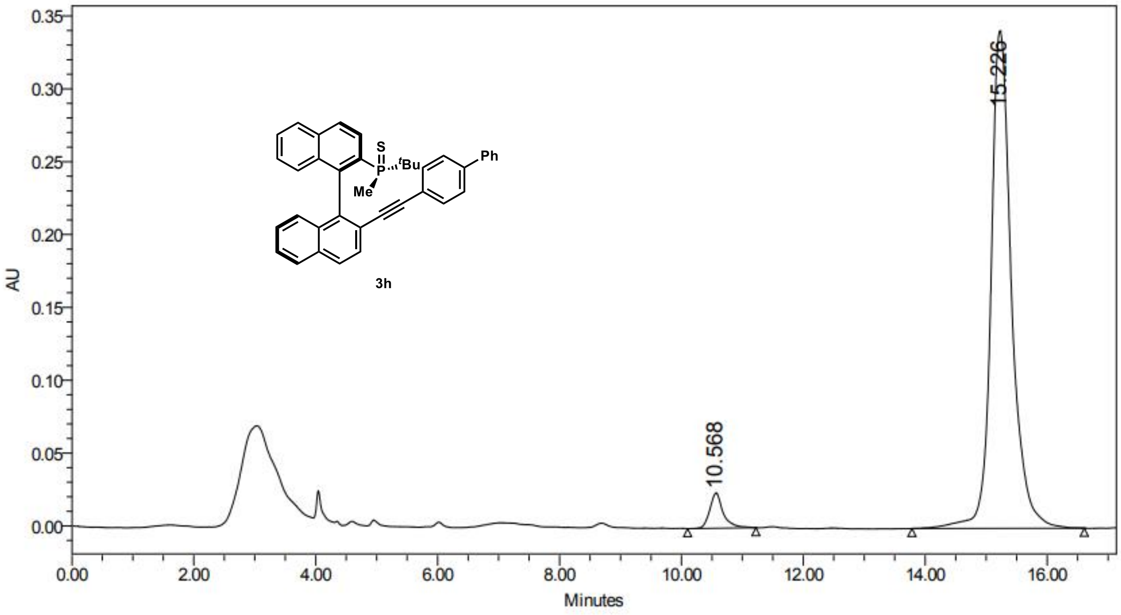

	RT (min)	Peak Type	Area (µV*sec)	% Area	Height (µV)	% Height	Integration Type	Points Across Peak	Start Time (min)	End Time (min)
1	10.568	Unknown	368668	4.40	24132	6.60	bb	673	10.100	11.222
2	15.226	Unknown	8019307	95.60	341426	93.40	bb	1697	13.782	16.610

Report Method: Detailed Individual Report
Page: 1 of 2

Printed: 5/19/2022
8:36:47 AM PRC

Supplementary Fig. 345. HPLC of **3h.**

??????
Project Name lk
Reported by User: Breeze user (Breeze)

Breeze 2 HPLC System

SAMPLE INFORMATION

Sample Name:	PLZ-7-44A-IA10%	Acquired By:	Breeze
Sample Type:	Unknown	Date Acquired:	5/20/2022 7:04:32 AM CST
Vial:	1	Acq. Method:	30% 254nm
Injection #:	1	Processed By:	Breeze
Injection Volume:	20.00 ul	Date Processed:	5/20/2022 7:19:38 AM CST
Run Time:	60.00 Minutes	Channel Name:	2998 Ch1 254nm@1.2nm
Sampling Rate:	10.00 per sec	Channel Desc.:	2998 Ch1 254nm@1.2nm
		Sample Set Name	

Sample Values
Used in Calculation:

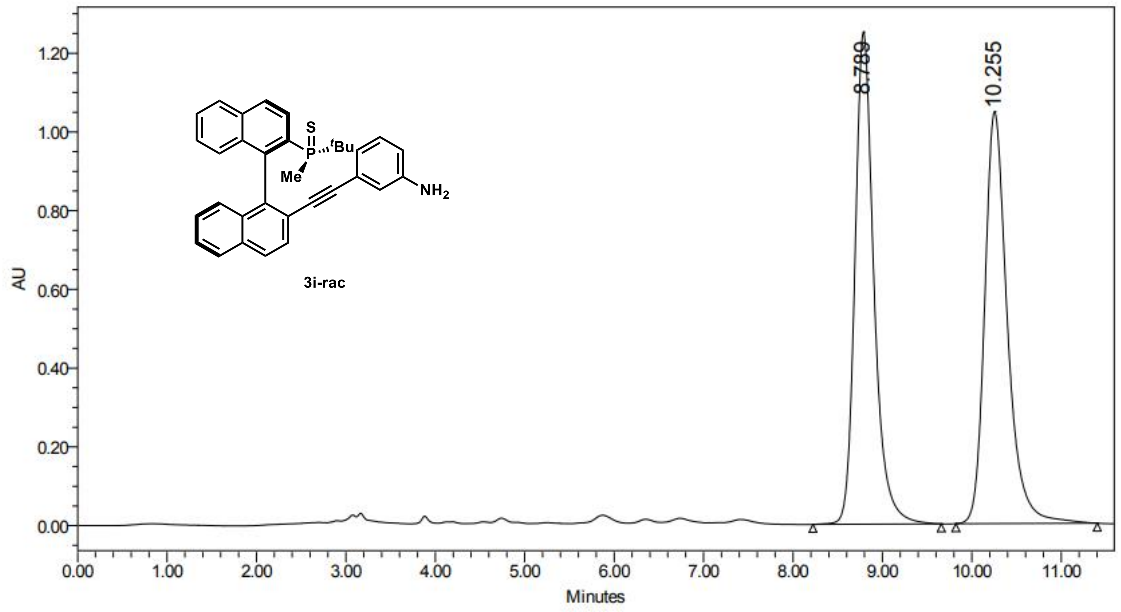

	RT (min)	Peak Type	Area (μV*sec)	% Area	Height (μV)	% Height	Integration Type	Points Across Peak	Start Time (min)
1	8.789	Unknown	18446668	49.97	1250811	54.45	bb	861	8.223
2	10.255	Unknown	18465963	50.03	1046197	45.55	bb	949	9.822

Report Method: Detailed Individual Report
Page: 1 of 2
Printed: 5/20/2022 7:20:00 AM PRC

Supplementary Fig. 346. HPLC of **3i-rac.**

??????
Project Name Ik
Reported by User: Breeze user (Breeze)

SAMPLE INFORMATION

Sample Name:	PLZ-7-97F-IA30%	Acquired By:	Breeze
Sample Type:	Unknown	Date Acquired:	5/20/2022 7:18:58 AM CST
Vial:	1	Acq. Method:	30% 254nm
Injection #:	2	Processed By:	Breeze
Injection Volume:	20.00 ul	Date Processed:	5/20/2022 7:40:33 AM CST
Run Time:	60.00 Minutes	Channel Name:	2998 Ch1 254nm@1.2nm
Sampling Rate:	10.00 per sec	Channel Desc.:	2998 Ch1 254nm@1.2nm
		Sample Set Name	

Sample Values
Used in Calculation:

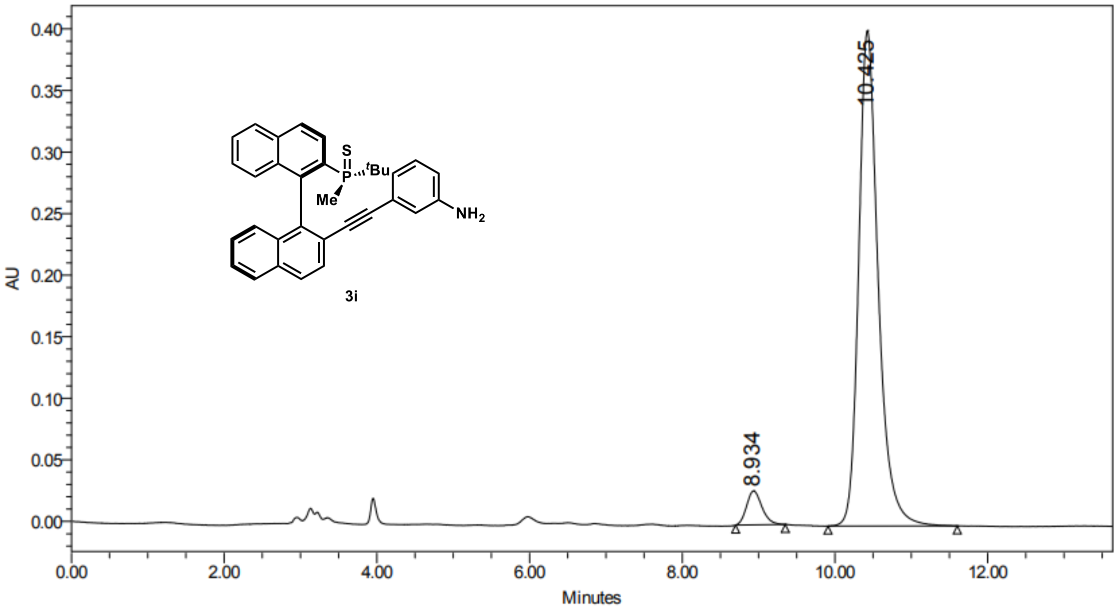

	RT (min)	Peak Type	Area (µV*sec)	% Area	Height (µV)	% Height	Integration Type	Points Across Peak	Start Time (min)	End Time (min)
1	8.934	Unknown	384655	5.10	27583	6.41	bb	390	8.700	9.350
2	10.425	Unknown	7158826	94.90	402420	93.59	bb	1015	9.910	11.602

Report Method: Detailed Individual Report
Page: 1 of 2

Printed: 5/20/2022
7:40:58 AM PRC

Supplementary Fig. 347. HPLC of **3i.**

??????

Project Name lk

Reported by User: Breeze user (Breeze)

SAMPLE INFORMATION

Sample Name:	PLZ-7-146E-IA10%	Acquired By:	Breeze
Sample Type:	Unknown	Date Acquired:	7/4/2022 7:32:58 AM CST
Vial:	1	Acq. Method:	10% 254nm
Injection #:	7	Processed By:	Breeze
Injection Volume:	20.00 ul	Date Processed:	7/4/2022 7:59:42 AM CST
Run Time:	60.00 Minutes	Channel Name:	2998 Ch1 254nm@1.2nm
Sampling Rate:	10.00 per sec	Channel Desc.:	2998 Ch1 254nm@1.2nm
		Sample Set Name	

Sample Values Used in Calculation:

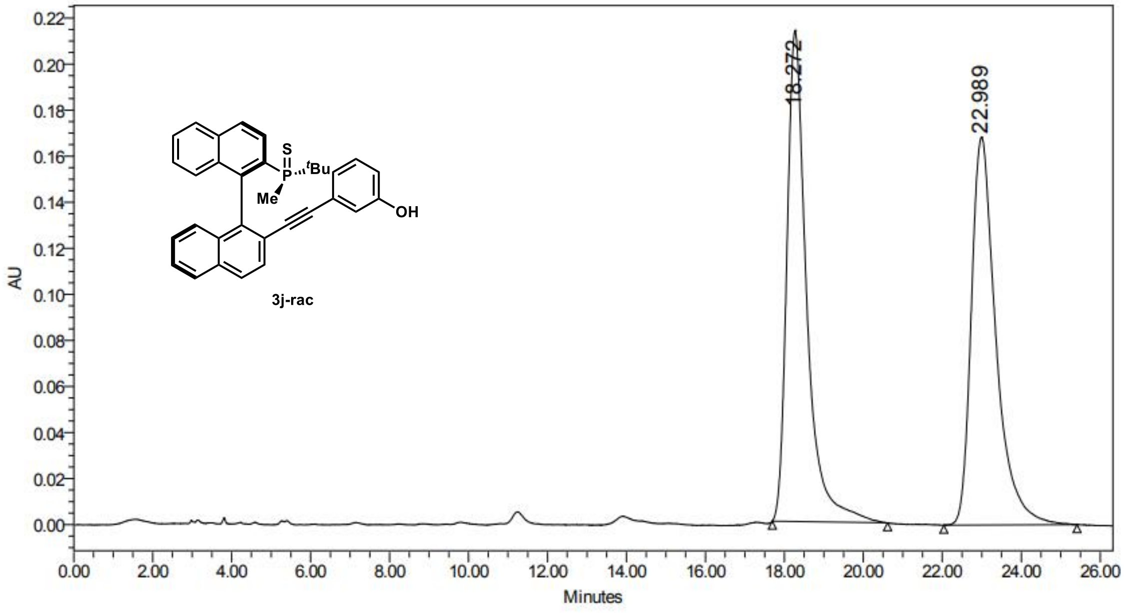

	RT (min)	Peak Type	Area (µV*sec)	% Area	Height (µV)	% Height	Integration Type	Points Across Peak	Start Time (min)	End Time (min)
1	18.272	Unknown	7409073	50.31	213242	55.86	bb	1754	17.693	20.617
2	22.989	Unknown	7316955	49.69	168468	44.14	bb	2026	22.037	25.413

Report Method: Detailed Individual Report

Page: 1 of 2

Printed: 7/4/2022 8:43:42 AM PRC

Supplementary Fig. 348. HPLC of **3j-rac.**

??????
Project Name lk
Reported by User: Breeze user (Breeze)

SAMPLE INFORMATION

Sample Name: PLZ-7-162A-IA10%
Sample Type: Unknown
Vial: 1
Injection #: 9
Injection Volume: 20.00 ul
Run Time: 60.00 Minutes
Sampling Rate: 10.00 per sec

Acquired By: Breeze
Date Acquired: 7/4/2022 8:15:56 AM CST
Acq. Method: 10% 254nm
Processed By: Breeze
Date Processed: 7/4/2022 8:45:19 AM CST
Channel Name: 2998 Ch1 254nm@1.2nm
Channel Desc.: 2998 Ch1 254nm@1.2nm
Sample Set Name

Sample Values
Used in Calculation:

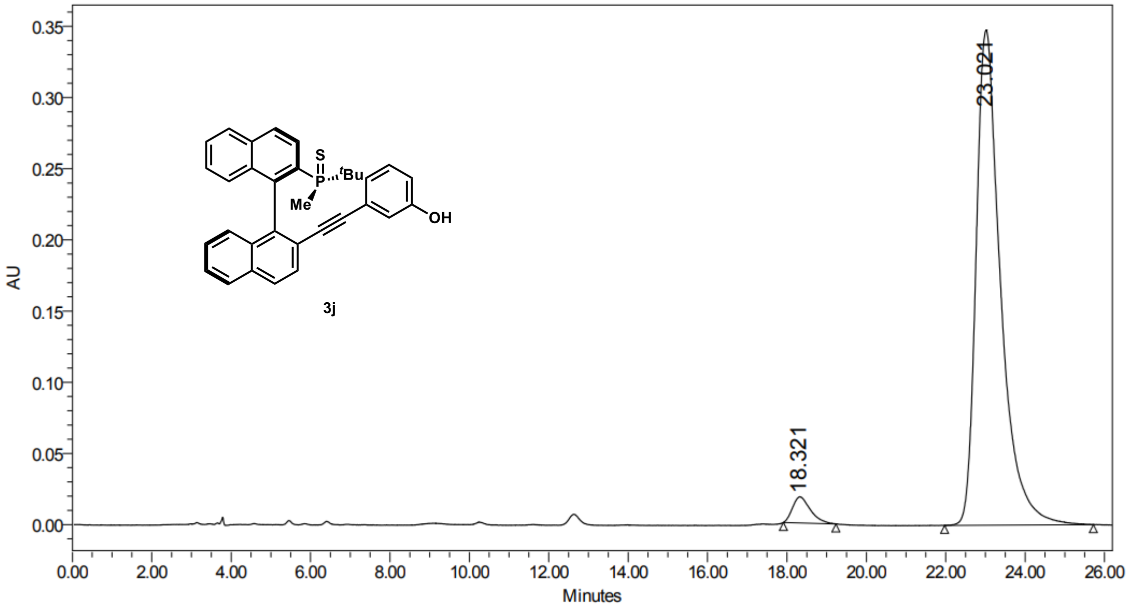

	RT (min)	Peak Type	Area (µV*sec)	% Area	Height (µV)	% Height	Integration Type	Points Across Peak	Start Time (min)	End Time (min)
1	18.321	Unknown	571956	3.70	18357	5.01	bb	792	17.913	19.233
2	23.021	Unknown	14896464	96.30	347888	94.99	bb	2250	21.970	25.720

Report Method: Detailed Individual Report
Page: 1 of 2
Printed: 7/4/2022 8:45:33 AM PRC

Supplementary Fig. 349. HPLC of **3j.**

??????

Project Name Ik

Reported by User: Breeze user (Breeze)

SAMPLE INFORMATION

Sample Name:	PLZ-7-37C-IA10%	Acquired By:	Breeze
Sample Type:	Unknown	Date Acquired:	5/28/2022 12:55:01 PM CST
Vial:	1	Acq. Method:	10% 254nm
Injection #:	2	Processed By:	Breeze
Injection Volume:	20.00 ul	Date Processed:	5/28/2022 1:06:40 PM CST
Run Time:	60.00 Minutes	Channel Name:	2998 Ch1 254nm@1.2nm
Sampling Rate:	10.00 per sec	Channel Desc.:	2998 Ch1 254nm@1.2nm
		Sample Set Name	

Sample Values Used in Calculation:

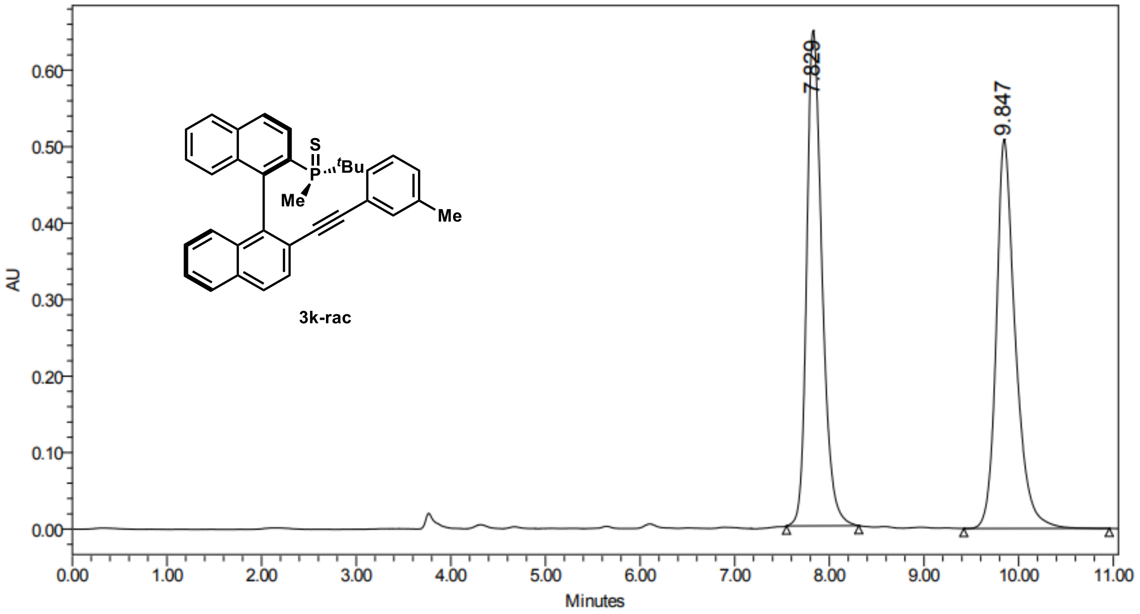

	RT (min)	Peak Type	Area (μV*sec)	% Area	Height (μV)	% Height	Integration Type	Points Across Peak	Start Time (min)	End Time (min)
1	7.829	Unknown	7293535	50.57	647520	56.01	bb	458	7.548	8.312
2	9.847	Unknown	7130008	49.43	508514	43.99	bb	922	9.422	10.958

Report Method: Detailed Individual Report
Page: 1 of 2

Printed: 5/28/2022 1:08:26 PM PRC

Supplementary Fig. 350. HPLC of **3k-rac.**

??????

Project Name Ik

Reported by User: Breeze user (Breeze)

SAMPLE INFORMATION

Sample Name:	PLZ-7-130-IA10%	Acquired By:	Breeze
Sample Type:	Unknown	Date Acquired:	5/28/2022 12:43:34 PM CST
Vial:	1	Acq. Method:	10% 254nm
Injection #:	1	Processed By:	Breeze
Injection Volume:	20.00 ul	Date Processed:	5/28/2022 1:07:49 PM CST
Run Time:	60.00 Minutes	Channel Name:	2998 Ch1 254nm@1.2nm
Sampling Rate:	10.00 per sec	Channel Desc.:	2998 Ch1 254nm@1.2nm
		Sample Set Name	

Sample Values
Used in Calculation:

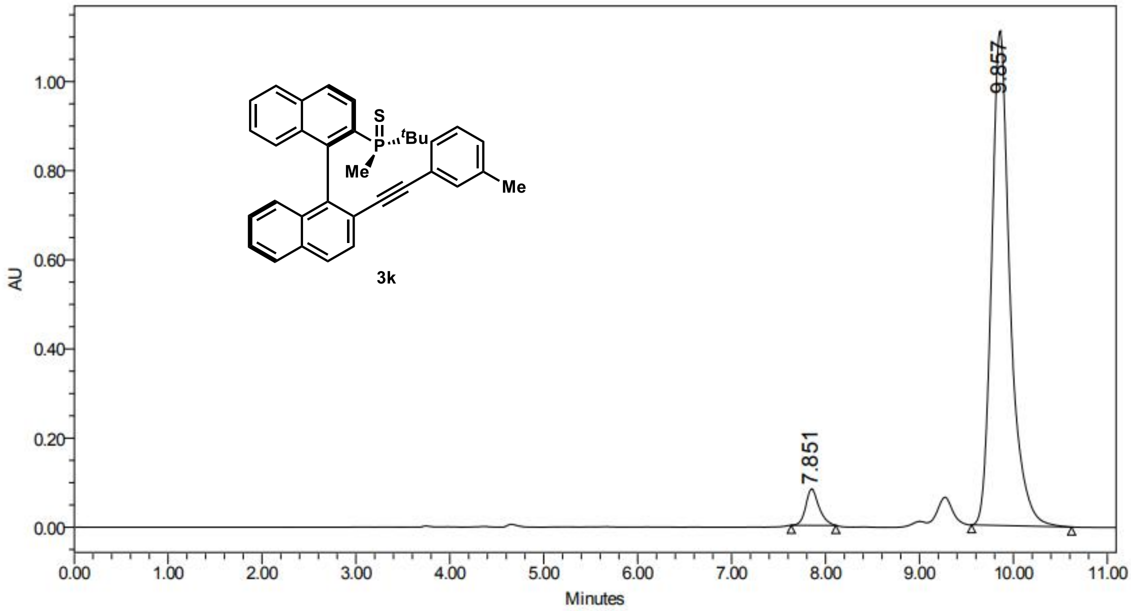

	RT (min)	Peak Type	Area (μV*sec)	% Area	Height (μV)	% Height	Integration Type	Points Across Peak	Start Time (min)	End Time (min)
1	7.851	Unknown	799921	5.03	81439	6.84	bb	285	7.633	8.108
2	9.857	Unknown	15112833	94.97	1109670	93.16	bb	639	9.553	10.618

Report Method: Detailed Individual Report
Page: 1 of 2

Printed: 5/28/2022
1:08:03 PM PRC

Supplementary Fig. 351. HPLC of **3k.**

??????

Project Name lk

Reported by User: Breeze user (Breeze)

SAMPLE INFORMATION

Sample Name:	PLZ-7-7A-IA10%	Acquired By:	Breeze
Sample Type:	Unknown	Date Acquired:	5/18/2022 4:53:01 AM CST
Vial:	1	Acq. Method:	10% 254nm
Injection #:	1	Processed By:	Breeze
Injection Volume:	20.00 ul	Date Processed:	5/18/2022 5:39:04 AM CST
Run Time:	60.00 Minutes	Channel Name:	2998 Ch1 254nm@1.2nm
Sampling Rate:	10.00 per sec	Channel Desc.:	2998 Ch1 254nm@1.2nm
		Sample Set Name	

Sample Values
Used in Calculation:

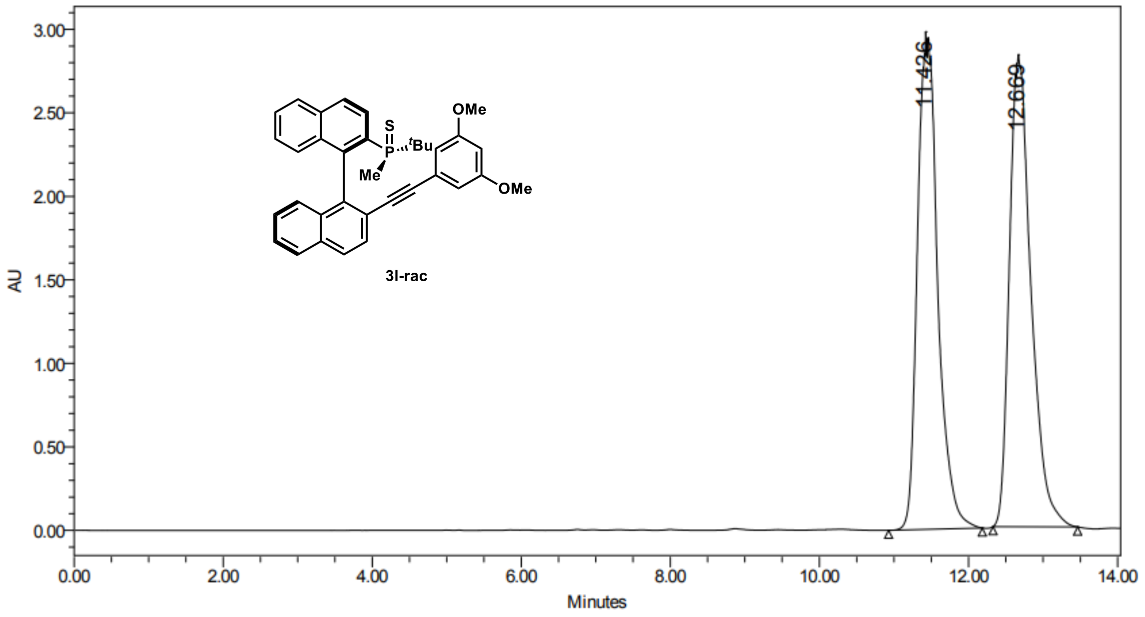

	RT (min)	Peak Type	Area (μV*sec)	% Area	Height (μV)	% Height	Integration Type	Points Across Peak	Start Time (min)
1	11.426	Unknown	56077587	49.70	2973945	51.28	bb	754	10.928
2	12.669	Unknown	56758074	50.30	2825417	48.72	bb	682	12.328

Report Method: Detailed Individual Report
Page: 1 of 2

Printed: 5/18/2022
5:39:20 AM PRC

Supplementary Fig. 352. HPLC of **3l-rac.**

??????
Project Name Ik
Reported by User: Breeze user (Breeze)

SAMPLE INFORMATION

Sample Name:	PLZ-7-105H-IA10%	Acquired By:	Breeze
Sample Type:	Unknown	Date Acquired:	5/18/2022 5:23:04 AM CST
Vial:	1	Acq. Method:	10% 254nm
Injection #:	3	Processed By:	Breeze
Injection Volume:	20.00 ul	Date Processed:	5/18/2022 5:39:57 AM CST
Run Time:	60.00 Minutes	Channel Name:	2998 Ch1 254nm@1.2nm
Sampling Rate:	10.00 per sec	Channel Desc.:	2998 Ch1 254nm@1.2nm
		Sample Set Name	

Sample Values
Used in Calculation:

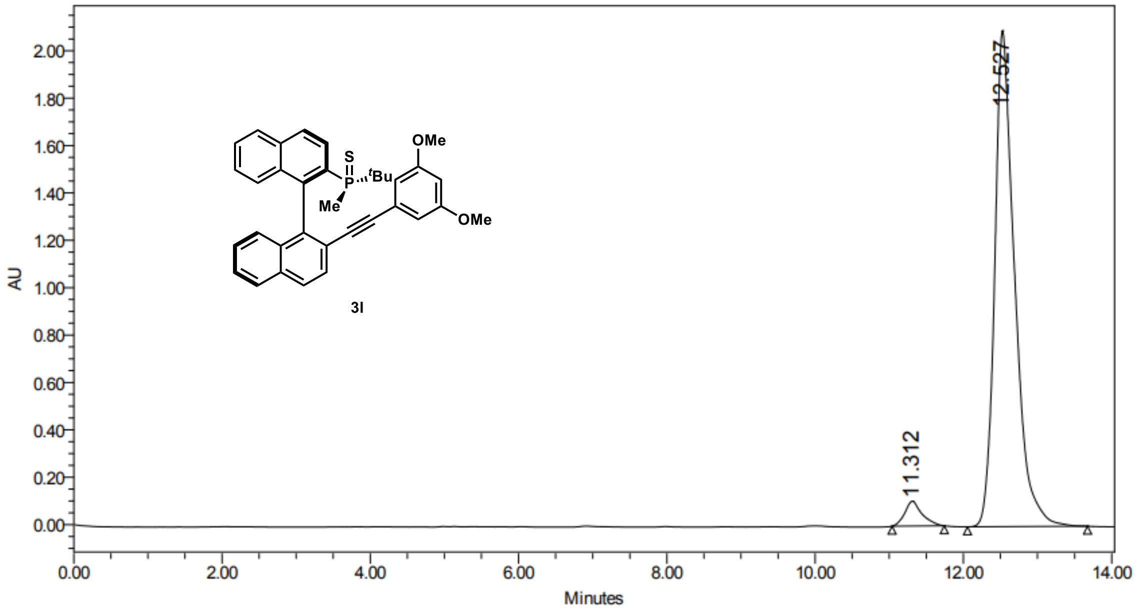

	RT (min)	Peak Type	Area (µV*sec)	% Area	Height (µV)	% Height	Integration Type	Points Across Peak	Start Time (min)
1	11.312	Unknown	1686693	4.13	104730	4.77	bb	422	11.038
2	12.527	Unknown	39118458	95.87	2093024	95.23	bb	972	12.055

Report Method: Detailed Individual Report
Page: 1 of 2
Printed: 5/18/2022 5:40:15 AM PRC

Supplementary Fig. 353. HPLC of **3l.**

??????

Project Name WR

Reported by User: Breeze user (Breeze)

SAMPLE INFORMATION

Sample Name:	PLZ-7-25D-ID10%	Acquired By:	Breeze
Sample Type:	Unknown	Date Acquired:	7/8/2022 12:45:48 PM CST
Vial:	1	Acq. Method:	10% 254nm
Injection #:	1	Date Processed:	7/8/2022 1:05:26 PM CST
Injection Volume:	20.00 ul	Channel Name:	2998 Ch1 254nm@1.2nm
Run Time:	60.00 Minutes	Sample Set Name	

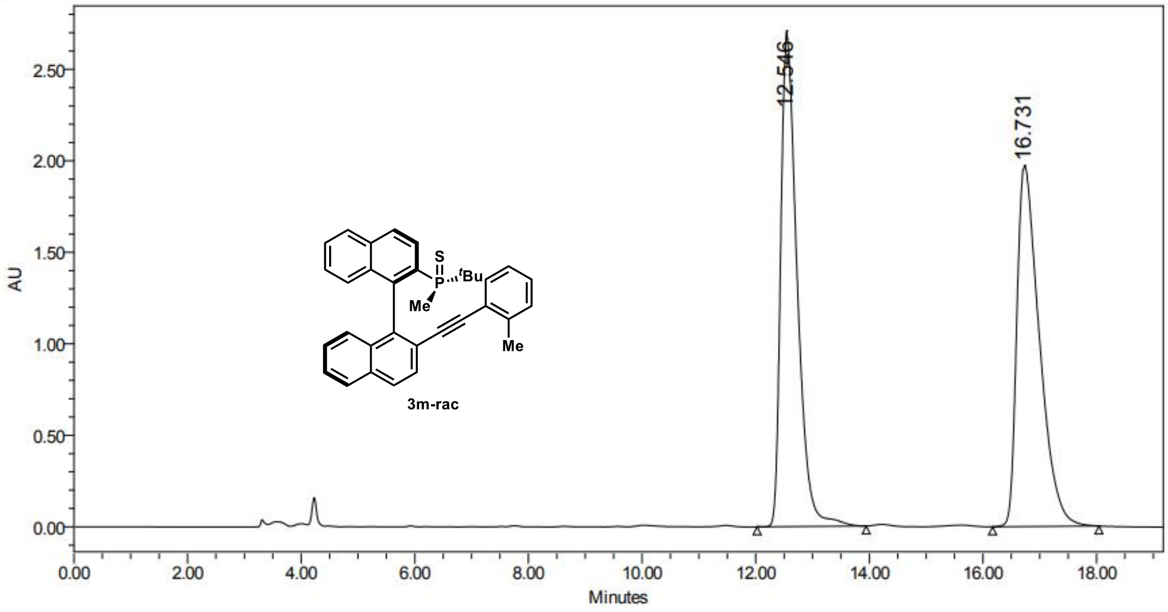

	RT (min)	Area (μV*sec)	% Area	Height (μV)	% Height
1	12.546	54081325	49.86	2710300	57.85
2	16.731	54374958	50.14	1974708	42.15

Report Method: Untitled
Page: 1 of 1

Printed: 7/8/2022
1:54:45 PM PRC

Supplementary Fig. 354. HPLC of **3m-rac.**

??????

Project Name WR

Reported by User: Breeze user (Breeze)

SAMPLE INFORMATION

Sample Name:	PLZ-7-105C-ID10%	Acquired By:	Breeze
Sample Type:	Unknown	Date Acquired:	7/8/2022 1:32:20 PM CST
Vial:	1	Acq. Method:	10% 254nm
Injection #:	3	Date Processed:	7/8/2022 1:53:23 PM CST
Injection Volume:	20.00 ul	Channel Name:	2998 Ch1 254nm@1.2nm
Run Time:	60.00 Minutes	Sample Set Name	

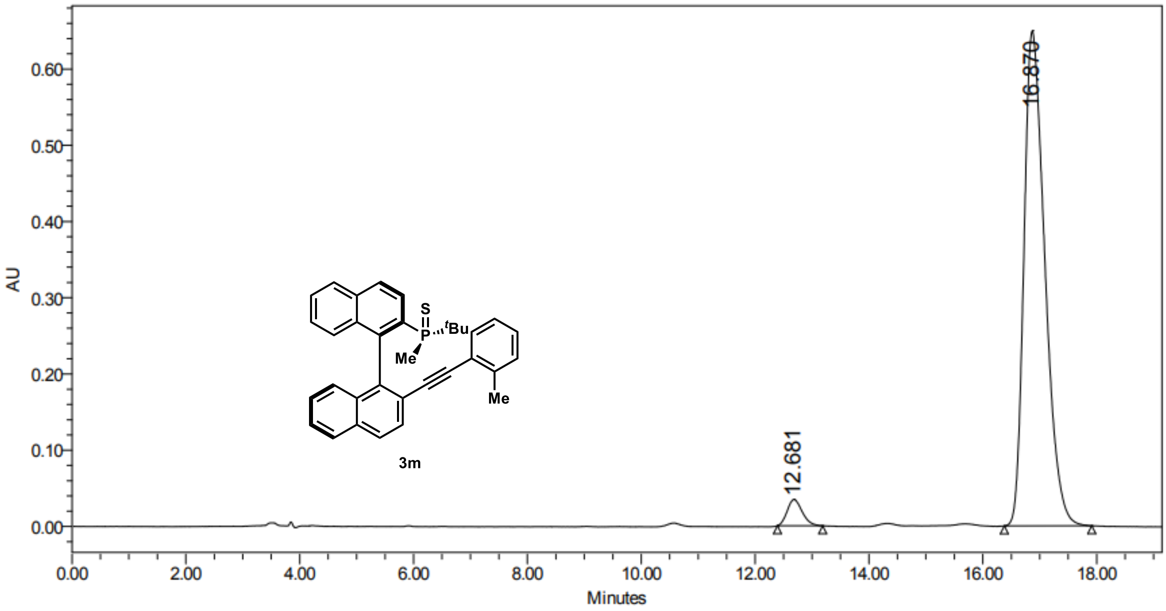

	RT (min)	Area (µV*sec)	% Area	Height (µV)	% Height
1	12.681	631519	3.68	34660	5.06
2	16.870	16518613	96.32	649705	94.94

Report Method: Untitled Printed: 7/8/2022

Page: 1 of 1 1:53:45 PM PRC

Supplementary Fig. 355. HPLC of **3m.**

SAMPLE INFORMATION

Sample Name:	PLZ-7-136C-IA10%	Acquired By:	Breeze
Sample Type:	Unknown	Date Acquired:	7/4/2022 6:22:18 AM CST
Vial:	1	Acq. Method:	10% 254nm
Injection #:	3	Processed By:	Breeze
Injection Volume:	20.00 ul	Date Processed:	7/4/2022 7:06:36 AM CST
Run Time:	60.00 Minutes	Channel Name:	2998 Ch1 254nm@1.2nm
Sampling Rate:	10.00 per sec	Channel Desc.:	2998 Ch1 254nm@1.2nm
		Sample Set Name	

Sample Values
Used in Calculation:

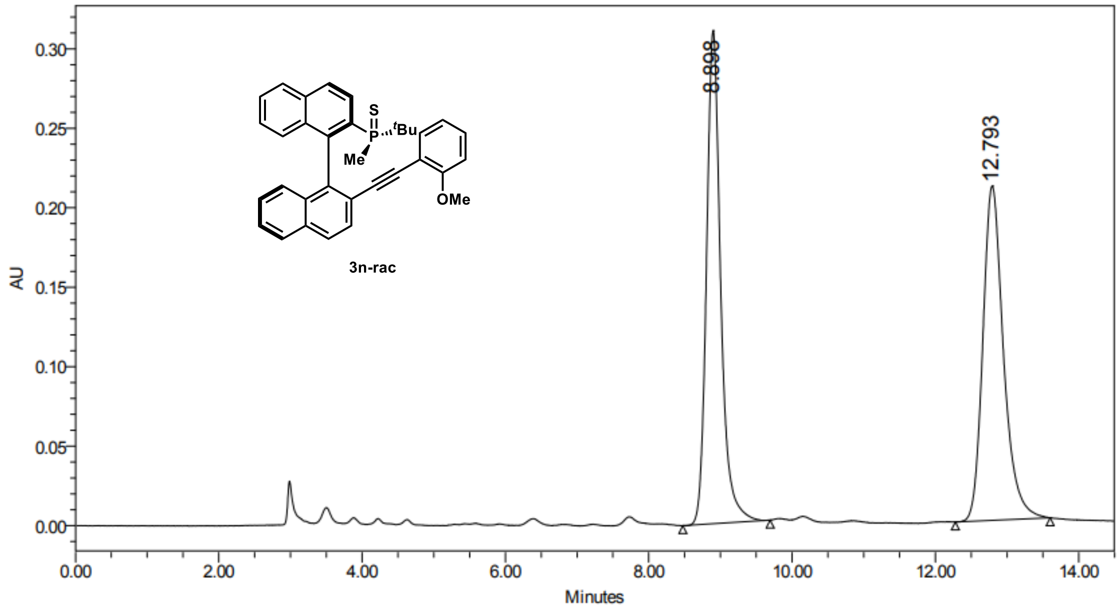

	RT (min)	Peak Type	Area (µV*sec)	% Area	Height (µV)	% Height	Integration Type	Points Across Peak	Start Time (min)	End Time (min)
1	8.898	Unknown	4257431	50.30	310321	59.61	bb	732	8.475	9.695
2	12.793	Unknown	4207182	49.70	210266	40.39	bb	794	12.278	13.602

Supplementary Fig. 356. HPLC of **3n-rac.**

??????

Project Name Ik

Reported by User: Breeze user (Breeze)

SAMPLE INFORMATION

Sample Name:	PLZ-7-142B-IA10%	Acquired By:	Breeze
Sample Type:	Unknown	Date Acquired:	7/4/2022 6:39:22 AM CST
Vial:	1	Acq. Method:	10% 254nm
Injection #:	4	Processed By:	Breeze
Injection Volume:	20.00 ul	Date Processed:	7/4/2022 7:07:51 AM CST
Run Time:	60.00 Minutes	Channel Name:	2998 Ch1 254nm@1.2nm
Sampling Rate:	10.00 per sec	Channel Desc.:	2998 Ch1 254nm@1.2nm
		Sample Set Name	

Sample Values
Used in Calculation:

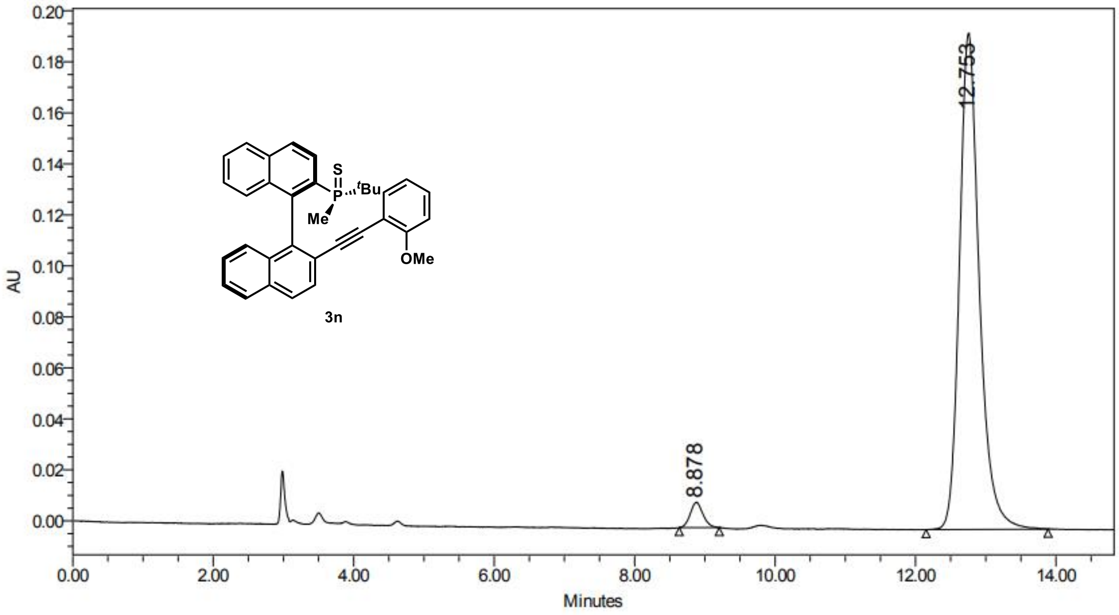

	RT (min)	Peak Type	Area (µV*sec)	% Area	Height (µV)	% Height	Integration Type	Points Across Peak	Start Time (min)	End Time (min)
1	8.878	Unknown	124223	3.15	9863	4.83	bb	343	8.635	9.207
2	12.753	Unknown	3821289	96.85	194512	95.17	bb	1041	12.150	13.885

Report Method: Detailed Individual Report
Page: 1 of 2

Printed: 7/4/2022
7:08:49 AM PRC

Supplementary Fig. 357. HPLC of **3n.**

??????

Project Name lk

Reported by User: Breeze user (Breeze)

SAMPLE INFORMATION

Sample Name:	PLZ-7-136B-ID10%	Acquired By:	Breeze
Sample Type:	Unknown	Date Acquired:	7/5/2022 9:07:26 AM CST
Vial:	1	Acq. Method:	10% 254nm
Injection #:	3	Processed By:	Breeze
Injection Volume:	20.00 ul	Date Processed:	7/5/2022 9:55:58 AM CST
Run Time:	60.00 Minutes	Channel Name:	2998 Ch1 254nm@1.2nm
Sampling Rate:	10.00 per sec	Channel Desc.:	2998 Ch1 254nm@1.2nm
		Sample Set Name	
Sample Values Used in Calculation:			

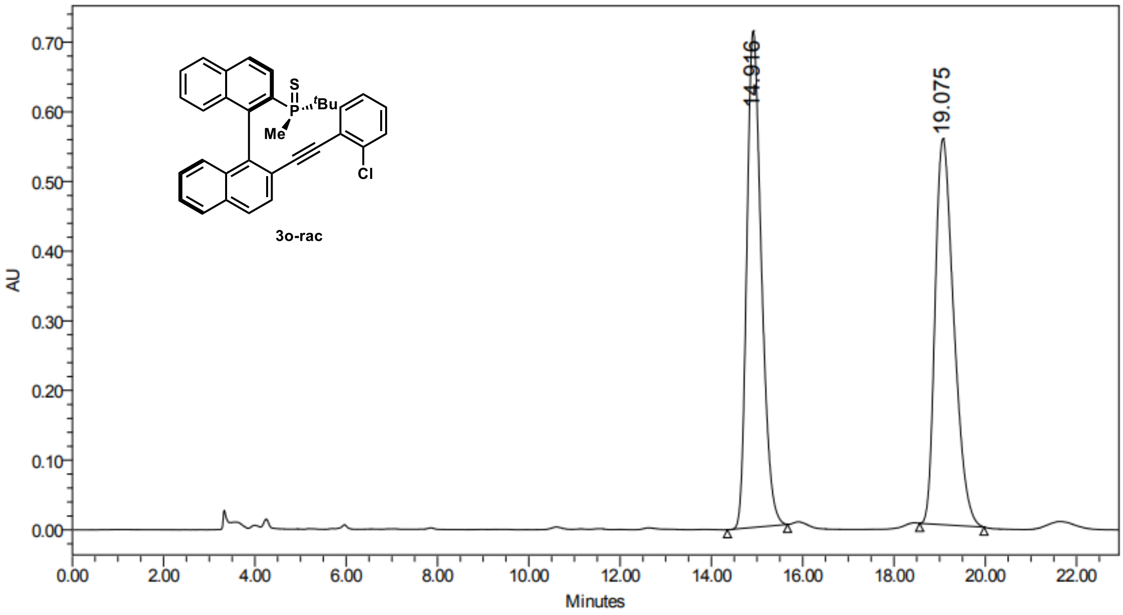

	RT (min)	Peak Type	Area (µV*sec)	% Area	Height (µV)	% Height	Integration Type	Points Across Peak	Start Time (min)	End Time (min)
1	14.916	Unknown	15843898	50.13	712843	56.27	bb	791	14.350	15.668
2	19.075	Unknown	15762127	49.87	554055	43.73	bb	848	18.562	19.975

Report Method: Detailed Individual Report

Page: 1 of 2

Printed: 7/5/2022 10:00:34 AM PRC

Supplementary Fig. 358. HPLC of **3o-rac.**

??????

Project Name Ik

Reported by User: Breeze user (Breeze)

Breeze 2 HPLC System

SAMPLE INFORMATION

Sample Name:	PLZ-7-142A-ID10%	Acquired By:	Breeze
Sample Type:	Unknown	Date Acquired:	7/5/2022 9:32:27 AM CST
Vial:	1	Acq. Method:	10% 254nm
Injection #:	4	Processed By:	Breeze
Injection Volume:	20.00 ul	Date Processed:	7/5/2022 9:59:49 AM CST
Run Time:	60.00 Minutes	Channel Name:	2998 Ch1 254nm@1.2nm
Sampling Rate:	10.00 per sec	Channel Desc.:	2998 Ch1 254nm@1.2nm
		Sample Set Name	

Sample Values Used in Calculation:

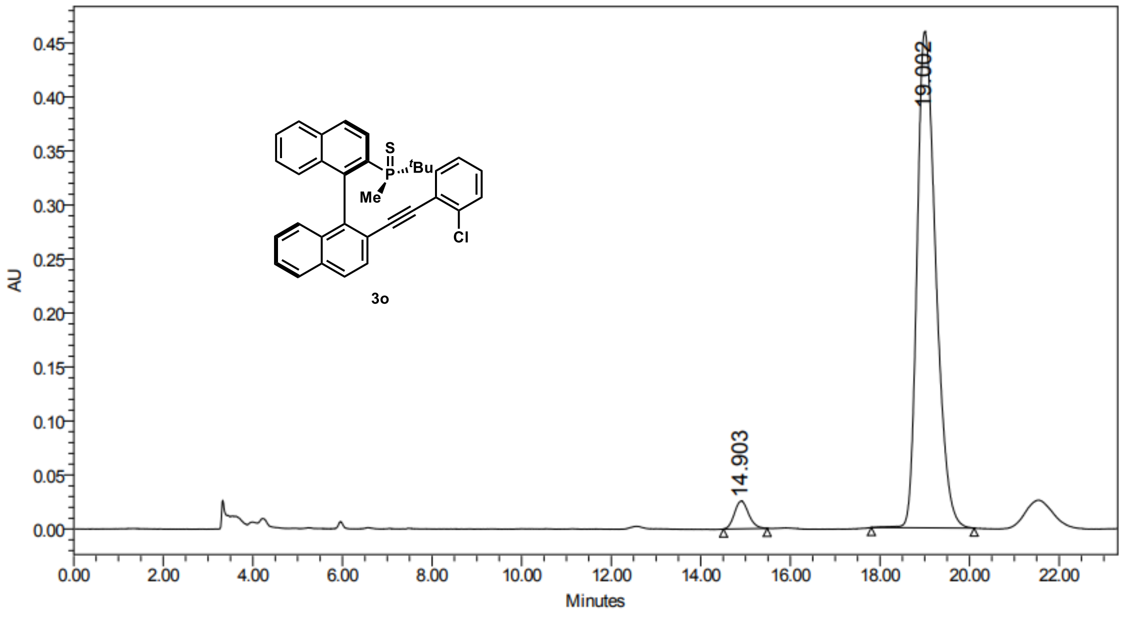

	RT (min)	Peak Type	Area (µV*sec)	% Area	Height (µV)	% Height	Integration Type	Points Across Peak	Start Time (min)	End Time (min)
1	14.903	Unknown	563884	4.06	25748	5.30	bb	587	14.507	15.485
2	19.002	Unknown	13335648	95.94	459660	94.70	bb	1375	17.810	20.102

Report Method: Detailed Individual Report

Page: 1 of 2

Printed: 7/5/2022 10:00:02 AM PRC

Supplementary Fig. 359. HPLC of **3o.**

??????

Project Name lk

Reported by User: Breeze user (Breeze)

SAMPLE INFORMATION

Sample Name:	PLZ-7-146B-IA10%	Acquired By:	Breeze
Sample Type:	Unknown	Date Acquired:	7/4/2022 6:55:39 AM CST
Vial:	1	Acq. Method:	10% 254nm
Injection #:	5	Processed By:	Breeze
Injection Volume:	20.00 ul	Date Processed:	7/4/2022 7:09:25 AM CST
Run Time:	60.00 Minutes	Channel Name:	2998 Ch1 254nm@1.2nm
Sampling Rate:	10.00 per sec	Channel Desc.:	2998 Ch1 254nm@1.2nm
		Sample Set Name	

Sample Values Used in Calculation:

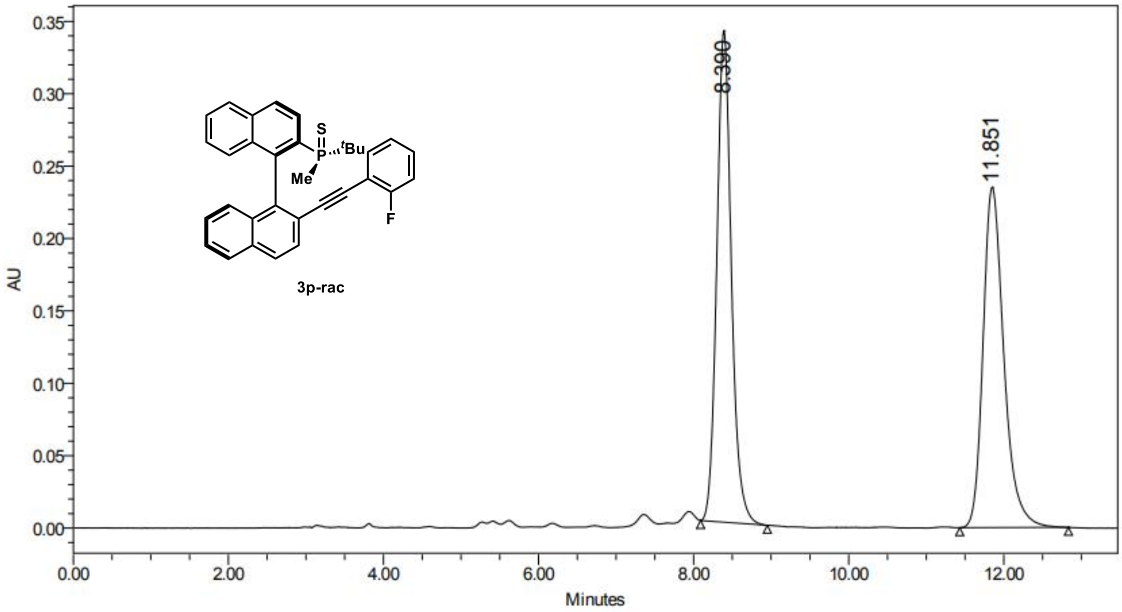

	RT (min)	Peak Type	Area (µV*sec)	% Area	Height (µV)	% Height	Integration Type	Points Across Peak	Start Time (min)	End Time (min)
1	8.390	Unknown	4441733	50.34	339493	59.09	bb	516	8.090	8.950
2	11.851	Unknown	4381924	49.66	235027	40.91	bb	841	11.432	12.833

Report Method: Detailed Individual Report
Page: 1 of 2

Printed: 7/4/2022 7:28:33 AM PRC

Supplementary Fig. 360. HPLC of **3p-rac.**

??????

Project Name Ik

Reported by User: Breeze user (Breeze)

SAMPLE INFORMATION

Sample Name:	PLZ-7-145B-IA10%	Acquired By:	Breeze
Sample Type:	Unknown	Date Acquired:	7/4/2022 7:13:08 AM CST
Vial:	1	Acq. Method:	10% 254nm
Injection #:	6	Processed By:	Breeze
Injection Volume:	20.00 ul	Date Processed:	7/4/2022 7:28:13 AM CST
Run Time:	60.00 Minutes	Channel Name:	2998 Ch1 254nm@1.2nm
Sampling Rate:	10.00 per sec	Channel Desc.:	2998 Ch1 254nm@1.2nm
		Sample Set Name	

Sample Values
Used in Calculation:

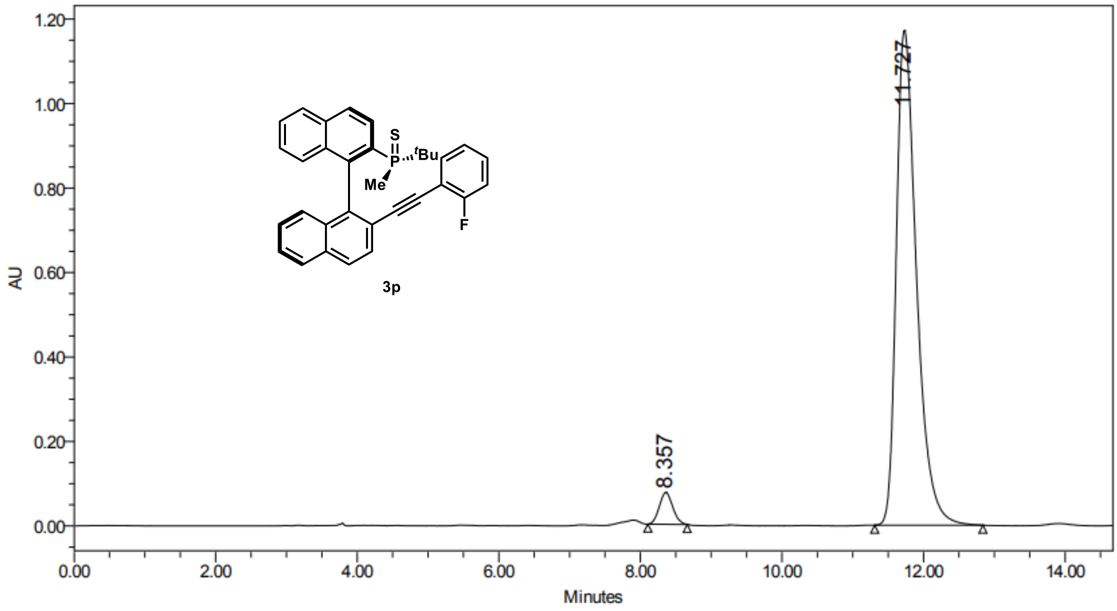

	RT (min)	Peak Type	Area (µV*sec)	% Area	Height (µV)	% Height	Integration Type	Points Across Peak	Start Time (min)
1	8.357	Unknown	997007	4.11	75436	6.05	bb	333	8.105
2	11.727	Unknown	23254798	95.89	1171212	93.95	bb	918	11.308

Report Method: Detailed Individual Report
Page: 1 of 2

Printed: 7/4/2022
7:28:49 AM PRC

Supplementary Fig. 361. HPLC of **3p.**

??????

Project Name lk

Reported by User: Breeze user (Breeze)

SAMPLE INFORMATION

Sample Name:	PLZ-7-28F-IA10%	Acquired By:	Breeze
Sample Type:	Unknown	Date Acquired:	5/19/2022 9:08:39 AM CST
Vial:	1	Acq. Method:	10% 254nm
Injection #:	4	Processed By:	Breeze
Injection Volume:	20.00 ul	Date Processed:	5/19/2022 10:09:43 AM CST
Run Time:	60.00 Minutes	Channel Name:	2998 Ch1 254nm@1.2nm
Sampling Rate:	10.00 per sec	Channel Desc.:	2998 Ch1 254nm@1.2nm
		Sample Set Name	

Sample Values
Used in Calculation:

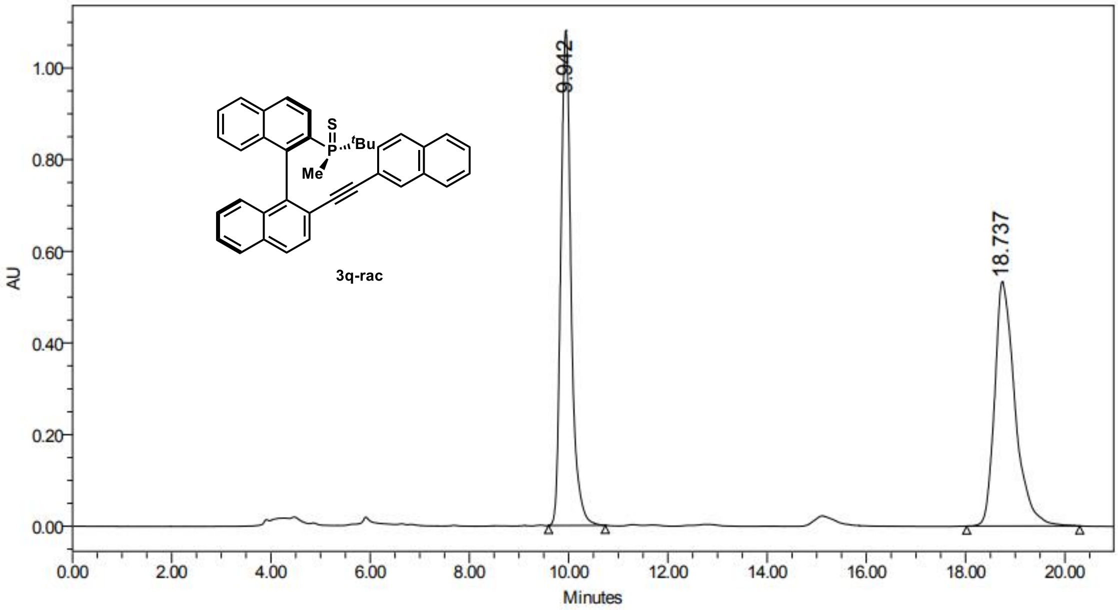

	RT (min)	Peak Type	Area (µV*sec)	% Area	Height (µV)	% Height	Integration Type	Points Across Peak	Start Time (min)
1	9.942	Unknown	15570928	50.56	1080319	66.97	bb	688	9.595
2	18.737	Unknown	15227489	49.44	532839	33.03	bb	1367	18.022

Report Method: Detailed Individual Report
Page: 1 of 2

Printed: 5/19/2022
10:09:57 AM PRC

Supplementary Fig. 362. HPLC of **3q-rac.**

??????

Project Name Ik

Reported by User: Breeze user (Breeze)

SAMPLE INFORMATION

Sample Name:	PLZ-7-98F-IA10%	Acquired By:	Breeze
Sample Type:	Unknown	Date Acquired:	5/19/2022 8:48:02 AM CST
Vial:	1	Acq. Method:	10% 254nm
Injection #:	3	Processed By:	Breeze
Injection Volume:	20.00 ul	Date Processed:	5/19/2022 10:07:49 AM CST
Run Time:	60.00 Minutes	Channel Name:	2998 Ch1 254nm@1.2nm
Sampling Rate:	10.00 per sec	Channel Desc.:	2998 Ch1 254nm@1.2nm
		Sample Set Name	

Sample Values Used in Calculation:

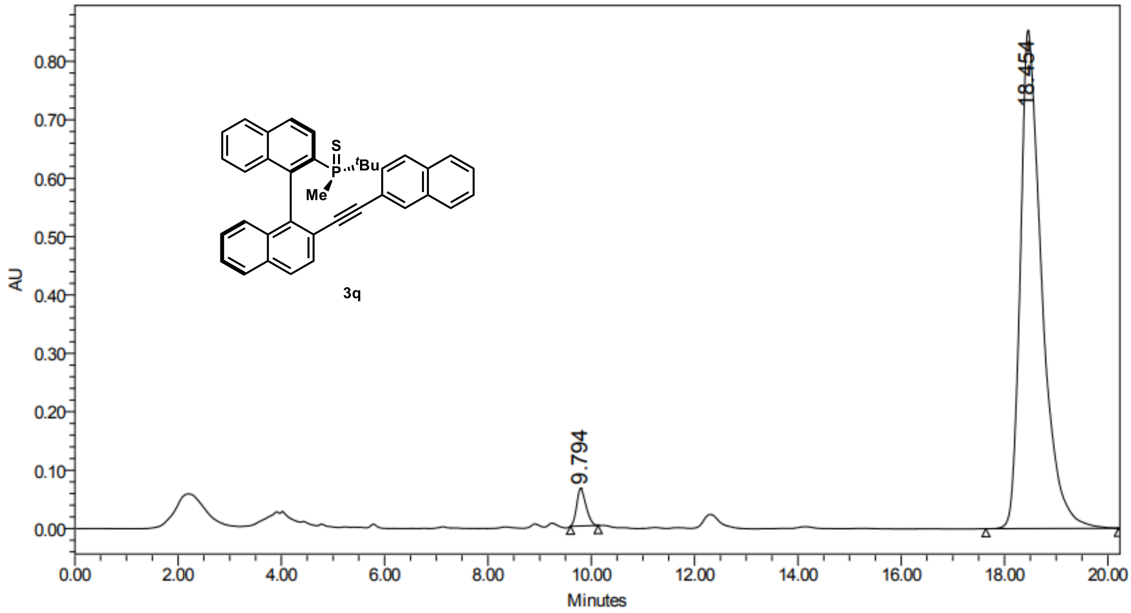

	RT (min)	Peak Type	Area (µV*sec)	% Area	Height (µV)	% Height	Integration Type	Points Across Peak	Start Time (min)	End Time (min)
1	9.794	Unknown	804625	3.16	64787	7.06	bb	321	9.597	10.132
2	18.454	Unknown	24628852	96.84	852935	92.94	bb	1539	17.640	20.205

Report Method: Detailed Individual Report

Page: 1 of 2

Printed: 5/19/2022 10:08:15 AM PRC

Supplementary Fig. 363. HPLC of **3q.**

??????

Project Name Ik

Reported by User: Breeze user (Breeze)

SAMPLE INFORMATION

Sample Name:	PLZ-7-94C-IA5%	Acquired By:	Breeze
Sample Type:	Unknown	Date Acquired:	7/4/2022 12:15:42 PM CST
Vial:	1	Acq. Method:	5% 254nm
Injection #:	1	Processed By:	Breeze
Injection Volume:	20.00 ul	Date Processed:	7/4/2022 12:59:22 PM CST
Run Time:	60.00 Minutes	Channel Name:	2998 Ch1 254nm@1.2nm
Sampling Rate:	10.00 per sec	Channel Desc.:	2998 Ch1 254nm@1.2nm
		Sample Set Name	

Sample Values Used in Calculation:

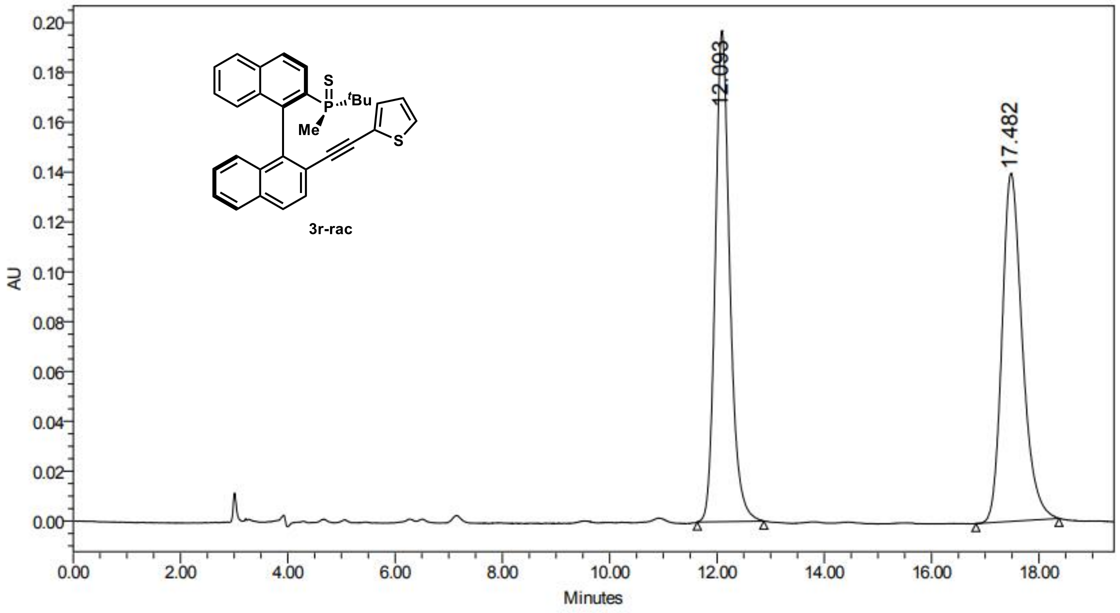

	RT (min)	Peak Type	Area (µV*sec)	% Area	Height (µV)	% Height	Integration Type	Points Across Peak	Start Time (min)	End Time (min)
1	12.093	Unknown	3707297	50.25	196999	58.52	bb	744	11.633	12.873
2	17.482	Unknown	3670429	49.75	139620	41.48	bb	929	16.827	18.375

Report Method: Detailed Individual Report

Page: 1 of 2

Printed: 7/4/2022 1:00:08 PM PRC

Supplementary Fig. 364. HPLC of **3r-rac.**

??????
Project Name Ik
Reported by User: Breeze user (Breeze)

Breeze 2 HPLC System

SAMPLE INFORMATION

Sample Name:	PLZ-7-165-IA5%	Acquired By:	Breeze
Sample Type:	Unknown	Date Acquired:	7/4/2022 12:36:46 PM CST
Vial:	1	Acq. Method:	5% 254nm
Injection #:	2	Processed By:	Breeze
Injection Volume:	20.00 ul	Date Processed:	7/4/2022 12:58:50 PM CST
Run Time:	60.00 Minutes	Channel Name:	2998 Ch1 254nm@1.2nm
Sampling Rate:	10.00 per sec	Channel Desc.:	2998 Ch1 254nm@1.2nm
		Sample Set Name	

Sample Values
Used in Calculation:

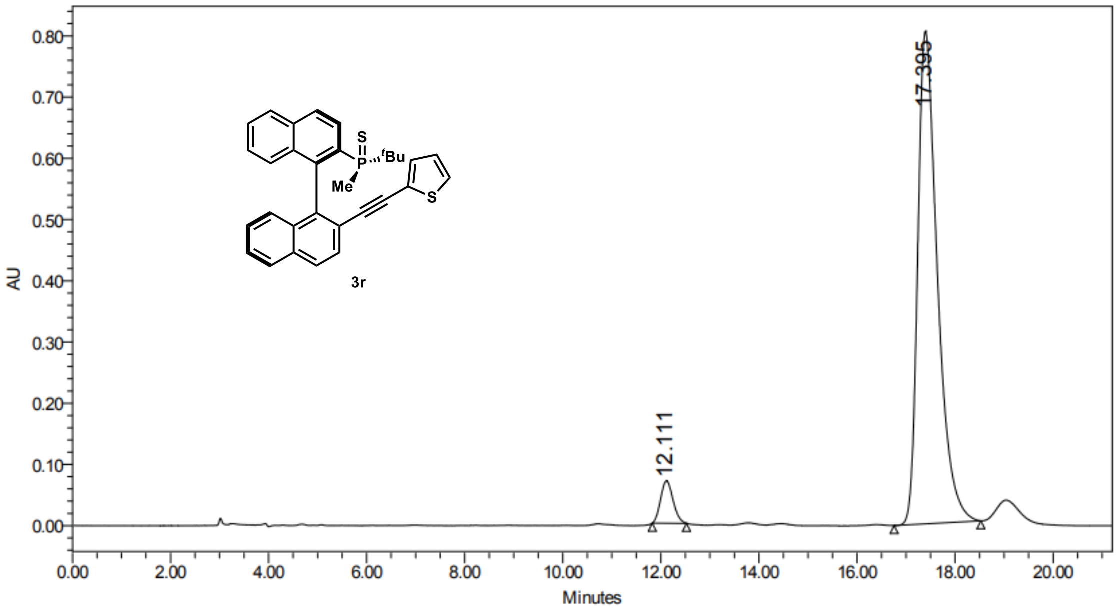

	RT (min)	Peak Type	Area (µV*sec)	% Area	Height (µV)	% Height	Integration Type	Points Across Peak	Start Time (min)	End Time (min)
1	12.111	Unknown	1222024	5.18	69378	7.94	bb	419	11.825	12.523
2	17.395	Unknown	22364945	94.82	804700	92.06	bb	1062	16.758	18.528

Report Method: Detailed Individual Report
Page: 1 of 2

Printed: 7/4/2022
12:59:37 PM PRC

Supplementary Fig. 365. HPLC of **3r.**

??????
Project Name Ik
Reported by User: Breeze user (Breeze)

SAMPLE INFORMATION

Sample Name: PLZ-7-106-IA10%
Sample Type: Unknown
Vial: 1
Injection #: 1
Injection Volume: 20.00 ul
Run Time: 60.00 Minutes
Sampling Rate: 10.00 per sec

Sample Values
Used in Calculation:

Acquired By: Breeze
Date Acquired: 5/20/2022 8:19:21 AM CST
Acq. Method: 10% 254nm
Processed By: Breeze
Date Processed: 5/20/2022 8:45:37 AM CST
Channel Name: 2998 Ch1 254nm@1.2nm
Channel Desc.: 2998 Ch1 254nm@1.2nm
Sample Set Name

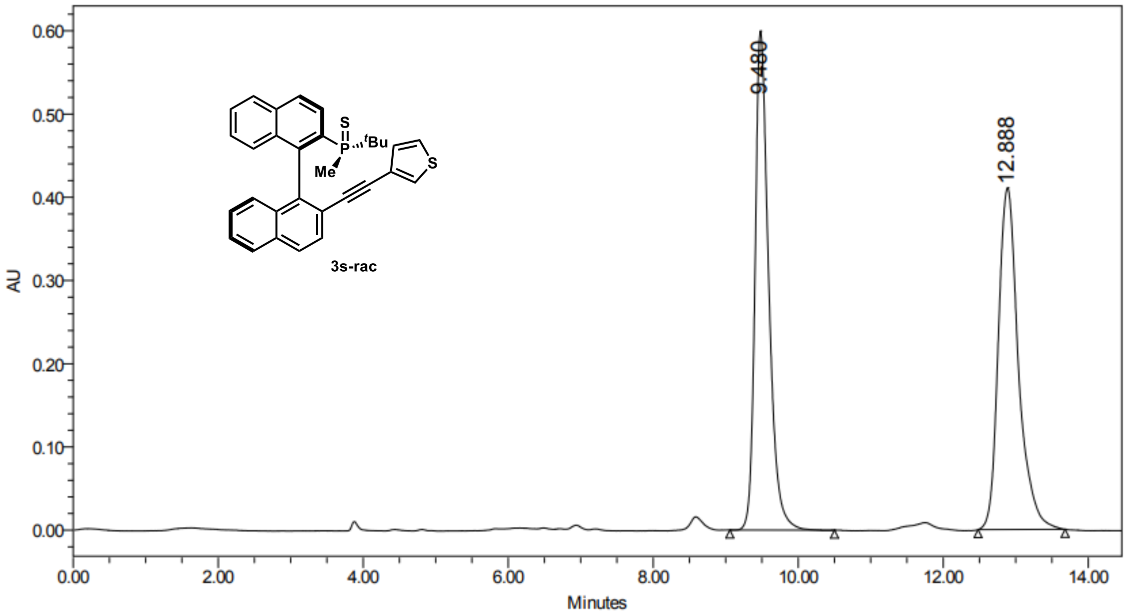

	RT (min)	Peak Type	Area (μV*sec)	% Area	Height (μV)	% Height	Integration Type	Points Across Peak	Start Time (min)	End Time (min)
1	9.480	Unknown	7816269	49.69	599606	59.36	bb	866	9.057	10.500
2	12.888	Unknown	7915034	50.31	410491	40.64	bb	720	12.480	13.680

Report Method: Detailed Individual Report
Page: 1 of 2

Printed: 5/20/2022
8:45:56 AM PRC

Supplementary Fig. 366. HPLC of **3s-rac.**

??????

Project Name lk

Reported by User: Breeze user (Breeze)

SAMPLE INFORMATION

Sample Name:	PLZ-7-97D-IA10%	Acquired By:	Breeze
Sample Type:	Unknown	Date Acquired:	5/20/2022 8:34:13 AM CST
Vial:	1	Acq. Method:	10% 254nm
Injection #:	2	Processed By:	Breeze
Injection Volume:	20.00 ul	Date Processed:	5/20/2022 8:49:24 AM CST
Run Time:	60.00 Minutes	Channel Name:	2998 Ch1 254nm@1.2nm
Sampling Rate:	10.00 per sec	Channel Desc.:	2998 Ch1 254nm@1.2nm
		Sample Set Name	

Sample Values Used in Calculation:

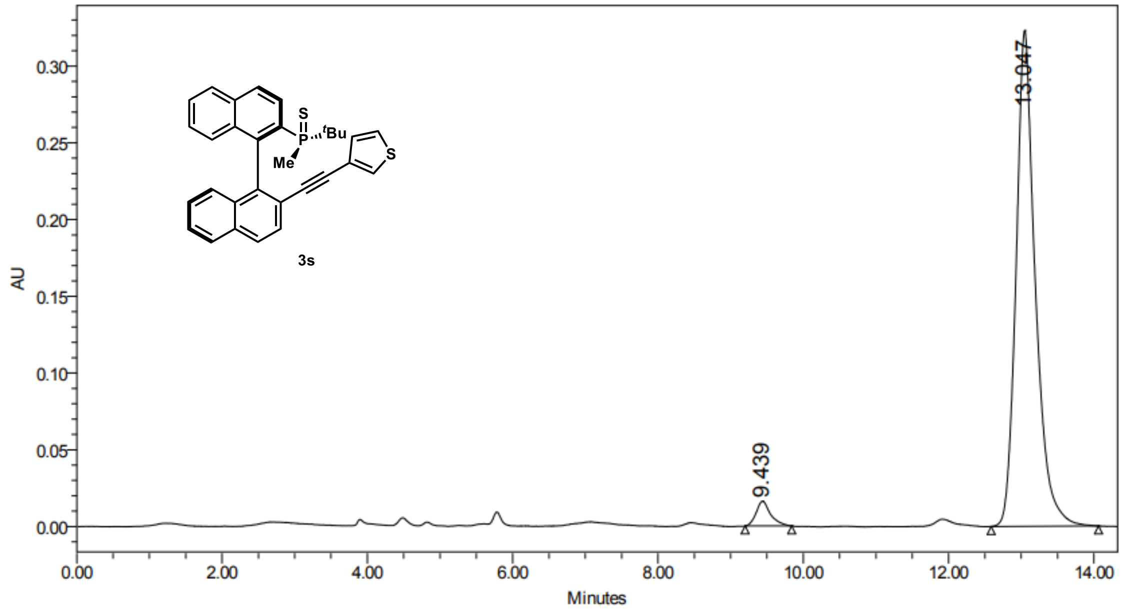

	RT (min)	Peak Type	Area (µV*sec)	% Area	Height (µV)	% Height	Integration Type	Points Across Peak	Start Time (min)	End Time (min)
1	9.439	Unknown	221888	3.60	16132	4.75	bb	385	9.200	9.842
2	13.047	Unknown	5939656	96.40	323133	95.25	bb	887	12.585	14.063

Report Method: Detailed Individual Report

Page: 1 of 2

Printed: 5/20/2022 8:49:46 AM PRC

Supplementary Fig. 367. HPLC of **3s.**

??????
Project Name WR
Reported by User: Breeze user (Breeze)

Breeze 2 HPLC System

SAMPLE INFORMATION

Sample Name:	PLZ-7-66-IE10%	Acquired By:	Breeze
Sample Type:	Unknown	Date Acquired:	7/2/2022 8:04:16 AM CST
Vial:	1	Acq. Method:	10% 254nm
Injection #:	3	Date Processed:	7/2/2022 8:29:01 AM CST
Injection Volume:	20.00 ul	Channel Name:	2998 Ch1 254nm@1.2nm
Run Time:	60.00 Minutes	Sample Set Name	

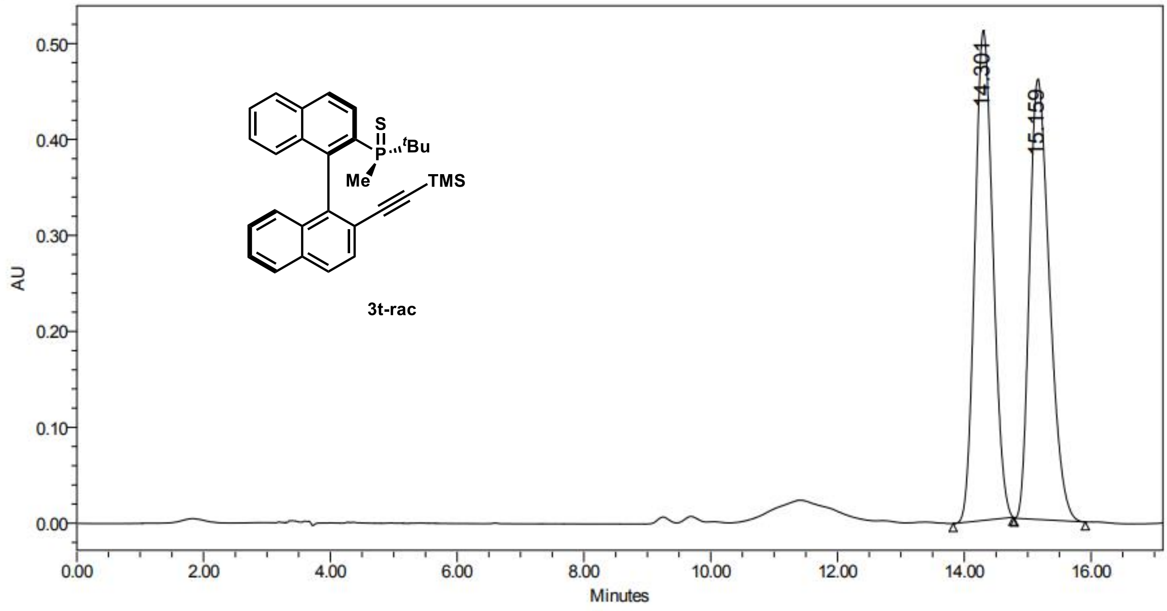

	RT (min)	Area (µV*sec)	% Area	Height (µV)	% Height
1	14.301	10106825	50.13	510451	52.68
2	15.159	10052891	49.87	458547	47.32

Report Method: Untitled
Page: 1 of 1

Printed: 7/2/2022
8:30:28 AM PRC

Supplementary Fig. 368. HPLC of **3t-rac.**

??????

Project Name WR

Reported by User: Breeze user (Breeze)

SAMPLE INFORMATION

Sample Name:	PLZ-7-193A-IE10%	Acquired By:	Breeze
Sample Type:	Unknown	Date Acquired:	7/2/2022 8:27:01 AM CST
Vial:	1	Acq. Method:	10% 254nm
Injection #:	4	Date Processed:	7/2/2022 8:44:35 AM CST
Injection Volume:	20.00 ul	Channel Name:	2998 Ch1 254nm@1.2nm
Run Time:	60.00 Minutes	Sample Set Name	

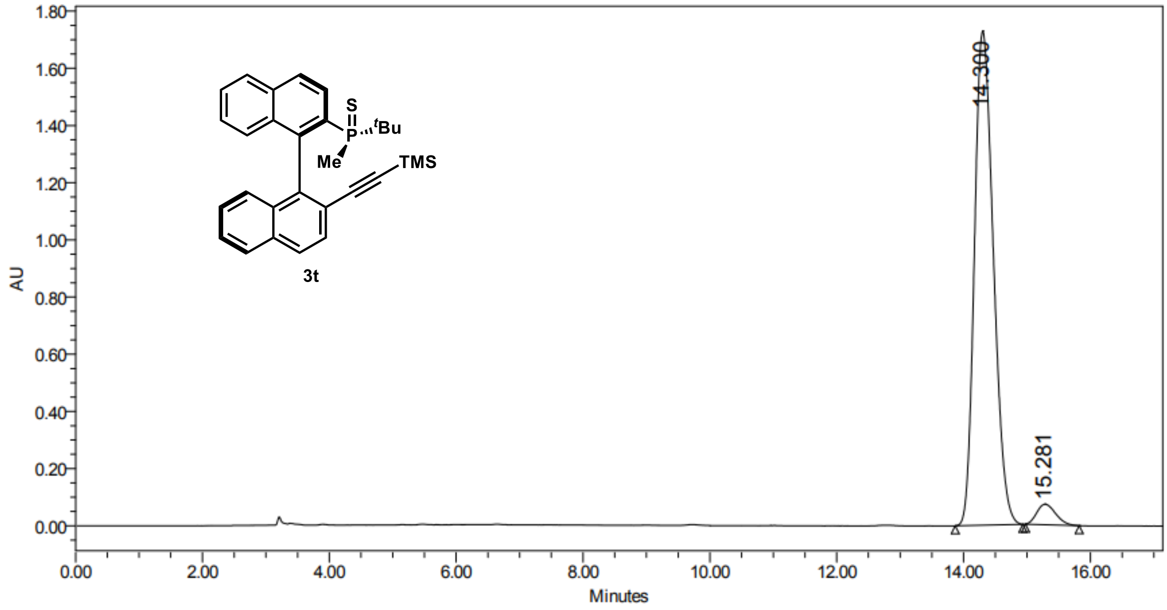

	RT (min)	Area (µV*sec)	% Area	Height (µV)	% Height
1	14.300	35761840	96.01	1728642	96.02
2	15.281	1487709	3.99	71691	3.98

Report Method: Untitled

Page: 1 of 1

Printed: 7/2/2022

8:44:50 AM PRC

Supplementary Fig. 369. HPLC of **3t.**

??????

Project Name WR

Reported by User: Breeze user (Breeze)

SAMPLE INFORMATION

Sample Name:	PLZ-7-135-IA10%	Acquired By:	Breeze
Sample Type:	Unknown	Date Acquired:	6/30/2022 5:51:27 AM CST
Vial:	1	Acq. Method:	10% 254nm
Injection #:	5	Date Processed:	6/30/2022 6:03:07 AM CST
Injection Volume:	20.00 ul	Channel Name:	2998 Ch1 254nm@1.2nm
Run Time:	60.00 Minutes	Sample Set Name	

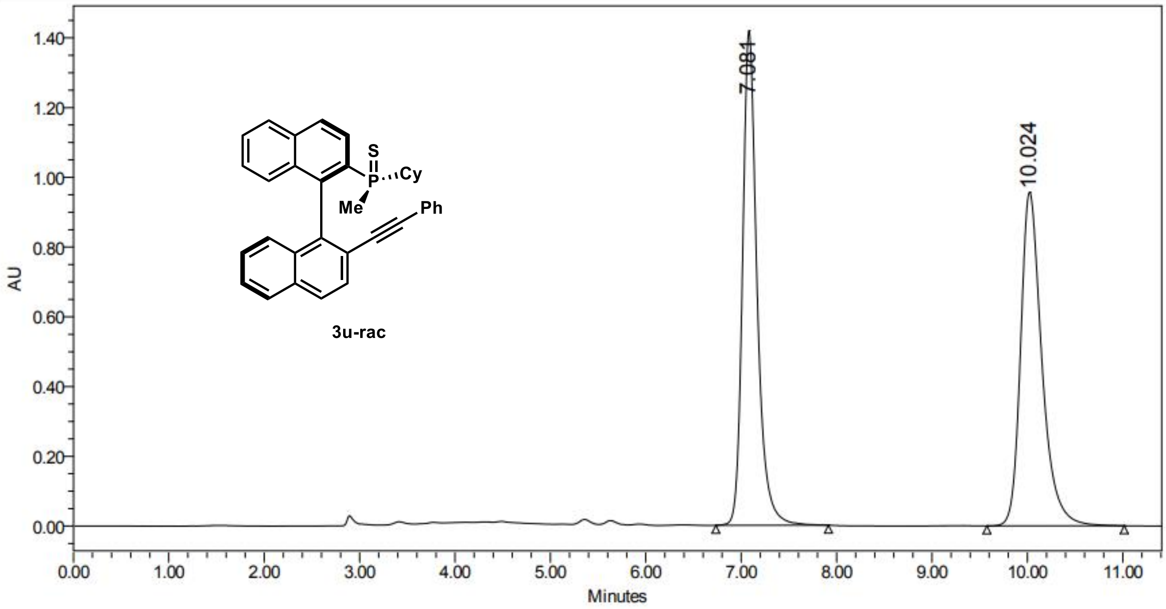

	RT (min)	Area (µV*sec)	% Area	Height (µV)	% Height
1	7.081	14952061	50.23	1418506	59.71
2	10.024	14813191	49.77	957119	40.29

Report Method: Untitled

Page: 1 of 1

Printed: 7/9/2022 12:35:40 AM PRC

Supplementary Fig. 370. HPLC of **3u-rac.**

??????

Project Name WR

Reported by User: Breeze user (Breeze)

Breeze 2 HPLC System

SAMPLE INFORMATION

Sample Name:	PLZ-7-126B-IA10%	Acquired By:	Breeze
Sample Type:	Unknown	Date Acquired:	6/30/2022 6:05:39 AM CST
Vial:	1	Acq. Method:	10% 254nm
Injection #:	6	Date Processed:	6/30/2022 6:17:16 AM CST
Injection Volume:	20.00 ul	Channel Name:	2998 Ch1 254nm@1.2nm
Run Time:	60.00 Minutes	Sample Set Name	

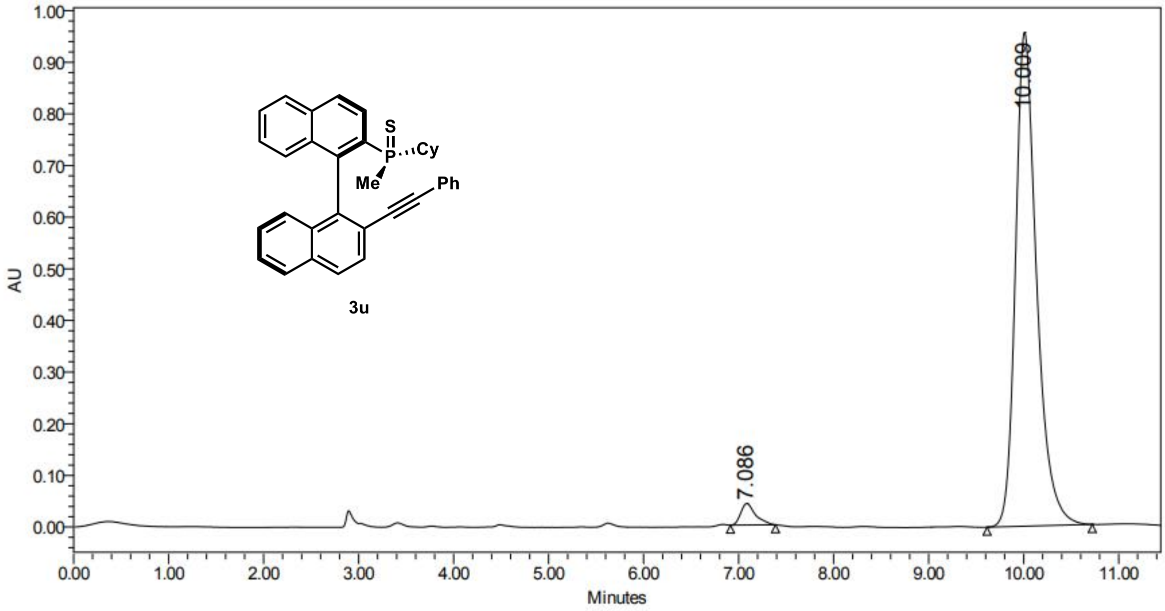

	RT (min)	Area (µV*sec)	% Area	Height (µV)	% Height
1	7.086	448709	2.94	41858	4.19
2	10.009	14803553	97.06	956035	95.81

Report Method: Untitled

Page: 1 of 1

Printed: 6/30/2022 6:17:35 AM PRC

Supplementary Fig. 371. HPLC of **3u.**

2022/8/2 9:55:19 1 / 1

SHIMADZU LabSolutions 分析报告

〈样品信息〉

样品名	:		
样品ID	: WXB-220418-37-1		
数据文件名	: PLZ-8-97A-IA10%1.lcd		
方法文件名	: 1.1cm		
批处理文件名	:		
样品瓶号	: 1-1	样品类型	: 未知
进样体积	: 1 uL		
分析日期	: 2022/7/25 18:48:58	分析者	: System Administrator
处理日期	: 2022/8/2 9:54:52	处理者	: System Administrator

〈色谱图〉

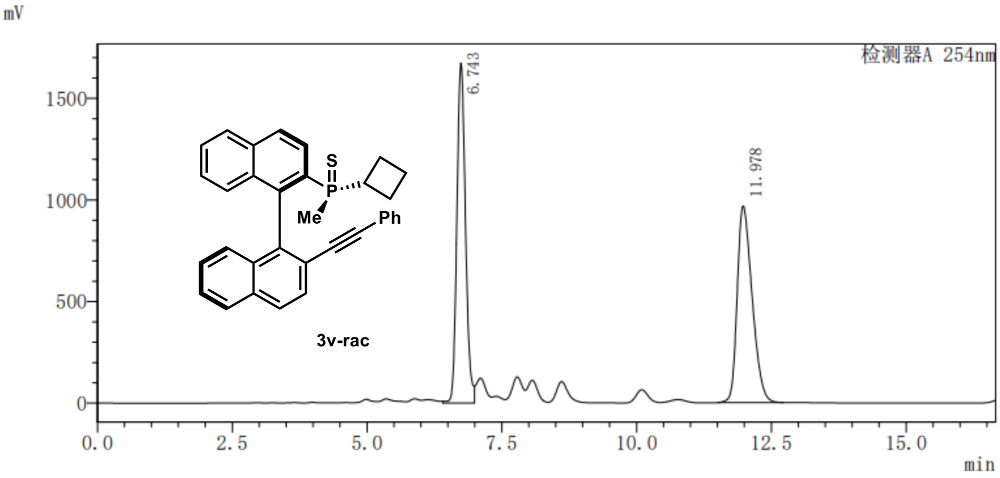

〈峰表〉

检测器A 254nm

峰号	保留时间	面积	高度	浓度	浓度单位	标记	化合物名
1	6.743	18552612	1674062	49.900			
2	11.978	18627090	967264	50.100		M	
总计		37179702	2641327				

C:\LabSolutions\Sample\PLZ-8-97A-IA10%1.lcd

Supplementary Fig. 372. HPLC of **3v-rac.**

SHIMADZU LabSolutions 分析报告

〈样品信息〉

样品名	:		
样品ID	: WXB-220418-37-1		
数据文件名	: PLZ-8-95A-IA10%2.lcd		
方法文件名	: 1.1cm		
批处理文件名	:		
样品瓶号	: 1-1	样品类型	: 未知
进样体积	: 1 uL		
分析日期	: 2022/7/25 19:07:26	分析者	: System Administrator
处理日期	: 2022/7/25 19:23:45	处理者	: System Administrator

〈色谱图〉

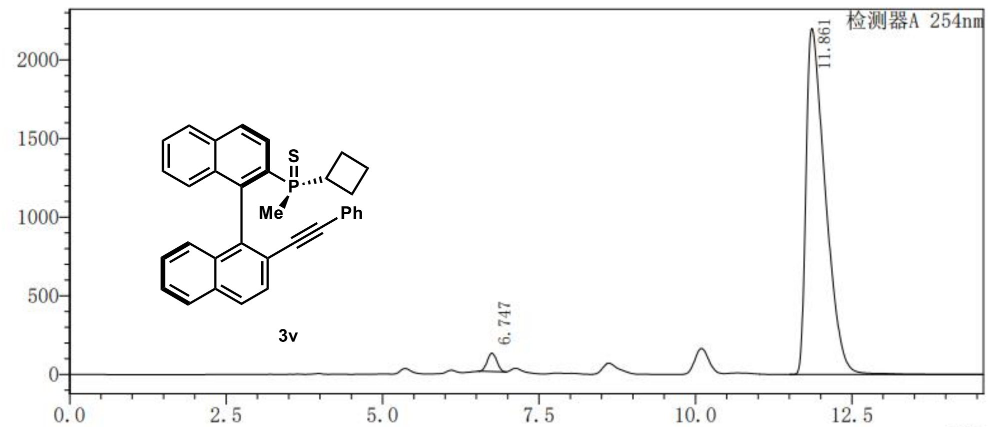

3v

〈峰表〉

检测器A 254nm

峰号	保留时间	面积	高度	浓度	浓度单位	标记	化合物名
1	6.747	1238233	116481	2.553		M	
2	11.861	47259064	2199173	97.447			
总计		48497297	2315655				

C:\LabSolutions\Sample\PLZ-8-95A-IA10%2.lcd

Supplementary Fig. 373. HPLC of **3v.**

SHIMADZU LabSolutions 分析报告

〈样品信息〉

样品名	: ZZY-4Br		
样品ID	: WXB-220418-37-1		
数据文件名	: PLZ-8-104A-IA10%1.lcd		
方法文件名	: 1.1cm		
批处理文件名	:		
样品瓶号	: 1-1	样品类型	: 未知
进样体积	: 1 uL		
分析日期	: 2022/7/28 13:09:13	分析者	: System Administrator
处理日期	: 2022/7/28 13:39:40	处理者	: System Administrator

〈色谱图〉

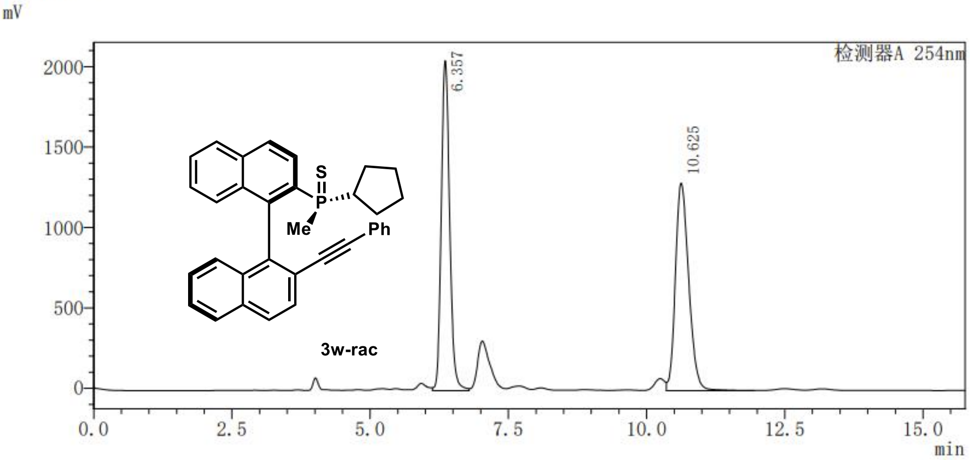

〈峰表〉

检测器A 254nm

峰号	保留时间	面积	高度	浓度	浓度单位	标记	化合物名
1	6.357	21583856	2050675	49.983			
2	10.625	21598451	1290329	50.017			
总计		43182307	3341004				

C:\LabSolutions\Sample\PLZ-8-104A-IA10%1.lcd

Supplementary Fig. 374. HPLC of **3w-rac.**

SHIMADZU LabSolutions 分析报告

<样品信息>

样品名	: WXB-220724-125-5		
样品ID	: WXB-220724-125-8		
数据文件名	: PLZ-8-106A-IA10%.lcd		
方法文件名	: 1.1cm		
批处理文件名	:		
样品瓶号	: 1-1	样品类型	: 未知
进样体积	: 1 uL		
分析日期	: 2022/7/30 10:18:31	分析者	: System Administrator
处理日期	: 2022/7/30 11:48:03	处理者	: System Administrator

<色谱图>

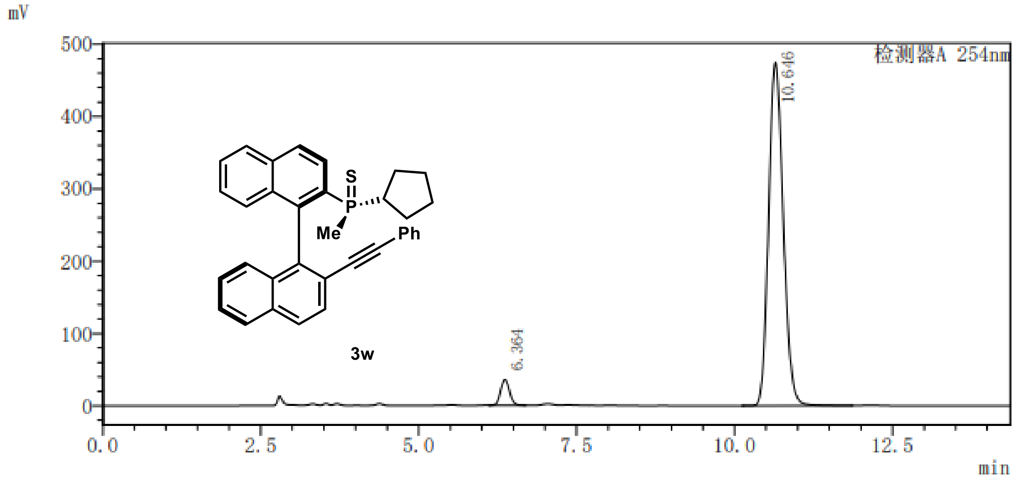

<峰表>

检测器A 254nm

峰号	保留时间	面积	高度	浓度	浓度单位	标记	化合物名
1	6.364	348228	35460	4.344		M	
2	10.646	7667163	474711	95.656		M	
总计		8015391	510171				

C:\LabSolutions\Sample\PLZ-8-106A-IA10%.lcd

Supplementary Fig. 375. HPLC of **3w.**

SHIMADZU LabSolutions 分析报告

<样品信息>

样品名	: ZZY-4Br		
样品ID	: WXB-220418-37-1		
数据文件名	: PLZ-8-101A-IA10%1.lcd		
方法文件名	: 1.1cm		
批处理文件名	:		
样品瓶号	: 1-1	样品类型	: 未知
进样体积	: 1 uL		
分析日期	: 2022/7/28 12:13:17	分析者	: System Administrator
处理日期	: 2022/8/2 10:21:04	处理者	: System Administrator

<色谱图>

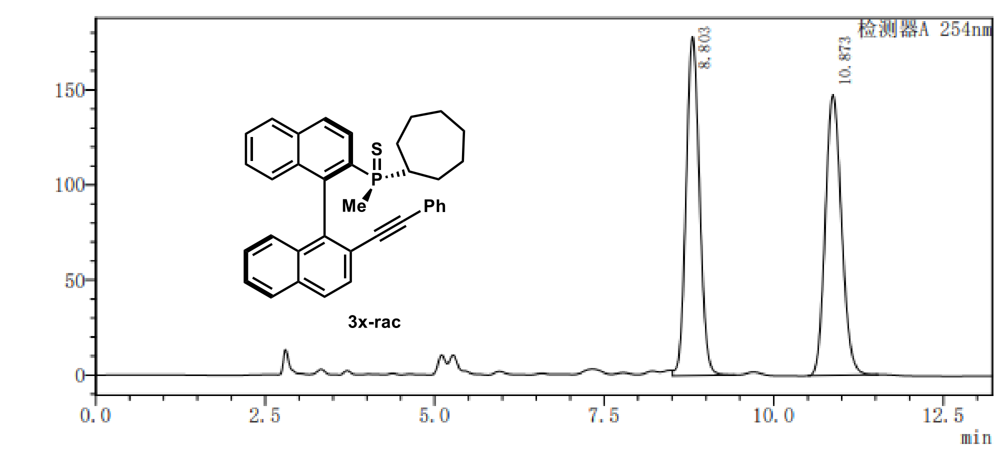

<峰表>

检测器A 254nm

峰号	保留时间	面积	高度	浓度	浓度单位	标记	化合物名
1	8.803	2438122	178047	49.867		M	
2	10.873	2451105	147494	50.133		M	
总计		4889227	325542				

C:\LabSolutions\Sample\PLZ-8-101A-IA10%1.lcd

Supplementary Fig. 376. HPLC of **3x-rac.**

SHIMADZU LabSolutions 分析报告

<样品信息>

样品名	: ZZY-4Br		
样品ID	: WXB-220418-37-1		
数据文件名	: PLZ-8-99A-IA10%1.lcd		
方法文件名	: 1.1cm		
批处理文件名	:		
样品瓶号	: 1-1	样品类型	: 未知
进样体积	: 1 uL		
分析日期	: 2022/7/28 12:27:13	分析者	: System Administrator
处理日期	: 2022/8/3 18:31:42	处理者	: System Administrator

<色谱图>

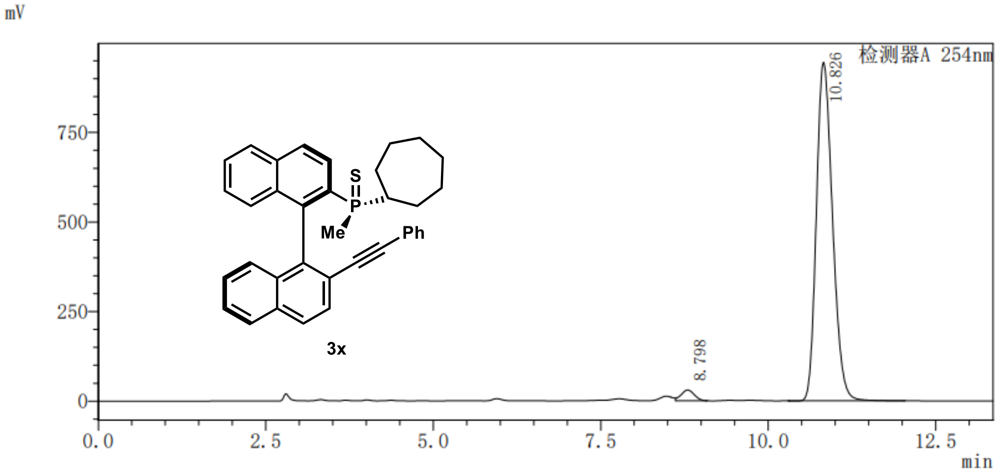

<峰表>

检测器A 254nm

峰号	保留时间	面积	高度	浓度	浓度单位	标记	化合物名
1	8.798	400433	29663	2.465		M	
2	10.826	15846982	944796	97.535		M	
总计		16247416	974459				

C:\LabSolutions\Sample\PLZ-8-99A-IA10%1.lcd

Supplementary Fig. 377. HPLC of **3x.**

SHIMADZU LabSolutions 分析报告

〈样品信息〉

样品名	: ZZY-4Br		
样品ID	: WXB-220418-37-1		
数据文件名	: PLZ-8-101C-IA10%1.lcd		
方法文件名	: 1.1cm		
批处理文件名	:		
样品瓶号	: 1-1	样品类型	: 未知
进样体积	: 1 uL		
分析日期	: 2022/7/28 14:16:19	分析者	: System Administrator
处理日期	: 2022/8/2 10:07:58	处理者	: System Administrator

〈色谱图〉

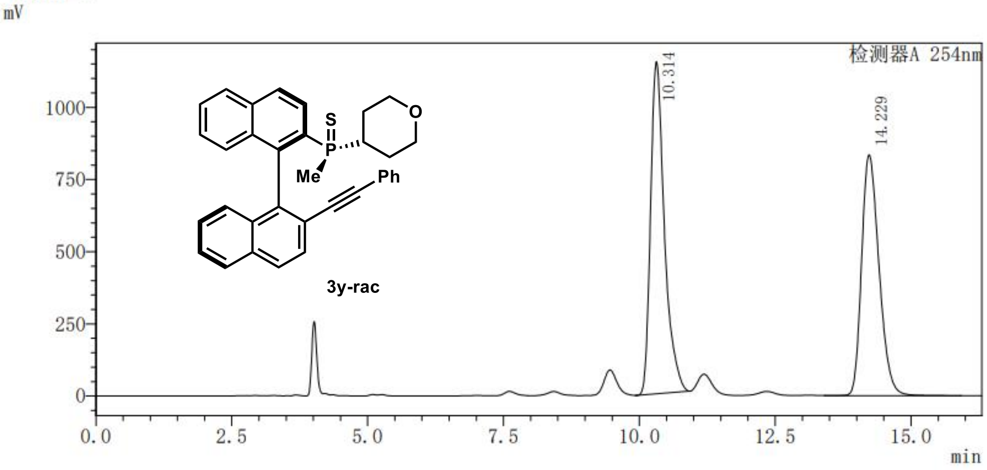

〈峰表〉

检测器A 254nm

峰号	保留时间	面积	高度	浓度	浓度单位	标记	化合物名
1	10.314	20408709	1149942	52.188		M	
2	14.229	18697354	834957	47.812			
总计		39106063	1984899				

C:\LabSolutions\Sample\PLZ-8-101C-IA10%1.lcd

Supplementary Fig. 378. HPLC of **3y-rac.**

SHIMADZU LabSolutions 分析报告

<样品信息>

样品名	: ZZY-4Br		
样品ID	: WXB-220418-37-1		
数据文件名	: PLZ-8-99C-IA10%1.lcd		
方法文件名	: 1.1cm		
批处理文件名	:		
样品瓶号	: 1-1	样品类型	: 未知
进样体积	: 1 uL		
分析日期	: 2022/7/28 14:33:15	分析者	: System Administrator
处理日期	: 2022/7/28 14:49:59	处理者	: System Administrator

<色谱图>

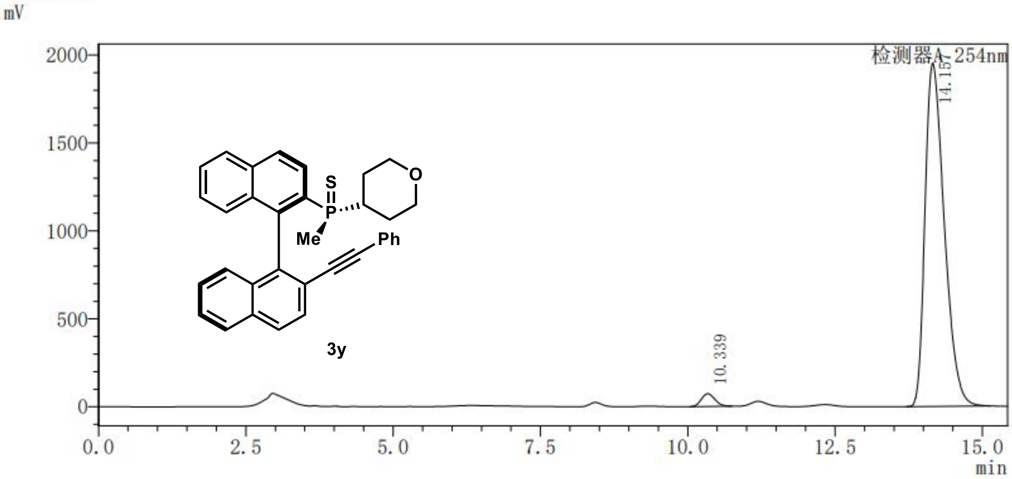

<峰表>

检测器A 254nm

峰号	保留时间	面积	高度	浓度	浓度单位	标记	化合物名
1	10.339	1164537	73002	2.557		M	
2	14.157	44385527	1951507	97.443		M	
总计		45550064	2024509				

C:\LabSolutions\Sample\PLZ-8-99C-IA10%1.lcd

Supplementary Fig. 379. HPLC of **3y.**

SHIMADZU LabSolutions 分析报告

<样品信息>

样品名 :
样品ID : WXB-220418-37-1
数据文件名 : PLZ-8-97B-IA10%3.lcd
方法文件名 : 1.1cm
批处理文件名 :
样品瓶号 : 1-1 样品类型 : 未知
进样体积 : 1 uL
分析日期 : 2022/7/25 19:22:57 分析者 : System Administrator
处理日期 : 2022/8/2 9:56:41 处理者 : System Administrator

<色谱图>

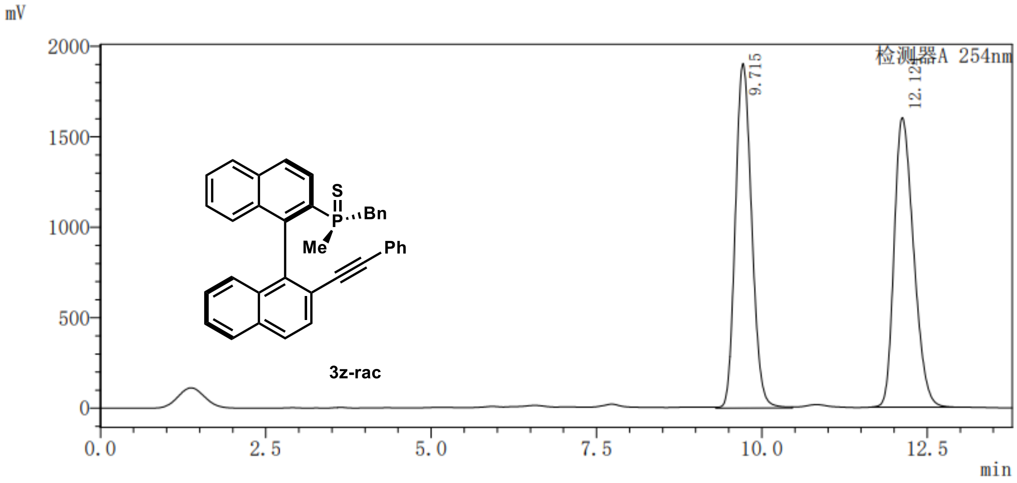

<峰表>

检测器A 254nm

峰号	保留时间	面积	高度	浓度	浓度单位	标记	化合物名
1	9.715	32704285	1903806	49.950			
2	12.124	32769544	1600264	50.050		M	
总计		65473829	3504069				

C:\LabSolutions\Sample\PLZ-8-97B-IA10%3.lcd

Supplementary Fig. 380. HPLC of **3z-rac.**

SHIMADZU LabSolutions 分析报告

〈样品信息〉

样品名	:		
样品ID	: WXB-220418-37-1		
数据文件名	: PLZ-8-95B-IA10%4.lcd		
方法文件名	: 1.1cm		
批处理文件名	:		
样品瓶号	: 1-1	样品类型	: 未知
进样体积	: 1 uL		
分析日期	: 2022/7/25 19:37:17	分析者	: System Administrator
处理日期	: 2022/7/25 19:55:37	处理者	: System Administrator

〈色谱图〉

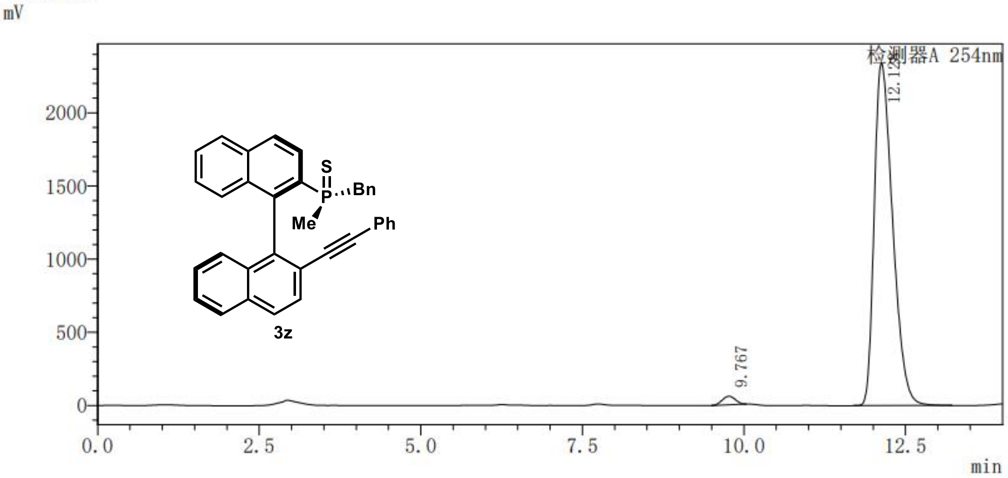

〈峰表〉

检测器A 254nm

峰号	保留时间	面积	高度	浓度	浓度单位	标记	化合物名
1	9.767	823089	58659	1.739		M	
2	12.128	46503538	2341333	98.261		M	
总计		47326628	2399993				

C:\LabSolutions\Sample\PLZ-8-95B-IA10%4.lcd

Supplementary Fig. 381. HPLC of **3z.**

SHIMADZU LabSolutions 分析报告

〈样品信息〉

样品名	:		
样品ID	: WXB-220418-37-1		
数据文件名	: PLZ-8-101B-IA10%2.lcd		
方法文件名	: 1.1cm		
批处理文件名	:		
样品瓶号	: 1-1	样品类型	: 未知
进样体积	: 1 uL		
分析日期	: 2022/7/26 18:18:14	分析者	: System Administrator
处理日期	: 2022/8/2 10:05:20	处理者	: System Administrator

〈色谱图〉

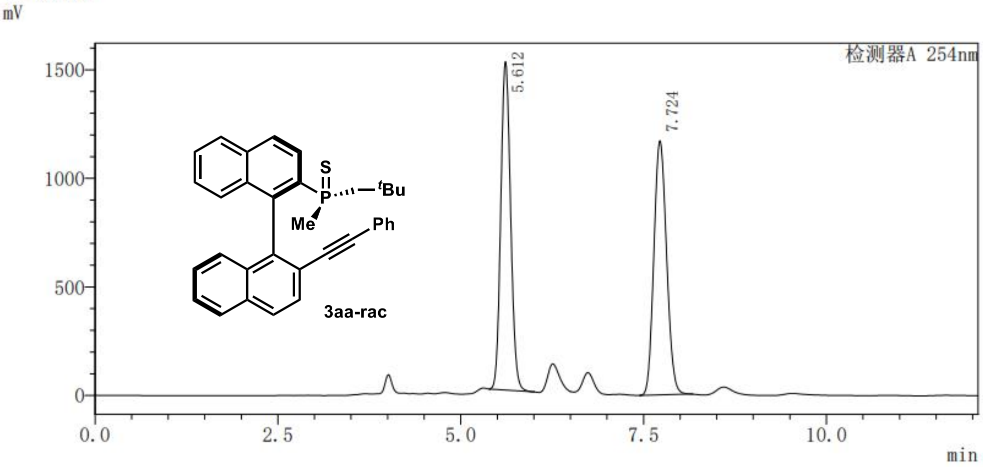

〈峰表〉

检测器A 254nm

峰号	保留时间	面积	高度	浓度	浓度单位	标记	化合物名
1	5.612	14110110	1511512	50.003		M	
2	7.724	14108253	1170587	49.997		M	
总计		28218363	2682099				

C:\LabSolutions\Sample\PLZ-8-101B-IA10%2.lcd

Supplementary Fig. 382. HPLC of **3aa-rac.**

SHIMADZU LabSolutions 分析报告

〈样品信息〉

样品名	: ZZY-4Br		
样品ID	: WXB-220418-37-1		
数据文件名	: PLZ-8-99B-IA10%1.lcd		
方法文件名	: 1.lcm		
批处理文件名	:		
样品瓶号	: 1-1	样品类型	: 未知
进样体积	: 1 uL		
分析日期	: 2022/7/28 12:55:45	分析者	: System Administrator
处理日期	: 2022/8/2 10:04:01	处理者	: System Administrator

〈色谱图〉

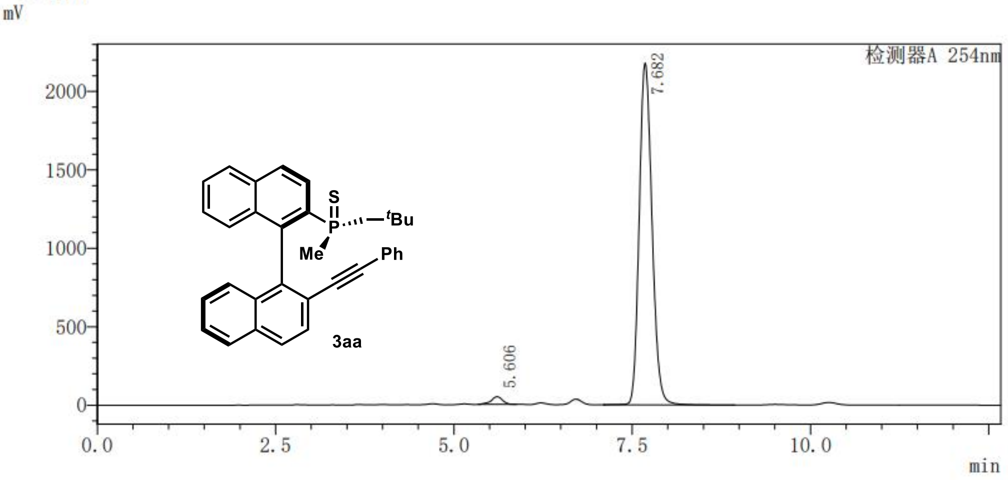

〈峰表〉

检测器A 254nm

峰号	保留时间	面积	高度	浓度	浓度单位	标记	化合物名
1	5.606	541797	49175	1.953		M	
2	7.682	27196550	2178548	98.047		M	
总计		27738347	2227724				

C:\LabSolutions\Sample\PLZ-8-99B-IA10%1.lcd

Supplementary Fig. 383. HPLC of **3aa.**

SHIMADZU LabSolutions 分析报告

〈样品信息〉

样品名	:	YCZ-3-40			
样品ID	:	WXB-220724-125-8			
数据文件名	:	PLZ-8-108A-ID10%5.lcd			
方法文件名	:	1.lcm			
批处理文件名	:				
样品瓶号	:	1-1	样品类型	:	未知
进样体积	:	1 uL			
分析日期	:	2022/8/3 9:28:53	分析者	:	System Administrator
处理日期	:	2022/8/3 10:01:48	处理者	:	System Administrator

〈色谱图〉

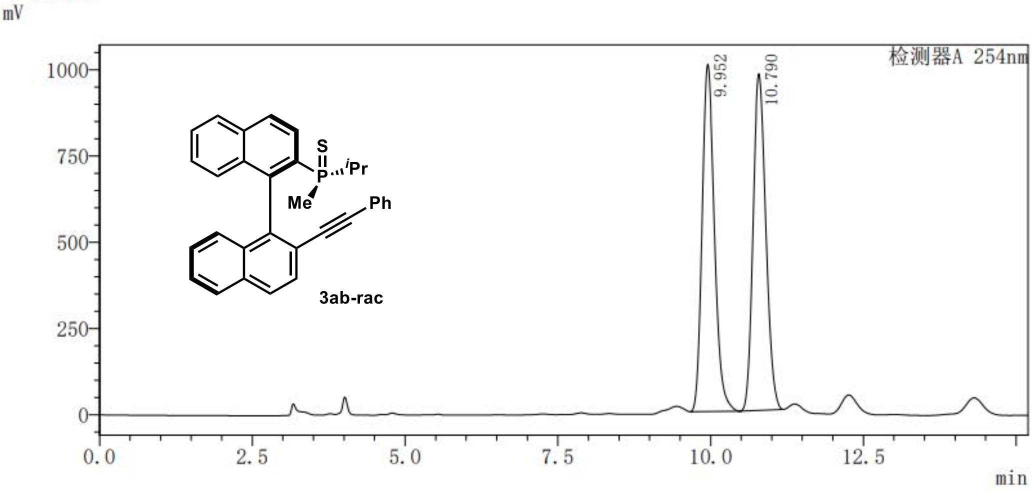

〈峰表〉

检测器A 254nm

峰号	保留时间	面积	高度	浓度	浓度单位	标记	化合物名
1	9.952	14093563	1005633	49.843		M	
2	10.790	14182329	975419	50.157		M	
总计		28275892	1981053				

C:\LabSolutions\Sample\PLZ-8-108A-ID10%5.lcd

Supplementary Fig. 384. HPLC of **3ab-rac.**

SHIMADZU LabSolutions 分析报告

〈样品信息〉

样品名	: YCZ-3-40		
样品ID	: WXB-220724-125-8		
数据文件名	: PLZ-8-107A-ID10%6.lcd		
方法文件名	: 1.1cm		
批处理文件名	:		
样品瓶号	: 1-1	样品类型	: 未知
进样体积	: 1 uL		
分析日期	: 2022/8/3 9:46:56	分析者	: System Administrator
处理日期	: 2022/8/3 10:01:17	处理者	: System Administrator

〈色谱图〉

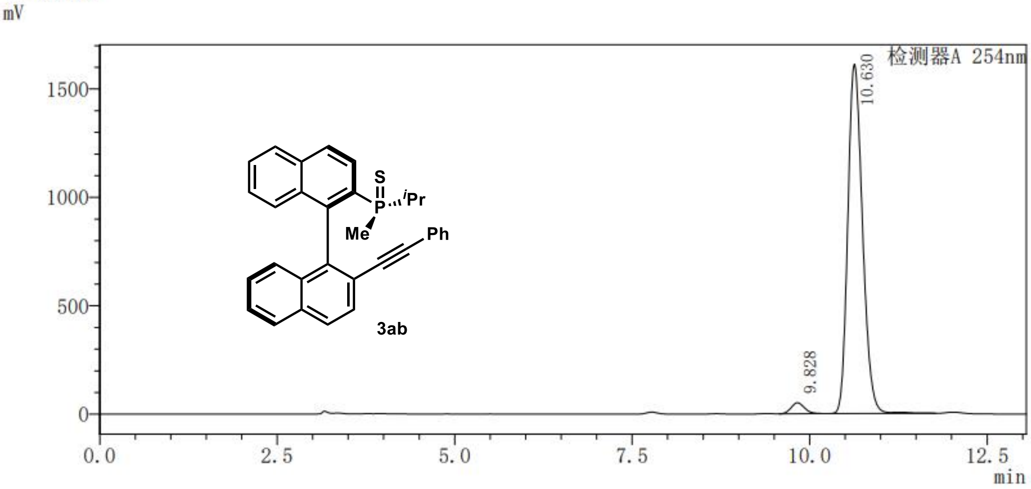

〈峰表〉

检测器A 254nm

峰号	保留时间	面积	高度	浓度	浓度单位	标记	化合物名
1	9.828	650382	50150	2.725		M	
2	10.630	23220626	1610722	97.275		M	
总计		23871008	1660872				

C:\LabSolutions\Sample\PLZ-8-107A-ID10%6.lcd

Supplementary Fig. 385. HPLC of **3ab.**

SHIMADZU LabSolutions 分析报告

<样品信息>

样品名	: YCZ-3-40		
样品ID	: WXB-220724-125-8		
数据文件名	: PLZ-8-108B-IA10%001.lcd		
方法文件名	: 1.lcm		
批处理文件名	:		
样品瓶号	: 1-1	样品类型	: 未知
进样体积	: 1 uL		
分析日期	: 2022/7/30 14:31:40	分析者	: System Administrator
处理日期	: 2022/7/30 16:35:56	处理者	: System Administrator

<色谱图>

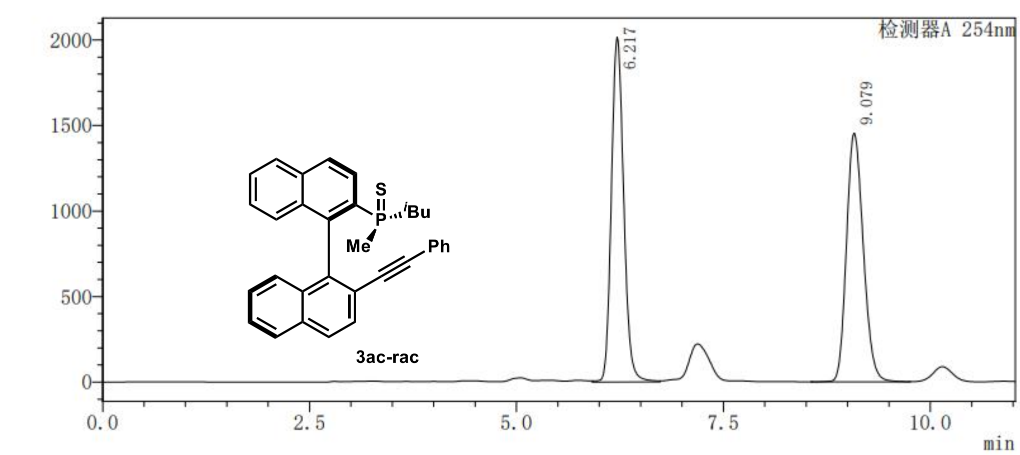

<峰表>

检测器A 254nm

峰号	保留时间	面积	高度	浓度	浓度单位	标记	化合物名
1	6.217	20876374	2015612	50.318			
2	9.079	20612160	1453142	49.682			
总计		41488534	3468754				

C:\LabSolutions\Sample\PLZ-8-108B-IA10%001.lcd

Supplementary Fig. 386. HPLC of **3ac-rac.**

SHIMADZU LabSolutions 分析报告

<样品信息>

样品名	: YCZ-3-40		
样品ID	: WXB-220724-125-8		
数据文件名	: PLZ-8-107B-IA10%.lcd		
方法文件名	: 1.1cm		
批处理文件名	:		
样品瓶号	: 1-1	样品类型	: 未知
进样体积	: 1 uL		
分析日期	: 2022/7/30 14:19:05	分析者	: System Administrator
处理日期	: 2022/8/2 10:16:02	处理者	: System Administrator

<色谱图>

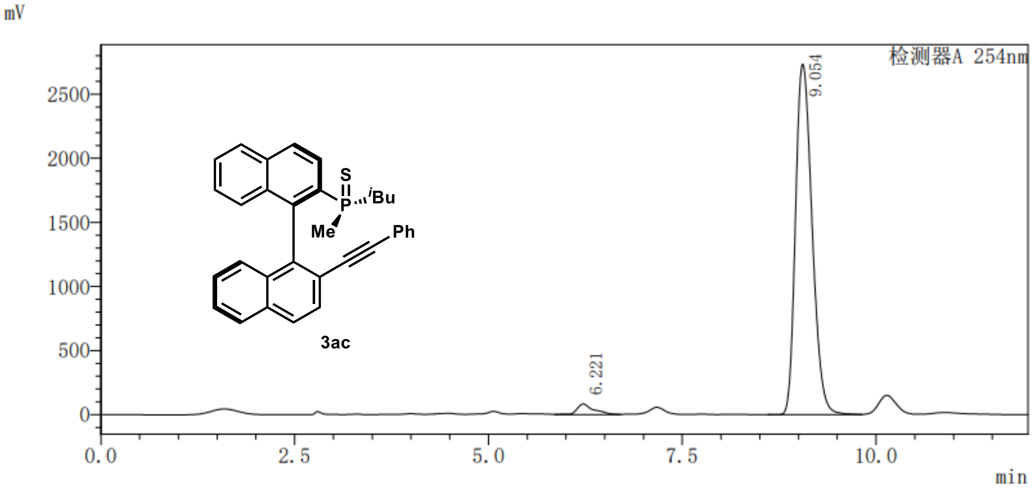

<峰表>

检测器A 254nm

峰号	保留时间	面积	高度	浓度	浓度单位	标记	化合物名
1	6.221	1322487	84018	3.138			
2	9.054	40816797	2734485	96.862			
总计		42139285	2818503				

C:\LabSolutions\Sample\PLZ-8-107B-IA10%.lcd

Supplementary Fig. 387. HPLC of **3ac.**

SHIMADZU LabSolutions 分析报告

<样品信息>

样品名	: YCZ-3-40		
样品ID	: WXB-220724-125-8		
数据文件名	: PLZ-8-104E-IA10%.lcd		
方法文件名	: 1.1cm		
批处理文件名	:		
样品瓶号	: 1-1	样品类型	: 未知
进样体积	: 1 uL		
分析日期	: 2022/7/30 13:37:09	分析者	: System Administrator
处理日期	: 2022/7/30 13:52:41	处理者	: System Administrator

<色谱图>

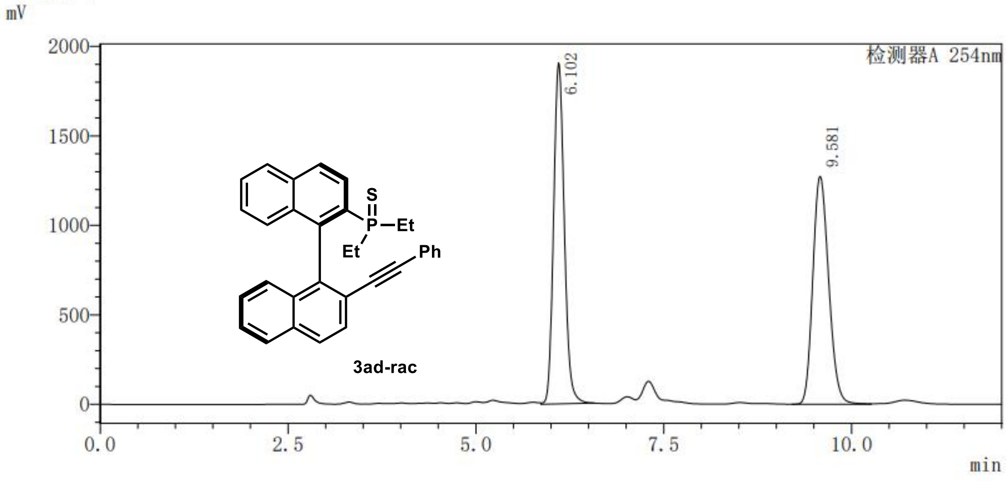

<峰表>

检测器A 254nm

峰号	保留时间	面积	高度	浓度	浓度单位	标记	化合物名
1	6.102	18830359	1904696	50.256		M	
2	9.581	18638169	1273611	49.744			
总计		37468528	3178307				

C:\LabSolutions\Sample\PLZ-8-104E-IA10%.lcd

Supplementary Fig. 388. HPLC of **3ad-rac.**

SHIMADZU LabSolutions 分析报告

〈样品信息〉

样品名 : YCZ-3-40
样品ID : WXB-220724-125-8
数据文件名 : PLZ-8-106E-IA10%.lcd
方法文件名 : 1.lcm
批处理文件名 :
样品瓶号 : 1-1　　样品类型 : 未知
进样体积 : 1 uL
分析日期 : 2022/7/30 13:23:53　　分析者 : System Administrator
处理日期 : 2022/8/1 9:18:34　　处理者 : System Administrator

〈色谱图〉

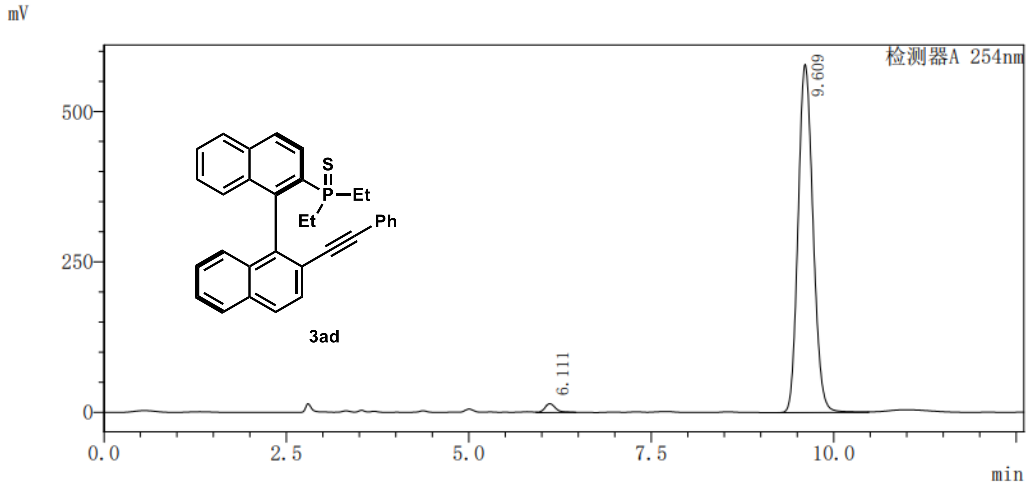

〈峰表〉

检测器A 254nm

峰号	保留时间	面积	高度	浓度	浓度单位	标记	化合物名
1	6.111	147751	14611	1.760		M	
2	9.609	8247665	578320	98.240			
总计		8395416	592931				

C:\LabSolutions\Sample\PLZ-8-106E-IA10%.lcd

Supplementary Fig. 389. HPLC of **3ad.**

??????

Project Name WR

Reported by User: Breeze user (Breeze)

Breeze 2 HPLC System

SAMPLE INFORMATION

Sample Name:	PLZ-7-50D-IE10%	Acquired By:	Breeze
Sample Type:	Unknown	Date Acquired:	7/2/2022 7:30:31 AM CST
Vial:	1	Acq. Method:	10% 254nm
Injection #:	1	Date Processed:	7/2/2022 7:44:50 AM CST
Injection Volume:	20.00 ul	Channel Name:	2998 Ch1 254nm@1.2nm
Run Time:	60.00 Minutes	Sample Set Name	

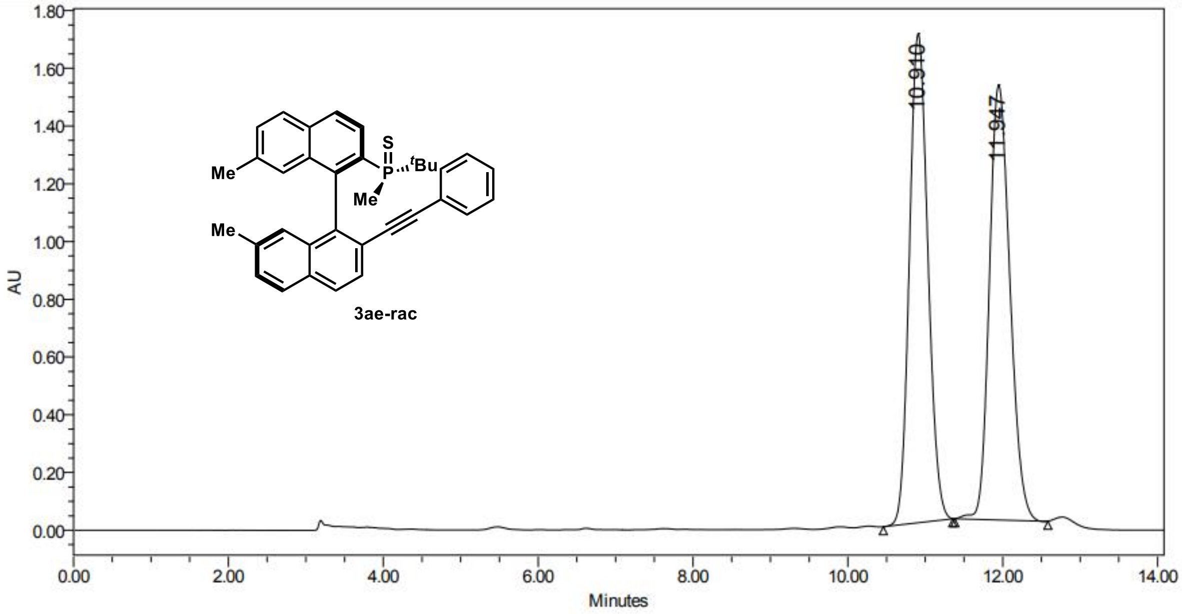

	RT (min)	Area (μV*sec)	% Area	Height (μV)	% Height
1	10.910	28014447	50.19	1694553	52.93
2	11.947	27800519	49.81	1506755	47.07

Report Method: Untitled

Page: 1 of 1

Printed: 7/2/2022 8:29:18 AM PRC

Supplementary Fig. 390. HPLC of **3ae-rac.**

??????

Project Name WR

Reported by User: Breeze user (Breeze)

SAMPLE INFORMATION

Sample Name:	PLZ-7-125C-IE10%	Acquired By:	Breeze
Sample Type:	Unknown	Date Acquired:	7/2/2022 7:47:59 AM CST
Vial:	1	Acq. Method:	10% 254nm
Injection #:	2	Date Processed:	7/2/2022 8:28:36 AM CST
Injection Volume:	20.00 ul	Channel Name:	2998 Ch1 254nm@1.2nm
Run Time:	60.00 Minutes	Sample Set Name	

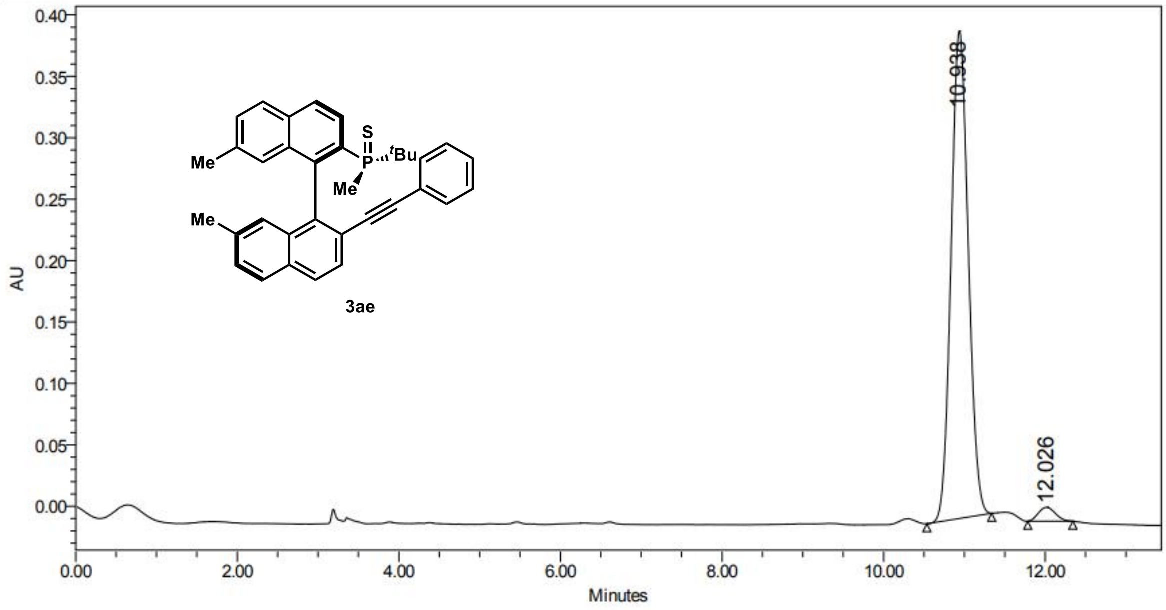

	RT (min)	Area (μV*sec)	% Area	Height (μV)	% Height
1	10.938	5860870	97.25	396788	97.22
2	12.026	165510	2.75	11354	2.78

Report Method: Untitled
Page: 1 of 1

Printed: 7/2/2022
8:29:54 AM PRC

Supplementary Fig. 391. HPLC of **3ae.**

??????

Project Name WR

Reported by User: Breeze user (Breeze)

SAMPLE INFORMATION

Sample Name:	PLZ-7-50B-IA10%	Acquired By:	Breeze
Sample Type:	Unknown	Date Acquired:	6/30/2022 7:02:32 AM CST
Vial:	1	Acq. Method:	10% 254nm
Injection #:	10	Date Processed:	6/30/2022 7:32:49 AM CST
Injection Volume:	20.00 ul	Channel Name:	2998 Ch1 254nm@1.2nm
Run Time:	60.00 Minutes	Sample Set Name	

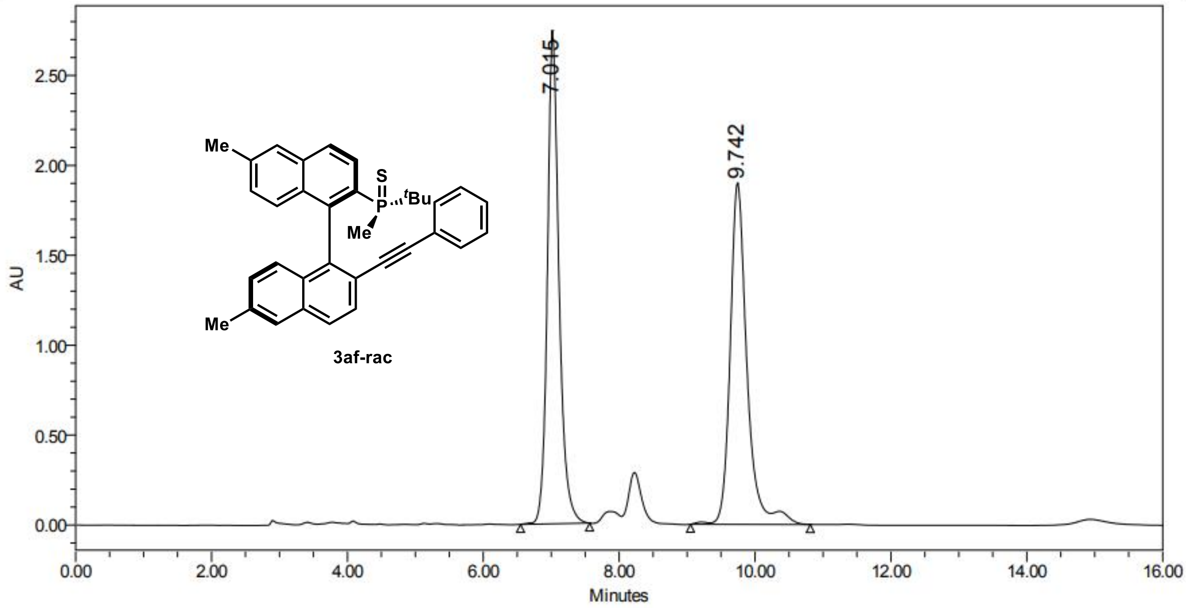

	RT (min)	Area (µV*sec)	% Area	Height (µV)	% Height
1	7.015	32526999	49.79	2744386	59.12
2	9.742	32805245	50.21	1897508	40.88

Report Method: Untitled
Page: 1 of 1

Printed: 7/9/2022
12:46:07 AM PRC

Supplementary Fig. 392. HPLC of **3af-rac.**

??????

Project Name WR

Reported by User: Breeze user (Breeze)

Breeze 2 HPLC System

SAMPLE INFORMATION

Sample Name:	PLZ-7-141B-IA10%	Acquired By:	Breeze
Sample Type:	Unknown	Date Acquired:	6/30/2022 7:20:52 AM CST
Vial:	1	Acq. Method:	10% 254nm
Injection #:	11	Date Processed:	6/30/2022 7:32:13 AM CST
Injection Volume:	20.00 ul	Channel Name:	2998 Ch1 254nm@1.2nm
Run Time:	60.00 Minutes	Sample Set Name	

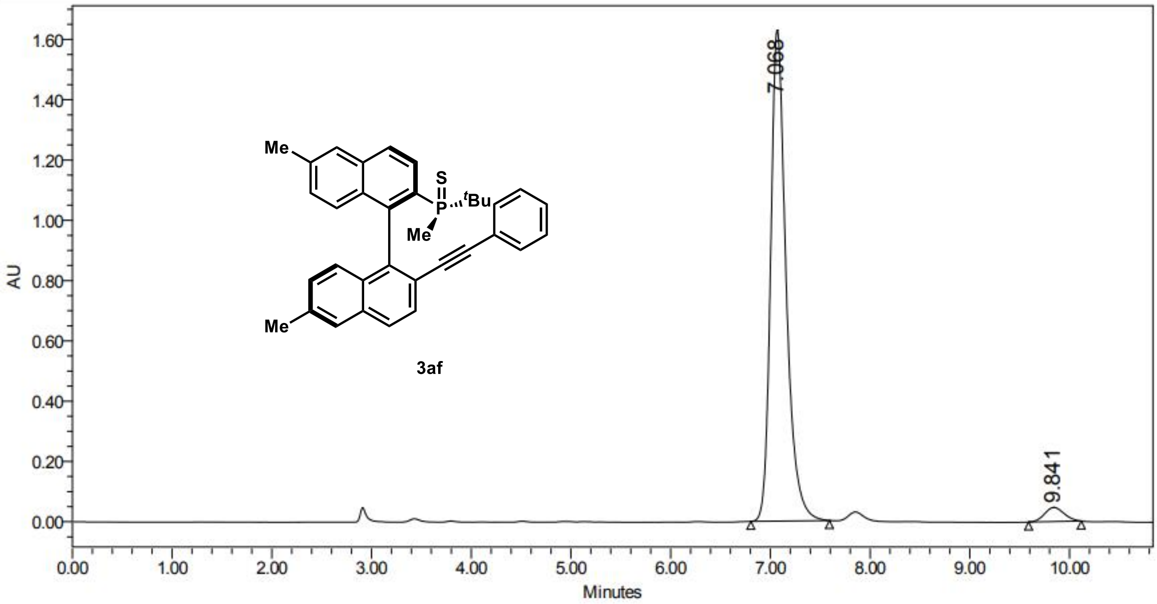

	RT (min)	Area (µV*sec)	% Area	Height (µV)	% Height
1	7.068	17511891	96.50	1628400	97.21
2	9.841	635457	3.50	46770	2.79

Report Method: Untitled

Page: 1 of 1

Printed: 7/9/2022
12:45:39 AM PRC

Supplementary Fig. 393. HPLC of **3af.**

??????

Project Name lk

Reported by User: Breeze user (Breeze)

SAMPLE INFORMATION

Sample Name:	PLZ-7-50C-ID10%	Acquired By:	Breeze
Sample Type:	Unknown	Date Acquired:	5/25/2022 6:32:00 AM CST
Vial:	1	Acq. Method:	10% 254nm
Injection #:	1	Processed By:	Breeze
Injection Volume:	20.00 ul	Date Processed:	5/25/2022 7:01:16 AM CST
Run Time:	60.00 Minutes	Channel Name:	2998 Ch1 254nm@1.2nm
Sampling Rate:	10.00 per sec	Channel Desc.:	2998 Ch1 254nm@1.2nm
		Sample Set Name	

Sample Values Used in Calculation:

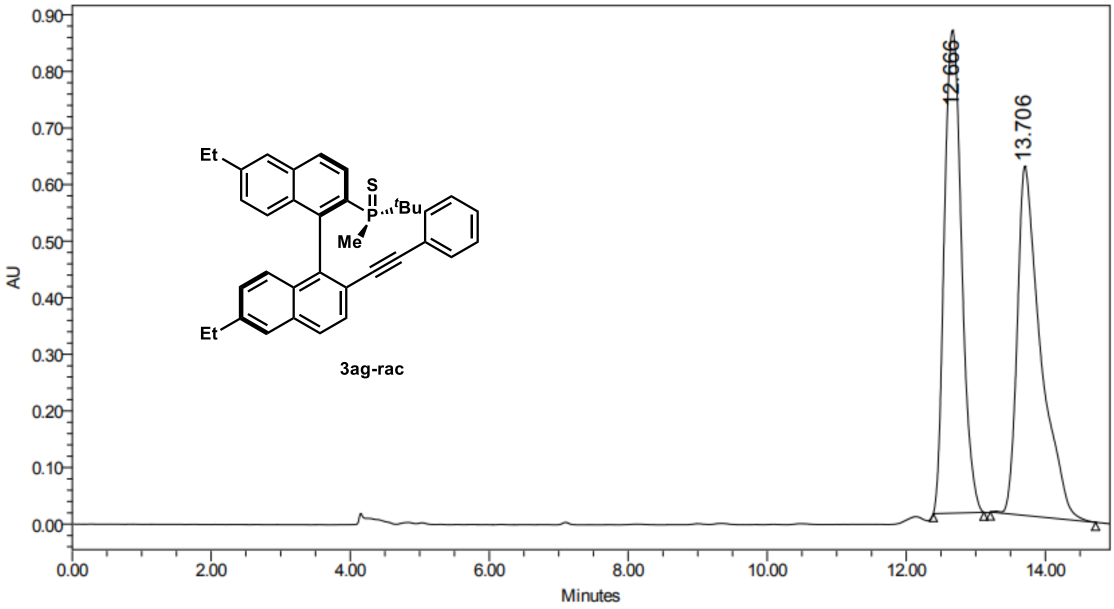

	RT (min)	Peak Type	Area (µV*sec)	% Area	Height (µV)	% Height	Integration Type	Points Across Peak	Start Time (min)	End Time (min)
1	12.666	Unknown	14706190	50.43	853325	58.03	bb	436	12.390	13.117
2	13.706	Unknown	14453015	49.57	617236	41.97	bb	909	13.210	14.725

Report Method: Detailed Individual Report

Page: 1 of 2

Printed: 5/25/2022 7:11:39 AM PRC

Supplementary Fig. 394. HPLC of **3ag-rac.**

??????

Project Name lk

Reported by User: Breeze user (Breeze)

SAMPLE INFORMATION

Sample Name:	PLZ-7-108C-ID10%	Acquired By:	Breeze
Sample Type:	Unknown	Date Acquired:	5/25/2022 6:53:48 AM CST
Vial:	1	Acq. Method:	10% 254nm
Injection #:	1	Processed By:	Breeze
Injection Volume:	20.00 ul	Date Processed:	5/25/2022 7:10:54 AM CST
Run Time:	60.00 Minutes	Channel Name:	2998 Ch1 254nm@1.2nm
Sampling Rate:	10.00 per sec	Channel Desc.:	2998 Ch1 254nm@1.2nm
		Sample Set Name	

Sample Values Used in Calculation:

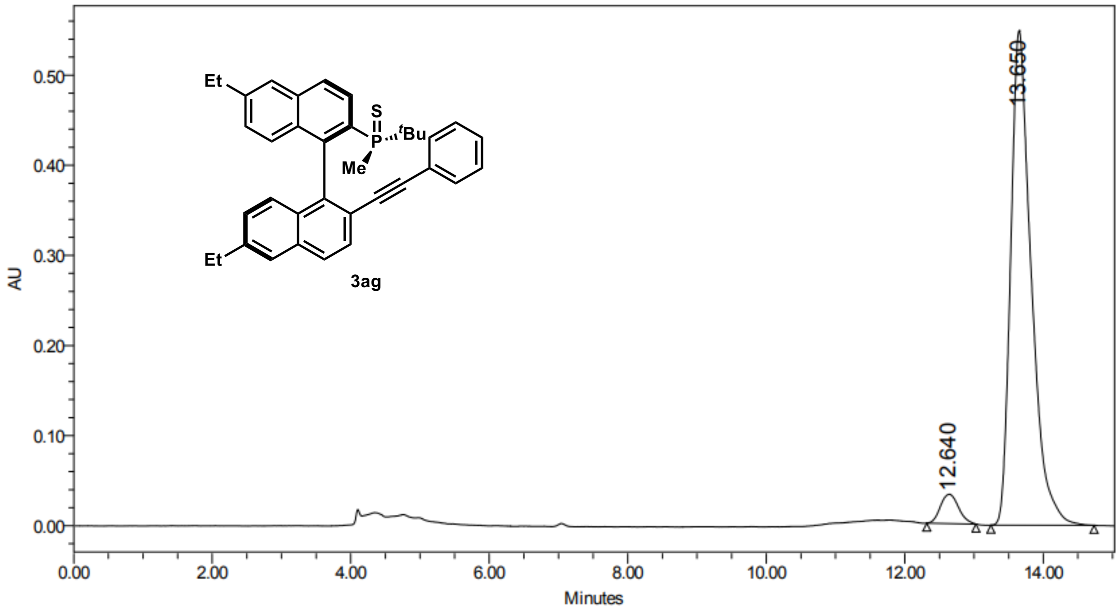

	RT (min)	Peak Type	Area (µV*sec)	% Area	Height (µV)	% Height	Integration Type	Points Across Peak	Start Time (min)	End Time (min)
1	12.640	Unknown	592385	4.98	32486	5.59	bb	426	12.317	13.027
2	13.650	Unknown	11299720	95.02	548973	94.41	bb	895	13.242	14.733

Report Method: Detailed Individual Report

Page: 1 of 2

Printed: 5/25/2022 7:12:16 AM PRC

Supplementary Fig. 395. HPLC of **3ag.**

??????

Project Name lk

Reported by User: Breeze user (Breeze)

SAMPLE INFORMATION

Sample Name:	PLZ-8-36B-IE20%	Acquired By:	Breeze
Sample Type:	Unknown	Date Acquired:	7/17/2022 8:52:00 AM CST
Vial:	1	Acq. Method:	20% 254nm
Injection #:	3	Processed By:	Breeze
Injection Volume:	20.00 ul	Date Processed:	7/17/2022 9:31:14 AM CST
Run Time:	60.00 Minutes	Channel Name:	2998 Ch1 254nm@1.2nm
Sampling Rate:	10.00 per sec	Channel Desc.:	2998 Ch1 254nm@1.2nm
		Sample Set Name	

Sample Values
Used in Calculation:

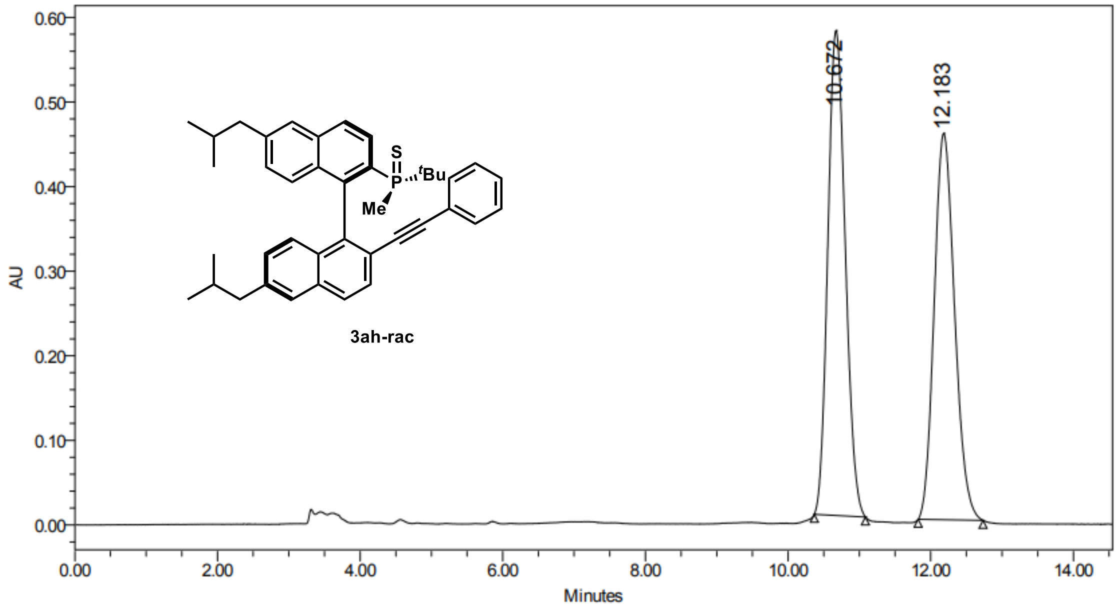

3ah-rac

	RT (min)	Peak Type	Area (µV*sec)	% Area	Height (µV)	% Height	Integration Type	Points Across Peak	Start Time (min)	End Time (min)
1	10.672	Unknown	9724746	51.70	573436	55.67	bb	429	10.368	11.083
2	12.183	Unknown	9086193	48.30	456682	44.33	bb	546	11.823	12.733

Report Method: Detailed Individual Report Printed: 7/17/2022 9:38:31 AM PRC

Page: 1 of 2

Supplementary Fig. 396. HPLC of **3ah-rac.**

??????

Project Name lk

Reported by User: Breeze user (Breeze)

SAMPLE INFORMATION

Sample Name:	PLZ-8-36A-IE20%	Acquired By:	Breeze
Sample Type:	Unknown	Date Acquired:	7/17/2022 9:17:51 AM CST
Vial:	1	Acq. Method:	20% 254nm
Injection #:	4	Processed By:	Breeze
Injection Volume:	20.00 ul	Date Processed:	7/17/2022 9:32:55 AM CST
Run Time:	60.00 Minutes	Channel Name:	2998 Ch1 254nm@1.2nm
Sampling Rate:	10.00 per sec	Channel Desc.:	2998 Ch1 254nm@1.2nm
		Sample Set Name	

Sample Values
Used in Calculation:

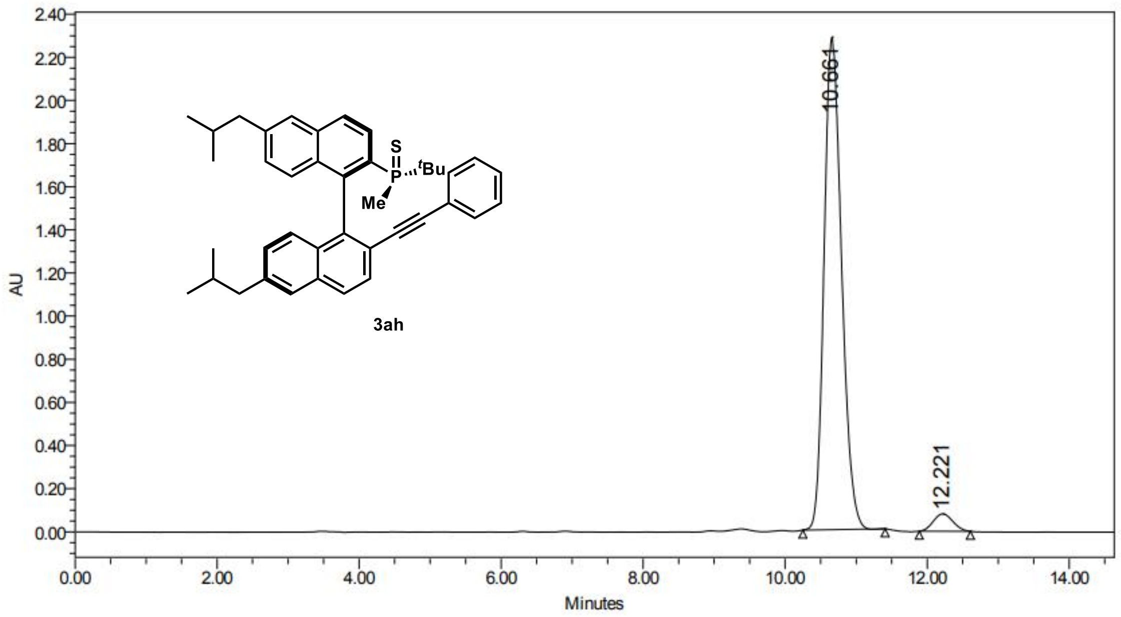

	RT (min)	Peak Type	Area (µV*sec)	% Area	Height (µV)	% Height	Integration Type	Points Across Peak	Start Time (min)
1	10.661	Unknown	39027560	96.19	2283892	96.60	bb	695	10.248
2	12.221	Unknown	1547933	3.81	80485	3.40	bb	432	11.888

Report Method: Detailed Individual Report
Page: 1 of 2

Printed: 7/17/2022
9:34:44 AM PRC

Supplementary Fig. 397. HPLC of **3ah.**

SHIMADZU LabSolutions 分析报告

<样品信息>

样品名	: YCZ-3-40		
样品ID	: WXB-220724-125-8		
数据文件名	: PLZ-8-77B-ID5%9. lcd		
方法文件名	: 1. lcm		
批处理文件名	:		
样品瓶号	: 1-1	样品类型	: 未知
进样体积	: 1 uL		
分析日期	: 2022/8/3 14:52:11	分析者	: System Administrator
处理日期	: 2022/8/3 15:16:13	处理者	: System Administrator

<色谱图>

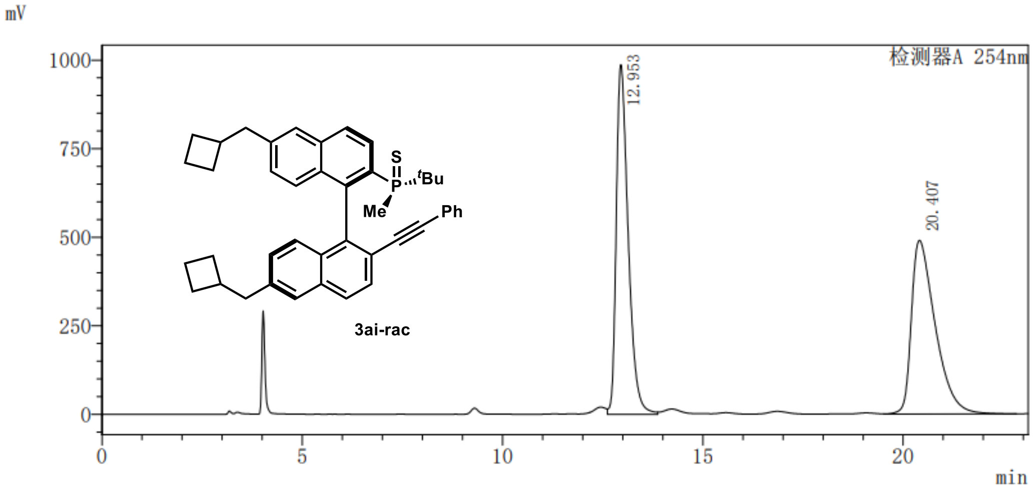

<峰表>

检测器A 254nm

峰号	保留时间	面积	高度	浓度	浓度单位	标记	化合物名
1	12. 953	20425494	986796	50. 194			
2	20. 407	20267209	490433	49. 806			
总计		40692703	1477229				

C:\LabSolutions\Sample\PLZ-8-77B-ID5%9.lcd

Supplementary Fig. 398. HPLC of **3ai-rac.**

SHIMADZU LabSolutions 分析报告

〈样品信息〉

样品名	: YCZ-3-40		
样品ID	: WXB-220724-125-8		
数据文件名	: PLZ-8-81B-ID5%10.lcd		
方法文件名	: 1.1cm		
批处理文件名	:		
样品瓶号	: 1-1	样品类型	: 未知
进样体积	: 1 uL		
分析日期	: 2022/8/3 15:15:54	分析者	: System Administrator
处理日期	: 2022/8/3 15:39:48	处理者	: System Administrator

〈色谱图〉

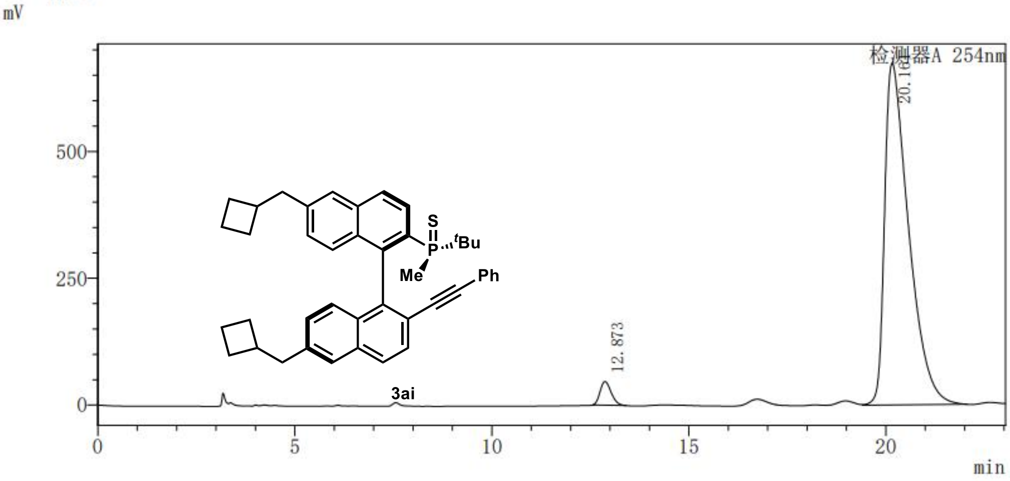

〈峰表〉

检测器A 254nm

峰号	保留时间	面积	高度	浓度	浓度单位	标记	化合物名
1	12.873	913968	46793	3.102		M	
2	20.161	28550123	673829	96.898		M	
总计		29464090	720622				

C:\LabSolutions\Sample\PLZ-8-81B-ID5%10.lcd

Supplementary Fig. 399. HPLC of **3ai.**

??????

Project Name lk

Reported by User: Breeze user (Breeze)

SAMPLE INFORMATION

Sample Name:	PLZ-8-57B-IA10%	Acquired By:	Breeze
Sample Type:	Unknown	Date Acquired:	7/12/2022 1:28:37 PM CST
Vial:	1	Acq. Method:	10% 254nm
Injection #:	3	Processed By:	Breeze
Injection Volume:	20.00 ul	Date Processed:	7/12/2022 1:54:12 PM CST
Run Time:	60.00 Minutes	Channel Name:	2998 Ch1 254nm@1.2nm
Sampling Rate:	10.00 per sec	Channel Desc.:	2998 Ch1 254nm@1.2nm
		Sample Set Name	
Sample Values Used in Calculation:			

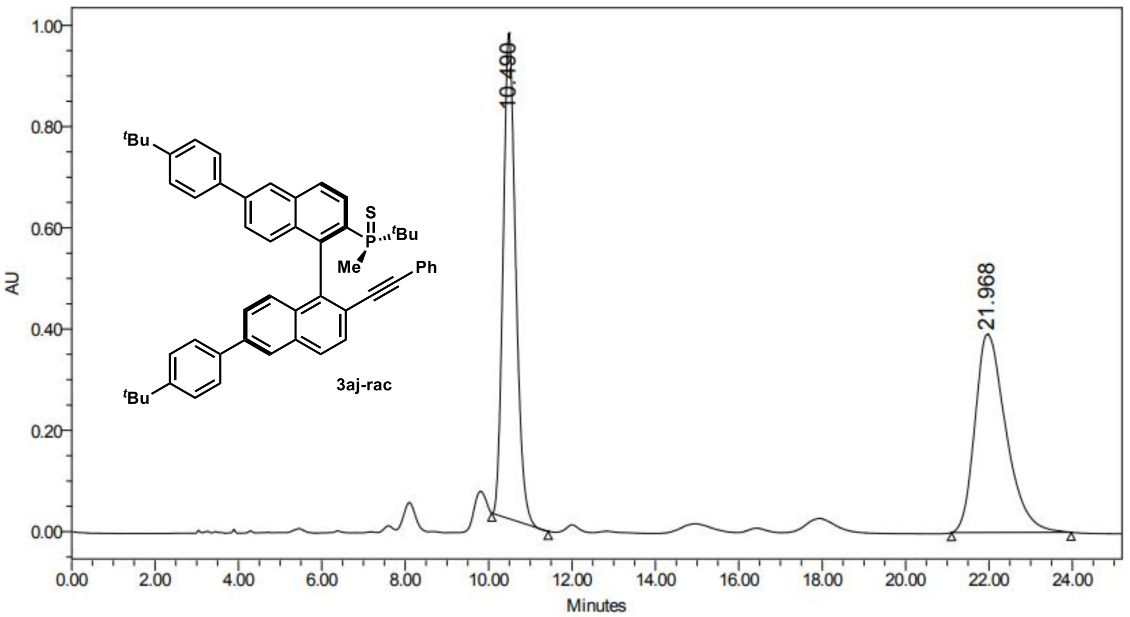

	RT (min)	Peak Type	Area (μV*sec)	% Area	Height (μV)	% Height	Integration Type	Points Across Peak	Start Time (min)	End Time (min)
1	10.490	Unknown	20116754	49.80	959824	71.04	bb	812	10.075	11.428
2	21.968	Unknown	20274580	50.20	391299	28.96	bb	1720	21.103	23.970

Report Method: Detailed Individual Report

Page: 1 of 2

Printed: 7/12/2022 1:54:25 PM PRC

Supplementary Fig. 400. HPLC of **3aj-rac.**

??????

Project Name Ik

Reported by User: Breeze user (Breeze)

SAMPLE INFORMATION

Sample Name:	PLZ-8-57A-IA10%	Acquired By:	Breeze
Sample Type:	Unknown	Date Acquired:	7/12/2022 2:28:45 PM CST
Vial:	1	Acq. Method:	10% 254nm
Injection #:	5	Processed By:	Breeze
Injection Volume:	20.00 ul	Date Processed:	7/12/2022 2:54:47 PM CST
Run Time:	60.00 Minutes	Channel Name:	2998 Ch1 254nm@1.2nm
Sampling Rate:	10.00 per sec	Channel Desc.:	2998 Ch1 254nm@1.2nm
		Sample Set Name	

Sample Values Used in Calculation:

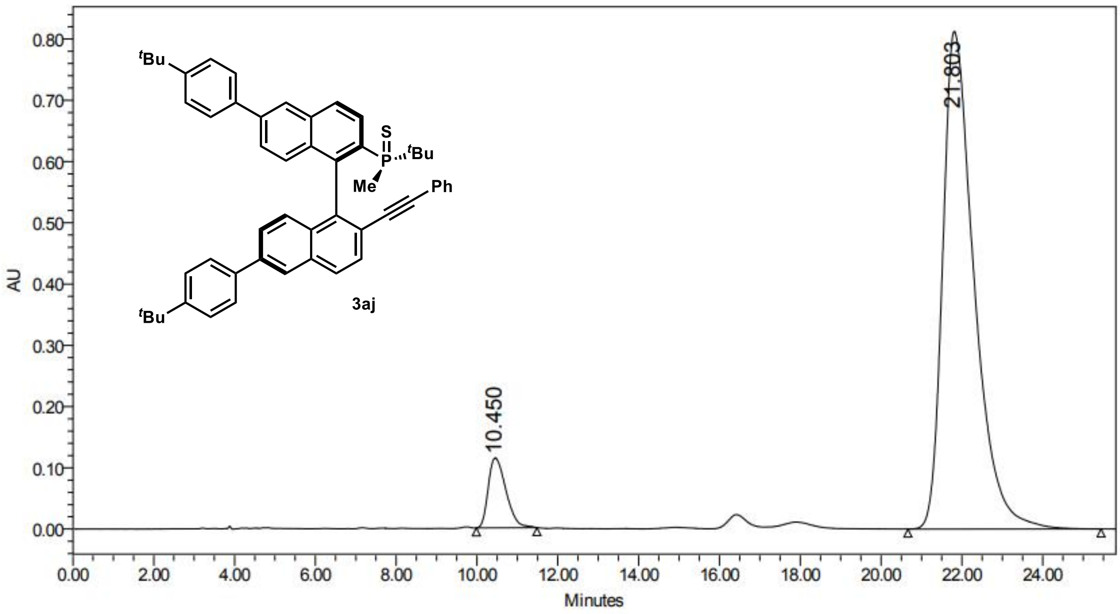

	RT (min)	Peak Type	Area (µV*sec)	% Area	Height (µV)	% Height	Integration Type	Points Across Peak	Start Time (min)	End Time (min)
1	10.450	Unknown	3464372	7.33	113822	12.29	bb	898	9.983	11.480
2	21.803	Unknown	43827130	92.67	812039	87.71	bb	2868	20.663	25.443

Report Method: Detailed Individual Report

Page: 1 of 2

Printed: 7/12/2022 2:55:07 PM PRC

Supplementary Fig. 401. HPLC of **3aj.**

SHIMADZU LabSolutions

分析报告

〈样品信息〉

样品名	: PLZ				
样品ID	: PLZ-220915				
数据文件名	: PLZ-10-183-AS10%230nm9.lcd				
方法文件名	: 322.lcm				
批处理文件名	:				
样品瓶号	: 1-1		样品类型	: 未知	
进样体积	: 10 uL				
分析日期	: 2023/5/6 17:22:46		分析者	: System Administrator	
处理日期	: 2023/5/6 17:49:16		处理者	: System Administrator	

〈色谱图〉

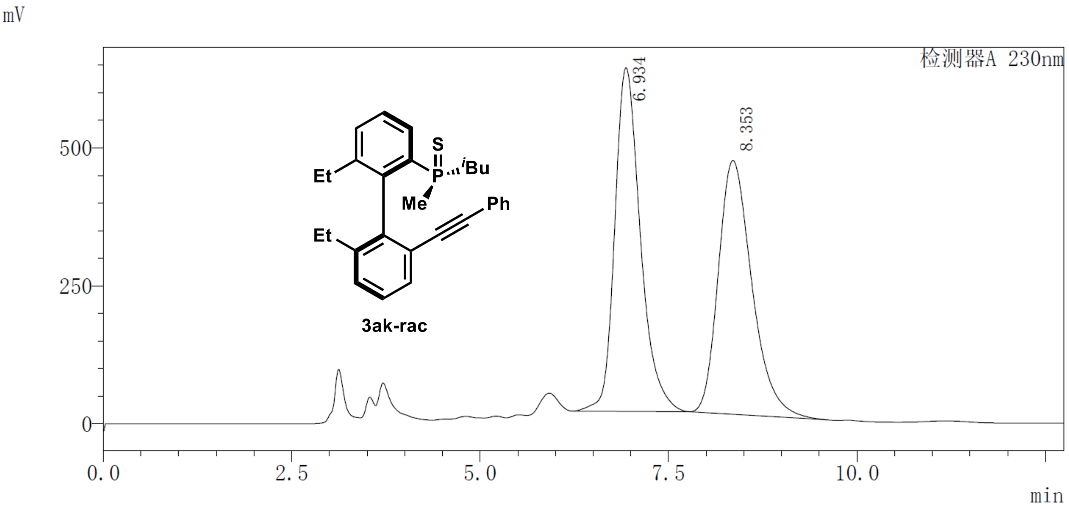

〈峰表〉

检测器A 230nm

峰号	保留时间	面积	高度	浓度	浓度单位	标记	化合物名
1	6.934	14745702	623147	50.822		M	
2	8.353	14268505	460336	49.178		M	
总计		29014206	1083483				

Supplementary Fig. 402. HPLC of **3ak-rac.**

SHIMADZU LabSolutions 分析报告

〈样品信息〉

样品名	: PLZ		
样品ID	: PLZ-220915		
数据文件名	: PLZ-10-179-AS10%230nm10.lcd		
方法文件名	: 322.1cm		
批处理文件名	:		
样品瓶号	: 1-1	样品类型	: 未知
进样体积	: 10 uL		
分析日期	: 2023/5/6 17:36:26	分析者	: System Administrator
处理日期	: 2023/5/6 17:48:18	处理者	: System Administrator

〈色谱图〉

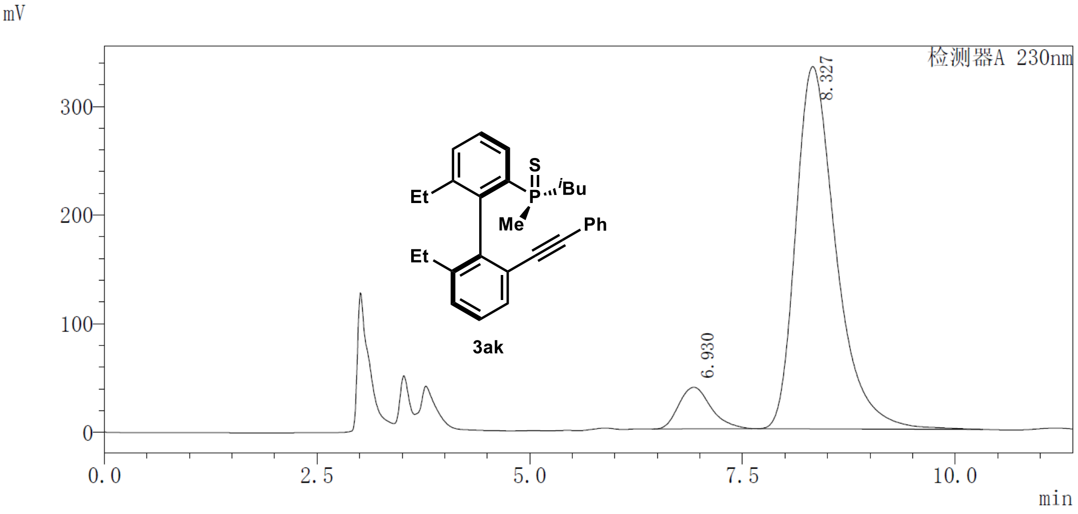

〈峰表〉

检测器A 230nm

峰号	保留时间	面积	高度	浓度	浓度单位	标记	化合物名
1	6.930	1011572	38283	8.434		M	
2	8.327	10982137	333373	91.566		M	
总计		11993709	371656				

Supplementary Fig. 403. HPLC of **3ak.**

SHIMADZU LabSolutions 分析报告

〈样品信息〉

样品名	: PLZ		
样品ID	: PLZ-220915		
数据文件名	: PLZ-10-168-IC5%3.lcd		
方法文件名	: 322.lcm		
批处理文件名	:		
样品瓶号	: 1-1	样品类型	: 未知
进样体积	: 1 uL		
分析日期	: 2023/4/24 10:24:45	分析者	: System Administrator
处理日期	: 2023/4/24 10:45:15	处理者	: System Administrator

〈色谱图〉

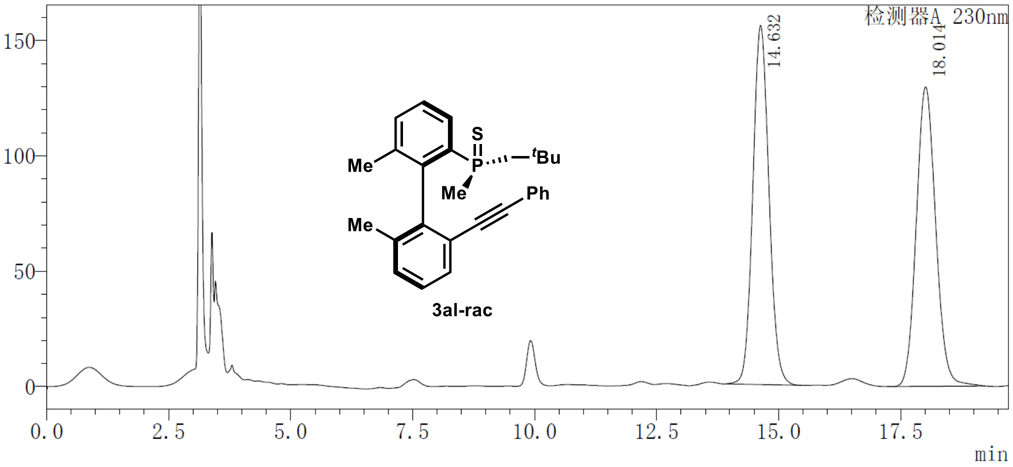

〈峰表〉

检测器A 230nm

峰号	保留时间	面积	高度	浓度	浓度单位	标记	化合物名
1	14.632	3580628	155648	49.906		M	
2	18.014	3594173	129830	50.094		M	
总计		7174802	285478				

Supplementary Fig. 404. HPLC of **3al-rac.**

SHIMADZU LabSolutions 分析报告

〈样品信息〉

样品名	: PLZ		
样品ID	: PLZ-220915		
数据文件名	: PLZ-10-161-IC5%4.lcd		
方法文件名	: 322.1cm		
批处理文件名	:		
样品瓶号	: 1-1	样品类型	: 未知
进样体积	: 1 uL		
分析日期	: 2023/4/24 10:45:34	分析者	: System Administrator
处理日期	: 2023/4/25 15:57:51	处理者	: System Administrator

〈色谱图〉

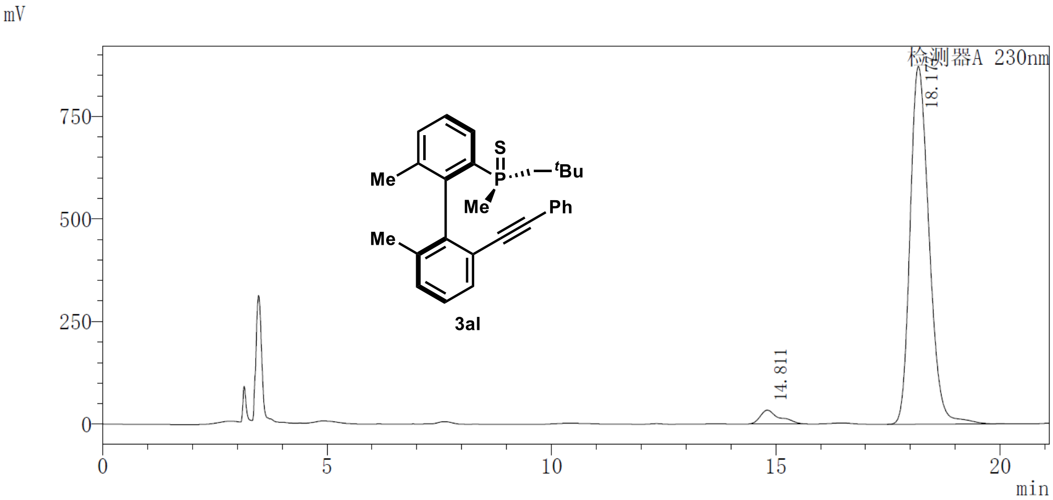

〈峰表〉

检测器A 230nm

峰号	保留时间	面积	高度	浓度	浓度单位	标记	化合物名
1	14.811	979158	33125	3.682		M	
2	18.177	25615765	872360	96.318		M	
总计		26594923	905485				

Supplementary Fig. 405. HPLC of **3al.**

SHIMADZU LabSolutions 分析报告

〈样品信息〉

样品名	: PLZ		
样品ID	: PLZ-220915		
数据文件名	: LG-1-36B-IA2.5%35.lcd		
方法文件名	: 1.1cm		
批处理文件名	:		
样品瓶号	: 1-1	样品类型	: 未知
进样体积	: 1 uL		
分析日期	: 2022/11/30 22:30:24	分析者	: System Administrator
处理日期	: 2022/11/30 22:49:00	处理者	: System Administrator

〈色谱图〉

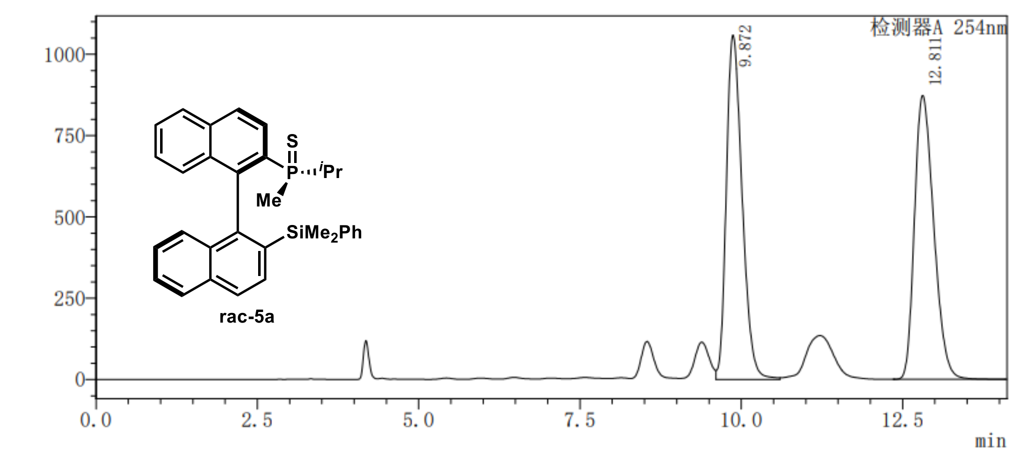

〈峰表〉

检测器A 254nm

峰号	保留时间	面积	高度	浓度	浓度单位	标记	化合物名
1	9.872	18029652	1058453	49.833			
2	12.811	18150315	872589	50.167			
总计		36179968	1931042				

C:\LabSolutions\Sample\LG-1-36B-IA2.5%35.lcd

Supplementary Fig. 406. HPLC of **5a-rac.**

SHIMADZU LabSolutions 分析报告

<样品信息>

样品名	: PLZ		
样品ID	: PLZ-220915		
数据文件名	: PLZ-9-92B-IA2.5%36.lcd		
方法文件名	: 1.lcm		
批处理文件名	:		
样品瓶号	: 1-1	样品类型	: 未知
进样体积	: 1 uL		
分析日期	: 2022/11/30 22:47:43	分析者	: System Administrator
处理日期	: 2022/11/30 23:01:51	处理者	: System Administrator

<色谱图>

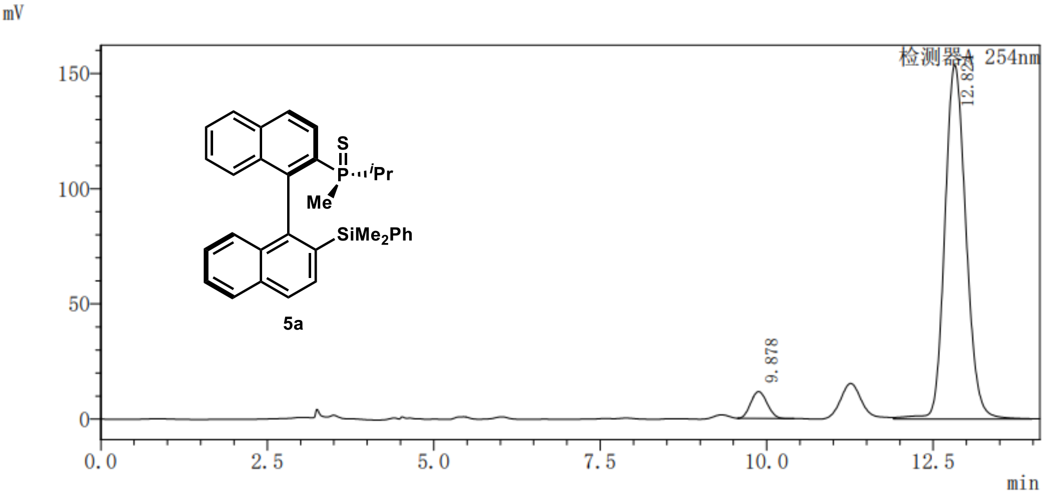

<峰表>

检测器A 254nm

峰号	保留时间	面积	高度	浓度	浓度单位	标记	化合物名
1	9.878	198747	11617	5.531		M	
2	12.824	3394308	153557	94.469		M	
总计		3593055	165173				

C:\LabSolutions\Sample\PLZ-9-92B-IA2.5%36.lcd

Supplementary Fig. 407. HPLC of **5a.**

SHIMADZU LabSolutions 分析报告

〈样品信息〉

样品名	: ZZY		
样品ID	: PLZ-220915		
数据文件名	: PLZ-9-91D-IE10%31.lcd		
方法文件名	: 1.lcm		
批处理文件名	:		
样品瓶号	: 1-1	样品类型	: 未知
进样体积	: 1 uL		
分析日期	: 2022/11/2 17:02:20	分析者	: System Administrator
处理日期	: 2022/11/2 17:22:23	处理者	: System Administrator

〈色谱图〉

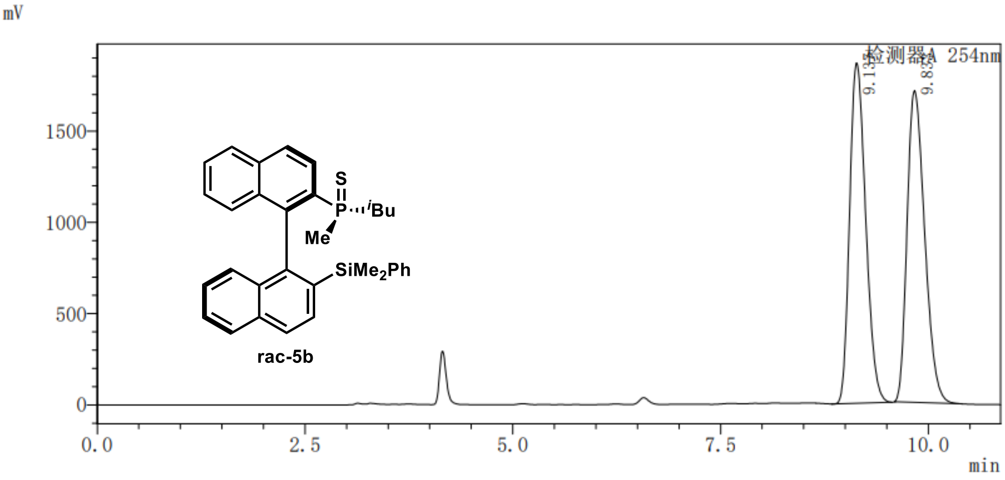

〈峰表〉

检测器A 254nm

峰号	保留时间	面积	高度	浓度	浓度单位	标记	化合物名
1	9.137	25073639	1863295	49.928		M	
2	9.833	25146376	1707483	50.072		M	
总计		50220015	3570778				

C:\LabSolutions\Sample\PLZ-9-91D-IE10%31.lcd

Supplementary Fig. 408. HPLC of **5b-rac.**

SHIMADZU LabSolutions 分析报告

〈样品信息〉

样品名	: ZZY		
样品ID	: PLZ-220915		
数据文件名	: PLZ-9-96D-IE10%32.lcd		
方法文件名	: 1.1cm		
批处理文件名	:		
样品瓶号	: 1-1	样品类型	: 未知
进样体积	: 1 uL		
分析日期	: 2022/11/2 17:16:25	分析者	: System Administrator
处理日期	: 2022/11/2 17:27:52	处理者	: System Administrator

〈色谱图〉

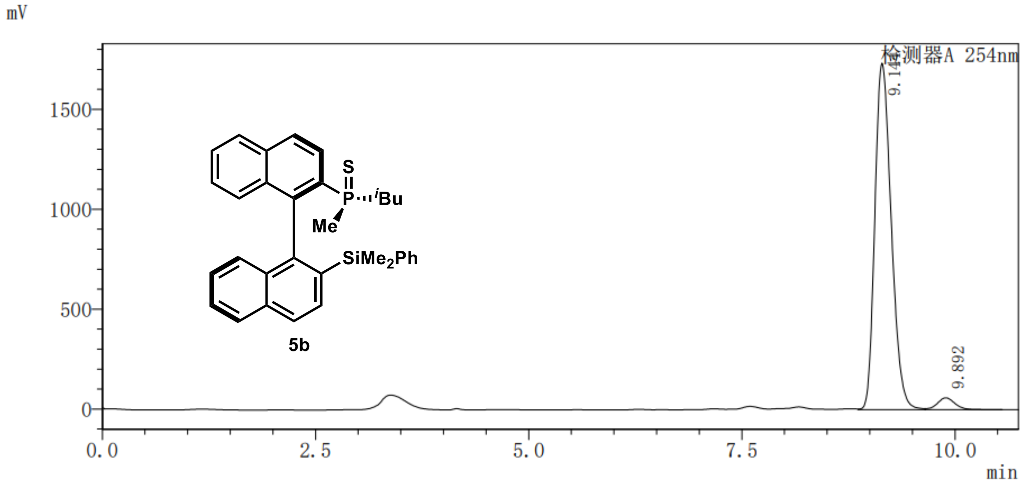

〈峰表〉

检测器A 254nm

峰号	保留时间	面积	高度	浓度	浓度单位	标记	化合物名
1	9.144	23258690	1733962	96.503			
2	9.892	842888	59012	3.497		V	
总计		24101578	1792974				

C:\LabSolutions\Sample\PLZ-9-96D-IE10%32.lcd

Supplementary Fig. 409. HPLC of **5b.**

SHIMADZU LabSolutions 分析报告

<样品信息>

样品名	: ZZY		
样品ID	: PLZ-220915		
数据文件名	: PLZ-9-89E-IE30%36.lcd		
方法文件名	: 1.lcm		
批处理文件名	:		
样品瓶号	: 1-1	样品类型	: 未知
进样体积	: 1 uL		
分析日期	: 2022/11/2 18:32:32	分析者	: System Administrator
处理日期	: 2022/11/2 19:31:35	处理者	: System Administrator

<色谱图>

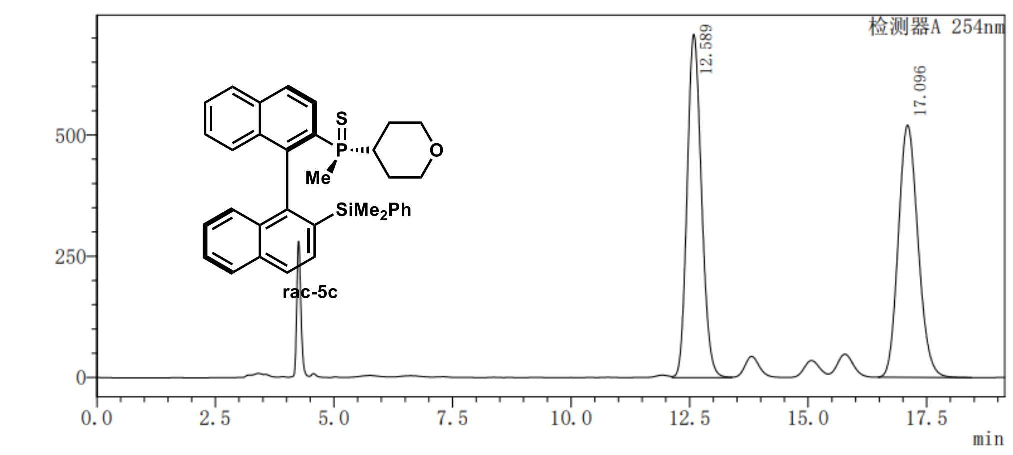

<峰表>

检测器A 254nm

峰号	保留时间	面积	高度	浓度	浓度单位	标记	化合物名
1	12.589	14967120	708071	49.895			
2	17.096	15029965	519979	50.105		M	
总计		29997085	1228049				

C:\LabSolutions\Sample\PLZ-9-89E-IE30%36.lcd

Supplementary Fig. 410. HPLC of **5c-rac.**

SHIMADZU LabSolutions 分析报告

〈样品信息〉

样品名	: ZZY		
样品ID	: PLZ-220915		
数据文件名	: PLZ-9-98E-IE30%44.lcd		
方法文件名	: 1.lcm		
批处理文件名	:		
样品瓶号	: 1-1	样品类型	: 未知
进样体积	: 1 uL		
分析日期	: 2022/11/2 21:13:58	分析者	: System Administrator
处理日期	: 2022/11/8 19:43:19	处理者	: System Administrator

〈色谱图〉

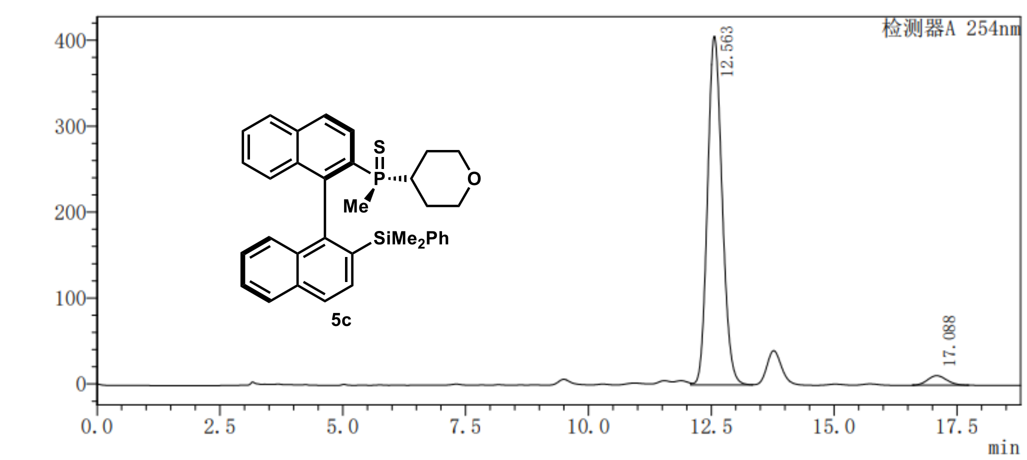

〈峰表〉

检测器A 254nm

峰号	保留时间	面积	高度	浓度	浓度单位	标记	化合物名
1	12.563	8402888	405806	96.531		M	
2	17.088	301961	11137	3.469		M	
总计		8704848	416943				

C:\LabSolutions\Sample\PLZ-9-98E-IE30%44.lcd

Supplementary Fig. 411. HPLC of **5c.**

SHIMADZU LabSolutions 分析报告

〈样品信息〉

样品名 : ZZY
样品ID : PLZ-220915
数据文件名 : PLZ-9-89C-IE30%35.lcd
方法文件名 : 1.1cm
批处理文件名 :
样品瓶号 : 1-1 样品类型 : 未知
进样体积 : 1 uL
分析日期 : 2022/11/2 18:19:50 分析者 : System Administrator
处理日期 : 2022/11/30 15:17:24 处理者 : System Administrator

〈色谱图〉

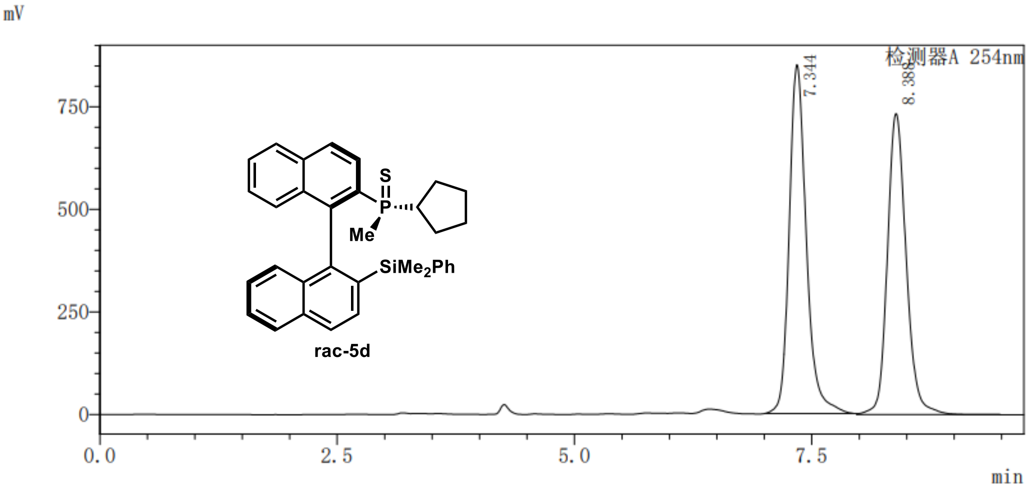

〈峰表〉

检测器A 254nm

峰号	保留时间	面积	高度	浓度	浓度单位	标记	化合物名
1	7.344	10675622	850200	51.365		M	
2	8.388	10108293	733411	48.635		M	
总计		20783915	1583612				

C:\LabSolutions\Sample\PLZ-9-89C-IE30%35.lcd

Supplementary Fig. 412. HPLC of **5d-rac.**

SHIMADZU LabSolutions 分析报告

<样品信息>

样品名 : ZZY
样品ID : PLZ-220915
数据文件名 : PLZ-9-98C-IE30%43.lcd
方法文件名 : 1.lcm
批处理文件名 :
样品瓶号 : 1-1 样品类型 : 未知
进样体积 : 1 uL
分析日期 : 2022/11/2 21:02:56 分析者 : System Administrator
处理日期 : 2022/11/30 14:36:40 处理者 : System Administrator

<色谱图>

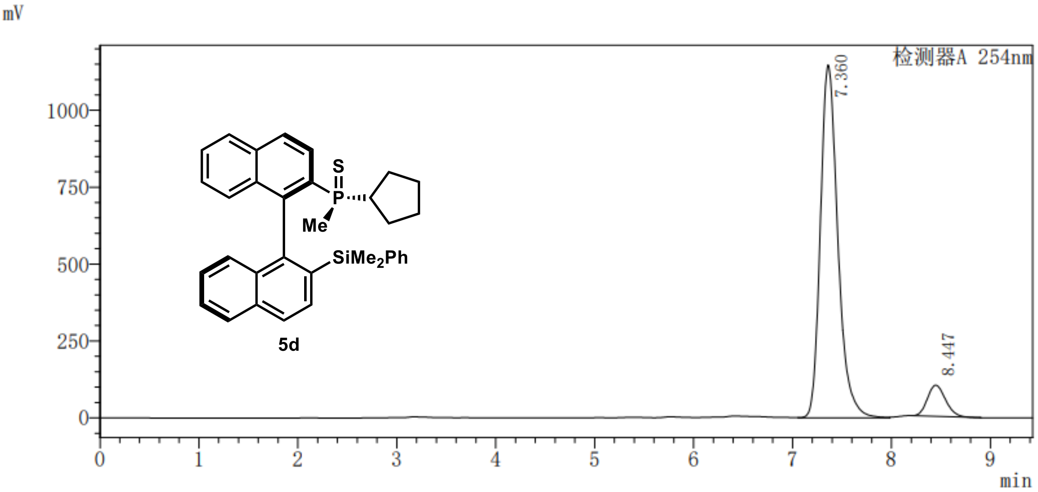

<峰表>

检测器A 254nm

峰号	保留时间	面积	高度	浓度	浓度单位	标记	化合物名
1	7.360	14073436	1147212	91.571		M	
2	8.447	1295448	101284	8.429		M	
总计		15368884	1248496				

C:\LabSolutions\Sample\PLZ-9-98C-IE30%43.lcd

Supplementary Fig. 413. HPLC of **5d.**

SHIMADZU LabSolutions 分析报告

〈样品信息〉

样品名	: ZZY			
样品ID	: PLZ-220915			
数据文件名	: PLZ-9-99E-ID10%10.lcd			
方法文件名	: 1.lcm			
批处理文件名	:			
样品瓶号	: 1-1	样品类型	: 未知	
进样体积	: 1 uL			
分析日期	: 2022/11/5 16:37:31	分析者	: System Administrator	
处理日期	: 2022/11/5 17:08:17	处理者	: System Administrator	

〈色谱图〉

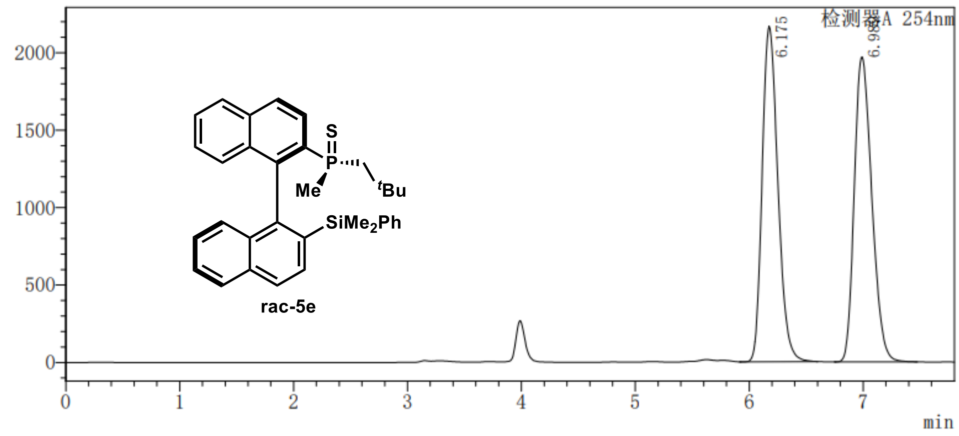

〈峰表〉

检测器A 254nm

峰号	保留时间	面积	高度	浓度	浓度单位	标记	化合物名
1	6.175	20740396	2166566	49.571		M	
2	6.989	21098961	1969207	50.429		M	
总计		41839357	4135772				

C:\LabSolutions\Sample\PLZ-9-99E-ID10%10.lcd

Supplementary Fig. 414. HPLC of **5e-rac.**

SHIMADZU LabSolutions 分析报告

〈样品信息〉

样品名	: ZZY		
样品ID	: PLZ-220915		
数据文件名	: PLZ-9-101E-ID10%11.lcd		
方法文件名	: 1.lcm		
批处理文件名	:		
样品瓶号	: 1-1	样品类型	: 未知
进样体积	: 1 uL		
分析日期	: 2022/11/5 16:59:10	分析者	: System Administrator
处理日期	: 2022/11/5 17:07:58	处理者	: System Administrator

〈色谱图〉

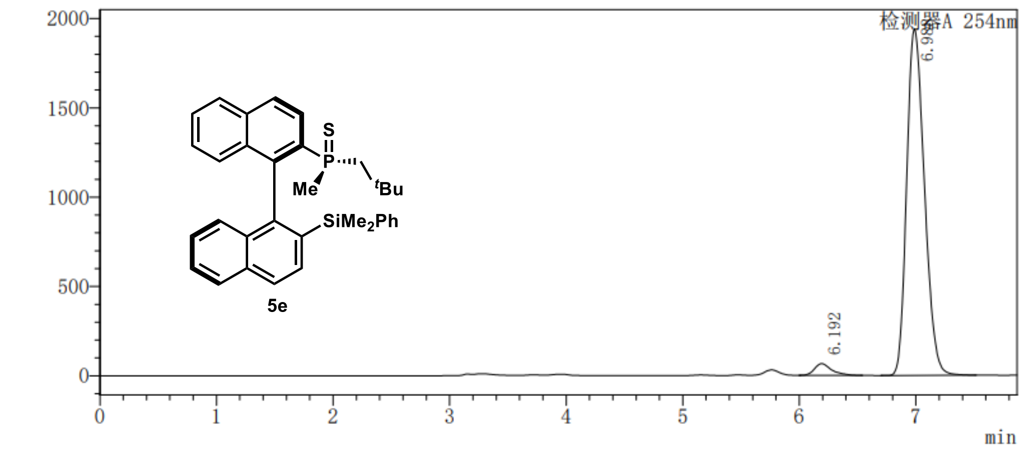

〈峰表〉

检测器A 254nm

峰号	保留时间	面积	高度	浓度	浓度单位	标记	化合物名
1	6.192	663217	65230	3.130		M	
2	6.988	20526622	1938400	96.870		M	
总计		21189840	2003630				

C:\LabSolutions\Sample\PLZ-9-101E-ID10%11.lcd

Supplementary Fig. 415. HPLC of **5e-rac.**

SHIMADZU LabSolutions 分析报告

<样品信息>

样品名	: ZZY			
样品ID	: PLZ-220915			
数据文件名	: PLZ-9-91C-IE10%6.lcd			
方法文件名	: 1.lcm			
批处理文件名	:			
样品瓶号	: 1-1	样品类型	: 未知	
进样体积	: 1 uL			
分析日期	: 2022/10/29 10:12:57	分析者	: System Administrator	
处理日期	: 2022/10/29 10:36:29	处理者	: System Administrator	

<色谱图>

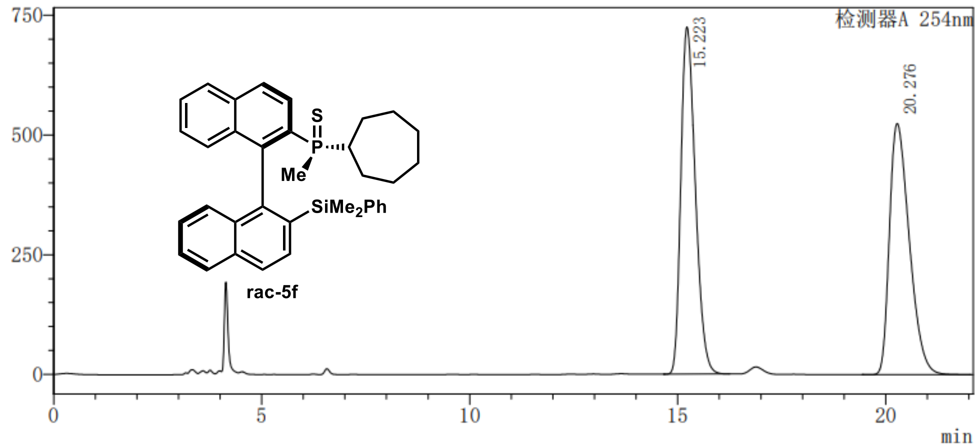

<峰表>

检测器A 254nm

峰号	保留时间	面积	高度	浓度	浓度单位	标记	化合物名
1	15.223	17554060	724652	50.207		M	
2	20.276	17409209	524470	49.793		M	
总计		34963270	1249122				

C:\LabSolutions\Sample\PLZ-9-91C-IE10%6.lcd

Supplementary Fig. 416. HPLC of **5f-rac.**

SHIMADZU LabSolutions 分析报告

〈样品信息〉

样品名	: ZZY			
样品ID	: PLZ-220915			
数据文件名	: PLZ-9-96C-IE10%28.lcd			
方法文件名	: 1.1cm			
批处理文件名	:			
样品瓶号	: 1-1	样品类型	: 未知	
进样体积	: 1 uL			
分析日期	: 2022/11/2 15:17:30	分析者	: System Administrator	
处理日期	: 2022/11/2 15:45:10	处理者	: System Administrator	

〈色谱图〉

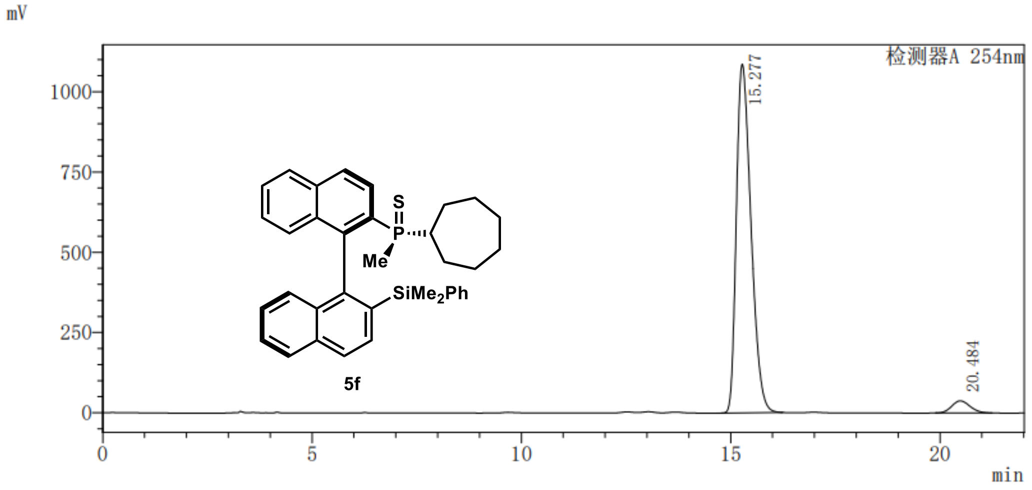

〈峰表〉

检测器A 254nm

峰号	保留时间	面积	高度	浓度	浓度单位	标记	化合物名
1	15.277	26400345	1085875	95.842		M	
2	20.484	1145401	37773	4.158		M	
总计		27545746	1123648				

C:\LabSolutions\Sample\PLZ-9-96C-IE10%28.lcd

Supplementary Fig. 417. HPLC of **5f.**

SHIMADZU LabSolutions 分析报告

〈样品信息〉

样品名 : ZZY
样品ID : PLZ-220915
数据文件名 : PLZ-9-91A-IE10%33.lcd
方法文件名 : 1.lcm
批处理文件名 :
样品瓶号 : 1-1 样品类型 : 未知
进样体积 : 1 uL
分析日期 : 2022/11/2 17:29:43 分析者 : System Administrator
处理日期 : 2022/11/2 17:52:39 处理者 : System Administrator

〈色谱图〉

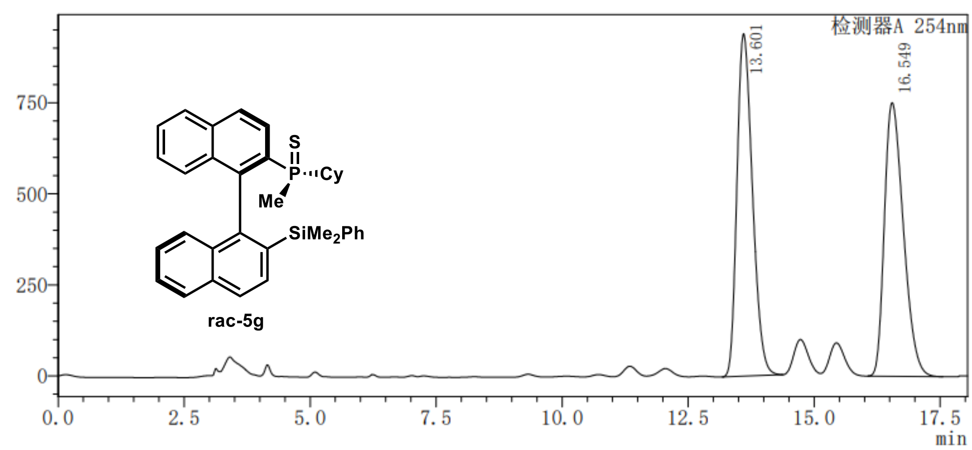

〈峰表〉

检测器A 254nm

峰号	保留时间	面积	高度	浓度	浓度单位	标记	化合物名
1	13.601	20065951	939516	50.487		M	
2	16.549	19678911	751144	49.513		M	
总计		39744863	1690660				

C:\LabSolutions\Sample\PLZ-9-91A-IE10%33.lcd

Supplementary Fig. 418. HPLC of **5g-rac.**

SHIMADZU LabSolutions 分析报告

<样品信息>

样品名 : PLZ
样品ID : PLZ-220915
数据文件名 : PLZ-9-110C-IE10%18.lcd
方法文件名 : 1.1cm
批处理文件名 :
样品瓶号 : 1-1　　样品类型 : 未知
进样体积 : 1 uL
分析日期 : 2022/11/30 14:54:27　　分析者 : System Administrator
处理日期 : 2022/11/30 15:12:31　　处理者 : System Administrator

<色谱图>

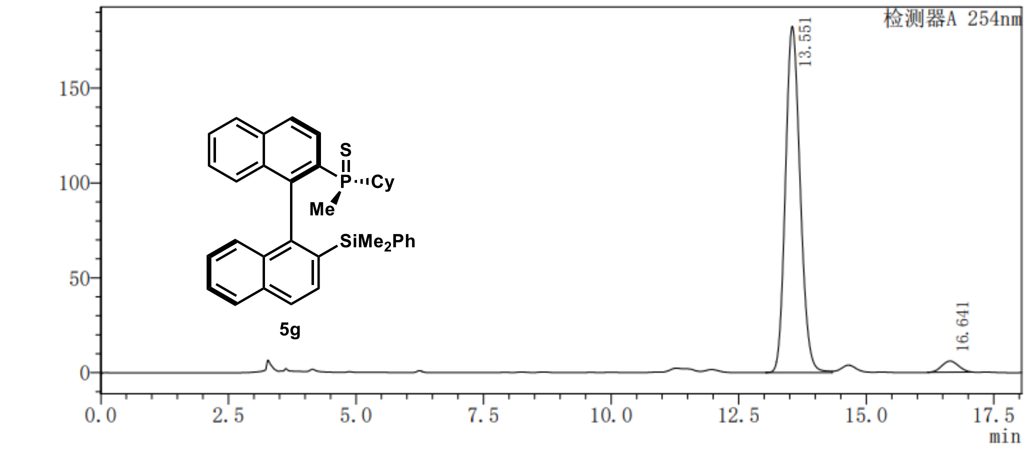

<峰表>

检测器A 254nm

峰号	保留时间	面积	高度	浓度	浓度单位	标记	化合物名
1	13.551	3670036	182521	96.478		V	
2	16.641	133989	5892	3.522		M	
总计		3804025	188414				

C:\LabSolutions\Sample\PLZ-9-110C-IE10%18.lcd

Supplementary Fig. 419. HPLC of **5g.**

SHIMADZU LabSolutions 分析报告

<样品信息>

样品名 : ZZY
样品ID : PLZ-220915
数据文件名 : LG-1-31B-IE10%14.lcd
方法文件名 : 1.lcm
批处理文件名 :
样品瓶号 : 1-1 样品类型 : 未知
进样体积 : 1 uL
分析日期 : 2022/11/4 10:28:38 分析者 : System Administrator
处理日期 : 2022/11/4 11:02:15 处理者 : System Administrator

<色谱图>

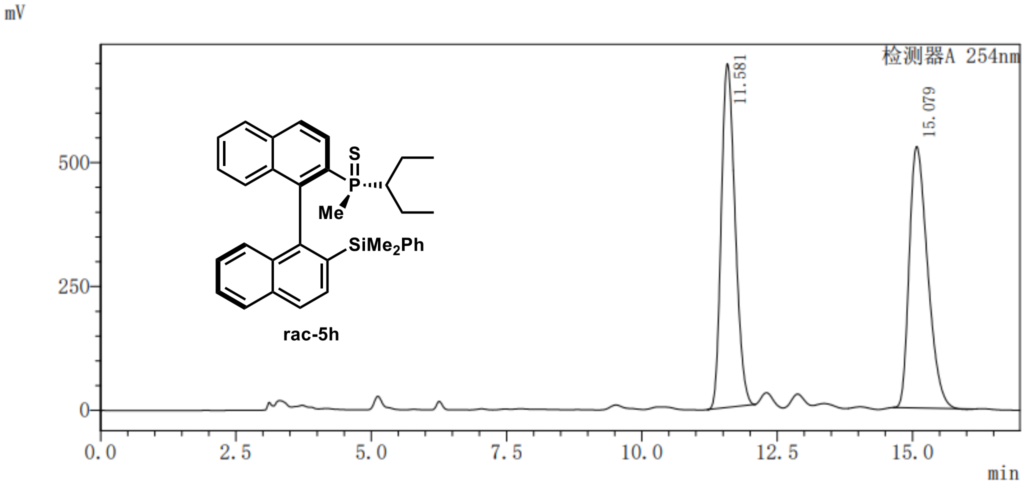

<峰表>

检测器A 254nm

峰号	保留时间	面积	高度	浓度	浓度单位	标记	化合物名
1	11.581	12350331	693581	49.810		M	
2	15.079	12444796	527472	50.190		M	
总计		24795127	1221053				

C:\LabSolutions\Sample\LG-1-31B-IE10%14.lcd

Supplementary Fig. 420. HPLC of **5h-rac.**

SHIMADZU LabSolutions 分析报告

〈样品信息〉

样品名	: ZZY			
样品ID	: PLZ-220915			
数据文件名	: PLZ-9-98B-IE10%6.lcd			
方法文件名	: 1.1cm			
批处理文件名	:			
样品瓶号	: 1-1	样品类型	: 未知	
进样体积	: 1 uL			
分析日期	: 2022/11/8 10:20:07	分析者	: System Administrator	
处理日期	: 2022/11/8 18:53:38	处理者	: System Administrator	

〈色谱图〉

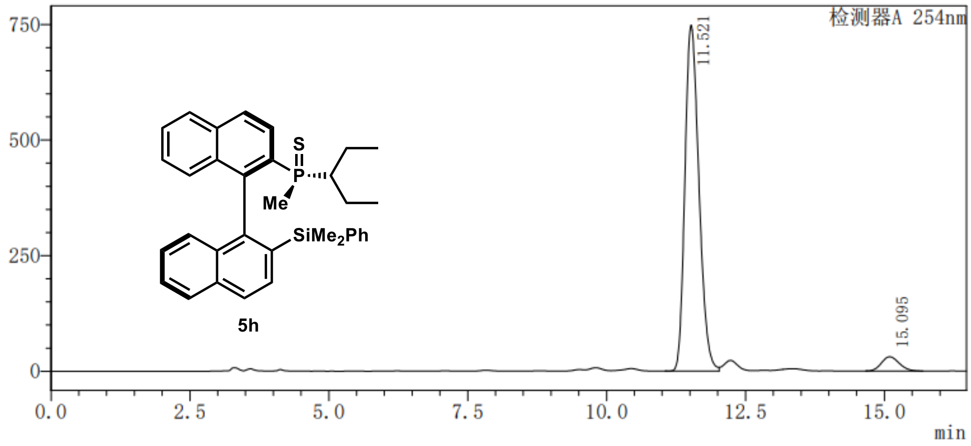

〈峰表〉

检测器A 254nm

峰号	保留时间	面积	高度	浓度	浓度单位	标记	化合物名
1	11.521	13145649	748333	95.055			
2	15.095	683942	30809	4.945		M	
总计		13829590	779142				

C:\LabSolutions\Sample\PLZ-9-98B-IE10%6.lcd

Supplementary Fig. 421. HPLC of **5h.**

SHIMADZU LabSolutions 分析报告

〈样品信息〉

样品名	: PLZ		
样品ID	: PLZ-220915		
数据文件名	: PLZ-9-89F-IG20%14. lcd		
方法文件名	: 1. lcm		
批处理文件名	:		
样品瓶号	: 1-1	样品类型	: 未知
进样体积	: 1 uL		
分析日期	: 2022/11/30 13:33:24	分析者	: System Administrator
处理日期	: 2022/11/30 13:46:25	处理者	: System Administrator

〈色谱图〉

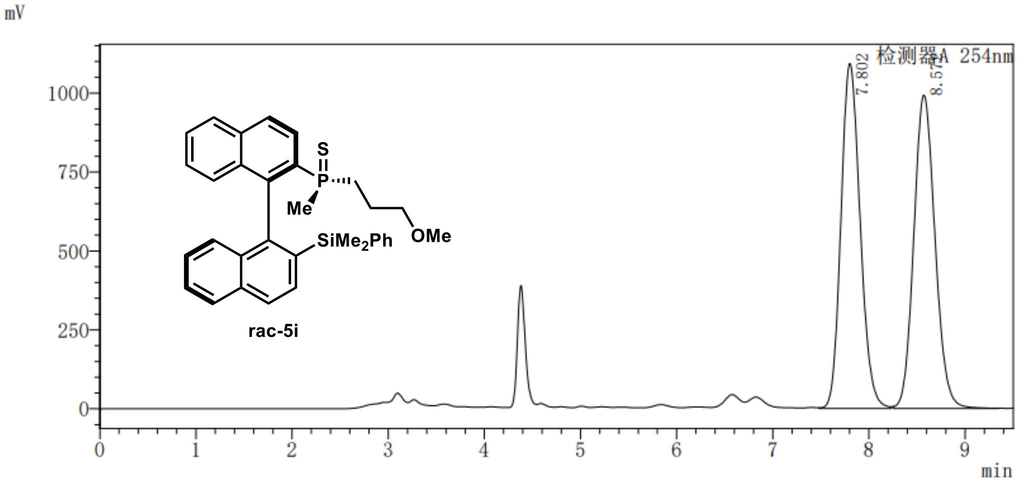

〈峰表〉

检测器A 254nm

峰号	保留时间	面积	高度	浓度	浓度单位	标记	化合物名
1	7. 802	15133558	1092194	49. 855			
2	8. 572	15221456	992259	50. 145		M	
总计		30355014	2084453				

C:\LabSolutions\Sample\PLZ-9-89F-IG20%14.lcd

Supplementary Fig. 422. HPLC of **5i-rac.**

SHIMADZU LabSolutions 分析报告

〈样品信息〉

样品名 : PLZ
样品ID : PLZ-220915
数据文件名 : PLZ-9-98F-IG20%17.lcd
方法文件名 : 1.lcm
批处理文件名 :
样品瓶号 : 1-1 样品类型 : 未知
进样体积 : 1 uL
分析日期 : 2022/11/30 14:14:20 分析者 : System Administrator
处理日期 : 2022/11/30 14:24:58 处理者 : System Administrator

〈色谱图〉

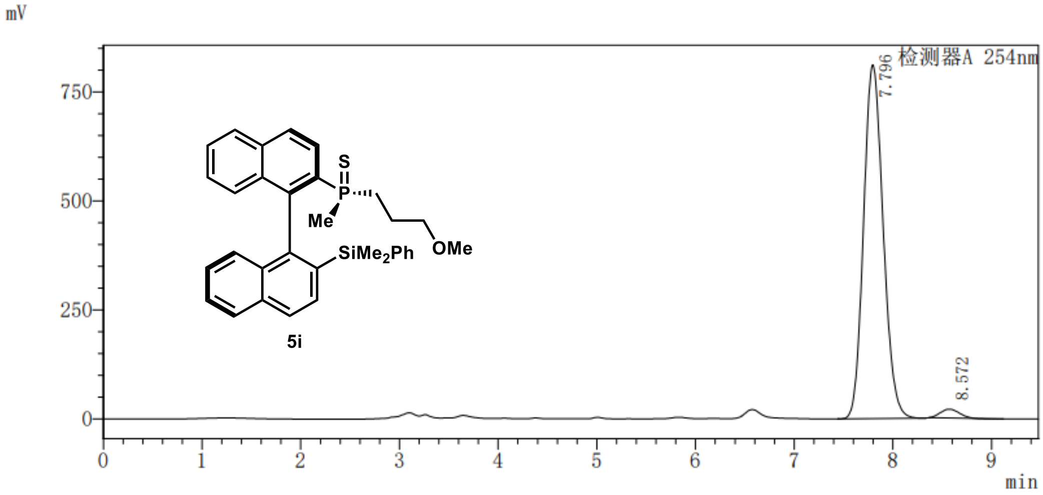

〈峰表〉

检测器A 254nm

峰号	保留时间	面积	高度	浓度	浓度单位	标记	化合物名
1	7.796	11189564	810899	97.664		M	
2	8.572	267697	20214	2.336		M	
总计		11457261	831113				

C:\LabSolutions\Sample\PLZ-9-98F-IG20%17.lcd

Supplementary Fig. 423. HPLC of **5i.**

SHIMADZU LabSolutions 分析报告

〈样品信息〉

样品名	: PLZ		
样品ID	: PLZ-220915		
数据文件名	: PLZ-9-118B-IG5%15.lcd		
方法文件名	: 1.lcm		
批处理文件名	:		
样品瓶号	: 1-1	样品类型	: 未知
进样体积	: 1 uL		
分析日期	: 2022/11/12 22:37:40	分析者	: System Administrator
处理日期	: 2022/11/12 22:49:55	处理者	: System Administrator

〈色谱图〉

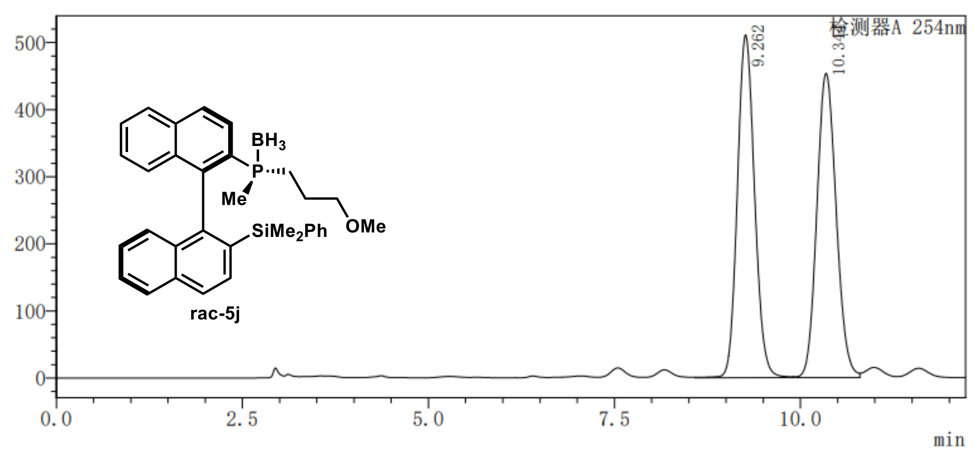

〈峰表〉

检测器A 254nm

峰号	保留时间	面积	高度	浓度	浓度单位	标记	化合物名
1	9.262	8218169	510846	50.039			
2	10.344	8205476	453753	49.961		V	
总计		16423644	964599				

C:\LabSolutions\Sample\PLZ-9-118B-IG5%15.lcd

Supplementary Fig. 424. HPLC of **5j-rac.**

SHIMADZU LabSolutions 分析报告

〈样品信息〉

样品名	: PLZ		
样品ID	: PLZ-220915		
数据文件名	: PLZ-9-118A-IG5%19. lcd		
方法文件名	: 1. lcm		
批处理文件名	:		
样品瓶号	: 1-1	样品类型	: 未知
进样体积	: 1 uL		
分析日期	: 2022/11/11 16:24:38	分析者	: System Administrator
处理日期	: 2022/11/12 22:33:53	处理者	: System Administrator

〈色谱图〉

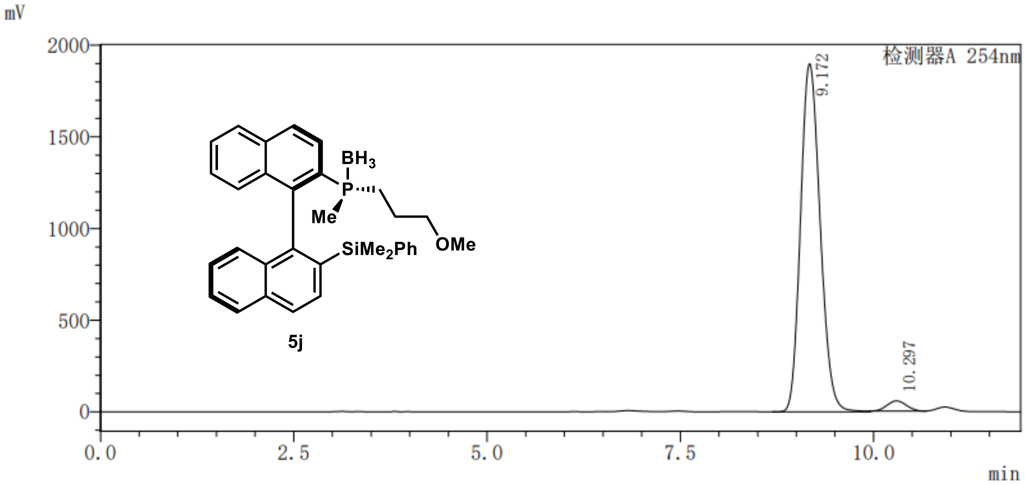

〈峰表〉

检测器A 254nm

峰号	保留时间	面积	高度	浓度	浓度单位	标记	化合物名
1	9.172	33511368	1898022	97.183		M	
2	10.297	971365	55500	2.817		M	
总计		34482732	1953522				

C:\LabSolutions\Sample\PLZ-9-118A-IG5%19.lcd

Supplementary Fig. 425. HPLC of **5j.**

SHIMADZU LabSolutions 分析报告

〈样品信息〉

样品名 : PLZ
样品ID : PLZ-220915
数据文件名 : PLZ-9-91B-IG10%6.lcd
方法文件名 : 1.lcm
批处理文件名 :
样品瓶号 : 1-1 样品类型 : 未知
进样体积 : 1 uL
分析日期 : 2022/12/1 18:09:12 分析者 : System Administrator
处理日期 : 2022/12/1 18:25:26 处理者 : System Administrator

〈色谱图〉

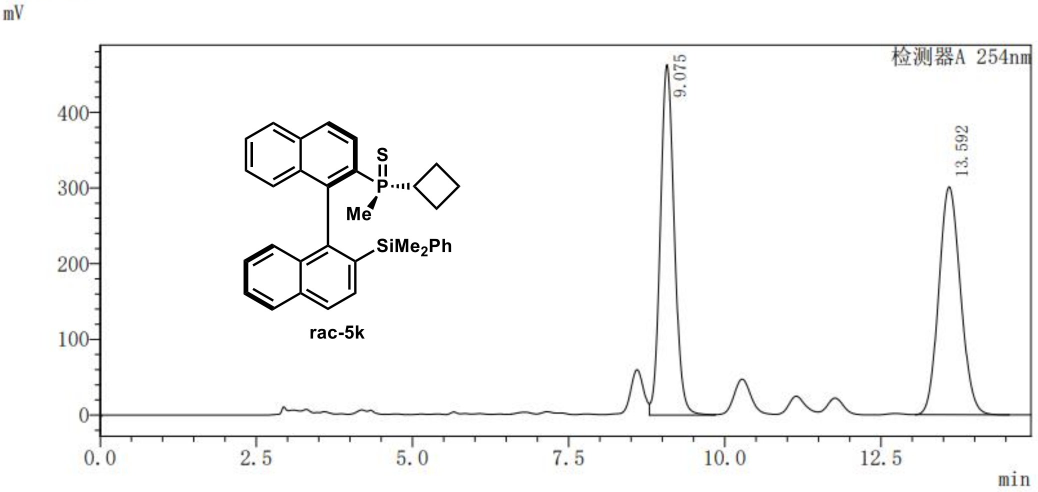

〈峰表〉

检测器A 254nm

峰号	保留时间	面积	高度	浓度	浓度单位	标记	化合物名
1	9.075	7186066	462709	49.698			
2	13.592	7273436	301020	50.302		M	
总计		14459502	763728				

C:\LabSolutions\Sample\PLZ-9-91B-IG10%6.lcd

Supplementary Fig. 426. HPLC of **5k-rac.**

SHIMADZU LabSolutions 分析报告

〈样品信息〉

样品名	: PLZ		
样品ID	: PLZ-220915		
数据文件名	: PLZ-9-98D-IG10%7.lcd		
方法文件名	: 1.1cm		
批处理文件名	:		
样品瓶号	: 1-1	样品类型	: 未知
进样体积	: 1 uL		
分析日期	: 2022/12/1 18:29:36	分析者	: System Administrator
处理日期	: 2022/12/1 18:49:03	处理者	: System Administrator

〈色谱图〉

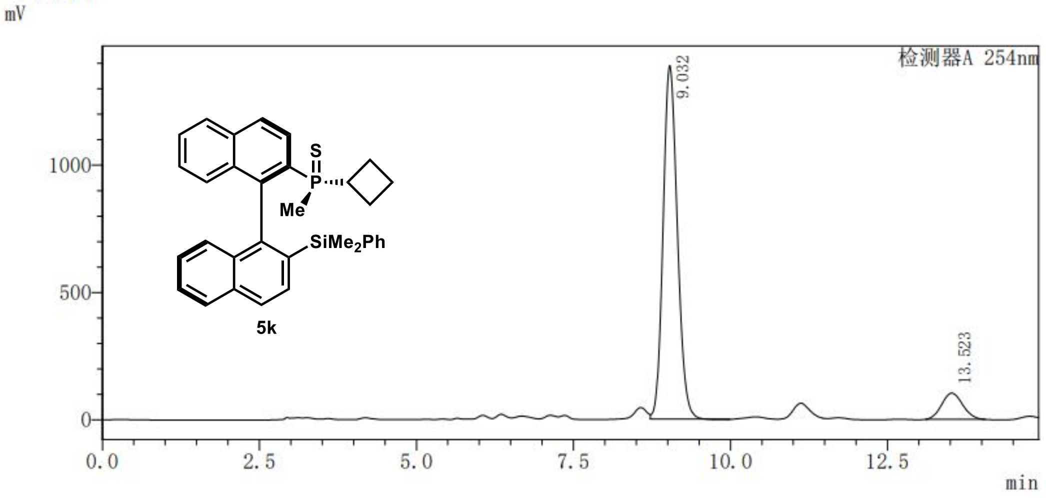

〈峰表〉

检测器A 254nm

峰号	保留时间	面积	高度	浓度	浓度单位	标记	化合物名
1	9.032	21968580	1388058	90.205		M	
2	13.523	2385509	102632	9.795		M	
总计		24354088	1490691				

C:\LabSolutions\Sample\PLZ-9-98D-IG10%7.lcd

Supplementary Fig. 427. HPLC of **5k.**

SHIMADZU LabSolutions 分析报告

〈样品信息〉

样品名	: ZZY		
样品ID	: PLZ-220915		
数据文件名	: PLZ-9-99B-ID10%7.lcd		
方法文件名	: 1.lcm		
批处理文件名	:		
样品瓶号	: 1-1	样品类型	: 未知
进样体积	: 1 uL		
分析日期	: 2022/11/5 16:06:35	分析者	: System Administrator
处理日期	: 2022/11/5 16:20:32	处理者	: System Administrator

〈色谱图〉

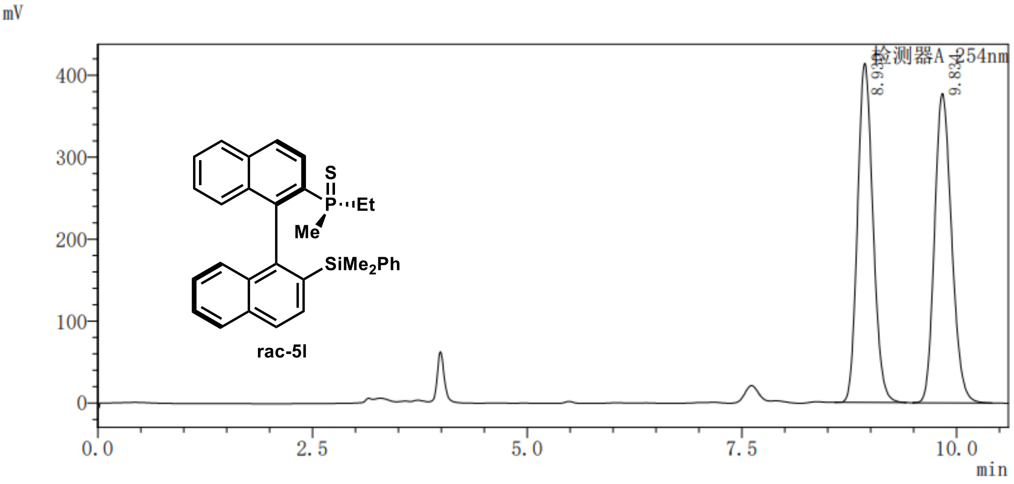

〈峰表〉

检测器A 254nm

峰号	保留时间	面积	高度	浓度	浓度单位	标记	化合物名
1	8.930	5177506	413649	49.727		M	
2	9.834	5234337	377643	50.273		M	
总计		10411842	791293				

C:\LabSolutions\Sample\PLZ-9-99B-ID10%7.lcd

Supplementary Fig. 428. HPLC of **5l-rac.**

SHIMADZU LabSolutions 分析报告

<样品信息>

样品名	: ZZY		
样品ID	: PLZ-220915		
数据文件名	: PLZ-9-101B-ID10%7.lcd		
方法文件名	: 1.lcm		
批处理文件名	:		
样品瓶号	: 1-1	样品类型	: 未知
进样体积	: 1 uL		
分析日期	: 2022/11/5 15:51:58	分析者	: System Administrator
处理日期	: 2022/11/5 16:08:12	处理者	: System Administrator

<色谱图>

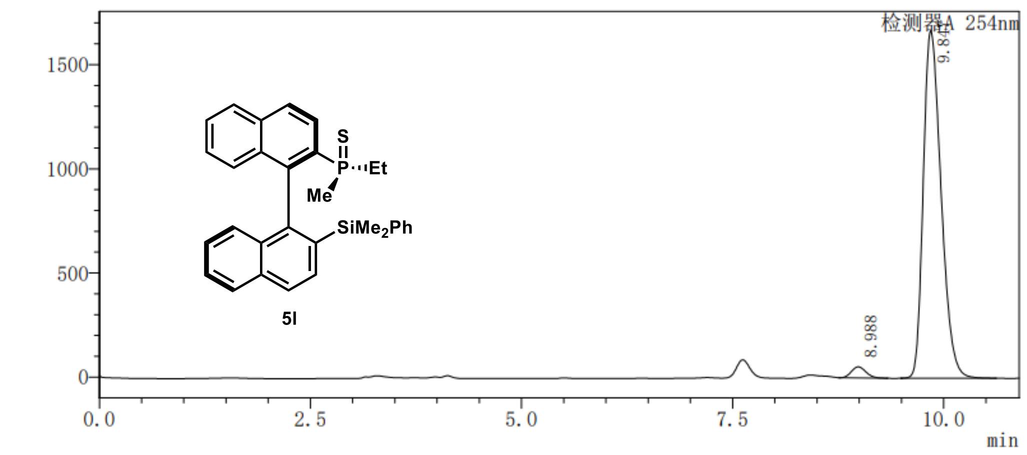

<峰表>

检测器A 254nm

峰号	保留时间	面积	高度	浓度	浓度单位	标记	化合物名
1	8.988	639703	53225	2.547		M	
2	9.847	24478515	1667447	97.453		M	
总计		25118218	1720672				

C:\LabSolutions\Sample\PLZ-9-101B-ID10%7.lcd

Supplementary Fig. 429. HPLC of **5l.**

SHIMADZU LabSolutions 分析报告

<样品信息>

样品名	: ZZY		
样品ID	: PLZ-220915		
数据文件名	: PLZ-9-95F-IE10%2.lcd		
方法文件名	: 1.1cm		
批处理文件名	:		
样品瓶号	: 1-1	样品类型	: 未知
进样体积	: 1 uL		
分析日期	: 2022/11/5 10:03:04	分析者	: System Administrator
处理日期	: 2022/11/5 10:29:14	处理者	: System Administrator

<色谱图>

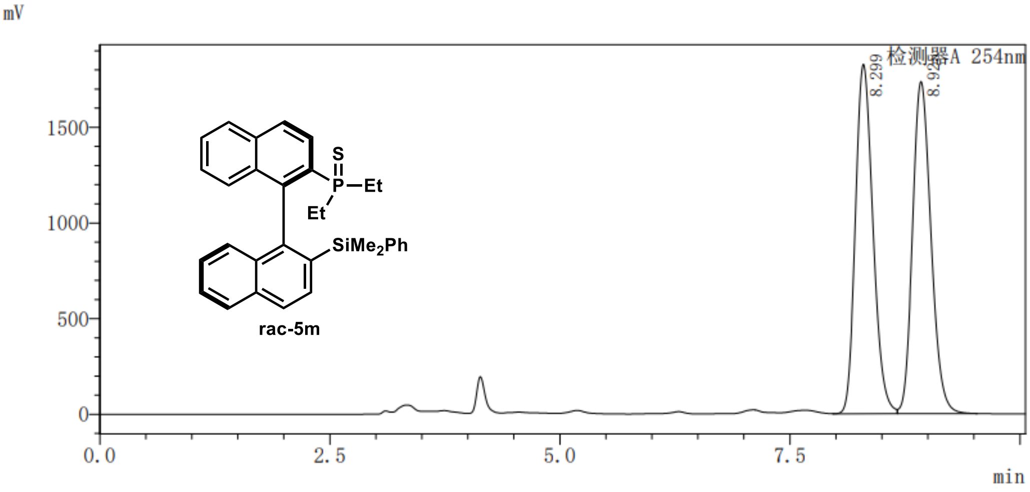

<峰表>

检测器A 254nm

峰号	保留时间	面积	高度	浓度	浓度单位	标记	化合物名
1	8.299	24245288	1826735	50.079			
2	8.925	24168929	1735970	49.921		V M	
总计		48414218	3562705				

C:\LabSolutions\Sample\PLZ-9-95F-IE10%2.lcd

Supplementary Fig. 430. HPLC of **5m-rac.**

SHIMADZU LabSolutions 分析报告

<样品信息>

样品名	: ZZY		
样品ID	: PLZ-220915		
数据文件名	: PLZ-9-100F-IE10%3.lcd		
方法文件名	: 1.lcm		
批处理文件名	:		
样品瓶号	: 1-1	样品类型	: 未知
进样体积	: 1 uL		
分析日期	: 2022/11/5 10:20:52	分析者	: System Administrator
处理日期	: 2022/11/5 10:36:07	处理者	: System Administrator

<色谱图>

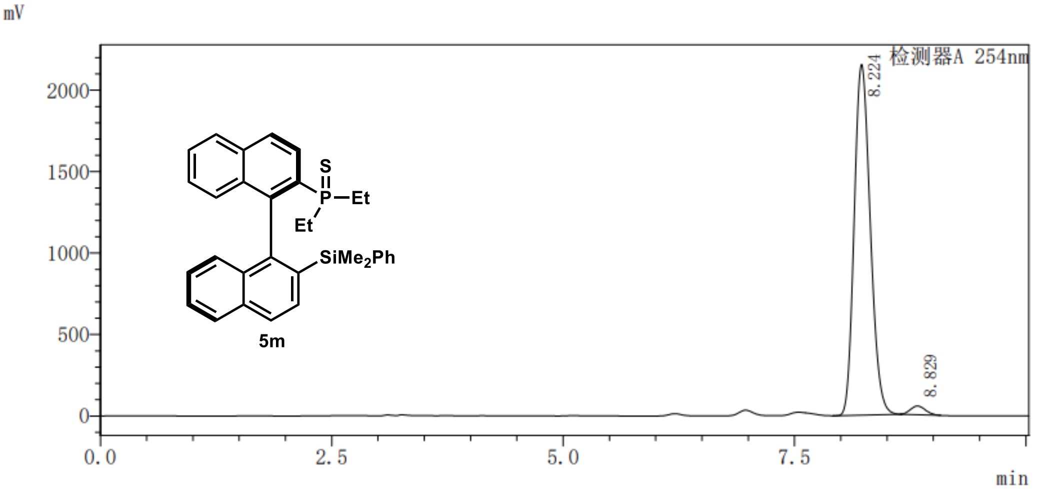

<峰表>

检测器A 254nm

峰号	保留时间	面积	高度	浓度	浓度单位	标记	化合物名
1	8.224	25996546	2152946	97.779		M	
2	8.829	590429	52714	2.221		M	
总计		26586975	2205661				

C:\LabSolutions\Sample\PLZ-9-100F-IE10%3.lcd

Supplementary Fig. 431. HPLC of **5m.**

SHIMADZU LabSolutions 分析报告

〈样品信息〉

样品名	: PLZ		
样品ID	: PLZ-220915		
数据文件名	: PLZ-9-145B-OD5%7.lcd		
方法文件名	: 1.lcm		
批处理文件名	:		
样品瓶号	: 1-1	样品类型	: 未知
进样体积	: 1 uL		
分析日期	: 2022/11/22 21:08:44	分析者	: System Administrator
处理日期	: 2022/11/22 21:21:00	处理者	: System Administrator

〈色谱图〉

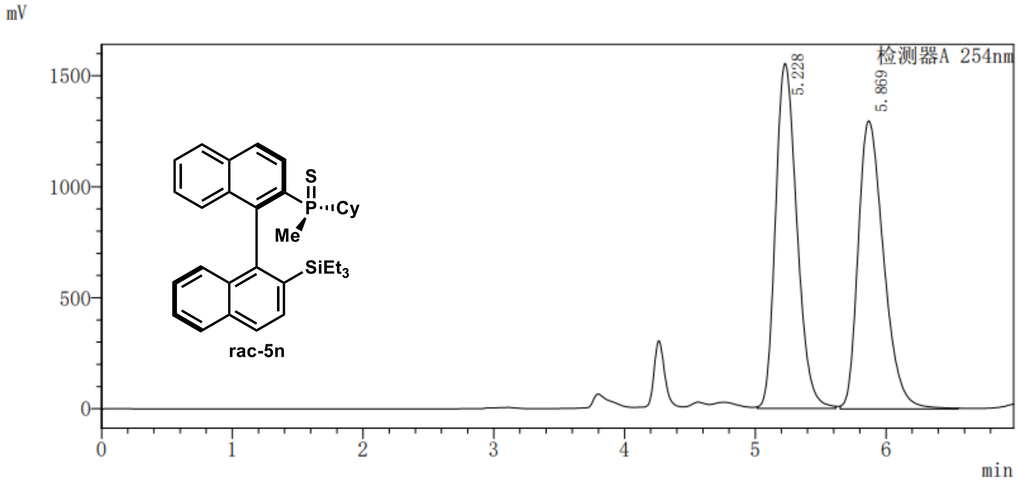

〈峰表〉

检测器A 254nm

峰号	保留时间	面积	高度	浓度	浓度单位	标记	化合物名
1	5.228	17673492	1553691	50.080		M	
2	5.869	17616874	1298147	49.920		M	
总计		35290366	2851838				

C:\LabSolutions\Sample\PLZ-9-145B-OD5%7.lcd

Supplementary Fig. 432. HPLC of **5n-rac.**

SHIMADZU LabSolutions 分析报告

<样品信息>

样品名	: PLZ		
样品ID	: PLZ-220915		
数据文件名	: PLZ-9-145A-OD5%10.lcd		
方法文件名	: 1.lcm		
批处理文件名	:		
样品瓶号	: 1-1	样品类型	: 未知
进样体积	: 1 uL		
分析日期	: 2022/11/22 21:37:57	分析者	: System Administrator
处理日期	: 2022/11/22 21:46:36	处理者	: System Administrator

<色谱图>

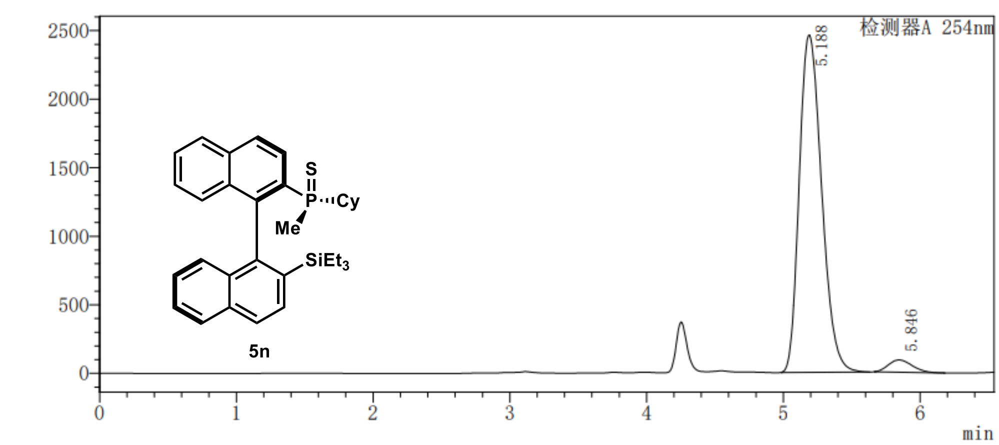

<峰表>

检测器A 254nm

峰号	保留时间	面积	高度	浓度	浓度单位	标记	化合物名
1	5.188	28005159	2459592	96.297		M	
2	5.846	1077059	89263	3.703		M	
总计		29082218	2548855				

C:\LabSolutions\Sample\PLZ-9-145A-OD5%10.lcd

Supplementary Fig. 433. HPLC of **5n.**

SHIMADZU LabSolutions 分析报告

<样品信息>

样品名	: PLZ		
样品ID	: PLZ-220915		
数据文件名	: PLZ-9-121B-IG5%20. lcd		
方法文件名	: 1. 1cm		
批处理文件名	:		
样品瓶号	: 1-1	样品类型	: 未知
进样体积	: 1 uL		
分析日期	: 2022/11/11 16:40:51	分析者	: System Administrator
处理日期	: 2022/11/11 18:23:40	处理者	: System Administrator

<色谱图>

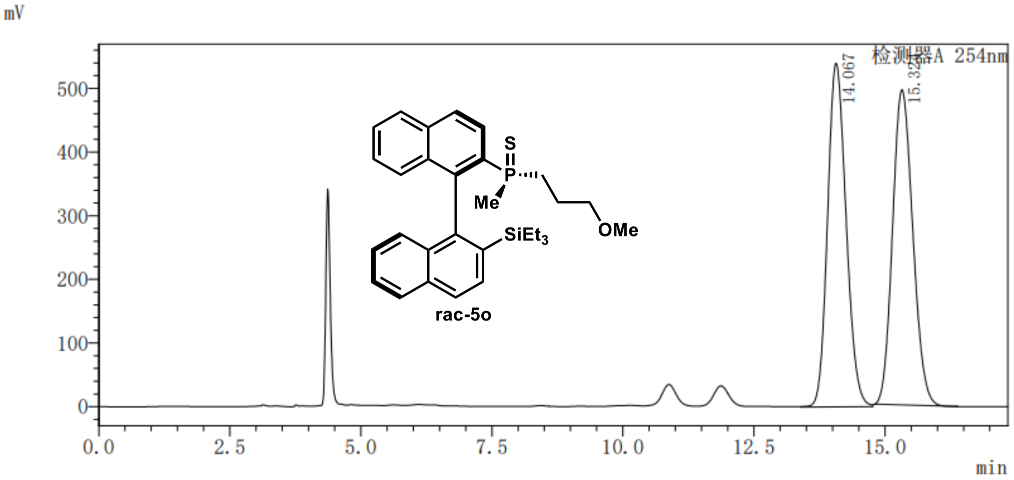

<峰表>

检测器A 254nm

峰号	保留时间	面积	高度	浓度	浓度单位	标记	化合物名
1	14. 067	13275938	539719	49. 919		M	
2	15. 324	13318756	495305	50. 081		M	
总计		26594694	1035023				

C:\LabSolutions\Sample\PLZ-9-121B-IG5%20.lcd

Supplementary Fig. 434. HPLC of **5o-rac.**

SHIMADZU LabSolutions 分析报告

<样品信息>

样品名	: PLZ		
样品ID	: PLZ-220915		
数据文件名	: PLZ-9-121A-IG5%21.lcd		
方法文件名	: 1.lcm		
批处理文件名	:		
样品瓶号	: 1-1	样品类型	: 未知
进样体积	: 1 uL		
分析日期	: 2022/11/11 16:58:55	分析者	: System Administrator
处理日期	: 2022/11/11 18:24:20	处理者	: System Administrator

<色谱图>

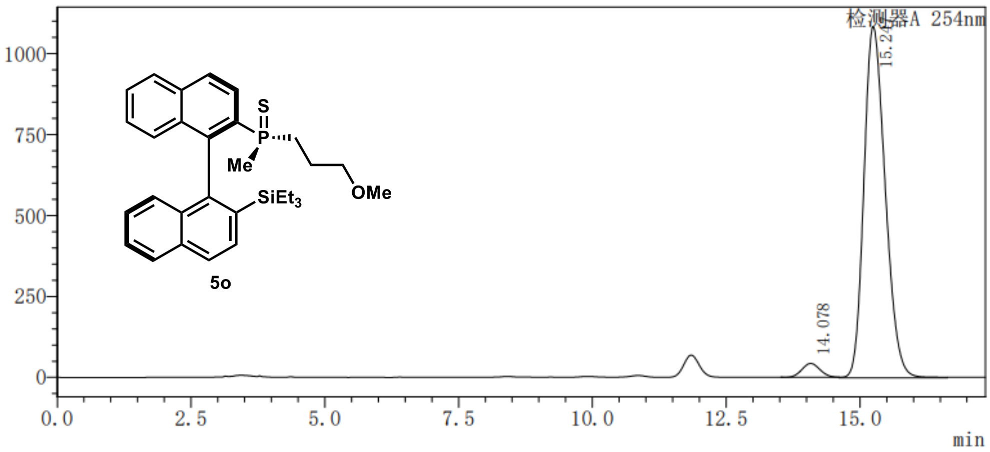

<峰表>

检测器A 254nm

峰号	保留时间	面积	高度	浓度	浓度单位	标记	化合物名
1	14.078	1003573	42810	3.231		M	
2	15.247	30055923	1084288	96.769		M	
总计		31059497	1127098				

C:\LabSolutions\Sample\PLZ-9-121A-IG5%21.lcd

Supplementary Fig. 435. HPLC of **5o**.

SHIMADZU LabSolutions 分析报告

〈样品信息〉

样品名	: PLZ
样品ID	: PLZ-220915
数据文件名	: PLZ-9-159B-IA5%20.lcd
方法文件名	: 1.lcm
批处理文件名	:
样品瓶号	: 1-1
样品类型	: 未知
进样体积	: 1 uL
分析日期	: 2022/11/24 21:16:59
分析者	: System Administrator
处理日期	: 2022/11/24 21:39:37
处理者	: System Administrator

〈色谱图〉

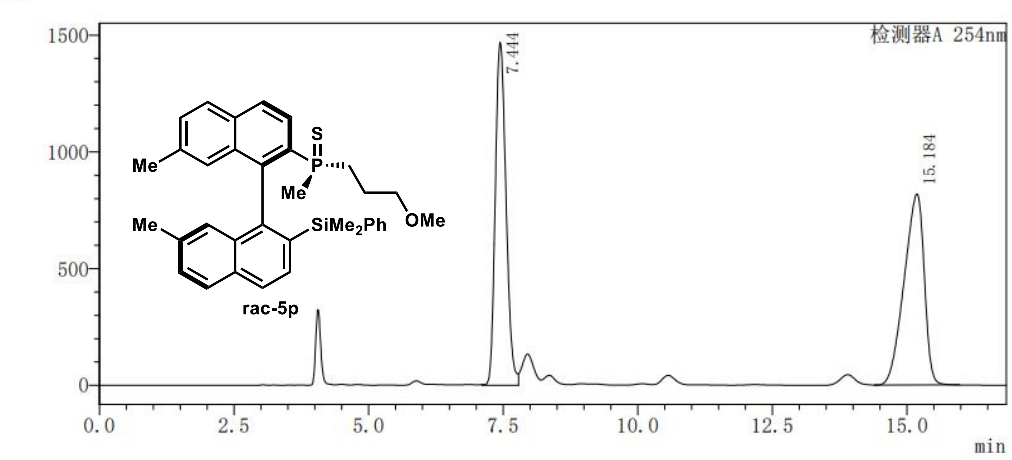

〈峰表〉

检测器A 254nm

峰号	保留时间	面积	高度	浓度	浓度单位	标记	化合物名
1	7.444	20387815	1468945	48.960		M	
2	15.184	21254348	818159	51.040		M	
总计		41642163	2287105				

C:\LabSolutions\Sample\PLZ-9-159B-IA5%20.lcd

Supplementary Fig. 436. HPLC of **5p-rac.**

SHIMADZU LabSolutions 分析报告

<样品信息>

样品名	: PLZ		
样品ID	: PLZ-220915		
数据文件名	: PLZ-9-159A-IA5%21.lcd		
方法文件名	: 1.1cm		
批处理文件名	:		
样品瓶号	: 1-1	样品类型	: 未知
进样体积	: 1 uL		
分析日期	: 2022/11/24 21:35:31	分析者	: System Administrator
处理日期	: 2022/11/24 21:54:04	处理者	: System Administrator

<色谱图>

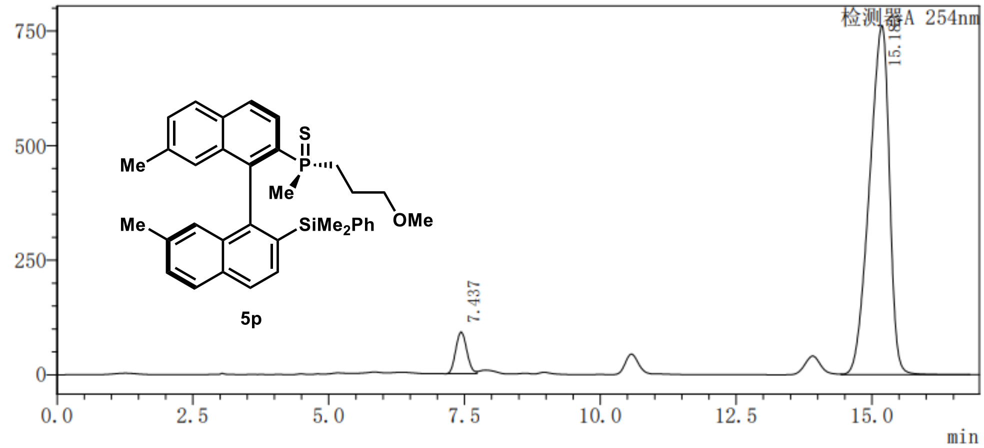

<峰表>

检测器A 254nm

峰号	保留时间	面积	高度	浓度	浓度单位	标记	化合物名
1	7.437	1286307	90529	6.194		M	
2	15.183	19481532	762214	93.806			
总计		20767839	852743				

C:\LabSolutions\Sample\PLZ-9-159A-IA5%21.lcd

Supplementary Fig. 437. HPLC of **5p.**

SHIMADZU LabSolutions 分析报告

<样品信息>

样品名	: PLZ				
样品ID	: PLZ-220915				
数据文件名	: PLZ-9-138B-IG5%.lcd				
方法文件名	: 1.1cm				
批处理文件名	:				
样品瓶号	: 1-1	样品类型	: 未知		
进样体积	: 1 uL				
分析日期	: 2022/11/21 15:16:04	分析者	: System Administrator		
处理日期	: 2022/11/21 15:51:40	处理者	: System Administrator		

<色谱图>

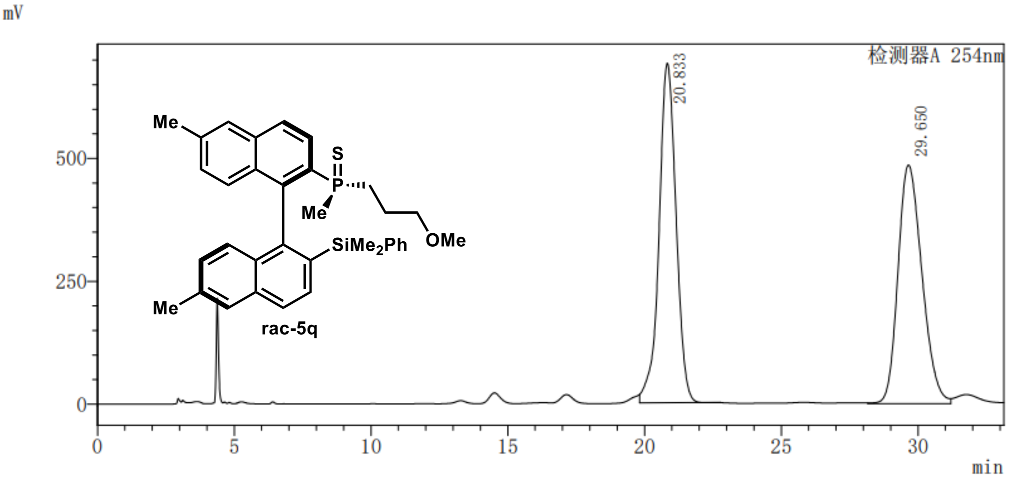

<峰表>

检测器A 254nm

峰号	保留时间	面积	高度	浓度	浓度单位	标记	化合物名
1	20.833	29923370	691003	50.228		M	
2	29.650	29651978	485659	49.772		M	
总计		59575349	1176662				

C:\LabSolutions\Sample\PLZ-9-138B-IG5%.lcd

Supplementary Fig. 438. HPLC of **5q-rac.**

SHIMADZU LabSolutions 分析报告

〈样品信息〉

样品名	: PLZ		
样品ID	: PLZ-220915		
数据文件名	: PLZ-9-138A-IG5%6.lcd		
方法文件名	: 1.1cm		
批处理文件名	:		
样品瓶号	: 1-1	样品类型	: 未知
进样体积	: 1 uL		
分析日期	: 2022/11/21 16:05:06	分析者	: System Administrator
处理日期	: 2022/11/21 17:05:11	处理者	: System Administrator

〈色谱图〉

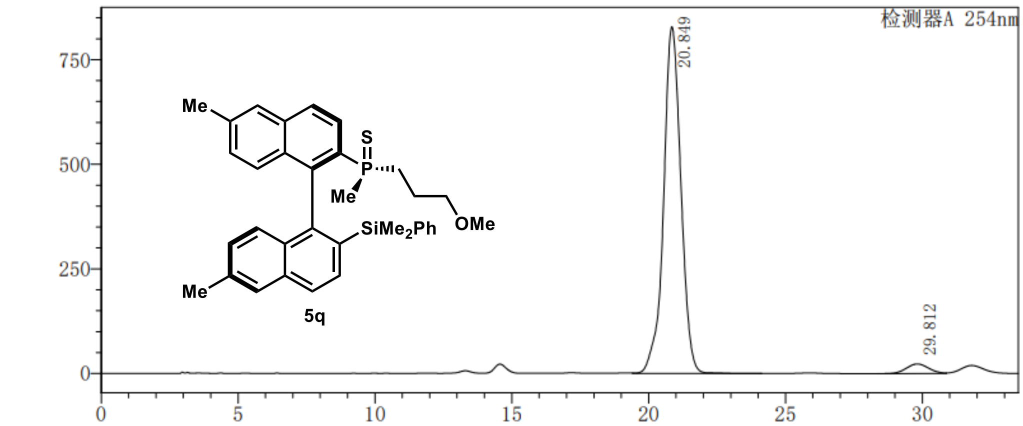

〈峰表〉

检测器A 254nm

峰号	保留时间	面积	高度	浓度	浓度单位	标记	化合物名
1	20.849	36516594	828649	96.510			
2	29.812	1320325	23078	3.490			
总计		37836919	851727				

C:\LabSolutions\Sample\PLZ-9-138A-IG5%6.lcd

Supplementary Fig. 439. HPLC of **5q.**

SHIMADZU LabSolutions 分析报告

〈样品信息〉

样品名	: PLZ		
样品ID	: PLZ-220915		
数据文件名	: PLZ-9-139B-IG5%5.lcd		
方法文件名	: 1.lcm		
批处理文件名	:		
样品瓶号	: 1-1	样品类型	: 未知
进样体积	: 1 uL		
分析日期	: 2022/11/30 10:44:00	分析者	: System Administrator
处理日期	: 2022/11/30 11:07:22	处理者	: System Administrator

〈色谱图〉

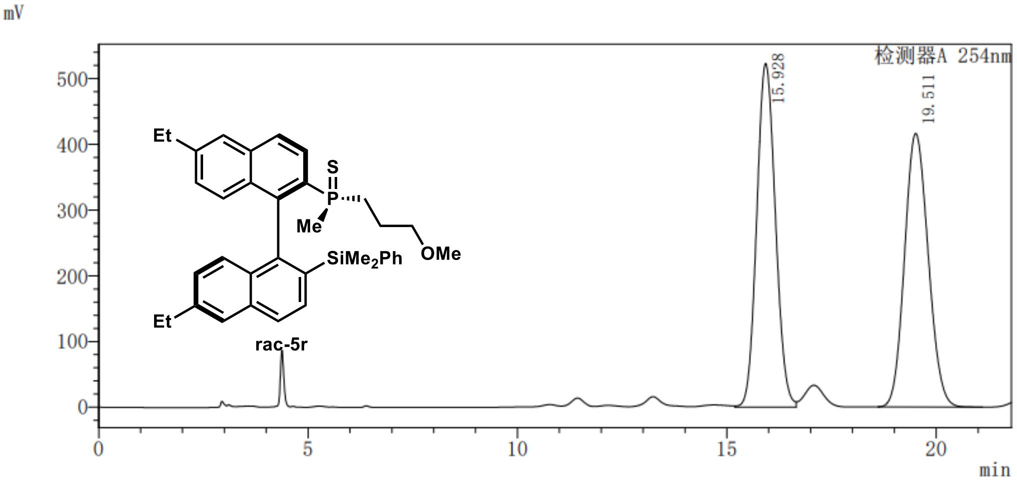

〈峰表〉

检测器A 254nm

峰号	保留时间	面积	高度	浓度	浓度单位	标记	化合物名
1	15.928	16248937	522957	49.849			
2	19.511	16347658	416193	50.151		M	
总计		32596595	939149				

C:\LabSolutions\Sample\PLZ-9-139B-IG5%5.lcd

Supplementary Fig. 440. HPLC of **5r-rac.**

SHIMADZU LabSolutions 分析报告

〈样品信息〉

样品名	: PLZ		
样品ID	: PLZ-220915		
数据文件名	: PLZ-9-139A-IG5%6.lcd		
方法文件名	: 1.lcm		
批处理文件名	:		
样品瓶号	: 1-1	样品类型	: 未知
进样体积	: 1 uL		
分析日期	: 2022/11/30 11:09:13	分析者	: System Administrator
处理日期	: 2022/11/30 11:32:33	处理者	: System Administrator

〈色谱图〉

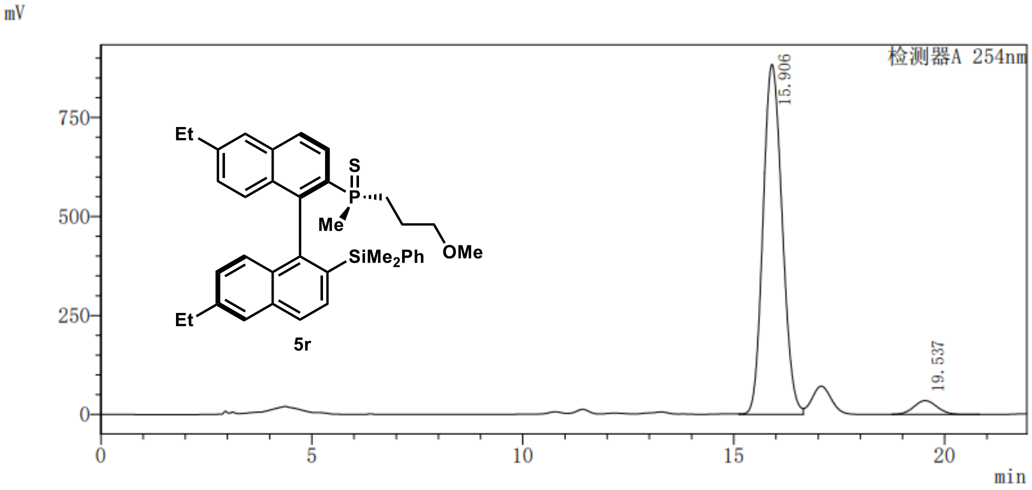

〈峰表〉

检测器A 254nm

峰号	保留时间	面积	高度	浓度	浓度单位	标记	化合物名
1	15.906	27908111	884062	95.427			
2	19.537	1337247	34877	4.573		M	
总计		29245358	918939				

C:\LabSolutions\Sample\PLZ-9-139A-IG5%6.lcd

Supplementary Fig. 441. HPLC of **5r.**

SHIMADZU LabSolutions 分析报告

〈样品信息〉

样品名	: PLZ			
样品ID	: PLZ-220915			
数据文件名	: PLZ-9-158B-IA2.5%22.lcd			
方法文件名	: 1.lcm			
批处理文件名	:			
样品瓶号	: 1-1	样品类型	: 未知	
进样体积	: 1 uL			
分析日期	: 2022/11/24 22:02:07	分析者	: System Administrator	
处理日期	: 2022/11/24 22:39:13	处理者	: System Administrator	

〈色谱图〉

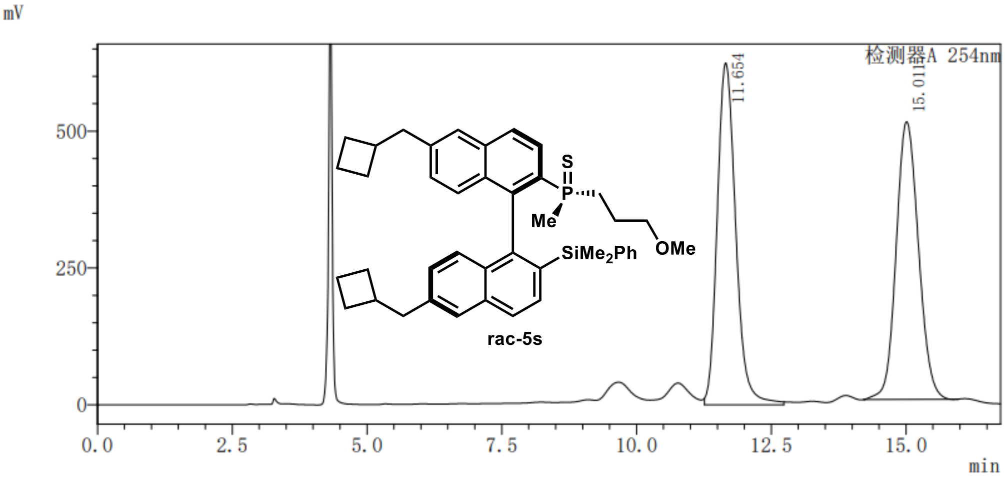

〈峰表〉

检测器A 254nm

峰号	保留时间	面积	高度	浓度	浓度单位	标记	化合物名
1	11.654	14888728	624045	50.468		M	
2	15.011	14612501	507095	49.532		M	
总计		29501229	1131139				

C:\LabSolutions\Sample\PLZ-9-158B-IA2.5%22.lcd

Supplementary Fig. 442. HPLC of **5s-rac.**

SHIMADZU LabSolutions 分析报告

<样品信息>

样品名	: PLZ		
样品ID	: PLZ-220915		
数据文件名	: PLZ-9-158A-IA2.5%23.lcd		
方法文件名	: 1.1cm		
批处理文件名	:		
样品瓶号	: 1-1	样品类型	: 未知
进样体积	: 1 uL		
分析日期	: 2022/11/24 22:19:27	分析者	: System Administrator
处理日期	: 2022/11/24 22:39:09	处理者	: System Administrator

<色谱图>

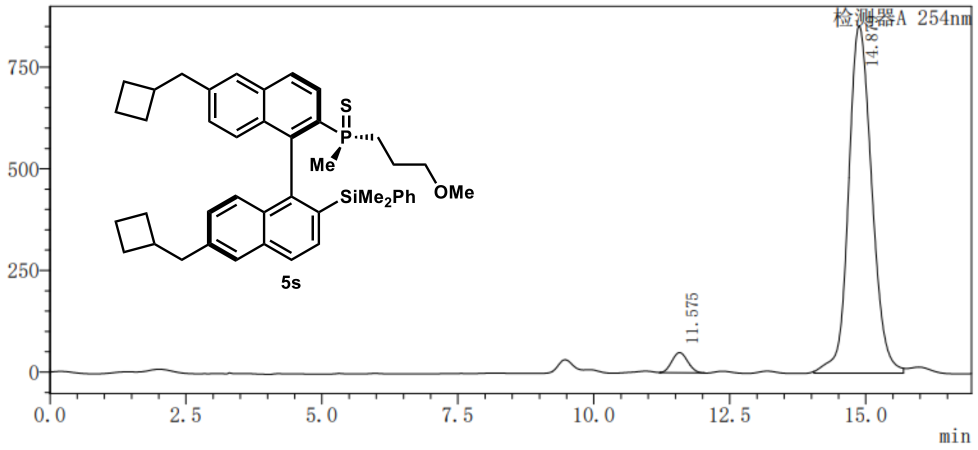

<峰表>

检测器A 254nm

峰号	保留时间	面积	高度	浓度	浓度单位	标记	化合物名
1	11.575	998584	49591	3.782		M	
2	14.879	25406698	854207	96.218		M	
总计		26405282	903798				

C:\LabSolutions\Sample\PLZ-9-158A-IA2.5%23.lcd

Supplementary Fig. 443. HPLC of **5s.**

SHIMADZU LabSolutions 分析报告

〈样品信息〉

样品名	: PLZ		
样品ID	: PLZ-220915		
数据文件名	: PLZ-10-180-IF2.5%1.lcd		
方法文件名	: 322.lcm		
批处理文件名	:		
样品瓶号	: 1-1	样品类型	: 未知
进样体积	: 10 uL		
分析日期	: 2023/5/5 20:22:29	分析者	: System Administrator
处理日期	: 2023/5/6 15:37:10	处理者	: System Administrator

〈色谱图〉

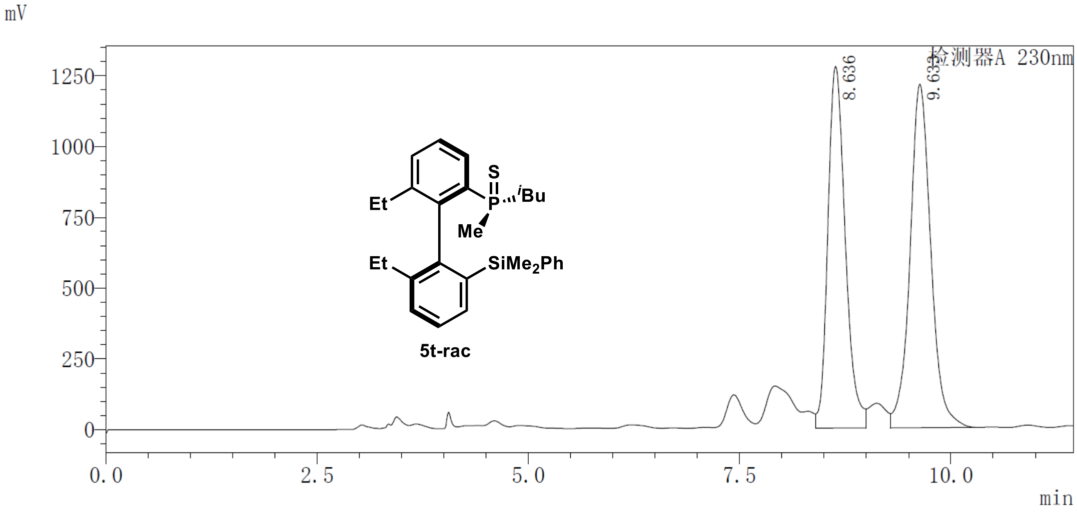

〈峰表〉

检测器A 230nm

峰号	保留时间	面积	高度	浓度	浓度单位	标记	化合物名
1	8.636	18314598	1277030	46.814			
2	9.633	20807375	1213382	53.186		M	
总计		39121972	2490412				

Supplementary Fig. 444. HPLC of **5t-rac.**

SHIMADZU LabSolutions 分析报告

〈样品信息〉

样品名	: PLZ			
样品ID	: PLZ-220915			
数据文件名	: PLZ-10-184-IF2.5%230nm11.lcd			
方法文件名	: 322.lcm			
批处理文件名	:			
样品瓶号	: 1-1	样品类型	: 未知
进样体积	: 10 uL			
分析日期	: 2023/5/6 18:00:48	分析者	: System Administrator
处理日期	: 2023/5/7 16:37:20	处理者	: System Administrator

〈色谱图〉

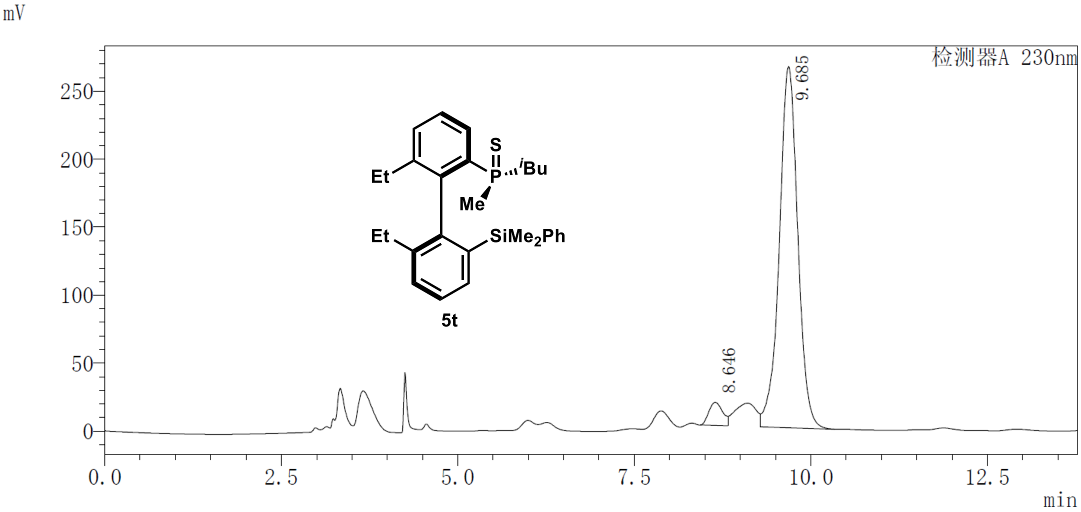

〈峰表〉

检测器A 230nm

峰号	保留时间	面积	高度	浓度	浓度单位	标记	化合物名
1	8.646	232827	17015	4.587		M	
2	9.685	4842709	265691	95.413		M	
总计		5075536	282706				

Supplementary Fig. 445. HPLC of **5t.**

SHIMADZU LabSolutions 分析报告

〈样品信息〉

样品名	:	PLZ			
样品ID	:	PLZ-220915			
数据文件名	:	PLZ-10-169-IC5%2.lcd			
方法文件名	:	322.lcm			
批处理文件名	:				
样品瓶号	:	1-1	样品类型	:	未知
进样体积	:	1 uL			
分析日期	:	2023/4/22 20:03:32	分析者	:	System Administrator
处理日期	:	2023/4/22 20:24:24	处理者	:	System Administrator

〈色谱图〉

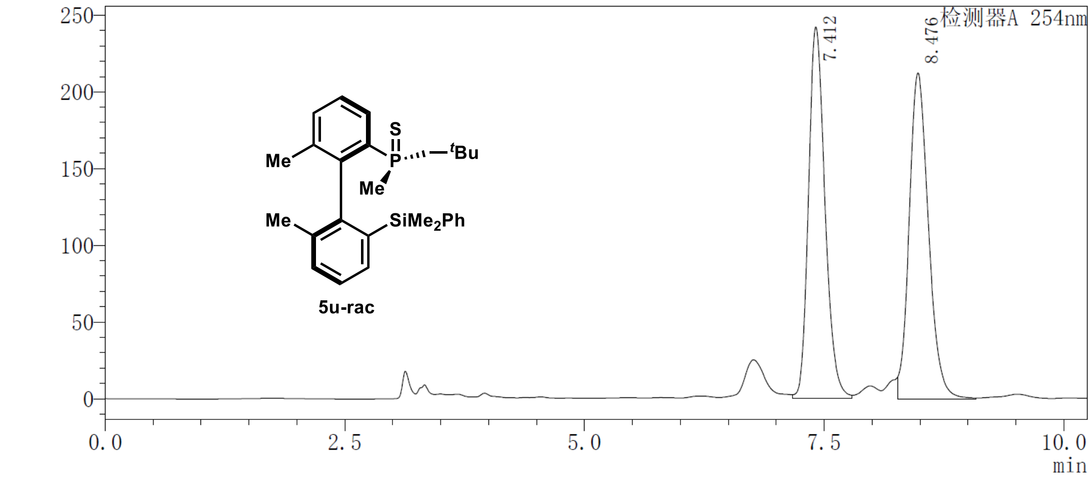

〈峰表〉

检测器A 254nm

峰号	保留时间	面积	高度	浓度	浓度单位	标记	化合物名
1	7.412	2907515	242160	49.447			
2	8.476	2972504	212495	50.553		M	
总计		5880019	454654				

Supplementary Fig. 446. HPLC of **5u-rac.**

SHIMADZU LabSolutions 分析报告

〈样品信息〉

样品名	: PLZ		
样品ID	: PLZ-220915		
数据文件名	: PLZ-10-162-IC5%3.lcd		
方法文件名	: 322.1cm		
批处理文件名	:		
样品瓶号	: 1-1	样品类型	: 未知
进样体积	: 1 uL		
分析日期	: 2023/4/22 20:15:19	分析者	: System Administrator
处理日期	: 2023/4/22 20:25:58	处理者	: System Administrator

〈色谱图〉

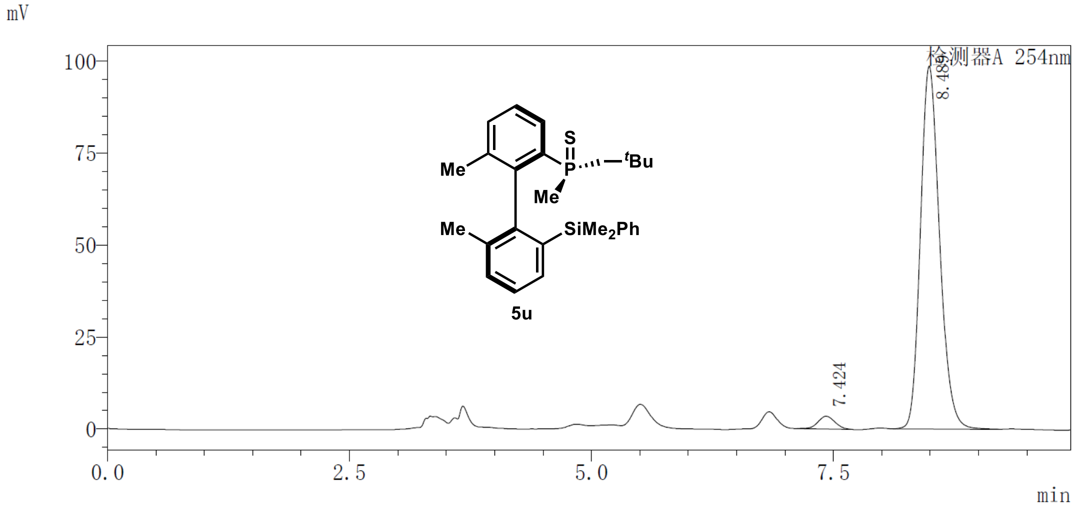

〈峰表〉

检测器A 254nm

峰号	保留时间	面积	高度	浓度	浓度单位	标记	化合物名
1	7.424	42049	3483	2.990		M	
2	8.489	1364131	98782	97.010		M	
总计		1406180	102265				

Supplementary Fig. 447. HPLC of **5u.**

SHIMADZU LabSolutions 分析报告

<样品信息>

样品名	: ZZY		
样品ID	: PLZ-220915		
数据文件名	: PLZ-7-157B-IE20%18.lcd		
方法文件名	: 1.lcm		
批处理文件名	:		
样品瓶号	: 1-1	样品类型	: 未知
进样体积	: 1 uL		
分析日期	: 2022/11/9 14:24:26	分析者	: System Administrator
处理日期	: 2022/11/9 14:51:36	处理者	: System Administrator

<色谱图>

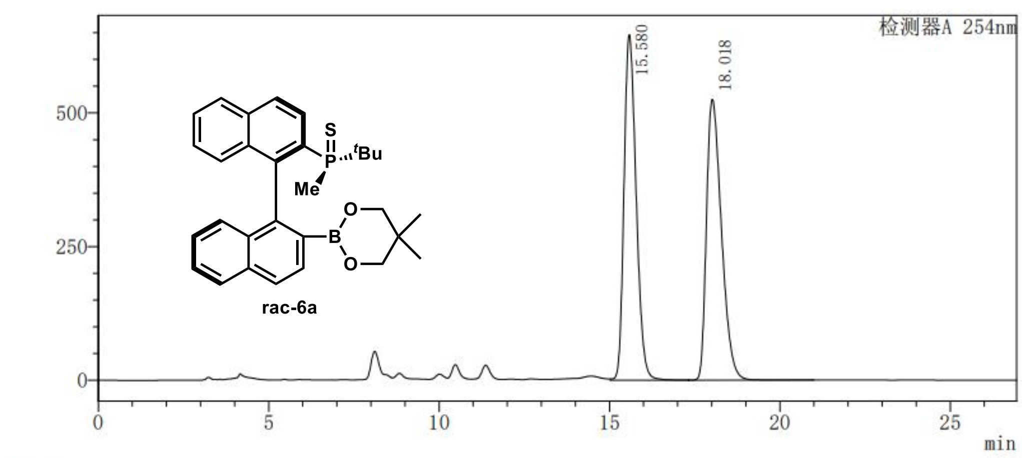

<峰表>

检测器A 254nm

峰号	保留时间	面积	高度	浓度	浓度单位	标记	化合物名
1	15.580	15969774	645811	49.830			
2	18.018	16078548	525248	50.170		V	
总计		32048323	1171058				

C:\LabSolutions\Sample\PLZ-7-157B-IE20%18.lcd

Supplementary Fig. 448. HPLC of **6a-rac.**

SHIMADZU LabSolutions 分析报告

〈样品信息〉

样品名	: PLZ		
样品ID	: PLZ-220915		
数据文件名	: PLZ-8-193A-IE20%16. lcd		
方法文件名	: AD30. 1cm		
批处理文件名	:		
样品瓶号	: 1-1	样品类型	: 未知
进样体积	: 1 uL		
分析日期	: 2022/9/21 19:16:55	分析者	: System Administrator
处理日期	: 2022/11/8 20:00:06	处理者	: System Administrator

〈色谱图〉

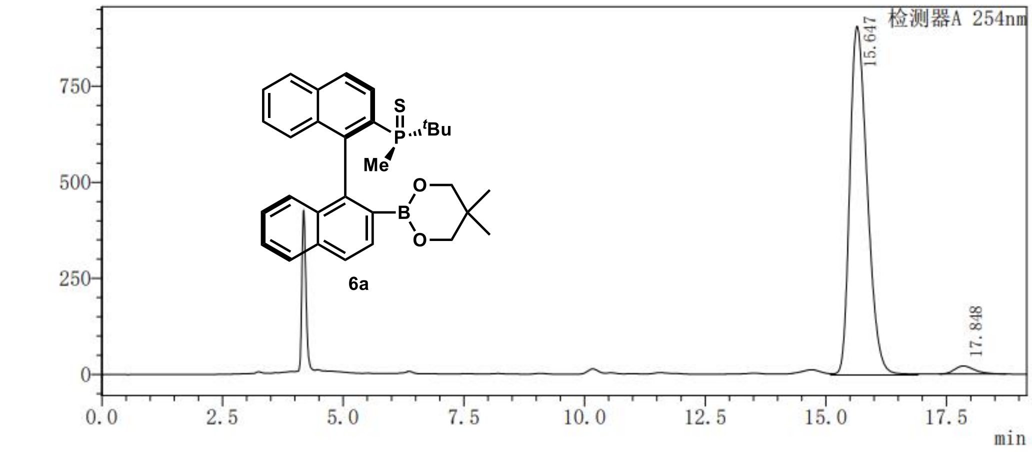

〈峰表〉

检测器A 254nm

峰号	保留时间	面积	高度	浓度	浓度单位	标记	化合物名
1	15. 647	22934227	907832	97. 415		M	
2	17. 848	608482	20886	2. 585		M	
总计		23542709	928718				

C:\LabSolutions\Sample\PLZ-8-193A-IE20%16.lcd

Supplementary Fig. 449. HPLC of **6a.**

SHIMADZU LabSolutions 分析报告

〈样品信息〉

样品名	: PLZ		
样品ID	: PLZ-220915		
数据文件名	: PLZ-9-130B-AD2.5%18.lcd		
方法文件名	: 1.lcm		
批处理文件名	:		
样品瓶号	: 1-1	样品类型	: 未知
进样体积	: 1 uL		
分析日期	: 2022/11/26 19:49:54	分析者	: System Administrator
处理日期	: 2022/11/26 20:12:25	处理者	: System Administrator

〈色谱图〉

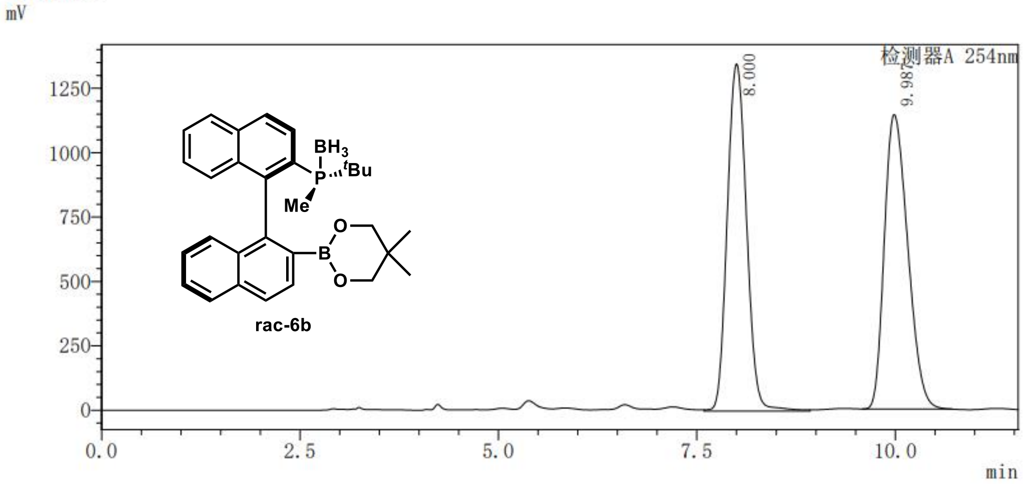

〈峰表〉

检测器A 254nm

峰号	保留时间	面积	高度	浓度	浓度单位	标记	化合物名
1	8.000	22827756	1348374	49.356		M	
2	9.987	23423878	1143507	50.644		M	
总计		46251634	2491881				

C:\LabSolutions\Sample\PLZ-9-130B-AD2.5%18.lcd

Supplementary Fig. 450. HPLC of **6b-rac.**

SHIMADZU LabSolutions 分析报告

〈样品信息〉

样品名	: PLZ		
样品ID	: PLZ-220915		
数据文件名	: PLZ-9-130A-AD2.5%19.lcd		
方法文件名	: 1.lcm		
批处理文件名	:		
样品瓶号	: 1-1	样品类型	: 未知
进样体积	: 1 uL		
分析日期	: 2022/11/26 20:03:47	分析者	: System Administrator
处理日期	: 2022/11/26 20:15:32	处理者	: System Administrator

〈色谱图〉

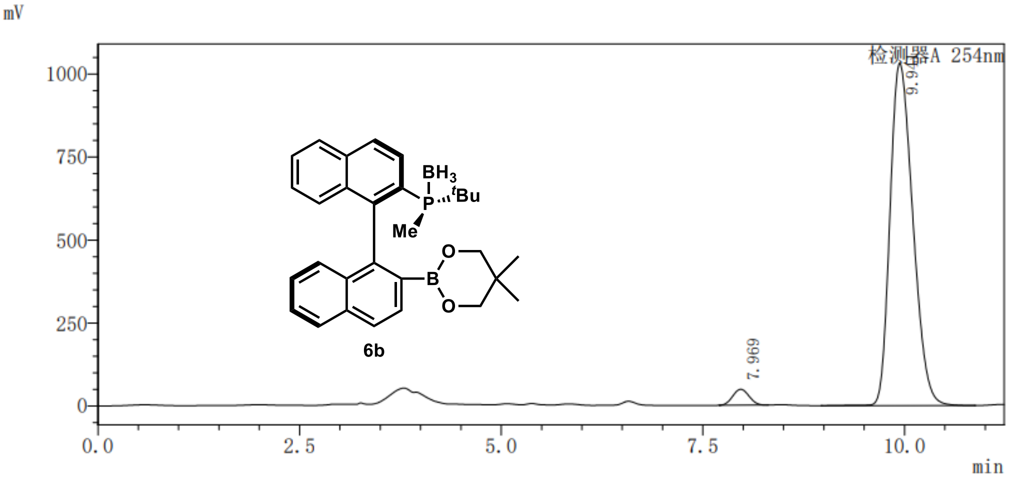

〈峰表〉

检测器A 254nm

峰号	保留时间	面积	高度	浓度	浓度单位	标记	化合物名
1	7.969	675879	48062	3.218		M	
2	9.941	20326963	1032439	96.782			
总计		21002842	1080501				

C:\LabSolutions\Sample\PLZ-9-130A-AD2.5%19.lcd

Supplementary Fig. 451. HPLC of **6b.**

SHIMADZU LabSolutions 分析报告

<样品信息>

样品名	: PLZ		
样品ID	: PLZ-220915		
数据文件名	: PLZ-9-8E-IG5%2. lcd		
方法文件名	: 1. 1cm		
批处理文件名	:		
样品瓶号	: 1-1	样品类型	: 未知
进样体积	: 1 uL		
分析日期	: 2022/11/15 21:50:52	分析者	: System Administrator
处理日期	: 2022/11/15 22:15:41	处理者	: System Administrator

<色谱图>

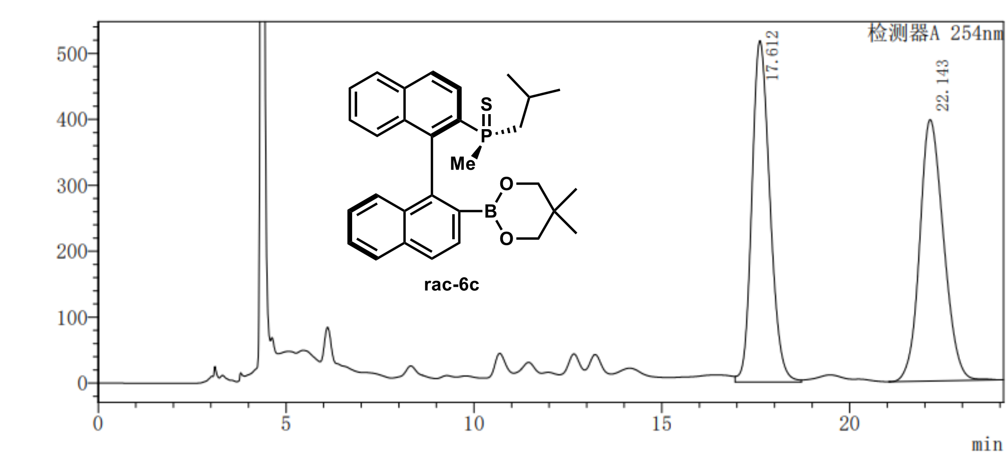

<峰表>

检测器A 254nm

峰号	保留时间	面积	高度	浓度	浓度单位	标记	化合物名
1	17.612	17252069	517517	49.553			
2	22.143	17563232	396503	50.447			
总计		34815302	914020				

C:\LabSolutions\Sample\PLZ-9-8E-IG5%2.lcd

Supplementary Fig. 452. HPLC of **6c-rac.**

SHIMADZU LabSolutions 分析报告

<样品信息>

样品名	: PLZ		
样品ID	: PLZ-220915		
数据文件名	: PLZ-9-117A-IG5%1.lcd		
方法文件名	: 1.lcm		
批处理文件名	:		
样品瓶号	: 1-1	样品类型	: 未知
进样体积	: 1 uL		
分析日期	: 2022/11/15 21:22:51	分析者	: System Administrator
处理日期	: 2022/11/15 22:08:50	处理者	: System Administrator

<色谱图>

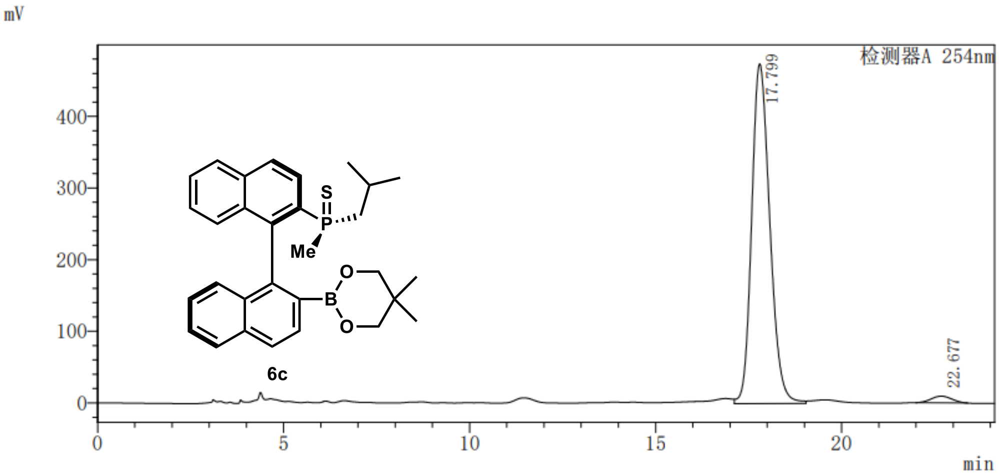

<峰表>

检测器A 254nm

峰号	保留时间	面积	高度	浓度	浓度单位	标记	化合物名
1	17.799	16073797	474150	97.861			
2	22.677	351286	9191	2.139		M	
总计		16425083	483341				

C:\LabSolutions\Sample\PLZ-9-117A-IG5%1.lcd

Supplementary Fig. 453. HPLC of **6c.**

SHIMADZU LabSolutions 分析报告

〈样品信息〉

样品名 : ZZY
样品ID : PLZ-220915
数据文件名 : PLZ-9-8A-IE20%19. lcd
方法文件名 : 1. lcm
批处理文件名 :
样品瓶号 : 1-1　　样品类型 : 未知
进样体积 : 1 uL
分析日期 : 2022/11/9 10:14:06　　分析者 : System Administrator
处理日期 : 2022/11/9 10:30:08　　处理者 : System Administrator

〈色谱图〉

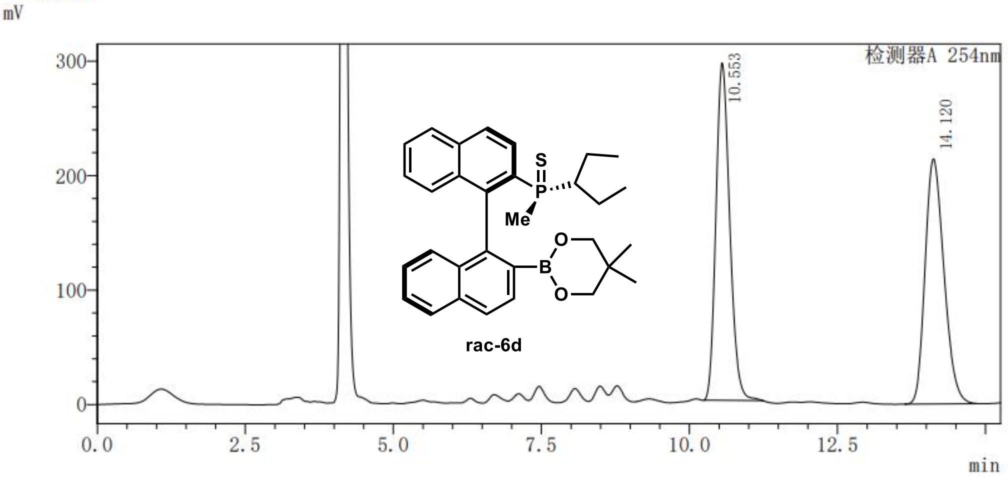

〈峰表〉

检测器A 254nm

峰号	保留时间	面积	高度	浓度	浓度单位	标记	化合物名
1	10.553	4779841	294646	50.171		M	
2	14.120	4747345	214124	49.829		M	
总计		9527186	508771				

C:\LabSolutions\Sample\PLZ-9-8A-IE20%19.lcd

Supplementary Fig. 454. HPLC of **6d-rac.**

SHIMADZU LabSolutions 分析报告

〈样品信息〉

样品名 : ZZY
样品ID : PLZ-220915
数据文件名 : PLZ-9-117B-IE20%16.lcd
方法文件名 : 1.1cm
批处理文件名 :
样品瓶号 : 1-1　　样品类型 : 未知
进样体积 : 1 uL
分析日期 : 2022/11/8 21:48:07　　分析者 : System Administrator
处理日期 : 2022/11/9 10:10:43　　处理者 : System Administrator

〈色谱图〉

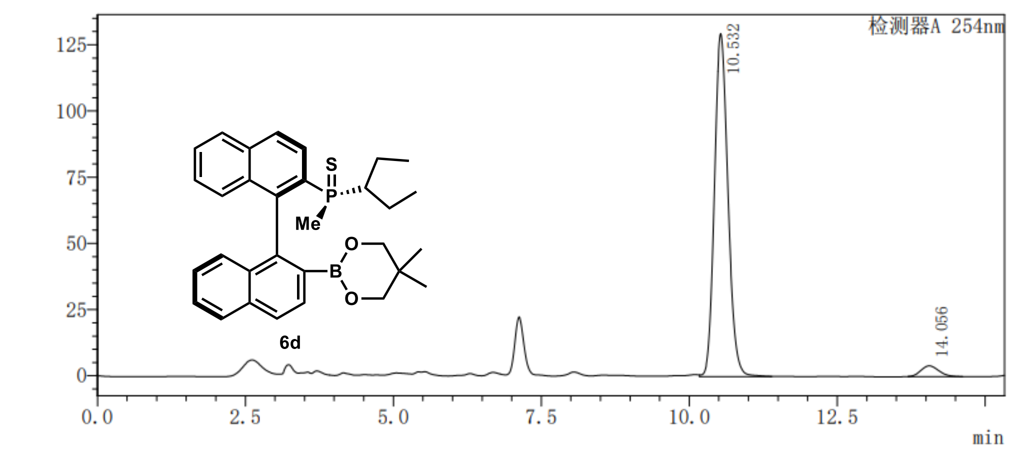

〈峰表〉

检测器A 254nm

峰号	保留时间	面积	高度	浓度	浓度单位	标记	化合物名
1	10.532	2061205	129448	96.040		M	
2	14.056	84987	4061	3.960		M	
总计		2146192	133508				

C:\LabSolutions\Sample\PLZ-9-117B-IE20%16.lcd

Supplementary Fig. 455. HPLC of **6d.**

SHIMADZU LabSolutions 分析报告

〈样品信息〉

样品名	: PLZ		
样品ID	: PLZ-220915		
数据文件名	: PLZ-9-186E-IC5%7.lcd		
方法文件名	: 1.1cm		
批处理文件名	:		
样品瓶号	: 1-1	样品类型	: 未知
进样体积	: 1 uL		
分析日期	: 2022/12/6 23:45:41	分析者	: System Administrator
处理日期	: 2022/12/11 22:37:30	处理者	: System Administrator

〈色谱图〉

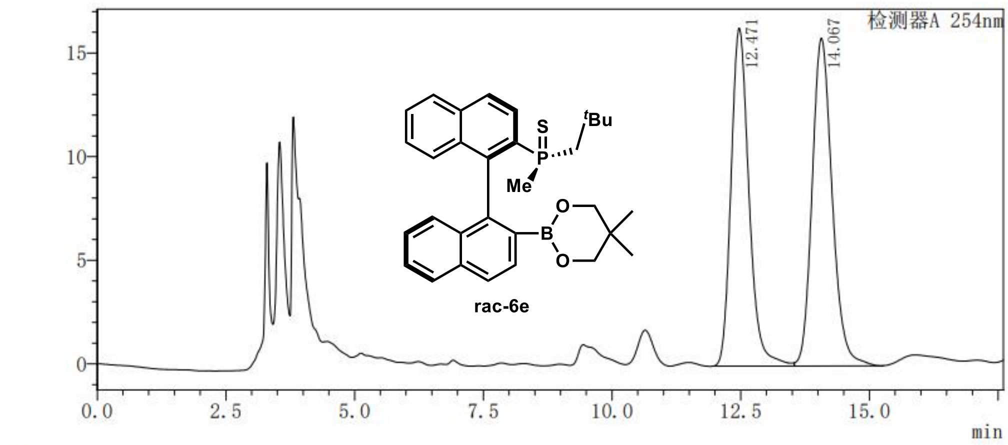

〈峰表〉

检测器A 254nm

峰号	保留时间	面积	高度	浓度	浓度单位	标记	化合物名
1	12.471	395937	16302	48.009		M	
2	14.067	428784	15819	51.991		V M	
总计		824722	32121				

C:\LabSolutions\Sample\PLZ-9-186E-IC5%7.lcd

Supplementary Fig. 456. HPLC of **6e-rac.**

SHIMADZU LabSolutions 分析报告

〈样品信息〉

样品名	: PLZ		
样品ID	: PLZ-220915		
数据文件名	: PLZ-9-180E-IC5%8.lcd		
方法文件名	: 1.lcm		
批处理文件名	:		
样品瓶号	: 1-1	样品类型	: 未知
进样体积	: 1 uL		
分析日期	: 2022/12/7 0:03:39	分析者	: System Administrator
处理日期	: 2022/12/7 0:19:30	处理者	: System Administrator

〈色谱图〉

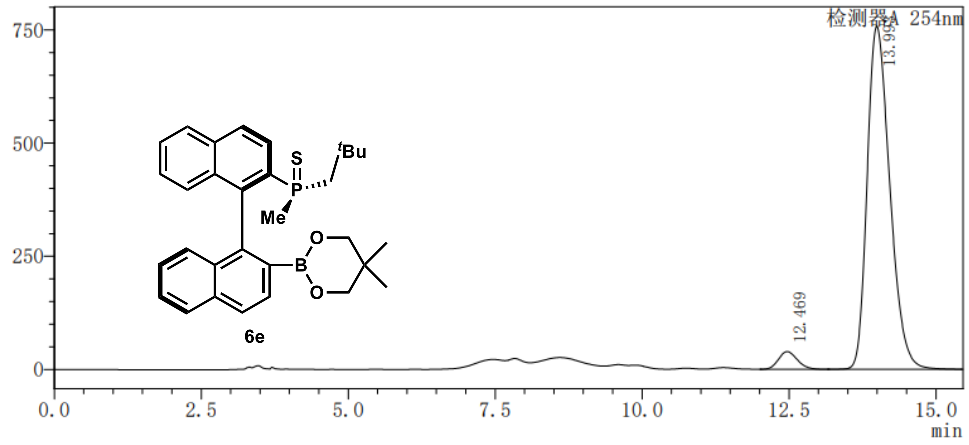

〈峰表〉

检测器A 254nm

峰号	保留时间	面积	高度	浓度	浓度单位	标记	化合物名
1	12.469	882016	39297	4.162			
2	13.993	20311341	758614	95.838			
总计		21193357	797910				

C:\LabSolutions\Sample\PLZ-9-180E-IC5%8.lcd

Supplementary Fig. 457. HPLC of **6e.**

SHIMADZU LabSolutions 分析报告

〈样品信息〉

样品名	: PLZ		
样品ID	: PLZ-220915		
数据文件名	: PLZ-9-8B-IE30%2.lcd		
方法文件名	: 1.1cm		
批处理文件名	:		
样品瓶号	: 1-1	样品类型	: 未知
进样体积	: 1 uL		
分析日期	: 2022/12/8 16:08:17	分析者	: System Administrator
处理日期	: 2022/12/11 22:33:37	处理者	: System Administrator

〈色谱图〉

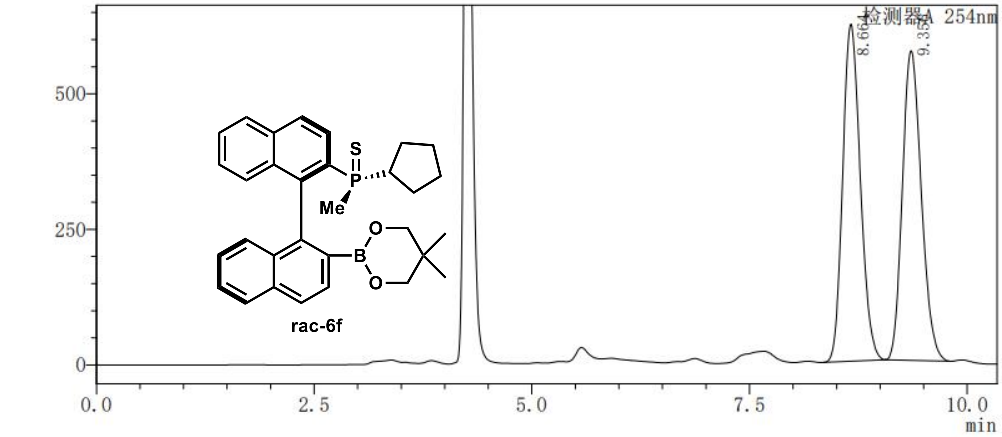

〈峰表〉

检测器A 254nm

峰号	保留时间	面积	高度	浓度	浓度单位	标记	化合物名
1	8.664	8740355	621566	50.279		M	
2	9.356	8643525	571063	49.721		M	
总计		17383880	1192629				

C:\LabSolutions\Sample\PLZ-9-8B-IE30%2.lcd

Supplementary Fig. 458. HPLC of **6f-rac.**

SHIMADZU LabSolutions 分析报告

〈样品信息〉

样品名	: PLZ		
样品ID	: PLZ-220915		
数据文件名	: PLZ-9-197B-IE30%4.lcd		
方法文件名	: 1.1cm		
批处理文件名	:		
样品瓶号	: 1-1	样品类型	: 未知
进样体积	: 1 uL		
分析日期	: 2022/12/8 16:34:47	分析者	: System Administrator
处理日期	: 2022/12/11 22:34:58	处理者	: System Administrator

〈色谱图〉

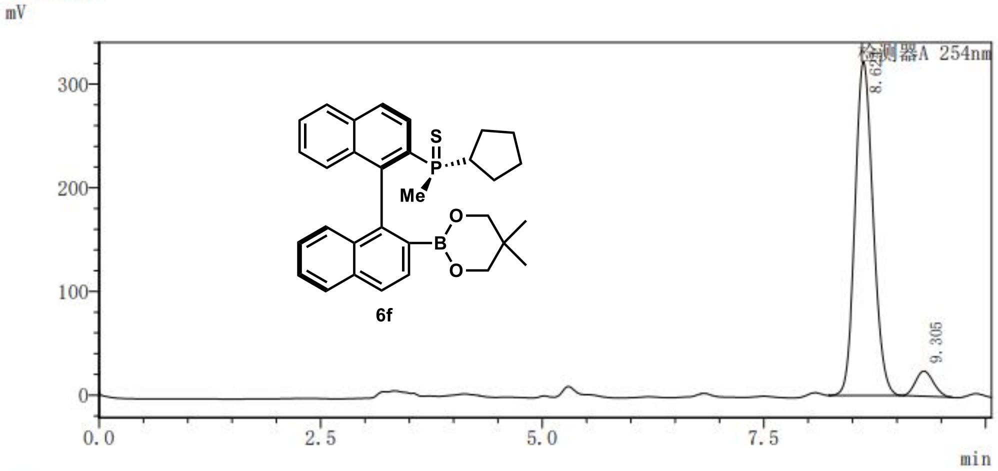

〈峰表〉

检测器A 254nm

峰号	保留时间	面积	高度	浓度	浓度单位	标记	化合物名
1	8.624	4566333	322279	93.050		M	
2	9.305	341058	24257	6.950		M	
总计		4907391	346535				

C:\LabSolutions\Sample\PLZ-9-197B-IE30%4.lcd

Supplementary Fig. 459. HPLC of **6f.**

SHIMADZU LabSolutions 分析报告

〈样品信息〉

样品名	: PLZ		
样品ID	: PLZ-220915		
数据文件名	: PLZ-10-170-IC20%20.lcd		
方法文件名	: 322.lcm		
批处理文件名	:		
样品瓶号	: 1-1	样品类型	: 未知
进样体积	: 1 uL		
分析日期	: 2023/4/23 16:53:07	分析者	: System Administrator
处理日期	: 2023/5/8 11:42:47	处理者	: System Administrator

〈色谱图〉

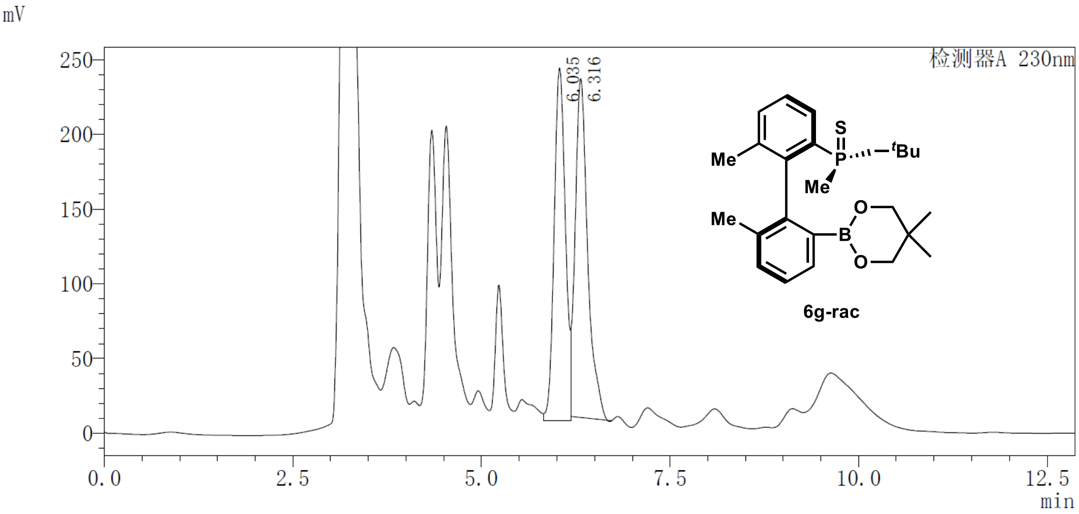

〈峰表〉

检测器A 230nm

峰号	保留时间	面积	高度	浓度	浓度单位	标记	化合物名
1	6.035	2449245	236386	48.399		M	
2	6.316	2611249	226875	51.601		M	
总计		5060494	463261				

Supplementary Fig. 460. HPLC of **6g-rac.**

SHIMADZU LabSolutions 分析报告

〈样品信息〉

样品名	: PLZ		
样品ID	: PLZ-220915		
数据文件名	: PLZ-10-163-IC20%21.lcd		
方法文件名	: 322.lcm		
批处理文件名	:		
样品瓶号	: 1-1	样品类型	: 未知
进样体积	: 1 uL		
分析日期	: 2023/4/23 17:06:47	分析者	: System Administrator
处理日期	: 2023/4/23 17:16:40	处理者	: System Administrator

〈色谱图〉

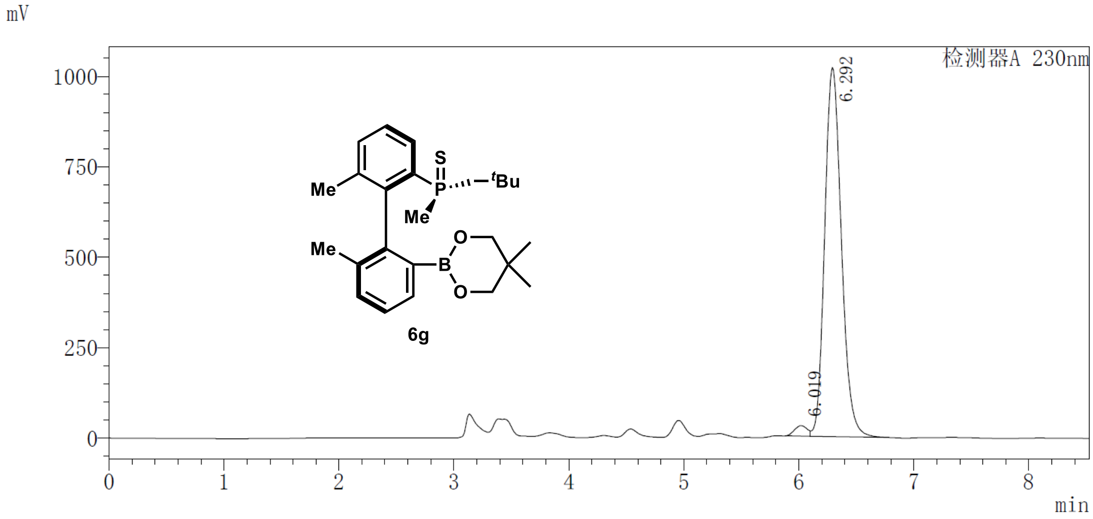

〈峰表〉

检测器A 230nm

峰号	保留时间	面积	高度	浓度	浓度单位	标记	化合物名
1	6.019	227942	29099	2.167		M	
2	6.292	10291729	1020222	97.833		V M	
总计		10519671	1049320				

Supplementary Fig. 461. HPLC of **6g.**

SHIMADZU LabSolutions 分析报告

<样品信息>

样品名	: PLZ		
样品ID	: PLZ-220915		
数据文件名	: SQL-5-70-2-IA5%10. lcd		
方法文件名	: AD30. lcm		
批处理文件名	:		
样品瓶号	: 1-1	样品类型	: 未知
进样体积	: 1 uL		
分析日期	: 2022/9/22 10:48:43	分析者	: System Administrator
处理日期	: 2022/9/22 11:11:30	处理者	: System Administrator

<色谱图>

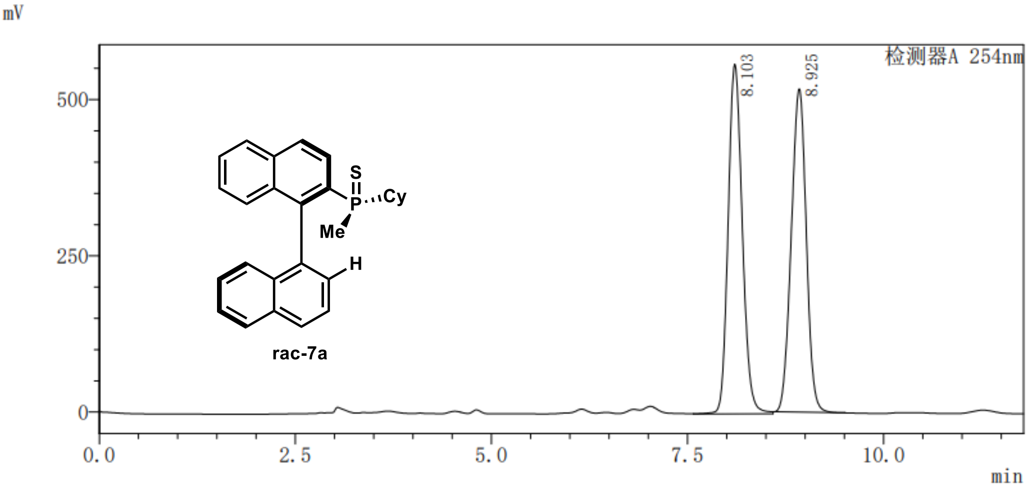

<峰表>

检测器A 254nm

峰号	保留时间	面积	高度	浓度	浓度单位	标记	化合物名
1	8.103	7036048	559567	50.569			
2	8.925	6877709	517623	49.431		M	
总计		13913756	1077190				

C:\LabSolutions\Sample\SQL-5-70-2-IA5%10.lcd

Supplementary Fig. 462. HPLC of **7a-rac.**

SHIMADZU LabSolutions 分析报告

〈样品信息〉

样品名	: ZZY			
样品ID	: PLZ-220915			
数据文件名	: PLZ-9-56-IA5%1.lcd			
方法文件名	: 1.lcm			
批处理文件名	:			
样品瓶号	: 1-1	样品类型	: 未知	
进样体积	: 1 uL			
分析日期	: 2022/10/13 21:10:03	分析者	: System Administrator	
处理日期	: 2022/10/13 21:21:45	处理者	: System Administrator	

〈色谱图〉

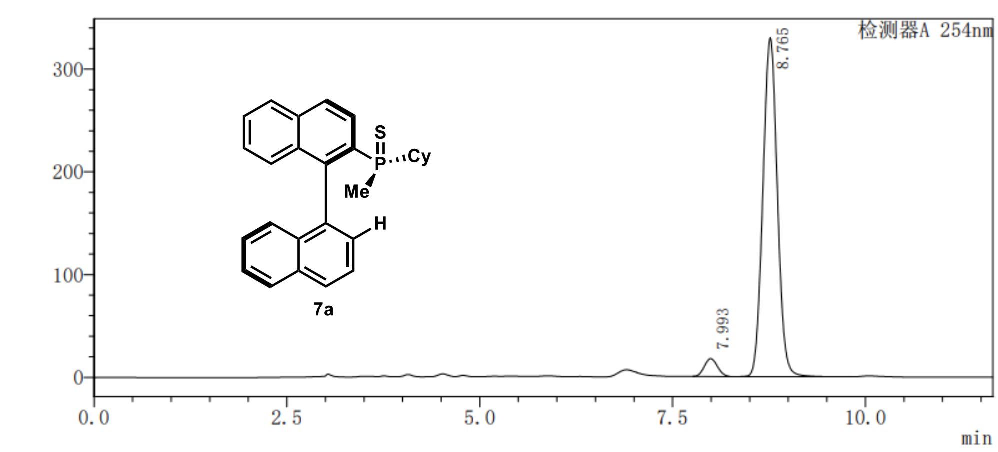

〈峰表〉

检测器A 254nm

峰号	保留时间	面积	高度	浓度	浓度单位	标记	化合物名
1	7.993	206769	17443	4.566		M	
2	8.765	4321687	329970	95.434		M	
总计		4528456	347413				

C:\LabSolutions\Sample\PLZ-9-56-IA5%1.lcd

Supplementary Fig. 463. HPLC of **7a.**

SHIMADZU LabSolutions 分析报告

<样品信息>

样品名	: ZZY			
样品ID	: PLZ-220915			
数据文件名	: PLZ-9-94B-IE10%4.lcd			
方法文件名	: 1.lcm			
批处理文件名	:			
样品瓶号	: 1-1	样品类型	: 未知	
进样体积	: 1 uL			
分析日期	: 2022/11/1 9:31:31	分析者	: System Administrator	
处理日期	: 2022/11/1 9:48:00	处理者	: System Administrator	

<色谱图>

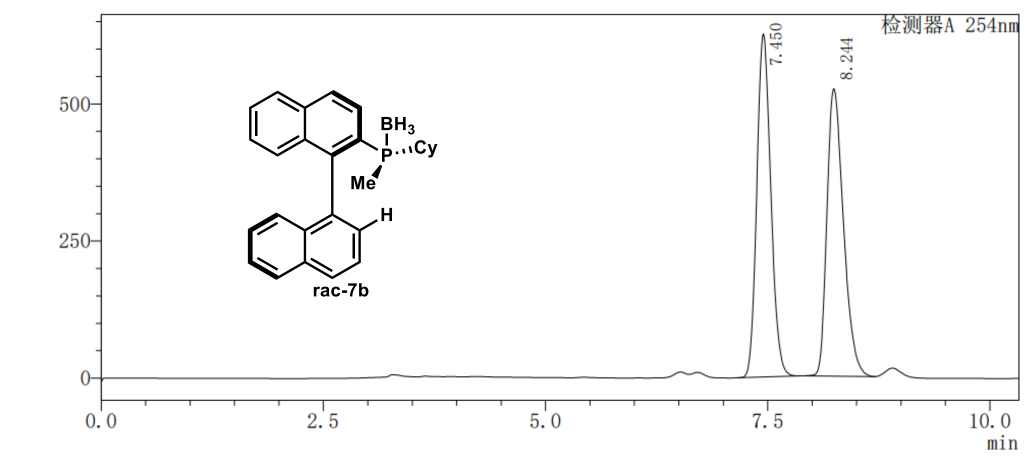

<峰表>

检测器A 254nm

峰号	保留时间	面积	高度	浓度	浓度单位	标记	化合物名
1	7.450	6863606	625738	50.220		M	
2	8.244	6803401	524332	49.780		M	
总计		13667008	1150070				

C:\LabSolutions\Sample\PLZ-9-94B-IE10%4.lcd

Supplementary Fig. 464. HPLC of **7b-rac.**

SHIMADZU LabSolutions 分析报告

〈样品信息〉

样品名	: ZZY		
样品ID	: PLZ-220915		
数据文件名	: PLZ-9-94A-IE10%5.lcd		
方法文件名	: 1.lcm		
批处理文件名	:		
样品瓶号	: 1-1	样品类型	: 未知
进样体积	: 1 uL		
分析日期	: 2022/11/1 9:45:25	分析者	: System Administrator
处理日期	: 2022/11/1 9:58:24	处理者	: System Administrator

〈色谱图〉

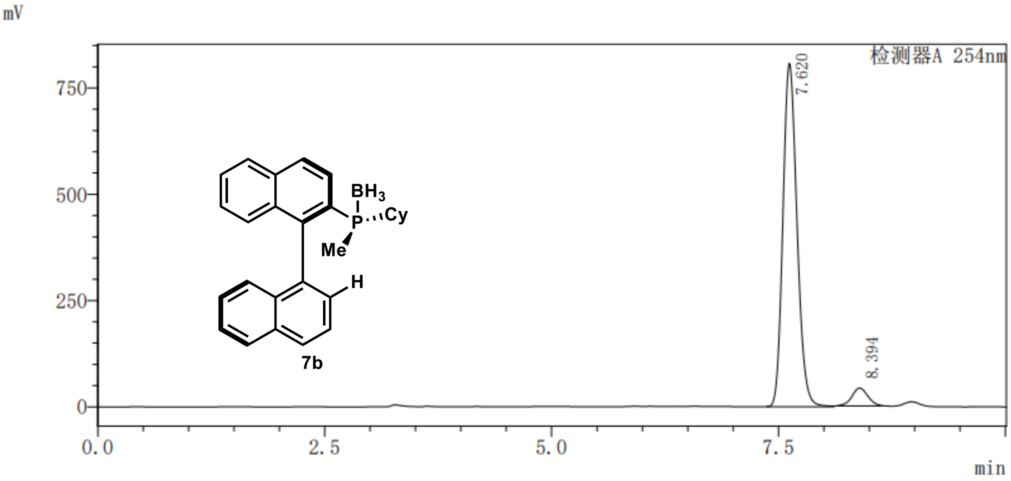

〈峰表〉

检测器A 254nm

峰号	保留时间	面积	高度	浓度	浓度单位	标记	化合物名
1	7.620	8728081	807839	94.352		M	
2	8.394	522516	42236	5.648		M	
总计		9250597	850075				

C:\LabSolutions\Sample\PLZ-9-94A-IE10%5.lcd

Supplementary Fig. 465. HPLC of **7b.**

SHIMADZU LabSolutions

分析报告

〈样品信息〉

样品名	: ZZY		
样品ID	: PLZ-220915		
数据文件名	: SQL-5-68-4-IA5%12.lcd		
方法文件名	: 1.lcm		
批处理文件名	:		
样品瓶号	: 1-1	样品类型	: 未知
进样体积	: 1 uL		
分析日期	: 2022/10/22 9:33:05	分析者	: System Administrator
处理日期	: 2022/10/22 10:18:43	处理者	: System Administrator

〈色谱图〉

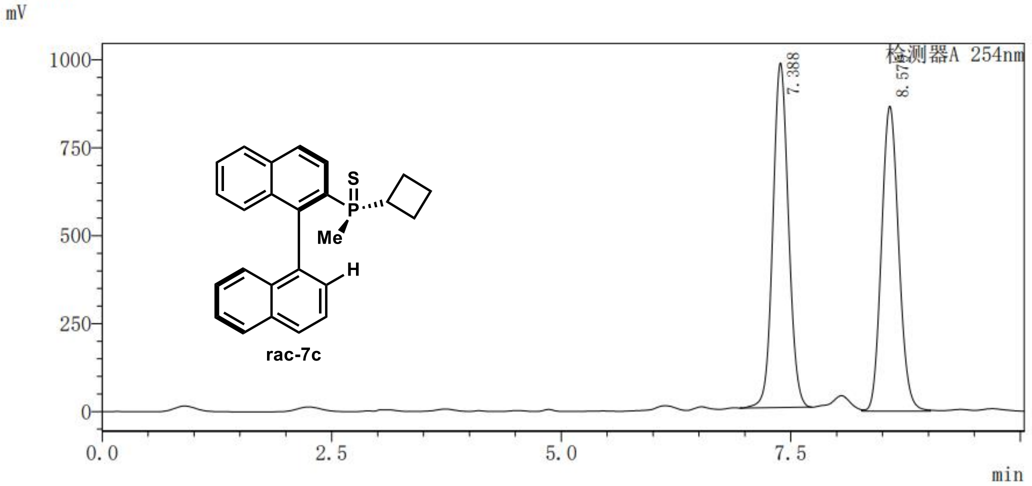

〈峰表〉

检测器A 254nm

峰号	保留时间	面积	高度	浓度	浓度单位	标记	化合物名
1	7.388	11535812	979888	50.531		M	
2	8.579	11293167	866685	49.469		M	
总计		22828980	1846572				

C:\LabSolutions\Sample\SQL-5-68-4-IA5%12.lcd

Supplementary Fig. 466. HPLC of **7c-rac.**

SHIMADZU LabSolutions 分析报告

〈样品信息〉

样品名	: ZZY			
样品ID	: PLZ-220915			
数据文件名	: PLZ-9-37A-IA5%13.lcd			
方法文件名	: 1.lcm			
批处理文件名	:			
样品瓶号	: 1-1	样品类型	: 未知	
进样体积	: 1 uL			
分析日期	: 2022/10/22 9:44:27	分析者	: System Administrator	
处理日期	: 2022/10/22 10:19:52	处理者	: System Administrator	

〈色谱图〉

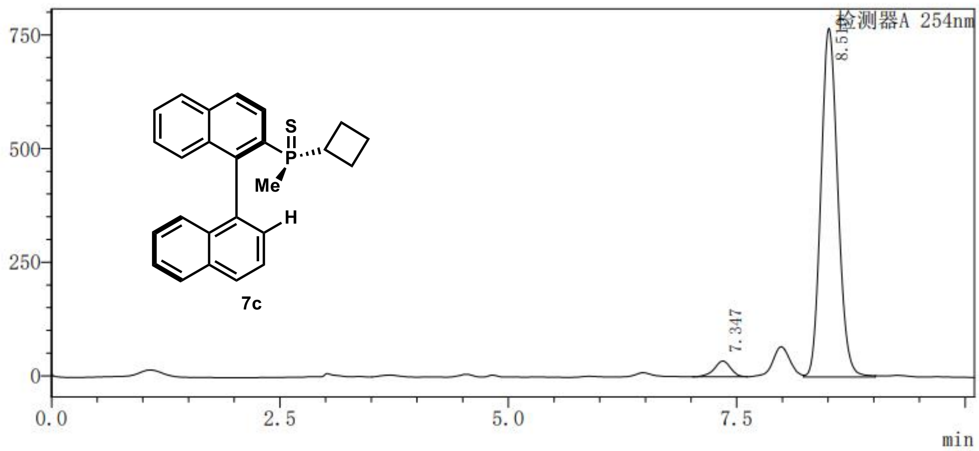

〈峰表〉

检测器A 254nm

峰号	保留时间	面积	高度	浓度	浓度单位	标记	化合物名
1	7.347	426657	34489	4.144		M	
2	8.510	9869336	766548	95.856		M	
总计		10295993	801037				

C:\LabSolutions\Sample\PLZ-9-37A-IA5%13.lcd

Supplementary Fig. 467. HPLC of **7c.**

SHIMADZU LabSolutions 分析报告

〈样品信息〉

样品名	: PLZ		
样品ID	: PLZ-220915		
数据文件名	: SQL-5-68-1-IA10%25.lcd		
方法文件名	: AD30.lcm		
批处理文件名	:		
样品瓶号	: 1-1	样品类型	: 未知
进样体积	: 1 uL		
分析日期	: 2022/9/23 13:01:41	分析者	: System Administrator
处理日期	: 2022/10/21 9:31:51	处理者	: System Administrator

〈色谱图〉

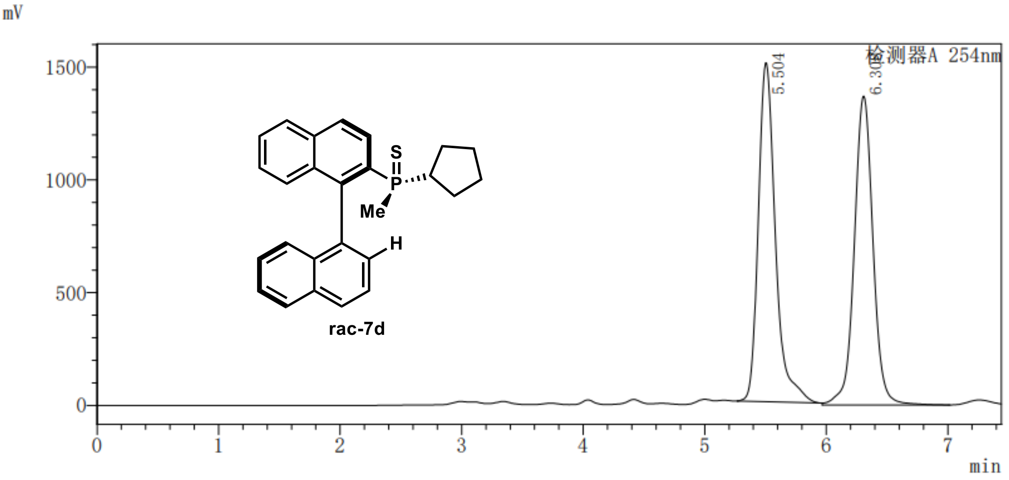

〈峰表〉

检测器A 254nm

峰号	保留时间	面积	高度	浓度	浓度单位	标记	化合物名
1	5.504	14722475	1502093	50.174		M	
2	6.308	14620292	1369821	49.826			
总计		29342767	2871915				

C:\LabSolutions\Sample\SQL-5-68-1-IA10%25.lcd

Supplementary Fig. 468. HPLC of **7d-rac.**

SHIMADZU LabSolutions 分析报告

<样品信息>

样品名	:		
样品ID	:		
数据文件名	: PLZ-9-82B-IA10%.lcd		
方法文件名	: test.lcm		
批处理文件名	:		
样品瓶号	: 1-1	样品类型	: 未知
进样体积	: 1 uL		
分析日期	: 2022/10/25 21:18:34	分析者	: System Administrator
处理日期	: 2022/10/26 11:45:00	处理者	: System Administrator

<色谱图>

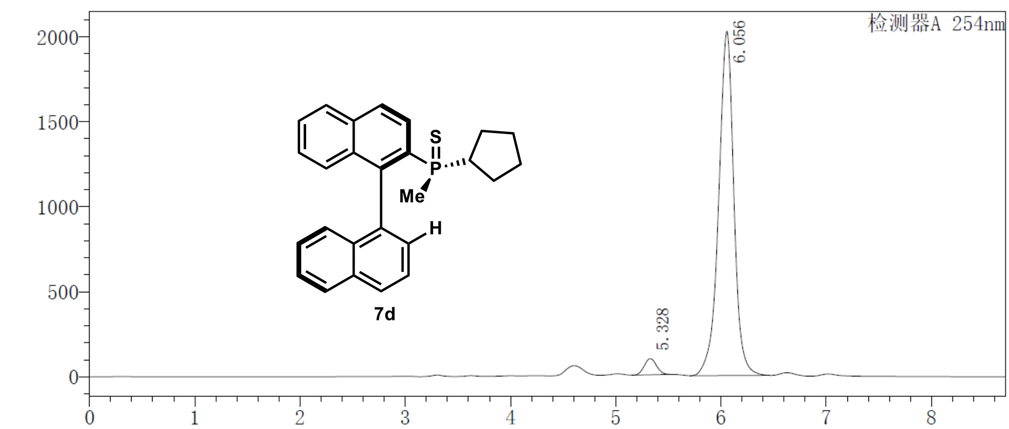

7d

<峰表>

检测器A 254nm

峰号	保留时间	面积	高度	浓度	浓度单位	标记	化合物名
1	5.328	769857	95019	3.634		M	
2	6.056	20412666	2026470	96.366		M	
总计		21182523	2121489				

C:\LabSolutions\Data\Project1\PLZ-9-82B-IA10%.lcd

Supplementary Fig. 469. HPLC of **7d.**

SHIMADZU LabSolutions 分析报告

〈样品信息〉

样品名	: PLZ			
样品ID	: PLZ-220915			
数据文件名	: SQL-5-68-6-IE10%1.lcd			
方法文件名	: AD30.lcm			
批处理文件名	:			
样品瓶号	: 1-1	样品类型	: 未知	
进样体积	: 1 uL			
分析日期	: 2022/9/20 19:57:08	分析者	: System Administrator	
处理日期	: 2022/9/20 20:24:49	处理者	: System Administrator	

〈色谱图〉

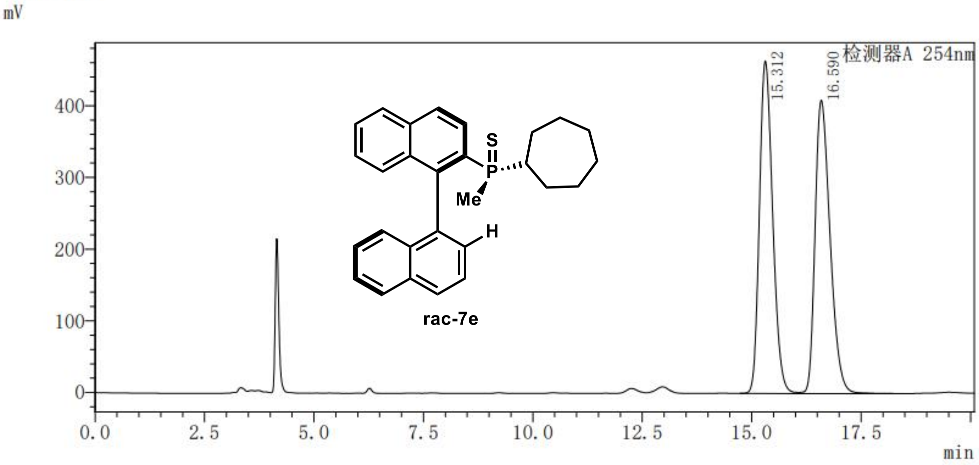

〈峰表〉

检测器A 254nm

峰号	保留时间	面积	高度	浓度	浓度单位	标记	化合物名
1	15.312	9756733	463368	50.049			
2	16.590	9737467	409001	49.951		V	
总计		19494200	872369				

C:\LabSolutions\Sample\SQL-5-68-6-IE10%1.lcd

Supplementary Fig. 470. HPLC of **7e-rac.**

SHIMADZU LabSolutions 分析报告

<样品信息>

样品名	: ZZY		
样品ID	: PLZ-220915		
数据文件名	: PLZ-9-78C-IE10%6. lcd		
方法文件名	: 1. lcm		
批处理文件名	:		
样品瓶号	: 1-1	样品类型	: 未知
进样体积	: 1 uL		
分析日期	: 2022/10/21 21:25:36	分析者	: System Administrator
处理日期	: 2022/10/21 21:44:02	处理者	: System Administrator

<色谱图>

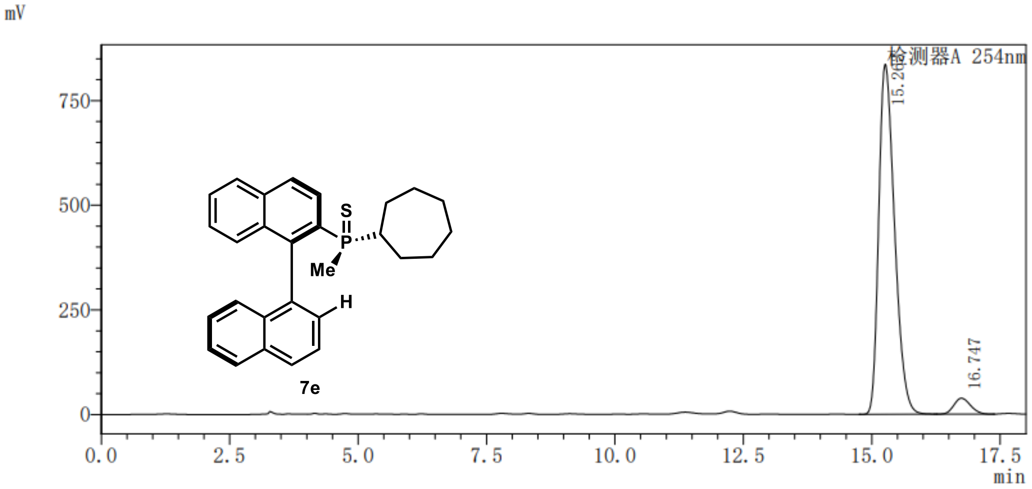

<峰表>

检测器A 254nm

峰号	保留时间	面积	高度	浓度	浓度单位	标记	化合物名
1	15. 263	17902845	835996	95. 457		M	
2	16. 747	851982	38027	4. 543		M	
总计		18754827	874023				

C:\LabSolutions\Sample\PLZ-9-78C-IE10%6.lcd

Supplementary Fig. 471. HPLC of **7e.**

SHIMADZU LabSolutions 分析报告

<样品信息>

样品名	: ZZY		
样品ID	: PLZ-220915		
数据文件名	: SQL-5-68-2-IC10%3.lcd		
方法文件名	: 1.lcm		
批处理文件名	:		
样品瓶号	: 1-1	样品类型	: 未知
进样体积	: 1 uL		
分析日期	: 2022/10/12 15:05:12	分析者	: System Administrator
处理日期	: 2022/10/12 15:21:04	处理者	: System Administrator

<色谱图>

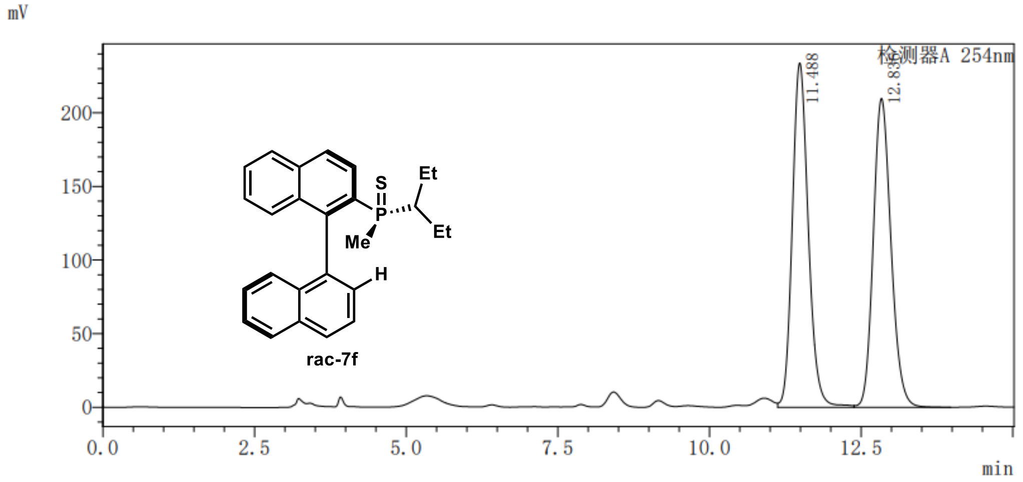

<峰表>

检测器A 254nm

峰号	保留时间	面积	高度	浓度	浓度单位	标记	化合物名
1	11.488	4280860	233741	50.154			
2	12.836	4254540	209765	49.846		V	
总计		8535399	443507				

C:\LabSolutions\Sample\SQL-5-68-2-IC10%3.lcd

Supplementary Fig. 472. HPLC of **7f-rac.**

SHIMADZU LabSolutions 分析报告

〈样品信息〉

样品名 : ZZY
样品ID : PLZ-220915
数据文件名 : PLZ-9-3B-IC10%2.lcd
方法文件名 : 1.lcm
批处理文件名 :
样品瓶号 : 1-1 样品类型 : 未知
进样体积 : 1 uL
分析日期 : 2022/10/12 14:50:13 分析者 : System Administrator
处理日期 : 2022/10/12 15:06:43 处理者 : System Administrator

〈色谱图〉

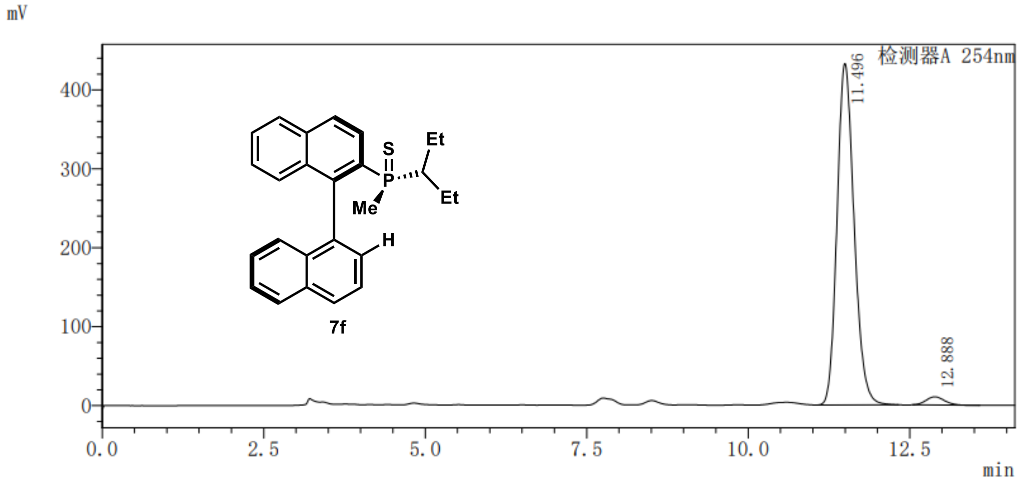

〈峰表〉

检测器A 254nm

峰号	保留时间	面积	高度	浓度	浓度单位	标记	化合物名
1	11.496	7949004	432331	97.478		M	
2	12.888	205653	10331	2.522		M	
总计		8154656	442663				

C:\LabSolutions\Sample\PLZ-9-3B-IC10%2.lcd

Supplementary Fig. 473. HPLC of **7f.**

SHIMADZU LabSolutions 分析报告

〈样品信息〉

样品名 : PLZ
样品ID : PLZ-220915
数据文件名 : SQL-5-68-3-IE30%1.lcd
方法文件名 : AD30.lcm
批处理文件名 :
样品瓶号 : 1-1 样品类型 : 未知
进样体积 : 1 uL
分析日期 : 2022/9/21 19:45:03 分析者 : System Administrator
处理日期 : 2022/10/12 15:08:08 处理者 : System Administrator

〈色谱图〉

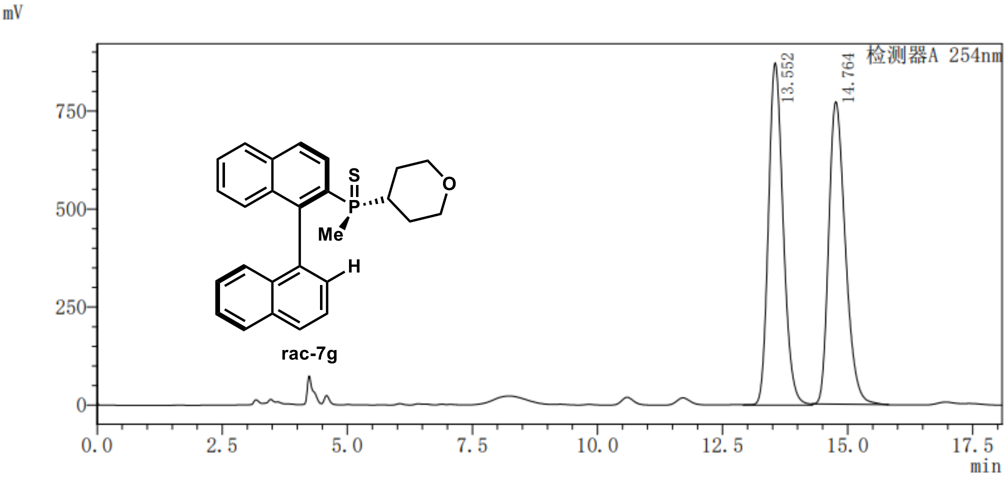

〈峰表〉

检测器A 254nm

峰号	保留时间	面积	高度	浓度	浓度单位	标记	化合物名
1	13.552	17909973	872511	50.197			
2	14.764	17769071	770897	49.803		M	
总计		35679043	1643408				

C:\LabSolutions\Sample\SQL-5-68-3-IE30%1.lcd

Supplementary Fig. 474. HPLC of **7g-rac.**

SHIMADZU LabSolutions 分析报告

〈样品信息〉

样品名	: ZZY			
样品ID	: PLZ-220915			
数据文件名	: PLZ-9-3C-IE30%2.lcd			
方法文件名	: 1.1cm			
批处理文件名	:			
样品瓶号	: 1-1	样品类型	: 未知	
进样体积	: 1 uL			
分析日期	: 2022/10/17 9:46:30	分析者	: System Administrator	
处理日期	: 2022/10/17 10:06:33	处理者	: System Administrator	

〈色谱图〉

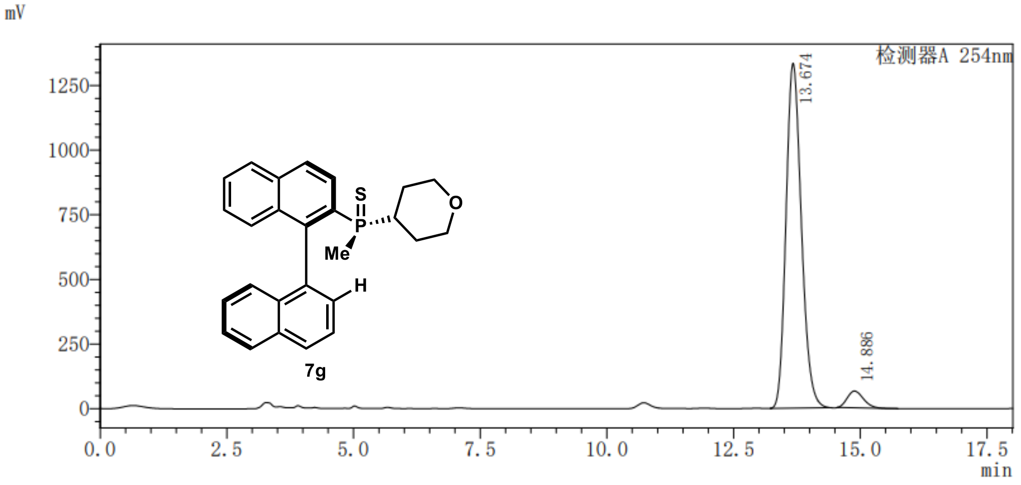

〈峰表〉

检测器A 254nm

峰号	保留时间	面积	高度	浓度	浓度单位	标记	化合物名
1	13.674	28143688	1333470	95.129		M	
2	14.886	1441188	64820	4.871		M	
总计		29584877	1398289				

C:\LabSolutions\Sample\PLZ-9-3C-IE30%2.lcd

Supplementary Fig. 475. HPLC of **7g.**

SHIMADZU LabSolutions 分析报告

<样品信息>

样品名	: ZZY		
样品ID	: PLZ-220915		
数据文件名	: SQL-5-69-4-AS5%4. lcd		
方法文件名	: 1. lcm		
批处理文件名	:		
样品瓶号	: 1-1	样品类型	: 未知
进样体积	: 1 uL		
分析日期	: 2022/10/13 10:29:31	分析者	: System Administrator
处理日期	: 2022/10/13 10:48:02	处理者	: System Administrator

<色谱图>

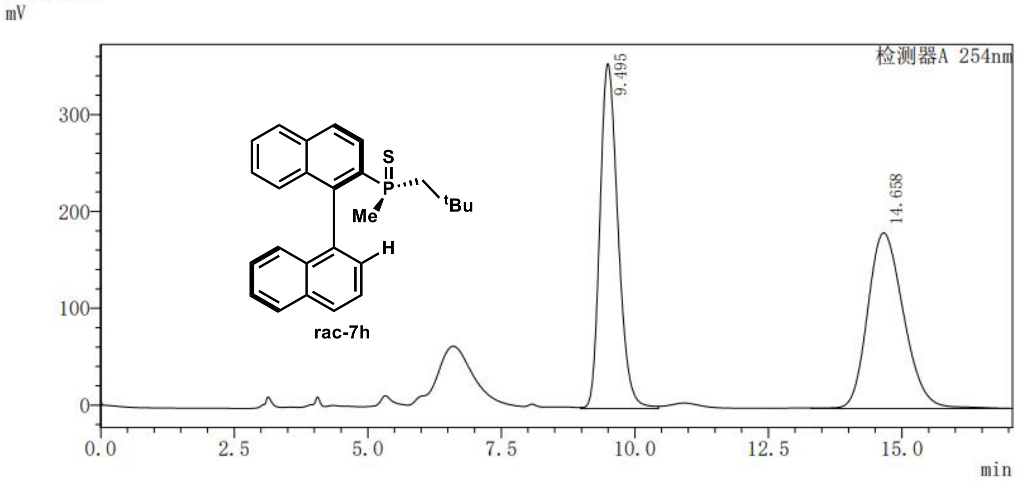

<峰表>

检测器A 254nm

峰号	保留时间	面积	高度	浓度	浓度单位	标记	化合物名
1	9. 495	8367151	355899	49. 694			
2	14. 658	8470334	181223	50. 306			
总计		16837485	537123				

C:\LabSolutions\Sample\SQL-5-69-4-AS5%4.lcd

Supplementary Fig. 476. HPLC of **7h-rac.**

SHIMADZU LabSolutions 分析报告

〈样品信息〉

样品名	: ZZY			
样品ID	: PLZ-220915			
数据文件名	: PLZ-9-4D-AS5%15.lcd			
方法文件名	: 1.1cm			
批处理文件名	:			
样品瓶号	: 1-1	样品类型	: 未知	
进样体积	: 1 uL			
分析日期	: 2022/10/22 10:32:22	分析者	: System Administrator	
处理日期	: 2022/10/22 10:49:40	处理者	: System Administrator	

〈色谱图〉

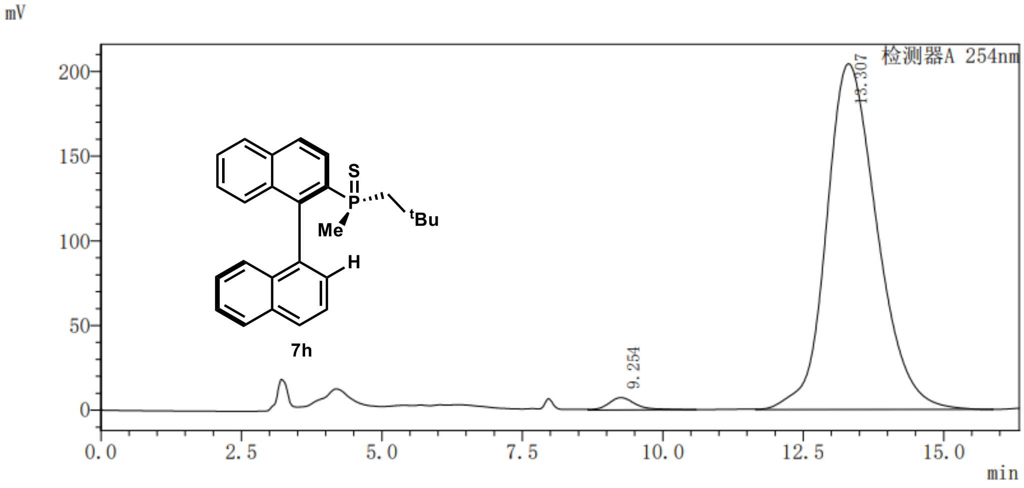

〈峰表〉

检测器A 254nm

峰号	保留时间	面积	高度	浓度	浓度单位	标记	化合物名
1	9.254	241271	7259	1.851			
2	13.307	12792458	204174	98.149			
总计		13033729	211433				

C:\LabSolutions\Sample\PLZ-9-4D-AS5%15.lcd

Supplementary Fig. 477. HPLC of **7h.**

SHIMADZU LabSolutions 分析报告

<样品信息>

样品名	: ZZY		
样品ID	: PLZ-220915		
数据文件名	: PLZ-6-85B-IC10%27.lcd		
方法文件名	: 1.1cm		
批处理文件名	:		
样品瓶号	: 1-1	样品类型	: 未知
进样体积	: 1 uL		
分析日期	: 2022/10/21 16:21:43	分析者	: System Administrator
处理日期	: 2022/10/21 16:57:50	处理者	: System Administrator

<色谱图>

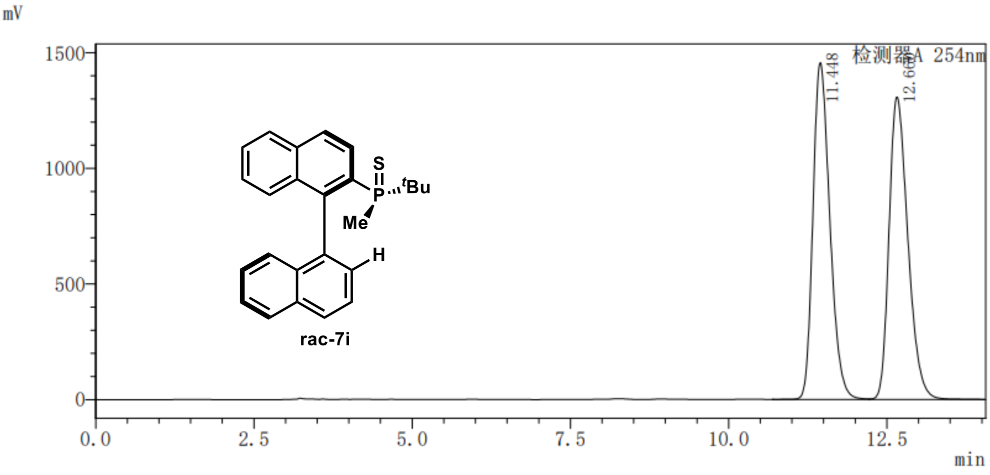

<峰表>

检测器A 254nm

峰号	保留时间	面积	高度	浓度	浓度单位	标记	化合物名
1	11.448	27109519	1455886	50.053			
2	12.660	27051983	1307637	49.947		V	
总计		54161503	2763523				

C:\LabSolutions\Sample\PLZ-6-85B-IC10%27.lcd

Supplementary Fig. 478. HPLC of **7i-rac.**

SHIMADZU LabSolutions 分析报告

〈样品信息〉

样品名	: ZZY		
样品ID	: PLZ-220915		
数据文件名	: PLZ-8-198-IC10%28.lcd		
方法文件名	: 1.lcm		
批处理文件名	:		
样品瓶号	: 1-1	样品类型	: 未知
进样体积	: 1 uL		
分析日期	: 2022/10/21 16:38:31	分析者	: System Administrator
处理日期	: 2022/10/21 16:56:59	处理者	: System Administrator

〈色谱图〉

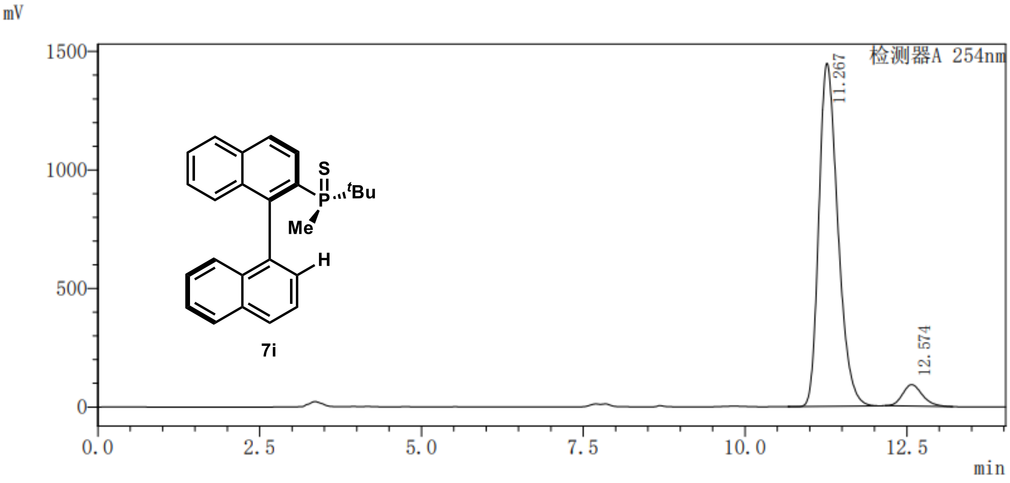

〈峰表〉

检测器A 254nm

峰号	保留时间	面积	高度	浓度	浓度单位	标记	化合物名
1	11.267	29587962	1447077	93.892		M	
2	12.574	1924864	90317	6.108		M	
总计		31512825	1537394				

C:\LabSolutions\Sample\PLZ-8-198-IC10%28.lcd

Supplementary Fig. 479. HPLC of **7i.**

SHIMADZU LabSolutions 分析报告

〈样品信息〉

样品名	: PLZ		
样品ID	: PLZ-220915		
数据文件名	: SQL-5-69-2-IA10%6.lcd		
方法文件名	: AD30.lcm		
批处理文件名	:		
样品瓶号	: 1-1	样品类型	: 未知
进样体积	: 1 uL		
分析日期	: 2022/9/22 10:06:00	分析者	: System Administrator
处理日期	: 2022/9/22 10:17:28	处理者	: System Administrator

〈色谱图〉

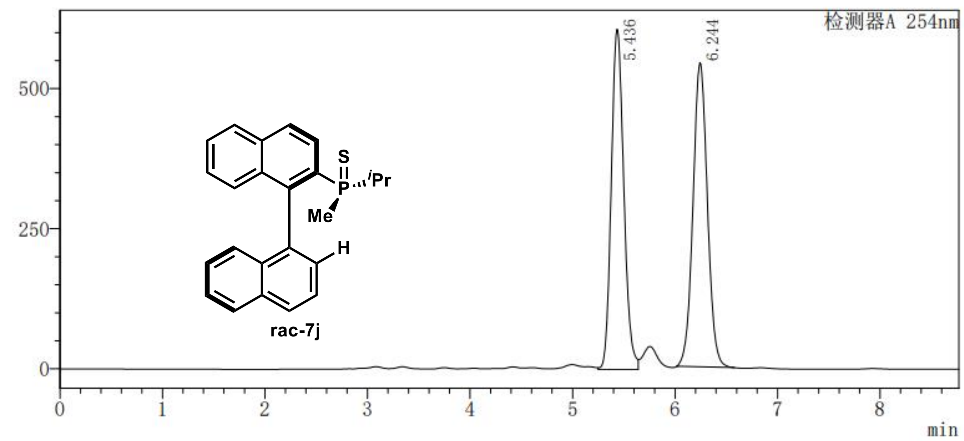

〈峰表〉

检测器A 254nm

峰号	保留时间	面积	高度	浓度	浓度单位	标记	化合物名
1	5.436	5148421	606873	49.315			
2	6.244	5291426	542296	50.685		M	
总计		10439847	1149170				

C:\LabSolutions\Sample\SQL-5-69-2-IA10%6.lcd

Supplementary Fig. 480. HPLC of **7j-rac.**

SHIMADZU LabSolutions 分析报告

<样品信息>

样品名	: ZZY		
样品ID	: PLZ-220915		
数据文件名	: PLZ-9-82A-IA10%5.lcd		
方法文件名	: 1.lcm		
批处理文件名	:		
样品瓶号	: 1-1	样品类型	: 未知
进样体积	: 1 uL		
分析日期	: 2022/10/25 20:14:39	分析者	: System Administrator
处理日期	: 2022/10/25 20:23:18	处理者	: System Administrator

<色谱图>

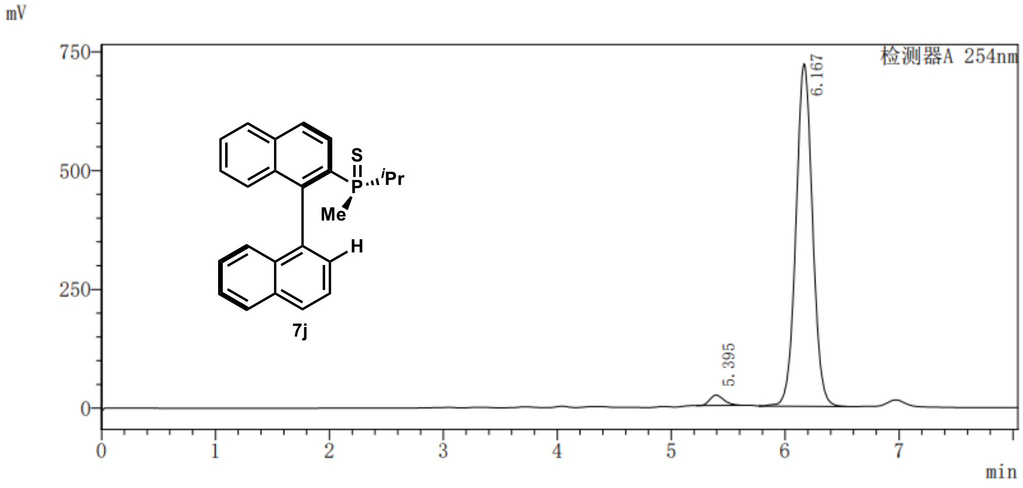

<峰表>

检测器A 254nm

峰号	保留时间	面积	高度	浓度	浓度单位	标记	化合物名
1	5.395	191541	21904	2.558		M	
2	6.167	7296049	720631	97.442		M	
总计		7487589	742535				

C:\LabSolutions\Sample\PLZ-9-82A-IA10%5.lcd

Supplementary Fig. 481. HPLC of **7j.**

SHIMADZU LabSolutions 分析报告

<样品信息>

样品名	: ZZY		
样品ID	: PLZ-220915		
数据文件名	: SQL-5-69-3-IA2.5%2.lcd		
方法文件名	: 1.lcm		
批处理文件名	:		
样品瓶号	: 1-1	样品类型	: 未知
进样体积	: 1 uL		
分析日期	: 2022/10/17 14:15:15	分析者	: System Administrator
处理日期	: 2022/10/17 14:34:50	处理者	: System Administrator

<色谱图>

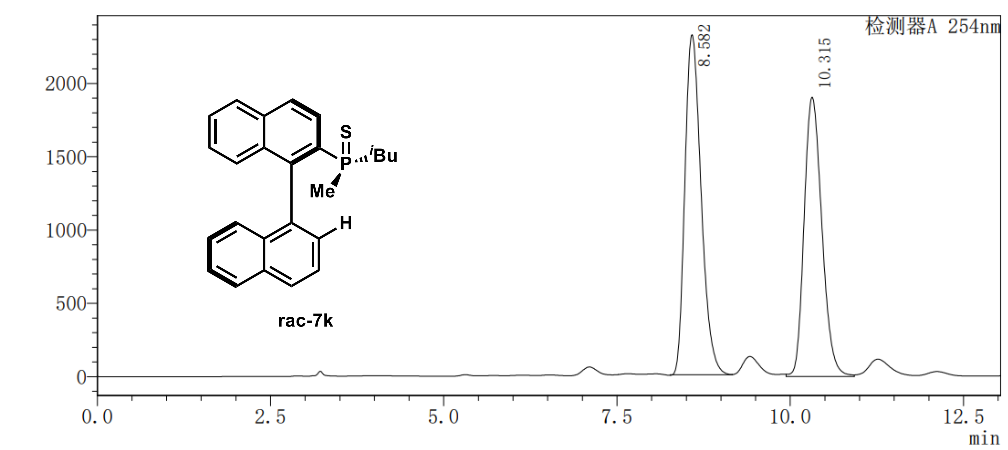

<峰表>

检测器A 254nm

峰号	保留时间	面积	高度	浓度	浓度单位	标记	化合物名
1	8.582	37903413	2319925	52.718		M	
2	10.315	33995059	1905818	47.282		M	
总计		71898472	4225743				

C:\LabSolutions\Sample\SQL-5-69-3-IA2.5%2.lcd

Supplementary Fig. 482. HPLC of **7k-rac.**

SHIMADZU LabSolutions 分析报告

〈样品信息〉

样品名	: PLZ			
样品ID	: PLZ-220915			
数据文件名	: PLZ-9-4C-IA2.5%17.lcd			
方法文件名	: 1.lcm			
批处理文件名	:			
样品瓶号	: 1-1	样品类型	: 未知	
进样体积	: 1 uL			
分析日期	: 2022/9/24 15:10:20	分析者	: System Administrator	
处理日期	: 2022/9/24 15:24:52	处理者	: System Administrator	

〈色谱图〉

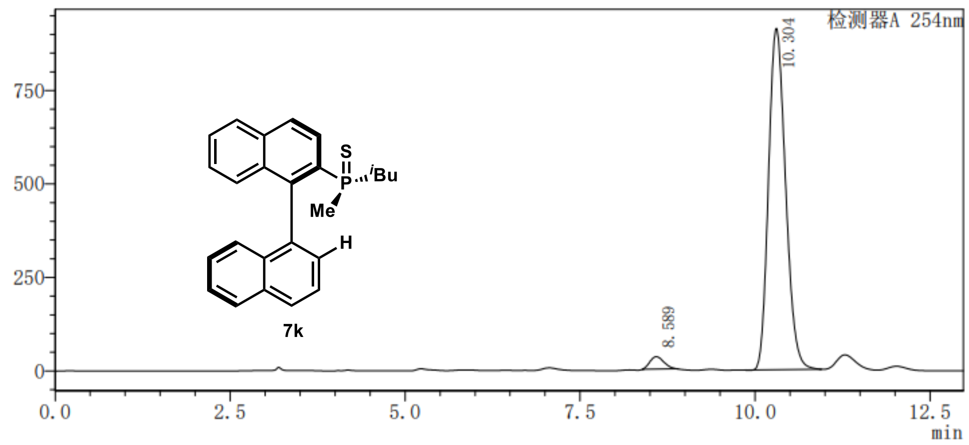

〈峰表〉

检测器A 254nm

峰号	保留时间	面积	高度	浓度	浓度单位	标记	化合物名
1	8.589	466213	33394	2.923		M	
2	10.304	15485908	912642	97.077		M	
总计		15952121	946036				

C:\LabSolutions\Sample\PLZ-9-4C-IA2.5%17.lcd

Supplementary Fig. 483. HPLC of **7k.**

SHIMADZU LabSolutions 分析报告

<样品信息>

样品名	: ZZY		
样品ID	: PLZ-220915		
数据文件名	: SQL-5-70-1-IA2.5%4.lcd		
方法文件名	: 1.lcm		
批处理文件名	:		
样品瓶号	: 1-1	样品类型	: 未知
进样体积	: 1 uL		
分析日期	: 2022/10/17 14:46:45	分析者	: System Administrator
处理日期	: 2022/10/17 19:05:33	处理者	: System Administrator

<色谱图>

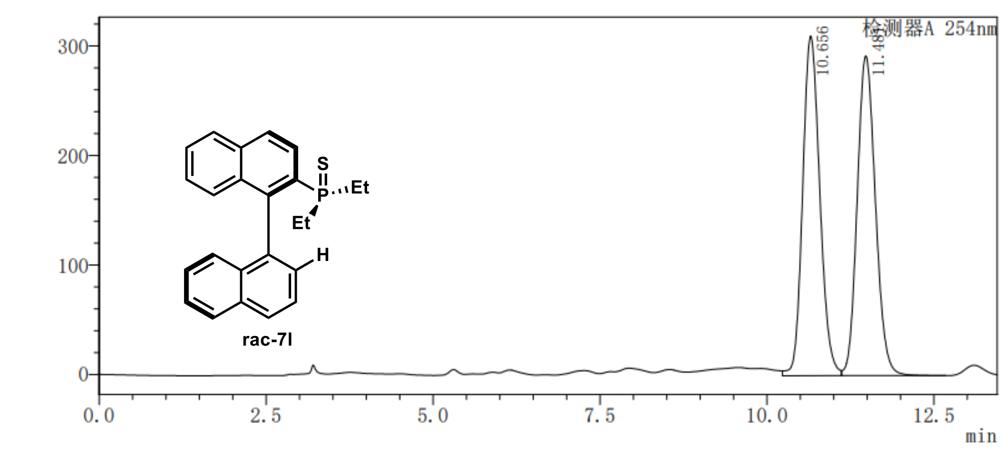

<峰表>

检测器A 254nm

峰号	保留时间	面积	高度	浓度	浓度单位	标记	化合物名
1	10.656	5540263	310057	49.954			
2	11.481	5550427	291966	50.046		V	
总计		11090689	602023				

C:\LabSolutions\Sample\SQL-5-70-1-IA2.5%4.lcd

Supplementary Fig. 484. HPLC of **7l-rac.**

SHIMADZU LabSolutions 分析报告

<样品信息>

样品名	: ZZY				
样品ID	: PLZ-220915				
数据文件名	: PLZ-9-37B-IA2.5%3.lcd				
方法文件名	: 1.lcm				
批处理文件名	:				
样品瓶号	: 1-1	样品类型	: 未知		
进样体积	: 1 uL				
分析日期	: 2022/10/17 14:31:50	分析者	: System Administrator		
处理日期	: 2022/10/17 14:48:02	处理者	: System Administrator		

<色谱图>

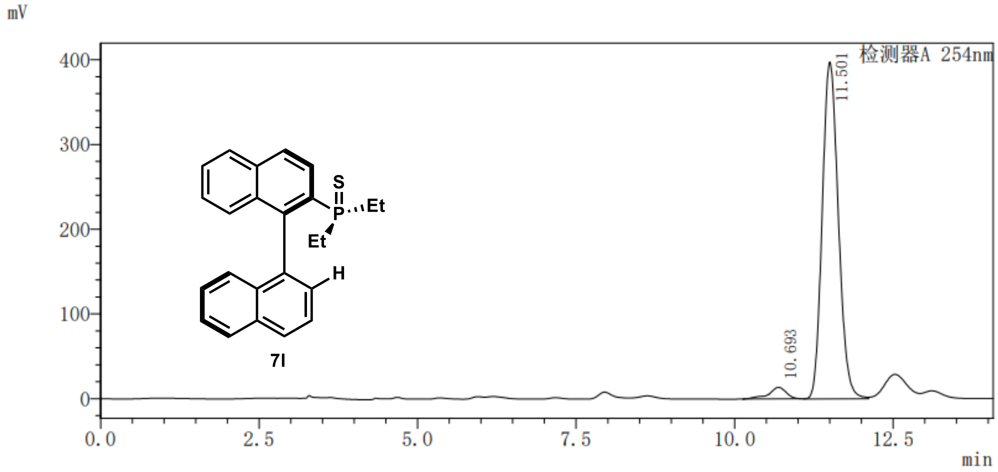

<峰表>

检测器A 254nm

峰号	保留时间	面积	高度	浓度	浓度单位	标记	化合物名
1	10.693	256924	13481	3.524		M	
2	11.501	7033301	397700	96.476			
总计		7290225	411180				

C:\LabSolutions\Sample\PLZ-9-37B-IA2.5%3.lcd

Supplementary Fig. 485. HPLC of **7l.**

SHIMADZU LabSolutions 分析报告

〈样品信息〉

样品名	: ZZY		
样品ID	: PLZ-220915		
数据文件名	: PLZ-9-58C-AS30%16.lcd		
方法文件名	: 1.lcm		
批处理文件名	:		
样品瓶号	: 1-1	样品类型	: 未知
进样体积	: 1 uL		
分析日期	: 2022/10/14 17:22:49	分析者	: System Administrator
处理日期	: 2022/10/14 17:38:10	处理者	: System Administrator

〈色谱图〉

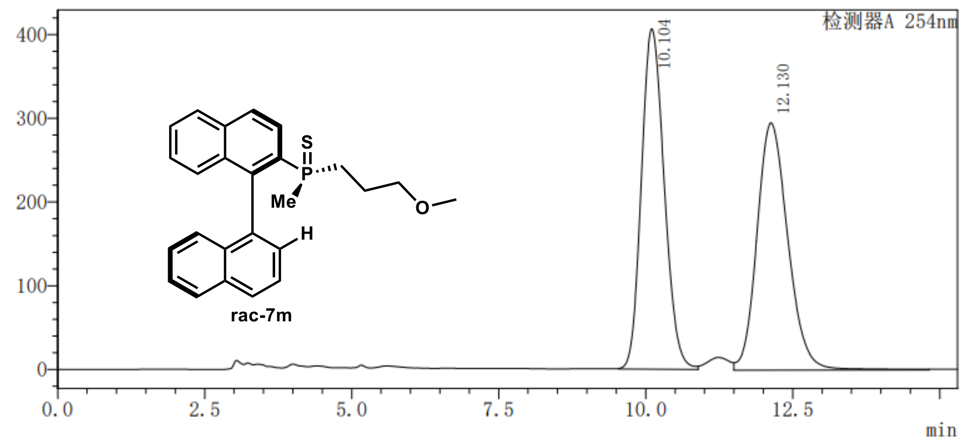

〈峰表〉

检测器A 254nm

峰号	保留时间	面积	高度	浓度	浓度单位	标记	化合物名
1	10.104	10981586	406527	50.324		M	
2	12.130	10840179	295477	49.676		M	
总计		21821765	702005				

C:\LabSolutions\Sample\PLZ-9-58C-AS30%16.lcd

Supplementary Fig. 486. HPLC of **7m-rac.**

SHIMADZU LabSolutions 分析报告

<样品信息>

样品名	: ZZY		
样品ID	: PLZ-220915		
数据文件名	: PLZ-9-5F-AS30%14.lcd		
方法文件名	: 1.1cm		
批处理文件名	:		
样品瓶号	: 1-1	样品类型	: 未知
进样体积	: 1 uL		
分析日期	: 2022/10/14 16:51:35	分析者	: System Administrator
处理日期	: 2022/10/14 17:07:57	处理者	: System Administrator

<色谱图>

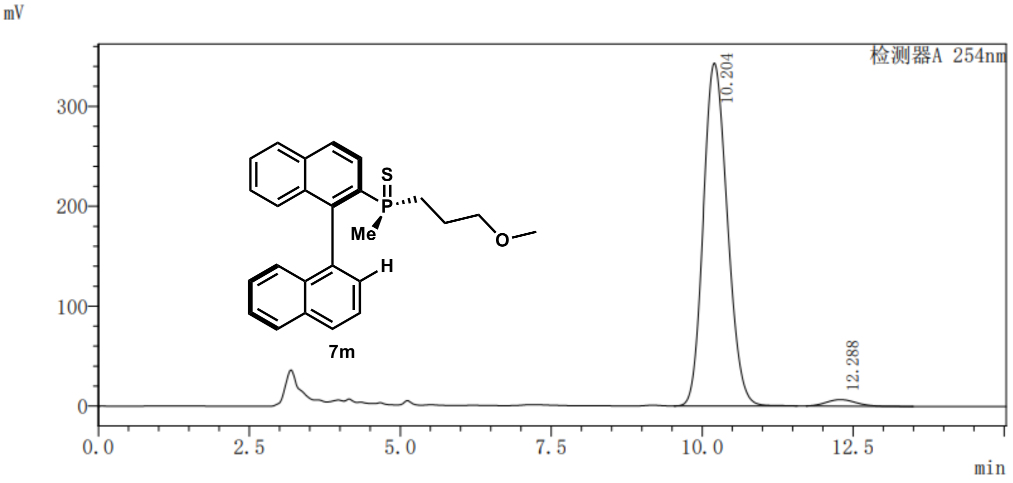

<峰表>

检测器A 254nm

峰号	保留时间	面积	高度	浓度	浓度单位	标记	化合物名
1	10.204	9532136	343015	97.764		M	
2	12.288	218049	6482	2.236		M	
总计		9750185	349496				

C:\LabSolutions\Sample\PLZ-9-5F-AS30%14.lcd

Supplementary Fig. 487. HPLC of **7m.**

SHIMADZU LabSolutions 分析报告

<样品信息>

样品名	: ZZY		
样品ID	: PLZ-220915		
数据文件名	: PLZ-9-64-IE20%25.lcd		
方法文件名	: 1.1cm		
批处理文件名	:		
样品瓶号	: 1-1	样品类型	: 未知
进样体积	: 1 uL		
分析日期	: 2022/10/21 15:36:48	分析者	: System Administrator
处理日期	: 2022/10/21 15:47:28	处理者	: System Administrator

<色谱图>

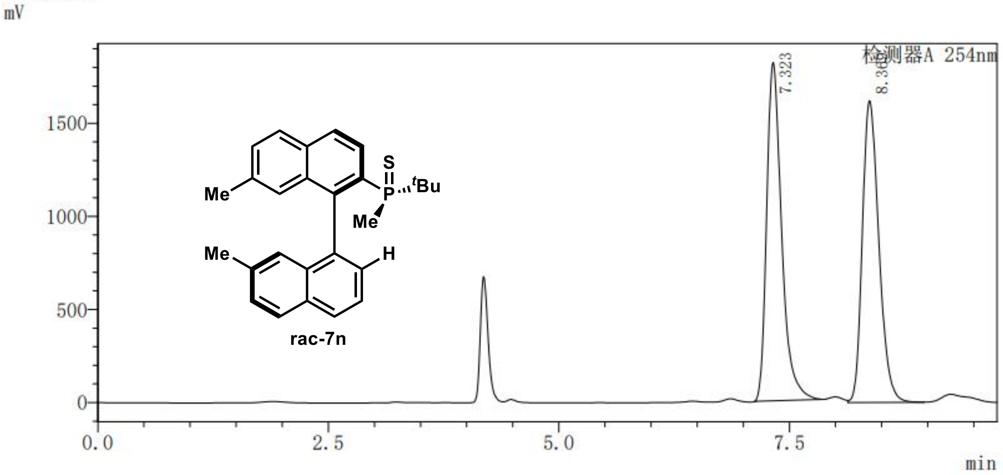

<峰表>

检测器A 254nm

峰号	保留时间	面积	高度	浓度	浓度单位	标记	化合物名
1	7.323	20477728	1816256	50.320		M	
2	8.369	20217583	1620550	49.680			
总计		40695311	3436806				

C:\LabSolutions\Sample\PLZ-9-64-IE20%25.lcd

Supplementary Fig. 488. HPLC of **7n-rac.**

SHIMADZU LabSolutions 分析报告

<样品信息>

样品名	: ZZY		
样品ID	: PLZ-220915		
数据文件名	: PLZ-9-72E-IE20%24.lcd		
方法文件名	: 1.lcm		
批处理文件名	:		
样品瓶号	: 1-1	样品类型	: 未知
进样体积	: 1 uL		
分析日期	: 2022/10/21 15:25:26	分析者	: System Administrator
处理日期	: 2022/10/21 15:37:10	处理者	: System Administrator

<色谱图>

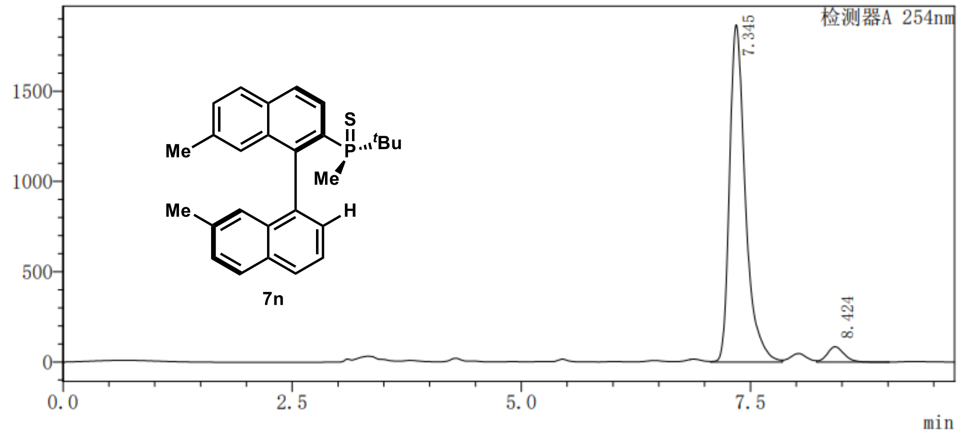

<峰表>

检测器A 254nm

峰号	保留时间	面积	高度	浓度	浓度单位	标记	化合物名
1	7.345	22473360	1867440	95.375			
2	8.424	1089840	84752	4.625			
总计		23563200	1952192				

C:\LabSolutions\Sample\PLZ-9-72E-IE20%24.lcd

Supplementary Fig. 489. HPLC of **7n.**

SHIMADZU LabSolutions 分析报告

<样品信息>

样品名	: ZZY		
样品ID	: PLZ-220915		
数据文件名	: PLZ-9-58B-IC10%9.lcd		
方法文件名	: 1.lcm		
批处理文件名	:		
样品瓶号	: 1-1	样品类型	: 未知
进样体积	: 1 uL		
分析日期	: 2022/10/23 19:00:09	分析者	: System Administrator
处理日期	: 2022/10/23 19:14:02	处理者	: System Administrator

<色谱图>

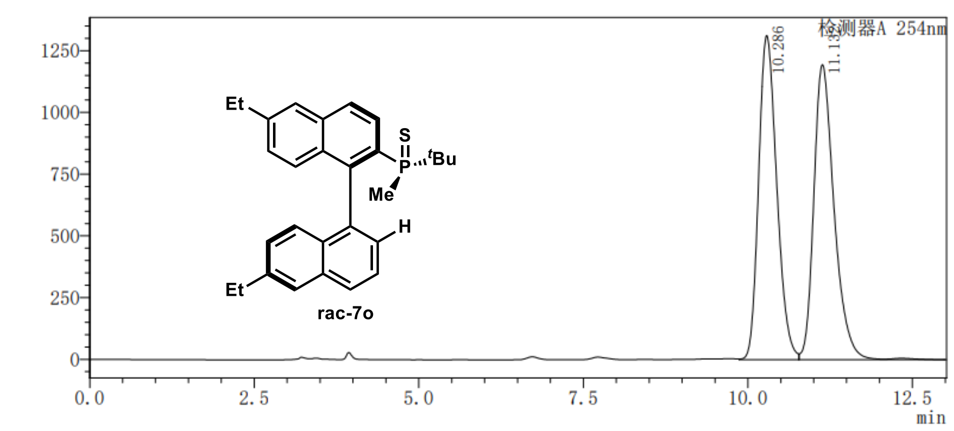

<峰表>

检测器A 254nm

峰号	保留时间	面积	高度	浓度	浓度单位	标记	化合物名
1	10.286	25203038	1312837	49.497			
2	11.132	25714982	1195454	50.503		V	
总计		50918019	2508291				

C:\LabSolutions\Sample\PLZ-9-58B-IC10%9.lcd

Supplementary Fig. 490. HPLC of **7o-rac.**

SHIMADZU LabSolutions 分析报告

<样品信息>

样品名	: ZZY		
样品ID	: PLZ-220915		
数据文件名	: PLZ-9-72D-IC10%11.lcd		
方法文件名	: 1.lcm		
批处理文件名	:		
样品瓶号	: 1-1	样品类型	: 未知
进样体积	: 1 uL		
分析日期	: 2022/10/23 19:27:41	分析者	: System Administrator
处理日期	: 2022/10/23 19:43:30	处理者	: System Administrator

<色谱图>

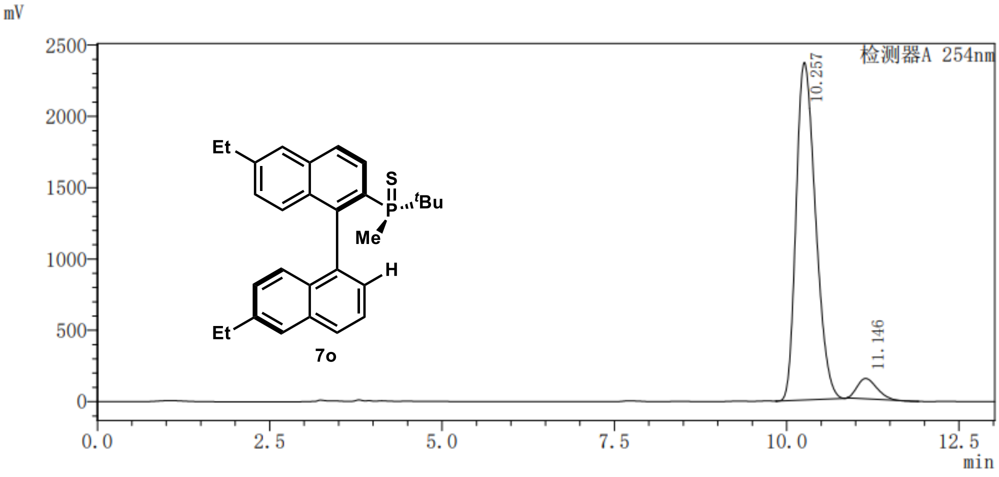

<峰表>

检测器A 254nm

峰号	保留时间	面积	高度	浓度	浓度单位	标记	化合物名
1	10.257	46399987	2366128	94.257		M	
2	11.146	2827172	142909	5.743		M	
总计		49227159	2509037				

C:\LabSolutions\Sample\PLZ-9-72D-IC10%11.lcd

Supplementary Fig. 491. HPLC of **7o.**

SHIMADZU LabSolutions 分析报告

〈样品信息〉

样品名 : ZZY
样品ID : PLZ-220915
数据文件名 : SQL-5-71-4-IE10%13. lcd
方法文件名 : 1. lcm
批处理文件名 :
样品瓶号 : 1-1 样品类型 : 未知
进样体积 : 1 uL
分析日期 : 2022/10/24 16:34:24 分析者 : System Administrator
处理日期 : 2022/10/24 16:55:19 处理者 : System Administrator

〈色谱图〉

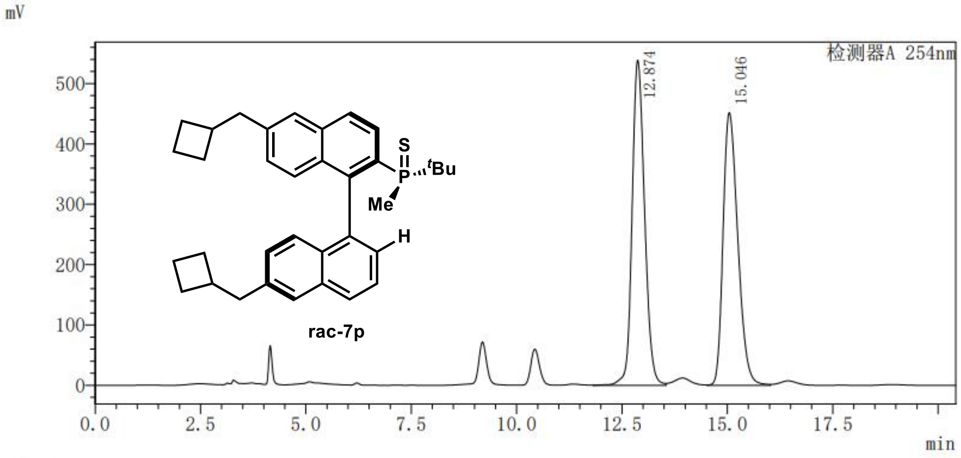

〈峰表〉

检测器A 254nm

峰号	保留时间	面积	高度	浓度	浓度单位	标记	化合物名
1	12. 874	11110665	538627	50. 282			
2	15. 046	10985969	451901	49. 718			
总计		22096633	990528				

C:\LabSolutions\Sample\SQL-5-71-4-IE10%13.lcd

Supplementary Fig. 492. HPLC of **7p-rac.**

SHIMADZU LabSolutions 分析报告

<样品信息>

样品名	: ZZY			
样品ID	: PLZ-220915			
数据文件名	: PLZ-9-77-IE10%12.lcd			
方法文件名	: 1.lcm			
批处理文件名	:			
样品瓶号	: 1-1		样品类型	: 未知
进样体积	: 1 uL			
分析日期	: 2022/10/24 16:14:00		分析者	: System Administrator
处理日期	: 2022/10/24 16:56:17		处理者	: System Administrator

<色谱图>

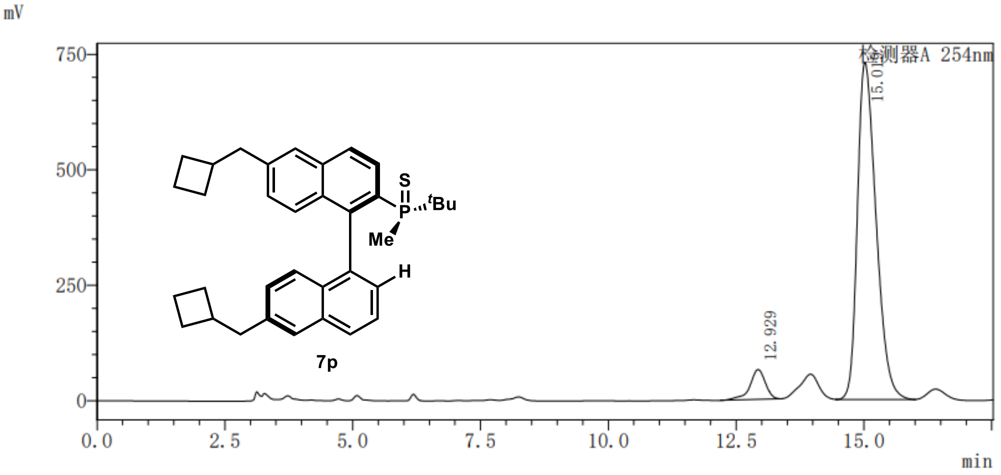

<峰表>

检测器A 254nm

峰号	保留时间	面积	高度	浓度	浓度单位	标记	化合物名
1	12.929	1358533	64548	6.830		M	
2	15.019	18531299	730311	93.170		M	
总计		19889832	794860				

C:\LabSolutions\Sample\PLZ-9-77-IE10%12.lcd

Supplementary Fig. 493. HPLC of **7p.**

SHIMADZU LabSolutions 分析报告

<样品信息>

样品名	: ZZY		
样品ID	: PLZ-220915		
数据文件名	: SQL-5-71-2-IE5%13.lcd		
方法文件名	: 1.lcm		
批处理文件名	:		
样品瓶号	: 1-1	样品类型	: 未知
进样体积	: 1 uL		
分析日期	: 2022/10/30 17:24:16	分析者	: System Administrator
处理日期	: 2022/10/30 17:59:32	处理者	: System Administrator

<色谱图>

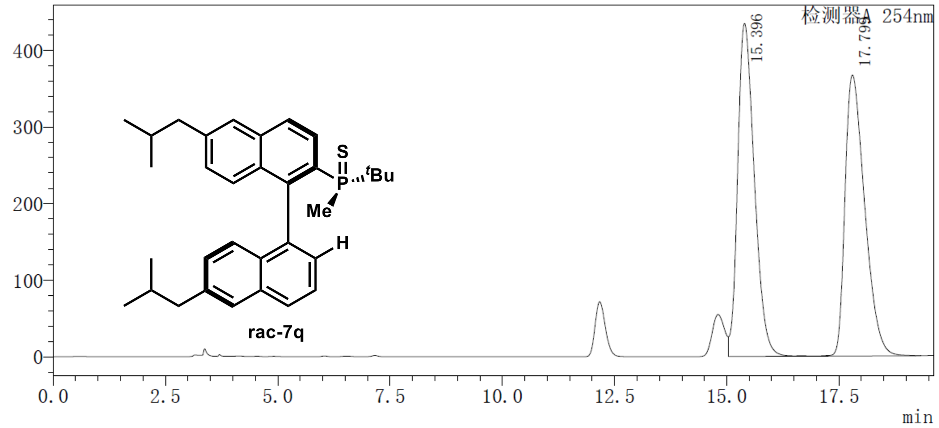

<峰表>

检测器A 254nm

峰号	保留时间	面积	高度	浓度	浓度单位	标记	化合物名
1	15.396	11247599	434751	50.137			
2	17.799	11185946	367001	49.863		V	
总计		22433545	801752				

C:\LabSolutions\Sample\SQL-5-71-2-IE5%13.lcd

Supplementary Fig. 494. HPLC of **7q-rac.**

分析报告

〈样品信息〉

样品名	: ZZY			
样品ID	: PLZ-220915			
数据文件名	: PLZ-9-60B-IE5%12.lcd			
方法文件名	: 1.1cm			
批处理文件名	:			
样品瓶号	: 1-1	样品类型	: 未知	
进样体积	: 1 uL			
分析日期	: 2022/10/30 16:59:18	分析者	: System Administrator	
处理日期	: 2022/10/30 17:19:36	处理者	: System Administrator	

〈色谱图〉

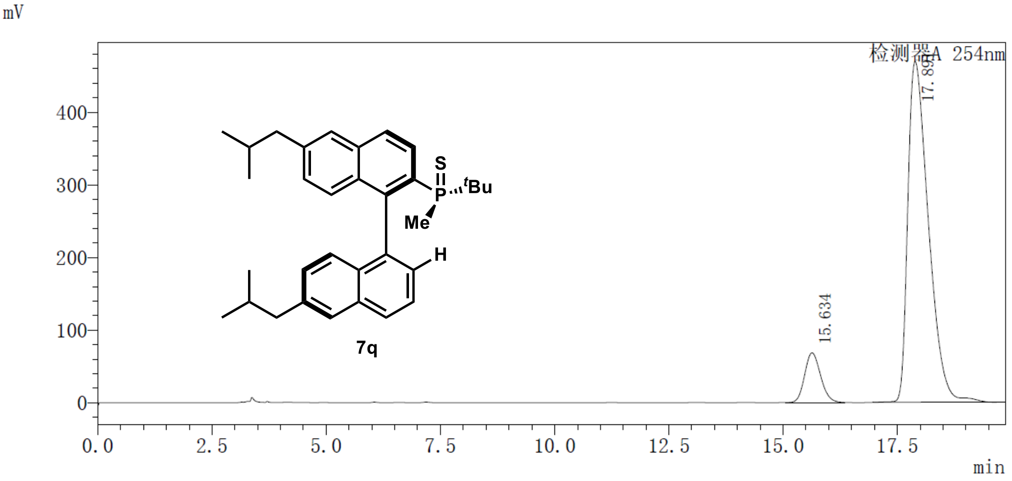

〈峰表〉

检测器A 254nm

峰号	保留时间	面积	高度	浓度	浓度单位	标记	化合物名
1	15.634	1652961	68285	10.094		M	
2	17.891	14722479	469062	89.906		M	
总计		16375440	537347				

C:\LabSolutions\Sample\PLZ-9-60B-IE5%12.lcd

Supplementary Fig. 495. HPLC of **7q.**

SHIMADZU LabSolutions 分析报告

<样品信息>

样品名 : PLZ
样品ID : PLZ-220915
数据文件名 : PLZ-9-142B-IA2.5%7.lcd
方法文件名 : 1.lcm
批处理文件名 :
样品瓶号 : 1-1 样品类型 : 未知
进样体积 : 1 uL
分析日期 : 2022/11/24 16:10:57 分析者 : System Administrator
处理日期 : 2022/11/27 17:22:52 处理者 : System Administrator

<色谱图>

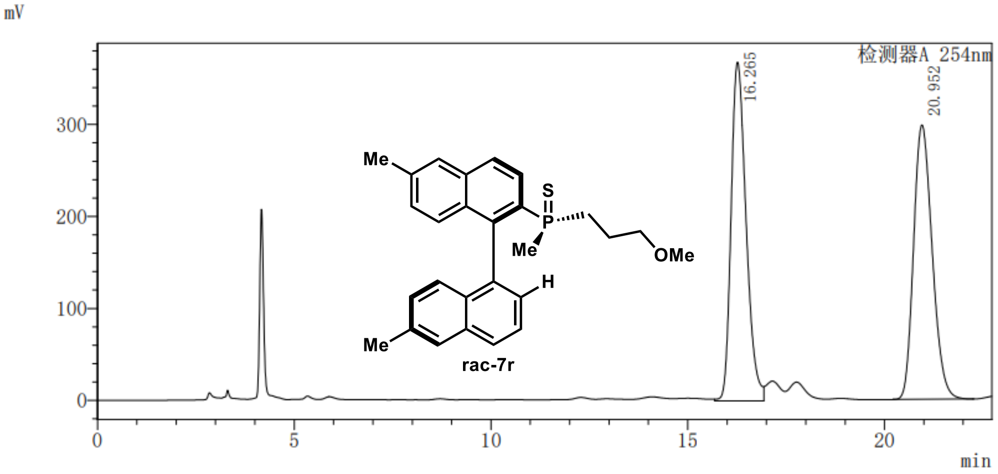

<峰表>

检测器A 254nm

峰号	保留时间	面积	高度	浓度	浓度单位	标记	化合物名
1	16.265	9778038	368292	49.899		M	
2	20.952	9817693	298353	50.101		M	
总计		19595731	666646				

C:\LabSolutions\Sample\PLZ-9-142B-IA2.5%7.lcd

Supplementary Fig. 496. HPLC of **7r-rac.**

SHIMADZU LabSolutions 分析报告

<样品信息>

样品名	:	PLZ			
样品ID	:	PLZ-220915			
数据文件名	:	PLZ-9-142A-IA2.5%8.lcd			
方法文件名	:	1.lcm			
批处理文件名	:				
样品瓶号	:	1-1	样品类型	:	未知
进样体积	:	1 uL			
分析日期	:	2022/11/24 16:36:46	分析者	:	System Administrator
处理日期	:	2022/11/24 17:00:36	处理者	:	System Administrator

<色谱图>

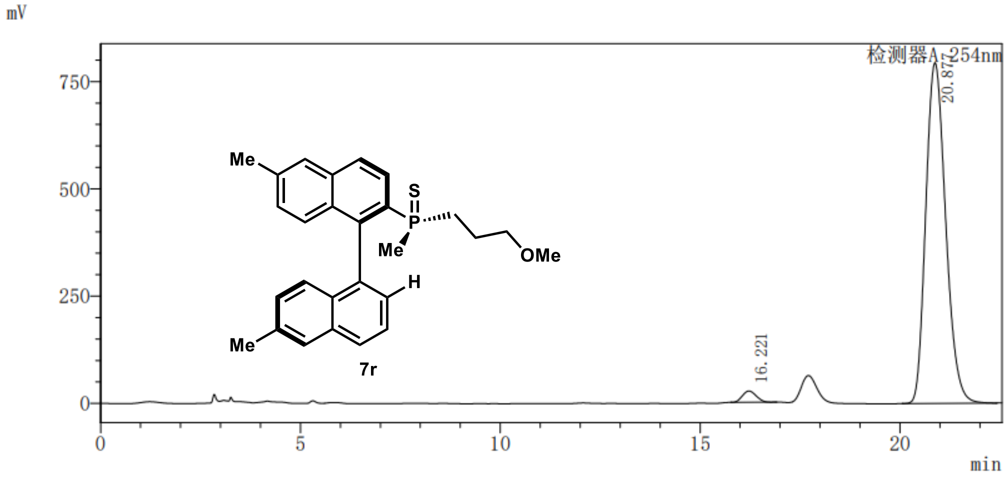

<峰表>

检测器A 254nm

峰号	保留时间	面积	高度	浓度	浓度单位	标记	化合物名
1	16.221	631184	26303	2.248		M	
2	20.877	27446072	793823	97.752		M	
总计		28077256	820126				

Supplementary Fig. 497. HPLC of **7r.**

SHIMADZU LabSolutions 分析报告

<样品信息>

样品名	: PLZ		
样品ID	: PLZ-220915		
数据文件名	: PLZ-9-143B-IA2.5%1.lcd		
方法文件名	: 1.lcm		
批处理文件名	:		
样品瓶号	: 1-1	样品类型	: 未知
进样体积	: 1 uL		
分析日期	: 2022/11/23 9:27:50	分析者	: System Administrator
处理日期	: 2022/11/27 17:21:41	处理者	: System Administrator

<色谱图>

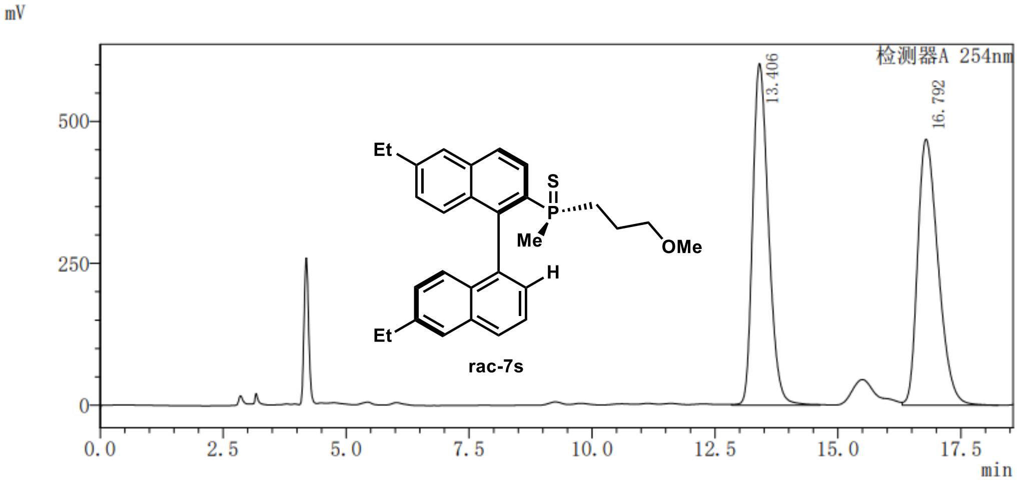

<峰表>

检测器A 254nm

峰号	保留时间	面积	高度	浓度	浓度单位	标记	化合物名
1	13.406	13440325	601802	49.693		M	
2	16.792	13606625	468582	50.307		M	
总计		27046950	1070384				

C:\LabSolutions\Sample\PLZ-9-143B-IA2.5%1.lcd

Supplementary Fig. 498. HPLC of **7s-rac.**

SHIMADZU LabSolutions 分析报告

<样品信息>

样品名	: PLZ		
样品ID	: PLZ-220915		
数据文件名	: PLZ-9-143A-IA2.5%2.lcd		
方法文件名	: 1.lcm		
批处理文件名	:		
样品瓶号	: 1-1	样品类型	: 未知
进样体积	: 1 uL		
分析日期	: 2022/11/23 9:48:57	分析者	: System Administrator
处理日期	: 2022/11/23 10:12:47	处理者	: System Administrator

<色谱图>

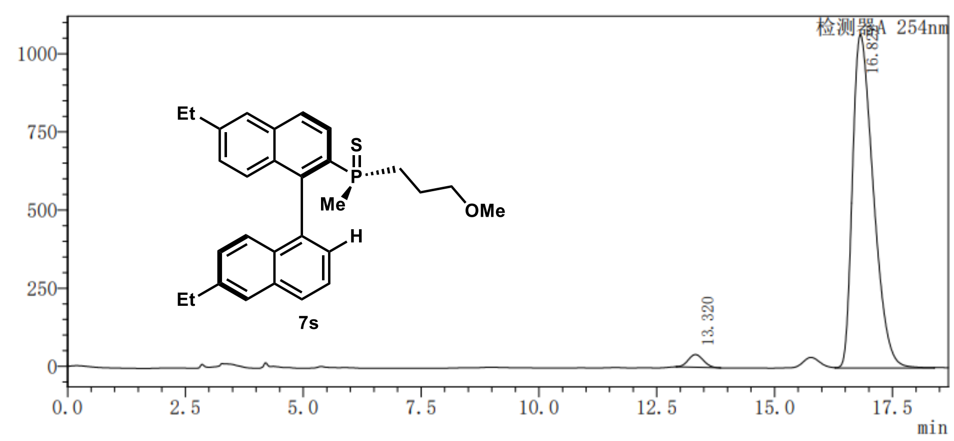

<峰表>

检测器A 254nm

峰号	保留时间	面积	高度	浓度	浓度单位	标记	化合物名
1	13.320	903819	40629	2.677		M	
2	16.822	32852392	1066366	97.323		M	
总计		33756211	1106995				

C:\LabSolutions\Sample\PLZ-9-143A-IA2.5%2.lcd

Supplementary Fig. 499. HPLC of **7s-rac.**

SHIMADZU LabSolutions 分析报告

<样品信息>

样品名	:	PLZ			
样品ID	:	PLZ-220915			
数据文件名	:	PLZ-10-182-IC10%230nm10.lcd			
方法文件名	:	322.1cm			
批处理文件名	:				
样品瓶号	:	1-1	样品类型	:	未知
进样体积	:	10 uL			
分析日期	:	2023/5/7 20:36:38	分析者	:	System Administrator
处理日期	:	2023/5/8 14:33:08	处理者	:	System Administrator

<色谱图>

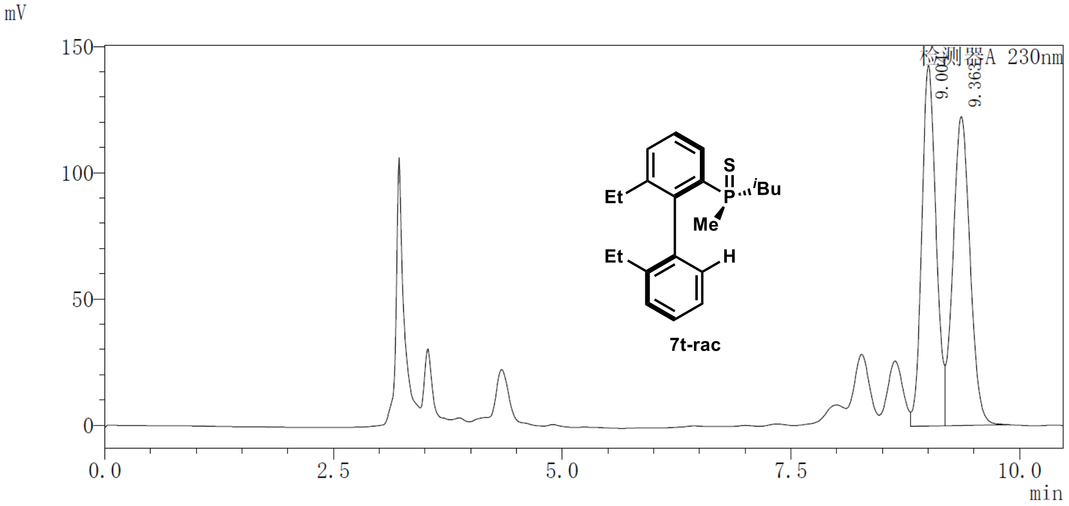

<峰表>

检测器A 230nm

峰号	保留时间	面积	高度	浓度	浓度单位	标记	化合物名
1	9.004	1555363	142786	49.390			
2	9.363	1593762	122295	50.610		V M	
总计		3149125	265081				

Supplementary Fig. 500. HPLC of **7t-rac.**

SHIMADZU LabSolutions

分析报告

<样品信息>

样品名	: PLZ		
样品ID	: PLZ-220915		
数据文件名	: PLZ-10-186-IC10%230nm9.lcd		
方法文件名	: 322.1cm		
批处理文件名	:		
样品瓶号	: 1-1	样品类型	: 未知
进样体积	: 10 uL		
分析日期	: 2023/5/7 20:14:16	分析者	: System Administrator
处理日期	: 2023/5/7 20:47:34	处理者	: System Administrator

<色谱图>

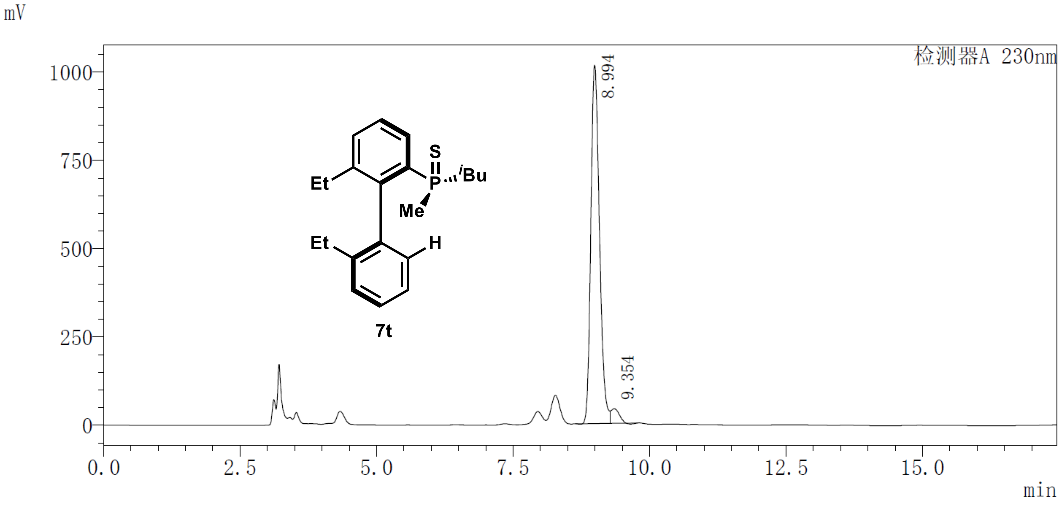

<峰表>

检测器A 230nm

峰号	保留时间	面积	高度	浓度	浓度单位	标记	化合物名
1	8.994	11185629	1015424	96.367		M	
2	9.354	421686	41242	3.633		V M	
总计		11607315	1056665				

Supplementary Fig. 501. HPLC of **7t.**

SHIMADZU LabSolutions 分析报告

<样品信息>

样品名 : PLZ
样品ID : PLZ-220915
数据文件名 : PLZ-10-171-IC20%18.lcd
方法文件名 : 322.lcm
批处理文件名 :
样品瓶号 : 1-1 样品类型 : 未知
进样体积 : 1 uL
分析日期 : 2023/4/23 16:29:14 分析者 : System Administrator
处理日期 : 2023/4/23 16:49:33 处理者 : System Administrator

<色谱图>

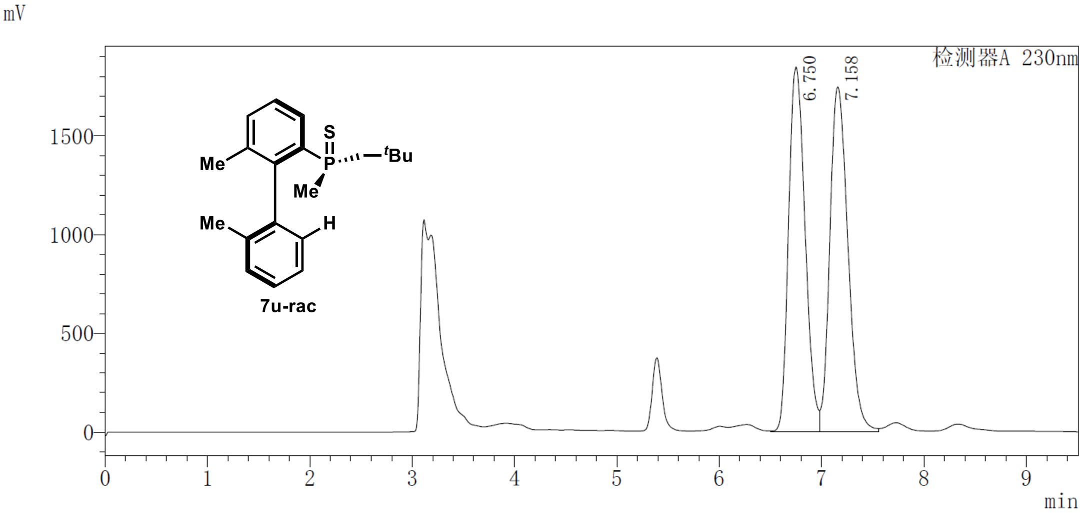

<峰表>

检测器A 230nm

峰号	保留时间	面积	高度	浓度	浓度单位	标记	化合物名
1	6.750	20777105	1848438	49.187			
2	7.158	21463598	1747050	50.813		V	
总计		42240704	3595488				

Supplementary Fig. 502. HPLC of **7u-rac.**

SHIMADZU LabSolutions 分析报告

〈样品信息〉

样品名 : PLZ
样品ID : PLZ-220915
数据文件名 : PLZ-10-164-IC20%19.lcd
方法文件名 : 322.lcm
批处理文件名 :
样品瓶号 : 1-1　　样品类型 : 未知
进样体积 : 1 uL
分析日期 : 2023/4/23 16:39:34　　分析者 : System Administrator
处理日期 : 2023/4/23 16:50:52　　处理者 : System Administrator

〈色谱图〉

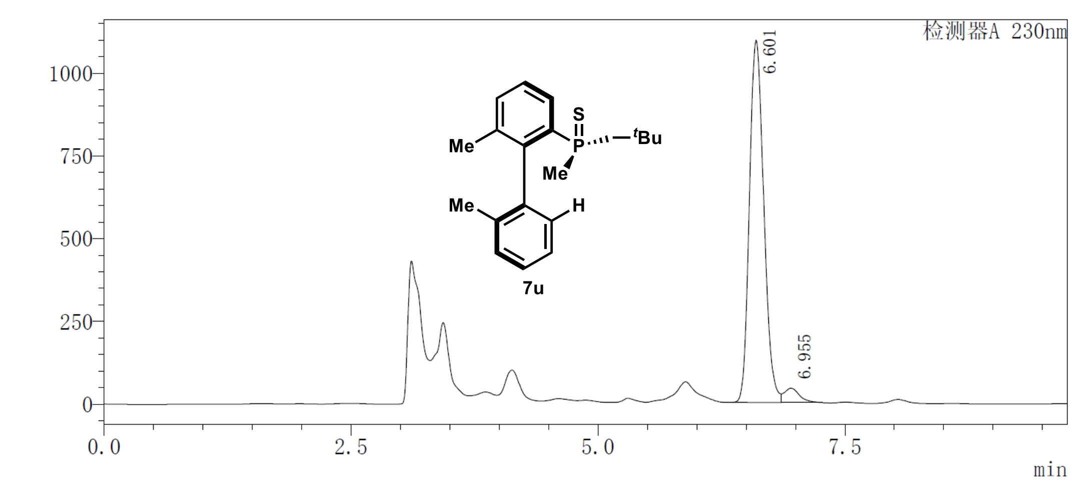

〈峰表〉

检测器A 230nm

峰号	保留时间	面积	高度	浓度	浓度单位	标记	化合物名
1	6.601	11088569	1094519	95.968		M	
2	6.955	465825	43449	4.032		V M	
总计		11554395	1137967				

Supplementary Fig. 503. HPLC of **7u.**

SHIMADZU LabSolutions 分析报告

<样品信息>

样品名	: PLZ				
样品ID	: PLZ-220915				
数据文件名	: PLZ-7-63B-ID10%2.lcd				
方法文件名	: 1.lcm				
批处理文件名	:				
样品瓶号	: 1-1	样品类型	: 未知		
进样体积	: 1 uL				
分析日期	: 2022/12/12 16:51:05	分析者	: System Administrator		
处理日期	: 2022/12/12 17:24:11	处理者	: System Administrator		

<色谱图>

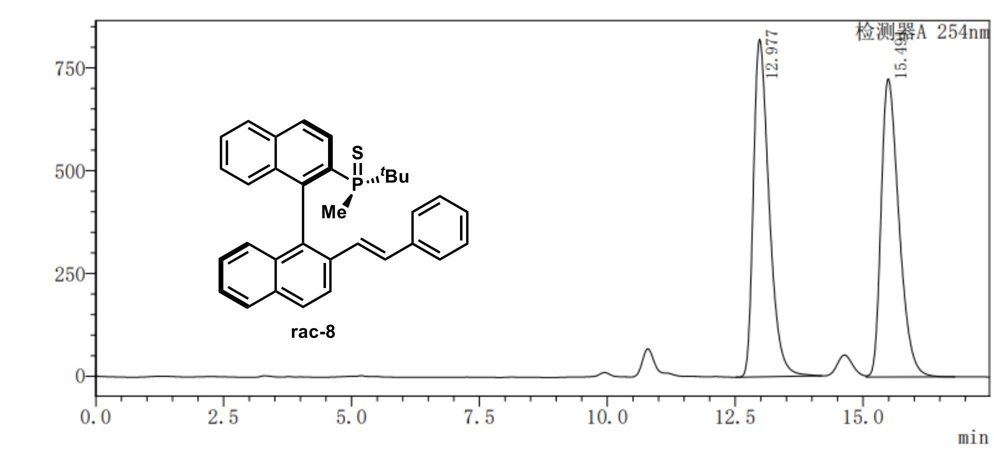

<峰表>

检测器A 254nm

峰号	保留时间	面积	高度	浓度	浓度单位	标记	化合物名
1	12.977	17568333	820373	49.940		M	
2	15.491	17610508	725049	50.060		M	
总计		35178841	1545423				

C:\LabSolutions\Sample\PLZ-7-63B-ID10%2.lcd

Supplementary Fig. 504. HPLC of **8-rac.**

SHIMADZU LabSolutions

分析报告

〈样品信息〉

样品名	: PLZ		
样品ID	: PLZ-220915		
数据文件名	: PLZ-8-13-ID10%3.lcd		
方法文件名	: 1.1cm		
批处理文件名	:		
样品瓶号	: 1-1	样品类型	: 未知
进样体积	: 1 uL		
分析日期	: 2022/12/12 17:12:59	分析者	: System Administrator
处理日期	: 2022/12/12 17:31:20	处理者	: System Administrator

〈色谱图〉

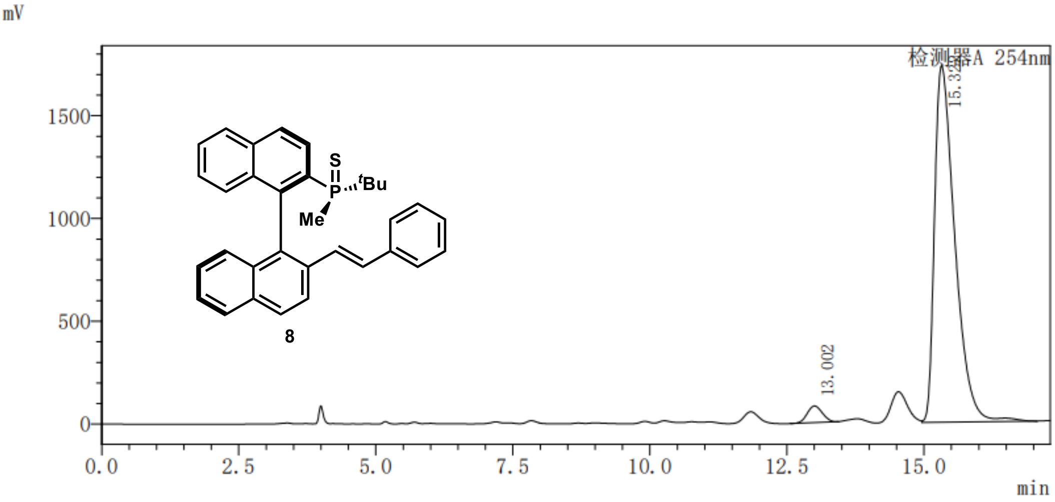

〈峰表〉

检测器A 254nm

峰号	保留时间	面积	高度	浓度	浓度单位	标记	化合物名
1	13.002	1557941	81112	3.365		M	
2	15.322	44738579	1734284	96.635			
总计		46296520	1815396				

C:\LabSolutions\Sample\PLZ-8-13-ID10%3.lcd

Supplementary Fig. 505. HPLC of **8.**

SHIMADZU LabSolutions 分析报告

<样品信息>

样品名 : PLZ
样品ID : PLZ-220915
数据文件名 : PLZ-9-185-IG10%12.lcd
方法文件名 : 1.lcm
批处理文件名 :
样品瓶号 : 1-1 样品类型 : 未知
进样体积 : 1 uL
分析日期 : 2022/12/4 22:37:35 分析者 : System Administrator
处理日期 : 2022/12/4 22:58:45 处理者 : System Administrator

<色谱图>

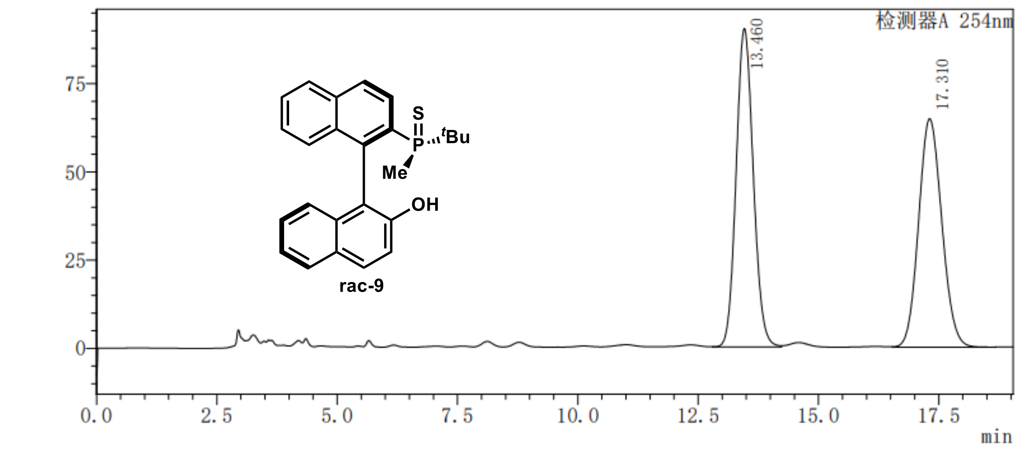

<峰表>

检测器A 254nm

峰号	保留时间	面积	高度	浓度	浓度单位	标记	化合物名
1	13.460	2238220	90181	50.785		M	
2	17.310	2169018	64719	49.215		M	
总计		4407238	154900				

C:\LabSolutions\Sample\PLZ-9-185-IG10%12.lcd

Supplementary Fig. 506. HPLC of **9-rac.**

SHIMADZU LabSolutions 分析报告

〈样品信息〉

样品名	: PLZ		
样品ID	: PLZ-220915		
数据文件名	: PLZ-9-163-IG10%13.lcd		
方法文件名	: 1.1cm		
批处理文件名	:		
样品瓶号	: 1-1	样品类型	: 未知
进样体积	: 1 uL		
分析日期	: 2022/12/4 22:57:06	分析者	: System Administrator
处理日期	: 2022/12/4 23:21:03	处理者	: System Administrator

〈色谱图〉

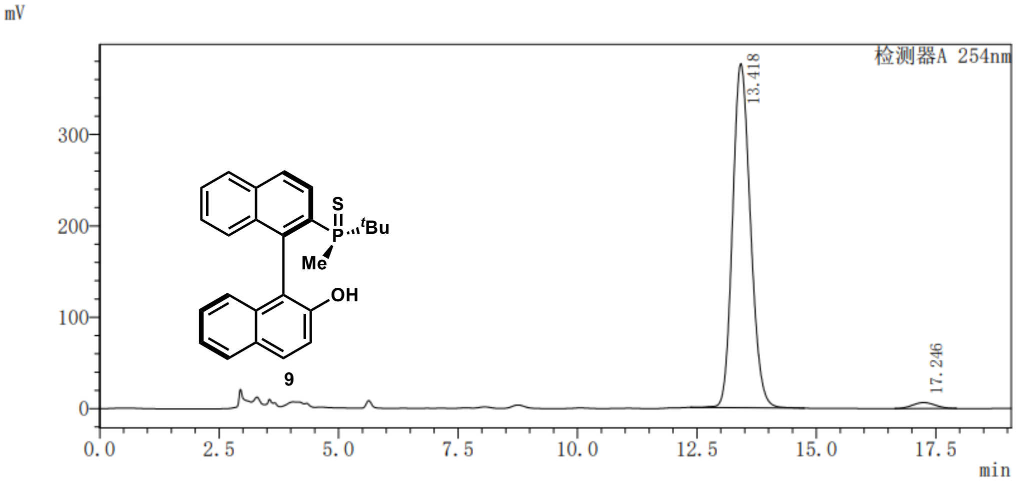

〈峰表〉

检测器A 254nm

峰号	保留时间	面积	高度	浓度	浓度单位	标记	化合物名
1	13.418	9793959	376432	97.838		M	
2	17.246	216385	6510	2.162		M	
总计		10010345	382942				

Supplementary Fig. 507. HPLC of **9.**

SHIMADZU LabSolutions 分析报告

<样品信息>

样品名	: ZZY			
样品ID	: PLZ-220915			
数据文件名	: PLZ-6-85B-IC10%27.lcd			
方法文件名	: 1.lcm			
批处理文件名	:			
样品瓶号	: 1-1	样品类型	: 未知	
进样体积	: 1 uL			
分析日期	: 2022/10/21 16:21:43	分析者	: System Administrator	
处理日期	: 2022/10/21 16:57:50	处理者	: System Administrator	

<色谱图>

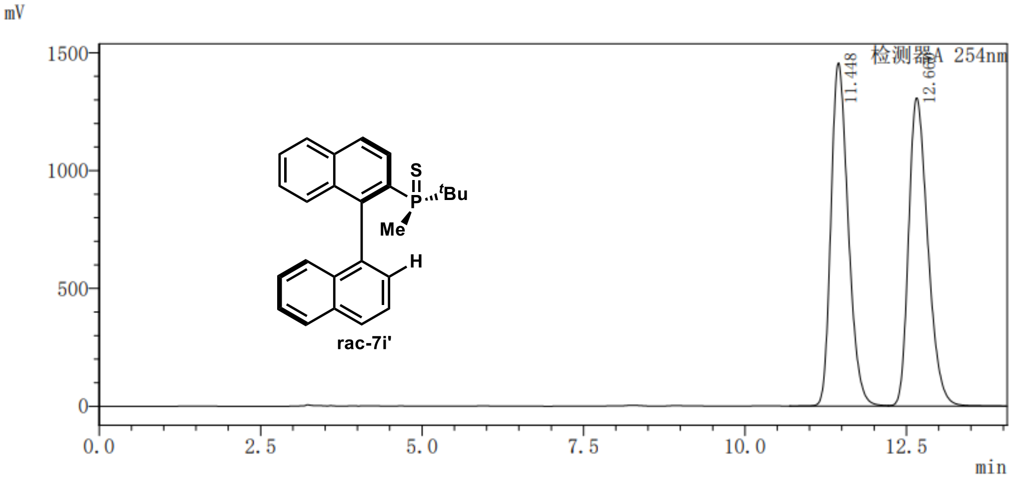

<峰表>

检测器A 254nm

峰号	保留时间	面积	高度	浓度	浓度单位	标记	化合物名
1	11.448	27109519	1455886	50.053			
2	12.660	27051983	1307637	49.947		V	
总计		54161503	2763523				

C:\LabSolutions\Sample\PLZ-6-85B-IC10%27.lcd

Supplementary Fig. 508. HPLC of **7i'-rac.**

SHIMADZU LabSolutions 分析报告

<样品信息>

样品名	: PLZ			
样品ID	: PLZ-220915			
数据文件名	: PLZ-9-192-IC10%4.lcd			
方法文件名	: 1.1cm			
批处理文件名	:			
样品瓶号	: 1-1	样品类型	: 未知	
进样体积	: 1 uL			
分析日期	: 2022/12/8 21:07:45	分析者	: System Administrator	
处理日期	: 2022/12/8 21:34:45	处理者	: System Administrator	

<色谱图>

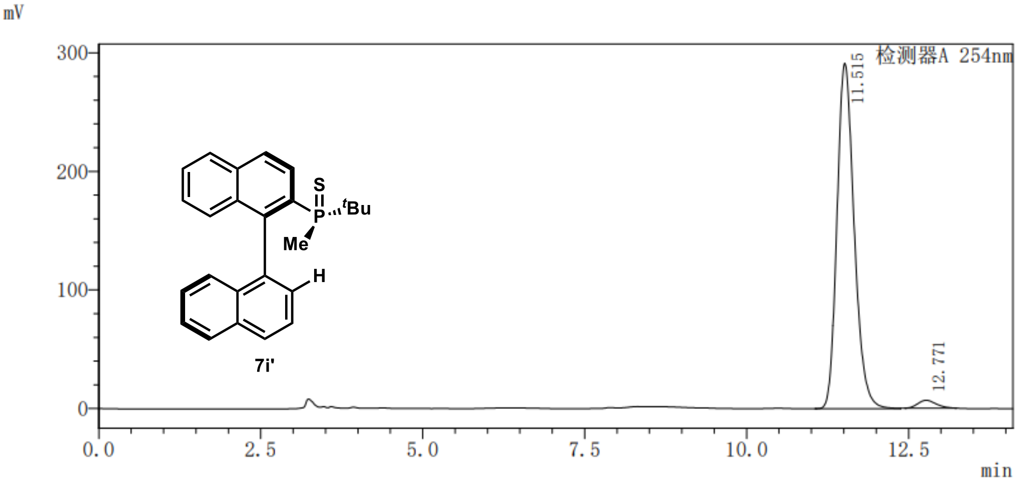

<峰表>

检测器A 254nm

峰号	保留时间	面积	高度	浓度	浓度单位	标记	化合物名
1	11.515	5380464	291336	97.633		M	
2	12.771	130429	6648	2.367		M	
总计		5510893	297983				

C:\LabSolutions\Sample\PLZ-9-192-IC10%4.lcd

Supplementary Fig. 509. HPLC of **7i'.**

SHIMADZU LabSolutions

分析报告

〈样品信息〉

样品名	: PLZ		
样品ID	: PLZ-220915		
数据文件名	: PLZ-9-172B-IC10%13.lcd		
方法文件名	: 1.lcm		
批处理文件名	:		
样品瓶号	: 1-1	样品类型	: 未知
进样体积	: 1 uL		
分析日期	: 2022/11/29 16:21:55	分析者	: System Administrator
处理日期	: 2022/11/29 23:50:13	处理者	: System Administrator

〈色谱图〉

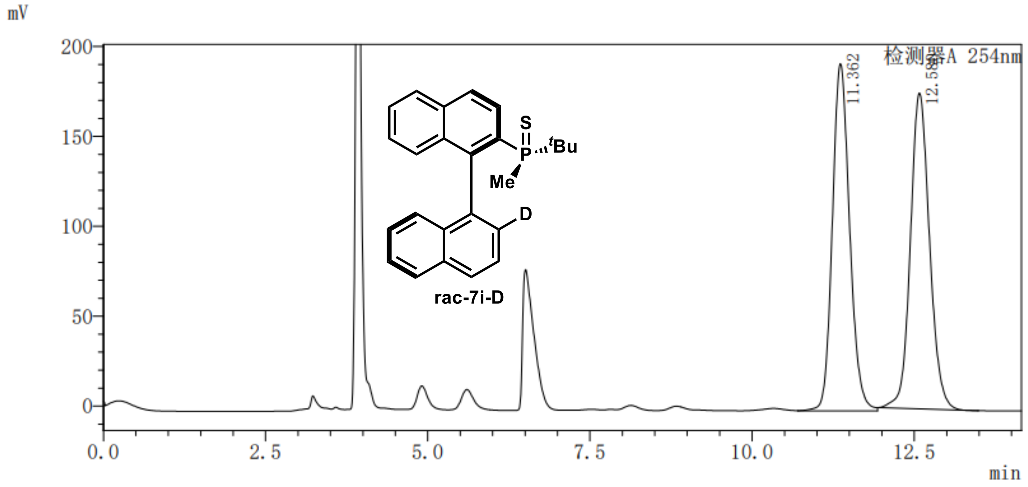

〈峰表〉

检测器A 254nm

峰号	保留时间	面积	高度	浓度	浓度单位	标记	化合物名
1	11.362	3585390	193086	49.861			
2	12.580	3605440	175635	50.139		M	
总计		7190830	368721				

C:\LabSolutions\Sample\PLZ-9-172B-IC10%13.lcd

Supplementary Fig. 510. HPLC of **7i-D-rac.**

SHIMADZU LabSolutions 分析报告

〈样品信息〉

样品名	: PLZ		
样品ID	: PLZ-220915		
数据文件名	: PLZ-9-174-IC10%5.lcd		
方法文件名	: 1.1cm		
批处理文件名	:		
样品瓶号	: 1-1	样品类型	: 未知
进样体积	: 1 uL		
分析日期	: 2022/12/8 21:24:09	分析者	: System Administrator
处理日期	: 2022/12/12 11:12:46	处理者	: System Administrator

〈色谱图〉

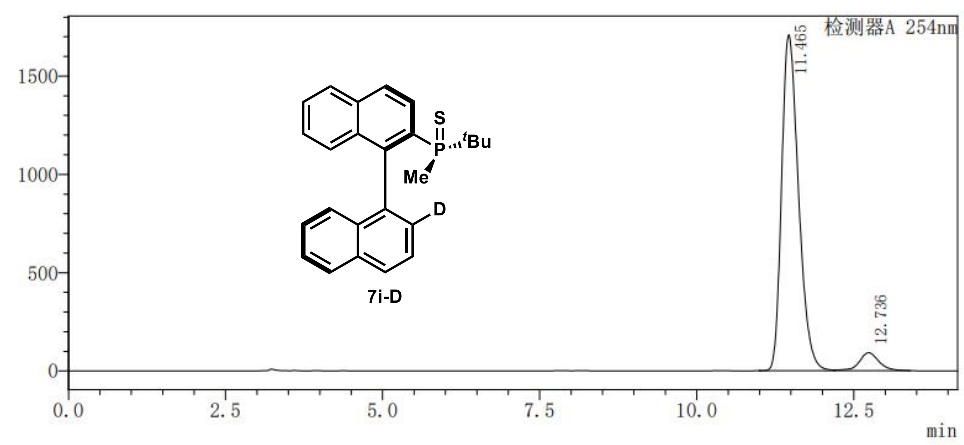

〈峰表〉

检测器A 254nm

峰号	保留时间	面积	高度	浓度	浓度单位	标记	化合物名
1	11.465	32005138	1708387	94.136		M	
2	12.736	1993753	90941	5.864		V M	
总计		33998891	1799328				

C:\LabSolutions\Sample\PLZ-9-174-IC10%5.lcd

Supplementary Fig. 511. HPLC of **7i-D.**

SHIMADZU LabSolutions 分析报告

<样品信息>

样品名	: PLZ		
样品ID	: PLZ-220915		
数据文件名	: PLZ-9-176A-AD-H30%3.lcd		
方法文件名	: 1.lcm		
批处理文件名	:		
样品瓶号	: 1-1	样品类型	: 未知
进样体积	: 1 uL		
分析日期	: 2022/12/13 21:25:33	分析者	: System Administrator
处理日期	: 2022/12/13 21:39:08	处理者	: System Administrator

<色谱图>

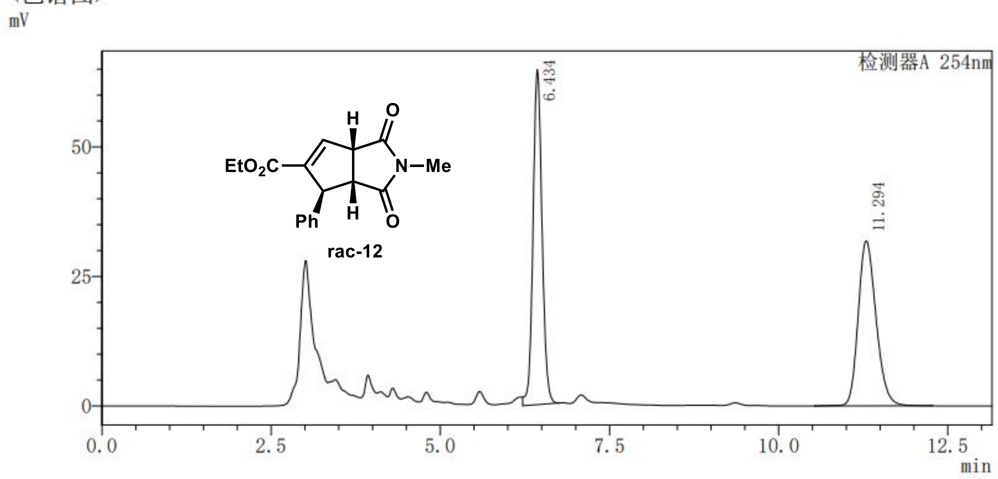

<峰表>

检测器A 254nm

峰号	保留时间	面积	高度	浓度	浓度单位	标记	化合物名
1	6.434	596877	64614	50.461		M	
2	11.294	585963	31852	49.539			
总计		1182840	96466				

C:\LabSolutions\Sample\PLZ-9-176A-AD-H30%3.lcd

Supplementary Fig. 512. HPLC of **12-rac.**

SHIMADZU LabSolutions 分析报告

〈样品信息〉

样品名	: PLZ		
样品ID	: PLZ-220915		
数据文件名	: PLZ-10-13B-AD-H30%2.lcd		
方法文件名	: 1.lcm		
批处理文件名	:		
样品瓶号	: 1-1	样品类型	: 未知
进样体积	: 1 uL		
分析日期	: 2022/12/13 21:08:02	分析者	: System Administrator
处理日期	: 2022/12/13 21:24:35	处理者	: System Administrator

〈色谱图〉

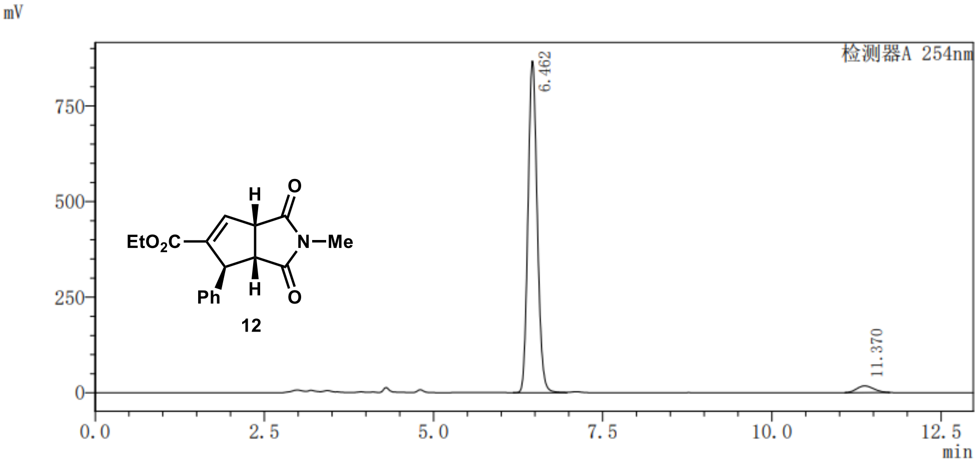

〈峰表〉

检测器A 254nm

峰号	保留时间	面积	高度	浓度	浓度单位	标记	化合物名
1	6.462	8261973	867552	96.471		M	
2	11.370	302269	17401	3.529		M	
总计		8564241	884952				

C:\LabSolutions\Sample\PLZ-10-13B-AD-H30%2.lcd

Supplementary Fig. 513. HPLC of **12.**

4. Supplementary References

[1]. Gladiali, S.; Dore, A.; Fabbri, D.; De Lucchi, O.; & Valle, G. Synthesis, crystal structure, dynamic behavior and reactivity of dinaphtho[2,l-6:l',2'-*d*]phospholes and related atropisomeric phosphacyclic derivatives, *J. Org. Chem.* **59**, 6363-6371 (1994).

[2] Pang, L.; Sun, Q.; Huang, Z.; Li, G.; Liu, J.; Guo, J.; Yao, C.; Yu, J. & Li, Q. Palladium-catalyzed stereoselective cleavage of C-P bond: enantioselective construction of atropisomers containing a P-stereogenic center, *Angew. Chem. Int. Ed.* **61**, e202211710 (2022).

[3] Zhong, F.; Chen, G.-Y.; Han, X.; Yao, W. & Lu, Y. Asymmetric construction of functionalized bicyclic imides via [3+2] annulation of MBH carbonates catalyzed by dipeptide-based phosphines, *Org. Lett.* **14**, 3764-3767 (2012).
